# Combined loss of *CDH1* and downstream regulatory sequences drive early-onset diffuse gastric cancer and increase penetrance of hereditary diffuse gastric cancer

**DOI:** 10.1007/s10120-023-01395-0

**Published:** 2023-05-30

**Authors:** Celina São José, José Garcia-Pelaez, Marta Ferreira, Oscar Arrieta, Ana André, Nelson Martins, Samantha Solís, Braulio Martínez-Benítez, María Luisa Ordóñez-Sánchez, Maribel Rodríguez-Torres, Anna K. Sommer, Iris B. A. W. te Paske, Carlos Caldas, Marc Tischkowitz, Maria Teresa Tusié, Solve-RD DITF-GENTURIS, Stefan Aretz, Gabriel Capella, Sérgio Castedo, Richarda M. de Voer, Gareth Evans, Susana Fernandes, José Garcia-Pelaez, Luzia Garrido, Elke Holinski-Feder, Nicoline Hoogerbrugge, David Huntsman, Arne Jahn, C. Marleen Kets, Andreas Laner, Marjolijn Ligtenberg, Andrea Meinhardt, Arjen Mensenkamp, Carla Oliveira, Sophia Peters, Isabel Quintana, Evelin Schröck, Anna Sommer, Isabel Spier, Liesbeth Spruijt, Verena Steinke-Lange, Iris te Paske, Marc Tischkowitz, Laura Valle, Rachel van der Post, Yasmijn van Herwaarden, Wendy van Zelst-Stams, Doreen William, Nicoline Hoogerbrugge, German Demidov, Richarda M. de Voer, Steve Laurie, Carla Oliveira

**Affiliations:** 1 i3S–Instituto de Investigação e Inovação em Saúde, Rua Alfredo Allen, 208, 4200-135 Porto, Portugal; 2 IPATIMUP–Instituto de Patologia e Imunologia Molecular da Universidade do Porto, Porto, Portugal; 3 Doctoral Programme in Biomedicine, Faculty of Medicine, University of Porto, Porto, Portugal; 4 Department Computer Science Faculty of Science, University of Porto, Porto, Portugal; 5 Thoracic Oncology Unit, Department of Thoracic Oncology, Instituto Nacional de Cancerología, Mexico City, Mexico; 6 Master Programme in Molecular Medicine and Oncology, Faculty of Medicine, University of Porto, Porto, Portugal; 7 INCMNSZ/Instituto de Investigaciones Biomédicas, Unidad de Biología Molecular y Medicina Genómica Instituto Nacional de Ciencias Médicas y Nutrición Salvador Zubirán, UNAM Mexico City, Mexico; 8 Pathology Department, Instituto Nacional de Ciencias Médicas y Nutrición Salvador Zubirán, INCMNSZ Mexico City, Mexico; 9 Institute of Human Genetics, Medical Faculty, University of Bonn, Bonn, Germany; 10 Department of Human Genetics, Radboud University Medical Center, Radboud Institute for Molecular Life Sciences, Nijmegen, The Netherlands; 11 Cancer Research UK Cambridge Institute, University of Cambridge, Li Ka Shing Centre, Cambridge, UK; 12 Department of Oncology, University of Cambridge, Cambridge, UK; 13 Cambridge Experimental Cancer Medicine Centre (ECMC), CRUK Cambridge Centre, NIHR Cambridge Biomedical Research Centre, University of Cambridge and Cambridge University Hospitals NHS Foundation Trust, Cambridge, UK; 14 Department of Medical Genetics, National Institute for Health Research Cambridge Biomedical Research Centre, University of Cambridge, Cambridge, UK; 15 Institute of Medical Genetics and Applied Genomics, Tübingen, Germany; 16 The Barcelona Institute of Science and Technology, CNAG-CRG, Centre for Genomic Regulation (CRG), Barcelona, Spain; 17 FMUP–Faculty of Medicine of the University of Porto, Porto, Portugal

**Keywords:** CDH1, Hereditary diffuse gastric cancer, Regulatory elements, Copy number variants, Deletion, Type-I interferon immune response

## Abstract

**Background:**

Germline *CDH1* pathogenic or likely pathogenic variants cause hereditary diffuse gastric cancer (HDGC). Once a genetic cause is identified, stomachs’ and breasts’ surveillance and/or prophylactic surgery is offered to asymptomatic *CDH1* carriers, which is life-saving. Herein, we characterized an inherited mechanism responsible for extremely early-onset gastric cancer and atypical HDGC high penetrance.

**Methods:**

Whole-exome sequencing (WES) re-analysis was performed in an unsolved HDGC family. Accessible chromatin and *CDH1* promoter interactors were evaluated in normal stomach by ATAC-seq and 4C-seq, and functional analysis was performed using CRISPR-Cas9, RNA-seq and pathway analysis.

**Results:**

We identified a germline heterozygous 23 Kb *CDH1*-*TANGO6* deletion in a family with eight diffuse gastric cancers, six before age 30. Atypical HDGC high penetrance and young cancer-onset argued towards a role for the deleted region downstream of *CDH1*, which we proved to present accessible chromatin, and *CDH1* promoter interactors in normal stomach. CRISPR-Cas9 edited cells mimicking the *CDH1*-*TANGO6* deletion display the strongest *CDH1* mRNA downregulation, more impacted adhesion-associated, type-I interferon immune-associated and oncogenic signalling pathways, compared to wild-type or *CDH1-*deleted cells. This finding solved an 18-year family odyssey and engaged carrier family members in a cancer prevention pathway of care.

**Conclusion:**

In this work, we demonstrated that regulatory elements lying down-stream of *CDH1* are part of a chromatin network that control *CDH1* expression and influence cell transcriptome and associated signalling pathways, likely explaining high disease penetrance and very young cancer-onset. This study highlights the importance of incorporating scientific–technological updates and clinical guidelines in routine diagnosis, given their impact in timely genetic diagnosis and disease prevention.

**Graphical abstract:**

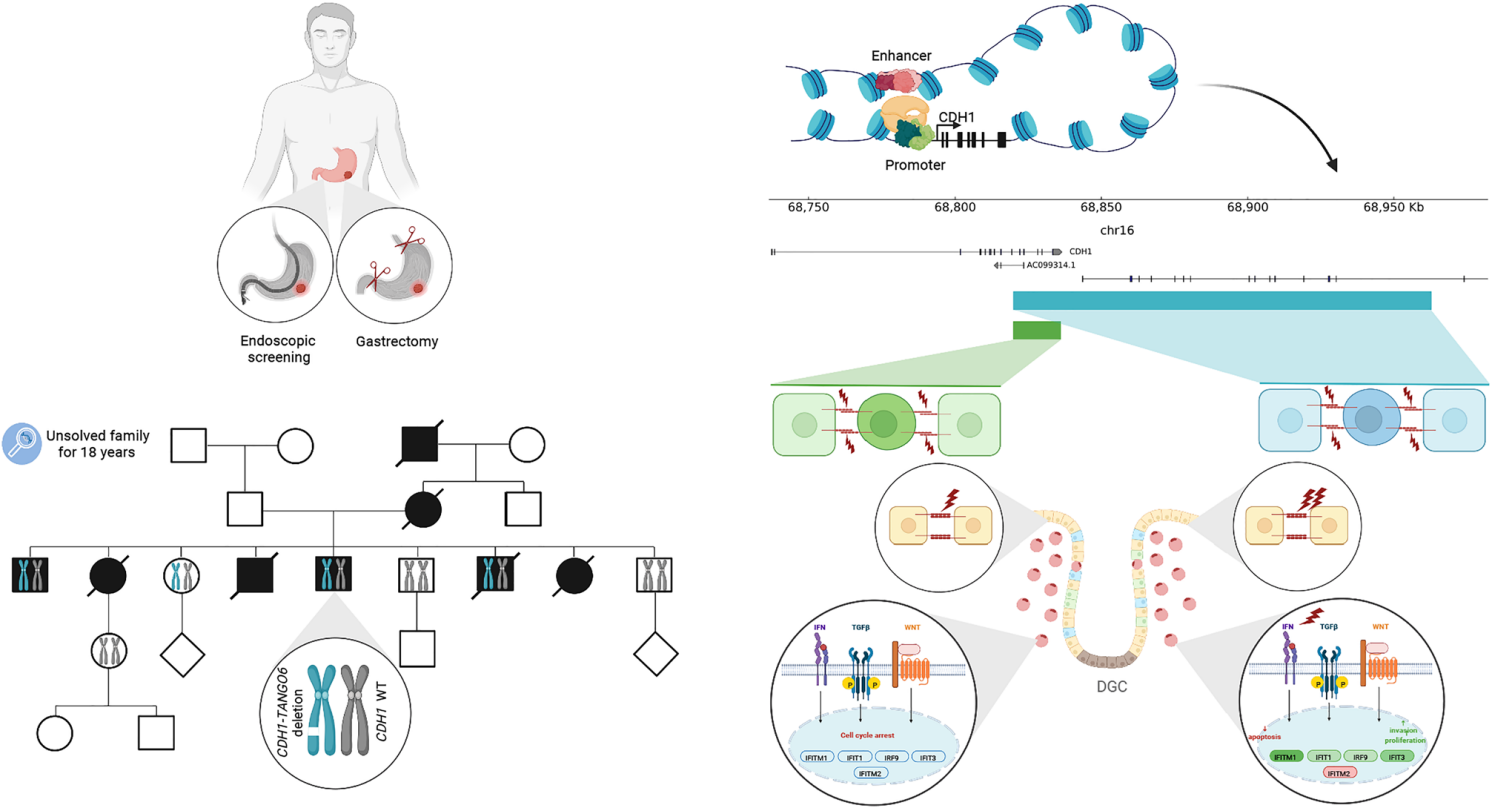

**Supplementary Information:**

The online version contains supplementary material available at 10.1007/s10120-023-01395-0.

## Introduction

Pathogenic and likely pathogenic germline truncating single-nucleotide (SNVs) and copy number variants (CNVs) affecting the *CDH1* coding sequence [1–3], as well as, truncating *CTNNA1* SNVs [4, 5] cause hereditary diffuse gastric cancer (HDGC) syndrome [3, 5, 6]. *CDH1* and *CTNNA1* genes encode epithelial adherens junctions proteins, E-cadherin and αE-catenin respectively [7, 8], whose loss of function reduces cell–cell adhesion and increases invasiveness and cell survival, amongst other consequences [7, 9]. HDGC is autosomal-dominant and predisposes to diffuse gastric cancer (DGC) and lobular breast cancer (LBC) [1, 3].

In *CDH1*-associated HDGC, the cumulative DGC incidence ranges from 42 to 72% in males [10, 11], and from 33 to 55% in females, whilst the risk of LBC ranges from 39 to 55% in females, by 80 years of age [10–13]. Even taking into account that estimates are likely to be biassed, either due to patient geographical origin or due to clinical ascertainment [11], the risk of developing DGC or LBC remains high across different cohorts and studies [3]. Individuals with suspected HDGC are identified and referred for genetic testing, according to guidelines and clinical criteria, which have been evolving since 1999 (supplementary Fig. 1) [14–17], based on technological advances and recognition of novel HDGC-related clinical presentations [3]. Identification of a *CDH1* variant in the proband, classified as pathogenic or likely pathogenic, according to the American College of Medical Genetics and Genomics and the Association for Molecular Pathology (ACMG/AMP) variant curation guidelines for *CDH1* germline variants [18], leads to cascade genetic testing in other family members [17]. Once carrier status is confirmed in asymptomatic relatives, endoscopic surveillance and/or prophylactic surgery are offered according to guidelines to prevent DGC in both genders and/or LBC in females [17]. 

Many families fulfilling the 2020 HDGC clinical criteria remain genetically unexplained, despite the use of candidate-gene, whole-exome (WES), and whole-genome (WGS) sequencing approaches [6, 19]. Missing hereditability in such families may derive from screening approaches excluding CNV analysis or by technical limitations still existing in next-generation sequencing (NGS) protocols and analyses pipelines [20, 21]. In addition, missing hereditability may be hidden in regulatory elements or unscreened genomic regions [22]. The 3D chromatin architecture, and enhancers, may drive gene expression across large genomic distances through physical proximity to the promoter [23]. These mechanisms are often cell-type specific and control overall gene expression, including that of important disease-causing genes [24, 25]. In fact, the gene expression profile of some disease-causing genes is controlled by regulatory elements located in gene bodies, intergenic regions in their vicinity, and other regions of the causal-gene topological-associating domain [26]. CNVs or other type of aberrations affecting these regulatory regions, which consequently interfere with the normal 3D chromatin architecture, may lead to misexpression patterns, causing or aggravating disease [26].

Given the advances in knowledge on all above-described layers, retesting families who tested negative prior to a syndrome-specific breakthrough, implementation of relevant technological improvements and integration of well-validated novel disease-causative mechanisms in routine testing are important aspects in diagnosis and management [27–29].

Herein, we wish to characterize a novel inherited mechanism, that is simultaneously disease-causing and likely responsible for extremely early-onset gastric cancer and atypical high penetrance of HDGC.

## Materials/subjects and methods

### Subjects

A HDGC-suspected family from Mexico was enrolled for meeting the 1999 HDGC clinical criteria [14]. The family presents eight DGC with signet-ring cells. WES of individuals III-1, III-3, III-5, III-6, III-7 and III-9 was available for CNVs re-analysis. Multiplex ligation-dependent probe amplification (MLPA) validation was performed for individuals III-1, III-5, III-6, III-9, IV-1 and IV-3.

### Whole-exome sequencing (WES) re-analysis

WES from HDGC-suspected family [19] was re-analyzed and screened for CNVs using three variant callers (ExomeDepth [30], ClinCNV [31] and Manta [32]) in tumour risk syndromes-associated genes (supplementary table 1) and annotated using AnnotSV [33]. Candidate calls were prioritized considering quality scores, calling by more than one caller and visual evaluation using Integrated Genome Viewer (IGV).

### Multiplex ligation-dependent probe amplification (MLPA)

Specific *CDH1* probes (SALSA MLPA-Probemix P083 *CDH1*, MRC-Holland) were used to identify gene dosage alterations, as previously described [2, 34] and following manufacturer’s recommendations. Probemix includes 20 *CDH1*-specific probes, 2 probes in *CDH1* flanking genes and 13 reference probes in relatively copy number stable regions. Data analysis was performed using Coffalyser.Net (MRC-Holland).

### ATAC-seq and 4C-seq

ATAC-seq data was collected from phase 3 ENCODE project [35]. For 4C-seq, gastric mucosa cells from bariatric surgeries were scraped and enzymatically dissociated. 4C-seq libraries were generated, as previously described [36, 37], digested with DnpII (New England Biolabs) and Csp6I (New England Biolabs) and re-circularized with T4 DNA Ligase (Thermo Fisher Scientific). 4C-seq libraries were PCR-amplified (supplementary Table 2) and sequenced on Illumina HiSeqX technology.

*CDH1* interactions on a genome scale were mapped, based on Pipe4C [37] and PeakC [38] with window size 2, alpha fdr 0.1 and minimal distance 500.

### Cell culture

MKN74 human gastric cancer cell line was purchased from the Japanese collection of research bioresources cell bank. MKN74 and isogenic clones were cultured in RPMI medium (Gibco), supplemented with 10% foetal bovine serum (Biowest) and 1% penicillin streptomycin (Gibco) and maintained at 37 °C under 5% CO_2_ humidified atmosphere.

### CRISPR-Cas9 editing

sgRNAs were designed to target the family CNV or the *CDH1* portion of that CNV using Benchling online platform (supplementary table 3). Individual sgRNAs (Invitrogen) were cloned in LentiCRISPRv2GFP (addgene 82416) or LentiCRISPRv2-mCherry (addgene 99154) vectors using BsmBIv2 (New England Biolabs). Plasmids were transformed into Stbl3 competent cells and colonies were sequenced (supplementary Table 4). Lentiviral particles were produced resourcing to HEK293T cell line with pMD2.G (addgene 12259) and pCMV-dR8.91 (addgene) vectors, following Lipofectamine 3000 manufacture’s protocol (Invitrogen) and collected at 48 h. MKN74 was infected with pairs of lentivirus particles in medium supplemented with 10 μg/μl hexadimethrine bromide (Merck Life Science S.L.U.) for 48 h. Transduced cells were selected for GFP and mCherry positive expression at 7 days post-infection using FACS ARIA (BD Biosciences).

### Genotyping of edited clones

gDNA was extracted using NZY Tissue gDNA isolation kit (NZYTech), according to the manufacturers’ protocol. gDNA was amplified using primers flanking the edition sites (supplementary table 4) and Multiplex PCR kit (Qiagen). PCR products were analysed in gel electrophoresis and Sanger sequenced on an ABI-3130 Genetic Analyzer (Applied Biosystems).

### *CDH1*/E-cadherin expression analysis

*CDH1* mRNA expression was assessed by qPCR in triplicates. RNA was extracted using *mir*Vana RNA Isolation Kit (Invitrogen), according to manufacturers’ protocol. cDNA was synthesized using SuperScriptII reverse transcriptase (Invitrogen), according to the manufacturers’ protocol. *CDH1* mRNA expression was analyzed by qPCR with KAPA PROBE FAST qPCR Master Mix (2X) Kit (Sigma-Aldrich) and probes for *CDH1* (Hs.PT.58.3324071, TaqMan) and 18S (custom assay, IDT). Reactions were sequenced on a 7500 Real-Time PCR System (Applied Biosystems). Relative expression was normalized for the endogenous 18S control and quantified using the 2^−∆∆Ct^ method.

E-cadherin expression was assessed by flow cytometry in triplicates. Cells were detached with Versene (Gibco) and blocked with 3% bovine serum albumin–phosphatase-buffered saline. Cells were incubated with primary mouse monoclonal antibody HECD-1 (Invitrogen), washed and incubated with secondary antibody anti-mouse Alexa Fluor 647 (Invitrogen). Fluorescence was measured using FACS ARIA (BD Biosciences) and Flow Jo version 10 software was used to analyse the data.

### Statistical analysis

Statistical analysis was performed using GraphPad Prism version 7.00 software (GraphPad Software Inc.). A Student’s t test was used for comparison analysis, assuming equal variance between clones and WT samples. Differences were considered significant when *p* value < 0.05.

### RNA-seq and whole transcriptome sequencing bioinformatics analysis

Duplicated RNA samples collected from control and CRISPR-cas9-edited cell lines were sequenced with TruSeq stranded total RNA with Ribo-Zero Gold library in NovaSeq6000. Unique reads were mapped to GRCh38 human genome using STAR and RSeQC and annotated with USCS GRCh38 (Genecode v36). Batch effect was assessed using a PCA and corrected with ComBat_seq from sba (v.3.44) R package for replicates and samples. Differential expression analysis was performed using Deseq2 (v.1.36.0) R package and canonical transcripts were selected considering |log2fold-change|≥ 1 and corrected p value < 0.05. ClusterProfiler (v.4.4.4) R package was used for enrichment GO terms analysis (*q* value < 0.05). Statistics were performed using R (v.4.2.0).

## Results

### The genetic odyssey of a HDGC-suspected family ends with the finding of a *CDH1-TANGO6* deletion

**Fig. 1:**
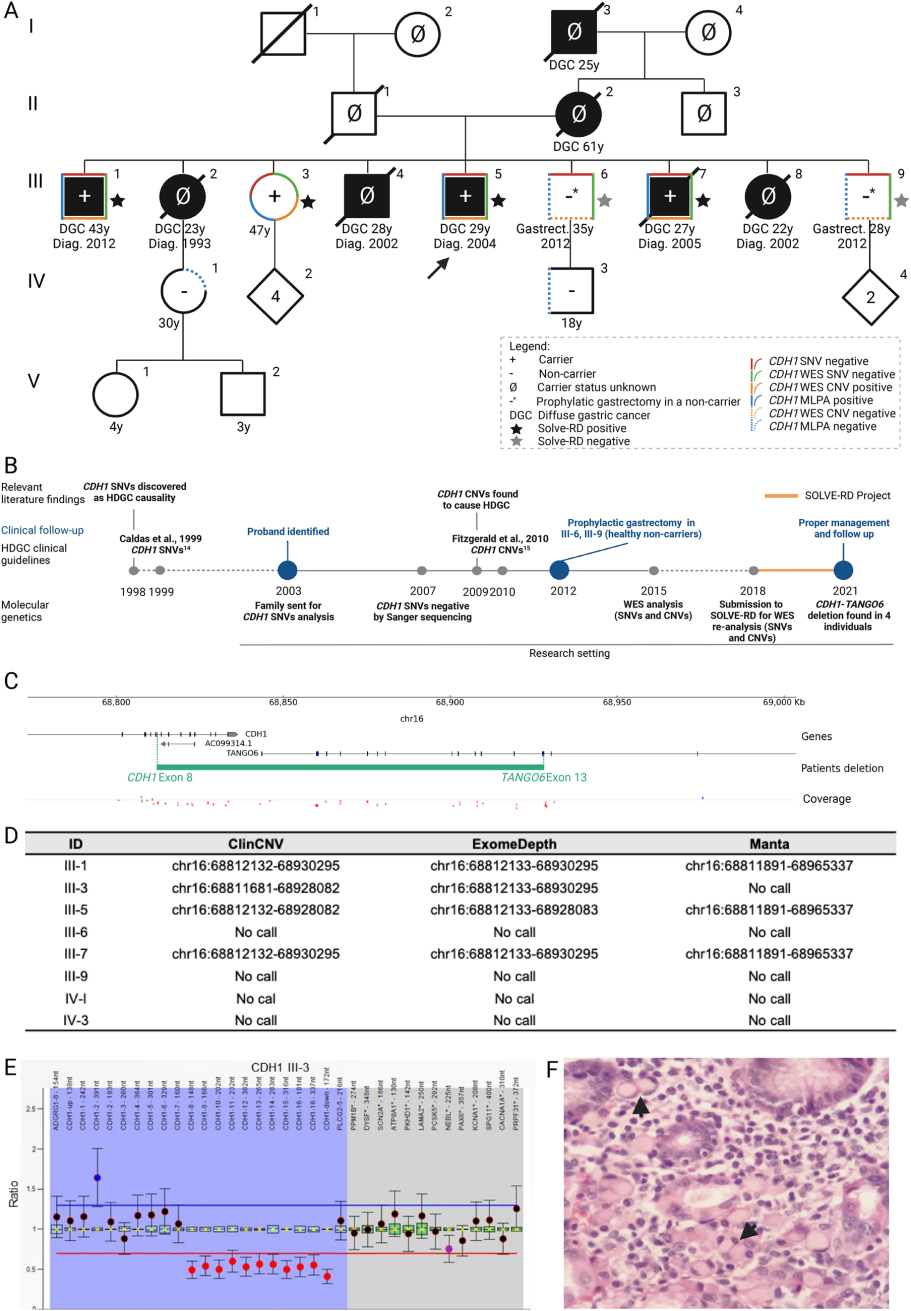
Diagnostic odyssey, pedigree, variant calling details and cancer histology of a HDGC family carrying a causative *CDH1* CNV. **A** Current family pedigree. Full black symbols: individuals with confirmed DGC; Red outline: negative for SNVs (Sanger sequencing); green outline: negative for SNVs (WES); orange outline: positive for CNVs (WES); blue outline: MLPA-positive for the *CDH1* deletion; traced orange outline: negative for CNVs (WES); traced blue outline: MLPA-negative for the CDH1 deletion. All family members, submitted to multiple genetic tests herein described, signed informed consents. The research work has been approved by the Ethics Committee of Centro Hospitalar Universitário São João, in Porto, Portugal, in the frame of the Solve-RD project with the reference CHUSJ_445/2020; **B** Timeline of events and diagnostic odyssey of the family; **C** *CDH1* CNV found by WES, encompassing part of the *CDH1* and *TANGO6* genes (represented in green) and IGV coverage; Green text represents first and last edited exons; **D** Detected *CDH1* CNV in ClinCNV [31], ExomeDepth [30] and Manta [32]; **E** MLPA analysis performed using SALSA MLPA-Probemix P083 *CDH1* (MRC Holland) in patient III-3; Ratio above blue line indicates increased copy number, whilst ratio below red line indicates reduced copy number; Blue highlight represents *CDH1* probes and 2 neighbour genes; Grey highlight represents reference probes; **F** Haematoxylin and eosin staining of DGC depicting signet-ring cells (arrow heads) (100 × magnification) from the proband III-5

A family of Mexican origin, meeting the 1999 HDGC clinical criteria [14], was identified in 2003 showing five DGC in three consecutive generations, three occurring in the same generation at ages 23 (III-2), 28 (III-4) and 22 (III-8) (Fig. 1A). In addition, individuals III-5 and III-7 also developed DGC at age 29 and 27, in 2004 and 2005, respectively. Given the highly suggestive HDGC pedigree, samples from family members that remained alive (III-1, III-3, III-5, III-6, III-7 and III-9), were all sent out for *CDH1* Sanger sequencing, under a research protocol (Fig. 1A, B). The genetic test was negative for the presence of *CDH1* SNVs in 2007. Meanwhile, all asymptomatic family members were integrated into a gastric surveillance protocol, similar to that offered to genetically unsolved HDGC families [14]. In 2012, at age 43, individual III-1 developed DGC and underwent gastric surgery with curative intent. Due to the absence of a genetic diagnosis to find individuals at higher risk of DGC, the clinical team decided to offer (presumably) prophylactic total gastrectomy to asymptomatic individuals III-6 and III-9 (Fig. 1A).

Although, *CDH1* CNVs were first described as a cause of HDGC in 2009 [2], and integrated in HDGC clinical guidelines in 2010 [15], samples from this family were not tested by MLPA. Samples were rather submitted to WES in 2015, again as part of a research protocol, and analysed for SNVs and CNVs which result turned out negative (Fig. 1B), likely due to technical limitations in WES analysis or sample/sequencing quality [39].

WES re-analysis led to the identification of a heterozygous deletion involving half of the *CDH1* gene (starting downstream of *CDH1* exon 7) and the downstream *TANGO6* gene (ending upstream of *TANGO6* exon 14) (Fig. 1B–D). This CNV was identified by several variant callers, namely ‘ClinCNV’ [31], ‘Manta’ [32] and ‘ExomeDepth’ [30] (Fig. 1C,D), and supported by Integrative Genomics Viewer (IGV) visual inspection [40], despite the low quality of the sequencing data (Fig. 1C). The CNV was after validated by MLPA (Fig. 1E) in individuals III-1, III-3, III-5 and III-7 (Fig. 1A). Individuals III-6 and III-9, who had opted for risk-reducing gastrectomy, were actually non-carriers of the CNV (Fig. 1A).

As the pedigree evidences (Fig. 1A), three out of four carriers in generation III, developed DGC with signet-ring cells at ages 27, 29 and 43 (Fig. 1F). Individual III-3 is the only carrier to our knowledge that remains asymptomatic at the age of 47. Besides these family members, there are five not-tested DGC patients, two (I-3 and II-2) died from DGC at ages 25 and 61, being in principle obligated carriers. There are three other relatives (III-2, III-4 and III-8) who died of DGC at age 23, 28 and 22 and were likely also carriers of this predisposing CNV (Fig. 1A). After the genetic diagnosis, a blood sample was obtained from individuals IV-1 and IV-3, who were old enough to be tested, and the CNV screening performed by MLPA returned a negative result for both (Fig. 1A).

### Chromatin conformation analysis highlights physical interactions between the *CDH1* promoter and sequences within the deleted region in normal stomach

Although highly penetrant, HDGC caused by *CDH1* pathogenic or likely pathogenic variants is rarely as penetrant as in this family, which presents an uncommonly high number of individuals in a single generation affected by DGC (six out of seven carriers/presumed carriers). Additionally, five out of six developed DGC before age 29, and average age of cancer onset was 29 ± 7.55 years in six carriers/presumed carriers from generation III. We therefore hypothesised that sequences within the deleted region, and extending into the downstream intergenic region, and the neighbouring *TANGO6* gene sequence, would affect *CDH1* expression and the *locus* 3D chromatin structure more strongly, than deletion of the *CDH1* alone.

**Fig. 2:**
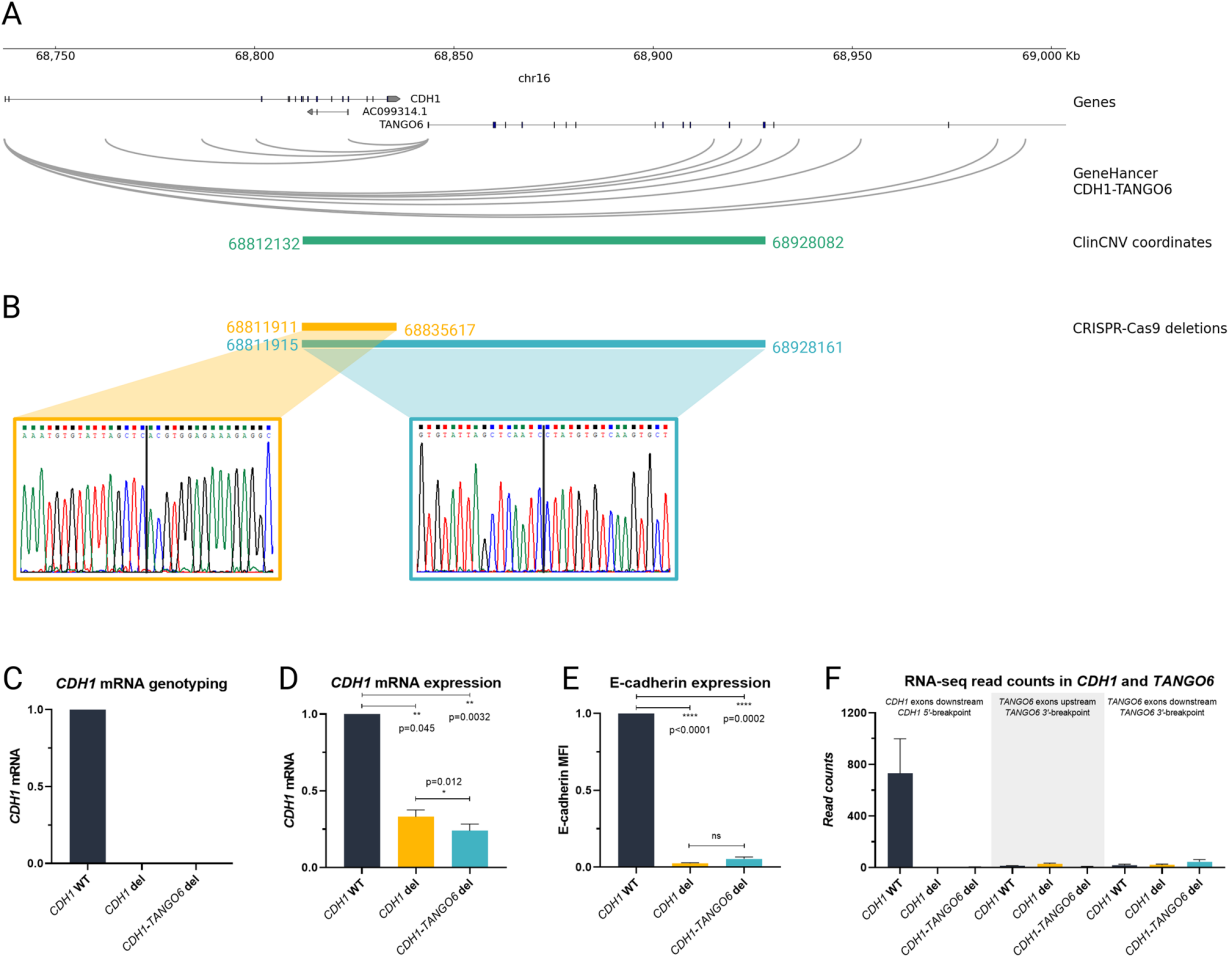
*CDH1* and *TANGO6* regulatory network and characterization of CRISPR-cas9-edited clones. **A** *CDH1*/*TANGO6* potential enhancers depicted in GeneHancer, accessible chromatin and *CDH1* promotor interactors in normal stomach tissue; **B** Sanger sequencing genotyping of the CNV breakpoints, and deletion coordinates in *CDH1* and *CDH1*-*TANGO6* CRISPR-cas9-edited clones; **C** mRNA genotyping of *CDH1* and *CDH1*-*TANGO6*-edited clones resourcing to a probe located in the deleted *CDH1* region; **D** *CDH1* mRNA expression of *CDH1*- and *CDH1*-*TANGO6*-edited clones resourcing to a probe located in exons 6–7 (Hs.PT.58.3324071, TaqMan); **E** E-cadherin protein expression of *CDH1*- and *CDH1*-*TANGO6*-edited clones measured by flow cytometry (monoclonal antibody HECD-1, Invitrogen, 1:100 dilution; and mouse Alexa Fluor 647, Invitrogen, 1:500 dilution); **F** RNA-seq expression read counts of *CDH1* and *TANGO6* genes. Data are represented as the mean ± SEM, MFI: median fluorescence intensity. Experiments depicted in panels C–E were performed in triplicates and differences considered statistically significant if p value < 0.05 in a t-test (details in Materials and Methods)

To address this hypothesis, we mined the GeneHancer database [41] and found that several enhancers within the deleted region, interact with either *TANGO6* or *CDH1* promoters (Fig. 2A). Additionally, we used normal epithelial cells from bariatric surgeries to profile *CDH1* promoter interactions using 4C-seq, and analysed accessible chromatin regions using ATAC-seq from the ENCODE project [35]. We found that the *CDH1* promoter interacts not only with downstream sequences within *CDH1*, but also *TANGO6* and the intergenic region, all deleted by the family CNV, and displaying regions of accessible chromatin (Fig. 2A). These results suggest a role of *CDH1*/*TANGO6* co-regulation in stomach, besides gene physical proximity.

### Deletion of *CDH1* and downstream regulatory sequences, present within the HDGC causal CNV, strongly impact *CDH1* expression and specific signalling pathways

To understand the impact of deleting several *CDH1* promoter-interacting sequences, beyond the *CDH1 locus*, we CRISPR-Cas9 deleted either the *CDH1* portion of the CNV, or mimicked the family CNV. This was performed in a human gastric cancer cell line bearing normal *CDH1/*E-cadherin expression and function [42]. We used sgRNAs flanking target regions (supplementary Table 3), and successful editing was confirmed by Sanger sequencing and qRT-PCR (Fig. 2B,C). A clone bearing a 23.705 bp homozygous deletion spanning from *CDH1* intron 7 and extending till exon 16 (chr16:68,811,911–68,835,617, hg38) and a clone bearing a 116.245 bp homozygous deletion starting in *CDH1* intron 7 and ending after *TANGO6* intron 13 (chr16:68,811,915–68,928,161, hg38) were produced (Fig. 2B).

We found that both *CDH1* and *CDH1*-*TANGO6* deletions led to a significant decrease in *CDH1* mRNA compared to the wild-type (WT) control (p value = 0.045 and p value = 0.0032, respectively), being this downregulation stronger for the *CDH1*-*TANGO6* deletion as compared to the *CDH1* deletion alone (*p* value = 0.012) (Fig. 2D). The level of protein downregulation in relation to the WT control was equivalent in both deletion clones, as assessed by flow cytometry (*p* value < 0.0001 and *p* value = 0.0002, respectively) (Fig. 2E).

**Fig. 3:**
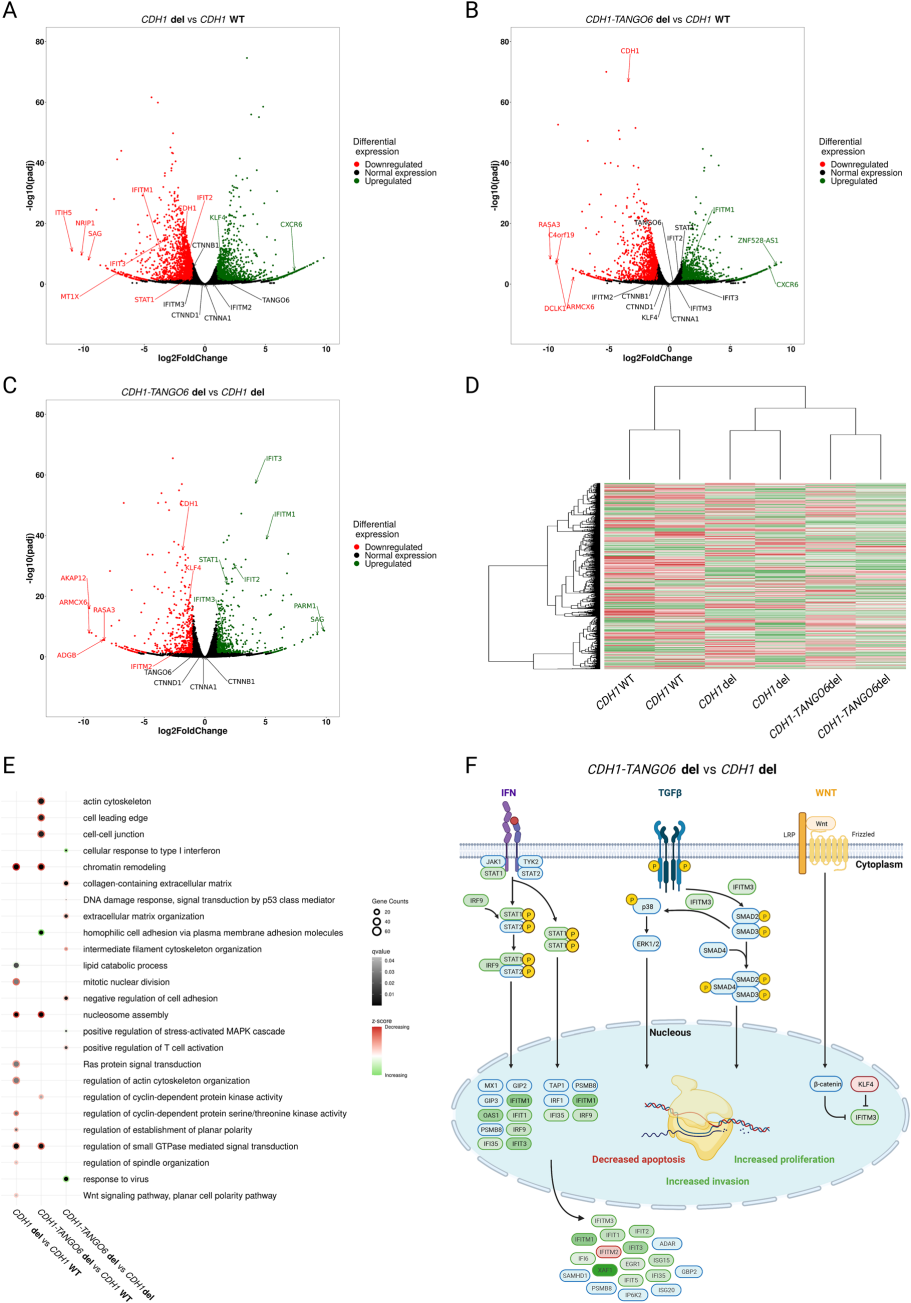
Genome-wide transcriptome regulation of *CDH1* CNV and *CDH1*-*TANGO6* CNV. A) Volcano-plot illustrating *CDH1* CNV differentially expressed genes with *CDH1* WT; **B** Volcano-plot illustrating *CDH1-TANGO6* CNV differentially expressed genes with *CDH1* WT; **C** Volcano-plot illustrating *CDH1-TANGO6* CNV differentially expressed genes with *CDH1* CNV; **D** Heatmap illustrating genome-wide differentially expressed genes; **E** Selected genome-wide transcriptomic misregulated pathways of *CDH1-TANGO6* CNV and *CDH1* CNV. Grey scale represents q value and red–green scale represents z-score (red for pathways with mainly downregulated genes, white for equally downregulated and upregulated genes and green for pathways with mainly upregulated genes); **F** Response to interferon, TGF-β and Wnt signalling pathways misregulated in *CDH1-TANGO6* CNV vs *CDH1* CNV, created with BioRender.com. Red–green scale represents down- to upregulated genes and blue represents normal expressed genes

We next explored the effect of both deletions in the cells’ transcriptomes. For this, we evaluated the differential transcriptome between *CDH1* or *CDH1*-*TANGO6* deletion clones and WT cells, and between *CDH1*-*TANGO6* and *CDH1* deletion clones. We confirmed that *CDH1* mRNA expression was more strongly downregulated (3.4-fold) in the *CDH1*-*TANGO6* deletion than in the *CDH1* deletion alone (1.6-fold), when each was compared to *CDH1* mRNA expression in WT cells (*p* value < 0.0001) (Figs. 2F, 3A,B). We also verified that *TANGO6* is not expressed in WT cells, and its expression remains, as expected, unchanged after the 5’-deletion of the *TANGO6* gene (Fig. 2F).

We also observed that the global patterns of differentially expressed genes clearly differ between clones bearing the *CDH1* deletion alone, the *CDH1*-*TANGO6* deletion and the *CDH1* WT cells (Fig. 3D). There were 3,272 differentially expressed genes between the *CDH1*-deleted clone and *CDH1* WT cells; 2 492 differentially expressed genes between the *CDH1*-*TANGO6*-deleted clone and *CDH1* WT cells; and 1,102 differentially expressed genes between the *CDH1*-*TANGO6* and the *CDH1*-deleted clones (supplementary Tables 5,6,7). Gene ontology analysis further revealed that ‘nucleosome assembly’, ‘chromatin remodelling’, ‘regulation of small GTPase-mediated signal transduction’ as well as pathways related to cyclin-dependent protein kinase activity and actin cytoskeleton were impacted in both in *CDH1* alone and in the *CDH1*-*TANGO6* deletion clones (Fig. 3E, supplementary Table 8).

*CDH1* deletion exclusive events encompass downregulation of ‘mitotic nuclear division’, ‘regulation of spindle organization’ and planar polarity-related pathways, as well as upregulation of ‘lipid catabolic processes’, amongst others (Fig. 3E). Events exclusively altered in the *CDH1*-*TANGO6* deletion clone include mainly impairment of adhesion- and cytoskeleton-associated pathways (Fig. 3E).

The direct comparison between the transcriptional profile of clones bearing the *CDH1*-*TANGO6* deletion and the *CDH1* deletion alone emphasises the negative impact in adhesion-, p53-mediated DNA damage response and cytoskeleton-associated pathways and the positive impact in MAPK cascade-associated proliferation, presented by the *CDH1*-*TANGO6* deletion clone (Fig. 3E). Additional positive and negative impacts were found in several immune-associated pathways, namely related to ‘cellular response to type I interferon’ and ‘response to virus’ in the *CDH1*-*TANGO6* deletion clone (Fig. 3E,F). The expression of *IFITM* genes (*IFITM1*, *IFITM3* and *IFITM2*) and some of their upstream regulators (*STAT1* and *KLF4*) was particularly affected in the *CDH1*-*TANGO6* deletion clone (Fig. 3C,F).

Altogether, and upon data integration, cancer cells arising in patients bearing the *CDH1*-*TANGO6* deletion are likely to present decreased apoptosis, increased proliferation and invasion, and no cell cycle arrest (Fig. 3F).

## Discussion

In this work, we demonstrated that regulatory elements, lying within the *CDH1*-*TANGO6* deletion, are part of a chromatin network that, not only control *CDH1* expression, but also influence the cell transcriptome and associated signalling pathways. We believe this finding explains the high penetrance and extremely young age of cancer-onset in patients from this family. In addition, we describe the diagnostic odyssey of a HDGC-suspected family, that remained genetically unsolved for 18 years, to highlight the importance of incorporating scientific–technological updates and clinical guidelines in routine diagnosis. This is expected to ultimately improve clinical management and care of hereditary cancer patients and families.

A distinctive feature of this family, in comparison with other *CDH1*-positive HDGC families, is the high disease penetrance reflected in generation III, with six out of seven individuals being CNV carriers or presumed carriers and developing DGC. The other is the very early age of cancer-onset (29 ± 7.55 years), compared to the average age reported for DGC in most HDGC families (46,7 ± 16,6 years) [3, 11, 43]. In accordance with our findings, an additional HDGC family has been reported in the literature, bearing a 275 kb *CDH1* (exon 7) and *TANGO6* (full gene) deletion, with four patients in four consecutive generations affected by gastric cancer, from which two were confirmed with signet ring cell carcinoma at ages 30 and 34 years old [44]. These features prompted the next question of this study, which was related to a potential role for the downstream intergenic region and the neighbouring *TANGO6* gene sequence, in modulating *CDH1* expression, the *CDH1 locus* 3D chromatin structure and the transcriptome, in aggravating the clinical presentation in this family.

CRISPR-Cas9 clones mimicking the *CDH1* deletion alone or the family full CNV, demonstrated that the full CNV was more efficient in promoting downregulation of *CDH1* mRNA than the *CDH1* deletion alone. This finding supports the existence of regulatory DNA elements controlling *CDH1* expression, downstream of the *CDH1 locus*, and enclosed in the CNV, which we have also proved to interact with the *CDH1* promoter*.* Similar findings have been previously reported, as for instance those related to neighbour murine *Shh* and *Lmbr1* genes, in which the ZRS enhancer located within the *Lmbr1* gene, regulates *Shh* gene expression [45].

Our data on differential expression and gene ontology analysis of RNA-seq data from clones mimicking the *CDH1* deletion alone or the family full CNV in comparison to *CDH1* WT, highlighted shared biological terms between these clones related to cell division and spindle orientation, and involving chromatin remodelling. These terms have been previously associated with E-cadherin function [46–49], and likely represent the contribution of the *CDH1* locus deletion itself to the differential transcriptome. The fact that adhesion-related (‘negative regulation of cell adhesion’) and actin-related pathways (‘actin cytoskeleton’) were found specifically downregulated upon *CDH1*-*TANGO6* deletion, and in comparison, to *CDH1*-deleted cells, suggests that the perturbations induced by the *CDH1*-*TANGO6* deletion are greater than those induced by the *CDH1* deletion alone in these pathways. Although it is widely known that E-cadherin deficiency leads to weaker adhesion and abnormal microtubule organization [50, 51], which may be involved in migration upon malignant transformation [52], our data specifically link homophilic cell adhesion to the longer *CDH1*-*TANGO6* deletion. Indeed, this clone presents upregulation of the pathway ‘homophilic cell adhesion via plasma membrane adhesion molecules’, which may reflect a yet to prove adhesion-compensatory mechanism, promoted by upregulation of protocadherins in the absence of E-cadherin, seen in our data. A similar switch has been previously demonstrated in lobular breast cancer, whereby E-cadherin is replaced by P-cadherin in cancer cells [53].

Immune-associated genes and pathways were particularly altered in the *CDH1*-*TANGO6* deletion clone, comparing to the clone bearing the *CDH1* deletion alone. *IFITM1* gene was particularly overexpressed in the *CDH1*-*TANGO6* deletion, a feature previously observed in several aggressive tumours, and shown to enhance tumour proliferation and invasion [42, 54]. In gastroesophageal tumours, for instance, *IFITM1* was found overexpressed comparing to normal adjacent tissues [55–57]. Additionally, *IFITM3* overexpression has been shown to promote gastric cancer growth, through the activation of several pathways, resulting in increased proliferation, invasion, and metastasis [58, 59]. IFITM3 has been shown to promote SMAD2/3/4 phosphorylation and interact with STAT1/2 and PIP3/PI3K. This results in activation of downstream proliferation and oncogenic signalling pathways, such as p38/MAPK [60], JAK/STAT [61–63] and PI3K [64]. IFITM3 plays an additional role in the Wnt signalling pathway, being regulated by β-catenin [58] and KLF4 [65]. Indeed, KLF4, known to promote *IFITM3* transcriptional inhibition [65], is downregulated in the *CDH1*-*TANGO6* deletion clone providing a rationale for the *IFITM3* upregulation seen in this clone. Finally, *IFITM2,* a pro-apoptotic gene, encoding a protein which has been linked to protection against tumour proliferation [66], was found downregulated in the *CDH1*-*TANGO6* deletion clone. Most abovementioned observations support a relationship between *IFITM* proteins misregulation and the severe phenotype, observed in this HDGC family. Increased proliferation associated with MAPK cascade [67, 68] and decreased DNA damage response mediated by p53 signalling [69–71], also found in cells bearing the family CNV, may hint to more aggressive DGC, than the commonly described for HDGC patients bearing *CDH1*-coding variants, who generally die by dissemination to the peritoneum [43]. Supporting this, is the fact that individual III-4 died at age 28 with DGC metastases in the lung and bone, a very unusual event in patients with coding *CDH1* variants.

Our data also seem to indicate that whilst a role has not been found for the *TANGO6* mRNA itself, the genomic sequences downstream of the *CDH1 locus*, and within the sequence deleted in this family, host positive regulators of *CDH1* expression. This would explain not only the lower *CDH1* mRNA expression, but also the wider transcriptional and signalling impact, which likely contribute to the clinical presentation in this family. Only two HDGC families have been reported to carry large deletions involving specifically *CDH1* and *TANGO6*, both presenting early-onset DGC and gastric cancer in consecutive generations [44]. Therefore, evidence for the role of regulatory *CDH1* downstream regions in early-onset DGC is still limited. Yet, this study provides the first evidence on the role of *CDH1* downstream sequences in *CDH1* expression control, which may apply to other HDGC families harbouring similar deletions.

This HDGC-suspected family with a severe clinical presentation was somehow lost in genetic follow-up, as clinical standardized diagnostic tests were never used in their clinical path. Negative tests for *CDH1*-coding SNVs and CNVs, both were obtained from research projects, although management guidelines proposed formal genetic testing with Sanger sequencing and MLPA since 2010 in families fulfilling HDGC clinical criteria [2, 15]. This highlights the risk associated with molecular diagnosis being performed in the frame of research studies, rather than in proper diagnostic and clinically approved laboratories, as part of patients’ and families’ pathways of care. In this case, the most obvious disease gene/causing mechanism was overlooked, as for research purposes, these should have been, in principle, excluded. The result of a WES data re-analysis 18 years after clinical diagnosis, revealed a CNV involving the most obvious HDGC-causing gene, *CDH1* [1, 3, 6, 17]. This case highlights the importance of revisiting, re-contacting and retesting families fulfilling clinical criteria but lacking molecular diagnosis, in diagnostic and clinically approved laboratories, as novel and consolidated genetic susceptibility causes of hereditary syndromes arise, and both technologies and management guidelines evolve.

This family odyssey also illustrates the importance of timely genetic diagnosis. For this family, the greater consequences included two carriers developing cancer, two and nine years after the family was recognized clinically as an HDGC-suspected family; and two non-carrier siblings undergoing unnecessary and presumably prophylactic total gastrectomy, nine years after clinical diagnosis, based on family history only. The Solve-RD project (https://solve-rd.eu/) emerged in this case, as a solution to overcome an unfortunate diagnostic odyssey. The finding of a causal *CDH1* CNV was reported to the clinical team at the hospital of origin in 2021, which promoted re-engagement with the family and effective genetic counselling and disease risk management.

Taken together, our data suggest that patients bearing the *CDH1*-*TANGO6* deletion develop high penetrance, earlier-onset and possibly more aggressive DGC, than patients with pathogenic variants confined to the *CDH1*, likely due to the impairment of regulatory elements able to activate oncogenic signalling pathways. It also illustrates the inequities and complexities of genetic diagnosis and integrated care in rare diseases, in a rapidly evolving field, which can impact timely genetic diagnosis and disease prevention.

## Supplementary Information

Below is the link to the electronic supplementary material.

Supplementary file1 (DOCX 37 KB)

Supplementary file2 (PDF 30 KB)

Supplementary file3 (PDF 16 KB)

Supplementary file4 (PDF 23 KB)

Supplementary file5 (PDF 25 KB)

Supplementary file6 (PDF 1120 KB)

Supplementary file7 (PDF 909 KB)

Supplementary file8 (PDF 397 KB)

Supplementary file9 (PDF 131 KB)

Supplementary file10 (PDF 233 KB)

## Data Availability

Raw data supporting the findings of this study are available upon request from the corresponding author. Processed data are available in supplementary materials.
