# Supplementary material for: Combined loss of CDH1 and downstream regulatory sequences drive early-onset diffuse gastric cancer and increase penetrance of hereditary diffuse gastric cancer
Source: Gastric Cancer. 2023 May 30;26(5):653–66. doi: 10.1007/s10120-023-01395-0 (PMC10361908; doi:10.1007/s10120-023-01395-0)
Supplement: Supplementary file 1 — Supplementary file1 (DOCX 37 KB) [file 10120_2023_1395_MOESM1_ESM.docx]

**Materials/Subjects and Methods**

*Subjects*

A HDGC-suspected family was enrolled for meeting the 1999 HDGC clinical criteria [14], which diagnostic odyssey presents eight DGC with signet-ring cells in three consecutive generations. WES bam files of individuals III-1, III-3, III-5, III-6, III-7, III-9 were available for CNVs re-analysis. Multiplex ligation-dependent probe amplification (MLPA) validation was performed for individuals III-1, III-3, III-5, III-6, III-7, III-9, IV-1, IV-3.

*Whole exome sequencing (WES) re-analysis*

Under the Solve-RD project, WES from genetically unsolved families with tumour risk syndromes (TRS) [19] were re-analyzed and screened for structural variants and CNVs using three different variant callers (ExomeDepth [32], ClinCNV [33] and Manta [34]) in TRS-associated genes (**supplementary table 1**) and annotated using AnnotSV [35]. Candidate calls were prioritized considering quality scores and calling by more than one caller. Visual support screenshots using the Integrated Genome Viewer (IGV) were generated for evaluation.

*Multiplex ligation-dependent probe amplification (MLPA)*

Specific-*CDH1* probes were used to identify gene dosage alterations, as previously described[2, 36]. Fifty ng of gDNA was analysed using SALSA MLPA-Probemix P083 *CDH1* (MRC-Holland) and following manufacturer’s recommendations. Data analysis was performed using Coffalyser.Net (MRC-Holland).

*ATAC-seq and 4C-seq*

ATAC-seq data was collected from phase 3 ENCODE project [37]. For 4C-seq, fresh gastric tissue from bariatric surgeries was collected and washed in HBSS 1x (Gibco). Gastric mucosa cells were scraped and enzymatically dissociated with collagenase type I (Merck), dispase (Merck), trypsin inhibitor (Sigma), bovine serum albumin (Nzytech), dithiothreitol (Invitrogen) and HBSS 1x at 37ºC and 150rpm for at least 1h. 4C-seq libraries were generated, as previously described [38,39], containing 1x10^7^ cells, cross-linked in 2% paraformaldehyde and lysed. Nuclei suspensions were digested with DnpII (New England Biolabs) as primary and Csp6I (New England Biolabs) as secondary restriction enzymes and re-circularized with T4 DNA Ligase (Thermo Fisher Scientific). 4C-seq libraries were purified using Amicon Ultra-15 10 kDa (MWCO) (Millipore) and PCR-amplified with 3.2µg per reaction (**supplementary table 2**). Samples were paired-end sequenced on Illumina HiSeqX technology (150bp reads) according to standard protocols.

*CDH1* interactions on a genome scale were mapped from sequenced 4C libraries, using a bioinformatics pipeline based on Pipe4C [39] and PeakC [40] with window size 2, alpha fdr 0.1 and minimal distance 500.

*Cell culture*

MKN74 human gastric cancer cell line was purchased from the japanese collection of research bioresources cell bank. MKN74 and isogenic clones were cultured in RPMI medium (Gibco), supplemented with 10% fetal bovine serum (Biowest) and 1% penicillin streptomycin (Gibco), and maintained at 37°C under 5% CO_2_ humidified atmosphere.

*CRISPR-Cas9 editing*

sgRNAs were designed to target the entire CNV found in the family or the *CDH1* portion of that CNV using Benchling online platform (**supplementary table 3**). Individual sgRNAs (Invitrogen) were cloned in LentiCRISPRv2GFP (addgene 82416) or LentiCRISPRv2-mCherry (addgene 99154) vectors using BsmBI restriction site (New England Biolabs). Plasmids were transformed into Stbl3 competent cells and colonies were screened by PCR and sequenced (**supplementary table 4**). Lentiviral particles were produced resourcing to HEK293T cell line. Briefly, cells were seeded in 6-well plates, grown for 24h until 90%-95% confluency and individual plasmids were co-transfected with pMD2.G (addgene 12259) and pCMV-dR8.91 (addgene), following Lipofectamine 3000 manufacture’s protocol (Invitrogen). Lentiviral particles were collected at 48h, centrifuged and filtered. For transduction, MKN74 were seeded in 6-well plates and grown for 24h until 80% confluency. MKN74 was infected with pairs of lentivirus particles in medium supplemented with 10μg/μl hexadimethrine bromide (Merck Life Science S.L.U.) for 48h. Transduced cells were selected for GFP and mCherry positive expression at 7 days post-infection using FACS ARIA (BD Biosciences).

*Genotyping of edited clones*

gDNA was extracted using NZY Tissue gDNA isolation kit (NZYTech), according to the manufacturers’ protocol. gDNA was amplified using primers flanking the edition sites (**supplementary table 4**) and Multiplex PCR kit (Qiagen). PCR products were analysed in gel electrophoresis and Sanger sequenced using BigDye Terminator v.3.1 cycle sequencing kit (Thermo Fisher Scientific) on an ABI-3130 Genetic Analyzer (Applied Biosystems).

*CDH1/E-cadherin expression analysis*

*CDH1* mRNA expression was assessed by qPCR in triplicates. Briefly, RNA was extracted using *mir*Vana RNA Isolation Kit (Invitrogen), according to manufacturers’ protocol. cDNA was synthesized using 1µg of template RNA and SuperScriptII reverse transcriptase (Invitrogen), according to the manufacturers’ protocol. *CDH1* mRNA expression was analyzed by qPCR with KAPA PROBE FAST qPCR Master Mix (2X) Kit (Sigma-Aldrich) and probes for *CDH1* (Hs.PT.58.3324071, TaqMan) and 18S (custom assay, IDT) as endogenous control. Reactions were sequenced on a 7500 Real-Time PCR System (Applied Biosystems). Relative expression was normalized for the endogenous 18S control and quantified using the 2−∆∆Ct method.

E-cadherin expression was assessed by flow cytometry in triplicates. Cells were detached with Versene (Gibco) and blocked with 3% bovine serum albumin-phosphatase buffer saline for 30 min. Cells were incubated with primary mouse monoclonal antibody HECD-1 (1:100 dilution; 1h at 4ºC; Invitrogen), washed and incubated with secondary antibody anti-mouse Alexa Fluor 647 (1:500; 45min at 4ºC; Invitrogen). Fluorescence was measured using FACS ARIA (BD Biosciences) and Flow Jo version 10 software was used to analyse the data.

*Statistical analysis*

Statistical analysis was performed using GraphPad Prism version 7.00 software (GraphPad Software Inc.). A T-student test was used for comparison analysis, assuming equal variance between clones and WT samples. Differences were considered significant when p-value<0.05.

*RNA-seq* and *whole transcriptome sequencing bioinformatics analysis*

Duplicated RNA samples collected previously were sequenced with TruSeq stranded total RNA with Ribo-Zero Gold library in NovaSeq6000 100bp paired-end reads after treatment with DNAse. Unique reads were mapped to GRCh38 human genome using STAR and RSeQC for quantification of the whole transcriptome using the annotation of USCS GRCh38 (Genecode v36) as a whole transcriptome reference. Batch effect was assessed using a PCA and corrected with ComBat_seq from sba (v.3.44) R package for replicates and samples. Deseq2 (v.1.36.0) R package was used to perform the differentially-expression analysis. Canonical transcripts of differentially expressed genes were selected by a |log2fold-change|≥1 and corrected p-value<0.05. Statistics was performed using R (v.4.2.0). ClusterProfiler (v.4.4.4) R package was used for assessment of significantly enriched GO terms (q-value<0.05).
