# Supplementary material for: Combined loss of CDH1 and downstream regulatory sequences drive early-onset diffuse gastric cancer and increase penetrance of hereditary diffuse gastric cancer
Source: Gastric Cancer. 2023 May 30;26(5):653–66. doi: 10.1007/s10120-023-01395-0 (PMC10361908; doi:10.1007/s10120-023-01395-0)
Supplement: Supplementary file 2 — Supplementary file2 (PDF 30 KB) [file 10120_2023_1395_MOESM2_ESM.pdf]

Supplementary table 1. Genes associated with gastrointestinal syndromes

Gene name	Reference sequence
A2ML1	NM_144670.6
ACD	NM_001082486.2
ADM	NM_001124.3
AIP	NM_003977.4
AKT1	NM_001014431.2
ALK	NM_004304.5
ANKRD26	NM_014915.2
APC	NM_001354896.2
ARMC5	NM_001288767.2
ASXL1	NM_015338.6
ATM	NM_000051.3
ATR	NM_001184.4
AXIN1	NM_003502.4
AXIN2	NM_004655.4
BAP1	NM_004656.4
BARD1	NM_000465.4
BLM	NM_000057.4
BMPR1A	NM_004329.2
BRAF	NM_001354609.2
BRCA1	NM_007300.4
BRCA2	NM_000059.3
BRIP1	NM_032043.3
BUB1	NM_004336.5
BUB1B	NM_001211.5
BUB3	NM_004725.4
CBL	NM_005188.4
CDC73	NM_024529.4
CDH1	NM_004360.5
CDK4	NM_000075.4
CDKN1A	NM_001291549.2
CDKN1B	NM_004064.4
CDKN1C	NM_000076.2
CDKN2A	NM_001195132.1
CDKN2B	NM_004936.4
CDKN2C	NM_001262.2
CEBPA	NM_001287424.2
CHEK1	NM_001330427.1
CHEK2	NM_001005735.2
CREBBP	NM_004380.3
CTC1	NM_025099.6
CTNNA1	NM_001323982.2
CTR9	NM_014633.5
CYLD	NM_015247.2
DDB2	NM_000107.2
DDX11	NM_001257144.2

DICER1	NM_001271282.3
DIS3L2	NM_152383.4
DKC1	NM_001363.5
DNAJC21	NM_194283.4
EGFR	NM_005228.5
ELANE	NM_001972.4
ENG	NM_001114753.2
EPCAM	NM_002354.3
ERCC1	NM_001369408.1
ERCC2	NM_000400.3
ERCC3	NM_000122.2
ERCC4	NM_005236.3
ERCC5	NM_000123.3
ERCC6	NM_000124.4
ESR2	NM_001437.2
ETV6	NM_001987.5
EXO1	NM_006027.4
EXT1	NM_000127.2
EXT2	NM_000401.3
EZH2	NM_004456.5
FAN1	NM_014967.5
FANCA	NM_000135.4
FANCB	NM_001018113.3
FANCC	NM_000136.3
FANCD2	NM_033084.5
FANCE	NM_021922.3
FANCF	NM_022725.4
FANCG	NM_004629.1
FANCI	NM_001113378.1
FANCL	NM_001114636.1
FANCM	NM_020937.4
FAS	NM_000043.6
FBXW7	NM_001349798.2
FH	NM_000143.3
FLCN	NM_001353229.2
FOXO3	NM_001455.4
G6PC3	NM_138387.3
GALNT12	NM_024642.5
GATA2	NM_001145661.2
GDNF	NM_001190468.1
GFI1	NM_001127215.2
GPC3	NM_001164617.2
GREM1	NM_001368719.1
GRHL2	NM_024915.4
HABP2	NM_004132.5
HAX1	NM_006118.4
HNF1A	NM_001306179.2
HOXB13	NM_006361.5

IPMK	NM_152230.5
KIF1B	NM_001365951.2
KIT	NM_000222.2
KLLN	NM_001126049.1
KRAS	NM_001369786.1
LIG4	NM_001352604.1
LZTR1	NM_006767.4
MAP2K1	NM_002755.3
MAP2K2	NM_030662.3
MAX	NM_002382.5
MDH2	NM_005918.4
MEN1	NM_001370251.1
MET	NM_001127500.3
MITF	NM_001354604.2
MLH1	NM_000249.3
MLH3	NM_001040108.1
MPL	NM_005373.3
MRE11A	NM_005591.3
**Gene name**	**Reference sequence**
MSH2	NM_000251.3
MSH3	NM_002439.5
MSH6	NM_000179.2
MUC5B	NM_002458.3
MUTYH	NM_001128425.1
NBN	NM_002485.4
NF1	NM_001042492.3
NF2	NM_000268.3
NHP2	NM_017838.3
NOP10	NM_018648.3
NPM1	NM_001355006.1
NRAS	NM_002524.5
NSD1	NM_022455.4
NTHL1	NM_002528.7
OGG1	NM_016821.2
PALB2	NM_024675.4
PARK2	NM_004562.3
PARN	NM_002582.4
PAX5	NM_016734.3
PDGFB	NM_002608.4
PDGFRA	NM_001347828.2
PHOX2B	NM_003924.4
PIK3CA	NM_006218.4
PMS1	NM_000534.4
PMS2	NM_001322014.2
PMS2CL	NR_002217.1
POLD1	NM_001308632.1
POLD2	NM_006230.3
POLD3	NM_006591.3

POLD4	NM_021173.5
POLE	NM_006231.4
POLE2	NM_002692.4
POLE3	NM_001278255.1
POLE4	NM_019896.4
POLH	NM_006502.3
POT1	NM_015450.3
POU6F2	NM_001370959.1
PPM1D	NM_003620.4
PRF1	NM_001083116.3
PRKAR1A	NM_001276289.1
PRSS1	NM_002769.5
PTCH1	NM_000264.5
PTCH2	NM_003738.5
PTEN	NM_001304717.5
PTPN11	NM_001330437.1
PTPN12	NM_002835.4
PTPRJ	NM_002843.4
RAD50	NM_005732.4
RAD51C	NM_058216.3
RAD51D	NM_001142571.2
RAF1	NM_001354689.3
RB1	NM_000321.2
RECQL	NM_002907.4
RECQL4	NM_004260.3
REST	NM_001193508.1
RET	NM_020975.6
RHBDF2	NM_024599.5
RINT1	NM_021930.6
RIT1	NM_001256821.2
RNF43	NM_001305544.2
RPL11	NM_000975.5
RPL35A	NM_000996.4
RPL5	NM_000969.5
RPS10	NM_001014.5
RPS17	NM_001021.6
RPS19	NM_001321485.2.2
RPS20	NM_001146227.2
RPS24	NM_001142285.2
RPS26	NM_001029.5
RPS7	NM_001011.4
RTEL1	NM_001283009.2
RUNX1	NM_001754.4
SAMD9	NM_001193307.1
SAMD9L	NM_001303496.3
SBDS	NM_016038.4
SDHA	NM_004168.4
SDHAF2	NM_017841.2

SDHB	NM_003000.3
SDHC	NM_003001.3
SDHD	NM_003002.4
SEC23B	NM_001172745.3
SEMA4A	NM_001193300.2
SFTPA1	NM_001093770.3
SFTPA2	NM_001320814.1
SH2B3	NM_005475.3
SHOC2	NM_001324336.1
SLC26A3	NM_000111.3
SLX4	NM_032444.4
SMAD4	NM_005359.6
SMAD9	NM_001127217.2
SMARCA4	NM_001128849.2
SMARCB1	NM_001362877.2
SMARCE1	NM_003079.5
SOS1	NM_005633.3
SPINK1	NM_001354966.1
SPRED1	NM_152594.3
SQSTM1	NM_003900.5
SRC	NM_005417.4
STK11	NM_000455.5
SUFU	NM_016169.3
TERC	NR_001566.1
TERF2IP	NM_018975.4
TERT	NM_198253.3
TINF2	NM_001099274.3
TMEM127	NM_001193304.3
TNFRSF11A	NM_003839.4
TP53	NM_000546.5
TRIP13	NM_004237.4
TSC1	NM_000368.5
TSC2	NM_000548.5
USB1	NM_024598.4
VHL	NM_000551.3
WAS	NM_000377.3
WRAP53	NM_001143990.1
WRN	NM_000553.6
WT1	NM_024426.6
XPA	NM_000380.3
XPC	NM_004628.4
