# Supplementary material for: Combined loss of CDH1 and downstream regulatory sequences drive early-onset diffuse gastric cancer and increase penetrance of hereditary diffuse gastric cancer
Source: Gastric Cancer. 2023 May 30;26(5):653–66. doi: 10.1007/s10120-023-01395-0 (PMC10361908; doi:10.1007/s10120-023-01395-0)
Supplement: Supplementary file 3 — Supplementary file3 (PDF 16 KB) [file 10120_2023_1395_MOESM3_ESM.pdf]

Supplementary table 2. Primers used for 4C-seq

Primer	Sequence
4C_prom_P5_N502	AATGATACGGCGACCACCGAGATCTACACCTCTCTATTCGTCGGCAGCGTCAGATGTGTATAAGAGACAGGTGAACCCTCAGCCAATCAG
4C_prom_P5_N504	AATGATACGGCGACCACCGAGATCTACACAGAGTAGATCGTCGGCAGCGTCAGATGTGTATAAGAGACAGGTGAACCCTCAGCCAATCAG
4C_prom_P5_N503	AATGATACGGCGACCACCGAGATCTACACTATCCTCTTCGTCGGCAGCGTCAGATGTGTATAAGAGACAGGTGAACCCTCAGCCAATCAG
4C_prom_P5_N501	AATGATACGGCGACCACCGAGATCTACACTAGATCGCTCGTCGGCAGCGTCAGATGTGTATAAGAGACAGGTGAACCCTCAGCCAATCAG
4C_prom_P7_N705	CAAGCAGAAGACGGCATACGAGATAGGAGTCCGTCTCGTGGGCTCGGAGATGTGTATAAGAGACAGGATCCCAGGTCTTAGTGAGCCA
4C_prom_P7_N707	CAAGCAGAAGACGGCATACGAGATGTAGAGAGGTCTCGTGGGCTCGGAGATGTGTATAAGAGACAGGATCCCAGGTCTTAGTGAGCCA
4C_prom_P7_N706	CAAGCAGAAGACGGCATACGAGATCATGCCTAGTCTCGTGGGCTCGGAGATGTGTATAAGAGACAGGATCCCAGGTCTTAGTGAGCCA
4C_prom_P7_N708	CAAGCAGAAGACGGCATACGAGATCCTCTCTGGTCTCGTGGGCTCGGAGATGTGTATAAGAGACAGGATCCCAGGTCTTAGTGAGCCA
