# Supplementary material for: Combined loss of CDH1 and downstream regulatory sequences drive early-onset diffuse gastric cancer and increase penetrance of hereditary diffuse gastric cancer
Source: Gastric Cancer. 2023 May 30;26(5):653–66. doi: 10.1007/s10120-023-01395-0 (PMC10361908; doi:10.1007/s10120-023-01395-0)
Supplement: Supplementary file 4 — Supplementary file4 (PDF 23 KB) [file 10120_2023_1395_MOESM4_ESM.pdf]

Supplementary table 3. sgRNAs used for CRISPR-Cas9

sgRNA	Sequence
CDH1 intron 7	TGTGTATTAGCTCAATCCCG
CDH1 3UTR	AGATGGCCTCAGTCAAAACG
TANGO6 5UTR	GCTTCTGAAATTGTGCGCGT
TANGO6 intron 13	CAGCACTTGACACATAGTCC
