# Supplementary material for: Combined loss of CDH1 and downstream regulatory sequences drive early-onset diffuse gastric cancer and increase penetrance of hereditary diffuse gastric cancer
Source: Gastric Cancer. 2023 May 30;26(5):653–66. doi: 10.1007/s10120-023-01395-0 (PMC10361908; doi:10.1007/s10120-023-01395-0)
Supplement: Supplementary file 5 — Supplementary file5 (PDF 25 KB) [file 10120_2023_1395_MOESM5_ESM.pdf]

Supplementary table 4. Primers used for CRISPR-Cas9

Primer	Sequence	Orientation	Used for
Cas9	GGGCCTATTTCCCATGATTCCTT	Forward	Colony PCR
Cas9	GACTCGGTGCCACTTTTTCAA	Reverse	Colony PCR
CDH1 del 5’	GGGCAGAATTGGATTAAGCA	Forward	Genotyping
CDH1 del 3’	TGGCCTGTACTCACTTGCTG	Reverse	Genotyping
CDH1-TANGO6 del 5’	GGGCAGAATTGGATTAAGCA	Forward	Genotyping
CDH1-TANGO6 del 3’	AACATGGTGCCCGTCTCTAC	Reverse	Genotyping
