# Supplementary material for: Combined loss of CDH1 and downstream regulatory sequences drive early-onset diffuse gastric cancer and increase penetrance of hereditary diffuse gastric cancer
Source: Gastric Cancer. 2023 May 30;26(5):653–66. doi: 10.1007/s10120-023-01395-0 (PMC10361908; doi:10.1007/s10120-023-01395-0)
Supplement: Supplementary file 6 — Supplementary file6 (PDF 1120 KB) [file 10120_2023_1395_MOESM6_ESM.pdf]

Supplementary table 5. Differentially expressed genes in CDH1 del vs CDH1 WT

Transcript.stable.ID	baseMean	log2FoldChange	lfcSE	stat	pvalue	padj	Gene.stable.ID	chr	start	end	Gene.name
ENST00000003100	1632.182159	-2.234992329	0.18711	-11.94	6.92E-33	5.42E-30	ENSG00000001630	7	92112152	92134477	CYP51A1
ENST00000005259	534.5836023	-1.337110833	0.21281	-6.283	3.32E-10	1.30E-08	ENSG00000075790	7	107580245	107620478	BCAP29
ENST00000012049	93.98003828	1.154271025	0.3744	3.083	0.0020496	0.0122673	ENSG00000011478	19	45692665	45703987	QPCTL
ENST00000035307	689.1049433	1.346515835	0.20235	6.6543	2.85E-11	1.42E-09	ENSG00000033100	7	151232482	151238822	CHPF2
ENST00000046640	250.4024207	1.362202567	0.29099	4.6812	2.85E-06	4.50E-05	ENSG00000040531	17	3636759	3663103	CTNS
ENST00000054650	551.4463179	1.087191269	0.21744	5	5.73E-07	1.09E-05	ENSG00000041988	1	6624867	6633562	THAP3
ENST00000054950	1455.246042	-1.864932313	0.19859	-9.391	5.95E-21	1.19E-18	ENSG00000049449	11	32091073	32105722	RCN1
ENST00000078429	483.6229349	-1.041521223	0.23312	-4.468	7.91E-06	0.0001097	ENSG00000088256	19	3094361	3123999	GNA11
ENST00000084798	22.16190986	-1.916003655	0.72899	-2.628	0.0085812	0.0387996	ENSG00000063180	19	48637945	48646187	CA11
ENST00000155926	209.228756	2.924912272	0.34569	8.461	2.65E-17	3.39E-15	ENSG00000071575	2	12716935	12742734	TRIB2
ENST00000161006	118.6223499	1.323168811	0.3367	3.9298	8.50E-05	0.0008575	ENSG00000005001	16	2852729	2858170	PRSS22
ENST00000164024	119.5052146	1.648723889	0.36324	4.539	5.65E-06	8.18E-05	ENSG00000008300	3	48636462	48662886	CELSR3
ENST00000164305	259.5153886	1.020587343	0.26314	3.8785	0.0001051	0.001022	ENSG00000069943	15	55319221	55355648	PIGB
ENST00000169551	85.87357501	-1.195949646	0.38154	-3.135	0.0017211	0.0106441	ENSG00000075336	18	74148522	74160531	TIMM21
ENST00000173527	136.2790492	-1.284971469	0.33306	-3.858	0.0001143	0.0010993	ENSG00000066583	5	129094748	129114028	ISOC1
ENST00000174618	114.8559549	1.083882132	0.36427	2.9755	0.0029256	0.0163589	ENSG00000070444	17	2384072	2401060	MNT
ENST00000190611	7.827928568	-6.443340524	1.8128	-3.554	0.0003789	0.0030355	ENSG00000079156	2	178194511	178402893	OSBPL6
ENST00000194155	235.680574	-1.044347715	0.26278	-3.974	7.06E-05	0.0007327	ENSG00000112852	5	141094614	141098703	PCDHB2
ENST00000202017	214.6771132	1.069301601	0.31994	3.3422	0.0008311	0.0058578	ENSG00000088356	20	31944336	31952046	PDRG1
ENST00000202917	235.6362525	-4.58846601	1.01617	-4.515	6.32E-06	9.00E-05	ENSG00000089127	12	112906961	112919903	OAS1
ENST00000204517	109.6091244	-1.66409051	0.36099	-4.61	4.03E-06	6.08E-05	ENSG00000090447	16	4257185	4273023	TFAP4
ENST00000204679	691.4259833	1.154852739	0.20202	5.7164	1.09E-08	3.15E-07	ENSG00000090581	16	1351930	1364113	GNPTG
ENST00000205194	110.2378476	-1.582679508	0.37168	-4.258	2.06E-05	0.0002514	ENSG00000090971	19	55485187	55487566	NAT14
ENST00000206595	1170.975074	-1.189528227	0.18591	-6.399	1.57E-10	6.58E-09	ENSG00000092140	14	30559157	30620064	G2E3
ENST00000209718	2581.504259	-1.136470592	0.17666	-6.433	1.25E-10	5.39E-09	ENSG00000108244	17	40922699	40937646	KRT23
ENST00000212355	2714.597923	-1.128224941	0.18576	-6.074	1.25E-09	4.42E-08	ENSG00000069702	1	91680342	91886151	TGFBR3
ENST00000214869	168.9823521	-1.395287837	0.3025	-4.612	3.98E-06	6.01E-05	ENSG00000099203	19	10832066	10836212	TMED1
ENST00000215730	1690.183089	1.314836931	0.18672	7.0417	1.90E-12	1.23E-10	ENSG00000099940	22	20859006	20891214	SNAP29
ENST00000215829	1722.046081	-1.807230337	0.23933	-7.551	4.31E-14	3.64E-12	ENSG00000100028	22	24555998	24574971	SNRPD3
ENST00000215909	884.3843459	-1.935147383	0.20544	-9.42	4.53E-21	9.16E-19	ENSG00000100097	22	37675635	37679802	LGALS1
ENST00000216014	76.37056086	-1.309403505	0.44114	-2.968	0.0029955	0.0166701	ENSG00000100196	22	38468095	38483447	KDELR3
ENST00000216085	379.0046914	1.922065981	0.2431	7.9066	2.65E-15	2.64E-13	ENSG00000100263	22	29259871	29267981	RHBDD3
ENST00000216124	96.20146041	2.059051443	0.44462	4.631	3.64E-06	5.56E-05	ENSG00000100299	22	50622753	50628152	ARSA
ENST00000216133	169.1646339	1.429704357	0.345	4.1441	3.41E-05	0.0003901	ENSG00000100307	22	39130771	39152680	CBX7
ENST00000216259	136.0436116	1.111055457	0.32817	3.3856	0.0007102	0.0051462	ENSG00000100417	22	41576899	41589840	PMM1
ENST00000216271	198.7334029	1.058075002	0.32739	3.2318	0.0012301	0.0080878	ENSG00000100429	22	50245182	50251265	HDAC10
ENST00000216367	279.9818204	-1.02114077	0.27631	-3.696	0.0002193	0.0019186	ENSG00000100479	14	49643554	49688214	POLE2

Gene.type	Canonical	MANE.Select	ensembl.version
protein_coding	Yes	NM_000786.4	108
protein_coding	Yes	NM_018844.4	108
protein_coding	Yes	NM_017659.4	108
protein_coding	Yes	NM_019015.3	108
protein_coding	Yes	NM_004937.3	108
protein_coding	Yes	NM_001195753.2	108
protein_coding	Yes	NM_002901.4	108
protein_coding	Yes	NM_002067.5	108
protein_coding	Yes	NM_001217.5	108
protein_coding	Yes	NM_021643.4	108
protein_coding	Yes	NM_022119.4	108
protein_coding	Yes	NM_001407.3	108
protein_coding	Yes	NM_004855.5	108
protein_coding	Yes	NM_014177.3	108
protein_coding	Yes	NM_016048.2	108
protein_coding	Yes	NM_020310.3	108
protein_coding	Yes	NM_032523.4	108
protein_coding	Yes	NM_018936.4	108
protein_coding	Yes	NM_030815.3	108
protein_coding	Yes	NM_016816.4	108
protein_coding	Yes	NM_003223.3	108
protein_coding	Yes	NM_032520.5	108
protein_coding	Yes	NM_020378.4	108
protein_coding	Yes	NM_017769.5	108
protein_coding	Yes	NM_015515.5	108
protein_coding	Yes	NM_003243.5	108
protein_coding	Yes	NM_006858.4	108
protein_coding	Yes	NM_004782.4	108
protein_coding	Yes	NM_004175.5	108
protein_coding	Yes	NM_002305.4	108
protein_coding	Yes	NM_006855.4	108
protein_coding	Yes	NM_012265.3	108
protein_coding	Yes	NM_000487.6	108
protein_coding	Yes	NM_175709.5	108
protein_coding	Yes	NM_002676.3	108
protein_coding	Yes	NM_032019.6	108
protein_coding	Yes	NM_002692.4	108

ENST00000216392	30.43492133	1.72883464	0.61718	2.8012	0.0050912	0.0256577	ENSG00000100504	14	50905216	50944483	PYGL
ENST00000216420	143.4160535	-1.393109181	0.31373	-4.441	8.97E-06	0.0001225	ENSG00000100532	14	54509905	54539292	CGRRF1
ENST00000216452	342.693754	1.236181898	0.24111	5.1271	2.94E-07	6.09E-06	ENSG00000100564	14	67589317	67600268	PIGH
ENST00000216465	128.204224	-1.527950213	0.33357	-4.581	4.64E-06	6.89E-05	ENSG00000100577	14	77321035	77331597	GSTZ1
ENST00000216807	149.1161717	-1.986265238	0.31692	-6.267	3.67E-10	1.42E-08	ENSG00000100916	14	35826337	35871963	BRMS1L
ENST00000216832	1364.842247	-1.753640028	0.19284	-9.094	9.58E-20	1.64E-17	ENSG00000100941	14	39175253	39183220	PNN
ENST00000216862	380.9673675	-3.431786461	0.27431	-12.51	6.52E-36	9.58E-33	ENSG00000019186	20	54153445	54173986	CYP24A1
ENST00000216923	337.0921772	1.289604196	0.23978	5.3782	7.52E-08	1.79E-06	ENSG00000020256	20	52151277	52191779	ZFP64
ENST00000217131	71.16606669	-2.65733634	0.45264	-5.871	4.34E-09	1.37E-07	ENSG00000101160	20	58995184	59007254	CTSZ
ENST00000217159	230.4938597	1.404606947	0.33094	4.2443	2.19E-05	0.0002655	ENSG00000101187	20	62642502	62672293	SLCO4A1
ENST00000217909	214.8943149	-2.57878148	0.29423	-8.764	1.88E-18	2.76E-16	ENSG00000077713	X	119399335	119454478	SLC25A43
ENST00000217971	790.3288772	-1.030788189	0.19838	-5.196	2.04E-07	4.37E-06	ENSG00000101856	X	119236284	119244466	PGRMC1
ENST00000218104	220.0491661	1.328700786	0.27025	4.9165	8.81E-07	1.59E-05	ENSG00000101986	X	153724855	153744755	ABCD1
ENST00000218176	31.02490732	3.18804469	0.78647	4.0536	5.04E-05	0.0005478	ENSG00000102057	X	48961379	48971844	KCND1
ENST00000218516	504.245868	1.381973246	0.24349	5.6757	1.38E-08	3.92E-07	ENSG00000102393	X	101397802	101407925	GLA
ENST00000219091	172.6858076	-1.503753856	0.31035	-4.845	1.26E-06	2.19E-05	ENSG00000122386	16	3112585	3120517	ZNF205
ENST00000219150	89.70106232	-1.317403495	0.3776	-3.489	0.0004851	0.0037426	ENSG00000102879	16	30183601	30189076	CORO1A
ENST00000219204	555.3031618	-1.189330736	0.21259	-5.594	2.21E-08	5.95E-07	ENSG00000102931	16	57245258	57253635	ARL2BP
ENST00000219207	11.24910983	-5.500397433	1.70317	-3.23	0.00124	0.0081444	ENSG00000102934	16	57256096	57284672	PLLP
ENST00000219240	91.39879805	-1.107882599	0.37924	-2.921	0.0034857	0.0188691	ENSG00000102967	16	72008743	72027659	DHODH
ENST00000219299	71.6854462	-1.297445518	0.41216	-3.148	0.0016443	0.0102617	ENSG00000103021	16	58249935	58283836	CCDC113
ENST00000219334	15.57068044	4.285644732	1.1324	3.7846	0.000154	0.0014194	ENSG00000103056	16	68358326	68448508	SMPD3
ENST00000219479	261.4248073	-1.735088381	0.26843	-6.464	1.02E-10	4.48E-09	ENSG00000103202	16	397198	400754	NME4
ENST00000220509	968.9468255	1.312703511	0.24069	5.4538	4.93E-08	1.22E-06	ENSG00000104142	15	40894449	40903975	VPS18
ENST00000220584	818.5915495	-1.122249443	0.19872	-5.647	1.63E-08	4.53E-07	ENSG00000079459	8	11802740	11839298	FDFT1
ENST00000220597	322.410277	-2.210533231	0.25439	-8.69	3.64E-18	5.18E-16	ENSG00000076641	8	80967809	81112068	PAG1
ENST00000220616	54.35498062	4.212836111	0.61313	6.871	6.38E-12	3.67E-10	ENSG00000042832	8	132866957	133134899	TG
ENST00000220659	374.0783089	1.229301556	0.2428	5.063	4.13E-07	8.21E-06	ENSG00000104221	8	37843267	37849861	BRF2
ENST00000220809	115.1288382	-2.442476411	0.41802	-5.843	5.13E-09	1.60E-07	ENSG00000104368	8	42174717	42207565	PLAT
ENST00000221086	324.4693835	-1.173793545	0.2566	-4.574	4.78E-06	7.07E-05	ENSG00000104643	8	11284815	11328146	MTMR9
ENST00000221138	395.1912593	-1.27293005	0.23172	-5.493	3.94E-08	9.98E-07	ENSG00000104695	8	30785615	30812818	PPP2CB
ENST00000221200	454.9167285	-1.04564819	0.25001	-4.182	2.88E-05	0.0003367	ENSG00000104756	8	25427846	25458433	KCTD9
ENST00000221315	799.4961588	1.014275596	0.19972	5.0785	3.80E-07	7.64E-06	ENSG00000256087	19	52031377	52048838	ZNF432
ENST00000221418	131.3535326	-1.616393225	0.33477	-4.828	1.38E-06	2.37E-05	ENSG00000104823	19	38815421	38831794	ECH1
ENST00000221452	242.682515	2.788264792	0.3019	9.2358	2.56E-20	4.62E-18	ENSG00000104856	19	45001463	45038192	RELB
ENST00000221466	230.8668283	1.569811511	0.27885	5.6295	1.81E-08	4.97E-07	ENSG00000104870	19	49512660	49526428	FCGRT
ENST00000221543	207.5181092	1.860019485	0.28818	6.4544	1.09E-10	4.75E-09	ENSG00000104946	19	49877701	49888750	TBC1D17
ENST00000221856	31.36527134	-3.909954824	0.75466	-5.181	2.21E-07	4.70E-06	ENSG00000105255	19	4304597	4323836	FSD1
ENST00000221930	33.36590215	-2.379034535	0.6134	-3.878	0.0001051	0.0010221	ENSG00000105329	19	41330322	41353922	TGFB1
ENST00000221980	136.9459117	1.524361737	0.3574	4.2652	2.00E-05	0.0002447	ENSG00000105376	19	10289951	10296778	ICAM5

protein_coding	Yes	NM_002863.5	108
protein_coding	Yes	NM_006568.3	108
protein_coding	Yes	NM_004569.5	108
protein_coding	Yes	NM_145870.3	108
protein_coding	Yes	NM_032352.4	108
protein_coding	Yes	NM_002687.4	108
protein_coding	Yes	NM_000782.5	108
protein_coding	Yes	NM_018197.3	108
protein_coding	Yes	NM_001336.4	108
protein_coding	Yes	NM_016354.4	108
protein_coding	Yes	NM_145305.3	108
protein_coding	Yes	NM_006667.5	108
protein_coding	Yes	NM_000033.4	108
protein_coding	Yes	NM_004979.6	108
protein_coding	Yes	NM_000169.3	108
protein_coding	Yes	NM_001042428.2	108
protein_coding	Yes	NM_007074.4	108
protein_coding	Yes	NM_012106.4	108
protein_coding	Yes	NM_015993.3	108
protein_coding	Yes	NM_001361.5	108
protein_coding	Yes	NM_014157.4	108
protein_coding	Yes	NM_018667.4	108
protein_coding	Yes	NM_005009.3	108
protein_coding	Yes	NM_020857.3	108
protein_coding	Yes	NM_004462.5	108
protein_coding	Yes	NM_018440.4	108
protein_coding	Yes	NM_003235.5	108
protein_coding	Yes	NM_018310.4	108
protein_coding	Yes	NM_000930.5	108
protein_coding	Yes	NM_015458.4	108
protein_coding	Yes	NM_001009552.2	108
protein_coding	Yes	NM_017634.4	108
protein_coding	Yes	NM_014650.4	108
protein_coding	Yes	NM_001398.3	108
protein_coding	Yes	NM_006509.4	108
protein_coding	Yes	NM_001136019.3	108
protein_coding	Yes	NM_024682.3	108
protein_coding	Yes	NM_024333.3	108
protein_coding	Yes	NM_000660.7	108
protein_coding	Yes	NM_003259.4	108

ENST00000222120	288.3818489	-2.53796352	0.28169	-9.01	2.06E-19	3.42E-17	ENSG00000105514	19	11322067	11339657	RAB3D
ENST00000222122	324.6955051	1.87867223	0.30048	6.2523	4.05E-10	1.55E-08	ENSG00000105516	19	48630029	48637379	DBP
ENST00000222139	223.3223509	1.531196075	0.30804	4.9708	6.67E-07	1.25E-05	ENSG00000187266	19	11377206	11384314	EPOR
ENST00000222145	237.2726857	1.081072964	0.33405	3.2362	0.0012112	0.0079889	ENSG00000105538	19	48720584	48740610	RASIP1
ENST00000222248	11.88150312	5.529182456	1.68479	3.2818	0.0010314	0.006988	ENSG00000105641	19	17871944	17895174	SLC5A5
ENST00000222543	48.85738043	-1.890536889	0.57251	-3.302	0.0009593	0.0065792	ENSG00000105825	7	93885395	93890753	TFPI2
ENST00000222573	2105.223525	2.078553466	0.18442	11.271	1.83E-29	1.06E-26	ENSG00000105855	7	20330899	20415754	ITGB8
ENST00000222673	1627.036969	1.112527302	0.19329	5.7557	8.63E-09	2.56E-07	ENSG00000105953	7	44606626	44709066	OGDH
ENST00000222693	974.0576691	-1.835197599	0.20992	-8.742	2.28E-18	3.32E-16	ENSG00000105971	7	116499737	116508541	CAV2
ENST00000222990	1181.806912	1.162702561	0.19117	6.082	1.19E-09	4.21E-08	ENSG00000106266	7	2251769	2314441	SNX8
ENST00000223073	473.6518672	-1.036659203	0.22182	-4.673	2.96E-06	4.65E-05	ENSG00000106344	7	128297684	128343908	RBM28
ENST00000223095	37.61577132	1.674733166	0.65904	2.5412	0.0110486	0.0473393	ENSG00000106366	7	101127103	101139247	SERPINE1
ENST00000223129	876.8601041	-1.073751616	0.19672	-5.458	4.81E-08	1.19E-06	ENSG00000106399	7	7636517	7718607	RPA3
ENST00000223145	179.2167674	-1.255294614	0.30736	-4.084	4.42E-05	0.0004891	ENSG00000106415	7	7968795	8089080	GLCCI1
ENST00000223210	163.1784711	1.16939069	0.39995	2.9239	0.0034571	0.0187506	ENSG00000106479	7	149838374	149867479	ZNF862
ENST00000223215	450.8213788	-2.692430506	0.24816	-10.85	2.00E-27	8.98E-25	ENSG00000106484	7	130492084	130506465	MEST
ENST00000223336	208.7796248	-1.957959085	0.28093	-6.969	3.18E-12	1.94E-10	ENSG00000106603	7	43639256	43729523	COA1
ENST00000223398	80.97912987	-2.232155468	0.4276	-5.22	1.79E-07	3.89E-06	ENSG00000106665	7	74289406	74405935	CLIP2
ENST00000224784	8.553731298	-5.095271988	1.7595	-2.896	0.0037812	0.0201516	ENSG00000107796	10	88935073	88952773	ACTA2
ENST00000225276	7.449567885	-4.867302743	1.85845	-2.619	0.0088185	0.0396945	ENSG00000070731	17	76565376	76585860	ST6GALNAC2
ENST00000226091	3.810374571	-5.403736545	2.13911	-2.526	0.0115316	0.0489523	ENSG00000108947	17	7705201	7711372	EFNB3
ENST00000226253	46.46405987	1.307316477	0.50078	2.6106	0.0090393	0.0404757	ENSG00000109107	17	28573119	28576895	ALDOC
ENST00000227135	108.7244302	-2.057346565	0.36715	-5.604	2.10E-08	5.68E-07	ENSG00000064199	11	124673903	124697518	SPA17
ENST00000227451	460.3133369	-2.067624123	0.28843	-7.168	7.58E-13	5.24E-11	ENSG00000110042	11	59172131	59208588	DTX4
ENST00000227520	369.4325392	1.466833233	0.32586	4.5015	6.75E-06	9.56E-05	ENSG00000110104	11	60842112	60851081	CCDC86
ENST00000228027	20.05174495	-2.044149911	0.7593	-2.692	0.0070991	0.0333913	ENSG00000062282	11	75768777	75801534	DGAT2
ENST00000228136	1674.110684	-1.082123143	0.18486	-5.854	4.81E-09	1.51E-07	ENSG00000110696	11	16738646	16758340	C11orf58
ENST00000228510	359.8666171	-1.303324245	0.24672	-5.283	1.27E-07	2.86E-06	ENSG00000110921	12	109573793	109598125	MVK
ENST00000228515	191.9129263	-1.176947456	0.31279	-3.763	0.0001681	0.0015304	ENSG00000110925	12	51061204	51083596	CSRNP2
ENST00000228534	61.93527816	3.082519443	0.50481	6.1063	1.02E-09	3.66E-08	ENSG00000110944	12	56338883	56340410	IL23A
ENST00000228799	122.7411943	1.128832579	0.34094	3.3109	0.0009298	0.0064119	ENSG00000111203	12	2812667	2825076	ITFG2
ENST00000228865	595.9532498	-2.104230173	0.24102	-8.731	2.53E-18	3.66E-16	ENSG00000111269	12	12611875	12645108	CREBL2
ENST00000228872	854.7000736	-1.564275753	0.20569	-7.605	2.85E-14	2.46E-12	ENSG00000111276	12	12717367	12722369	CDKN1B
ENST00000228916	275.2866301	-3.186064429	0.33868	-9.407	5.09E-21	1.02E-18	ENSG00000111319	12	6346846	6375563	SCNN1A
ENST00000228945	281.1667953	-1.908440539	0.25665	-7.436	1.04E-13	8.26E-12	ENSG00000111348	12	14942030	14961601	ARHGDIB
ENST00000229264	15.94487534	7.42191278	1.63839	4.53	5.90E-06	8.50E-05	ENSG00000111664	12	6840924	6847393	GNB3
ENST00000229266	123.8392548	-1.752268278	0.33543	-5.224	1.75E-07	3.82E-06	ENSG00000111666	12	101697639	101729074	CHPT1
ENST00000229281	763.5972516	1.008425918	0.19919	5.0625	4.14E-07	8.23E-06	ENSG00000111678	12	6944021	6946002	C12orf57
ENST00000229330	277.5750854	-1.280606188	0.25616	-4.999	5.76E-07	1.10E-05	ENSG00000111727	12	104064530	104106524	HCFC2
ENST00000229595	375.2560339	-1.269831564	0.25069	-5.065	4.08E-07	8.12E-06	ENSG00000111875	6	118894151	118909171	ASF1A

protein_coding	Yes	NM_004283.4	108
protein_coding	Yes	NM_001352.5	108
protein_coding	Yes	NM_000121.4	108
protein_coding	Yes	NM_017805.3	108
protein_coding	Yes	NM_000453.3	108
protein_coding	Yes	NM_006528.4	108
protein_coding	Yes	NM_002214.3	108
protein_coding	Yes	NM_002541.4	108
protein_coding	Yes	NM_001233.5	108
protein_coding	Yes	NM_013321.4	108
protein_coding	Yes	NM_018077.3	108
protein_coding	Yes	NM_000602.5	108
protein_coding	Yes	NM_002947.5	108
protein_coding	Yes	NM_138426.4	108
protein_coding	Yes	NM_001099220.3	108
protein_coding	Yes	NM_002402.4	108
protein_coding	Yes	NM_018224.4	108
protein_coding	Yes	NM_003388.5	108
protein_coding	Yes	NM_001613.4	108
protein_coding	Yes	NM_006456.3	108
protein_coding	Yes	NM_001406.4	108
protein_coding	Yes	NM_005165.3	108
protein_coding	Yes	NM_017425.4	108
protein_coding	Yes	NM_015177.2	108
protein_coding	Yes	NM_024098.4	108
protein_coding	Yes	NM_032564.5	108
protein_coding	Yes	NM_014267.6	108
protein_coding	Yes	NM_000431.4	108
protein_coding	Yes	NM_030809.3	108
protein_coding	Yes	NM_016584.3	108
protein_coding	Yes	NM_018463.4	108
protein_coding	Yes	NM_001310.4	108
protein_coding	Yes	NM_004064.5	108
protein_coding	Yes	NM_001038.6	108
protein_coding	Yes	NM_001175.7	108
protein_coding	Yes	NM_002075.4	108
protein_coding	Yes	NM_020244.3	108
protein_coding	Yes	NM_138425.4	108
protein_coding	Yes	NM_013320.3	108
protein_coding	Yes	NM_014034.3	108

ENST00000229758	421.3312962	-2.043100065	0.24714	-8.267	1.37E-16	1.60E-14	ENSG00000112029	6	152970534	152983041	FBXO5
ENST00000229769	171.7547629	-1.305805831	0.29682	-4.399	1.09E-05	0.0001445	ENSG00000112039	6	35452337	35467102	FANCE
ENST00000230792	270.5818314	-2.012097257	0.31972	-6.293	3.11E-10	1.22E-08	ENSG00000112874	5	103548854	103562789	NUDT12
ENST00000230895	373.1923654	1.195648669	0.23101	5.1758	2.27E-07	4.82E-06	ENSG00000112977	5	10679229	10761234	DAP
ENST00000231198	152.9419022	-2.019066938	0.31401	-6.43	1.28E-10	5.48E-09	ENSG00000113272	5	157731419	157741449	THG1L
ENST00000231484	7.041538405	6.244278276	1.85047	3.3744	0.0007397	0.0053257	ENSG00000113555	5	141943580	141958202	PCDH12
ENST00000231948	460.6146961	-1.030691503	0.22494	-4.582	4.60E-06	6.84E-05	ENSG00000113851	3	3150010	3179691	CRBN
ENST00000232854	89.89828557	-1.129487504	0.39501	-2.859	0.004245	0.0221575	ENSG00000114735	3	50569521	50596166	HEMK1
ENST00000232975	129.9287304	1.483217733	0.34079	4.3523	1.35E-05	0.0001745	ENSG00000114854	3	52451099	52454041	TNNC1
ENST00000233072	15.36788849	-4.326484236	1.13398	-3.815	0.000136	0.0012752	ENSG00000021826	2	210556598	210679107	CPS1
ENST00000233084	1324.204917	-1.320756331	0.18836	-7.012	2.35E-12	1.49E-10	ENSG00000079785	2	15591867	15631101	DDX1
ENST00000233114	1507.771086	-1.653149277	0.18351	-9.008	2.09E-19	3.46E-17	ENSG00000014641	2	63588962	63607197	MDH1
ENST00000233143	4549.571368	-1.606044038	0.18492	-8.685	3.78E-18	5.38E-16	ENSG00000034510	2	84905655	84906671	TMSB10
ENST00000233336	392.5767184	-1.165074458	0.23464	-4.965	6.85E-07	1.28E-05	ENSG00000114999	2	112482155	112541739	TTL
ENST00000233809	4.536014061	-5.656442023	2.03958	-2.773	0.0055484	0.027444	ENSG00000115457	2	216633419	216664436	IGFBP2
ENST00000233957	34.68525089	-3.297497614	0.73528	-4.485	7.30E-06	0.0001025	ENSG00000115604	2	102355795	102398776	IL18R1
ENST00000234256	101.4157552	-1.138173346	0.37258	-3.055	0.0022517	0.0132531	ENSG00000115902	2	64989398	65023865	SLC1A4
ENST00000235307	70.68574809	-8.172038521	1.48225	-5.513	3.52E-08	9.03E-07	ENSG00000116667	1	184387028	184629019	C1orf21
ENST00000236147	11.24996959	-6.964873858	1.74054	-4.002	6.29E-05	0.0006634	ENSG00000188404	1	169690666	169711620	SELL
ENST00000237455	652.8364509	1.296959658	0.22436	5.7808	7.44E-09	2.25E-07	ENSG00000239305	2	86603397	86623865	RNF103
ENST00000237596	290.2974042	-2.439562364	0.2587	-9.43	4.10E-21	8.37E-19	ENSG00000118762	4	88007634	88077777	PKD2
ENST00000237612	116.0620253	-5.412151873	0.59015	-9.171	4.70E-20	8.18E-18	ENSG00000118777	4	88090268	88158639	ABCG2
ENST00000237642	111.9464867	-1.061439899	0.35033	-3.03	0.0024469	0.0141694	ENSG00000118804	4	76306732	76311130	STBD1
ENST00000237889	456.0477112	-1.024155764	0.2218	-4.618	3.88E-06	5.88E-05	ENSG00000119013	2	201072000	201085750	NDUFB3
ENST00000238081	5231.825753	-1.331874177	0.16729	-7.961	1.70E-15	1.73E-13	ENSG00000134308	2	9583966	9630997	YWHAQ
ENST00000238714	352.9773875	-1.044910406	0.24253	-4.308	1.64E-05	0.0002068	ENSG00000115421	2	60756267	60802086	PAPOLG
ENST00000238721	151.7272372	-1.533657061	0.32027	-4.789	1.68E-06	2.82E-05	ENSG00000115129	2	24077432	24084834	TP53I3
ENST00000239223	123.4807435	1.081875155	0.33003	3.2781	0.001045	0.0070635	ENSG00000120129	5	172768095	172771195	DUSP1
ENST00000239444	26.41253221	-6.745994262	1.55171	-4.347	1.38E-05	0.0001778	ENSG00000120322	5	141177789	141180539	PCDHB8
ENST00000239449	45.38089237	-2.093922581	0.58583	-3.574	0.0003512	0.0028468	ENSG00000120327	5	141223342	141227759	PCDHB14
ENST00000240093	430.1668297	-1.570711569	0.29072	-5.403	6.56E-08	1.58E-06	ENSG00000104290	8	28494211	28574258	FZD3
ENST00000240123	24.23948016	-1.884571194	0.70887	-2.659	0.0078473	0.0361018	ENSG00000120896	8	22551926	22575788	SORBS3
ENST00000240651	242.1290663	1.216769994	0.27122	4.4862	7.25E-06	0.0001018	ENSG00000121350	12	21437654	21471250	PYROXD1
ENST00000241052	401.6744908	-1.024736973	0.28581	-3.585	0.0003366	0.0027494	ENSG00000121691	11	34438933	34472060	CAT
ENST00000241071	15.28177591	2.323884504	0.90165	2.5774	0.0099557	0.0436831	ENSG00000106336	7	100586327	100601117	FBXO24
ENST00000241125	58.89178377	1.821129394	0.53098	3.4297	0.0006042	0.004491	ENSG00000121743	13	20138254	20161052	GJA3
ENST00000241453	4.218300497	5.501896295	2.08875	2.6341	0.0084371	0.0383068	ENSG00000122025	13	28003273	28100576	FLT3
ENST00000241463	128.6858945	1.28624335	0.35853	3.5875	0.0003338	0.0027296	ENSG00000122035	13	27270829	27275192	RASL11A
ENST00000241502	1728.211194	-1.002864441	0.19571	-5.124	2.99E-07	6.18E-06	ENSG00000122068	3	197749882	197787596	FYTTD1
ENST00000242351	2043.552267	1.040740832	0.22158	4.6969	2.64E-06	4.21E-05	ENSG00000105939	7	139043514	139109720	ZC3HAV1

protein_coding	Yes	NM_012177.5	108
protein_coding	Yes	NM_021922.3	108
protein_coding	Yes	NM_031438.4	108
protein_coding	Yes	NM_004394.3	108
protein_coding	Yes	NM_017872.5	108
protein_coding	Yes	NM_016580.4	108
protein_coding	Yes	NM_016302.4	108
protein_coding	Yes	NM_016173.5	108
protein_coding	Yes	NM_003280.3	108
protein_coding	Yes	NM_001875.5	108
protein_coding	Yes	NM_004939.3	108
protein_coding	Yes	NM_005917.4	108
protein_coding	Yes	NM_021103.4	108
protein_coding	Yes	NM_153712.5	108
protein_coding	Yes	NM_000597.3	108
protein_coding	Yes	NM_003855.5	108
protein_coding	Yes	NM_003038.5	108
protein_coding	Yes	NM_030806.4	108
protein_coding	Yes	NM_000655.5	108
protein_coding	Yes	NM_005667.4	108
protein_coding	Yes	NM_000297.4	108
protein_coding	Yes	NM_004827.3	108
protein_coding	Yes	NM_003943.5	108
protein_coding	Yes	NM_002491.3	108
protein_coding	Yes	NM_006826.4	108
protein_coding	Yes	NM_022894.4	108
protein_coding	Yes	NM_004881.5	108
protein_coding	Yes	NM_004417.4	108
protein_coding	Yes	NM_019120.5	108
protein_coding	Yes	NM_018934.4	108
protein_coding	Yes	NM_017412.4	108
protein_coding	Yes	NM_005775.5	108
protein_coding	Yes	NM_024854.5	108
protein_coding	Yes	NM_001752.4	108
protein_coding	Yes	NM_033506.3	108
protein_coding	Yes	NM_021954.4	108
protein_coding	Yes	NM_004119.3	108
protein_coding	Yes	NM_206827.2	108
protein_coding	Yes	NM_032288.7	108
protein_coding	Yes	NM_020119.4	108

ENST00000242577	1264.215333	-1.894706805	0.20094	-9.429	4.14E-21	8.43E-19	ENSG00000088986	12	120496112	120498493	DYNLL1
ENST00000242728	22.8832007	4.451281631	1.00991	4.4076	1.05E-05	0.0001396	ENSG00000123095	12	26120029	26125037	BHLHE41
ENST00000242827	192.2432279	-1.042369105	0.28383	-3.672	0.0002402	0.0020725	ENSG00000123179	13	49660673	49691486	EBPL
ENST00000243167	57.7234258	-1.559364774	0.45838	-3.402	0.0006691	0.0048955	ENSG00000117480	1	46394316	46413845	FAAH
ENST00000243349	126.441568	-3.24974303	0.42793	-7.594	3.10E-14	2.66E-12	ENSG00000123612	2	157526766	157628864	ACVR1C
ENST00000243563	504.6101753	-1.219292114	0.22037	-5.533	3.15E-08	8.16E-07	ENSG00000077312	19	40751202	40765389	SNRPA
ENST00000243643	7.041538405	6.244278276	1.85047	3.3744	0.0007397	0.0053257	ENSG00000170954	19	53107878	53132910	ZNF415
ENST00000243673	26.62970088	3.252953652	0.74975	4.3387	1.43E-05	0.000184	ENSG00000123901	11	94377315	94401419	GPR83
ENST00000243776	1555.874438	1.243063956	0.18012	6.9011	5.16E-12	3.02E-10	ENSG00000123989	2	219538953	219543809	CHPF
ENST00000244061	3396.475096	1.022111619	0.17912	5.7064	1.15E-08	3.33E-07	ENSG00000124226	20	49936396	49953885	RNF114
ENST00000244519	117.303698	2.29656968	0.39269	5.8484	4.96E-09	1.55E-07	ENSG00000111801	6	26440503	26453415	BTN3A3
ENST00000244534	2116.530129	-1.475907574	0.20377	-7.243	4.39E-13	3.17E-11	ENSG00000124575	6	26234211	26234987	H1-3
ENST00000244565	39.32285879	2.521702341	0.58389	4.3188	1.57E-05	0.0001988	ENSG00000124602	6	41026894	41039221	UNC5CL
ENST00000244571	366.9695134	-1.175236254	0.26189	-4.488	7.20E-06	0.0001013	ENSG00000124608	6	44298730	44313347	AARS2
ENST00000244741	316.0388704	-1.084940818	0.24909	-4.356	1.33E-05	0.0001721	ENSG00000124762	6	36678713	36687332	CDKN1A
ENST00000245157	559.5279399	-1.473672304	0.21627	-6.814	9.49E-12	5.26E-10	ENSG00000125124	16	56484384	56520024	BBS2
ENST00000245185	234.8321904	-3.968889178	1.54679	-2.566	0.0102912	0.0448538	ENSG00000125148	16	56608583	56609497	MT2A
ENST00000245206	709.5657749	-1.898234571	0.21036	-9.024	1.82E-19	3.04E-17	ENSG00000125166	16	58707130	58734316	GOT2
ENST00000245222	149.5663603	1.969600889	0.3314	5.9434	2.79E-09	9.20E-08	ENSG00000063176	19	48619505	48630405	SPHK2
ENST00000245451	277.0667062	2.440348422	0.33391	7.3083	2.70E-13	2.03E-11	ENSG00000125378	14	53949735	53956825	BMP4
ENST00000245817	202.4470255	2.638677503	0.2976	8.8664	7.56E-19	1.15E-16	ENSG00000125657	19	6531025	6535924	TNFSF9
ENST00000245838	1223.984379	-1.085879359	0.18807	-5.774	7.75E-09	2.33E-07	ENSG00000125676	X	123600568	123733052	THOC2
ENST00000245907	106.8324321	2.641353793	0.43132	6.1238	9.14E-10	3.30E-08	ENSG00000125730	19	6677703	6720650	C3
ENST00000246151	876.1890365	1.153697414	0.25004	4.614	3.95E-06	5.98E-05	ENSG00000057757	1	23778417	23788232	PITHD1
ENST00000246166	189.826132	-1.183330257	0.32366	-3.656	0.0002561	0.0021917	ENSG00000257365	14	64986894	65062650	FNTB
ENST00000246190	396.5948857	1.10046892	0.22959	4.7933	1.64E-06	2.77E-05	ENSG00000125967	20	33657086	33674428	NECAB3
ENST00000246672	49.19509519	3.212723015	0.55823	5.7552	8.65E-09	2.57E-07	ENSG00000126368	17	40092792	40100589	NR1D1
ENST00000246802	1003.848691	1.080306226	0.19442	5.5566	2.75E-08	7.23E-07	ENSG00000105373	19	47745545	47757058	NOP53
ENST00000247026	139.8925547	-1.610188604	0.32723	-4.921	8.62E-07	1.56E-05	ENSG00000126653	17	30116815	30186475	NSRP1
ENST00000247138	395.998242	1.204727372	0.23095	5.2164	1.82E-07	3.96E-06	ENSG00000102100	X	48903182	48911646	SLC35A2
ENST00000247191	2205.260842	-1.94034996	0.20138	-9.635	5.68E-22	1.29E-19	ENSG00000126787	14	55148134	55191585	DLGAP5
ENST00000247225	1291.286959	-1.210753586	0.21312	-5.681	1.34E-08	3.80E-07	ENSG00000126821	14	63684215	63728065	SGPP1
ENST00000247291	416.7917074	-1.220954816	0.22834	-5.347	8.94E-08	2.08E-06	ENSG00000126878	9	131096530	131123144	AIF1L
ENST00000247470	50.92611486	-3.689836406	0.57862	-6.377	1.81E-10	7.50E-09	ENSG00000103490	16	31201485	31202760	PYCARD
ENST00000247706	409.5485477	1.103170696	0.24874	4.4351	9.20E-06	0.0001252	ENSG00000127220	19	17292130	17303425	ABHD8
ENST00000248151	403.0288884	-1.067385575	0.24545	-4.349	1.37E-05	0.0001769	ENSG00000127589	8	30352007	30353342	TUBBP1
ENST00000248248	515.8365073	1.281723195	0.25581	5.0105	5.43E-07	1.04E-05	ENSG00000103111	16	77191189	77202398	MON1B
ENST00000248484	564.8257306	-1.391427686	0.2259	-6.159	7.30E-10	2.69E-08	ENSG00000127863	13	23570411	23676093	TNFRSF19
ENST00000248846	766.2477522	1.120155715	0.19982	5.6058	2.07E-08	5.62E-07	ENSG00000128159	22	50217693	50245023	TUBGCP6
ENST00000249071	31.33884913	-1.779253145	0.60685	-2.932	0.0033685	0.0183823	ENSG00000128340	22	37225269	37244269	RAC2

protein_coding	Yes	NM_003746.3	108
protein_coding	Yes	NM_030762.3	108
protein_coding	Yes	NM_032565.5	108
protein_coding	Yes	NM_001441.3	108
protein_coding	Yes	NM_145259.3	108
protein_coding	Yes	NM_004596.5	108
protein_coding	Yes	NM_018355.4	108
protein_coding	Yes	NM_016540.4	108
protein_coding	Yes	NM_024536.6	108
protein_coding	Yes	NM_018683.4	108
protein_coding	Yes	NM_006994.5	108
protein_coding	Yes	NM_005320.3	108
protein_coding	Yes	NM_173561.3	108
protein_coding	Yes	NM_020745.4	108
protein_coding	Yes	NM_000389.5	108
protein_coding	Yes	NM_031885.5	108
protein_coding	Yes	NM_005953.5	108
protein_coding	Yes	NM_002080.4	108
protein_coding	Yes	NM_020126.5	108
protein_coding	Yes	NM_001202.6	108
protein_coding	Yes	NM_003811.4	108
protein_coding	Yes	NM_001081550.2	108
protein_coding	Yes	NM_000064.4	108
protein_coding	Yes	NM_020362.5	108
protein_coding	Yes	NM_002028.4	108
protein_coding	Yes	NM_031232.4	108
protein_coding	Yes	NM_021724.5	108
protein_coding	Yes	NM_015710.5	108
protein_coding	Yes	NM_032141.4	108
protein_coding	Yes	NM_005660.3	108
protein_coding	Yes	NM_014750.5	108
protein_coding	Yes	NM_030791.4	108
protein_coding	Yes	NM_031426.4	108
protein_coding	Yes	NM_013258.5	108
protein_coding	Yes	NM_024527.5	108
ped_processed_pse	Yes	-	108
protein_coding	Yes	NM_014940.4	108
protein_coding	Yes	NM_148957.4	108
protein_coding	Yes	NM_020461.4	108
protein_coding	Yes	NM_002872.5	108

ENST00000249330	109.9254588	3.669461912	0.48927	7.4998	6.39E-14	5.27E-12	ENSG00000128564	7	101162508	101165569	VGF
ENST00000249700	102.4770438	-3.973295555	0.49295	-8.06	7.61E-16	8.13E-14	ENSG00000128872	15	51751596	51816363	TMOD2
ENST00000249861	56.26104919	1.255488567	0.46579	2.6954	0.0070311	0.0331578	ENSG00000129028	15	70881341	70892433	THAP10
ENST00000250024	266.6127697	-1.322233387	0.26052	-5.075	3.87E-07	7.75E-06	ENSG00000129173	11	19224062	19240945	E2F8
ENST00000250263	747.0196743	-1.264249002	0.20746	-6.094	1.10E-09	3.93E-08	ENSG00000104626	8	9002896	9033338	ERI1
ENST00000250383	1039.621364	2.577822373	0.19536	13.195	9.36E-40	1.83E-36	ENSG00000100867	14	23636363	23645639	DHRS2
ENST00000251170	47.26811218	1.961943251	0.53224	3.6862	0.0002276	0.0019798	ENSG00000103184	16	4958329	5019157	SEC14L5
ENST00000251363	127.8688614	-3.109110821	0.4425	-7.026	2.12E-12	1.36E-10	ENSG00000090661	19	8209369	8262421	CERS4
ENST00000251453	511.6959134	2.530212528	0.24391	10.373	3.27E-25	1.09E-22	ENSG00000105193	19	39433136	39435949	RPS16
ENST00000251496	2241.621164	-1.176664099	0.18308	-6.427	1.30E-10	5.57E-09	ENSG00000109805	4	17810978	17844865	NCAPG
ENST00000251642	106.4743408	1.621158799	0.36919	4.3911	1.13E-05	0.0001492	ENSG00000108771	17	42101410	42112714	DHX58
ENST00000251822	95.68454292	-1.631682922	0.37916	-4.303	1.68E-05	0.0002108	ENSG00000008853	8	22999547	23020199	RHOBTB2
ENST00000251900	76.27396176	-1.672063594	0.4263	-3.922	8.77E-05	0.0008813	ENSG00000102098	X	18239312	18354688	SCML2
ENST00000251973	271.2667655	-1.374424578	0.2709	-5.074	3.90E-07	7.81E-06	ENSG00000100065	22	37490361	37519415	CARD10
ENST00000252136	1755.68248	1.013256327	0.23611	4.2915	1.77E-05	0.0002209	ENSG00000099899	22	20111874	20117226	TRMT2A
ENST00000252242	232.1469741	2.440916308	0.92038	2.6521	0.0079998	0.0367039	ENSG00000186081	12	52514574	52520394	KRT5
ENST00000252288	13.34626208	-3.676069614	1.09793	-3.348	0.0008135	0.0057556	ENSG00000130005	19	1397025	1401542	GAMT
ENST00000252505	7.064360679	6.248469536	1.84774	3.3817	0.0007204	0.0052065	ENSG00000151360	2	3658199	3702671	ALLC
ENST00000252506	90.76137929	2.420510015	0.44706	5.4143	6.15E-08	1.49E-06	ENSG00000130222	9	89605011	89606555	GADD45G
ENST00000252590	4.149833674	5.480651158	2.08437	2.6294	0.0085535	0.0387013	ENSG00000130300	19	17351454	17377342	PLVAP
ENST00000252826	103.6269649	-1.302225644	0.36331	-3.584	0.0003379	0.0027586	ENSG00000130529	19	49157791	49211836	TRPM4
ENST00000252945	25.49876792	2.110317197	0.68294	3.09	0.0020013	0.0120251	ENSG00000130649	10	133527362	133539123	CYP2E1
ENST00000253079	43.60673913	1.868739425	0.53012	3.5251	0.0004233	0.0033346	ENSG00000130783	12	122774571	122827528	CCDC62
ENST00000253122	152.8097013	1.884973101	0.31326	6.0172	1.77E-09	6.11E-08	ENSG00000130821	X	153687925	153696588	SLC6A8
ENST00000253193	233.2349627	-1.417431706	0.2786	-5.088	3.63E-07	7.33E-06	ENSG00000130881	19	33194329	33208864	LRP3
ENST00000253255	9.694729938	6.702286926	1.76618	3.7948	0.0001478	0.0013686	ENSG00000130943	22	46255662	46263343	PKDREJ
ENST00000253413	3204.373529	1.034350505	0.23173	4.4637	8.06E-06	0.0001116	ENSG00000131100	22	17592135	17628749	ATP6V1E1
ENST00000253462	333.669073	-1.113210162	0.27246	-4.086	4.39E-05	0.000486	ENSG00000131153	16	85676197	85688954	GINS2
ENST00000253697	19.0392568	2.253966268	0.80295	2.8071	0.0049991	0.0252795	ENSG00000131379	3	14675140	14773036	C3orf20
ENST00000253799	66.23163218	1.42512803	0.43202	3.2988	0.0009711	0.0066479	ENSG00000131480	17	42844579	42850707	AOC2
ENST00000254043	127.518697	1.539346601	0.41025	3.7522	0.0001753	0.0015853	ENSG00000171346	17	41513744	41518890	KRT15
ENST00000254101	304.9366856	-1.332098317	0.26219	-5.081	3.76E-07	7.57E-06	ENSG00000131791	1	147155105	147172470	PRKAB2
ENST00000254190	275.4274258	-1.840609008	0.26872	-6.85	7.41E-12	4.20E-10	ENSG00000131873	15	101175726	101252048	CHSY1
ENST00000254286	581.5262965	-1.126504439	0.21092	-5.341	9.25E-08	2.15E-06	ENSG00000131966	14	58200148	58235636	ACTR10
ENST00000254579	148.3349785	1.51749003	0.35912	4.2256	2.38E-05	0.0002853	ENSG00000179532	11	6497279	6572020	DNHD1
ENST00000254661	6.803025208	6.189760723	1.92278	3.2192	0.0012856	0.0083938	ENSG00000132329	2	237859622	237912106	RAMP1
ENST00000254691	37.24487072	2.04387266	0.60697	3.3673	0.0007589	0.0054401	ENSG00000132357	5	40841366	40855354	CARD6
ENST00000254695	156.2250071	-2.148991427	0.32033	-6.709	1.96E-11	1.02E-09	ENSG00000132359	17	2796437	3037741	RAP1GAP2
ENST00000254759	73.62800683	-1.9574412	0.43698	-4.48	7.48E-06	0.0001047	ENSG00000132423	6	99369400	99394195	COQ3
ENST00000254806	457.1058553	1.312275848	0.25789	5.0886	3.61E-07	7.29E-06	ENSG00000132471	17	75845698	75855335	WBP2

protein_coding	Yes	NM_003378.4	108
protein_coding	Yes	NM_014548.4	108
protein_coding	Yes	NM_020147.4	108
protein_coding	Yes	NM_024680.4	108
protein_coding	Yes	NM_153332.4	108
protein_coding	Yes	NM_005794.4	108
protein_coding	Yes	NM_014692.2	108
protein_coding	Yes	NM_024552.3	108
protein_coding	Yes	NM_001020.6	108
protein_coding	Yes	NM_022346.5	108
protein_coding	Yes	NM_024119.3	108
protein_coding	Yes	NM_015178.3	108
protein_coding	Yes	NM_006089.3	108
protein_coding	Yes	NM_014550.4	108
protein_coding	Yes	NM_022727.6	108
protein_coding	Yes	NM_000424.4	108
protein_coding	Yes	NM_000156.6	108
protein_coding	Yes	NM_018436.4	108
protein_coding	Yes	NM_006705.4	108
protein_coding	Yes	NM_031310.3	108
protein_coding	Yes	NM_017636.4	108
protein_coding	Yes	NM_000773.4	108
protein_coding	Yes	NM_201435.5	108
protein_coding	Yes	NM_005629.4	108
protein_coding	Yes	NM_002333.4	108
protein_coding	Yes	NM_006071.2	108
protein_coding	Yes	NM_001696.4	108
protein_coding	Yes	NM_016095.3	108
protein_coding	Yes	NM_032137.5	108
protein_coding	Yes	NM_009590.4	108
protein_coding	Yes	NM_002275.4	108
protein_coding	Yes	NM_005399.5	108
protein_coding	Yes	NM_014918.5	108
protein_coding	Yes	NM_018477.3	108
protein_coding	Yes	NM_144666.3	108
protein_coding	Yes	NM_005855.4	108
protein_coding	Yes	NM_032587.4	108
protein_coding	Yes	NM_015085.5	108
protein_coding	Yes	NM_017421.4	108
protein_coding	Yes	NM_012478.4	108

ENST00000254816	333.4536034	1.240777864	0.25522	4.8617	1.16E-06	2.04E-05	ENSG00000132481	17	75874163	75878581	TRIM47
ENST00000254898	249.1701271	-2.918551678	0.28775	-10.14	3.57E-24	1.10E-21	ENSG00000132561	8	97869063	98036724	MATN2
ENST00000254908	103.3285668	-1.70621778	0.37077	-4.602	4.19E-06	6.30E-05	ENSG00000132570	5	134905130	134962644	PCBD2
ENST00000254928	154.5284839	-1.971109859	0.32242	-6.113	9.75E-10	3.51E-08	ENSG00000132591	17	28855015	28861061	ERAL1
ENST00000254950	550.8969629	-1.171519084	0.25	-4.686	2.78E-06	4.41E-05	ENSG00000132612	16	69311349	69326939	VPS4A
ENST00000255006	1589.391925	1.244602542	0.18768	6.6314	3.32E-11	1.63E-09	ENSG00000132669	20	19758257	20002456	RIN2
ENST00000255078	244.2178792	1.216507864	0.26056	4.6688	3.03E-06	4.75E-05	ENSG00000132740	11	68903890	68940601	IGHMBP2
ENST00000255152	201.156991	1.538043002	0.27997	5.4937	3.94E-08	9.96E-07	ENSG00000132801	20	45857613	45879122	ZSWIM3
ENST00000255174	1163.066094	1.116944615	0.21904	5.0993	3.41E-07	6.95E-06	ENSG00000132823	20	44195938	44210771	OSER1
ENST00000255320	1447.855548	-1.735501623	0.22978	-7.553	4.26E-14	3.60E-12	ENSG00000132967	3	22381818	22382460	HMGB1P5
ENST00000255389	79.48735989	-1.146035032	0.3951	-2.901	0.0037241	0.0199092	ENSG00000133027	17	17505562	17591708	PEMT
ENST00000255681	99.67973278	-1.026004254	0.36171	-2.837	0.004561	0.0234581	ENSG00000133315	11	63998557	64166113	MACROD1
ENST00000255764	117.6201116	1.037280974	0.34139	3.0384	0.0023785	0.0138516	ENSG00000133398	5	6371873	6378547	MED10
ENST00000256010	114.2948084	-5.516184404	0.56168	-9.821	9.16E-23	2.38E-20	ENSG00000133636	12	85874294	85882992	NTS
ENST00000256084	75.1808972	-1.733120131	0.41973	-4.129	3.64E-05	0.0004131	ENSG00000133710	5	148063979	148137382	SPINK5
ENST00000256196	352.3178541	-1.116242547	0.23719	-4.706	2.53E-06	4.05E-05	ENSG00000133818	11	14277921	14359183	RRAS2
ENST00000256319	166.1053195	-1.102246622	0.29726	-3.708	0.0002089	0.001841	ENSG00000133935	14	75649790	75660876	ERG28
ENST00000256429	406.1362182	-1.216543939	0.23952	-5.079	3.79E-07	7.63E-06	ENSG00000134046	18	54151605	54224669	MBD2
ENST00000256442	3706.245858	-1.132243076	0.20424	-5.544	2.96E-08	7.73E-07	ENSG00000134057	5	69167149	69178245	CCNB1
ENST00000256458	93.4032475	1.214267339	0.37777	3.2143	0.0013078	0.0085099	ENSG00000134070	3	10164918	10243745	IRAK2
ENST00000256538	99.81712903	-1.440221256	0.38757	-3.716	0.0002024	0.0017919	ENSG00000134146	15	35370879	35546165	DPH6
ENST00000256544	1838.336046	1.560462835	0.20673	7.5482	4.41E-14	3.72E-12	ENSG00000134152	15	34140673	34210096	KATNBL1
ENST00000256722	45.69086291	-4.913089988	0.80789	-6.081	1.19E-09	4.23E-08	ENSG00000134326	2	6848315	6865907	CMPK2
ENST00000256733	24.13043132	8.02059739	1.57683	5.0865	3.65E-07	7.36E-06	ENSG00000134339	11	18245239	18248668	SAA2
ENST00000256951	287.4625467	-2.571615725	0.26962	-9.538	1.46E-21	3.17E-19	ENSG00000134531	12	13196725	13219941	EMP1
ENST00000256996	116.7109237	-1.832549549	0.3541	-5.175	2.28E-07	4.84E-06	ENSG00000134574	11	47214973	47239217	DDB2
ENST00000257497	5110.060305	-2.279545079	0.21469	-10.62	2.46E-26	9.55E-24	ENSG00000135046	9	73151864	73170393	ANXA1
ENST00000257570	23.91016342	-6.590327937	1.62902	-4.046	5.22E-05	0.0005641	ENSG00000135114	12	121019110	121039246	OASL
ENST00000257572	52.54965778	-1.37198064	0.4713	-2.911	0.0036021	0.0193747	ENSG00000135116	12	116856143	116881441	HRK
ENST00000257663	129.1761585	-1.187002344	0.35132	-3.379	0.0007284	0.0052557	ENSG00000135211	7	77793727	77798434	TMEM60
ENST00000257776	16.64497925	7.48421753	1.63007	4.5913	4.40E-06	6.58E-05	ENSG00000135324	6	84033771	84090881	MRAP2
ENST00000257868	100.9498352	-1.890636281	0.3673	-5.147	2.64E-07	5.54E-06	ENSG00000135414	12	55743121	55757264	GDF11
ENST00000257909	239.5283976	-1.184079127	0.26422	-4.481	7.41E-06	0.0001038	ENSG00000135451	12	49323254	49331731	TROAP
ENST00000257934	528.3507822	-1.757455711	0.21903	-8.024	1.03E-15	1.07E-13	ENSG00000135476	12	53268298	53293638	ESPL1
ENST00000258080	493.0769235	1.115061851	0.21739	5.1293	2.91E-07	6.03E-06	ENSG00000115317	2	74529932	74533319	HTRA2
ENST00000258091	2901.491363	-1.719330282	0.21939	-7.837	4.62E-15	4.47E-13	ENSG00000135624	2	73234307	73253005	CCT7
ENST00000258111	562.2791258	1.051824065	0.2447	4.2983	1.72E-05	0.0002152	ENSG00000135643	12	70366289	70434292	KCNMB4
ENST00000258149	6123.312251	-1.266185884	0.20728	-6.109	1.00E-09	3.61E-08	ENSG00000135679	12	68808176	68845544	MDM2
ENST00000258169	172.4224847	-1.00211309	0.30622	-3.272	0.0010661	0.0071852	ENSG00000135698	16	82148161	82170224	MPHOSPH6
ENST00000258173	78.86674081	-1.124681909	0.40841	-2.754	0.0058907	0.0287823	ENSG00000205084	16	75536740	75556252	TMEM231

protein_coding	Yes	NM_033452.3	108
protein_coding	Yes	NM_002380.5	108
protein_coding	Yes	NM_032151.5	108
protein_coding	Yes	NM_005702.4	108
protein_coding	Yes	NM_013245.3	108
protein_coding	Yes	NM_018993.4	108
protein_coding	Yes	NM_002180.3	108
protein_coding	Yes	NM_080752.4	108
protein_coding	Yes	NM_016470.8	108
ped_processed_pse	Yes	-	108
protein_coding	Yes	NM_148172.3	108
protein_coding	Yes	NM_014067.4	108
protein_coding	Yes	NM_032286.3	108
protein_coding	Yes	NM_006183.5	108
protein_coding	Yes	NM_006846.4	108
protein_coding	Yes	NM_012250.6	108
protein_coding	Yes	NM_007176.4	108
protein_coding	Yes	NM_003927.5	108
protein_coding	Yes	NM_031966.4	108
protein_coding	Yes	NM_001570.4	108
protein_coding	Yes	NM_080650.4	108
protein_coding	Yes	NM_024713.3	108
protein_coding	Yes	NM_207315.4	108
protein_coding	Yes	NM_030754.5	108
protein_coding	Yes	NM_001423.3	108
protein_coding	Yes	NM_000107.3	108
protein_coding	Yes	NM_000700.3	108
protein_coding	Yes	NM_003733.4	108
protein_coding	Yes	NM_003806.4	108
protein_coding	Yes	NM_032936.4	108
protein_coding	Yes	NM_138409.4	108
protein_coding	Yes	NM_005811.5	108
protein_coding	Yes	NM_005480.4	108
protein_coding	Yes	NM_012291.5	108
protein_coding	Yes	NM_013247.5	108
protein_coding	Yes	NM_006429.4	108
protein_coding	Yes	NM_014505.6	108
protein_coding	Yes	NM_002392.6	108
protein_coding	Yes	NM_005792.2	108
protein_coding	Yes	NM_001077418.3	108

ENST00000258198	1962.264133	-1.229477175	0.17924	-6.859	6.92E-12	3.95E-10	ENSG00000135720	16	66720892	66751609	DYNC1LI2
ENST00000258200	40.73194034	1.896919652	0.55168	3.4384	0.0005851	0.0043805	ENSG00000135722	16	67159956	67164174	FBXL8
ENST00000258214	60.06378251	-2.087854767	0.46512	-4.489	7.16E-06	0.0001008	ENSG00000135736	16	57512180	57536571	CCDC102A
ENST00000258341	3815.600897	-1.341265897	0.17742	-7.56	4.04E-14	3.42E-12	ENSG00000135862	1	183023419	183145592	LAMC1
ENST00000258400	4.918404405	5.72469864	2.00033	2.8619	0.0042113	0.0220231	ENSG00000135914	2	231108229	231125042	HTR2B
ENST00000258411	584.9627423	2.368727034	0.24554	9.6471	5.06E-22	1.16E-19	ENSG00000135925	2	218880851	218893928	WNT10A
ENST00000258415	20.79404027	2.837791811	0.84216	3.3697	0.0007526	0.0054017	ENSG00000135929	2	218782146	218815293	CYP27A1
ENST00000258443	80.00397488	1.712569787	0.51451	3.3285	0.000873	0.0060925	ENSG00000135960	2	108894470	108989220	EDAR
ENST00000258607	1352.507042	-1.554589842	0.18358	-8.468	2.49E-17	3.21E-15	ENSG00000136108	13	52455477	52476627	CKAP2
ENST00000258729	3199.102318	-1.06383808	0.17541	-6.065	1.32E-09	4.66E-08	ENSG00000136231	7	23310208	23470491	IGF2BP3
ENST00000258733	163.5559333	1.370347657	0.36114	3.7945	0.000148	0.0013701	ENSG00000136235	7	23246774	23275108	GPNMB
ENST00000258796	846.9696322	1.000360501	0.20876	4.7919	1.65E-06	2.78E-05	ENSG00000136295	7	2631985	2664802	TTYH3
ENST00000258821	223.7653605	-2.833218154	0.29622	-9.565	1.13E-21	2.49E-19	ENSG00000136319	14	20286226	20305951	TTC5
ENST00000258888	55.61121693	1.252081876	0.46056	2.7186	0.0065552	0.0313837	ENSG00000136383	15	84817355	84873479	ALPK3
ENST00000258962	5862.516915	-1.172775575	0.17543	-6.685	2.30E-11	1.18E-09	ENSG00000136450	17	58000918	58007246	SRSF1
ENST00000258975	123.8139093	-1.974091478	0.33962	-5.813	6.15E-09	1.89E-07	ENSG00000136463	17	63600894	63608365	TACO1
ENST00000259154	809.4747181	-1.243842133	0.19704	-6.313	2.74E-10	1.09E-08	ENSG00000136636	1	215567303	215621807	KCTD3
ENST00000259241	389.4177139	-1.459825049	0.24669	-5.918	3.27E-09	1.06E-07	ENSG00000136720	2	128265479	128318868	HS6ST1
ENST00000259667	197.3034369	-1.345273858	0.2872	-4.684	2.81E-06	4.45E-05	ENSG00000137133	9	35812959	35815042	HINT2
ENST00000259818	267.8449304	-1.409795173	0.33072	-4.263	2.02E-05	0.0002472	ENSG00000137285	6	3224276	3227653	TUBB2B
ENST00000259939	280.0183636	-1.144273411	0.2741	-4.175	2.98E-05	0.0003468	ENSG00000137393	6	18387349	18468870	RNF144B
ENST00000260113	213.0706007	-3.004625188	0.41501	-7.24	4.49E-13	3.24E-11	ENSG00000137558	8	74824533	74855029	PI15
ENST00000260197	939.1989058	-1.113810093	0.1998	-5.575	2.48E-08	6.60E-07	ENSG00000137642	11	121452313	121633763	SORL1
ENST00000260324	368.6186797	1.04919511	0.29887	3.5105	0.0004473	0.0034938	ENSG00000137767	15	45635028	45691281	SQOR
ENST00000260356	3767.450393	2.609708232	0.85541	3.0508	0.0022822	0.0133892	ENSG00000137801	15	39581078	39599466	THBS1
ENST00000260382	147.2025959	1.212711254	0.31323	3.8716	0.0001081	0.0010468	ENSG00000137821	15	70892825	71053658	LRRC49
ENST00000260403	871.9946189	1.250272826	0.19415	6.4399	1.20E-10	5.18E-09	ENSG00000137842	15	43133585	43185144	TMEM62
ENST00000260442	4.406023918	5.566668149	2.0528	2.7117	0.006693	0.0318868	ENSG00000137875	15	52109262	52112775	BCL2L10
ENST00000260453	30.71765794	-2.497123681	0.66047	-3.781	0.0001563	0.0014384	ENSG00000138587	15	56428723	56465137	MNS1
ENST00000260505	16.88608428	-6.096649554	1.66512	-3.661	0.0002509	0.0021517	ENSG00000137941	1	83865023	83999132	TTLL7
ENST00000260526	11939.0143	-1.424933308	0.2365	-6.025	1.69E-09	5.84E-08	ENSG00000137962	1	94168904	94237584	ARHGAP29
ENST00000260682	16.27670068	3.285168514	0.95293	3.4475	0.0005659	0.0042589	ENSG00000138109	10	94938657	94990091	CYP2C9
ENST00000260702	1018.475883	2.098820732	0.21296	9.8553	6.50E-23	1.74E-20	ENSG00000138131	10	98247689	98268194	LOXL4
ENST00000260731	2533.53757	-1.324869789	0.18263	-7.254	4.04E-13	2.94E-11	ENSG00000138160	10	92593129	92655395	KIF11
ENST00000260843	106.5261569	-3.468135068	0.41224	-8.413	4.00E-17	4.99E-15	ENSG00000138271	3	151294085	151316820	GPR87
ENST00000260947	789.166885	-1.656854544	0.1989	-8.33	8.07E-17	9.67E-15	ENSG00000138376	2	214725645	214809683	BARD1
ENST00000260970	1043.693567	-1.642151079	0.19429	-8.452	2.87E-17	3.64E-15	ENSG00000138398	2	169584350	169641406	PPIG
ENST00000261024	59.88439538	-3.846746201	0.55246	-6.963	3.33E-12	2.03E-10	ENSG00000138449	2	189560589	189580786	SLC40A1
ENST00000261192	425.832093	-2.647247316	0.92125	-2.874	0.0040591	0.0213739	ENSG00000060982	12	24810023	24949101	BCAT1
ENST00000261245	588.3613145	-1.772762817	0.24902	-7.119	1.09E-12	7.34E-11	ENSG00000020426	14	60734760	60969965	MNAT1

protein_coding	Yes	NM_006141.3	108
protein_coding	Yes	NM_018378.3	108
protein_coding	Yes	NM_033212.4	108
protein_coding	Yes	NM_002293.4	108
protein_coding	Yes	NM_000867.5	108
protein_coding	Yes	NM_025216.3	108
protein_coding	Yes	NM_000784.4	108
protein_coding	Yes	NM_022336.4	108
protein_coding	Yes	NM_018204.5	108
protein_coding	Yes	NM_006547.3	108
protein_coding	Yes	NM_002510.3	108
protein_coding	Yes	NM_025250.3	108
protein_coding	Yes	NM_138376.3	108
protein_coding	Yes	NM_020778.5	108
protein_coding	Yes	NM_006924.5	108
protein_coding	Yes	NM_016360.4	108
protein_coding	Yes	NM_016121.5	108
protein_coding	Yes	NM_004807.3	108
protein_coding	Yes	NM_032593.3	108
protein_coding	Yes	NM_178012.5	108
protein_coding	Yes	NM_182757.4	108
protein_coding	Yes	NM_015886.5	108
protein_coding	Yes	NM_003105.6	108
protein_coding	Yes	NM_021199.4	108
protein_coding	Yes	NM_003246.4	108
protein_coding	Yes	NM_017691.5	108
protein_coding	Yes	NM_024956.4	108
protein_coding	Yes	NM_020396.4	108
protein_coding	Yes	NM_018365.4	108
protein_coding	Yes	NM_024686.6	108
protein_coding	Yes	NM_004815.4	108
protein_coding	Yes	NM_000771.4	108
protein_coding	Yes	NM_032211.7	108
protein_coding	Yes	NM_004523.4	108
protein_coding	Yes	NM_023915.4	108
protein_coding	Yes	NM_000465.4	108
protein_coding	Yes	NM_004792.3	108
protein_coding	Yes	NM_014585.6	108
protein_coding	Yes	NM_005504.7	108
protein_coding	Yes	NM_002431.4	108

ENST00000261254	20.20147402	-3.97585039	0.97497	-4.078	4.54E-05	0.0005003	ENSG00000118971	12	4273761	4305353	CCND2
ENST00000261332	1158.938166	-1.190272221	0.18861	-6.311	2.78E-10	1.11E-08	ENSG00000172466	18	35332226	35344420	ZNF24
ENST00000261349	1211.291408	-1.099377769	0.20258	-5.427	5.73E-08	1.40E-06	ENSG00000070018	12	12116024	12267044	LRP6
ENST00000261353	2256.536725	1.979282908	0.20898	9.4711	2.77E-21	5.77E-19	ENSG00000119801	2	30147006	30160533	YPEL5
ENST00000261366	1917.726491	-1.746433557	0.1901	-9.187	4.03E-20	7.10E-18	ENSG00000113368	5	126777135	126837020	LMNB1
ENST00000261402	364.2359579	1.949169266	0.23958	8.1358	4.09E-16	4.50E-14	ENSG00000074590	12	106063344	106138954	NUAK1
ENST00000261448	9.795117545	-3.19114086	1.19691	-2.666	0.0076727	0.0354672	ENSG00000118729	1	115700020	115768714	CASQ2
ENST00000261464	29.40346542	-4.871396565	0.92668	-5.257	1.47E-07	3.24E-06	ENSG00000082512	1	211326832	211374946	TRAF5
ENST00000261483	854.6541411	-1.104439029	0.20568	-5.37	7.88E-08	1.87E-06	ENSG00000112893	5	109689926	109869625	MAN2A1
ENST00000261486	22.1215785	-6.476505268	1.64338	-3.941	8.12E-05	0.0008239	ENSG00000129595	5	112162618	112419277	EPB41L4A
ENST00000261507	1037.952135	-1.47572017	0.20023	-7.37	1.71E-13	1.32E-11	ENSG00000052802	4	165327668	165343164	MSMO1
ENST00000261534	203.9298722	-1.283426983	0.28811	-4.455	8.40E-06	0.0001157	ENSG00000009830	14	77274955	77320883	POMT2
ENST00000261558	787.9291267	-1.226049428	0.19787	-6.196	5.78E-10	2.17E-08	ENSG00000053770	14	57268970	57298742	AP5M1
ENST00000261593	404.7590825	-1.679448401	0.23037	-7.29	3.09E-13	2.30E-11	ENSG00000134758	18	32091873	32131561	RNF138
ENST00000261597	576.3763403	-1.171765808	0.21411	-5.473	4.43E-08	1.11E-06	ENSG00000080986	18	2571556	2616635	NDC80
ENST00000261654	4.946371907	5.738428866	2.09357	2.741	0.0061256	0.0297031	ENSG00000111452	12	130953906	131141469	ADGRD1
ENST00000261769	5430.622834	-1.609753945	0.18634	-8.639	5.68E-18	7.94E-16	ENSG00000039068	16	68737291	68835537	CDH1
ENST00000261783	434.5474354	2.409239777	0.26993	8.9254	4.44E-19	7.08E-17	ENSG00000081181	14	67619919	67651708	ARG2
ENST00000261799	8.254022801	6.473511846	1.79719	3.602	0.0003158	0.0026029	ENSG00000113721	5	150113838	150155845	PDGFRB
ENST00000261862	158.4415502	-1.541552211	0.32231	-4.783	1.73E-06	2.90E-05	ENSG00000187720	15	71115470	71783383	THSD4
ENST00000261867	97.14999393	-1.32106855	0.39707	-3.327	0.0008776	0.0061161	ENSG00000104154	15	45479605	45522755	SLC30A4
ENST00000261879	64.34902277	-1.517039655	0.44671	-3.396	0.0006837	0.0049867	ENSG00000138613	15	63277604	63309126	APH1B
ENST00000261883	13.63798317	3.01545374	1.02657	2.9374	0.0033096	0.0181095	ENSG00000138615	15	65194759	65211473	CILP
ENST00000261908	297.4842253	-1.316037566	0.29681	-4.434	9.25E-06	0.0001258	ENSG00000067141	15	73052462	73305205	NEO1
ENST00000261965	288.9137362	-1.602792277	0.25884	-6.192	5.93E-10	2.22E-08	ENSG00000126216	13	112485010	112588153	TUBGCP3
ENST00000261973	1006.030122	-1.679093113	0.19167	-8.76	1.95E-18	2.85E-16	ENSG00000119707	14	73058533	73123899	RBM25
ENST00000262094	176.5460614	-3.284405086	0.37753	-8.7	3.33E-18	4.74E-16	ENSG00000041353	18	54828476	54895516	RAB27B
ENST00000262096	414.9364742	-1.952094392	0.29359	-6.649	2.95E-11	1.47E-09	ENSG00000104219	8	17156481	17224799	ZDHHC2
ENST00000262120	472.9118732	-1.672564017	0.22428	-7.457	8.82E-14	7.09E-12	ENSG00000128791	18	9334772	9402420	TWSG1
ENST00000262133	711.363327	-2.248004142	0.23806	-9.443	3.63E-21	7.46E-19	ENSG00000103479	16	53434470	53491648	RBL2
ENST00000262134	776.0005671	-1.540565625	0.21735	-7.088	1.36E-12	9.04E-11	ENSG00000087253	16	55509071	55586666	LPCAT2
ENST00000262138	21.07164572	-6.405580149	1.64044	-3.905	9.43E-05	0.0009344	ENSG00000075461	17	66964706	67033398	CACNG4
ENST00000262139	111.0388314	1.768005891	0.40434	4.3726	1.23E-05	0.0001608	ENSG00000070540	17	68421280	68457496	WIPI1
ENST00000262207	90.16198573	-1.236744161	0.45662	-2.708	0.0067592	0.0321495	ENSG00000121005	8	74984504	75034558	CRISPLD1
ENST00000262213	3182.385721	-1.06460001	0.19323	-5.509	3.60E-08	9.20E-07	ENSG00000067167	8	70573217	70608416	TRAM1
ENST00000262233	93.51580377	-2.446578764	0.41995	-5.826	5.68E-09	1.76E-07	ENSG00000066629	14	99793412	99942060	EML1
ENST00000262244	116.9128401	-1.04203041	0.33974	-3.067	0.0021612	0.012816	ENSG00000120162	9	27325208	27529814	MOB3B
ENST00000262293	1202.539158	-1.197481801	0.18601	-6.438	1.21E-10	5.24E-09	ENSG00000068489	17	59155745	59206709	PRR11
ENST00000262384	581.0235629	-1.248700096	0.2087	-5.983	2.19E-09	7.37E-08	ENSG00000102921	16	48538725	48610180	N4BP1
ENST00000262429	433.3705747	-1.283423493	0.25	-5.134	2.84E-07	5.91E-06	ENSG00000064270	16	84368537	84464187	ATP2C2

protein_coding	Yes	NM_001759.4	108
protein_coding	Yes	NM_006965.4	108
protein_coding	Yes	NM_002336.3	108
protein_coding	Yes	NM_016061.3	108
protein_coding	Yes	NM_005573.4	108
protein_coding	Yes	NM_014840.3	108
protein_coding	Yes	NM_001232.4	108
protein_coding	Yes	NM_001033910.3	108
protein_coding	Yes	NM_002372.4	108
protein_coding	Yes	NM_022140.5	108
protein_coding	Yes	NM_006745.5	108
protein_coding	Yes	NM_013382.7	108
protein_coding	Yes	NM_018229.4	108
protein_coding	Yes	NM_016271.5	108
protein_coding	Yes	NM_006101.3	108
protein_coding	Yes	NM_198827.5	108
protein_coding	Yes	NM_004360.5	108
protein_coding	Yes	NM_001172.4	108
protein_coding	Yes	NM_002609.4	108
protein_coding	Yes	NM_024817.3	108
protein_coding	Yes	NM_013309.6	108
protein_coding	Yes	NM_031301.4	108
protein_coding	Yes	NM_003613.4	108
protein_coding	Yes	NM_002499.4	108
protein_coding	Yes	NM_006322.6	108
protein_coding	Yes	NM_021239.3	108
protein_coding	Yes	NM_004163.4	108
protein_coding	Yes	NM_016353.5	108
protein_coding	Yes	NM_020648.6	108
protein_coding	Yes	NM_005611.4	108
protein_coding	Yes	NM_017839.5	108
protein_coding	Yes	NM_014405.4	108
protein_coding	Yes	NM_017983.7	108
protein_coding	Yes	NM_031461.6	108
protein_coding	Yes	NM_014294.6	108
protein_coding	Yes	NM_004434.3	108
protein_coding	Yes	NM_024761.5	108
protein_coding	Yes	NM_018304.4	108
protein_coding	Yes	NM_153029.4	108
protein_coding	Yes	NM_014861.4	108

ENST00000262450	37.21131183	1.572643401	0.56864	2.7656	0.0056816	0.0279588	ENSG00000116254	1	6101786	6180321	CHD5
ENST00000262460	1106.261751	-1.740165568	0.20829	-8.355	6.56E-17	7.97E-15	ENSG00000101003	20	25407672	25448563	GINS1
ENST00000262498	409.3298495	-1.284341983	0.23213	-5.533	3.15E-08	8.16E-07	ENSG00000070761	16	58113591	58129381	CFAP20
ENST00000262507	312.9749415	-1.01110799	0.24841	-4.07	4.69E-05	0.0005146	ENSG00000088682	16	57447478	57461270	COQ9
ENST00000262519	141.6490789	1.237914241	0.31365	3.9468	7.92E-05	0.0008068	ENSG00000099381	16	30957753	30984664	SETD1A
ENST00000262525	292.3321132	-2.302978372	0.25983	-8.864	7.75E-19	1.18E-16	ENSG00000102870	16	30778455	30787205	ZNF629
ENST00000262545	137.5851525	2.736489234	0.34396	7.9557	1.78E-15	1.81E-13	ENSG00000125851	20	17227039	17484578	PCSK2
ENST00000262646	1852.411396	-1.002667882	0.19996	-5.014	5.32E-07	1.03E-05	ENSG00000104388	8	60516986	60623644	RAB2A
ENST00000262776	2827.355948	1.547776652	0.23389	6.6175	3.65E-11	1.78E-09	ENSG00000108679	17	78971254	78979923	LGALS3BP
ENST00000262805	21.13474978	2.699141294	0.87739	3.0763	0.0020958	0.0124968	ENSG00000105650	19	18207964	18226438	PDE4C
ENST00000262844	421.2668748	-1.095310996	0.23039	-4.754	1.99E-06	3.28E-05	ENSG00000101935	X	110194185	110318085	AMMECR1
ENST00000263071	14.11562632	2.804973193	0.97086	2.8892	0.0038626	0.0205191	ENSG00000074660	17	1633857	1645732	SCARF1
ENST00000263094	467.2957891	1.118807483	0.26486	4.2241	2.40E-05	0.000287	ENSG00000064687	19	1040106	1065572	ABCA7
ENST00000263102	2398.288444	1.00287644	0.20266	4.9485	7.48E-07	1.38E-05	ENSG00000108091	10	59788746	59906556	CCDC6
ENST00000263125	48.55825325	-6.031548452	0.95482	-6.317	2.67E-10	1.07E-08	ENSG00000065675	10	6427147	6580276	PRKCQ
ENST00000263205	1235.5076	1.058126659	0.18523	5.7126	1.11E-08	3.22E-07	ENSG00000099917	22	20507609	20587619	MED15
ENST00000263233	62.77726195	4.179283653	0.58665	7.124	1.05E-12	7.10E-11	ENSG00000102003	X	49187814	49200193	SYP
ENST00000263266	54.16715395	1.718292869	0.47273	3.6348	0.0002782	0.0023404	ENSG00000105523	19	48599960	48615076	FAM83E
ENST00000263277	101.9547439	-2.233824701	0.39687	-5.629	1.82E-08	5.00E-07	ENSG00000024422	19	47713421	47743134	EHD2
ENST00000263368	1153.194909	1.377054016	0.21804	6.3156	2.69E-10	1.07E-08	ENSG00000090013	19	40447767	40465745	BLVRB
ENST00000263464	763.1408682	1.889732628	0.20094	9.4046	5.22E-21	1.05E-18	ENSG00000023445	11	102317483	102339403	BIRC3
ENST00000263620	570.7599973	-1.486332965	0.21737	-6.838	8.04E-12	4.53E-10	ENSG00000116017	19	926034	975939	ARID3A
ENST00000263650	110.0222937	-2.013013425	0.38758	-5.194	2.06E-07	4.42E-06	ENSG00000021762	11	3087106	3165310	OSBPL5
ENST00000263663	688.3630778	-1.00245752	0.22064	-4.543	5.53E-06	8.03E-05	ENSG00000115750	2	9843473	9934416	TAF1B
ENST00000263681	608.9037342	-1.295145497	0.21191	-6.112	9.86E-10	3.55E-08	ENSG00000077514	11	74592581	74643076	POLD3
ENST00000263726	109.527672	-1.213246338	0.34516	-3.515	0.0004398	0.003444	ENSG00000121454	1	180230263	180278984	LHX4
ENST00000263736	406.227078	-1.005663463	0.22847	-4.402	1.07E-05	0.000143	ENSG00000068784	2	45388679	45611267	SRBD1
ENST00000263754	299.411969	-1.281010013	0.28621	-4.476	7.61E-06	0.0001062	ENSG00000114166	3	20040445	20154404	KAT2B
ENST00000263849	19.95441983	-2.534776183	0.79976	-3.169	0.0015274	0.0096594	ENSG00000104427	8	78666088	78719765	ZC2HC1A
ENST00000263867	576.2046212	-1.509323339	0.21315	-7.081	1.43E-12	9.44E-11	ENSG00000042493	2	85394752	85410366	CAPG
ENST00000263932	41.00480286	3.925419662	0.67033	5.856	4.74E-09	1.49E-07	ENSG00000120949	1	12063302	12144207	TNFRSF8
ENST00000263955	1426.997189	-1.573888694	0.18977	-8.294	1.10E-16	1.30E-14	ENSG00000081320	2	196133582	196171578	STK17B
ENST00000263966	205.106473	-1.894098674	0.28378	-6.674	2.48E-11	1.26E-09	ENSG00000058056	3	179653039	179789401	USP13
ENST00000264001	229.5198586	-1.049159601	0.30228	-3.471	0.0005189	0.003958	ENSG00000217555	16	66552575	66566068	CKLF
ENST00000264021	145.0601477	-3.245387642	0.40818	-7.951	1.85E-15	1.88E-13	ENSG00000118096	11	118544542	118565931	IFT46
ENST00000264025	115.5348574	-2.168142253	0.36054	-6.014	1.82E-09	6.22E-08	ENSG00000110400	11	119660991	119729200	NECTIN1
ENST00000264059	61.09431327	-1.719764825	0.45578	-3.773	0.0001611	0.001476	ENSG00000115468	2	232633603	232682776	EFHD1
ENST00000264079	221.5157008	2.398604848	0.28475	8.4235	3.65E-17	4.60E-15	ENSG00000090674	19	7522623	7534009	MCOLN1
ENST00000264080	576.0715774	1.103679632	0.22496	4.906	9.29E-07	1.67E-05	ENSG00000125734	19	6729913	6737580	GPR108
ENST00000264094	35.51169519	-1.949271144	0.5818	-3.35	0.0008068	0.0057194	ENSG00000115318	2	74532257	74553942	LOXL3

protein_coding	Yes	NM_015557.3	108
protein_coding	Yes	NM_021067.5	108
protein_coding	Yes	NM_013242.3	108
protein_coding	Yes	NM_020312.4	108
protein_coding	Yes	NM_014712.3	108
protein_coding	Yes	NM_001080417.3	108
protein_coding	Yes	NM_002594.5	108
protein_coding	Yes	NM_002865.3	108
protein_coding	Yes	NM_005567.4	108
protein_coding	Yes	NM_001098818.4	108
protein_coding	Yes	NM_015365.3	108
protein_coding	Yes	NM_003693.4	108
protein_coding	Yes	NM_019112.4	108
protein_coding	Yes	NM_005436.5	108
protein_coding	Yes	NM_006257.5	108
protein_coding	Yes	NM_001003891.3	108
protein_coding	Yes	NM_003179.3	108
protein_coding	Yes	NM_017708.4	108
protein_coding	Yes	NM_014601.4	108
protein_coding	Yes	NM_000713.3	108
protein_coding	Yes	NM_001165.5	108
protein_coding	Yes	NM_005224.3	108
protein_coding	Yes	NM_020896.4	108
protein_coding	Yes	NM_005680.3	108
protein_coding	Yes	NM_006591.3	108
protein_coding	Yes	NM_033343.4	108
protein_coding	Yes	NM_018079.5	108
protein_coding	Yes	NM_003884.5	108
protein_coding	Yes	NM_016010.3	108
protein_coding	Yes	NM_001747.4	108
protein_coding	Yes	NM_001243.5	108
protein_coding	Yes	NM_004226.4	108
protein_coding	Yes	NM_003940.3	108
protein_coding	Yes	NM_016951.4	108
protein_coding	Yes	NM_001168618.2	108
protein_coding	Yes	NM_002855.5	108
protein_coding	Yes	NM_025202.4	108
protein_coding	Yes	NM_020533.3	108
protein_coding	Yes	NM_001080452.2	108
protein_coding	Yes	NM_032603.5	108

ENST00000264158	1744.340338	-1.046577559	0.19553	-5.352	8.67E-08	2.03E-06	ENSG00000115839	2	135052291	135170710	RAB3GAP1
ENST00000264159	400.4841239	1.090482623	0.22937	4.7543	1.99E-06	3.28E-05	ENSG00000121988	2	135196968	135531218	ZRANB3
ENST00000264167	1618.393999	-1.062562688	0.17922	-5.929	3.05E-09	9.97E-08	ENSG00000018510	2	177392772	177543834	AGPS
ENST00000264202	513.3663228	-1.166082491	0.21766	-5.357	8.45E-08	1.98E-06	ENSG00000077549	1	19338774	19485539	CAPZB
ENST00000264218	59.08526979	-3.722026674	0.54582	-6.819	9.16E-12	5.10E-10	ENSG00000109255	4	55595230	55636298	NMU
ENST00000264228	67.40316961	-2.178314922	0.44231	-4.925	8.44E-07	1.54E-05	ENSG00000128039	4	55346241	55373100	SRD5A3
ENST00000264234	214.8116982	-3.915108431	0.39418	-9.932	3.01E-23	8.43E-21	ENSG00000114638	3	119173597	119205143	UPK1B
ENST00000264279	2146.386874	-1.029889064	0.2132	-4.831	1.36E-06	2.34E-05	ENSG00000055044	2	202265762	202303661	NOP58
ENST00000264389	682.8521819	-1.082241058	0.20361	-5.315	1.07E-07	2.43E-06	ENSG00000138663	4	83035182	83075818	COPS4
ENST00000264712	127.7342146	-1.232077399	0.32954	-3.739	0.0001849	0.0016577	ENSG00000084731	2	25926597	25982497	KIF3C
ENST00000264735	14.6719459	4.822067205	1.32106	3.6502	0.0002621	0.0022334	ENSG00000127252	3	193241221	193270855	PLAAT1
ENST00000264741	41.85881831	3.225964358	0.60029	5.374	7.70E-08	1.83E-06	ENSG00000144668	3	37452140	37823507	ITGA9
ENST00000264832	1773.508008	2.273353765	0.18065	12.584	2.59E-36	3.91E-33	ENSG00000090339	19	10271119	10286615	ICAM1
ENST00000264866	94.30998621	-2.122148218	0.38471	-5.516	3.46E-08	8.89E-07	ENSG00000109680	4	26584083	26756223	TBC1D19
ENST00000264893	1892.308483	-1.474881969	0.17705	-8.33	8.06E-17	9.67E-15	ENSG00000138758	4	76949751	77038615	SEPTIN11
ENST00000264930	540.0929657	1.608024874	0.23117	6.9559	3.50E-12	2.12E-10	ENSG00000113504	5	1050383	1112063	SLC12A7
ENST00000265026	56.97880328	1.84341347	0.47289	3.8982	9.69E-05	0.0009567	ENSG00000073803	3	185363135	185489094	MAP3K13
ENST00000265036	527.5504981	-1.180763352	0.22429	-5.264	1.41E-07	3.12E-06	ENSG00000035499	5	60596911	60700166	DEPDC1B
ENST00000265052	66.85590051	-1.386255564	0.43113	-3.215	0.0013026	0.0084826	ENSG00000074416	3	127689065	127822515	MGLL
ENST00000265073	1622.01932	-1.805844098	0.18322	-9.856	6.46E-23	1.73E-20	ENSG00000113387	5	32585556	32604079	SUB1
ENST00000265107	512.8613493	-1.176848888	0.21454	-5.485	4.12E-08	1.04E-06	ENSG00000082068	5	37379317	37753435	WDR70
ENST00000265109	613.1336408	-1.581408515	0.24578	-6.434	1.24E-10	5.35E-09	ENSG00000039560	5	34656327	34832612	RAI14
ENST00000265138	813.3174535	1.032187548	0.20947	4.9276	8.32E-07	1.52E-05	ENSG00000113369	5	91368630	91383317	ARRDC3
ENST00000265148	2850.581681	-1.860474017	0.17252	-10.78	4.10E-27	1.77E-24	ENSG00000138778	4	103105810	103198343	CENPE
ENST00000265162	170.4764924	-1.567259947	0.29799	-5.259	1.45E-07	3.20E-06	ENSG00000138792	4	110476154	110565285	ENPEP
ENST00000265260	1264.810236	-1.18284964	0.2052	-5.765	8.19E-09	2.45E-07	ENSG00000081154	3	101574179	101594465	PCNP
ENST00000265294	35.90165544	-5.139989156	0.92461	-5.559	2.71E-08	7.14E-07	ENSG00000094755	5	170783724	170814047	GABRP
ENST00000265310	8.606647369	6.535899283	1.81087	3.6093	0.0003071	0.0025431	ENSG00000127412	7	142908100	142933746	TRPV5
ENST00000265381	18.07709588	-3.074157739	0.9123	-3.37	0.0007526	0.0054017	ENSG00000107282	9	69427531	69672371	APBA1
ENST00000265382	8.107104327	-3.933906617	1.47779	-2.662	0.0077674	0.035826	ENSG00000107242	9	68705239	69009176	PIP5K1B
ENST00000265394	77.01115018	-2.195109874	0.49598	-4.426	9.61E-06	0.0001299	ENSG00000106066	7	28995234	29146537	CPVL
ENST00000265440	5.527219217	5.896413602	1.95983	3.0086	0.0026242	0.0150026	ENSG00000105967	7	115935151	116030763	TFEC
ENST00000265471	357.4434089	1.176488834	0.23356	5.0372	4.72E-07	9.24E-06	ENSG00000149541	11	62615295	62621986	B3GAT3
ENST00000265562	98.62069209	1.153780153	0.42152	2.7372	0.0061972	0.0299787	ENSG00000076201	3	47381020	47413435	PTPN23
ENST00000265593	177.7365929	1.357213722	0.29517	4.598	4.26E-06	6.40E-05	ENSG00000114859	3	184346184	184361605	CLCN2
ENST00000265616	180.3185089	1.116875532	0.29402	3.7986	0.0001455	0.0013512	ENSG00000104691	8	30744163	30767006	UBXN8
ENST00000265678	34.81179777	1.617625378	0.58668	2.7572	0.0058292	0.0285301	ENSG00000071242	6	166409363	166627251	RPS6KA2
ENST00000265689	1230.021936	1.00513703	0.19577	5.1343	2.83E-07	5.90E-06	ENSG00000110721	11	68052858	68121388	CHKA
ENST00000265728	488.7649546	-1.697408596	0.23379	-7.26	3.86E-13	2.82E-11	ENSG00000006634	7	87876492	87909553	DBF4
ENST00000265806	197.1754809	-3.069300772	0.30714	-9.993	1.63E-23	4.64E-21	ENSG00000104679	8	23288107	23296279	R3HCC1

protein_coding	Yes	NM_012233.3	108
protein_coding	Yes	NM_032143.4	108
protein_coding	Yes	NM_003659.4	108
protein_coding	Yes	NM_004930.5	108
protein_coding	Yes	NM_006681.4	108
protein_coding	Yes	NM_024592.5	108
protein_coding	Yes	NM_006952.4	108
protein_coding	Yes	NM_015934.5	108
protein_coding	Yes	NM_016129.3	108
protein_coding	Yes	NM_002254.8	108
protein_coding	Yes	NM_020386.5	108
protein_coding	Yes	NM_002207.3	108
protein_coding	Yes	NM_000201.3	108
protein_coding	Yes	NM_018317.4	108
protein_coding	Yes	NM_018243.4	108
protein_coding	Yes	NM_006598.3	108
protein_coding	Yes	NM_004721.5	108
protein_coding	Yes	NM_018369.3	108
protein_coding	Yes	NM_007283.7	108
protein_coding	Yes	NM_006713.4	108
protein_coding	Yes	NM_018034.4	108
protein_coding	Yes	NM_015577.3	108
protein_coding	Yes	NM_020801.4	108
protein_coding	Yes	NM_001813.3	108
protein_coding	Yes	NM_001977.4	108
protein_coding	Yes	NM_020357.3	108
protein_coding	Yes	NM_014211.3	108
protein_coding	Yes	NM_019841.7	108
protein_coding	Yes	NM_001163.4	108
protein_coding	Yes	NM_003558.4	108
protein_coding	Yes	NM_031311.5	108
protein_coding	Yes	NM_012252.4	108
protein_coding	Yes	NM_012200.4	108
protein_coding	Yes	NM_015466.4	108
protein_coding	Yes	NM_004366.6	108
protein_coding	Yes	NM_005671.4	108
protein_coding	Yes	NM_021135.6	108
protein_coding	Yes	NM_001277.3	108
protein_coding	Yes	NM_006716.4	108
protein_coding	Yes	NM_001136108.3	108

ENST00000265807	346.9725356	-1.040046823	0.24366	-4.268	1.97E-05	0.0002417	ENSG00000104611	8	19313692	19396218	SH2D4A
ENST00000265896	1393.385691	-1.478369869	0.1921	-7.696	1.41E-14	1.27E-12	ENSG00000104549	8	124998504	125022283	SQLE
ENST00000265981	2656.76678	-1.446743593	0.24241	-5.968	2.40E-09	8.02E-08	ENSG00000110315	11	10511672	10541187	RNF141
ENST00000266263	91.59134885	-1.987967692	0.43312	-4.59	4.44E-06	6.63E-05	ENSG00000242114	22	30425767	30429054	MTFP1
ENST00000266304	631.6524006	1.56349957	0.22787	6.8613	6.82E-12	3.90E-10	ENSG00000167074	22	41381941	41399326	TEF
ENST00000266383	22.88082307	-2.755025275	0.80536	-3.421	0.0006243	0.0046167	ENSG00000139044	12	459938	563509	B4GALNT3
ENST00000266397	23.12346738	-2.295192564	0.72147	-3.181	0.0014663	0.0093545	ENSG00000139055	12	14914038	14938537	ERP27
ENST00000266483	157.210563	-1.793243457	0.31248	-5.739	9.54E-09	2.80E-07	ENSG00000139133	12	34022495	34028302	ALG10
ENST00000266508	511.445054	-1.756780581	0.26808	-6.553	5.63E-11	2.60E-09	ENSG00000139154	12	19439491	19522227	AEBP2
ENST00000266517	1564.188922	-1.438681791	0.21324	-6.747	1.51E-11	7.99E-10	ENSG00000139163	12	22625170	22690665	ETNK1
ENST00000266556	10.90674731	-3.783385083	1.23447	-3.065	0.0021782	0.0128983	ENSG00000139192	12	6452041	6462316	TAPBPL
ENST00000266712	1361.363748	-1.398499136	0.19835	-7.051	1.78E-12	1.16E-10	ENSG00000139324	12	88142306	88199887	TMTC3
ENST00000266943	164.8378444	1.519301189	0.30123	5.0436	4.57E-07	8.97E-06	ENSG00000139508	13	28700079	28718970	SLC46A3
ENST00000266970	1651.112946	-1.131686759	0.17873	-6.332	2.43E-10	9.77E-09	ENSG00000123374	12	55966829	55972789	CDK2
ENST00000266987	259.5470859	-1.317700391	0.30308	-4.348	1.38E-05	0.0001777	ENSG00000139546	12	53501282	53506431	TARBP2
ENST00000267485	26.64302958	1.753694361	0.6666	2.6308	0.0085179	0.0386018	ENSG00000139971	14	58000760	58152213	ARMH4
ENST00000267807	399.4357212	-1.335991835	0.22816	-5.855	4.76E-09	1.49E-07	ENSG00000137871	15	56630175	56733509	ZNF280D
ENST00000267838	69.2147893	-1.3324841	0.43385	-3.071	0.0021311	0.012668	ENSG00000140280	15	51723010	51737655	LYSMD2
ENST00000267853	109.895506	-1.030339255	0.34321	-3.002	0.0026812	0.0152605	ENSG00000263155	15	57591903	57685364	MYZAP
ENST00000267970	1011.889196	-1.263651074	0.23137	-5.462	4.72E-08	1.17E-06	ENSG00000140391	15	77041403	77071109	TSPAN3
ENST00000268057	349.1040221	1.415860925	0.25364	5.5821	2.38E-08	6.34E-07	ENSG00000140463	15	72686206	72738473	BBS4
ENST00000268097	23.5216165	7.982985701	1.58028	5.0516	4.38E-07	8.64E-06	ENSG00000213614	15	72340923	72376014	HEXA
ENST00000268459	114.7644865	-1.293918358	0.34464	-3.754	0.0001737	0.0015734	ENSG00000140807	16	50548395	50649249	NKD1
ENST00000268483	212.1692905	1.154346459	0.27506	4.1966	2.71E-05	0.0003189	ENSG00000140830	16	72084856	72093620	TXNL4B
ENST00000268607	428.6608175	1.045325463	0.24107	4.3362	1.45E-05	0.0001856	ENSG00000140941	16	87392335	87404774	MAP1LC3B
ENST00000269195	940.9589489	-1.210992152	0.21308	-5.683	1.32E-08	3.76E-07	ENSG00000141429	18	35581739	35711834	GALNT1
ENST00000269228	671.2788223	1.303233943	0.20367	6.3987	1.57E-10	6.57E-09	ENSG00000141458	18	23531441	23586506	NPC1
ENST00000269298	267.9285761	1.183745903	0.25817	4.5851	4.54E-06	6.76E-05	ENSG00000141504	17	7626233	7627821	SAT2
ENST00000269389	113.422639	-3.246154765	0.46292	-7.012	2.34E-12	1.49E-10	ENSG00000141574	17	82321023	82333766	SECTM1
ENST00000269394	89.73253719	-1.312440473	0.43447	-3.021	0.0025215	0.0145206	ENSG00000141579	17	82829433	82840022	ZNF750
ENST00000269466	23.77135744	-2.143979447	0.70834	-3.027	0.002472	0.0142864	ENSG00000141642	18	50968039	50988121	ELAC1
ENST00000269593	325.4448757	1.759996039	0.25753	6.8342	8.25E-12	4.62E-10	ENSG00000141753	17	40443449	40457725	IGFBP4
ENST00000269881	12.71083649	7.097983543	1.70266	4.1688	3.06E-05	0.0003548	ENSG00000269058	19	16479060	16496167	CALR3
ENST00000270061	155.8865767	-1.883924434	0.32809	-5.742	9.35E-09	2.75E-07	ENSG00000130511	19	18419397	18434562	SSBP4
ENST00000270176	2693.124805	1.438163187	0.19214	7.485	7.16E-14	5.84E-12	ENSG00000142186	11	65525082	65538704	SCYL1
ENST00000270233	517.4735281	-1.022722694	0.31567	-3.24	0.001196	0.0079047	ENSG00000187244	19	44809102	44821421	BCAM
ENST00000270257	122.5608101	-1.787575095	0.3416	-5.233	1.67E-07	3.65E-06	ENSG00000142252	19	45079259	45091518	GEMIN7
ENST00000270747	89.75188939	-1.324720134	0.37524	-3.53	0.000415	0.0032798	ENSG00000142632	1	16197853	16212652	ARHGEF19
ENST00000271002	253.2406503	-1.36479265	0.25842	-5.281	1.28E-07	2.87E-06	ENSG00000142856	1	63440769	63523225	ITGB3BP
ENST00000271332	326.5907985	-1.342435834	0.25894	-5.184	2.17E-07	4.63E-06	ENSG00000143126	1	109249538	109275751	CELSR2

protein_coding	Yes	NM_022071.4	108
protein_coding	Yes	NM_003129.4	108
protein_coding	Yes	NM_016422.4	108
protein_coding	Yes	NM_016498.5	108
protein_coding	Yes	NM_003216.4	108
protein_coding	Yes	NM_173593.4	108
protein_coding	Yes	NM_152321.4	108
protein_coding	Yes	NM_032834.4	108
protein_coding	Yes	NM_153207.5	108
protein_coding	Yes	NM_018638.5	108
protein_coding	Yes	NM_018009.5	108
protein_coding	Yes	NM_181783.4	108
protein_coding	Yes	NM_181785.4	108
protein_coding	Yes	NM_001798.5	108
protein_coding	Yes	NM_134323.2	108
protein_coding	Yes	NM_001001872.4	108
protein_coding	Yes	NM_017661.4	108
protein_coding	Yes	NM_153374.3	108
protein_coding	Yes	NM_001018100.5	108
protein_coding	Yes	NM_005724.6	108
protein_coding	Yes	NM_033028.5	108
protein_coding	Yes	NM_000520.6	108
protein_coding	Yes	NM_033119.5	108
protein_coding	Yes	NM_017853.3	108
protein_coding	Yes	NM_022818.5	108
protein_coding	Yes	NM_020474.4	108
protein_coding	Yes	NM_000271.5	108
protein_coding	Yes	NM_133491.5	108
protein_coding	Yes	NM_003004.3	108
protein_coding	Yes	NM_024702.3	108
protein_coding	Yes	NM_018696.3	108
protein_coding	Yes	NM_001552.3	108
protein_coding	Yes	NM_145046.5	108
protein_coding	Yes	NM_032627.5	108
protein_coding	Yes	NM_020680.4	108
protein_coding	Yes	NM_005581.5	108
protein_coding	Yes	NM_024707.3	108
protein_coding	Yes	NM_153213.5	108
protein_coding	Yes	NM_014288.5	108
protein_coding	Yes	NM_001408.3	108

ENST00000271417	21.70700032	-2.179103163	0.77305	-2.819	0.0048196	0.0245348	ENSG00000143195	1	166908186	166975540	ILDR2
ENST00000271452	610.6137335	-1.791558787	0.20996	-8.533	1.43E-17	1.90E-15	ENSG00000143228	1	163321953	163355759	NUF2
ENST00000271588	92.3244517	1.557108372	0.38257	4.0701	4.70E-05	0.0005151	ENSG00000143341	1	185734390	186190949	HMCN1
ENST00000271638	847.4440462	-1.698049894	0.19851	-8.554	1.19E-17	1.61E-15	ENSG00000163191	1	152032505	152037004	S100A11
ENST00000271643	113.9289333	-1.236371775	0.40122	-3.082	0.0020592	0.0123147	ENSG00000143382	1	150549407	150560933	ADAMTSL4
ENST00000271843	882.4156066	-1.01732261	0.20546	-4.951	7.37E-07	1.36E-05	ENSG00000143543	1	153974268	153977674	JTB
ENST00000271915	7.787286862	6.389180714	1.81459	3.521	0.0004299	0.0033787	ENSG00000143603	1	154697454	154870281	KCNN3
ENST00000272133	9.579765069	3.073172464	1.20393	2.5526	0.010692	0.0462117	ENSG00000143786	1	224616316	224740554	CNIH3
ENST00000272163	1818.196272	-1.489727166	0.17818	-8.361	6.23E-17	7.60E-15	ENSG00000143815	1	225401501	225428038	LBR
ENST00000272217	126.4050625	-1.162334078	0.32637	-3.561	0.0003689	0.0029671	ENSG00000143862	1	202133403	202144737	ARL8A
ENST00000272298	6836.135784	-1.13716538	0.17281	-6.581	4.69E-11	2.22E-09	ENSG00000143933	2	47160083	47176511	CALM2
ENST00000272321	93.6840375	-1.071764405	0.37178	-2.883	0.0039421	0.0208566	ENSG00000143951	2	63119558	63588477	WDPCP
ENST00000272322	984.1849224	-1.016787555	0.19243	-5.284	1.26E-07	2.84E-06	ENSG00000143952	2	63892149	64019428	VPS54
ENST00000272418	628.5424832	-1.325469221	0.21379	-6.2	5.66E-10	2.12E-08	ENSG00000144029	2	95085368	95121808	MRPS5
ENST00000272438	280.4156304	-2.103914966	0.25778	-8.162	3.30E-16	3.67E-14	ENSG00000144043	2	70985941	70994873	TEX261
ENST00000272519	742.1590411	-1.480697966	0.20229	-7.32	2.49E-13	1.88E-11	ENSG00000144118	2	120252851	120294710	RALB
ENST00000272521	79.10569864	-1.214937925	0.41367	-2.937	0.0033144	0.018131	ENSG00000144120	2	119679200	119682118	TMEM177
ENST00000272542	793.3277332	1.331366075	0.26228	5.0761	3.85E-07	7.72E-06	ENSG00000144136	2	112645938	112663825	SLC20A1
ENST00000272748	788.4612113	-1.463848997	0.23584	-6.207	5.40E-10	2.03E-08	ENSG00000144320	2	175923881	176002267	LNPK
ENST00000272895	442.6245576	-2.339397006	0.263	-8.895	5.84E-19	9.16E-17	ENSG00000144452	2	214931541	215138626	ABCA12
ENST00000273067	40.36243824	1.553360683	0.58381	2.6607	0.0077967	0.03592	ENSG00000144583	2	216257864	216372483	MARCHF4
ENST00000273153	468.4441098	1.107690656	0.22673	4.8855	1.03E-06	1.83E-05	ENSG00000144655	3	39141854	39153591	CSRNP1
ENST00000273375	429.683475	1.170930688	0.23171	5.0535	4.34E-07	8.58E-06	ENSG00000144840	3	120684937	120742516	RABL3
ENST00000273550	3537.683527	1.196315803	0.21675	5.5194	3.40E-08	8.75E-07	ENSG00000167996	11	61964284	61967634	FTH1
ENST00000273857	60.03643633	2.894856825	0.51453	5.6262	1.84E-08	5.06E-07	ENSG00000145244	4	47594000	47838067	CORIN
ENST00000274026	1670.573862	-2.137148288	0.19261	-11.1	1.31E-28	6.89E-26	ENSG00000145386	4	121816443	121823883	CCNA2
ENST00000274093	18.58393007	-2.888931435	0.90071	-3.207	0.0013395	0.0086735	ENSG00000145451	4	174636919	174829247	GLRA3
ENST00000274137	287.8600503	-1.275212358	0.33131	-3.849	0.0001186	0.0011345	ENSG00000145494	5	1801406	1816048	NDUFS6
ENST00000274217	56.86633327	-1.395771465	0.45637	-3.058	0.0022252	0.0131305	ENSG00000145569	5	14581791	14616180	OTULINL
ENST00000274255	1243.478149	-1.052468924	0.19733	-5.334	9.63E-08	2.23E-06	ENSG00000145604	5	36152110	36184319	SKP2
ENST00000274364	654.2010546	-2.758653795	0.23134	-11.92	8.81E-33	6.73E-30	ENSG00000145703	5	76403284	76708132	IQGAP2
ENST00000274458	144.1344603	-1.673772941	0.31716	-5.277	1.31E-07	2.93E-06	ENSG00000145781	5	116085024	116293287	COMMD10
ENST00000274764	5.060483278	5.768759615	1.9946	2.8922	0.0038256	0.020352	ENSG00000146047	6	25726776	25727345	H2BC1
ENST00000274773	50.95597972	-1.580617253	0.51966	-3.042	0.0023529	0.0137279	ENSG00000146054	5	181193923	181205196	TRIM7
ENST00000274813	437.2668543	1.073371546	0.23742	4.521	6.15E-06	8.80E-05	ENSG00000146085	6	49430359	49463253	MMUT
ENST00000275230	268.9889248	1.947281692	0.29744	6.5468	5.88E-11	2.70E-09	ENSG00000146411	6	133987580	134052624	SLC2A12
ENST00000276202	24.58711231	-3.735282204	0.83303	-4.484	7.33E-06	0.0001028	ENSG00000147251	X	118495814	118686147	DOCK11
ENST00000276569	981.5581852	-1.189827109	0.19989	-5.952	2.64E-09	8.76E-08	ENSG00000104442	8	65602457	65634177	ARMC1
ENST00000276654	28.44276139	-3.256017593	0.71897	-4.529	5.93E-06	8.54E-05	ENSG00000147650	8	104489235	104589258	LRP12
ENST00000277225	155.4045129	-4.097431311	0.40345	-10.16	3.12E-24	9.61E-22	ENSG00000148143	9	106863165	107013634	ZNF462

protein_coding	Yes	NM_199351.3	108
protein_coding	Yes	NM_145697.3	108
protein_coding	Yes	NM_031935.3	108
protein_coding	Yes	NM_005620.2	108
protein_coding	Yes	NM_019032.6	108
protein_coding	Yes	NM_006694.4	108
protein_coding	Yes	NM_002249.6	108
protein_coding	Yes	NM_152495.2	108
protein_coding	Yes	NM_002296.4	108
protein_coding	Yes	NM_138795.4	108
protein_coding	Yes	NM_001743.6	108
protein_coding	Yes	NM_015910.7	108
protein_coding	Yes	NM_016516.3	108
protein_coding	Yes	NM_031902.5	108
protein_coding	Yes	NM_144582.3	108
protein_coding	Yes	NM_002881.3	108
protein_coding	Yes	NM_030577.3	108
protein_coding	Yes	NM_005415.5	108
protein_coding	Yes	NM_030650.3	108
protein_coding	Yes	NM_173076.3	108
protein_coding	Yes	NM_020814.3	108
protein_coding	Yes	NM_033027.4	108
protein_coding	Yes	NM_173825.5	108
protein_coding	Yes	NM_002032.3	108
protein_coding	Yes	NM_006587.4	108
protein_coding	Yes	NM_001237.5	108
protein_coding	Yes	NM_006529.4	108
protein_coding	Yes	NM_004553.6	108
protein_coding	Yes	NM_019018.3	108
protein_coding	Yes	NM_005983.4	108
protein_coding	Yes	NM_006633.5	108
protein_coding	Yes	NM_016144.4	108
protein_coding	Yes	NM_170610.3	108
protein_coding	Yes	NM_203293.3	108
protein_coding	Yes	NM_000255.4	108
protein_coding	Yes	NM_145176.3	108
protein_coding	Yes	NM_144658.4	108
protein_coding	Yes	NM_018120.6	108
protein_coding	Yes	NM_013437.5	108
protein_coding	Yes	NM_021224.6	108

ENST00000278060	60.69817168	1.68804758	0.45064	3.7459	0.0001798	0.0016188	ENSG00000148832	10	133379261	133391694	PAOX
ENST00000278317	12.62317266	-7.132744071	1.68356	-4.237	2.27E-05	0.0002734	ENSG00000130595	11	1919702	1938702	TNNT3
ENST00000278353	790.3635231	-1.26729618	0.1976	-6.414	1.42E-10	6.02E-09	ENSG00000149084	11	43680737	43856615	HSD17B12
ENST00000278412	2722.9268	-2.073488908	0.21517	-9.636	5.62E-22	1.28E-19	ENSG00000149136	11	57325987	57335892	SSRP1
ENST00000278505	659.7513085	-2.510243139	0.21598	-11.62	3.17E-31	2.07E-28	ENSG00000149218	11	95089845	95132645	ENDOD1
ENST00000278903	1412.362106	-1.498842091	0.21823	-6.868	6.51E-12	3.74E-10	ENSG00000149547	11	125569476	125584684	EI24
ENST00000278937	741.5007855	-1.709350975	0.20857	-8.196	2.49E-16	2.81E-14	ENSG00000149573	11	118253415	118264297	MPZL2
ENST00000278980	281.5785964	-1.591783187	0.30937	-5.145	2.67E-07	5.61E-06	ENSG00000149600	20	32702698	32743467	COMMD7
ENST00000279249	181.5765611	1.391243922	0.29883	4.6556	3.23E-06	5.01E-05	ENSG00000149798	11	65314865	65322417	CDC42EP2
ENST00000279263	498.2492122	1.13236526	0.21948	5.1592	2.48E-07	5.23E-06	ENSG00000149809	11	65111871	65116230	TM7SF2
ENST00000279281	1055.280867	-1.183523939	0.19849	-5.963	2.48E-09	8.27E-08	ENSG00000149823	11	65096213	65111862	VPS51
ENST00000279396	543.9161708	1.045222525	0.21105	4.9524	7.33E-07	1.36E-05	ENSG00000149932	16	29962078	29973048	TMEM219
ENST00000279488	86.36903435	1.045696869	0.38903	2.688	0.007189	0.033709	ENSG00000139318	12	89347234	89352501	DUSP6
ENST00000280258	10131.65001	1.461064609	0.16585	8.8098	1.25E-18	1.87E-16	ENSG00000150687	11	86800541	86811233	PRSS23
ENST00000280350	69.27766775	2.089085077	0.44561	4.6881	2.76E-06	4.37E-05	ENSG00000150773	11	112067797	112074017	PIH1D2
ENST00000280481	41.72549355	-4.78590078	0.77025	-6.213	5.19E-10	1.95E-08	ENSG00000150893	13	38687076	38887131	FREM2
ENST00000280551	112.0906524	1.52711363	0.35342	4.321	1.55E-05	0.0001971	ENSG00000150961	4	118722822	118836126	SEC24D
ENST00000280571	133.4026153	1.406880615	0.34077	4.1285	3.65E-05	0.0004139	ENSG00000150977	12	123415038	123436684	RILPL2
ENST00000280665	12.46804384	-7.113771255	1.69731	-4.191	2.77E-05	0.0003256	ENSG00000151065	12	1946052	2004457	DCP1B
ENST00000280734	33.22257883	2.746757398	0.64056	4.2881	1.80E-05	0.0002239	ENSG00000151117	11	18698778	18704785	TMEM86A
ENST00000280800	135.0920625	1.338772365	0.38932	3.4387	0.0005844	0.0043763	ENSG00000151176	12	113358586	113391629	PLBD2
ENST00000280871	168.1974242	-2.668758508	0.33026	-8.081	6.44E-16	6.95E-14	ENSG00000151229	12	39755024	40106081	SLC2A13
ENST00000281172	556.2460772	-2.377309381	0.21784	-10.91	9.95E-28	4.65E-25	ENSG00000151491	12	15620133	15789388	EPS8
ENST00000281282	20.57798116	-3.724990813	0.91026	-4.092	4.27E-05	0.0004745	ENSG00000128849	15	57376504	57550717	CGNL1
ENST00000281441	470.7411727	1.652765337	0.22469	7.3557	1.90E-13	1.46E-11	ENSG00000151715	11	129815847	129860003	TMEM45B
ENST00000281471	243.455403	2.935967223	0.30159	9.735	2.14E-22	5.28E-20	ENSG00000151743	12	31671141	31729021	AMN1
ENST00000281513	817.6244549	-1.567639568	0.20103	-7.798	6.28E-15	5.92E-13	ENSG00000151779	2	15166915	15561334	NBAS
ENST00000281772	299.8842689	1.257426735	0.24581	5.1154	3.13E-07	6.44E-06	ENSG00000144445	2	210021420	210171409	KANSL1L
ENST00000281828	535.8224914	-1.446299888	0.23173	-6.241	4.34E-10	1.66E-08	ENSG00000116120	2	222566898	222656092	FARSB
ENST00000281834	87.95544269	1.5898317	0.38564	4.1225	3.75E-05	0.0004236	ENSG00000117586	1	173183730	173207331	TNFSF4
ENST00000281924	5.664152862	5.928166255	1.93769	3.0594	0.0022178	0.0130945	ENSG00000152128	2	134455758	134719000	TMEM163
ENST00000281938	137.1667999	1.686066145	0.40872	4.1252	3.70E-05	0.0004192	ENSG00000152137	12	119178930	119194746	HSPB8
ENST00000282041	537.8503059	1.322557615	0.22481	5.8829	4.03E-09	1.29E-07	ENSG00000152223	18	45847608	45967329	EPG5
ENST00000282074	155.2979144	-1.949785598	0.3129	-6.231	4.62E-10	1.76E-08	ENSG00000152253	2	168870890	168890430	SPC25
ENST00000282272	47.23132082	-2.816237232	0.55275	-5.095	3.49E-07	7.09E-06	ENSG00000065413	2	196986661	197310780	ANKRD44
ENST00000282441	4487.725393	-1.062519591	0.17957	-5.917	3.28E-09	1.06E-07	ENSG00000137693	11	102110446	102233424	YAP1
ENST00000282470	36.73570247	-1.556757947	0.57114	-2.726	0.0064165	0.0308395	ENSG00000152583	4	87473334	87529376	SPARCL1
ENST00000282541	262.0534818	-1.281931262	0.26699	-4.801	1.58E-06	2.67E-05	ENSG00000152642	3	32106619	32168709	GPD1L
ENST00000282633	604.9353371	1.086643485	0.21592	5.0326	4.84E-07	9.44E-06	ENSG00000099290	10	50067953	50133509	WASHC2A
ENST00000282869	335.5160894	1.25925708	0.32704	3.8505	0.0001179	0.0011285	ENSG00000152926	7	64971771	64991036	ZNF117

protein_coding	Yes	NM_152911.4	108
protein_coding	Yes	NM_006757.4	108
protein_coding	Yes	NM_016142.3	108
protein_coding	Yes	NM_003146.3	108
protein_coding	Yes	NM_015036.3	108
protein_coding	Yes	NM_004879.5	108
protein_coding	Yes	NM_005797.4	108
protein_coding	Yes	NM_053041.3	108
protein_coding	Yes	NM_006779.4	108
protein_coding	Yes	NM_003273.6	108
protein_coding	Yes	NM_013265.4	108
protein_coding	Yes	NM_001083613.2	108
protein_coding	Yes	NM_001946.4	108
protein_coding	Yes	NM_007173.6	108
protein_coding	Yes	NM_138789.4	108
protein_coding	Yes	NM_207361.6	108
protein_coding	Yes	NM_014822.4	108
protein_coding	Yes	NM_145058.3	108
protein_coding	Yes	NM_152640.5	108
protein_coding	Yes	NM_153347.3	108
protein_coding	Yes	NM_173542.4	108
protein_coding	Yes	NM_052885.4	108
protein_coding	Yes	NM_004447.6	108
protein_coding	Yes	NM_032866.5	108
protein_coding	Yes	NM_138788.5	108
protein_coding	Yes	NM_001113402.2	108
protein_coding	Yes	NM_015909.4	108
protein_coding	Yes	NM_152519.4	108
protein_coding	Yes	NM_005687.5	108
protein_coding	Yes	NM_003326.5	108
protein_coding	Yes	NM_030923.5	108
protein_coding	Yes	NM_014365.3	108
protein_coding	Yes	NM_020964.3	108
protein_coding	Yes	NM_020675.4	108
protein_coding	Yes	NM_001195144.2	108
protein_coding	Yes	NM_001130145.3	108
protein_coding	Yes	NM_004684.6	108
protein_coding	Yes	NM_015141.4	108
protein_coding	Yes	NM_001005751.3	108
protein_coding	Yes	-	108

ENST00000283109	237.2725866	-1.900371224	0.28895	-6.577	4.81E-11	2.27E-09	ENSG00000058729	5	97160866	97183247	RIOK2
ENST00000283441	107.4299909	1.620821057	0.37866	4.2804	1.87E-05	0.0002307	ENSG00000188818	5	795604	850986	ZDHHC11
ENST00000283871	18.58224963	3.257957555	0.89344	3.6465	0.0002658	0.0022606	ENSG00000113924	3	120628171	120682239	HGD
ENST00000283928	23.42055539	-3.108237916	0.77569	-4.007	6.15E-05	0.0006496	ENSG00000153814	7	27830576	28180795	JAZF1
ENST00000283946	92.52756895	-1.238385032	0.39976	-3.098	0.0019494	0.0117656	ENSG00000153832	2	229922502	230013119	FBXO36
ENST00000284031	675.1075375	-1.701056588	0.21055	-8.079	6.53E-16	7.02E-14	ENSG00000153904	1	85318484	85465159	DDAH1
ENST00000284136	237.3926145	-1.145040718	0.30915	-3.704	0.0002124	0.0018667	ENSG00000153993	7	84995552	85187056	SEMA3D
ENST00000284245	46.84558034	-1.321395817	0.5044	-2.62	0.0087997	0.0396302	ENSG00000154102	16	85707516	85751096	C16orf74
ENST00000284878	655.8595735	-1.011338641	0.20398	-4.958	7.12E-07	1.32E-05	ENSG00000154639	21	17513042	17570100	CXADR
ENST00000284881	312.5513116	-1.337770257	0.25558	-5.234	1.66E-07	3.63E-06	ENSG00000154642	21	17788973	17819356	C21orf91
ENST00000285039	363.5163946	-1.647406586	0.26346	-6.253	4.02E-10	1.55E-08	ENSG00000167306	18	49822788	50195147	MYO5B
ENST00000285046	10.23507793	6.783303863	1.73351	3.9131	9.11E-05	0.0009078	ENSG00000154783	3	14818961	14934571	FGD5
ENST00000285106	609.9685022	1.622980275	0.2157	7.5243	5.30E-14	4.43E-12	ENSG00000154832	18	50282346	50287692	CXXC1
ENST00000285238	148.5457323	1.099945417	0.30806	3.5706	0.0003562	0.0028797	ENSG00000108846	17	50634880	50692253	ABCC3
ENST00000285407	861.2456933	-1.066980384	0.22657	-4.709	2.49E-06	4.00E-05	ENSG00000155090	8	102648783	102655725	KLF10
ENST00000285667	572.9049861	-1.389771729	0.21459	-6.476	9.39E-11	4.16E-09	ENSG00000155304	21	14371114	14383146	HSPA13
ENST00000285697	259.3918161	-2.390764608	0.2674	-8.941	3.86E-19	6.21E-17	ENSG00000155330	16	46796602	46831180	C16orf87
ENST00000285737	1148.455457	-1.772403323	0.19258	-9.203	3.47E-20	6.15E-18	ENSG00000102910	16	48244299	48357349	LONP2
ENST00000285873	548.1415579	-1.118032317	0.22114	-5.056	4.29E-07	8.49E-06	ENSG00000082516	5	154887410	154938211	GEMIN5
ENST00000285947	68.15898374	-1.370615234	0.42409	-3.232	0.0012298	0.0080872	ENSG00000155542	5	56909595	56917348	SETD9
ENST00000285968	2429.936894	-1.894127196	0.17802	-10.64	1.94E-26	7.70E-24	ENSG00000155561	7	135557916	135648753	NUP205
ENST00000285979	20.43540809	1.90765847	0.74792	2.5506	0.010753	0.0464138	ENSG00000108242	10	94683728	94736190	CYP2C18
ENST00000286049	67.83643058	-1.740962112	0.42923	-4.056	4.99E-05	0.0005429	ENSG00000155622	X	52369020	52375680	XAGE2
ENST00000286070	143.7884543	-1.304432109	0.31595	-4.129	3.65E-05	0.0004138	ENSG00000155636	2	178112436	178129656	RBM45
ENST00000286201	509.0500805	1.137210061	0.23556	4.8276	1.38E-06	2.37E-05	ENSG00000155760	2	202033854	202038441	FZD7
ENST00000286544	136.2699328	2.114125918	0.33717	6.2702	3.61E-10	1.40E-08	ENSG00000156050	14	73932275	73950094	FAM161B
ENST00000286548	1251.578729	-1.547927733	0.18539	-8.35	6.84E-17	8.28E-15	ENSG00000156052	9	77716096	78031811	GNAQ
ENST00000286648	572.1291943	-1.117182169	0.21138	-5.285	1.26E-07	2.82E-06	ENSG00000156136	4	70993648	71030914	DCK
ENST00000286733	146.4320806	-2.51066839	0.33699	-7.45	9.31E-14	7.46E-12	ENSG00000138744	4	75913659	75941013	NAAA
ENST00000286760	191.0269935	1.084301427	0.28944	3.7462	0.0001795	0.0016172	ENSG00000156232	15	82809627	82836108	WHAMM
ENST00000287078	444.2546137	1.103504306	0.22202	4.9704	6.68E-07	1.25E-05	ENSG00000156521	10	70137980	70146700	TYSND1
ENST00000287097	8.584759444	3.984507129	1.45601	2.7366	0.0062078	0.0300167	ENSG00000156535	6	73696202	73828313	CD109
ENST00000287598	1589.205015	-1.150790603	0.20451	-5.627	1.83E-08	5.04E-07	ENSG00000156970	15	40161068	40221123	BUB1B
ENST00000287934	63.3723263	-2.583457294	0.48	-5.382	7.36E-08	1.76E-06	ENSG00000157240	7	91264432	91271326	FZD1
ENST00000287936	1589.511302	-1.293250127	0.18372	-7.039	1.93E-12	1.25E-10	ENSG00000113161	5	75337219	75362101	HMGCR
ENST00000288040	20.25782077	2.108164207	0.77831	2.7087	0.0067556	0.0321351	ENSG00000157322	16	69951333	69963986	CLEC18A
ENST00000288050	627.0995504	1.000197641	0.21563	4.6385	3.51E-06	5.38E-05	ENSG00000090857	16	70114331	70162537	PDPR
ENST00000288087	64.13164699	-1.734027029	0.45141	-3.841	0.0001223	0.0011642	ENSG00000213920	14	24213942	24216066	MDP1
ENST00000288532	313.6619567	-1.064664086	0.24286	-4.384	1.17E-05	0.0001536	ENSG00000110871	12	120503278	120529158	COQ5
ENST00000289166	485.8604898	1.472395024	0.23356	6.3043	2.90E-10	1.15E-08	ENSG00000158246	1	27005019	27012850	TENT5B

protein_coding	Yes	NM_018343.3	108
protein_coding	Yes	NM_024786.3	108
protein_coding	Yes	NM_000187.4	108
protein_coding	Yes	NM_175061.4	108
protein_coding	Yes	NM_174899.5	108
protein_coding	Yes	NM_012137.4	108
protein_coding	Yes	NM_001384900.1	108
protein_coding	Yes	NM_206967.3	108
protein_coding	Yes	NM_001338.5	108
protein_coding	Yes	NM_001100420.2	108
protein_coding	Yes	NM_001080467.3	108
protein_coding	Yes	NM_152536.4	108
protein_coding	Yes	NM_014593.4	108
protein_coding	Yes	NM_003786.4	108
protein_coding	Yes	NM_005655.4	108
protein_coding	Yes	NM_006948.5	108
protein_coding	Yes	NM_001001436.4	108
protein_coding	Yes	NM_031490.5	108
protein_coding	Yes	NM_015465.5	108
protein_coding	Yes	NM_153706.4	108
protein_coding	Yes	NM_015135.3	108
protein_coding	Yes	NM_000772.3	108
protein_coding	Yes	NM_130777.3	108
protein_coding	Yes	NM_152945.4	108
protein_coding	Yes	NM_003507.2	108
protein_coding	Yes	NM_152445.3	108
protein_coding	Yes	NM_002072.5	108
protein_coding	Yes	NM_000788.3	108
protein_coding	Yes	NM_014435.4	108
protein_coding	Yes	NM_001080435.3	108
protein_coding	Yes	NM_173555.4	108
protein_coding	Yes	NM_133493.5	108
protein_coding	Yes	NM_001211.6	108
protein_coding	Yes	NM_003505.2	108
protein_coding	Yes	NM_000859.3	108
protein_coding	Yes	NM_001370523.4	108
protein_coding	Yes	NM_017990.5	108
protein_coding	Yes	NM_138476.4	108
protein_coding	Yes	NM_032314.4	108
protein_coding	Yes	NM_052943.4	108

ENST00000289269	424.0686386	1.028118888	0.23302	4.4122	1.02E-05	0.0001371	ENSG00000243232	5	140966475	141012347	PCDHAC2
ENST00000289272	456.6374006	1.075745533	0.23287	4.6196	3.85E-06	5.83E-05	ENSG00000239389	5	140882123	141012347	PCDHA13
ENST00000289361	459.4229803	1.080502988	0.30001	3.6016	0.0003163	0.0026067	ENSG00000026950	6	26402274	26415208	BTN3A1
ENST00000289431	93.36591654	1.369047302	0.37283	3.6721	0.0002406	0.002075	ENSG00000158480	20	49903390	49915529	SPATA2
ENST00000289528	504.492493	1.080555189	0.22853	4.7282	2.27E-06	3.68E-05	ENSG00000158552	2	219206781	219209648	ZFAND2B
ENST00000290310	6.108066527	6.038185527	1.90263	3.1736	0.0015056	0.0095516	ENSG00000159197	21	34364005	34371381	KCNE2
ENST00000290354	59.08989217	-2.486037999	0.4954	-5.018	5.21E-07	1.01E-05	ENSG00000159231	21	36135078	36146562	CBR3
ENST00000290438	19.93495309	7.745418588	1.60304	4.8317	1.35E-06	2.33E-05	ENSG00000159289	15	74069856	74082550	GOLGA6A
ENST00000290510	96.53097952	1.045218614	0.36586	2.8569	0.0042782	0.0222922	ENSG00000110811	12	6828406	6839847	P3H3
ENST00000290551	15.07384027	-2.745437649	0.91903	-2.987	0.0028142	0.0158587	ENSG00000159388	1	203305518	203309602	BTG2
ENST00000290573	16.36596673	7.460133325	1.63268	4.5693	4.89E-06	7.22E-05	ENSG00000159399	2	74834126	74893359	HK2
ENST00000290649	1759.306397	-1.365771031	0.17823	-7.663	1.81E-14	1.62E-12	ENSG00000159461	16	56361451	56425545	AMFR
ENST00000290776	264.4760567	-1.201585359	0.26579	-4.521	6.16E-06	8.81E-05	ENSG00000140848	16	57092582	57148369	CPNE2
ENST00000290810	1104.630416	-1.207151263	0.19685	-6.132	8.66E-10	3.14E-08	ENSG00000159593	16	66802877	66830976	NAE1
ENST00000290902	105.4662136	-1.359051399	0.35373	-3.842	0.000122	0.0011615	ENSG00000159674	4	1166931	1172583	SPON2
ENST00000291539	14.26355558	-2.842533013	1.03533	-2.746	0.0060414	0.0293835	ENSG00000160191	21	42653620	42775509	PDE9A
ENST00000292147	374.7728231	-1.261878025	0.2373	-5.318	1.05E-07	2.40E-06	ENSG00000105755	19	43506718	43527201	ETHE1
ENST00000292430	353.428269	-2.04695703	0.26109	-7.84	4.50E-15	4.36E-13	ENSG00000160886	8	142700110	142705127	LY6K
ENST00000292475	751.8370079	-1.401142406	0.24457	-5.729	1.01E-08	2.94E-07	ENSG00000241468	7	99458194	99466167	ATP5MF
ENST00000292476	317.4692043	-1.026856097	0.24791	-4.142	3.44E-05	0.0003932	ENSG00000160917	7	99438942	99457373	CPSF4
ENST00000292494	3802.94671	-1.470819763	0.18904	-7.78	7.22E-15	6.74E-13	ENSG00000160932	8	143018528	143022409	LY6E
ENST00000292562	629.6022156	1.259574107	0.22407	5.6214	1.89E-08	5.18E-07	ENSG00000198169	8	144720908	144755531	ZNF251
ENST00000292616	433.8808575	1.078181033	0.2631	4.0979	4.17E-05	0.0004645	ENSG00000161036	7	102464983	102473168	LRWD1
ENST00000292778	190.8527528	-1.220449402	0.32379	-3.769	0.0001637	0.0014957	ENSG00000161179	22	21628088	21630022	YDJC
ENST00000292894	30.00147025	1.972918449	0.63921	3.0865	0.0020254	0.0121458	ENSG00000161277	19	36034983	36054285	THAP8
ENST00000292928	106.787827	1.868430264	0.4387	4.259	2.05E-05	0.0002508	ENSG00000161298	19	36605312	36634114	ZNF382
ENST00000293261	33.60571943	1.541038541	0.58343	2.6413	0.0082579	0.0376677	ENSG00000161558	19	48332355	48363940	TMEM143
ENST00000293303	4.172655949	5.487752313	2.08271	2.6349	0.0084159	0.0382366	ENSG00000161594	17	41837824	41848384	KLHL10
ENST00000293379	196.5224742	-1.10347784	0.30155	-3.659	0.0002528	0.002166	ENSG00000161638	12	54395260	54419266	ITGA5
ENST00000293406	187.2213096	-1.090367467	0.28355	-3.845	0.0001204	0.0011488	ENSG00000161654	17	44034327	44066671	LSM12
ENST00000293443	37.31771933	-2.025453952	0.58154	-3.483	0.0004959	0.0038117	ENSG00000161682	17	44353214	44363853	FAM171A2
ENST00000293778	234.9618024	1.582630432	0.28364	5.5797	2.41E-08	6.42E-07	ENSG00000161921	17	4733532	4739928	CXCL16
ENST00000293826	77.21813081	1.779428369	0.40716	4.3704	1.24E-05	0.0001623	ENSG00000248871	17	7549098	7561601	TNFSF12-TNFSF13
ENST00000293829	109.1081676	1.87162568	0.40877	4.5787	4.68E-06	6.94E-05	ENSG00000161958	17	7439511	7444937	FGF11
ENST00000294072	755.8720431	1.256419914	0.20875	6.0188	1.76E-09	6.06E-08	ENSG00000162144	11	61348804	61361882	CYB561A3
ENST00000294172	509.5481351	1.385450815	0.21732	6.3752	1.83E-10	7.57E-09	ENSG00000162231	11	62792129	62805440	NXF1
ENST00000294258	493.4766299	1.11475859	0.21956	5.0773	3.83E-07	7.68E-06	ENSG00000162300	11	65084221	65088398	ZFPL1
ENST00000294785	447.3791939	1.173382553	0.23273	5.0418	4.61E-07	9.05E-06	ENSG00000162736	1	160343382	160358949	NCSTN
ENST00000294889	177.3192361	-5.140352471	0.43052	-11.94	7.32E-33	5.71E-30	ENSG00000162817	1	220690362	220699153	C1orf115
ENST00000295030	611.5449665	-1.922498769	0.21659	-8.876	6.91E-19	1.06E-16	ENSG00000162928	2	61017719	61051990	PEX13

protein_coding	Yes	NM_018899.6	108
protein_coding	Yes	NM_018904.3	108
protein_coding	Yes	NM_007048.6	108
protein_coding	Yes	NM_006038.4	108
protein_coding	Yes	NM_138802.3	108
protein_coding	Yes	NM_172201.2	108
protein_coding	Yes	NM_001236.4	108
protein_coding	Yes	NM_001038640.2	108
protein_coding	Yes	NM_014262.5	108
protein_coding	Yes	NM_006763.3	108
protein_coding	Yes	NM_000189.5	108
protein_coding	Yes	NM_001144.6	108
protein_coding	Yes	NM_152727.6	108
protein_coding	Yes	NM_003905.4	108
protein_coding	Yes	NM_012445.4	108
protein_coding	Yes	NM_002606.3	108
protein_coding	Yes	NM_014297.5	108
protein_coding	Yes	NM_017527.4	108
protein_coding	Yes	NM_004889.5	108
protein_coding	Yes	NM_006693.4	108
protein_coding	Yes	NM_002346.3	108
protein_coding	Yes	NM_138367.2	108
protein_coding	Yes	NM_152892.3	108
protein_coding	Yes	NM_001017964.2	108
protein_coding	Yes	NM_152658.3	108
protein_coding	Yes	NM_032825.5	108
protein_coding	Yes	NM_018273.4	108
protein_coding	Yes	NM_152467.5	108
protein_coding	Yes	NM_002205.5	108
protein_coding	Yes	NM_001371445.1	108
protein_coding	Yes	NM_198475.3	108
protein_coding	Yes	NM_001386809.1	108
protein_coding	Yes	-	108
protein_coding	Yes	NM_004112.4	108
protein_coding	Yes	NM_153611.6	108
protein_coding	Yes	NM_006362.5	108
protein_coding	Yes	NM_006782.4	108
protein_coding	Yes	NM_015331.3	108
protein_coding	Yes	NM_024709.5	108
protein_coding	Yes	NM_002618.4	108

ENST00000295190	36.82394135	-2.468529806	0.59657	-4.138	3.51E-05	0.0003996	ENSG00000163053	2	230034981	230068924	SLC16A14
ENST00000295304	60.02003507	-2.411376668	0.4843	-4.979	6.39E-07	1.20E-05	ENSG00000143942	2	53767803	53775196	CHAC2
ENST00000295448	104.7569975	1.227379331	0.35369	3.4702	0.0005201	0.0039651	ENSG00000163281	4	44701794	44726556	GNPDA2
ENST00000295453	80.71229981	2.604807023	0.43239	6.0242	1.70E-09	5.87E-08	ENSG00000163286	2	232406843	232410714	ALPG
ENST00000295461	313.5398716	-2.294762433	0.25707	-8.926	4.40E-19	7.02E-17	ENSG00000163293	4	48016771	48040173	NIPAL1
ENST00000295770	5052.359349	-1.030871342	0.17039	-6.05	1.45E-09	5.07E-08	ENSG00000163527	3	31532924	31637616	STT3B
ENST00000295899	253.053693	-1.577340365	0.27599	-5.715	1.10E-08	3.17E-07	ENSG00000163634	3	63833869	63863805	THOC7
ENST00000296026	3.916465705	5.396742222	2.11677	2.5495	0.0107872	0.0464746	ENSG00000163734	4	74036588	74038689	CXCL3
ENST00000296233	20.02624219	7.751353633	1.60097	4.8417	1.29E-06	2.23E-05	ENSG00000163884	3	126342634	126357408	KLF15
ENST00000296318	98.63175735	-1.03895628	0.36364	-2.857	0.0042754	0.0222819	ENSG00000144730	3	57089981	57165353	IL17RD
ENST00000296358	4.337557096	5.546371557	2.07077	2.6784	0.0073974	0.034454	ENSG00000163982	4	4188725	4226929	OTOP1
ENST00000296417	5078.847911	-1.038195211	0.19381	-5.357	8.47E-08	1.99E-06	ENSG00000164032	4	99948087	99950275	H2AZ1
ENST00000296474	283.1811814	1.314401706	0.26213	5.0143	5.32E-07	1.03E-05	ENSG00000164078	3	49887001	49903873	MST1R
ENST00000296498	182.7316974	-1.373162038	0.37091	-3.702	0.0002138	0.001877	ENSG00000164099	4	118280037	118353003	PRSS12
ENST00000296503	1781.62589	-1.799983723	0.18196	-9.892	4.50E-23	1.23E-20	ENSG00000164104	4	173331375	173334358	HMGB2
ENST00000296504	191.6508933	1.292650992	0.30935	4.1786	2.93E-05	0.0003415	ENSG00000164105	4	173370953	173377532	SAP30
ENST00000296509	1180.476845	-1.097204508	0.19391	-5.658	1.53E-08	4.29E-07	ENSG00000164109	4	120055622	120066848	MAD2L1
ENST00000296550	37.63053329	-2.658385047	0.60356	-4.405	1.06E-05	0.0001413	ENSG00000170390	4	150078444	150257438	DCLK2
ENST00000296581	182.4141502	-1.462699528	0.29211	-5.007	5.52E-07	1.06E-05	ENSG00000164167	4	146175717	146191535	LSM6
ENST00000296603	587.7120119	-1.406953946	0.25717	-5.471	4.48E-08	1.12E-06	ENSG00000164187	5	36098406	36151887	LMBRD2
ENST00000296684	400.6663017	-1.404423619	0.2279	-6.162	7.17E-10	2.64E-08	ENSG00000164258	5	53560638	53683338	NDUFS4
ENST00000296741	4.149833674	5.480651158	2.08437	2.6294	0.0085535	0.0387013	ENSG00000164303	4	184088705	184217873	ENPP6
ENST00000296930	9800.270691	-1.002028778	0.18721	-5.352	8.68E-08	2.03E-06	ENSG00000181163	5	171387848	171410900	NPM1
ENST00000296978	382.3883151	-6.898323994	0.47292	-14.59	3.42E-48	1.14E-44	ENSG00000164484	6	130366016	130443067	TMEM200A
ENST00000297012	6.620447014	6.153868073	1.87202	3.2873	0.0010116	0.0068826	ENSG00000164508	6	25726062	25726562	H2AC1
ENST00000297071	591.6236958	-1.111964856	0.20891	-5.323	1.02E-07	2.35E-06	ENSG00000164548	7	23504779	23531981	TRA2A
ENST00000297164	245.8443359	2.693972872	0.28389	9.4895	2.32E-21	4.92E-19	ENSG00000164620	5	141636996	141641064	RELL2
ENST00000297494	90.03375539	5.113718621	0.58367	8.7613	1.93E-18	2.83E-16	ENSG00000164867	7	150991016	151014588	NOS3
ENST00000297632	436.5884346	-1.386014714	0.26488	-5.233	1.67E-07	3.66E-06	ENSG00000164983	8	124306188	124372701	TMEM65
ENST00000297668	7.384504626	-4.856360459	1.81353	-2.678	0.0074097	0.0344957	ENSG00000106714	9	39064709	39288167	CNTNAP3
ENST00000297875	13.12862007	-7.189378827	1.67514	-4.292	1.77E-05	0.0002206	ENSG00000147041	X	38006552	38128816	SYTL5
ENST00000297991	725.8495444	-1.739081098	0.29697	-5.856	4.74E-09	1.49E-07	ENSG00000165272	9	33441159	33447593	AQP3
ENST00000298105	4.36037937	5.553185115	2.06176	2.6934	0.0070723	0.0332879	ENSG00000250878	13	102891310	102895685	METTL21EP
ENST00000298130	406.3554416	-2.211454915	0.23709	-9.328	1.08E-20	2.08E-18	ENSG00000165389	14	34432787	34462240	SPTSSA
ENST00000298159	537.3514448	-1.789113075	0.21456	-8.338	7.53E-17	9.06E-15	ENSG00000165410	14	34709112	34714593	CFL2
ENST00000298288	350.7968571	-1.052003705	0.27506	-3.825	0.000131	0.0012352	ENSG00000165501	14	49598939	49614672	LRR1
ENST00000298299	253.020398	-3.237720316	0.36288	-8.922	4.57E-19	7.27E-17	ENSG00000165512	10	45000922	45005326	ZNF22
ENST00000298316	2668.336585	-1.384022655	0.18467	-7.495	6.65E-14	5.46E-12	ENSG00000165527	14	49893081	49897054	ARF6
ENST00000298838	623.9861021	-1.179434144	0.21556	-5.472	4.46E-08	1.12E-06	ENSG00000165912	11	47177521	47186434	PACSIN3
ENST00000298894	194.2540231	1.000417848	0.28093	3.5611	0.0003692	0.0029696	ENSG00000165943	14	93182198	93184897	MOAP1

protein_coding	Yes	NM_152527.5	108
protein_coding	Yes	NM_001008708.4	108
protein_coding	Yes	NM_138335.3	108
protein_coding	Yes	NM_031313.3	108
protein_coding	Yes	NM_207330.3	108
protein_coding	Yes	NM_178862.3	108
protein_coding	Yes	NM_025075.4	108
protein_coding	Yes	NM_002090.3	108
protein_coding	Yes	NM_014079.4	108
protein_coding	Yes	NM_017563.5	108
protein_coding	Yes	NM_177998.3	108
protein_coding	Yes	NM_002106.4	108
protein_coding	Yes	NM_002447.4	108
protein_coding	Yes	NM_003619.4	108
protein_coding	Yes	NM_002129.4	108
protein_coding	Yes	NM_003864.4	108
protein_coding	Yes	NM_002358.4	108
protein_coding	Yes	NM_001040260.4	108
protein_coding	Yes	NM_007080.3	108
protein_coding	Yes	NM_001007527.2	108
protein_coding	Yes	NM_002495.4	108
protein_coding	Yes	NM_153343.4	108
protein_coding	Yes	NM_002520.7	108
protein_coding	Yes	NM_001258277.2	108
protein_coding	Yes	NM_170745.3	108
protein_coding	Yes	NM_013293.5	108
protein_coding	Yes	NM_173828.5	108
protein_coding	Yes	NM_000603.5	108
protein_coding	Yes	NM_194291.3	108
protein_coding	Yes	NM_033655.5	108
protein_coding	Yes	NM_138780.3	108
protein_coding	Yes	NM_004925.5	108
ed_unprocessed_ps	Yes	-	108
protein_coding	Yes	NM_138288.4	108
protein_coding	Yes	NM_138638.5	108
protein_coding	Yes	NM_152329.4	108
protein_coding	Yes	NM_006963.5	108
protein_coding	Yes	NM_001663.4	108
protein_coding	Yes	NM_016223.5	108
protein_coding	Yes	NM_022151.5	108

ENST00000298912	46.22939882	-2.338372021	0.60218	-3.883	0.0001031	0.0010071	ENSG00000165959	14	95181939	95319908	CLMN
ENST00000299045	93.85524582	1.440769252	0.44909	3.2082	0.0013358	0.008654	ENSG00000166046	12	106302732	106347003	TCP11L2
ENST00000299084	626.9535556	-1.924154819	0.20913	-9.201	3.56E-20	6.29E-18	ENSG00000166068	15	38252835	38357249	SPRED1
ENST00000299138	2632.516784	-1.221151122	0.17243	-7.082	1.42E-12	9.39E-11	ENSG00000069329	16	46656131	46689178	VPS35
ENST00000299157	185.1500523	-1.746636892	0.30241	-5.776	7.66E-09	2.31E-07	ENSG00000166130	12	98624248	98644788	IKBIP
ENST00000299174	5.106127827	5.780412549	1.98129	2.9175	0.0035284	0.0190517	ENSG00000166143	15	40815450	40828708	PPP1R14D
ENST00000299192	560.2676191	-1.0046761	0.22039	-4.559	5.15E-06	7.55E-05	ENSG00000155393	16	50065969	50107272	HEATR3
ENST00000299335	342.8971833	-1.040655824	0.23658	-4.399	1.09E-05	0.0001448	ENSG00000166260	17	54960421	54968654	COX11
ENST00000299353	378.4213629	1.743016463	0.23561	7.398	1.38E-13	1.09E-11	ENSG00000270316	10	102854271	102901899	BORCS7-ASMT
ENST00000299427	106.6094115	1.284633626	0.42095	3.0517	0.0022754	0.0133612	ENSG00000166340	11	6612767	6619422	TPP1
ENST00000299440	197.3424447	-1.345741671	0.32635	-4.124	3.73E-05	0.0004219	ENSG00000166349	11	36568006	36579762	RAG1
ENST00000299492	35.84714132	-1.567255231	0.56425	-2.778	0.0054763	0.0271754	ENSG00000166387	11	7513998	7653756	PPFIBP2
ENST00000299578	35.82293822	1.904657501	0.58103	3.2781	0.0010452	0.0070642	ENSG00000166455	16	81060979	81077238	C16orf46
ENST00000299601	470.8994681	-1.375341092	0.23006	-5.978	2.26E-09	7.57E-08	ENSG00000166477	15	51938024	51971778	LEO1
ENST00000300030	721.4548075	-1.056092859	0.23283	-4.536	5.74E-06	8.29E-05	ENSG00000166797	15	64072564	64093838	CIAO2A
ENST00000300035	983.52896	-1.450367242	0.19639	-7.385	1.52E-13	1.19E-11	ENSG00000166803	15	64364303	64381441	PCLAF
ENST00000300055	17.00664079	2.507468156	0.87011	2.8818	0.0039543	0.0209114	ENSG00000166819	15	89664366	89679367	PLIN1
ENST00000300093	340.2120235	-2.002150271	0.2667	-7.507	6.04E-14	5.01E-12	ENSG00000166851	16	23678888	23690367	PLK1
ENST00000300101	194.8956427	-1.25251458	0.2814	-4.451	8.55E-06	0.0001175	ENSG00000166860	12	56998835	57006546	ZBTB39
ENST00000300119	22.67873304	1.891253646	0.71561	2.6429	0.0082209	0.0375337	ENSG00000166866	12	57028516	57050129	MYO1A
ENST00000300258	41.08829495	2.027066204	0.54559	3.7154	0.0002029	0.0017956	ENSG00000242220	21	32573720	32585523	TCP10L
ENST00000300291	3052.433467	-2.417959064	0.17246	-14.02	1.17E-44	3.25E-41	ENSG00000167005	16	56429132	56451332	NUDT21
ENST00000300403	2534.766247	-1.066804329	0.1868	-5.711	1.12E-08	3.25E-07	ENSG00000088325	20	31739289	31801800	TPX2
ENST00000300413	791.8557619	-1.547985948	0.23495	-6.589	4.44E-11	2.11E-09	ENSG00000167088	18	21612313	21633520	SNRPD1
ENST00000300456	552.2958987	-1.236107001	0.2239	-5.521	3.37E-08	8.69E-07	ENSG00000167114	9	128340526	128361470	SLC27A4
ENST00000300557	102.2941367	-1.078326257	0.36434	-2.96	0.00308	0.0170554	ENSG00000167183	17	47951966	47957883	PRR15L
ENST00000300571	46.35030216	-2.160159516	0.53258	-4.056	4.99E-05	0.0005429	ENSG00000167191	16	19856690	19884848	GPRC5B
ENST00000301071	335.90802	-1.669792912	0.25455	-6.56	5.39E-11	2.51E-09	ENSG00000167552	12	49184794	49189080	TUBA1A
ENST00000301178	1006.908979	-1.423770021	0.21055	-6.762	1.36E-11	7.26E-10	ENSG00000167601	19	41219222	41261766	AXL
ENST00000301263	22.71283858	-2.042213304	0.73172	-2.791	0.005255	0.0263287	ENSG00000167656	8	142784881	142786539	LY6D
ENST00000301335	197.3356863	2.387422327	0.31726	7.5251	5.27E-14	4.41E-12	ENSG00000167703	17	1569267	1628834	SLC43A2
ENST00000301452	4.616569613	5.635108028	2.03024	2.7756	0.0055102	0.0273019	ENSG00000167769	19	6306141	6333612	ACER1
ENST00000301459	31.09015211	-3.549859773	0.71949	-4.934	8.06E-07	1.47E-05	ENSG00000167771	11	63911229	63917164	RCOR2
ENST00000301480	9.910420861	6.737973712	1.74829	3.854	0.0001162	0.0011142	ENSG00000198028	19	9466354	9498616	ZNF560
ENST00000301585	237.318477	-1.463740712	0.26951	-5.431	5.60E-08	1.37E-06	ENSG00000167862	17	75012669	75021261	MRPL58
ENST00000301686	165.9088689	-1.096970607	0.29768	-3.685	0.0002287	0.0019871	ENSG00000130731	16	634429	636305	METTL26
ENST00000301873	782.249951	1.070672778	0.2133	5.0196	5.18E-07	1.00E-05	ENSG00000168056	11	65538558	65558359	LTBP3
ENST00000301891	14.64597905	-3.436089333	1.02885	-3.34	0.0008385	0.0059015	ENSG00000168065	11	64555940	64572875	SLC22A11
ENST00000301905	563.0249763	-2.429799238	0.21682	-11.21	3.78E-29	2.12E-26	ENSG00000168078	8	27809623	27837817	PBK
ENST00000302060	76.29403343	-1.579237457	0.43016	-3.671	0.0002413	0.0020804	ENSG00000170464	5	139410202	139439525	DNAJC18

protein_coding	Yes	NM_024734.4	108
protein_coding	Yes	NM_152772.3	108
protein_coding	Yes	NM_152594.3	108
protein_coding	Yes	NM_018206.6	108
protein_coding	Yes	NM_153687.4	108
protein_coding	Yes	NM_017726.8	108
protein_coding	Yes	NM_182922.4	108
protein_coding	Yes	NM_004375.5	108
protein_coding	Yes	-	108
protein_coding	Yes	NM_000391.4	108
protein_coding	Yes	NM_000448.3	108
protein_coding	Yes	NM_003621.5	108
protein_coding	Yes	NM_152337.3	108
protein_coding	Yes	NM_138792.4	108
protein_coding	Yes	NM_032231.7	108
protein_coding	Yes	NM_014736.6	108
protein_coding	Yes	NM_002666.5	108
protein_coding	Yes	NM_005030.6	108
protein_coding	Yes	NM_014830.3	108
protein_coding	Yes	NM_005379.4	108
protein_coding	Yes	NM_144659.7	108
protein_coding	Yes	NM_007006.3	108
protein_coding	Yes	NM_012112.5	108
protein_coding	Yes	NM_006938.4	108
protein_coding	Yes	NM_005094.4	108
protein_coding	Yes	NM_024320.4	108
protein_coding	Yes	NM_016235.3	108
protein_coding	Yes	NM_006009.4	108
protein_coding	Yes	NM_021913.5	108
protein_coding	Yes	NM_003695.3	108
protein_coding	Yes	NM_152346.3	108
protein_coding	Yes	NM_133492.3	108
protein_coding	Yes	NM_173587.4	108
protein_coding	Yes	NM_152476.3	108
protein_coding	Yes	NM_001545.3	108
protein_coding	Yes	NM_032366.5	108
protein_coding	Yes	NM_001130144.3	108
protein_coding	Yes	NM_018484.4	108
protein_coding	Yes	NM_018492.4	108
protein_coding	Yes	NM_152686.4	108

ENST00000302118	17.62559618	-7.61473449	1.62258	-4.693	2.69E-06	4.29E-05	ENSG00000169174	1	55039547	55064852	PCSK9
ENST00000302236	216.3915458	1.025347707	0.27175	3.7732	0.0001612	0.0014763	ENSG00000168152	4	82900734	82919969	THAP9
ENST00000302271	2510.190504	-1.453419644	0.18542	-7.838	4.56E-15	4.42E-13	ENSG00000125944	1	23304687	23344284	HNRNPR
ENST00000302450	463.0137922	-1.44471093	0.25226	-5.727	1.02E-08	2.98E-07	ENSG00000169607	2	112736348	112764609	CKAP2L
ENST00000302586	90.39266191	1.771898219	0.38172	4.6419	3.45E-06	5.31E-05	ENSG00000171097	9	128832941	128881950	KYAT1
ENST00000302628	39.27359345	1.795600703	0.57739	3.1099	0.0018718	0.0113841	ENSG00000172137	16	71358722	71390433	CALB2
ENST00000302631	400.4436676	1.232565398	0.23078	5.3409	9.25E-08	2.15E-06	ENSG00000116521	1	155255980	155262360	SCAMP3
ENST00000302754	825.6404834	1.221151435	0.21279	5.7387	9.54E-09	2.80E-07	ENSG00000171223	19	12791485	12793315	JUNB
ENST00000303142	45.64620737	2.216088426	0.52584	4.2144	2.50E-05	0.0002978	ENSG00000171984	20	5750400	5864395	SHLD1
ENST00000303251	163.9700392	2.273568231	0.31639	7.1859	6.68E-13	4.67E-11	ENSG00000169228	5	177301197	177303719	RAB24
ENST00000303375	62.5659015	-3.823577352	0.5645	-6.773	1.26E-11	6.77E-10	ENSG00000011028	17	62627669	62693597	MRC2
ENST00000303383	448.4442101	-2.519814551	0.23289	-10.82	2.77E-27	1.22E-24	ENSG00000171241	16	46578590	46621379	SHCBP1
ENST00000303460	61.70713627	2.732991621	0.48061	5.6865	1.30E-08	3.70E-07	ENSG00000180818	12	53985145	53990279	HOXC10
ENST00000303562	154.5029074	-1.110545452	0.35219	-3.153	0.0016146	0.0101049	ENSG00000170345	14	75278827	75282230	FOS
ENST00000303775	222.4260211	1.331978799	0.27196	4.8976	9.70E-07	1.73E-05	ENSG00000156239	21	28872190	28885367	N6AMT1
ENST00000303892	10.74745841	6.853433659	1.72068	3.983	6.81E-05	0.00071	ENSG00000213760	6	31544443	31546608	ATP6V1G2
ENST00000303910	2248.797902	-1.402106466	0.17685	-7.928	2.22E-15	2.23E-13	ENSG00000277075	6	26216920	26217437	H2AC8
ENST00000303921	42.407892	-3.739409109	0.64316	-5.814	6.10E-09	1.87E-07	ENSG00000170775	7	124743884	124765792	GPR37
ENST00000304116	99.08289266	1.682440215	0.36927	4.5562	5.21E-06	7.62E-05	ENSG00000196357	19	36182275	36214665	ZNF565
ENST00000304195	67.84748736	1.627606109	0.4455	3.6534	0.0002587	0.0022104	ENSG00000172159	9	83244621	83538401	FRMD3
ENST00000304218	5812.506024	-1.197817535	0.16617	-7.208	5.67E-13	4.01E-11	ENSG00000168298	6	26156328	26157115	H1-4
ENST00000304222	200.055923	-2.268045217	0.29923	-7.58	3.47E-14	2.95E-12	ENSG00000170425	17	15945129	15975746	ADORA2B
ENST00000304283	213.5674885	1.205374021	0.30055	4.0106	6.06E-05	0.0006417	ENSG00000168096	16	4696510	4734271	ANKS3
ENST00000304330	424.5431832	1.059911686	0.22304	4.7521	2.01E-06	3.31E-05	ENSG00000103932	15	41517175	41544257	RPAP1
ENST00000304414	6641.178472	-1.421950037	0.17404	-8.17	3.08E-16	3.43E-14	ENSG00000170540	16	18791668	18801549	ARL6IP1
ENST00000304494	68.05898261	-1.411639097	0.42292	-3.338	0.0008443	0.0059342	ENSG00000147889	9	21967751	21974857	CDKN2A
ENST00000304552	42.26994726	7.376742962	1.51446	4.8709	1.11E-06	1.95E-05	ENSG00000172215	3	45943463	45948351	CXCR6
ENST00000304567	1725.823289	-1.413939106	0.19317	-7.32	2.49E-13	1.88E-11	ENSG00000171848	2	10122738	10131414	RRM2
ENST00000304698	1901.547007	-1.865529669	0.2174	-8.581	9.40E-18	1.28E-15	ENSG00000144369	2	186694059	186765959	FAM171B
ENST00000304735	687.419034	1.506143985	0.22814	6.602	4.06E-11	1.95E-09	ENSG00000168140	16	4371847	4383538	VASN
ENST00000304749	8.888536434	4.069872663	1.47057	2.7676	0.0056479	0.0278337	ENSG00000170373	20	23747561	23750935	CST1
ENST00000304786	3263.657571	-1.395249275	0.17711	-7.878	3.33E-15	3.29E-13	ENSG00000143742	1	225777825	225790464	SRP9
ENST00000304800	328.2939595	1.10097735	0.24395	4.5131	6.39E-06	9.09E-05	ENSG00000168701	16	67227129	67229278	TMEM208
ENST00000304952	35.36113322	-2.265989364	0.63231	-3.584	0.0003388	0.002765	ENSG00000188290	1	998963	1000097	HES4
ENST00000305123	481.1732552	1.152563735	0.21714	5.308	1.11E-07	2.52E-06	ENSG00000169047	2	226731311	226799820	IRS1
ENST00000305202	4.172655949	5.487752313	2.08271	2.6349	0.0084159	0.0382366	ENSG00000103154	16	83968243	84002776	NECAB2
ENST00000305352	16.21500369	3.577918279	1.01164	3.5368	0.0004051	0.003213	ENSG00000170989	1	101237018	101241518	S1PR1
ENST00000305428	262.3353934	2.399599908	0.28247	8.4952	1.98E-17	2.58E-15	ENSG00000169330	15	79432335	79472304	MINAR1
ENST00000305494	652.9156329	-1.237367154	0.26445	-4.679	2.88E-06	4.55E-05	ENSG00000166575	11	87037933	87328824	TMEM135
ENST00000305560	372.5505185	1.113727036	0.3136	3.5514	0.0003832	0.0030658	ENSG00000171763	15	45402335	45421415	SPATA5L1

protein_coding	Yes	NM_174936.4	108
protein_coding	Yes	NM_024672.6	108
protein_coding	Yes	NM_005826.5	108
protein_coding	Yes	NM_152515.5	108
protein_coding	Yes	NM_004059.5	108
protein_coding	Yes	NM_001740.5	108
protein_coding	Yes	NM_005698.4	108
protein_coding	Yes	NM_002229.3	108
protein_coding	Yes	NM_152504.4	108
protein_coding	Yes	NM_001031677.4	108
protein_coding	Yes	NM_006039.5	108
protein_coding	Yes	NM_024745.5	108
protein_coding	Yes	NM_017409.4	108
protein_coding	Yes	NM_005252.4	108
protein_coding	Yes	NM_013240.6	108
protein_coding	Yes	NM_130463.4	108
protein_coding	Yes	NM_021052.4	108
protein_coding	Yes	NM_005302.5	108
protein_coding	Yes	NM_152477.5	108
protein_coding	Yes	NM_174938.6	108
protein_coding	Yes	NM_005321.3	108
protein_coding	Yes	NM_000676.4	108
protein_coding	Yes	NM_133450.4	108
protein_coding	Yes	NM_015540.4	108
protein_coding	Yes	NM_015161.3	108
protein_coding	Yes	NM_000077.5	108
protein_coding	Yes	NM_006564.2	108
protein_coding	Yes	NM_001034.4	108
protein_coding	Yes	NM_177454.4	108
protein_coding	Yes	NM_138440.3	108
protein_coding	Yes	NM_001898.3	108
protein_coding	Yes	NM_003133.6	108
protein_coding	Yes	NM_014187.4	108
protein_coding	Yes	NM_021170.4	108
protein_coding	Yes	NM_005544.3	108
protein_coding	Yes	NM_019065.3	108
protein_coding	Yes	NM_001400.5	108
protein_coding	Yes	NM_015206.3	108
protein_coding	Yes	NM_022918.4	108
protein_coding	Yes	NM_024063.3	108

ENST00000305709	43.69821864	-1.84125247	0.52439	-3.511	0.0004461	0.0034867	ENSG00000170161	9	62897448	62900104	FAM88B
ENST00000305747	735.043287	-3.297340534	0.24695	-13.35	1.15E-40	2.50E-37	ENSG00000172292	2	168456271	168775134	CERS6
ENST00000305784	444.6668192	-1.193701165	0.25169	-4.743	2.11E-06	3.45E-05	ENSG00000168393	2	241675746	241686815	DTYMK
ENST00000305786	2317.894758	-1.319089867	0.19637	-6.717	1.85E-11	9.61E-10	ENSG00000172115	7	25118655	25125260	CYCS
ENST00000305883	71.38084884	-2.043073848	0.42841	-4.769	1.85E-06	3.08E-05	ENSG00000172059	2	10043549	10054836	KLF11
ENST00000306072	166.9102859	2.834339376	0.3318	8.5424	1.31E-17	1.76E-15	ENSG00000172183	15	88639015	88656483	ISG20
ENST00000306100	4.690181664	5.661923415	2.16519	2.615	0.0089233	0.0400604	ENSG00000168843	4	161383896	162164000	FSTL5
ENST00000306121	18.99678246	-6.25670033	1.59137	-3.932	8.44E-05	0.0008515	ENSG00000162627	1	98661720	98760500	SNX7
ENST00000306243	70.18134397	-1.520375343	0.42105	-3.611	0.0003051	0.00253	ENSG00000169105	15	40470983	40473158	CHST14
ENST00000306357	136.8498974	-1.582139651	0.32913	-4.807	1.53E-06	2.60E-05	ENSG00000170310	17	9250470	9575820	STX8
ENST00000306390	81.83031895	2.088047608	0.42657	4.895	9.83E-07	1.76E-05	ENSG00000171236	19	4536401	4540036	LRG1
ENST00000306406	4.795244093	-5.736391506	2.01115	-2.852	0.0043405	0.0225418	ENSG00000171227	2	119431853	119438504	TMEM37
ENST00000306467	548.6466029	-1.030155519	0.21099	-4.882	1.05E-06	1.86E-05	ENSG00000168944	5	123344891	123423403	CEP120
ENST00000306601	460.590634	1.038783751	0.23739	4.3759	1.21E-05	0.0001587	ENSG00000171163	1	248850007	248859085	ZNF692
ENST00000306627	1771.252526	-1.045711157	0.17943	-5.828	5.61E-09	1.74E-07	ENSG00000170142	3	23805954	23891640	UBE2E1
ENST00000306721	527.8174111	-2.127195996	0.21815	-9.751	1.82E-22	4.55E-20	ENSG00000144354	2	173354871	173368997	CDCA7
ENST00000306796	361.2988527	-2.427958981	0.24277	-10	1.51E-23	4.29E-21	ENSG00000169718	17	82057505	82065804	DUS1L
ENST00000306858	695.1105115	-1.435290896	0.20266	-7.082	1.42E-12	9.37E-11	ENSG00000168143	6	54846770	54945099	FAM83B
ENST00000307227	440.4764034	-1.422194156	0.23832	-5.967	2.41E-09	8.05E-08	ENSG00000172340	3	67374718	67654612	SUCLG2
ENST00000307407	52.89703064	2.478815911	0.5192	4.7743	1.80E-06	3.01E-05	ENSG00000169429	4	73740568	73743716	CXCL8
ENST00000307428	21.32004313	-6.433080219	1.58217	-4.066	4.78E-05	0.0005229	ENSG00000169116	4	74933115	75050113	PARM1
ENST00000307439	31.02190251	-2.306840501	0.70998	-3.249	0.0011574	0.007693	ENSG00000169435	4	73571549	73620436	RASSF6
ENST00000307471	301.2834898	-1.443367252	0.25479	-5.665	1.47E-08	4.15E-07	ENSG00000169193	7	23597381	23644708	CCDC126
ENST00000307526	82.18652456	-1.509292292	0.43348	-3.482	0.0004981	0.0038251	ENSG00000170293	3	32238679	32370321	CMTM8
ENST00000307677	2863.756852	1.09283025	0.17672	6.184	6.25E-10	2.33E-08	ENSG00000171552	20	31664457	31722868	BCL2L1
ENST00000307741	531.5346574	-2.162341648	0.21977	-9.839	7.62E-23	2.02E-20	ENSG00000172009	19	2785502	2815807	THOP1
ENST00000307998	30.17330447	2.138806862	0.64714	3.305	0.0009497	0.0065252	ENSG00000172638	11	65866440	65872800	EFEMP2
ENST00000308027	26.65300245	1.831614181	0.6746	2.7151	0.0066254	0.0316529	ENSG00000174327	17	7036014	7040117	SLC16A13
ENST00000308108	410.5376826	-1.741342227	0.24205	-7.194	6.28E-13	4.41E-11	ENSG00000175305	8	94880223	94895201	CCNE2
ENST00000308159	881.0500219	-1.790500717	0.221	-8.102	5.41E-16	5.88E-14	ENSG00000102900	16	56730128	56850286	NUP93
ENST00000308418	302.5282329	-1.533618344	0.29197	-5.253	1.50E-07	3.31E-06	ENSG00000172922	11	65717672	65720798	RNASEH2C
ENST00000308423	36.2758169	1.85719553	0.59303	3.1317	0.0017378	0.0107287	ENSG00000131471	17	42851198	42858124	AOC3
ENST00000308521	245.8336218	1.328713342	0.31125	4.2689	1.96E-05	0.0002414	ENSG00000128394	22	39040863	39055972	APOBEC3F
ENST00000308742	628.1340296	-2.25755968	0.2239	-10.08	6.57E-24	1.94E-21	ENSG00000175548	12	38316773	38329721	ALG10B
ENST00000308748	11.84591229	2.751240817	1.07448	2.5605	0.0104509	0.04542	ENSG00000174939	16	29900487	29906056	ASPHD1
ENST00000308919	49.17521989	1.820534389	0.49638	3.6676	0.0002448	0.0021068	ENSG00000073067	7	983180	989640	CYP2W1
ENST00000309048	67.62041471	-1.434267472	0.42997	-3.336	0.0008507	0.0059644	ENSG00000175279	1	10430432	10442808	CENPS
ENST00000309137	477.8455524	-1.264457191	0.21831	-5.792	6.96E-09	2.11E-07	ENSG00000172775	16	57152465	57186039	PSME3IP1
ENST00000309241	59.74696601	-1.545953651	0.45713	-3.382	0.0007199	0.0052042	ENSG00000155846	5	149730309	149855022	PPARGC1B
ENST00000309285	14.5041682	7.287386779	1.66135	4.3864	1.15E-05	0.000152	ENSG00000173578	3	46017006	46027483	XCR1

lncRNA	Yes	-	108
protein_coding	Yes	NM_203463.3	108
protein_coding	Yes	NM_012145.4	108
protein_coding	Yes	NM_018947.6	108
protein_coding	Yes	NM_003597.5	108
protein_coding	Yes	NM_002201.6	108
protein_coding	Yes	NM_020116.5	108
protein_coding	Yes	NM_015976.5	108
protein_coding	Yes	NM_130468.4	108
protein_coding	Yes	NM_004853.3	108
protein_coding	Yes	NM_052972.3	108
protein_coding	Yes	NM_183240.3	108
protein_coding	Yes	NM_001375405.1	108
protein_coding	Yes	NM_017865.4	108
protein_coding	Yes	NM_003341.5	108
protein_coding	Yes	NM_031942.5	108
protein_coding	Yes	NM_022156.5	108
protein_coding	Yes	NM_001010872.3	108
protein_coding	Yes	NM_003848.4	108
protein_coding	Yes	NM_000584.4	108
protein_coding	Yes	NM_015393.4	108
protein_coding	Yes	NM_177532.5	108
protein_coding	Yes	NM_138771.4	108
protein_coding	Yes	NM_178868.5	108
protein_coding	Yes	NM_138578.3	108
protein_coding	Yes	NM_003249.5	108
protein_coding	Yes	NM_016938.5	108
protein_coding	Yes	NM_201566.3	108
protein_coding	Yes	NM_057749.3	108
protein_coding	Yes	NM_014669.5	108
protein_coding	Yes	NM_032193.4	108
protein_coding	Yes	NM_003734.4	108
protein_coding	Yes	NM_145298.6	108
protein_coding	Yes	NM_001013620.4	108
protein_coding	Yes	NM_181718.4	108
protein_coding	Yes	NM_017781.3	108
protein_coding	Yes	NM_199294.3	108
protein_coding	Yes	NM_024946.4	108
protein_coding	Yes	NM_133263.4	108
protein_coding	Yes	NM_001024644.2	108

ENST00000309602	567.022781	1.602852263	0.28196	5.6846	1.31E-08	3.74E-07	ENSG00000173621	11	66857063	66860475	LRFN4
ENST00000309731	491.0743998	-2.344302861	0.22318	-10.5	8.26E-26	2.93E-23	ENSG00000175318	15	72159805	72197787	GRAMD2A
ENST00000309733	160.2748298	-2.931262092	0.3306	-8.866	7.55E-19	1.15E-16	ENSG00000174749	4	112145453	112195256	FAM241A
ENST00000309758	23.26153297	4.888953004	1.04694	4.6697	3.02E-06	4.73E-05	ENSG00000173110	1	161524539	161526894	HSPA6
ENST00000309868	135.70956	-1.454240319	0.32591	-4.462	8.12E-06	0.0001123	ENSG00000173210	5	149141492	149260542	ABLIM3
ENST00000309880	62.66386616	2.393459843	0.5068	4.7226	2.33E-06	3.77E-05	ENSG00000173825	11	65354750	65357613	TIGD3
ENST00000310046	428.9583161	1.008461708	0.22255	4.5314	5.86E-06	8.44E-05	ENSG00000173914	11	66664997	66677887	RBM4B
ENST00000310054	60.04799086	-1.841041998	0.48942	-3.762	0.0001688	0.0015355	ENSG00000167600	19	41193218	41207539	CYP2S1
ENST00000310078	1270.700186	-1.165701877	0.18817	-6.195	5.83E-10	2.18E-08	ENSG00000173744	2	227472155	227561217	AGFG1
ENST00000310389	3.870821157	5.381562504	2.12805	2.5289	0.011443	0.0486717	ENSG00000175414	5	176365486	176381909	ARL10
ENST00000310392	114.5613469	-1.400087489	0.34113	-4.104	4.06E-05	0.0004538	ENSG00000171806	1	169792528	169794907	METTL18
ENST00000310455	234.3209388	-1.394860198	0.35361	-3.945	7.99E-05	0.000813	ENSG00000173281	8	9136254	9150658	PPP1R3B
ENST00000310521	8.615754769	-4.054230447	1.43753	-2.82	0.0047983	0.02444	ENSG00000174015	13	45702319	45714559	CBY2
ENST00000310571	156.1443298	1.636124626	0.3189	5.1305	2.89E-07	6.00E-06	ENSG00000175575	11	73876998	73931114	PAAF1
ENST00000310614	288.9104049	-2.310094653	0.26049	-8.868	7.42E-19	1.13E-16	ENSG00000175581	11	73787873	73865133	MRPL48
ENST00000310803	28.20343353	-2.351189873	0.67942	-3.461	0.000539	0.0040857	ENSG00000172575	15	38488102	38564814	RASGRP1
ENST00000310806	1265.958111	-1.47555914	0.18494	-7.979	1.48E-15	1.52E-13	ENSG00000129534	14	45203189	45253202	MIS18BP1
ENST00000310833	4.406023918	5.566668149	2.0528	2.7117	0.006693	0.0318868	ENSG00000174059	1	207880971	207911125	CD34
ENST00000310836	23.47077305	-8.027586385	1.58031	-5.08	3.78E-07	7.60E-06	ENSG00000174607	4	114598806	114678225	UGT8
ENST00000311008	665.1286764	1.076848866	0.21548	4.9974	5.81E-07	1.11E-05	ENSG00000169682	16	28974777	28984543	SPNS1
ENST00000311066	233.1733206	1.141335793	0.3066	3.7226	0.0001972	0.0017518	ENSG00000116731	1	13700187	13825079	PRDM2
ENST00000311412	56.49313271	-3.565009458	0.54335	-6.561	5.34E-11	2.49E-09	ENSG00000173083	4	83292460	83334848	HPSE
ENST00000311502	975.4571999	-1.118714189	0.22227	-5.033	4.82E-07	9.41E-06	ENSG00000196642	9	136807947	136841187	RABL6
ENST00000311812	8.766403288	6.559948751	1.7771	3.6914	0.000223	0.0019465	ENSG00000174226	8	100572888	100649665	SNX31
ENST00000311916	228.4264929	1.07937255	0.29744	3.6288	0.0002847	0.0023863	ENSG00000107815	10	100987542	100994403	TWNK
ENST00000311936	935.9329373	-1.549524975	0.22086	-7.016	2.29E-12	1.45E-10	ENSG00000133703	12	25205245	25250929	KRAS
ENST00000311966	8.589892706	-3.431448655	1.31207	-2.615	0.0089151	0.0400318	ENSG00000135423	12	56470951	56488161	GLS2
ENST00000312251	243.331958	1.981838024	0.27307	7.2576	3.94E-13	2.88E-11	ENSG00000103174	16	5024843	5033935	NAGPA
ENST00000312377	2169.833527	-1.157046285	0.1839	-6.292	3.14E-10	1.24E-08	ENSG00000161800	12	49989161	50025494	RACGAP1
ENST00000312499	207.8777876	-1.431606108	0.33996	-4.211	2.54E-05	0.0003018	ENSG00000175643	16	11345458	11351760	RMI2
ENST00000312655	96.72793917	1.422499751	0.44552	3.1929	0.0014084	0.0090422	ENSG00000175155	17	59331654	59401729	YPEL2
ENST00000312814	206.3345368	-1.781344673	0.28953	-6.153	7.62E-10	2.79E-08	ENSG00000146802	7	112762376	112790393	TMEM168
ENST00000312849	22.71651016	1.899824417	0.72138	2.6336	0.0084486	0.0383472	ENSG00000181016	7	112480990	112491062	LSMEM1
ENST00000313005	85.56971773	-1.96760348	0.42691	-4.609	4.05E-06	6.11E-05	ENSG00000186318	11	117285697	117316256	BACE1
ENST00000313056	262.6335407	-1.068076967	0.25722	-4.152	3.29E-05	0.0003779	ENSG00000181396	17	82389209	82418586	OGFOD3
ENST00000313143	1105.593624	1.163975041	0.18961	6.1389	8.31E-10	3.03E-08	ENSG00000161202	3	184155376	184173614	DVL3
ENST00000313182	1238.913894	1.162662325	0.19709	5.8992	3.65E-09	1.18E-07	ENSG00000138614	15	65578756	65611131	INTS14
ENST00000313250	39.36588763	-3.284957709	0.62168	-5.284	1.26E-07	2.84E-06	ENSG00000157326	14	23953769	23969279	DHRS4
ENST00000313401	179.9490125	1.379648189	0.33272	4.1466	3.37E-05	0.0003862	ENSG00000176749	17	32486992	32491253	CDK5R1
ENST00000313546	211.28411	-2.229380438	0.28359	-7.861	3.80E-15	3.72E-13	ENSG00000139323	12	89419717	89526047	POC1B

protein_coding	Yes	NM_024036.5	108
protein_coding	Yes	NM_001012642.3	108
protein_coding	Yes	NM_152400.3	108
protein_coding	Yes	NM_002155.5	108
protein_coding	Yes	NM_014945.5	108
protein_coding	Yes	NM_145719.3	108
protein_coding	Yes	NM_031492.4	108
protein_coding	Yes	NM_030622.8	108
protein_coding	Yes	NM_004504.5	108
protein_coding	Yes	NM_173664.6	108
protein_coding	Yes	NM_033418.4	108
protein_coding	Yes	NM_024607.4	108
protein_coding	Yes	NM_152719.3	108
protein_coding	Yes	NM_025155.3	108
protein_coding	Yes	NM_016055.6	108
protein_coding	Yes	NM_005739.4	108
protein_coding	Yes	NM_018353.5	108
protein_coding	Yes	NM_001025109.2	108
protein_coding	Yes	NM_001128174.3	108
protein_coding	Yes	NM_032038.3	108
protein_coding	Yes	NM_001393986.1	108
protein_coding	Yes	NM_001098540.3	108
protein_coding	Yes	NM_024718.5	108
protein_coding	Yes	NM_152628.4	108
protein_coding	Yes	NM_021830.5	108
protein_coding	Yes	NM_004985.5	108
protein_coding	Yes	NM_013267.4	108
protein_coding	Yes	NM_016256.4	108
protein_coding	Yes	NM_001319999.2	108
protein_coding	Yes	NM_152308.3	108
protein_coding	Yes	NM_001005404.4	108
protein_coding	Yes	NM_022484.6	108
protein_coding	Yes	NM_182597.3	108
protein_coding	Yes	NM_012104.6	108
protein_coding	Yes	NM_024648.3	108
protein_coding	Yes	NM_004423.4	108
protein_coding	Yes	NM_001394796.1	108
protein_coding	Yes	NM_021004.4	108
protein_coding	Yes	NM_003885.3	108
protein_coding	Yes	NM_172240.3	108

ENST00000313578	74.85978099	2.998624072	0.4525	6.6268	3.43E-11	1.68E-09	ENSG00000178301	11	77589952	77610356	AQP11
ENST00000313582	244.7515126	1.074866017	0.27555	3.9007	9.59E-05	0.0009481	ENSG00000104825	19	38899968	38908889	NFKBIB
ENST00000313624	6.341434496	6.092535286	1.88786	3.2272	0.00125	0.0081997	ENSG00000133106	13	42886387	42992241	EPSTI1
ENST00000313766	35.6878196	-3.134000911	0.63832	-4.91	9.12E-07	1.64E-05	ENSG00000177706	7	192570	260772	FAM20C
ENST00000314032	66.98821896	2.623569481	0.45776	5.7314	9.96E-09	2.91E-07	ENSG00000175564	11	74000276	74009085	UCP3
ENST00000314124	244.6079875	1.058740718	0.26145	4.0495	5.13E-05	0.0005566	ENSG00000088726	3	12733527	12759259	TMEM40
ENST00000314256	345.8811004	1.049181771	0.31248	3.3576	0.0007863	0.0056038	ENSG00000177728	17	75456634	75500452	TMEM94
ENST00000314485	183.6823567	1.15968554	0.30256	3.8329	0.0001266	0.0011996	ENSG00000162729	1	160091342	160098619	IGSF8
ENST00000314574	1384.860279	-1.107743941	0.18364	-6.032	1.62E-09	5.61E-08	ENSG00000176105	18	721587	812242	YES1
ENST00000314727	26.05022396	-6.725077932	1.55194	-4.333	1.47E-05	0.0001878	ENSG00000177570	8	118377983	118621963	SAMD12
ENST00000314761	916.5739785	-1.114188325	0.20395	-5.463	4.68E-08	1.17E-06	ENSG00000115368	2	189441473	189475552	WDR75
ENST00000315127	386.7035537	1.031943398	0.24955	4.1353	3.55E-05	0.0004036	ENSG00000179151	15	74630557	74696024	EDC3
ENST00000315367	2056.337112	-1.597305911	0.20442	-7.814	5.54E-15	5.26E-13	ENSG00000175895	8	95133784	95156685	PLEKHF2
ENST00000315392	45.24689311	2.120250547	0.64704	3.2769	0.0010497	0.00709	ENSG00000161381	17	39063312	39151637	PLXDC1
ENST00000315567	523.7644145	-1.431108992	0.25088	-5.704	1.17E-08	3.36E-07	ENSG00000169288	4	77862829	77952785	MRPL1
ENST00000315580	600.725352	-1.409802991	0.22909	-6.154	7.56E-10	2.77E-08	ENSG00000182196	12	122980680	122982909	ARL6IP4
ENST00000315717	2606.1214	-1.355270763	0.17299	-7.835	4.71E-15	4.54E-13	ENSG00000163466	2	218217188	218254348	ARPC2
ENST00000315869	445.3063541	1.244031019	0.22223	5.5979	2.17E-08	5.84E-07	ENSG00000068323	X	49028725	49043357	TFE3
ENST00000315872	2220.930008	-1.09801078	0.18384	-5.973	2.34E-09	7.82E-08	ENSG00000134318	2	11179758	11344636	ROCK2
ENST00000316105	29.67944441	-6.914287251	1.54278	-4.482	7.40E-06	0.0001037	ENSG00000177839	5	141187160	141191541	PCDHB9
ENST00000316185	154.793154	1.044216075	0.3028	3.4485	0.0005637	0.0042449	ENSG00000133460	22	23857874	23886312	SLC2A11
ENST00000316218	119.9310442	-1.027921196	0.33432	-3.075	0.0021071	0.0125471	ENSG00000065485	3	123067024	123162104	PDIA5
ENST00000316407	231.4134328	1.009077385	0.3302	3.0559	0.0022435	0.0132145	ENSG00000176542	3	113648384	113696642	USF3
ENST00000316509	61.29880774	1.583663563	0.53132	2.9806	0.0028767	0.0161448	ENSG00000220205	17	8159148	8162948	VAMP2
ENST00000316586	828.4715057	-1.403441399	0.20135	-6.97	3.17E-12	1.94E-10	ENSG00000115694	2	241492669	241508584	STK25
ENST00000316610	34.84981929	-2.880901043	0.63421	-4.542	5.56E-06	8.06E-05	ENSG00000176055	5	90458208	90474771	MBLAC2
ENST00000316634	270.7007364	1.000596966	0.28693	3.4873	0.0004879	0.0037593	ENSG00000174446	15	66493467	66497762	SNAPC5
ENST00000316660	152.3585598	-1.187079658	0.33811	-3.511	0.0004466	0.0034894	ENSG00000141682	18	59899995	59904305	PMAIP1
ENST00000316724	85.23235724	-1.293220521	0.41543	-3.113	0.0018524	0.0112916	ENSG00000136717	2	127048031	127107154	BIN1
ENST00000316752	89.00095363	1.153120438	0.38087	3.0276	0.0024648	0.0142534	ENSG00000162927	2	60940222	61018255	PUS10
ENST00000316757	158.174565	1.122116845	0.32271	3.4772	0.0005066	0.0038799	ENSG00000011132	19	3750771	3761692	APBA3
ENST00000316963	2885.576342	-1.839030973	0.20278	-9.069	1.20E-19	2.04E-17	ENSG00000120992	8	54046366	54101947	LYPLA1
ENST00000317008	9.768341988	6.715654819	1.74567	3.847	0.0001196	0.0011422	ENSG00000178412	18	79638927	79679745	CTDP1-DT
ENST00000317012	70.3834809	-3.407914403	0.57659	-5.91	3.41E-09	1.10E-07	ENSG00000177494	3	111592899	111595346	ZBED2
ENST00000317058	89.3284873	-1.026210754	0.38381	-2.674	0.0075008	0.0348256	ENSG00000176476	16	28553919	28591790	SGF29
ENST00000317114	35.50476807	1.531274588	0.57402	2.6676	0.0076388	0.0353405	ENSG00000177337	18	3594098	3598352	DLGAP1-AS1
ENST00000317147	3659.595614	-1.21662905	0.18748	-6.489	8.63E-11	3.84E-09	ENSG00000125107	16	58519950	58629826	CNOT1
ENST00000317276	154.3132674	2.275520386	0.35	6.5014	7.96E-11	3.56E-09	ENSG00000179094	17	8140471	8152404	PER1
ENST00000317479	292.617889	-2.653687233	0.28539	-9.298	1.43E-20	2.69E-18	ENSG00000176894	12	132687586	132704985	PXMP2
ENST00000317571	127.7031814	1.123915033	0.32518	3.4563	0.0005476	0.004141	ENSG00000168234	18	24014753	24135600	TTC39C

protein_coding	Yes	NM_173039.3	108
protein_coding	Yes	NM_002503.5	108
protein_coding	Yes	NM_033255.5	108
protein_coding	Yes	NM_020223.4	108
protein_coding	Yes	NM_003356.4	108
protein_coding	Yes	NM_018306.4	108
protein_coding	Yes	NM_014738.6	108
protein_coding	Yes	NM_052868.6	108
protein_coding	Yes	NM_005433.4	108
protein_coding	Yes	NM_207506.3	108
protein_coding	Yes	NM_032168.3	108
protein_coding	Yes	NM_025083.5	108
protein_coding	Yes	NM_024613.4	108
protein_coding	Yes	NM_020405.5	108
protein_coding	Yes	NM_020236.4	108
protein_coding	Yes	NM_018694.4	108
protein_coding	Yes	NM_152862.3	108
protein_coding	Yes	NM_006521.6	108
protein_coding	Yes	NM_004850.5	108
protein_coding	Yes	NM_019119.5	108
protein_coding	Yes	NM_001024939.4	108
protein_coding	Yes	NM_006810.4	108
protein_coding	Yes	NM_001009899.4	108
protein_coding	Yes	NM_014232.3	108
protein_coding	Yes	NM_001271977.2	108
protein_coding	Yes	NM_203406.2	108
protein_coding	Yes	NM_001329615.2	108
protein_coding	Yes	NM_021127.3	108
protein_coding	Yes	NM_139343.3	108
protein_coding	Yes	NM_144709.4	108
protein_coding	Yes	NM_004886.4	108
protein_coding	Yes	NM_006330.4	108
lncRNA	Yes	-	108
protein_coding	Yes	NM_024508.5	108
protein_coding	Yes	NM_138414.3	108
lncRNA	Yes	-	108
protein_coding	Yes	NM_016284.5	108
protein_coding	Yes	NM_002616.3	108
protein_coding	Yes	NM_018663.3	108
protein_coding	Yes	NM_001135993.2	108

ENST00000317615	322.1596787	-1.572359373	0.24267	-6.479	9.22E-11	4.08E-09	ENSG00000128789	18	12703041	12725740	PSMG2
ENST00000317811	174.0531431	-2.200256091	0.34973	-6.291	3.15E-10	1.24E-08	ENSG00000179431	11	35618459	35620865	FJX1
ENST00000317827	49.59048776	3.400293069	0.56835	5.9828	2.19E-09	7.38E-08	ENSG00000147526	8	38787235	38853028	TACC1
ENST00000318003	572.1554859	1.139976894	0.2239	5.0915	3.55E-07	7.20E-06	ENSG00000132024	19	13906200	13930879	CC2D1A
ENST00000318121	689.2657828	-1.094008289	0.23526	-4.65	3.32E-06	5.13E-05	ENSG00000092853	1	35732111	35769978	CLSPN
ENST00000318129	137.949465	-3.103170654	0.35465	-8.75	2.13E-18	3.12E-16	ENSG00000181938	16	58392470	58406147	GINS3
ENST00000318348	615.0173124	-1.073070323	0.20727	-5.177	2.25E-07	4.79E-06	ENSG00000139433	12	109850944	109880541	GLTP
ENST00000318522	2000.563647	-1.011416214	0.19195	-5.269	1.37E-07	3.05E-06	ENSG00000143924	2	42169352	42332548	EML4
ENST00000318562	4063.461675	1.328319454	0.17252	7.6997	1.36E-14	1.23E-12	ENSG00000177106	11	706230	727727	EPS8L2
ENST00000318579	117.0588822	2.625082012	0.39692	6.6136	3.75E-11	1.82E-09	ENSG00000005102	17	43640388	43661922	MEOX1
ENST00000318683	351.4780547	1.189730706	0.24585	4.8393	1.30E-06	2.26E-05	ENSG00000179913	19	17795137	17813576	B3GNT3
ENST00000318948	282.5112099	-10.17242422	1.45918	-6.971	3.14E-12	1.92E-10	ENSG00000180530	21	14961234	15065000	NRIP1
ENST00000319211	77.57142802	-2.28882483	0.4174	-5.484	4.17E-08	1.05E-06	ENSG00000181104	5	76716125	76735770	F2R
ENST00000319286	78.79820829	-1.046522694	0.39378	-2.658	0.0078695	0.0361908	ENSG00000180938	8	124973294	124979389	ZNF572
ENST00000319296	143.9367171	1.527259508	0.31666	4.823	1.41E-06	2.42E-05	ENSG00000179965	16	30407605	30419414	ZNF771
ENST00000319481	274.5488936	-1.08487181	0.25197	-4.306	1.67E-05	0.0002091	ENSG00000141447	18	24162051	24397824	OSBPL1A
ENST00000319560	5.339495796	5.845156443	1.96201	2.9792	0.0028903	0.0162004	ENSG00000179363	X	103710908	103714032	TMEM31
ENST00000319694	46.68370855	-2.074277893	0.53312	-3.891	9.99E-05	0.0009812	ENSG00000176532	7	29563834	29567293	PRR15
ENST00000319933	145.6674587	-2.080316261	0.32133	-6.474	9.53E-11	4.22E-09	ENSG00000181751	5	103258762	103278660	MACIR
ENST00000319974	530.671852	-1.095213178	0.21211	-5.163	2.43E-07	5.12E-06	ENSG00000178074	2	199911292	199928273	C2orf69
ENST00000320640	525.7664379	-1.286173389	0.21315	-6.034	1.60E-09	5.55E-08	ENSG00000129636	16	47154390	47461063	ITFG1
ENST00000320892	28.83483751	-5.26458984	1.056	-4.985	6.18E-07	1.17E-05	ENSG00000151692	2	6917411	7044179	RNF144A
ENST00000320954	1106.088807	-1.00233083	0.21362	-4.692	2.70E-06	4.30E-05	ENSG00000170275	3	33114013	33147773	CRTAP
ENST00000320955	154.802574	1.151272317	0.31833	3.6166	0.0002986	0.0024859	ENSG00000137877	15	41848145	41894053	SPTBN5
ENST00000321276	320.8426729	1.631978403	0.33311	4.8992	9.62E-07	1.72E-05	ENSG00000163082	2	222424542	222562621	SGPP2
ENST00000321348	12.92502985	3.219628036	1.06655	3.0187	0.0025384	0.0146031	ENSG00000071967	2	171522473	171558129	CYBRD1
ENST00000321358	3490.449193	-1.013141163	0.20459	-4.952	7.34E-07	1.36E-05	ENSG00000065978	1	42682417	42703805	YBX1
ENST00000321556	1271.330526	1.313748573	0.1865	7.044	1.87E-12	1.21E-10	ENSG00000158828	1	20633457	20651511	PINK1
ENST00000321949	100.0444009	1.060006381	0.36575	2.8982	0.0037531	0.0200334	ENSG00000105662	19	18683679	18782333	CRTC1
ENST00000322048	338.9174129	1.750445058	0.24341	7.1913	6.42E-13	4.50E-11	ENSG00000067836	16	4796967	4802633	ROGDI
ENST00000322165	26.51108681	1.897515712	0.7027	2.7003	0.0069276	0.0327674	ENSG00000025423	12	56763323	56787790	HSD17B6
ENST00000322349	1302.366769	-1.471122366	0.22968	-6.405	1.50E-10	6.33E-09	ENSG00000102189	12	92770636	92929295	EEA1
ENST00000322563	128.0806077	1.123394027	0.33211	3.3826	0.0007181	0.0051931	ENSG00000085741	11	76186324	76206502	WNT11
ENST00000322564	15.53796615	2.352531857	0.897	2.6227	0.0087242	0.039347	ENSG00000175985	14	69484727	69531551	PLEKHD1
ENST00000322623	819.2831168	-1.335645944	0.22827	-5.851	4.88E-09	1.53E-07	ENSG00000175792	3	128080809	128123812	RUVBL1
ENST00000322652	1194.392848	-1.045573876	0.20705	-5.05	4.42E-07	8.72E-06	ENSG00000178691	17	31937006	32001038	SUZ12
ENST00000322723	3569.814959	-1.494162994	0.209	-7.149	8.74E-13	5.99E-11	ENSG00000115053	2	231453530	231464484	NCL
ENST00000322886	241.6922393	-1.082244607	0.2635	-4.107	4.01E-05	0.0004491	ENSG00000165891	12	77021250	77065569	E2F7
ENST00000322927	946.7337082	2.092909689	0.20716	10.103	5.37E-24	1.61E-21	ENSG00000198026	20	45948659	45972203	ZNF335
ENST00000322954	894.3667234	-1.605147288	0.20884	-7.686	1.52E-14	1.36E-12	ENSG00000137831	15	70654553	70763558	UACA

protein_coding	Yes	NM_020232.5	108
protein_coding	Yes	NM_014344.4	108
protein_coding	Yes	NM_006283.3	108
protein_coding	Yes	NM_017721.5	108
protein_coding	Yes	NM_022111.4	108
protein_coding	Yes	NM_022770.4	108
protein_coding	Yes	NM_016433.4	108
protein_coding	Yes	NM_019063.5	108
protein_coding	Yes	NM_022772.4	108
protein_coding	Yes	NM_004527.4	108
protein_coding	Yes	NM_014256.4	108
protein_coding	Yes	NM_003489.4	108
protein_coding	Yes	NM_001992.5	108
protein_coding	Yes	NM_152412.3	108
protein_coding	Yes	NM_001142305.2	108
protein_coding	Yes	NM_080597.4	108
protein_coding	Yes	NM_182541.2	108
protein_coding	Yes	NM_175887.3	108
protein_coding	Yes	NM_033211.4	108
protein_coding	Yes	NM_153689.6	108
protein_coding	Yes	NM_030790.5	108
protein_coding	Yes	NM_014746.6	108
protein_coding	Yes	NM_006371.5	108
protein_coding	Yes	NM_016642.4	108
protein_coding	Yes	NM_152386.4	108
protein_coding	Yes	NM_024843.4	108
protein_coding	Yes	NM_004559.5	108
protein_coding	Yes	NM_032409.3	108
protein_coding	Yes	NM_015321.3	108
protein_coding	Yes	NM_024589.3	108
protein_coding	Yes	NM_003725.4	108
protein_coding	Yes	NM_003566.4	108
protein_coding	Yes	NM_004626.3	108
protein_coding	Yes	NM_001161498.2	108
protein_coding	Yes	NM_003707.3	108
protein_coding	Yes	NM_015355.4	108
protein_coding	Yes	NM_005381.3	108
protein_coding	Yes	NM_203394.3	108
protein_coding	Yes	NM_022095.4	108
protein_coding	Yes	NM_018003.4	108

ENST00000323019	521.9004175	1.027146544	0.21291	4.8243	1.40E-06	2.41E-05	ENSG00000177427	17	18260661	18266552	MIEF2
ENST00000323022	4.428846192	5.573362803	2.05277	2.715	0.0066267	0.0316529	ENSG00000102001	X	49205062	49233340	CACNA1F
ENST00000323206	325.6481548	1.314547648	0.24569	5.3503	8.78E-08	2.05E-06	ENSG00000181284	17	7435434	7437679	TMEM102
ENST00000323274	158.7629594	-1.127613442	0.33601	-3.356	0.0007912	0.005633	ENSG00000176890	18	657652	673578	TYMS
ENST00000323372	11.39786125	3.37287051	1.15272	2.926	0.0034333	0.0186594	ENSG00000179761	17	29043140	29057216	PIPOX
ENST00000323380	20.25031713	2.258628917	0.8095	2.7901	0.0052684	0.026376	ENSG00000179141	13	29476514	29490105	MTUS2-AS1
ENST00000323468	783.3222733	-2.357196083	0.20342	-11.59	4.74E-31	3.02E-28	ENSG00000178202	11	108472115	108498384	POGLUT3
ENST00000323523	76.21407778	1.652522623	0.41567	3.9756	7.02E-05	0.0007291	ENSG00000181458	3	100492618	100577444	TMEM45A
ENST00000323662	89.19215392	1.776150449	0.47202	3.7629	0.0001679	0.0015292	ENSG00000179698	8	144107738	144118328	WDR97
ENST00000323666	1530.306563	-1.632623222	0.18139	-9.001	2.25E-19	3.69E-17	ENSG00000111142	12	95474151	95515839	METAP2
ENST00000323760	422.550003	-1.745385286	0.22858	-7.636	2.25E-14	1.97E-12	ENSG00000158402	5	138285268	138331804	CDC25C
ENST00000323776	4.946371907	5.738428866	2.09357	2.741	0.0061256	0.0297031	ENSG00000136449	17	50508438	50531501	MYCBPAP
ENST00000323777	5.897520832	5.986731667	1.91891	3.1199	0.0018094	0.0110864	ENSG00000176160	17	58420166	58488408	HSF5
ENST00000323851	3525.91699	1.769990085	0.17534	10.095	5.83E-24	1.73E-21	ENSG00000104419	8	133237174	133297252	NDRG1
ENST00000323963	3373.768989	-1.228103199	0.16998	-7.225	5.02E-13	3.59E-11	ENSG00000156976	3	186783576	186789897	EIF4A2
ENST00000324001	77.46783322	-1.869942554	0.41626	-4.492	7.05E-06	9.93E-05	ENSG00000105227	19	40393767	40413378	PRX
ENST00000324093	310.6630387	-1.292176488	0.25404	-5.086	3.65E-07	7.36E-06	ENSG00000004399	3	129555213	129606676	PLXND1
ENST00000324194	749.1210321	-1.37377383	0.21898	-6.274	3.53E-10	1.37E-08	ENSG00000114120	3	140941835	140980995	SLC25A36
ENST00000324210	2104.061789	-1.070013529	0.18059	-5.925	3.12E-09	1.02E-07	ENSG00000152601	3	152268928	152465780	MBNL1
ENST00000324219	864.9387178	-2.514063886	0.20826	-12.07	1.49E-33	1.25E-30	ENSG00000071054	2	101697706	101894690	MAP4K4
ENST00000324453	565.6517437	1.566194101	0.28941	5.4116	6.25E-08	1.51E-06	ENSG00000180354	7	30134985	30162762	MTURN
ENST00000324787	180.8979831	-1.150782873	0.29694	-3.876	0.0001064	0.0010323	ENSG00000090263	7	141002609	141014952	MRPS33
ENST00000324894	139.310118	-1.308720826	0.32268	-4.056	5.00E-05	0.0005435	ENSG00000130299	19	17337552	17342731	GTPBP3
ENST00000325000	1529.042813	-1.227418964	0.1855	-6.617	3.67E-11	1.79E-09	ENSG00000178464	19	12643274	12643919	RPL10P16
ENST00000325110	1047.599842	-1.192887438	0.18992	-6.281	3.36E-10	1.32E-08	ENSG00000112972	5	43287469	43313412	HMGCS1
ENST00000325167	74.57530756	-1.738282912	0.4317	-4.027	5.66E-05	0.0006047	ENSG00000176108	17	80991840	81000133	CHMP6
ENST00000325307	850.2994058	-2.50027072	0.20523	-12.18	3.85E-34	3.80E-31	ENSG00000029993	X	150983334	150990771	HMGB3
ENST00000325327	2100.896654	-1.004681096	0.20249	-4.962	6.99E-07	1.30E-05	ENSG00000176619	19	2428165	2456959	LMNB2
ENST00000325495	1444.168502	-1.004898689	0.19986	-5.028	4.96E-07	9.63E-06	ENSG00000099783	19	8444974	8489114	HNRNPM
ENST00000325617	38.94578611	-1.808938604	0.59403	-3.045	0.0023253	0.0136014	ENSG00000153132	4	140388452	140427648	CLGN
ENST00000325805	1301.286807	-1.52953521	0.21177	-7.223	5.10E-13	3.64E-11	ENSG00000114439	3	107522961	107811339	BBX
ENST00000325888	10.98082638	6.884571106	1.7154	4.0134	5.99E-05	0.0006354	ENSG00000128591	7	128830405	128859272	FLNC
ENST00000326047	321.5078705	-1.236645047	0.24744	-4.998	5.80E-07	1.10E-05	ENSG00000163808	3	44761793	44853256	KIF15
ENST00000326134	879.3849563	1.1392734	0.21098	5.3999	6.67E-08	1.60E-06	ENSG00000179832	8	144148015	144261926	MROH1
ENST00000326194	50.98905747	1.232974741	0.48726	2.5304	0.0113931	0.0485038	ENSG00000102312	X	48508991	48520808	PORCN
ENST00000326266	154.5214819	1.016485615	0.30594	3.3225	0.0008923	0.0062012	ENSG00000131652	16	3024034	3027750	THOC6
ENST00000326446	491.8429078	-1.14963917	0.22085	-5.206	1.93E-07	4.17E-06	ENSG00000177917	2	152718619	152762396	ARL6IP6
ENST00000326505	292.0500238	-1.941261614	0.25239	-7.691	1.45E-14	1.31E-12	ENSG00000176597	3	183253252	183273385	B3GNT5
ENST00000326587	854.6212125	1.245351126	0.23434	5.3143	1.07E-07	2.44E-06	ENSG00000179222	X	51893622	51902354	MAGED1
ENST00000326674	518.5880247	-1.21861087	0.22666	-5.376	7.60E-08	1.81E-06	ENSG00000110536	11	47565598	47573461	PTPMT1

protein_coding	Yes	NM_139162.4	108
protein_coding	Yes	NM_001256789.3	108
protein_coding	Yes	NM_178518.3	108
protein_coding	Yes	NM_001071.4	108
protein_coding	Yes	NM_016518.3	108
lncRNA	Yes	-	108
protein_coding	Yes	NM_153705.5	108
protein_coding	Yes	NM_018004.3	108
protein_coding	Yes	NM_001316309.2	108
protein_coding	Yes	NM_006838.4	108
protein_coding	Yes	NM_001790.5	108
protein_coding	Yes	NM_032133.6	108
protein_coding	Yes	NM_001080439.3	108
protein_coding	Yes	NM_006096.4	108
protein_coding	Yes	NM_001967.4	108
protein_coding	Yes	NM_181882.3	108
protein_coding	Yes	NM_015103.3	108
protein_coding	Yes	NM_001104647.3	108
protein_coding	Yes	NM_021038.5	108
protein_coding	Yes	NM_001395002.1	108
protein_coding	Yes	NM_152793.3	108
protein_coding	Yes	NM_053035.3	108
protein_coding	Yes	NM_032620.4	108
ocessed_pseudoge	Yes	-	108
protein_coding	Yes	NM_001098272.3	108
protein_coding	Yes	NM_024591.5	108
protein_coding	Yes	NM_005342.4	108
protein_coding	Yes	NM_032737.4	108
protein_coding	Yes	NM_005968.5	108
protein_coding	Yes	NM_004362.3	108
protein_coding	Yes	NM_001142568.3	108
protein_coding	Yes	NM_001458.5	108
protein_coding	Yes	NM_020242.3	108
protein_coding	Yes	NM_032450.3	108
protein_coding	Yes	NM_203475.3	108
protein_coding	Yes	NM_024339.5	108
protein_coding	Yes	NM_152522.7	108
protein_coding	Yes	NM_032047.5	108
protein_coding	Yes	NM_006986.4	108
protein_coding	Yes	NM_175732.3	108

ENST00000326735	312.5581299	-1.195262297	0.24845	-4.811	1.50E-06	2.56E-05	ENSG00000159363	1	16985957	17011928	ATP13A2
ENST00000326840	2049.621472	-1.254906738	0.20744	-6.049	1.45E-09	5.09E-08	ENSG00000057019	3	98795940	98901695	DCBLD2
ENST00000327040	254.3588543	-1.199179499	0.29385	-4.081	4.49E-05	0.000495	ENSG00000156011	8	18527302	19013703	PSD3
ENST00000327158	171.0433202	-1.030586574	0.30084	-3.426	0.0006132	0.0045466	ENSG00000177370	17	997128	1003671	TIMM22
ENST00000327259	7.855753684	6.400517567	1.81236	3.5316	0.0004131	0.0032672	ENSG00000179292	11	66291893	66296664	TMEM151A
ENST00000327347	880.0832604	-1.379488748	0.19685	-7.008	2.42E-12	1.53E-10	ENSG00000086475	10	13317427	13348293	SEPHS1
ENST00000327428	97.13719174	-3.127276985	0.4289	-7.291	3.07E-13	2.29E-11	ENSG00000163170	2	74135399	74147912	BOLA3
ENST00000327435	780.3065394	-1.833688031	0.23251	-7.887	3.10E-15	3.08E-13	ENSG00000182551	2	3497365	3519531	ADI1
ENST00000327490	142.3636082	-2.653462588	0.33584	-7.901	2.77E-15	2.76E-13	ENSG00000185262	17	76265347	76271298	UBALD2
ENST00000327835	807.0532477	-1.666645747	0.21637	-7.703	1.33E-14	1.21E-12	ENSG00000164684	8	80628450	80874781	ZNF704
ENST00000327906	36.28096213	1.858888419	0.57292	3.2446	0.0011762	0.0077968	ENSG00000010318	3	52410659	52423641	PHF7
ENST00000328111	426.3329709	-2.895785764	0.23809	-12.16	4.91E-34	4.72E-31	ENSG00000088305	20	32762384	32809356	DNMT3B
ENST00000328300	20.54640956	-3.976292752	0.94055	-4.228	2.36E-05	0.0002831	ENSG00000188153	X	108439837	108697545	COL4A5
ENST00000328434	286.8595075	-1.01123909	0.2674	-3.782	0.0001557	0.0014337	ENSG00000183978	17	42797624	42798704	COA3
ENST00000328459	85.92128617	-1.433227238	0.38379	-3.734	0.0001882	0.0016828	ENSG00000165914	14	90524563	90816430	TTC7B
ENST00000328666	513.8221711	1.147552719	0.25597	4.4831	7.36E-06	0.0001031	ENSG00000187531	17	81911938	81918176	SIRT7
ENST00000328697	141.420054	-3.754257093	0.37574	-9.992	1.66E-23	4.69E-21	ENSG00000109881	11	27338511	27363215	CCDC34
ENST00000328703	854.157458	-1.097466076	0.19511	-5.625	1.86E-08	5.09E-07	ENSG00000143575	1	154272628	154275875	HAX1
ENST00000328771	515.4842418	1.115680716	0.21523	5.1837	2.17E-07	4.64E-06	ENSG00000134815	19	47349314	47382704	DHX34
ENST00000328827	2125.189383	-1.000545114	0.17671	-5.662	1.50E-08	4.21E-07	ENSG00000177425	12	79584878	79690964	PAWR
ENST00000328880	19.19484252	-4.193812826	1.02764	-4.081	4.48E-05	0.0004947	ENSG00000182782	12	122701292	122703357	HCAR2
ENST00000328965	49.81762812	-1.435411773	0.48352	-2.969	0.0029909	0.016649	ENSG00000184232	11	120211031	120230334	OAF
ENST00000329003	27.06778059	8.18625291	1.56366	5.2353	1.65E-07	3.61E-06	ENSG00000182687	17	76074780	76077537	GALR2
ENST00000329078	448.2954765	1.714506017	0.22512	7.616	2.62E-14	2.27E-12	ENSG00000183018	17	4498880	4539035	SPNS2
ENST00000329146	64.97645052	-1.159732671	0.43839	-2.645	0.008158	0.0373033	ENSG00000182809	14	105474820	105480162	CRIP2
ENST00000329321	90.31158413	-1.128053365	0.37308	-3.024	0.0024977	0.0144034	ENSG00000183840	2	132416804	132646582	GPR39
ENST00000329492	628.4544335	3.10611964	0.23959	12.964	1.95E-38	3.31E-35	ENSG00000008735	22	50600792	50613978	MAPK8IP2
ENST00000329565	233.8370465	-1.313914229	0.3017	-4.355	1.33E-05	0.0001726	ENSG00000184602	16	11668454	11679152	SNN
ENST00000329600	84.84524245	1.367741788	0.38822	3.5231	0.0004265	0.0033556	ENSG00000183208	15	90234208	90245811	GDPGP1
ENST00000329608	43.40567812	2.497086006	0.56792	4.3969	1.10E-05	0.0001458	ENSG00000184371	1	109910848	109930992	CSF1
ENST00000330062	1043.217013	-1.063438997	0.21094	-5.041	4.62E-07	9.07E-06	ENSG00000182054	15	90083044	90102468	IDH2
ENST00000330133	114.2442574	-2.665543661	0.39333	-6.777	1.23E-11	6.63E-10	ENSG00000183617	19	3762681	3767565	MRPL54
ENST00000330137	1270.362363	-1.456497993	0.18776	-7.757	8.69E-15	8.04E-13	ENSG00000182628	17	59109856	59155186	SKA2
ENST00000330263	685.960015	-1.660051712	0.27367	-6.066	1.31E-09	4.63E-08	ENSG00000053372	1	19251804	19260128	MRTO4
ENST00000330333	535.2404307	-1.142448442	0.21804	-5.24	1.61E-07	3.54E-06	ENSG00000182240	21	41168159	41282530	BACE2
ENST00000330368	71.79877125	1.594960455	0.4314	3.6972	0.000218	0.0019084	ENSG00000184378	3	169766920	169769561	ACTRT3
ENST00000330475	25.59403115	1.844367301	0.68953	2.6748	0.0074767	0.034737	ENSG00000185761	19	1505021	1513019	ADAMTSL5
ENST00000330560	530.7094947	-1.584419464	0.22413	-7.069	1.56E-12	1.02E-10	ENSG00000184661	8	25459232	25507911	CDCA2
ENST00000330651	118.2650998	1.510644462	0.34934	4.3243	1.53E-05	0.0001946	ENSG00000185386	22	50263712	50270380	MAPK11
ENST00000330676	93.11146642	1.113676229	0.38379	2.9018	0.0037105	0.0198488	ENSG00000185561	17	1702815	1710377	TLCD2

protein_coding	Yes	NM_022089.4	108
protein_coding	Yes	NM_080927.4	108
protein_coding	Yes	NM_015310.4	108
protein_coding	Yes	NM_013337.4	108
protein_coding	Yes	NM_153266.4	108
protein_coding	Yes	NM_012247.5	108
protein_coding	Yes	NM_212552.3	108
protein_coding	Yes	NM_018269.4	108
protein_coding	Yes	NM_182565.4	108
protein_coding	Yes	NM_001033723.3	108
protein_coding	Yes	NM_016483.7	108
protein_coding	Yes	NM_006892.4	108
protein_coding	Yes	NM_033380.3	108
protein_coding	Yes	NM_001040431.3	108
protein_coding	Yes	NM_001010854.2	108
protein_coding	Yes	NM_016538.3	108
protein_coding	Yes	NM_030771.2	108
protein_coding	Yes	NM_006118.4	108
protein_coding	Yes	NM_014681.6	108
protein_coding	Yes	NM_002583.4	108
protein_coding	Yes	NM_177551.4	108
protein_coding	Yes	NM_178507.4	108
protein_coding	Yes	NM_003857.4	108
protein_coding	Yes	NM_001124758.3	108
protein_coding	Yes	NM_001312.4	108
protein_coding	Yes	NM_001508.3	108
protein_coding	Yes	NM_012324.6	108
protein_coding	Yes	NM_003498.6	108
protein_coding	Yes	NM_001013657.3	108
protein_coding	Yes	NM_000757.6	108
protein_coding	Yes	NM_002168.4	108
protein_coding	Yes	NM_172251.3	108
protein_coding	Yes	NM_182620.4	108
protein_coding	Yes	NM_016183.4	108
protein_coding	Yes	NM_012105.5	108
protein_coding	Yes	NM_032487.5	108
protein_coding	Yes	NM_213604.3	108
protein_coding	Yes	NM_152562.4	108
protein_coding	Yes	NM_002751.7	108
protein_coding	Yes	NM_001164407.2	108

ENST00000330714	17.43715564	-2.159007465	0.83512	-2.585	0.00973	0.0428834	ENSG00000183486	21	41362026	41409393	MX2
ENST00000330794	93.20179313	-2.694824333	0.40131	-6.715	1.88E-11	9.75E-10	ENSG00000184584	5	139475532	139482758	STING1
ENST00000330877	7.404906945	3.769962125	1.48719	2.535	0.011246	0.0480061	ENSG00000185100	14	104724228	104747310	ADSS1
ENST00000330889	62.37310091	-2.906519157	0.49236	-5.903	3.57E-09	1.15E-07	ENSG00000184060	17	30921944	30959322	ADAP2
ENST00000331128	683.9448173	-1.277555355	0.20217	-6.319	2.63E-10	1.05E-08	ENSG00000184270	1	149887468	149887965	H2AC21
ENST00000331238	44.33697265	-2.181173505	0.56604	-3.853	0.0001165	0.0011169	ENSG00000185924	17	1934676	2025334	RTN4RL1
ENST00000331302	12.03069321	-3.482654407	1.14373	-3.045	0.0023269	0.013607	ENSG00000181031	17	212388	352807	RPH3AL
ENST00000331334	298.5988736	-1.017508843	0.2714	-3.749	0.0001775	0.0016015	ENSG00000182512	14	95535049	95544714	GLRX5
ENST00000331366	105.6453948	-3.691574424	0.44219	-8.348	6.92E-17	8.37E-15	ENSG00000139117	12	38652202	38905591	CPNE8
ENST00000331380	1826.138253	-1.319933521	0.17831	-7.403	1.33E-13	1.05E-11	ENSG00000184260	1	149886917	149887411	H2AC20
ENST00000331442	5800.09176	-1.542686143	0.17541	-8.795	1.43E-18	2.13E-16	ENSG00000184357	6	27866791	27867588	H1-5
ENST00000331628	18.01526847	4.521498144	1.11692	4.0482	5.16E-05	0.0005592	ENSG00000183379	14	74405898	74426210	SYNDIG1L
ENST00000331782	140.2779392	-3.025994561	0.36631	-8.261	1.45E-16	1.68E-14	ENSG00000184916	14	105140994	105168776	JAG2
ENST00000331835	3529.413085	-1.040426208	0.19203	-5.418	6.02E-08	1.47E-06	ENSG00000183291	1	86862444	86914126	SELENOF
ENST00000331890	339.0720011	1.129559052	0.26531	4.2575	2.07E-05	0.000252	ENSG00000182325	8	144355430	144358472	FBXL6
ENST00000332118	988.6475308	1.03111546	0.19529	5.2798	1.29E-07	2.89E-06	ENSG00000182934	11	126262937	126268895	SRPRA
ENST00000332145	4.149833674	5.480651158	2.08437	2.6294	0.0085535	0.0387013	ENSG00000182950	15	75724040	75727688	ODF3L1
ENST00000332160	2045.07651	-1.698224515	0.25077	-6.772	1.27E-11	6.82E-10	ENSG00000185432	12	50925014	50932508	METTL7A
ENST00000332235	8.048120645	-6.483967359	1.80712	-3.588	0.0003332	0.0027255	ENSG00000183186	19	405444	409147	C2CD4C
ENST00000332439	92.3836615	1.916584108	0.42938	4.4636	8.06E-06	0.0001117	ENSG00000182224	17	7858002	7862282	CYB5D1
ENST00000332582	6.505916345	-4.689701638	1.85471	-2.529	0.0114541	0.0487039	ENSG00000184194	X	53048788	53080615	GPR173
ENST00000332598	811.6784167	1.036130213	0.20839	4.9721	6.62E-07	1.24E-05	ENSG00000184840	5	177592202	177597242	TMED9
ENST00000332780	8.41377872	6.498399027	1.79903	3.6122	0.0003037	0.0025203	ENSG00000185909	3	49171597	49176486	KLHDC8B
ENST00000332884	56.36991867	-2.631825118	0.51408	-5.119	3.06E-07	6.32E-06	ENSG00000155016	4	107931548	107953461	CYP2U1
ENST00000333017	14.33926705	7.270141237	1.65842	4.3838	1.17E-05	0.0001536	ENSG00000185674	2	99242245	99255666	LYG2
ENST00000333090	301.3743106	-1.504486317	0.28954	-5.196	2.03E-07	4.37E-06	ENSG00000182704	11	76783355	76798144	TSKU
ENST00000333130	288.4128671	-2.058400175	0.30713	-6.702	2.06E-11	1.06E-09	ENSG00000185608	22	19432544	19436075	MRPL40
ENST00000333151	2979.188217	-1.280347248	0.17258	-7.419	1.18E-13	9.34E-12	ENSG00000276368	6	27814301	27814777	H2AC14
ENST00000333202	581.2393792	1.271565071	0.2133	5.9614	2.50E-09	8.32E-08	ENSG00000184277	15	101641847	101652381	TM2D3
ENST00000333279	8.578679867	6.527755504	1.78379	3.6595	0.0002527	0.0021653	ENSG00000183831	1	173608335	173669851	ANKRD45
ENST00000333421	347.8288252	-1.091994008	0.24495	-4.458	8.27E-06	0.0001141	ENSG00000107362	9	71865151	71911193	ABHD17B
ENST00000333467	283.2005182	1.386941556	0.25065	5.5333	3.14E-08	8.14E-07	ENSG00000179750	22	38982346	38992779	APOBEC3B
ENST00000333482	4.451668466	5.580042745	2.05553	2.7147	0.0066346	0.0316755	ENSG00000249021	5	115691461	115692167	-
ENST00000333751	274.8124578	1.273817316	0.26488	4.8091	1.52E-06	2.58E-05	ENSG00000213859	17	7352161	7354944	KCTD11
ENST00000333762	394.4312716	-1.404748756	0.24557	-5.72	1.06E-08	3.09E-07	ENSG00000184897	3	129314770	129316286	H1-10
ENST00000333845	100.1193126	1.272447947	0.35877	3.5467	0.0003901	0.0031124	ENSG00000241404	6	32164594	32168281	EGFL8
ENST00000333861	27.09067329	1.950426713	0.65828	2.9629	0.0030475	0.0169105	ENSG00000182791	11	66590175	66593063	CCDC87
ENST00000333891	18.72737277	-3.872757775	1.00966	-3.836	0.0001252	0.0011875	ENSG00000186472	7	82754011	83162884	PCLO
ENST00000334062	111.311167	-1.573589882	0.35782	-4.398	1.09E-05	0.0001453	ENSG00000185989	13	113977782	114132623	RASA3
ENST00000334126	1590.5634	1.917648341	0.21226	9.0342	1.65E-19	2.77E-17	ENSG00000168014	11	74012717	74171002	C2CD3

protein_coding	Yes	NM_002463.2	108
protein_coding	Yes	NM_198282.4	108
protein_coding	Yes	NM_152328.5	108
protein_coding	Yes	NM_018404.3	108
protein_coding	Yes	NM_175065.3	108
protein_coding	Yes	NM_178568.4	108
protein_coding	Yes	NM_006987.4	108
protein_coding	Yes	NM_016417.3	108
protein_coding	Yes	NM_153634.3	108
protein_coding	Yes	NM_003517.3	108
protein_coding	Yes	NM_005322.3	108
protein_coding	Yes	NM_001105579.2	108
protein_coding	Yes	NM_002226.5	108
protein_coding	Yes	NM_004261.5	108
protein_coding	Yes	NM_012162.4	108
protein_coding	Yes	NM_003139.4	108
protein_coding	Yes	NM_175881.5	108
protein_coding	Yes	NM_014033.4	108
protein_coding	Yes	NM_001136263.2	108
protein_coding	Yes	NM_144607.6	108
protein_coding	Yes	NM_018969.6	108
protein_coding	Yes	NM_017510.6	108
protein_coding	Yes	NM_173546.3	108
protein_coding	Yes	NM_183075.3	108
protein_coding	Yes	NM_175735.4	108
protein_coding	Yes	NM_015516.4	108
protein_coding	Yes	NM_003776.4	108
protein_coding	Yes	NM_021066.3	108
protein_coding	Yes	NM_078474.3	108
protein_coding	Yes	NM_198493.3	108
protein_coding	Yes	NM_001025780.3	108
protein_coding	Yes	NM_004900.5	108
lncRNA	Yes	-	108
protein_coding	Yes	NM_001363642.1	108
protein_coding	Yes	NM_006026.4	108
protein_coding	Yes	NM_030652.4	108
protein_coding	Yes	NM_018219.3	108
protein_coding	Yes	NM_033026.6	108
protein_coding	Yes	NM_007368.4	108
protein_coding	Yes	NM_001286577.2	108

ENST00000334166	19.16812696	-6.278827026	1.58988	-3.949	7.84E-05	0.0007999	ENSG00000187123	2	149330586	149474138	LYPD6
ENST00000334179	4.639391888	5.641491239	2.02631	2.7841	0.0053674	0.0267625	ENSG00000186150	1	110112442	110113947	UBL4B
ENST00000334186	248.9338945	1.272942399	0.26318	4.8368	1.32E-06	2.28E-05	ENSG00000177380	19	49119543	49151026	PPFIA3
ENST00000334232	198.1448695	-1.021578542	0.28458	-3.59	0.0003309	0.0027103	ENSG00000186197	1	236394285	236484930	EDARADD
ENST00000334274	91.04128014	-1.07192855	0.37102	-2.889	0.0038634	0.0205222	ENSG00000176148	11	33039571	33073550	TCP11L1
ENST00000334306	113.2359359	-1.67311733	0.36499	-4.584	4.56E-06	6.79E-05	ENSG00000186212	4	76894151	76898144	SOWAHB
ENST00000334307	43.23540912	-3.140438225	0.59435	-5.284	1.27E-07	2.84E-06	ENSG00000166352	11	36594501	36659272	IFTAP
ENST00000334351	1748.59409	-1.022973061	0.17768	-5.757	8.54E-09	2.54E-07	ENSG00000189266	1	23959827	23963462	PNRC2
ENST00000334414	41.27820466	1.767737695	0.5419	3.2621	0.0011059	0.0074105	ENSG00000127329	12	70515869	70637429	PTPRB
ENST00000334456	34.39541587	2.073997038	0.60943	3.4032	0.000666	0.0048759	ENSG00000186642	11	72576140	72674422	PDE2A
ENST00000334462	46.73358925	1.390027571	0.50117	2.7735	0.005545	0.0274346	ENSG00000168890	2	85598546	85602698	TMEM150A
ENST00000334529	7.320550923	6.299607737	1.83481	3.4334	0.0005961	0.0044428	ENSG00000186265	3	112463965	112499472	BTLA
ENST00000334583	11.6669009	3.056442659	1.10364	2.7694	0.0056156	0.0277085	ENSG00000159753	16	67645143	67657569	CARMIL2
ENST00000334635	304.4389604	-1.449672784	0.25466	-5.692	1.25E-08	3.59E-07	ENSG00000109184	4	51843152	51916837	DCUN1D4
ENST00000334661	95.38022812	1.553932605	0.44379	3.5015	0.0004626	0.0035975	ENSG00000187091	3	38007495	38029642	PLCD1
ENST00000334696	146.4272091	-1.223640082	0.31593	-3.873	0.0001075	0.0010414	ENSG00000187097	14	73963229	74019288	ENTPD5
ENST00000334816	56.87275098	1.743874101	0.47609	3.6629	0.0002494	0.0021411	ENSG00000186132	2	119302224	119366834	C2orf76
ENST00000335007	2605.978205	-1.462790407	0.17774	-8.23	1.88E-16	2.16E-14	ENSG00000186298	12	110719679	110742891	PPP1CC
ENST00000335125	31.07356639	-3.020333452	0.67791	-4.455	8.37E-06	0.0001154	ENSG00000187630	14	23988928	24006403	DHRS4L2
ENST00000335154	80.10223189	-2.307676836	0.41347	-5.581	2.39E-08	6.37E-07	ENSG00000161791	12	49636498	49707405	FMNL3
ENST00000335174	31.18894608	2.454322003	0.6413	3.8271	0.0001297	0.0012244	ENSG00000186352	4	185396840	185400241	ANKRD37
ENST00000335183	602.4090323	-1.328564257	0.21435	-6.198	5.72E-10	2.14E-08	ENSG00000100526	14	54397006	54420218	CDKN3
ENST00000335185	17.53101873	5.077330748	1.27607	3.9789	6.92E-05	0.0007208	ENSG00000186714	11	32602720	32794662	CCDC73
ENST00000335350	52.6449658	1.20486167	0.46991	2.564	0.0103473	0.0450513	ENSG00000107731	10	71212569	71302864	UNC5B
ENST00000335464	123.8467818	1.095832058	0.34629	3.1645	0.0015537	0.0097913	ENSG00000109066	17	74776498	74839753	TMEM104
ENST00000335473	7.434662294	6.319710193	1.84158	3.4317	0.0005998	0.0044641	ENSG00000133454	22	25742187	26031045	MYO18B
ENST00000335509	453.0042433	1.385865553	0.28237	4.9079	9.20E-07	1.66E-05	ENSG00000186815	12	113221463	113298585	TPCN1
ENST00000335613	38.73126597	1.800139851	0.57219	3.1461	0.0016548	0.010316	ENSG00000167992	11	61258285	61295316	VWCE
ENST00000335659	274.5197795	-1.833085664	0.26127	-7.016	2.28E-12	1.45E-10	ENSG00000186106	8	100520771	100559759	ANKRD46
ENST00000335670	2544.325586	2.137450358	0.203	10.53	6.31E-26	2.32E-23	ENSG00000069667	15	60488283	61229302	RORA
ENST00000335678	8.48739077	6.513895718	1.78947	3.6401	0.0002725	0.0023022	ENSG00000185974	13	113667218	113737736	GRK1
ENST00000335756	377.2796609	-1.533231474	0.24991	-6.135	8.51E-10	3.10E-08	ENSG00000115163	2	26786055	26794589	CENPA
ENST00000335987	121.926928	-1.605498718	0.40211	-3.993	6.53E-05	0.0006856	ENSG00000172818	11	65787062	65797214	OVOL1
ENST00000336023	11450.38166	-1.183988372	0.20471	-5.784	7.30E-09	2.21E-07	ENSG00000123416	12	49127781	49131395	TUBA1B
ENST00000336119	37.15045945	1.69128488	0.58359	2.8981	0.0037549	0.0200401	ENSG00000162711	1	247416172	247448817	NLRP3
ENST00000336219	340.3273123	-1.706372739	0.27455	-6.215	5.13E-10	1.93E-08	ENSG00000170779	14	105009572	105021083	CDCA4
ENST00000336374	15.36800217	4.253509571	1.16681	3.6454	0.000267	0.0022683	ENSG00000160870	7	99705035	99735196	CYP3A7
ENST00000336375	63.83943619	-4.380591386	0.58646	-7.47	8.05E-14	6.49E-12	ENSG00000014257	3	132317406	132358841	ACP3
ENST00000336395	451.9419008	1.973409113	0.23428	8.4231	3.67E-17	4.60E-15	ENSG00000107140	9	35605261	35610033	TESK1
ENST00000336505	521.5999551	-1.240404034	0.22595	-5.49	4.02E-08	1.02E-06	ENSG00000173611	9	124942607	125143528	SCAI

protein_coding	Yes	NM_194317.5	108
protein_coding	Yes	NM_203412.2	108
protein_coding	Yes	NM_003660.4	108
protein_coding	Yes	NM_145861.4	108
protein_coding	Yes	NM_018393.4	108
protein_coding	Yes	NM_001029870.3	108
protein_coding	Yes	NM_138787.4	108
protein_coding	Yes	NM_017761.4	108
protein_coding	Yes	NM_001109754.4	108
protein_coding	Yes	NM_002599.5	108
protein_coding	Yes	NM_001031738.3	108
protein_coding	Yes	NM_181780.4	108
protein_coding	Yes	NM_001013838.3	108
protein_coding	Yes	NM_001040402.3	108
protein_coding	Yes	NM_006225.4	108
protein_coding	Yes	NM_001249.5	108
protein_coding	Yes	NM_001322331.2	108
protein_coding	Yes	NM_002710.4	108
protein_coding	Yes	NM_198083.4	108
protein_coding	Yes	NM_175736.5	108
protein_coding	Yes	NM_181726.4	108
protein_coding	Yes	NM_005192.4	108
protein_coding	Yes	NM_001008391.4	108
protein_coding	Yes	NM_170744.5	108
protein_coding	Yes	NM_017728.4	108
protein_coding	Yes	NM_032608.7	108
protein_coding	Yes	NM_017901.6	108
protein_coding	Yes	NM_152718.2	108
protein_coding	Yes	NM_001270377.2	108
protein_coding	Yes	NM_134261.3	108
protein_coding	Yes	NM_002929.3	108
protein_coding	Yes	NM_001809.4	108
protein_coding	Yes	NM_004561.4	108
protein_coding	Yes	NM_006082.3	108
protein_coding	Yes	NM_001243133.2	108
protein_coding	Yes	NM_017955.4	108
protein_coding	Yes	NM_000765.5	108
protein_coding	Yes	NM_001099.5	108
protein_coding	Yes	NM_006285.3	108
protein_coding	Yes	NM_001144877.3	108

ENST00000336615	342.2636332	1.461749143	0.24793	5.8959	3.73E-09	1.20E-07	ENSG00000177666	11	818913	825573	PNPLA2
ENST00000336689	155.1716173	-1.159951989	0.3143	-3.691	0.0002237	0.0019519	ENSG00000088280	1	23428562	23484179	ASAP3
ENST00000337080	142.8256087	1.17777747	0.31621	3.7247	0.0001955	0.001739	ENSG00000167685	19	56141286	56160893	ZNF444
ENST00000337109	633.0110124	-1.29037647	0.21535	-5.992	2.07E-09	7.01E-08	ENSG00000132313	2	86199456	86213790	MRPL35
ENST00000337491	140.9602102	1.4041238	0.3159	4.4448	8.80E-06	0.0001204	ENSG00000077463	19	4174108	4182563	SIRT6
ENST00000337523	147.6298477	1.12431475	0.30933	3.6347	0.0002783	0.0023411	ENSG00000204348	6	31969814	31972138	DXO
ENST00000337539	59.34969842	2.158143583	0.46702	4.6211	3.82E-06	5.79E-05	ENSG00000128271	22	24427598	24442357	ADORA2A
ENST00000337554	329.9371412	1.661427469	0.24999	6.6459	3.01E-11	1.50E-09	ENSG00000100300	22	43151558	43163242	TSPO
ENST00000337576	75.31611434	1.579871931	0.41735	3.7855	0.0001534	0.0014147	ENSG00000088899	20	3162616	3173549	LZTS3
ENST00000337619	1722.271324	1.163963624	0.19785	5.883	4.03E-09	1.29E-07	ENSG00000106367	7	101154475	101161276	AP1S1
ENST00000337664	28.49898821	-2.113938091	0.653	-3.237	0.0012068	0.0079671	ENSG00000109576	4	170060221	170089956	AADAT
ENST00000337702	240.3754973	-1.433266876	0.29366	-4.881	1.06E-06	1.87E-05	ENSG00000113638	5	40711575	40755961	TTC33
ENST00000337843	89.75986607	-1.520965236	0.37948	-4.008	6.12E-05	0.0006479	ENSG00000133466	22	37180165	37188247	C1QTNF6
ENST00000337975	153.7787969	1.153575308	0.34439	3.3496	0.0008093	0.0057317	ENSG00000183655	15	85759325	85794925	KLHL25
ENST00000338033	66.84289885	-2.103910708	0.49821	-4.223	2.41E-05	0.0002883	ENSG00000181085	8	143716348	143722458	MAPK15
ENST00000338037	805.3497258	-1.514686792	0.30169	-5.021	5.15E-07	9.97E-06	ENSG00000182621	20	8132265	8884900	PLCB1
ENST00000338074	98.40167952	-1.243311894	0.40031	-3.106	0.001897	0.0115102	ENSG00000088367	20	36154739	36232799	EPB41L1
ENST00000338134	144.9578482	1.684234565	0.32676	5.1543	2.55E-07	5.36E-06	ENSG00000118162	19	47475149	47484219	KPTN
ENST00000338244	551.8273377	-1.258920272	0.22168	-5.679	1.36E-08	3.85E-07	ENSG00000089057	20	4852357	5001510	SLC23A2
ENST00000338415	5.897520832	5.986731667	1.91891	3.1199	0.0018094	0.0110864	ENSG00000115828	2	37344629	37373322	QPCT
ENST00000338432	15.12301433	-2.373102972	0.892	-2.66	0.0078042	0.0359466	ENSG00000076555	12	109116586	109268226	ACACB
ENST00000338483	316.3471998	1.28023305	0.27303	4.6889	2.75E-06	4.36E-05	ENSG00000185022	22	38201993	38216507	MAFF
ENST00000338492	41.44627417	2.2234855	0.55827	3.9828	6.81E-05	0.0007105	ENSG00000151640	10	132186947	132205759	DPYSL4
ENST00000338513	77.42559794	-1.043795268	0.40336	-2.588	0.0096603	0.0426415	ENSG00000189280	1	34755046	34758512	GJB5
ENST00000338523	313.2231771	-1.205145347	0.2584	-4.664	3.10E-06	4.84E-05	ENSG00000086300	7	26291861	26374383	SNX10
ENST00000338745	74.86566359	-3.690780824	0.49635	-7.436	1.04E-13	8.27E-12	ENSG00000182568	3	18345376	18425339	SATB1
ENST00000338784	91.00435327	1.740084117	0.37972	4.5826	4.59E-06	6.83E-05	ENSG00000161955	17	7558749	7561601	TNFSF13
ENST00000338961	412.8313714	-1.423666153	0.2523	-5.643	1.67E-08	4.64E-07	ENSG00000148334	9	128120692	128127729	PTGES2
ENST00000339235	164.8223534	1.001264987	0.3496	2.864	0.0041829	0.0219079	ENSG00000171823	12	1565992	1594581	FBXL14
ENST00000339276	1322.810667	-1.355461532	0.21807	-6.216	5.11E-10	1.93E-08	ENSG00000175793	1	26863148	26864456	SFN
ENST00000339394	29.22212119	-2.313657592	0.65229	-3.547	0.0003897	0.0031098	ENSG00000162512	1	30869465	30908758	SDC3
ENST00000339399	269.9543522	-1.181063861	0.25704	-4.595	4.33E-06	6.49E-05	ENSG00000136938	9	97983340	98015943	ANP32B
ENST00000339450	1065.569266	-1.119105459	0.21032	-5.321	1.03E-07	2.37E-06	ENSG00000099219	9	5784571	5833117	ERMP1
ENST00000339562	27.9489174	2.15769724	0.74472	2.8973	0.0037637	0.0200792	ENSG00000153234	2	156324436	156332721	NR4A2
ENST00000339686	511.6125074	-1.409317059	0.21517	-6.55	5.76E-11	2.65E-09	ENSG00000161249	19	35497219	35513649	DMKN
ENST00000339732	18.21008821	7.615038659	1.61743	4.7081	2.50E-06	4.02E-05	ENSG00000131386	3	16174679	16230165	GALNT15
ENST00000339812	1737.832104	-1.598677782	0.18196	-8.786	1.55E-18	2.29E-16	ENSG00000124635	6	27132315	27132795	H2BC11
ENST00000339818	121.5415745	1.066400037	0.34146	3.1231	0.0017898	0.0109891	ENSG00000160226	21	44328943	44339390	CFAP410
ENST00000339824	113.3817932	1.445065247	0.43437	3.3268	0.0008786	0.0061218	ENSG00000171435	12	117453011	117968990	KSR2
ENST00000339834	1272.615058	1.276727489	0.19304	6.6139	3.74E-11	1.82E-09	ENSG00000166275	10	102854258	102864961	BORCS7

protein_coding	Yes	NM_020376.4	108
protein_coding	Yes	NM_017707.4	108
protein_coding	Yes	NM_018337.4	108
protein_coding	Yes	NM_016622.4	108
protein_coding	Yes	NM_016539.4	108
protein_coding	Yes	NM_005510.4	108
protein_coding	Yes	NM_000675.6	108
protein_coding	Yes	NM_000714.6	108
protein_coding	Yes	NM_001365618.1	108
protein_coding	Yes	NM_001283.5	108
protein_coding	Yes	NM_016228.4	108
protein_coding	Yes	NM_012382.3	108
protein_coding	Yes	NM_031910.4	108
protein_coding	Yes	NM_022480.4	108
protein_coding	Yes	NM_139021.3	108
protein_coding	Yes	NM_015192.4	108
protein_coding	Yes	NM_012156.2	108
protein_coding	Yes	NM_007059.4	108
protein_coding	Yes	NM_005116.6	108
protein_coding	Yes	NM_012413.4	108
protein_coding	Yes	NM_001093.4	108
protein_coding	Yes	NM_012323.4	108
protein_coding	Yes	NM_006426.3	108
protein_coding	Yes	NM_005268.4	108
protein_coding	Yes	NM_013322.3	108
protein_coding	Yes	NM_002971.6	108
protein_coding	Yes	NM_003808.4	108
protein_coding	Yes	NM_025072.7	108
protein_coding	Yes	NM_152441.3	108
protein_coding	Yes	NM_006142.5	108
protein_coding	Yes	NM_014654.4	108
protein_coding	Yes	NM_006401.3	108
protein_coding	Yes	NM_024896.3	108
protein_coding	Yes	NM_006186.4	108
protein_coding	Yes	NM_033317.5	108
protein_coding	Yes	NM_054110.5	108
protein_coding	Yes	NM_021058.4	108
protein_coding	Yes	NM_004928.3	108
protein_coding	Yes	NM_173598.6	108
protein_coding	Yes	NM_001136200.2	108

ENST00000339950	3167.044437	-1.835961131	0.1711	-10.73	7.35E-27	3.06E-24	ENSG00000162607	1	62437048	62451804	USP1
ENST00000340006	396.5329119	-2.960705868	0.81612	-3.628	0.0002859	0.0023941	ENSG00000159176	1	201483529	201507123	CSRP1
ENST00000340083	42.85285497	-1.881842646	0.61429	-3.063	0.002188	0.0129494	ENSG00000175920	4	3463305	3494482	DOK7
ENST00000340093	615.6009147	1.004141506	0.20785	4.8312	1.36E-06	2.34E-05	ENSG00000011422	19	43648578	43670169	PLAUR
ENST00000340099	186.7763132	-1.526129984	0.28644	-5.328	9.93E-08	2.29E-06	ENSG00000125170	16	57471921	57486493	DOK4
ENST00000340124	1307.199366	-1.089171274	0.18451	-5.903	3.57E-09	1.15E-07	ENSG00000166123	16	46884361	46931289	GPT2
ENST00000340157	448.7758793	-1.130413952	0.22259	-5.079	3.80E-07	7.64E-06	ENSG00000028116	2	58046805	58159871	VRK2
ENST00000340181	3.916465705	5.396742222	2.11677	2.5495	0.0107872	0.0464746	ENSG00000010030	6	36366193	36387669	ETV7
ENST00000340249	12.96188151	7.123531504	1.67747	4.2466	2.17E-05	0.0002631	ENSG00000291004	15	22610352	22617854	GOLGA8IP
ENST00000340510	30.93165671	1.555466335	0.61056	2.5476	0.0108468	0.0466852	ENSG00000188185	7	39733631	39793092	LINC00265
ENST00000340607	534.9134813	-4.402395214	0.25614	-17.19	3.29E-66	2.51E-62	ENSG00000148344	9	129738348	129753042	PTGES
ENST00000340828	1657.0652	-1.299726528	0.18844	-6.897	5.30E-12	3.10E-10	ENSG00000113328	5	163437594	163445016	CCNG1
ENST00000340857	1706.867523	-2.059928566	0.2011	-10.24	1.27E-24	4.00E-22	ENSG00000189060	22	37805228	37807432	H1-0
ENST00000340866	467.0808698	-2.641373983	0.25787	-10.24	1.27E-24	4.00E-22	ENSG00000116791	1	74705485	74733050	CRYZ
ENST00000341023	1970.08051	-1.191566561	0.18235	-6.534	6.38E-11	2.92E-09	ENSG00000196866	6	26198783	26199293	H2AC7
ENST00000341049	1821.674148	-1.521982463	0.19306	-7.884	3.18E-15	3.15E-13	ENSG00000105974	7	116525035	116561179	CAV1
ENST00000341119	369.4396686	-1.071602745	0.23331	-4.593	4.37E-06	6.54E-05	ENSG00000075303	7	87833567	87876360	SLC25A40
ENST00000341156	245.4795404	2.132798377	0.28042	7.6058	2.83E-14	2.45E-12	ENSG00000135454	12	57623408	57633201	B4GALNT1
ENST00000341184	246.7431188	1.308294158	0.27843	4.6989	2.62E-06	4.18E-05	ENSG00000128268	22	39457011	39492194	MGAT3
ENST00000341259	114.5816576	1.747611927	0.34734	5.0314	4.87E-07	9.48E-06	ENSG00000111252	12	111405922	111451623	SH2B3
ENST00000341420	9.810843504	-5.297814641	1.7224	-3.076	0.0020991	0.0125084	ENSG00000125848	20	14322984	14337612	FLRT3
ENST00000341423	4722.352603	-2.025824969	0.18601	-10.89	1.27E-27	5.86E-25	ENSG00000189403	13	30456703	30465936	HMGB1
ENST00000341469	207.0328743	-1.853203813	0.32106	-5.772	7.82E-09	2.35E-07	ENSG00000106785	9	98084352	98119222	TRIM14
ENST00000341695	999.1697889	-1.087654392	0.19262	-5.647	1.64E-08	4.54E-07	ENSG00000138777	4	105369076	105474062	PPA2
ENST00000341864	207.0984909	-1.608684763	0.28463	-5.652	1.59E-08	4.44E-07	ENSG00000187325	X	78129747	78139650	TAF9B
ENST00000341911	52.88010108	-2.373113789	0.50067	-4.74	2.14E-06	3.49E-05	ENSG00000118513	6	135181307	135219172	MYB
ENST00000341947	55.43513608	1.566848179	0.49591	3.1595	0.0015803	0.0099264	ENSG00000204248	6	33162693	33192467	COL11A2
ENST00000341948	9.911904929	-4.267384033	1.40272	-3.042	0.0023483	0.0137075	ENSG00000187372	5	141213918	141218979	PCDHB13
ENST00000342203	571.4735365	-1.346516696	0.21042	-6.399	1.56E-10	6.56E-09	ENSG00000117155	1	84643705	84690461	SSX2IP
ENST00000342232	53.68150007	-3.571853201	0.5712	-6.253	4.02E-10	1.55E-08	ENSG00000104369	8	74234699	74321540	JPH1
ENST00000342291	73.03168802	1.094129273	0.42216	2.5917	0.0095492	0.0422413	ENSG00000182324	19	48455573	48466980	KCNJ14
ENST00000342435	692.0955055	-1.389409394	0.3049	-4.557	5.19E-06	7.60E-05	ENSG00000188313	3	146515179	146544607	PLSCR1
ENST00000342456	30.14092741	-1.890920359	0.67406	-2.805	0.0050276	0.0253966	ENSG00000006534	11	68010326	68029276	ALDH3B1
ENST00000342462	106.2996584	1.553683761	0.3541	4.3877	1.15E-05	0.0001512	ENSG00000188167	3	33090421	33097146	TMPPE
ENST00000342494	608.2837598	1.074629103	0.21322	5.0401	4.65E-07	9.12E-06	ENSG00000151148	12	109477633	109536702	UBE3B
ENST00000342665	111.6114098	1.439742891	0.44934	3.2041	0.0013547	0.0087559	ENSG00000177732	20	325551	330224	SOX12
ENST00000342679	197.0233295	1.549090592	0.28489	5.4375	5.40E-08	1.33E-06	ENSG00000034152	17	21284710	21315232	MAP2K3
ENST00000342683	104.2094184	1.096726989	0.36742	2.9849	0.0028363	0.0159565	ENSG00000189227	15	67521130	67530146	C15orf61
ENST00000342784	53.59674088	-1.958839768	0.48134	-4.07	4.71E-05	0.0005161	ENSG00000105137	19	15107400	15114974	SYDE1
ENST00000342951	10.53430297	-5.384345116	1.76458	-3.051	0.0022782	0.0133729	ENSG00000204941	19	43167742	43186536	PSG5

protein_coding	Yes	NM_003368.5	108
protein_coding	Yes	NM_004078.3	108
protein_coding	Yes	NM_173660.5	108
protein_coding	Yes	NM_002659.4	108
protein_coding	Yes	NM_018110.5	108
protein_coding	Yes	NM_133443.4	108
protein_coding	Yes	NM_006296.7	108
protein_coding	Yes	NM_016135.4	108
lncRNA	Yes	-	108
lncRNA	Yes	-	108
protein_coding	Yes	NM_004878.5	108
protein_coding	Yes	NM_004060.4	108
protein_coding	Yes	NM_005318.4	108
protein_coding	Yes	NM_001889.4	108
protein_coding	Yes	NM_021065.3	108
protein_coding	Yes	NM_001753.5	108
protein_coding	Yes	NM_018843.4	108
protein_coding	Yes	NM_001478.5	108
protein_coding	Yes	NM_002409.5	108
protein_coding	Yes	NM_005475.3	108
protein_coding	Yes	NM_198391.3	108
protein_coding	Yes	NM_002128.7	108
protein_coding	Yes	NM_014788.4	108
protein_coding	Yes	NM_176869.3	108
protein_coding	Yes	NM_015975.5	108
protein_coding	Yes	NM_001130173.2	108
protein_coding	Yes	NM_080680.3	108
protein_coding	Yes	NM_018933.4	108
protein_coding	Yes	NM_001166293.2	108
protein_coding	Yes	NM_020647.4	108
protein_coding	Yes	NM_013348.4	108
protein_coding	Yes	NM_021105.3	108
protein_coding	Yes	NM_000694.4	108
protein_coding	Yes	NM_001039770.3	108
protein_coding	Yes	NM_130466.4	108
protein_coding	Yes	NM_006943.4	108
protein_coding	Yes	NM_145109.3	108
protein_coding	Yes	NM_001143936.2	108
protein_coding	Yes	NM_033025.6	108
protein_coding	Yes	NM_002781.4	108

ENST00000343410	7.625425503	4.860669716	1.79883	2.7021	0.0068896	0.032621	ENSG00000143512	1	222522263	222548104	HHIPL2
ENST00000343677	6513.311425	-1.290886994	0.16626	-7.764	8.20E-15	7.61E-13	ENSG00000187837	6	26055739	26056470	H1-2
ENST00000343736	44.88683685	1.730327256	0.52281	3.3096	0.0009342	0.0064358	ENSG00000005379	17	58301230	58328795	TSPOAP1
ENST00000343959	56.77107568	9.254922164	1.50671	6.1425	8.13E-10	2.97E-08	ENSG00000188100	10	87020293	87024730	FAM25A
ENST00000344096	608.5638554	-1.533553729	0.23172	-6.618	3.63E-11	1.77E-09	ENSG00000127334	12	67648744	67665406	DYRK2
ENST00000344175	183.0272203	1.110760147	0.29609	3.7515	0.0001758	0.001589	ENSG00000204673	19	49869038	49877355	AKT1S1
ENST00000344279	718.9410857	3.817952186	0.23302	16.385	2.46E-60	1.17E-56	ENSG00000235109	6	28324736	28336148	ZSCAN31
ENST00000344318	287.2898152	1.906368413	0.25979	7.338	2.17E-13	1.65E-11	ENSG00000100319	22	29730955	29767007	ZMAT5
ENST00000344320	302.8011196	4.276275685	0.31628	13.52	1.19E-41	2.75E-38	ENSG00000188277	15	40769979	40772449	C15orf62
ENST00000344517	620.4522287	-1.62839168	0.20729	-7.856	3.98E-15	3.89E-13	ENSG00000135776	1	229516581	229558707	ABCB10
ENST00000344532	61.45195086	-1.567120749	0.47291	-3.314	0.0009205	0.0063585	ENSG00000136161	13	48488962	48533080	RCBTB2
ENST00000344629	137.6018892	1.440754752	0.32373	4.4505	8.57E-06	0.0001177	ENSG00000114026	3	9749951	9757407	OGG1
ENST00000344646	35.14031083	1.852004074	0.59853	3.0942	0.0019731	0.0118846	ENSG00000172671	10	45615499	45672772	ZFAND4
ENST00000344686	358.4110428	1.98833901	0.24141	8.2364	1.78E-16	2.05E-14	ENSG00000006062	17	45263118	45317020	MAP3K14
ENST00000344726	1015.545365	-1.159517654	0.1974	-5.874	4.26E-09	1.35E-07	ENSG00000118418	6	79201244	79234689	HMGN3
ENST00000344754	5.686975136	5.933397777	1.94052	3.0576	0.0022309	0.0131548	ENSG00000088827	20	3686969	3712600	SIGLEC1
ENST00000344924	787.0320724	1.712703594	0.20167	8.4928	2.02E-17	2.63E-15	ENSG00000172331	7	134646852	134679816	BPGM
ENST00000345136	15324.33893	1.316151329	0.18079	7.2799	3.34E-13	2.47E-11	ENSG00000178209	8	143915152	143939597	PLEC
ENST00000345146	2361.455591	-1.105378801	0.22864	-4.835	1.33E-06	2.30E-05	ENSG00000138413	2	208236228	208255071	IDH1
ENST00000345254	2994.987618	1.489123056	0.17717	8.4049	4.28E-17	5.31E-15	ENSG00000176986	10	73744371	73772161	SEC24C
ENST00000345264	300.354803	-1.113920963	0.24515	-4.544	5.52E-06	8.02E-05	ENSG00000148484	10	16590610	16817424	RSU1
ENST00000345363	81.32255206	1.083030424	0.39712	2.7272	0.0063872	0.0307198	ENSG00000213903	14	24311501	24318036	LTB4R
ENST00000345365	197.6016741	-1.008862499	0.28898	-3.491	0.0004809	0.0037154	ENSG00000185379	17	35092220	35119860	RAD51D
ENST00000345851	103.3178326	1.100061681	0.35352	3.1118	0.0018597	0.0113286	ENSG00000110171	11	6448612	6473941	TRIM3
ENST00000346436	202.4135612	1.004384557	0.2893	3.4718	0.000517	0.0039478	ENSG00000011021	1	11806190	11843130	CLCN6
ENST00000346473	603.6084937	1.839991977	0.21374	8.6085	7.41E-18	1.03E-15	ENSG00000175197	12	57516592	57520514	DDIT3
ENST00000346872	4.337557096	5.546371557	2.07077	2.6784	0.0073974	0.034454	ENSG00000161405	17	39757717	39864312	IKZF3
ENST00000347055	164.642295	-1.136915921	0.31927	-3.561	0.0003695	0.0029717	ENSG00000172086	2	88027204	88055783	KRCC1
ENST00000347077	28.80423252	2.842670604	0.69531	4.0883	4.34E-05	0.0004815	ENSG00000187187	19	39997059	40021038	ZNF546
ENST00000347162	245.273456	1.561198742	0.31727	4.9207	8.62E-07	1.56E-05	ENSG00000137171	6	43059630	43075093	KLC4
ENST00000347230	814.9364073	1.060541413	0.19603	5.41	6.30E-08	1.53E-06	ENSG00000072121	14	67746521	67816590	ZFYVE26
ENST00000347512	1806.916857	-1.065112875	0.18072	-5.894	3.77E-09	1.21E-07	ENSG00000130255	19	5690294	5691875	RPL36
ENST00000348227	72.84356359	-1.6543504	0.41427	-3.993	6.51E-05	0.0006838	ENSG00000005448	2	74421729	74425755	WDR54
ENST00000348354	211.32696	-1.32472496	0.27327	-4.848	1.25E-06	2.17E-05	ENSG00000154640	21	17593652	17612901	BTG3
ENST00000348993	297.1931907	1.83059855	0.25136	7.2826	3.27E-13	2.43E-11	ENSG00000291159	15	84631450	84647478	-
ENST00000349064	13.59055966	3.273322578	1.05555	3.101	0.0019284	0.0116541	ENSG00000213199	7	151048530	151052754	ASIC3
ENST00000349077	9.489329471	6.674387606	1.75452	3.8041	0.0001423	0.0013258	ENSG00000118004	2	3595111	3644644	COLEC11
ENST00000349721	553.6536171	-1.033058819	0.21972	-4.702	2.58E-06	4.13E-05	ENSG00000080503	9	2015346	2193620	SMARCA2
ENST00000349847	277.3881859	-2.566861837	0.33358	-7.695	1.42E-14	1.27E-12	ENSG00000136155	13	77535705	77645263	SCEL
ENST00000349880	325.3765222	-1.118601363	0.34592	-3.234	0.0012221	0.008047	ENSG00000166833	11	19712836	20121601	NAV2

protein_coding	Yes	NM_024746.4	108
protein_coding	Yes	NM_005319.4	108
protein_coding	Yes	NM_004758.4	108
protein_coding	Yes	NM_001146157.3	108
protein_coding	Yes	NM_006482.3	108
protein_coding	Yes	NM_001098633.4	108
protein_coding	Yes	NM_030899.5	108
protein_coding	Yes	NM_001003692.2	108
protein_coding	Yes	NM_001130448.3	108
protein_coding	Yes	NM_012089.3	108
protein_coding	Yes	NM_001268.4	108
protein_coding	Yes	NM_002542.6	108
protein_coding	Yes	NM_174890.4	108
protein_coding	Yes	NM_003954.5	108
protein_coding	Yes	-	108
protein_coding	Yes	NM_023068.4	108
protein_coding	Yes	NM_001724.5	108
protein_coding	Yes	NM_201384.3	108
protein_coding	Yes	NM_005896.4	108
protein_coding	Yes	NM_198597.3	108
protein_coding	Yes	NM_012425.4	108
protein_coding	Yes	NM_001143919.3	108
protein_coding	Yes	NM_002878.4	108
protein_coding	Yes	NM_033278.4	108
protein_coding	Yes	NM_001286.5	108
protein_coding	Yes	NM_004083.6	108
protein_coding	Yes	NM_012481.5	108
protein_coding	Yes	NM_016618.3	108
protein_coding	Yes	NM_178544.5	108
protein_coding	Yes	NM_201521.3	108
protein_coding	Yes	NM_015346.4	108
protein_coding	Yes	NM_033643.3	108
protein_coding	Yes	NM_032118.4	108
protein_coding	Yes	NM_006806.5	108
lncRNA	Yes	-	108
protein_coding	Yes	NM_004769.4	108
protein_coding	Yes	NM_024027.5	108
protein_coding	Yes	NM_003070.5	108
protein_coding	Yes	NM_144777.3	108
protein_coding	Yes	NM_145117.5	108

ENST00000350030	1183.277147	-1.786239288	0.19356	-9.228	2.75E-20	4.95E-18	ENSG00000132780	1	45584040	45618893	NASP
ENST00000350051	1332.321664	-1.736690474	0.20009	-8.679	3.98E-18	5.65E-16	ENSG00000089685	17	78214252	78225635	BIRC5
ENST00000350060	24.62030558	2.569201796	0.73933	3.4751	0.0005108	0.0039057	ENSG00000089820	X	153907377	153926264	ARHGAP4
ENST00000350320	2883.218378	-1.054902504	0.17164	-6.146	7.94E-10	2.90E-08	ENSG00000122545	7	35801056	35907110	SEPTIN7
ENST00000350881	34.88489323	2.558799704	0.61213	4.1802	2.91E-05	0.0003395	ENSG00000113296	5	80035347	80083287	THBS4
ENST00000350997	573.4486826	-1.189078404	0.22776	-5.221	1.78E-07	3.88E-06	ENSG00000149485	11	61799626	61817003	FADS1
ENST00000351298	114.2567764	-2.191851923	0.354	-6.192	5.95E-10	2.23E-08	ENSG00000075651	3	171600403	171810483	PLD1
ENST00000351486	98.54705815	-1.228284444	0.36376	-3.377	0.0007339	0.0052886	ENSG00000113734	5	173144530	173164387	BNIP1
ENST00000352241	8.123156767	-4.999925483	1.77654	-2.814	0.0048867	0.0248173	ENSG00000187098	3	69739463	69968332	MITF
ENST00000352410	173.395179	-1.477846628	0.29737	-4.97	6.71E-07	1.25E-05	ENSG00000178802	15	74890041	74902219	MPI
ENST00000352433	938.9130781	-1.346270739	0.19531	-6.893	5.47E-12	3.18E-10	ENSG00000164611	5	160421854	160428739	PTTG1
ENST00000353267	788.1028416	-2.268976619	0.20619	-11	3.64E-28	1.82E-25	ENSG00000118260	2	207529961	207605988	CREB1
ENST00000354181	634.0637953	1.157356911	0.2049	5.6483	1.62E-08	4.51E-07	ENSG00000140199	15	34229783	34337781	SLC12A6
ENST00000354193	779.5232045	-1.007445527	0.20757	-4.854	1.21E-06	2.11E-05	ENSG00000198168	11	22818926	22829801	SVIP
ENST00000354258	486.8967283	1.793555216	0.23588	7.6035	2.88E-14	2.48E-12	ENSG00000168394	6	32845208	32853704	TAP1
ENST00000354287	1880.013801	1.999754183	0.1902	10.514	7.43E-26	2.70E-23	ENSG00000116661	1	11648386	11654429	FBXO2
ENST00000354421	2197.628119	1.506063491	0.21747	6.9253	4.35E-12	2.58E-10	ENSG00000182919	11	93741671	93764749	C11orf54
ENST00000354586	690.8595409	-1.024289707	0.21012	-4.875	1.09E-06	1.92E-05	ENSG00000198252	14	52730165	52774989	STYX
ENST00000354764	302.8075547	-1.067114879	0.2829	-3.772	0.0001619	0.0014823	ENSG00000197121	2	196833003	196926707	PGAP1
ENST00000354817	735.0579758	-1.352793572	0.19988	-6.768	1.30E-11	6.99E-10	ENSG00000197601	11	13668667	13732346	FAR1
ENST00000354852	314.5991427	-1.075387971	0.24703	-4.353	1.34E-05	0.0001738	ENSG00000258588	11	5596724	5644398	TRIM6-TRIM34
ENST00000354919	363.7533158	-1.850598382	0.24346	-7.601	2.93E-14	2.52E-12	ENSG00000152455	10	14878865	14904315	SUV39H2
ENST00000354960	132.8772917	-3.093408801	0.36043	-8.583	9.28E-18	1.27E-15	ENSG00000138735	4	119494402	119628804	PDE5A
ENST00000355029	1868.833284	-2.211203922	0.19038	-11.61	3.46E-31	2.25E-28	ENSG00000173848	10	5412556	5459056	NET1
ENST00000355057	120.4452416	-1.607101224	0.34564	-4.65	3.33E-06	5.14E-05	ENSG00000197238	6	27824091	27824480	H4C11
ENST00000355059	86.63481571	1.438406313	0.40231	3.5753	0.0003498	0.0028371	ENSG00000140398	15	75347038	75357115	NEIL1
ENST00000355085	17.70784229	2.398336263	0.93629	2.5615	0.010421	0.0453142	ENSG00000197405	19	47309860	47322066	C5AR1
ENST00000355086	224.9514849	1.293580748	0.39181	3.3015	0.0009616	0.0065922	ENSG00000196935	12	63844699	64162217	SRGAP1
ENST00000355202	692.6048803	1.290212481	0.20204	6.386	1.70E-10	7.09E-09	ENSG00000198182	19	37696370	37719761	ZNF607
ENST00000355285	152.835611	-1.118669283	0.36512	-3.064	0.0021854	0.0129368	ENSG00000154856	18	10454634	10489949	APCDD1
ENST00000355303	212.0991522	-3.090642149	0.30526	-10.12	4.29E-24	1.30E-21	ENSG00000131620	11	70078316	70189530	ANO1
ENST00000355315	183.7523679	-1.217732759	0.28822	-4.225	2.39E-05	0.0002859	ENSG00000170791	8	56211788	56218809	CHCHD7
ENST00000355485	2257.536466	-1.370116176	0.17637	-7.768	7.95E-15	7.39E-13	ENSG00000173218	1	115641969	115698221	VANGL1
ENST00000355502	21.13474978	2.699141294	0.87739	3.0763	0.0020958	0.0124968	ENSG00000285188	19	18207960	18255419	-
ENST00000355522	122.8901509	2.498033065	0.45949	5.4366	5.43E-08	1.34E-06	ENSG00000198535	15	62066976	62070917	C2CD4A
ENST00000355526	8.579593361	-6.57572381	1.78386	-3.686	0.0002276	0.0019796	ENSG00000011332	19	38211005	38224222	DPF1
ENST00000355527	595.0646642	-1.157388516	0.22735	-5.091	3.56E-07	7.22E-06	ENSG00000172893	11	71434410	71448393	DHCR7
ENST00000355577	92.50295123	1.451295185	0.45675	3.1774	0.001486	0.0094538	ENSG00000156042	10	73253761	73358864	CFAP70
ENST00000355610	291.6241611	-1.280691699	0.26199	-4.888	1.02E-06	1.81E-05	ENSG00000133131	X	106940737	107000212	MORC4
ENST00000355673	113.8010983	1.640766991	0.38087	4.3079	1.65E-05	0.0002072	ENSG00000166987	12	57522823	57530148	MBD6

protein_coding	Yes	NM_002482.4	108
protein_coding	Yes	NM_001168.3	108
protein_coding	Yes	NM_001666.5	108
protein_coding	Yes	NM_001788.6	108
protein_coding	Yes	NM_003248.6	108
protein_coding	Yes	NM_013402.7	108
protein_coding	Yes	NM_002662.5	108
protein_coding	Yes	NM_001205.3	108
protein_coding	Yes	NM_001354604.2	108
protein_coding	Yes	NM_002435.3	108
protein_coding	Yes	NM_004219.4	108
protein_coding	Yes	NM_004379.5	108
protein_coding	Yes	NM_001365088.1	108
protein_coding	Yes	NM_148893.3	108
protein_coding	Yes	NM_000593.6	108
protein_coding	Yes	NM_012168.6	108
protein_coding	Yes	NM_001286069.2	108
protein_coding	Yes	NM_145251.4	108
protein_coding	Yes	NM_024989.4	108
protein_coding	Yes	NM_032228.6	108
protein_coding	Yes	-	108
protein_coding	Yes	NM_001193424.2	108
protein_coding	Yes	NM_001083.4	108
protein_coding	Yes	NM_001047160.3	108
protein_coding	Yes	NM_021968.4	108
protein_coding	Yes	NM_024608.4	108
protein_coding	Yes	NM_001736.4	108
protein_coding	Yes	NM_020762.4	108
protein_coding	Yes	NM_032689.5	108
protein_coding	Yes	NM_153000.5	108
protein_coding	Yes	NM_018043.7	108
protein_coding	Yes	NM_001011671.3	108
protein_coding	Yes	NM_138959.3	108
protein_coding	Yes	-	108
protein_coding	Yes	NM_207322.3	108
protein_coding	Yes	NM_001135155.3	108
protein_coding	Yes	NM_001360.3	108
protein_coding	Yes	NM_001367801.1	108
protein_coding	Yes	NM_024657.5	108
protein_coding	Yes	NM_052897.4	108

ENST00000355703	1331.697383	1.069893817	0.18499	5.7835	7.32E-09	2.21E-07	ENSG00000197136	11	65615775	65637439	PCNX3
ENST00000355716	90.54921542	-1.128824198	0.38534	-2.929	0.0033961	0.0185027	ENSG00000157873	1	2556370	2563829	TNFRSF14
ENST00000355749	742.0604653	-1.754963033	0.22294	-7.872	3.50E-15	3.44E-13	ENSG00000128609	7	123536996	123557818	NDUFA5
ENST00000355773	1152.371826	1.429705233	0.18733	7.6321	2.31E-14	2.02E-12	ENSG00000119899	6	73593378	73653992	SLC17A5
ENST00000355857	513.438494	-1.36935034	0.21985	-6.229	4.70E-10	1.79E-08	ENSG00000155368	2	119366976	119372543	DBI
ENST00000355897	105.2496116	-3.167002047	0.43989	-7.199	6.05E-13	4.26E-11	ENSG00000173890	3	170037994	170085392	GPR160
ENST00000355899	2674.266626	-1.677645234	0.17563	-9.552	1.27E-21	2.79E-19	ENSG00000102024	X	115561173	115650861	PLS3
ENST00000355904	445.6402851	-1.335318367	0.22883	-5.835	5.36E-09	1.67E-07	ENSG00000197265	8	30578317	30658236	GTF2E2
ENST00000355999	100.2246255	-1.109764392	0.35671	-3.111	0.0018638	0.0113491	ENSG00000198648	2	167954021	168247595	STK39
ENST00000356031	19.51909225	-2.813231568	0.82656	-3.404	0.0006652	0.0048706	ENSG00000152582	5	35617862	35814611	SPEF2
ENST00000356080	1728.960073	-1.569952599	0.17868	-8.787	1.54E-18	2.29E-16	ENSG00000148700	10	110007983	110135565	ADD3
ENST00000356083	28.70414011	1.830055061	0.63669	2.8743	0.0040491	0.0213317	ENSG00000196739	9	114155536	114312511	COL27A1
ENST00000356151	86.28768407	-1.844228653	0.38972	-4.732	2.22E-06	3.61E-05	ENSG00000168297	3	58332891	58426127	PXK
ENST00000356166	326.7382111	1.137505878	0.25403	4.4778	7.54E-06	0.0001054	ENSG00000156860	16	30658430	30670810	FBRS
ENST00000356231	735.2450075	1.227828724	0.25272	4.8585	1.18E-06	2.07E-05	ENSG00000140326	15	42723543	42737128	CDAN1
ENST00000356232	320.8871933	-1.404870612	0.24754	-5.675	1.38E-08	3.93E-07	ENSG00000081307	3	132660328	132679794	UBA5
ENST00000356244	1760.199927	-1.034276334	0.21958	-4.71	2.47E-06	3.98E-05	ENSG00000100401	22	41244778	41286187	RANGAP1
ENST00000356371	20.22702629	-2.393644792	0.81678	-2.931	0.0033831	0.0184497	ENSG00000109794	4	186105076	186172662	FAM149A
ENST00000356404	538.1488583	-1.04749619	0.21219	-4.937	7.95E-07	1.46E-05	ENSG00000110697	11	67491767	67505363	PITPNM1
ENST00000356443	15.47930895	2.352422329	0.88436	2.66	0.0078137	0.0359795	ENSG00000101605	18	3066806	3219968	MYOM1
ENST00000356524	148.346429	10.64045483	1.47352	7.2211	5.16E-13	3.67E-11	ENSG00000173432	11	18266263	18269967	SAA1
ENST00000356530	740.7800555	-1.593661209	0.2046	-7.789	6.75E-15	6.34E-13	ENSG00000277224	6	26199515	26199988	H2BC7
ENST00000356575	209.5106525	2.000531534	0.31254	6.4009	1.54E-10	6.50E-09	ENSG00000162591	1	3487950	3611508	MEGF6
ENST00000356591	147.0442843	-1.177567197	0.3269	-3.602	0.0003155	0.0026014	ENSG00000178878	12	12785658	12791466	APOLD1
ENST00000356660	50.80931226	1.348002865	0.49229	2.7382	0.006177	0.0299049	ENSG00000176697	11	27654892	27700455	BDNF
ENST00000356708	1903.126156	-1.320231254	0.17742	-7.441	9.97E-14	7.95E-12	ENSG00000165629	10	7788176	7807801	ATP5F1C
ENST00000356709	143.4554295	1.204518966	0.33648	3.5797	0.000344	0.0027975	ENSG00000124508	6	26383107	26394874	BTN2A2
ENST00000356839	1430.459803	-2.760068777	0.2024	-13.64	2.43E-42	5.99E-39	ENSG00000072778	17	7219937	7225266	ACADVL
ENST00000356884	921.7042933	-1.089542112	0.19563	-5.569	2.56E-08	6.78E-07	ENSG00000185963	9	92711362	92764833	BICD2
ENST00000356950	2362.446485	-1.612571361	0.17462	-9.235	2.59E-20	4.66E-18	ENSG00000197903	6	27146360	27146855	H2BC12
ENST00000356956	4779.589201	1.748483199	0.16816	10.398	2.54E-25	8.57E-23	ENSG00000197081	6	159969081	160111504	IGF2R
ENST00000356978	2069.670615	-1.688499966	0.17751	-9.512	1.87E-21	4.01E-19	ENSG00000198668	14	90397028	90408268	CALM1
ENST00000357003	87.38197781	1.145781137	0.41146	2.7847	0.0053582	0.0267383	ENSG00000144843	3	119579491	119589945	ADPRH
ENST00000357060	53.61943869	2.540870209	0.51184	4.9642	6.90E-07	1.28E-05	ENSG00000197748	10	104129887	104232364	CFAP43
ENST00000357068	81.82051704	-2.689273211	0.42265	-6.363	1.98E-10	8.13E-09	ENSG00000100033	22	18912780	18936293	PRODH
ENST00000357195	10.47158298	-4.377013434	1.41765	-3.088	0.0020183	0.0121078	ENSG00000127152	14	99169286	99272197	BCL11B
ENST00000357214	3518.68042	-1.118411253	0.17277	-6.473	9.58E-11	4.23E-09	ENSG00000116560	1	35182935	35193145	SFPQ
ENST00000357242	9.862894111	-5.304764612	1.72233	-3.08	0.00207	0.0123689	ENSG00000133138	X	106802672	106876150	TBC1D8B
ENST00000357388	3195.821425	-1.767128711	0.17244	-10.25	1.21E-24	3.83E-22	ENSG00000113810	3	160399649	160434953	SMC4
ENST00000357430	4453.708964	-1.636339159	0.17007	-9.622	6.47E-22	1.46E-19	ENSG00000123983	2	222861035	222944639	ACSL3

protein_coding	Yes	NM_032223.4	108
protein_coding	Yes	NM_003820.4	108
protein_coding	Yes	NM_005000.5	108
protein_coding	Yes	NM_012434.5	108
protein_coding	Yes	NM_001079862.4	108
protein_coding	Yes	NM_014373.3	108
protein_coding	Yes	NM_005032.7	108
protein_coding	Yes	NM_002095.6	108
protein_coding	Yes	NM_013233.3	108
protein_coding	Yes	NM_024867.4	108
protein_coding	Yes	NM_016824.5	108
protein_coding	Yes	NM_032888.4	108
protein_coding	Yes	NM_017771.5	108
protein_coding	Yes	NM_001105079.3	108
protein_coding	Yes	NM_138477.4	108
protein_coding	Yes	NM_024818.6	108
protein_coding	Yes	NM_002883.4	108
protein_coding	Yes	-	108
protein_coding	Yes	NM_004910.3	108
protein_coding	Yes	NM_003803.4	108
protein_coding	Yes	NM_199161.5	108
protein_coding	Yes	NM_003522.4	108
protein_coding	Yes	NM_001409.4	108
protein_coding	Yes	NM_030817.3	108
protein_coding	Yes	NM_001709.5	108
protein_coding	Yes	NM_001001973.3	108
protein_coding	Yes	NM_006995.5	108
protein_coding	Yes	NM_000018.4	108
protein_coding	Yes	NM_001003800.2	108
protein_coding	Yes	NM_001312653.2	108
protein_coding	Yes	NM_000876.4	108
protein_coding	Yes	NM_006888.6	108
protein_coding	Yes	NM_001125.4	108
protein_coding	Yes	NM_025145.7	108
protein_coding	Yes	NM_016335.6	108
protein_coding	Yes	NM_138576.4	108
protein_coding	Yes	NM_005066.3	108
protein_coding	Yes	NM_017752.3	108
protein_coding	Yes	NM_001002800.3	108
protein_coding	Yes	NM_004457.5	108

ENST00000357447	128.1616028	-1.092816679	0.3411	-3.204	0.0013565	0.0087663	ENSG00000151474	10	13643705	14330924	FRMD4A
ENST00000357484	262.2086641	-1.845909345	0.26111	-7.069	1.56E-12	1.02E-10	ENSG00000242498	15	89895005	89912952	ARPIN
ENST00000357560	153.7506455	1.274510937	0.30923	4.1215	3.76E-05	0.0004252	ENSG00000113108	5	140558267	140564598	APBB3
ENST00000357578	228.4989091	-1.397422805	0.27205	-5.137	2.80E-07	5.83E-06	ENSG00000112294	6	24494968	24537207	ALDH5A1
ENST00000357640	5.339495796	5.845156443	1.96201	2.9792	0.0028903	0.0162004	ENSG00000162777	1	111185968	111200646	DENND2D
ENST00000357681	424.6114214	-2.027067777	0.27431	-7.39	1.47E-13	1.15E-11	ENSG00000058668	1	203626831	203744081	ATP2B4
ENST00000357685	22.47224387	2.290776322	0.75144	3.0485	0.0022998	0.0134737	ENSG00000197580	11	112175511	112218946	BCO2
ENST00000357700	137.7917094	-2.382938877	0.33998	-7.009	2.40E-12	1.52E-10	ENSG00000196372	10	5638866	5666595	ASB13
ENST00000357736	960.5129535	1.156702497	0.19138	6.0441	1.50E-09	5.24E-08	ENSG00000197063	17	81918269	81927735	MAFG
ENST00000357742	215.5104164	-2.169498852	0.30231	-7.176	7.16E-13	4.97E-11	ENSG00000140563	15	94231560	94483952	MCTP2
ENST00000357814	584.415381	1.113810062	0.21036	5.2948	1.19E-07	2.69E-06	ENSG00000109065	17	74770528	74776345	NAT9
ENST00000357947	62.11190874	1.308385795	0.44904	2.9137	0.0035716	0.0192414	ENSG00000095587	10	96364607	96513926	TLL2
ENST00000357998	325.5407133	-2.696128293	0.26046	-10.35	4.12E-25	1.36E-22	ENSG00000106692	9	105558130	105641118	FKTN
ENST00000358056	1123.514958	-2.691843431	0.19422	-13.86	1.11E-43	2.86E-40	ENSG00000197045	14	54474484	54488980	GMFB
ENST00000358227	98.56608443	-2.066794544	0.38072	-5.429	5.68E-08	1.39E-06	ENSG00000137038	9	7796499	7799778	DMAC1
ENST00000358230	753.0009483	-1.286376405	0.19893	-6.466	1.00E-10	4.42E-09	ENSG00000155957	12	66135845	66169996	TMBIM4
ENST00000358290	1065.585662	-1.23282049	0.21598	-5.708	1.14E-08	3.30E-07	ENSG00000198189	4	87336514	87391188	HSD17B11
ENST00000358395	579.7987741	1.302782384	0.22663	5.7485	9.00E-09	2.66E-07	ENSG00000101236	20	3927310	4015558	RNF24
ENST00000358399	27.86142436	-4.187471669	0.84572	-4.951	7.37E-07	1.36E-05	ENSG00000119125	9	72149439	72252224	GDA
ENST00000358464	127.1438779	-1.161965835	0.32582	-3.566	0.0003621	0.0029195	ENSG00000282826	20	28580708	28602664	FRG1CP
ENST00000358491	212.7375981	-1.053242863	0.31065	-3.39	0.0006979	0.0050735	ENSG00000197013	19	21505601	21540668	ZNF429
ENST00000358495	83.88279738	-1.556394194	0.39764	-3.914	9.08E-05	0.0009055	ENSG00000002016	12	911735	949694	RAD52
ENST00000358502	425.7183922	1.192386185	0.22354	5.3341	9.60E-08	2.22E-06	ENSG00000128335	22	36226208	36239527	APOL2
ENST00000358572	318.0244021	-1.54909563	0.27422	-5.649	1.61E-08	4.49E-07	ENSG00000198498	4	163494689	163520539	TMA16
ENST00000358582	39.81180759	1.956472407	0.59374	3.2952	0.0009836	0.0067218	ENSG00000290988	19	37667750	37692315	ZNF781
ENST00000358607	87.46489312	-1.321928474	0.41533	-3.183	0.0014585	0.0093109	ENSG00000006015	19	18588797	18592337	REX1BD
ENST00000358731	1763.028058	-1.101901267	0.18211	-6.051	1.44E-09	5.05E-08	ENSG00000145734	5	71455650	71567820	BDP1
ENST00000358739	2062.852207	-1.65521792	0.17713	-9.345	9.23E-21	1.80E-18	ENSG00000196747	6	27808172	27808667	H2AC13
ENST00000358755	3423.173153	-1.066994959	0.17198	-6.204	5.50E-10	2.07E-08	ENSG00000164930	8	103298897	103332866	FZD6
ENST00000358763	20.33719635	4.68043495	1.0861	4.3094	1.64E-05	0.0002059	ENSG00000185666	22	32507819	33058381	SYN3
ENST00000358776	615.1859903	1.084702404	0.23485	4.6187	3.86E-06	5.85E-05	ENSG00000196177	10	123009005	123058290	ACADSB
ENST00000358789	68.55414105	1.860769218	0.50304	3.699	0.0002164	0.0018967	ENSG00000129451	19	51012738	51019709	KLK10
ENST00000358812	230.9017968	-2.101250472	0.31164	-6.743	1.56E-11	8.21E-10	ENSG00000172469	6	95577534	95609452	MANEA
ENST00000358821	5.151772375	5.791933645	1.97706	2.9296	0.0033943	0.0184954	ENSG00000150656	18	74534499	74587212	CNDP1
ENST00000358869	329.6180892	-1.01258579	0.2605	-3.887	0.0001014	0.000994	ENSG00000197712	4	38867805	38945739	FAM114A1
ENST00000358913	7.343373197	6.303637464	1.83393	3.4372	0.0005877	0.0043945	ENSG00000138347	10	68109487	68212017	MYPN
ENST00000359003	377.7010033	1.121185366	0.31859	3.5192	0.0004328	0.0033987	ENSG00000198646	20	34714773	34825651	NCOA6
ENST00000359039	388.3501854	-1.088931262	0.22844	-4.767	1.87E-06	3.11E-05	ENSG00000198642	9	21329664	21335404	KLHL9
ENST00000359128	32.00900727	8.427869882	1.5468	5.4486	5.08E-08	1.26E-06	ENSG00000167984	16	3539032	3577403	NLRC3
ENST00000359154	757.9024346	1.022368017	0.20524	4.9813	6.31E-07	1.19E-05	ENSG00000198373	16	69762331	69941739	WWP2

protein_coding	Yes	NM_018027.5	108
protein_coding	Yes	NM_182616.4	108
protein_coding	Yes	NM_133173.3	108
protein_coding	Yes	NM_001080.3	108
protein_coding	Yes	NM_024901.5	108
protein_coding	Yes	NM_001684.5	108
protein_coding	Yes	NM_031938.7	108
protein_coding	Yes	NM_024701.4	108
protein_coding	Yes	NM_002359.4	108
protein_coding	Yes	NM_001385001.1	108
protein_coding	Yes	NM_015654.5	108
protein_coding	Yes	NM_012465.4	108
protein_coding	Yes	NM_001079802.2	108
protein_coding	Yes	NM_004124.3	108
protein_coding	Yes	NM_033428.3	108
protein_coding	Yes	NM_016056.4	108
protein_coding	Yes	NM_016245.5	108
protein_coding	Yes	NM_001134337.3	108
protein_coding	Yes	NM_004293.5	108
ed_unprocessed_ps	Yes	-	108
protein_coding	Yes	NM_001001415.4	108
protein_coding	Yes	NM_134424.4	108
protein_coding	Yes	NM_030882.4	108
protein_coding	Yes	NM_018352.3	108
lncRNA	Yes	-	108
protein_coding	Yes	NM_001100418.2	108
protein_coding	Yes	NM_018429.3	108
protein_coding	Yes	NM_003509.3	108
protein_coding	Yes	NM_003506.4	108
protein_coding	Yes	NM_003490.4	108
protein_coding	Yes	NM_001609.4	108
protein_coding	Yes	NM_145888.3	108
protein_coding	Yes	NM_024641.4	108
protein_coding	Yes	NM_032649.6	108
protein_coding	Yes	NM_138389.4	108
protein_coding	Yes	NM_032578.4	108
protein_coding	Yes	NM_014071.5	108
protein_coding	Yes	NM_018847.4	108
protein_coding	Yes	NM_178844.4	108
protein_coding	Yes	NM_001270454.2	108

ENST00000359187	300.1764826	1.887409156	0.26662	7.079	1.45E-12	9.57E-11	ENSG00000175265	15	34379067	34437808	GOLGA8A
ENST00000359204	109.5760362	-1.33711369	0.35262	-3.792	0.0001495	0.0013833	ENSG00000148690	10	93667882	93702592	FRA10AC1
ENST00000359246	541.1958791	-1.020450801	0.21717	-4.699	2.62E-06	4.18E-05	ENSG00000197724	9	93576583	93679587	PHF2
ENST00000359303	1122.205492	-1.151903558	0.18921	-6.088	1.14E-09	4.07E-08	ENSG00000197153	6	27890314	27890826	H3C12
ENST00000359314	2092.156048	-1.529570235	0.17992	-8.501	1.87E-17	2.45E-15	ENSG00000198087	6	47477788	47627263	CD2AP
ENST00000359365	126.8890154	1.509767868	0.3392	4.4509	8.55E-06	0.0001175	ENSG00000068028	3	50329787	50340836	RASSF1
ENST00000359396	28.58894035	-3.417755204	0.73985	-4.62	3.85E-06	5.83E-05	ENSG00000165125	7	142871207	142885745	TRPV6
ENST00000359429	1125.092275	-1.613955275	0.18906	-8.537	1.38E-17	1.84E-15	ENSG00000197713	2	210002637	210022260	RPE
ENST00000359441	13.49193904	7.178659797	1.70101	4.2202	2.44E-05	0.0002915	ENSG00000158815	8	22042819	22048809	FGF17
ENST00000359543	300.9956581	-1.559318976	0.28331	-5.504	3.71E-08	9.46E-07	ENSG00000213853	16	10528421	10580598	EMP2
ENST00000359623	211.9565808	2.16547568	0.33523	6.4597	1.05E-10	4.60E-09	ENSG00000127399	7	150323354	150338156	LRRC61
ENST00000359858	651.9868289	-1.949636302	0.21958	-8.879	6.74E-19	1.04E-16	ENSG00000197056	1	35079384	35115859	ZMYM1
ENST00000359988	8.418923948	6.503335082	1.80303	3.6069	0.0003099	0.0025624	ENSG00000197893	10	113588713	113664041	NRAP
ENST00000360004	70.43288081	-2.20602892	0.49345	-4.471	7.80E-06	0.0001084	ENSG00000196126	6	32578774	32589848	HLA-DRB1
ENST00000360032	11.96969275	-3.926488398	1.22376	-3.209	0.0013341	0.0086447	ENSG00000197587	1	46489835	46516216	DMBX1
ENST00000360215	4.451668466	5.580042745	2.05553	2.7147	0.0066346	0.0316755	ENSG00000197753	6	35805351	35824070	LHFPL5
ENST00000360273	6.922281806	6.217012516	1.86297	3.3371	0.0008464	0.0059445	ENSG00000157087	3	10324022	10505586	ATP2B2
ENST00000360403	291.409072	-1.43001738	0.26151	-5.468	4.54E-08	1.13E-06	ENSG00000070785	1	44850521	44986595	EIF2B3
ENST00000360482	33.78372451	2.180174641	0.63941	3.4097	0.0006504	0.0047796	ENSG00000134668	1	31790421	31816022	SPOCD1
ENST00000360490	142.4488804	-2.170671915	0.43786	-4.957	7.14E-07	1.32E-05	ENSG00000196549	3	155080318	155183704	MME
ENST00000360509	953.3880398	-1.583009939	0.20231	-7.825	5.10E-15	4.88E-13	ENSG00000166415	15	53513740	53759663	WDR72
ENST00000360534	47.40946202	-4.538993803	0.6861	-6.616	3.70E-11	1.80E-09	ENSG00000197635	2	161992244	162074215	DPP4
ENST00000360564	45.16280958	1.788505501	0.52195	3.4266	0.0006112	0.004535	ENSG00000198598	12	131828392	131851771	MMP17
ENST00000360570	246.9721519	-1.5730424	0.27272	-5.768	8.02E-09	2.40E-07	ENSG00000158234	3	138608770	138633376	FAIM
ENST00000360589	9.045415806	6.604569004	1.76737	3.737	0.0001863	0.0016679	ENSG00000144031	2	70978629	70985499	ANKRD53
ENST00000360668	278.7341538	1.886556748	0.27703	6.8099	9.77E-12	5.39E-10	ENSG00000101460	20	34558717	34560345	MAP1LC3A
ENST00000360779	202.7201515	3.318838527	0.31499	10.536	5.88E-26	2.18E-23	ENSG00000125775	20	1309908	1329139	SDCBP2
ENST00000360803	1260.607941	-1.345499763	0.23193	-5.801	6.58E-09	2.00E-07	ENSG00000107937	10	988433	1019932	GTPBP4
ENST00000360909	215.5100786	-1.19783292	0.27093	-4.421	9.81E-06	0.0001322	ENSG00000100592	14	59188666	59371403	DAAM1
ENST00000360986	10.78286361	-4.419158299	1.38926	-3.181	0.001468	0.0093599	ENSG00000109625	4	8592764	8619752	CPZ
ENST00000361066	169.1183558	1.999464603	0.39135	5.1092	3.24E-07	6.64E-06	ENSG00000134202	1	109733936	109741038	GSTM3
ENST00000361131	187.4481047	-2.323487082	0.32609	-7.125	1.04E-12	7.05E-11	ENSG00000198729	6	150143043	150250392	PPP1R14C
ENST00000361138	1667.057464	-1.094241313	0.19479	-5.617	1.94E-08	5.29E-07	ENSG00000198887	9	70258977	70354873	SMC5
ENST00000361170	499.0764174	-1.598758932	0.2255	-7.09	1.34E-12	8.93E-11	ENSG00000183856	1	156525404	156572565	IQGAP3
ENST00000361219	4315.372895	-1.146457826	0.17801	-6.44	1.19E-10	5.17E-09	ENSG00000142864	1	67407809	67430402	SERBP1
ENST00000361226	469.383911	1.209216535	0.23736	5.0945	3.50E-07	7.10E-06	ENSG00000198853	9	35490110	35561895	RUSC2
ENST00000361271	173.4965445	-1.977103242	0.29939	-6.604	4.01E-11	1.93E-09	ENSG00000165996	10	17589031	17617374	HACD1
ENST00000361272	1100.622067	-1.03338893	0.18823	-5.49	4.02E-08	1.02E-06	ENSG00000198791	8	17224965	17246857	CNOT7
ENST00000361418	451.4037268	-1.821728389	0.23146	-7.871	3.53E-15	3.47E-13	ENSG00000139116	12	39293227	39443120	KIF21A
ENST00000361428	452.2839792	1.005026261	0.2317	4.3376	1.44E-05	0.0001846	ENSG00000171467	6	43336069	43369647	ZNF318

protein_coding	Yes	NM_181077.5	108
protein_coding	Yes	NM_145246.5	108
protein_coding	Yes	NM_005392.4	108
protein_coding	Yes	NM_003535.3	108
protein_coding	Yes	NM_012120.3	108
protein_coding	Yes	NM_007182.5	108
protein_coding	Yes	NM_018646.6	108
protein_coding	Yes	NM_199229.3	108
protein_coding	Yes	NM_003867.4	108
protein_coding	Yes	NM_001424.6	108
protein_coding	Yes	NM_001142928.2	108
protein_coding	Yes	NM_024772.5	108
protein_coding	Yes	NM_198060.4	108
protein_coding	Yes	NM_002124.4	108
protein_coding	Yes	NM_172225.2	108
protein_coding	Yes	NM_182548.4	108
protein_coding	Yes	NM_001001331.4	108
protein_coding	Yes	NM_020365.5	108
protein_coding	Yes	NM_144569.7	108
protein_coding	Yes	NM_007289.4	108
protein_coding	Yes	NM_182758.4	108
protein_coding	Yes	NM_001935.4	108
protein_coding	Yes	NM_016155.7	108
protein_coding	Yes	NM_001033031.2	108
protein_coding	Yes	NM_001115116.2	108
protein_coding	Yes	NM_032514.4	108
protein_coding	Yes	NM_080489.5	108
protein_coding	Yes	NM_012341.3	108
protein_coding	Yes	NM_001270520.2	108
protein_coding	Yes	NM_001014447.3	108
protein_coding	Yes	NM_000849.5	108
protein_coding	Yes	NM_030949.3	108
protein_coding	Yes	NM_015110.4	108
protein_coding	Yes	NM_178229.5	108
protein_coding	Yes	NM_001018069.2	108
protein_coding	Yes	NM_014806.5	108
protein_coding	Yes	NM_014241.4	108
protein_coding	Yes	NM_013354.7	108
protein_coding	Yes	NM_001173464.2	108
protein_coding	Yes	NM_014345.3	108

ENST00000361475	3339.326422	-1.498576196	0.27075	-5.535	3.11E-08	8.08E-07	ENSG00000198959	20	38127384	38165270	TGM2
ENST00000361573	256.7897233	1.06629913	0.28519	3.7389	0.0001848	0.0016574	ENSG00000197818	20	49812827	49892242	SLC9A8
ENST00000361618	861.6545695	-1.276112864	0.20842	-6.123	9.19E-10	3.32E-08	ENSG00000198948	4	169986601	170026395	MFAP3L
ENST00000361627	1658.023265	-1.675264659	0.20324	-8.243	1.68E-16	1.94E-14	ENSG00000198826	15	32615503	32639941	ARHGAP11A
ENST00000361729	122.7689508	-2.133327072	0.35469	-6.015	1.80E-09	6.19E-08	ENSG00000007968	1	23506437	23531233	E2F2
ENST00000361757	745.2889532	1.015122656	0.19965	5.0845	3.69E-07	7.43E-06	ENSG00000064932	19	1107637	1174268	SBNO2
ENST00000361794	79.36607608	-1.488979857	0.40557	-3.671	0.0002413	0.00208	ENSG00000198945	6	130018580	130141438	L3MBTL3
ENST00000361840	164.4325321	-1.325880961	0.29837	-4.444	8.84E-06	0.0001209	ENSG00000123178	13	49912701	49936340	SPRYD7
ENST00000361842	24.39160232	-8.082161719	1.58295	-5.106	3.29E-07	6.74E-06	ENSG00000132530	17	6756045	6775647	XAF1
ENST00000361871	137.3265695	1.473892725	0.32023	4.6025	4.17E-06	6.28E-05	ENSG00000198736	16	1938228	1943199	MSRB1
ENST00000362007	22.86219761	-2.920963927	0.83043	-3.517	0.0004358	0.0034169	ENSG00000198715	1	156292687	156295679	GLMP
ENST00000362012	16.0817177	2.441674447	0.90826	2.6883	0.0071818	0.033683	ENSG00000095303	9	122371023	122395703	PTGS1
ENST00000362077	169.3299673	1.219169079	0.35705	3.4146	0.0006389	0.0047086	ENSG00000272657	21	34073591	34360033	-
ENST00000362477	37.16468968	-1.662880097	0.58521	-2.842	0.0044901	0.0231606	ENSG00000199347	1	11908151	11908271	RNU5E-1
ENST00000362512	47.56975692	-1.906029342	0.50745	-3.756	0.0001726	0.0015644	ENSG00000276027	22	42615243	42615393	RNU12
ENST00000362698	42.73680767	-2.175423694	0.55453	-3.923	8.74E-05	0.0008792	ENSG00000199568	15	65296050	65296166	RNU5A-1
ENST00000363009	12.16065649	-3.933002761	1.22415	-3.213	0.0013142	0.0085407	ENSG00000199879	1	148263475	148263632	RNVU1-22
ENST00000363373	15.68665162	2.386693209	0.87929	2.7143	0.0066407	0.031694	ENSG00000200243	5	6848126	6848462	RN7SKP79
ENST00000363442	336.0232149	1.715579554	0.24499	7.0026	2.51E-12	1.58E-10	ENSG00000200312	14	89712510	89712823	RN7SKP255
ENST00000363593	27.88101674	-1.98114919	0.64816	-3.057	0.0022387	0.0131942	ENSG00000200463	17	8173453	8173587	SNORD118
ENST00000363673	81.82260126	1.103485408	0.38835	2.8415	0.0044903	0.0231611	ENSG00000260682	16	81961925	81962243	RN7SKP176
ENST00000364102	4.0435793	-5.490211719	2.09993	-2.614	0.0089364	0.0401122	ENSG00000200972	1	210374153	210374267	RNU5A-8P
ENST00000364127	12.09186319	-5.605113907	1.67236	-3.352	0.0008034	0.0056999	ENSG00000200997	17	58679526	58679690	RNVU1-34
ENST00000364313	7.827928568	-6.443340524	1.8128	-3.554	0.0003789	0.0030355	ENSG00000201183	1	148402714	148402875	RNVU1-3
ENST00000364488	46.41674115	8.964063801	1.51808	5.9049	3.53E-09	1.14E-07	ENSG00000201358	14	50068567	50068873	RN7SKP193
ENST00000364688	17.80554346	-2.49496305	0.83052	-3.004	0.0026638	0.0151792	ENSG00000201558	1	146052080	146052244	RNVU1-6
ENST00000364923	25.76205448	2.422429168	0.69761	3.4725	0.0005157	0.0039391	ENSG00000201793	13	99205707	99206006	RN7SKP9
ENST00000365031	33.0606262	1.880326014	0.60008	3.1335	0.0017276	0.0106757	ENSG00000201901	4	85100495	85100823	RN7SKP48
ENST00000365188	702.55337	1.174178884	0.22336	5.2569	1.46E-07	3.24E-06	ENSG00000202058	22	42565047	42565330	RN7SKP80
ENST00000365465	8.317650022	-3.381148219	1.3198	-2.562	0.0104112	0.0452851	ENSG00000202335	12	110496351	110496421	-
ENST00000366376	4.337557096	5.546371557	2.07077	2.6784	0.0073974	0.034454	ENSG00000203565	10	33684754	33709868	-
ENST00000366451	70.72952209	-1.462214983	0.4196	-3.485	0.0004926	0.003789	ENSG00000203644	3	129847047	129847957	-
ENST00000366513	1006.886933	1.007178041	0.19629	5.1311	2.88E-07	5.98E-06	ENSG00000162852	1	246566455	246668595	CNST
ENST00000366534	63.71819238	3.389728666	0.50821	6.67	2.56E-11	1.29E-09	ENSG00000179397	1	244461272	244640504	CATSPERE
ENST00000366559	18.21912519	2.62407541	0.85021	3.0864	0.0020261	0.0121489	ENSG00000117009	1	241532377	241595642	KMO
ENST00000366577	442.3996759	-1.068721557	0.22582	-4.733	2.22E-06	3.61E-05	ENSG00000116984	1	236795291	236903981	MTR
ENST00000366587	197.380192	-1.013255629	0.28518	-3.553	0.0003809	0.0030498	ENSG00000244457	1	236483164	236484468	ENO1P1
ENST00000366592	53.01761726	-1.315575459	0.48426	-2.717	0.0065937	0.0315295	ENSG00000077585	1	236142538	236208907	GPR137B
ENST00000366691	760.3937476	-1.539811648	0.21741	-7.082	1.42E-12	9.37E-11	ENSG00000116574	1	228735478	228746664	RHOU
ENST00000366843	4487.792348	-1.333435158	0.18522	-7.199	6.06E-13	4.26E-11	ENSG00000154380	1	225486828	225653123	ENAH

protein_coding	Yes	NM_004613.4	108
protein_coding	Yes	NM_015266.3	108
protein_coding	Yes	NM_021647.8	108
protein_coding	Yes	NM_014783.6	108
protein_coding	Yes	NM_004091.4	108
protein_coding	Yes	NM_014963.3	108
protein_coding	Yes	NM_032438.4	108
protein_coding	Yes	NM_020456.4	108
protein_coding	Yes	NM_017523.5	108
protein_coding	Yes	NM_016332.4	108
protein_coding	Yes	NM_144580.3	108
protein_coding	Yes	NM_000962.4	108
lncRNA	Yes	-	108
snRNA	Yes	-	108
snRNA	Yes	-	108
snRNA	Yes	-	108
snRNA	Yes	-	108
misc_RNA	Yes	-	108
misc_RNA	Yes	-	108
snoRNA	Yes	-	108
misc_RNA	Yes	-	108
snRNA	Yes	-	108
snRNA	Yes	-	108
snRNA	Yes	-	108
misc_RNA	Yes	-	108
snRNA	Yes	-	108
misc_RNA	Yes	-	108
misc_RNA	Yes	-	108
misc_RNA	Yes	-	108
snoRNA	Yes	-	108
lncRNA	Yes	-	108
lncRNA	Yes	-	108
protein_coding	Yes	NM_152609.3	108
protein_coding	Yes	NM_001130957.2	108
protein_coding	Yes	NM_003679.5	108
protein_coding	Yes	NM_000254.3	108
bed_processed_pse	Yes	-	108
protein_coding	Yes	NM_003272.4	108
protein_coding	Yes	NM_021205.6	108
protein_coding	Yes	NM_018212.6	108

ENST00000366899	133.860139	1.150594769	0.33217	3.4639	0.0005324	0.0040452	ENSG00000143507	1	221701423	221742089	DUSP10
ENST00000366910	81.69315757	-5.359477681	0.65512	-8.181	2.82E-16	3.16E-14	ENSG00000186205	1	220786912	220819659	MTARC1
ENST00000366932	418.5404903	-1.427654954	0.27215	-5.246	1.56E-07	3.42E-06	ENSG00000067533	1	218285292	218337983	RRP15
ENST00000366955	5309.044599	-1.60542038	0.1696	-9.466	2.91E-21	6.07E-19	ENSG00000117724	1	214603194	214664571	CENPF
ENST00000366958	10.2579002	6.786188357	1.73262	3.9167	8.98E-05	0.0008979	ENSG00000117707	1	213987972	214041510	PROX1
ENST00000366974	117.5718725	-1.156194647	0.33611	-3.44	0.0005818	0.0043595	ENSG00000203705	1	212791893	212816830	TATDN3
ENST00000366977	352.5590966	-1.780936165	0.24401	-7.299	2.91E-13	2.18E-11	ENSG00000117697	1	212726152	212791777	NSL1
ENST00000366999	1154.723415	-1.093843283	0.19467	-5.619	1.92E-08	5.25E-07	ENSG00000117650	1	211662771	211675621	NEK2
ENST00000367001	837.8120446	-1.989775627	0.19796	-10.05	9.06E-24	2.63E-21	ENSG00000170385	1	211571567	211579161	SLC30A1
ENST00000367021	1118.026188	-1.017791649	0.19847	-5.128	2.92E-07	6.06E-06	ENSG00000117595	1	209785616	209806142	IRF6
ENST00000367033	204.2804931	-2.316346062	0.30079	-7.701	1.35E-14	1.22E-12	ENSG00000076356	1	208022241	208244384	PLXNA2
ENST00000367042	2801.325587	-1.162209302	0.1773	-6.555	5.56E-11	2.57E-09	ENSG00000117335	1	207752053	207795504	CD46
ENST00000367078	28.22990052	1.89547605	0.72809	2.6033	0.009232	0.0411466	ENSG00000123843	1	207088859	207099993	C4BPB
ENST00000367142	4810.474084	-2.62601347	0.16968	-15.48	5.01E-54	1.82E-50	ENSG00000069275	1	205712821	205750182	NUCKS1
ENST00000367162	234.4605474	-1.808945369	0.27175	-6.657	2.80E-11	1.40E-09	ENSG00000133059	1	205142504	205211702	DSTYK
ENST00000367194	20.14160998	-2.082685836	0.80077	-2.601	0.0092989	0.0413844	ENSG00000170498	1	204190340	204196491	KISS1
ENST00000367202	143.1255348	-1.305171815	0.3144	-4.151	3.31E-05	0.0003795	ENSG00000143845	1	204131061	204152044	ETNK2
ENST00000367255	18.50297761	-2.549141635	0.84765	-3.007	0.0026356	0.0150554	ENSG00000131018	6	152121686	152637362	SYNE1
ENST00000367339	45.49068016	-2.33129276	0.54262	-4.296	1.74E-05	0.0002168	ENSG00000131019	6	150061052	150069121	ULBP3
ENST00000367350	1811.311723	-1.337106219	0.17768	-7.525	5.25E-14	4.40E-12	ENSG00000118193	1	200551499	200620751	KIF14
ENST00000367409	3843.622313	-1.703268364	0.16998	-10.02	1.24E-23	3.54E-21	ENSG00000066279	1	197084126	197146669	ASPM
ENST00000367425	8.100334493	-5.013651376	1.77922	-2.818	0.0048339	0.0245922	ENSG00000116785	1	196774839	196795407	CFHR3
ENST00000367466	104.6699049	-2.253765548	0.38641	-5.833	5.46E-09	1.69E-07	ENSG00000116711	1	186828948	186988981	PLA2G4A
ENST00000367500	228.1608677	1.946517882	0.30961	6.287	3.24E-10	1.27E-08	ENSG00000116668	1	185157166	185291781	SWT1
ENST00000367510	202.5579207	-2.232903317	0.31396	-7.112	1.14E-12	7.70E-11	ENSG00000121481	1	185045557	185102603	RNF2
ENST00000367511	97.31182247	-2.726972501	0.41516	-6.568	5.08E-11	2.38E-09	ENSG00000135842	1	184791031	184974508	NIBAN1
ENST00000367519	105.1057076	2.146368226	0.38402	5.5892	2.28E-08	6.11E-07	ENSG00000112425	6	145625304	145735520	EPM2A
ENST00000367545	1785.711368	-1.08847713	0.17949	-6.064	1.33E-09	4.67E-08	ENSG00000152818	6	144285334	144853034	UTRN
ENST00000367590	1292.064316	-1.009113353	0.23891	-4.224	2.40E-05	0.0002873	ENSG00000143324	1	180632021	180890279	XPR1
ENST00000367603	1511.304346	1.687142964	0.18525	9.1076	8.43E-20	1.44E-17	ENSG00000010818	6	142751468	142945176	HIVEP2
ENST00000367635	906.0871629	-2.150140977	0.2284	-9.414	4.79E-21	9.66E-19	ENSG00000116191	1	178725243	178921840	RALGPS2
ENST00000367660	273.4667736	-1.993386888	0.25849	-7.712	1.24E-14	1.13E-12	ENSG00000146386	6	139028744	139043302	ABRACL
ENST00000367815	1778.468082	1.280753099	0.20444	6.2647	3.74E-10	1.45E-08	ENSG00000143153	1	169106689	169132719	ATP1B1
ENST00000367921	33.87670128	1.507292803	0.5828	2.5863	0.009702	0.0427933	ENSG00000162733	1	162632463	162787405	DDR2
ENST00000367942	669.5218313	-1.3882179	0.20811	-6.67	2.55E-11	1.29E-09	ENSG00000118217	1	161766319	161964070	ATF6
ENST00000367943	227.7053489	-2.99854889	0.31228	-9.602	7.83E-22	1.76E-19	ENSG00000081721	1	161749785	161757238	DUSP12
ENST00000368003	1269.473605	1.543397826	0.1952	7.9067	2.64E-15	2.64E-13	ENSG00000143222	1	161153977	161158856	UFC1
ENST00000368009	360.0001756	1.022354458	0.26369	3.8771	0.0001057	0.0010266	ENSG00000158793	1	161118104	161121194	NIT1
ENST00000368223	61.91599466	-3.894285926	0.54567	-7.137	9.56E-13	6.52E-11	ENSG00000132688	1	156668762	156677407	NES
ENST00000368232	198.9569146	-1.639662663	0.32245	-5.085	3.68E-07	7.41E-06	ENSG00000160818	1	156594300	156601479	GPATCH4

protein_coding	Yes	NM_007207.6	108
protein_coding	Yes	NM_022746.4	108
protein_coding	Yes	NM_016052.4	108
protein_coding	Yes	NM_016343.4	108
protein_coding	Yes	NM_001270616.2	108
protein_coding	Yes	NM_001042552.3	108
protein_coding	Yes	NM_015471.4	108
protein_coding	Yes	NM_002497.4	108
protein_coding	Yes	NM_021194.3	108
protein_coding	Yes	NM_006147.4	108
protein_coding	Yes	NM_025179.4	108
protein_coding	Yes	NM_172351.3	108
protein_coding	Yes	NM_001017365.3	108
protein_coding	Yes	NM_022731.5	108
protein_coding	Yes	NM_015375.3	108
protein_coding	Yes	NM_002256.4	108
protein_coding	Yes	NM_018208.4	108
protein_coding	Yes	NM_182961.4	108
protein_coding	Yes	NM_024518.3	108
protein_coding	Yes	NM_014875.3	108
protein_coding	Yes	NM_018136.5	108
protein_coding	Yes	NM_021023.6	108
protein_coding	Yes	NM_024420.3	108
protein_coding	Yes	NM_017673.7	108
protein_coding	Yes	NM_007212.4	108
protein_coding	Yes	NM_052966.4	108
protein_coding	Yes	NM_005670.4	108
protein_coding	Yes	NM_007124.3	108
protein_coding	Yes	NM_004736.4	108
protein_coding	Yes	NM_006734.4	108
protein_coding	Yes	NM_152663.5	108
protein_coding	Yes	NM_021243.3	108
protein_coding	Yes	NM_001677.4	108
protein_coding	Yes	NM_006182.4	108
protein_coding	Yes	NM_007348.4	108
protein_coding	Yes	NM_007240.3	108
protein_coding	Yes	NM_016406.4	108
protein_coding	Yes	NM_005600.3	108
protein_coding	Yes	NM_006617.2	108
protein_coding	Yes	-	108

ENST00000368321	728.6003297	1.169427618	0.20506	5.7029	1.18E-08	3.39E-07	ENSG00000132680	1	155913044	155934413	KHDC4
ENST00000368324	23.18223979	3.384186318	0.81479	4.1535	3.27E-05	0.0003763	ENSG00000132718	1	155859566	155885199	SYT11
ENST00000368373	829.6608855	1.950830524	0.21406	9.1133	7.99E-20	1.37E-17	ENSG00000177628	1	155234451	155241249	GBA
ENST00000368409	94.26152002	1.201959839	0.36688	3.2762	0.0010522	0.0071043	ENSG00000243364	1	155063739	155069553	EFNA4
ENST00000368468	215.3813677	3.210449039	0.30561	10.505	8.19E-26	2.92E-23	ENSG00000111885	6	119177204	119349761	MAN1A1
ENST00000368476	12.75133582	7.099514716	1.6818	4.2214	2.43E-05	0.0002901	ENSG00000160716	1	154567777	154580013	CHRNB2
ENST00000368491	114.9712959	-1.241062495	0.33887	-3.662	0.0002499	0.0021446	ENSG00000111860	6	118460771	118651591	CEP85L
ENST00000368564	288.7327972	-1.852049706	0.26043	-7.111	1.15E-12	7.73E-11	ENSG00000196911	6	116681210	116741867	KPNA5
ENST00000368633	132.4386973	1.079144985	0.37113	2.9077	0.0036409	0.0195444	ENSG00000160741	1	153947674	153958612	CRTC2
ENST00000368656	16.82712355	-3.66570766	1.02005	-3.594	0.0003261	0.0026763	ENSG00000203778	6	112087590	112102790	FAM229B
ENST00000368685	195.8229362	-2.267498723	0.2912	-7.787	6.88E-15	6.44E-13	ENSG00000143553	1	153658702	153661852	SNAPIN
ENST00000368713	98.96126735	5.787988265	0.68225	8.4837	2.18E-17	2.84E-15	ENSG00000188015	1	153547328	153549258	S100A3
ENST00000368716	656.3365333	2.670756739	0.21472	12.438	1.62E-35	2.26E-32	ENSG00000196154	1	153543620	153545806	S100A4
ENST00000368738	163.5806484	-1.040991093	0.32194	-3.233	0.0012229	0.0080504	ENSG00000163220	1	153357853	153361023	S100A9
ENST00000368799	15.63111659	3.874347386	1.11261	3.4822	0.0004973	0.0038199	ENSG00000143631	1	152302164	152325239	FLG
ENST00000368830	395.2251674	-1.651779336	0.24744	-6.676	2.46E-11	1.25E-09	ENSG00000143436	1	151759646	151763496	MRPL9
ENST00000368836	399.0867593	-3.308147626	0.2509	-13.19	1.07E-39	2.03E-36	ENSG00000203791	10	124757833	124791887	EEF1AKMT2
ENST00000368892	371.3189817	-1.014217683	0.24276	-4.178	2.94E-05	0.0003425	ENSG00000163159	1	151176303	151190197	VPS72
ENST00000368904	305.0573122	1.460041206	0.25849	5.6484	1.62E-08	4.51E-07	ENSG00000286088	10	122832166	122855773	-
ENST00000368908	122.1587253	1.899959606	0.34422	5.5197	3.40E-08	8.74E-07	ENSG00000163155	1	151159747	151165902	LYSMD1
ENST00000368914	34.08782798	2.345228631	0.64377	3.643	0.0002695	0.0022814	ENSG00000143434	1	151131706	151146631	SEMA6C
ENST00000368921	148.6129503	1.448199831	0.312	4.6416	3.46E-06	5.31E-05	ENSG00000213190	1	151060396	151069544	MLLT11
ENST00000368924	8.390956446	6.494851013	1.79611	3.6161	0.0002991	0.0024898	ENSG00000203797	6	110391783	110415575	DDO
ENST00000369005	196.8121671	1.039634557	0.29782	3.4908	0.0004816	0.0037196	ENSG00000138162	10	121989162	122254542	TACC2
ENST00000369085	525.2303633	1.302424014	0.21333	6.1053	1.03E-09	3.68E-08	ENSG00000151929	10	119651379	119677819	BAG3
ENST00000369155	2008.666137	-1.761995905	0.18755	-9.395	5.74E-21	1.15E-18	ENSG00000184678	1	149884458	149886682	H2BC21
ENST00000369159	5502.742149	-1.262959453	0.16766	-7.533	4.97E-14	4.17E-12	ENSG00000288825	1	149842217	149842750	H2AC18
ENST00000369167	3074.452943	-1.510077553	0.18089	-8.348	6.95E-17	8.39E-15	ENSG00000203814	1	149811878	149812370	H2BC18
ENST00000369258	499.8352267	-1.02003043	0.21664	-4.708	2.50E-06	4.01E-05	ENSG00000131778	1	147242683	147295762	CHD1L
ENST00000369295	11.10699366	-5.479952937	1.69153	-3.24	0.0011968	0.0079076	ENSG00000043591	10	114043865	114046904	ADRB1
ENST00000369306	104.3245456	-1.489981425	0.35841	-4.157	3.22E-05	0.0003712	ENSG00000131779	1	145911349	145918717	PEX11B
ENST00000369390	130.4051504	-1.016294742	0.32371	-3.14	0.0016921	0.0105057	ENSG00000188610	1	121167645	121184340	FAM72B
ENST00000369448	86.39471673	1.688045533	0.38677	4.3645	1.27E-05	0.0001662	ENSG00000183508	1	117606047	117628389	TENT5C
ENST00000369458	249.2774139	-2.495839751	0.85537	-2.918	0.0035247	0.0190421	ENSG00000134258	1	117143586	117210927	VTCN1
ENST00000369516	173.7903226	1.012690552	0.28994	3.4928	0.0004781	0.0036973	ENSG00000134198	1	115048010	115089503	TSPAN2
ENST00000369552	407.7976296	-1.548340099	0.23261	-6.656	2.81E-11	1.40E-09	ENSG00000164414	6	87472973	87512336	SLC35A1
ENST00000369604	2389.154684	1.053410953	0.18159	5.8009	6.59E-09	2.01E-07	ENSG00000116793	1	113696830	113759486	PHTF1
ENST00000369622	1395.135163	-1.041982598	0.22205	-4.692	2.70E-06	4.29E-05	ENSG00000135316	6	85613975	85642886	SYNCRIP
ENST00000369684	165.155044	3.974620984	0.37385	10.631	2.13E-26	8.40E-24	ENSG00000134245	1	112508964	112530165	WNT2B
ENST00000369700	10.27453965	-3.26753936	1.18226	-2.764	0.0057133	0.0280814	ENSG00000146250	6	83512533	83525704	PRSS35

protein_coding	Yes	NM_014949.4	108
protein_coding	Yes	NM_152280.5	108
protein_coding	Yes	NM_000157.4	108
protein_coding	Yes	NM_005227.3	108
protein_coding	Yes	NM_005907.4	108
protein_coding	Yes	NM_000748.3	108
protein_coding	Yes	NM_001042475.3	108
protein_coding	Yes	NM_001366306.2	108
protein_coding	Yes	NM_181715.3	108
protein_coding	Yes	NM_001033564.3	108
protein_coding	Yes	NM_012437.6	108
protein_coding	Yes	NM_002960.2	108
protein_coding	Yes	NM_002961.3	108
protein_coding	Yes	NM_002965.4	108
protein_coding	Yes	NM_002016.2	108
protein_coding	Yes	NM_031420.4	108
protein_coding	Yes	NM_212554.4	108
protein_coding	Yes	NM_005997.3	108
protein_coding	Yes	-	108
protein_coding	Yes	NM_212551.5	108
protein_coding	Yes	NM_030913.6	108
protein_coding	Yes	NM_006818.4	108
protein_coding	Yes	NM_001372108.2	108
protein_coding	Yes	NM_206862.4	108
protein_coding	Yes	NM_004281.4	108
protein_coding	Yes	NM_003528.3	108
protein_coding	Yes	NM_003516.3	108
protein_coding	Yes	NM_001024599.5	108
protein_coding	Yes	NM_004284.6	108
protein_coding	Yes	NM_000684.3	108
protein_coding	Yes	NM_003846.3	108
protein_coding	Yes	NM_001100910.2	108
protein_coding	Yes	NM_017709.4	108
protein_coding	Yes	NM_024626.4	108
protein_coding	Yes	NM_005725.6	108
protein_coding	Yes	NM_006416.5	108
protein_coding	Yes	NM_001323043.2	108
protein_coding	Yes	NM_006372.5	108
protein_coding	Yes	NM_024494.3	108
protein_coding	Yes	NM_153362.3	108

ENST00000369724	93.40174104	1.072514585	0.3683	2.9121	0.0035901	0.0193254	ENSG00000013392	6	83193356	83198935	RWDD2A
ENST00000369732	98.62044691	1.343632296	0.37124	3.6193	0.0002954	0.0024639	ENSG00000085465	1	111414318	111427735	OVGP1
ENST00000369800	156.5233416	-2.146381047	0.32226	-6.66	2.73E-11	1.37E-09	ENSG00000214357	5	172641262	172691540	NEURL1B
ENST00000369817	1851.841942	-1.251932048	0.17912	-6.989	2.77E-12	1.72E-10	ENSG00000147403	X	154398376	154402332	RPL10
ENST00000369836	61.85750788	1.766293747	0.47702	3.7028	0.0002132	0.0018735	ENSG00000168765	1	109656098	109661700	GSTM4
ENST00000369864	19.2511129	-2.928746227	0.86846	-3.372	0.0007454	0.0053611	ENSG00000181754	1	109504177	109509727	AMIGO1
ENST00000369878	374.1085986	1.533081341	0.26224	5.8462	5.03E-09	1.57E-07	ENSG00000148842	10	102918293	103090222	CNNM2
ENST00000369880	54.82939989	1.978118576	0.47775	4.1405	3.47E-05	0.0003954	ENSG00000214435	10	102869469	102901899	AS3MT
ENST00000369909	284.6805818	-2.081489978	0.25595	-8.133	4.20E-16	4.61E-14	ENSG00000134222	1	109279555	109283145	PSRC1
ENST00000369940	44.89959376	-2.296446357	0.54336	-4.226	2.38E-05	0.0002845	ENSG00000146243	6	78867550	78903102	IRAK1BP1
ENST00000369956	319.8230698	2.937393155	0.26324	11.159	6.50E-29	3.56E-26	ENSG00000107872	10	102420835	102423136	FBXL15
ENST00000370005	31.38368343	2.127162285	0.64002	3.3236	0.0008886	0.0061806	ENSG00000119915	10	102226298	102229589	ELOVL3
ENST00000370035	916.8327572	-2.764758594	0.20488	-13.49	1.69E-41	3.85E-38	ENSG00000162636	1	108560099	108639322	FAM102B
ENST00000370053	56.99400074	-2.592432045	0.49912	-5.194	2.06E-07	4.42E-06	ENSG00000197457	20	63639711	63653424	STMN3
ENST00000370060	246.1680929	1.841800519	0.35102	5.247	1.55E-07	3.41E-06	ENSG00000198910	X	153861515	153886173	L1CAM
ENST00000370078	253.8939986	-2.93890938	0.27893	-10.54	5.88E-26	2.18E-23	ENSG00000198890	1	107056673	107059294	PRMT6
ENST00000370192	401.8396126	-2.10101883	0.23363	-8.993	2.41E-19	3.96E-17	ENSG00000188641	1	97077742	97921034	DPYD
ENST00000370200	269.8128089	1.348188096	0.25468	5.2937	1.20E-07	2.70E-06	ENSG00000107821	10	101061988	101067059	KAZALD1
ENST00000370277	269.3967402	-1.004941444	0.26431	-3.802	0.0001434	0.001334	ENSG00000147400	X	152826993	152830757	CETN2
ENST00000370282	4194.434832	-1.658773737	0.17959	-9.236	2.55E-20	4.61E-18	ENSG00000117500	1	93149741	93180413	TMED5
ENST00000370298	2308.662245	-1.322891908	0.17411	-7.598	3.01E-14	2.59E-12	ENSG00000143033	1	93079282	93139076	MTF2
ENST00000370310	117.2313806	-2.428987391	0.36774	-6.605	3.97E-11	1.91E-09	ENSG00000154511	1	92842166	92961462	DIPK1A
ENST00000370314	134.7417227	-2.829348633	0.34885	-8.11	5.04E-16	5.49E-14	ENSG00000011677	X	152166233	152451315	GABRA3
ENST00000370355	6644.130497	-1.240792714	0.21617	-5.74	9.48E-09	2.78E-07	ENSG00000099194	10	100347232	100364826	SCD
ENST00000370435	632.0157438	-2.205719048	0.21573	-10.22	1.54E-24	4.81E-22	ENSG00000119900	6	71288810	71309059	OGFRL1
ENST00000370460	8.191259814	-6.506927732	1.83422	-3.548	0.0003889	0.0031044	ENSG00000155966	X	148500616	149000663	AFF2
ENST00000370501	7.82488878	-4.944953966	1.78395	-2.772	0.0055727	0.0275463	ENSG00000101188	20	62708835	62762771	NTSR1
ENST00000370509	1128.148367	-1.2022127	0.20857	-5.764	8.21E-09	2.45E-07	ENSG00000143162	1	167541012	167553762	CREG1
ENST00000370544	1828.168168	-1.244450998	0.17934	-6.939	3.95E-12	2.36E-10	ENSG00000143013	1	87328879	87348923	LMO4
ENST00000370550	3468.796969	-1.404874203	0.17884	-7.855	3.99E-15	3.90E-13	ENSG00000153936	1	86914634	87109982	HS2ST1
ENST00000370574	271.6864766	-1.127148217	0.25522	-4.416	1.00E-05	0.0001348	ENSG00000117174	1	85649416	85708433	ZNHIT6
ENST00000370589	153.282678	-2.778755851	0.33281	-8.349	6.86E-17	8.30E-15	ENSG00000055732	1	85018081	85048500	MCOLN3
ENST00000370602	76.2591442	2.972963109	0.45468	6.5386	6.21E-11	2.84E-09	ENSG00000155265	10	97849842	97871578	GOLGA7B
ENST00000370608	131.6092777	-1.009160352	0.33498	-3.013	0.0025902	0.0148447	ENSG00000153898	1	84925582	84997113	MCOLN2
ENST00000370626	210.4236276	1.954831502	0.28179	6.9372	4.00E-12	2.39E-10	ENSG00000119986	10	97677423	97687241	AVPI1
ENST00000370733	274.3599138	-1.483343413	0.25298	-5.864	4.53E-09	1.43E-07	ENSG00000168116	6	57046692	57055225	KIAA1586
ENST00000370747	303.6933882	-5.537700292	0.89947	-6.157	7.43E-10	2.73E-08	ENSG00000137965	1	78649830	78664073	IFI44
ENST00000370751	381.4085104	-5.26591552	1.16364	-4.525	6.03E-06	8.65E-05	ENSG00000137959	1	78620447	78646145	IFI44L
ENST00000370853	5.806138918	-6.012331624	1.9244	-3.124	0.0017825	0.0109512	ENSG00000076770	X	132369319	132489038	MBNL3
ENST00000370859	603.2844235	-1.896198959	0.28792	-6.586	4.52E-11	2.15E-09	ENSG00000137968	1	75202128	75611114	SLC44A5

protein_coding	Yes	NM_033411.5	108
protein_coding	Yes	NM_002557.4	108
protein_coding	Yes	NM_001142651.3	108
protein_coding	Yes	NM_006013.5	108
protein_coding	Yes	NM_000850.5	108
protein_coding	Yes	NM_020703.4	108
protein_coding	Yes	NM_017649.5	108
protein_coding	Yes	NM_020682.4	108
protein_coding	Yes	NM_001032291.3	108
protein_coding	Yes	NM_001010844.4	108
protein_coding	Yes	NM_024326.4	108
protein_coding	Yes	NM_152310.3	108
protein_coding	Yes	NM_001010883.3	108
protein_coding	Yes	NM_015894.4	108
protein_coding	Yes	NM_001278116.2	108
protein_coding	Yes	NM_018137.3	108
protein_coding	Yes	NM_000110.4	108
protein_coding	Yes	NM_030929.5	108
protein_coding	Yes	NM_004344.3	108
protein_coding	Yes	NM_016040.5	108
protein_coding	Yes	NM_007358.4	108
protein_coding	Yes	NM_001006605.5	108
protein_coding	Yes	NM_000808.4	108
protein_coding	Yes	NM_005063.5	108
protein_coding	Yes	NM_024576.5	108
protein_coding	Yes	NM_002025.4	108
protein_coding	Yes	NM_002531.3	108
protein_coding	Yes	NM_003851.3	108
protein_coding	Yes	NM_006769.4	108
protein_coding	Yes	NM_012262.4	108
protein_coding	Yes	NM_017953.4	108
protein_coding	Yes	NM_018298.11	108
protein_coding	Yes	NM_001010917.3	108
protein_coding	Yes	NM_153259.4	108
protein_coding	Yes	NM_021732.3	108
protein_coding	Yes	NM_020931.4	108
protein_coding	Yes	NM_006417.5	108
protein_coding	Yes	NM_006820.4	108
protein_coding	Yes	NM_001386889.1	108
protein_coding	Yes	NM_001130058.2	108

ENST00000370867	356.7058732	-1.546185401	0.23678	-6.53	6.58E-11	2.99E-09	ENSG00000162623	1	74733151	74766677	TYW3
ENST00000370938	161.0944153	2.171850349	0.31383	6.9205	4.50E-12	2.66E-10	ENSG00000116761	1	70411267	70439851	CTH
ENST00000371019	220.4827596	1.061072113	0.26884	3.9469	7.92E-05	0.0008066	ENSG00000181274	10	97332496	97334729	FRAT2
ENST00000371084	363.4108276	-2.3887997	0.24532	-9.738	2.08E-22	5.15E-20	ENSG00000079739	1	63593410	63660245	PGM1
ENST00000371117	14.51671229	-3.757703198	1.12535	-3.339	0.0008403	0.0059116	ENSG00000170927	6	51615298	52087613	PKHD1
ENST00000371153	153.8435136	-3.918819166	0.37029	-10.58	3.57E-26	1.34E-23	ENSG00000132854	1	62236164	62319434	KANK4
ENST00000371204	16.6669152	-6.074569605	1.61166	-3.769	0.0001638	0.001496	ENSG00000134716	1	59893307	59926773	CYP2J2
ENST00000371208	495.8453848	-1.707533343	0.27933	-6.113	9.77E-10	3.52E-08	ENSG00000134709	1	59814948	59876322	HOOK1
ENST00000371247	1031.195266	2.556018868	0.20497	12.47	1.09E-35	1.57E-32	ENSG00000095637	10	95311772	95561371	SORBS1
ENST00000371253	43.95218499	1.430520791	0.51437	2.7811	0.005417	0.0269539	ENSG00000153292	6	46997707	47042332	ADGRF1
ENST00000371269	993.5347257	-3.877018044	0.22896	-16.93	2.57E-64	1.40E-60	ENSG00000116133	1	54849626	54887195	DHCR24
ENST00000371270	9.888532936	4.200290524	1.41374	2.971	0.0029679	0.0165451	ENSG00000138115	10	95036771	95069497	CYP2C8
ENST00000371281	254.1268778	-1.288200454	0.26894	-4.79	1.67E-06	2.81E-05	ENSG00000243725	1	54715860	54742657	TTC4
ENST00000371380	46.80518868	2.424260885	0.53548	4.5273	5.98E-06	8.59E-05	ENSG00000138193	10	93993930	94332823	PLCE1
ENST00000371410	120.6554814	-1.823098135	0.34183	-5.333	9.64E-08	2.23E-06	ENSG00000101882	X	119920671	119943751	NKAP
ENST00000371429	1685.863487	-1.975135448	0.18434	-10.71	8.69E-27	3.60E-24	ENSG00000058804	1	53765477	53838296	NDC1
ENST00000371505	3.916465705	5.396742222	2.11677	2.5495	0.0107872	0.0464746	ENSG00000146221	6	44278733	44297698	TCTE1
ENST00000371634	16.36409649	2.629672554	0.8822	2.9808	0.002875	0.0161364	ENSG00000176919	9	136945242	136946975	C8G
ENST00000371655	1249.619885	2.202228071	0.27248	8.0822	6.36E-16	6.87E-14	ENSG00000169213	1	51907955	51990700	RAB3B
ENST00000371692	37.01388835	-2.889062442	0.60994	-4.737	2.17E-06	3.54E-05	ENSG00000165716	9	136712571	136724742	DIPK1B
ENST00000371696	242.3317269	-1.465673855	0.26838	-5.461	4.73E-08	1.18E-06	ENSG00000169692	9	136673142	136687457	AGPAT2
ENST00000371728	2336.712627	-1.393599036	0.17402	-8.008	1.16E-15	1.21E-13	ENSG00000138182	10	89701589	89774934	KIF20B
ENST00000371755	61.83654866	-3.128631824	0.49571	-6.311	2.76E-10	1.10E-08	ENSG00000112759	6	44223594	44234142	SLC29A1
ENST00000371761	75.74038514	-1.353732026	0.40762	-3.321	0.0008968	0.0062249	ENSG00000123080	1	50970244	50974634	CDKN2C
ENST00000371818	308.5653396	-3.187812949	0.37305	-8.545	1.28E-17	1.72E-15	ENSG00000119917	10	89327996	89340968	IFIT3
ENST00000371826	652.9558025	-1.647239182	0.28709	-5.738	9.59E-09	2.81E-07	ENSG00000119922	10	89302045	89309271	IFIT2
ENST00000371850	267.4883633	-1.642093745	0.31376	-5.234	1.66E-07	3.64E-06	ENSG00000160293	9	133761893	133992324	VAV2
ENST00000371899	134.6264442	1.48567576	0.32755	4.5357	5.74E-06	8.30E-05	ENSG00000160326	9	133471093	133479099	SLC2A6
ENST00000371930	417.2498632	1.101251787	0.2331	4.7245	2.31E-06	3.74E-05	ENSG00000152766	10	88819895	88851844	ANKRD22
ENST00000371941	9.134753815	3.488452153	1.35165	2.5809	0.0098548	0.0433423	ENSG00000124126	20	48624251	48827999	PREX1
ENST00000372230	686.0647043	1.264378418	0.2026	6.2409	4.35E-10	1.66E-08	ENSG00000080189	20	46345983	46364425	SLC35C2
ENST00000372232	278.6611543	1.256298517	0.25279	4.9698	6.70E-07	1.25E-05	ENSG00000101844	X	108091777	108154671	ATG4A
ENST00000372271	112.8968896	-2.261363143	0.36575	-6.183	6.29E-10	2.34E-08	ENSG00000126882	9	131258077	131276510	FAM78A
ENST00000372281	103.5770134	-1.19824403	0.35468	-3.378	0.0007291	0.0052602	ENSG00000133678	10	80078664	80092551	TMEM254
ENST00000372371	682.8757106	1.224606309	0.20564	5.9551	2.60E-09	8.63E-08	ENSG00000148606	10	77975148	78029515	POLR3A
ENST00000372398	319.606878	-1.017477098	0.25711	-3.957	7.58E-05	0.0007781	ENSG00000107130	9	130172403	130237303	NCS1
ENST00000372431	50.36886811	3.42697004	0.56715	6.0425	1.52E-09	5.28E-08	ENSG00000100979	20	45898621	45912155	PLTP
ENST00000372517	96.36662687	-1.021861181	0.36656	-2.788	0.0053086	0.0265292	ENSG00000164010	1	42817121	42844991	ERMAP
ENST00000372521	201.7367919	1.187603566	0.287	4.138	3.50E-05	0.0003994	ENSG00000177868	1	42807051	42817397	SVBP
ENST00000372523	208.9056533	1.152815625	0.27666	4.1669	3.09E-05	0.0003574	ENSG00000168612	20	45881226	45885266	ZSWIM1

protein_coding	Yes	NM_138467.3	108
protein_coding	Yes	NM_001902.6	108
protein_coding	Yes	NM_012083.3	108
protein_coding	Yes	NM_002633.3	108
protein_coding	Yes	NM_138694.4	108
protein_coding	Yes	NM_181712.5	108
protein_coding	Yes	NM_000775.4	108
protein_coding	Yes	NM_015888.6	108
protein_coding	Yes	NM_001034954.3	108
protein_coding	Yes	NM_153840.4	108
protein_coding	Yes	NM_014762.4	108
protein_coding	Yes	NM_000770.3	108
protein_coding	Yes	NM_004623.5	108
protein_coding	Yes	NM_016341.4	108
protein_coding	Yes	NM_024528.4	108
protein_coding	Yes	NM_018087.5	108
protein_coding	Yes	NM_182539.4	108
protein_coding	Yes	NM_000606.3	108
protein_coding	Yes	NM_002867.4	108
protein_coding	Yes	NM_152421.4	108
protein_coding	Yes	NM_006412.4	108
protein_coding	Yes	NM_001284259.2	108
protein_coding	Yes	NM_001372327.1	108
protein_coding	Yes	NM_078626.3	108
protein_coding	Yes	NM_001549.6	108
protein_coding	Yes	NM_001547.5	108
protein_coding	Yes	NM_001134398.2	108
protein_coding	Yes	NM_017585.4	108
protein_coding	Yes	NM_144590.3	108
protein_coding	Yes	NM_020820.4	108
protein_coding	Yes	NM_015945.12	108
protein_coding	Yes	NM_052936.5	108
protein_coding	Yes	NM_033387.4	108
protein_coding	Yes	NM_025125.4	108
protein_coding	Yes	NM_007055.4	108
protein_coding	Yes	NM_014286.4	108
protein_coding	Yes	NM_006227.4	108
protein_coding	Yes	NM_001017922.2	108
protein_coding	Yes	NM_199342.4	108
protein_coding	Yes	NM_080603.5	108

ENST00000372548	26.03616771	-2.855127302	0.73827	-3.867	0.00011	0.001063	ENSG00000147231	X	106611977	106679439	RADX
ENST00000372706	70.66015251	-2.513642431	0.45514	-5.523	3.34E-08	8.60E-07	ENSG00000187815	1	40477289	40496343	ZFP69
ENST00000372841	60.95743012	1.551442472	0.51308	3.0238	0.0024962	0.0143969	ENSG00000196968	10	73772275	73776219	FUT11
ENST00000372886	199.4912761	-1.010889502	0.29619	-3.413	0.0006426	0.0047312	ENSG00000168734	20	44582578	44619037	PKIG
ENST00000372936	394.0685441	1.067432682	0.22717	4.6988	2.62E-06	4.18E-05	ENSG00000188917	X	101009349	101052111	TRMT2B
ENST00000372956	21.76395053	-3.350196867	0.83998	-3.988	6.65E-05	0.0006964	ENSG00000182489	X	100913444	100928917	XKRX
ENST00000372989	280.9506227	-1.557251906	0.27141	-5.738	9.60E-09	2.81E-07	ENSG00000102362	X	100674490	100732121	SYTL4
ENST00000373001	541.5093592	1.055085796	0.22728	4.6423	3.45E-06	5.30E-05	ENSG00000116954	1	38838197	38859772	RRAGC
ENST00000373042	336.9028977	1.1729677	0.27714	4.2324	2.31E-05	0.0002778	ENSG00000197982	1	37807789	37809454	C1orf122
ENST00000373055	433.4294848	-1.83334668	0.23513	-7.797	6.33E-15	5.96E-13	ENSG00000134690	1	37692515	37709719	CDCA8
ENST00000373087	115.4717521	1.568908629	0.34728	4.5177	6.25E-06	8.92E-05	ENSG00000163874	1	37474579	37484377	ZC3H12A
ENST00000373100	57.77575933	-1.322367613	0.46449	-2.847	0.0044146	0.0228399	ENSG00000101049	20	43559026	43585627	SGK2
ENST00000373109	79.22505996	2.736606363	0.43342	6.314	2.72E-10	1.09E-08	ENSG00000107742	10	72059033	72088551	SPOCK2
ENST00000373202	7138.801612	2.037586084	0.1674	12.172	4.37E-34	4.25E-31	ENSG00000166224	10	70815947	70881184	SGPL1
ENST00000373233	388.3168528	-2.053156392	0.2557	-8.03	9.78E-16	1.03E-13	ENSG00000124177	20	41402082	41618377	CHD6
ENST00000373257	153.4320874	1.467659969	0.31091	4.7205	2.35E-06	3.81E-05	ENSG00000132793	20	41340820	41360582	LPIN3
ENST00000373279	67.63772474	2.524747529	0.50121	5.0373	4.72E-07	9.23E-06	ENSG00000171224	10	69630246	69633596	FAM241B
ENST00000373284	45.14924727	-1.447406554	0.55496	-2.608	0.009104	0.0407117	ENSG00000160404	9	127731523	127735294	TOR2A
ENST00000373327	151.768619	-2.064111878	0.31539	-6.545	5.97E-11	2.74E-09	ENSG00000204104	2	238320517	238400897	TRAF3IP1
ENST00000373345	117.6344228	-1.797954451	0.3554	-5.059	4.21E-07	8.36E-06	ENSG00000124143	20	38601808	38650653	ARHGAP40
ENST00000373358	88.19780886	-1.04926897	0.37995	-2.762	0.0057523	0.0282169	ENSG00000102390	X	76173061	76178314	PBDC1
ENST00000373366	415.9938245	1.107534048	0.22456	4.932	8.14E-07	1.49E-05	ENSG00000188910	1	34781213	34786364	GJB3
ENST00000373383	20.6435777	-4.768387131	1.10602	-4.311	1.62E-05	0.0002044	ENSG00000094841	X	75274084	75304885	UPRT
ENST00000373394	302.9613443	-2.059621449	0.25088	-8.209	2.22E-16	2.52E-14	ENSG00000131269	X	75051047	75156283	ABCB7
ENST00000373489	413.0952278	-1.230023947	0.25001	-4.92	8.66E-07	1.57E-05	ENSG00000167081	9	125747372	125967377	PBX3
ENST00000373510	158.8745575	-1.461058105	0.31255	-4.675	2.94E-06	4.63E-05	ENSG00000160062	1	32539426	32605941	ZBTB8A
ENST00000373573	199.8104143	-1.497743826	0.27996	-5.35	8.80E-08	2.05E-06	ENSG00000147099	X	72329515	72572843	HDAC8
ENST00000373664	500.5425386	-1.613083047	0.22243	-7.252	4.11E-13	2.98E-11	ENSG00000080839	20	36996348	37095997	RBL1
ENST00000373672	50.36861359	2.626693012	0.55579	4.7261	2.29E-06	3.71E-05	ENSG00000084636	1	31652262	31704017	COL16A1
ENST00000373715	3458.51812	-1.080590117	0.17867	-6.048	1.47E-09	5.13E-08	ENSG00000112081	6	36594361	36605600	SRSF3
ENST00000373764	8.185555978	6.462666024	1.81019	3.5702	0.0003568	0.0028829	ENSG00000185681	9	122159907	122200083	MORN5
ENST00000373855	868.7784511	-1.549765968	0.1995	-7.768	7.95E-15	7.39E-13	ENSG00000119397	9	121074954	121177610	CNTRL
ENST00000373883	173.0984094	3.159013232	0.32604	9.6891	3.35E-22	8.01E-20	ENSG00000188760	2	219544027	219550595	TMEM198
ENST00000373887	21.2109119	2.197781646	0.75125	2.9255	0.0034391	0.0186848	ENSG00000056558	9	120902392	120926796	TRAF1
ENST00000373896	655.1535853	-1.186037993	0.22482	-5.275	1.32E-07	2.96E-06	ENSG00000119403	9	120855650	120877188	PHF19
ENST00000373944	1221.315169	-1.119106918	0.20778	-5.386	7.21E-08	1.72E-06	ENSG00000122952	10	56357226	56361259	ZWINT
ENST00000373953	250.1032258	1.841108224	0.30145	6.1076	1.01E-09	3.63E-08	ENSG00000065029	6	35259734	35295985	ZNF76
ENST00000374045	64.92796671	-1.12832196	0.43403	-2.6	0.0093317	0.041492	ENSG00000181634	9	114784651	114806039	TNFSF15
ENST00000374193	45.73846425	-1.686029717	0.55267	-3.051	0.002283	0.0133922	ENSG00000148225	9	113313221	113340275	WDR31
ENST00000374227	22.96532564	-7.996182894	1.58303	-5.051	4.39E-07	8.66E-06	ENSG00000136866	9	113038376	113056724	ZFP37

protein_coding	Yes	NM_018015.6	108
protein_coding	Yes	NM_001320179.2	108
protein_coding	Yes	NM_173540.3	108
protein_coding	Yes	NM_001281445.2	108
protein_coding	Yes	NM_024917.6	108
protein_coding	Yes	NM_212559.3	108
protein_coding	Yes	NM_001370165.1	108
protein_coding	Yes	NM_022157.4	108
protein_coding	Yes	NM_198446.3	108
protein_coding	Yes	NM_001256875.2	108
protein_coding	Yes	NM_025079.3	108
protein_coding	Yes	NM_170693.3	108
protein_coding	Yes	NM_001244950.2	108
protein_coding	Yes	NM_003901.4	108
protein_coding	Yes	NM_032221.5	108
protein_coding	Yes	NM_022896.3	108
protein_coding	Yes	NM_145306.3	108
protein_coding	Yes	NM_001085347.3	108
protein_coding	Yes	NM_015650.4	108
protein_coding	Yes	-	108
protein_coding	Yes	NM_016500.5	108
protein_coding	Yes	NM_024009.3	108
protein_coding	Yes	NM_145052.4	108
protein_coding	Yes	NM_001271696.3	108
protein_coding	Yes	NM_006195.6	108
protein_coding	Yes	NM_001040441.3	108
protein_coding	Yes	NM_018486.3	108
protein_coding	Yes	NM_002895.5	108
protein_coding	Yes	NM_001856.4	108
protein_coding	Yes	NM_003017.5	108
protein_coding	Yes	NM_198469.4	108
protein_coding	Yes	NM_007018.6	108
protein_coding	Yes	NM_001005209.3	108
protein_coding	Yes	NM_005658.5	108
protein_coding	Yes	NM_015651.3	108
protein_coding	Yes	NM_007057.4	108
protein_coding	Yes	NM_003427.5	108
protein_coding	Yes	NM_005118.4	108
protein_coding	Yes	NM_001012361.4	108
protein_coding	Yes	NM_003408.3	108

ENST00000374242	581.2722492	-1.002697305	0.20842	-4.811	1.50E-06	2.56E-05	ENSG00000148153	9	112683925	112718117	INIP
ENST00000374257	7434.317668	-1.366215113	0.18588	-7.35	1.98E-13	1.52E-11	ENSG00000119314	9	112218434	112333619	PTBP3
ENST00000374279	1660.187873	-1.13903081	0.17895	-6.365	1.95E-10	8.03E-09	ENSG00000148154	9	111896813	111935369	UGCG
ENST00000374293	1598.103066	-2.839550365	0.19234	-14.76	2.52E-49	8.74E-46	ENSG00000242616	9	111661604	111670226	GNG10
ENST00000374294	1595.571406	-2.714238738	0.18743	-14.48	1.59E-47	4.85E-44	ENSG00000244115	9	111631385	111670229	DNAJC25-GNG10
ENST00000374304	4.662214162	5.647844874	2.02495	2.7891	0.0052851	0.026438	ENSG00000290837	9	111603288	111631289	LRRC37A5P
ENST00000374395	606.468258	-1.072891328	0.21455	-5.001	5.72E-07	1.09E-05	ENSG00000117602	1	24502835	24541040	RCAN3
ENST00000374403	1085.95108	-1.162003269	0.19887	-5.843	5.13E-09	1.60E-07	ENSG00000090889	X	70290103	70420886	KIF4A
ENST00000374433	60.9391409	-1.533805179	0.4468	-3.433	0.0005972	0.00445	ENSG00000169740	10	43643861	43648881	ZNF32
ENST00000374446	22.70131569	-2.386760752	0.75327	-3.169	0.0015321	0.0096833	ENSG00000196793	10	43556343	43574616	ZNF239
ENST00000374469	214.9045704	-1.583026023	0.27653	-5.725	1.04E-08	3.02E-07	ENSG00000165124	9	110365247	110579741	SVEP1
ENST00000374490	270.2628869	-1.278283891	0.25385	-5.036	4.76E-07	9.31E-06	ENSG00000117305	1	23801884	23825429	HMGCL
ENST00000374516	370.1056195	1.018867391	0.23105	4.4097	1.04E-05	0.0001385	ENSG00000112511	6	33411013	33416439	PHF1
ENST00000374530	1034.441875	-4.357375215	0.21482	-20.28	1.78E-91	3.40E-87	ENSG00000157654	9	109780197	110172512	PALM2AKAP2
ENST00000374561	42.82213687	-1.721557077	0.55036	-3.128	0.0017595	0.0108413	ENSG00000117318	1	23557925	23559501	ID3
ENST00000374586	3185.838622	-1.134456979	0.17233	-6.583	4.61E-11	2.19E-09	ENSG00000106771	9	109015134	109119947	TMEM245
ENST00000374630	431.2569116	-1.630754142	0.2433	-6.703	2.05E-11	1.06E-09	ENSG00000133216	1	22710838	22921500	EPHB2
ENST00000374672	575.7115909	1.302394315	0.2194	5.9362	2.92E-09	9.58E-08	ENSG00000136826	9	107484851	107489769	KLF4
ENST00000374677	1647.408063	1.309608222	0.1923	6.8102	9.75E-12	5.38E-10	ENSG00000112473	6	33200866	33204437	SLC39A7
ENST00000374680	356.497062	1.109556356	0.24201	4.5848	4.55E-06	6.77E-05	ENSG00000204231	6	33193587	33200665	RXRB
ENST00000374692	779.7074533	-1.092219323	0.20072	-5.442	5.28E-08	1.30E-06	ENSG00000095209	9	105694540	105776629	TMEM38B
ENST00000374767	132.272132	-1.475296597	0.33262	-4.435	9.19E-06	0.0001251	ENSG00000136783	9	104747682	104760120	NIPSNAP3A
ENST00000374810	76.82779353	2.622618801	0.52108	5.033	4.83E-07	9.42E-06	ENSG00000078804	20	34704343	34713436	TP53INP2
ENST00000374811	348.038726	-1.115398449	0.2633	-4.236	2.27E-05	0.0002739	ENSG00000001497	X	65512581	65534787	LAS1L
ENST00000374859	44.86378091	2.975702376	0.56718	5.2465	1.55E-07	3.41E-06	ENSG00000240065	6	32854191	32859851	PSMB9
ENST00000374865	515.5684073	-1.288533458	0.27383	-4.706	2.53E-06	4.06E-05	ENSG00000136897	9	101387632	101398618	MRPL50
ENST00000374867	516.5058021	2.392234818	0.22975	10.412	2.18E-25	7.44E-23	ENSG00000099250	10	33177492	33334667	NRP1
ENST00000375011	138.6579714	-1.941511742	0.33697	-5.762	8.32E-09	2.48E-07	ENSG00000119514	9	98807669	98850081	GALNT12
ENST00000375077	372.7035565	-1.488145153	0.25059	-5.939	2.87E-09	9.45E-08	ENSG00000106789	9	98120974	98192637	CORO2A
ENST00000375095	71.6598178	-1.842770853	0.42062	-4.381	1.18E-05	0.0001554	ENSG00000181264	11	120325298	120333686	TLCD5
ENST00000375153	220.4269473	1.315327544	0.28411	4.6297	3.66E-06	5.59E-05	ENSG00000040487	1	19312325	19329300	SLC66A1
ENST00000375200	136.3541464	2.69452755	0.35277	7.6381	2.20E-14	1.94E-12	ENSG00000131061	20	33731995	33792269	ZNF341
ENST00000375254	8416.935662	1.4382494	0.21628	6.6498	2.93E-11	1.46E-09	ENSG00000127481	1	19074509	19210266	UBR4
ENST00000375258	300.8760095	-1.256516257	0.24572	-5.114	3.16E-07	6.50E-06	ENSG00000123600	2	171315745	171433990	METTL8
ENST00000375431	119.8839684	-1.764584565	0.34988	-5.043	4.57E-07	8.99E-06	ENSG00000139835	13	113324162	113364130	GRTP1
ENST00000375472	35.93352039	-2.021130507	0.57834	-3.495	0.0004746	0.0036742	ENSG00000157303	9	93058700	93085133	SUSD3
ENST00000375631	1758.335742	2.340658953	0.19018	12.308	8.22E-35	1.03E-31	ENSG00000204386	6	31857658	31862821	NEU1
ENST00000375645	1770.323975	1.04281049	0.20766	5.0217	5.12E-07	9.92E-06	ENSG00000068650	13	112690037	112887168	ATP11A
ENST00000375650	9630.953234	1.392946316	0.19879	7.007	2.43E-12	1.53E-10	ENSG00000204388	6	31827737	31830254	HSPA1B
ENST00000375651	383.8624816	1.516137037	0.24895	6.09	1.13E-09	4.02E-08	ENSG00000204389	6	31815542	31817942	HSPA1A

protein_coding	Yes	NM_021218.3	108
protein_coding	Yes	NM_001163788.4	108
protein_coding	Yes	NM_003358.3	108
protein_coding	Yes	NM_001017998.4	108
protein_coding	Yes	-	108
lncRNA	Yes	-	108
protein_coding	Yes	NM_013441.4	108
protein_coding	Yes	NM_012310.5	108
protein_coding	Yes	NM_006973.3	108
protein_coding	Yes	NM_001099282.2	108
protein_coding	Yes	NM_153366.4	108
protein_coding	Yes	NM_000191.3	108
protein_coding	Yes	NM_024165.3	108
protein_coding	Yes	NM_007203.5	108
protein_coding	Yes	NM_002167.5	108
protein_coding	Yes	NM_032012.4	108
protein_coding	Yes	NM_017449.5	108
protein_coding	Yes	NM_004235.6	108
protein_coding	Yes	NM_006979.3	108
protein_coding	Yes	NM_021976.5	108
protein_coding	Yes	NM_018112.3	108
protein_coding	Yes	NM_015469.3	108
protein_coding	Yes	NM_021202.3	108
protein_coding	Yes	NM_031206.7	108
protein_coding	Yes	NM_002800.5	108
protein_coding	Yes	NM_019051.3	108
protein_coding	Yes	NM_003873.7	108
protein_coding	Yes	NM_024642.5	108
protein_coding	Yes	NM_052820.4	108
protein_coding	Yes	NM_001198671.2	108
protein_coding	Yes	NM_001040125.2	108
protein_coding	Yes	NM_001282933.2	108
protein_coding	Yes	NM_020765.3	108
protein_coding	Yes	NM_001321154.2	108
protein_coding	Yes	NM_024719.4	108
protein_coding	Yes	NM_145006.4	108
protein_coding	Yes	NM_000434.4	108
protein_coding	Yes	NM_015205.3	108
protein_coding	Yes	NM_005346.6	108
protein_coding	Yes	NM_005345.6	108

ENST00000375654	54.6150433	2.142516164	0.48618	4.4069	1.05E-05	0.00014	ENSG00000204390	6	31809618	31815283	HSPA1L
ENST00000375688	45.50688536	1.793166989	0.5545	3.2339	0.0012213	0.0080424	ENSG00000204396	6	31765589	31777328	VWA7
ENST00000375749	1167.226604	-1.899725341	0.2005	-9.475	2.67E-21	5.63E-19	ENSG00000101346	20	32207879	32238658	POFUT1
ENST00000375898	285.2797848	-1.237380779	0.25656	-4.823	1.41E-06	2.42E-05	ENSG00000139826	13	108218391	108234243	ABHD13
ENST00000375980	221.7794515	-1.316712126	0.3411	-3.86	0.0001133	0.0010906	ENSG00000142634	1	15409887	15430339	EFHD2
ENST00000376004	89.30124945	-1.487503445	0.38239	-3.89	0.0001002	0.0009837	ENSG00000134901	13	102784280	102798976	POGLUT2
ENST00000376008	337.4682687	-1.067442003	0.23748	-4.495	6.96E-06	9.83E-05	ENSG00000171729	1	15153746	15220478	TMEM51
ENST00000376032	157.1591648	-1.272081575	0.30843	-4.124	3.72E-05	0.0004206	ENSG00000151287	13	102765887	102773786	TEX30
ENST00000376104	44.05134305	2.045115485	0.52822	3.8717	0.0001081	0.0010467	ENSG00000150672	11	83455172	85627344	DLG2
ENST00000376112	312.2890035	-3.142160502	0.36851	-8.527	1.51E-17	2.00E-15	ENSG00000125968	20	31605288	31606510	ID1
ENST00000376350	30.37481166	1.919220779	0.63794	3.0085	0.0026255	0.0150085	ENSG00000080031	19	55181246	55209501	PTPRH
ENST00000376358	341.8430599	1.455865134	0.24148	6.0289	1.65E-09	5.72E-08	ENSG00000288053	X	49071469	49079887	-
ENST00000376371	54.39321342	-1.708181227	0.48915	-3.492	0.0004791	0.0037044	ENSG00000165113	9	83739424	83817769	GKAP1
ENST00000376372	614.3895351	1.489492985	0.21022	7.0856	1.38E-12	9.19E-11	ENSG00000196998	X	49074441	49079887	WDR45
ENST00000376389	461.7697335	-1.473733821	0.23357	-6.31	2.80E-10	1.11E-08	ENSG00000137312	6	30727708	30742687	FLOT1
ENST00000376447	757.5349677	-1.009316959	0.19797	-5.098	3.43E-07	6.98E-06	ENSG00000165105	9	82979589	83063142	RASEF
ENST00000376568	3942.838275	1.077037412	0.19087	5.6427	1.67E-08	4.64E-07	ENSG00000204580	6	30884518	30900156	DDR1
ENST00000376573	131.1551622	-1.000107389	0.32065	-3.119	0.0018149	0.0111115	ENSG00000150867	10	22534853	22714578	PIP4K2A
ENST00000376588	371.5002981	-7.237265285	0.5122	-14.13	2.49E-45	7.05E-42	ENSG00000135069	9	78297124	78330093	PSAT1
ENST00000376854	155.416904	-1.953439651	0.36443	-5.36	8.31E-08	1.95E-06	ENSG00000135045	9	74946582	74952912	C9orf40
ENST00000377185	7.359262396	3.76151506	1.48496	2.5331	0.0113068	0.0482051	ENSG00000204710	11	65170232	65173374	SPDYC
ENST00000377190	278.5812593	1.37761532	0.28383	4.8537	1.21E-06	2.11E-05	ENSG00000162298	11	65127278	65134519	SYVN1
ENST00000377275	1613.641357	-1.700635128	0.19906	-8.543	1.30E-17	1.75E-15	ENSG00000165997	10	18659430	18681639	ARL5B
ENST00000377294	54.08094291	-2.674525449	0.50319	-5.315	1.07E-07	2.43E-06	ENSG00000187626	6	28241696	28252269	ZKSCAN4
ENST00000377401	1651.564957	-1.544512423	0.1836	-8.412	4.02E-17	5.01E-15	ENSG00000185130	6	27807478	27807929	H2BC13
ENST00000377459	1866.233591	-1.110351847	0.18519	-5.996	2.03E-09	6.87E-08	ENSG00000274997	6	27147105	27147562	H2AC12
ENST00000377474	8.87482156	-5.132363723	1.7492	-2.934	0.0033449	0.0182734	ENSG00000178695	13	76880174	76886405	KCTD12
ENST00000377482	6269.359506	-1.154167471	0.17584	-6.564	5.25E-11	2.45E-09	ENSG00000116285	1	8011726	8026309	ERRFI1
ENST00000377507	68.01663514	1.311244189	0.47129	2.7823	0.0053981	0.0268749	ENSG00000049249	1	7915870	7940839	TNFRSF9
ENST00000377524	596.0532325	-1.30751506	0.20988	-6.23	4.67E-10	1.77E-08	ENSG00000136738	10	17644150	17716824	STAM
ENST00000377526	1180.138875	-1.124635431	0.19583	-5.743	9.30E-09	2.74E-07	ENSG00000115307	2	74526651	74529706	AUP1
ENST00000377532	1287.923647	1.024563543	0.25506	4.0169	5.90E-05	0.000627	ENSG00000049246	1	7784290	7845177	PER3
ENST00000377561	5.287678854	-5.877776934	1.96677	-2.989	0.0028031	0.0158171	ENSG00000154529	9	41890535	42129426	CNTNAP3B
ENST00000377658	1907.42282	2.619668538	0.24201	10.825	2.63E-27	1.17E-24	ENSG00000162413	1	6590723	6602869	KLHL21
ENST00000377669	293.6514653	-1.856297652	0.35252	-5.266	1.40E-07	3.10E-06	ENSG00000118922	13	73686088	74133929	KLF12
ENST00000377697	142.9992817	4.327303888	0.43873	9.8633	6.01E-23	1.62E-20	ENSG00000204869	19	46039181	46041002	IGFL4
ENST00000377708	705.5393701	1.178034998	0.22292	5.2846	1.26E-07	2.83E-06	ENSG00000186470	6	26365197	26378312	BTN3A2
ENST00000377727	1148.839018	-2.005531835	0.2228	-9.001	2.23E-19	3.68E-17	ENSG00000158406	6	26285125	26285534	H4C8
ENST00000377733	1335.342564	-1.277796187	0.18445	-6.927	4.28E-12	2.54E-10	ENSG00000278588	6	26272930	26273412	H2BC10
ENST00000377745	1127.441642	-1.40188527	0.18736	-7.482	7.30E-14	5.94E-12	ENSG00000278705	6	26026895	26027283	H4C2

protein_coding	Yes	NM_005527.4	108
protein_coding	Yes	NM_025258.3	108
protein_coding	Yes	NM_015352.2	108
protein_coding	Yes	NM_032859.3	108
protein_coding	Yes	NM_024329.6	108
protein_coding	Yes	NM_024089.3	108
protein_coding	Yes	NM_001136218.2	108
protein_coding	Yes	NM_138779.5	108
protein_coding	Yes	NM_001142699.3	108
protein_coding	Yes	NM_002165.4	108
protein_coding	Yes	NM_002842.5	108
protein_coding	Yes	-	108
protein_coding	Yes	NM_025211.4	108
protein_coding	Yes	NM_001029896.2	108
protein_coding	Yes	NM_005803.4	108
protein_coding	Yes	NM_152573.4	108
protein_coding	Yes	NM_001297654.2	108
protein_coding	Yes	NM_005028.5	108
protein_coding	Yes	NM_058179.4	108
protein_coding	Yes	NM_017998.3	108
protein_coding	Yes	NM_001008778.3	108
protein_coding	Yes	NM_172230.3	108
protein_coding	Yes	NM_178815.5	108
protein_coding	Yes	NM_019110.5	108
protein_coding	Yes	NM_003519.4	108
protein_coding	Yes	NM_080596.3	108
protein_coding	Yes	NM_138444.4	108
protein_coding	Yes	NM_018948.4	108
protein_coding	Yes	NM_001561.6	108
protein_coding	Yes	NM_003473.4	108
protein_coding	Yes	NM_181575.5	108
protein_coding	Yes	NM_001377275.1	108
protein_coding	Yes	NM_001201380.3	108
protein_coding	Yes	NM_014851.4	108
protein_coding	Yes	-	108
protein_coding	Yes	NM_001002923.3	108
protein_coding	Yes	NM_007047.5	108
protein_coding	Yes	NM_003543.4	108
protein_coding	Yes	NM_003525.3	108
protein_coding	Yes	NM_003544.3	108

ENST00000377791	2144.289218	-1.029653674	0.17575	-5.859	4.67E-09	1.47E-07	ENSG00000180573	6	26124171	26124690	H2AC6
ENST00000377799	59.32014422	-3.057766228	0.50676	-6.034	1.60E-09	5.55E-08	ENSG00000107614	10	17137335	17201672	TRDMT1
ENST00000377803	3766.338172	-1.148135871	0.1779	-6.454	1.09E-10	4.76E-09	ENSG00000197061	6	26103932	26104337	H4C3
ENST00000377818	784.1680293	-1.693353839	0.21533	-7.864	3.72E-15	3.65E-13	ENSG00000204899	13	72708366	72727629	MZT1
ENST00000377834	102.8386555	-3.863830679	0.45249	-8.539	1.35E-17	1.81E-15	ENSG00000069812	1	6415231	6419919	HES2
ENST00000377838	950.2129255	1.91651076	0.19611	9.7728	1.47E-22	3.74E-20	ENSG00000066827	8	134477787	134713031	ZFAT
ENST00000377873	119.8440853	1.87407848	0.352	5.324	1.02E-07	2.33E-06	ENSG00000125864	20	17493904	17531379	BFSP1
ENST00000377939	327.1327366	1.592612012	0.31131	5.1158	3.12E-07	6.43E-06	ENSG00000158286	1	6206118	6221299	RNF207
ENST00000377953	110.1680668	-2.952708062	0.44822	-6.588	4.47E-11	2.13E-09	ENSG00000204922	11	62671672	62673686	UQCC3
ENST00000378043	11.96516889	2.769822116	1.0744	2.578	0.0099367	0.0436194	ENSG00000167995	11	61950350	61964461	BEST1
ENST00000378083	39.54001108	2.538110638	0.57499	4.4142	1.01E-05	0.000136	ENSG00000069424	1	6045887	6101180	KCNAB2
ENST00000378165	84.20871055	-1.152000585	0.38256	-3.011	0.0026015	0.0148959	ENSG00000152465	10	15105769	15168693	NMT2
ENST00000378198	524.7640483	1.014510452	0.2174	4.6665	3.06E-06	4.79E-05	ENSG00000111802	6	24649978	24666899	TDP2
ENST00000378214	86.95785387	2.254682358	0.42324	5.3272	9.98E-08	2.29E-06	ENSG00000137261	6	24544106	24646191	KIAA0319
ENST00000378247	67.26474141	1.454219384	0.43263	3.3613	0.0007757	0.0055431	ENSG00000204991	16	89828474	89871319	SPIRE2
ENST00000378279	4.149833674	5.480651158	2.08437	2.6294	0.0085535	0.0387013	ENSG00000205002	8	116938206	116944487	AARD
ENST00000378295	213.3767986	-1.7495896	0.28876	-6.059	1.37E-09	4.82E-08	ENSG00000078900	1	3652515	3736201	TP73
ENST00000378364	169.9356122	-1.010452656	0.32259	-3.132	0.0017344	0.0107108	ENSG00000198931	16	88809338	88811928	APRT
ENST00000378372	291.5368054	-1.370182442	0.26252	-5.219	1.80E-07	3.90E-06	ENSG00000187522	10	14838305	14871741	HSPA14
ENST00000378387	347.1497493	-1.613120028	0.2442	-6.606	3.96E-11	1.91E-09	ENSG00000137135	9	35659342	35665195	ARHGEF39
ENST00000378444	263.1037679	-1.390125651	0.26495	-5.247	1.55E-07	3.41E-06	ENSG00000183337	X	40051250	40097958	BCOR
ENST00000378473	257.3801643	1.664976615	0.26229	6.3479	2.18E-10	8.90E-09	ENSG00000101333	20	9069086	9480808	PLCB4
ENST00000378486	12.75352211	3.172844833	1.0702	2.9647	0.0030296	0.0168276	ENSG00000149527	1	2476288	2505532	PLCH2
ENST00000378509	118.6633605	-2.298613699	0.35223	-6.526	6.76E-11	3.07E-09	ENSG00000205060	7	134289331	134316930	SLC35B4
ENST00000378526	1070.182376	1.26751959	0.1913	6.6256	3.46E-11	1.70E-09	ENSG00000005156	17	34980511	35009743	LIG3
ENST00000378572	296.3472654	-1.111259531	0.24517	-4.533	5.83E-06	8.41E-05	ENSG00000165630	10	13586964	13630859	PRPF18
ENST00000378578	489.0573068	-1.158119394	0.23071	-5.02	5.17E-07	1.00E-05	ENSG00000165169	X	37838835	37847571	DYNLT3
ENST00000378585	8.787099268	-4.111194082	1.45409	-2.827	0.0046939	0.0240101	ENSG00000187730	1	2019344	2030758	GABRD
ENST00000378616	63.22302726	-5.654867708	0.80284	-7.044	1.87E-12	1.21E-10	ENSG00000047597	X	37685790	37732130	XK
ENST00000378693	15.11137177	-7.392663835	1.6489	-4.483	7.35E-06	0.000103	ENSG00000198944	5	132813301	132816786	SOWAHA
ENST00000378700	19.15186772	-3.378429401	0.94548	-3.573	0.0003526	0.0028565	ENSG00000172201	6	19837369	19842197	ID4
ENST00000378714	688.0915857	-1.776800442	0.2305	-7.708	1.27E-14	1.16E-12	ENSG00000065328	10	13161557	13211110	MCM10
ENST00000378842	667.0827075	1.537839982	0.20566	7.4776	7.57E-14	6.13E-12	ENSG00000213930	9	34646674	34651035	GALT
ENST00000378850	16.45858862	2.626681693	0.90562	2.9004	0.0037267	0.019921	ENSG00000205129	4	185426253	185449826	C4orf47
ENST00000379028	23.41240473	3.409271351	0.81137	4.2019	2.65E-05	0.0003125	ENSG00000091129	7	108147648	108456436	NRCAM
ENST00000379063	5.128950101	5.786184995	1.97811	2.9251	0.0034433	0.0186933	ENSG00000205184	8	81634356	81635662	SLC10A5P1
ENST00000379214	1717.530885	-1.408978336	0.18495	-7.618	2.57E-14	2.23E-12	ENSG00000205213	11	27365960	27472790	LGR4
ENST00000379287	372.1262131	-1.202951728	0.23579	-5.102	3.36E-07	6.86E-06	ENSG00000145990	6	13357829	13487600	GFOD1
ENST00000379289	3.916465705	5.396742222	2.11677	2.5495	0.0107872	0.0464746	ENSG00000162571	1	1173879	1197936	TTLL10
ENST00000379410	14.41683085	-2.443446038	0.95133	-2.568	0.010215	0.0445863	ENSG00000187583	1	966481	975865	PLEKHN1

protein_coding	Yes	NM_003512.4	108
protein_coding	Yes	NM_004412.7	108
protein_coding	Yes	NM_003542.4	108
protein_coding	Yes	NM_001071775.3	108
protein_coding	Yes	NM_019089.5	108
protein_coding	Yes	NM_020863.4	108
protein_coding	Yes	NM_001195.5	108
protein_coding	Yes	NM_207396.3	108
protein_coding	Yes	NM_001085372.3	108
protein_coding	Yes	NM_004183.4	108
protein_coding	Yes	NM_001199862.2	108
protein_coding	Yes	NM_004808.3	108
protein_coding	Yes	NM_016614.3	108
protein_coding	Yes	NM_014809.4	108
protein_coding	Yes	NM_032451.2	108
protein_coding	Yes	NM_001025357.3	108
protein_coding	Yes	NM_005427.4	108
protein_coding	Yes	NM_000485.3	108
protein_coding	Yes	NM_016299.4	108
protein_coding	Yes	NM_032818.3	108
protein_coding	Yes	NM_001123385.2	108
protein_coding	Yes	NM_001377142.1	108
protein_coding	Yes	NM_014638.4	108
protein_coding	Yes	NM_032826.5	108
protein_coding	Yes	NM_013975.4	108
protein_coding	Yes	NM_003675.4	108
protein_coding	Yes	NM_006520.3	108
protein_coding	Yes	NM_000815.5	108
protein_coding	Yes	NM_021083.4	108
protein_coding	Yes	NM_175873.6	108
protein_coding	Yes	NM_001546.4	108
protein_coding	Yes	NM_018518.5	108
protein_coding	Yes	NM_000155.4	108
protein_coding	Yes	NM_001114357.3	108
protein_coding	Yes	NM_001037132.4	108
rocessed_pseudog	Yes	-	108
protein_coding	Yes	NM_018490.5	108
protein_coding	Yes	NM_018988.4	108
protein_coding	Yes	NM_001130045.2	108
protein_coding	Yes	NM_032129.3	108

ENST00000379426	93.46962594	-2.367731992	0.39644	-5.972	2.34E-09	7.82E-08	ENSG00000205269	6	11537748	11583524	TMEM170B
ENST00000379483	254.6915658	-1.088169302	0.26551	-4.098	4.16E-05	0.0004637	ENSG00000120696	13	41189833	41194569	KBTBD7
ENST00000379484	128.4609911	-1.643163697	0.36125	-4.549	5.40E-06	7.86E-05	ENSG00000012174	X	21839616	21885423	MBTPS2
ENST00000379589	9.921714552	-4.244859811	1.41668	-2.996	0.0027325	0.0155021	ENSG00000183722	13	39342891	39603193	LHFPL6
ENST00000379607	1099.393811	-1.316837482	0.19314	-6.818	9.23E-12	5.14E-10	ENSG00000173674	X	20124524	20141838	EIF1AX
ENST00000379661	155.628347	-1.405200942	0.30726	-4.573	4.80E-06	7.10E-05	ENSG00000140854	16	57735769	57757244	KATNB1
ENST00000379672	49.10862808	-3.436999396	0.5688	-6.043	1.52E-09	5.28E-08	ENSG00000006740	17	12789497	12991643	ARHGAP44
ENST00000379705	19.10591338	-1.956149709	0.7735	-2.529	0.0114398	0.0486647	ENSG00000133107	13	37632062	37869772	TRPC4
ENST00000379888	565.2315248	-1.856089462	0.21991	-8.44	3.16E-17	4.01E-15	ENSG00000134453	10	6089033	6117447	RBM17
ENST00000379915	11.5896412	6.963932258	1.71057	4.0711	4.68E-05	0.0005132	ENSG00000260729	15	72284726	72375981	-
ENST00000380071	687.177976	-1.103818116	0.21662	-5.096	3.48E-07	7.07E-06	ENSG00000133119	13	33818148	33837500	RFC3
ENST00000380097	112.5262946	-2.902090225	0.40938	-7.089	1.35E-12	8.98E-11	ENSG00000121236	11	5596636	5612952	TRIM6
ENST00000380118	9.550863218	4.162067965	1.39822	2.9767	0.0029137	0.0163046	ENSG00000198721	6	4115705	4135575	ECI2
ENST00000380191	4754.740957	-1.258271659	0.17066	-7.373	1.67E-13	1.29E-11	ENSG00000057608	10	5765222	5813434	GDI2
ENST00000380338	47.9106628	-3.136610521	0.55754	-5.626	1.85E-08	5.07E-07	ENSG00000171843	9	20341668	20622499	MLLT3
ENST00000380412	511.3425156	-1.168932802	0.23447	-4.986	6.18E-07	1.16E-05	ENSG00000169957	16	30524003	30526566	ZNF768
ENST00000380526	74.93492637	2.608457718	0.45359	5.7507	8.89E-09	2.63E-07	ENSG00000228175	1	89993592	89994321	GEMIN8P4
ENST00000380554	115.2330617	-1.202706123	0.37513	-3.206	0.0013454	0.0087047	ENSG00000196139	10	5094413	5107686	AKR1C3
ENST00000380615	283.6794488	-1.289821021	0.25205	-5.117	3.10E-07	6.39E-06	ENSG00000102781	13	30202629	30307551	KATNAL1
ENST00000380625	44.87959969	8.915693773	1.5204	5.864	4.52E-09	1.43E-07	ENSG00000187268	X	13035616	13044620	FAM9C
ENST00000380629	770.6808962	-1.286121422	0.27767	-4.632	3.63E-06	5.54E-05	ENSG00000104765	8	26383053	26413127	BNIP3L
ENST00000380668	1141.010829	-1.827065506	0.18866	-9.684	3.52E-22	8.35E-20	ENSG00000101911	X	12791411	12824222	PRPS2
ENST00000380698	57.80756516	-5.263382746	0.73522	-7.159	8.13E-13	5.59E-11	ENSG00000170542	6	2887269	2903309	SERPINB9
ENST00000380733	530.0324993	-1.320071149	0.21743	-6.071	1.27E-09	4.49E-08	ENSG00000164985	9	15464065	15510970	PSIP1
ENST00000380770	27.40341684	3.299918468	0.73837	4.4692	7.85E-06	0.000109	ENSG00000105371	19	10286954	10288520	ICAM4
ENST00000380861	124.5591009	-4.763222126	0.54337	-8.766	1.85E-18	2.72E-16	ENSG00000047644	X	10015253	10144474	WWC3
ENST00000380877	476.2288725	-1.001612169	0.23295	-4.3	1.71E-05	0.0002139	ENSG00000104064	15	50275388	50355198	GABPB1
ENST00000380916	394.996136	-1.269949309	0.24284	-5.23	1.70E-07	3.72E-06	ENSG00000175893	9	14611070	14693432	ZDHHC21
ENST00000380950	452.1650685	-2.250466371	0.22907	-9.824	8.85E-23	2.30E-20	ENSG00000103995	15	48737937	48811069	CEP152
ENST00000380956	15.80794169	7.410722009	1.63993	4.5189	6.22E-06	8.88E-05	ENSG00000137265	6	391751	411443	IRF4
ENST00000381125	1512.86995	-1.738025702	0.21785	-7.978	1.49E-15	1.52E-13	ENSG00000067057	10	3067547	3136802	PFKP
ENST00000381151	5488.431423	1.28182618	0.16925	7.5736	3.63E-14	3.09E-12	ENSG00000198743	21	34073577	34106260	SLC5A3
ENST00000381160	7.132827502	6.260964677	1.84667	3.3904	0.0006979	0.0050735	ENSG00000015520	7	44512534	44541330	NPC1L1
ENST00000381249	271.1575873	-1.011641907	0.25226	-4.01	6.06E-05	0.0006422	ENSG00000240857	2	18554722	18560653	RDH14
ENST00000381273	28.35488181	1.87208707	0.64567	2.8994	0.0037384	0.0199729	ENSG00000205693	12	27762426	27780236	MANSC4
ENST00000381323	125.9209753	-1.277136783	0.43289	-2.95	0.0031748	0.0174949	ENSG00000197872	2	16549458	16665834	CYRIA
ENST00000381344	1106.906324	-1.607233618	0.18945	-8.484	2.18E-17	2.84E-15	ENSG00000067064	10	1039418	1049119	IDI1
ENST00000381346	1382.756302	-1.079871665	0.18486	-5.842	5.17E-09	1.61E-07	ENSG00000072042	14	67676799	67695764	RDH11
ENST00000381356	94.03088683	-1.01562426	0.38091	-2.666	0.0076692	0.0354543	ENSG00000205707	12	25195236	25205143	ETFRF1
ENST00000381431	15.49566548	-2.977446845	0.98536	-3.022	0.0025138	0.014484	ENSG00000163069	4	52020705	52038299	SGCB

protein_coding	Yes	NM_001100829.3	108
protein_coding	Yes	NM_032138.7	108
protein_coding	Yes	NM_015884.4	108
protein_coding	Yes	NM_005780.3	108
protein_coding	Yes	NM_001412.4	108
protein_coding	Yes	NM_005886.3	108
protein_coding	Yes	NM_014859.6	108
protein_coding	Yes	NM_016179.4	108
protein_coding	Yes	NM_032905.5	108
protein_coding	Yes	-	108
protein_coding	Yes	NM_002915.4	108
protein_coding	Yes	NM_001003818.3	108
protein_coding	Yes	NM_206836.3	108
protein_coding	Yes	NM_001494.4	108
protein_coding	Yes	NM_004529.4	108
protein_coding	Yes	NM_024671.4	108
ocessed_pseudoge	Yes	-	108
protein_coding	Yes	NM_003739.6	108
protein_coding	Yes	NM_032116.5	108
protein_coding	Yes	NM_174901.6	108
protein_coding	Yes	NM_004331.3	108
protein_coding	Yes	NM_002765.5	108
protein_coding	Yes	NM_004155.6	108
protein_coding	Yes	NM_033222.5	108
protein_coding	Yes	NM_001544.5	108
protein_coding	Yes	-	108
protein_coding	Yes	NM_016654.5	108
protein_coding	Yes	NM_178566.6	108
protein_coding	Yes	NM_001194998.2	108
protein_coding	Yes	NM_002460.4	108
protein_coding	Yes	NM_002627.5	108
protein_coding	Yes	NM_006933.7	108
protein_coding	Yes	NM_001101648.2	108
protein_coding	Yes	NM_020905.4	108
protein_coding	Yes	NM_001146221.5	108
protein_coding	Yes	NM_030797.4	108
protein_coding	Yes	NM_004508.4	108
protein_coding	Yes	NM_016026.4	108
protein_coding	Yes	NM_001001660.3	108
protein_coding	Yes	NM_000232.5	108

ENST00000381486	46.69286669	3.609303143	0.59955	6.0201	1.74E-09	6.01E-08	ENSG00000196208	2	11534044	11642788	GREB1
ENST00000381604	1592.825889	-2.007693677	0.18197	-11.03	2.65E-28	1.34E-25	ENSG00000015171	10	135454	254637	ZMYND11
ENST00000381620	14.52699047	7.289441104	1.65918	4.3934	1.12E-05	0.0001479	ENSG00000151834	4	46243547	46390128	GABRA2
ENST00000381624	4.428846192	5.573362803	2.05277	2.715	0.0066267	0.0316529	ENSG00000174898	19	5720636	5778734	CATSPERD
ENST00000381668	28.82818705	1.963043013	0.71927	2.7292	0.0063489	0.030574	ENSG00000124406	4	42408372	42657105	ATP8A1
ENST00000381801	932.8993775	-1.363314145	0.19671	-6.93	4.20E-12	2.49E-10	ENSG00000139505	13	25246221	25287488	MTMR6
ENST00000381809	494.5231372	-1.179908317	0.22597	-5.221	1.77E-07	3.86E-06	ENSG00000147853	9	4709555	4741202	AK3
ENST00000381867	57.54627422	-1.772404773	0.49424	-3.586	0.0003356	0.0027425	ENSG00000101298	20	1266293	1309327	SNPH
ENST00000381962	4553.13204	2.161219106	0.21795	9.9161	3.54E-23	9.85E-21	ENSG00000271303	20	646614	653200	SRXN1
ENST00000381967	912.7041866	-1.142792113	0.19958	-5.726	1.03E-08	2.99E-07	ENSG00000169299	4	37826685	37862937	PGM2
ENST00000381980	57.52703992	-6.867272623	1.10612	-6.208	5.35E-10	2.01E-08	ENSG00000154274	4	37453924	37593510	C4orf19
ENST00000382038	566.9891753	-1.280869711	0.22557	-5.678	1.36E-08	3.86E-07	ENSG00000113456	5	34905259	34915504	RAD1
ENST00000382040	65.11114655	-4.672331412	0.72917	-6.408	1.48E-10	6.24E-09	ENSG00000134321	2	6877776	6898239	RSAD2
ENST00000382065	50.79399364	1.302093761	0.4778	2.7252	0.0064267	0.0308797	ENSG00000082196	5	34017857	34043213	C1QTNF3
ENST00000382349	19.9666491	2.625417875	0.80135	3.2762	0.001052	0.0071036	ENSG00000205922	19	1753505	1780988	ONECUT3
ENST00000382361	1564.811479	-1.619636599	0.23744	-6.821	9.02E-12	5.03E-10	ENSG00000075618	7	5592815	5606655	FSCN1
ENST00000382448	236.5415278	1.350827236	0.27535	4.9058	9.31E-07	1.67E-05	ENSG00000168970	15	41828115	41848143	JMJD7-PLA2G4B
ENST00000382554	189.5056949	-2.657256544	0.35525	-7.48	7.44E-14	6.04E-12	ENSG00000205978	14	24399002	24419283	NYNRIN
ENST00000382723	636.2218921	3.496118884	1.27903	2.7334	0.0062682	0.0302529	ENSG00000163132	4	4859664	4863936	MSX1
ENST00000382762	239.74696	1.059540474	0.26944	3.9324	8.41E-05	0.0008491	ENSG00000177951	11	202923	207399	BET1L
ENST00000382774	6.688913837	6.167252662	1.87877	3.2826	0.0010286	0.0069733	ENSG00000109758	4	3441967	3449486	HGFAC
ENST00000383472	61.63910843	-2.056695715	0.45524	-4.518	6.25E-06	8.92E-05	ENSG00000088756	18	6729715	6915716	ARHGAP28
ENST00000383678	197.4440258	-1.729528866	0.30152	-5.736	9.69E-09	2.84E-07	ENSG00000163607	3	112990983	113001969	GTPBP8
ENST00000383759	77.84088065	1.054056707	0.40202	2.6219	0.0087448	0.0394236	ENSG00000136059	3	37990785	38007185	VILL
ENST00000383836	43.24877769	1.980771575	0.73154	2.7077	0.006776	0.032211	ENSG00000196220	3	8980590	9249646	SRGAP3
ENST00000383869	130.2963024	-1.104630466	0.33032	-3.344	0.0008255	0.0058247	ENSG00000206596	14	34546713	34546877	RNU1-27P
ENST00000384101	26.36519109	-3.468868026	0.77016	-4.504	6.67E-06	9.45E-05	ENSG00000206828	1	149636765	149636929	RNVU1-30
ENST00000384263	68.11692269	-1.065118623	0.42231	-2.522	0.0116639	0.0493831	ENSG00000206990	1	165662584	165662687	Y_RNA
ENST00000384476	27.49692983	-3.201491601	0.7268	-4.405	1.06E-05	0.0001411	ENSG00000207205	1	144412575	144412740	RNVU1-15
ENST00000384770	38.62403246	-3.249614854	0.62532	-5.197	2.03E-07	4.36E-06	ENSG00000207501	1	145281115	145281279	RNVU1-14
ENST00000387069	35.67306682	-2.025643681	0.59645	-3.396	0.0006834	0.0049853	ENSG00000274978	1	28648599	28648733	RNU11
ENST00000388747	83.25570654	-1.447196651	0.40897	-3.539	0.0004022	0.0031932	ENSG00000167103	9	127920880	127930777	PIP5KL1
ENST00000388934	90.84536729	-2.561239799	0.40008	-6.402	1.54E-10	6.46E-09	ENSG00000172478	2	240886047	240896156	MAB21L4
ENST00000388995	1115.959461	1.049553574	0.22814	4.6005	4.21E-06	6.33E-05	ENSG00000188522	17	18968788	19004764	FAM83G
ENST00000389005	1122.002157	-1.046482852	0.18946	-5.524	3.32E-08	8.57E-07	ENSG00000074356	17	3802157	3846246	NCBP3
ENST00000389176	13.6066121	2.970218599	1.08378	2.7406	0.0061325	0.0297301	ENSG00000197599	16	1434382	1444556	CCDC154
ENST00000389221	540.4004654	1.223211428	0.25663	4.7665	1.87E-06	3.12E-05	ENSG00000059145	16	1363204	1414704	UNKL
ENST00000389313	34.42312136	-3.876112609	0.71674	-5.408	6.38E-08	1.54E-06	ENSG00000188176	17	4584528	4608319	SMTNL2
ENST00000389420	252.1740953	-1.294704658	0.28486	-4.545	5.49E-06	7.98E-05	ENSG00000173157	12	43353865	43552203	ADAMTS20
ENST00000389622	721.7543635	-1.006165479	0.20122	-5	5.72E-07	1.09E-05	ENSG00000080200	3	97822010	97944984	CRYBG3

protein_coding	Yes	NM_014668.4	108
protein_coding	Yes	NM_001370100.5	108
protein_coding	Yes	NM_000807.4	108
protein_coding	Yes	NM_152784.4	108
protein_coding	Yes	NM_006095.2	108
protein_coding	Yes	NM_004685.5	108
protein_coding	Yes	NM_016282.4	108
protein_coding	Yes	NM_001318234.2	108
protein_coding	Yes	NM_080725.3	108
protein_coding	Yes	NM_018290.4	108
protein_coding	Yes	NM_001104629.2	108
protein_coding	Yes	NM_002853.4	108
protein_coding	Yes	NM_080657.5	108
protein_coding	Yes	NM_181435.6	108
protein_coding	Yes	NM_001080488.2	108
protein_coding	Yes	NM_003088.4	108
protein_coding	Yes	-	108
protein_coding	Yes	NM_025081.3	108
protein_coding	Yes	NM_002448.3	108
protein_coding	Yes	NM_001098787.2	108
protein_coding	Yes	NM_001528.4	108
protein_coding	Yes	NM_001366230.1	108
protein_coding	Yes	NM_014170.4	108
protein_coding	Yes	NM_015873.4	108
protein_coding	Yes	NM_014850.4	108
snRNA	Yes	-	108
snRNA	Yes	-	108
misc_RNA	Yes	-	108
snRNA	Yes	-	108
snRNA	Yes	-	108
snRNA	Yes	-	108
protein_coding	Yes	NM_001135219.2	108
protein_coding	Yes	NM_001085437.3	108
protein_coding	Yes	NM_001039999.3	108
protein_coding	Yes	NM_001114118.3	108
protein_coding	Yes	NM_001143980.3	108
protein_coding	Yes	NM_001372107.1	108
protein_coding	Yes	NM_001114974.2	108
protein_coding	Yes	NM_025003.5	108
protein_coding	Yes	NM_153605.4	108

ENST00000389658	145.7912637	1.0730671	0.34188	3.1387	0.0016967	0.0105254	ENSG00000101680	18	6941741	7117797	LAMA1
ENST00000389758	6.085244253	6.033340122	1.9048	3.1674	0.0015379	0.0097108	ENSG00000185038	2	233778329	233833418	MROH2A
ENST00000389759	1452.821131	-1.057457904	0.18275	-5.786	7.19E-09	2.18E-07	ENSG00000144283	2	158456951	158681429	PKP4
ENST00000389805	11148.28043	1.788826799	0.24269	7.3707	1.70E-13	1.31E-11	ENSG00000161011	5	179820904	179838078	SQSTM1
ENST00000389834	4199.033278	1.183095	0.17482	6.7676	1.31E-11	7.02E-10	ENSG00000103978	15	42210446	42273433	TMEM87A
ENST00000389840	25.66866148	4.616087091	0.95523	4.8325	1.35E-06	2.32E-05	ENSG00000187775	17	78423696	78577396	DNAH17
ENST00000389912	352.1005863	-1.70113537	0.24806	-6.858	7.00E-12	3.99E-10	ENSG00000133983	14	70325080	70359683	COX16
ENST00000390168	2205.2381	-1.761793191	0.17768	-9.915	3.57E-23	9.90E-21	ENSG00000284010	11	1996758	1996831	MIR675
ENST00000390667	491.4690848	-1.633462604	0.22207	-7.356	1.90E-13	1.46E-11	ENSG00000136122	13	72727922	72756196	BORA
ENST00000390893	29.65677912	-4.033888684	0.7876	-5.122	3.03E-07	6.25E-06	ENSG00000212195	17	58631640	58631836	U3
ENST00000391759	107.5950867	2.09854112	0.38078	5.5112	3.56E-08	9.12E-07	ENSG00000105619	19	54107019	54115657	TFPT
ENST00000391826	4.662214162	5.647844874	2.02495	2.7891	0.0052851	0.026438	ENSG00000104951	19	49889653	49896887	IL4I1
ENST00000391854	134.2960152	-1.93127165	0.33659	-5.738	9.59E-09	2.81E-07	ENSG00000168243	1	235547684	235649784	GNG4
ENST00000391946	148.0169511	-1.081391932	0.31401	-3.444	0.0005735	0.0043047	ENSG00000104892	19	45340749	45351519	KLC3
ENST00000392027	999.7860865	2.814691338	0.23764	11.844	2.31E-32	1.67E-29	ENSG00000163283	2	232378750	232382889	ALPP
ENST00000392219	376.2155754	-1.263460738	0.23098	-5.47	4.50E-08	1.12E-06	ENSG00000089327	19	35154734	35169881	FXYD5
ENST00000392318	45.790537	-1.608701374	0.5482	-2.935	0.0033408	0.0182527	ENSG00000128641	2	191245403	191425386	MYO1B
ENST00000392413	5.407962619	5.8616559	1.95628	2.9963	0.0027326	0.0155021	ENSG00000130518	19	18257097	18274452	IQCN
ENST00000392452	737.0669542	-1.670461943	0.20351	-8.208	2.24E-16	2.54E-14	ENSG00000180611	3	192796814	192917856	MB21D2
ENST00000392487	4.872759857	5.712624018	2.00261	2.8526	0.0043365	0.0225272	ENSG00000163352	1	154993585	154994315	LENEP
ENST00000392521	702.0628073	-1.164902491	0.20972	-5.555	2.78E-08	7.31E-07	ENSG00000122966	12	119685790	119877320	CIT
ENST00000392552	45.93531716	1.709622573	0.61263	2.7906	0.0052608	0.0263533	ENSG00000163328	2	174431570	174487029	GPR155
ENST00000392568	54.30038473	-1.24250755	0.46262	-2.686	0.0072351	0.03388	ENSG00000183828	14	105172938	105181312	NUDT14
ENST00000392583	55.67188763	-4.161039803	0.67781	-6.139	8.31E-10	3.03E-08	ENSG00000111335	12	112978518	113011723	OAS2
ENST00000392627	180.4523941	-1.59372857	0.29091	-5.478	4.29E-08	1.08E-06	ENSG00000170412	17	74432087	74447429	GPRC5C
ENST00000392634	495.0642616	1.296666222	0.2238	5.7938	6.88E-09	2.09E-07	ENSG00000203485	14	104689617	104722535	INF2
ENST00000392666	8.795141898	3.413101333	1.29772	2.6301	0.0085364	0.0386726	ENSG00000151704	11	128838019	128867296	KCNJ1
ENST00000392668	353.6260051	1.555665387	0.30653	5.0751	3.87E-07	7.75E-06	ENSG00000134954	11	128458764	128587558	ETS1
ENST00000392692	2925.592516	-1.50935402	0.17282	-8.734	2.47E-18	3.57E-16	ENSG00000114346	3	172750725	172821474	ECT2
ENST00000392790	298.7667588	1.480213738	0.26474	5.5912	2.26E-08	6.05E-07	ENSG00000138161	10	122832157	122845857	CUZD1
ENST00000392793	20.98768003	2.41854209	0.78628	3.0759	0.0020985	0.012506	ENSG00000109927	11	121101242	121191490	TECTA
ENST00000392863	64.61149939	-1.69666657	0.47673	-3.559	0.0003723	0.0029906	ENSG00000111850	6	87322587	87342329	SMIM8
ENST00000392929	13.96296671	3.671783979	1.08337	3.3892	0.0007009	0.0050917	ENSG00000224043	2	134735463	134918670	CCNT2-AS1
ENST00000392951	72.68974161	2.957309501	0.47426	6.2357	4.50E-10	1.72E-08	ENSG00000149591	11	117199369	117207464	TAGLN
ENST00000393073	489.1790777	-1.310978194	0.21884	-5.991	2.09E-09	7.07E-08	ENSG00000214182	13	81690067	81690400	PTMAP5
ENST00000393110	281.8059733	-1.066235297	0.26617	-4.006	6.18E-05	0.0006527	ENSG00000115107	2	119223833	119265652	STEAP3
ENST00000393196	1179.279687	-1.823121823	0.27433	-6.646	3.02E-11	1.50E-09	ENSG00000239672	17	51153558	51162168	NME1
ENST00000393200	139.4875492	-4.121920238	0.41026	-10.05	9.46E-24	2.73E-21	ENSG00000136695	2	113059197	113064744	IL36RN
ENST00000393203	1224.329655	-1.214988442	0.19506	-6.229	4.70E-10	1.79E-08	ENSG00000134247	1	116909915	116990353	PTGFRN
ENST00000393217	6.298573679	-6.130078773	1.89232	-3.239	0.0011976	0.0079107	ENSG00000133640	12	85036350	85245105	LRRIQ1

protein_coding	Yes	NM_005559.4	108
protein_coding	Yes	NM_001394639.1	108
protein_coding	Yes	NM_003628.6	108
protein_coding	Yes	NM_003900.5	108
protein_coding	Yes	NM_015497.5	108
protein_coding	Yes	NM_173628.4	108
protein_coding	Yes	NM_016468.7	108
miRNA	Yes	-	108
protein_coding	Yes	NM_024808.5	108
snoRNA	Yes	-	108
protein_coding	Yes	NM_013342.4	108
protein_coding	Yes	NM_152899.2	108
protein_coding	Yes	NM_001098722.2	108
protein_coding	Yes	NM_177417.3	108
protein_coding	Yes	NM_001632.5	108
protein_coding	Yes	NM_014164.6	108
protein_coding	Yes	NM_001130158.3	108
protein_coding	Yes	NM_001145304.2	108
protein_coding	Yes	NM_178496.4	108
protein_coding	Yes	NM_001394530.1	108
protein_coding	Yes	NM_001206999.2	108
protein_coding	Yes	NM_152529.7	108
protein_coding	Yes	NM_177533.5	108
protein_coding	Yes	NM_002535.3	108
protein_coding	Yes	NM_022036.4	108
protein_coding	Yes	NM_022489.4	108
protein_coding	Yes	NM_153766.3	108
protein_coding	Yes	NM_001143820.2	108
protein_coding	Yes	NM_001258315.2	108
protein_coding	Yes	NM_022034.6	108
protein_coding	Yes	NM_005422.4	108
protein_coding	Yes	NM_001042493.3	108
lncRNA	Yes	-	108
protein_coding	Yes	NM_003186.5	108
ped_processed_pse	Yes	-	108
protein_coding	Yes	NM_182915.3	108
protein_coding	Yes	NM_000269.3	108
protein_coding	Yes	NM_012275.3	108
protein_coding	Yes	NM_020440.4	108
protein_coding	Yes	NM_001079910.2	108

ENST00000393306	182.9546475	3.041033236	0.36806	8.2623	1.43E-16	1.67E-14	ENSG00000140961	16	83953239	83966332	OSGIN1
ENST00000393316	26.3335279	1.757577127	0.69421	2.5318	0.0113495	0.0483586	ENSG00000188761	1	113876815	113887581	BCL2L15
ENST00000393432	6049.329561	-1.038282668	0.16575	-6.264	3.75E-10	1.45E-08	ENSG00000169045	5	179614177	179623671	HNRNPH1
ENST00000393459	449.5972424	1.384345248	0.22859	6.056	1.40E-09	4.91E-08	ENSG00000107819	10	101031233	101041241	SFXN3
ENST00000393565	332.8389321	-2.150693572	0.30185	-7.125	1.04E-12	7.05E-11	ENSG00000113758	5	177456609	177473634	DBN1
ENST00000393567	28.2515968	2.283844753	0.6627	3.4463	0.0005684	0.0042741	ENSG00000157423	16	70802083	71230722	HYDIN
ENST00000393597	117.8741418	-3.342972934	0.39684	-8.424	3.64E-17	4.59E-15	ENSG00000175591	11	73218280	73242427	P2RY2
ENST00000393674	285.8173081	-1.003076202	0.24785	-4.047	5.19E-05	0.0005612	ENSG00000164117	4	174236657	174283667	FBXO8
ENST00000393791	8.299667349	6.480655131	1.79333	3.6138	0.0003018	0.0025071	ENSG00000123329	12	57472268	57479866	ARHGAP9
ENST00000393818	402.6144724	1.085928366	0.22747	4.7739	1.81E-06	3.01E-05	ENSG00000068120	17	42562147	42566277	COASY
ENST00000393868	670.2919763	-1.247796268	0.20404	-6.116	9.62E-10	3.47E-08	ENSG00000163541	2	84423527	84459280	SUCLG1
ENST00000393909	823.604296	-1.911140268	0.19866	-9.62	6.56E-22	1.48E-19	ENSG00000115364	2	75646782	75662206	MRPL19
ENST00000393913	17.48785676	-2.446825077	0.87413	-2.799	0.0051236	0.0257925	ENSG00000115363	2	75492317	75560939	EVA1A
ENST00000393965	371.5217926	1.688964519	0.23465	7.1977	6.13E-13	4.30E-11	ENSG00000135637	2	74472831	74482998	CCDC142
ENST00000394133	4.149833674	5.480651158	2.08437	2.6294	0.0085535	0.0387013	ENSG00000213432	10	93284003	93284558	RPL17P34
ENST00000394166	3519.777338	-1.645399959	0.24088	-6.831	8.45E-12	4.73E-10	ENSG00000185551	15	96330699	96340258	NR2F2
ENST00000394198	804.7035349	-1.225851537	0.20416	-6.004	1.92E-09	6.56E-08	ENSG00000170854	3	97941817	97972431	RIOX2
ENST00000394222	320.5680661	-1.225532594	0.24768	-4.948	7.50E-07	1.38E-05	ENSG00000169379	3	93980154	94055678	ARL13B
ENST00000394308	2123.59581	-1.308974283	0.17615	-7.431	1.08E-13	8.54E-12	ENSG00000070669	7	97851676	97872408	ASNS
ENST00000394323	127.4706221	1.696985872	0.39142	4.3355	1.45E-05	0.0001861	ENSG00000213462	7	64990355	65006687	ERV3-1
ENST00000394334	620.0566792	-1.657354991	0.20802	-7.967	1.62E-15	1.65E-13	ENSG00000134602	X	132023301	132075943	STK26
ENST00000394391	476.1569562	-1.044761128	0.26479	-3.946	7.96E-05	0.0008102	ENSG00000005194	16	57428186	57447385	CIAPIN1
ENST00000394420	515.7032168	-1.078934453	0.21623	-4.99	6.05E-07	1.14E-05	ENSG00000159579	16	57186328	57240469	RSPRY1
ENST00000394485	34.43258154	-7.133563714	1.60664	-4.44	8.99E-06	0.0001227	ENSG00000187193	16	56682469	56684196	MT1X
ENST00000394621	51.3884346	1.800097923	0.53532	3.3626	0.000772	0.0055191	ENSG00000157214	7	90211739	90237683	STEAP2
ENST00000394650	348.5622273	-1.35720999	0.23604	-5.75	8.93E-09	2.64E-07	ENSG00000005059	4	109560245	109688719	MCUB
ENST00000394662	14.20543166	-3.72505629	1.11749	-3.333	0.0008579	0.0060041	ENSG00000225492	1	89410318	89426243	GBP1P1
ENST00000394688	937.7913286	-1.769734206	0.1936	-9.141	6.17E-20	1.07E-17	ENSG00000166164	16	50315956	50368988	BRD7
ENST00000394705	33.26627076	1.572442714	0.60249	2.6099	0.0090568	0.0405408	ENSG00000119737	2	53852911	53859967	GPR75
ENST00000394718	228.3813431	-2.184108863	0.28258	-7.729	1.08E-14	9.87E-13	ENSG00000179841	14	64465498	64474503	AKAP5
ENST00000394725	330.9846195	-1.460006479	0.24162	-6.042	1.52E-09	5.28E-08	ENSG00000196470	16	48360530	48385413	SIAH1
ENST00000394852	590.4669447	1.472579943	0.22194	6.6352	3.24E-11	1.60E-09	ENSG00000140403	15	78264561	78282191	DNAJA4
ENST00000394854	1033.373004	-1.230964377	0.21801	-5.646	1.64E-08	4.55E-07	ENSG00000138814	4	101023417	101347526	PPP3CA
ENST00000394900	222.3257255	-1.271868618	0.26985	-4.713	2.44E-06	3.93E-05	ENSG00000149476	11	61333227	61349242	TKFC
ENST00000394908	323.7292784	-1.646135032	0.24257	-6.786	1.15E-11	6.25E-10	ENSG00000132589	17	28879338	28897733	FLOT2
ENST00000394936	13852.01037	1.07267702	0.16428	6.5297	6.59E-11	3.00E-09	ENSG00000197746	10	71816297	71851251	PSAP
ENST00000394945	26.04410083	-3.458277394	0.79014	-4.377	1.20E-05	0.0001581	ENSG00000152377	5	136975297	137499326	SPOCK1
ENST00000394957	95.05441831	-2.727882563	0.39601	-6.888	5.64E-12	3.28E-10	ENSG00000107738	10	71747555	71773520	VSIR
ENST00000394963	352.4876267	-1.557540135	0.23615	-6.596	4.23E-11	2.03E-09	ENSG00000066117	12	50085341	50100707	SMARCD1
ENST00000394975	128.2984983	-1.137340414	0.35003	-3.249	0.0011569	0.007691	ENSG00000167397	16	31090853	31094797	VKORC1

protein_coding	Yes	NM_182981.3	108
protein_coding	Yes	NM_001010922.3	108
protein_coding	Yes	NM_001257293.2	108
protein_coding	Yes	NM_030971.6	108
protein_coding	Yes	NM_001363541.2	108
protein_coding	Yes	NM_001270974.2	108
protein_coding	Yes	NM_002564.4	108
protein_coding	Yes	NM_012180.3	108
protein_coding	Yes	NM_032496.4	108
protein_coding	Yes	NM_025233.7	108
protein_coding	Yes	NM_003849.4	108
protein_coding	Yes	NM_014763.4	108
protein_coding	Yes	NM_001135032.2	108
protein_coding	Yes	NM_001365575.2	108
ocessed_pseudoge	Yes	-	108
protein_coding	Yes	NM_021005.4	108
protein_coding	Yes	NM_153182.4	108
protein_coding	Yes	NM_001174150.2	108
protein_coding	Yes	NM_001673.5	108
protein_coding	Yes	NM_001007253.4	108
protein_coding	Yes	NM_016542.4	108
protein_coding	Yes	NM_020313.4	108
protein_coding	Yes	NM_133368.3	108
protein_coding	Yes	NM_005952.4	108
protein_coding	Yes	NM_001244944.2	108
protein_coding	Yes	NM_017918.5	108
ed_unprocessed_ps	Yes	-	108
protein_coding	Yes	NM_013263.5	108
protein_coding	Yes	NM_006794.4	108
protein_coding	Yes	NM_004857.3	108
protein_coding	Yes	NM_003031.4	108
protein_coding	Yes	NM_001130182.2	108
protein_coding	Yes	NM_000944.5	108
protein_coding	Yes	NM_015533.4	108
protein_coding	Yes	NM_004475.3	108
protein_coding	Yes	NM_002778.4	108
protein_coding	Yes	NM_004598.4	108
protein_coding	Yes	NM_022153.2	108
protein_coding	Yes	NM_003076.5	108
protein_coding	Yes	NM_024006.6	108

ENST00000395080	83.72570885	-1.930818676	0.50879	-3.795	0.0001477	0.0013681	ENSG00000118785	4	87975713	87983411	SPP1
ENST00000395159	114.3911222	-1.387683273	0.35642	-3.893	9.88E-05	0.0009729	ENSG00000100575	14	58408494	58427531	TIMM9
ENST00000395284	2362.160427	-1.064974571	0.17967	-5.927	3.08E-09	1.01E-07	ENSG00000170312	10	60778477	60794852	CDK1
ENST00000395748	104.7774255	-2.090759289	0.37645	-5.554	2.79E-08	7.34E-07	ENSG00000109321	4	74445135	74455005	AREG
ENST00000395810	71.37428581	-2.237210369	0.44744	-5	5.73E-07	1.09E-05	ENSG00000198915	10	43194534	43267065	RASGEF1A
ENST00000395823	20.89721161	-2.474230978	0.76944	-3.216	0.0013016	0.0084783	ENSG00000213707	22	26560525	26561088	HMGB1P10
ENST00000395841	1508.103148	1.274677046	0.21241	6.001	1.96E-09	6.68E-08	ENSG00000101146	20	57351254	57379202	RAE1
ENST00000395842	107.433125	2.363473958	0.37692	6.2705	3.60E-10	1.40E-08	ENSG00000073150	22	50170730	50180295	PANX2
ENST00000395906	234.8208795	1.623968383	0.26872	6.0434	1.51E-09	5.26E-08	ENSG00000241322	17	15583885	15619704	FBXW10B
ENST00000395925	25.70425077	2.9068011	0.72925	3.986	6.72E-05	0.0007024	ENSG00000106571	7	41960948	42237209	GLI3
ENST00000395930	6.950249308	6.227256985	1.87488	3.3214	0.0008956	0.0062183	ENSG00000125409	17	15303811	15341597	TEKT3
ENST00000396005	47.46306891	-5.229459831	0.84042	-6.222	4.90E-10	1.85E-08	ENSG00000148942	11	26667019	26722060	SLC5A12
ENST00000396077	234.8737322	-1.231505732	0.26773	-4.6	4.23E-06	6.35E-05	ENSG00000185219	3	44431704	44477670	ZNF445
ENST00000396386	3012.230948	-1.038543119	0.17248	-6.021	1.73E-09	5.97E-08	ENSG00000122565	7	26201744	26213607	CBX3
ENST00000396396	22.73630308	5.452809061	1.20986	4.507	6.58E-06	9.33E-05	ENSG00000154330	9	68356610	68531061	PGM5
ENST00000396402	22.14937619	7.897211649	1.58803	4.9729	6.59E-07	1.23E-05	ENSG00000137869	15	51208056	51338596	CYP19A1
ENST00000396551	212.7616288	1.114123453	0.2821	3.9493	7.84E-05	0.0007997	ENSG00000204599	6	30326926	30343729	TRIM39
ENST00000396602	1487.854794	2.027528003	0.19101	10.615	2.55E-26	9.86E-24	ENSG00000166478	11	9461011	9528524	ZNF143
ENST00000396654	152.9299063	-1.945817242	0.31287	-6.219	5.00E-10	1.89E-08	ENSG00000152056	2	223755325	223837582	AP1S3
ENST00000396679	527.2494361	-1.599735838	0.21927	-7.296	2.97E-13	2.22E-11	ENSG00000123219	5	65517765	65563168	CENPK
ENST00000396872	34.61301463	-1.444736499	0.57246	-2.524	0.0116121	0.0492075	ENSG00000164638	7	5282942	5306912	SLC29A4
ENST00000396895	5.550041491	5.90178657	1.9511	3.0249	0.0024876	0.0143535	ENSG00000213931	11	5268344	5269945	HBE1
ENST00000396946	530.153303	-1.239987616	0.21621	-5.735	9.75E-09	2.85E-07	ENSG00000198286	7	2906141	3043867	CARD11
ENST00000396984	3034.452741	-1.346734188	0.17391	-7.744	9.63E-15	8.85E-13	ENSG00000180596	6	26123466	26123926	H2BC4
ENST00000397061	190.8955595	-3.344612656	0.32087	-10.42	1.94E-25	6.67E-23	ENSG00000213965	19	32691820	32713792	NUDT19
ENST00000397066	518.2282856	-1.314123135	0.22853	-5.75	8.91E-09	2.63E-07	ENSG00000162063	16	2429446	2458854	CCNF
ENST00000397098	1079.074878	-1.112745076	0.26711	-4.166	3.10E-05	0.0003587	ENSG00000146540	7	997005	1138247	C7orf50
ENST00000397128	1035.747972	-1.266492629	0.19208	-6.594	4.29E-11	2.05E-09	ENSG00000132356	5	40759388	40798374	PRKAA1
ENST00000397146	179.1656808	-10.9593391	1.47817	-7.414	1.22E-13	9.66E-12	ENSG00000123243	10	7559269	7666966	ITIH5
ENST00000397163	40.19175559	1.922728401	0.57575	3.3395	0.0008392	0.0059049	ENSG00000092529	15	42359500	42412317	CAPN3
ENST00000397166	910.5738	-1.252989296	0.20578	-6.089	1.14E-09	4.05E-08	ENSG00000085788	8	38231584	38262836	DDHD2
ENST00000397298	154.6676698	-1.508379627	0.31	-4.866	1.14E-06	2.00E-05	ENSG00000214026	11	1947331	1956600	MRPL23
ENST00000397397	308.7123152	1.250441385	0.24446	5.1151	3.14E-07	6.45E-06	ENSG00000214063	11	842851	867111	TSPAN4
ENST00000397510	263.4010582	1.457535558	0.25862	5.6358	1.74E-08	4.81E-07	ENSG00000177042	11	695815	704129	TMEM80
ENST00000397536	293.5554843	2.012885411	0.27234	7.391	1.46E-13	1.14E-11	ENSG00000188549	15	40331451	40340939	CCDC9B
ENST00000397560	1204.790828	2.877584554	0.20297	14.177	1.26E-45	3.64E-42	ENSG00000006459	7	140084745	140176983	KDM7A
ENST00000397572	410.2444941	-1.720203476	0.23692	-7.261	3.85E-13	2.82E-11	ENSG00000214114	1	38862492	38873348	MYCBP
ENST00000397627	628.5037161	-1.374486037	0.2198	-6.253	4.02E-10	1.55E-08	ENSG00000197744	5	118973795	118974122	PTMAP2
ENST00000397764	129.2876706	-1.52894317	0.33736	-4.532	5.84E-06	8.42E-05	ENSG00000214194	7	113116717	113118554	SMIM30
ENST00000397766	206.318311	1.829458997	0.28618	6.3927	1.63E-10	6.82E-09	ENSG00000182405	15	34102082	34108686	PGBD4

protein_coding	Yes	NM_001040058.2	108
protein_coding	Yes	NM_012460.4	108
protein_coding	Yes	NM_001786.5	108
protein_coding	Yes	NM_001657.4	108
protein_coding	Yes	NM_145313.4	108
ocessed_pseudoge	Yes	-	108
protein_coding	Yes	NM_003610.4	108
protein_coding	Yes	NM_052839.4	108
protein_coding	Yes	NM_006382.4	108
protein_coding	Yes	NM_000168.6	108
protein_coding	Yes	NM_031898.3	108
protein_coding	Yes	NM_178498.4	108
protein_coding	Yes	NM_181489.6	108
protein_coding	Yes	NM_016587.4	108
protein_coding	Yes	NM_021965.4	108
protein_coding	Yes	NM_000103.4	108
protein_coding	Yes	NM_001369521.2	108
protein_coding	Yes	NM_003442.6	108
protein_coding	Yes	NM_001039569.2	108
protein_coding	Yes	NM_022145.5	108
protein_coding	Yes	NM_153247.4	108
protein_coding	Yes	NM_005330.4	108
protein_coding	Yes	NM_032415.7	108
protein_coding	Yes	NM_003526.3	108
protein_coding	Yes	NM_001105570.2	108
protein_coding	Yes	NM_001761.3	108
protein_coding	Yes	NM_001318252.2	108
protein_coding	Yes	NM_006251.6	108
protein_coding	Yes	NM_030569.7	108
protein_coding	Yes	NM_000070.3	108
protein_coding	Yes	NM_015214.3	108
protein_coding	Yes	NM_021134.4	108
protein_coding	Yes	NM_003271.5	108
protein_coding	Yes	NM_001042463.3	108
protein_coding	Yes	NM_207380.3	108
protein_coding	Yes	NM_030647.2	108
protein_coding	Yes	NM_012333.5	108
ocessed_pseudoge	Yes	-	108
protein_coding	Yes	NM_001352688.2	108
protein_coding	Yes	NM_152595.5	108

ENST00000397820	55.50880782	4.247409562	0.6103	6.9595	3.41E-12	2.07E-10	ENSG00000214212	19	10848414	10869790	C19orf38
ENST00000397942	1780.266864	-1.317527296	0.19344	-6.811	9.69E-12	5.36E-10	ENSG00000170677	18	70289044	70330199	SOCS6
ENST00000397944	30.25406945	6.888298054	1.55516	4.4293	9.45E-06	0.000128	ENSG00000118492	6	146598971	146815462	ADGB
ENST00000397985	45.13061342	1.450066561	0.51361	2.8233	0.0047533	0.024259	ENSG00000166401	18	63970080	63989374	SERPINB8
ENST00000398146	77.28192127	1.083670131	0.39685	2.7307	0.0063203	0.0304633	ENSG00000185304	2	87755959	87825796	RGPD2
ENST00000398297	10.88953729	6.87373648	1.72265	3.9902	6.60E-05	0.0006919	ENSG00000215120	X	71527813	71530225	SOCS6P1
ENST00000398357	348.8316086	-1.360858893	0.24086	-5.65	1.60E-08	4.47E-07	ENSG00000214455	13	45390352	45391267	RCN1P2
ENST00000398389	86.51818114	1.173582505	0.3828	3.0658	0.0021708	0.0128629	ENSG00000161647	17	43800810	43833146	MPP3
ENST00000398458	10.95800411	6.881875989	1.7164	4.0095	6.09E-05	0.0006444	ENSG00000186452	12	50842925	50887884	TMPRSS12
ENST00000398516	22.01531755	2.2584718	0.75078	3.0082	0.0026283	0.0150206	ENSG00000145217	4	987656	993404	SLC26A1
ENST00000398540	411.7216315	-1.421470263	0.22728	-6.254	4.00E-10	1.54E-08	ENSG00000114541	3	69168781	69386088	FRMD4B
ENST00000398631	665.6758456	1.2646254	0.21064	6.0037	1.93E-09	6.58E-08	ENSG00000251664	5	140875307	141012347	PCDHA12
ENST00000398806	83.10454714	-1.122321451	0.38781	-2.894	0.0038038	0.0202519	ENSG00000056998	X	2828929	2882818	GYG2
ENST00000398841	350.2363209	1.827906801	0.33041	5.5322	3.16E-08	8.19E-07	ENSG00000090238	16	30092313	30096213	YPEL3
ENST00000398882	788.7444863	1.479113887	0.23159	6.3867	1.70E-10	7.06E-09	ENSG00000204839	8	143566191	143572772	MROH6
ENST00000398992	9.278783775	6.64149485	1.75999	3.7736	0.0001609	0.0014747	ENSG00000214788	11	59942878	59970146	-
ENST00000399002	85.40332656	-3.463408374	0.45382	-7.632	2.32E-14	2.03E-12	ENSG00000172296	20	13008971	13169103	SPTLC3
ENST00000399069	384.0787492	1.017038242	0.22856	4.4497	8.60E-06	0.000118	ENSG00000205629	16	25111741	25178228	LCMT1
ENST00000399220	9.745519714	6.71262581	1.74667	3.8431	0.0001215	0.0011578	ENSG00000168329	3	39263495	39280036	CX3CR1
ENST00000399312	417.8788703	1.078237731	0.27909	3.8634	0.0001118	0.0010775	ENSG00000243927	21	34073577	34143030	MRPS6
ENST00000399506	105.131643	2.929629577	0.41692	7.0268	2.11E-12	1.35E-10	ENSG00000132535	17	7187186	7217627	DLG4
ENST00000399808	382.7257887	-1.006338631	0.25902	-3.885	0.0001022	0.0009999	ENSG00000142089	11	319675	320860	IFITM3
ENST00000399815	42.35447757	-3.196225885	0.62247	-5.135	2.82E-07	5.88E-06	ENSG00000288681	11	308407	315272	-
ENST00000399878	1273.353754	1.461556115	0.22213	6.5797	4.71E-11	2.23E-09	ENSG00000091490	4	25747432	25862988	SEL1L3
ENST00000400181	2014.39232	-1.022568598	0.1859	-5.501	3.78E-08	9.61E-07	ENSG00000004487	1	23019467	23083689	KDM1A
ENST00000400324	617.4407726	-1.357308159	0.20774	-6.534	6.41E-11	2.93E-09	ENSG00000139734	13	59665582	60163928	DIAPH3
ENST00000400382	23.19431656	1.925194945	0.72781	2.6452	0.0081643	0.0373287	ENSG00000100031	22	24603192	24628996	GGT1
ENST00000400522	11.04827647	2.957517909	1.09751	2.6948	0.007044	0.0331934	ENSG00000215529	20	32858922	32961845	EFCAB8
ENST00000400577	11.00071242	-5.449717958	1.72419	-3.161	0.0015736	0.0098938	ENSG00000185272	21	14216156	14228372	RBM11
ENST00000400590	3596.926652	-1.108841577	0.18117	-6.12	9.34E-10	3.38E-08	ENSG00000180776	13	21372570	21459303	ZDHHC20
ENST00000400999	10.27970574	2.840366073	1.1217	2.5322	0.0113351	0.0483093	ENSG00000143450	1	151766654	151771334	OAZ3
ENST00000401061	67.80671879	-1.983650188	0.435	-4.56	5.11E-06	7.51E-05	ENSG00000132763	1	45500299	45513382	MMACHC
ENST00000401408	67.31378413	-1.250415015	0.4748	-2.634	0.0084496	0.0383487	ENSG00000162994	2	55172546	55232293	CLHC1
ENST00000401827	626.070227	-1.418685903	0.21124	-6.716	1.87E-11	9.70E-10	ENSG00000205268	8	65714333	65842064	PDE7A
ENST00000402119	8.607581718	3.988140193	1.45586	2.7394	0.0061556	0.0298241	ENSG00000171903	19	15912376	15934529	CYP4F11
ENST00000402142	13.19524948	7.149399567	1.67362	4.2718	1.94E-05	0.0002386	ENSG00000100346	22	39570752	39689735	CACNA1I
ENST00000402743	33.08384985	-2.369219359	0.61579	-3.847	0.0001194	0.0011406	ENSG00000233514	1	44122152	44122512	-
ENST00000402802	314.4468903	-1.060639796	0.3046	-3.482	0.0004976	0.0038216	ENSG00000197461	7	497257	519846	PDGFA
ENST00000402868	747.2850269	-1.028240284	0.20458	-5.026	5.01E-07	9.73E-06	ENSG00000183955	12	123384131	123409353	KMT5A
ENST00000402938	113.6601232	2.757315025	0.37083	7.4355	1.04E-13	8.28E-12	ENSG00000111886	6	89254463	89315299	GABRR2

protein_coding	Yes	NM_001136482.3	108
protein_coding	Yes	NM_004232.4	108
protein_coding	Yes	NM_024694.4	108
protein_coding	Yes	NM_002640.4	108
protein_coding	Yes	NM_001078170.3	108
ocessed_pseudoge	Yes	-	108
ocessed_pseudoge	Yes	-	108
protein_coding	Yes	NM_001932.6	108
protein_coding	Yes	NM_182559.3	108
protein_coding	Yes	NM_022042.4	108
protein_coding	Yes	NM_015123.3	108
protein_coding	Yes	NM_018903.4	108
protein_coding	Yes	NM_001079855.2	108
protein_coding	Yes	NM_031477.5	108
protein_coding	Yes	NM_001100878.2	108
lncRNA	Yes	-	108
protein_coding	Yes	NM_018327.4	108
protein_coding	Yes	NM_016309.3	108
protein_coding	Yes	NM_001337.4	108
protein_coding	Yes	NM_032476.4	108
protein_coding	Yes	NM_001321075.3	108
protein_coding	Yes	NM_021034.3	108
protein_coding	Yes	-	108
protein_coding	Yes	NM_015187.5	108
protein_coding	Yes	NM_001009999.3	108
protein_coding	Yes	NM_001042517.2	108
protein_coding	Yes	NM_001288833.2	108
protein_coding	Yes	NM_001143967.2	108
protein_coding	Yes	NM_144770.5	108
protein_coding	Yes	NM_001330059.2	108
protein_coding	Yes	-	108
protein_coding	Yes	NM_015506.3	108
protein_coding	Yes	NM_152385.4	108
protein_coding	Yes	NM_001242318.3	108
protein_coding	Yes	NM_021187.4	108
protein_coding	Yes	NM_021096.4	108
ocessed_pseudoge	Yes	-	108
protein_coding	Yes	NM_033023.5	108
protein_coding	Yes	NM_020382.7	108
protein_coding	Yes	NM_002043.5	108

ENST00000403590	4.451668466	5.580042745	2.05553	2.7147	0.0066346	0.0316755	ENSG00000218226	6	158609705	158621636	TATDN2P2
ENST00000403683	385.3529724	-1.453545195	0.23355	-6.224	4.85E-10	1.84E-08	ENSG00000203852	1	149852607	149853125	H3C15
ENST00000403687	329.2730623	1.564053385	0.24439	6.3998	1.56E-10	6.54E-09	ENSG00000152689	2	33476648	33564731	RASGRP3
ENST00000403888	19.04996949	-2.331575667	0.79633	-2.928	0.0034127	0.0185736	ENSG00000100379	22	37051741	37063390	KCTD17
ENST00000403903	78.25772541	1.323656748	0.41764	3.1694	0.0015275	0.0096596	ENSG00000220201	19	10304802	10309880	ZGLP1
ENST00000404077	41.94450712	2.359132333	0.55957	4.216	2.49E-05	0.000296	ENSG00000169402	7	6754102	6798765	RSPH10B2
ENST00000404327	85.12483032	-1.332223501	0.38377	-3.471	0.0005178	0.0039505	ENSG00000218416	2	240449417	240456714	GPC1-AS1
ENST00000404406	26.05569536	2.253168101	0.70483	3.1968	0.0013898	0.0089437	ENSG00000155026	7	5926135	5970689	RSPH10B
ENST00000404589	33.68658508	-2.060734007	0.62198	-3.313	0.0009224	0.006369	ENSG00000168301	3	58492095	58502356	KCTD6
ENST00000404816	345.2247118	-1.36774733	0.23654	-5.782	7.37E-09	2.23E-07	ENSG00000049323	2	32946952	33399509	LTBP1
ENST00000404826	4.289796681	-5.575720003	2.06784	-2.696	0.0070093	0.0330813	ENSG00000146555	7	3301251	4269000	SDK1
ENST00000404933	1881.545049	-1.229950575	0.1965	-6.259	3.86E-10	1.49E-08	ENSG00000102172	X	21940708	21994837	SMS
ENST00000405166	8.976948983	6.594725308	1.77224	3.7211	0.0001983	0.0017608	ENSG00000218803	6	111046867	111047521	GSTM2P1
ENST00000405633	8.322489623	6.484210624	1.79271	3.617	0.0002981	0.0024823	ENSG00000166961	11	60756866	60776733	MS4A15
ENST00000405876	105.4686873	-2.634370102	0.37763	-6.976	3.04E-12	1.86E-10	ENSG00000139437	12	109900273	109918069	TCHP
ENST00000406189	609.3311036	-1.073323093	0.20624	-5.204	1.95E-07	4.20E-06	ENSG00000176624	18	51174549	51197681	MEX3C
ENST00000406217	53.57397382	1.250165834	0.47432	2.6357	0.0083962	0.0381797	ENSG00000179988	10	122980400	122990390	PSTK
ENST00000406246	549.4559594	1.199272812	0.21532	5.5698	2.55E-08	6.76E-07	ENSG00000173039	11	65653600	65662916	RELA
ENST00000406427	30.77166713	3.487032975	0.71855	4.8529	1.22E-06	2.12E-05	ENSG00000130653	9	137459951	137550402	PNPLA7
ENST00000406438	1368.694811	1.555675934	0.18372	8.4678	2.50E-17	3.22E-15	ENSG00000176994	17	18315292	18328056	SMCR8
ENST00000406489	34.66132445	1.493429108	0.57282	2.6072	0.0091297	0.0407944	ENSG00000219891	6	28092426	28092845	ZSCAN12P1
ENST00000406610	4.263771377	-5.567856417	2.07953	-2.677	0.0074183	0.0345221	ENSG00000116117	2	204545474	205620162	PARD3B
ENST00000406875	148.1103734	-3.380761013	0.37893	-8.922	4.58E-19	7.28E-17	ENSG00000064393	7	139561569	139777998	HIPK2
ENST00000407010	184.9985776	-1.396440544	0.29969	-4.66	3.17E-06	4.93E-05	ENSG00000214960	7	16087524	16421538	CRPPA
ENST00000407071	52.57627574	2.809677324	0.51875	5.4163	6.09E-08	1.48E-06	ENSG00000162849	1	245154984	245709432	KIF26B
ENST00000407693	239.8305092	-2.120151563	0.27807	-7.624	2.45E-14	2.13E-12	ENSG00000106853	9	111562566	111599647	PTGR1
ENST00000407997	1549.358107	2.767095042	0.92285	2.9984	0.0027139	0.0154153	ENSG00000239713	22	39077274	39087743	APOBEC3G
ENST00000408593	3.916465705	5.396742222	2.11677	2.5495	0.0107872	0.0464746	ENSG00000221520	7	92204014	92204098	MIR1285-1
ENST00000408903	54.60496596	-2.108796717	0.48553	-4.343	1.40E-05	0.0001807	ENSG00000171444	5	113022105	113488453	MCC
ENST00000408936	617.8510464	1.399078459	0.21535	6.4967	8.21E-11	3.66E-09	ENSG00000136848	9	121651633	121785530	DAB2IP
ENST00000408954	317.6824434	-2.359178626	0.2825	-8.351	6.77E-17	8.21E-15	ENSG00000196730	9	87497866	87708634	DAPK1
ENST00000408968	86.0166115	-3.643478343	0.48941	-7.445	9.72E-14	7.77E-12	ENSG00000185885	11	314039	315272	IFITM1
ENST00000409110	69.05008274	-9.584934579	1.50422	-6.372	1.87E-10	7.70E-09	ENSG00000130561	2	233307815	233347055	SAG
ENST00000409115	13218.37275	-1.258692885	0.16759	-7.511	5.88E-14	4.89E-12	ENSG00000187514	2	231708524	231713551	PTMA
ENST00000409150	120.6445846	-1.538533316	0.34212	-4.497	6.89E-06	9.74E-05	ENSG00000189362	2	190504337	190534722	NEMP2
ENST00000409175	2806.00564	-1.103510833	0.18613	-5.929	3.06E-09	9.99E-08	ENSG00000136536	2	159712505	159771027	MARCHF7
ENST00000409235	68.37732638	2.269252555	0.51956	4.3677	1.26E-05	0.0001641	ENSG00000099338	19	38335829	38370943	CATSPERG
ENST00000409318	231.919004	-1.254034395	0.27625	-4.54	5.64E-06	8.17E-05	ENSG00000204634	2	101007227	101151382	TBC1D8
ENST00000409359	319.8410138	-1.116180322	0.24485	-4.559	5.15E-06	7.55E-05	ENSG00000136002	2	130836913	131047253	ARHGEF4
ENST00000409548	4058.971306	4.941805193	0.20545	24.054	7.61E-128	2.91E-123	ENSG00000142102	11	289125	296107	PGGHG

ocessed_pseudoge	Yes	-	108
protein_coding	Yes	NM_001005464.3	108
protein_coding	Yes	NM_001139488.2	108
protein_coding	Yes	NM_001282684.2	108
protein_coding	Yes	-	108
protein_coding	Yes	NM_001099697.2	108
lncRNA	Yes	-	108
protein_coding	Yes	NM_173565.5	108
protein_coding	Yes	NM_001128214.2	108
protein_coding	Yes	NM_206943.4	108
protein_coding	Yes	NM_152744.4	108
protein_coding	Yes	NM_004595.5	108
ocessed_pseudoge	Yes	-	108
protein_coding	Yes	NM_001098835.2	108
protein_coding	Yes	NM_001143852.2	108
protein_coding	Yes	NM_016626.5	108
protein_coding	Yes	NM_001363531.2	108
protein_coding	Yes	NM_021975.4	108
protein_coding	Yes	NM_001098537.3	108
protein_coding	Yes	NM_144775.3	108
ed_unprocessed_ps	Yes	-	108
protein_coding	Yes	NM_001302769.2	108
protein_coding	Yes	NM_022740.5	108
protein_coding	Yes	NM_001101426.4	108
protein_coding	Yes	NM_018012.4	108
protein_coding	Yes	NM_001146108.2	108
protein_coding	Yes	NM_021822.4	108
miRNA	Yes	-	108
protein_coding	Yes	NM_001085377.2	108
protein_coding	Yes	NM_001395010.1	108
protein_coding	Yes	NM_004938.4	108
protein_coding	Yes	NM_003641.5	108
protein_coding	Yes	NM_000541.5	108
protein_coding	Yes	NM_002823.5	108
protein_coding	Yes	NM_001142645.2	108
protein_coding	Yes	NM_001282805.2	108
protein_coding	Yes	NM_021185.5	108
protein_coding	Yes	NM_001330348.2	108
protein_coding	Yes	NM_001367493.1	108
protein_coding	Yes	NM_025092.5	108

ENST00000409600	3430.900679	-1.008397737	0.18591	-5.424	5.83E-08	1.42E-06	ENSG00000082153	2	200811909	200827338	BZW1
ENST00000409644	616.2916668	1.339644265	0.20657	6.485	8.87E-11	3.94E-09	ENSG00000167716	17	1724703	1738585	WDR81
ENST00000409687	1781.014442	-1.755321893	0.23367	-7.512	5.82E-14	4.84E-12	ENSG00000186193	9	137062126	137070557	SAPCD2
ENST00000409746	687.8850391	1.222268347	0.23062	5.3	1.16E-07	2.62E-06	ENSG00000152229	18	45983535	46072260	PSTPIP2
ENST00000409753	1636.839908	-1.110776137	0.22713	-4.89	1.01E-06	1.79E-05	ENSG00000189159	17	75135247	75154512	JPT1
ENST00000409806	5.874698558	5.981707796	1.91881	3.1174	0.0018245	0.0111548	ENSG00000178199	6	149446794	149485014	ZC3H12D
ENST00000409871	137.586353	-1.162590315	0.3593	-3.236	0.0012135	0.0080007	ENSG00000188177	2	112275596	112340059	ZC3H6
ENST00000409878	52.94199228	1.861331213	0.51556	3.6103	0.0003059	0.0025345	ENSG00000213901	2	219161464	219170029	SLC23A3
ENST00000410023	79.71833508	-3.470852319	0.46166	-7.518	5.56E-14	4.63E-12	ENSG00000115594	2	102142745	102179874	IL1R1
ENST00000410062	270.1901167	-1.162913307	0.28121	-4.135	3.54E-05	0.0004034	ENSG00000170035	2	180980604	181063425	UBE2E3
ENST00000410067	131.6144157	-1.653943134	0.32783	-5.045	4.53E-07	8.92E-06	ENSG00000197223	2	68041129	68063004	C1D
ENST00000410792	14.39976167	-3.409058792	1.03395	-3.297	0.0009769	0.0066807	ENSG00000222724	2	88016353	88016547	RNU2-63P
ENST00000412037	3.916465705	5.396742222	2.11677	2.5495	0.0107872	0.0464746	ENSG00000236794	22	23179917	23180172	BCRP8
ENST00000412232	17.66175759	-5.135000295	1.24887	-4.112	3.93E-05	0.0004417	ENSG00000020181	8	37796882	37844896	ADGRA2
ENST00000412298	35.6108968	-1.500656148	0.5654	-2.654	0.0079509	0.0365167	ENSG00000230091	10	80046859	80078895	TMEM254-AS1
ENST00000412916	969.4428018	-1.665095711	0.19433	-8.568	1.05E-17	1.43E-15	ENSG00000067955	16	67029148	67101058	CBFB
ENST00000412976	95.05199363	-1.589370638	0.40627	-3.912	9.15E-05	0.0009112	ENSG00000235489	10	64168958	64170850	DBF4P1
ENST00000413027	202.7570131	-1.247826015	0.27635	-4.515	6.32E-06	9.00E-05	ENSG00000227939	6	31280316	31281519	RPL3P2
ENST00000413053	11.74939712	6.981713195	1.69945	4.1082	3.99E-05	0.0004476	ENSG00000229719	11	64889559	64893449	MIR194-2HG
ENST00000413139	28.85798	-3.425470898	0.74022	-4.628	3.70E-06	5.64E-05	ENSG00000008277	7	87934250	88202889	ADAM22
ENST00000413148	66.01576912	1.315256561	0.42712	3.0793	0.0020746	0.0123918	ENSG00000241326	1	9983140	9984568	-
ENST00000414150	247.1494891	-1.188927746	0.26759	-4.443	8.87E-06	0.0001213	ENSG00000271723	1	54641799	54742231	MROH7-TTC4
ENST00000414634	6.318612222	6.08786734	1.89057	3.2201	0.0012814	0.0083693	ENSG00000236508	3	193307243	193314362	ATP13A5-AS1
ENST00000414645	1725.051879	-1.190963905	0.20272	-5.875	4.23E-09	1.34E-07	ENSG00000156162	8	94719899	94793836	DPY19L4
ENST00000414656	31.31116008	1.837194004	0.6173	2.9762	0.0029188	0.0163274	ENSG00000228544	9	136803926	136808848	CCDC183-AS1
ENST00000414799	4.451668466	5.580042745	2.05553	2.7147	0.0066346	0.0316755	ENSG00000227113	7	65075022	65078780	-
ENST00000415083	924.3695384	-1.097957621	0.20493	-5.358	8.43E-08	1.98E-06	ENSG00000141380	18	26016252	26090613	SS18
ENST00000415187	88.30351382	-1.68476304	0.38473	-4.379	1.19E-05	0.0001567	ENSG00000230043	20	50840614	50840749	TMSB4XP6
ENST00000415278	72.21325451	1.266457081	0.41006	3.0884	0.002012	0.0120777	ENSG00000228502	1	96446929	96448318	EEF1A1P11
ENST00000415550	49.84226665	-1.482711549	0.49728	-2.982	0.0028672	0.0160977	ENSG00000164002	1	40508766	40516038	EXO5
ENST00000416167	59.17772844	-5.067400544	0.68998	-7.344	2.07E-13	1.58E-11	ENSG00000167244	11	2129116	2139389	IGF2
ENST00000416247	473.5908939	-1.402592988	0.22125	-6.339	2.31E-10	9.34E-09	ENSG00000224531	6	11093833	11138733	SMIM13
ENST00000416575	14.94293664	7.328481766	1.64951	4.4428	8.88E-06	0.0001214	ENSG00000227210	2	19990210	20004795	WDR35-DT
ENST00000416599	41.97453374	-1.81306596	0.5357	-3.384	0.0007131	0.0051633	ENSG00000291010	13	52167708	52194465	MRPS31P5
ENST00000416617	46.90271017	1.594912799	0.5106	3.1236	0.0017866	0.0109724	ENSG00000166578	12	113195445	113221094	IQCD
ENST00000416841	203.7589198	1.64255533	0.29545	5.5596	2.70E-08	7.13E-07	ENSG00000179304	X	52897329	52908560	FAM156B
ENST00000417410	129.7019906	-1.729372942	0.33105	-5.224	1.75E-07	3.82E-06	ENSG00000176209	8	42541584	42555195	SMIM19
ENST00000417550	199.6787007	-1.123767426	0.28034	-4.009	6.11E-05	0.0006465	ENSG00000196150	8	144876954	144901453	ZNF250
ENST00000417626	326.2949392	-1.200084233	0.27771	-4.321	1.55E-05	0.0001969	ENSG00000214706	3	50287731	50292429	IFRD2
ENST00000417765	128.3919143	-1.216544329	0.326	-3.732	0.0001902	0.0016983	ENSG00000240963	1	244375099	244409592	-

protein_coding	Yes	NM_001207067.2	108
protein_coding	Yes	NM_001163809.2	108
protein_coding	Yes	NM_178448.4	108
protein_coding	Yes	NM_024430.4	108
protein_coding	Yes	NM_016185.4	108
protein_coding	Yes	NM_207360.3	108
protein_coding	Yes	NM_198581.3	108
protein_coding	Yes	NM_001144889.2	108
protein_coding	Yes	NM_000877.4	108
protein_coding	Yes	NM_006357.4	108
protein_coding	Yes	NM_173177.3	108
snRNA	Yes	-	108
ocessed_pseudoge	Yes	-	108
protein_coding	Yes	NM_032777.10	108
lncRNA	Yes	-	108
protein_coding	Yes	NM_022845.3	108
ocessed_pseudoge	Yes	-	108
ocessed_pseudoge	Yes	-	108
lncRNA	Yes	-	108
protein_coding	Yes	NM_001324418.2	108
lncRNA	Yes	-	108
protein_coding	Yes	-	108
lncRNA	Yes	-	108
protein_coding	Yes	NM_181787.3	108
lncRNA	Yes	-	108
rocessed_pseudog	Yes	-	108
protein_coding	Yes	NM_001007559.3	108
ocessed_pseudoge	Yes	-	108
ocessed_pseudoge	Yes	-	108
protein_coding	Yes	NM_001346953.2	108
protein_coding	Yes	NM_000612.6	108
protein_coding	Yes	NM_001135575.2	108
lncRNA	Yes	-	108
lncRNA	Yes	-	108
protein_coding	Yes	NM_001330452.2	108
protein_coding	Yes	NM_001321178.3	108
protein_coding	Yes	NM_001135674.2	108
protein_coding	Yes	NM_001109689.4	108
protein_coding	Yes	NM_006764.5	108
lncRNA	Yes	-	108

ENST00000417927	65.69473285	1.637641136	0.4452	3.6785	0.0002346	0.0020321	ENSG00000227145	4	122618982	122689156	IL21-AS1
ENST00000418273	328.7813222	2.003015646	0.26258	7.6283	2.38E-14	2.07E-12	ENSG00000272734	10	86965739	86971311	ADIRF-AS1
ENST00000418539	178.0409641	-1.697621887	0.35356	-4.802	1.57E-06	2.67E-05	ENSG00000236824	2	47335314	47335514	BCYRN1
ENST00000418927	39.09863859	2.075890367	0.62797	3.3057	0.0009473	0.006515	ENSG00000235269	14	53685138	53850882	LINC02331
ENST00000419025	22.1344933	3.130468606	0.80813	3.8737	0.0001072	0.0010391	ENSG00000223529	3	184026368	184027756	EEF1A1P8
ENST00000419089	34.2302978	-2.953889376	0.63622	-4.643	3.44E-06	5.28E-05	ENSG00000225071	X	24429572	24429920	RPS26P58
ENST00000419457	91.07635886	-1.962947382	0.38337	-5.12	3.05E-07	6.30E-06	ENSG00000233588	13	28722385	28724021	CYP51A1P2
ENST00000420022	31.17118665	1.900402873	0.62034	3.0635	0.0021878	0.0129494	ENSG00000224420	19	49689593	49690575	ADM5
ENST00000420195	123.1918823	1.248751277	0.4483	2.7855	0.0053442	0.0266774	ENSG00000224660	3	15254183	15264493	SH3BP5-AS1
ENST00000420323	59.76123317	2.076051395	0.52327	3.9674	7.27E-05	0.0007506	ENSG00000114841	3	52316318	52400492	DNAH1
ENST00000420330	42.85815142	1.900996811	0.54481	3.4893	0.0004844	0.003738	ENSG00000236255	2	117833936	117841658	-
ENST00000420470	4.94122668	5.730713439	2.00267	2.8615	0.0042159	0.0220395	ENSG00000236699	4	105552619	105680914	ARHGEF38
ENST00000420638	51.3188162	1.437632299	0.48787	2.9467	0.0032115	0.0176591	ENSG00000223875	22	15852989	15893942	NBEAP3
ENST00000420658	221.4395124	-1.413916148	0.27162	-5.206	1.93E-07	4.17E-06	ENSG00000196526	4	7758713	7939861	AFAP1
ENST00000420696	262.9941809	-1.071095721	0.32167	-3.33	0.0008691	0.0060708	ENSG00000185630	1	164559183	164851831	PBX1
ENST00000420713	144.6765155	-1.012280035	0.31569	-3.207	0.0013435	0.0086945	ENSG00000182111	7	57450176	57473559	ZNF716
ENST00000420843	3327.883216	1.182928224	0.16997	6.9596	3.41E-12	2.07E-10	ENSG00000137486	11	75260121	75351661	ARRB1
ENST00000421553	93.76889398	-2.550996684	0.39299	-6.491	8.51E-11	3.79E-09	ENSG00000197062	6	28267142	28278204	ZSCAN26
ENST00000421828	72.31942803	1.859660221	0.4258	4.3674	1.26E-05	0.0001642	ENSG00000187186	9	34664162	34666045	-
ENST00000421865	186.5044707	-3.344041518	0.39433	-8.48	2.24E-17	2.92E-15	ENSG00000196569	6	128883137	129516566	LAMA2
ENST00000421999	339.5161168	-2.268668266	0.28925	-7.843	4.39E-15	4.26E-13	ENSG00000184220	3	99817861	100181732	CMSS1
ENST00000422048	14.27679742	2.390581788	0.92915	2.5729	0.0100856	0.0441297	ENSG00000228933	X	27174919	27398997	-
ENST00000422848	5.965987654	6.001782396	1.9297	3.1102	0.0018695	0.0113751	ENSG00000231970	10	97401114	97419524	-
ENST00000423158	4.616569613	5.635108028	2.03024	2.7756	0.0055102	0.0273019	ENSG00000185652	12	5432107	5495299	NTF3
ENST00000423313	144.5722675	-1.410150176	0.31272	-4.509	6.51E-06	9.24E-05	ENSG00000235750	1	175156985	175192776	KIAA0040
ENST00000423485	11163.19161	-1.320671101	0.16388	-8.059	7.71E-16	8.23E-14	ENSG00000131747	17	40388524	40417896	TOP2A
ENST00000424120	68.75012444	-6.538894252	0.9252	-7.068	1.58E-12	1.03E-10	ENSG00000048342	4	15469881	15601552	CC2D2A
ENST00000424177	5.851876283	5.976669435	1.92036	3.1123	0.0018565	0.0113116	ENSG00000231193	9	32840597	32841762	-
ENST00000424296	70.59452432	1.46495215	0.43085	3.4001	0.0006735	0.0049236	ENSG00000155085	6	109492854	109691202	AK9
ENST00000424460	50.10664663	-2.279820767	0.5103	-4.468	7.91E-06	0.0001098	ENSG00000229970	7	8262263	8344516	-
ENST00000424848	2684.708676	-1.629043532	0.17419	-9.352	8.61E-21	1.69E-18	ENSG00000105810	7	92604920	92836573	CDK6
ENST00000425159	107.7027863	1.448675675	0.35913	4.0339	5.49E-05	0.0005884	ENSG00000291120	17	47450567	47492493	MRPL45P2
ENST00000425270	546.1498814	-1.346350244	0.22413	-6.007	1.89E-09	6.45E-08	ENSG00000213080	1	160894979	160896076	-
ENST00000425346	102.0247626	1.596274024	0.39374	4.0542	5.03E-05	0.0005467	ENSG00000114395	3	50350861	50354069	CYB561D2
ENST00000425597	108.3617242	2.172207825	0.36626	5.9308	3.02E-09	9.87E-08	ENSG00000198794	15	74995562	75021495	SCAMP5
ENST00000425699	925.9799743	1.989946112	0.20136	9.8823	4.97E-23	1.36E-20	ENSG00000188613	10	119029713	119033730	NANOS1
ENST00000425953	122.4345404	-1.654759293	0.39115	-4.23	2.33E-05	0.0002799	ENSG00000233757	2	95207520	95223839	-
ENST00000425988	3.916465705	5.396742222	2.11677	2.5495	0.0107872	0.0464746	ENSG00000226507	13	22837025	22837880	IPMKP1
ENST00000426263	8556.682184	-1.205809296	0.17568	-6.864	6.70E-12	3.84E-10	ENSG00000117394	1	42925352	42958868	SLC2A1
ENST00000426361	1007.440032	-1.081078898	0.19016	-5.685	1.31E-08	3.73E-07	ENSG00000291080	8	144973588	145002895	ZNF252P

lncRNA	Yes	-	108
lncRNA	Yes	-	108
scRNA	Yes	-	108
lncRNA	Yes	-	108
ocessed_pseudoge	Yes	-	108
ocessed_pseudoge	Yes	-	108
ocessed_pseudoge	Yes	-	108
protein_coding	Yes	NM_001101340.2	108
lncRNA	Yes	-	108
protein_coding	Yes	NM_015512.5	108
lncRNA	Yes	-	108
protein_coding	Yes	NM_001242729.2	108
rocessed_pseudog	Yes	-	108
protein_coding	Yes	NM_001134647.2	108
protein_coding	Yes	NM_002585.4	108
protein_coding	Yes	NM_001159279.1	108
protein_coding	Yes	NM_004041.5	108
protein_coding	Yes	NM_001023560.4	108
protein_coding	Yes	NM_001320038.2	108
protein_coding	Yes	NM_000426.4	108
protein_coding	Yes	NM_032359.4	108
lncRNA	Yes	-	108
lncRNA	Yes	-	108
protein_coding	Yes	NM_001102654.2	108
protein_coding	Yes	NM_014656.3	108
protein_coding	Yes	NM_001067.4	108
protein_coding	Yes	NM_001378615.1	108
lncRNA	Yes	-	108
protein_coding	Yes	NM_001145128.3	108
lncRNA	Yes	-	108
protein_coding	Yes	NM_001145306.2	108
lncRNA	Yes	-	108
ocessed_pseudoge	Yes	-	108
protein_coding	Yes	NM_001291284.2	108
protein_coding	Yes	NM_138967.4	108
protein_coding	Yes	NM_199461.4	108
protein_coding	Yes	NM_001395961.1	108
ocessed_pseudoge	Yes	-	108
protein_coding	Yes	NM_006516.4	108
lncRNA	Yes	-	108

ENST00000426656	17.7925308	-2.493884997	0.83214	-2.997	0.0027269	0.0154818	ENSG00000233435	10	133640788	133642894	AGGF1P2
ENST00000426991	104.9786206	-1.276573646	0.40233	-3.173	0.0015089	0.0095676	ENSG00000229152	13	110894638	110899172	ANKRD10-IT1
ENST00000427199	241.7496497	1.73692802	0.26728	6.4985	8.11E-11	3.62E-09	ENSG00000243414	5	115578495	115602479	TICAM2
ENST00000427219	61.05512225	-2.93317544	0.50743	-5.78	7.45E-09	2.25E-07	ENSG00000234354	13	100539900	100540248	RPS26P47
ENST00000427277	41.15290609	-1.402888162	0.53122	-2.641	0.0082692	0.0377077	ENSG00000165238	9	93184138	93320569	WNK2
ENST00000427478	178.2058559	-1.352797965	0.29449	-4.594	4.35E-06	6.52E-05	ENSG00000205423	16	50025224	50037081	CNEP1R1
ENST00000427587	105.0758729	-1.141382049	0.38333	-2.978	0.002906	0.016274	ENSG00000118894	16	5084283	5097795	EEF2KMT
ENST00000428039	173.9747759	-1.291396322	0.296	-4.363	1.28E-05	0.0001673	ENSG00000215086	10	72917640	72918534	NPM1P24
ENST00000428066	31014.17635	-1.687929981	0.18717	-9.018	1.91E-19	3.18E-17	ENSG00000130600	11	1995175	2001470	H19
ENST00000428084	29.49163983	1.666712225	0.62155	2.6815	0.0073288	0.034238	ENSG00000128512	7	111726109	112206399	DOCK4
ENST00000428149	258.1901799	-1.635273911	0.26541	-6.161	7.21E-10	2.66E-08	ENSG00000137760	11	107502726	107565735	ALKBH8
ENST00000428289	49.26793459	2.514514744	0.52266	4.811	1.50E-06	2.56E-05	ENSG00000233030	1	149785658	149793020	-
ENST00000428304	297.4322085	1.541583842	0.24794	6.2176	5.05E-10	1.91E-08	ENSG00000100439	14	22598299	22612963	ABHD4
ENST00000428443	34.71954206	-4.519812464	0.87822	-5.147	2.65E-07	5.57E-06	ENSG00000187231	2	179101677	179264832	SESTD1
ENST00000428512	181.2690407	-1.149005729	0.31497	-3.648	0.0002643	0.0022493	ENSG00000229097	10	70163684	70164124	CALM2P2
ENST00000428667	5.851876283	5.976669435	1.92036	3.1123	0.0018565	0.0113116	ENSG00000233818	21	36445730	36532408	CLDN14-AS1
ENST00000428826	575.5704941	1.029484065	0.27333	3.7664	0.0001656	0.0015109	ENSG00000108799	17	42700274	42745040	EZH1
ENST00000429027	420.5550175	-1.157305494	0.22431	-5.159	2.48E-07	5.23E-06	ENSG00000052126	12	19129732	19376400	PLEKHA5
ENST00000429289	53.35208395	-1.284881777	0.49226	-2.61	0.0090501	0.0405168	ENSG00000229153	7	143407812	143523449	EPHA1-AS1
ENST00000429299	10.00170996	6.749876023	1.73939	3.8806	0.0001042	0.0010154	ENSG00000227507	6	31580557	31582424	LTB
ENST00000429317	18.27855503	7.619916423	1.61488	4.7186	2.38E-06	3.84E-05	ENSG00000231420	2	150234891	150257007	LINC01817
ENST00000429345	6.739703613	6.182311911	1.87712	3.2935	0.0009894	0.0067542	ENSG00000216863	6	6346464	6622744	LY86-AS1
ENST00000429538	34.62955648	2.919346267	0.63387	4.6056	4.11E-06	6.20E-05	ENSG00000125618	2	113215996	113278921	PAX8
ENST00000429584	14.42323691	-2.466246004	0.93255	-2.645	0.0081784	0.0373776	ENSG00000230797	X	21855986	21858740	YY2
ENST00000429588	5.128950101	5.786184995	1.97811	2.9251	0.0034433	0.0186933	ENSG00000230479	21	36430359	36481070	-
ENST00000429818	20.06796063	-2.052328178	0.75895	-2.704	0.0068474	0.0324668	ENSG00000204802	9	62374461	62376780	FAM88C
ENST00000429979	16.94368297	2.712394826	0.94159	2.8807	0.0039684	0.0209731	ENSG00000102445	13	46334680	46387252	RUBCNL
ENST00000430034	20.38332466	-4.750717761	1.07386	-4.424	9.69E-06	0.0001308	ENSG00000240801	11	2129120	2129964	-
ENST00000430070	745.8514849	1.870719155	0.20187	9.2667	1.92E-20	3.50E-18	ENSG00000123444	11	47572196	47578970	KBTBD4
ENST00000430166	32.74866573	3.052732484	0.66638	4.5811	4.63E-06	6.87E-05	ENSG00000225733	3	14920346	14948424	FGD5-AS1
ENST00000430385	527.6061296	1.092790615	0.23226	4.705	2.54E-06	4.07E-05	ENSG00000196466	19	12390015	12401271	ZNF799
ENST00000430970	14.92011436	7.326499128	1.6494	4.4419	8.92E-06	0.0001218	ENSG00000234134	10	125718770	125719365	-
ENST00000431010	231.8941185	1.293540323	0.28321	4.5675	4.94E-06	7.27E-05	ENSG00000196639	3	11154492	11263557	HRH1
ENST00000431378	41.00749531	1.389082645	0.53375	2.6025	0.0092545	0.0412241	ENSG00000225380	9	40641840	40642117	IGKV1OR9-2
ENST00000431401	9.31445546	5.168326991	1.74097	2.9687	0.0029911	0.016649	ENSG00000231056	6	10474499	10478502	LINC02522
ENST00000431500	3.939287979	5.404271542	2.11648	2.5534	0.010667	0.046131	ENSG00000230790	2	11740996	11745301	-
ENST00000431670	2534.238976	-1.455592689	0.17311	-8.408	4.16E-17	5.16E-15	ENSG00000144824	3	111859264	111976517	PHLDB2
ENST00000431679	26.00523207	2.441871751	0.69285	3.5244	0.0004244	0.0033419	ENSG00000244219	7	99598266	99611045	TMEM225B
ENST00000431924	13.25896952	2.689174531	0.99179	2.7114	0.0066995	0.0319115	ENSG00000228274	22	38667584	38681860	-
ENST00000431955	11.78133875	3.846281611	1.23302	3.1194	0.0018122	0.0110975	ENSG00000241720	1	109725819	109775252	-

ocessed_pseudoge	Yes	-	108
lncRNA	Yes	-	108
protein_coding	Yes	NM_021649.7	108
ocessed_pseudoge	Yes	-	108
protein_coding	Yes	NM_006648.4	108
protein_coding	Yes	NM_001281789.2	108
protein_coding	Yes	NM_201400.4	108
ocessed_pseudoge	Yes	-	108
lncRNA	Yes	-	108
protein_coding	Yes	NM_001363540.2	108
protein_coding	Yes	NM_138775.3	108
lncRNA	Yes	-	108
protein_coding	Yes	NM_022060.3	108
protein_coding	Yes	NM_178123.5	108
ocessed_pseudoge	Yes	-	108
lncRNA	Yes	-	108
protein_coding	Yes	NM_001991.5	108
protein_coding	Yes	NM_001256470.2	108
lncRNA	Yes	-	108
protein_coding	Yes	NM_002341.2	108
lncRNA	Yes	-	108
lncRNA	Yes	-	108
protein_coding	Yes	NM_003466.4	108
protein_coding	Yes	NM_206923.4	108
lncRNA	Yes	-	108
lncRNA	Yes	-	108
protein_coding	Yes	NM_025113.5	108
lncRNA	Yes	-	108
protein_coding	Yes	NM_018095.6	108
lncRNA	Yes	-	108
protein_coding	Yes	NM_001080821.3	108
lncRNA	Yes	-	108
protein_coding	Yes	NM_001098212.2	108
IG_V_pseudogene	Yes	-	108
lncRNA	Yes	-	108
lncRNA	Yes	-	108
protein_coding	Yes	NM_001134438.2	108
protein_coding	Yes	NM_001195541.3	108
lncRNA	Yes	-	108
lncRNA	Yes	-	108

ENST00000432176	1187.19652	-1.828267522	0.18814	-9.718	2.54E-22	6.17E-20	ENSG00000122591	7	22934210	23014130	FAM126A
ENST00000432230	16.38149663	-7.508997428	1.63383	-4.596	4.31E-06	6.45E-05	ENSG00000235609	21	14818842	15014430	-
ENST00000432264	65.7400784	-4.186199565	0.55939	-7.484	7.24E-14	5.89E-12	ENSG00000177873	3	40477121	40491053	ZNF619
ENST00000432294	129.4295271	-3.277069173	0.41443	-7.907	2.63E-15	2.62E-13	ENSG00000241073	1	100057989	100084471	-
ENST00000432377	5.641330588	5.922929416	1.93661	3.0584	0.0022253	0.0131305	ENSG00000225611	3	42770599	42773785	CCDC13-AS2
ENST00000432564	249.7223062	-3.181604491	0.30028	-10.6	3.13E-26	1.19E-23	ENSG00000196917	12	122726075	122730844	HCAR1
ENST00000432901	254.452531	-1.16922898	0.25788	-4.534	5.79E-06	8.36E-05	ENSG00000232838	20	18137862	18143169	PET117
ENST00000432994	12.79698036	7.104160334	1.68355	4.2198	2.45E-05	0.0002919	ENSG00000236908	12	3318717	3325343	LINC02827
ENST00000433060	1681.39638	-1.020736445	0.19238	-5.306	1.12E-07	2.55E-06	ENSG00000166025	11	94768355	94876748	AMOTL1
ENST00000433096	135.7558695	-1.02883313	0.31712	-3.244	0.0011772	0.0078034	ENSG00000236456	20	34245952	34246537	-
ENST00000433132	38.0360557	-1.956142132	0.56261	-3.477	0.0005072	0.0038833	ENSG00000235912	1	27649418	27649610	-
ENST00000433197	226.2855578	1.042618251	0.27601	3.7775	0.0001584	0.0014545	ENSG00000178607	17	64039141	64130144	ERN1
ENST00000433398	204.897156	-1.209710094	0.28582	-4.232	2.31E-05	0.0002778	ENSG00000225573	7	66606737	66607107	RPL35P5
ENST00000433529	1162.278435	-1.04125044	0.20251	-5.142	2.72E-07	5.70E-06	ENSG00000116001	2	70209443	70248628	TIA1
ENST00000433688	266.0045747	-1.168667705	0.25695	-4.548	5.41E-06	7.87E-05	ENSG00000188070	11	63759891	63768775	ZFTA
ENST00000433976	195.5080864	1.715711297	0.28893	5.9382	2.88E-09	9.47E-08	ENSG00000170100	16	89217702	89237141	ZNF778
ENST00000434010	3.916465705	5.396742222	2.11677	2.5495	0.0107872	0.0464746	ENSG00000225246	20	34122469	34123290	RPS2P1
ENST00000434325	699.4139469	1.037981839	0.23268	4.4611	8.16E-06	0.0001128	ENSG00000141934	19	281042	291403	PLPP2
ENST00000434498	30.71378135	-1.864796837	0.62323	-2.992	0.0027701	0.0156669	ENSG00000214429	2	78412792	78413094	CYCSP6
ENST00000434651	8.578679867	6.527755504	1.78379	3.6595	0.0002527	0.0021653	ENSG00000179344	6	32659466	32666657	HLA-DQB1
ENST00000434687	664.3057339	-1.994364623	0.26463	-7.537	4.83E-14	4.06E-12	ENSG00000030419	2	212999697	213151612	IKZF2
ENST00000434705	67.71235677	-1.328742536	0.42244	-3.145	0.0016584	0.0103337	ENSG00000215210	9	30689104	30690272	RBMXP2
ENST00000434707	8.18041075	6.457587909	1.81064	3.5665	0.0003618	0.002918	ENSG00000224715	22	47345568	47373541	-
ENST00000434910	72.52340295	-1.236995949	0.44345	-2.79	0.0052789	0.0264221	ENSG00000234241	20	6213698	6215381	-
ENST00000435033	46.42628429	1.852424614	0.5163	3.5879	0.0003333	0.0027266	ENSG00000268744	19	12379188	12401274	-
ENST00000435363	40.6239894	1.487592127	0.55683	2.6716	0.0075501	0.0350107	ENSG00000224389	6	32014794	32035418	C4B
ENST00000435881	751.6497015	1.044814647	0.20069	5.2062	1.93E-07	4.16E-06	ENSG00000108788	17	42567099	42573203	MLX
ENST00000435962	197.991931	-4.746585369	0.4508	-10.53	6.34E-26	2.33E-23	ENSG00000165929	14	91779745	91867536	TC2N
ENST00000435967	13.90781663	-2.609568596	0.95612	-2.729	0.0063462	0.0305679	ENSG00000272328	7	8303740	8341343	-
ENST00000436066	761.3895094	1.55878799	0.21487	7.2544	4.03E-13	2.94E-11	ENSG00000166289	19	29665460	29675477	PLEKHF1
ENST00000436088	1057.233444	-1.538219688	0.19599	-7.848	4.21E-15	4.09E-13	ENSG00000108312	17	44205039	44219675	UBTF
ENST00000436346	146.4586848	-3.330726449	0.38734	-8.599	8.05E-18	1.11E-15	ENSG00000115355	2	55287841	55419856	CCDC88A
ENST00000436571	47.49100745	-1.564198601	0.50474	-3.099	0.0019416	0.0117225	ENSG00000242756	13	18837767	18838098	RHOT1P3
ENST00000436587	2601.757756	-1.160970805	0.17851	-6.504	7.84E-11	3.51E-09	ENSG00000106392	7	7182543	7248616	C1GALT1
ENST00000436857	182.5669429	1.079118255	0.28652	3.7663	0.0001657	0.0015111	ENSG00000290832	7	72969695	73005721	-
ENST00000436885	11.93712054	7.005282867	1.69594	4.1306	3.62E-05	0.0004109	ENSG00000228251	2	112590795	112591939	-
ENST00000437139	257.6043467	-1.473926814	0.25989	-5.671	1.42E-08	4.01E-07	ENSG00000179029	17	8172456	8176380	TMEM107
ENST00000437605	16.09903098	2.245869172	0.88655	2.5333	0.0113007	0.0481886	ENSG00000214946	17	15732282	15744782	TBC1D26
ENST00000437920	4.616569613	5.635108028	2.03024	2.7756	0.0055102	0.0273019	ENSG00000224565	20	47391928	47412327	LINC01754
ENST00000437936	37.56224291	5.611189757	1.00736	5.5702	2.54E-08	6.75E-07	ENSG00000188293	19	46229741	46231243	IGFL1

protein_coding	Yes	NM_032581.4	108
lncRNA	Yes	-	108
protein_coding	Yes	NM_001145093.4	108
lncRNA	Yes	-	108
lncRNA	Yes	-	108
protein_coding	Yes	NM_032554.4	108
protein_coding	Yes	NM_001164811.2	108
lncRNA	Yes	-	108
protein_coding	Yes	NM_130847.3	108
ocessed_pseudoge	Yes	-	108
ocessed_pseudoge	Yes	-	108
protein_coding	Yes	NM_001433.5	108
ocessed_pseudoge	Yes	-	108
protein_coding	Yes	NM_022173.4	108
protein_coding	Yes	NM_001144936.2	108
protein_coding	Yes	NM_001201407.2	108
ocessed_pseudoge	Yes	-	108
protein_coding	Yes	NM_003712.4	108
ocessed_pseudoge	Yes	-	108
protein_coding	Yes	NM_002123.5	108
protein_coding	Yes	NM_001387220.1	108
ocessed_pseudoge	Yes	-	108
lncRNA	Yes	-	108
ocessed_pseudoge	Yes	-	108
lncRNA	Yes	-	108
protein_coding	Yes	NM_001002029.4	108
protein_coding	Yes	NM_198204.2	108
protein_coding	Yes	NM_001128596.3	108
lncRNA	Yes	-	108
protein_coding	Yes	NM_024310.5	108
protein_coding	Yes	NM_014233.4	108
protein_coding	Yes	NM_001365480.1	108
ocessed_pseudoge	Yes	-	108
protein_coding	Yes	NM_020156.5	108
lncRNA	Yes	-	108
lncRNA	Yes	-	108
protein_coding	Yes	NM_183065.4	108
protein_coding	Yes	NM_001388465.1	108
lncRNA	Yes	-	108
protein_coding	Yes	NM_198541.2	108

ENST00000438110	3.916465705	5.396742222	2.11677	2.5495	0.0107872	0.0464746	ENSG00000226874	7	30544052	30544431	TRPC6P10
ENST00000438607	214.7178091	-1.297142021	0.28232	-4.595	4.34E-06	6.49E-05	ENSG00000232112	3	48440256	48444208	TMA7
ENST00000438793	913.5063365	-1.327378013	0.20408	-6.504	7.81E-11	3.50E-09	ENSG00000145730	5	102755304	103029716	PAM
ENST00000438938	7.320550923	6.299607737	1.83481	3.4334	0.0005961	0.0044428	ENSG00000223657	7	136938279	136938553	KRT8P51
ENST00000438980	273.3673015	1.134398989	0.25283	4.4869	7.23E-06	0.0001016	ENSG00000173264	11	64285847	64289494	GPR137
ENST00000439099	7.338227969	6.298766487	1.91964	3.2812	0.0010336	0.0070013	ENSG00000227383	9	34191554	34191881	RPL35AP2
ENST00000439174	2826.736454	-1.009345946	0.21763	-4.638	3.52E-06	5.40E-05	ENSG00000120063	17	65009288	65056740	GNA13
ENST00000439236	4.849937583	5.706544394	2.00745	2.8427	0.0044735	0.0230953	ENSG00000197301	12	65851339	65882167	HMGA2-AS1
ENST00000439298	32.48250108	1.842687337	0.61313	3.0054	0.0026524	0.0151321	ENSG00000225032	9	127816065	127822520	-
ENST00000439741	119.3311861	-1.70850073	0.36212	-4.718	2.38E-06	3.85E-05	ENSG00000014914	1	149928650	149936879	MTMR11
ENST00000439742	114.1891855	-1.306640792	0.34301	-3.809	0.0001393	0.0013021	ENSG00000213347	5	177307209	177311898	MXD3
ENST00000439838	105.5204109	-1.761384659	0.40157	-4.386	1.15E-05	0.0001521	ENSG00000249590	22	30409261	30428987	-
ENST00000440460	69.94331725	2.328473071	0.43884	5.3059	1.12E-07	2.54E-06	ENSG00000150764	11	111937342	112022653	DIXDC1
ENST00000440486	214.4470831	-1.461518037	0.30271	-4.828	1.38E-06	2.37E-05	ENSG00000068078	4	1793292	1808867	FGFR3
ENST00000440863	760.3851284	1.209699857	0.22306	5.4233	5.85E-08	1.43E-06	ENSG00000140474	15	74836117	74843156	ULK3
ENST00000440944	108.627218	-1.205673488	0.38151	-3.16	0.0015765	0.0099069	ENSG00000160360	9	136327538	136359601	GPSM1
ENST00000441095	10.74745841	6.853433659	1.72068	3.983	6.81E-05	0.00071	ENSG00000233922	21	45593653	45603056	LINC01694
ENST00000441178	49.02246151	-1.388291856	0.50696	-2.738	0.0061728	0.029889	ENSG00000235376	10	103245886	103248016	RPEL1
ENST00000441207	5.760587187	5.956234167	1.94463	3.0629	0.002192	0.0129702	ENSG00000227009	22	39155524	39155945	FUNDC2P4
ENST00000441322	31.23665128	-2.803572555	0.65525	-4.279	1.88E-05	0.0002323	ENSG00000237289	15	43594026	43599406	CKMT1B
ENST00000441339	235.7578882	1.044131529	0.32279	3.2348	0.0012175	0.0080238	ENSG00000172927	11	69294154	69297287	MYEOV
ENST00000441377	113.764738	-1.80654521	0.36881	-4.898	9.67E-07	1.73E-05	ENSG00000278419	10	3134565	3145166	-
ENST00000441545	83.85141527	1.23354453	0.38791	3.18	0.0014729	0.009385	ENSG00000137070	9	34652184	34661902	IL11RA
ENST00000441699	6.125743573	6.037873955	2.07228	2.9136	0.0035724	0.019245	ENSG00000176826	7	55682651	55713252	FKBP9P1
ENST00000442055	5.481574669	5.885502205	1.98526	2.9646	0.0030308	0.0168332	ENSG00000228172	1	25816748	25819772	-
ENST00000442218	78.16302084	-1.892659007	0.40703	-4.65	3.32E-06	5.14E-05	ENSG00000211584	12	47773237	47782751	SLC48A1
ENST00000442241	167.907252	-2.086588193	0.31644	-6.594	4.28E-11	2.05E-09	ENSG00000073536	17	35128729	35142303	NLE1
ENST00000442253	55.47005187	-2.259626434	0.49432	-4.571	4.85E-06	7.16E-05	ENSG00000170915	6	52362150	52407777	PAQR8
ENST00000442305	5.874698558	5.981707796	1.91881	3.1174	0.0018245	0.0111548	ENSG00000229393	1	2493436	2494479	-
ENST00000442396	38.72128715	-1.476115966	0.54426	-2.712	0.0066843	0.0318591	ENSG00000022976	14	102319745	102342367	ZNF839
ENST00000442673	61.14202535	3.558210789	0.5312	6.6985	2.11E-11	1.08E-09	ENSG00000236423	1	3900402	3917225	LINC01134
ENST00000442697	1512.30555	-1.35933578	0.19885	-6.836	8.15E-12	4.58E-10	ENSG00000164031	4	99896247	99946618	DNAJB14
ENST00000442969	3.870821157	5.381562504	2.12805	2.5289	0.011443	0.0486717	ENSG00000290660	13	23939556	23946782	-
ENST00000443035	1306.971246	1.042766266	0.18979	5.4943	3.92E-08	9.93E-07	ENSG00000174485	15	65659122	65792293	DENND4A
ENST00000443090	145.6852238	-1.488888151	0.31559	-4.718	2.38E-06	3.85E-05	ENSG00000224837	1	168055900	168056422	GCSHP5
ENST00000443236	12.63426551	3.155777798	1.08217	2.9162	0.0035437	0.0191213	ENSG00000160111	19	16892950	17026810	CPAMD8
ENST00000443279	5.851876283	5.976669435	1.92036	3.1123	0.0018565	0.0113116	ENSG00000213338	X	154622427	154623500	ATF4P1
ENST00000443585	592.0621669	-1.2524766	0.24837	-5.043	4.59E-07	9.01E-06	ENSG00000235162	12	105330690	105371518	C12orf75
ENST00000443759	97.33511268	1.212019532	0.36451	3.3251	0.000884	0.0061538	ENSG00000242294	7	100338196	100338886	STAG3L5P
ENST00000443935	26.14715659	2.666207091	0.74606	3.5737	0.000352	0.0028517	ENSG00000236754	22	17580156	17589192	-

ocessed_pseudoge	Yes	-	108
protein_coding	Yes	NM_015933.6	108
protein_coding	Yes	NM_001177306.2	108
ocessed_pseudoge	Yes	-	108
protein_coding	Yes	NM_001170880.2	108
ocessed_pseudoge	Yes	-	108
protein_coding	Yes	NM_006572.6	108
lncRNA	Yes	-	108
lncRNA	Yes	-	108
protein_coding	Yes	NM_001145862.2	108
protein_coding	Yes	NM_031300.4	108
protein_coding	Yes	-	108
protein_coding	Yes	NM_001037954.4	108
protein_coding	Yes	NM_000142.5	108
protein_coding	Yes	NM_001099436.4	108
protein_coding	Yes	NM_001145638.3	108
lncRNA	Yes	-	108
protein_coding	Yes	NM_001143909.1	108
ocessed_pseudoge	Yes	-	108
protein_coding	Yes	NM_001375484.1	108
protein_coding	Yes	NM_001293291.2	108
lncRNA	Yes	-	108
protein_coding	Yes	NM_001142784.3	108
ed_unprocessed_ps	Yes	-	108
lncRNA	Yes	-	108
protein_coding	Yes	NM_017842.3	108
protein_coding	Yes	NM_018096.5	108
protein_coding	Yes	NM_133367.5	108
lncRNA	Yes	-	108
protein_coding	Yes	NM_018335.6	108
lncRNA	Yes	-	108
protein_coding	Yes	NM_001031723.4	108
lncRNA	Yes	-	108
protein_coding	Yes	NM_001320835.1	108
ocessed_pseudoge	Yes	-	108
protein_coding	Yes	NM_015692.5	108
ocessed_pseudoge	Yes	-	108
protein_coding	Yes	NM_001145199.2	108
ed_unprocessed_ps	Yes	-	108
lncRNA	Yes	-	108

ENST00000444180	19.29840598	3.096276503	0.85247	3.6321	0.0002811	0.0023608	ENSG00000272508	10	86993431	87024732	-
ENST00000444623	64.17506944	2.014765769	0.49097	4.1036	4.07E-05	0.0004549	ENSG00000139631	12	53157662	53180925	CSAD
ENST00000444643	974.938826	1.055542247	0.19136	5.516	3.47E-08	8.90E-07	ENSG00000148719	10	72332862	72354919	DNAJB12
ENST00000444815	19.33911811	-1.978399905	0.76848	-2.574	0.0100408	0.0439829	ENSG00000214077	2	131423800	131424867	GNAQP1
ENST00000444896	7.82505202	-3.907159311	1.46424	-2.668	0.0076217	0.0352796	ENSG00000213070	1	164356766	164357364	HMGB3P6
ENST00000445105	154.4457382	-1.839338287	0.31398	-5.858	4.68E-09	1.47E-07	ENSG00000114279	3	192139389	192727541	FGF12
ENST00000445118	89.2982526	1.339787655	0.38096	3.5169	0.0004367	0.0034227	ENSG00000228794	1	827597	859446	LINC01128
ENST00000445131	56.84483752	-1.53250354	0.47091	-3.254	0.0011366	0.007583	ENSG00000233994	1	72274551	72275159	GDI2P2
ENST00000445224	259.6435479	-1.400079284	0.26362	-5.311	1.09E-07	2.48E-06	ENSG00000151967	3	159839860	159897360	SCHIP1
ENST00000445323	293.8383261	-2.767496259	0.26495	-10.45	1.54E-25	5.37E-23	ENSG00000063601	X	150693395	150765108	MTMR1
ENST00000445355	1206.18322	2.486165418	0.57153	4.35	1.36E-05	0.0001761	ENSG00000151651	10	133262422	133276868	ADAM8
ENST00000445716	209.8486059	-1.173376894	0.27315	-4.296	1.74E-05	0.0002174	ENSG00000234545	7	92560757	92590390	FAM133B
ENST00000445808	3.984932528	5.419288392	2.12719	2.5476	0.010846	0.0466832	ENSG00000225850	10	97334563	97343204	-
ENST00000445822	807.5855381	-1.064665902	0.22638	-4.703	2.56E-06	4.10E-05	ENSG00000224861	14	66012829	66013789	YBX1P1
ENST00000446378	71.42373021	1.144043002	0.4199	2.7245	0.006439	0.030931	ENSG00000164309	5	79689835	79800222	CMYA5
ENST00000446477	8.317486781	-4.001237197	1.44309	-2.773	0.0055594	0.0274896	ENSG00000224940	7	128350325	128361685	PRRT4
ENST00000446597	346.0664498	-1.588279188	0.28401	-5.592	2.24E-08	6.01E-07	ENSG00000111731	12	22448582	22544542	C2CD5
ENST00000446897	37.46705149	-1.507518918	0.55382	-2.722	0.0064885	0.0311225	ENSG00000234335	10	32102521	32103293	RPS4XP11
ENST00000446969	28.51363296	8.261412682	1.55834	5.3014	1.15E-07	2.60E-06	ENSG00000235959	2	95640180	95640604	-
ENST00000447052	8.385811218	6.491250825	1.90868	3.4009	0.0006716	0.0049113	ENSG00000223822	21	23390257	23390996	EEF1A1P1
ENST00000447107	10.95378434	2.964385401	1.17694	2.5187	0.0117783	0.0497896	ENSG00000234911	14	64346170	64374666	TEX21P
ENST00000447166	8.299667349	6.480655131	1.79333	3.6138	0.0003018	0.0025071	ENSG00000221843	2	27537385	27582722	C2orf16
ENST00000447467	92.58977082	-1.248007207	0.3813	-3.273	0.0010642	0.0071743	ENSG00000158560	7	95772553	96098424	DYNC1I1
ENST00000447510	5.385140344	5.856176292	1.95624	2.9936	0.0027572	0.0156051	ENSG00000007062	4	15968227	16084023	PROM1
ENST00000447535	23.65988325	-5.553427812	1.21602	-4.567	4.95E-06	7.29E-05	ENSG00000233056	21	42916802	42925630	ERVH48-1
ENST00000447540	759.0827745	-1.343938357	0.22293	-6.029	1.65E-09	5.72E-08	ENSG00000177200	16	53054990	53327497	CHD9
ENST00000447648	453.6428639	1.628995019	0.2228	7.3115	2.64E-13	1.99E-11	ENSG00000205356	7	98214623	98252232	TECPR1
ENST00000448387	58.80124364	1.667068384	0.56339	2.959	0.0030866	0.0170866	ENSG00000232653	15	32593455	32607310	GOLGA8N
ENST00000449058	341.974555	1.332017132	0.24599	5.4149	6.13E-08	1.49E-06	ENSG00000100284	22	35299893	35347973	TOM1
ENST00000449111	32.92868343	2.002191946	0.60316	3.3195	0.0009018	0.0062516	ENSG00000235703	X	149938627	150224580	EOLA2-DT
ENST00000449264	14.12872136	7.248467186	1.66018	4.3661	1.26E-05	0.000165	ENSG00000232810	6	31575564	31578336	TNF
ENST00000449291	199.0296761	1.590746125	0.29124	5.4619	4.71E-08	1.17E-06	ENSG00000147813	8	143574784	143578330	NAPRT
ENST00000449339	10.74745841	6.853433659	1.72068	3.983	6.81E-05	0.00071	ENSG00000234509	21	31653592	31659500	SOD1-DT
ENST00000449428	710.4866589	-1.057086452	0.24573	-4.302	1.69E-05	0.0002122	ENSG00000182534	17	76679565	76710965	MXRA7
ENST00000449549	96.26207471	1.132187882	0.37274	3.0375	0.0023857	0.0138852	ENSG00000237172	16	67148103	67150998	B3GNT9
ENST00000449599	5.559921538	-5.949645846	1.94296	-3.062	0.0021975	0.0129968	ENSG00000163508	3	27715952	27722323	EOMES
ENST00000449862	19.58533549	-2.00134356	0.76472	-2.617	0.0088682	0.0398659	ENSG00000249242	4	82483175	82561988	TMEM150C
ENST00000450114	955.930107	-1.143054149	0.19665	-5.813	6.15E-09	1.89E-07	ENSG00000166483	11	9573669	9589985	WEE1
ENST00000450169	595.9511652	-1.016879461	0.21412	-4.749	2.04E-06	3.36E-05	ENSG00000106355	7	32485337	32490380	LSM5
ENST00000450253	756.386531	-1.247922855	0.24644	-5.064	4.11E-07	8.18E-06	ENSG00000151247	4	98879275	98929133	EIF4E

lncRNA	Yes	-	108
protein_coding	Yes	NM_001244705.2	108
protein_coding	Yes	NM_017626.7	108
ocessed_pseudoge	Yes	-	108
ocessed_pseudoge	Yes	-	108
protein_coding	Yes	NM_004113.6	108
lncRNA	Yes	-	108
ocessed_pseudoge	Yes	-	108
protein_coding	Yes	-	108
protein_coding	Yes	NM_001306144.3	108
protein_coding	Yes	NM_001109.5	108
protein_coding	Yes	NM_152789.4	108
lncRNA	Yes	-	108
ocessed_pseudoge	Yes	-	108
protein_coding	Yes	NM_153610.5	108
protein_coding	Yes	-	108
protein_coding	Yes	NM_001286176.2	108
ocessed_pseudoge	Yes	-	108
ocessed_pseudoge	Yes	-	108
ocessed_pseudoge	Yes	-	108
ribed_unitary_pseu	Yes	-	108
protein_coding	Yes	NM_032266.5	108
protein_coding	Yes	NM_001135556.2	108
protein_coding	Yes	NM_006017.3	108
protein_coding	Yes	NM_001308491.2	108
protein_coding	Yes	NM_001308319.2	108
protein_coding	Yes	NM_015395.3	108
protein_coding	Yes	-	108
protein_coding	Yes	NM_005488.3	108
lncRNA	Yes	-	108
protein_coding	Yes	NM_000594.4	108
protein_coding	Yes	NM_145201.6	108
lncRNA	Yes	-	108
protein_coding	Yes	NM_198530.4	108
protein_coding	Yes	NM_033309.3	108
protein_coding	Yes	NM_001278182.2	108
protein_coding	Yes	NM_001080506.3	108
protein_coding	Yes	NM_003390.4	108
protein_coding	Yes	NM_012322.3	108
protein_coding	Yes	NM_001968.5	108

ENST00000450676	128.2824698	1.06670737	0.35039	3.0443	0.0023322	0.013629	ENSG00000232187	13	22696022	22696574	FTH1P7
ENST00000450689	425.3062986	-1.222492062	0.26895	-4.545	5.48E-06	7.97E-05	ENSG00000164659	7	86876905	87059654	ELAPOR2
ENST00000450708	221.1661453	-1.267307514	0.26927	-4.706	2.52E-06	4.05E-05	ENSG00000133895	11	64803515	64810551	MEN1
ENST00000450791	4951.722045	1.12295639	0.20041	5.6032	2.10E-08	5.69E-07	ENSG00000090615	12	132768913	132828869	GOLGA3
ENST00000451013	45.27927048	1.608381691	0.53207	3.0229	0.0025039	0.0144331	ENSG00000196275	7	74796150	74851605	GTF2IRD2
ENST00000451137	220.0269277	1.290646443	0.29697	4.3461	1.39E-05	0.0001787	ENSG00000142871	1	85580760	85583950	CCN1
ENST00000451298	36.86666579	-1.951148321	0.56751	-3.438	0.0005858	0.0043849	ENSG00000217576	13	52167708	52291557	-
ENST00000451311	3948.929844	-1.086605743	0.18083	-6.009	1.87E-09	6.38E-08	ENSG00000205542	X	12975109	12977223	TMSB4X
ENST00000451794	98.28224098	3.435169259	0.42717	8.0417	8.86E-16	9.34E-14	ENSG00000163121	2	96497645	96505357	NEURL3
ENST00000452015	35.04269652	1.505822378	0.58927	2.5554	0.0106067	0.0459563	ENSG00000235194	14	23295651	23302859	PPP1R3E
ENST00000452097	12.2617776	7.043023371	1.69042	4.1664	3.09E-05	0.000358	ENSG00000237017	19	54119510	54125343	PRPF31-AS1
ENST00000452984	3.916465705	5.396742222	2.11677	2.5495	0.0107872	0.0464746	ENSG00000225933	2	87654889	87655243	RPS14P5
ENST00000453066	18.34498838	2.651950978	0.83581	3.1729	0.0015092	0.0095684	ENSG00000167711	17	1742870	1755265	SERPINF2
ENST00000453097	73.33714668	2.121903455	0.43896	4.834	1.34E-06	2.31E-05	ENSG00000050438	12	51424830	51515763	SLC4A8
ENST00000453153	248.5468043	-1.509104077	0.26278	-5.743	9.31E-09	2.74E-07	ENSG00000138399	2	169528507	169573865	FASTKD1
ENST00000453347	5.829054009	5.971610018	1.92358	3.1044	0.0019065	0.0115479	ENSG00000228420	1	109828354	109871436	LINC01768
ENST00000453386	1860.715713	-1.290377117	0.1864	-6.923	4.44E-12	2.62E-10	ENSG00000136527	3	185914557	185938014	TRA2B
ENST00000453407	147.9718465	1.075798945	0.31483	3.4171	0.0006329	0.0046691	ENSG00000285064	6	33321385	33329286	-
ENST00000453433	277.2200861	2.011343791	0.28067	7.1663	7.71E-13	5.32E-11	ENSG00000228283	6	146802794	146803709	KATNBL1P6
ENST00000453848	332.0434146	-1.95256461	0.27571	-7.082	1.42E-12	9.38E-11	ENSG00000006118	11	60924459	60937159	TMEM132A
ENST00000453895	5.897520832	5.986731667	1.91891	3.1199	0.0018094	0.0110864	ENSG00000240710	1	204603034	204616565	-
ENST00000453996	59.94866483	-1.70121255	0.45796	-3.715	0.0002034	0.0017997	ENSG00000174177	16	88706502	88715396	CTU2
ENST00000454078	136.4776814	-1.124416406	0.34291	-3.279	0.0010418	0.0070467	ENSG00000232389	6	70608233	70609334	-
ENST00000454158	236.3921289	-1.938953815	0.26959	-7.192	6.37E-13	4.47E-11	ENSG00000181826	4	37610633	37686376	RELL1
ENST00000454366	357.8502603	-1.345132517	0.24321	-5.531	3.19E-08	8.26E-07	ENSG00000075218	22	46296869	46330810	GTSE1
ENST00000454544	92.14433076	1.037919917	0.38566	2.6913	0.0071171	0.0334591	ENSG00000225871	1	148435104	148436926	-
ENST00000454584	55.9504755	1.767381866	0.4899	3.6076	0.000309	0.0025571	ENSG00000148935	11	22666610	22813001	GAS2
ENST00000454741	171.9637568	1.36739596	0.31645	4.3211	1.55E-05	0.000197	ENSG00000235954	22	27919420	28002679	TTC28-AS1
ENST00000454872	230.7381084	-1.496416113	0.31587	-4.737	2.16E-06	3.53E-05	ENSG00000177694	3	174859333	175810548	NAALADL2
ENST00000455036	877.9568782	-1.670764233	0.23587	-7.083	1.41E-12	9.31E-11	ENSG00000205189	8	80486225	80526265	ZBTB10
ENST00000455201	20.12982086	-2.050167947	0.76062	-2.695	0.0070308	0.0331578	ENSG00000236565	13	65787931	65788764	HNRNPA3P5
ENST00000455537	7.947042781	6.415594013	1.82329	3.5187	0.0004337	0.0034042	ENSG00000155980	12	57550043	57586633	KIF5A
ENST00000455809	22.49125972	-2.151027665	0.73135	-2.941	0.0032697	0.017926	ENSG00000144559	3	11790441	11846885	TAMM41
ENST00000455965	29.47056811	1.851635114	0.70079	2.6422	0.0082363	0.0375914	ENSG00000235092	2	8666662	8681864	ID2-AS1
ENST00000456187	8.022258582	-5.00092053	1.78287	-2.805	0.0050318	0.0254128	ENSG00000234161	X	91414877	91434999	PABPC5-AS1
ENST00000456315	2091.173063	-1.364387816	0.17646	-7.732	1.06E-14	9.69E-13	ENSG00000024526	1	68474151	68497082	DEPDC1
ENST00000456555	4.149833674	5.480651158	2.08437	2.6294	0.0085535	0.0387013	ENSG00000233487	3	196986675	196987558	RPSAP69
ENST00000456806	126.6187357	-1.931743779	0.39149	-4.934	8.04E-07	1.47E-05	ENSG00000214652	7	64045433	64085339	ZNF727
ENST00000456849	8.094091675	-3.931734452	1.47729	-2.661	0.0077803	0.0358649	ENSG00000198682	10	87659877	87747705	PAPSS2
ENST00000456936	2347.617117	-1.044593291	0.20581	-5.076	3.86E-07	7.74E-06	ENSG00000134970	5	115613209	115626036	TMED7

ocessed_pseudoge	Yes	-	108
protein_coding	Yes	NM_001142749.3	108
protein_coding	Yes	NM_001370259.2	108
protein_coding	Yes	NM_001389683.1	108
protein_coding	Yes	NM_173537.5	108
protein_coding	Yes	NM_001554.5	108
lncRNA	Yes	-	108
protein_coding	Yes	NM_021109.4	108
protein_coding	Yes	NM_001285485.2	108
protein_coding	Yes	NM_001276318.2	108
lncRNA	Yes	-	108
ocessed_pseudoge	Yes	-	108
protein_coding	Yes	NM_000934.4	108
protein_coding	Yes	NM_001039960.3	108
protein_coding	Yes	NM_024622.6	108
lncRNA	Yes	-	108
protein_coding	Yes	NM_004593.3	108
protein_coding	Yes	-	108
ped_processed_pse	Yes	-	108
protein_coding	Yes	NM_178031.3	108
lncRNA	Yes	-	108
protein_coding	Yes	NM_001012759.3	108
ocessed_pseudoge	Yes	-	108
protein_coding	Yes	NM_001085400.2	108
protein_coding	Yes	NM_016426.7	108
ocessed_pseudoge	Yes	-	108
protein_coding	Yes	NM_001143830.3	108
lncRNA	Yes	-	108
protein_coding	Yes	NM_207015.3	108
protein_coding	Yes	NM_001105539.3	108
ocessed_pseudoge	Yes	-	108
protein_coding	Yes	NM_004984.4	108
protein_coding	Yes	NM_001284401.2	108
lncRNA	Yes	-	108
lncRNA	Yes	-	108
protein_coding	Yes	NM_001114120.3	108
ped_processed_pse	Yes	-	108
protein_coding	Yes	NM_001159522.3	108
protein_coding	Yes	NM_001015880.2	108
protein_coding	Yes	NM_181836.6	108

ENST00000457052	5.476429441	5.878068943	1.96884	2.9856	0.0028307	0.0159316	ENSG00000237452	19	45733438	45764541	MEIOSIN
ENST00000457231	3.893643431	5.3891778	2.12058	2.5414	0.0110418	0.0473143	ENSG00000186676	7	125033452	125035301	EEF1GP1
ENST00000457354	2158.37493	-1.071549295	0.18483	-5.797	6.73E-09	2.05E-07	ENSG00000139921	14	51240246	51257655	TMX1
ENST00000457497	334.2586979	-1.369230831	0.23842	-5.743	9.30E-09	2.74E-07	ENSG00000230076	2	214847127	214847445	RPL10P6
ENST00000457511	18.82948375	4.586912642	1.10542	4.1495	3.33E-05	0.0003821	ENSG00000041515	13	108629610	109208005	MYO16
ENST00000457996	26.6723776	2.289488726	0.68295	3.3524	0.0008013	0.0056877	ENSG00000233029	1	121090288	121097655	-
ENST00000458108	337.5065978	-1.131883973	0.31365	-3.609	0.0003077	0.0025474	ENSG00000234737	3	32258939	32260230	KRT18P15
ENST00000458198	232.4023651	-3.748005908	0.31796	-11.79	4.52E-32	3.23E-29	ENSG00000237190	5	134402064	134411881	CDKN2AIPNL
ENST00000458200	57.47453867	-1.642997706	0.461	-3.564	0.0003653	0.0029426	ENSG00000231752	1	121519344	121568791	EMBP1
ENST00000458483	152.5244491	1.485565334	0.34084	4.3586	1.31E-05	0.0001701	ENSG00000243708	15	41838846	41848143	PLA2G4B
ENST00000459742	7.110005227	6.256808197	1.84581	3.3897	0.0006996	0.0050839	ENSG00000259628	7	77043720	77198626	-
ENST00000460698	922.3379434	-1.289434575	0.19874	-6.488	8.69E-11	3.86E-09	ENSG00000122417	1	86346836	86396323	ODF2L
ENST00000461366	7.800136273	4.906034241	1.79157	2.7384	0.006174	0.0298939	ENSG00000128482	17	19411231	19417276	RNF112
ENST00000461517	9.813986537	6.721695078	1.74555	3.8507	0.0001178	0.0011275	ENSG00000241175	15	50244627	50244931	RN7SL494P
ENST00000461771	7.622385715	6.356779134	1.8235	3.486	0.0004902	0.0037741	ENSG00000243352	7	104911927	104912193	RN7SL8P
ENST00000462248	26.45396448	2.280248003	0.71217	3.2018	0.0013655	0.008812	ENSG00000241170	11	74919586	74919965	RPL31P46
ENST00000462792	12.96182182	-2.707613769	1.02363	-2.645	0.0081667	0.0373384	ENSG00000239572	3	87641411	87793629	-
ENST00000463397	31.36645101	1.778787878	0.60988	2.9166	0.0035383	0.0190962	ENSG00000239899	2	11584772	11585047	RN7SL674P
ENST00000463745	177.1148624	-2.247340002	0.3062	-7.339	2.14E-13	1.63E-11	ENSG00000113966	3	97764757	97801229	ARL6
ENST00000463779	7.67410984	3.809124575	1.50488	2.5312	0.0113681	0.0484216	ENSG00000239437	3	129591348	129591635	RN7SL752P
ENST00000463781	15.19295603	2.734263264	1.04805	2.6089	0.009083	0.0406333	ENSG00000145113	3	195746770	195811929	MUC4
ENST00000464755	40.07536665	1.666530358	0.54007	3.0858	0.0020302	0.0121706	ENSG00000276490	10	94688153	94853073	-
ENST00000464848	132.4163128	-4.20194307	0.41797	-10.05	8.89E-24	2.58E-21	ENSG00000105948	7	139133777	139191986	TTC26
ENST00000465093	40.46682115	-2.059605726	0.54929	-3.75	0.0001771	0.0015988	ENSG00000114790	3	154121389	154257825	ARHGEF26
ENST00000465139	1559.390741	-1.543815051	0.19897	-7.759	8.55E-15	7.92E-13	ENSG00000140350	15	68778534	68820895	ANP32A
ENST00000465271	615.2542852	-1.045830149	0.21277	-4.915	8.86E-07	1.60E-05	ENSG00000143771	1	224356878	224379452	CNIH4
ENST00000465829	35.94977932	1.495799376	0.58912	2.5391	0.0111153	0.0475664	ENSG00000170667	7	102479975	102517777	RASA4B
ENST00000466086	254.8385145	1.221174043	0.27609	4.4231	9.73E-06	0.0001312	ENSG00000234975	1	228687414	228687826	FTH1P2
ENST00000466133	219.5253968	-1.817017443	0.2769	-6.562	5.31E-11	2.47E-09	ENSG00000184100	3	160100849	160102793	BRD7P2
ENST00000466444	347.2879095	-1.462773161	0.23822	-6.14	8.24E-10	3.01E-08	ENSG00000111832	6	116571503	116597675	RWDD1
ENST00000467460	14.3849116	7.274264453	1.65643	4.3915	1.13E-05	0.000149	ENSG00000114757	3	179794957	180036937	PEX5L
ENST00000467482	4.149833674	5.480651158	2.08437	2.6294	0.0085535	0.0387013	ENSG00000101850	X	9725345	9765847	GPR143
ENST00000467883	25.79266332	3.202637337	0.75151	4.2616	2.03E-05	0.0002483	ENSG00000240869	6	20421582	20421887	RN7SL128P
ENST00000469408	647.4973322	-1.322742615	0.22772	-5.809	6.30E-09	1.93E-07	ENSG00000091127	7	105456502	105522271	PUS7
ENST00000470205	66.08162024	-1.494463345	0.42961	-3.479	0.000504	0.0038639	ENSG00000212829	9	9090897	9091245	RPS26P3
ENST00000470487	452.4352748	-2.187122899	0.2607	-8.389	4.89E-17	6.03E-15	ENSG00000173905	3	168008688	168095924	GOLIM4
ENST00000471086	17.92483134	2.293516998	0.84376	2.7182	0.0065638	0.0314155	ENSG00000244642	8	119862661	119862956	RN7SL396P
ENST00000471089	4.895582131	5.718672064	2.00026	2.859	0.0042502	0.0221757	ENSG00000240520	1	84365427	84397831	UOX
ENST00000471227	216.1412364	-1.080899401	0.26964	-4.009	6.10E-05	0.0006462	ENSG00000225067	19	15611656	15612122	RPL23AP2
ENST00000471496	78.83974233	-1.356552173	0.40159	-3.378	0.0007304	0.0052682	ENSG00000291106	3	183447581	183456014	LINC00888

protein_coding	Yes	NM_001310124.2	108
ocessed_pseudoge	Yes	-	108
protein_coding	Yes	NM_030755.5	108
ocessed_pseudoge	Yes	-	108
protein_coding	Yes	NM_001198950.3	108
lncRNA	Yes	-	108
ocessed_pseudoge	Yes	-	108
protein_coding	Yes	NM_080656.3	108
ed_unprocessed_ps	Yes	-	108
protein_coding	Yes	NM_001114633.2	108
lncRNA	Yes	-	108
protein_coding	Yes	NM_001366781.1	108
protein_coding	Yes	NM_007148.5	108
misc_RNA	Yes	-	108
misc_RNA	Yes	-	108
bed_processed_pse	Yes	-	108
lncRNA	Yes	-	108
misc_RNA	Yes	-	108
protein_coding	Yes	NM_001278293.3	108
misc_RNA	Yes	-	108
protein_coding	Yes	NM_018406.7	108
protein_coding	Yes	-	108
protein_coding	Yes	NM_024926.4	108
protein_coding	Yes	NM_015595.4	108
protein_coding	Yes	NM_006305.4	108
protein_coding	Yes	NM_014184.4	108
protein_coding	Yes	NM_001367767.2	108
ocessed_pseudoge	Yes	-	108
ocessed_pseudoge	Yes	-	108
protein_coding	Yes	NM_015952.4	108
protein_coding	Yes	NM_016559.3	108
protein_coding	Yes	NM_000273.3	108
misc_RNA	Yes	-	108
protein_coding	Yes	NM_019042.5	108
ocessed_pseudoge	Yes	-	108
protein_coding	Yes	NM_014498.5	108
misc_RNA	Yes	-	108
ribed_unitary_pseud	Yes	-	108
ocessed_pseudoge	Yes	-	108
lncRNA	Yes	-	108

ENST00000472174	1448.008489	1.052903972	0.22173	4.7486	2.05E-06	3.36E-05	ENSG00000117410	1	43974959	43978295	ATP6V0B
ENST00000472924	61.54736839	1.582319568	0.44424	3.5619	0.0003682	0.0029631	ENSG00000229320	3	160567851	160569248	KRT8P12
ENST00000473864	91.54428048	-1.095777117	0.38605	-2.838	0.0045337	0.0233445	ENSG00000230457	3	156809550	156810732	PA2G4P4
ENST00000473968	167.2251659	-1.505138908	0.30903	-4.87	1.11E-06	1.96E-05	ENSG00000125962	X	102599514	102604159	ARMCX5
ENST00000474844	202.9980315	-2.184214854	0.28632	-7.628	2.38E-14	2.07E-12	ENSG00000117481	1	46340806	46365018	NSUN4
ENST00000474889	6.817033743	-6.243872208	1.86108	-3.355	0.0007937	0.0056454	ENSG00000144724	3	61561570	62297609	PTPRG
ENST00000475156	196.456699	-1.45474072	0.29912	-4.863	1.15E-06	2.02E-05	ENSG00000212994	8	100895770	100896118	RPS26P6
ENST00000475668	24.63088631	2.240922738	0.70137	3.1951	0.001398	0.0089882	ENSG00000257335	7	141995878	142106747	MGAM
ENST00000476941	87.05777795	-1.370203406	0.41021	-3.34	0.0008371	0.0058951	ENSG00000144935	3	142724033	142807888	TRPC1
ENST00000477717	33.32278081	-1.53367927	0.58206	-2.635	0.0084163	0.0382372	ENSG00000117069	1	76867479	77067546	ST6GALNAC5
ENST00000477922	8.789225562	6.563315237	1.7758	3.696	0.000219	0.0019164	ENSG00000257743	7	142111717	142222324	MGAM2
ENST00000478945	17.83055202	-3.502672412	0.94558	-3.704	0.000212	0.0018644	ENSG00000163530	3	109293787	109316517	DPPA2
ENST00000479066	7.297728649	6.295565165	1.83679	3.4275	0.0006092	0.004523	ENSG00000243537	8	133325996	133326404	RPL32P20
ENST00000480336	82.15185292	-1.629289746	0.40157	-4.057	4.97E-05	0.0005404	ENSG00000254553	1	32465056	32600792	-
ENST00000481490	356.3531089	-1.74195231	0.24964	-6.978	3.00E-12	1.84E-10	ENSG00000265817	8	94427715	94436944	FSBP
ENST00000481504	24.21494462	-2.183208207	0.71371	-3.059	0.002221	0.0131122	ENSG00000187952	1	21428302	21429536	HS6ST1P1
ENST00000481739	1087.089414	-1.11609833	0.19135	-5.833	5.45E-09	1.69E-07	ENSG00000186350	9	134326454	134440585	RXRA
ENST00000481909	287.5896362	1.398548037	0.24955	5.6043	2.09E-08	5.66E-07	ENSG00000119965	10	122930900	122954227	C10orf88
ENST00000483720	33.30842493	-1.954282256	0.59584	-3.28	0.0010386	0.0070284	ENSG00000158301	X	102712447	102717733	GPRASP2
ENST00000483910	5.806138918	-6.012331624	1.9244	-3.124	0.0017825	0.0109512	ENSG00000064225	3	98763339	98795852	ST3GAL6
ENST00000484259	102.0056781	-1.867342153	0.37192	-5.021	5.15E-07	9.96E-06	ENSG00000165060	9	69035751	69079076	FXN
ENST00000484558	31.67380554	-1.540569911	0.60412	-2.55	0.0107696	0.046467	ENSG00000250479	22	23765833	23767972	CHCHD10
ENST00000484902	159.8776595	-1.486418341	0.30343	-4.899	9.64E-07	1.73E-05	ENSG00000227063	20	21755269	21755350	RPL41P1
ENST00000485303	75.08591175	-5.648789724	0.75988	-7.434	1.06E-13	8.39E-12	ENSG00000177707	3	111071815	111137560	NECTIN3
ENST00000485827	651.8039137	-1.755530658	0.22824	-7.692	1.45E-14	1.30E-12	ENSG00000196656	4	113214045	113214393	-
ENST00000486442	100.3725843	1.660302959	0.38804	4.2787	1.88E-05	0.0002322	ENSG00000119771	2	23385178	23708606	KLHL29
ENST00000486874	8.254022801	6.473511846	1.79719	3.602	0.0003158	0.0026029	ENSG00000240270	17	80976453	80976948	RPL12P37
ENST00000487137	143.8790096	-1.271589096	0.32068	-3.965	7.33E-05	0.0007567	ENSG00000139880	14	23047066	23057520	CDH24
ENST00000488788	4540.318244	2.159932974	0.21753	9.9293	3.10E-23	8.67E-21	ENSG00000270299	20	646625	675800	-
ENST00000489288	356.7380981	-1.299290317	0.23409	-5.55	2.85E-08	7.47E-07	ENSG00000171792	12	2877222	2889524	RHNO1
ENST00000489612	100.7693335	-1.087503598	0.39256	-2.77	0.005601	0.0276576	ENSG00000231167	7	105582257	105583256	YBX1P2
ENST00000489711	109.4207274	-1.534826192	0.34891	-4.399	1.09E-05	0.0001447	ENSG00000163406	3	121894400	121944188	SLC15A2
ENST00000490594	11.18404657	-5.493002831	1.73452	-3.167	0.0015409	0.0097254	ENSG00000251012	3	119147374	119187807	-
ENST00000491143	17.07382439	-2.123261433	0.8242	-2.576	0.0099908	0.0438107	ENSG00000119547	18	57435373	57491298	ONECUT2
ENST00000491614	963.9168943	-1.055808108	0.19122	-5.521	3.36E-08	8.66E-07	ENSG00000165609	10	12165329	12195891	NUDT5
ENST00000492267	62.24869435	-2.100084768	0.45777	-4.588	4.48E-06	6.68E-05	ENSG00000241095	3	82806514	82808021	CYP51A1P1
ENST00000493013	9.353166934	3.508322019	1.27876	2.7435	0.006078	0.0295266	ENSG00000241529	3	113632703	113632998	RN7SL767P
ENST00000493111	115.8251373	1.273224234	0.34103	3.7334	0.0001889	0.0016889	ENSG00000189077	7	75986830	75994595	TMEM120A
ENST00000494359	5.618508314	5.917680498	1.93743	3.0544	0.0022551	0.0132659	ENSG00000197149	11	17228278	17229151	-
ENST00000494857	76.32526242	-3.506655663	0.50959	-6.881	5.93E-12	3.43E-10	ENSG00000003989	8	17497087	17570566	SLC7A2

protein_coding	Yes	NM_004047.5	108
ped_processed_pse	Yes	-	108
ocessed_pseudoge	Yes	-	108
protein_coding	Yes	NM_001168478.2	108
protein_coding	Yes	NM_199044.4	108
protein_coding	Yes	NM_002841.4	108
ocessed_pseudoge	Yes	-	108
protein_coding	Yes	NM_001365693.1	108
protein_coding	Yes	NM_001251845.2	108
protein_coding	Yes	NM_030965.3	108
protein_coding	Yes	NM_001293626.2	108
protein_coding	Yes	NM_138815.4	108
ocessed_pseudoge	Yes	-	108
protein_coding	Yes	-	108
protein_coding	Yes	NM_001256141.2	108
ocessed_pseudoge	Yes	-	108
protein_coding	Yes	NM_002957.6	108
protein_coding	Yes	NM_024942.4	108
protein_coding	Yes	NM_001004051.4	108
protein_coding	Yes	NM_001323368.2	108
protein_coding	Yes	NM_000144.5	108
protein_coding	Yes	NM_213720.3	108
ocessed_pseudoge	Yes	-	108
protein_coding	Yes	NM_015480.3	108
ped_processed_pse	Yes	-	108
protein_coding	Yes	NM_052920.2	108
ocessed_pseudoge	Yes	-	108
protein_coding	Yes	NM_144985.4	108
protein_coding	Yes	-	108
protein_coding	Yes	NM_001252499.3	108
ocessed_pseudoge	Yes	-	108
protein_coding	Yes	NM_021082.4	108
protein_coding	Yes	-	108
protein_coding	Yes	NM_004852.3	108
protein_coding	Yes	NM_014142.4	108
ocessed_pseudoge	Yes	-	108
misc_RNA	Yes	-	108
protein_coding	Yes	NM_031925.3	108
ocessed_pseudoge	Yes	-	108
protein_coding	Yes	NM_001370338.1	108

ENST00000494861	14.85599146	-2.542960112	0.99281	-2.561	0.0104262	0.0453308	ENSG00000242169	4	188349375	188350024	RPL7AP27
ENST00000495231	69.86800299	-1.337244579	0.41687	-3.208	0.0013373	0.0086614	ENSG00000243824	14	34740335	34740833	RPL12P6
ENST00000495723	55.81102755	1.414830367	0.47908	2.9533	0.0031444	0.0173517	ENSG00000258461	15	42348102	42412317	-
ENST00000496040	198.5102673	-1.122328637	0.27816	-4.035	5.46E-05	0.0005864	ENSG00000240395	8	42341766	42342634	RPL5P23
ENST00000496677	501.459922	-1.260552676	0.23423	-5.382	7.38E-08	1.76E-06	ENSG00000157224	7	90403460	90415954	CLDN12
ENST00000497454	1516.141239	1.051757739	0.18398	5.7167	1.09E-08	3.15E-07	ENSG00000237441	6	33291653	33298942	RGL2
ENST00000497538	996.0345024	-1.14031884	0.21243	-5.368	7.96E-08	1.88E-06	ENSG00000240489	3	155987303	155988176	SETP14
ENST00000498907	156.3339219	-3.904978275	0.37893	-10.31	6.67E-25	2.16E-22	ENSG00000245848	19	33299933	33302534	CEBPA
ENST00000499418	63.12018154	1.59975829	0.44028	3.6335	0.0002796	0.0023501	ENSG00000245149	8	124462484	124474564	RNF139-DT
ENST00000499521	3563.630857	-1.148010658	0.17587	-6.528	6.68E-11	3.04E-09	ENSG00000230551	5	149494313	149504670	-
ENST00000499522	62.62182001	1.260606773	0.43547	2.8948	0.0037934	0.0202047	ENSG00000247081	8	103165928	103285907	BAALC-AS1
ENST00000499966	30.82788229	-3.383317166	0.70712	-4.785	1.71E-06	2.88E-05	ENSG00000246898	16	66408515	66412135	LINC00920
ENST00000501071	5.897520832	5.986731667	1.91891	3.1199	0.0018094	0.0110864	ENSG00000245729	5	8333480	8457558	LINC02226
ENST00000501211	7.190337852	-4.814188838	1.86679	-2.579	0.0099126	0.04354	ENSG00000253284	12	19147073	19154659	-
ENST00000501817	132.2718649	1.541473695	0.36738	4.1959	2.72E-05	0.0003198	ENSG00000245571	11	59130132	59142755	FAM111A-DT
ENST00000502082	126.9704668	-1.006166552	0.32554	-3.091	0.0019963	0.0119989	ENSG00000246228	8	127289816	127482139	CASC8
ENST00000502160	10.12798048	3.15058536	1.23664	2.5477	0.0108438	0.0466769	ENSG00000247213	12	108434129	108492585	LINC01498
ENST00000502390	7.320550923	6.299607737	1.83481	3.4334	0.0005961	0.0044428	ENSG00000231683	6	53561288	53617007	-
ENST00000502732	1884.660754	1.219326882	0.19377	6.2927	3.12E-10	1.23E-08	ENSG00000143322	1	179099329	179229677	ABL2
ENST00000502773	213.121319	-2.319083878	0.28702	-8.08	6.48E-16	6.98E-14	ENSG00000145431	4	156760453	156971799	PDGFC
ENST00000503057	122.5452638	-2.330181369	0.37384	-6.233	4.57E-10	1.74E-08	ENSG00000064042	4	41538156	41700044	LIMCH1
ENST00000503176	197.0795295	1.264290539	0.32785	3.8563	0.0001151	0.0011056	ENSG00000005882	17	50095350	50112152	PDK2
ENST00000503362	2669.737659	-1.177823827	0.18621	-6.325	2.53E-10	1.02E-08	ENSG00000129128	4	176319965	176332245	SPCS3
ENST00000503452	29.93697911	2.894433953	0.69739	4.1504	3.32E-05	0.0003808	ENSG00000251141	5	44744899	44808777	MRPS30-DT
ENST00000503602	25.22755271	-1.963434324	0.69396	-2.829	0.0046645	0.0238992	ENSG00000250132	12	974132	990041	-
ENST00000503611	15.90070443	-2.458815389	0.88082	-2.791	0.0052464	0.0262935	ENSG00000248429	4	158170751	158202877	GASK1B-AS1
ENST00000503731	42.1074067	-3.621490459	0.65895	-5.496	3.89E-08	9.86E-07	ENSG00000104723	8	15540262	15766638	TUSC3
ENST00000504081	31.61668167	-2.137435094	0.61633	-3.468	0.0005243	0.0039916	ENSG00000248268	5	111277516	111302567	-
ENST00000504120	417.0958126	1.023693933	0.23456	4.3643	1.28E-05	0.0001662	ENSG00000204970	5	140786139	141012347	PCDHA1
ENST00000504548	347.6463416	1.126292307	0.25526	4.4123	1.02E-05	0.0001371	ENSG00000179886	8	143597830	143603224	TIGD5
ENST00000504748	4.639391888	5.641491239	2.02631	2.7841	0.0053674	0.0267625	ENSG00000254094	4	1356580	1358075	-
ENST00000504771	446.5779315	-2.110582257	0.22628	-9.327	1.08E-20	2.08E-18	ENSG00000145779	5	119356018	119399688	TNFAIP8
ENST00000504924	191.3451358	-1.619064954	0.37228	-4.349	1.37E-05	0.0001768	ENSG00000185305	5	53883941	54310573	ARL15
ENST00000505065	1490.745024	-1.192338902	0.19257	-6.192	5.95E-10	2.22E-08	ENSG00000158985	5	131264052	131394672	CDC42SE2
ENST00000505658	1588.539739	-1.308560994	0.20382	-6.42	1.36E-10	5.79E-09	ENSG00000108848	17	50719602	50756219	LUC7L3
ENST00000506729	13.93147571	-5.815081318	1.68398	-3.453	0.000554	0.0041825	ENSG00000205359	5	102371773	102499001	SLCO6A1
ENST00000507003	28.02973503	1.643106397	0.63936	2.5699	0.0101726	0.0444329	ENSG00000248406	19	12379745	12383687	-
ENST00000507248	8.772482865	4.018889651	1.45077	2.7702	0.0056025	0.0276622	ENSG00000234492	4	108538189	108620413	RPL34-DT
ENST00000507707	61.17063237	-1.640186039	0.45366	-3.615	0.0002998	0.0024947	ENSG00000248873	5	42465399	42468868	SERBP1P6
ENST00000507866	5.897520832	5.986731667	1.91891	3.1199	0.0018094	0.0110864	ENSG00000184985	4	7192537	7742827	SORCS2

ocessed_pseudoge	Yes	-	108
ocessed_pseudoge	Yes	-	108
protein_coding	Yes	-	108
ocessed_pseudoge	Yes	-	108
protein_coding	Yes	NM_001185072.3	108
protein_coding	Yes	NM_004761.5	108
ocessed_pseudoge	Yes	-	108
protein_coding	Yes	NM_004364.5	108
lncRNA	Yes	-	108
lncRNA	Yes	-	108
lncRNA	Yes	-	108
lncRNA	Yes	-	108
lncRNA	Yes	-	108
lncRNA	Yes	-	108
lncRNA	Yes	-	108
lncRNA	Yes	-	108
lncRNA	Yes	-	108
lncRNA	Yes	-	108
protein_coding	Yes	NM_007314.4	108
protein_coding	Yes	NM_016205.3	108
protein_coding	Yes	NM_001330672.2	108
protein_coding	Yes	NM_002611.5	108
protein_coding	Yes	NM_021928.4	108
lncRNA	Yes	-	108
lncRNA	Yes	-	108
lncRNA	Yes	-	108
protein_coding	Yes	NM_006765.4	108
lncRNA	Yes	-	108
protein_coding	Yes	NM_018900.4	108
protein_coding	Yes	NM_032862.5	108
lncRNA	Yes	-	108
protein_coding	Yes	NM_014350.4	108
protein_coding	Yes	NM_019087.3	108
protein_coding	Yes	NM_001375635.1	108
protein_coding	Yes	NM_016424.5	108
protein_coding	Yes	NM_173488.5	108
ed_unprocessed_ps	Yes	-	108
lncRNA	Yes	-	108
ocessed_pseudoge	Yes	-	108
protein_coding	Yes	NM_020777.3	108

ENST00000508195	9.41571742	6.660556914	1.76803	3.7672	0.0001651	0.0015063	ENSG00000250130	15	44354690	44355692	GAPDHP43
ENST00000508254	132.8996039	1.775399889	0.32754	5.4204	5.95E-08	1.45E-06	ENSG00000174130	4	38822896	38856817	TLR6
ENST00000508821	402.2391554	-1.905594352	0.25377	-7.509	5.95E-14	4.93E-12	ENSG00000164181	5	60751790	60844269	ELOVL7
ENST00000508859	19.19613617	2.547275516	0.85846	2.9673	0.0030046	0.0167114	ENSG00000206077	5	710354	784729	ZDHHC11B
ENST00000509176	82.196051	-3.1058078	0.43859	-7.081	1.43E-12	9.42E-11	ENSG00000184305	4	90127393	91605295	CCSER1
ENST00000509437	449.9640584	-1.054744803	0.25323	-4.165	3.11E-05	0.0003596	ENSG00000155329	5	132996984	133026549	ZCCHC10
ENST00000509685	31.01321262	-2.262478376	0.63496	-3.563	0.0003664	0.0029502	ENSG00000250299	13	52617557	52637446	MRPS31P4
ENST00000509801	18.47337323	2.633177923	0.93005	2.8312	0.004637	0.0237828	ENSG00000166763	15	43699487	43718184	STRCP1
ENST00000509834	22.84720099	2.015524662	0.71885	2.8038	0.0050499	0.0254889	ENSG00000251432	4	128292932	128474996	LINC02615
ENST00000510025	1122.926713	-1.705034829	0.20608	-8.273	1.30E-16	1.53E-14	ENSG00000133835	5	119452496	119542332	HSD17B4
ENST00000510708	159.8914122	-1.575092397	0.3213	-4.902	9.48E-07	1.70E-05	ENSG00000169570	5	118836073	118988547	DTWD2
ENST00000510898	29.57490715	1.805829098	0.6463	2.7941	0.0052044	0.0261257	ENSG00000146094	5	177501903	177509826	DOK3
ENST00000511437	201.4038095	-1.513404863	0.27962	-5.412	6.22E-08	1.51E-06	ENSG00000150756	5	10225506	10249888	ATPSCKMT
ENST00000511689	1798.224142	-1.012673357	0.17855	-5.672	1.41E-08	4.01E-07	ENSG00000113648	5	135334380	135399231	MACROH2A1
ENST00000511893	81.4782157	1.295039579	0.40497	3.1979	0.0013844	0.0089146	ENSG00000251660	7	30157530	30159534	-
ENST00000511912	530.5123011	1.606600269	0.2441	6.5817	4.65E-11	2.20E-09	ENSG00000171503	4	158672295	158709623	ETFDH
ENST00000511928	156.6443639	2.769702548	0.34415	8.0481	8.41E-16	8.93E-14	ENSG00000250986	4	3758747	3763390	LINC02600
ENST00000512123	659.5852998	1.167537241	0.21795	5.3569	8.47E-08	1.98E-06	ENSG00000138759	4	78057322	78544269	FRAS1
ENST00000512369	1051.438302	1.023222598	0.20285	5.0441	4.56E-07	8.95E-06	ENSG00000070190	4	99816826	99870190	DAPP1
ENST00000512467	22.64424133	2.675439515	0.80702	3.3152	0.0009158	0.0063314	ENSG00000183775	5	144170872	144485686	KCTD16
ENST00000512551	93.41643382	1.542551668	0.39627	3.8926	9.92E-05	0.0009757	ENSG00000288559	10	73247341	73248268	-
ENST00000512737	3578.099066	-1.083871052	0.19325	-5.609	2.04E-08	5.54E-07	ENSG00000243678	17	51166410	51171744	NME2
ENST00000512856	12.46906058	-3.176397566	1.10042	-2.887	0.003895	0.0206552	ENSG00000248734	5	96784776	96785999	-
ENST00000513489	21.38405652	-1.946624632	0.7354	-2.647	0.0081206	0.0371557	ENSG00000250546	4	83796435	84284327	LINC02994
ENST00000513638	6.557803711	-6.188097437	1.87593	-3.299	0.0009714	0.0066492	ENSG00000290525	1	89407678	89424934	GBP1P1
ENST00000514057	37.62004384	-1.807785978	0.5881	-3.074	0.0021126	0.0125758	ENSG00000248527	1	633695	634376	MTATP6P1
ENST00000514254	63.81505783	1.410331849	0.45389	3.1072	0.0018887	0.0114677	ENSG00000250091	12	123925460	123934984	DNAH10OS
ENST00000514618	200.9749547	-1.138637934	0.31334	-3.634	0.0002792	0.0023477	ENSG00000145416	4	163524717	164384019	MARCHF1
ENST00000514975	69.87888456	1.122815946	0.41411	2.7114	0.0067004	0.031915	ENSG00000249264	4	105484697	105486080	EEF1A1P9
ENST00000515279	438.0473063	-1.234698842	0.2267	-5.446	5.14E-08	1.27E-06	ENSG00000138780	4	105708783	105847725	GSTCD
ENST00000515393	17.37954204	-6.136122389	1.60521	-3.823	0.000132	0.0012433	ENSG00000175471	5	94703689	95285094	MCTP1
ENST00000515425	420.6736617	-1.888442713	0.2331	-8.101	5.43E-16	5.89E-14	ENSG00000169247	5	148982149	149063062	SH3TC2
ENST00000515604	107.5707887	-1.270232139	0.3593	-3.535	0.0004074	0.0032284	ENSG00000272414	4	76251720	76311120	FAM47E-STBD1
ENST00000517356	5.36231807	5.850679104	1.95813	2.9879	0.0028091	0.015836	ENSG00000248964	8	29815003	29854543	-
ENST00000517392	350.6730991	-1.282955971	0.23657	-5.423	5.86E-08	1.43E-06	ENSG00000183137	6	109095506	109174418	CEP57L1
ENST00000517833	53.82087607	-2.30383402	0.51684	-4.458	8.29E-06	0.0001144	ENSG00000247317	8	142981737	143018447	LY6E-DT
ENST00000517956	632.8811335	1.759518677	0.23391	7.5224	5.38E-14	4.49E-12	ENSG00000156804	8	123497888	123541206	FBXO32
ENST00000517992	3307.941442	1.201868749	0.17988	6.6816	2.36E-11	1.20E-09	ENSG00000172164	8	120535755	120812046	SNTB1
ENST00000518297	1733.904475	-1.264102311	0.17985	-7.029	2.08E-12	1.33E-10	ENSG00000155506	5	154755376	154817605	LARP1
ENST00000518322	52.21785514	-2.849940096	0.52453	-5.433	5.53E-08	1.36E-06	ENSG00000188343	8	93700549	93731527	CIBAR1

ocessed_pseudoge	Yes	-	108
protein_coding	Yes	NM_006068.5	108
protein_coding	Yes	NM_024930.3	108
protein_coding	Yes	NM_001351303.2	108
protein_coding	Yes	NM_001145065.2	108
protein_coding	Yes	NM_001300816.3	108
ed_unprocessed_ps	Yes	-	108
ed_unprocessed_ps	Yes	-	108
lncRNA	Yes	-	108
protein_coding	Yes	NM_000414.4	108
protein_coding	Yes	NM_173666.4	108
protein_coding	Yes	NM_001308236.3	108
protein_coding	Yes	NM_199133.4	108
protein_coding	Yes	NM_138610.3	108
lncRNA	Yes	-	108
protein_coding	Yes	NM_004453.4	108
lncRNA	Yes	-	108
protein_coding	Yes	NM_025074.7	108
protein_coding	Yes	NM_014395.3	108
protein_coding	Yes	NM_020768.4	108
lncRNA	Yes	-	108
protein_coding	Yes	NM_002512.4	108
lncRNA	Yes	-	108
lncRNA	Yes	-	108
lncRNA	Yes	-	108
rocessed_pseudog	Yes	-	108
lncRNA	Yes	-	108
protein_coding	Yes	NM_001394959.1	108
ocessed_pseudoge	Yes	-	108
protein_coding	Yes	NM_001370181.1	108
protein_coding	Yes	NM_024717.7	108
protein_coding	Yes	NM_024577.4	108
protein_coding	Yes	-	108
lncRNA	Yes	-	108
protein_coding	Yes	NM_001271852.3	108
lncRNA	Yes	-	108
protein_coding	Yes	NM_058229.4	108
protein_coding	Yes	NM_021021.4	108
protein_coding	Yes	NM_033551.3	108
protein_coding	Yes	NM_145269.5	108

ENST00000518937	1396.309816	-1.435430303	0.19312	-7.433	1.06E-13	8.45E-12	ENSG00000076554	8	80034744	80171564	TPD52
ENST00000519106	40.57444561	-1.70342511	0.53651	-3.175	0.0014983	0.0095142	ENSG00000253958	8	8701936	8704096	CLDN23
ENST00000519564	8.18041075	6.457587909	1.81064	3.5665	0.0003618	0.002918	ENSG00000178187	5	178941197	178966433	ZNF454
ENST00000519676	129.8699264	1.030689026	0.322	3.2009	0.00137	0.0088364	ENSG00000174032	13	45393315	45418373	SLC25A30
ENST00000519793	4.172655949	5.487752313	2.08271	2.6349	0.0084159	0.0382366	ENSG00000254084	8	101411872	101452452	-
ENST00000520242	458.4607623	-1.032585199	0.21984	-4.697	2.64E-06	4.21E-05	ENSG00000154582	8	73945118	73972191	ELOC
ENST00000520248	142.0817965	-1.018234412	0.34132	-2.983	0.0028519	0.0160296	ENSG00000253919	8	78760141	78762431	THAP12P7
ENST00000520433	129.5671137	1.122995918	0.33687	3.3336	0.0008574	0.0060016	ENSG00000251003	8	105780245	106060524	ZFPM2-AS1
ENST00000520530	27.93176768	-3.357564879	0.77987	-4.305	1.67E-05	0.0002092	ENSG00000163013	2	73254689	73284478	FBXO41
ENST00000520547	336.0179248	-1.024922866	0.2694	-3.804	0.0001421	0.0013242	ENSG00000253626	10	79512532	79516440	EIF5AL1
ENST00000520566	70.39229227	-1.14874733	0.42991	-2.672	0.0075384	0.0349756	ENSG00000253683	5	172656521	172656713	-
ENST00000521088	390.0427814	-1.082867157	0.24947	-4.341	1.42E-05	0.0001827	ENSG00000213881	8	61202349	61203220	NPM1P6
ENST00000521381	150.2225817	-1.9961271	0.32642	-6.115	9.65E-10	3.48E-08	ENSG00000145675	5	68215755	68301821	PIK3R1
ENST00000521400	24.61582406	-1.911509714	0.75458	-2.533	0.0113023	0.0481939	ENSG00000120915	8	27491142	27545564	EPHX2
ENST00000521604	2194.012668	-1.736070615	0.20481	-8.476	2.32E-17	3.00E-15	ENSG00000187735	8	53966555	54022448	TCEA1
ENST00000522551	1064.773025	-1.254269231	0.1922	-6.526	6.76E-11	3.07E-09	ENSG00000233913	5	168616351	168616996	RPL10P9
ENST00000522652	2199.121802	1.01390415	0.17909	5.6615	1.50E-08	4.22E-07	ENSG00000008513	8	133454847	133571887	ST3GAL1
ENST00000523101	3.893643431	5.3891778	2.12058	2.5414	0.0110418	0.0473143	ENSG00000253347	17	47409321	47423526	-
ENST00000523860	61.03834252	-1.298418106	0.45553	-2.85	0.0043671	0.0226468	ENSG00000253706	8	74798783	74866925	-
ENST00000524325	107.7260428	1.10775043	0.34838	3.1797	0.0014744	0.0093924	ENSG00000171045	8	142212079	142403182	TSNARE1
ENST00000524555	19.7244074	7.729865044	1.60377	4.8198	1.44E-06	2.46E-05	ENSG00000255071	11	18231422	18248635	SAA2-SAA4
ENST00000524717	251.2377039	-1.545635177	0.3114	-4.964	6.92E-07	1.29E-05	ENSG00000184384	11	95976597	96343195	MAML2
ENST00000524732	32.22775736	-2.483515386	0.65006	-3.82	0.0001332	0.0012523	ENSG00000234964	11	59781317	59781722	FABP5P7
ENST00000524803	200.8324766	-2.426723215	0.29645	-8.186	2.70E-16	3.04E-14	ENSG00000166788	11	18080291	18106082	SAAL1
ENST00000525539	40.45293825	2.969399722	0.62198	4.7741	1.81E-06	3.01E-05	ENSG00000166473	16	81101122	81220370	PKD1L2
ENST00000525643	78.36570476	1.435036144	0.40513	3.5421	0.0003969	0.0031582	ENSG00000008517	16	3065639	3069530	IL32
ENST00000526036	134.9912532	1.049867692	0.31811	3.3003	0.0009657	0.0066159	ENSG00000254837	11	74493365	74498533	LIPT2-AS1
ENST00000526049	811.8596529	1.078216884	0.24994	4.3139	1.60E-05	0.0002024	ENSG00000131871	15	101272190	101277485	SELENOS
ENST00000526355	52.24376168	4.301693636	0.63512	6.773	1.26E-11	6.78E-10	ENSG00000152402	11	106674018	107018476	GUCY1A2
ENST00000526635	52.6439222	1.610532959	0.5165	3.1182	0.0018199	0.0111354	ENSG00000245849	15	40686182	40695101	RAD51-AS1
ENST00000526692	3.893643431	5.3891778	2.12058	2.5414	0.0110418	0.0473143	ENSG00000197254	11	58685085	58691861	-
ENST00000527043	12.40673302	4.569899564	1.34484	3.3981	0.0006786	0.0049564	ENSG00000173227	11	67023133	67050863	SYT12
ENST00000527524	5.925488334	5.998401328	1.97514	3.037	0.0023898	0.0139041	ENSG00000149403	11	120511747	120988906	GRIK4
ENST00000527620	145.2322924	1.576677914	0.39961	3.9455	7.96E-05	0.0008103	ENSG00000254815	11	557594	560106	LMNTD2-AS1
ENST00000527799	4.172655949	5.487752313	2.08271	2.6349	0.0084159	0.0382366	ENSG00000255158	11	665909	678391	-
ENST00000527879	68.0033819	-1.155362126	0.43256	-2.671	0.0075624	0.0350564	ENSG00000255150	12	104303738	104305205	EID3
ENST00000528080	1806.240946	-1.070801795	0.20141	-5.316	1.06E-07	2.42E-06	ENSG00000166471	11	9280653	9314593	TMEM41B
ENST00000528667	1945.017796	1.665522459	0.1774	9.3884	6.09E-21	1.21E-18	ENSG00000116337	1	109619836	109632051	AMPD2
ENST00000528887	6.574802466	6.14491106	1.87434	3.2784	0.0010438	0.007057	ENSG00000254501	11	65110713	65111695	-
ENST00000529104	128.4805043	1.905575139	0.35075	5.4328	5.55E-08	1.36E-06	ENSG00000291008	6	28091136	28093664	-

protein_coding	Yes	NM_001025253.3	108
protein_coding	Yes	NM_194284.3	108
protein_coding	Yes	NM_001178089.3	108
protein_coding	Yes	NM_001010875.4	108
lncRNA	Yes	-	108
protein_coding	Yes	NM_005648.4	108
ocessed_pseudoge	Yes	-	108
lncRNA	Yes	-	108
protein_coding	Yes	NM_001371389.2	108
protein_coding	Yes	NM_001099692.2	108
ocessed_pseudoge	Yes	-	108
ocessed_pseudoge	Yes	-	108
protein_coding	Yes	NM_181523.3	108
protein_coding	Yes	NM_001979.6	108
protein_coding	Yes	NM_006756.4	108
ocessed_pseudoge	Yes	-	108
protein_coding	Yes	NM_173344.3	108
lncRNA	Yes	-	108
lncRNA	Yes	-	108
protein_coding	Yes	NM_145003.5	108
protein_coding	Yes	-	108
protein_coding	Yes	NM_032427.4	108
ocessed_pseudoge	Yes	-	108
protein_coding	Yes	NM_138421.3	108
protein_coding	Yes	-	108
protein_coding	Yes	NM_001376923.1	108
lncRNA	Yes	-	108
protein_coding	Yes	NM_018445.6	108
protein_coding	Yes	NM_000855.3	108
lncRNA	Yes	-	108
ocessed_pseudoge	Yes	-	108
protein_coding	Yes	NM_177963.4	108
protein_coding	Yes	NM_014619.5	108
lncRNA	Yes	-	108
lncRNA	Yes	-	108
protein_coding	Yes	NM_001008394.3	108
protein_coding	Yes	NM_015012.4	108
protein_coding	Yes	NM_001368809.2	108
lncRNA	Yes	-	108
lncRNA	Yes	-	108

ENST00000529415	151.1475382	1.52795721	0.30969	4.9339	8.06E-07	1.47E-05	ENSG00000254402	8	144522387	144527033	LRRC24
ENST00000529689	362.7038416	-1.193338141	0.23251	-5.132	2.86E-07	5.95E-06	ENSG00000137500	11	83259080	83286367	CCDC90B
ENST00000530349	348.6112805	1.093712119	0.2386	4.584	4.56E-06	6.79E-05	ENSG00000176973	11	65572537	65574190	FAM89B
ENST00000530835	111.9282623	-1.070612435	0.36965	-2.896	0.0037761	0.0201321	ENSG00000214391	11	90282559	90284172	TUBAP2
ENST00000530997	1234.89308	-1.437924257	0.19201	-7.489	6.96E-14	5.69E-12	ENSG00000100503	14	50719762	50831162	NIN
ENST00000531224	2955.260238	-1.236418934	0.17578	-7.034	2.01E-12	1.29E-10	ENSG00000029363	6	136256626	136289846	BCLAF1
ENST00000531225	75.27342719	1.378196745	0.40873	3.3719	0.0007464	0.0053682	ENSG00000271698	8	144353227	144355609	-
ENST00000531301	55.56144544	1.87997759	0.47145	3.9876	6.67E-05	0.0006984	ENSG00000213252	11	106826391	106827890	-
ENST00000531380	44.25292723	-2.365310114	0.62733	-3.77	0.000163	0.0014905	ENSG00000174804	11	86945678	86955395	FZD4
ENST00000531678	4.276784029	-5.571798857	2.07206	-2.689	0.0071662	0.0336256	ENSG00000255374	12	11091286	11092313	TAS2R43
ENST00000531730	16.83971659	3.951898722	1.0734	3.6817	0.0002317	0.0020095	ENSG00000255050	8	143573489	143577397	-
ENST00000531738	161.8726745	1.901887432	0.31155	6.1047	1.03E-09	3.69E-08	ENSG00000064309	11	125956820	126062866	CDON
ENST00000532090	318.6731295	-1.094834165	0.24605	-4.45	8.60E-06	0.000118	ENSG00000254470	11	65773897	65780976	AP5B1
ENST00000532414	64.05467493	-1.93972585	0.44716	-4.338	1.44E-05	0.0001845	ENSG00000254726	1	156072012	156082465	MEX3A
ENST00000532599	27.90099529	1.76783967	0.64528	2.7397	0.0061504	0.0298003	ENSG00000254860	11	8965047	8977472	TMEM9B-AS1
ENST00000532602	438.5413429	1.011657537	0.23365	4.3298	1.49E-05	0.0001904	ENSG00000204961	5	140848381	141012347	PCDHA9
ENST00000532615	75.66278226	-1.551849049	0.4115	-3.771	0.0001624	0.0014864	ENSG00000254719	11	10272051	10272259	-
ENST00000532942	668.4045466	-1.841662317	0.2162	-8.518	1.62E-17	2.14E-15	ENSG00000285283	11	31816383	32104665	-
ENST00000533028	3398.430131	-1.366724965	0.18825	-7.26	3.87E-13	2.83E-11	ENSG00000117620	1	99969995	100035634	SLC35A3
ENST00000533244	403.109568	1.079643401	0.22748	4.7461	2.07E-06	3.40E-05	ENSG00000173992	11	66593184	66606019	CCS
ENST00000533293	42.74808615	1.345129061	0.5263	2.5558	0.0105942	0.0459174	ENSG00000213906	14	24310139	24312038	LTB4R2
ENST00000533400	3.784349268	-5.394885692	2.13765	-2.524	0.0116112	0.0492063	ENSG00000137752	11	105025507	105035144	CASP1
ENST00000533544	426.7805947	1.007796435	0.23959	4.2064	2.60E-05	0.0003073	ENSG00000175376	11	65996544	66002157	EIF1AD
ENST00000533601	59.27618998	-3.25936312	0.51482	-6.331	2.44E-10	9.80E-09	ENSG00000139998	14	64945815	64972336	RAB15
ENST00000533924	221.7793979	5.952039282	0.4867	12.229	2.16E-34	2.31E-31	ENSG00000255026	11	287304	288298	-
ENST00000533945	10.63334704	6.839576028	1.7303	3.9528	7.72E-05	0.00079	ENSG00000255507	11	75803430	75814576	UVRAG-DT
ENST00000534313	164.821834	1.323133578	0.3023	4.3769	1.20E-05	0.0001581	ENSG00000213445	11	65638100	65650912	SIPA1
ENST00000534348	357.3324693	-1.082021457	0.24947	-4.337	1.44E-05	0.0001848	ENSG00000255526	14	24214095	24232352	NEDD8-MDP1
ENST00000534431	4.895582131	5.718672064	2.00026	2.859	0.0042502	0.0221757	ENSG00000255202	11	33665219	33696701	-
ENST00000534526	496.9601286	-1.386844961	0.21778	-6.368	1.91E-10	7.88E-09	ENSG00000139132	12	32399557	32646050	FGD4
ENST00000535113	62.56280793	1.304259256	0.46218	2.822	0.0047731	0.0243389	ENSG00000184949	22	38578117	38656392	FAM227A
ENST00000535199	8.533035318	6.520846942	1.78492	3.6533	0.0002589	0.0022104	ENSG00000230212	21	36069641	36126640	CBR1-AS1
ENST00000535358	9.937217273	3.577988108	1.30619	2.7393	0.0061579	0.0298322	ENSG00000198520	1	44674713	44725591	ARMH1
ENST00000535420	843.2914447	1.021290669	0.20266	5.0393	4.67E-07	9.16E-06	ENSG00000160685	1	155002801	155018523	ZBTB7B
ENST00000535784	711.8397067	-1.197813644	0.2043	-5.863	4.55E-09	1.43E-07	ENSG00000165983	10	16437009	16513745	PTER
ENST00000535817	4.895582131	5.718672064	2.00026	2.859	0.0042502	0.0221757	ENSG00000256690	11	62832233	62834043	STX5-DT
ENST00000535829	52.25517984	-3.060739971	0.53363	-5.736	9.71E-09	2.84E-07	ENSG00000166532	12	8697990	8777191	RIMKLB
ENST00000535902	142.719898	1.143409105	0.34739	3.2914	0.0009967	0.0067972	ENSG00000111261	12	12326055	12350242	MANSC1
ENST00000535913	7.155649776	6.265118335	1.84863	3.3891	0.0007013	0.0050939	ENSG00000204044	20	46013499	46022073	SLC12A5-AS1
ENST00000537262	253.423373	-2.282962654	0.28813	-7.923	2.31E-15	2.32E-13	ENSG00000256632	12	132688048	132714912	-

protein_coding	Yes	NM_001024678.4	108
protein_coding	Yes	NM_021825.5	108
protein_coding	Yes	NM_001098785.2	108
ocessed_pseudoge	Yes	-	108
protein_coding	Yes	NM_020921.4	108
protein_coding	Yes	NM_014739.3	108
protein_coding	Yes	-	108
ocessed_pseudoge	Yes	-	108
protein_coding	Yes	NM_012193.4	108
protein_coding	Yes	NM_176884.2	108
lncRNA	Yes	-	108
protein_coding	Yes	NM_001378964.1	108
protein_coding	Yes	NM_138368.5	108
protein_coding	Yes	NM_001093725.2	108
lncRNA	Yes	-	108
protein_coding	Yes	NM_031857.2	108
ocessed_pseudoge	Yes	-	108
protein_coding	Yes	-	108
protein_coding	Yes	NM_012243.3	108
protein_coding	Yes	NM_005125.2	108
protein_coding	Yes	NM_019839.5	108
protein_coding	Yes	NM_001257118.3	108
protein_coding	Yes	NM_001242481.2	108
protein_coding	Yes	NM_001308154.2	108
lncRNA	Yes	-	108
lncRNA	Yes	-	108
protein_coding	Yes	NM_006747.4	108
protein_coding	Yes	-	108
lncRNA	Yes	-	108
protein_coding	Yes	NM_001370298.3	108
protein_coding	Yes	NM_001013647.2	108
lncRNA	Yes	-	108
protein_coding	Yes	NM_001145636.2	108
protein_coding	Yes	NM_001256455.2	108
protein_coding	Yes	NM_001261836.2	108
lncRNA	Yes	-	108
protein_coding	Yes	NM_001297776.2	108
protein_coding	Yes	NM_018050.4	108
lncRNA	Yes	-	108
lncRNA	Yes	-	108

ENST00000537384	672.7716825	-1.41542969	0.31154	-4.543	5.54E-06	8.04E-05	ENSG00000188191	7	549197	727281	PRKAR1B
ENST00000537570	545.4858539	-1.011299901	0.23603	-4.285	1.83E-05	0.000227	ENSG00000256664	12	68841945	68842384	-
ENST00000537668	58.94151674	-1.257021971	0.45351	-2.772	0.0055756	0.027559	ENSG00000256594	12	9653682	9657043	-
ENST00000537763	39.9104655	1.658205337	0.54033	3.0689	0.0021486	0.0127542	ENSG00000146955	7	140404057	140427974	RAB19
ENST00000538077	109.6928606	-2.209472711	0.36545	-6.046	1.49E-09	5.19E-08	ENSG00000255874	13	110863986	110870330	PRECSIT
ENST00000538779	23.58806059	-2.192353732	0.77284	-2.837	0.0045577	0.0234437	ENSG00000139144	12	18261517	18648416	PIK3C2G
ENST00000538862	299.1962287	-1.429350618	0.25828	-5.534	3.13E-08	8.12E-07	ENSG00000111665	12	6848827	6851286	CDCA3
ENST00000538936	660.3102379	1.185356705	0.24758	4.7879	1.69E-06	2.83E-05	ENSG00000185813	17	81900957	81911399	PCYT2
ENST00000539652	654.1489177	-1.090293152	0.2054	-5.308	1.11E-07	2.52E-06	ENSG00000228144	12	66123916	66169985	-
ENST00000539664	7.711141184	-3.91566413	1.52954	-2.56	0.0104666	0.0454779	ENSG00000255690	7	28953357	28958330	TRIL
ENST00000539745	623.569626	-1.486636161	0.24183	-6.148	7.87E-10	2.88E-08	ENSG00000197837	12	14770719	14771131	H4-16
ENST00000540175	165.7547709	-1.582951447	0.3611	-4.384	1.17E-05	0.0001537	ENSG00000256663	12	20551589	20553012	-
ENST00000541036	323.7198967	-2.722324554	0.26126	-10.42	2.01E-25	6.91E-23	ENSG00000156299	21	31118417	31344261	TIAM1
ENST00000541103	62.80160405	1.527852729	0.43951	3.4763	0.0005084	0.0038913	ENSG00000176700	15	84631962	84642066	SCAND2P
ENST00000541790	2301.905811	-1.213921762	0.17556	-6.914	4.70E-12	2.77E-10	ENSG00000273802	6	26216199	26216688	H2BC8
ENST00000541899	5.385140344	5.856176292	1.95624	2.9936	0.0027572	0.0156051	ENSG00000284844	11	72105923	72109596	TOMT
ENST00000542575	5233.869193	1.099795925	0.16642	6.6085	3.88E-11	1.87E-09	ENSG00000105281	19	46774882	46788594	SLC1A5
ENST00000542754	116.5985686	-1.236862858	0.36812	-3.36	0.0007797	0.005565	ENSG00000100478	14	31025648	31096450	AP4S1
ENST00000542869	90.25394931	1.788821489	0.38734	4.6182	3.87E-06	5.87E-05	ENSG00000101213	20	63528000	63537376	PTK6
ENST00000543262	88.80008272	1.066315977	0.38379	2.7784	0.0054627	0.0271228	ENSG00000151376	11	86441109	86672636	ME3
ENST00000543484	1178.522908	-1.204634923	0.24647	-4.888	1.02E-06	1.82E-05	ENSG00000165685	12	10179031	10191804	TMEM52B
ENST00000543942	33.81403559	-1.919662716	0.59018	-3.253	0.0011432	0.0076167	ENSG00000185306	12	64264761	64390758	C12orf56
ENST00000544040	4032.273149	-1.503833885	0.16904	-8.896	5.78E-19	9.08E-17	ENSG00000111642	12	6570081	6607379	CHD4
ENST00000544216	1596.606004	-1.269231991	0.18624	-6.815	9.42E-12	5.23E-10	ENSG00000257103	19	34172503	34229288	LSM14A
ENST00000544421	5.476429441	5.878068943	1.96884	2.9856	0.0028307	0.0159316	ENSG00000257052	11	60906788	60909742	PRPF19-DT
ENST00000544898	118.5429565	-2.063232832	0.35672	-5.784	7.30E-09	2.21E-07	ENSG00000166548	16	66508002	66550122	TK2
ENST00000544920	14.48126354	2.842236572	0.95174	2.9864	0.0028232	0.0159027	ENSG00000256338	15	28315650	28316059	RPL41P2
ENST00000545001	73.81674847	1.219278425	0.40954	2.9772	0.0029092	0.0162869	ENSG00000256293	12	64338177	64338797	ATP6V1E1P3
ENST00000545069	12.24774821	3.132902768	1.08269	2.8936	0.0038083	0.0202725	ENSG00000256625	12	27121909	27147664	-
ENST00000545202	40.25273962	8.757150316	1.53555	5.7029	1.18E-08	3.39E-07	ENSG00000255980	11	69425677	69429621	LINC02953
ENST00000545287	959.2607562	-1.194968989	0.21417	-5.58	2.41E-08	6.43E-07	ENSG00000126858	17	32142501	32225727	RHOT1
ENST00000545904	16.19023922	3.559113316	0.985	3.6133	0.0003023	0.0025108	ENSG00000256747	12	27779820	27781067	-
ENST00000548203	22.64282173	-2.287839034	0.77217	-2.963	0.0030478	0.0169111	ENSG00000257622	14	105152193	105181194	-
ENST00000548861	454.2664799	-1.027617015	0.2329	-4.412	1.02E-05	0.0001371	ENSG00000257411	12	56101330	56109289	-
ENST00000549441	12.58211792	-5.64759507	1.71999	-3.283	0.0010253	0.006956	ENSG00000178401	12	49346887	49353690	DNAJC22
ENST00000549521	3.870821157	5.381562504	2.12805	2.5289	0.011443	0.0486717	ENSG00000258112	12	75257634	75258634	CCNG2P1
ENST00000549807	9.557796293	6.683699054	1.7523	3.8142	0.0001366	0.00128	ENSG00000257732	12	104262313	104280722	-
ENST00000549982	1327.164221	-1.037290164	0.18376	-5.645	1.65E-08	4.59E-07	ENSG00000198015	12	93467513	93516214	MRPL42
ENST00000549994	129.2198037	1.403538734	0.41126	3.4128	0.0006431	0.0047338	ENSG00000135407	12	57797379	57818734	AVIL
ENST00000550402	7.83293141	6.396747334	1.81214	3.5299	0.0004156	0.0032834	ENSG00000174600	12	108288045	108339311	CMKLR1

protein_coding	Yes	NM_001164760.2	108
ocessed_pseudoge	Yes	-	108
ed_unprocessed_ps	Yes	-	108
protein_coding	Yes	NM_001008749.3	108
lncRNA	Yes	-	108
protein_coding	Yes	NM_001288772.2	108
protein_coding	Yes	NM_031299.7	108
protein_coding	Yes	NM_002861.5	108
protein_coding	Yes	-	108
protein_coding	Yes	NM_014817.4	108
protein_coding	Yes	NM_175054.2	108
ocessed_pseudoge	Yes	-	108
protein_coding	Yes	NM_001353694.2	108
ed_unprocessed_ps	Yes	-	108
protein_coding	Yes	NM_003518.4	108
protein_coding	Yes	NM_001393500.2	108
protein_coding	Yes	NM_005628.3	108
protein_coding	Yes	NM_001128126.3	108
protein_coding	Yes	NM_005975.4	108
protein_coding	Yes	-	108
protein_coding	Yes	NM_001384896.1	108
protein_coding	Yes	NM_001170633.2	108
protein_coding	Yes	NM_001273.5	108
protein_coding	Yes	NM_015578.4	108
lncRNA	Yes	-	108
protein_coding	Yes	NM_004614.5	108
ocessed_pseudoge	Yes	-	108
ocessed_pseudoge	Yes	-	108
processed_pseudog	Yes	-	108
lncRNA	Yes	-	108
protein_coding	Yes	NM_001033566.3	108
lncRNA	Yes	-	108
lncRNA	Yes	-	108
protein_coding	Yes	-	108
protein_coding	Yes	NM_001304944.2	108
ocessed_pseudoge	Yes	-	108
lncRNA	Yes	-	108
protein_coding	Yes	NM_014050.4	108
protein_coding	Yes	NM_006576.4	108
protein_coding	Yes	NM_001142343.2	108

ENST00000550948	31.70318638	1.935942847	0.60955	3.176	0.001493	0.009487	ENSG00000110876	12	108621894	108633894	SELPLG
ENST00000551380	12.00558736	7.012693587	1.6948	4.1378	3.51E-05	0.0003996	ENSG00000166704	19	57977075	58002820	ZNF606
ENST00000551556	37.41502775	-2.510937694	0.61589	-4.077	4.56E-05	0.0005022	ENSG00000120833	12	93572674	93576745	SOCS2
ENST00000551956	20.66277887	3.211827601	0.84537	3.7993	0.0001451	0.0013478	ENSG00000170477	12	52806548	52814116	KRT4
ENST00000552695	97.75988013	1.075022624	0.36892	2.914	0.0035686	0.0192283	ENSG00000198855	12	108515276	108521210	FICD
ENST00000552810	432.4586846	-1.526076623	0.2277	-6.702	2.05E-11	1.06E-09	ENSG00000198707	12	88049015	88142088	CEP290
ENST00000553443	22.21446631	2.078806042	0.76601	2.7138	0.0066512	0.0317299	ENSG00000139865	14	37595628	37842717	TTC6
ENST00000553458	248.0148404	1.044824616	0.37644	2.7755	0.0055109	0.0273034	ENSG00000119711	14	74056846	74084453	ALDH6A1
ENST00000553946	78.62205676	-1.310904791	0.41764	-3.139	0.0016963	0.0105239	ENSG00000258892	14	60879713	60982585	-
ENST00000554452	9.791164263	6.718677158	1.74529	3.8496	0.0001183	0.0011321	ENSG00000258590	15	20657637	20688408	NBEAP1
ENST00000554659	793.5301577	-2.494025791	0.21237	-11.74	7.61E-32	5.29E-29	ENSG00000100568	14	67647084	67674632	VTI1B
ENST00000554737	19.55832627	3.585396817	0.92107	3.8926	9.92E-05	0.0009757	ENSG00000258813	14	73057924	73059415	RBM25-AS1
ENST00000554922	25.55305285	-3.23203757	0.76419	-4.229	2.34E-05	0.0002812	ENSG00000165521	14	88612430	88792953	EML5
ENST00000554937	125.4707157	1.230818147	0.44323	2.7769	0.0054874	0.0272206	ENSG00000179899	12	55411726	55414787	PHC1P1
ENST00000555043	6.295789948	6.083175617	1.89489	3.2103	0.0013259	0.008602	ENSG00000258377	14	49620814	49623480	-
ENST00000555147	72.94217942	1.386990739	0.41493	3.3427	0.0008295	0.0058486	ENSG00000258839	16	89918861	89920972	MC1R
ENST00000555265	241.6425977	-1.102847903	0.26551	-4.154	3.27E-05	0.0003759	ENSG00000100629	14	80496478	80941738	CEP128
ENST00000555572	4274.574891	-1.214084418	0.20305	-5.979	2.24E-09	7.54E-08	ENSG00000011052	17	51153635	51171742	NME1-NME2
ENST00000556029	5337.713787	-1.602846101	0.17452	-9.184	4.14E-20	7.27E-18	ENSG00000120802	12	98515589	98550351	TMPO
ENST00000556143	262.8751204	1.020705381	0.28079	3.6351	0.0002779	0.0023381	ENSG00000165861	14	72969450	73027106	ZFYVE1
ENST00000556147	34.27086909	-4.253690618	0.75996	-5.597	2.18E-08	5.86E-07	ENSG00000165795	14	21016771	21025061	NDRG2
ENST00000556278	307.0677334	1.393765937	0.24488	5.6915	1.26E-08	3.61E-07	ENSG00000258728	9	34646644	34657113	-
ENST00000556509	33.31550456	-2.561287017	0.68127	-3.76	0.0001702	0.0015466	ENSG00000205683	14	72609033	72894101	DPF3
ENST00000556529	42.71935797	2.279219093	0.55174	4.131	3.61E-05	0.0004103	ENSG00000258102	12	116559380	116576606	MAP1LC3B2
ENST00000556667	4.289796681	-5.575720003	2.06784	-2.696	0.0070093	0.0330813	ENSG00000258708	14	37171887	37173811	SLC25A21-AS1
ENST00000556738	19.84034576	-2.676789956	0.80063	-3.343	0.0008277	0.0058377	ENSG00000258768	14	20260479	20264308	-
ENST00000557185	486.374362	-1.482791076	0.21783	-6.807	9.95E-12	5.48E-10	ENSG00000100612	14	60144118	60165398	DHRS7
ENST00000558342	6.83099271	6.199783511	1.85961	3.3339	0.0008563	0.0059955	ENSG00000259442	15	82744222	82750289	-
ENST00000558391	4.94122668	5.730713439	2.00267	2.8615	0.0042159	0.0220395	ENSG00000259315	15	82726306	82727425	ACTG1P17
ENST00000558480	113.514915	-2.074689284	0.35798	-5.796	6.81E-09	2.07E-07	ENSG00000058335	15	78959905	79090780	RASGRF1
ENST00000558555	62.96147426	-1.214875796	0.47157	-2.576	0.0099878	0.0438018	ENSG00000105419	19	47403123	47419527	MEIS3
ENST00000558616	28.03987113	1.783396262	0.63938	2.7893	0.0052826	0.0264358	ENSG00000259580	15	39770558	39801333	-
ENST00000558833	10.44477012	3.214361628	1.18335	2.7163	0.0066009	0.0315543	ENSG00000259657	15	59676756	59677321	PIGHP1
ENST00000559000	259.3583032	-1.003435115	0.25488	-3.937	8.25E-05	0.0008355	ENSG00000285253	15	56652487	56918571	-
ENST00000559052	9.063092852	6.603810156	1.82587	3.6168	0.0002983	0.0024836	ENSG00000259186	15	73483195	73483943	MRPS15P1
ENST00000559239	259.6995872	-1.172088002	0.26651	-4.398	1.09E-05	0.0001452	ENSG00000140451	15	64815631	64825647	PIF1
ENST00000559321	50.87728967	-1.252625819	0.48488	-2.583	0.0097845	0.0430875	ENSG00000259485	15	97215811	97432086	LINC02253
ENST00000559594	3.893643431	5.3891778	2.12058	2.5414	0.0110418	0.0473143	ENSG00000259502	14	67610985	67613864	-
ENST00000559717	1132.845186	1.335867701	0.21087	6.3349	2.37E-10	9.58E-09	ENSG00000196547	15	90903301	90922579	MAN2A2
ENST00000560489	35.41992232	1.482977724	0.56675	2.6166	0.0088806	0.0399148	ENSG00000259531	15	49365123	49366685	-

protein_coding	Yes	NM_003006.4	108
protein_coding	Yes	NM_001348022.3	108
protein_coding	Yes	NM_001270471.2	108
protein_coding	Yes	NM_002272.4	108
protein_coding	Yes	NM_007076.3	108
protein_coding	Yes	NM_025114.4	108
protein_coding	Yes	NM_001310135.5	108
protein_coding	Yes	NM_005589.4	108
lncRNA	Yes	-	108
ed_unprocessed_ps	Yes	-	108
protein_coding	Yes	NM_006370.3	108
lncRNA	Yes	-	108
protein_coding	Yes	NM_183387.3	108
ocessed_pseudoge	Yes	-	108
lncRNA	Yes	-	108
protein_coding	Yes	NM_002386.4	108
protein_coding	Yes	NM_152446.5	108
protein_coding	Yes	-	108
protein_coding	Yes	NM_001032283.3	108
protein_coding	Yes	NM_021260.4	108
protein_coding	Yes	NM_001320329.2	108
protein_coding	Yes	-	108
protein_coding	Yes	NM_001280542.3	108
protein_coding	Yes	NM_001085481.3	108
lncRNA	Yes	-	108
lncRNA	Yes	-	108
protein_coding	Yes	NM_016029.4	108
lncRNA	Yes	-	108
ped_processed_pse	Yes	-	108
protein_coding	Yes	NM_001145648.3	108
protein_coding	Yes	NM_001301059.2	108
lncRNA	Yes	-	108
ocessed_pseudoge	Yes	-	108
protein_coding	Yes	-	108
ocessed_pseudoge	Yes	-	108
protein_coding	Yes	NM_001286496.2	108
lncRNA	Yes	-	108
ocessed_pseudoge	Yes	-	108
protein_coding	Yes	NM_006122.4	108
ocessed_pseudoge	Yes	-	108

ENST00000560794	4.872759857	5.712624018	2.00261	2.8526	0.0043365	0.0225272	ENSG00000259637	15	82531652	82534139	DNM1P38
ENST00000561100	309.4826229	1.357770674	0.25979	5.2264	1.73E-07	3.77E-06	ENSG00000166073	15	39799007	39920266	GPR176
ENST00000561652	51.45075774	-1.567727018	0.48257	-3.249	0.0011593	0.0077029	ENSG00000260966	11	103050686	103055799	-
ENST00000561678	580.3282706	-1.833008823	0.21128	-8.676	4.11E-18	5.81E-16	ENSG00000121274	16	50153305	50235310	TENT4B
ENST00000561705	18.44232423	-7.679588552	1.61356	-4.759	1.94E-06	3.21E-05	ENSG00000260265	4	75081701	75084717	LINC02562
ENST00000562057	3950.518449	1.131150937	0.17079	6.623	3.52E-11	1.72E-09	ENSG00000166145	15	40844047	40858207	SPINT1
ENST00000562102	214.173782	1.104136789	0.29439	3.7506	0.0001764	0.0015931	ENSG00000166669	16	10386060	10483638	ATF7IP2
ENST00000562280	12.14860058	4.512915957	1.35799	3.3232	0.0008898	0.0061872	ENSG00000260034	16	25140576	25149032	LCMT1-AS2
ENST00000562532	21.19316289	3.685912064	0.90532	4.0714	4.67E-05	0.0005129	ENSG00000260903	20	31968150	32003387	XKR7
ENST00000562631	365.6461738	-1.418609159	0.3132	-4.529	5.92E-06	8.51E-05	ENSG00000205336	16	57628629	57665567	ADGRG1
ENST00000562782	4.149833674	5.480651158	2.08437	2.6294	0.0085535	0.0387013	ENSG00000261546	16	89113174	89115279	-
ENST00000562783	48.43900856	2.201608853	0.54255	4.0579	4.95E-05	0.0005392	ENSG00000178115	15	30552049	30565684	GOLGA8Q
ENST00000562834	188.6817617	-1.443656268	0.28719	-5.027	4.99E-07	9.69E-06	ENSG00000261116	6	54943166	54945099	-
ENST00000563027	13.76154038	2.743537994	1.00903	2.719	0.0065482	0.0313552	ENSG00000188626	15	28698582	28712421	GOLGA8M
ENST00000563225	62.69568658	1.580623493	0.44776	3.5301	0.0004154	0.0032823	ENSG00000261211	6	6680308	6686948	-
ENST00000563377	79.98200929	1.092196743	0.39175	2.788	0.0053036	0.0265077	ENSG00000214331	16	74332566	74360859	PDPR2P
ENST00000563449	25.87085738	2.327775423	0.71824	3.2409	0.0011914	0.0078805	ENSG00000261613	16	2554059	2556060	-
ENST00000563594	176.1147063	-1.552180762	0.3093	-5.018	5.21E-07	1.01E-05	ENSG00000140995	16	89948754	89968060	DEF8
ENST00000563777	101.2478692	1.865094167	0.38011	4.9067	9.26E-07	1.67E-05	ENSG00000260852	16	30919318	30923269	FBXL19-AS1
ENST00000563867	6.341434496	6.092535286	1.88786	3.2272	0.00125	0.0081997	ENSG00000214646	15	77941441	77942747	COMMD4P1
ENST00000563962	22.44248852	2.069156919	0.73647	2.8096	0.0049609	0.0251185	ENSG00000260448	16	25085167	25111539	LCMT1-AS1
ENST00000564224	82.42036648	1.850628456	0.40236	4.5995	4.24E-06	6.36E-05	ENSG00000261215	9	34661902	34666029	-
ENST00000564385	4.616569613	5.635108028	2.03024	2.7756	0.0055102	0.0273019	ENSG00000260611	16	35785568	35786887	-
ENST00000564629	89.42655814	1.750917876	0.38379	4.5622	5.06E-06	7.44E-05	ENSG00000260267	16	31456710	31459736	-
ENST00000564741	4.383201644	5.55994647	2.05576	2.7046	0.0068394	0.0324384	ENSG00000261037	5	6019028	6022283	-
ENST00000565246	340.0930769	2.032495133	0.24803	8.1946	2.51E-16	2.83E-14	ENSG00000261556	16	70219869	70246610	SMG1P7
ENST00000565433	31.48541873	-3.276410097	0.6891	-4.755	1.99E-06	3.28E-05	ENSG00000260401	11	73238974	73242335	-
ENST00000565563	4.70785871	5.660506682	2.03011	2.7883	0.005299	0.0264916	ENSG00000260179	1	1249776	1251334	-
ENST00000565617	39.44356632	1.588722359	0.54228	2.9297	0.0033929	0.0184948	ENSG00000261087	8	101166804	101169629	ZNNT1
ENST00000566085	5.783409461	5.961399914	1.93554	3.08	0.0020702	0.0123689	ENSG00000260874	16	2456251	2459966	-
ENST00000566155	4.593747339	5.6286836	2.037	2.7632	0.0057235	0.0281071	ENSG00000260166	16	88087055	88089975	-
ENST00000566157	396.6372944	-1.522980733	0.25648	-5.938	2.89E-09	9.48E-08	ENSG00000087263	16	56451553	56479104	OGFOD1
ENST00000566383	10.67945892	-6.890458721	1.73181	-3.979	6.93E-05	0.0007209	ENSG00000261804	16	53373478	53385142	-
ENST00000566699	96.68635993	3.102898491	0.4145	7.4859	7.11E-14	5.80E-12	ENSG00000260231	7	140177183	140179640	KDM7A-DT
ENST00000566732	253.7768944	1.032148522	0.28039	3.6811	0.0002322	0.0020133	ENSG00000103168	16	84177854	84187057	TAF1C
ENST00000566740	17.77894157	2.961429316	0.87826	3.3719	0.0007465	0.0053684	ENSG00000261794	15	30604027	30617752	GOLGA8H
ENST00000566802	82.69190694	1.101226753	0.38613	2.852	0.0043446	0.0225574	ENSG00000290890	16	74332401	74368161	-
ENST00000567180	42.39705932	2.191634486	0.56616	3.8711	0.0001084	0.001049	ENSG00000260428	8	144266452	144268481	SCX
ENST00000567305	5.897520832	5.986731667	1.91891	3.1199	0.0018094	0.0110864	ENSG00000261379	2	226804035	226805061	-
ENST00000567458	5.128950101	5.786184995	1.97811	2.9251	0.0034433	0.0186933	ENSG00000259303	16	32847712	32848156	IGHV2OR16-5

rocessed_pseudog	Yes	-	108
protein_coding	Yes	NM_007223.3	108
lncRNA	Yes	-	108
protein_coding	Yes	NM_001365324.3	108
lncRNA	Yes	-	108
protein_coding	Yes	NM_003710.4	108
protein_coding	Yes	NM_001393719.1	108
lncRNA	Yes	-	108
protein_coding	Yes	NM_001011718.2	108
protein_coding	Yes	NM_201525.4	108
lncRNA	Yes	-	108
protein_coding	Yes	NM_001355476.2	108
lncRNA	Yes	-	108
protein_coding	Yes	NM_001282468.3	108
lncRNA	Yes	-	108
ed_unprocessed_ps	Yes	-	108
lncRNA	Yes	-	108
protein_coding	Yes	NM_001242818.2	108
lncRNA	Yes	-	108
ed_unprocessed_ps	Yes	-	108
lncRNA	Yes	-	108
lncRNA	Yes	-	108
lncRNA	Yes	-	108
lncRNA	Yes	-	108
lncRNA	Yes	-	108
ed_unprocessed_ps	Yes	-	108
lncRNA	Yes	-	108
lncRNA	Yes	-	108
lncRNA	Yes	-	108
lncRNA	Yes	-	108
lncRNA	Yes	-	108
protein_coding	Yes	NM_018233.4	108
lncRNA	Yes	-	108
lncRNA	Yes	-	108
protein_coding	Yes	NM_001243156.2	108
protein_coding	Yes	NM_001282490.2	108
lncRNA	Yes	-	108
protein_coding	Yes	NM_001080514.3	108
lncRNA	Yes	-	108
IG_V_gene	Yes	-	108

ENST00000567563	21.52517655	-6.451224887	1.66932	-3.865	0.0001113	0.001073	ENSG00000259827	16	56682469	56687807	-
ENST00000567572	83.30928396	-2.802032099	0.44227	-6.336	2.36E-10	9.55E-09	ENSG00000140931	16	66604703	66613887	CMTM3
ENST00000567701	129.8910567	-1.073392055	0.33828	-3.173	0.0015082	0.0095653	ENSG00000234337	16	52655306	52656045	-
ENST00000567819	48.72989104	1.893548147	0.51575	3.6714	0.0002412	0.0020798	ENSG00000260942	2	240582699	240586699	CAPN10-DT
ENST00000568093	10.08785383	6.758305894	1.80499	3.7442	0.0001809	0.001628	ENSG00000260648	15	40232081	40236109	-
ENST00000568150	6.93110782	-4.759039391	1.87626	-2.536	0.0111985	0.0478528	ENSG00000260086	16	48623414	48746318	-
ENST00000568184	4.383201644	5.55994647	2.05576	2.7046	0.0068394	0.0324384	ENSG00000290666	15	77941898	77944582	-
ENST00000568248	293.5015151	-2.096674603	0.32588	-6.434	1.24E-10	5.37E-09	ENSG00000259820	8	134792019	134798272	MIR30DHG
ENST00000568449	664.0808968	1.660644521	0.20833	7.9712	1.57E-15	1.60E-13	ENSG00000197774	16	1772809	1781702	EME2
ENST00000568855	230.6619293	2.09386217	0.27987	7.4816	7.34E-14	5.97E-12	ENSG00000291219	16	70229803	70246612	-
ENST00000568943	5.106127827	5.780412549	1.98129	2.9175	0.0035284	0.0190517	ENSG00000103546	16	55655987	55706192	SLC6A2
ENST00000569046	557.534075	-1.598984202	0.24412	-6.55	5.75E-11	2.65E-09	ENSG00000259781	15	71164769	71165415	HMGB1P6
ENST00000569134	15.13890353	-2.751661941	0.92121	-2.987	0.0028171	0.0158734	ENSG00000260317	8	80541299	80543104	LINC02986
ENST00000569188	30.82891803	4.537738597	0.92585	4.9011	9.53E-07	1.71E-05	ENSG00000103044	16	69105652	69117660	HAS3
ENST00000569580	5.407962619	5.8616559	1.95628	2.9963	0.0027326	0.0155021	ENSG00000260927	16	58129518	58163246	-
ENST00000569831	7.576741166	6.348994831	1.82299	3.4827	0.0004963	0.0038137	ENSG00000260425	16	1358899	1361405	-
ENST00000569969	686.6104069	1.102510705	0.21578	5.1095	3.23E-07	6.63E-06	ENSG00000261067	16	28974803	28990775	-
ENST00000570010	168.0225681	-1.262878555	0.30676	-4.117	3.84E-05	0.0004329	ENSG00000184517	16	75148523	75172234	ZFP1
ENST00000570054	325.7084305	-1.039679022	0.27731	-3.749	0.0001774	0.0016012	ENSG00000260914	16	69299681	69322700	-
ENST00000570444	3.916465705	5.396742222	2.11677	2.5495	0.0107872	0.0464746	ENSG00000262624	17	7436556	7437523	-
ENST00000571340	11.7033664	-7.022470355	1.71127	-4.104	4.07E-05	0.0004549	ENSG00000262714	16	53386943	53389085	-
ENST00000572272	37.40427176	2.154843941	0.59622	3.6141	0.0003013	0.0025047	ENSG00000091592	17	5514117	5584509	NLRP1
ENST00000572447	368.709012	-2.152700395	0.24961	-8.624	6.46E-18	9.01E-16	ENSG00000129195	17	6444454	6451469	PIMREG
ENST00000573312	67.0446495	1.850148804	0.52698	3.5109	0.0004466	0.0034896	ENSG00000261888	17	83104254	83106910	-
ENST00000573318	91.34858186	1.423189889	0.38104	3.735	0.0001877	0.001679	ENSG00000228782	17	47482991	47492459	-
ENST00000573622	58.21283747	-1.201384273	0.45856	-2.62	0.008796	0.0396185	ENSG00000272917	12	9642871	9657070	-
ENST00000574428	261.9242993	-2.193632716	0.27933	-7.853	4.06E-15	3.95E-13	ENSG00000123472	1	46632736	46668364	ATPAF1
ENST00000574616	1549.113925	-1.558261111	0.19788	-7.875	3.41E-15	3.36E-13	ENSG00000263244	16	9104847	9113181	-
ENST00000575018	82.14096631	-1.889911061	0.41587	-4.544	5.51E-06	8.00E-05	ENSG00000262519	17	4572205	4572515	TXNP4
ENST00000575094	182.8000437	-1.480533102	0.30121	-4.915	8.87E-07	1.60E-05	ENSG00000291107	12	9648046	9658412	-
ENST00000575310	64.43381864	1.985836335	0.49264	4.031	5.55E-05	0.0005949	ENSG00000262880	17	7420102	7444081	-
ENST00000575331	158.4598879	1.063747915	0.32663	3.2567	0.0011271	0.0075307	ENSG00000272884	17	7439505	7445966	-
ENST00000576060	5.613363086	5.911749373	2.10274	2.8115	0.0049318	0.025002	ENSG00000182896	17	7355155	7357219	TMEM95
ENST00000576634	125.1935787	-1.901905446	0.34314	-5.543	2.98E-08	7.77E-07	ENSG00000103351	16	3501003	3539048	CLUAP1
ENST00000576742	324.4802694	2.185997948	0.2558	8.5458	1.28E-17	1.71E-15	ENSG00000167723	17	3510501	3557812	TRPV3
ENST00000577135	6.039599704	6.023575137	1.9141	3.147	0.0016498	0.0102909	ENSG00000262497	19	35232290	35233003	FAM187B2P
ENST00000577164	290.132069	1.519138003	0.26367	5.7615	8.34E-09	2.49E-07	ENSG00000186594	17	1711502	1717174	MIR22HG
ENST00000577502	3.916465705	5.396742222	2.11677	2.5495	0.0107872	0.0464746	ENSG00000284575	3	48644193	48644280	MIR4793
ENST00000577660	101.4180176	-1.022396808	0.35383	-2.89	0.0038585	0.0205005	ENSG00000265303	17	59197565	59215226	-
ENST00000577781	68.16411664	4.075783334	0.57043	7.1451	8.99E-13	6.15E-11	ENSG00000263826	3	186781779	186784179	-

lncRNA	Yes	-	108
protein_coding	Yes	NM_181553.4	108
ocessed_pseudoge	Yes	-	108
lncRNA	Yes	-	108
lncRNA	Yes	-	108
lncRNA	Yes	-	108
lncRNA	Yes	-	108
lncRNA	Yes	-	108
protein_coding	Yes	NM_001257370.2	108
lncRNA	Yes	-	108
protein_coding	Yes	NM_001172501.3	108
ocessed_pseudoge	Yes	-	108
lncRNA	Yes	-	108
protein_coding	Yes	NM_001199280.2	108
lncRNA	Yes	-	108
lncRNA	Yes	-	108
lncRNA	Yes	-	108
protein_coding	Yes	NM_153688.4	108
protein_coding	Yes	-	108
lncRNA	Yes	-	108
lncRNA	Yes	-	108
protein_coding	Yes	NM_033004.4	108
protein_coding	Yes	NM_019013.3	108
lncRNA	Yes	-	108
ed_unprocessed_ps	Yes	-	108
lncRNA	Yes	-	108
protein_coding	Yes	NM_001394565.1	108
lncRNA	Yes	-	108
ocessed_pseudoge	Yes	-	108
lncRNA	Yes	-	108
lncRNA	Yes	-	108
lncRNA	Yes	-	108
protein_coding	Yes	NM_001320436.2	108
protein_coding	Yes	NM_015041.3	108
protein_coding	Yes	NM_145068.4	108
rocessed_pseudog	Yes	-	108
lncRNA	Yes	-	108
miRNA	Yes	-	108
protein_coding	Yes	-	108
lncRNA	Yes	-	108

ENST00000577809	222.378655	-2.865869022	0.2949	-9.718	2.52E-22	6.14E-20	ENSG00000108666	17	32328440	32342173	C17orf75
ENST00000577863	5.829054009	5.971610018	1.92358	3.1044	0.0019065	0.0115479	ENSG00000179136	17	12549940	12642854	LINC00670
ENST00000577970	59.28133484	5.846633617	0.83645	6.9898	2.75E-12	1.71E-10	ENSG00000263567	17	31762439	31769048	-
ENST00000577988	169.1091395	-1.126967494	0.29467	-3.824	0.0001311	0.0012356	ENSG00000265185	17	19061911	19062669	SNORD3B-1
ENST00000578186	4755.029862	-1.356984575	0.18927	-7.17	7.52E-13	5.21E-11	ENSG00000270882	1	149832656	149833052	H4C14
ENST00000579209	65.38645317	1.277055099	0.4772	2.6761	0.0074474	0.0346279	ENSG00000263647	17	64761911	64762710	BPTFP1
ENST00000579212	91.449446	-1.25782203	0.39283	-3.202	0.0013649	0.0088084	ENSG00000284368	17	64501213	64501313	MIR5047
ENST00000579774	724.2290735	2.40962785	0.24374	9.886	4.79E-23	1.31E-20	ENSG00000265107	1	147756198	147760602	GJA5
ENST00000579849	21.40435559	-4.050828562	0.92022	-4.402	1.07E-05	0.0001428	ENSG00000006042	17	32928152	32944315	TMEM98
ENST00000580114	27.07215164	1.852844895	0.70467	2.6294	0.0085539	0.0387013	ENSG00000265690	16	67163384	67165808	-
ENST00000580170	35.52445805	-8.624785781	1.54248	-5.591	2.25E-08	6.04E-07	ENSG00000173482	18	7567315	8406856	PTPRM
ENST00000580533	314.6699586	2.015116462	0.35166	5.7303	1.00E-08	2.92E-07	ENSG00000264940	17	19189664	19190245	SNORD3C
ENST00000581050	90.43608288	1.908108066	0.39898	4.7825	1.73E-06	2.90E-05	ENSG00000291263	16	70219580	70226033	SMG1P7
ENST00000581792	626.3758359	4.458726299	0.27422	16.26	1.91E-59	8.57E-56	ENSG00000264462	21	8986998	8987178	MIR3648-2
ENST00000581978	5.857021511	5.982236288	2.07982	2.8763	0.0040233	0.0212165	ENSG00000266467	10	68007717	68008017	RN7SL220P
ENST00000582263	4.383201644	5.55994647	2.05576	2.7046	0.0068394	0.0324384	ENSG00000264968	17	39927866	39939601	-
ENST00000582558	8.647146689	6.538091578	1.78835	3.6559	0.0002563	0.002193	ENSG00000264569	17	82037871	82039380	DCXR-DT
ENST00000582866	126.3133377	-1.064529896	0.32689	-3.257	0.0011277	0.0075338	ENSG00000263731	17	81878424	81881106	-
ENST00000582970	3367.312113	1.807922716	0.59467	3.0402	0.0023643	0.0137837	ENSG00000173821	17	80260851	80398794	RNF213
ENST00000583467	9.794204051	5.234538996	1.72627	3.0323	0.0024271	0.0140825	ENSG00000265345	12	124915546	124915659	MIR5188
ENST00000583931	2198.309778	-2.25653124	0.19063	-11.84	2.50E-32	1.80E-29	ENSG00000143401	1	150218416	150236112	ANP32E
ENST00000585281	137.4381307	-2.078210934	0.39712	-5.233	1.67E-07	3.65E-06	ENSG00000265190	10	47467992	47484115	ANXA8
ENST00000585482	40.31227456	2.786578476	0.6357	4.3835	1.17E-05	0.0001538	ENSG00000004139	17	28371693	28404049	SARM1
ENST00000585682	338.7586388	-2.966575509	0.27009	-10.98	4.58E-28	2.28E-25	ENSG00000164125	4	158124473	158173025	GASK1B
ENST00000585807	196.1812906	1.371687494	0.29167	4.7029	2.57E-06	4.11E-05	ENSG00000108786	17	42552922	42555214	HSD17B1
ENST00000586076	11.29178054	3.780265695	1.24717	3.0311	0.0024369	0.0141264	ENSG00000267801	17	75876371	75879546	-
ENST00000586569	19.88648003	-2.264744783	0.77538	-2.921	0.0034911	0.018893	ENSG00000141655	18	62325309	62391288	TNFRSF11A
ENST00000586606	78.75841088	1.179917262	0.41813	2.8219	0.0047742	0.0243421	ENSG00000267552	19	37689882	37719125	-
ENST00000587128	7.416985247	6.321223928	1.85635	3.4052	0.0006612	0.0048445	ENSG00000267058	19	43891803	43901805	-
ENST00000587230	574.116152	1.066514385	0.20983	5.0827	3.72E-07	7.50E-06	ENSG00000104915	19	13144057	13150375	STX10
ENST00000587682	107.6929468	1.283922109	0.4186	3.0672	0.0021609	0.0128149	ENSG00000159905	19	43951227	43967709	ZNF221
ENST00000588387	24.38466893	2.327014529	0.71113	3.2723	0.0010669	0.0071884	ENSG00000267169	19	14137145	14171264	ADGRL1-AS1
ENST00000588776	93.16432815	-2.437063154	0.47282	-5.154	2.55E-07	5.36E-06	ENSG00000105519	19	5914253	5916211	CAPS
ENST00000588789	4.593747339	5.6286836	2.037	2.7632	0.0057235	0.0281071	ENSG00000290719	19	9265473	9268819	-
ENST00000588891	24.73377473	-2.63700739	0.726	-3.632	0.000281	0.00236	ENSG00000267314	19	5904840	5914707	-
ENST00000588981	118.390831	-1.987077153	0.40954	-4.852	1.22E-06	2.13E-05	ENSG00000105048	19	55132697	55149206	TNNT1
ENST00000589942	4.593747339	5.6286836	2.037	2.7632	0.0057235	0.0281071	ENSG00000225980	19	9265439	9266283	OR7E19P
ENST00000590474	117.348811	-4.516525037	0.50806	-8.89	6.12E-19	9.53E-17	ENSG00000108984	17	69414696	69553865	MAP2K6
ENST00000590508	252.5324263	2.167501106	0.30715	7.0569	1.70E-12	1.11E-10	ENSG00000132003	19	13795442	13832254	ZSWIM4
ENST00000590513	213.3027258	1.08469533	0.27709	3.9146	9.05E-05	0.000904	ENSG00000266962	17	42552430	42554841	HSD17B1-AS1

protein_coding	Yes	NM_022344.4	108
lncRNA	Yes	-	108
lncRNA	Yes	-	108
snoRNA	Yes	-	108
protein_coding	Yes	NM_003548.2	108
rocessed_pseudog	Yes	-	108
miRNA	Yes	-	108
protein_coding	Yes	NM_181703.4	108
protein_coding	Yes	NM_015544.3	108
protein_coding	Yes	-	108
protein_coding	Yes	NM_001105244.2	108
snoRNA	Yes	-	108
lncRNA	Yes	-	108
miRNA	Yes	-	108
misc_RNA	Yes	-	108
lncRNA	Yes	-	108
lncRNA	Yes	-	108
lncRNA	Yes	-	108
protein_coding	Yes	NM_001256071.3	108
miRNA	Yes	-	108
protein_coding	Yes	NM_030920.5	108
protein_coding	Yes	NM_001040084.3	108
protein_coding	Yes	NM_015077.4	108
protein_coding	Yes	NM_001128424.2	108
protein_coding	Yes	NM_000413.4	108
lncRNA	Yes	-	108
protein_coding	Yes	NM_003839.4	108
protein_coding	Yes	-	108
lncRNA	Yes	-	108
protein_coding	Yes	NM_003765.3	108
protein_coding	Yes	NM_001297588.2	108
lncRNA	Yes	-	108
protein_coding	Yes	NM_004058.5	108
lncRNA	Yes	-	108
protein_coding	Yes	-	108
protein_coding	Yes	NM_003283.6	108
d_unprocessed_ps	Yes	-	108
protein_coding	Yes	NM_002758.4	108
protein_coding	Yes	NM_001367834.3	108
lncRNA	Yes	-	108

ENST00000590734	42.08398041	1.351476661	0.52231	2.5875	0.0096677	0.0426681	ENSG00000237854	17	68101907	68114527	LINC00674
ENST00000590949	30.25875809	1.905740142	0.66638	2.8598	0.0042388	0.0221325	ENSG00000126561	17	42288497	42311943	STAT5A
ENST00000591022	605.3402633	1.667455219	0.24788	6.7269	1.73E-11	9.06E-10	ENSG00000068137	17	42667913	42676994	PLEKHH3
ENST00000591504	36.5921067	-3.390934024	0.65062	-5.212	1.87E-07	4.05E-06	ENSG00000166510	18	54901508	54959461	CCDC68
ENST00000592087	23.24243032	2.15089885	0.7256	2.9643	0.0030338	0.016845	ENSG00000267309	19	36489648	36491040	ZNF566-AS1
ENST00000592431	262.537869	3.765742902	0.30656	12.284	1.11E-34	1.34E-31	ENSG00000267475	19	32686917	32691764	NUDT19-DT
ENST00000592540	645.0987147	-1.229872255	0.24008	-5.123	3.01E-07	6.22E-06	ENSG00000161888	19	11145492	11155782	SPC24
ENST00000592792	599.1826214	-1.031477734	0.20711	-4.98	6.35E-07	1.19E-05	ENSG00000196922	8	144976189	144999723	ZNF252P
ENST00000593537	124.1237491	1.163092064	0.34993	3.3238	0.000888	0.0061777	ENSG00000225950	19	49061127	49064818	NTF4
ENST00000594254	68.16452109	1.492213793	0.43418	3.4368	0.0005886	0.0043998	ENSG00000213976	19	21382864	21387177	-
ENST00000594653	65.50889764	1.33380902	0.44391	3.0047	0.0026583	0.0151549	ENSG00000269107	19	23257254	23274232	-
ENST00000595224	26.02011496	3.774862192	0.80963	4.6624	3.12E-06	4.87E-05	ENSG00000269313	X	49162757	49168774	MAGIX
ENST00000595562	130.623409	1.15526807	0.34545	3.3443	0.000825	0.0058219	ENSG00000268870	19	12391948	12441082	-
ENST00000595607	532.4110259	4.805305516	0.28697	16.745	6.17E-63	3.14E-59	ENSG00000178150	19	48270080	48287608	ZNF114
ENST00000596288	1837.255926	-1.165960977	0.22532	-5.175	2.28E-07	4.85E-06	ENSG00000112118	6	52264014	52284742	MCM3
ENST00000596326	7.323590711	4.801434251	1.8106	2.6518	0.0080053	0.0367236	ENSG00000269289	19	23919080	23957930	-
ENST00000596946	3.916465705	5.396742222	2.11677	2.5495	0.0107872	0.0464746	ENSG00000267852	19	7390521	7395039	ARHGEF18-AS1
ENST00000597126	5.220239197	5.809124567	1.98721	2.9233	0.0034639	0.018777	ENSG00000268598	19	21063923	21064261	VN1R80P
ENST00000597229	125.0667588	-2.831579207	0.36378	-7.784	7.03E-15	6.57E-13	ENSG00000198816	19	7516102	7521025	ZNF358
ENST00000597336	75.79206093	1.772292047	0.41035	4.319	1.57E-05	0.0001986	ENSG00000269069	19	40023383	40025502	-
ENST00000597420	3.784349268	-5.394885692	2.13765	-2.524	0.0116112	0.0492063	ENSG00000269564	19	53788781	53789168	-
ENST00000597850	12.00558736	7.012693587	1.6948	4.1378	3.51E-05	0.0003996	ENSG00000171649	19	57584144	57593890	ZIK1
ENST00000598418	126.0235253	1.199450583	0.33569	3.573	0.0003528	0.0028579	ENSG00000161677	19	50505997	50511191	JOSD2
ENST00000598887	4.149833674	5.480651158	2.08437	2.6294	0.0085535	0.0387013	ENSG00000268475	19	41425358	41426237	-
ENST00000599312	31.24256762	-3.144921543	0.67836	-4.636	3.55E-06	5.44E-05	ENSG00000267952	19	7507081	7519526	-
ENST00000599795	69.94298507	1.254625024	0.47649	2.6331	0.0084615	0.0383864	ENSG00000283663	19	49055792	49065076	-
ENST00000599889	6.892069865	-4.75201281	1.84504	-2.576	0.0100078	0.0438636	ENSG00000269054	19	58362568	58367620	ZNF497-AS1
ENST00000599921	171.7496394	4.897126147	0.42917	11.411	3.70E-30	2.24E-27	ENSG00000105499	19	48047845	48110817	PLA2G4C
ENST00000599944	8.231200526	6.469917093	1.80057	3.5933	0.0003266	0.0026796	ENSG00000267924	19	23817598	23874701	-
ENST00000600303	18.41361844	2.824648907	0.84652	3.3368	0.0008476	0.0059503	ENSG00000268093	19	48619271	48623858	-
ENST00000600737	2209.346269	1.508851187	0.18393	8.2035	2.34E-16	2.64E-14	ENSG00000032444	19	7535716	7561764	PNPLA6
ENST00000600996	108.3075596	-1.060045754	0.37315	-2.841	0.0045003	0.0232047	ENSG00000268034	19	41506151	41506898	TPM3P5
ENST00000601870	289.9924445	1.456859374	0.25419	5.7313	9.97E-09	2.91E-07	ENSG00000268614	19	7533594	7540059	-
ENST00000602172	49.25324181	1.395501873	0.50591	2.7584	0.0058085	0.0284463	ENSG00000268001	19	48255674	48258199	CARD8-AS1
ENST00000602255	6.17653335	6.052649114	1.90546	3.1765	0.0014907	0.0094747	ENSG00000267892	19	38738283	38739863	-
ENST00000602478	94.82178986	-1.881486887	0.37949	-4.958	7.12E-07	1.32E-05	ENSG00000270022	22	42615243	42615907	-
ENST00000602508	7.064360679	6.248469536	1.84774	3.3817	0.0007204	0.0052065	ENSG00000269911	X	72777607	72779097	FAM226B
ENST00000602575	28.35484899	-1.650148878	0.63165	-2.612	0.0089899	0.0402994	ENSG00000269918	8	11104690	11106704	-
ENST00000602739	11.38610943	3.338156294	1.19537	2.7926	0.0052293	0.0262271	ENSG00000270020	16	86520382	86523897	-
ENST00000602787	55.92486802	-1.532083538	0.46677	-3.282	0.0010295	0.0069779	ENSG00000251503	1	10430434	10451411	CENPS-CORT

ed_unprocessed_ps	Yes	-	108
protein_coding	Yes	NM_001288718.2	108
protein_coding	Yes	NM_024927.5	108
protein_coding	Yes	NM_025214.3	108
lncRNA	Yes	-	108
lncRNA	Yes	-	108
protein_coding	Yes	NM_182513.4	108
ed_unprocessed_ps	Yes	-	108
protein_coding	Yes	NM_006179.5	108
rocessed_pseudog	Yes	-	108
lncRNA	Yes	-	108
protein_coding	Yes	NM_024859.4	108
protein_coding	Yes	-	108
protein_coding	Yes	NM_153608.4	108
protein_coding	Yes	NM_002388.6	108
lncRNA	Yes	-	108
lncRNA	Yes	-	108
rocessed_pseudog	Yes	-	108
protein_coding	Yes	NM_018083.5	108
ocessed_pseudoge	Yes	-	108
lncRNA	Yes	-	108
protein_coding	Yes	NM_001010879.4	108
protein_coding	Yes	NM_001270639.2	108
lncRNA	Yes	-	108
protein_coding	Yes	-	108
protein_coding	Yes	-	108
lncRNA	Yes	-	108
protein_coding	Yes	NM_003706.3	108
lncRNA	Yes	-	108
lncRNA	Yes	-	108
protein_coding	Yes	NM_001166114.2	108
ocessed_pseudoge	Yes	-	108
protein_coding	Yes	-	108
lncRNA	Yes	-	108
lncRNA	Yes	-	108
lncRNA	Yes	-	108
lncRNA	Yes	-	108
lncRNA	Yes	-	108
lncRNA	Yes	-	108
protein_coding	Yes	-	108

ENST00000602813	33.36955135	-1.789600806	0.61513	-2.909	0.0036223	0.0194635	ENSG00000270103	1	28648599	28648730	-
ENST00000603208	32.75077852	2.200118139	0.67792	3.2454	0.001173	0.0077805	ENSG00000270393	22	19018042	19018916	-
ENST00000603357	327.8327431	-1.781140642	0.25811	-6.901	5.17E-12	3.03E-10	ENSG00000180346	4	89111532	89114901	TIGD2
ENST00000603504	37.69665181	2.06190433	0.58345	3.534	0.0004093	0.0032424	ENSG00000271394	17	35889557	35889804	7SK
ENST00000603536	5.339495796	5.845156443	1.96201	2.9792	0.0028903	0.0162004	ENSG00000271142	12	76619385	76620043	YWHAQP7
ENST00000603540	22.08994634	2.943721325	0.79948	3.682	0.0002314	0.0020072	ENSG00000139985	14	70452173	70459905	ADAM21
ENST00000603791	27.81359948	1.998215186	0.65208	3.0644	0.0021814	0.0129162	ENSG00000270504	6	3751110	3753871	-
ENST00000603928	129.027325	-1.239077953	0.32961	-3.759	0.0001705	0.0015487	ENSG00000235106	9	134025480	134031587	BRD3OS
ENST00000604030	612.0860396	-1.173564193	0.21434	-5.475	4.37E-08	1.10E-06	ENSG00000109686	4	151120280	151325605	SH3D19
ENST00000604404	378.2719677	-1.30979366	0.28726	-4.56	5.12E-06	7.52E-05	ENSG00000089356	19	35115822	35124324	FXYD3
ENST00000604716	34.46047913	2.063874442	0.67173	3.0725	0.0021229	0.0126277	ENSG00000270605	1	28239508	28241453	-
ENST00000604729	1497.759343	2.372780472	0.19981	11.875	1.59E-32	1.18E-29	ENSG00000214655	10	73785605	73801793	ZSWIM8
ENST00000604818	12.76273876	-3.608053048	1.11867	-3.225	0.0012584	0.0082456	ENSG00000270659	2	213151971	213153661	-
ENST00000604845	37.59081551	-1.8028766	0.58176	-3.099	0.0019417	0.0117227	ENSG00000270696	2	75660461	75662208	-
ENST00000605757	7.297728649	6.295565165	1.83679	3.4275	0.0006092	0.004523	ENSG00000270326	6	28319659	28319852	SMIM15P2
ENST00000606025	57.87064836	-2.018552718	0.46692	-4.323	1.54E-05	0.0001955	ENSG00000258881	2	70939317	70995336	-
ENST00000606162	4.509988757	-5.648997521	2.05305	-2.752	0.0059321	0.0289538	ENSG00000122378	10	80408550	80437115	PRXL2A
ENST00000606173	100.2596561	1.194759083	0.38427	3.1092	0.0018759	0.0114017	ENSG00000290683	1	247199398	247210856	-
ENST00000606253	74.11106317	2.14927438	0.42338	5.0765	3.85E-07	7.71E-06	ENSG00000167604	19	35888240	35902303	NFKBID
ENST00000606379	248.4273253	1.468284871	0.26396	5.5624	2.66E-08	7.02E-07	ENSG00000272084	1	19072109	19075511	-
ENST00000606432	9.317332008	3.51552763	1.31917	2.665	0.0076997	0.0355749	ENSG00000272217	6	33246074	33246856	-
ENST00000606924	65.49845021	1.178111219	0.45283	2.6016	0.0092779	0.0413019	ENSG00000272341	6	16764345	16766883	-
ENST00000607355	605.1596872	-1.682149621	0.23513	-7.154	8.41E-13	5.77E-11	ENSG00000288859	1	149851060	149851594	H2AC19
ENST00000607357	114.6439026	-1.302551527	0.37339	-3.488	0.0004859	0.0037471	ENSG00000172428	2	240130827	240136305	COPS9
ENST00000607452	103.011534	1.098573734	0.35446	3.0993	0.0019397	0.0117133	ENSG00000170634	2	53971112	54305300	ACYP2
ENST00000607496	10.22520235	-5.360419569	1.73487	-3.09	0.0020028	0.0120321	ENSG00000271851	17	9553322	9555696	-
ENST00000607727	38.8519959	4.893608756	0.81772	5.9844	2.17E-09	7.32E-08	ENSG00000271755	6	27404009	27406964	-
ENST00000607876	11.27844141	3.010275552	1.16428	2.5855	0.0097228	0.0428649	ENSG00000272848	6	169426419	169452475	-
ENST00000607927	46.02234447	2.406222716	0.53564	4.4923	7.05E-06	9.93E-05	ENSG00000225335	22	18077185	18078884	-
ENST00000608005	16.46949583	2.438223365	0.96103	2.5371	0.0111779	0.0477883	ENSG00000272630	10	73098043	73101297	-
ENST00000608049	89.98121752	-1.175549881	0.38451	-3.057	0.0022339	0.0131701	ENSG00000290877	18	109064	122219	ROCK1P1
ENST00000608684	10.0959356	-6.810482975	1.73745	-3.92	8.86E-05	0.000889	ENSG00000273338	1	78004345	78004554	-
ENST00000608770	53.38741872	-1.976589298	0.49317	-4.008	6.12E-05	0.0006481	ENSG00000272732	7	7550103	7552440	-
ENST00000608984	4.593747339	5.6286836	2.037	2.7632	0.0057235	0.0281071	ENSG00000272911	17	2688472	2688960	-
ENST00000609104	779.1861893	-1.200980608	0.21567	-5.569	2.57E-08	6.80E-07	ENSG00000148429	10	11460509	11611650	USP6NL
ENST00000609162	24.48617499	1.923091502	0.76168	2.5248	0.0115769	0.0491166	ENSG00000272720	22	38090126	38091559	-
ENST00000609363	62.17722652	1.628719718	0.44797	3.6358	0.0002771	0.0023335	ENSG00000273413	10	86970236	86970826	-
ENST00000609375	113.0230192	-1.487405241	0.3547	-4.193	2.75E-05	0.0003228	ENSG00000285508	20	10413519	10431922	MKKS
ENST00000609514	4.872759857	5.712624018	2.00261	2.8526	0.0043365	0.0225272	ENSG00000273143	10	110428839	110496187	DUSP5-DT
ENST00000609632	30.55908795	2.342111421	0.64423	3.6355	0.0002774	0.0023354	ENSG00000273139	22	20320103	20321208	-

lncRNA	Yes	-	108
ocessed_pseudoge	Yes	-	108
protein_coding	Yes	NM_145715.3	108
misc_RNA	Yes	-	108
ocessed_pseudoge	Yes	-	108
protein_coding	Yes	NM_003813.4	108
lncRNA	Yes	-	108
protein_coding	Yes	NM_001355256.2	108
protein_coding	Yes	NM_001378122.1	108
protein_coding	Yes	NM_005971.4	108
lncRNA	Yes	-	108
protein_coding	Yes	NM_001367799.1	108
lncRNA	Yes	-	108
lncRNA	Yes	-	108
ocessed_pseudoge	Yes	-	108
protein_coding	Yes	-	108
protein_coding	Yes	NM_032333.5	108
lncRNA	Yes	-	108
protein_coding	Yes	-	108
lncRNA	Yes	-	108
lncRNA	Yes	-	108
lncRNA	Yes	-	108
protein_coding	Yes	NM_001040874.1	108
protein_coding	Yes	NM_001163424.2	108
protein_coding	Yes	NM_001320586.2	108
lncRNA	Yes	-	108
lncRNA	Yes	-	108
lncRNA	Yes	-	108
lncRNA	Yes	-	108
lncRNA	Yes	-	108
lncRNA	Yes	-	108
lncRNA	Yes	-	108
lncRNA	Yes	-	108
lncRNA	Yes	-	108
protein_coding	Yes	NM_014688.5	108
lncRNA	Yes	-	108
lncRNA	Yes	-	108
protein_coding	Yes	-	108
lncRNA	Yes	-	108
lncRNA	Yes	-	108

ENST00000609682	28.53498638	-2.033662265	0.64449	-3.155	0.0016024	0.0100394	ENSG00000256628	3	101676423	101679217	ZBTB11-AS1
ENST00000609684	9.51039549	-5.232221858	1.79368	-2.917	0.0035338	0.0190767	ENSG00000272674	5	141182386	141186226	PCDHB16
ENST00000609713	25.4552377	2.504906602	0.72342	3.4626	0.000535	0.0040622	ENSG00000157542	21	37607372	37916457	KCNJ6
ENST00000609744	43.97735085	-1.505798903	0.51452	-2.927	0.0034271	0.0186316	ENSG00000273420	19	33207128	33207639	-
ENST00000609924	12.15070602	5.554890085	1.67811	3.3102	0.0009322	0.0064265	ENSG00000272688	18	3245287	3247433	-
ENST00000609936	20.02624219	7.751353633	1.60097	4.8417	1.29E-06	2.23E-05	ENSG00000272682	22	19124308	19128449	-
ENST00000610050	19.44024965	7.707436411	1.60874	4.791	1.66E-06	2.80E-05	ENSG00000272836	22	50205584	50206062	-
ENST00000610495	39.20565241	-2.243768416	0.56337	-3.983	6.81E-05	0.0007106	ENSG00000288722	X	154886354	154888061	F8A1
ENST00000610533	743.4299454	-1.387804386	0.20388	-6.807	9.97E-12	5.49E-10	ENSG00000106628	7	44114679	44123548	POLD2
ENST00000610578	71.82342493	1.502957343	0.41833	3.5928	0.0003272	0.0026842	ENSG00000291158	1	149607343	149679523	LINC00869
ENST00000610674	34640.20293	1.538613323	0.21091	7.2953	2.98E-13	2.23E-11	ENSG00000278771	14	49853615	49853914	RN7SL3
ENST00000610745	45.09852375	-5.17590101	0.8048	-6.431	1.27E-10	5.44E-09	ENSG00000138061	2	38067508	38076151	CYP1B1
ENST00000610828	10.01530962	-3.227184932	1.19246	-2.706	0.006803	0.0323133	ENSG00000136098	13	52132646	52159597	NEK3
ENST00000611299	71.08204906	1.225899327	0.42151	2.9083	0.0036338	0.0195121	ENSG00000164674	6	158650053	158764871	SYTL3
ENST00000611582	210.7381733	-1.26312137	0.27331	-4.622	3.81E-06	5.78E-05	ENSG00000135931	2	231198630	231376848	ARMC9
ENST00000611635	7.359262396	3.76151506	1.48496	2.5331	0.0113068	0.0482051	ENSG00000277561	15	22607539	22617756	GOLGA8IP
ENST00000611639	57.226195	-3.199174799	0.5177	-6.18	6.43E-10	2.39E-08	ENSG00000189366	3	125929271	125990537	ALG1L
ENST00000611884	467.4214121	-1.845094425	0.22288	-8.278	1.25E-16	1.47E-14	ENSG00000174891	3	158110088	158545730	RSRC1
ENST00000611956	7.064360679	6.248469536	1.84774	3.3817	0.0007204	0.0052065	ENSG00000273980	10	133257143	133257551	-
ENST00000611969	50.04960931	-1.456142353	0.50057	-2.909	0.0036264	0.0194779	ENSG00000175701	2	110211528	110212547	MTLN
ENST00000612078	4.36037937	5.553185115	2.06176	2.6934	0.0070723	0.0332879	ENSG00000186526	19	15615217	15630639	CYP4F8
ENST00000612480	202.4184967	1.714251807	0.28238	6.0707	1.27E-09	4.50E-08	ENSG00000275131	1	120489859	120701289	PDE4DIPP2
ENST00000612725	5.106127827	5.780412549	1.98129	2.9175	0.0035284	0.0190517	ENSG00000278740	17	68188546	68189165	-
ENST00000612813	36.84797673	-4.355391136	0.7721	-5.641	1.69E-08	4.68E-07	ENSG00000165949	14	94110814	94116695	IFI27
ENST00000612820	5.151772375	5.791933645	1.97706	2.9296	0.0033943	0.0184954	ENSG00000276626	3	124792318	124792562	7SK
ENST00000612829	620.7080244	-2.64241967	0.23323	-11.33	9.38E-30	5.58E-27	ENSG00000284378	2	231713313	231713398	MIR1244-1
ENST00000612899	249.0412568	1.972083397	0.2839	6.9464	3.75E-12	2.25E-10	ENSG00000118503	6	137867258	137883312	TNFAIP3
ENST00000612985	9.486876695	-3.611424218	1.32206	-2.732	0.0063013	0.0303855	ENSG00000277610	1	120942599	120942763	RNVU1-4
ENST00000613153	4.639391888	5.641491239	2.02631	2.7841	0.0053674	0.0267625	ENSG00000274918	18	35317395	35317848	-
ENST00000613161	8.208378252	6.466302924	1.8049	3.5826	0.0003401	0.0027734	ENSG00000273674	15	50839875	50908599	-
ENST00000613174	896.1141675	-1.125494872	0.19793	-5.686	1.30E-08	3.70E-07	ENSG00000276903	6	27865316	27865798	H2AC16
ENST00000613361	38.79263647	1.647472499	0.55791	2.9529	0.0031478	0.0173661	ENSG00000277938	20	25229149	25231933	-
ENST00000613769	25.25879783	-2.064060559	0.68618	-3.008	0.0026295	0.0150255	ENSG00000290856	13	52600118	52637368	-
ENST00000613868	16.38886985	3.278606645	0.98606	3.325	0.0008843	0.0061554	ENSG00000275491	20	3808356	3812434	LINC01730
ENST00000613986	80.67541883	-1.251848346	0.44727	-2.799	0.0051286	0.0258085	ENSG00000196684	19	16143684	16158575	HSH2D
ENST00000614097	46.52188597	3.845207312	0.62188	6.1832	6.28E-10	2.34E-08	ENSG00000274290	6	26183760	26184230	H2BC6
ENST00000614247	2843.522057	-1.413835447	0.17524	-8.068	7.15E-16	7.66E-14	ENSG00000277157	6	26188709	26189112	H4C4
ENST00000614302	35.63451859	-1.849442363	0.5955	-3.106	0.0018984	0.0115155	ENSG00000176715	16	89093851	89156233	ACSF3
ENST00000614341	8.825810741	-6.616648809	1.77564	-3.726	0.0001943	0.0017294	ENSG00000182040	17	74916082	74923255	USH1G
ENST00000614378	1985.787791	-1.195497582	0.17646	-6.775	1.24E-11	6.70E-10	ENSG00000273983	6	26270917	26271413	H3C8

lncRNA	Yes	-	108
protein_coding	Yes	NM_020957.4	108
protein_coding	Yes	NM_002240.5	108
lncRNA	Yes	-	108
lncRNA	Yes	-	108
lncRNA	Yes	-	108
lncRNA	Yes	-	108
protein_coding	Yes	NM_012151.4	108
protein_coding	Yes	NM_006230.4	108
lncRNA	Yes	-	108
misc_RNA	Yes	-	108
protein_coding	Yes	NM_000104.4	108
protein_coding	Yes	NM_002498.3	108
protein_coding	Yes	NM_001242394.2	108
protein_coding	Yes	NM_001352754.2	108
ed_unprocessed_ps	Yes	-	108
ed_unprocessed_ps	Yes	-	108
protein_coding	Yes	NM_001271838.2	108
lncRNA	Yes	-	108
protein_coding	Yes	NM_001384134.1	108
protein_coding	Yes	NM_007253.4	108
ed_unprocessed_ps	Yes	-	108
lncRNA	Yes	-	108
protein_coding	Yes	-	108
misc_RNA	Yes	-	108
miRNA	Yes	-	108
protein_coding	Yes	NM_001270508.2	108
snRNA	Yes	-	108
lncRNA	Yes	-	108
lncRNA	Yes	-	108
protein_coding	Yes	NM_003511.3	108
lncRNA	Yes	-	108
lncRNA	Yes	-	108
lncRNA	Yes	-	108
protein_coding	Yes	NM_001382417.1	108
protein_coding	Yes	NM_003523.3	108
protein_coding	Yes	NM_003539.4	108
protein_coding	Yes	NM_001243279.3	108
protein_coding	Yes	NM_173477.5	108
protein_coding	Yes	NM_003534.3	108

ENST00000614492	49.69349982	2.364896786	0.67576	3.4996	0.0004659	0.0036176	ENSG00000277437	21	8208843	8208904	-
ENST00000614646	1594.232475	-1.384728687	0.18251	-7.587	3.27E-14	2.80E-12	ENSG00000140022	14	81260651	81400324	STON2
ENST00000614758	412.6937541	-3.255106191	0.78819	-4.13	3.63E-05	0.000412	ENSG00000284570	8	100702967	100703024	MIR7705
ENST00000614771	31.16012508	2.117005594	0.63593	3.329	0.0008716	0.0060848	ENSG00000278709	20	57710155	57712780	NKILA
ENST00000615164	1713.303814	-2.634193444	0.18229	-14.45	2.49E-47	7.47E-44	ENSG00000276966	6	26204609	26205021	H4C5
ENST00000615189	56.06443412	-1.410372467	0.47124	-2.993	0.0027635	0.0156376	ENSG00000100003	22	30397017	30425303	SEC14L2
ENST00000615353	265.4423505	-7.492515685	0.64033	-11.7	1.26E-31	8.67E-29	ENSG00000276180	6	27139281	27139678	H4C9
ENST00000615531	56.75674986	1.643543021	0.52253	3.1454	0.0016588	0.0103355	ENSG00000088340	20	35558865	35607494	FER1L4
ENST00000615648	4075.826648	1.265190102	0.16932	7.4723	7.88E-14	6.37E-12	ENSG00000261150	8	143857323	143878467	EPPK1
ENST00000615665	58.88333314	-3.309113153	0.55499	-5.962	2.48E-09	8.27E-08	ENSG00000145687	5	81412803	81751097	SSBP2
ENST00000615674	18.27855503	7.619916423	1.61488	4.7186	2.38E-06	3.84E-05	ENSG00000273712	6	28315612	28315883	-
ENST00000615868	829.9676889	-1.21400304	0.20461	-5.933	2.97E-09	9.73E-08	ENSG00000278463	6	26033091	26033618	H2AC4
ENST00000615959	30.513753	4.857025217	1.0975	4.4255	9.62E-06	0.00013	ENSG00000275708	21	8208472	8208652	MIR3648-1
ENST00000616016	3.79736192	-5.399314655	2.13639	-2.527	0.011494	0.0488313	ENSG00000187634	1	923922	944574	SAMD11
ENST00000616106	33.36619992	2.241500228	0.6437	3.4822	0.0004973	0.0038201	ENSG00000278341	16	88708955	88710437	-
ENST00000616113	4.616569613	5.635108028	2.03024	2.7756	0.0055102	0.0273019	ENSG00000274363	17	4267690	4268430	-
ENST00000616182	1210.54202	-1.327019918	0.18715	-7.091	1.33E-12	8.88E-11	ENSG00000274641	6	27893424	27893891	H2BC17
ENST00000616428	60.30842449	2.309868891	0.47188	4.8951	9.83E-07	1.76E-05	ENSG00000274828	18	79677284	79679364	-
ENST00000616558	517.2905066	-1.283451063	0.23827	-5.386	7.18E-08	1.72E-06	ENSG00000142765	1	27342021	27353932	SYTL1
ENST00000616572	6.153711075	6.047833923	1.90298	3.1781	0.0014825	0.0094353	ENSG00000273998	20	16576067	16579615	-
ENST00000616721	69.55503296	4.022933761	0.5348	7.5223	5.38E-14	4.49E-12	ENSG00000275395	19	39863322	39934626	FCGBP
ENST00000616818	4.451668466	5.580042745	2.05553	2.7147	0.0066346	0.0316755	ENSG00000289133	13	21287633	21287828	-
ENST00000616842	14.38888574	4.152629944	1.1864	3.5002	0.0004649	0.0036119	ENSG00000274717	22	42791813	42794313	-
ENST00000617058	26.00794538	2.15145203	0.68547	3.1386	0.0016974	0.0105282	ENSG00000273599	10	124996063	125001491	-
ENST00000617224	6.17653335	6.052649114	1.90546	3.1765	0.0014907	0.0094747	ENSG00000276430	10	47995321	47999791	FAM25C
ENST00000617275	1600.960383	-1.124849721	0.18032	-6.238	4.43E-10	1.69E-08	ENSG00000169499	8	38901345	38973912	PLEKHA2
ENST00000617533	130.0241569	1.453520731	0.35222	4.1268	3.68E-05	0.0004166	ENSG00000174938	16	29871158	29899550	SEZ6L2
ENST00000617673	4.149833674	5.480651158	2.08437	2.6294	0.0085535	0.0387013	ENSG00000284280	8	27610600	27610751	MIR6843
ENST00000617804	94.12117808	1.5408182	0.41553	3.7081	0.0002088	0.0018407	ENSG00000274322	20	1318372	1393096	-
ENST00000617896	4.662214162	5.647844874	2.02495	2.7891	0.0052851	0.026438	ENSG00000275329	9	136122520	136124363	-
ENST00000618183	2509.891711	-1.956335398	0.18344	-10.66	1.49E-26	6.04E-24	ENSG00000122566	7	26189926	26200746	HNRNPA2B1
ENST00000618282	1844.873278	-1.002772065	0.18276	-5.487	4.09E-08	1.03E-06	ENSG00000102158	X	77825746	77895435	MAGT1
ENST00000618302	7.645207989	6.360665917	1.82528	3.4848	0.0004926	0.003789	ENSG00000284800	X	102360397	102463164	-
ENST00000618305	502.6851896	-2.623616544	0.33903	-7.739	1.01E-14	9.20E-13	ENSG00000275126	6	27873147	27873534	H4C13
ENST00000618526	10.07017678	6.758717162	1.73927	3.886	0.0001019	0.0009977	ENSG00000275756	9	92104424	92105451	-
ENST00000618589	86.20591124	-2.511000948	0.41712	-6.02	1.75E-09	6.02E-08	ENSG00000276216	1	145281115	145281462	-
ENST00000619162	157.4271888	-2.404131154	0.35328	-6.805	1.01E-11	5.55E-10	ENSG00000264230	10	46375775	46391778	ANXA8L1
ENST00000619168	199.4050557	-1.996445295	0.31482	-6.342	2.28E-10	9.23E-09	ENSG00000183049	10	12349546	12835545	CAMK1D
ENST00000619208	66.26791481	2.093272038	0.45461	4.6045	4.13E-06	6.22E-05	ENSG00000186862	10	101007678	101031129	PDZD7
ENST00000619360	340.6679107	2.60249745	0.31077	8.3743	5.56E-17	6.83E-15	ENSG00000146232	6	44258165	44265551	NFKBIE

miRNA	Yes	-	108
protein_coding	Yes	NM_001394390.1	108
miRNA	Yes	-	108
lncRNA	Yes	-	108
protein_coding	Yes	NM_003545.4	108
protein_coding	Yes	NM_012429.5	108
protein_coding	Yes	NM_003495.3	108
ribed_unitary_pseud	Yes	-	108
protein_coding	Yes	NM_031308.4	108
protein_coding	Yes	NM_001256732.3	108
ocessed_pseudoge	Yes	-	108
protein_coding	Yes	NM_003513.3	108
miRNA	Yes	-	108
protein_coding	Yes	NM_001385641.1	108
lncRNA	Yes	-	108
lncRNA	Yes	-	108
protein_coding	Yes	NM_003527.4	108
lncRNA	Yes	-	108
protein_coding	Yes	NM_001193308.2	108
lncRNA	Yes	-	108
protein_coding	Yes	-	108
ed_unprocessed_ps	Yes	-	108
ocessed_pseudoge	Yes	-	108
lncRNA	Yes	-	108
protein_coding	Yes	NM_001137548.3	108
protein_coding	Yes	NM_021623.2	108
protein_coding	Yes	NM_001243332.2	108
miRNA	Yes	-	108
protein_coding	Yes	-	108
lncRNA	Yes	-	108
protein_coding	Yes	NM_002137.4	108
protein_coding	Yes	NM_001367916.1	108
protein_coding	Yes	-	108
protein_coding	Yes	NM_003546.3	108
ocessed_pseudoge	Yes	-	108
lncRNA	Yes	-	108
protein_coding	Yes	NM_001098845.3	108
protein_coding	Yes	NM_153498.4	108
protein_coding	Yes	NM_001195263.2	108
protein_coding	Yes	NM_004556.3	108

ENST00000619642	23.81634455	1.994439306	0.70688	2.8215	0.0047806	0.0243674	ENSG00000273945	9	35732921	35732995	MIR6853
ENST00000619654	8.806902609	6.56246325	1.83193	3.5823	0.0003406	0.0027765	ENSG00000274383	15	64950915	64951435	-
ENST00000619766	4.406023918	5.566668149	2.0528	2.7117	0.006693	0.0318868	ENSG00000274269	20	1186091	1207036	-
ENST00000619867	15.84361338	5.950632972	1.62607	3.6595	0.0002527	0.0021653	ENSG00000278811	1	147258884	147517875	LINC00624
ENST00000620149	20.03588262	-6.344593661	1.59499	-3.978	6.95E-05	0.0007233	ENSG00000196542	3	161344797	161371517	SPTSSB
ENST00000620220	9.304645838	5.158344356	1.73967	2.9651	0.0030256	0.0168074	ENSG00000282301	7	99684956	99735102	CYP3A7-CYP3A51P
ENST00000620266	820.4365447	1.072747967	0.2297	4.6701	3.01E-06	4.72E-05	ENSG00000278730	17	68126665	68129586	-
ENST00000620414	75.44875688	-1.903357029	0.4198	-4.534	5.79E-06	8.36E-05	ENSG00000277534	18	26542970	26545791	-
ENST00000620680	4.94122668	5.730713439	2.00267	2.8615	0.0042159	0.0220395	ENSG00000277264	6	32179815	32179876	MIR6833
ENST00000620793	10.84472384	-4.40500879	1.36204	-3.234	0.0012202	0.0080371	ENSG00000274286	2	96112875	96116571	ADRA2B
ENST00000621053	12.99594349	-3.636506787	1.1185	-3.251	0.0011491	0.0076481	ENSG00000276302	6	28267120	28281580	-
ENST00000621103	42.5932927	1.572667767	0.588	2.6746	0.0074817	0.0347527	ENSG00000233006	5	132311284	132369916	MIR3936HG
ENST00000621112	758.2105174	-1.577178578	0.20138	-7.832	4.81E-15	4.62E-13	ENSG00000273703	6	27815021	27815489	H2BC14
ENST00000621166	6.364256771	6.097185577	1.88659	3.2319	0.0012299	0.0080872	ENSG00000283836	1	43364647	43364715	MIR6734
ENST00000621281	130.2083676	-1.493647347	0.32843	-4.548	5.42E-06	7.89E-05	ENSG00000274944	1	38864528	38881602	-
ENST00000621282	82.26729082	-1.723844708	0.39741	-4.338	1.44E-05	0.0001846	ENSG00000231607	13	49982551	50125541	DLEU2
ENST00000621410	201.9440649	-1.313011122	0.28178	-4.66	3.17E-06	4.92E-05	ENSG00000274349	9	66900724	66921426	ZNF658
ENST00000621441	1778.902972	-1.165787804	0.18191	-6.409	1.47E-10	6.20E-09	ENSG00000070367	14	57200506	57268905	EXOC5
ENST00000621525	393.215576	-1.391013396	0.24136	-5.763	8.25E-09	2.47E-07	ENSG00000258644	14	70326063	70417074	SYNJ2BP-COX16
ENST00000621583	15.63789532	7.392324853	1.65662	4.4623	8.11E-06	0.0001122	ENSG00000274678	16	30821337	30821884	-
ENST00000621597	25.86517625	1.868755682	0.6675	2.7996	0.0051162	0.0257609	ENSG00000275223	20	33655700	33656423	-
ENST00000621997	5.156917603	5.798707483	2.12677	2.7265	0.0064004	0.0307729	ENSG00000277368	13	26221926	26222654	-
ENST00000621999	1171.630282	-1.878813636	0.25205	-7.454	9.05E-14	7.26E-12	ENSG00000085365	5	78360616	78480739	SCAMP1
ENST00000622235	71.61632636	1.361725403	0.43549	3.1269	0.0017666	0.0108749	ENSG00000244486	22	20424583	20437825	SCARF2
ENST00000622285	33.0968625	-2.369809499	0.61486	-3.854	0.0001161	0.0011134	ENSG00000274210	1	148522600	148522765	RNVU1-27
ENST00000622447	178.2363039	1.498043375	0.30005	4.9927	5.95E-07	1.13E-05	ENSG00000268350	X	52947253	52958518	FAM156A
ENST00000622750	60.42592746	1.302653467	0.45019	2.8935	0.0038091	0.0202764	ENSG00000277476	17	68133200	68135935	-
ENST00000622931	61.52909972	1.471091126	0.52728	2.7899	0.0052718	0.0263904	ENSG00000280248	17	80339897	80342058	-
ENST00000623057	5.476429441	5.878068943	1.96884	2.9856	0.0028307	0.0159316	ENSG00000279255	16	707649	709816	-
ENST00000623146	88.09136438	-1.062442885	0.37567	-2.828	0.0046819	0.0239687	ENSG00000279730	13	18297553	18298733	KMT5AP1
ENST00000623192	23.4085923	2.147912687	0.71764	2.993	0.0027621	0.0156315	ENSG00000278952	11	65118309	65119111	-
ENST00000623250	62.55176465	-1.138223032	0.43551	-2.614	0.0089605	0.0402003	ENSG00000279348	2	216211403	216213519	-
ENST00000623257	32.82912846	2.550121169	0.62945	4.0513	5.09E-05	0.0005525	ENSG00000279605	8	143578357	143583304	-
ENST00000623334	13.59749273	4.066473662	1.19547	3.4016	0.00067	0.004901	ENSG00000277661	6	29036020	29076479	OR2W1-AS1
ENST00000623400	535.5553038	1.127360326	0.22506	5.0091	5.47E-07	1.05E-05	ENSG00000172661	10	45727265	45792964	WASHC2C
ENST00000623435	9.49434305	-4.173743692	1.48365	-2.813	0.0049059	0.024896	ENSG00000279513	1	117493514	117495006	-
ENST00000623463	20.51277135	-2.068241135	0.78732	-2.627	0.0086153	0.0389335	ENSG00000279458	10	46370758	46382626	-
ENST00000623483	70.60003179	1.164192844	0.42083	2.7664	0.0056675	0.0278995	ENSG00000279145	15	55171377	55176739	-
ENST00000623535	164.8735964	-1.013541177	0.30893	-3.281	0.0010352	0.0070104	ENSG00000008086	X	18425607	18640196	CDKL5
ENST00000623543	23.25055226	3.820387538	0.96014	3.979	6.92E-05	0.0007205	ENSG00000280007	22	18110758	18131154	-

miRNA	Yes	-	108
lncRNA	Yes	-	108
lncRNA	Yes	-	108
lncRNA	Yes	-	108
protein_coding	Yes	NM_001040100.2	108
protein_coding	Yes	-	108
lncRNA	Yes	-	108
lncRNA	Yes	-	108
miRNA	Yes	-	108
protein_coding	Yes	NM_000682.7	108
protein_coding	Yes	-	108
lncRNA	Yes	-	108
protein_coding	Yes	NM_003521.3	108
miRNA	Yes	-	108
protein_coding	Yes	-	108
lncRNA	Yes	-	108
protein_coding	Yes	NM_033160.7	108
protein_coding	Yes	NM_006544.4	108
protein_coding	Yes	-	108
lncRNA	Yes	-	108
lncRNA	Yes	-	108
lncRNA	Yes	-	108
protein_coding	Yes	NM_004866.6	108
protein_coding	Yes	NM_182895.5	108
snRNA	Yes	-	108
protein_coding	Yes	NM_001387706.1	108
lncRNA	Yes	-	108
TEC	Yes	-	108
TEC	Yes	-	108
ocessed_pseudoge	Yes	-	108
TEC	Yes	-	108
TEC	Yes	-	108
TEC	Yes	-	108
lncRNA	Yes	-	108
protein_coding	Yes	NM_001330074.2	108
TEC	Yes	-	108
lncRNA	Yes	-	108
lncRNA	Yes	-	108
protein_coding	Yes	NM_001323289.2	108
lncRNA	Yes	-	108

ENST00000623633	34.117176	2.895097076	0.63638	4.5494	5.38E-06	7.84E-05	ENSG00000280238	10	73791217	73791490	-
ENST00000623789	11.02647093	6.889945269	1.71492	4.0176	5.88E-05	0.0006251	ENSG00000278920	22	31051629	31068327	-
ENST00000623877	5.780113615	-6.006529656	1.93079	-3.111	0.001865	0.0113555	ENSG00000280027	16	53391088	53392473	-
ENST00000623936	1807.91637	-1.405153635	0.18019	-7.798	6.28E-15	5.92E-13	ENSG00000279483	7	137513858	137513933	-
ENST00000623995	4.315821984	-5.583530402	2.06921	-2.698	0.0069678	0.0329308	ENSG00000279472	5	141177848	141182369	-
ENST00000624004	4.895582131	5.718672064	2.00026	2.859	0.0042502	0.0221757	ENSG00000279311	3	158869897	158871821	-
ENST00000624087	45.82873035	-1.678525	0.54612	-3.074	0.0021154	0.0125899	ENSG00000280287	12	132550728	132554947	-
ENST00000624153	42.75247959	2.461370476	0.55145	4.4634	8.07E-06	0.0001117	ENSG00000280330	21	7129102	7130287	CTBP2P10
ENST00000624171	96.54532785	1.859967464	0.4115	4.52	6.18E-06	8.84E-05	ENSG00000280046	17	7858942	7866083	-
ENST00000624246	12.62303181	2.899701559	1.11014	2.612	0.0090013	0.0403409	ENSG00000279539	19	42242774	42244973	-
ENST00000624248	4.383201644	5.55994647	2.05576	2.7046	0.0068394	0.0324384	ENSG00000278998	7	155222902	155223851	-
ENST00000624431	4.872759857	5.712624018	2.00261	2.8526	0.0043365	0.0225272	ENSG00000279928	1	182695	184174	DDX11L17
ENST00000624624	1293.87376	1.266875564	0.18453	6.8656	6.62E-12	3.80E-10	ENSG00000279117	11	75260128	75262466	-
ENST00000624682	57.01023312	-2.088902628	0.49413	-4.227	2.36E-05	0.0002833	ENSG00000280206	16	15701236	15702118	-
ENST00000624705	294.9718154	-1.668936717	0.27934	-5.975	2.31E-09	7.73E-08	ENSG00000279602	17	43360032	43361361	-
ENST00000624818	5.36231807	5.850679104	1.95813	2.9879	0.0028091	0.015836	ENSG00000280384	22	46163302	46165347	-
ENST00000624861	5.618508314	5.917680498	1.93743	3.0544	0.0022551	0.0132659	ENSG00000279853	7	44367139	44369292	-
ENST00000624956	535.7006534	-1.17545045	0.22475	-5.23	1.70E-07	3.71E-06	ENSG00000279800	16	34266040	34268649	BCLAF1P2
ENST00000625022	8.578679867	6.527755504	1.78379	3.6595	0.0002527	0.0021653	ENSG00000280053	3	126973064	126976426	-
ENST00000625293	56.96014679	1.639540911	0.46947	3.4923	0.0004789	0.0037026	ENSG00000213689	3	48466229	48467645	TREX1
ENST00000626300	15.92205306	7.420052311	1.63797	4.53	5.90E-06	8.49E-05	ENSG00000281491	9	34985409	34989379	DNAJB5-DT
ENST00000626948	4.428846192	5.573362803	2.05277	2.715	0.0066267	0.0316529	ENSG00000242808	3	180989769	181791029	SOX2-OT
ENST00000627116	4.662214162	5.647844874	2.02495	2.7891	0.0052851	0.026438	ENSG00000228242	3	14148401	14165978	XPC-AS1
ENST00000627981	2141.551876	-7.384042996	0.27346	-27	1.40E-160	2.14E-155	ENSG00000281181	21	8437628	8438551	-
ENST00000628067	572.4018735	1.548161001	0.21202	7.3018	2.84E-13	2.13E-11	ENSG00000177542	11	790474	798281	SLC25A22
ENST00000628224	784.4241649	-1.157355299	0.20092	-5.76	8.40E-09	2.50E-07	ENSG00000204843	2	74361154	74380311	DCTN1
ENST00000629291	8.797072131	-3.469805575	1.29871	-2.672	0.0075459	0.0349973	ENSG00000232006	7	42971540	43113931	-
ENST00000629807	34.68820326	1.718262373	0.57773	2.9742	0.0029377	0.0164093	ENSG00000242086	3	195708182	195739964	MUC20-OT1
ENST00000629969	4438.603688	-8.945865397	0.81716	-10.95	6.84E-28	3.32E-25	ENSG00000281383	21	8254591	8255514	-
ENST00000630421	12.32939093	3.475396001	1.12303	3.0947	0.0019703	0.0118712	ENSG00000280734	13	99486961	99496772	LINC01232
ENST00000630721	336.6415459	-1.509740553	0.24195	-6.24	4.38E-10	1.67E-08	ENSG00000198689	X	135985434	136047269	SLC9A6
ENST00000631190	37.66766788	-1.822373704	0.56093	-3.249	0.0011587	0.0076995	ENSG00000280798	11	33076148	33079454	LINC00294
ENST00000631195	16.36596673	7.460133325	1.63268	4.5693	4.89E-06	7.22E-05	ENSG00000281097	11	129612115	129617269	LINC01395
ENST00000633077	63.71633556	-2.558838273	0.48677	-5.257	1.47E-07	3.25E-06	ENSG00000048740	10	11017871	11336675	CELF2
ENST00000634258	516.3985073	1.005304072	0.30636	3.2814	0.0010328	0.006997	ENSG00000198198	1	43389898	43454247	SZT2
ENST00000634734	743.6545889	1.054721238	0.26373	3.9993	6.35E-05	0.000669	ENSG00000107262	9	33252470	33264708	BAG1
ENST00000635120	73.29355739	-3.110187142	0.49829	-6.242	4.33E-10	1.65E-08	ENSG00000183117	8	2935360	4994914	CSMD1
ENST00000635271	6.06536895	-6.075177298	1.9064	-3.187	0.0014389	0.0092077	ENSG00000234546	1	9182003	9196284	LNCTAM34A
ENST00000635687	104.9477702	-1.166050994	0.37196	-3.135	0.001719	0.0106334	ENSG00000228526	1	9148010	9182392	MIR34AHG
ENST00000635767	340.5634114	-1.388394545	0.24182	-5.741	9.39E-09	2.76E-07	ENSG00000178177	4	17841186	18021755	LCORL

TEC	Yes	-	108
lncRNA	Yes	-	108
TEC	Yes	-	108
ocessed_pseudoge	Yes	-	108
lncRNA	Yes	-	108
TEC	Yes	-	108
TEC	Yes	-	108
ocessed_pseudoge	Yes	-	108
TEC	Yes	-	108
TEC	Yes	-	108
TEC	Yes	-	108
processed_pseudog	Yes	-	108
TEC	Yes	-	108
lncRNA	Yes	-	108
TEC	Yes	-	108
lncRNA	Yes	-	108
TEC	Yes	-	108
ocessed_pseudoge	Yes	-	108
TEC	Yes	-	108
protein_coding	Yes	NM_033629.6	108
lncRNA	Yes	-	108
lncRNA	Yes	-	108
lncRNA	Yes	-	108
lncRNA	Yes	-	108
protein_coding	Yes	NM_001191061.2	108
protein_coding	Yes	NM_004082.5	108
lncRNA	Yes	-	108
lncRNA	Yes	-	108
lncRNA	Yes	-	108
lncRNA	Yes	-	108
protein_coding	Yes	NM_001379110.1	108
lncRNA	Yes	-	108
lncRNA	Yes	-	108
protein_coding	Yes	NM_001326342.2	108
protein_coding	Yes	NM_001365999.1	108
protein_coding	Yes	NM_004323.6	108
protein_coding	Yes	NM_033225.6	108
lncRNA	Yes	-	108
lncRNA	Yes	-	108
protein_coding	Yes	NM_001394446.1	108

ENST00000635826	16.61215129	-4.012410766	1.03881	-3.863	0.0001122	0.0010811	ENSG00000283676	1	148334478	148334554	MIR5087
ENST00000635852	58.83312944	9.305114976	1.50845	6.1686	6.89E-10	2.55E-08	ENSG00000283646	3	46416523	46423591	LINC02009
ENST00000636147	601.4690036	1.514266681	0.2355	6.43	1.28E-10	5.48E-09	ENSG00000188603	16	28477278	28492074	CLN3
ENST00000636174	170.3090905	-1.225361383	0.29375	-4.171	3.03E-05	0.0003512	ENSG00000188878	17	75909573	75941042	FBF1
ENST00000636215	356.0269717	-1.662830578	0.23638	-7.035	2.00E-12	1.28E-10	ENSG00000175105	3	88059254	88144660	ZNF654
ENST00000636341	46.91203038	-1.27653512	0.50135	-2.546	0.0108904	0.0468268	ENSG00000225868	19	37829049	37836964	WDR87BP
ENST00000636449	59.10571721	-1.857150821	0.4951	-3.751	0.0001761	0.0015913	ENSG00000283498	5	118974585	118974670	MIR1244-2
ENST00000637378	122.4937521	1.582033721	0.34715	4.5573	5.18E-06	7.59E-05	ENSG00000261832	16	28456718	28482634	-
ENST00000637412	37.29100346	1.473773598	0.55535	2.6538	0.0079598	0.0365463	ENSG00000283426	3	195701617	195711643	SMBD1P
ENST00000637601	113.9588854	1.498221727	0.34377	4.3582	1.31E-05	0.0001703	ENSG00000290999	1	120528386	120548357	-
ENST00000638164	25.42905958	8.094479415	1.57766	5.1307	2.89E-07	5.99E-06	ENSG00000214725	16	29864307	29865442	CDIPTOSP
ENST00000638394	88.44572999	1.968453093	0.46075	4.2723	1.93E-05	0.0002382	ENSG00000163637	3	64092235	64225466	PRICKLE2
ENST00000638749	350.4449953	-1.274797602	0.25848	-4.932	8.14E-07	1.49E-05	ENSG00000283154	3	159273243	159897359	IQCJ-SCHIP1
ENST00000638797	56.03867083	1.438023199	0.46626	3.0841	0.0020414	0.0122257	ENSG00000260220	9	136249972	136304094	CCDC187
ENST00000639259	5.197416923	5.803394709	1.98153	2.9287	0.0034033	0.0185294	ENSG00000279317	2	210171550	210233966	-
ENST00000639579	12.01758327	3.085525587	1.07008	2.8835	0.0039334	0.0208192	ENSG00000181652	7	151015150	151024494	ATG9B
ENST00000639662	5.858189525	-6.023886158	1.93385	-3.115	0.0018396	0.0112291	ENSG00000228623	9	112997119	112998259	ZNF883
ENST00000640019	450.4588891	-1.101459277	0.23369	-4.713	2.44E-06	3.93E-05	ENSG00000284024	10	14838305	14846999	-
ENST00000640237	42.39498855	1.701900058	0.60194	2.8274	0.0046932	0.0240101	ENSG00000214279	10	133453944	133469760	SCART1
ENST00000640769	697.3718919	1.18956944	0.20211	5.8856	3.97E-09	1.27E-07	ENSG00000176225	18	70003030	70205687	RTTN
ENST00000640959	108.8720683	1.06942169	0.3834	2.7893	0.0052822	0.0264347	ENSG00000283977	11	6481500	6498239	-
ENST00000641151	15.66873937	4.916528989	1.28552	3.8245	0.000131	0.0012354	ENSG00000204701	6	29108057	29114770	OR2J3
ENST00000641199	37.82137858	1.454238641	0.55384	2.6257	0.0086463	0.0390582	ENSG00000284626	15	90234199	90265462	-
ENST00000641304	159.473127	-1.354118574	0.31916	-4.243	2.21E-05	0.0002671	ENSG00000284634	12	9448497	9658392	-
ENST00000641417	5.100982599	5.773555224	2.1388	2.6994	0.0069456	0.0328423	ENSG00000204700	6	29170906	29175811	OR2J2
ENST00000641539	437.5656484	-1.509523052	0.23274	-6.486	8.82E-11	3.92E-09	ENSG00000167747	19	50798193	50798910	C19orf48
ENST00000641659	4.639391888	5.641491239	2.02631	2.7841	0.0053674	0.0267625	ENSG00000204702	6	29099489	29102701	OR2J1
ENST00000641784	2139.989688	-1.29016265	0.17625	-7.32	2.48E-13	1.87E-11	ENSG00000284707	7	97851687	97972985	-
ENST00000641841	45.56996286	2.044367671	0.54454	3.7543	0.0001738	0.0015743	ENSG00000243896	7	144257662	144264792	OR2A7
ENST00000641969	4.593747339	5.6286836	2.037	2.7632	0.0057235	0.0281071	ENSG00000225969	7	73735406	73736073	ABHD11-AS1
ENST00000642384	132.7208092	-1.213322899	0.35316	-3.436	0.0005913	0.0044168	ENSG00000119866	2	60457193	60553654	BCL11A
ENST00000642603	7.10245232	-4.818172334	1.82626	-2.638	0.0083331	0.0379528	ENSG00000187554	1	223109403	223143248	TLR5
ENST00000643273	1252.848247	-1.128657473	0.1902	-5.934	2.95E-09	9.68E-08	ENSG00000148019	9	78236074	78279690	CEP78
ENST00000643349	12.76327204	-5.670130919	1.68097	-3.373	0.0007432	0.005347	ENSG00000284779	11	2131394	2149603	-
ENST00000643943	98.00128503	1.013088279	0.37474	2.7035	0.0068623	0.0325207	ENSG00000215790	1	1734689	1739557	SLC35E2A
ENST00000644144	177.1825904	-1.078501504	0.29534	-3.652	0.0002604	0.002222	ENSG00000106992	9	127866485	127877675	AK1
ENST00000644269	25.16685129	1.747943665	0.69061	2.531	0.0113736	0.0484383	ENSG00000091137	7	107660827	107717809	SLC26A4
ENST00000644384	315.2645447	2.799192945	0.73242	3.8219	0.0001325	0.0012463	ENSG00000160183	21	42371889	42396052	TMPRSS3
ENST00000644480	4228.089736	-1.314202821	0.16867	-7.791	6.62E-15	6.23E-13	ENSG00000285238	12	6556885	6607367	-
ENST00000644823	28.68959413	2.6018307	0.67987	3.8269	0.0001297	0.001225	ENSG00000119715	14	76376136	76501837	ESRRB

miRNA	Yes	-	108
lncRNA	Yes	-	108
protein_coding	Yes	NM_001042432.2	108
protein_coding	Yes	NM_001319193.2	108
protein_coding	Yes	NM_001350134.2	108
ed_unprocessed_ps	Yes	-	108
miRNA	Yes	-	108
protein_coding	Yes	-	108
ribed_unitary_pseud	Yes	-	108
lncRNA	Yes	-	108
ribed_unitary_pseud	Yes	-	108
protein_coding	Yes	NM_198859.4	108
protein_coding	Yes	NM_014575.4	108
protein_coding	Yes	NM_001378188.1	108
lncRNA	Yes	-	108
protein_coding	Yes	NM_001317056.2	108
ed_unprocessed_ps	Yes	-	108
protein_coding	Yes	NM_001378785.1	108
protein_coding	Yes	NM_001396050.1	108
protein_coding	Yes	NM_173630.4	108
protein_coding	Yes	-	108
protein_coding	Yes	NM_001005216.4	108
protein_coding	Yes	-	108
lncRNA	Yes	-	108
protein_coding	Yes	NM_030905.3	108
ribed_unitary_pseud	Yes	-	108
protein_coding	Yes	NM_001348294.2	108
lncRNA	Yes	-	108
protein_coding	Yes	NM_001005328.2	108
ribed_unitary_pseud	Yes	-	108
protein_coding	Yes	NM_022893.4	108
protein_coding	Yes	NM_003268.6	108
protein_coding	Yes	NM_001330691.3	108
protein_coding	Yes	-	108
ed_unprocessed_ps	Yes	-	108
protein_coding	Yes	NM_000476.3	108
protein_coding	Yes	NM_000441.2	108
protein_coding	Yes	NM_001256317.3	108
protein_coding	Yes	-	108
protein_coding	Yes	NM_001379180.1	108

ENST00000644917	35.71690138	-2.266880682	0.63067	-3.594	0.0003251	0.0026693	ENSG00000070182	14	64746282	64879907	SPTB
ENST00000645041	182.403102	1.072473288	0.29704	3.6106	0.0003055	0.0025324	ENSG00000285509	11	121045738	121113108	TBCEL-TECTA
ENST00000645495	4006.643256	-1.065237269	0.16863	-6.317	2.67E-10	1.07E-08	ENSG00000137710	11	110229440	110296614	RDX
ENST00000645590	4.149833674	5.480651158	2.08437	2.6294	0.0085535	0.0387013	ENSG00000285010	11	59964032	59970343	OOSP4A
ENST00000645668	581.6330987	-2.489420254	0.21595	-11.53	9.54E-31	5.97E-28	ENSG00000198860	1	184051729	184074212	TSEN15
ENST00000645831	131.7474886	-1.436309618	0.33279	-4.316	1.59E-05	0.000201	ENSG00000054598	6	1609914	1613897	FOXC1
ENST00000646209	222.2101504	-3.629008657	0.31173	-11.64	2.53E-31	1.66E-28	ENSG00000151090	3	24117152	24494850	THRB
ENST00000646241	1783.211786	2.174817451	0.17986	12.092	1.16E-33	1.01E-30	ENSG00000064601	20	45891334	45898820	CTSA
ENST00000646357	102.0599207	-1.312635459	0.39997	-3.282	0.0010314	0.006988	ENSG00000285382	20	32632182	32743523	-
ENST00000646449	140.5752594	1.028076966	0.31504	3.2633	0.0011013	0.0073856	ENSG00000197728	12	56041917	56044697	RPS26
ENST00000646685	7.599563441	6.352889973	1.82269	3.4854	0.0004913	0.0037808	ENSG00000284873	11	59938431	59957480	OOSP1
ENST00000647029	17.45842789	-2.471615854	0.85616	-2.887	0.003891	0.0206396	ENSG00000121742	13	20221961	20232319	GJB6
ENST00000647043	150.1003954	1.20556421	0.32387	3.7224	0.0001973	0.0017531	ENSG00000290854	1	1724837	1745919	-
ENST00000647211	164.59926	-1.16601699	0.29667	-3.93	8.48E-05	0.0008557	ENSG00000103494	16	53598152	53703859	RPGRIP1L
ENST00000647468	335.4850357	1.195870904	0.2527	4.7324	2.22E-06	3.61E-05	ENSG00000122335	6	158109518	158168262	SERAC1
ENST00000647584	181.7039063	-1.277626874	0.29461	-4.337	1.45E-05	0.0001853	ENSG00000132199	18	670317	712630	ENOSF1
ENST00000647589	17.19022683	2.915956972	0.90364	3.2269	0.0012514	0.0082078	ENSG00000261305	7	150337482	150343346	-
ENST00000647814	230.3565891	2.437832654	0.32709	7.4531	9.11E-14	7.31E-12	ENSG00000023839	10	99782639	99852594	ABCC2
ENST00000647856	62.86978794	1.314922531	0.45384	2.8973	0.0037634	0.0200787	ENSG00000285796	20	45926517	45935055	-
ENST00000647893	53.77743841	-3.333017097	0.54601	-6.104	1.03E-09	3.70E-08	ENSG00000197594	6	131808019	131895155	ENPP1
ENST00000647941	301.5144394	-2.474863322	0.28828	-8.585	9.09E-18	1.25E-15	ENSG00000080819	3	98579445	98593611	CPOX
ENST00000647952	7.668030263	6.364553999	1.82816	3.4814	0.0004988	0.0038288	ENSG00000290870	6	29849549	29885615	-
ENST00000647991	7.648247777	4.864633516	1.80017	2.7023	0.0068857	0.0326084	ENSG00000291261	15	22606021	22608126	-
ENST00000648011	84.51885477	-1.398740896	0.39034	-3.583	0.0003391	0.002767	ENSG00000291141	1	121519122	121575702	EMBP1
ENST00000648076	36.92101611	-1.591731316	0.62286	-2.556	0.0106034	0.0459457	ENSG00000065618	10	104031285	104085880	COL17A1
ENST00000648193	7.599563441	6.352889973	1.82269	3.4854	0.0004913	0.0037808	ENSG00000285777	1	173596059	173669837	-
ENST00000648240	383.6088206	-1.329027323	0.28737	-4.625	3.75E-06	5.70E-05	ENSG00000285526	19	35106509	35124297	-
ENST00000648610	262.294665	1.557906842	0.25907	6.0135	1.82E-09	6.22E-08	ENSG00000172375	11	119107343	119118544	C2CD2L
ENST00000648922	166.0751013	-1.325130501	0.29643	-4.47	7.81E-06	0.0001086	ENSG00000285547	X	72301810	72572843	-
ENST00000648936	8.333375981	-6.533603578	1.79271	-3.645	0.0002679	0.0022699	ENSG00000127990	7	94584979	94656133	SGCE
ENST00000649015	136.2361478	1.746765694	0.32758	5.3323	9.70E-08	2.24E-06	ENSG00000150995	3	4493347	4847506	ITPR1
ENST00000649017	33.20863183	2.958839927	0.65128	4.5431	5.54E-06	8.04E-05	ENSG00000275216	13	109272202	109278512	-
ENST00000649031	5.520883582	-5.940542468	1.95346	-3.041	0.0023577	0.013751	ENSG00000106031	7	27194363	27200091	HOXA13
ENST00000649063	1054.211388	1.14309697	0.19487	5.8661	4.46E-09	1.41E-07	ENSG00000242802	7	4775622	4794397	AP5Z1
ENST00000649141	143.7165015	-1.398504814	0.31428	-4.45	8.59E-06	0.0001179	ENSG00000206418	18	8609436	8639383	RAB12
ENST00000649169	37.00102373	8.637475072	1.53509	5.6267	1.84E-08	5.05E-07	ENSG00000268926	9	95772588	95776282	-
ENST00000649286	160.6525208	-3.318923692	0.341	-9.733	2.19E-22	5.38E-20	ENSG00000006530	7	141551409	141655244	AGK
ENST00000649488	8.464568496	6.510396325	1.79303	3.631	0.0002824	0.0023693	ENSG00000108556	17	4897770	4903098	CHRNE
ENST00000649529	776.8218561	-1.626564185	0.2878	-5.652	1.59E-08	4.44E-07	ENSG00000187608	1	1013496	1014540	ISG15
ENST00000649556	250.0535278	2.039327551	0.28373	7.1876	6.59E-13	4.62E-11	ENSG00000285952	16	4777938	4801423	-

protein_coding	Yes	NM_001355436.2	108
protein_coding	Yes	-	108
protein_coding	Yes	NM_002906.4	108
protein_coding	Yes	NM_001395277.1	108
protein_coding	Yes	NM_052965.4	108
protein_coding	Yes	NM_001453.3	108
protein_coding	Yes	NM_001354712.2	108
protein_coding	Yes	NM_000308.4	108
protein_coding	Yes	-	108
protein_coding	Yes	NM_001029.5	108
protein_coding	Yes	NM_001395276.1	108
protein_coding	Yes	NM_001110219.3	108
lncRNA	Yes	-	108
protein_coding	Yes	NM_015272.5	108
protein_coding	Yes	NM_032861.4	108
protein_coding	Yes	NM_017512.7	108
lncRNA	Yes	-	108
protein_coding	Yes	NM_000392.5	108
lncRNA	Yes	-	108
protein_coding	Yes	NM_006208.3	108
protein_coding	Yes	NM_000097.7	108
lncRNA	Yes	-	108
lncRNA	Yes	-	108
lncRNA	Yes	-	108
protein_coding	Yes	NM_000494.4	108
protein_coding	Yes	-	108
protein_coding	Yes	-	108
protein_coding	Yes	NM_001290474.2	108
protein_coding	Yes	-	108
protein_coding	Yes	NM_003919.3	108
protein_coding	Yes	NM_001378452.1	108
lncRNA	Yes	-	108
protein_coding	Yes	NM_000522.5	108
protein_coding	Yes	NM_014855.3	108
protein_coding	Yes	NM_001025300.3	108
lncRNA	Yes	-	108
protein_coding	Yes	NM_018238.4	108
protein_coding	Yes	NM_000080.4	108
protein_coding	Yes	NM_005101.4	108
lncRNA	Yes	-	108

ENST00000649796	90.47044926	-1.185494203	0.38056	-3.115	0.0018386	0.011225	ENSG00000127955	7	80134830	80226181	GNAI1
ENST00000649802	4606.862863	1.285532647	0.18739	6.8601	6.88E-12	3.92E-10	ENSG00000285565	6	31828833	31839719	-
ENST00000649903	4.104189126	5.466321343	2.09779	2.6058	0.0091672	0.0409201	ENSG00000285656	11	62116469	62123767	-
ENST00000649912	47.11615332	-1.681813291	0.51796	-3.247	0.0011664	0.0077434	ENSG00000285723	20	10420545	10420737	MKKS
ENST00000650150	15.07369943	2.719614437	1.02992	2.6406	0.008276	0.0377312	ENSG00000248323	5	91151210	91314402	LUCAT1
ENST00000650255	4.685036436	5.654179995	2.02627	2.7904	0.0052637	0.026362	ENSG00000285871	10	68896919	68900768	-
ENST00000650284	193.1059357	-1.400913933	0.28988	-4.833	1.35E-06	2.32E-05	ENSG00000285645	9	35812959	35828732	-
ENST00000650446	12.97067439	3.2245724	1.06425	3.0299	0.0024464	0.0141675	ENSG00000285877	17	67244281	67273503	-
ENST00000650553	16.64302816	4.404449846	1.15147	3.8251	0.0001307	0.0012333	ENSG00000234147	6	140575811	140898381	-
ENST00000650579	7.901398233	6.408050316	1.81583	3.529	0.0004171	0.0032923	ENSG00000205293	8	57855499	57984114	LINC01602
ENST00000650711	42.58684941	-7.439043391	1.5083	-4.932	8.14E-07	1.49E-05	ENSG00000108576	17	30194318	30235697	SLC6A4
ENST00000650758	4.1270114	5.473512357	2.08944	2.6196	0.0088031	0.0396375	ENSG00000286269	4	117648831	117657312	-
ENST00000650785	497.6248601	-1.026365626	0.217	-4.73	2.25E-06	3.65E-05	ENSG00000237188	1	147173361	147295734	-
ENST00000650932	430.7248927	-1.750041989	0.28444	-6.153	7.63E-10	2.79E-08	ENSG00000276043	19	4909500	4962154	UHRF1
ENST00000651006	30.45896119	-2.762016935	0.65958	-4.188	2.82E-05	0.00033	ENSG00000251363	14	40954692	41149309	LINC02315
ENST00000651192	85.85750736	1.11142201	0.39301	2.828	0.0046844	0.0239793	ENSG00000164011	1	42846612	42852477	ZNF691
ENST00000651205	7.901398233	6.408050316	1.81583	3.529	0.0004171	0.0032923	ENSG00000286067	12	120218069	120222668	-
ENST00000651314	65.02092674	1.365285287	0.43028	3.173	0.0015086	0.0095663	ENSG00000286007	17	7428860	7436278	-
ENST00000651358	853.9920196	2.03040172	0.21766	9.3281	1.08E-20	2.07E-18	ENSG00000164970	9	34398183	34458570	FAM219A
ENST00000651514	7.690852537	6.368446707	1.83208	3.4761	0.0005088	0.0038932	ENSG00000160868	7	99756966	99784184	CYP3A4
ENST00000651636	17.3907277	7.547354557	1.62299	4.6503	3.32E-06	5.13E-05	ENSG00000267549	19	56315261	56365316	ZSCAN5A-AS1
ENST00000651663	44.18329625	2.955151195	0.56915	5.1922	2.08E-07	4.46E-06	ENSG00000286042	6	79307668	79313384	LCAL1
ENST00000651687	87.38121179	-4.510826095	0.52645	-8.568	1.05E-17	1.43E-15	ENSG00000113356	5	90474863	90514557	POLR3G
ENST00000651706	39.23036136	-5.305861255	0.8939	-5.936	2.93E-09	9.60E-08	ENSG00000286231	1	220786989	220884503	-
ENST00000651973	9.318571984	-4.199231659	1.43347	-2.929	0.003396	0.0185027	ENSG00000286172	1	147084615	147084756	RNVU1-8
ENST00000651982	21.72982886	-2.818504598	0.78589	-3.586	0.0003353	0.0027398	ENSG00000178662	2	165469697	165689407	CSRNP3
ENST00000652155	19.69067637	2.453031592	0.795	3.0856	0.0020317	0.012178	ENSG00000235888	21	38998650	39062894	LINC02940
ENST00000652248	858.6604587	-1.61261352	0.24534	-6.573	4.93E-11	2.32E-09	ENSG00000286070	22	24556006	24628987	-
ENST00000652326	584.5374909	-1.051620915	0.21933	-4.795	1.63E-06	2.75E-05	ENSG00000100578	14	58427718	58551297	KIAA0586
ENST00000652361	104.1744136	-2.500838425	0.37662	-6.64	3.13E-11	1.55E-09	ENSG00000176928	5	75025345	75052558	GCNT4
ENST00000652409	36.7416188	-1.715738398	0.57271	-2.996	0.0027372	0.015521	ENSG00000286237	X	102712494	102753530	ARMCX5-GPRASP2
ENST00000652477	97.86703444	2.488672372	0.46467	5.3558	8.52E-08	2.00E-06	ENSG00000180155	8	142771201	142777202	LYNX1
ENST00000652629	4.172655949	5.487752313	2.08271	2.6349	0.0084159	0.0382366	ENSG00000163879	1	37556939	37566857	DNALI1
ENST00000652658	446.9354084	-1.25182309	0.23858	-5.247	1.55E-07	3.41E-06	ENSG00000082438	2	164680187	164841823	COBLL1
ENST00000652894	11.12290526	6.904434065	1.71844	4.0179	5.87E-05	0.0006246	ENSG00000272690	3	75443563	75538029	LINC02018
ENST00000653163	600.4488272	-1.218158342	0.21578	-5.645	1.65E-08	4.57E-07	ENSG00000272888	15	92819539	92898743	CHASERR
ENST00000653345	40.42661959	1.519740432	0.58182	2.612	0.0090005	0.0403385	ENSG00000238197	21	32728599	32746781	PAXBP1-AS1
ENST00000653385	15.74547206	2.557659629	0.89928	2.8441	0.0044536	0.0230104	ENSG00000215067	17	6875231	7012334	ALOX12-AS1
ENST00000653713	21.21192864	2.881023323	0.80258	3.5897	0.0003311	0.0027107	ENSG00000287985	7	64369240	64375584	-
ENST00000654272	5.151772375	5.791933645	1.97706	2.9296	0.0033943	0.0184954	ENSG00000226087	2	47225780	47240005	-

protein_coding	Yes	NM_002069.6	108
lncRNA	Yes	-	108
lncRNA	Yes	-	108
protein_coding	Yes	-	108
lncRNA	Yes	-	108
lncRNA	Yes	-	108
protein_coding	Yes	-	108
lncRNA	Yes	-	108
lncRNA	Yes	-	108
lncRNA	Yes	-	108
protein_coding	Yes	NM_001045.6	108
lncRNA	Yes	-	108
lncRNA	Yes	-	108
protein_coding	Yes	NM_001048201.3	108
lncRNA	Yes	-	108
protein_coding	Yes	NM_001242739.2	108
lncRNA	Yes	-	108
protein_coding	Yes	-	108
protein_coding	Yes	NM_001184940.2	108
protein_coding	Yes	NM_017460.6	108
lncRNA	Yes	-	108
lncRNA	Yes	-	108
protein_coding	Yes	NM_006467.3	108
protein_coding	Yes	-	108
snRNA	Yes	-	108
protein_coding	Yes	NM_001172173.2	108
lncRNA	Yes	-	108
protein_coding	Yes	-	108
protein_coding	Yes	NM_001329943.3	108
protein_coding	Yes	NM_001366737.1	108
protein_coding	Yes	-	108
protein_coding	Yes	NM_177477.4	108
protein_coding	Yes	NM_003462.5	108
protein_coding	Yes	NM_001365672.2	108
lncRNA	Yes	-	108
lncRNA	Yes	-	108
lncRNA	Yes	-	108
lncRNA	Yes	-	108
lncRNA	Yes	-	108
lncRNA	Yes	-	108

ENST00000654602	139.3741343	2.205824329	0.33838	6.5188	7.09E-11	3.20E-09	ENSG00000233461	1	231520728	231528604	-
ENST00000654773	4.639391888	5.641491239	2.02631	2.7841	0.0053674	0.0267625	ENSG00000288002	5	8207018	8366437	-
ENST00000655618	8.697936465	6.549769111	1.78595	3.6674	0.000245	0.0021081	ENSG00000237596	6	136053350	136225618	-
ENST00000655684	56.62809883	-1.894880003	0.47678	-3.974	7.06E-05	0.0007325	ENSG00000227403	2	161244719	161249095	LINC01806
ENST00000655709	24.4018205	4.181197253	0.92832	4.504	6.67E-06	9.45E-05	ENSG00000276462	9	41101041	41119909	LINC03025
ENST00000655811	10.32636702	6.794814477	1.73348	3.9198	8.86E-05	0.0008891	ENSG00000287530	13	23954492	23970188	-
ENST00000656097	10.53995251	5.343212318	1.70826	3.1279	0.0017607	0.0108448	ENSG00000287426	11	129761712	129814690	-
ENST00000656133	13.34434228	3.589798681	1.13856	3.1529	0.0016164	0.0101156	ENSG00000258017	12	49130908	49188484	-
ENST00000656249	7.599563441	6.352889973	1.82269	3.4854	0.0004913	0.0037808	ENSG00000245293	4	107863667	107978799	CYP2U1-AS1
ENST00000656384	79.46209675	1.17929502	0.40459	2.9148	0.0035594	0.0191914	ENSG00000230606	2	97416164	97429862	LINC02969
ENST00000656462	48.30432567	-1.411993579	0.48954	-2.884	0.0039227	0.0207737	ENSG00000215256	14	23938761	24052509	DHRS4-AS1
ENST00000656698	1836.302395	2.402092217	0.19216	12.5	7.45E-36	1.08E-32	ENSG00000250920	4	103550588	103559277	-
ENST00000656825	4566.740833	-1.107893775	0.1849	-5.992	2.08E-09	7.03E-08	ENSG00000070831	1	22052708	22101360	CDC42
ENST00000656931	4.895582131	5.718672064	2.00026	2.859	0.0042502	0.0221757	ENSG00000227431	20	49040463	49046168	CSE1L-DT
ENST00000656939	50.19472792	3.144447455	0.59582	5.2775	1.31E-07	2.93E-06	ENSG00000287126	2	3568504	3575100	-
ENST00000657113	8.782292487	4.034661031	1.42511	2.8311	0.0046385	0.0237899	ENSG00000288568	16	87493305	87516940	ZCCHC14-DT
ENST00000657329	15.28134618	-2.402254369	0.88842	-2.704	0.0068521	0.0324855	ENSG00000227885	6	53739265	53794904	-
ENST00000657350	7.625425503	4.860669716	1.79883	2.7021	0.0068896	0.032621	ENSG00000286403	5	139503241	139511374	-
ENST00000657417	4.616569613	5.635108028	2.03024	2.7756	0.0055102	0.0273019	ENSG00000287529	2	219198639	219200034	-
ENST00000657742	5.316673522	5.839599379	1.9681	2.9671	0.003006	0.0167165	ENSG00000236404	9	2421604	2622373	VLDLR-AS1
ENST00000657887	56.55685839	1.68168267	0.47393	3.5484	0.0003876	0.0030965	ENSG00000286540	6	120462510	120706789	-
ENST00000658424	8.020654831	6.431963051	1.80557	3.5623	0.0003676	0.0029587	ENSG00000288079	3	113259785	113266824	-
ENST00000658766	31.3070227	1.986572504	0.62073	3.2004	0.0013725	0.0088501	ENSG00000241764	7	107739998	107743595	CBLL1-AS1
ENST00000658780	25.06328783	-2.793934579	0.73034	-3.826	0.0001305	0.0012313	ENSG00000110427	11	33376107	33674102	KIAA1549L
ENST00000658787	18.27372586	2.048872211	0.80817	2.5352	0.0112381	0.0479856	ENSG00000227619	9	129575183	129584553	-
ENST00000658822	6.478368142	6.120364618	1.90225	3.2174	0.0012934	0.0084367	ENSG00000230550	1	204141403	204143327	ERLNC1
ENST00000658933	8.231200526	6.469917093	1.80057	3.5933	0.0003266	0.0026796	ENSG00000255553	11	62049862	62081968	LINC02733
ENST00000658960	7.835807958	3.873995268	1.49384	2.5933	0.0095054	0.0420912	ENSG00000287721	17	28204615	28223735	-
ENST00000659148	64.31702088	-1.588263198	0.45085	-3.523	0.0004269	0.0033579	ENSG00000174365	20	38446342	38450940	SNHG11
ENST00000659152	88.65460954	-3.333660026	0.4455	-7.483	7.27E-14	5.91E-12	ENSG00000287190	1	146050440	146052244	-
ENST00000659750	27.71522449	3.481822468	0.75293	4.6244	3.76E-06	5.71E-05	ENSG00000228192	1	42832521	42846414	ZNF691-DT
ENST00000660525	5.755441959	5.949133731	1.96147	3.033	0.0024214	0.014054	ENSG00000249771	4	41883270	41935075	-
ENST00000660618	170.7411671	-1.86568831	0.32833	-5.682	1.33E-08	3.78E-07	ENSG00000285646	1	11908151	11914298	-
ENST00000661181	82.67716165	9.797468036	1.49102	6.571	5.00E-11	2.35E-09	ENSG00000286299	6	125268086	125274828	-
ENST00000661266	12.2617776	7.043023371	1.69042	4.1664	3.09E-05	0.000358	ENSG00000288067	8	134190382	134390487	-
ENST00000661295	5.829054009	5.971610018	1.92358	3.1044	0.0019065	0.0115479	ENSG00000253123	8	37326605	37331991	-
ENST00000661319	76.35041244	1.837991486	0.41123	4.4696	7.84E-06	0.0001089	ENSG00000287356	1	2315039	2323085	-
ENST00000661493	22.11139258	-3.015867445	0.78827	-3.826	0.0001303	0.0012297	ENSG00000245694	16	54852902	54928843	CRNDE
ENST00000661498	3.893643431	5.3891778	2.12058	2.5414	0.0110418	0.0473143	ENSG00000287549	X	41008426	41029306	-
ENST00000661543	1952.278658	3.469966609	0.18394	18.865	2.21E-79	2.68E-75	ENSG00000077150	10	102395704	102402524	NFKB2

lncRNA	Yes	-	108
lncRNA	Yes	-	108
lncRNA	Yes	-	108
lncRNA	Yes	-	108
lncRNA	Yes	-	108
lncRNA	Yes	-	108
lncRNA	Yes	-	108
lncRNA	Yes	-	108
lncRNA	Yes	-	108
lncRNA	Yes	-	108
lncRNA	Yes	-	108
lncRNA	Yes	-	108
protein_coding	Yes	NM_001791.4	108
lncRNA	Yes	-	108
lncRNA	Yes	-	108
lncRNA	Yes	-	108
lncRNA	Yes	-	108
lncRNA	Yes	-	108
lncRNA	Yes	-	108
lncRNA	Yes	-	108
lncRNA	Yes	-	108
lncRNA	Yes	-	108
lncRNA	Yes	-	108
protein_coding	Yes	NM_012194.3	108
lncRNA	Yes	-	108
lncRNA	Yes	-	108
lncRNA	Yes	-	108
lncRNA	Yes	-	108
lncRNA	Yes	-	108
lncRNA	Yes	-	108
lncRNA	Yes	-	108
lncRNA	Yes	-	108
lncRNA	Yes	-	108
lncRNA	Yes	-	108
lncRNA	Yes	-	108
lncRNA	Yes	-	108
lncRNA	Yes	-	108
lncRNA	Yes	-	108
lncRNA	Yes	-	108
protein_coding	Yes	NM_001322934.2	108

ENST00000661978	4.616569613	5.635108028	2.03024	2.7756	0.0055102	0.0273019	ENSG00000287624	1	156768104	156769233	-
ENST00000662437	53.20506793	-2.824542082	0.51612	-5.473	4.43E-08	1.11E-06	ENSG00000250682	5	102608478	102671765	LINC00491
ENST00000662507	12.70256909	2.880606562	1.04304	2.7617	0.0057494	0.0282075	ENSG00000286782	9	35012962	35016076	-
ENST00000662856	10.08233593	3.14410663	1.23568	2.5444	0.0109454	0.0470148	ENSG00000233058	3	194487145	194521154	ATP13A3-DT
ENST00000662918	6.17653335	6.052649114	1.90546	3.1765	0.0014907	0.0094747	ENSG00000286590	6	20321460	20333193	-
ENST00000663040	34.77824195	2.935563898	0.64599	4.5443	5.51E-06	8.01E-05	ENSG00000287245	11	109946580	109975996	-
ENST00000663232	299.6084086	1.019202023	0.25207	4.0434	5.27E-05	0.0005685	ENSG00000224032	5	112160525	112164818	EPB41L4A-AS1
ENST00000663724	11.39194491	5.467316812	1.6945	3.2265	0.0012531	0.0082174	ENSG00000232618	6	142946405	142957945	HIVEP2-DT
ENST00000664044	4.662214162	5.647844874	2.02495	2.7891	0.0052851	0.026438	ENSG00000267535	17	76849839	76861836	LINC00868
ENST00000664962	9.164672404	6.625423341	1.77325	3.7363	0.0001867	0.0016714	ENSG00000179935	20	18786085	18794574	LINC00652
ENST00000665340	27.31610188	2.86519437	0.71122	4.0286	5.61E-05	0.0006003	ENSG00000228775	7	141704002	141738230	WEE2-AS1
ENST00000665637	57.95694088	-4.504874952	0.62714	-7.183	6.81E-13	4.75E-11	ENSG00000136237	7	22118235	22357154	RAPGEF5
ENST00000666035	4.195478223	5.494829583	2.0841	2.6366	0.0083753	0.0380939	ENSG00000287760	16	68448842	68450002	-
ENST00000666136	390.0049079	3.483743743	0.26693	13.051	6.24E-39	1.11E-35	ENSG00000268621	19	46189028	46203083	IGFL2-AS1
ENST00000666402	24.70738534	4.979074693	1.03655	4.8035	1.56E-06	2.65E-05	ENSG00000268460	19	46201579	46214838	-
ENST00000666693	11.30548345	6.925561846	1.71059	4.0486	5.15E-05	0.0005583	ENSG00000286833	2	132347472	132365130	-
ENST00000666926	8.697936465	6.549769111	1.78595	3.6674	0.000245	0.0021081	ENSG00000255197	11	47381508	47409271	-
ENST00000666966	7.787286862	6.389180714	1.81459	3.521	0.0004299	0.0033787	ENSG00000287170	19	55116419	55123169	-
ENST00000667279	4.337557096	5.546371557	2.07077	2.6784	0.0073974	0.034454	ENSG00000277047	13	54938603	54986717	-
ENST00000667941	38.08469648	-1.695306111	0.57675	-2.939	0.0032884	0.0180088	ENSG00000287713	12	7838913	7844395	-
ENST00000668057	9.7848182	-5.294381543	1.7266	-3.066	0.0021668	0.0128417	ENSG00000286984	8	88809948	88813144	-
ENST00000668344	15.69662448	2.564384656	0.89006	2.8811	0.0039624	0.0209486	ENSG00000258733	14	85934608	86161539	LINC02328
ENST00000668940	4.964048954	5.736725682	2.00756	2.8576	0.0042692	0.0222548	ENSG00000286599	4	55889742	55903177	-
ENST00000669254	20.00020646	1.994547837	0.78212	2.5502	0.0107662	0.0464588	ENSG00000272695	13	113864111	113866834	GAS6-DT
ENST00000669835	20.74916837	7.802583231	1.59601	4.8888	1.01E-06	1.81E-05	ENSG00000287382	4	55938152	55947996	-
ENST00000669870	215.4711277	-1.287641649	0.2763	-4.66	3.16E-06	4.91E-05	ENSG00000269994	9	87008514	87042419	LINC02893
ENST00000669890	7.320550923	6.299607737	1.83481	3.4334	0.0005961	0.0044428	ENSG00000235142	6	106705333	106787521	LINC02532
ENST00000670397	148.9999286	1.01429113	0.31705	3.1992	0.0013783	0.0088802	ENSG00000231074	6	30286702	30327362	HCG18
ENST00000670559	5.385140344	5.856176292	1.95624	2.9936	0.0027572	0.0156051	ENSG00000223704	22	26860552	26968763	LINC01422
ENST00000670972	22.73435045	1.991427734	0.7253	2.7457	0.006039	0.029379	ENSG00000288095	5	141320979	141325985	-
ENST00000671199	129.1012149	1.629343217	0.33032	4.9326	8.12E-07	1.48E-05	ENSG00000223891	20	44210906	44226027	OSER1-DT
ENST00000671505	9.063092852	6.603810156	1.82587	3.6168	0.0002983	0.0024836	ENSG00000287853	20	34132269	34136485	-
ENST00000672341	817.2428626	-1.26348221	0.1974	-6.401	1.55E-10	6.51E-09	ENSG00000164022	4	106316549	106349456	AIMP1
ENST00000672957	846.1977927	1.184918319	0.20016	5.9199	3.22E-09	1.05E-07	ENSG00000138303	10	72096031	72216276	ASCC1
ENST00000673801	574.4121022	-2.451499444	0.21726	-11.28	1.58E-29	9.25E-27	ENSG00000121281	16	50266550	50318135	ADCY7
ENST00000673955	8.999771257	6.598017852	1.76981	3.7281	0.0001929	0.001719	ENSG00000236719	1	180509379	180566523	OVAAL
ENST00000673966	6.856071698	-6.25125206	1.8644	-3.353	0.0007996	0.005678	ENSG00000132746	11	67662154	67674623	ALDH3B2
ENST00000674020	5.857021511	5.982236288	2.07982	2.8763	0.0040233	0.0212165	ENSG00000260239	6	4488797	4494009	LINC02533
ENST00000674022	11.03846685	2.947591878	1.09765	2.6854	0.007245	0.0339168	ENSG00000263072	16	3100695	3143734	ZNF213-AS1
ENST00000674313	47.70282563	2.459013882	0.52744	4.6622	3.13E-06	4.87E-05	ENSG00000155465	14	22773221	22815435	SLC7A7

lncRNA	Yes	-	108
lncRNA	Yes	-	108
lncRNA	Yes	-	108
lncRNA	Yes	-	108
lncRNA	Yes	-	108
lncRNA	Yes	-	108
lncRNA	Yes	-	108
lncRNA	Yes	-	108
lncRNA	Yes	-	108
lncRNA	Yes	-	108
lncRNA	Yes	-	108
protein_coding	Yes	NM_012294.5	108
lncRNA	Yes	-	108
lncRNA	Yes	-	108
lncRNA	Yes	-	108
lncRNA	Yes	-	108
lncRNA	Yes	-	108
lncRNA	Yes	-	108
lncRNA	Yes	-	108
lncRNA	Yes	-	108
lncRNA	Yes	-	108
lncRNA	Yes	-	108
lncRNA	Yes	-	108
lncRNA	Yes	-	108
lncRNA	Yes	-	108
lncRNA	Yes	-	108
lncRNA	Yes	-	108
lncRNA	Yes	-	108
lncRNA	Yes	-	108
lncRNA	Yes	-	108
lncRNA	Yes	-	108
lncRNA	Yes	-	108
protein_coding	Yes	NM_001142416.2	108
protein_coding	Yes	NM_001198800.3	108
protein_coding	Yes	NM_001114.5	108
lncRNA	Yes	-	108
protein_coding	Yes	NM_001393402.2	108
lncRNA	Yes	-	108
lncRNA	Yes	-	108
protein_coding	Yes	NM_003982.4	108

ENST00000675225	15.63796728	2.564557289	0.90084	2.8468	0.0044155	0.0228428	ENSG00000288612	6	3023471	3027426	-
ENST00000675367	182.0895031	1.592720305	0.30485	5.2245	1.75E-07	3.81E-06	ENSG00000129667	17	76470895	76501423	RHBDF2
ENST00000675561	115.5601754	-1.011822947	0.33642	-3.008	0.0026333	0.0150443	ENSG00000182923	3	134486056	134565031	CEP63
ENST00000675830	18.24000526	2.611575887	0.93719	2.7866	0.0053264	0.0266046	ENSG00000290691	15	43699440	43718260	STRCP1
ENST00000676095	9.699875166	6.706538294	1.75072	3.8307	0.0001278	0.0012082	ENSG00000228509	2	190672296	190841864	-
ENST00000676153	287.4569682	-1.094831943	0.25059	-4.369	1.25E-05	0.0001632	ENSG00000133019	1	239386567	239915450	CHRM3
ENST00000676302	159.6599142	-2.037954502	0.30962	-6.582	4.64E-11	2.20E-09	ENSG00000188158	X	17375199	17735994	NHS
ENST00000678225	683.5213244	-1.864370299	0.21887	-8.518	1.62E-17	2.14E-15	ENSG00000085982	2	233475525	233566782	USP40
ENST00000679707	27.14807414	-1.795978863	0.66783	-2.689	0.007161	0.0336082	ENSG00000288695	4	55346227	55388139	-
ENST00000679887	33.40454916	3.184967743	0.69187	4.6034	4.16E-06	6.26E-05	ENSG00000087589	20	56412259	56460382	CASS4
ENST00000680972	639.6706374	1.11113477	0.21036	5.2821	1.28E-07	2.86E-06	ENSG00000089060	12	113298770	113335109	SLC8B1
ENST00000681994	4.428846192	5.573362803	2.05277	2.715	0.0066267	0.0316529	ENSG00000288704	19	1245889	1249559	-
ENST00000682035	5.339495796	5.845156443	1.96201	2.9792	0.0028903	0.0162004	ENSG00000188729	3	191199240	191265615	OSTN
ENST00000682144	10.98732973	-3.023205349	1.12743	-2.681	0.0073294	0.0342388	ENSG00000137338	6	28281571	28302549	PGBD1
ENST00000682506	182.2729806	-2.094399972	0.29836	-7.02	2.22E-12	1.42E-10	ENSG00000160145	3	124033368	124726325	KALRN
ENST00000682553	391.07272	-1.20247979	0.22818	-5.27	1.36E-07	3.04E-06	ENSG00000179046	4	188091271	188109603	TRIML2
ENST00000682710	220.1781757	-1.293234354	0.28102	-4.602	4.19E-06	6.29E-05	ENSG00000219545	7	7640751	7879223	UMAD1
ENST00000682763	126.7899853	-1.88860125	0.37837	-4.991	5.99E-07	1.13E-05	ENSG00000176438	14	95407265	95516650	SYNE3
ENST00000682840	37.2276475	-1.733405651	0.56812	-3.051	0.00228	0.0133785	ENSG00000150722	2	181985852	182117756	PPP1R1C
ENST00000682928	872.1846288	-1.071870613	0.20345	-5.268	1.38E-07	3.06E-06	ENSG00000182986	19	52876179	52897635	ZNF320
ENST00000682950	31.03300106	2.113314369	0.62654	3.373	0.0007435	0.0053492	ENSG00000140839	16	74408630	74421478	CLEC18B
ENST00000682992	219.37772	1.348059363	0.27039	4.9857	6.17E-07	1.16E-05	ENSG00000164663	6	41789895	41895375	USP49
ENST00000683033	132.1441655	-1.895014491	0.38562	-4.914	8.91E-07	1.61E-05	ENSG00000150471	4	61200325	62078335	ADGRL3
ENST00000683046	138.5818087	-1.332746027	0.31774	-4.195	2.73E-05	0.0003215	ENSG00000186314	5	145756343	145835342	PRELID2
ENST00000683051	38.54762625	-2.476346144	0.58088	-4.263	2.02E-05	0.0002469	ENSG00000228956	3	18527206	18923280	SATB1-AS1
ENST00000683139	1083.998419	-1.116857478	0.18814	-5.936	2.91E-09	9.57E-08	ENSG00000206560	3	15667235	15797979	ANKRD28
ENST00000683415	276.1942801	1.559489924	0.27135	5.7473	9.07E-09	2.67E-07	ENSG00000215252	15	34525094	34583651	GOLGA8B
ENST00000683636	21.17118368	1.869674022	0.733	2.5507	0.0107505	0.0464041	ENSG00000268500	19	51610959	51630401	SIGLEC5
ENST00000683666	383.7812138	1.14255739	0.24779	4.611	4.01E-06	6.05E-05	ENSG00000143409	1	150996834	151006902	MINDY1
ENST00000683778	766.9283733	1.16052971	0.20286	5.7208	1.06E-08	3.08E-07	ENSG00000197279	6	28080567	28089563	ZNF165
ENST00000683779	276.2577201	1.346010059	0.25302	5.3198	1.04E-07	2.38E-06	ENSG00000172687	19	21358999	21388582	ZNF738
ENST00000683842	34.10268406	-3.303428133	0.6854	-4.82	1.44E-06	2.46E-05	ENSG00000139973	14	61812702	62112550	SYT16
ENST00000683939	52.2276379	-1.981947713	0.50939	-3.891	9.99E-05	0.0009812	ENSG00000084453	12	21264599	21334917	SLCO1A2
ENST00000683975	26.47643844	-6.739296611	1.61578	-4.171	3.03E-05	0.0003518	ENSG00000134864	13	100530179	100588789	GGACT
ENST00000684293	2388.225582	-1.802871556	0.17802	-10.13	4.18E-24	1.27E-21	ENSG00000091409	2	172427585	172506459	ITGA6
ENST00000684302	9.946092546	5.267013857	1.72621	3.0512	0.0022793	0.0133761	ENSG00000111181	12	190080	214157	SLC6A12
ENST00000684388	2329.860975	-2.202035679	0.1766	-12.47	1.10E-35	1.58E-32	ENSG00000288725	16	56430555	56501497	-
ENST00000684578	12.4026765	2.861614542	1.05982	2.7001	0.006932	0.0327867	ENSG00000100505	14	50975265	51095105	TRIM9
ENST00000684583	107.6013489	2.528100281	0.37288	6.7799	1.20E-11	6.50E-10	ENSG00000063438	5	321713	438285	AHRR
ENST00000684770	113.7576918	-1.301116304	0.35817	-3.633	0.0002805	0.0023569	ENSG00000170379	7	143620973	143730410	TCAF2

lncRNA	Yes	-	108
protein_coding	Yes	NM_001005498.4	108
protein_coding	Yes	NM_001353108.3	108
lncRNA	Yes	-	108
lncRNA	Yes	-	108
protein_coding	Yes	NM_001375978.1	108
protein_coding	Yes	NM_001291867.2	108
protein_coding	Yes	NM_001365479.2	108
protein_coding	Yes	-	108
protein_coding	Yes	NM_020356.4	108
protein_coding	Yes	NM_001358345.2	108
lncRNA	Yes	-	108
protein_coding	Yes	NM_198184.2	108
protein_coding	Yes	NM_032507.4	108
protein_coding	Yes	NM_001388419.1	108
protein_coding	Yes	NM_173553.4	108
protein_coding	Yes	NM_001302348.2	108
protein_coding	Yes	NM_152592.6	108
protein_coding	Yes	NM_001080545.3	108
protein_coding	Yes	NM_001351774.2	108
protein_coding	Yes	NM_001385193.1	108
protein_coding	Yes	NM_001286554.2	108
protein_coding	Yes	NM_001387552.1	108
protein_coding	Yes	NM_205846.3	108
lncRNA	Yes	-	108
protein_coding	Yes	NM_001349278.2	108
protein_coding	Yes	NM_001023567.5	108
protein_coding	Yes	NM_003830.4	108
protein_coding	Yes	NM_001376665.1	108
protein_coding	Yes	NM_001376491.1	108
protein_coding	Yes	NM_001355237.2	108
protein_coding	Yes	NM_001367656.1	108
protein_coding	Yes	NM_001386879.1	108
protein_coding	Yes	NM_001195087.2	108
protein_coding	Yes	NM_000210.4	108
protein_coding	Yes	NM_001122848.3	108
protein_coding	Yes	-	108
protein_coding	Yes	NM_001387360.1	108
protein_coding	Yes	NM_001377236.1	108
protein_coding	Yes	NM_001363538.2	108

ENST00000684848	265.8070276	1.657693354	0.25875	6.4064	1.49E-10	6.29E-09	ENSG00000188385	10	132065945	132184858	JAKMIP3
ENST00000685419	159.7421089	1.107960824	0.36497	3.0358	0.0023994	0.013949	ENSG00000214021	3	9810261	9836365	TTLL3
ENST00000685586	5.128950101	5.786184995	1.97811	2.9251	0.0034433	0.0186933	ENSG00000288806	3	123701307	123713035	-
ENST00000685924	462.9290134	-1.099118864	0.22471	-4.891	1.00E-06	1.79E-05	ENSG00000284906	15	30626128	30773002	-
ENST00000685964	300.2144549	-1.78255532	0.27247	-6.542	6.06E-11	2.78E-09	ENSG00000089682	X	107061884	107118822	RBM41
ENST00000686162	55.40399549	1.926707303	0.50718	3.7989	0.0001454	0.00135	ENSG00000289562	11	62574173	62575097	-
ENST00000686237	40.08254536	2.221026122	0.56628	3.9221	8.78E-05	0.0008818	ENSG00000284713	11	69155934	69162440	SMIM38
ENST00000686413	192.7369976	2.365769932	0.35331	6.6961	2.14E-11	1.10E-09	ENSG00000048052	7	18495748	19002416	HDAC9
ENST00000686815	13.9977849	2.357067435	0.93554	2.5195	0.0117535	0.0496991	ENSG00000289009	21	44873984	44875807	-
ENST00000687007	62.58013948	-1.251694312	0.444	-2.819	0.0048152	0.0245188	ENSG00000288811	22	18349941	18361190	-
ENST00000687343	21.39101646	-4.81092922	1.06491	-4.518	6.25E-06	8.92E-05	ENSG00000237943	10	6580505	6615954	PRKCQ-AS1
ENST00000687749	32.18621885	1.562867581	0.61822	2.528	0.0114709	0.0487563	ENSG00000289554	1	26881108	26882235	-
ENST00000688186	22.42234673	-3.387224758	0.81622	-4.15	3.33E-05	0.0003815	ENSG00000289357	8	25508778	25509735	-
ENST00000688188	581.6835397	-1.501155831	0.21614	-6.945	3.78E-12	2.27E-10	ENSG00000149474	20	18137862	18188035	KAT14
ENST00000688475	17.39461056	2.110512236	0.82252	2.5659	0.010291	0.0448538	ENSG00000271133	7	20328288	20331781	ITGB8-AS1
ENST00000688549	4.149833674	5.480651158	2.08437	2.6294	0.0085535	0.0387013	ENSG00000288956	9	129884327	129885874	-
ENST00000688585	708.3338207	1.616573122	0.20521	7.8777	3.33E-15	3.29E-13	ENSG00000289316	8	133237170	133237927	-
ENST00000688720	153.1139275	2.439708809	0.37399	6.5235	6.87E-11	3.11E-09	ENSG00000196562	20	47657405	47785481	SULF2
ENST00000688948	35.61685639	1.879156995	0.5894	3.1883	0.0014312	0.0091668	ENSG00000064787	20	53943540	54070594	BCAS1
ENST00000689451	33.75639513	-1.620857567	0.58227	-2.784	0.0053745	0.0267895	ENSG00000289174	12	88781255	88847391	-
ENST00000689524	58.70652889	-1.916388101	0.51716	-3.706	0.0002109	0.0018554	ENSG00000289396	3	197073878	197075341	-
ENST00000689584	311.6350889	-1.262764113	0.26975	-4.681	2.85E-06	4.50E-05	ENSG00000181218	1	228457363	228457873	H2AW
ENST00000689635	296.7306707	-2.173032072	0.26176	-8.302	1.03E-16	1.22E-14	ENSG00000123094	12	25958681	26072869	RASSF8
ENST00000689782	17.30458383	7.542762631	1.63985	4.5997	4.23E-06	6.35E-05	ENSG00000215039	12	6439160	6451568	CD27-AS1
ENST00000689808	6.085244253	6.033340122	1.9048	3.1674	0.0015379	0.0097108	ENSG00000257496	12	46384288	46494474	-
ENST00000690102	39.07919147	2.309117897	0.56814	4.0643	4.82E-05	0.000526	ENSG00000291043	10	73730572	73737790	GLUD1P3
ENST00000690414	13.38935657	-2.545695668	0.96451	-2.639	0.0083061	0.0378459	ENSG00000289463	2	71419973	71422314	-
ENST00000690974	93.60969311	1.246867944	0.36919	3.3773	0.0007321	0.0052779	ENSG00000291214	17	68101873	68161227	-
ENST00000690982	1619.716	-1.832457111	0.19169	-9.559	1.18E-21	2.61E-19	ENSG00000164023	4	107824931	107915047	SGMS2
ENST00000691001	17.90310819	7.589066861	1.61891	4.6878	2.76E-06	4.38E-05	ENSG00000228063	1	218976671	219173768	LYPLAL1-DT
ENST00000691134	4.328834636	-5.58743528	2.0747	-2.693	0.0070785	0.033314	ENSG00000289171	16	24550350	24550660	-
ENST00000691431	18.20134336	-2.668254672	0.85511	-3.12	0.0018063	0.0110751	ENSG00000289145	8	17226028	17228085	-
ENST00000691526	38.86094159	2.36727434	0.57624	4.1081	3.99E-05	0.0004476	ENSG00000285533	11	65662987	65671718	RELA-DT
ENST00000691638	19.46659101	2.152898338	0.82296	2.6161	0.0088953	0.0399691	ENSG00000188766	19	38388696	38399587	SPRED3
ENST00000691740	18.98785916	2.857734832	0.89661	3.1873	0.0014363	0.0091934	ENSG00000289291	1	28736043	28737670	-
ENST00000691844	8.969852668	3.458238549	1.33908	2.5826	0.0098071	0.04316	ENSG00000289332	13	23715151	23735387	-
ENST00000692061	16.45803921	-2.688980562	0.89436	-3.007	0.0026418	0.0150799	ENSG00000278175	9	39709534	39810112	GLIDR
ENST00000692248	559.1450308	-1.390731878	0.23953	-5.806	6.40E-09	1.95E-07	ENSG00000288864	15	32615447	32697095	ARHGAP11A-SCG5
ENST00000692281	2261.626422	-1.106537047	0.1798	-6.154	7.54E-10	2.77E-08	ENSG00000289027	7	92112175	92245924	-
ENST00000692495	3.893643431	5.3891778	2.12058	2.5414	0.0110418	0.0473143	ENSG00000254812	8	143620585	143632783	-

protein_coding	Yes	NM_001323087.2	108
protein_coding	Yes	NM_001387446.1	108
lncRNA	Yes	-	108
protein_coding	Yes	-	108
protein_coding	Yes	NM_001324242.2	108
lncRNA	Yes	-	108
protein_coding	Yes	NM_001369201.2	108
protein_coding	Yes	NM_178425.4	108
lncRNA	Yes	-	108
lncRNA	Yes	-	108
lncRNA	Yes	-	108
lncRNA	Yes	-	108
lncRNA	Yes	-	108
protein_coding	Yes	NM_001392073.1	108
lncRNA	Yes	-	108
lncRNA	Yes	-	108
lncRNA	Yes	-	108
protein_coding	Yes	NM_001387048.1	108
protein_coding	Yes	NM_001366298.2	108
lncRNA	Yes	-	108
lncRNA	Yes	-	108
protein_coding	Yes	NM_033445.3	108
protein_coding	Yes	NM_001394098.1	108
lncRNA	Yes	-	108
lncRNA	Yes	-	108
lncRNA	Yes	-	108
lncRNA	Yes	-	108
lncRNA	Yes	-	108
protein_coding	Yes	NM_001375905.1	108
lncRNA	Yes	-	108
lncRNA	Yes	-	108
lncRNA	Yes	-	108
lncRNA	Yes	-	108
protein_coding	Yes	NM_001394336.1	108
lncRNA	Yes	-	108
lncRNA	Yes	-	108
lncRNA	Yes	-	108
protein_coding	Yes	-	108
protein_coding	Yes	-	108
lncRNA	Yes	-	108

ENST00000692570	127.7674067	-1.504160626	0.33599	-4.477	7.58E-06	0.0001058	ENSG00000289194	11	62153729	62154783	-
ENST00000692721	381.8442035	1.057915301	0.24447	4.3273	1.51E-05	0.0001923	ENSG00000289001	21	36070023	36070700	-
ENST00000692801	65.06138312	-3.147526578	0.48743	-6.457	1.07E-10	4.67E-09	ENSG00000291096	3	125928688	125990573	-
ENST00000693095	604.7665918	-1.492677934	0.2723	-5.482	4.21E-08	1.06E-06	ENSG00000196890	1	228458102	228458558	H2BU1
ENST00000693548	105.6701671	2.073469764	0.42734	4.8521	1.22E-06	2.13E-05	ENSG00000144821	3	108380367	108510596	MYH15
ENST00000693711	210.1717628	-1.271145176	0.27377	-4.643	3.43E-06	5.28E-05	ENSG00000285077	15	30625965	30649529	ARHGAP11B
ENST00000695012	3.893643431	5.3891778	2.12058	2.5414	0.0110418	0.0473143	ENSG00000133958	14	93430938	93707876	UNC79
ENST00000695562	39.16768641	3.022925093	0.60516	4.9952	5.88E-07	1.12E-05	ENSG00000289688	13	23505237	23535314	-
ENST00000695563	19.22004715	2.018788295	0.78101	2.5849	0.0097421	0.0429256	ENSG00000198156	16	28342516	28363508	NPIPB6
ENST00000695795	591.3890795	2.249266316	0.21405	10.508	7.94E-26	2.83E-23	ENSG00000178104	1	148844427	149033016	PDE4DIP
ENST00000695855	1648.647224	-1.047643024	0.19303	-5.427	5.72E-08	1.40E-06	ENSG00000289694	1	22025141	22091559	-
ENST00000695929	526.159892	-1.33436587	0.26973	-4.947	7.53E-07	1.39E-05	ENSG00000289695	12	50924968	50970700	-
ENST00000695948	87.89430437	-1.231830355	0.3909	-3.151	0.0016256	0.0101632	ENSG00000124496	6	42224930	42451926	TRERF1
ENST00000696032	8.100334493	-5.013651376	1.77922	-2.818	0.0048339	0.0245922	ENSG00000289697	1	196651851	196795393	-
ENST00000696146	20.18311113	2.234019843	0.76863	2.9065	0.003655	0.019607	ENSG00000261587	8	144353227	144354931	TMEM249
ENST00000696174	73.57803474	1.205100797	0.40751	2.9573	0.0031039	0.0171638	ENSG00000188897	16	11372014	11527247	-
ENST00000696247	290.344121	1.983994716	0.34226	5.7967	6.76E-09	2.06E-07	ENSG00000188707	7	150329870	150331115	ZBED10P
ENST00000697408	37.92206997	1.675353279	0.57222	2.9278	0.0034134	0.0185758	ENSG00000289728	1	247332330	247448603	-
ENST00000697736	25.35685229	2.190236519	0.69034	3.1727	0.0015103	0.0095738	ENSG00000196381	19	37668578	37676393	ZNF781
ENST00000697990	1369.655984	2.147906439	0.18523	11.596	4.32E-31	2.77E-28	ENSG00000289738	10	70815988	70958701	-
ENST00000698129	163.8084925	1.195865017	0.41813	2.8601	0.0042355	0.0221226	ENSG00000289740	11	65499311	65507432	TALAM1
ENST00000698562	108.4853429	1.646942261	0.35246	4.6727	2.97E-06	4.67E-05	ENSG00000289748	19	37696862	37697402	-
ENST00000698857	455.4903379	-2.99585476	0.26546	-11.29	1.55E-29	9.11E-27	ENSG00000065882	4	37891083	38139173	TBC1D1
ENST00000699294	168.1236692	1.756200881	0.34447	5.0982	3.43E-07	6.99E-06	ENSG00000180881	12	75275978	75330324	CAPS2
ENST00000701183	18.27466175	4.527721946	1.09485	4.1355	3.54E-05	0.0004033	ENSG00000287865	7	155203670	155215013	-
ENST00000701211	3.810374571	-5.403736545	2.13911	-2.526	0.0115316	0.0489523	ENSG00000289866	14	50668404	50669009	-
ENST00000701348	9.466507196	6.671265375	1.75669	3.7976	0.0001461	0.001355	ENSG00000289885	3	153161418	153162036	-
ENST00000702115	11.93712054	7.005282867	1.69594	4.1306	3.62E-05	0.0004109	ENSG00000289959	X	13089739	13091216	-
ENST00000702229	8.069339168	4.946277106	1.77997	2.7789	0.0054551	0.0270968	ENSG00000289975	6	33247975	33249215	-
ENST00000702504	950.2867846	-1.686271641	0.2387	-7.065	1.61E-12	1.05E-10	ENSG00000290018	2	174719820	174720853	-
ENST00000702591	5.59568604	5.912412081	1.94004	3.0476	0.002307	0.0135058	ENSG00000290035	3	127218942	127227213	-
ENST00000702614	10.51045769	-5.400273412	1.71525	-3.148	0.0016417	0.0102475	ENSG00000290039	17	58659124	58659306	-
ENST00000702711	333.4299967	2.013599957	0.26547	7.5851	3.32E-14	2.84E-12	ENSG00000290058	19	32693040	32693529	-
ENST00000702873	30.71544031	2.335721973	0.65033	3.5916	0.0003286	0.0026942	ENSG00000290082	17	29567822	29568533	-
ENST00000703064	4.536014061	-5.656442023	2.03958	-2.773	0.0055484	0.027444	ENSG00000290124	1	247189575	247189725	-

lncRNA	Yes	-	108
lncRNA	Yes	-	108
lncRNA	Yes	-	108
protein_coding	Yes	NM_175055.3	108
protein_coding	Yes	NM_014981.3	108
protein_coding	Yes	-	108
protein_coding	Yes	NM_001395159.1	108
lncRNA	Yes	-	108
protein_coding	Yes	NM_001395275.1	108
protein_coding	Yes	NM_001395426.1	108
protein_coding	Yes	-	108
protein_coding	Yes	-	108
protein_coding	Yes	NM_001395490.1	108
protein_coding	Yes	-	108
protein_coding	Yes	NM_001280561.2	108
protein_coding	Yes	NM_001370704.1	108
ribed_unitary_pseud	Yes	-	108
lncRNA	Yes	-	108
ed_unprocessed_ps	Yes	-	108
lncRNA	Yes	-	108
lncRNA	Yes	-	108
bed_processed_pse	Yes	-	108
protein_coding	Yes	NM_001396959.1	108
protein_coding	Yes	NM_001355024.4	108
lncRNA	Yes	-	108
lncRNA	Yes	-	108
lncRNA	Yes	-	108
lncRNA	Yes	-	108
lncRNA	Yes	-	108
lncRNA	Yes	-	108
lncRNA	Yes	-	108
lncRNA	Yes	-	108
lncRNA	Yes	-	108
lncRNA	Yes	-	108
lncRNA	Yes	-	108
