# Supplementary material for: Combined loss of CDH1 and downstream regulatory sequences drive early-onset diffuse gastric cancer and increase penetrance of hereditary diffuse gastric cancer
Source: Gastric Cancer. 2023 May 30;26(5):653–66. doi: 10.1007/s10120-023-01395-0 (PMC10361908; doi:10.1007/s10120-023-01395-0)
Supplement: Supplementary file 7 — Supplementary file7 (PDF 909 KB) [file 10120_2023_1395_MOESM7_ESM.pdf]

Supplementary table 6. Differentially expressed genes in CDH1-TANGO6 del vs CDH1 WT

Transcript.stable.ID	baseMean	log2FoldChange	lfcSE	stat	pvalue	padj	Gene.stable.ID	chr	start	end
ENST00000002501	105.77206	-1.149474377	0.346965	-3.312939	0.00092321	0.007983848	ENSG00000003249	16	90004870	90019456
ENST00000003100	1729.9602	-1.825061602	0.178021	-10.25194	1.16E-24	8.46E-22	ENSG00000001630	7	92112152	92134477
ENST00000054950	1583.7758	-1.381613554	0.195493	-7.067342	1.58E-12	1.91E-10	ENSG00000049449	11	32091073	32105722
ENST00000064780	129.36703	-1.044391787	0.363729	-2.871347	0.004087266	0.025668113	ENSG00000054967	11	73376398	73397474
ENST00000075120	14070.248	-1.130014187	0.168758	-6.696077	2.14E-11	2.08E-09	ENSG00000059804	12	7919229	7936187
ENST00000078429	467.04908	-1.210840443	0.231388	-5.232937	1.67E-07	5.78E-06	ENSG00000088256	19	3094361	3123999
ENST00000164024	117.85741	1.619536437	0.338821	4.779911	1.75E-06	4.40E-05	ENSG00000008300	3	48636462	48662886
ENST00000190611	7.8454031	-6.445625875	1.776181	-3.628923	0.000284606	0.003153546	ENSG00000079156	2	178194511	178402893
ENST00000198536	18.480345	2.065593378	0.772988	2.672219	0.007535156	0.041096462	ENSG00000085514	7	100373486	100400096
ENST00000200181	5743.535	1.050142534	0.213325	4.922731	8.53E-07	2.36E-05	ENSG00000132470	17	75721458	75757818
ENST00000202017	234.40383	1.248012081	0.289636	4.308895	1.64E-05	0.000296215	ENSG00000088356	20	31944336	31952046
ENST00000204517	122.99618	-1.080237553	0.325662	-3.317051	0.000909731	0.007894608	ENSG00000090447	16	4257185	4273023
ENST00000205194	110.02396	-1.614393138	0.352208	-4.583634	4.57E-06	0.000100182	ENSG00000090971	19	55485187	55487566
ENST00000206765	146.4624	-1.536959091	0.314196	-4.891724	1.00E-06	2.69E-05	ENSG00000092295	14	24249113	24263177
ENST00000210633	4.6671419	5.645964836	1.970163	2.865735	0.004160419	0.026003428	ENSG00000095539	10	100969503	100985616
ENST00000214869	165.73412	-1.510431854	0.298089	-5.067042	4.04E-07	1.24E-05	ENSG00000099203	19	10832066	10836212
ENST00000215829	1929.1158	-1.192715939	0.227749	-5.236981	1.63E-07	5.68E-06	ENSG00000100028	22	24555998	24574971
ENST00000215909	988.6571	-1.296401425	0.192914	-6.720108	1.82E-11	1.79E-09	ENSG00000100097	22	37675635	37679802
ENST00000215912	36.117992	1.682728249	0.548925	3.065499	0.002173073	0.015665512	ENSG00000100100	22	31281599	31292488
ENST00000216014	76.50865	-1.323214582	0.43471	-3.043901	0.002335316	0.016581779	ENSG00000100196	22	38468095	38483447
ENST00000216027	200.62851	1.303749002	0.272868	4.777953	1.77E-06	4.43E-05	ENSG00000100209	22	28742038	28757510
ENST00000216085	272.5599	1.285193551	0.249805	5.144781	2.68E-07	8.70E-06	ENSG00000100263	22	29259871	29267981
ENST00000216124	101.38782	2.147807592	0.426075	5.040915	4.63E-07	1.40E-05	ENSG00000100299	22	50622753	50628152
ENST00000216133	176.26051	1.505282438	0.333954	4.507449	6.56E-06	0.00013635	ENSG00000100307	22	39130771	39152680
ENST00000216185	205.95137	1.325023189	0.281167	4.712582	2.45E-06	5.85E-05	ENSG00000100348	22	36467045	36481640
ENST00000216241	11.316236	3.33671911	1.112078	3.000436	0.002695932	0.01856011	ENSG00000100399	22	41229512	41240931
ENST00000216259	154.62874	1.369262553	0.302324	4.529126	5.92E-06	0.000124941	ENSG00000100417	22	41576899	41589840
ENST00000216271	195.02239	1.014098619	0.292415	3.468014	0.00052432	0.005117327	ENSG00000100429	22	50245182	50251265
ENST00000216367	244.45277	-1.733821074	0.278639	-6.22246	4.89E-10	3.43E-08	ENSG00000100479	14	49643554	49688214
ENST00000216392	48.821022	2.561230793	0.507248	5.049271	4.43E-07	1.34E-05	ENSG00000100504	14	50905216	50944483
ENST00000216446	102.53816	-1.912379111	0.452279	-4.228314	2.35E-05	0.000399076	ENSG00000100558	14	67386983	67412165

Gene.name	Gene.type	Canonical	MANE.Select	ensembl.version
DBNDD1	protein_coding	Yes	NM_001042610.3	108
CYP51A1	protein_coding	Yes	NM_000786.4	108
RCN1	protein_coding	Yes	NM_002901.4	108
RELT	protein_coding	Yes	NM_152222.2	108
SLC2A3	protein_coding	Yes	NM_006931.3	108
GNA11	protein_coding	Yes	NM_002067.5	108
CELSR3	protein_coding	Yes	NM_001407.3	108
OSBPL6	protein_coding	Yes	NM_032523.4	108
PILRA	protein_coding	Yes	NM_013439.3	108
ITGB4	protein_coding	Yes	NM_000213.5	108
PDRG1	protein_coding	Yes	NM_030815.3	108
TFAP4	protein_coding	Yes	NM_003223.3	108
NAT14	protein_coding	Yes	NM_020378.4	108
TGM1	protein_coding	Yes	NM_000359.3	108
SEMA4G	protein_coding	Yes	NM_017893.4	108
TMED1	protein_coding	Yes	NM_006858.4	108
SNRPD3	protein_coding	Yes	NM_004175.5	108
LGALS1	protein_coding	Yes	NM_002305.4	108
PIK3IP1	protein_coding	Yes	NM_052880.5	108
KDELR3	protein_coding	Yes	NM_006855.4	108
HSCB	protein_coding	Yes	NM_172002.5	108
RHBDD3	protein_coding	Yes	NM_012265.3	108
ARSA	protein_coding	Yes	NM_000487.6	108
CBX7	protein_coding	Yes	NM_175709.5	108
TXN2	protein_coding	Yes	NM_012473.4	108
CHADL	protein_coding	Yes	NM_138481.2	108
PMM1	protein_coding	Yes	NM_002676.3	108
HDAC10	protein_coding	Yes	NM_032019.6	108
POLE2	protein_coding	Yes	NM_002692.4	108
PYGL	protein_coding	Yes	NM_002863.5	108
PLEK2	protein_coding	Yes	NM_016445.3	108

ENST00000216714	1114.3448	1.932465858	0.202156	9.559272	1.19E-21	5.43E-19	ENSG00000100823	14	20455225	20457767
ENST00000216756	62.147159	-1.101347786	0.422784	-2.604987	0.009187784	0.047673689	ENSG00000100865	14	102348281	102362890
ENST00000216807	168.05222	-1.293062112	0.285666	-4.526478	6.00E-06	0.000126255	ENSG00000100916	14	35826337	35871963
ENST00000216862	412.57639	-2.470801527	0.2517	-9.816458	9.56E-23	5.26E-20	ENSG00000019186	20	54153445	54173986
ENST00000217073	347.31234	1.69814183	0.293575	5.784356	7.28E-09	3.79E-07	ENSG00000101104	20	44910059	44939316
ENST00000217182	124.68238	-4.045709512	0.455664	-8.878714	6.76E-19	2.23E-16	ENSG00000101210	20	63488013	63499083
ENST00000217289	1015.2339	-1.382091763	0.20091	-6.879163	6.02E-12	6.53E-10	ENSG00000101311	20	6074844	6123030
ENST00000218176	28.839181	3.071110982	0.724679	4.237893	2.26E-05	0.000384899	ENSG00000102057	X	48961379	48971844
ENST00000218516	476.07944	1.261719753	0.23721	5.319002	1.04E-07	3.86E-06	ENSG00000102393	X	101397802	101407925
ENST00000219091	176.50381	-1.392348742	0.301395	-4.61968	3.84E-06	8.64E-05	ENSG00000122386	16	3112585	3120517
ENST00000219150	69.549057	-3.575277646	0.482852	-7.404507	1.32E-13	1.97E-11	ENSG00000102879	16	30183601	30189076
ENST00000219479	280.98293	-1.341538502	0.249435	-5.378319	7.52E-08	2.92E-06	ENSG00000103202	16	397198	400754
ENST00000220166	344.96515	-1.473240041	0.23506	-6.267498	3.67E-10	2.69E-08	ENSG00000103811	15	78921057	78945046
ENST00000220584	821.49957	-1.117206407	0.201535	-5.543474	2.97E-08	1.28E-06	ENSG00000079459	8	11802740	11839298
ENST00000220597	307.93959	-2.653501379	0.254156	-10.44043	1.62E-25	1.26E-22	ENSG00000076641	8	80967809	81112068
ENST00000220616	22.624521	2.829894947	0.74251	3.811253	0.000138264	0.001748939	ENSG00000042832	8	132866957	133134899
ENST00000220809	108.69391	-3.118738849	0.413572	-7.540976	4.66E-14	7.54E-12	ENSG00000104368	8	42174717	42207565
ENST00000221138	371.80951	-1.612668583	0.231147	-6.976795	3.02E-12	3.45E-10	ENSG00000104695	8	30785615	30812818
ENST00000221200	454.02745	-1.06452913	0.232824	-4.57224	4.83E-06	0.000104898	ENSG00000104756	8	25427846	25458433
ENST00000221452	184.64831	2.323370456	0.306637	7.576946	3.54E-14	5.93E-12	ENSG00000104856	19	45001463	45038192
ENST00000221466	67.042735	-2.730733329	0.451216	-6.051941	1.43E-09	8.94E-08	ENSG00000104870	19	49512660	49526428
ENST00000221543	159.7023	1.352760897	0.300281	4.504976	6.64E-06	0.000137573	ENSG00000104946	19	49877701	49888750
ENST00000221856	34.402609	-2.585936638	0.601057	-4.302319	1.69E-05	0.000303995	ENSG00000105255	19	4304597	4323836
ENST00000221930	33.588741	-2.329906865	0.596007	-3.909197	9.26E-05	0.001249672	ENSG00000105329	19	41330322	41353922
ENST00000222120	350.92947	-1.241542859	0.253733	-4.893104	9.93E-07	2.68E-05	ENSG00000105514	19	11322067	11339657
ENST00000222139	181.09069	1.101574904	0.317617	3.468251	0.000523858	0.005115117	ENSG00000187266	19	11377206	11384314
ENST00000222374	173.88261	-1.921997134	0.306882	-6.262984	3.78E-10	2.75E-08	ENSG00000105767	19	43622367	43639850
ENST00000222543	46.236824	-2.322800067	0.584496	-3.974021	7.07E-05	0.001000164	ENSG00000105825	7	93885395	93890753
ENST00000222573	1817.1728	1.806603927	0.188389	9.589755	8.83E-22	4.14E-19	ENSG00000105855	7	20330899	20415754
ENST00000222693	975.29924	-1.843932763	0.211477	-8.719324	2.80E-18	8.55E-16	ENSG00000105971	7	116499737	116508541
ENST00000222747	13.299837	-3.011780551	0.978276	-3.078661	0.002079334	0.015143033	ENSG00000106025	7	120787319	120858144
ENST00000223129	882.17154	-1.057741355	0.194402	-5.440993	5.30E-08	2.16E-06	ENSG00000106399	7	7636517	7718607
ENST00000223210	157.50042	1.090917879	0.35469	3.075697	0.002100111	0.015255711	ENSG00000106479	7	149838374	149867479
ENST00000223215	458.83438	-2.532347986	0.242002	-10.46416	1.26E-25	1.00E-22	ENSG00000106484	7	130492084	130506465

APEX1	protein_coding	Yes	NM_001641.4	108
CINP	protein_coding	Yes	NM_032630.3	108
BRMS1L	protein_coding	Yes	NM_032352.4	108
CYP24A1	protein_coding	Yes	NM_000782.5	108
PABPC1L	protein_coding	Yes	NM_001372179.1	108
EEF1A2	protein_coding	Yes	NM_001958.5	108
FERMT1	protein_coding	Yes	NM_017671.5	108
KCND1	protein_coding	Yes	NM_004979.6	108
GLA	protein_coding	Yes	NM_000169.3	108
ZNF205	protein_coding	Yes	NM_001042428.2	108
CORO1A	protein_coding	Yes	NM_007074.4	108
NME4	protein_coding	Yes	NM_005009.3	108
CTSH	protein_coding	Yes	NM_004390.5	108
FDFT1	protein_coding	Yes	NM_004462.5	108
PAG1	protein_coding	Yes	NM_018440.4	108
TG	protein_coding	Yes	NM_003235.5	108
PLAT	protein_coding	Yes	NM_000930.5	108
PPP2CB	protein_coding	Yes	NM_001009552.2	108
KCTD9	protein_coding	Yes	NM_017634.4	108
RELB	protein_coding	Yes	NM_006509.4	108
FCGRT	protein_coding	Yes	NM_001136019.3	108
TBC1D17	protein_coding	Yes	NM_024682.3	108
FSD1	protein_coding	Yes	NM_024333.3	108
TGFB1	protein_coding	Yes	NM_000660.7	108
RAB3D	protein_coding	Yes	NM_004283.4	108
EPOR	protein_coding	Yes	NM_000121.4	108
CADM4	protein_coding	Yes	NM_145296.2	108
TFPI2	protein_coding	Yes	NM_006528.4	108
ITGB8	protein_coding	Yes	NM_002214.3	108
CAV2	protein_coding	Yes	NM_001233.5	108
TSPAN12	protein_coding	Yes	NM_012338.4	108
RPA3	protein_coding	Yes	NM_002947.5	108
ZNF862	protein_coding	Yes	NM_001099220.3	108
MEST	protein_coding	Yes	NM_002402.4	108

ENST00000223336	238.34985	-1.210504442	0.253767	-4.770147	1.84E-06	4.59E-05	ENSG00000106603	7	43639256	43729523
ENST00000224784	9.3431217	-3.075471452	1.17504	-2.617333	0.008861991	0.04637373	ENSG00000107796	10	88935073	88952773
ENST00000227135	122.48924	-1.344448811	0.331479	-4.055913	4.99E-05	0.000753066	ENSG00000064199	11	124673903	124697518
ENST00000227451	434.16639	-2.59839754	0.29227	-8.890396	6.09E-19	2.04E-16	ENSG00000110042	11	59172131	59208588
ENST00000227525	572.12917	-1.013459999	0.21471	-4.720131	2.36E-06	5.68E-05	ENSG00000110108	11	60914157	60923443
ENST00000228136	1701.3864	-1.02121388	0.189585	-5.386578	7.18E-08	2.81E-06	ENSG00000110696	11	16738646	16758340
ENST00000228510	365.87158	-1.231941727	0.24057	-5.120928	3.04E-07	9.69E-06	ENSG00000110921	12	109573793	109598125
ENST00000228534	55.205495	2.892996299	0.506416	5.712688	1.11E-08	5.44E-07	ENSG00000110944	12	56338883	56340410
ENST00000228916	275.29628	-3.220060987	0.306632	-10.5014	8.51E-26	7.13E-23	ENSG00000111319	12	6346846	6375563
ENST00000228945	310.00412	-1.347696032	0.239009	-5.638684	1.71E-08	7.84E-07	ENSG00000111348	12	14942030	14961601
ENST00000229266	116.02696	-2.236600453	0.340717	-6.564387	5.22E-11	4.70E-09	ENSG00000111666	12	101697639	101729074
ENST00000229277	128.58535	1.935233006	0.3297	5.869685	4.37E-09	2.39E-07	ENSG00000111674	12	6914579	6923697
ENST00000229281	797.28981	1.095887113	0.192182	5.702352	1.18E-08	5.74E-07	ENSG00000111678	12	6944021	6946002
ENST00000229758	453.39886	-1.580583617	0.230581	-6.854781	7.14E-12	7.62E-10	ENSG00000112029	6	152970534	152983041
ENST00000230792	302.55266	-1.352142838	0.305273	-4.429287	9.45E-06	0.000185048	ENSG00000112874	5	103548854	103562789
ENST00000231134	6.5773687	6.14230891	1.832599	3.351693	0.000803189	0.007154161	ENSG00000113209	5	141135205	141138615
ENST00000231198	177.03228	-1.185492539	0.278833	-4.251628	2.12E-05	0.000366224	ENSG00000113272	5	157731419	157741449
ENST00000232744	79.723971	1.900638781	0.437055	4.348744	1.37E-05	0.00025424	ENSG00000114626	3	127672951	127680926
ENST00000233072	15.167587	-4.907795196	1.256238	-3.906741	9.35E-05	0.001259759	ENSG00000021826	2	210556598	210679107
ENST00000233114	1719.5256	-1.00125818	0.177016	-5.656304	1.55E-08	7.16E-07	ENSG00000014641	2	63588962	63607197
ENST00000233143	4647.5673	-1.497282081	0.176397	-8.488125	2.10E-17	5.58E-15	ENSG00000034510	2	84905655	84906671
ENST00000233331	166.94973	1.016652675	0.311782	3.260785	0.001111041	0.009232586	ENSG00000115274	2	74455086	74457944
ENST00000233615	332.76996	1.263786193	0.234398	5.39163	6.98E-08	2.74E-06	ENSG00000239779	2	74458437	74460881
ENST00000234179	526.19803	-1.284829131	0.206582	-6.21947	4.99E-10	3.49E-08	ENSG00000115825	2	37250501	37324833
ENST00000235307	71.393676	-6.582320551	0.91703	-7.177867	7.08E-13	9.13E-11	ENSG00000116667	1	184387028	184629019
ENST00000236877	21.842704	3.081754569	0.836799	3.68279	0.000230696	0.002655831	ENSG00000118160	19	47428016	47471893
ENST00000237455	651.57591	1.287753136	0.228939	5.624866	1.86E-08	8.40E-07	ENSG00000239305	2	86603397	86623865
ENST00000237596	319.81518	-1.728716727	0.236468	-7.310561	2.66E-13	3.74E-11	ENSG00000118762	4	88007634	88077777
ENST00000237612	115.6435	-5.85783919	0.630478	-9.291114	1.53E-20	5.94E-18	ENSG00000118777	4	88090268	88158639
ENST00000237642	112.73101	-1.045814158	0.354714	-2.948333	0.003194924	0.021147463	ENSG00000118804	4	76306732	76311130
ENST00000237889	457.05912	-1.02308563	0.218684	-4.67837	2.89E-06	6.75E-05	ENSG00000119013	2	201072000	201085750
ENST00000239243	267.28825	-1.928861801	0.261651	-7.37189	1.68E-13	2.47E-11	ENSG00000120149	5	174724581	174730896
ENST00000239367	1765.6903	1.024495955	0.178007	5.755359	8.65E-09	4.39E-07	ENSG00000120256	6	149818756	149864359
ENST00000240100	212.62247	-1.317558351	0.339711	-3.878468	0.000105117	0.001387727	ENSG00000120875	8	29333063	29350684

COA1	protein_coding	Yes	NM_018224.4	108
ACTA2	protein_coding	Yes	NM_001613.4	108
SPA17	protein_coding	Yes	NM_017425.4	108
DTX4	protein_coding	Yes	NM_015177.2	108
TMEM109	protein_coding	Yes	NM_024092.3	108
C11orf58	protein_coding	Yes	NM_014267.6	108
MVK	protein_coding	Yes	NM_000431.4	108
IL23A	protein_coding	Yes	NM_016584.3	108
SCNN1A	protein_coding	Yes	NM_001038.6	108
ARHGDIB	protein_coding	Yes	NM_001175.7	108
CHPT1	protein_coding	Yes	NM_020244.3	108
ENO2	protein_coding	Yes	NM_001975.3	108
C12orf57	protein_coding	Yes	NM_138425.4	108
FBXO5	protein_coding	Yes	NM_012177.5	108
NUDT12	protein_coding	Yes	NM_031438.4	108
PCDHB5	protein_coding	Yes	NM_015669.5	108
THG1L	protein_coding	Yes	NM_017872.5	108
ABTB1	protein_coding	Yes	NM_172027.3	108
CPS1	protein_coding	Yes	NM_001875.5	108
MDH1	protein_coding	Yes	NM_005917.4	108
TMSB10	protein_coding	Yes	NM_021103.4	108
INO80B	protein_coding	Yes	NM_031288.4	108
WBP1	protein_coding	Yes	NM_012477.4	108
PRKD3	protein_coding	Yes	NM_005813.6	108
C1orf21	protein_coding	Yes	NM_030806.4	108
SLC8A2	protein_coding	Yes	NM_015063.3	108
RNF103	protein_coding	Yes	NM_005667.4	108
PKD2	protein_coding	Yes	NM_000297.4	108
ABCG2	protein_coding	Yes	NM_004827.3	108
STBD1	protein_coding	Yes	NM_003943.5	108
NDUFB3	protein_coding	Yes	NM_002491.3	108
MSX2	protein_coding	Yes	NM_002449.5	108
LRP11	protein_coding	Yes	NM_032832.6	108
DUSP4	protein_coding	Yes	NM_001394.7	108

ENST00000240651	249.90504	1.276400809	0.254487	5.015585	5.29E-07	1.56E-05	ENSG00000121350	12	21437654	21471250
ENST00000241125	52.187211	1.589970832	0.528718	3.007222	0.002636475	0.018254113	ENSG00000121743	13	20138254	20161052
ENST00000241261	44.074096	2.010792058	0.671855	2.992895	0.002763448	0.01892093	ENSG00000121858	3	172505507	172523430
ENST00000241337	4.6423127	5.639148354	1.971416	2.860456	0.004230322	0.026324826	ENSG00000213366	1	109668056	109675286
ENST00000242067	172.27422	1.047328567	0.351027	2.983615	0.002848654	0.019360813	ENSG00000122507	7	33129563	33606069
ENST00000242317	28.037757	1.909901998	0.6811	2.804144	0.005045035	0.030206986	ENSG00000122735	9	34458804	34520984
ENST00000242351	2111.7505	1.105600837	0.199731	5.535447	3.10E-08	1.33E-06	ENSG00000105939	7	139043514	139109720
ENST00000242480	7.6814572	6.363442284	1.791188	3.552638	0.000381389	0.003972922	ENSG00000122877	10	62811995	62816366
ENST00000242810	1585.9308	1.200797799	0.256467	4.682077	2.84E-06	6.65E-05	ENSG00000114796	3	183635622	183684519
ENST00000242827	193.33828	-1.028360348	0.270515	-3.801498	0.000143824	0.001806522	ENSG00000123179	13	49660673	49691486
ENST00000243189	651.52288	1.221865529	0.205272	5.952432	2.64E-09	1.53E-07	ENSG00000117616	1	25242248	25247454
ENST00000243349	165.50752	-1.175493948	0.361769	-3.249298	0.001156901	0.009544462	ENSG00000123612	2	157526766	157628864
ENST00000243643	8.8194833	6.564562155	1.742507	3.76731	0.000165016	0.002020754	ENSG00000170954	19	53107878	53132910
ENST00000243673	16.410256	2.455065705	0.83304	2.947115	0.003207543	0.021219012	ENSG00000123901	11	94377315	94401419
ENST00000244519	134.50433	2.52709365	0.368732	6.853474	7.21E-12	7.68E-10	ENSG00000111801	6	26440503	26453415
ENST00000244534	1970.1757	-1.930086667	0.205925	-9.372766	7.07E-21	2.85E-18	ENSG00000124575	6	26234211	26234987
ENST00000244565	38.148414	2.466152534	0.568576	4.337418	1.44E-05	0.000265464	ENSG00000124602	6	41026894	41039221
ENST00000244571	374.63099	-1.088377468	0.265707	-4.096156	4.20E-05	0.000651764	ENSG00000124608	6	44298730	44313347
ENST00000245105	95.271383	-2.834458917	0.390931	-7.250535	4.15E-13	5.60E-11	ENSG00000125089	4	8199289	8241103
ENST00000245206	836.38993	-1.024257709	0.194268	-5.272397	1.35E-07	4.80E-06	ENSG00000125166	16	58707130	58734316
ENST00000245222	133.52095	1.756718937	0.326976	5.372626	7.76E-08	3.00E-06	ENSG00000063176	19	48619505	48630405
ENST00000245907	17.767577	-2.334178754	0.82136	-2.841846	0.004485321	0.027527274	ENSG00000125730	19	6677703	6720650
ENST00000246802	1308.7939	1.609519837	0.182333	8.827357	1.07E-18	3.43E-16	ENSG00000105373	19	47745545	47757058
ENST00000246914	25.570909	1.926977576	0.674338	2.857586	0.004268767	0.026494827	ENSG00000126562	17	42780609	42797066
ENST00000246957	1169.8264	-1.059844871	0.1966	-5.390862	7.01E-08	2.75E-06	ENSG00000126602	16	3658036	3717524
ENST00000247026	151.77412	-1.19524793	0.30587	-3.907703	9.32E-05	0.001256131	ENSG00000126653	17	30116815	30186475
ENST00000247191	2348.2118	-1.558744127	0.191437	-8.142347	3.88E-16	8.89E-14	ENSG00000126787	14	55148134	55191585
ENST00000247225	1299.7282	-1.192279449	0.236774	-5.035511	4.77E-07	1.43E-05	ENSG00000126821	14	63684215	63728065
ENST00000247291	366.56387	-1.978443696	0.234219	-8.446992	2.99E-17	7.80E-15	ENSG00000126878	9	131096530	131123144
ENST00000247470	65.967566	-1.349582999	0.410234	-3.289785	0.00100264	0.008500017	ENSG00000103490	16	31201485	31202760
ENST00000248248	508.12946	1.245996335	0.24763	5.031683	4.86E-07	1.45E-05	ENSG00000103111	16	77191189	77202398
ENST00000248306	92.021938	1.187162267	0.35752	3.320548	0.000898408	0.007817712	ENSG00000127720	12	82358528	82479239
ENST00000249071	30.002137	-2.105948001	0.611805	-3.442187	0.000577031	0.005512025	ENSG00000128340	22	37225269	37244269
ENST00000249700	118.05421	-2.171807787	0.406766	-5.339204	9.34E-08	3.52E-06	ENSG00000128872	15	51751596	51816363

PYROXD1	protein_coding	Yes	NM_024854.5	108
GJA3	protein_coding	Yes	NM_021954.4	108
TNFSF10	protein_coding	Yes	NM_003810.4	108
GSTM2	protein_coding	Yes	NM_000848.4	108
BBS9	protein_coding	Yes	NM_198428.3	108
DNAI1	protein_coding	Yes	NM_012144.4	108
ZC3HAV1	protein_coding	Yes	NM_020119.4	108
EGR2	protein_coding	Yes	NM_000399.5	108
KLHL24	protein_coding	Yes	NM_017644.3	108
EBPL	protein_coding	Yes	NM_032565.5	108
RSRP1	protein_coding	Yes	NM_020317.5	108
ACVR1C	protein_coding	Yes	NM_145259.3	108
ZNF415	protein_coding	Yes	NM_018355.4	108
GPR83	protein_coding	Yes	NM_016540.4	108
BTN3A3	protein_coding	Yes	NM_006994.5	108
H1-3	protein_coding	Yes	NM_005320.3	108
UNC5CL	protein_coding	Yes	NM_173561.3	108
AARS2	protein_coding	Yes	NM_020745.4	108
SH3TC1	protein_coding	Yes	NM_018986.5	108
GOT2	protein_coding	Yes	NM_002080.4	108
SPHK2	protein_coding	Yes	NM_020126.5	108
C3	protein_coding	Yes	NM_000064.4	108
NOP53	protein_coding	Yes	NM_015710.5	108
WNK4	protein_coding	Yes	NM_032387.5	108
TRAP1	protein_coding	Yes	NM_016292.3	108
NSRP1	protein_coding	Yes	NM_032141.4	108
DLGAP5	protein_coding	Yes	NM_014750.5	108
SGPP1	protein_coding	Yes	NM_030791.4	108
AIF1L	protein_coding	Yes	NM_031426.4	108
PYCARD	protein_coding	Yes	NM_013258.5	108
MON1B	protein_coding	Yes	NM_014940.4	108
METTL25	protein_coding	Yes	NM_032230.3	108
RAC2	protein_coding	Yes	NM_002872.5	108
TMOD2	protein_coding	Yes	NM_014548.4	108

ENST00000250024	257.28404	-1.524978954	0.251671	-6.059417	1.37E-09	8.59E-08	ENSG00000129173	11	19224062	19240945
ENST00000250263	750.20037	-1.256238563	0.213154	-5.89356	3.78E-09	2.10E-07	ENSG00000104626	8	9002896	9033338
ENST00000251170	38.344459	1.566730215	0.54433	2.878273	0.003998591	0.025237254	ENSG00000103184	16	4958329	5019157
ENST00000251363	164.52209	-1.211184026	0.384601	-3.1492	0.001637182	0.012538769	ENSG00000090661	19	8209369	8262421
ENST00000251453	1525.513	4.259370758	0.207627	20.51452	1.60E-93	1.22E-88	ENSG00000105193	19	39433136	39435949
ENST00000251496	2244.1946	-1.180977392	0.189077	-6.246015	4.21E-10	3.01E-08	ENSG00000109805	4	17810978	17844865
ENST00000251642	141.82979	2.143253205	0.331527	6.464801	1.01E-10	8.61E-09	ENSG00000108771	17	42101410	42112714
ENST00000251822	94.627559	-1.70779192	0.360693	-4.734753	2.19E-06	5.35E-05	ENSG00000008853	8	22999547	23020199
ENST00000251900	74.306344	-1.848936046	0.406186	-4.551944	5.32E-06	0.00011413	ENSG00000102098	X	18239312	18354688
ENST00000251973	259.37811	-1.633051388	0.270454	-6.038176	1.56E-09	9.57E-08	ENSG00000100065	22	37490361	37519415
ENST00000252029	108.89937	1.311744843	0.352266	3.72373	0.000196301	0.002332456	ENSG00000025708	22	50525751	50530012
ENST00000252087	56.59393	1.163081068	0.435373	2.67146	0.007552205	0.041167348	ENSG00000240764	5	141489080	141512975
ENST00000252288	14.660524	-2.482295448	0.889556	-2.790487	0.005262883	0.031185462	ENSG00000130005	19	1397025	1401542
ENST00000252486	288.07078	-1.381996668	0.35831	-3.856989	0.000114792	0.001494715	ENSG00000130203	19	44905795	44909393
ENST00000252505	7.5821404	6.346741434	1.785802	3.554001	0.000379418	0.003956724	ENSG00000151360	2	3658199	3702671
ENST00000252506	51.63636	1.384534866	0.502541	2.755067	0.005868018	0.03392703	ENSG00000130222	9	89605011	89606555
ENST00000252590	8.8194833	6.564562155	1.742507	3.76731	0.000165016	0.002020754	ENSG00000130300	19	17351454	17377342
ENST00000252729	174.25157	-1.018989242	0.32124	-3.172054	0.001513648	0.011779852	ENSG00000130433	19	53991636	54012666
ENST00000252809	210.35	-2.097559413	0.272213	-7.705593	1.30E-14	2.40E-12	ENSG00000130513	19	18386157	18389176
ENST00000252945	25.384625	2.097347683	0.658015	3.187385	0.001435654	0.011308348	ENSG00000130649	10	133527362	133539123
ENST00000253079	57.44815	2.354743281	0.468105	5.030376	4.90E-07	1.46E-05	ENSG00000130783	12	122774571	122827528
ENST00000253193	254.19151	-1.015719744	0.258084	-3.935615	8.30E-05	0.001142856	ENSG00000130881	19	33194329	33208864
ENST00000253255	7.6905655	6.37002583	1.795323	3.548122	0.000387989	0.004030107	ENSG00000130943	22	46255662	46263343
ENST00000253462	324.27603	-1.257164896	0.270297	-4.65105	3.30E-06	7.56E-05	ENSG00000131153	16	85676197	85688954
ENST00000253697	27.247448	2.858028159	0.686586	4.162668	3.15E-05	0.000509903	ENSG00000131379	3	14675140	14773036
ENST00000253799	84.406059	1.882982622	0.382538	4.922336	8.55E-07	2.37E-05	ENSG00000131480	17	42844579	42850707
ENST00000253807	579.23018	1.261869225	0.224921	5.610288	2.02E-08	9.06E-07	ENSG00000248383	5	140926298	141012347
ENST00000253812	56.125812	1.205492415	0.43825	2.750693	0.005946927	0.034246848	ENSG00000254245	5	141343828	141512975
ENST00000254043	191.10264	2.275501494	0.371506	6.125073	9.06E-10	5.99E-08	ENSG00000171346	17	41513744	41518890
ENST00000254190	264.52236	-2.1461301	0.263813	-8.135032	4.12E-16	9.40E-14	ENSG00000131873	15	101175726	101252048
ENST00000254436	287.138	1.145481279	0.254877	4.494244	6.98E-06	0.000143506	ENSG00000132109	11	4384896	4393702
ENST00000254579	153.69582	1.582015262	0.33592	4.709494	2.48E-06	5.93E-05	ENSG00000179532	11	6497279	6572020
ENST00000254695	173.1415	-1.493537524	0.288893	-5.169856	2.34E-07	7.73E-06	ENSG00000132359	17	2796437	3037741
ENST00000254759	85.691154	-1.119067767	0.383573	-2.917485	0.003528668	0.022854319	ENSG00000132423	6	99369400	99394195

E2F8	protein_coding	Yes	NM_024680.4	108
ERI1	protein_coding	Yes	NM_153332.4	108
SEC14L5	protein_coding	Yes	NM_014692.2	108
CERS4	protein_coding	Yes	NM_024552.3	108
RPS16	protein_coding	Yes	NM_001020.6	108
NCAPG	protein_coding	Yes	NM_022346.5	108
DHX58	protein_coding	Yes	NM_024119.3	108
RHOBTB2	protein_coding	Yes	NM_015178.3	108
SCML2	protein_coding	Yes	NM_006089.3	108
CARD10	protein_coding	Yes	NM_014550.4	108
TYMP	protein_coding	Yes	NM_001953.5	108
PCDHGC5	protein_coding	Yes	NM_018929.3	108
GAMT	protein_coding	Yes	NM_000156.6	108
APOE	protein_coding	Yes	NM_000041.4	108
ALLC	protein_coding	Yes	NM_018436.4	108
GADD45G	protein_coding	Yes	NM_006705.4	108
PLVAP	protein_coding	Yes	NM_031310.3	108
CACNG6	protein_coding	Yes	NM_145814.2	108
GDF15	protein_coding	Yes	NM_004864.4	108
CYP2E1	protein_coding	Yes	NM_000773.4	108
CCDC62	protein_coding	Yes	NM_201435.5	108
LRP3	protein_coding	Yes	NM_002333.4	108
PKDREJ	protein_coding	Yes	NM_006071.2	108
GINS2	protein_coding	Yes	NM_016095.3	108
C3orf20	protein_coding	Yes	NM_032137.5	108
AOC2	protein_coding	Yes	NM_009590.4	108
PCDHAC1	protein_coding	Yes	NM_018898.5	108
PCDHGA3	protein_coding	Yes	NM_018916.4	108
KRT15	protein_coding	Yes	NM_002275.4	108
CHSY1	protein_coding	Yes	NM_014918.5	108
TRIM21	protein_coding	Yes	NM_003141.4	108
DNHD1	protein_coding	Yes	NM_144666.3	108
RAP1GAP2	protein_coding	Yes	NM_015085.5	108
COQ3	protein_coding	Yes	NM_017421.4	108

ENST00000254806	445.67025	1.255962284	0.246218	5.101014	3.38E-07	1.06E-05	ENSG00000132471	17	75845698	75855335
ENST00000254816	336.96722	1.258524135	0.255481	4.926101	8.39E-07	2.33E-05	ENSG00000132481	17	75874163	75878581
ENST00000255320	1579.6515	-1.266067915	0.218375	-5.797669	6.72E-09	3.53E-07	ENSG00000132967	3	22381818	22382460
ENST00000255389	79.822394	-1.132507725	0.385259	-2.939604	0.003286321	0.021631246	ENSG00000133027	17	17505562	17591708
ENST00000255681	99.409279	-1.050798577	0.346019	-3.036825	0.002390844	0.016888716	ENSG00000133315	11	63998557	64166113
ENST00000255764	119.69363	1.070592881	0.328993	3.254152	0.001137314	0.009413958	ENSG00000133398	5	6371873	6378547
ENST00000256010	116.98258	-4.520567702	0.446699	-10.11994	4.51E-24	3.04E-21	ENSG00000133636	12	85874294	85882992
ENST00000256458	95.08707	1.245906872	0.362408	3.437859	0.000586333	0.005582081	ENSG00000134070	3	10164918	10243745
ENST00000256538	99.903373	-1.448715524	0.358948	-4.036001	5.44E-05	0.000808132	ENSG00000134146	15	35370879	35546165
ENST00000256722	169.80756	1.501954116	0.403385	3.723379	0.000196574	0.002335339	ENSG00000134326	2	6848315	6865907
ENST00000256733	6.1122266	6.035898091	1.858312	3.248054	0.001161974	0.009572323	ENSG00000134339	11	18245239	18248668
ENST00000256951	279.28477	-2.918685428	0.27114	-10.76449	5.06E-27	4.74E-24	ENSG00000134531	12	13196725	13219941
ENST00000257497	4968.4129	-2.554963082	0.206381	-12.37984	3.36E-35	5.51E-32	ENSG00000135046	9	73151864	73170393
ENST00000257637	90.517095	-1.211424435	0.370374	-3.270813	0.001072388	0.008968955	ENSG00000135185	7	87196159	87219732
ENST00000257663	128.50107	-1.228946156	0.329333	-3.731624	0.000190249	0.002272244	ENSG00000135211	7	77793727	77798434
ENST00000257770	27.447591	-5.182137549	1.059116	-4.892891	9.94E-07	2.68E-05	ENSG00000135318	6	85450082	85495784
ENST00000257776	5.8796555	5.979596508	1.873074	3.192397	0.001410971	0.011152758	ENSG00000135324	6	84033771	84090881
ENST00000257868	107.1881	-1.537671581	0.341234	-4.506213	6.60E-06	0.00013696	ENSG00000135414	12	55743121	55757264
ENST00000257940	160.57093	1.127228696	0.289264	3.89688	9.74E-05	0.001303054	ENSG00000135482	12	56118265	56127514
ENST00000258091	3184.5851	-1.224347777	0.209366	-5.847892	4.98E-09	2.69E-07	ENSG00000135624	2	73234307	73253005
ENST00000258341	3813.3157	-1.356018073	0.16895	-8.026155	1.01E-15	2.15E-13	ENSG00000135862	1	183023419	183145592
ENST00000258443	266.38443	3.725568065	0.855945	4.352577	1.35E-05	0.000250413	ENSG00000135960	2	108894470	108989220
ENST00000258607	1487.8391	-1.085645802	0.176181	-6.162111	7.18E-10	4.86E-08	ENSG00000136108	13	52455477	52476627
ENST00000258662	790.80581	-1.26399483	0.228982	-5.520064	3.39E-08	1.44E-06	ENSG00000136159	13	48037725	48047221
ENST00000258733	232.01352	2.026421789	0.320138	6.329828	2.45E-10	1.91E-08	ENSG00000136235	7	23246774	23275108
ENST00000258821	262.49392	-1.578704928	0.256603	-6.152326	7.64E-10	5.12E-08	ENSG00000136319	14	20286226	20305951
ENST00000258888	72.608056	1.768700173	0.403527	4.383104	1.17E-05	0.000222152	ENSG00000136383	15	84817355	84873479
ENST00000258975	143.61918	-1.146089067	0.299275	-3.829557	0.000128374	0.001641686	ENSG00000136463	17	63600894	63608365
ENST00000259241	406.94333	-1.246086672	0.228071	-5.463586	4.67E-08	1.93E-06	ENSG00000136720	2	128265479	128318868
ENST00000259392	77.173297	-1.057108217	0.395623	-2.672011	0.00753982	0.04111721	ENSG00000136867	9	113151006	113164140
ENST00000259667	195.00479	-1.414748224	0.288031	-4.911787	9.03E-07	2.48E-05	ENSG00000137133	9	35812959	35815042
ENST00000259881	17.095632	3.094318171	0.91599	3.378114	0.000729848	0.006637046	ENSG00000204540	6	31114799	31140092
ENST00000259951	150.40056	2.288230165	0.393259	5.818636	5.93E-09	3.14E-07	ENSG00000204642	6	29723433	29727296
ENST00000260197	939.69944	-1.120538354	0.202436	-5.535264	3.11E-08	1.33E-06	ENSG00000137642	11	121452313	121633763

WBP2	protein_coding	Yes	NM_012478.4	108
TRIM47	protein_coding	Yes	NM_033452.3	108
HMGB1P5	d_processed_ps	Yes	-	108
PEMT	protein_coding	Yes	NM_148172.3	108
MACROD1	protein_coding	Yes	NM_014067.4	108
MED10	protein_coding	Yes	NM_032286.3	108
NTS	protein_coding	Yes	NM_006183.5	108
IRAK2	protein_coding	Yes	NM_001570.4	108
DPH6	protein_coding	Yes	NM_080650.4	108
CMPK2	protein_coding	Yes	NM_207315.4	108
SAA2	protein_coding	Yes	NM_030754.5	108
EMP1	protein_coding	Yes	NM_001423.3	108
ANXA1	protein_coding	Yes	NM_000700.3	108
TMEM243	protein_coding	Yes	NM_024315.4	108
TMEM60	protein_coding	Yes	NM_032936.4	108
NT5E	protein_coding	Yes	NM_002526.4	108
MRAP2	protein_coding	Yes	NM_138409.4	108
GDF11	protein_coding	Yes	NM_005811.5	108
ZC3H10	protein_coding	Yes	NM_032786.3	108
CCT7	protein_coding	Yes	NM_006429.4	108
LAMC1	protein_coding	Yes	NM_002293.4	108
EDAR	protein_coding	Yes	NM_022336.4	108
CKAP2	protein_coding	Yes	NM_018204.5	108
NUDT15	protein_coding	Yes	NM_018283.4	108
GPNMB	protein_coding	Yes	NM_002510.3	108
TTC5	protein_coding	Yes	NM_138376.3	108
ALPK3	protein_coding	Yes	NM_020778.5	108
TACO1	protein_coding	Yes	NM_016360.4	108
HS6ST1	protein_coding	Yes	NM_004807.3	108
SLC31A2	protein_coding	Yes	NM_001860.3	108
HINT2	protein_coding	Yes	NM_032593.3	108
PSORS1C1	protein_coding	Yes	NM_014068.3	108
HLA-F	protein_coding	Yes	NM_001098479.2	108
SORL1	protein_coding	Yes	NM_003105.6	108

ENST00000260228	11.53788	3.015965305	1.048123	2.877493	0.004008488	0.025289246	ENSG00000137674	11	102576831	102625332
ENST00000260324	449.19117	1.449333981	0.289642	5.003884	5.62E-07	1.65E-05	ENSG00000137767	15	45635028	45691281
ENST00000260379	2979.1566	1.089859515	0.185465	5.876367	4.19E-09	2.31E-07	ENSG00000137818	15	69452817	69456205
ENST00000260442	3.8949411	5.386514595	2.058112	2.617211	0.008865141	0.04637373	ENSG00000137875	15	52109262	52112775
ENST00000260682	14.076127	3.045625658	0.957717	3.180091	0.001472289	0.01153158	ENSG00000138109	10	94938657	94990091
ENST00000260731	2537.5445	-1.327315241	0.190877	-6.953775	3.56E-12	4.02E-10	ENSG00000138160	10	92593129	92655395
ENST00000260843	109.60538	-3.062084836	0.375525	-8.154148	3.52E-16	8.08E-14	ENSG00000138271	3	151294085	151316820
ENST00000260947	880.04629	-1.103429714	0.189252	-5.830489	5.53E-09	2.95E-07	ENSG00000138376	2	214725645	214809683
ENST00000261024	60.288881	-3.756728385	0.526124	-7.140382	9.31E-13	1.17E-10	ENSG00000138449	2	189560589	189580786
ENST00000261245	637.22437	-1.332372306	0.234496	-5.681866	1.33E-08	6.31E-07	ENSG00000020426	14	60734760	60969965
ENST00000261254	20.931292	-3.31021824	0.874879	-3.783629	0.000154558	0.001916242	ENSG00000118971	12	4273761	4305353
ENST00000261292	91.494604	-1.324978415	0.484129	-2.736827	0.006203494	0.03543117	ENSG00000101670	18	49562056	49599185
ENST00000261326	110.40079	1.424924564	0.342597	4.159184	3.19E-05	0.000516806	ENSG00000075643	18	36187496	36272157
ENST00000261353	1534.8759	1.234961866	0.214609	5.754469	8.69E-09	4.41E-07	ENSG00000119801	2	30147006	30160533
ENST00000261366	1943.0898	-1.677789142	0.201223	-8.337969	7.56E-17	1.89E-14	ENSG00000113368	5	126777135	126837020
ENST00000261402	241.98796	1.152339017	0.253159	4.551847	5.32E-06	0.000114151	ENSG00000074590	12	106063344	106138954
ENST00000261448	10.095937	-2.849600518	1.091488	-2.610748	0.009034436	0.047044567	ENSG00000118729	1	115700020	115768714
ENST00000261507	938.8893	-2.138459143	0.19627	-10.89548	1.21E-27	1.21E-24	ENSG00000052802	4	165327668	165343164
ENST00000261593	456.66581	-1.068011926	0.216206	-4.93979	7.82E-07	2.19E-05	ENSG00000134758	18	32091873	32131561
ENST00000261758	831.80699	-1.014766454	0.236702	-4.287105	1.81E-05	0.000321849	ENSG00000117899	15	80975751	80989819
ENST00000261769	4484.2745	-3.416309175	0.190763	-17.90869	1.01E-71	1.10E-67	ENSG00000039068	16	68737291	68835537
ENST00000261783	351.81148	2.035624328	0.266723	7.631993	2.31E-14	4.05E-12	ENSG00000081181	14	67619919	67651708
ENST00000261862	157.09564	-1.598912535	0.329884	-4.846895	1.25E-06	3.28E-05	ENSG00000187720	15	71115470	71783383
ENST00000261867	87.792325	-1.93277562	0.409186	-4.72346	2.32E-06	5.61E-05	ENSG00000104154	15	45479605	45522755
ENST00000261884	298.90932	1.229415726	0.237048	5.186353	2.14E-07	7.17E-06	ENSG00000103671	15	64387835	64455303
ENST00000261908	240.54749	-2.938697565	0.318446	-9.228239	2.75E-20	1.04E-17	ENSG00000067141	15	73052462	73305205
ENST00000261965	289.30765	-1.604040679	0.25897	-6.193928	5.87E-10	4.05E-08	ENSG00000126216	13	112485010	112588153
ENST00000262055	996.21073	1.472167812	0.236182	6.233201	4.57E-10	3.23E-08	ENSG00000050426	12	51048326	51060424
ENST00000262094	211.57687	-1.653608896	0.335772	-4.924795	8.44E-07	2.34E-05	ENSG00000041353	18	54828476	54895516
ENST00000262096	358.87415	-3.547481623	0.314559	-11.27764	1.69E-29	1.92E-26	ENSG00000104219	8	17156481	17224799
ENST00000262134	864.49045	-1.019298171	0.211625	-4.816538	1.46E-06	3.75E-05	ENSG00000087253	16	55509071	55586666
ENST00000262139	87.435318	1.297900728	0.422033	3.075355	0.002102522	0.01526813	ENSG00000070540	17	68421280	68457496
ENST00000262213	3197.4002	-1.054582665	0.213076	-4.949321	7.45E-07	2.10E-05	ENSG00000067167	8	70573217	70608416
ENST00000262233	100.15219	-1.920616897	0.388335	-4.94578	7.58E-07	2.13E-05	ENSG00000066629	14	99793412	99942060

MMP20	protein_coding	Yes	NM_004771.4	108
SQOR	protein_coding	Yes	NM_021199.4	108
RPLP1	protein_coding	Yes	NM_001003.3	108
BCL2L10	protein_coding	Yes	NM_020396.4	108
CYP2C9	protein_coding	Yes	NM_000771.4	108
KIF11	protein_coding	Yes	NM_004523.4	108
GPR87	protein_coding	Yes	NM_023915.4	108
BARD1	protein_coding	Yes	NM_000465.4	108
SLC40A1	protein_coding	Yes	NM_014585.6	108
MNAT1	protein_coding	Yes	NM_002431.4	108
CCND2	protein_coding	Yes	NM_001759.4	108
LIPG	protein_coding	Yes	NM_006033.4	108
MOCOS	protein_coding	Yes	NM_017947.4	108
YPEL5	protein_coding	Yes	NM_016061.3	108
LMNB1	protein_coding	Yes	NM_005573.4	108
NUAK1	protein_coding	Yes	NM_014840.3	108
CASQ2	protein_coding	Yes	NM_001232.4	108
MSMO1	protein_coding	Yes	NM_006745.5	108
RNF138	protein_coding	Yes	NM_016271.5	108
MESD	protein_coding	Yes	NM_015154.3	108
CDH1	protein_coding	Yes	NM_004360.5	108
ARG2	protein_coding	Yes	NM_001172.4	108
THSD4	protein_coding	Yes	NM_024817.3	108
SLC30A4	protein_coding	Yes	NM_013309.6	108
TRIP4	protein_coding	Yes	NM_016213.5	108
NEO1	protein_coding	Yes	NM_002499.4	108
TUBGCP3	protein_coding	Yes	NM_006322.6	108
LETMD1	protein_coding	Yes	NM_015416.5	108
RAB27B	protein_coding	Yes	NM_004163.4	108
ZDHHC2	protein_coding	Yes	NM_016353.5	108
LPCAT2	protein_coding	Yes	NM_017839.5	108
WIPI1	protein_coding	Yes	NM_017983.7	108
TRAM1	protein_coding	Yes	NM_014294.6	108
EML1	protein_coding	Yes	NM_004434.3	108

ENST00000262244	116.66656	-1.062085819	0.328585	-3.232304	0.001227965	0.009998683	ENSG00000120162	9	27325208	27529814
ENST00000262302	279.81702	-1.05268411	0.274234	-3.838628	0.000123724	0.001593981	ENSG00000095906	16	1782959	1789186
ENST00000262315	360.06641	-1.111927409	0.262236	-4.240184	2.23E-05	0.000382203	ENSG00000127586	16	788619	798074
ENST00000262428	738.27724	-1.682277246	0.242932	-6.924903	4.36E-12	4.82E-10	ENSG00000103187	16	84565595	84618078
ENST00000262429	389.4691	-1.917608785	0.25485	-7.524457	5.29E-14	8.50E-12	ENSG00000064270	16	84368537	84464187
ENST00000262450	36.77668	1.548908581	0.544302	2.84568	0.004431676	0.027268594	ENSG00000116254	1	6101786	6180321
ENST00000262460	1142.7011	-1.559116473	0.2036	-7.657745	1.89E-14	3.40E-12	ENSG00000101003	20	25407672	25448563
ENST00000262483	76.833975	1.540935279	0.436283	3.531962	0.000412488	0.004234376	ENSG00000091622	17	6451262	6556555
ENST00000262525	311.31899	-1.847659246	0.241298	-7.657183	1.90E-14	3.40E-12	ENSG00000102870	16	30778455	30787205
ENST00000262545	98.493152	2.161231946	0.364091	5.935971	2.92E-09	1.67E-07	ENSG00000125851	20	17227039	17484578
ENST00000262580	349.27253	-1.758920648	0.230961	-7.615667	2.62E-14	4.54E-12	ENSG00000104518	8	143558345	143563062
ENST00000262776	4244.6175	2.28562797	0.233253	9.798905	1.14E-22	6.13E-20	ENSG00000108679	17	78971254	78979923
ENST00000262844	421.64076	-1.103585593	0.227367	-4.853771	1.21E-06	3.18E-05	ENSG00000101935	X	110194185	110318085
ENST00000263038	317.4696	1.932238727	0.27961	6.910486	4.83E-12	5.30E-10	ENSG00000107537	10	13277798	13300064
ENST00000263071	14.074308	2.800849486	0.939686	2.980622	0.002876634	0.019504879	ENSG00000074660	17	1633857	1645732
ENST00000263125	63.099757	-1.661492991	0.429528	-3.868187	0.000109648	0.001438537	ENSG00000065675	10	6427147	6580276
ENST00000263228	928.98939	-1.120419508	0.187113	-5.987928	2.13E-09	1.26E-07	ENSG00000107341	9	33817159	33920399
ENST00000263233	62.028374	4.154964501	0.570861	7.278418	3.38E-13	4.62E-11	ENSG00000102003	X	49187814	49200193
ENST00000263268	293.376	-1.153343347	0.258242	-4.466134	7.96E-06	0.000160207	ENSG00000118242	2	215942583	216013551
ENST00000263270	747.52504	-1.021756857	0.203579	-5.01897	5.19E-07	1.54E-05	ENSG00000042753	19	46838166	46850846
ENST00000263368	972.55435	1.018841152	0.221225	4.605448	4.12E-06	9.15E-05	ENSG00000090013	19	40447767	40465745
ENST00000263464	856.05275	2.093036136	0.192542	10.87054	1.59E-27	1.56E-24	ENSG00000023445	11	102317483	102339403
ENST00000263576	12.597754	2.598413806	0.97445	2.666545	0.007663537	0.041644645	ENSG00000109832	11	125904507	125928843
ENST00000263650	123.83054	-1.31924195	0.355103	-3.715093	0.000203129	0.002394179	ENSG00000021762	11	3087106	3165310
ENST00000263681	617.11663	-1.239289462	0.209159	-5.925097	3.12E-09	1.77E-07	ENSG00000077514	11	74592581	74643076
ENST00000263726	108.92656	-1.250021308	0.332787	-3.756215	0.000172503	0.002097097	ENSG00000121454	1	180230263	180278984
ENST00000263736	404.91549	-1.029665418	0.219568	-4.689508	2.74E-06	6.45E-05	ENSG00000068784	2	45388679	45611267
ENST00000263864	603.3464	1.02164326	0.200506	5.095329	3.48E-07	1.09E-05	ENSG00000118640	2	85577585	85582031
ENST00000263932	20.700392	2.837501858	0.779076	3.642138	0.000270384	0.003024545	ENSG00000120949	1	12063302	12144207
ENST00000263955	1442.5387	-1.526358738	0.191722	-7.961325	1.70E-15	3.55E-13	ENSG00000081320	2	196133582	196171578
ENST00000264021	167.1773	-1.880770925	0.355197	-5.295006	1.19E-07	4.32E-06	ENSG00000118096	11	118544542	118565931
ENST00000264039	1188.2009	-1.111536849	0.181821	-6.113368	9.76E-10	6.40E-08	ENSG00000063660	2	240435662	240468076
ENST00000264051	10.323532	-5.372408003	1.682623	-3.192878	0.001408627	0.011138272	ENSG00000066248	2	232878700	233013256
ENST00000264059	64.883626	-1.391216129	0.420744	-3.306562	0.000944483	0.008126777	ENSG00000115468	2	232633603	232682776

MOB3B	protein_coding	Yes	NM_024761.5	108
NUBP2	protein_coding	Yes	NM_012225.4	108
CHTF18	protein_coding	Yes	NM_022092.3	108
COTL1	protein_coding	Yes	NM_021149.5	108
ATP2C2	protein_coding	Yes	NM_014861.4	108
CHD5	protein_coding	Yes	NM_015557.3	108
GINS1	protein_coding	Yes	NM_021067.5	108
PITPNM3	protein_coding	Yes	NM_031220.4	108
ZNF629	protein_coding	Yes	NM_001080417.3	108
PCSK2	protein_coding	Yes	NM_002594.5	108
GSDMD	protein_coding	Yes	NM_024736.7	108
LGALS3BP	protein_coding	Yes	NM_005567.4	108
AMMECR1	protein_coding	Yes	NM_015365.3	108
PHYH	protein_coding	Yes	NM_006214.4	108
SCARF1	protein_coding	Yes	NM_003693.4	108
PRKCQ	protein_coding	Yes	NM_006257.5	108
UBE2R2	protein_coding	Yes	NM_017811.4	108
SYP	protein_coding	Yes	NM_003179.3	108
MREG	protein_coding	Yes	NM_018000.3	108
AP2S1	protein_coding	Yes	NM_004069.6	108
BLVRB	protein_coding	Yes	NM_000713.3	108
BIRC3	protein_coding	Yes	NM_001165.5	108
DDX25	protein_coding	Yes	NM_013264.5	108
OSBPL5	protein_coding	Yes	NM_020896.4	108
POLD3	protein_coding	Yes	NM_006591.3	108
LHX4	protein_coding	Yes	NM_033343.4	108
SRBD1	protein_coding	Yes	NM_018079.5	108
VAMP8	protein_coding	Yes	NM_003761.5	108
TNFRSF8	protein_coding	Yes	NM_001243.5	108
STK17B	protein_coding	Yes	NM_004226.4	108
IFT46	protein_coding	Yes	NM_001168618.2	108
GPC1	protein_coding	Yes	NM_002081.3	108
NGEF	protein_coding	Yes	NM_019850.3	108
EFHD1	protein_coding	Yes	NM_025202.4	108

ENST00000264079	145.27571	1.634574965	0.304929	5.360515	8.30E-08	3.19E-06	ENSG00000090674	19	7522623	7534009
ENST00000264159	408.33157	1.126396835	0.222172	5.06993	3.98E-07	1.23E-05	ENSG00000121988	2	135196968	135531218
ENST00000264218	62.415056	-2.90376955	0.470199	-6.175613	6.59E-10	4.49E-08	ENSG00000109255	4	55595230	55636298
ENST00000264220	1215.9643	-1.088374771	0.181544	-5.995108	2.03E-09	1.21E-07	ENSG00000128059	4	56393361	56435615
ENST00000264233	492.88212	-1.226548837	0.219915	-5.577374	2.44E-08	1.08E-06	ENSG00000051341	3	121431430	121545988
ENST00000264234	242.10969	-2.324891166	0.343602	-6.76623	1.32E-11	1.34E-09	ENSG00000114638	3	119173597	119205143
ENST00000264245	27.597733	-1.967321817	0.626066	-3.142357	0.001675935	0.012768994	ENSG00000031081	3	119294382	119420714
ENST00000264433	187.84273	-1.292144242	0.289165	-4.468538	7.88E-06	0.000158879	ENSG00000052795	4	158769025	158908050
ENST00000264711	132.77675	1.157579651	0.314166	3.684609	0.000229054	0.00264072	ENSG00000115137	2	24943641	24971955
ENST00000264741	30.694125	2.717172905	0.634493	4.28243	1.85E-05	0.000327329	ENSG00000144668	3	37452140	37823507
ENST00000264832	1124.0131	1.427309597	0.182256	7.831366	4.83E-15	9.48E-13	ENSG00000090339	19	10271119	10286615
ENST00000264866	112.68276	-1.102607835	0.333491	-3.306256	0.000945518	0.008132008	ENSG00000109680	4	26584083	26756223
ENST00000264893	1944.9202	-1.341977028	0.171616	-7.819664	5.30E-15	1.04E-12	ENSG00000138758	4	76949751	77038615
ENST00000265052	54.324194	-3.048582628	0.506888	-6.014316	1.81E-09	1.09E-07	ENSG00000074416	3	127689065	127822515
ENST00000265073	1893.5069	-1.006015135	0.173297	-5.805157	6.43E-09	3.38E-07	ENSG00000113387	5	32585556	32604079
ENST00000265104	42.158239	2.25088889	0.530523	4.242774	2.21E-05	0.000378497	ENSG00000039139	5	13690327	13944688
ENST00000265109	608.16079	-1.641352512	0.23045	-7.122387	1.06E-12	1.32E-10	ENSG00000039560	5	34656327	34832612
ENST00000265138	815.28946	1.031678501	0.218054	4.7313	2.23E-06	5.42E-05	ENSG00000113369	5	91368630	91383317
ENST00000265148	3127.5285	-1.335956925	0.166253	-8.035674	9.31E-16	2.00E-13	ENSG00000138778	4	103105810	103198343
ENST00000265239	92.810304	1.640530657	0.3616	4.536871	5.71E-06	0.000121196	ENSG00000114473	3	197889076	197959991
ENST00000265294	35.200495	-7.160846107	1.524336	-4.697681	2.63E-06	6.23E-05	ENSG00000094755	5	170783724	170814047
ENST00000265381	19.006443	-2.56558321	0.828179	-3.097859	0.00194924	0.014382542	ENSG00000107282	9	69427531	69672371
ENST00000265382	7.6125781	-6.401712062	1.786348	-3.583688	0.000338777	0.003613507	ENSG00000107242	9	68705239	69009176
ENST00000265394	69.868175	-3.272885688	0.529974	-6.175561	6.59E-10	4.49E-08	ENSG00000106066	7	28995234	29146537
ENST00000265440	4.2019998	5.493492792	2.024643	2.713315	0.006661379	0.037489128	ENSG00000105967	7	115935151	116030763
ENST00000265562	95.984702	1.096762826	0.400884	2.735863	0.006221696	0.035513837	ENSG00000076201	3	47381020	47413435
ENST00000265616	180.28902	1.110159492	0.290389	3.823014	0.00013183	0.001679439	ENSG00000104691	8	30744163	30767006
ENST00000265643	20.249522	-2.04611143	0.775593	-2.638126	0.008336549	0.044429045	ENSG00000069482	11	68684543	68691175
ENST00000265678	39.369447	1.848094434	0.565892	3.265809	0.001091517	0.009097628	ENSG00000071242	6	166409363	166627251
ENST00000265728	495.02924	-1.632174071	0.236427	-6.903499	5.07E-12	5.55E-10	ENSG00000006634	7	87876492	87909553
ENST00000265753	2154.1084	-1.263642484	0.187005	-6.757252	1.41E-11	1.42E-09	ENSG00000106682	7	74174355	74197096
ENST00000265806	222.07623	-1.957092304	0.265707	-7.365613	1.76E-13	2.58E-11	ENSG00000104679	8	23288107	23296279
ENST00000265896	1294.9699	-1.942449737	0.187157	-10.37873	3.10E-25	2.33E-22	ENSG00000104549	8	124998504	125022283
ENST00000266058	34.07056	2.270585857	0.591141	3.841021	0.000122524	0.001581063	ENSG00000187122	10	96998037	97185959

MCOLN1	protein_coding	Yes	NM_020533.3	108
ZRANB3	protein_coding	Yes	NM_032143.4	108
NMU	protein_coding	Yes	NM_006681.4	108
PPAT	protein_coding	Yes	NM_002703.5	108
POLQ	protein_coding	Yes	NM_199420.4	108
UPK1B	protein_coding	Yes	NM_006952.4	108
ARHGAP31	protein_coding	Yes	NM_020754.4	108
FNIP2	protein_coding	Yes	NM_020840.3	108
DNAJC27	protein_coding	Yes	NM_016544.3	108
ITGA9	protein_coding	Yes	NM_002207.3	108
ICAM1	protein_coding	Yes	NM_000201.3	108
TBC1D19	protein_coding	Yes	NM_018317.4	108
SEPTIN11	protein_coding	Yes	NM_018243.4	108
MGLL	protein_coding	Yes	NM_007283.7	108
SUB1	protein_coding	Yes	NM_006713.4	108
DNAH5	protein_coding	Yes	NM_001369.3	108
RAI14	protein_coding	Yes	NM_015577.3	108
ARRDC3	protein_coding	Yes	NM_020801.4	108
CENPE	protein_coding	Yes	NM_001813.3	108
IQCG	protein_coding	Yes	NM_032263.5	108
GABRP	protein_coding	Yes	NM_014211.3	108
APBA1	protein_coding	Yes	NM_001163.4	108
PIP5K1B	protein_coding	Yes	NM_003558.4	108
CPVL	protein_coding	Yes	NM_031311.5	108
TFEC	protein_coding	Yes	NM_012252.4	108
PTPN23	protein_coding	Yes	NM_015466.4	108
UBXN8	protein_coding	Yes	NM_005671.4	108
GAL	protein_coding	Yes	NM_015973.5	108
RPS6KA2	protein_coding	Yes	NM_021135.6	108
DBF4	protein_coding	Yes	NM_006716.4	108
EIF4H	protein_coding	Yes	NM_022170.2	108
R3HCC1	protein_coding	Yes	NM_001136108.3	108
SQLE	protein_coding	Yes	NM_003129.4	108
SLIT1	protein_coding	Yes	NM_003061.3	108

ENST00000266397	22.70525	-2.484746276	0.715863	-3.470982	0.00051856	0.005073123	ENSG00000139055	12	14914038	14938537
ENST00000266483	181.81329	-1.039232797	0.2814	-3.693084	0.000221551	0.00257036	ENSG00000139133	12	34022495	34028302
ENST00000266503	979.84724	-1.068132471	0.186792	-5.718297	1.08E-08	5.30E-07	ENSG00000029153	12	27332835	27425286
ENST00000266508	554.07123	-1.320728753	0.262141	-5.038234	4.70E-07	1.41E-05	ENSG00000139154	12	19439491	19522227
ENST00000266542	310.47995	1.197726604	0.328975	3.640785	0.000271808	0.003039143	ENSG00000139178	12	7094553	7109214
ENST00000266970	1657.9277	-1.123148767	0.173919	-6.457874	1.06E-10	8.96E-09	ENSG00000123374	12	55966829	55972789
ENST00000267328	221.0536	-1.371832292	0.284924	-4.814725	1.47E-06	3.78E-05	ENSG00000139832	13	110523065	110561722
ENST00000267853	97.908202	-1.626093346	0.352245	-4.616373	3.91E-06	8.75E-05	ENSG00000263155	15	57591903	57685364
ENST00000267970	935.94705	-1.701227088	0.224134	-7.590235	3.19E-14	5.42E-12	ENSG00000140391	15	77041403	77071109
ENST00000267978	354.95401	-1.574821729	0.247014	-6.375432	1.82E-10	1.47E-08	ENSG00000140400	15	75355791	75368607
ENST00000268261	424.45535	-1.086295035	0.225081	-4.826236	1.39E-06	3.59E-05	ENSG00000140650	16	8797838	8849325
ENST00000268483	221.21963	1.236173205	0.26251	4.709053	2.49E-06	5.94E-05	ENSG00000140830	16	72084856	72093620
ENST00000268607	425.60205	1.025992518	0.223324	4.594181	4.34E-06	9.58E-05	ENSG00000140941	16	87392335	87404774
ENST00000268766	71.957994	1.19367853	0.414236	2.881639	0.003956122	0.025029329	ENSG00000160602	17	28728787	28743455
ENST00000269195	963.62212	-1.111326697	0.214531	-5.180251	2.22E-07	7.38E-06	ENSG00000141429	18	35581739	35711834
ENST00000269298	279.88149	1.267884884	0.252138	5.028542	4.94E-07	1.47E-05	ENSG00000141504	17	7626233	7627821
ENST00000269389	145.60548	-1.268219817	0.397084	-3.193832	0.001403979	0.011109593	ENSG00000141574	17	82321023	82333766
ENST00000269881	12.472745	7.061946817	1.68312	4.195747	2.72E-05	0.000450759	ENSG00000269058	19	16479060	16496167
ENST00000270061	157.16565	-1.846492734	0.302427	-6.105585	1.02E-09	6.66E-08	ENSG00000130511	19	18419397	18434562
ENST00000270233	473.98269	-1.461873348	0.318101	-4.595623	4.31E-06	9.53E-05	ENSG00000187244	19	44809102	44821421
ENST00000270257	140.85564	-1.062365635	0.304055	-3.493989	0.00047586	0.004742411	ENSG00000142252	19	45079259	45091518
ENST00000270349	20.24189	2.812172382	0.785925	3.578167	0.000346012	0.003672427	ENSG00000142319	5	1392793	1445440
ENST00000270776	1839.4719	1.110470789	0.213925	5.19094	2.09E-07	7.02E-06	ENSG00000142657	1	10399063	10420511
ENST00000271002	267.74943	-1.106608564	0.244369	-4.528427	5.94E-06	0.000125286	ENSG00000142856	1	63440769	63523225
ENST00000271064	1579.8474	-1.496011328	0.178212	-8.394537	4.68E-17	1.19E-14	ENSG00000142910	1	31576514	31587686
ENST00000271375	1321.8533	-1.052740615	0.21444	-4.909261	9.14E-07	2.50E-05	ENSG00000213064	1	168226003	168253021
ENST00000271417	19.220135	-3.621644163	0.926314	-3.909736	9.24E-05	0.001247123	ENSG00000143195	1	166908186	166975540
ENST00000271452	709.68006	-1.015755465	0.194293	-5.227961	1.71E-07	5.92E-06	ENSG00000143228	1	163321953	163355759
ENST00000271636	615.07051	-1.416123787	0.202499	-6.993236	2.69E-12	3.09E-10	ENSG00000143375	1	151511386	151538692
ENST00000271915	7.8395407	6.394636132	1.775957	3.600671	0.000317397	0.003439132	ENSG00000143603	1	154697454	154870281
ENST00000272163	1957.1084	-1.131799457	0.172902	-6.545885	5.91E-11	5.22E-09	ENSG00000143815	1	225401501	225428038
ENST00000272217	125.79051	-1.197174031	0.315622	-3.793061	0.000148802	0.001857259	ENSG00000143862	1	202133403	202144737
ENST00000272462	810.66748	-1.809919073	0.19317	-9.369586	7.28E-21	2.93E-18	ENSG00000144063	2	110083869	110115822
ENST00000272519	756.74582	-1.389014809	0.19383	-7.166159	7.71E-13	9.85E-11	ENSG00000144118	2	120252851	120294710

ERP27	protein_coding	Yes	NM_152321.4	108
ALG10	protein_coding	Yes	NM_032834.4	108
ARNTL2	protein_coding	Yes	NM_020183.6	108
AEBP2	protein_coding	Yes	NM_153207.5	108
C1RL	protein_coding	Yes	NM_016546.4	108
CDK2	protein_coding	Yes	NM_001798.5	108
RAB20	protein_coding	Yes	NM_017817.3	108
MYZAP	protein_coding	Yes	NM_001018100.5	108
TSPAN3	protein_coding	Yes	NM_005724.6	108
MAN2C1	protein_coding	Yes	NM_006715.4	108
PMM2	protein_coding	Yes	NM_000303.3	108
TXNL4B	protein_coding	Yes	NM_017853.3	108
MAP1LC3B	protein_coding	Yes	NM_022818.5	108
NEK8	protein_coding	Yes	NM_178170.3	108
GALNT1	protein_coding	Yes	NM_020474.4	108
SAT2	protein_coding	Yes	NM_133491.5	108
SECTM1	protein_coding	Yes	NM_003004.3	108
CALR3	protein_coding	Yes	NM_145046.5	108
SSBP4	protein_coding	Yes	NM_032627.5	108
BCAM	protein_coding	Yes	NM_005581.5	108
GEMIN7	protein_coding	Yes	NM_024707.3	108
SLC6A3	protein_coding	Yes	NM_001044.5	108
PGD	protein_coding	Yes	NM_002631.4	108
ITGB3BP	protein_coding	Yes	NM_014288.5	108
TINAGL1	protein_coding	Yes	NM_022164.3	108
SFT2D2	protein_coding	Yes	NM_199344.3	108
ILDR2	protein_coding	Yes	NM_199351.3	108
NUF2	protein_coding	Yes	NM_145697.3	108
CGN	protein_coding	Yes	NM_020770.3	108
KCNN3	protein_coding	Yes	NM_002249.6	108
LBR	protein_coding	Yes	NM_002296.4	108
ARL8A	protein_coding	Yes	NM_138795.4	108
MALL	protein_coding	Yes	NM_005434.5	108
RALB	protein_coding	Yes	NM_002881.3	108

ENST00000272895	520.90795	-1.300863663	0.24874	-5.229816	1.70E-07	5.87E-06	ENSG00000144452	2	214931541	215138626
ENST00000273375	398.1039	1.003961965	0.227839	4.406457	1.05E-05	0.000202589	ENSG00000144840	3	120684937	120742516
ENST00000273857	44.962854	2.405732848	0.543334	4.427727	9.52E-06	0.000186081	ENSG00000145244	4	47594000	47838067
ENST00000274026	1672.4499	-2.144495433	0.195316	-10.97964	4.79E-28	5.00E-25	ENSG00000145386	4	121816443	121823883
ENST00000274031	1036.4943	-1.604208311	0.187688	-8.547211	1.26E-17	3.46E-15	ENSG00000145391	4	139506037	139556219
ENST00000274137	284.4278	-1.347173306	0.285222	-4.723245	2.32E-06	5.61E-05	ENSG00000145494	5	1801406	1816048
ENST00000274217	52.821082	-1.84267359	0.463103	-3.978968	6.92E-05	0.000983417	ENSG00000145569	5	14581791	14616180
ENST00000274255	1185.0846	-1.287922363	0.19236	-6.69539	2.15E-11	2.09E-09	ENSG00000145604	5	36152110	36184319
ENST00000274364	688.19992	-2.287414604	0.217071	-10.53764	5.79E-26	4.93E-23	ENSG00000145703	5	76403284	76708132
ENST00000274458	138.07302	-1.969313115	0.314403	-6.263659	3.76E-10	2.74E-08	ENSG00000145781	5	116085024	116293287
ENST00000274813	436.1221	1.062972745	0.227463	4.673171	2.97E-06	6.89E-05	ENSG00000146085	6	49430359	49463253
ENST00000275230	255.11826	1.845100209	0.306285	6.024131	1.70E-09	1.03E-07	ENSG00000146411	6	133987580	134052624
ENST00000276202	29.314081	-1.843824184	0.620289	-2.972526	0.002953602	0.019909835	ENSG00000147251	X	118495814	118686147
ENST00000276609	42.520433	1.659806066	0.552965	3.00165	0.002685205	0.01851377	ENSG00000147606	8	91249318	91398155
ENST00000276646	250.47195	1.129435026	0.253583	4.453907	8.43E-06	0.000167841	ENSG00000147642	8	109573977	109644822
ENST00000276654	29.432956	-2.820611803	0.652994	-4.319505	1.56E-05	0.000284721	ENSG00000147650	8	104489235	104589258
ENST00000277165	2601.4274	-1.149637542	0.172456	-6.666253	2.62E-11	2.49E-09	ENSG00000048828	9	93451684	93566112
ENST00000277225	148.4077	-6.909851868	0.718593	-9.615807	6.86E-22	3.27E-19	ENSG00000148143	9	106863165	107013634
ENST00000278060	50.794975	1.336498683	0.460241	2.903911	0.003685327	0.023652857	ENSG00000148832	10	133379261	133391694
ENST00000278317	12.651234	-7.135020687	1.659247	-4.300156	1.71E-05	0.000306434	ENSG00000130595	11	1919702	1938702
ENST00000278353	814.8068	-1.133041044	0.190028	-5.9625	2.48E-09	1.44E-07	ENSG00000149084	11	43680737	43856615
ENST00000278412	2842.7655	-1.788777622	0.205493	-8.704792	3.18E-18	9.43E-16	ENSG00000149136	11	57325987	57335892
ENST00000278903	1566.9327	-1.003210899	0.206488	-4.858455	1.18E-06	3.12E-05	ENSG00000149547	11	125569476	125584684
ENST00000278937	737.1336	-1.760231848	0.198969	-8.846769	9.01E-19	2.92E-16	ENSG00000149573	11	118253415	118264297
ENST00000278980	306.8789	-1.156704491	0.290234	-3.98542	6.74E-05	0.000961741	ENSG00000149600	20	32702698	32743467
ENST00000279281	965.69804	-1.665902291	0.193647	-8.602768	7.78E-18	2.21E-15	ENSG00000149823	11	65096213	65111862
ENST00000280350	62.975113	1.911018689	0.450984	4.237446	2.26E-05	0.000385501	ENSG00000150773	11	112067797	112074017
ENST00000280357	602.16131	1.208287846	0.240801	5.017776	5.23E-07	1.55E-05	ENSG00000150782	11	112143259	112164094
ENST00000280481	43.303671	-3.782188603	0.619119	-6.108989	1.00E-09	6.54E-08	ENSG00000150893	13	38687076	38887131
ENST00000280571	120.63602	1.197250911	0.343195	3.488544	0.000485659	0.004818994	ENSG00000150977	12	123415038	123436684
ENST00000280756	740.05343	-1.02560565	0.203597	-5.037431	4.72E-07	1.42E-05	ENSG00000151135	12	106955906	106974035
ENST00000280758	6.7503216	-6.230668702	1.851215	-3.365718	0.000763448	0.006880196	ENSG00000151136	12	107318433	107659642
ENST00000280772	549.34101	1.226059739	0.258222	4.748075	2.05E-06	5.05E-05	ENSG00000151150	10	60026297	60389875
ENST00000280800	129.27311	1.248023539	0.34306	3.637917	0.000274852	0.003066109	ENSG00000151176	12	113358586	113391629

ABCA12	protein_coding	Yes	NM_173076.3	108
RABL3	protein_coding	Yes	NM_173825.5	108
CORIN	protein_coding	Yes	NM_006587.4	108
CCNA2	protein_coding	Yes	NM_001237.5	108
SETD7	protein_coding	Yes	NM_030648.4	108
NDUFS6	protein_coding	Yes	NM_004553.6	108
OTULINL	protein_coding	Yes	NM_019018.3	108
SKP2	protein_coding	Yes	NM_005983.4	108
IQGAP2	protein_coding	Yes	NM_006633.5	108
COMMD10	protein_coding	Yes	NM_016144.4	108
MMUT	protein_coding	Yes	NM_000255.4	108
SLC2A12	protein_coding	Yes	NM_145176.3	108
DOCK11	protein_coding	Yes	NM_144658.4	108
SLC26A7	protein_coding	Yes	NM_052832.4	108
SYBU	protein_coding	Yes	NM_001099754.2	108
LRP12	protein_coding	Yes	NM_013437.5	108
FAM120A	protein_coding	Yes	NM_014612.5	108
ZNF462	protein_coding	Yes	NM_021224.6	108
PAOX	protein_coding	Yes	NM_152911.4	108
TNNT3	protein_coding	Yes	NM_006757.4	108
HSD17B12	protein_coding	Yes	NM_016142.3	108
SSRP1	protein_coding	Yes	NM_003146.3	108
EI24	protein_coding	Yes	NM_004879.5	108
MPZL2	protein_coding	Yes	NM_005797.4	108
COMMD7	protein_coding	Yes	NM_053041.3	108
VPS51	protein_coding	Yes	NM_013265.4	108
PIH1D2	protein_coding	Yes	NM_138789.4	108
IL18	protein_coding	Yes	NM_001562.4	108
FREM2	protein_coding	Yes	NM_207361.6	108
RILPL2	protein_coding	Yes	NM_145058.3	108
TMEM263	protein_coding	Yes	NM_152261.4	108
ABTB3	protein_coding	Yes	NM_001018072.2	108
ANK3	protein_coding	Yes	NM_020987.5	108
PLBD2	protein_coding	Yes	NM_173542.4	108

ENST00000280871	170.91136	-2.532202084	0.331098	-7.6479	2.04E-14	3.62E-12	ENSG00000151229	12	39755024	40106081
ENST00000281172	684.70475	-1.106118573	0.19567	-5.652992	1.58E-08	7.28E-07	ENSG00000151491	12	15620133	15789388
ENST00000281282	21.131084	-3.302085648	0.808666	-4.083376	4.44E-05	0.000682913	ENSG00000128849	15	57376504	57550717
ENST00000281455	1254.8147	-1.08768544	0.192641	-5.646179	1.64E-08	7.54E-07	ENSG00000151726	4	184755594	184825968
ENST00000281471	235.56187	2.876793471	0.316025	9.103066	8.78E-20	3.19E-17	ENSG00000151743	12	31671141	31729021
ENST00000281631	194.06709	-6.617854335	0.580542	-11.39944	4.21E-30	5.05E-27	ENSG00000151883	5	50666636	50846519
ENST00000281703	7.4579944	6.325573025	1.803012	3.508337	0.000450918	0.004542892	ENSG00000151948	12	128853477	128984968
ENST00000281772	271.91255	1.047633995	0.243413	4.303937	1.68E-05	0.000301995	ENSG00000144445	2	210021420	210171409
ENST00000281828	584.68313	-1.033854446	0.217176	-4.760447	1.93E-06	4.79E-05	ENSG00000116120	2	222566898	222656092
ENST00000281834	70.798803	1.151047083	0.397543	2.895403	0.003786725	0.024167271	ENSG00000117586	1	173183730	173207331
ENST00000281923	643.36613	-1.128938796	0.199247	-5.666035	1.46E-08	6.83E-07	ENSG00000152127	2	134254071	134454621
ENST00000281924	4.5926543	5.625398721	1.981882	2.838412	0.004533861	0.02775253	ENSG00000152128	2	134455758	134719000
ENST00000282041	534.59479	1.306009414	0.212209	6.154349	7.54E-10	5.07E-08	ENSG00000152223	18	45847608	45967329
ENST00000282074	167.59944	-1.492248272	0.28721	-5.195661	2.04E-07	6.87E-06	ENSG00000152253	2	168870890	168890430
ENST00000282272	57.652006	-1.356926062	0.44904	-3.02184	0.002512435	0.017571708	ENSG00000065413	2	196986661	197310780
ENST00000282588	13.830754	-2.600869825	0.912546	-2.850125	0.0043702	0.02697859	ENSG00000213949	5	52787915	52959209
ENST00000282633	1060.7736	2.158682162	0.200978	10.74086	6.54E-27	5.97E-24	ENSG00000099290	10	50067953	50133509
ENST00000283109	279.89322	-1.022104802	0.26	-3.931165	8.45E-05	0.001160207	ENSG00000058729	5	97160866	97183247
ENST00000283441	105.74393	1.588090177	0.352617	4.503728	6.68E-06	0.000138252	ENSG00000188818	5	795604	850986
ENST00000283871	14.674777	2.874267334	0.926647	3.101792	0.00192353	0.014230031	ENSG00000113924	3	120628171	120682239
ENST00000283928	21.76672	-4.847297202	1.040712	-4.657673	3.20E-06	7.36E-05	ENSG00000153814	7	27830576	28180795
ENST00000283946	92.394907	-1.264264995	0.380802	-3.320004	0.000900163	0.007828069	ENSG00000153832	2	229922502	230013119
ENST00000284031	689.19029	-1.591863572	0.199408	-7.982948	1.43E-15	3.03E-13	ENSG00000153904	1	85318484	85465159
ENST00000284116	160.04977	1.603070789	0.380561	4.212386	2.53E-05	0.000424191	ENSG00000153982	17	59220510	59275970
ENST00000284245	41.321513	-2.104469675	0.531959	-3.956071	7.62E-05	0.001064884	ENSG00000154102	16	85707516	85751096
ENST00000284268	160.65414	-1.006340887	0.29032	-3.466322	0.000527632	0.005145032	ENSG00000154122	5	14704799	14871778
ENST00000284273	1922.1778	-1.616112194	0.219126	-7.375264	1.64E-13	2.41E-11	ENSG00000154127	11	122655721	122814473
ENST00000285106	488.88373	1.178244484	0.214121	5.502699	3.74E-08	1.58E-06	ENSG00000154832	18	50282346	50287692
ENST00000285176	35.695106	1.817157066	0.568139	3.198435	0.001381758	0.010970164	ENSG00000154874	17	18542154	18625447
ENST00000285238	61.311604	-1.76393928	0.431082	-4.091887	4.28E-05	0.000662263	ENSG00000108846	17	50634880	50692253
ENST00000285419	194.61649	-1.028044819	0.284468	-3.613918	0.000301605	0.00330324	ENSG00000155099	8	90993801	91040859
ENST00000285697	286.44859	-1.681501506	0.244802	-6.868832	6.47E-12	6.94E-10	ENSG00000155330	16	46796602	46831180
ENST00000285968	2648.6091	-1.39524976	0.169532	-8.230026	1.87E-16	4.45E-14	ENSG00000155561	7	135557916	135648753
ENST00000285979	25.331992	2.286427774	0.668344	3.421033	0.000623837	0.005867297	ENSG00000108242	10	94683728	94736190

SLC2A13	protein_coding	Yes	NM_052885.4	108
EPS8	protein_coding	Yes	NM_004447.6	108
CGNL1	protein_coding	Yes	NM_032866.5	108
ACSL1	protein_coding	Yes	NM_001995.5	108
AMN1	protein_coding	Yes	NM_001113402.2	108
PARP8	protein_coding	Yes	NM_024615.4	108
GLT1D1	protein_coding	Yes	NM_144669.3	108
KANSL1L	protein_coding	Yes	NM_152519.4	108
FARSB	protein_coding	Yes	NM_005687.5	108
TNFSF4	protein_coding	Yes	NM_003326.5	108
MGAT5	protein_coding	Yes	NM_002410.5	108
TMEM163	protein_coding	Yes	NM_030923.5	108
EPG5	protein_coding	Yes	NM_020964.3	108
SPC25	protein_coding	Yes	NM_020675.4	108
ANKRD44	protein_coding	Yes	NM_001195144.2	108
ITGA1	protein_coding	Yes	NM_181501.2	108
WASHC2A	protein_coding	Yes	NM_001005751.3	108
RIOK2	protein_coding	Yes	NM_018343.3	108
ZDHHC11	protein_coding	Yes	NM_024786.3	108
HGD	protein_coding	Yes	NM_000187.4	108
JAZF1	protein_coding	Yes	NM_175061.4	108
FBXO36	protein_coding	Yes	NM_174899.5	108
DDAH1	protein_coding	Yes	NM_012137.4	108
GDPD1	protein_coding	Yes	NM_182569.4	108
C16orf74	protein_coding	Yes	NM_206967.3	108
ANKH	protein_coding	Yes	NM_054027.6	108
UBASH3B	protein_coding	Yes	NM_032873.5	108
CXXC1	protein_coding	Yes	NM_014593.4	108
CCDC144BP	unprocessed_p	Yes	-	108
ABCC3	protein_coding	Yes	NM_003786.4	108
PIP4P2	protein_coding	Yes	NM_018710.3	108
C16orf87	protein_coding	Yes	NM_001001436.4	108
NUP205	protein_coding	Yes	NM_015135.3	108
CYP2C18	protein_coding	Yes	NM_000772.3	108

ENST00000286049	59.16502	-2.936125354	0.479401	-6.12457	9.09E-10	6.00E-08	ENSG00000155622	X	52369020	52375680
ENST00000286063	4.8194797	-5.742264601	1.956346	-2.935198	0.003333346	0.02185018	ENSG00000128655	2	177623243	178072777
ENST00000286234	56.385366	1.627389609	0.501618	3.244283	0.001177466	0.009670159	ENSG00000155792	8	119873721	120050918
ENST00000286298	651.39805	1.078232419	0.201719	5.345225	9.03E-08	3.42E-06	ENSG00000155850	5	149960757	149987400
ENST00000286544	143.2173	2.197068274	0.324696	6.766537	1.32E-11	1.34E-09	ENSG00000156050	14	73932275	73950094
ENST00000286548	1292.5588	-1.385491472	0.180252	-7.686412	1.51E-14	2.76E-12	ENSG00000156052	9	77716096	78031811
ENST00000286648	572.58732	-1.125140987	0.213567	-5.268332	1.38E-07	4.90E-06	ENSG00000156136	4	70993648	71030914
ENST00000286733	146.36852	-2.528135285	0.325493	-7.767104	8.03E-15	1.54E-12	ENSG00000138744	4	75913659	75941013
ENST00000287078	499.42509	1.340671268	0.213794	6.270847	3.59E-10	2.64E-08	ENSG00000156521	10	70137980	70146700
ENST00000287097	13.516898	4.672766021	1.29007	3.622104	0.000292217	0.003224595	ENSG00000156535	6	73696202	73828313
ENST00000287387	187.26101	-1.164187912	0.274758	-4.237136	2.26E-05	0.000385867	ENSG00000156795	8	123416773	123442240
ENST00000287598	1572.8985	-1.20864771	0.217298	-5.562162	2.66E-08	1.16E-06	ENSG00000156970	15	40161068	40221123
ENST00000287814	8.3902222	-5.047496769	1.732615	-2.913225	0.003577172	0.023108564	ENSG00000157150	3	12153067	12158912
ENST00000287934	72.616318	-1.583708002	0.415793	-3.808883	0.000139596	0.001761981	ENSG00000157240	7	91264432	91271326
ENST00000288040	20.418122	2.119242542	0.745245	2.843685	0.004459511	0.027391118	ENSG00000157322	16	69951333	69963986
ENST00000288050	633.07831	1.016822707	0.218128	4.661579	3.14E-06	7.24E-05	ENSG00000090857	16	70114331	70162537
ENST00000288087	69.625213	-1.290245201	0.409952	-3.14731	0.001647801	0.012597459	ENSG00000213920	14	24213942	24216066
ENST00000288235	1568.4556	-1.35803988	0.181535	-7.480869	7.38E-14	1.15E-11	ENSG00000157483	15	59132433	59372871
ENST00000289269	469.47124	1.236665974	0.226008	5.471785	4.46E-08	1.85E-06	ENSG00000243232	5	140966475	141012347
ENST00000289272	495.49055	1.241131936	0.228499	5.431684	5.58E-08	2.26E-06	ENSG00000239389	5	140882123	141012347
ENST00000289431	79.456163	1.030173806	0.377848	2.726423	0.006402482	0.036337555	ENSG00000158480	20	49903390	49915529
ENST00000289734	86.60577	-2.08889117	0.452479	-4.616553	3.90E-06	8.74E-05	ENSG00000029534	8	41653224	41797622
ENST00000290354	59.491251	-2.460836637	0.480307	-5.123468	3.00E-07	9.58E-06	ENSG00000159231	21	36135078	36146562
ENST00000290438	17.663796	7.566376516	1.603116	4.719795	2.36E-06	5.69E-05	ENSG00000159289	15	74069856	74082550
ENST00000290510	36.215804	-2.76612054	0.589431	-4.692867	2.69E-06	6.35E-05	ENSG00000110811	12	6828406	6839847
ENST00000290573	3.9197703	5.394631232	2.054307	2.626011	0.008639213	0.045513691	ENSG00000159399	2	74834126	74893359
ENST00000290776	270.86048	-1.100176211	0.256988	-4.281037	1.86E-05	0.00032895	ENSG00000140848	16	57092582	57148369
ENST00000290868	4.6423127	5.639148354	1.971416	2.860456	0.004230322	0.026324826	ENSG00000159650	3	126481165	126517773
ENST00000290902	81.679794	-3.757940803	0.460748	-8.156172	3.46E-16	7.96E-14	ENSG00000159674	4	1166931	1172583
ENST00000291539	12.747321	-5.683828113	1.659778	-3.42445	0.000616045	0.005811588	ENSG00000160191	21	42653620	42775509
ENST00000292090	214.90504	1.137313813	0.292194	3.892324	9.93E-05	0.001324291	ENSG00000160606	17	28724347	28726233
ENST00000292430	384.1078	-1.527279864	0.242535	-6.297145	3.03E-10	2.29E-08	ENSG00000160886	8	142700110	142705127
ENST00000292475	780.01957	-1.226042696	0.239097	-5.127814	2.93E-07	9.38E-06	ENSG00000241468	7	99458194	99466167
ENST00000292476	293.95164	-1.406153314	0.244571	-5.749478	8.95E-09	4.51E-07	ENSG00000160917	7	99438942	99457373

XAGE2	protein_coding	Yes	NM_130777.3	108
PDE11A	protein_coding	Yes	NM_016953.4	108
DEPTOR	protein_coding	Yes	NM_022783.4	108
SLC26A2	protein_coding	Yes	NM_000112.4	108
FAM161B	protein_coding	Yes	NM_152445.3	108
GNAQ	protein_coding	Yes	NM_002072.5	108
DCK	protein_coding	Yes	NM_000788.3	108
NAAA	protein_coding	Yes	NM_014435.4	108
TYSND1	protein_coding	Yes	NM_173555.4	108
CD109	protein_coding	Yes	NM_133493.5	108
NTAQ1	protein_coding	Yes	NM_018024.3	108
BUB1B	protein_coding	Yes	NM_001211.6	108
TIMP4	protein_coding	Yes	NM_003256.4	108
FZD1	protein_coding	Yes	NM_003505.2	108
CLEC18A	protein_coding	Yes	NM_001370523.4	108
PDPR	protein_coding	Yes	NM_017990.5	108
MDP1	protein_coding	Yes	NM_138476.4	108
MYO1E	protein_coding	Yes	NM_004998.4	108
PCDHAC2	protein_coding	Yes	NM_018899.6	108
PCDHA13	protein_coding	Yes	NM_018904.3	108
SPATA2	protein_coding	Yes	NM_006038.4	108
ANK1	protein_coding	Yes	NM_000037.4	108
CBR3	protein_coding	Yes	NM_001236.4	108
GOLGA6A	protein_coding	Yes	NM_001038640.2	108
P3H3	protein_coding	Yes	NM_014262.5	108
HK2	protein_coding	Yes	NM_000189.5	108
CPNE2	protein_coding	Yes	NM_152727.6	108
UROC1	protein_coding	Yes	NM_144639.3	108
SPON2	protein_coding	Yes	NM_012445.4	108
PDE9A	protein_coding	Yes	NM_002606.3	108
TLCD1	protein_coding	Yes	NM_138463.4	108
LY6K	protein_coding	Yes	NM_017527.4	108
ATP5MF	protein_coding	Yes	NM_004889.5	108
CPSF4	protein_coding	Yes	NM_006693.4	108

ENST00000292494	3831.7922	-1.442782235	0.184885	-7.803656	6.01E-15	1.17E-12	ENSG00000160932	8	143018528	143022409
ENST00000292841	264.24664	1.085315508	0.266908	4.066256	4.78E-05	0.000725814	ENSG00000196967	19	37145539	37172695
ENST00000292894	28.708427	1.887061606	0.6265	3.01207	0.002594727	0.018028945	ENSG00000161277	19	36034983	36054285
ENST00000293230	9.4169363	3.490409746	1.276719	2.733891	0.006259075	0.035683237	ENSG00000161544	17	76527355	76537709
ENST00000293379	200.40996	-1.026262228	0.283131	-3.624696	0.000289302	0.00319814	ENSG00000161638	12	54395260	54419266
ENST00000293826	70.457561	1.602073766	0.403573	3.969726	7.20E-05	0.001015442	ENSG00000248871	17	7549098	7561601
ENST00000293829	182.42101	2.760344348	0.357159	7.72861	1.09E-14	2.04E-12	ENSG00000161958	17	7439511	7444937
ENST00000294172	473.9234	1.234114949	0.211969	5.822141	5.81E-09	3.09E-07	ENSG00000162231	11	62792129	62805440
ENST00000294454	23.2937	2.026957425	0.702686	2.884587	0.003919279	0.024843669	ENSG00000162461	1	15736257	15741392
ENST00000294484	25.228542	2.854860537	0.735546	3.881282	0.000103907	0.001375184	ENSG00000204624	1	11479154	11537551
ENST00000294785	444.07944	1.153607987	0.219932	5.245287	1.56E-07	5.47E-06	ENSG00000162736	1	160343382	160358949
ENST00000294889	189.79617	-3.35250739	0.319528	-10.49207	9.39E-26	7.78E-23	ENSG00000162817	1	220690362	220699153
ENST00000295030	660.26648	-1.469005728	0.208076	-7.059933	1.67E-12	2.01E-10	ENSG00000162928	2	61017719	61051990
ENST00000295156	7.1332798	-6.30724078	1.812898	-3.479093	0.000503114	0.0049528	ENSG00000163032	2	17540695	17657018
ENST00000295228	14.209283	-3.775560809	1.088579	-3.468337	0.00052369	0.005114774	ENSG00000163083	2	120346135	120351803
ENST00000295246	754.66025	1.110718915	0.206628	5.375449	7.64E-08	2.96E-06	ENSG00000174501	2	95849547	95991824
ENST00000295304	65.415769	-1.773218208	0.439472	-4.034883	5.46E-05	0.000811435	ENSG00000143942	2	53767803	53775196
ENST00000295448	33.396091	-4.005837764	0.720194	-5.562165	2.66E-08	1.16E-06	ENSG00000163281	4	44701794	44726556
ENST00000295453	58.806188	2.049782257	0.468218	4.377833	1.20E-05	0.000226687	ENSG00000163286	2	232406843	232410714
ENST00000295461	320.94769	-2.124416205	0.241891	-8.782553	1.60E-18	5.03E-16	ENSG00000163293	4	48016771	48040173
ENST00000295887	1023.5749	-1.239626179	0.186697	-6.639765	3.14E-11	2.95E-09	ENSG00000163624	4	84583126	84651334
ENST00000295899	280.6249	-1.066927783	0.254042	-4.199812	2.67E-05	0.00044431	ENSG00000163634	3	63833869	63863805
ENST00000296233	19.524364	7.711695216	1.588569	4.854491	1.21E-06	3.17E-05	ENSG00000163884	3	126342634	126357408
ENST00000296370	477.78962	-2.229363095	0.251875	-8.851063	8.67E-19	2.82E-16	ENSG00000163993	4	6693877	6697170
ENST00000296417	4803.5698	-1.309693346	0.185638	-7.055085	1.72E-12	2.07E-10	ENSG00000164032	4	99948087	99950275
ENST00000296452	23.034735	1.824979292	0.682896	2.672411	0.007530825	0.041079793	ENSG00000164061	3	49554476	49671549
ENST00000296474	283.4517	1.313450963	0.265079	4.954935	7.24E-07	2.05E-05	ENSG00000164078	3	49887001	49903873
ENST00000296498	141.56037	-3.822182582	0.43595	-8.76748	1.83E-18	5.71E-16	ENSG00000164099	4	118280037	118353003
ENST00000296503	1872.2203	-1.516099602	0.174111	-8.707661	3.10E-18	9.25E-16	ENSG00000164104	4	173331375	173334358
ENST00000296509	1112.6205	-1.395732364	0.187901	-7.428037	1.10E-13	1.66E-11	ENSG00000164109	4	120055622	120066848
ENST00000296513	143.67542	1.066991114	0.303921	3.510757	0.000446832	0.00451516	ENSG00000164113	4	122379010	122429784
ENST00000296550	43.130211	-1.62471397	0.514217	-3.159588	0.001579926	0.012193547	ENSG00000170390	4	150078444	150257438
ENST00000296581	182.01669	-1.484742303	0.287511	-5.164119	2.42E-07	7.94E-06	ENSG00000164167	4	146175717	146191535
ENST00000296684	417.75524	-1.206446499	0.217106	-5.556939	2.75E-08	1.20E-06	ENSG00000164258	5	53560638	53683338

LY6E	protein_coding	Yes	NM_002346.3	108
ZNF585A	protein_coding	Yes	NM_001288800.2	108
THAP8	protein_coding	Yes	NM_152658.3	108
CYGB	protein_coding	Yes	NM_134268.5	108
ITGA5	protein_coding	Yes	NM_002205.5	108
TNFSF12-TNFSF13	protein_coding	Yes	-	108
FGF11	protein_coding	Yes	NM_004112.4	108
NXF1	protein_coding	Yes	NM_006362.5	108
SLC25A34	protein_coding	Yes	NM_207348.3	108
DISP3	protein_coding	Yes	NM_020780.2	108
NCSTN	protein_coding	Yes	NM_015331.3	108
C1orf115	protein_coding	Yes	NM_024709.5	108
PEX13	protein_coding	Yes	NM_002618.4	108
VSNL1	protein_coding	Yes	NM_003385.5	108
INHBB	protein_coding	Yes	NM_002193.4	108
ANKRD36C	protein_coding	Yes	NM_001393982.1	108
CHAC2	protein_coding	Yes	NM_001008708.4	108
GNPDA2	protein_coding	Yes	NM_138335.3	108
ALPG	protein_coding	Yes	NM_031313.3	108
NIPAL1	protein_coding	Yes	NM_207330.3	108
CDS1	protein_coding	Yes	NM_001263.4	108
THOC7	protein_coding	Yes	NM_025075.4	108
KLF15	protein_coding	Yes	NM_014079.4	108
S100P	protein_coding	Yes	NM_005980.3	108
H2AZ1	protein_coding	Yes	NM_002106.4	108
BSN	protein_coding	Yes	NM_003458.4	108
MST1R	protein_coding	Yes	NM_002447.4	108
PRSS12	protein_coding	Yes	NM_003619.4	108
HMGB2	protein_coding	Yes	NM_002129.4	108
MAD2L1	protein_coding	Yes	NM_002358.4	108
ADAD1	protein_coding	Yes	NM_139243.4	108
DCLK2	protein_coding	Yes	NM_001040260.4	108
LSM6	protein_coding	Yes	NM_007080.3	108
NDUFS4	protein_coding	Yes	NM_002495.4	108

ENST00000296978	484.26694	-1.868669917	0.244571	-7.640614	2.16E-14	3.82E-12	ENSG00000164484	6	130366016	130443067
ENST00000297164	147.06547	1.789515317	0.313242	5.71289	1.11E-08	5.44E-07	ENSG00000164620	5	141636996	141641064
ENST00000297205	28.890577	1.923489026	0.637829	3.015683	0.002564009	0.017858662	ENSG00000164647	7	90154468	90164827
ENST00000297477	931.6047	1.010860313	0.285412	3.541755	0.000397475	0.00410904	ENSG00000164855	7	1542234	1556205
ENST00000297494	89.626216	5.105426807	0.57469	8.883792	6.46E-19	2.15E-16	ENSG00000164867	7	150991016	151014588
ENST00000297533	126.50607	-1.364585899	0.322147	-4.235908	2.28E-05	0.000387766	ENSG00000164897	7	151081084	151083493
ENST00000297625	405.29002	-1.075604996	0.218635	-4.919629	8.67E-07	2.39E-05	ENSG00000164976	9	34366665	34376898
ENST00000297632	440.89909	-1.349880241	0.293348	-4.601629	4.19E-06	9.30E-05	ENSG00000164983	8	124306188	124372701
ENST00000297689	164.66612	-1.379449931	0.286962	-4.807089	1.53E-06	3.91E-05	ENSG00000165030	9	91409044	91423832
ENST00000297785	4.8997129	5.716560879	1.947521	2.935301	0.00333224	0.021846691	ENSG00000165092	9	72900670	72953053
ENST00000297875	13.41523	-5.744949461	1.626299	-3.532529	0.000411605	0.004227019	ENSG00000147041	X	38006552	38128816
ENST00000297991	588.49416	-4.273954172	0.31082	-13.75056	5.05E-43	1.24E-39	ENSG00000165272	9	33441159	33447593
ENST00000298048	2782.1234	-1.496333358	0.215867	-6.931751	4.16E-12	4.63E-10	ENSG00000165304	9	36572894	36677682
ENST00000298159	570.58926	-1.450309012	0.20378	-7.117018	1.10E-12	1.37E-10	ENSG00000165410	14	34709112	34714593
ENST00000298288	344.06859	-1.148532642	0.272923	-4.208263	2.57E-05	0.000430628	ENSG00000165501	14	49598939	49614672
ENST00000298299	299.14507	-1.712583286	0.322029	-5.318111	1.05E-07	3.87E-06	ENSG00000165512	10	45000922	45005326
ENST00000298316	2670.2732	-1.392511714	0.182522	-7.629284	2.36E-14	4.12E-12	ENSG00000165527	14	49893081	49897054
ENST00000298596	184.66515	1.362923727	0.278393	4.895684	9.80E-07	2.65E-05	ENSG00000165730	10	68827530	68893060
ENST00000298838	636.97641	-1.094015317	0.209634	-5.218691	1.80E-07	6.17E-06	ENSG00000165912	11	47177521	47186434
ENST00000298966	18.370512	-2.393840525	0.781083	-3.064771	0.002178364	0.015688512	ENSG00000166002	11	93478471	93543391
ENST00000299001	28.607694	-1.810379244	0.608329	-2.975989	0.002920452	0.019736133	ENSG00000134627	11	94567367	94621421
ENST00000299045	113.31396	1.796519273	0.41795	4.298409	1.72E-05	0.000308422	ENSG00000166046	12	106302732	106347003
ENST00000299084	560.42306	-2.979197949	0.2163	-13.77347	3.68E-43	9.51E-40	ENSG00000166068	15	38252835	38357249
ENST00000299157	188.87235	-1.641948254	0.28246	-5.813035	6.14E-09	3.24E-07	ENSG00000166130	12	98624248	98644788
ENST00000299213	17.667315	-3.464279939	0.896872	-3.862623	0.000112176	0.001465531	ENSG00000166173	15	70829129	70854157
ENST00000299353	289.30004	1.211644785	0.240056	5.047333	4.48E-07	1.36E-05	ENSG00000270316	10	102854271	102901899
ENST00000299427	102.99295	1.211346775	0.370395	3.270421	0.001073874	0.008977482	ENSG00000166340	11	6612767	6619422
ENST00000299498	25.384118	2.282715739	0.679422	3.35979	0.000780017	0.006999299	ENSG00000166394	11	7665103	7673500
ENST00000299601	493.84621	-1.152697027	0.217109	-5.309296	1.10E-07	4.04E-06	ENSG00000166477	15	51938024	51971778
ENST00000299698	9.4197829	-6.708485174	1.731387	-3.87463	0.000106787	0.001406331	ENSG00000166535	12	8822620	8876787
ENST00000299798	28.432665	2.935050531	0.673217	4.35974	1.30E-05	0.000243247	ENSG00000135740	16	67248978	67272191
ENST00000300030	712.07269	-1.125169445	0.213674	-5.265829	1.40E-07	4.96E-06	ENSG00000166797	15	64072564	64093838
ENST00000300093	356.96391	-1.692848106	0.268514	-6.304502	2.89E-10	2.20E-08	ENSG00000166851	16	23678888	23690367
ENST00000300119	25.594835	2.105989143	0.659628	3.192694	0.001409521	0.011144185	ENSG00000166866	12	57028516	57050129

TMEM200A	protein_coding	Yes	NM_001258277.2	108
RELL2	protein_coding	Yes	NM_173828.5	108
STEAP1	protein_coding	Yes	NM_012449.3	108
TMEM184A	protein_coding	Yes	NM_001097620.2	108
NOS3	protein_coding	Yes	NM_000603.5	108
TMUB1	protein_coding	Yes	NM_001136044.2	108
MYORG	protein_coding	Yes	NM_020702.5	108
TMEM65	protein_coding	Yes	NM_194291.3	108
NFIL3	protein_coding	Yes	NM_005384.3	108
ALDH1A1	protein_coding	Yes	NM_000689.5	108
SYTL5	protein_coding	Yes	NM_138780.3	108
AQP3	protein_coding	Yes	NM_004925.5	108
MELK	protein_coding	Yes	NM_014791.4	108
CFL2	protein_coding	Yes	NM_138638.5	108
LRR1	protein_coding	Yes	NM_152329.4	108
ZNF22	protein_coding	Yes	NM_006963.5	108
ARF6	protein_coding	Yes	NM_001663.4	108
STOX1	protein_coding	Yes	NM_152709.5	108
PACSIN3	protein_coding	Yes	NM_016223.5	108
SMCO4	protein_coding	Yes	NM_020179.3	108
PIWIL4	protein_coding	Yes	NM_152431.3	108
TCP11L2	protein_coding	Yes	NM_152772.3	108
SPRED1	protein_coding	Yes	NM_152594.3	108
IKBIP	protein_coding	Yes	NM_153687.4	108
LARP6	protein_coding	Yes	NM_018357.4	108
BORCS7-ASMT	protein_coding	Yes	-	108
TPP1	protein_coding	Yes	NM_000391.4	108
CYB5R2	protein_coding	Yes	NM_016229.5	108
LEO1	protein_coding	Yes	NM_138792.4	108
A2ML1	protein_coding	Yes	NM_144670.6	108
SLC9A5	protein_coding	Yes	NM_004594.3	108
CIAO2A	protein_coding	Yes	NM_032231.7	108
PLK1	protein_coding	Yes	NM_005030.6	108
MYO1A	protein_coding	Yes	NM_005379.4	108

ENST00000300179	50.873147	-1.51557382	0.461787	-3.281973	0.001030835	0.008679181	ENSG00000166924	7	100483926	100494802
ENST00000300291	3667.9464	-1.240321649	0.16522	-7.507103	6.05E-14	9.58E-12	ENSG00000167005	16	56429132	56451332
ENST00000300527	45.968341	-3.057261337	0.622731	-4.909438	9.13E-07	2.50E-05	ENSG00000142173	21	46098111	46132848
ENST00000300571	46.423477	-2.180347114	0.514429	-4.23838	2.25E-05	0.000384294	ENSG00000167191	16	19856690	19884848
ENST00000300784	31.747785	1.65958706	0.591062	2.807807	0.004988007	0.029947887	ENSG00000167363	17	82735614	82751196
ENST00000301178	880.46765	-2.337509453	0.210239	-11.11837	1.02E-28	1.10E-25	ENSG00000167601	19	41219222	41261766
ENST00000301200	7.4862194	-4.872719536	1.836805	-2.652823	0.00798217	0.042970137	ENSG00000167617	19	54465025	54473296
ENST00000301263	23.279874	-1.888169403	0.697805	-2.70587	0.006812569	0.038152509	ENSG00000167656	8	142784881	142786539
ENST00000301305	55.601342	-1.257960051	0.440093	-2.858395	0.004257901	0.026443543	ENSG00000147804	8	144412413	144416844
ENST00000301335	194.59108	2.360749179	0.294594	8.013567	1.11E-15	2.38E-13	ENSG00000167703	17	1569267	1628834
ENST00000301459	36.808291	-1.827536691	0.550092	-3.322236	0.000892993	0.007783477	ENSG00000167771	11	63911229	63917164
ENST00000301480	13.353371	7.161550732	1.655575	4.325719	1.52E-05	0.000277706	ENSG00000198028	19	9466354	9498616
ENST00000301686	160.36446	-1.26961146	0.29015	-4.375701	1.21E-05	0.000228572	ENSG00000130731	16	634429	636305
ENST00000301891	14.605636	-3.472753467	0.997305	-3.482138	0.000497428	0.00490734	ENSG00000168065	11	64555940	64572875
ENST00000301905	597.04924	-1.975142585	0.204605	-9.653434	4.75E-22	2.34E-19	ENSG00000168078	8	27809623	27837817
ENST00000302118	18.617707	-4.185632317	0.989251	-4.231114	2.33E-05	0.000394688	ENSG00000169174	1	55039547	55064852
ENST00000302262	755.35977	1.645044161	0.286938	5.733093	9.86E-09	4.92E-07	ENSG00000171298	17	80101580	80119881
ENST00000302274	22.027096	-5.460961553	1.192876	-4.577981	4.69E-06	0.000102543	ENSG00000170522	4	110045845	110198615
ENST00000302327	129.853	-1.144392135	0.33156	-3.45154	0.000557398	0.005370438	ENSG00000168556	4	183505057	183512429
ENST00000302450	472.63491	-1.34593074	0.234936	-5.728931	1.01E-08	5.02E-07	ENSG00000169607	2	112736348	112764609
ENST00000302586	79.171514	1.515235857	0.383194	3.954224	7.68E-05	0.001071767	ENSG00000171097	9	128832941	128881950
ENST00000302631	376.28017	1.098061014	0.224813	4.88434	1.04E-06	2.78E-05	ENSG00000116521	1	155255980	155262360
ENST00000302851	578.95902	1.101460497	0.206506	5.333784	9.62E-08	3.61E-06	ENSG00000171469	19	9607328	9621232
ENST00000303037	9.7994259	6.716566272	1.715337	3.915596	9.02E-05	0.001221306	ENSG00000168928	16	75204102	75207161
ENST00000303142	37.196744	1.843914122	0.541761	3.403558	0.000665142	0.006173118	ENSG00000171984	20	5750400	5864395
ENST00000303151	309.02017	-1.174676591	0.273428	-4.296106	1.74E-05	0.000311166	ENSG00000172336	7	100706120	100707486
ENST00000303251	120.09797	1.705912503	0.334642	5.097724	3.44E-07	1.08E-05	ENSG00000169228	5	177301197	177303719
ENST00000303375	67.909062	-2.65382694	0.46697	-5.683083	1.32E-08	6.28E-07	ENSG00000011028	17	62627669	62693597
ENST00000303383	477.496	-2.01362218	0.217114	-9.274504	1.78E-20	6.89E-18	ENSG00000171241	16	46578590	46621379
ENST00000303562	156.72004	-1.060878696	0.371337	-2.856918	0.004277768	0.026536647	ENSG00000170345	14	75278827	75282230
ENST00000303746	198.72007	-2.302508572	0.280494	-8.208774	2.23E-16	5.28E-14	ENSG00000131650	16	2964274	2968380
ENST00000303892	11.219681	6.912554508	1.68378	4.105378	4.04E-05	0.000630018	ENSG00000213760	6	31544443	31546608
ENST00000303910	2292.2955	-1.315040893	0.170361	-7.719124	1.17E-14	2.18E-12	ENSG00000277075	6	26216920	26217437
ENST00000303921	41.216919	-4.53147959	0.716448	-6.324924	2.53E-10	1.96E-08	ENSG00000170775	7	124743884	124765792

NYAP1	protein_coding	Yes	NM_173564.4	108
NUDT21	protein_coding	Yes	NM_007006.3	108
COL6A2	protein_coding	Yes	NM_001849.4	108
GPRC5B	protein_coding	Yes	NM_016235.3	108
FN3K	protein_coding	Yes	NM_022158.4	108
AXL	protein_coding	Yes	NM_021913.5	108
CDC42EP5	protein_coding	Yes	NM_145057.4	108
LY6D	protein_coding	Yes	NM_003695.3	108
SLC39A4	protein_coding	Yes	NM_130849.4	108
SLC43A2	protein_coding	Yes	NM_152346.3	108
RCOR2	protein_coding	Yes	NM_173587.4	108
ZNF560	protein_coding	Yes	NM_152476.3	108
METTL26	protein_coding	Yes	NM_032366.5	108
SLC22A11	protein_coding	Yes	NM_018484.4	108
PBK	protein_coding	Yes	NM_018492.4	108
PCSK9	protein_coding	Yes	NM_174936.4	108
GAA	protein_coding	Yes	NM_000152.5	108
ELOVL6	protein_coding	Yes	NM_024090.3	108
ING2	protein_coding	Yes	NM_001564.4	108
CKAP2L	protein_coding	Yes	NM_152515.5	108
KYAT1	protein_coding	Yes	NM_004059.5	108
SCAMP3	protein_coding	Yes	NM_005698.4	108
ZNF561	protein_coding	Yes	NM_152289.3	108
CTRB2	protein_coding	Yes	NM_001025200.4	108
SHLD1	protein_coding	Yes	NM_152504.4	108
POP7	protein_coding	Yes	NM_005837.3	108
RAB24	protein_coding	Yes	NM_001031677.4	108
MRC2	protein_coding	Yes	NM_006039.5	108
SHCBP1	protein_coding	Yes	NM_024745.5	108
FOS	protein_coding	Yes	NM_005252.4	108
KREMEN2	protein_coding	Yes	NM_172229.3	108
ATP6V1G2	protein_coding	Yes	NM_130463.4	108
H2AC8	protein_coding	Yes	NM_021052.4	108
GPR37	protein_coding	Yes	NM_005302.5	108

ENST00000304116	78.971208	1.23055693	0.383663	3.20739	0.001339452	0.010712455	ENSG00000196357	19	36182275	36214665
ENST00000304218	5508.1165	-1.483268248	0.162398	-9.133524	6.63E-20	2.43E-17	ENSG00000168298	6	26156328	26157115
ENST00000304222	207.05728	-2.014130839	0.288837	-6.973255	3.10E-12	3.53E-10	ENSG00000170425	17	15945129	15975746
ENST00000304312	343.28528	-1.24432726	0.243529	-5.10956	3.23E-07	1.02E-05	ENSG00000169020	4	672435	674276
ENST00000304385	335.60461	-1.742255137	0.285095	-6.111136	9.89E-10	6.48E-08	ENSG00000170006	4	152618627	152679997
ENST00000304414	6814.9044	-1.300237273	0.165646	-7.84949	4.18E-15	8.30E-13	ENSG00000170540	16	18791668	18801549
ENST00000304494	74.096952	-1.012607223	0.391395	-2.587172	0.009676737	0.049592901	ENSG00000147889	9	21967751	21974857
ENST00000304552	74.893842	8.202821258	1.47897	5.546305	2.92E-08	1.26E-06	ENSG00000172215	3	45943463	45948351
ENST00000304567	1729.8058	-1.412208351	0.204333	-6.911297	4.80E-12	5.28E-10	ENSG00000171848	2	10122738	10131414
ENST00000304611	85.259412	1.296435654	0.384501	3.371734	0.000746965	0.00676491	ENSG00000124587	6	42963864	42979181
ENST00000304661	693.72107	1.051828817	0.20382	5.160567	2.46E-07	8.08E-06	ENSG00000171155	X	120625673	120630054
ENST00000304698	2131.1182	-1.235277513	0.2166	-5.703026	1.18E-08	5.72E-07	ENSG00000144369	2	186694059	186765959
ENST00000304874	139.97127	-1.007745196	0.301791	-3.339212	0.000840165	0.007414614	ENSG00000126522	7	66075818	66093576
ENST00000304952	37.227042	-1.885092529	0.614083	-3.069767	0.002142257	0.015493194	ENSG00000188290	1	998963	1000097
ENST00000305428	139.81915	1.225926144	0.314164	3.902191	9.53E-05	0.001279497	ENSG00000169330	15	79432335	79472304
ENST00000305544	1092.4827	1.039610713	0.272131	3.820253	0.000133315	0.001695195	ENSG00000172037	3	49121113	49133050
ENST00000305784	445.35559	-1.19431109	0.27195	-4.39166	1.12E-05	0.000214681	ENSG00000168393	2	241675746	241686815
ENST00000305798	109.82227	-1.729375964	0.339052	-5.100619	3.39E-07	1.06E-05	ENSG00000168785	4	98470366	98658611
ENST00000305883	84.056913	-1.121220984	0.372005	-3.013997	0.002578302	0.017938535	ENSG00000172059	2	10043549	10054836
ENST00000305997	2913.6938	-1.172558386	0.218839	-5.358075	8.41E-08	3.22E-06	ENSG00000143797	2	8852689	9003684
ENST00000306072	106.49978	2.061300347	0.363371	5.672709	1.41E-08	6.60E-07	ENSG00000172183	15	88639015	88656483
ENST00000306121	19.972656	-3.955138221	0.923818	-4.281294	1.86E-05	0.000328684	ENSG00000162627	1	98661720	98760500
ENST00000306125	2145.754	1.285446178	0.178738	7.191799	6.39E-13	8.33E-11	ENSG00000172053	3	49095931	49104757
ENST00000306243	69.935421	-1.549670974	0.403474	-3.84082	0.000122624	0.001582089	ENSG00000169105	15	40470983	40473158
ENST00000306357	149.93613	-1.126818061	0.305638	-3.686769	0.000227119	0.002622979	ENSG00000170310	17	9250470	9575820
ENST00000306390	15.595267	-7.437799993	1.628917	-4.566102	4.97E-06	0.000107524	ENSG00000171236	19	4536401	4540036
ENST00000306406	4.8058312	-5.738655542	1.956982	-2.932401	0.003363524	0.021987476	ENSG00000171227	2	119431853	119438504
ENST00000306593	57.310775	1.280023386	0.43501	2.942512	0.003255612	0.021474554	ENSG00000242419	5	141485029	141512975
ENST00000306601	499.36055	1.203950186	0.228933	5.258952	1.45E-07	5.13E-06	ENSG00000171163	1	248850007	248859085
ENST00000306721	610.06426	-1.261320243	0.2	-6.306597	2.85E-10	2.17E-08	ENSG00000144354	2	173354871	173368997
ENST00000306796	417.83852	-1.441215194	0.2183	-6.602	4.06E-11	3.72E-09	ENSG00000169718	17	82057505	82065804
ENST00000306897	146.35347	-1.276861246	0.300938	-4.242934	2.21E-05	0.000378311	ENSG00000169750	17	82031677	82034204
ENST00000307360	594.80637	1.239620718	0.217607	5.696601	1.22E-08	5.89E-07	ENSG00000250120	5	140855896	141012347
ENST00000307428	235.62462	3.345356367	0.331567	10.08953	6.15E-24	4.09E-21	ENSG00000169116	4	74933115	75050113

ZNF565	protein_coding	Yes	NM_152477.5	108
H1-4	protein_coding	Yes	NM_005321.3	108
ADORA2B	protein_coding	Yes	NM_000676.4	108
ATP5ME	protein_coding	Yes	NM_007100.4	108
TMEM154	protein_coding	Yes	NM_152680.3	108
ARL6IP1	protein_coding	Yes	NM_015161.3	108
CDKN2A	protein_coding	Yes	NM_000077.5	108
CXCR6	protein_coding	Yes	NM_006564.2	108
RRM2	protein_coding	Yes	NM_001034.4	108
PEX6	protein_coding	Yes	NM_000287.4	108
C1GALT1C1	protein_coding	Yes	NM_001011551.3	108
FAM171B	protein_coding	Yes	NM_177454.4	108
ASL	protein_coding	Yes	NM_000048.4	108
HES4	protein_coding	Yes	NM_021170.4	108
MINAR1	protein_coding	Yes	NM_015206.3	108
LAMB2	protein_coding	Yes	NM_002292.4	108
DTYMK	protein_coding	Yes	NM_012145.4	108
TSPAN5	protein_coding	Yes	NM_005723.4	108
KLF11	protein_coding	Yes	NM_003597.5	108
MBOAT2	protein_coding	Yes	NM_138799.4	108
ISG20	protein_coding	Yes	NM_002201.6	108
SNX7	protein_coding	Yes	NM_015976.5	108
QARS1	protein_coding	Yes	NM_005051.3	108
CHST14	protein_coding	Yes	NM_130468.4	108
STX8	protein_coding	Yes	NM_004853.3	108
LRG1	protein_coding	Yes	NM_052972.3	108
TMEM37	protein_coding	Yes	NM_183240.3	108
PCDHGC4	protein_coding	Yes	NM_018928.3	108
ZNF692	protein_coding	Yes	NM_017865.4	108
CDCA7	protein_coding	Yes	NM_031942.5	108
DUS1L	protein_coding	Yes	NM_022156.5	108
RAC3	protein_coding	Yes	NM_005052.3	108
PCDHA10	protein_coding	Yes	NM_018901.4	108
PARM1	protein_coding	Yes	NM_015393.4	108

ENST00000307439	33.946149	-1.67450214	0.61174	-2.737276	0.006195033	0.035390799	ENSG00000169435	4	73571549	73620436
ENST00000307465	50.84265	2.515599166	0.502245	5.008712	5.48E-07	1.61E-05	ENSG00000138769	4	75576495	75630528
ENST00000307630	1677.9562	-1.116244519	0.174507	-6.396576	1.59E-10	1.30E-08	ENSG00000170027	7	76326798	76358991
ENST00000307741	585.8946	-1.533299653	0.204065	-7.513777	5.74E-14	9.13E-12	ENSG00000172009	19	2785502	2815807
ENST00000307998	28.276319	2.016331102	0.641956	3.140917	0.001684199	0.012821062	ENSG00000172638	11	65866440	65872800
ENST00000308108	370.61391	-2.55419255	0.245971	-10.38411	2.93E-25	2.22E-22	ENSG00000175305	8	94880223	94895201
ENST00000308177	56.615783	1.223437224	0.436892	2.800319	0.005105215	0.030495749	ENSG00000240184	5	141475965	141512975
ENST00000308278	180.60008	-1.238043582	0.309423	-4.001132	6.30E-05	0.000910022	ENSG00000167695	17	732595	742968
ENST00000308418	303.8435	-1.518704839	0.303454	-5.004727	5.59E-07	1.64E-05	ENSG00000172922	11	65717672	65720798
ENST00000308423	36.345261	1.860972161	0.567548	3.278967	0.001041878	0.008755237	ENSG00000131471	17	42851198	42858124
ENST00000308521	240.90794	1.284640878	0.274628	4.677746	2.90E-06	6.76E-05	ENSG00000128394	22	39040863	39055972
ENST00000308742	693.19335	-1.594521094	0.214976	-7.417215	1.20E-13	1.80E-11	ENSG00000175548	12	38316773	38329721
ENST00000308874	150.99425	-1.285414446	0.300101	-4.283267	1.84E-05	0.000326313	ENSG00000172889	9	136662915	136672678
ENST00000308919	12.573811	-2.671603873	0.961297	-2.779167	0.005449852	0.032012081	ENSG00000073067	7	983180	989640
ENST00000309241	59.645351	-1.562459239	0.435114	-3.590923	0.000329509	0.003536175	ENSG00000155846	5	149730309	149855022
ENST00000309285	16.550599	7.471409325	1.618025	4.617611	3.88E-06	8.71E-05	ENSG00000173578	3	46017006	46027483
ENST00000309602	445.75418	1.116385073	0.277972	4.016179	5.91E-05	0.000863919	ENSG00000173621	11	66857063	66860475
ENST00000309733	175.59957	-2.081691192	0.293985	-7.080937	1.43E-12	1.75E-10	ENSG00000174749	4	112145453	112195256
ENST00000309739	9.4896053	3.056320542	1.175404	2.60023	0.009316122	0.048180506	ENSG00000172602	12	48857144	48865870
ENST00000309755	43.621472	2.02147939	0.511283	3.953739	7.69E-05	0.001073649	ENSG00000146021	5	137617499	137736089
ENST00000309758	9.3288002	3.489856355	1.239461	2.815624	0.004868254	0.029397976	ENSG00000173110	1	161524539	161526894
ENST00000309868	136.00844	-1.449950865	0.317325	-4.569289	4.89E-06	0.000106144	ENSG00000173210	5	149141492	149260542
ENST00000309880	48.651423	1.944219225	0.525384	3.700566	0.00021512	0.002509148	ENSG00000173825	11	65354750	65357613
ENST00000310054	59.374731	-1.922828491	0.456358	-4.21342	2.52E-05	0.000422493	ENSG00000167600	19	41193218	41207539
ENST00000310144	838.64656	1.148968767	0.191413	6.002571	1.94E-09	1.16E-07	ENSG00000087191	17	63827430	63832019
ENST00000310389	4.4345708	5.571739292	1.995746	2.791808	0.005241441	0.031089836	ENSG00000175414	5	176365486	176381909
ENST00000310581	8.5409023	-4.042135823	1.40572	-2.875491	0.004033993	0.025401786	ENSG00000164362	5	1253166	1295068
ENST00000310614	287.46282	-2.375052604	0.251092	-9.458888	3.11E-21	1.31E-18	ENSG00000175581	11	73787873	73865133
ENST00000310803	25.567605	-3.602468789	0.783846	-4.595887	4.31E-06	9.52E-05	ENSG00000172575	15	38488102	38564814
ENST00000310836	23.522561	-8.029847206	1.566357	-5.126447	2.95E-07	9.44E-06	ENSG00000174607	4	114598806	114678225
ENST00000311066	247.59561	1.262752553	0.274338	4.602908	4.17E-06	9.25E-05	ENSG00000116731	1	13700187	13825079
ENST00000311083	22.039769	-2.840481888	0.75743	-3.75016	0.000176722	0.002138155	ENSG00000175183	12	76858708	76879019
ENST00000311124	137.87882	-1.108659732	0.330746	-3.352001	0.000802297	0.007149973	ENSG00000173638	21	45512564	45542440
ENST00000311412	65.66841	-1.955991326	0.423793	-4.615437	3.92E-06	8.78E-05	ENSG00000173083	4	83292460	83334848

RASSF6	protein_coding	Yes	NM_177532.5	108
CDKL2	protein_coding	Yes	NM_001330724.2	108
YWHAG	protein_coding	Yes	NM_012479.4	108
THOP1	protein_coding	Yes	NM_003249.5	108
EFEMP2	protein_coding	Yes	NM_016938.5	108
CCNE2	protein_coding	Yes	NM_057749.3	108
PCDHGC3	protein_coding	Yes	NM_002588.4	108
TLCD3A	protein_coding	Yes	NM_024792.3	108
RNASEH2C	protein_coding	Yes	NM_032193.4	108
AOC3	protein_coding	Yes	NM_003734.4	108
APOBEC3F	protein_coding	Yes	NM_145298.6	108
ALG10B	protein_coding	Yes	NM_001013620.4	108
EGFL7	protein_coding	Yes	NM_016215.5	108
CYP2W1	protein_coding	Yes	NM_017781.3	108
PPARGC1B	protein_coding	Yes	NM_133263.4	108
XCR1	protein_coding	Yes	NM_001024644.2	108
LRFN4	protein_coding	Yes	NM_024036.5	108
FAM241A	protein_coding	Yes	NM_152400.3	108
RND1	protein_coding	Yes	NM_014470.4	108
KLHL3	protein_coding	Yes	NM_017415.3	108
HSPA6	protein_coding	Yes	NM_002155.5	108
ABLIM3	protein_coding	Yes	NM_014945.5	108
TIGD3	protein_coding	Yes	NM_145719.3	108
CYP2S1	protein_coding	Yes	NM_030622.8	108
PSMC5	protein_coding	Yes	NM_002805.6	108
ARL10	protein_coding	Yes	NM_173664.6	108
TERT	protein_coding	Yes	NM_198253.3	108
MRPL48	protein_coding	Yes	NM_016055.6	108
RASGRP1	protein_coding	Yes	NM_005739.4	108
UGT8	protein_coding	Yes	NM_001128174.3	108
PRDM2	protein_coding	Yes	NM_001393986.1	108
CSRP2	protein_coding	Yes	NM_001321.3	108
SLC19A1	protein_coding	Yes	NM_194255.4	108
HPSE	protein_coding	Yes	NM_001098540.3	108

ENST00000311502	972.70208	-1.142044226	0.200827	-5.686698	1.30E-08	6.17E-07	ENSG00000196642	9	136807947	136841187
ENST00000311812	8.0721117	6.437066362	1.767079	3.642772	0.000269718	0.003019315	ENSG00000174226	8	100572888	100649665
ENST00000312108	558.95413	1.656310862	0.225763	7.336512	2.19E-13	3.15E-11	ENSG00000205560	22	50568860	50578427
ENST00000312134	195.61847	-1.682217789	0.311915	-5.39319	6.92E-08	2.72E-06	ENSG00000175315	11	66012007	66013505
ENST00000312251	166.91072	1.25586017	0.288395	4.35465	1.33E-05	0.00024833	ENSG00000103174	16	5024843	5033935
ENST00000312428	41.857419	1.500423008	0.514536	2.916071	0.003544702	0.022933802	ENSG00000118997	2	195737702	196068837
ENST00000312475	267.04066	-1.033222889	0.302037	-3.420846	0.000624267	0.005870263	ENSG00000259494	15	88459477	88467388
ENST00000312499	205.23699	-1.508230382	0.295222	-5.108796	3.24E-07	1.02E-05	ENSG00000175643	16	11345458	11351760
ENST00000312655	149.61169	2.226731522	0.395732	5.626868	1.84E-08	8.32E-07	ENSG00000175155	17	59331654	59401729
ENST00000313093	129.35653	-1.384127354	0.327217	-4.229998	2.34E-05	0.000396367	ENSG00000180448	19	1067166	1086628
ENST00000313236	24.704439	1.700131763	0.657901	2.584176	0.009761191	0.049897319	ENSG00000149582	11	118531191	118535829
ENST00000313250	46.569404	-1.731994542	0.48973	-3.536631	0.000405266	0.004175423	ENSG00000157326	14	23953769	23969279
ENST00000313546	252.42316	-1.164761689	0.250211	-4.655109	3.24E-06	7.44E-05	ENSG00000139323	12	89419717	89526047
ENST00000313578	86.817353	3.233046706	0.4211	7.677618	1.62E-14	2.94E-12	ENSG00000178301	11	77589952	77610356
ENST00000313766	34.080918	-4.037360157	0.716324	-5.636223	1.74E-08	7.94E-07	ENSG00000177706	7	192570	260772
ENST00000314032	50.976739	2.149626267	0.480353	4.475095	7.64E-06	0.000154776	ENSG00000175564	11	74000276	74009085
ENST00000314128	1719.2735	1.256555962	0.221036	5.684851	1.31E-08	6.22E-07	ENSG00000170581	12	56341596	56360107
ENST00000314256	356.9492	1.111163408	0.284503	3.905631	9.40E-05	0.001264214	ENSG00000177728	17	75456634	75500452
ENST00000314727	29.614164	-2.805036865	0.664588	-4.220718	2.44E-05	0.000410893	ENSG00000177570	8	118377983	118621963
ENST00000314759	407.19143	-1.259487116	0.220088	-5.722654	1.05E-08	5.17E-07	ENSG00000165480	13	21153594	21176552
ENST00000315087	7.11972	4.755060134	1.783421	2.666258	0.00767009	0.0416654	ENSG00000101638	18	46667820	46757053
ENST00000315127	382.75031	1.006031742	0.230526	4.364074	1.28E-05	0.000239061	ENSG00000179151	15	74630557	74696024
ENST00000315323	264.62027	-1.150546216	0.255863	-4.496728	6.90E-06	0.000142186	ENSG00000180340	17	44557483	44561262
ENST00000315367	2285.2432	-1.073963852	0.20341	-5.279797	1.29E-07	4.64E-06	ENSG00000175895	8	95133784	95156685
ENST00000315392	62.676635	2.676898324	0.573705	4.665981	3.07E-06	7.10E-05	ENSG00000161381	17	39063312	39151637
ENST00000315453	11.269553	2.682549864	1.019053	2.632395	0.008478532	0.044989886	ENSG00000180658	6	131699643	131701401
ENST00000315567	516.65414	-1.517782474	0.229871	-6.602751	4.04E-11	3.70E-09	ENSG00000169288	4	77862829	77952785
ENST00000315580	651.20006	-1.032642551	0.215215	-4.798197	1.60E-06	4.05E-05	ENSG00000182196	12	122980680	122982909
ENST00000315707	41.966138	1.698611786	0.509811	3.331846	0.000862721	0.007575952	ENSG00000178977	17	8220623	8224043
ENST00000315717	2779.8588	-1.058136826	0.167212	-6.328098	2.48E-10	1.92E-08	ENSG00000163466	2	218217188	218254348
ENST00000316218	119.54929	-1.052950495	0.320333	-3.28705	0.001012428	0.008564897	ENSG00000065485	3	123067024	123162104
ENST00000316308	399.60091	1.077286356	0.219752	4.902284	9.47E-07	2.58E-05	ENSG00000113240	5	178602663	178627050
ENST00000316341	597.88077	-1.09006816	0.208103	-5.238117	1.62E-07	5.65E-06	ENSG00000124067	16	67943473	67968637
ENST00000316509	62.103375	1.602149222	0.525821	3.046946	0.002311789	0.016444605	ENSG00000220205	17	8159148	8162948

RABL6	protein_coding	Yes	NM_024718.5	108
SNX31	protein_coding	Yes	NM_152628.4	108
CPT1B	protein_coding	Yes	NM_152246.3	108
CST6	protein_coding	Yes	NM_001323.4	108
NAGPA	protein_coding	Yes	NM_016256.4	108
DNAH7	protein_coding	Yes	NM_018897.3	108
MRPL46	protein_coding	Yes	NM_022163.4	108
RMI2	protein_coding	Yes	NM_152308.3	108
YPEL2	protein_coding	Yes	NM_001005404.4	108
ARHGAP45	protein_coding	Yes	NM_012292.5	108
TMEM25	protein_coding	Yes	NM_032780.4	108
DHRS4	protein_coding	Yes	NM_021004.4	108
POC1B	protein_coding	Yes	NM_172240.3	108
AQP11	protein_coding	Yes	NM_173039.3	108
FAM20C	protein_coding	Yes	NM_020223.4	108
UCP3	protein_coding	Yes	NM_003356.4	108
STAT2	protein_coding	Yes	NM_005419.4	108
TMEM94	protein_coding	Yes	NM_014738.6	108
SAMD12	protein_coding	Yes	NM_207506.3	108
SKA3	protein_coding	Yes	NM_145061.6	108
ST8SIA5	protein_coding	Yes	NM_013305.6	108
EDC3	protein_coding	Yes	NM_025083.5	108
FZD2	protein_coding	Yes	NM_001466.4	108
PLEKHF2	protein_coding	Yes	NM_024613.4	108
PLXDC1	protein_coding	Yes	NM_020405.5	108
OR2A4	protein_coding	Yes	NM_030908.2	108
MRPL1	protein_coding	Yes	NM_020236.4	108
ARL6IP4	protein_coding	Yes	NM_018694.4	108
LINC00324	lncRNA	Yes	-	108
ARPC2	protein_coding	Yes	NM_152862.3	108
PDIA5	protein_coding	Yes	NM_006810.4	108
CLK4	protein_coding	Yes	NM_020666.3	108
SLC12A4	protein_coding	Yes	NM_005072.5	108
VAMP2	protein_coding	Yes	NM_014232.3	108

ENST00000316586	845.78198	-1.309153165	0.191413	-6.839427	7.95E-12	8.35E-10	ENSG00000115694	2	241492669	241508584
ENST00000316660	148.70434	-1.318727967	0.311535	-4.233	2.31E-05	0.000392069	ENSG00000141682	18	59899995	59904305
ENST00000316724	87.823492	-1.158972364	0.389301	-2.977062	0.002910251	0.019683776	ENSG00000136717	2	127048031	127107154
ENST00000316803	5.489001	5.877568208	1.920128	3.061029	0.002205778	0.015834621	ENSG00000157782	12	120640625	120667324
ENST00000316881	2452.0203	1.113715576	0.190531	5.845325	5.06E-09	2.73E-07	ENSG00000121060	17	56887908	56914049
ENST00000316963	3185.0664	-1.290338517	0.205701	-6.272876	3.54E-10	2.61E-08	ENSG00000120992	8	54046366	54101947
ENST00000317012	75.778102	-2.503821774	0.521951	-4.797039	1.61E-06	4.07E-05	ENSG00000177494	3	111592899	111595346
ENST00000317114	35.152969	1.506532999	0.554921	2.71486	0.006630375	0.037370077	ENSG00000177337	18	3594098	3598352
ENST00000317122	38.345697	1.957036004	0.539038	3.630612	0.00028275	0.003137321	ENSG00000179743	1	15834473	15848147
ENST00000317276	99.076688	1.453681068	0.383519	3.790371	0.000150422	0.001873348	ENSG00000179094	17	8140471	8152404
ENST00000317479	307.32696	-2.220173136	0.265705	-8.355769	6.50E-17	1.64E-14	ENSG00000176894	12	132687586	132704985
ENST00000317551	246.9437	1.003276761	0.255174	3.93174	8.43E-05	0.001157853	ENSG00000139624	12	50129288	50167369
ENST00000317578	143.22415	1.018028625	0.326372	3.119225	0.001813275	0.013589843	ENSG00000177045	19	45764784	45769252
ENST00000317811	183.9575	-1.813259619	0.334352	-5.423201	5.85E-08	2.36E-06	ENSG00000179431	11	35618459	35620865
ENST00000317827	47.624452	3.3320365	0.556147	5.991289	2.08E-09	1.24E-07	ENSG00000147526	8	38787235	38853028
ENST00000318121	694.75045	-1.065950308	0.26391	-4.039063	5.37E-05	0.00079945	ENSG00000092853	1	35732111	35769978
ENST00000318129	150.19083	-2.232793419	0.310575	-7.18923	6.52E-13	8.47E-11	ENSG00000181938	16	58392470	58406147
ENST00000318222	9.0181169	6.593459268	1.767306	3.730797	0.000190875	0.002279001	ENSG00000107593	10	100288148	100330228
ENST00000318357	73.814897	1.197584684	0.416876	2.872758	0.004069057	0.025575037	ENSG00000163126	2	96837911	96844021
ENST00000318407	370.39021	-1.041461278	0.228893	-4.549993	5.36E-06	0.000115049	ENSG00000176720	2	241558744	241574131
ENST00000318522	1920.2381	-1.207824375	0.184253	-6.555253	5.55E-11	4.95E-09	ENSG00000143924	2	42169352	42332548
ENST00000318579	16.824658	-5.063319504	1.248046	-4.056996	4.97E-05	0.000750324	ENSG00000005102	17	43640388	43661922
ENST00000318673	195.45797	1.19765482	0.275444	4.34809	1.37E-05	0.000254813	ENSG00000280537	2	219075972	219169987
ENST00000318911	1812.0952	-1.346361889	0.200896	-6.701802	2.06E-11	2.01E-09	ENSG00000179091	8	144095075	144097525
ENST00000318948	402.66563	-1.241575484	0.286585	-4.332313	1.48E-05	0.000270782	ENSG00000180530	21	14961234	15065000
ENST00000319211	95.851637	-1.044070913	0.347918	-3.000914	0.002691709	0.018538844	ENSG00000181104	5	76716125	76735770
ENST00000319286	74.997464	-1.288458391	0.388346	-3.317813	0.000907253	0.007879388	ENSG00000180938	8	124973294	124979389
ENST00000319349	200.05823	-2.21042816	0.399271	-5.536159	3.09E-08	1.33E-06	ENSG00000175832	17	43527845	43546340
ENST00000319420	11.266012	-5.488038059	1.68396	-3.259007	0.00111803	0.009280548	ENSG00000180730	13	26044596	26052016
ENST00000319518	75.705382	-1.043798059	0.385745	-2.705931	0.00681132	0.038152509	ENSG00000178773	16	89575757	89597246
ENST00000319636	6.8238421	4.703472476	1.797799	2.616239	0.008890432	0.04646775	ENSG00000185177	7	57117675	57132436
ENST00000319694	44.200493	-2.572639162	0.533387	-4.823217	1.41E-06	3.64E-05	ENSG00000176532	7	29563834	29567293
ENST00000319928	152.80366	1.038560781	0.304185	3.414238	0.000639606	0.005985175	ENSG00000142961	1	46607718	46616811
ENST00000320078	3.9197703	5.394631232	2.054307	2.626011	0.008639213	0.045513691	ENSG00000243130	19	43007655	43026469

STK25	protein_coding	Yes	NM_001271977.2	108
PMAIP1	protein_coding	Yes	NM_021127.3	108
BIN1	protein_coding	Yes	NM_139343.3	108
CABP1	protein_coding	Yes	NM_001033677.2	108
TRIM25	protein_coding	Yes	NM_005082.5	108
LYPLA1	protein_coding	Yes	NM_006330.4	108
ZBED2	protein_coding	Yes	NM_024508.5	108
DLGAP1-AS1	lncRNA	Yes	-	108
SPEN-AS1	lncRNA	Yes	-	108
PER1	protein_coding	Yes	NM_002616.3	108
PXMP2	protein_coding	Yes	NM_018663.3	108
CERS5	protein_coding	Yes	NM_147190.5	108
SIX5	protein_coding	Yes	NM_175875.5	108
FJX1	protein_coding	Yes	NM_014344.4	108
TACC1	protein_coding	Yes	NM_006283.3	108
CLSPN	protein_coding	Yes	NM_022111.4	108
GINS3	protein_coding	Yes	NM_022770.4	108
PKD2L1	protein_coding	Yes	NM_016112.3	108
ANKRD23	protein_coding	Yes	NM_144994.8	108
BOK	protein_coding	Yes	NM_032515.5	108
EML4	protein_coding	Yes	NM_019063.5	108
MEOX1	protein_coding	Yes	NM_004527.4	108
-	protein_coding	Yes	-	108
CYC1	protein_coding	Yes	NM_001916.5	108
NRIP1	protein_coding	Yes	NM_003489.4	108
F2R	protein_coding	Yes	NM_001992.5	108
ZNF572	protein_coding	Yes	NM_152412.3	108
ETV4	protein_coding	Yes	NM_001079675.5	108
SHISA2	protein_coding	Yes	NM_001007538.2	108
CPNE7	protein_coding	Yes	NM_153636.3	108
ZNF479	protein_coding	Yes	NM_001370129.2	108
PRR15	protein_coding	Yes	NM_175887.3	108
MOB3C	protein_coding	Yes	NM_201403.3	108
PSG11	protein_coding	Yes	NM_002785.3	108

ENST00000320248	182.99858	-1.778161518	0.280939	-6.329353	2.46E-10	1.91E-08	ENSG00000176595	8	1973676	2006936
ENST00000320892	96.318649	1.274615614	0.453346	2.811573	0.004929991	0.029683751	ENSG00000151692	2	6917411	7044179
ENST00000321297	88.798077	1.283495935	0.458985	2.796376	0.005167917	0.030780656	ENSG00000168010	11	72814410	72829635
ENST00000321331	708.41232	-1.250066822	0.212297	-5.888292	3.90E-09	2.16E-07	ENSG00000181061	3	42782907	42804490
ENST00000321418	4.9493713	5.729481077	1.950121	2.938013	0.003303235	0.021714444	ENSG00000178715	1	15828231	15828669
ENST00000321562	712.52713	-2.051416702	0.19864	-10.3273	5.30E-25	3.92E-22	ENSG00000141756	17	41813003	41823213
ENST00000321826	56.185127	1.308003007	0.44184	2.960357	0.003072829	0.020555347	ENSG00000244165	19	10111692	10115372
ENST00000321854	90.299214	1.643096492	0.394122	4.169009	3.06E-05	0.000497987	ENSG00000181513	17	45135686	45144176
ENST00000322048	237.06694	1.032360754	0.25724	4.013224	5.99E-05	0.000872514	ENSG00000067836	16	4796967	4802633
ENST00000322165	39.390043	2.587609745	0.597644	4.329684	1.49E-05	0.000273408	ENSG00000025423	12	56763323	56787790
ENST00000322507	303.50877	-1.789452547	0.353599	-5.060681	4.18E-07	1.28E-05	ENSG00000111799	6	75084325	75206053
ENST00000322623	875.24124	-1.033680743	0.215973	-4.786162	1.70E-06	4.28E-05	ENSG00000175792	3	128080809	128123812
ENST00000322886	217.96519	-1.623458025	0.267829	-6.061542	1.35E-09	8.50E-08	ENSG00000165891	12	77021250	77065569
ENST00000322927	601.35048	1.224393399	0.216633	5.651929	1.59E-08	7.32E-07	ENSG00000198026	20	45948659	45972203
ENST00000322954	895.4495	-1.60976411	0.212094	-7.589864	3.20E-14	5.43E-12	ENSG00000137831	15	70654553	70763558
ENST00000323061	57.613568	-1.203492857	0.445575	-2.700988	0.006913392	0.03857927	ENSG00000177432	4	88695912	88697829
ENST00000323169	65.472974	1.12604411	0.417082	2.699815	0.006937813	0.038675899	ENSG00000177599	19	11798522	11808503
ENST00000323274	160.01352	-1.094672105	0.344968	-3.173258	0.001507384	0.011743692	ENSG00000176890	18	657652	673578
ENST00000323372	11.828569	3.424688023	1.102395	3.106588	0.001892601	0.014051008	ENSG00000179761	17	29043140	29057216
ENST00000323380	18.373625	2.083449658	0.802299	2.596849	0.009408317	0.048555159	ENSG00000179141	13	29476514	29490105
ENST00000323468	931.35291	-1.259192205	0.187514	-6.715184	1.88E-11	1.85E-09	ENSG00000178202	11	108472115	108498384
ENST00000323662	86.307352	1.711275673	0.433291	3.949486	7.83E-05	0.00109061	ENSG00000179698	8	144107738	144118328
ENST00000323760	464.2649	-1.240624347	0.212955	-5.825772	5.68E-09	3.03E-07	ENSG00000158402	5	138285268	138331804
ENST00000323777	7.9976241	6.425211053	1.773124	3.623668	0.000290455	0.003207862	ENSG00000176160	17	58420166	58488408
ENST00000323853	3170.6864	1.06940233	0.170911	6.25707	3.92E-10	2.84E-08	ENSG00000144028	2	96274337	96305546
ENST00000323944	389.16733	-1.510980125	0.245839	-6.146212	7.94E-10	5.30E-08	ENSG00000168952	14	24809453	25050147
ENST00000324093	251.81141	-2.844777513	0.27842	-10.21758	1.65E-24	1.18E-21	ENSG00000004399	3	129555213	129606676
ENST00000324219	1030.0958	-1.335951672	0.195565	-6.831245	8.42E-12	8.81E-10	ENSG00000071054	2	101697706	101894690
ENST00000324348	84.480718	1.55690479	0.38524	4.041387	5.31E-05	0.000792729	ENSG00000179859	17	7913338	7916289
ENST00000324453	641.84869	1.800416314	0.285611	6.30373	2.91E-10	2.21E-08	ENSG00000180354	7	30134985	30162762
ENST00000324607	122.26709	-1.345808257	0.322835	-4.168716	3.06E-05	0.000498469	ENSG00000172197	6	20099683	20212469
ENST00000324765	119.61374	-2.392461608	0.359529	-6.65443	2.84E-11	2.69E-09	ENSG00000147202	X	96684841	97604997
ENST00000324787	186.32583	-1.028311312	0.276979	-3.712595	0.000205145	0.002413269	ENSG00000090263	7	141002609	141014952
ENST00000324873	13.306042	-5.733922045	1.640922	-3.49433	0.000475254	0.004737336	ENSG00000176046	16	28532707	28538974

KBTBD11	protein_coding	Yes	NM_014867.3	108
RNF144A	protein_coding	Yes	NM_014746.6	108
ATG16L2	protein_coding	Yes	NM_033388.2	108
HIGD1A	protein_coding	Yes	NM_014056.4	108
-	essed_pseudog	Yes	-	108
FKBP10	protein_coding	Yes	NM_021939.4	108
P2RY11	protein_coding	Yes	NM_002566.5	108
ACBD4	protein_coding	Yes	NM_001135705.3	108
ROGDI	protein_coding	Yes	NM_024589.3	108
HSD17B6	protein_coding	Yes	NM_003725.4	108
COL12A1	protein_coding	Yes	NM_004370.6	108
RUVBL1	protein_coding	Yes	NM_003707.3	108
E2F7	protein_coding	Yes	NM_203394.3	108
ZNF335	protein_coding	Yes	NM_022095.4	108
UACA	protein_coding	Yes	NM_018003.4	108
NAP1L5	protein_coding	Yes	NM_153757.4	108
ZNF491	protein_coding	Yes	NM_152356.4	108
TYMS	protein_coding	Yes	NM_001071.4	108
PIPOX	protein_coding	Yes	NM_016518.3	108
MTUS2-AS1	lncRNA	Yes	-	108
POGLUT3	protein_coding	Yes	NM_153705.5	108
WDR97	protein_coding	Yes	NM_001316309.2	108
CDC25C	protein_coding	Yes	NM_001790.5	108
HSF5	protein_coding	Yes	NM_001080439.3	108
SNRNP200	protein_coding	Yes	NM_014014.5	108
STXBP6	protein_coding	Yes	NM_001394410.1	108
PLXND1	protein_coding	Yes	NM_015103.3	108
MAP4K4	protein_coding	Yes	NM_001395002.1	108
RNF227	protein_coding	Yes	NM_001358699.2	108
MTURN	protein_coding	Yes	NM_152793.3	108
MBOAT1	protein_coding	Yes	NM_001080480.3	108
DIAPH2	protein_coding	Yes	NM_006729.5	108
MRPS33	protein_coding	Yes	NM_053035.3	108
NUPR1	protein_coding	Yes	NM_012385.3	108

ENST00000324894	135.61208	-1.457243924	0.322471	-4.518988	6.21E-06	0.000130139	ENSG00000130299	19	17337552	17342731
ENST00000325102	121.25499	1.02998322	0.326729	3.152412	0.001619275	0.012434896	ENSG00000125457	17	75266227	75271231
ENST00000325110	1057.2737	-1.160411505	0.183478	-6.32453	2.54E-10	1.96E-08	ENSG00000112972	5	43287469	43313412
ENST00000325123	15.573592	2.956232169	0.905441	3.264964	0.001094781	0.009118349	ENSG00000114812	3	42502642	42537568
ENST00000325167	75.147627	-1.708786154	0.400027	-4.271672	1.94E-05	0.000340547	ENSG00000176108	17	80991840	81000133
ENST00000325307	943.56393	-1.722916769	0.190938	-9.023438	1.82E-19	6.46E-17	ENSG00000029993	X	150983334	150990771
ENST00000325327	2102.3226	-1.010872142	0.192426	-5.253303	1.49E-07	5.27E-06	ENSG00000176619	19	2428165	2456959
ENST00000325421	90.086104	-1.349251146	0.364671	-3.699916	0.00021567	0.002513423	ENSG00000218891	19	55576773	55580848
ENST00000325617	35.511085	-2.559059091	0.608328	-4.206711	2.59E-05	0.000432882	ENSG00000153132	4	140388452	140427648
ENST00000325805	1446.1139	-1.021528579	0.209346	-4.879608	1.06E-06	2.84E-05	ENSG00000114439	3	107522961	107811339
ENST00000325897	7.0582317	6.239158391	1.858704	3.356726	0.000788713	0.007057803	ENSG00000177182	8	61287969	61501629
ENST00000326047	332.09129	-1.092726449	0.250149	-4.368304	1.25E-05	0.000235171	ENSG00000163808	3	44761793	44853256
ENST00000326172	65.068934	1.557120424	0.423658	3.675419	0.00023746	0.00272055	ENSG00000144802	3	101849513	101861022
ENST00000326194	62.793317	1.640370985	0.435484	3.766773	0.000165372	0.002024243	ENSG00000102312	X	48508991	48520808
ENST00000326294	33.958579	2.994574729	0.627964	4.768702	1.85E-06	4.62E-05	ENSG00000213402	11	67435509	67437682
ENST00000326427	242.43431	-1.158324744	0.319534	-3.625047	0.000288909	0.003194762	ENSG00000135916	2	230864941	230879248
ENST00000326840	2120.0756	-1.105515879	0.196805	-5.617313	1.94E-08	8.74E-07	ENSG00000057019	3	98795940	98901695
ENST00000327040	262.97914	-1.057892287	0.291884	-3.624358	0.00028968	0.00320186	ENSG00000156011	8	18527302	19013703
ENST00000327111	14.647128	-3.197616281	1.027764	-3.111235	0.001863064	0.013879047	ENSG00000175745	5	93583221	93594611
ENST00000327428	112.61022	-1.788631583	0.36263	-4.932388	8.12E-07	2.27E-05	ENSG00000163170	2	74135399	74147912
ENST00000327435	894.53923	-1.104852887	0.217833	-5.072016	3.94E-07	1.21E-05	ENSG00000182551	2	3497365	3519531
ENST00000327490	170.71594	-1.370943624	0.284396	-4.82054	1.43E-06	3.68E-05	ENSG00000185262	17	76265347	76271298
ENST00000327741	1251.1935	-2.111723282	0.193265	-10.92659	8.60E-28	8.68E-25	ENSG00000205426	12	52285912	52291534
ENST00000327906	40.488495	2.055359542	0.530547	3.874039	0.000107046	0.001409263	ENSG00000010318	3	52410659	52423641
ENST00000327949	182.38281	1.286064379	0.284132	4.526285	6.00E-06	0.000126352	ENSG00000169660	17	82418346	82442645
ENST00000328111	466.32638	-2.07125134	0.21649	-9.567427	1.10E-21	5.06E-19	ENSG00000088305	20	32762384	32809356
ENST00000328300	19.35981	-7.747742057	1.597341	-4.8504	1.23E-06	3.23E-05	ENSG00000188153	X	108439837	108697545
ENST00000328697	155.8465	-2.464748377	0.310953	-7.92643	2.26E-15	4.65E-13	ENSG00000109881	11	27338511	27363215
ENST00000328747	147.51802	-1.016920777	0.313599	-3.242737	0.001183873	0.009711782	ENSG00000146757	7	65373854	65401136
ENST00000328850	108.06699	1.48232077	0.3519	4.212341	2.53E-05	0.00042423	ENSG00000182511	15	90884503	90895776
ENST00000328880	19.372989	-3.922420279	0.954736	-4.108381	3.98E-05	0.000623288	ENSG00000182782	12	122701292	122703357
ENST00000328965	45.009648	-2.090439558	0.503271	-4.153702	3.27E-05	0.00052756	ENSG00000184232	11	120211031	120230334
ENST00000329003	14.416909	7.273983685	1.634557	4.450124	8.58E-06	0.000170312	ENSG00000182687	17	76074780	76077537
ENST00000329078	133.94854	-1.852563213	0.316731	-5.849014	4.94E-09	2.67E-07	ENSG00000183018	17	4498880	4539035

GTPBP3	protein_coding	Yes	NM_032620.4	108
MIF4GD	protein_coding	Yes	NM_001370592.1	108
HMGCS1	protein_coding	Yes	NM_001098272.3	108
VIPR1	protein_coding	Yes	NM_004624.4	108
CHMP6	protein_coding	Yes	NM_024591.5	108
HMGB3	protein_coding	Yes	NM_005342.4	108
LMNB2	protein_coding	Yes	NM_032737.4	108
ZNF579	protein_coding	Yes	NM_152600.3	108
CLGN	protein_coding	Yes	NM_004362.3	108
BBX	protein_coding	Yes	NM_001142568.3	108
CLVS1	protein_coding	Yes	NM_173519.3	108
KIF15	protein_coding	Yes	NM_020242.3	108
NFKBIZ	protein_coding	Yes	NM_031419.4	108
PORCN	protein_coding	Yes	NM_203475.3	108
PTPRCAP	protein_coding	Yes	NM_005608.3	108
ITM2C	protein_coding	Yes	NM_030926.6	108
DCBLD2	protein_coding	Yes	NM_080927.4	108
PSD3	protein_coding	Yes	NM_015310.4	108
NR2F1	protein_coding	Yes	NM_005654.6	108
BOLA3	protein_coding	Yes	NM_212552.3	108
ADI1	protein_coding	Yes	NM_018269.4	108
UBALD2	protein_coding	Yes	NM_182565.4	108
KRT81	protein_coding	Yes	NM_002281.4	108
PHF7	protein_coding	Yes	NM_016483.7	108
HEXD	protein_coding	Yes	NM_001330542.2	108
DNMT3B	protein_coding	Yes	NM_006892.4	108
COL4A5	protein_coding	Yes	NM_033380.3	108
CCDC34	protein_coding	Yes	NM_030771.2	108
ZNF92	protein_coding	Yes	NM_152626.4	108
FES	protein_coding	Yes	NM_002005.4	108
HCAR2	protein_coding	Yes	NM_177551.4	108
OAF	protein_coding	Yes	NM_178507.4	108
GALR2	protein_coding	Yes	NM_003857.4	108
SPNS2	protein_coding	Yes	NM_001124758.3	108

ENST00000329146	54.814897	-2.197304188	0.47598	-4.61638	3.90E-06	8.75E-05	ENSG00000182809	14	105474820	105480162
ENST00000329363	33.195164	2.548837179	0.629164	4.051147	5.10E-05	0.000766751	ENSG00000177989	22	50530425	50532498
ENST00000329492	386.33108	2.290463773	0.254245	9.00888	2.08E-19	7.36E-17	ENSG00000008735	22	50600792	50613978
ENST00000329565	232.53913	-1.355569815	0.271977	-4.984137	6.22E-07	1.80E-05	ENSG00000184602	16	11668454	11679152
ENST00000329858	8.1052711	-5.014011868	1.742947	-2.876744	0.00401801	0.025322069	ENSG00000184574	12	6618834	6635959
ENST00000329962	59.3373	-2.720918789	0.470609	-5.781702	7.39E-09	3.84E-07	ENSG00000182272	11	369498	382117
ENST00000330133	126.15108	-1.859357022	0.350293	-5.308006	1.11E-07	4.06E-06	ENSG00000183617	19	3762681	3767565
ENST00000330263	698.03075	-1.568779126	0.272434	-5.75838	8.49E-09	4.33E-07	ENSG00000053372	1	19251804	19260128
ENST00000330333	524.64307	-1.247280605	0.208185	-5.991208	2.08E-09	1.24E-07	ENSG00000182240	21	41168159	41282530
ENST00000330560	550.71852	-1.391984957	0.211619	-6.577793	4.77E-11	4.32E-09	ENSG00000184661	8	25459232	25507911
ENST00000330676	98.065335	1.217051605	0.362639	3.356092	0.000790521	0.007070666	ENSG00000185561	17	1702815	1710377
ENST00000330714	62.911512	1.770442182	0.450524	3.929741	8.50E-05	0.001164894	ENSG00000183486	21	41362026	41409393
ENST00000330722	9.9132767	-5.3160037	1.767609	-3.007455	0.002634454	0.018244267	ENSG00000205420	12	52487175	52493257
ENST00000330852	199.86139	-1.171016067	0.270042	-4.336418	1.45E-05	0.000266579	ENSG00000157869	4	13367723	13484340
ENST00000330877	8.5042194	3.982928496	1.402983	2.838901	0.004526927	0.027726788	ENSG00000185100	14	104724228	104747310
ENST00000330889	77.419944	-1.309006178	0.394489	-3.318236	0.000905878	0.007867896	ENSG00000184060	17	30921944	30959322
ENST00000330891	9.2258587	6.626889885	1.750513	3.785685	0.000153286	0.00190294	ENSG00000127325	12	69653608	69699303
ENST00000331128	631.86564	-1.727690074	0.201156	-8.588817	8.79E-18	2.46E-15	ENSG00000184270	1	149887468	149887965
ENST00000331302	11.747271	-3.939031856	1.216814	-3.237169	0.001207219	0.009861373	ENSG00000181031	17	212388	352807
ENST00000331334	297.57154	-1.04331604	0.247321	-4.218462	2.46E-05	0.000414428	ENSG00000182512	14	95535049	95544714
ENST00000331380	1871.6347	-1.210403821	0.174739	-6.926927	4.30E-12	4.76E-10	ENSG00000184260	1	149886917	149887411
ENST00000331442	5465.1529	-1.927209203	0.168674	-11.42562	3.11E-30	3.80E-27	ENSG00000184357	6	27866791	27867588
ENST00000331491	713.84829	-1.343466535	0.199792	-6.72434	1.76E-11	1.75E-09	ENSG00000183598	1	149813224	149813693
ENST00000331628	10.489188	3.696506866	1.235894	2.990958	0.002781036	0.019014019	ENSG00000183379	14	74405898	74426210
ENST00000331782	133.72576	-3.87540878	0.394874	-9.814289	9.77E-23	5.36E-20	ENSG00000184916	14	105140994	105168776
ENST00000331817	3311.6359	-1.470709189	0.202005	-7.280565	3.32E-13	4.56E-11	ENSG00000135480	12	52233242	52248921
ENST00000331835	3521.8523	-1.059872355	0.17602	-6.021306	1.73E-09	1.05E-07	ENSG00000183291	1	86862444	86914126
ENST00000331979	4.102683	5.46289539	2.037889	2.680664	0.007347629	0.040289962	ENSG00000183239	6	44089241	44089696
ENST00000332235	8.5297216	-4.066527656	1.428773	-2.846168	0.004424879	0.027238588	ENSG00000183186	19	405444	409147
ENST00000332439	90.324914	1.871646284	0.409162	4.574344	4.78E-06	0.000104019	ENSG00000182224	17	7858002	7862282
ENST00000332499	1194.4456	1.028808706	0.220111	4.674046	2.95E-06	6.87E-05	ENSG00000186834	17	45148474	45152099
ENST00000332509	57.748186	1.221139617	0.466622	2.61698	0.008871141	0.046398086	ENSG00000184381	22	38111494	38181830
ENST00000332884	59.098018	-2.228267625	0.495551	-4.496544	6.91E-06	0.000142275	ENSG00000155016	4	107931548	107953461
ENST00000333017	11.78414	6.982303814	1.674241	4.17043	3.04E-05	0.000495634	ENSG00000185674	2	99242245	99255666

CRIP2	protein_coding	Yes	NM_001312.4	108
ODF3B	protein_coding	Yes	NM_001014440.4	108
MAPK8IP2	protein_coding	Yes	NM_012324.6	108
SNN	protein_coding	Yes	NM_003498.6	108
LPAR5	protein_coding	Yes	NM_020400.6	108
B4GALNT4	protein_coding	Yes	NM_178537.5	108
MRPL54	protein_coding	Yes	NM_172251.3	108
MRTO4	protein_coding	Yes	NM_016183.4	108
BACE2	protein_coding	Yes	NM_012105.5	108
CDCA2	protein_coding	Yes	NM_152562.4	108
TLCD2	protein_coding	Yes	NM_001164407.2	108
MX2	protein_coding	Yes	NM_002463.2	108
KRT6A	protein_coding	Yes	NM_005554.4	108
RAB28	protein_coding	Yes	NM_001017979.3	108
ADSS1	protein_coding	Yes	NM_152328.5	108
ADAP2	protein_coding	Yes	NM_018404.3	108
BEST3	protein_coding	Yes	NM_032735.3	108
H2AC21	protein_coding	Yes	NM_175065.3	108
RPH3AL	protein_coding	Yes	NM_006987.4	108
GLRX5	protein_coding	Yes	NM_016417.3	108
H2AC20	protein_coding	Yes	NM_003517.3	108
H1-5	protein_coding	Yes	NM_005322.3	108
H3C13	protein_coding	Yes	NM_001123375.3	108
SYNDIG1L	protein_coding	Yes	NM_001105579.2	108
JAG2	protein_coding	Yes	NM_002226.5	108
KRT7	protein_coding	Yes	NM_005556.4	108
SELENOF	protein_coding	Yes	NM_004261.5	108
-	essed_pseudog	Yes	-	108
C2CD4C	protein_coding	Yes	NM_001136263.2	108
CYB5D1	protein_coding	Yes	NM_144607.6	108
HEXIM1	protein_coding	Yes	NM_006460.3	108
PLA2G6	protein_coding	Yes	NM_003560.4	108
CYP2U1	protein_coding	Yes	NM_183075.3	108
LYG2	protein_coding	Yes	NM_175735.4	108

ENST00000333090	297.62527	-1.587220777	0.264807	-5.993882	2.05E-09	1.22E-07	ENSG00000182704	11	76783355	76798144
ENST00000333130	341.20392	-1.107525437	0.280575	-3.94734	7.90E-05	0.001098819	ENSG00000185608	22	19432544	19436075
ENST00000333151	2934.3822	-1.368289318	0.167312	-8.178074	2.88E-16	6.73E-14	ENSG00000276368	6	27814301	27814777
ENST00000333167	242.63083	1.182856707	0.251763	4.698287	2.62E-06	6.22E-05	ENSG00000060971	3	38122714	38137127
ENST00000333279	9.4923672	6.67170838	1.723743	3.870477	0.000108623	0.001426564	ENSG00000183831	1	173608335	173669851
ENST00000333421	335.30785	-1.277978994	0.235893	-5.417626	6.04E-08	2.43E-06	ENSG00000107362	9	71865151	71911193
ENST00000333467	648.75204	2.860576883	0.209376	13.66242	1.70E-42	3.99E-39	ENSG00000179750	22	38982346	38992779
ENST00000333640	42.073914	1.754265833	0.515424	3.403537	0.000665195	0.006173233	ENSG00000184271	12	51186935	51218062
ENST00000333762	401.78054	-1.318635847	0.231716	-5.690746	1.26E-08	6.07E-07	ENSG00000184897	3	129314770	129316286
ENST00000333845	94.399735	1.14608717	0.351696	3.258742	0.001119073	0.009285662	ENSG00000241404	6	32164594	32168281
ENST00000333850	4.4097416	5.564557071	1.995673	2.788312	0.005298356	0.031321376	ENSG00000139287	12	71938844	72032440
ENST00000333861	24.412848	1.754441652	0.656598	2.672017	0.007539692	0.04111721	ENSG00000182791	11	66590175	66593063
ENST00000333868	193.23926	1.10098938	0.280215	3.929093	8.53E-05	0.00116741	ENSG00000132906	1	15491400	15524215
ENST00000333891	18.856523	-3.832309045	0.94823	-4.041541	5.31E-05	0.000792384	ENSG00000186472	7	82754011	83162884
ENST00000334062	83.507136	-9.858017936	1.487138	-6.628852	3.38E-11	3.16E-09	ENSG00000185989	13	113977782	114132623
ENST00000334126	1572.0769	1.892344065	0.189694	9.975783	1.95E-23	1.20E-20	ENSG00000168014	11	74012717	74171002
ENST00000334179	5.8548263	5.974190633	1.874526	3.187041	0.001437365	0.011313862	ENSG00000186150	1	110112442	110113947
ENST00000334306	118.97603	-1.406486522	0.335617	-4.190743	2.78E-05	0.000459667	ENSG00000186212	4	76894151	76898144
ENST00000334307	53.159144	-1.452196458	0.467392	-3.10702	0.001889838	0.014034207	ENSG00000166352	11	36594501	36659272
ENST00000334456	36.341129	2.169507795	0.571132	3.798609	0.00014551	0.001823797	ENSG00000186642	11	72576140	72674422
ENST00000334529	10.497139	6.816375511	1.698639	4.012846	6.00E-05	0.000873053	ENSG00000186265	3	112463965	112499472
ENST00000334635	314.17784	-1.300359201	0.255113	-5.097188	3.45E-07	1.08E-05	ENSG00000109184	4	51843152	51916837
ENST00000334651	73.754463	-1.234926937	0.393434	-3.138838	0.001696194	0.012893055	ENSG00000169084	X	2219505	2500976
ENST00000334816	46.981202	1.370513912	0.487162	2.813263	0.004904148	0.029566697	ENSG00000186132	2	119302224	119366834
ENST00000334955	278.05455	-1.219003467	0.247301	-4.929229	8.26E-07	2.30E-05	ENSG00000187257	7	77696458	77783022
ENST00000335125	36.986053	-1.58175482	0.542655	-2.914842	0.003558688	0.023005733	ENSG00000187630	14	23988928	24006403
ENST00000335154	79.90495	-2.343679235	0.401347	-5.839532	5.23E-09	2.81E-07	ENSG00000161791	12	49636498	49707405
ENST00000335185	14.914396	4.828004014	1.275534	3.785085	0.000153656	0.001906607	ENSG00000186714	11	32602720	32794662
ENST00000335473	7.4579944	6.325573025	1.803012	3.508337	0.000450918	0.004542892	ENSG00000133454	22	25742187	26031045
ENST00000335659	306.57415	-1.228626	0.243903	-5.037345	4.72E-07	1.42E-05	ENSG00000186106	8	100520771	100559759
ENST00000335670	1555.8652	1.198633721	0.197898	6.056814	1.39E-09	8.72E-08	ENSG00000069667	15	60488283	61229302
ENST00000335987	125.95671	-1.434576285	0.382476	-3.750765	0.000176296	0.002134186	ENSG00000172818	11	65787062	65797214
ENST00000336023	11632.337	-1.119975002	0.220445	-5.08052	3.76E-07	1.17E-05	ENSG00000123416	12	49127781	49131395
ENST00000336032	509.23465	1.289837246	0.247019	5.221612	1.77E-07	6.09E-06	ENSG00000146278	6	89080750	89085160

TSKU	protein_coding	Yes	NM_015516.4	108
MRPL40	protein_coding	Yes	NM_003776.4	108
H2AC14	protein_coding	Yes	NM_021066.3	108
ACAA1	protein_coding	Yes	NM_001607.4	108
ANKRD45	protein_coding	Yes	NM_198493.3	108
ABHD17B	protein_coding	Yes	NM_001025780.3	108
APOBEC3B	protein_coding	Yes	NM_004900.5	108
POU6F1	protein_coding	Yes	NM_001330422.2	108
H1-10	protein_coding	Yes	NM_006026.4	108
EGFL8	protein_coding	Yes	NM_030652.4	108
TPH2	protein_coding	Yes	NM_173353.4	108
CCDC87	protein_coding	Yes	NM_018219.3	108
CASP9	protein_coding	Yes	NM_001229.5	108
PCLO	protein_coding	Yes	NM_033026.6	108
RASA3	protein_coding	Yes	NM_007368.4	108
C2CD3	protein_coding	Yes	NM_001286577.2	108
UBL4B	protein_coding	Yes	NM_203412.2	108
SOWAHB	protein_coding	Yes	NM_001029870.3	108
IFTAP	protein_coding	Yes	NM_138787.4	108
PDE2A	protein_coding	Yes	NM_002599.5	108
BTLA	protein_coding	Yes	NM_181780.4	108
DCUN1D4	protein_coding	Yes	NM_001040402.3	108
DHRSX	protein_coding	Yes	NM_145177.3	108
C2orf76	protein_coding	Yes	NM_001322331.2	108
RSBN1L	protein_coding	Yes	NM_198467.3	108
DHRS4L2	protein_coding	Yes	NM_198083.4	108
FMNL3	protein_coding	Yes	NM_175736.5	108
CCDC73	protein_coding	Yes	NM_001008391.4	108
MYO18B	protein_coding	Yes	NM_032608.7	108
ANKRD46	protein_coding	Yes	NM_001270377.2	108
RORA	protein_coding	Yes	NM_134261.3	108
OVOL1	protein_coding	Yes	NM_004561.4	108
TUBA1B	protein_coding	Yes	NM_006082.3	108
PNRC1	protein_coding	Yes	NM_006813.3	108

ENST00000336219	331.10209	-1.897850688	0.261517	-7.257096	3.95E-13	5.35E-11	ENSG00000170779	14	105009572	105021083
ENST00000336334	42.592174	3.57926701	0.605272	5.913488	3.35E-09	1.88E-07	ENSG00000167754	19	50943302	50953038
ENST00000336375	68.352271	-3.061550442	0.462197	-6.623902	3.50E-11	3.26E-09	ENSG00000014257	3	132317406	132358841
ENST00000336689	127.33308	-2.436062752	0.34487	-7.063715	1.62E-12	1.96E-10	ENSG00000088280	1	23428562	23484179
ENST00000337523	156.98527	1.247069295	0.291653	4.275863	1.90E-05	0.000335457	ENSG00000204348	6	31969814	31972138
ENST00000337539	47.716418	1.759014368	0.482242	3.647574	0.000264728	0.002975478	ENSG00000128271	22	24427598	24442357
ENST00000337554	284.63114	1.368849961	0.252221	5.427181	5.73E-08	2.32E-06	ENSG00000100300	22	43151558	43163242
ENST00000337843	91.00673	-1.454915122	0.36624	-3.972576	7.11E-05	0.00100495	ENSG00000133466	22	37180165	37188247
ENST00000338033	63.84493	-2.511161799	0.477629	-5.257561	1.46E-07	5.16E-06	ENSG00000181085	8	143716348	143722458
ENST00000338134	113.01355	1.188717533	0.329994	3.602245	0.000315481	0.003422991	ENSG00000118162	19	47475149	47484219
ENST00000338333	6.1370558	6.041079267	1.857764	3.251801	0.001146762	0.009476202	ENSG00000188573	5	168529304	168530634
ENST00000338380	66.941898	-2.222539196	0.46859	-4.743041	2.11E-06	5.16E-05	ENSG00000124107	20	45252238	45254564
ENST00000338492	39.717491	2.147651149	0.554157	3.875529	0.000106394	0.001402001	ENSG00000151640	10	132186947	132205759
ENST00000338625	246.79852	-1.178460652	0.28426	-4.145713	3.39E-05	0.000543421	ENSG00000102743	13	40789610	40812460
ENST00000338702	20.917391	-2.755267109	0.761117	-3.620031	0.000294568	0.003243886	ENSG00000189221	X	43656298	43746817
ENST00000338745	77.330687	-3.18998991	0.458326	-6.960091	3.40E-12	3.86E-10	ENSG00000182568	3	18345376	18425339
ENST00000338779	497.2219	-1.024646896	0.212959	-4.811475	1.50E-06	3.83E-05	ENSG00000132613	16	70661203	70686053
ENST00000338784	86.781459	1.645856656	0.370851	4.438049	9.08E-06	0.000178639	ENSG00000161955	17	7558749	7561601
ENST00000338821	638.85547	1.044418768	0.211143	4.946502	7.56E-07	2.13E-05	ENSG00000054793	20	51596513	51768390
ENST00000338961	413.98675	-1.418455421	0.261094	-5.432734	5.55E-08	2.25E-06	ENSG00000148334	9	128120692	128127729
ENST00000339276	1279.3817	-1.546373944	0.209102	-7.395309	1.41E-13	2.10E-11	ENSG00000175793	1	26863148	26864456
ENST00000339381	11.308031	2.679410218	1.025197	2.613555	0.008960556	0.0467477	ENSG00000169962	1	1331279	1335314
ENST00000339394	30.596361	-1.981183016	0.618379	-3.203831	0.00135612	0.010814545	ENSG00000162512	1	30869465	30908758
ENST00000339468	55.516376	1.207514107	0.453839	2.660669	0.007798559	0.042181646	ENSG00000100027	22	21697535	21735794
ENST00000339562	32.145923	2.399662307	0.660555	3.632795	0.000280368	0.00311539	ENSG00000153234	2	156324436	156332721
ENST00000339728	634.28965	-1.172558681	0.220677	-5.313458	1.08E-07	3.96E-06	ENSG00000188042	2	234493040	234497081
ENST00000339732	5.364855	5.848166865	1.909051	3.063389	0.002188451	0.015739149	ENSG00000131386	3	16174679	16230165
ENST00000339738	4.335254	5.542764563	2.013314	2.753055	0.005904191	0.034056399	ENSG00000149599	20	31861076	31870527
ENST00000339812	1828.7884	-1.333905216	0.174066	-7.663228	1.81E-14	3.27E-12	ENSG00000124635	6	27132315	27132795
ENST00000339824	112.17495	1.423364008	0.40034	3.555385	0.000377426	0.003939448	ENSG00000171435	12	117453011	117968990
ENST00000339852	7.656628	6.359266538	1.788275	3.55609	0.000376415	0.003931058	ENSG00000188505	19	39196963	39201884
ENST00000339950	3298.5819	-1.598297465	0.166221	-9.61548	6.88E-22	3.27E-19	ENSG00000162607	1	62437048	62451804
ENST00000340006	385.35086	-3.398647068	0.792809	-4.286842	1.81E-05	0.000322194	ENSG00000159176	1	201483529	201507123
ENST00000340083	40.320264	-2.368086177	0.602797	-3.928494	8.55E-05	0.001170006	ENSG00000175920	4	3463305	3494482

CDCA4	protein_coding	Yes	NM_017955.4	108
KLK5	protein_coding	Yes	NM_012427.5	108
ACP3	protein_coding	Yes	NM_001099.5	108
ASAP3	protein_coding	Yes	NM_017707.4	108
DXO	protein_coding	Yes	NM_005510.4	108
ADORA2A	protein_coding	Yes	NM_000675.6	108
TSPO	protein_coding	Yes	NM_000714.6	108
C1QTNF6	protein_coding	Yes	NM_031910.4	108
MAPK15	protein_coding	Yes	NM_139021.3	108
KPTN	protein_coding	Yes	NM_007059.4	108
FBLL1	protein_coding	Yes	NM_001355274.2	108
SLPI	protein_coding	Yes	NM_003064.4	108
DPYSL4	protein_coding	Yes	NM_006426.3	108
SLC25A15	protein_coding	Yes	NM_014252.4	108
MAOA	protein_coding	Yes	NM_000240.4	108
SATB1	protein_coding	Yes	NM_002971.6	108
MTSS2	protein_coding	Yes	NM_138383.3	108
TNFSF13	protein_coding	Yes	NM_003808.4	108
ATP9A	protein_coding	Yes	NM_006045.3	108
PTGES2	protein_coding	Yes	NM_025072.7	108
SFN	protein_coding	Yes	NM_006142.5	108
TAS1R3	protein_coding	Yes	NM_152228.3	108
SDC3	protein_coding	Yes	NM_014654.4	108
YPEL1	protein_coding	Yes	NM_013313.5	108
NR4A2	protein_coding	Yes	NM_006186.4	108
ARL4C	protein_coding	Yes	NM_001282431.2	108
GALNT15	protein_coding	Yes	NM_054110.5	108
DUSP15	protein_coding	Yes	NM_080611.5	108
H2BC11	protein_coding	Yes	NM_021058.4	108
KSR2	protein_coding	Yes	NM_173598.6	108
NCCRP1	protein_coding	Yes	NM_001001414.2	108
USP1	protein_coding	Yes	NM_003368.5	108
CSRP1	protein_coding	Yes	NM_004078.3	108
DOK7	protein_coding	Yes	NM_173660.5	108

ENST00000340099	198.00746	-1.233807489	0.268625	-4.593042	4.37E-06	9.63E-05	ENSG00000125170	16	57471921	57486493
ENST00000340368	678.70754	-1.619661548	0.20378	-7.9481	1.89E-15	3.93E-13	ENSG00000186480	7	155297877	155310235
ENST00000340434	1979.1836	-1.045049634	0.18404	-5.678389	1.36E-08	6.42E-07	ENSG00000188647	9	69709524	69760011
ENST00000340510	32.222289	1.631416927	0.575817	2.833221	0.004608152	0.028118069	ENSG00000188185	7	39733631	39793092
ENST00000340607	606.28795	-2.439471426	0.209493	-11.64462	2.44E-31	3.18E-28	ENSG00000148344	9	129738348	129753042
ENST00000340635	44.651304	-1.997025416	0.594864	-3.357112	0.000787613	0.007049205	ENSG00000113448	5	58969037	59893726
ENST00000340857	1845.4623	-1.568026089	0.199164	-7.873034	3.46E-15	6.93E-13	ENSG00000189060	22	37805228	37807432
ENST00000340866	465.37078	-2.705801443	0.234722	-11.52768	9.57E-31	1.22E-27	ENSG00000116791	1	74705485	74733050
ENST00000340940	7.1169983	6.254848675	1.806594	3.462232	0.000535715	0.005208558	ENSG00000188883	7	139452689	139483673
ENST00000340958	1998.1185	-1.190543672	0.17865	-6.664113	2.66E-11	2.53E-09	ENSG00000189143	7	73830995	73832690
ENST00000341023	1959.2956	-1.228433213	0.174098	-7.055985	1.71E-12	2.06E-10	ENSG00000196866	6	26198783	26199293
ENST00000341156	191.03969	1.670171524	0.285281	5.854474	4.79E-09	2.60E-07	ENSG00000135454	12	57623408	57633201
ENST00000341420	9.830585	-5.300099766	1.692773	-3.131016	0.001742024	0.013158729	ENSG00000125848	20	14322984	14337612
ENST00000341423	5293.1676	-1.34683638	0.176848	-7.615806	2.62E-14	4.54E-12	ENSG00000189403	13	30456703	30465936
ENST00000341590	299.56068	-1.094715764	0.313057	-3.496862	0.000470765	0.004700283	ENSG00000073712	14	52857272	52951050
ENST00000341695	955.59628	-1.310218604	0.18716	-7.000544	2.55E-12	2.96E-10	ENSG00000138777	4	105369076	105474062
ENST00000341911	61.088477	-1.415702401	0.430501	-3.288499	0.00100723	0.008528495	ENSG00000118513	6	135181307	135219172
ENST00000341947	65.103714	1.865233303	0.454232	4.106346	4.02E-05	0.000627773	ENSG00000204248	6	33162693	33192467
ENST00000342232	59.480975	-2.336566071	0.473922	-4.930279	8.21E-07	2.29E-05	ENSG00000104369	8	74234699	74321540
ENST00000342291	80.3573	1.290540876	0.38942	3.31401	0.000919683	0.007962834	ENSG00000182324	19	48455573	48466980
ENST00000342456	78.039981	1.190730652	0.446637	2.665993	0.007676127	0.041690006	ENSG00000006534	11	68010326	68029276
ENST00000342462	86.72108	1.139641957	0.363724	3.133264	0.001728741	0.013079133	ENSG00000188167	3	33090421	33097146
ENST00000342494	589.62336	1.00299095	0.20581	4.873392	1.10E-06	2.92E-05	ENSG00000151148	12	109477633	109536702
ENST00000342665	105.53864	1.325489627	0.384279	3.449291	0.00056206	0.005407067	ENSG00000177732	20	325551	330224
ENST00000342679	151.91748	1.014936828	0.294877	3.441898	0.000577649	0.005516679	ENSG00000034152	17	21284710	21315232
ENST00000342782	11.087527	-4.438266481	1.328352	-3.341182	0.000834225	0.007370304	ENSG00000130829	X	153647202	153651326
ENST00000342784	58.436148	-1.446270711	0.435576	-3.320367	0.000898992	0.007821005	ENSG00000105137	19	15107400	15114974
ENST00000343433	12.106258	3.474478446	1.136556	3.057023	0.002235468	0.016005554	ENSG00000187889	1	56718788	56819404
ENST00000343677	6127.8594	-1.633288244	0.162036	-10.07978	6.79E-24	4.50E-21	ENSG00000187837	6	26055739	26056470
ENST00000343736	45.893819	1.767634307	0.498769	3.543992	0.000394117	0.004081247	ENSG00000005379	17	58301230	58328795
ENST00000343959	9.0520543	6.602338713	1.735317	3.804687	0.000141984	0.001787121	ENSG00000188100	10	87020293	87024730
ENST00000343986	935.19324	1.057993799	0.191366	5.528639	3.23E-08	1.38E-06	ENSG00000171858	20	62387102	62388520
ENST00000344279	555.64436	3.411922196	0.237725	14.35237	1.03E-46	4.48E-43	ENSG00000235109	6	28324736	28336148
ENST00000344318	228.05343	1.465333489	0.262639	5.579271	2.42E-08	1.07E-06	ENSG00000100319	22	29730955	29767007

DOK4	protein_coding	Yes	NM_018110.5	108
INSIG1	protein_coding	Yes	NM_005542.6	108
PTAR1	protein_coding	Yes	NM_001099666.2	108
LINC00265	lncRNA	Yes	-	108
PTGES	protein_coding	Yes	NM_004878.5	108
PDE4D	protein_coding	Yes	NM_001104631.2	108
H1-0	protein_coding	Yes	NM_005318.4	108
CRYZ	protein_coding	Yes	NM_001889.4	108
KLRG2	protein_coding	Yes	NM_198508.4	108
CLDN4	protein_coding	Yes	NM_001305.5	108
H2AC7	protein_coding	Yes	NM_021065.3	108
B4GALNT1	protein_coding	Yes	NM_001478.5	108
FLRT3	protein_coding	Yes	NM_198391.3	108
HMGB1	protein_coding	Yes	NM_002128.7	108
FERMT2	protein_coding	Yes	NM_006832.3	108
PPA2	protein_coding	Yes	NM_176869.3	108
MYB	protein_coding	Yes	NM_001130173.2	108
COL11A2	protein_coding	Yes	NM_080680.3	108
JPH1	protein_coding	Yes	NM_020647.4	108
KCNJ14	protein_coding	Yes	NM_013348.4	108
ALDH3B1	protein_coding	Yes	NM_000694.4	108
TMPPE	protein_coding	Yes	NM_001039770.3	108
UBE3B	protein_coding	Yes	NM_130466.4	108
SOX12	protein_coding	Yes	NM_006943.4	108
MAP2K3	protein_coding	Yes	NM_145109.3	108
DUSP9	protein_coding	Yes	NM_001318503.2	108
SYDE1	protein_coding	Yes	NM_033025.6	108
FYB2	protein_coding	Yes	NM_001004303.5	108
H1-2	protein_coding	Yes	NM_005319.4	108
TSPOAP1	protein_coding	Yes	NM_004758.4	108
FAM25A	protein_coding	Yes	NM_001146157.3	108
RPS21	protein_coding	Yes	NM_001024.4	108
ZSCAN31	protein_coding	Yes	NM_030899.5	108
ZMAT5	protein_coding	Yes	NM_001003692.2	108

ENST00000344320	120.7418	2.830647178	0.363302	7.791453	6.62E-15	1.28E-12	ENSG00000188277	15	40769979	40772449
ENST00000344363	23.211563	7.96108018	1.567461	5.078966	3.79E-07	1.18E-05	ENSG00000221880	17	41033883	41034874
ENST00000344532	65.178017	-1.266051621	0.440021	-2.877252	0.004011551	0.025304871	ENSG00000136161	13	48488962	48533080
ENST00000344629	131.43037	1.345425241	0.317406	4.238813	2.25E-05	0.000383812	ENSG00000114026	3	9749951	9757407
ENST00000344646	86.446198	3.368787194	0.448806	7.506109	6.09E-14	9.61E-12	ENSG00000172671	10	45615499	45672772
ENST00000344686	284.60204	1.553819131	0.243994	6.368274	1.91E-10	1.53E-08	ENSG00000006062	17	45263118	45317020
ENST00000344887	4.3600832	5.550086197	2.004404	2.768946	0.005623788	0.032777457	ENSG00000129991	19	55151766	55157732
ENST00000345363	80.716263	1.063775634	0.378284	2.812111	0.00492175	0.029650536	ENSG00000213903	14	24311501	24318036
ENST00000346234	474.14431	-1.078112909	0.213162	-5.057705	4.24E-07	1.30E-05	ENSG00000134996	9	75088513	75147265
ENST00000346473	545.43734	1.645618354	0.21223	7.75395	8.91E-15	1.68E-12	ENSG00000175197	12	57516592	57520514
ENST00000347077	29.347482	2.872783667	0.681227	4.217074	2.47E-05	0.000416602	ENSG00000187187	19	39997059	40021038
ENST00000347162	248.1689	1.578879141	0.308852	5.112087	3.19E-07	1.01E-05	ENSG00000137171	6	43059630	43075093
ENST00000347624	3.8701119	5.378338053	2.06548	2.603917	0.009216513	0.047791845	ENSG00000172061	3	194355248	194369743
ENST00000349004	1161.1354	1.068072854	0.18594	5.744179	9.24E-09	4.64E-07	ENSG00000101079	20	36651770	36746090
ENST00000349064	15.886783	3.519632512	0.975863	3.606687	0.000310132	0.003378178	ENSG00000213199	7	151048530	151052754
ENST00000349077	8.329512	6.482099485	1.758318	3.686535	0.000227329	0.002624006	ENSG00000118004	2	3595111	3644644
ENST00000349394	16.360755	-2.507385047	0.833721	-3.007462	0.002634394	0.018244267	ENSG00000182379	12	57216793	57226449
ENST00000349752	9.0520543	6.602338713	1.735317	3.804687	0.000141984	0.001787121	ENSG00000158089	2	30910466	31138440
ENST00000349847	265.78556	-3.096876539	0.338793	-9.140909	6.19E-20	2.28E-17	ENSG00000136155	13	77535705	77645263
ENST00000349880	319.93562	-1.20734996	0.295588	-4.084564	4.42E-05	0.00067977	ENSG00000166833	11	19712836	20121601
ENST00000350051	1518.8085	-1.062379128	0.188373	-5.639769	1.70E-08	7.79E-07	ENSG00000089685	17	78214252	78225635
ENST00000350796	4583.7628	-1.174164063	0.175021	-6.708707	1.96E-11	1.93E-09	ENSG00000008256	7	6161778	6272624
ENST00000350881	24.421053	1.930476557	0.664218	2.906392	0.003656232	0.023513188	ENSG00000113296	5	80035347	80083287
ENST00000350997	523.98198	-1.683857362	0.229753	-7.32898	2.32E-13	3.32E-11	ENSG00000149485	11	61799626	61817003
ENST00000352909	6.3665626	-6.143247956	1.850648	-3.319511	0.000901752	0.00783716	ENSG00000180176	11	2163928	2171815
ENST00000353267	897.03322	-1.430008344	0.195806	-7.303179	2.81E-13	3.93E-11	ENSG00000118260	2	207529961	207605988
ENST00000354193	780.02079	-1.015712944	0.202252	-5.022028	5.11E-07	1.52E-05	ENSG00000198168	11	22818926	22829801
ENST00000354258	529.57139	1.944058367	0.226146	8.596469	8.22E-18	2.31E-15	ENSG00000168394	6	32845208	32853704
ENST00000354287	1461.1654	1.524634961	0.184741	8.252842	1.55E-16	3.73E-14	ENSG00000116661	1	11648386	11654429
ENST00000354960	153.09619	-1.816591656	0.306996	-5.917312	3.27E-09	1.85E-07	ENSG00000138735	4	119494402	119628804
ENST00000355057	129.14607	-1.252134787	0.335277	-3.734625	0.000187995	0.002249016	ENSG00000197238	6	27824091	27824480
ENST00000355085	16.982372	2.324513519	0.893228	2.602373	0.009258109	0.047961477	ENSG00000197405	19	47309860	47322066
ENST00000355086	217.88174	1.224922417	0.313029	3.913129	9.11E-05	0.001232108	ENSG00000196935	12	63844699	64162217
ENST00000355202	611.10448	1.023771705	0.199637	5.128167	2.93E-07	9.37E-06	ENSG00000198182	19	37696370	37719761

C15orf62	protein_coding	Yes	NM_001130448.3	108
KRTAP1-3	protein_coding	Yes	NM_030966.2	108
RCBTB2	protein_coding	Yes	NM_001268.4	108
OGG1	protein_coding	Yes	NM_002542.6	108
ZFAND4	protein_coding	Yes	NM_174890.4	108
MAP3K14	protein_coding	Yes	NM_003954.5	108
TNNI3	protein_coding	Yes	NM_000363.5	108
LTB4R	protein_coding	Yes	NM_001143919.3	108
OSTF1	protein_coding	Yes	NM_012383.5	108
DDIT3	protein_coding	Yes	NM_004083.6	108
ZNF546	protein_coding	Yes	NM_178544.5	108
KLC4	protein_coding	Yes	NM_201521.3	108
LRRC15	protein_coding	Yes	NM_130830.5	108
NDRG3	protein_coding	Yes	NM_032013.4	108
ASIC3	protein_coding	Yes	NM_004769.4	108
COLEC11	protein_coding	Yes	NM_024027.5	108
NXPH4	protein_coding	Yes	NM_007224.4	108
GALNT14	protein_coding	Yes	NM_024572.4	108
SCEL	protein_coding	Yes	NM_144777.3	108
NAV2	protein_coding	Yes	NM_145117.5	108
BIRC5	protein_coding	Yes	NM_001168.3	108
CYTH3	protein_coding	Yes	NM_004227.4	108
THBS4	protein_coding	Yes	NM_003248.6	108
FADS1	protein_coding	Yes	NM_013402.7	108
TH	protein_coding	Yes	NM_000360.4	108
CREB1	protein_coding	Yes	NM_004379.5	108
SVIP	protein_coding	Yes	NM_148893.3	108
TAP1	protein_coding	Yes	NM_000593.6	108
FBXO2	protein_coding	Yes	NM_012168.6	108
PDE5A	protein_coding	Yes	NM_001083.4	108
H4C11	protein_coding	Yes	NM_021968.4	108
C5AR1	protein_coding	Yes	NM_001736.4	108
SRGAP1	protein_coding	Yes	NM_020762.4	108
ZNF607	protein_coding	Yes	NM_032689.5	108

ENST00000355303	220.32855	-2.660562432	0.281626	-9.44714	3.48E-21	1.45E-18	ENSG00000131620	11	70078316	70189530
ENST00000355417	112.20666	-1.178659111	0.348684	-3.380311	0.000724039	0.006597994	ENSG00000198624	5	151181051	151224092
ENST00000355476	1209.8042	-1.099707135	0.192339	-5.717534	1.08E-08	5.32E-07	ENSG00000168214	4	26321013	26434923
ENST00000355485	2391.3714	-1.102497183	0.171763	-6.418723	1.37E-10	1.14E-08	ENSG00000173218	1	115641969	115698221
ENST00000355526	8.5984718	-6.577980513	1.749942	-3.75897	0.000170614	0.002079115	ENSG00000011332	19	38211005	38224222
ENST00000355527	571.13402	-1.36911401	0.219561	-6.235697	4.50E-10	3.18E-08	ENSG00000172893	11	71434410	71448393
ENST00000355577	102.28332	1.640999645	0.432358	3.795467	0.000147366	0.001841296	ENSG00000156042	10	73253761	73358864
ENST00000355591	105.5909	-1.107217694	0.335429	-3.300898	0.000963758	0.008248857	ENSG00000170516	4	46734826	46909245
ENST00000355610	291.38992	-1.292113413	0.252774	-5.111741	3.19E-07	1.01E-05	ENSG00000133131	X	106940737	107000212
ENST00000355673	101.37015	1.413727484	0.357733	3.951903	7.75E-05	0.001081132	ENSG00000166987	12	57522823	57530148
ENST00000355699	23.304627	2.127344236	0.715839	2.97182	0.002960406	0.01994819	ENSG00000160323	9	133422366	133459386
ENST00000355749	747.60322	-1.725528872	0.234769	-7.349909	1.98E-13	2.88E-11	ENSG00000128609	7	123536996	123557818
ENST00000355832	427.19467	1.290927601	0.216989	5.949268	2.69E-09	1.55E-07	ENSG00000225830	10	49454469	49539121
ENST00000355841	78.293245	-1.125245681	0.391033	-2.877621	0.004006867	0.025283205	ENSG00000196923	5	177483393	177497604
ENST00000355897	141.59876	-1.026008115	0.366308	-2.800947	0.005095296	0.030445725	ENSG00000173890	3	170037994	170085392
ENST00000355899	2935.2903	-1.192573586	0.168327	-7.084882	1.39E-12	1.70E-10	ENSG00000102024	X	115561173	115650861
ENST00000355904	469.74048	-1.094571535	0.221575	-4.93996	7.81E-07	2.19E-05	ENSG00000197265	8	30578317	30658236
ENST00000356083	93.312504	3.78302361	0.439953	8.598702	8.06E-18	2.27E-15	ENSG00000196739	9	114155536	114312511
ENST00000356091	443.87467	1.090153624	0.215241	5.064807	4.09E-07	1.25E-05	ENSG00000215440	20	58692799	58715844
ENST00000356151	99.449422	-1.089312044	0.3441	-3.165686	0.001547179	0.011988162	ENSG00000168297	3	58332891	58426127
ENST00000356189	512.23217	1.279387988	0.307208	4.164563	3.12E-05	0.000506169	ENSG00000176244	10	15075474	15088776
ENST00000356244	1768.013	-1.02352732	0.221927	-4.611998	3.99E-06	8.90E-05	ENSG00000100401	22	41244778	41286187
ENST00000356371	18.657891	-3.33397228	0.88641	-3.761209	0.000169094	0.002064383	ENSG00000109794	4	186105076	186172662
ENST00000356388	259.93756	-1.056732195	0.254616	-4.150303	3.32E-05	0.000534099	ENSG00000198369	2	65310850	65432599
ENST00000356404	469.84827	-1.773221496	0.214535	-8.265422	1.39E-16	3.38E-14	ENSG00000110697	11	67491767	67505363
ENST00000356443	18.458277	2.647498107	0.80557	3.286489	0.001014446	0.008576254	ENSG00000101605	18	3066806	3219968
ENST00000356524	54.030096	9.179909155	1.50259	6.109391	1.00E-09	6.52E-08	ENSG00000173432	11	18266263	18269967
ENST00000356530	730.37213	-1.690801975	0.196585	-8.600861	7.91E-18	2.24E-15	ENSG00000277224	6	26199515	26199988
ENST00000356575	368.59053	2.959957056	0.277364	10.67176	1.38E-26	1.22E-23	ENSG00000162591	1	3487950	3611508
ENST00000356660	93.891792	2.46778644	0.387576	6.367229	1.92E-10	1.54E-08	ENSG00000176697	11	27654892	27700455
ENST00000356709	134.75279	1.067175676	0.337757	3.159596	0.00157988	0.012193547	ENSG00000124508	6	26383107	26394874
ENST00000356736	516.30092	-1.831811051	0.210057	-8.720526	2.77E-18	8.47E-16	ENSG00000138821	4	102261665	102345482
ENST00000356860	6.380211	-6.145998772	1.855124	-3.312985	0.00092306	0.007983001	ENSG00000153956	7	81946443	82443777
ENST00000356884	842.74725	-1.552094961	0.192602	-8.058566	7.72E-16	1.70E-13	ENSG00000185963	9	92711362	92764833

ANO1	protein_coding	Yes	NM_018043.7	108
CCDC69	protein_coding	Yes	NM_015621.3	108
RBPJ	protein_coding	Yes	NM_015874.6	108
VANGL1	protein_coding	Yes	NM_138959.3	108
DPF1	protein_coding	Yes	NM_001135155.3	108
DHCR7	protein_coding	Yes	NM_001360.3	108
CFAP70	protein_coding	Yes	NM_001367801.1	108
COX7B2	protein_coding	Yes	NM_130902.3	108
MORC4	protein_coding	Yes	NM_024657.5	108
MBD6	protein_coding	Yes	NM_052897.4	108
ADAMTS13	protein_coding	Yes	NM_139027.6	108
NDUFA5	protein_coding	Yes	NM_005000.5	108
ERCC6	protein_coding	Yes	NM_000124.4	108
PDLIM7	protein_coding	Yes	NM_005451.5	108
GPR160	protein_coding	Yes	NM_014373.3	108
PLS3	protein_coding	Yes	NM_005032.7	108
GTF2E2	protein_coding	Yes	NM_002095.6	108
COL27A1	protein_coding	Yes	NM_032888.4	108
NPEPL1	protein_coding	Yes	NM_024663.4	108
PXK	protein_coding	Yes	NM_017771.5	108
ACBD7	protein_coding	Yes	NM_001039844.3	108
RANGAP1	protein_coding	Yes	NM_002883.4	108
FAM149A	protein_coding	Yes	-	108
SPRED2	protein_coding	Yes	NM_181784.3	108
PITPNM1	protein_coding	Yes	NM_004910.3	108
MYOM1	protein_coding	Yes	NM_003803.4	108
SAA1	protein_coding	Yes	NM_199161.5	108
H2BC7	protein_coding	Yes	NM_003522.4	108
MEGF6	protein_coding	Yes	NM_001409.4	108
BDNF	protein_coding	Yes	NM_001709.5	108
BTN2A2	protein_coding	Yes	NM_006995.5	108
SLC39A8	protein_coding	Yes	NM_001135146.2	108
CACNA2D1	protein_coding	Yes	NM_000722.4	108
BICD2	protein_coding	Yes	NM_001003800.2	108

ENST00000356950	2388.4401	-1.562508003	0.170638	-9.156833	5.34E-20	1.98E-17	ENSG00000197903	6	27146360	27146855
ENST00000356956	3489.5399	1.121665926	0.167575	6.693524	2.18E-11	2.11E-09	ENSG00000197081	6	159969081	160111504
ENST00000356978	2348.3755	-1.049094288	0.172507	-6.081448	1.19E-09	7.61E-08	ENSG00000198668	14	90397028	90408268
ENST00000356994	2436.4034	-1.298096616	0.172658	-7.518303	5.55E-14	8.87E-12	ENSG00000180900	8	143790924	143815773
ENST00000357060	70.923381	3.000287709	0.458043	6.550235	5.74E-11	5.09E-09	ENSG00000197748	10	104129887	104232364
ENST00000357068	84.587437	-2.381134101	0.396663	-6.002921	1.94E-09	1.16E-07	ENSG00000100033	22	18912780	18936293
ENST00000357162	2616.9229	1.090269264	0.201797	5.402815	6.56E-08	2.60E-06	ENSG00000132549	8	99013273	99877580
ENST00000357175	9.9673236	-6.789424446	1.72795	-3.92918	8.52E-05	0.001167093	ENSG00000157502	X	106168304	106208956
ENST00000357195	10.022718	-6.800561588	1.728492	-3.934389	8.34E-05	0.001147638	ENSG00000127152	14	99169286	99272197
ENST00000357242	9.6526078	-6.744075694	1.72161	-3.917307	8.95E-05	0.00121516	ENSG00000133138	X	106802672	106876150
ENST00000357430	5028.8647	-1.032298178	0.166806	-6.188617	6.07E-10	4.18E-08	ENSG00000123983	2	222861035	222944639
ENST00000357560	153.5941	1.266500102	0.299171	4.233363	2.30E-05	0.000391525	ENSG00000113108	5	140558267	140564598
ENST00000357727	26.115333	1.879498008	0.641079	2.931771	0.003370354	0.022017963	ENSG00000146592	7	28412517	28825894
ENST00000357742	254.83519	-1.178749624	0.275152	-4.283995	1.84E-05	0.0003257	ENSG00000140563	15	94231560	94483952
ENST00000357814	612.00982	1.205433439	0.201057	5.995478	2.03E-09	1.21E-07	ENSG00000109065	17	74770528	74776345
ENST00000357847	386.32902	1.171606147	0.23352	5.017157	5.24E-07	1.55E-05	ENSG00000146416	6	143060888	143341058
ENST00000357947	101.6739	2.225256151	0.368311	6.041787	1.52E-09	9.41E-08	ENSG00000095587	10	96364607	96513926
ENST00000357998	354.72919	-1.972766209	0.240682	-8.196571	2.47E-16	5.83E-14	ENSG00000106692	9	105558130	105641118
ENST00000358015	4504.9574	-1.616218782	0.170901	-9.457046	3.17E-21	1.33E-18	ENSG00000119318	9	107283278	107332189
ENST00000358024	12.57109	-2.438899941	0.940786	-2.592405	0.009530738	0.049008831	ENSG00000133069	1	205227945	205273343
ENST00000358056	1319.169	-1.503039669	0.180999	-8.304136	1.01E-16	2.46E-14	ENSG00000197045	14	54474484	54488980
ENST00000358171	1163.2788	-1.93346814	0.259077	-7.462908	8.46E-14	1.30E-11	ENSG00000149257	11	75562252	75572783
ENST00000358173	462.27103	-1.883025506	0.216333	-8.704288	3.20E-18	9.46E-16	ENSG00000196411	7	100802564	100827523
ENST00000358227	109.49471	-1.422784156	0.34126	-4.16921	3.06E-05	0.000497763	ENSG00000137038	9	7796499	7799778
ENST00000358242	15.205343	-2.359332866	0.876004	-2.693291	0.007075058	0.039191565	ENSG00000158856	8	22056813	22082525
ENST00000358299	30.55415	1.84877455	0.629457	2.937096	0.003313017	0.021763729	ENSG00000163596	2	202773175	202871766
ENST00000358385	96.52853	1.331863076	0.366535	3.633657	0.000279432	0.003106762	ENSG00000198513	14	50560144	50633045
ENST00000358399	28.404117	-3.772966686	0.760452	-4.961479	7.00E-07	1.99E-05	ENSG00000119125	9	72149439	72252224
ENST00000358432	2132.8147	-1.004080243	0.195505	-5.135819	2.81E-07	9.05E-06	ENSG00000142627	1	16124336	16156069
ENST00000358572	314.24424	-1.631111955	0.254294	-6.414272	1.41E-10	1.17E-08	ENSG00000198498	4	163494689	163520539
ENST00000358582	41.469454	2.026419493	0.561643	3.608019	0.000308544	0.003362794	ENSG00000290988	19	37667750	37692315
ENST00000358656	197.3694	-1.140803376	0.269017	-4.240637	2.23E-05	0.000381561	ENSG00000181135	8	143684478	143695385
ENST00000358739	2041.1059	-1.734207273	0.176825	-9.807479	1.05E-22	5.69E-20	ENSG00000196747	6	27808172	27808667
ENST00000358752	216.40922	-1.553139045	0.263583	-5.892409	3.81E-09	2.11E-07	ENSG00000196371	11	94543920	94549895

H2BC12	protein_coding	Yes	NM_001312653.2	108
IGF2R	protein_coding	Yes	NM_000876.4	108
CALM1	protein_coding	Yes	NM_006888.6	108
SCRIB	protein_coding	Yes	NM_182706.5	108
CFAP43	protein_coding	Yes	NM_025145.7	108
PRODH	protein_coding	Yes	NM_016335.6	108
VPS13B	protein_coding	Yes	NM_152564.5	108
PWWP3B	protein_coding	Yes	NM_001171020.2	108
BCL11B	protein_coding	Yes	NM_138576.4	108
TBC1D8B	protein_coding	Yes	NM_017752.3	108
ACSL3	protein_coding	Yes	NM_004457.5	108
APBB3	protein_coding	Yes	NM_133173.3	108
CREB5	protein_coding	Yes	NM_182898.4	108
MCTP2	protein_coding	Yes	NM_001385001.1	108
NAT9	protein_coding	Yes	NM_015654.5	108
AIG1	protein_coding	Yes	NM_016108.4	108
TLL2	protein_coding	Yes	NM_012465.4	108
FKTN	protein_coding	Yes	NM_001079802.2	108
RAD23B	protein_coding	Yes	NM_002874.5	108
TMCC2	protein_coding	Yes	NM_014858.4	108
GMFB	protein_coding	Yes	NM_004124.3	108
SERPINH1	protein_coding	Yes	NM_001235.5	108
EPHB4	protein_coding	Yes	NM_004444.5	108
DMAC1	protein_coding	Yes	NM_033428.3	108
DMTN	protein_coding	Yes	NM_001387751.1	108
ICA1L	protein_coding	Yes	NM_001288622.3	108
ATL1	protein_coding	Yes	NM_015915.5	108
GDA	protein_coding	Yes	NM_004293.5	108
EPHA2	protein_coding	Yes	NM_004431.5	108
TMA16	protein_coding	Yes	NM_018352.3	108
ZNF781	lncRNA	Yes	-	108
ZNF707	protein_coding	Yes	NM_001100598.2	108
H2AC13	protein_coding	Yes	NM_003509.3	108
FUT4	protein_coding	Yes	NM_002033.4	108

ENST00000358763	14.964704	4.211854158	1.130537	3.725535	0.000194901	0.002318538	ENSG00000185666	22	32507819	33058381
ENST00000358771	48.217549	-1.55509808	0.474303	-3.2787	0.001042864	0.008762068	ENSG00000198053	20	1895406	1940592
ENST00000358776	640.21739	1.163332638	0.233341	4.985553	6.18E-07	1.79E-05	ENSG00000196177	10	123009005	123058290
ENST00000358812	247.03791	-1.663062547	0.300003	-5.543484	2.97E-08	1.28E-06	ENSG00000172469	6	95577534	95609452
ENST00000358869	284.26937	-1.802963608	0.269286	-6.695342	2.15E-11	2.09E-09	ENSG00000197712	4	38867805	38945739
ENST00000358894	8.0996626	4.948174263	1.746296	2.833526	0.004603761	0.028095772	ENSG00000101203	20	63293185	63334806
ENST00000358913	6.3199684	6.085074819	1.847495	3.293689	0.000988818	0.008410912	ENSG00000138347	10	68109487	68212017
ENST00000358987	79.496467	1.42299391	0.39119	3.637599	0.000275192	0.003069542	ENSG00000197054	19	11965039	11980617
ENST00000359003	365.32953	1.046331644	0.265803	3.936491	8.27E-05	0.001139281	ENSG00000198646	20	34714773	34825651
ENST00000359128	28.78416	8.271767527	1.545834	5.351008	8.75E-08	3.33E-06	ENSG00000167984	16	3539032	3577403
ENST00000359227	9.7745967	6.713323002	1.71565	3.912992	9.12E-05	0.001232477	ENSG00000196361	19	11451325	11481046
ENST00000359246	540.514	-1.03447711	0.208765	-4.955235	7.22E-07	2.05E-05	ENSG00000197724	9	93576583	93679587
ENST00000359263	13737.576	1.055564676	0.167974	6.284079	3.30E-10	2.45E-08	ENSG00000138119	10	93306428	93482334
ENST00000359303	1042.4963	-1.539401393	0.185141	-8.314749	9.19E-17	2.27E-14	ENSG00000197153	6	27890314	27890826
ENST00000359314	2300.8501	-1.067206753	0.176776	-6.037066	1.57E-09	9.62E-08	ENSG00000198087	6	47477788	47627263
ENST00000359365	120.74811	1.408331489	0.332854	4.231077	2.33E-05	0.000394688	ENSG00000068028	3	50329787	50340836
ENST00000359429	1160.696	-1.452000737	0.184375	-7.875272	3.40E-15	6.83E-13	ENSG00000197713	2	210002637	210022260
ENST00000359441	12.906445	7.117554916	1.680965	4.234208	2.29E-05	0.00039025	ENSG00000158815	8	22042819	22048809
ENST00000359600	105.13708	1.384912905	0.403397	3.43313	0.000596657	0.005655289	ENSG00000162415	1	45016398	45206605
ENST00000359611	1597.5617	-1.01978653	0.174159	-5.855484	4.76E-09	2.59E-07	ENSG00000278677	6	27892698	27893185
ENST00000359623	131.92175	1.268563919	0.356174	3.561644	0.00036854	0.003866663	ENSG00000127399	7	150323354	150338156
ENST00000359697	99.495333	-1.383256369	0.353164	-3.916758	8.97E-05	0.001216953	ENSG00000127952	7	75996341	76048000
ENST00000359858	734.98019	-1.26634212	0.210728	-6.009356	1.86E-09	1.12E-07	ENSG00000197056	1	35079384	35115859
ENST00000360403	316.87256	-1.03600974	0.2432	-4.259912	2.05E-05	0.000355319	ENSG00000070785	1	44850521	44986595
ENST00000360465	23.230179	2.722672614	0.739241	3.683064	0.000230447	0.00265357	ENSG00000167555	19	52397848	52418401
ENST00000360482	37.085525	2.341532193	0.581502	4.026698	5.66E-05	0.00083434	ENSG00000134668	1	31790421	31816022
ENST00000360509	955.58934	-1.584568963	0.211093	-7.506485	6.07E-14	9.60E-12	ENSG00000166415	15	53513740	53759663
ENST00000360534	48.052147	-4.210310748	0.635793	-6.622137	3.54E-11	3.28E-09	ENSG00000197635	2	161992244	162074215
ENST00000360589	5.8548263	5.974190633	1.874526	3.187041	0.001437365	0.011313862	ENSG00000144031	2	70978629	70985499
ENST00000360631	60.070216	-7.948332545	2.422343	-3.281259	0.001033449	0.008696869	ENSG00000133083	13	35768651	36131382
ENST00000360668	279.99779	1.890860124	0.264309	7.153985	8.43E-13	1.07E-10	ENSG00000101460	20	34558717	34560345
ENST00000360768	329.80998	-1.13489964	0.23096	-4.913831	8.93E-07	2.45E-05	ENSG00000196715	7	65873073	65959558
ENST00000360774	39.329911	-1.609106315	0.554476	-2.90203	0.003707535	0.023771365	ENSG00000119121	9	74722494	74887921
ENST00000360779	86.447947	1.875103153	0.38072	4.925153	8.43E-07	2.34E-05	ENSG00000125775	20	1309908	1329139

SYN3	protein_coding	Yes	NM_003490.4	108
SIRPA	protein_coding	Yes	NM_001040023.2	108
ACADSB	protein_coding	Yes	NM_001609.4	108
MANEA	protein_coding	Yes	NM_024641.4	108
FAM114A1	protein_coding	Yes	NM_138389.4	108
COL20A1	protein_coding	Yes	NM_020882.4	108
MYPN	protein_coding	Yes	NM_032578.4	108
ZNF763	protein_coding	Yes	NM_001367172.2	108
NCOA6	protein_coding	Yes	NM_014071.5	108
NLRC3	protein_coding	Yes	NM_178844.4	108
ELAVL3	protein_coding	Yes	NM_001420.4	108
PHF2	protein_coding	Yes	NM_005392.4	108
MYOF	protein_coding	Yes	NM_013451.4	108
H3C12	protein_coding	Yes	NM_003535.3	108
CD2AP	protein_coding	Yes	NM_012120.3	108
RASSF1	protein_coding	Yes	NM_007182.5	108
RPE	protein_coding	Yes	NM_199229.3	108
FGF17	protein_coding	Yes	NM_003867.4	108
ZSWIM5	protein_coding	Yes	NM_020883.2	108
H2AC17	protein_coding	Yes	NM_003514.2	108
LRRC61	protein_coding	Yes	NM_001142928.2	108
STYXL1	protein_coding	Yes	NM_001317785.2	108
ZMYM1	protein_coding	Yes	NM_024772.5	108
EIF2B3	protein_coding	Yes	NM_020365.5	108
ZNF528	protein_coding	Yes	NM_032423.3	108
SPOCD1	protein_coding	Yes	NM_144569.7	108
WDR72	protein_coding	Yes	NM_182758.4	108
DPP4	protein_coding	Yes	NM_001935.4	108
ANKRD53	protein_coding	Yes	NM_001115116.2	108
DCLK1	protein_coding	Yes	NM_001330071.2	108
MAP1LC3A	protein_coding	Yes	NM_032514.4	108
VKORC1L1	protein_coding	Yes	NM_173517.6	108
TRPM6	protein_coding	Yes	NM_017662.5	108
SDCBP2	protein_coding	Yes	NM_080489.5	108

ENST00000360947	300.99194	-1.27659855	0.239297	-5.334784	9.57E-08	3.60E-06	ENSG00000165804	14	21090076	21098655
ENST00000360986	10.337434	-6.844212568	1.705449	-4.013145	5.99E-05	0.000872514	ENSG00000109625	4	8592764	8619752
ENST00000361066	161.6237	1.911586094	0.339873	5.624419	1.86E-08	8.42E-07	ENSG00000134202	1	109733936	109741038
ENST00000361131	188.71794	-2.274200002	0.338136	-6.72569	1.75E-11	1.74E-09	ENSG00000198729	6	150143043	150250392
ENST00000361138	1630.404	-1.208707967	0.196292	-6.157703	7.38E-10	4.98E-08	ENSG00000198887	9	70258977	70354873
ENST00000361143	63.373851	1.425811322	0.419808	3.396339	0.000682936	0.006297012	ENSG00000114779	3	51968514	51974049
ENST00000361170	523.61174	-1.347329942	0.219701	-6.132555	8.65E-10	5.73E-08	ENSG00000183856	1	156525404	156572565
ENST00000361362	36.322545	2.180365849	0.562838	3.873881	0.000107116	0.001409851	ENSG00000198939	5	178895912	178933212
ENST00000361418	492.91538	-1.331022412	0.220624	-6.032991	1.61E-09	9.84E-08	ENSG00000139116	12	39293227	39443120
ENST00000361428	611.84066	1.612869766	0.211033	7.642751	2.13E-14	3.76E-12	ENSG00000171467	6	43336069	43369647
ENST00000361490	229.38034	1.128634057	0.258322	4.369095	1.25E-05	0.000234494	ENSG00000107521	10	98416197	98446935
ENST00000361565	1419.7246	-1.161212418	0.176539	-6.577654	4.78E-11	4.32E-09	ENSG00000198700	1	201829156	201884291
ENST00000361618	813.1622	-1.59875147	0.208948	-7.651421	1.99E-14	3.54E-12	ENSG00000198948	4	169986601	170026395
ENST00000361627	1662.2923	-1.67162681	0.21639	-7.725079	1.12E-14	2.09E-12	ENSG00000198826	15	32615503	32639941
ENST00000361675	839.07386	-1.324411289	0.189916	-6.973652	3.09E-12	3.52E-10	ENSG00000122786	7	134779633	134970729
ENST00000361717	247.17136	-1.078213137	0.253631	-4.251113	2.13E-05	0.000367027	ENSG00000145365	4	112274536	112285904
ENST00000361729	123.09498	-2.121549093	0.356385	-5.952966	2.63E-09	1.52E-07	ENSG00000007968	1	23506437	23531233
ENST00000361794	74.652388	-1.876181442	0.410547	-4.569957	4.88E-06	0.000105851	ENSG00000198945	6	130018580	130141438
ENST00000361840	166.73106	-1.27001471	0.292388	-4.343593	1.40E-05	0.000259142	ENSG00000123178	13	49912701	49936340
ENST00000361842	108.07096	1.775455681	0.368439	4.818864	1.44E-06	3.71E-05	ENSG00000132530	17	6756045	6775647
ENST00000361866	129.60618	-3.02510484	0.346977	-8.718463	2.82E-18	8.58E-16	ENSG00000142156	21	45981769	46005048
ENST00000361910	59.408937	-9.366543764	1.498474	-6.250721	4.09E-10	2.93E-08	ENSG00000198960	X	101615124	101618000
ENST00000361927	18.091141	2.181352697	0.792525	2.752408	0.005915872	0.034110846	ENSG00000198756	1	183935833	184037728
ENST00000362477	37.28567	-1.671876303	0.556252	-3.00561	0.002650489	0.018325336	ENSG00000199347	1	11908151	11908271
ENST00000362512	49.260504	-1.689788163	0.480811	-3.514456	0.000440656	0.00446429	ENSG00000276027	22	42615243	42615393
ENST00000362698	43.397045	-2.079435839	0.51444	-4.042135	5.30E-05	0.000791092	ENSG00000199568	15	65296050	65296166
ENST00000363009	12.191068	-3.935450412	1.187994	-3.312685	0.000924048	0.007990192	ENSG00000199879	1	148263475	148263632
ENST00000363450	64.292639	-1.107388232	0.422823	-2.619034	0.00881791	0.046222767	ENSG00000200320	3	186787299	186787431
ENST00000363593	29.847505	-1.559132742	0.588982	-2.647165	0.008116983	0.043505326	ENSG00000200463	17	8173453	8173587
ENST00000364043	45.836036	2.163694736	0.50465	4.287512	1.81E-05	0.000321373	ENSG00000200913	1	44776489	44776593
ENST00000364127	11.884517	-7.044934281	1.672044	-4.213366	2.52E-05	0.000422493	ENSG00000200997	17	58679526	58679690
ENST00000364313	7.8454031	-6.445625875	1.776181	-3.628923	0.000284606	0.003153546	ENSG00000201183	1	148402714	148402875
ENST00000364488	18.104109	7.602343856	1.598864	4.754841	1.99E-06	4.91E-05	ENSG00000201358	14	50068567	50068873
ENST00000364688	16.626828	-3.368799636	0.912437	-3.692089	0.00022242	0.002578673	ENSG00000201558	1	146052080	146052244

ZNF219	protein_coding	Yes	NM_016423.3	108
CPZ	protein_coding	Yes	NM_001014447.3	108
GSTM3	protein_coding	Yes	NM_000849.5	108
PPP1R14C	protein_coding	Yes	NM_030949.3	108
SMC5	protein_coding	Yes	NM_015110.4	108
ABHD14B	protein_coding	Yes	NM_001146314.2	108
IQGAP3	protein_coding	Yes	NM_178229.5	108
ZFP2	protein_coding	Yes	NM_030613.4	108
KIF21A	protein_coding	Yes	NM_001173464.2	108
ZNF318	protein_coding	Yes	NM_014345.3	108
HPS1	protein_coding	Yes	NM_000195.5	108
IPO9	protein_coding	Yes	NM_018085.5	108
MFAP3L	protein_coding	Yes	NM_021647.8	108
ARHGAP11A	protein_coding	Yes	NM_014783.6	108
CALD1	protein_coding	Yes	NM_033138.4	108
TIFA	protein_coding	Yes	NM_052864.3	108
E2F2	protein_coding	Yes	NM_004091.4	108
L3MBTL3	protein_coding	Yes	NM_032438.4	108
SPRYD7	protein_coding	Yes	NM_020456.4	108
XAF1	protein_coding	Yes	NM_017523.5	108
COL6A1	protein_coding	Yes	NM_001848.3	108
ARMCX6	protein_coding	Yes	NM_019007.4	108
COLGALT2	protein_coding	Yes	NM_015101.4	108
RNU5E-1	snRNA	Yes	-	108
RNU12	snRNA	Yes	-	108
RNU5A-1	snRNA	Yes	-	108
RNVU1-22	snRNA	Yes	-	108
SNORA63	snoRNA	Yes	-	108
SNORD118	snoRNA	Yes	-	108
SNORD46	snoRNA	Yes	-	108
RNVU1-34	snRNA	Yes	-	108
RNVU1-3	snRNA	Yes	-	108
RN7SKP193	misc_RNA	Yes	-	108
RNVU1-6	snRNA	Yes	-	108

ENST00000364923	20.492864	2.015938253	0.726596	2.774496	0.005528737	0.032365676	ENSG00000201793	13	99205707	99206006
ENST00000365031	30.156995	1.70334806	0.594504	2.86516	0.004167982	0.026037887	ENSG00000201901	4	85100495	85100823
ENST00000365574	4.0527625	-5.492532258	2.038239	-2.694745	0.007044262	0.039077783	ENSG00000202444	1	44819882	44819997
ENST00000365668	57.343809	1.34322016	0.436515	3.077146	0.002089929	0.015196232	ENSG00000202538	12	120291762	120291903
ENST00000366523	53.649154	-1.310375827	0.447435	-2.928638	0.003404511	0.022197012	ENSG00000203666	1	244969681	245087837
ENST00000366534	73.615251	3.612061812	0.475085	7.602976	2.89E-14	4.95E-12	ENSG00000179397	1	244461272	244640504
ENST00000366559	24.633811	3.116439645	0.749789	4.156423	3.23E-05	0.000522348	ENSG00000117009	1	241532377	241595642
ENST00000366691	810.06853	-1.223474354	0.213099	-5.741334	9.39E-09	4.70E-07	ENSG00000116574	1	228735478	228746664
ENST00000366843	4791.4129	-1.035743524	0.187128	-5.534947	3.11E-08	1.34E-06	ENSG00000154380	1	225486828	225653123
ENST00000366910	84.037156	-4.273591791	0.520525	-8.210164	2.21E-16	5.24E-14	ENSG00000186205	1	220786912	220819659
ENST00000366932	446.59826	-1.11806792	0.256574	-4.357683	1.31E-05	0.000245273	ENSG00000067533	1	218285292	218337983
ENST00000366958	10.007168	6.747441554	1.709973	3.945935	7.95E-05	0.001103172	ENSG00000117707	1	213987972	214041510
ENST00000366963	11.203525	2.987237096	1.077682	2.77191	0.005572852	0.032561393	ENSG00000175003	6	160121814	160158718
ENST00000366977	406.06636	-1.048815589	0.226823	-4.623948	3.77E-06	8.50E-05	ENSG00000117697	1	212726152	212791777
ENST00000367001	886.46102	-1.637526626	0.190158	-8.611393	7.22E-18	2.06E-15	ENSG00000170385	1	211571567	211579161
ENST00000367033	207.55167	-2.198213397	0.298687	-7.359588	1.84E-13	2.69E-11	ENSG00000076356	1	208022241	208244384
ENST00000367142	5351.7816	-1.786886522	0.164952	-10.83279	2.41E-27	2.34E-24	ENSG00000069275	1	205712821	205750182
ENST00000367194	16.605736	-6.059834508	1.598495	-3.790962	0.000150065	0.001869965	ENSG00000170498	1	204190340	204196491
ENST00000367202	140.01714	-1.433321983	0.313301	-4.574903	4.76E-06	0.00010385	ENSG00000143845	1	204131061	204152044
ENST00000367339	48.691617	-1.832296058	0.497022	-3.68655	0.000227315	0.002624006	ENSG00000131019	6	150061052	150069121
ENST00000367350	1935.6989	-1.034236901	0.171928	-6.015533	1.79E-09	1.08E-07	ENSG00000118193	1	200551499	200620751
ENST00000367409	4261.1336	-1.165625335	0.164919	-7.067863	1.57E-12	1.90E-10	ENSG00000066279	1	197084126	197146669
ENST00000367463	82.387967	-3.153959397	0.426834	-7.389188	1.48E-13	2.19E-11	ENSG00000111962	6	148747029	149076990
ENST00000367466	92.369621	-3.944369276	0.461384	-8.548995	1.24E-17	3.41E-15	ENSG00000116711	1	186828948	186988981
ENST00000367468	18.431057	-3.072960035	0.835811	-3.676623	0.000236342	0.002708969	ENSG00000073756	1	186671790	186680423
ENST00000367500	229.89456	1.954747827	0.318087	6.14533	7.98E-10	5.33E-08	ENSG00000116668	1	185157166	185291781
ENST00000367510	233.81494	-1.335327958	0.289487	-4.612732	3.97E-06	8.87E-05	ENSG00000121481	1	185045557	185102603
ENST00000367511	103.20911	-2.199713283	0.382251	-5.754637	8.68E-09	4.41E-07	ENSG00000135842	1	184791031	184974508
ENST00000367519	87.735596	1.815449635	0.383689	4.731564	2.23E-06	5.42E-05	ENSG00000112425	6	145625304	145735520
ENST00000367603	1309.6812	1.405229976	0.184745	7.606329	2.82E-14	4.84E-12	ENSG00000010818	6	142751468	142945176
ENST00000367635	1013.7093	-1.444383186	0.223393	-6.465648	1.01E-10	8.56E-09	ENSG00000116191	1	178725243	178921840
ENST00000367942	700.47766	-1.176221073	0.202412	-5.811021	6.21E-09	3.28E-07	ENSG00000118217	1	161766319	161964070
ENST00000367943	249.37594	-2.122736595	0.280389	-7.570698	3.71E-14	6.20E-12	ENSG00000081721	1	161749785	161757238
ENST00000368003	1270.744	1.540592154	0.19627	7.849336	4.18E-15	8.30E-13	ENSG00000143222	1	161153977	161158856

RN7SKP9	misc_RNA	Yes	-	108
RN7SKP48	misc_RNA	Yes	-	108
RNU5E-6P	snRNA	Yes	-	108
RNU4-2	snRNA	Yes	-	108
EFCAB2	protein_coding	Yes	NM_032328.4	108
CATSPERE	protein_coding	Yes	NM_001130957.2	108
KMO	protein_coding	Yes	NM_003679.5	108
RHOU	protein_coding	Yes	NM_021205.6	108
ENAH	protein_coding	Yes	NM_018212.6	108
MTARC1	protein_coding	Yes	NM_022746.4	108
RRP15	protein_coding	Yes	NM_016052.4	108
PROX1	protein_coding	Yes	NM_001270616.2	108
SLC22A1	protein_coding	Yes	NM_003057.3	108
NSL1	protein_coding	Yes	NM_015471.4	108
SLC30A1	protein_coding	Yes	NM_021194.3	108
PLXNA2	protein_coding	Yes	NM_025179.4	108
NUCKS1	protein_coding	Yes	NM_022731.5	108
KISS1	protein_coding	Yes	NM_002256.4	108
ETNK2	protein_coding	Yes	NM_018208.4	108
ULBP3	protein_coding	Yes	NM_024518.3	108
KIF14	protein_coding	Yes	NM_014875.3	108
ASPM	protein_coding	Yes	NM_018136.5	108
UST	protein_coding	Yes	NM_005715.3	108
PLA2G4A	protein_coding	Yes	NM_024420.3	108
PTGS2	protein_coding	Yes	NM_000963.4	108
SWT1	protein_coding	Yes	NM_017673.7	108
RNF2	protein_coding	Yes	NM_007212.4	108
NIBAN1	protein_coding	Yes	NM_052966.4	108
EPM2A	protein_coding	Yes	NM_005670.4	108
HIVEP2	protein_coding	Yes	NM_006734.4	108
RALGPS2	protein_coding	Yes	NM_152663.5	108
ATF6	protein_coding	Yes	NM_007348.4	108
DUSP12	protein_coding	Yes	NM_007240.3	108
UFC1	protein_coding	Yes	NM_016406.4	108

ENST00000368009	359.46651	1.014107406	0.252981	4.008625	6.11E-05	0.00088557	ENSG00000158793	1	161118104	161121194
ENST00000368223	63.256728	-3.505290275	0.498092	-7.03744	1.96E-12	2.31E-10	ENSG00000132688	1	156668762	156677407
ENST00000368232	205.96956	-1.454346671	0.312007	-4.661257	3.14E-06	7.25E-05	ENSG00000160818	1	156594300	156601479
ENST00000368321	677.93461	1.011771763	0.197931	5.111737	3.19E-07	1.01E-05	ENSG00000132680	1	155913044	155934413
ENST00000368324	26.40534	3.583657573	0.766949	4.672613	2.97E-06	6.91E-05	ENSG00000132718	1	155859566	155885199
ENST00000368373	810.81373	1.904607535	0.209847	9.076178	1.12E-19	4.05E-17	ENSG00000177628	1	155234451	155241249
ENST00000368378	211.39847	1.498851746	0.371509	4.034492	5.47E-05	0.000812549	ENSG00000169231	1	155195587	155207897
ENST00000368440	38.708138	-1.52415427	0.532108	-2.864372	0.004178372	0.026091031	ENSG00000172594	6	122789257	122809720
ENST00000368468	146.55378	2.575417082	0.325185	7.919861	2.38E-15	4.86E-13	ENSG00000111885	6	119177204	119349761
ENST00000368607	172.05324	1.311120367	0.284462	4.609131	4.04E-06	9.01E-05	ENSG00000143578	1	153967901	153974359
ENST00000368633	130.55556	1.04722389	0.34153	3.066274	0.002167443	0.015635276	ENSG00000160741	1	153947674	153958612
ENST00000368685	239.24841	-1.082801947	0.25176	-4.300935	1.70E-05	0.000305575	ENSG00000143553	1	153658702	153661852
ENST00000368706	855.80738	-1.232909608	0.222079	-5.551679	2.83E-08	1.23E-06	ENSG00000188643	1	153606885	153613137
ENST00000368719	1120.0259	-1.05778118	0.19757	-5.353952	8.61E-08	3.29E-06	ENSG00000197956	1	153534598	153535991
ENST00000368738	118.10643	-3.821435703	0.412641	-9.260932	2.03E-20	7.76E-18	ENSG00000163220	1	153357853	153361023
ENST00000368830	436.96636	-1.138186625	0.229957	-4.949564	7.44E-07	2.10E-05	ENSG00000143436	1	151759646	151763496
ENST00000368836	445.15863	-2.150053531	0.221303	-9.715441	2.59E-22	1.31E-19	ENSG00000203791	10	124757833	124791887
ENST00000368851	110.29302	1.657030631	0.33774	4.906239	9.28E-07	2.53E-05	ENSG00000112394	6	111087502	111231194
ENST00000368914	50.169412	2.988708113	0.554726	5.387716	7.14E-08	2.79E-06	ENSG00000143434	1	151131706	151146631
ENST00000368921	162.85655	1.621272359	0.292105	5.550306	2.85E-08	1.23E-06	ENSG00000213190	1	151060396	151069544
ENST00000368924	7.0176815	6.236877671	1.814974	3.436345	0.00058962	0.005607074	ENSG00000203797	6	110391783	110415575
ENST00000368966	15.21985	2.508488866	0.86717	2.89273	0.00381909	0.024326967	ENSG00000203799	6	109183954	109355046
ENST00000369005	244.3688	1.477938291	0.273034	5.41302	6.20E-08	2.48E-06	ENSG00000138162	10	121989162	122254542
ENST00000369155	2026.3869	-1.722190991	0.196061	-8.783973	1.58E-18	4.98E-16	ENSG00000184678	1	149884458	149886682
ENST00000369158	3028.0354	-1.428066804	0.171484	-8.327678	8.24E-17	2.05E-14	ENSG00000203811	1	149840686	149841208
ENST00000369159	5247.5374	-1.523264604	0.165672	-9.194475	3.77E-20	1.41E-17	ENSG00000288825	1	149842217	149842750
ENST00000369167	3065.6176	-1.538993042	0.172446	-8.924504	4.48E-19	1.52E-16	ENSG00000203814	1	149811878	149812370
ENST00000369448	66.517929	1.165370897	0.406624	2.86597	0.00415734	0.026000322	ENSG00000183508	1	117606047	117628389
ENST00000369500	18.756623	2.657292619	0.863625	3.076907	0.002091604	0.015204057	ENSG00000173212	1	116111398	116138149
ENST00000369516	85.113302	-1.075834913	0.365217	-2.945744	0.003221786	0.021291982	ENSG00000134198	1	115048010	115089503
ENST00000369534	106.51228	1.458776555	0.343046	4.252423	2.11E-05	0.000365133	ENSG00000130830	X	154778683	154805485
ENST00000369684	80.190907	2.826340331	0.428911	6.589567	4.41E-11	4.01E-09	ENSG00000134245	1	112508964	112530165
ENST00000369774	94.850904	-1.934051663	0.396588	-4.876732	1.08E-06	2.88E-05	ENSG00000107957	10	103594026	103855576
ENST00000369779	70.764512	1.399514556	0.487112	2.873084	0.004064856	0.025556008	ENSG00000168679	1	110362856	110391026

NIT1	protein_coding	Yes	NM_005600.3	108
NES	protein_coding	Yes	NM_006617.2	108
GPATCH4	protein_coding	Yes	-	108
KHDC4	protein_coding	Yes	NM_014949.4	108
SYT11	protein_coding	Yes	NM_152280.5	108
GBA	protein_coding	Yes	NM_000157.4	108
THBS3	protein_coding	Yes	NM_007112.5	108
SMPDL3A	protein_coding	Yes	NM_006714.5	108
MAN1A1	protein_coding	Yes	NM_005907.4	108
CREB3L4	protein_coding	Yes	NM_001255978.2	108
CRTC2	protein_coding	Yes	NM_181715.3	108
SNAPIN	protein_coding	Yes	NM_012437.6	108
S100A16	protein_coding	Yes	NM_080388.3	108
S100A6	protein_coding	Yes	NM_014624.4	108
S100A9	protein_coding	Yes	NM_002965.4	108
MRPL9	protein_coding	Yes	NM_031420.4	108
EEF1AKMT2	protein_coding	Yes	NM_212554.4	108
SLC16A10	protein_coding	Yes	NM_018593.5	108
SEMA6C	protein_coding	Yes	NM_030913.6	108
MLLT11	protein_coding	Yes	NM_006818.4	108
DDO	protein_coding	Yes	NM_001372108.2	108
CCDC162P	ed_unitary_pse	Yes	-	108
TACC2	protein_coding	Yes	NM_206862.4	108
H2BC21	protein_coding	Yes	NM_003528.3	108
H3C14	protein_coding	Yes	NM_021059.3	108
H2AC18	protein_coding	Yes	NM_003516.3	108
H2BC18	protein_coding	Yes	NM_001024599.5	108
TENT5C	protein_coding	Yes	NM_017709.4	108
MAB21L3	protein_coding	Yes	NM_152367.3	108
TSPAN2	protein_coding	Yes	NM_005725.6	108
MPP1	protein_coding	Yes	NM_002436.4	108
WNT2B	protein_coding	Yes	NM_024494.3	108
SH3PXD2A	protein_coding	Yes	NM_001394015.1	108
SLC16A4	protein_coding	Yes	NM_004696.3	108

ENST00000369800	186.22168	-1.136009067	0.28373	-4.003838	6.23E-05	0.000901037	ENSG00000214357	5	172641262	172691540
ENST00000369836	90.416254	2.437243351	0.407588	5.979677	2.24E-09	1.32E-07	ENSG00000168765	1	109656098	109661700
ENST00000369864	19.041905	-3.103964193	0.85146	-3.645459	0.000266915	0.002994094	ENSG00000181754	1	109504177	109509727
ENST00000369878	337.54701	1.325568138	0.245381	5.402091	6.59E-08	2.61E-06	ENSG00000148842	10	102918293	103090222
ENST00000369880	47.459271	1.70823567	0.482984	3.536834	0.000404954	0.004173615	ENSG00000214435	10	102869469	102901899
ENST00000369888	198.16269	-1.101841476	0.27061	-4.071689	4.67E-05	0.000712352	ENSG00000196700	20	63956703	63969930
ENST00000369909	344.15329	-1.025824579	0.226871	-4.521612	6.14E-06	0.000128695	ENSG00000134222	1	109279555	109283145
ENST00000369956	188.5365	2.032849701	0.287545	7.069683	1.55E-12	1.88E-10	ENSG00000107872	10	102420835	102423136
ENST00000369977	3582.0066	1.07010746	0.215898	4.956532	7.18E-07	2.04E-05	ENSG00000196586	6	75749238	75919537
ENST00000370005	51.382179	2.959125075	0.532905	5.552817	2.81E-08	1.22E-06	ENSG00000119915	10	102226298	102229589
ENST00000370035	1150.4898	-1.197285771	0.191578	-6.2496	4.12E-10	2.95E-08	ENSG00000162636	1	108560099	108639322
ENST00000370053	55.232625	-2.956156602	0.507359	-5.82656	5.66E-09	3.02E-07	ENSG00000197457	20	63639711	63653424
ENST00000370060	311.30226	2.258993748	0.298418	7.569907	3.73E-14	6.21E-12	ENSG00000198910	X	153861515	153886173
ENST00000370078	305.80364	-1.480352556	0.237416	-6.235281	4.51E-10	3.19E-08	ENSG00000198890	1	107056673	107059294
ENST00000370096	13.763371	4.092625477	1.132201	3.614751	0.000300636	0.003295236	ENSG00000060718	1	102876472	103108522
ENST00000370162	315.38507	1.036492651	0.247506	4.187745	2.82E-05	0.000465174	ENSG00000166169	10	101578881	101588257
ENST00000370192	369.46558	-2.931527774	0.242301	-12.09872	1.07E-33	1.67E-30	ENSG00000188641	1	97077742	97921034
ENST00000370205	486.07597	1.000961023	0.213819	4.681347	2.85E-06	6.67E-05	ENSG00000172339	1	94974404	95072951
ENST00000370206	2716.7174	-1.167152114	0.245025	-4.763392	1.90E-06	4.72E-05	ENSG00000117519	1	94896956	94927110
ENST00000370278	26.218199	-6.728316557	1.572202	-4.279551	1.87E-05	0.00033077	ENSG00000221867	X	152698793	152702347
ENST00000370282	4690.5042	-1.0924217	0.17927	-6.093734	1.10E-09	7.11E-08	ENSG00000117500	1	93149741	93180413
ENST00000370310	134.45242	-1.488149627	0.325713	-4.568902	4.90E-06	0.000106276	ENSG00000154511	1	92842166	92961462
ENST00000370314	146.32073	-2.084748121	0.311774	-6.686735	2.28E-11	2.20E-09	ENSG00000011677	X	152166233	152451315
ENST00000370355	6842.0714	-1.114359145	0.217712	-5.118512	3.08E-07	9.80E-06	ENSG00000099194	10	100347232	100364826
ENST00000370435	677.0556	-1.734068802	0.202253	-8.573742	1.00E-17	2.77E-15	ENSG00000119900	6	71288810	71309059
ENST00000370501	7.8178522	-4.961274561	1.751445	-2.832676	0.004616019	0.028159303	ENSG00000101188	20	62708835	62762771
ENST00000370548	725.48094	-1.175372669	0.194311	-6.048932	1.46E-09	9.07E-08	ENSG00000267561	1	86993008	87169204
ENST00000370550	3534.8975	-1.319776088	0.180164	-7.325404	2.38E-13	3.40E-11	ENSG00000153936	1	86914634	87109982
ENST00000370589	154.64415	-2.714613065	0.332798	-8.156946	3.44E-16	7.92E-14	ENSG00000055732	1	85018081	85048500
ENST00000370602	76.926267	2.984296013	0.437717	6.817874	9.24E-12	9.63E-10	ENSG00000155265	10	97849842	97871578
ENST00000370608	129.56337	-1.0911406	0.315378	-3.459787	0.000540603	0.005246009	ENSG00000153898	1	84925582	84997113
ENST00000370626	149.52205	1.297162272	0.297154	4.36528	1.27E-05	0.000237863	ENSG00000119986	10	97677423	97687241
ENST00000370853	5.8190218	-6.014605244	1.877722	-3.20314	0.001359378	0.010832026	ENSG00000076770	X	132369319	132489038
ENST00000370867	388.51467	-1.124104067	0.221146	-5.083086	3.71E-07	1.16E-05	ENSG00000162623	1	74733151	74766677

NEURL1B	protein_coding	Yes	NM_001142651.3	108
GSTM4	protein_coding	Yes	NM_000850.5	108
AMIGO1	protein_coding	Yes	NM_020703.4	108
CNNM2	protein_coding	Yes	NM_017649.5	108
AS3MT	protein_coding	Yes	NM_020682.4	108
ZNF512B	protein_coding	Yes	NM_020713.3	108
PSRC1	protein_coding	Yes	NM_001032291.3	108
FBXL15	protein_coding	Yes	NM_024326.4	108
MYO6	protein_coding	Yes	NM_004999.4	108
ELOVL3	protein_coding	Yes	NM_152310.3	108
FAM102B	protein_coding	Yes	NM_001010883.3	108
STMN3	protein_coding	Yes	NM_015894.4	108
L1CAM	protein_coding	Yes	NM_001278116.2	108
PRMT6	protein_coding	Yes	NM_018137.3	108
COL11A1	protein_coding	Yes	NM_001854.4	108
POLL	protein_coding	Yes	NM_001174084.2	108
DPYD	protein_coding	Yes	NM_000110.4	108
ALG14	protein_coding	Yes	NM_144988.4	108
CNN3	protein_coding	Yes	NM_001839.5	108
MAGEA3	protein_coding	Yes	NM_005362.4	108
TMED5	protein_coding	Yes	NM_016040.5	108
DIPK1A	protein_coding	Yes	NM_001006605.5	108
GABRA3	protein_coding	Yes	NM_000808.4	108
SCD	protein_coding	Yes	NM_005063.5	108
OGFRL1	protein_coding	Yes	NM_024576.5	108
NTSR1	protein_coding	Yes	NM_002531.3	108
-	protein_coding	Yes	-	108
HS2ST1	protein_coding	Yes	NM_012262.4	108
MCOLN3	protein_coding	Yes	NM_018298.11	108
GOLGA7B	protein_coding	Yes	NM_001010917.3	108
MCOLN2	protein_coding	Yes	NM_153259.4	108
AVPI1	protein_coding	Yes	NM_021732.3	108
MBNL3	protein_coding	Yes	NM_001386889.1	108
TYW3	protein_coding	Yes	NM_138467.3	108

ENST00000370938	103.79903	1.344803283	0.342141	3.930556	8.47E-05	0.001162102	ENSG00000116761	1	70411267	70439851
ENST00000370963	21.343471	2.213950795	0.723042	3.061994	0.002198681	0.0157993	ENSG00000170899	6	52977952	52995284
ENST00000370982	725.50268	-2.018065171	0.198359	-10.17379	2.60E-24	1.80E-21	ENSG00000172380	1	67701474	67833467
ENST00000371084	418.10634	-1.445617752	0.221016	-6.540794	6.12E-11	5.39E-09	ENSG00000079739	1	63593410	63660245
ENST00000371085	3052.3634	-1.018419755	0.188466	-5.403731	6.53E-08	2.59E-06	ENSG00000087460	20	58891420	58911192
ENST00000371113	336.26234	-1.232720719	0.231447	-5.326147	1.00E-07	3.74E-06	ENSG00000122126	X	129540258	129592556
ENST00000371121	510.3622	-5.234120594	0.28604	-18.29855	8.50E-75	1.01E-70	ENSG00000102038	X	129446505	129523490
ENST00000371153	151.23952	-4.451933002	0.394032	-11.29841	1.34E-29	1.53E-26	ENSG00000132854	1	62236164	62319434
ENST00000371205	24.986242	2.997001591	0.720747	4.158191	3.21E-05	0.000518893	ENSG00000138185	10	95756162	95877266
ENST00000371208	565.37313	-1.04235338	0.272088	-3.830938	0.000127656	0.001634009	ENSG00000134709	1	59814948	59876322
ENST00000371247	634.33955	1.688046474	0.214564	7.867321	3.62E-15	7.25E-13	ENSG00000095637	10	95311772	95561371
ENST00000371269	1079.5761	-2.66077599	0.209799	-12.68251	7.39E-37	1.33E-33	ENSG00000116133	1	54849626	54887195
ENST00000371281	268.59834	-1.039047728	0.255709	-4.063404	4.84E-05	0.000732991	ENSG00000243725	1	54715860	54742657
ENST00000371321	27.098505	1.717966276	0.623517	2.755283	0.00586414	0.03391233	ENSG00000165841	10	94762680	94855547
ENST00000371380	29.968091	1.615635059	0.600241	2.691642	0.007110112	0.039348817	ENSG00000138193	10	93993930	94332823
ENST00000371429	1815.2995	-1.524225091	0.175405	-8.689748	3.63E-18	1.07E-15	ENSG00000058804	1	53765477	53838296
ENST00000371505	11.036769	6.88793291	1.687885	4.080808	4.49E-05	0.000689528	ENSG00000146221	6	44278733	44297698
ENST00000371634	18.787444	2.852687722	0.818585	3.484902	0.000492317	0.004869525	ENSG00000176919	9	136945242	136946975
ENST00000371692	39.101507	-2.354254547	0.553434	-4.253904	2.10E-05	0.00036316	ENSG00000165716	9	136712571	136724742
ENST00000371696	246.65265	-1.382091298	0.261427	-5.286724	1.25E-07	4.49E-06	ENSG00000169692	9	136673142	136687457
ENST00000371755	69.211346	-2.029202664	0.419962	-4.831871	1.35E-06	3.51E-05	ENSG00000112759	6	44223594	44234142
ENST00000371850	216.83819	-3.870209029	0.359273	-10.77233	4.65E-27	4.40E-24	ENSG00000160293	9	133761893	133992324
ENST00000371899	134.88566	1.483953548	0.317657	4.671561	2.99E-06	6.93E-05	ENSG00000160326	9	133471093	133479099
ENST00000372086	180.0522	1.05547035	0.351594	3.001958	0.002682493	0.018497939	ENSG00000070759	1	45343882	45491163
ENST00000372232	277.94831	1.246576147	0.244073	5.107396	3.27E-07	1.03E-05	ENSG00000101844	X	108091777	108154671
ENST00000372289	107.8796	1.127749827	0.334878	3.367645	0.000758131	0.006840373	ENSG00000178028	1	44213470	44220673
ENST00000372318	89.695172	1.108512654	0.36367	3.048127	0.002302725	0.016393901	ENSG00000159214	1	43991598	43996528
ENST00000372371	680.2447	1.212395597	0.198442	6.109557	9.99E-10	6.52E-08	ENSG00000148606	10	77975148	78029515
ENST00000372398	273.63453	-1.855097626	0.262159	-7.076232	1.48E-12	1.80E-10	ENSG00000107130	9	130172403	130237303
ENST00000372521	207.28229	1.237629284	0.271882	4.552083	5.31E-06	0.000114072	ENSG00000177868	1	42807051	42817397
ENST00000372548	24.466494	-3.948504512	0.862726	-4.576777	4.72E-06	0.000103061	ENSG00000147231	X	106611977	106679439
ENST00000372625	372.25176	1.025678418	0.26053	3.936899	8.25E-05	0.001137965	ENSG00000172465	X	103628974	103630953
ENST00000372980	56.223368	-1.418720281	0.479416	-2.959269	0.003083699	0.020604541	ENSG00000149596	20	44106589	44187188
ENST00000372991	568.64897	-1.15144356	0.237489	-4.848415	1.24E-06	3.26E-05	ENSG00000112576	6	41934951	41941808

CTH	protein_coding	Yes	NM_001902.6	108
GSTA4	protein_coding	Yes	NM_001512.4	108
GNG12	protein_coding	Yes	NM_018841.6	108
PGM1	protein_coding	Yes	NM_002633.3	108
GNAS	protein_coding	Yes	NM_000516.7	108
OCRL	protein_coding	Yes	NM_000276.4	108
SMARCA1	protein_coding	Yes	NM_001282874.2	108
KANK4	protein_coding	Yes	NM_181712.5	108
ENTPD1	protein_coding	Yes	NM_001776.6	108
HOOK1	protein_coding	Yes	NM_015888.6	108
SORBS1	protein_coding	Yes	NM_001034954.3	108
DHCR24	protein_coding	Yes	NM_014762.4	108
TTC4	protein_coding	Yes	NM_004623.5	108
CYP2C19	protein_coding	Yes	NM_000769.4	108
PLCE1	protein_coding	Yes	NM_016341.4	108
NDC1	protein_coding	Yes	NM_018087.5	108
TCTE1	protein_coding	Yes	NM_182539.4	108
C8G	protein_coding	Yes	NM_000606.3	108
DIPK1B	protein_coding	Yes	NM_152421.4	108
AGPAT2	protein_coding	Yes	NM_006412.4	108
SLC29A1	protein_coding	Yes	NM_001372327.1	108
VAV2	protein_coding	Yes	NM_001134398.2	108
SLC2A6	protein_coding	Yes	NM_017585.4	108
TESK2	protein_coding	Yes	NM_007170.3	108
ATG4A	protein_coding	Yes	NM_052936.5	108
DMAP1	protein_coding	Yes	NM_019100.5	108
CCDC24	protein_coding	Yes	NM_152499.4	108
POLR3A	protein_coding	Yes	NM_007055.4	108
NCS1	protein_coding	Yes	NM_014286.4	108
SVBP	protein_coding	Yes	NM_199342.4	108
RADX	protein_coding	Yes	NM_018015.6	108
TCEAL1	protein_coding	Yes	NM_004780.3	108
JPH2	protein_coding	Yes	NM_020433.5	108
CCND3	protein_coding	Yes	NM_001760.5	108

ENST00000373001	538.00782	1.037254819	0.213639	4.855166	1.20E-06	3.16E-05	ENSG00000116954	1	38838197	38859772
ENST00000373055	491.8575	-1.15185057	0.2163	-5.325251	1.01E-07	3.76E-06	ENSG00000134690	1	37692515	37709719
ENST00000373100	52.854115	-1.850367236	0.467323	-3.959505	7.51E-05	0.001053168	ENSG00000101049	20	43559026	43585627
ENST00000373109	47.672497	1.849190784	0.486783	3.7988	0.000145399	0.001822696	ENSG00000107742	10	72059033	72088551
ENST00000373202	5864.8852	1.671239105	0.165995	10.06799	7.65E-24	5.03E-21	ENSG00000166224	10	70815947	70881184
ENST00000373257	184.90755	1.81864788	0.286693	6.343541	2.25E-10	1.75E-08	ENSG00000132793	20	41340820	41360582
ENST00000373279	57.972058	2.256895754	0.504823	4.470664	7.80E-06	0.000157433	ENSG00000171224	10	69630246	69633596
ENST00000373312	5133.8427	-1.102418515	0.170373	-6.470604	9.76E-11	8.31E-09	ENSG00000136830	9	127505342	127569073
ENST00000373345	125.1811	-1.445366252	0.328503	-4.399857	1.08E-05	0.000207771	ENSG00000124143	20	38601808	38650653
ENST00000373509	361.50628	-1.569194388	0.251395	-6.241951	4.32E-10	3.07E-08	ENSG00000137193	6	37170151	37175428
ENST00000373573	215.44008	-1.131116994	0.261207	-4.330351	1.49E-05	0.000272779	ENSG00000147099	X	72329515	72572843
ENST00000373580	43.22375	-1.960371122	0.528319	-3.71058	0.000206785	0.002429565	ENSG00000185585	9	124777132	124814882
ENST00000373658	7.6508019	-4.908674251	1.763972	-2.782739	0.005390212	0.03174863	ENSG00000121753	1	31727116	31764340
ENST00000373709	105.27843	-1.007976147	0.335482	-3.004559	0.002659658	0.018370625	ENSG00000168528	1	31413212	31434678
ENST00000373883	78.875209	1.813359746	0.389255	4.658544	3.18E-06	7.34E-05	ENSG00000188760	2	219544027	219550595
ENST00000373887	28.596191	2.706133742	0.659143	4.105531	4.03E-05	0.00062986	ENSG00000056558	9	120902392	120926796
ENST00000373944	1160.8556	-1.376471786	0.200156	-6.87699	6.11E-12	6.59E-10	ENSG00000122952	10	56357226	56361259
ENST00000373953	247.08315	1.814942497	0.27963	6.490514	8.55E-11	7.36E-09	ENSG00000065029	6	35259734	35295985
ENST00000374045	62.249118	-1.341525949	0.422149	-3.177849	0.001483722	0.011599074	ENSG00000181634	9	114784651	114806039
ENST00000374198	866.49976	-1.102754839	0.240579	-4.583758	4.57E-06	0.000100151	ENSG00000136875	9	113275657	113292905
ENST00000374212	518.48983	-1.095150417	0.211584	-5.175955	2.27E-07	7.51E-06	ENSG00000136868	9	113221543	113264492
ENST00000374227	23.015965	-7.998442436	1.568803	-5.098437	3.42E-07	1.07E-05	ENSG00000136866	9	113038376	113056724
ENST00000374242	545.61128	-1.307967463	0.205068	-6.378201	1.79E-10	1.44E-08	ENSG00000148153	9	112683925	112718117
ENST00000374257	7380.7696	-1.416548202	0.194536	-7.281682	3.30E-13	4.52E-11	ENSG00000119314	9	112218434	112333619
ENST00000374270	240.27047	-1.454955854	0.258782	-5.622331	1.88E-08	8.51E-07	ENSG00000106868	9	112040782	112175297
ENST00000374279	1610.0437	-1.29718151	0.175739	-7.381308	1.57E-13	2.32E-11	ENSG00000148154	9	111896813	111935369
ENST00000374293	1718.4029	-2.167439113	0.188416	-11.50351	1.27E-30	1.58E-27	ENSG00000242616	9	111661604	111670226
ENST00000374294	1714.9953	-2.084680831	0.182798	-11.40428	3.98E-30	4.81E-27	ENSG00000244115	9	111631385	111670229
ENST00000374466	122.96627	1.448909367	0.325444	4.452104	8.50E-06	0.000168926	ENSG00000169826	10	43138444	43185302
ENST00000374472	3.9197703	5.394631232	2.054307	2.626011	0.008639213	0.045513691	ENSG00000188822	1	23870514	23913362
ENST00000374530	1378.5331	-1.342105345	0.179417	-7.480384	7.41E-14	1.16E-11	ENSG00000157654	9	109780197	110172512
ENST00000374586	3067.4164	-1.328514284	0.17043	-7.795094	6.44E-15	1.24E-12	ENSG00000106771	9	109015134	109119947
ENST00000374630	429.14607	-1.674807751	0.243784	-6.87006	6.42E-12	6.89E-10	ENSG00000133216	1	22710838	22921500
ENST00000374692	711.49738	-1.566875513	0.197537	-7.932072	2.16E-15	4.46E-13	ENSG00000095209	9	105694540	105776629

RRAGC	protein_coding	Yes	NM_022157.4	108
CDCA8	protein_coding	Yes	NM_001256875.2	108
SGK2	protein_coding	Yes	NM_170693.3	108
SPOCK2	protein_coding	Yes	NM_001244950.2	108
SGPL1	protein_coding	Yes	NM_003901.4	108
LPIN3	protein_coding	Yes	NM_022896.3	108
FAM241B	protein_coding	Yes	NM_145306.3	108
NIBAN2	protein_coding	Yes	NM_022833.4	108
ARHGAP40	protein_coding	Yes	-	108
PIM1	protein_coding	Yes	NM_002648.4	108
HDAC8	protein_coding	Yes	NM_018486.3	108
OLFML2A	protein_coding	Yes	NM_182487.4	108
ADGRB2	protein_coding	Yes	NM_001364857.2	108
SERINC2	protein_coding	Yes	NM_178865.5	108
TMEM198	protein_coding	Yes	NM_001005209.3	108
TRAF1	protein_coding	Yes	NM_005658.5	108
ZWINT	protein_coding	Yes	NM_007057.4	108
ZNF76	protein_coding	Yes	NM_003427.5	108
TNFSF15	protein_coding	Yes	NM_005118.4	108
PRPF4	protein_coding	Yes	NM_001244926.2	108
SLC31A1	protein_coding	Yes	NM_001859.4	108
ZFP37	protein_coding	Yes	NM_003408.3	108
INIP	protein_coding	Yes	NM_021218.3	108
PTBP3	protein_coding	Yes	NM_001163788.4	108
SUSD1	protein_coding	Yes	NM_022486.5	108
UGCG	protein_coding	Yes	NM_003358.3	108
GNG10	protein_coding	Yes	NM_001017998.4	108
DNAJC25-GNG10	protein_coding	Yes	-	108
CSGALNACT2	protein_coding	Yes	NM_018590.5	108
CNR2	protein_coding	Yes	NM_001841.3	108
PALM2AKAP2	protein_coding	Yes	NM_007203.5	108
TMEM245	protein_coding	Yes	NM_032012.4	108
EPHB2	protein_coding	Yes	NM_017449.5	108
TMEM38B	protein_coding	Yes	NM_018112.3	108

ENST00000374736	81.137199	2.452360451	0.481655	5.091527	3.55E-07	1.11E-05	ENSG00000165029	9	104781005	104928155
ENST00000374839	8.5124246	6.514633966	1.752987	3.716305	0.000202158	0.002384955	ENSG00000126970	X	64915806	64976450
ENST00000374843	89.783172	2.769806204	0.445411	6.218543	5.02E-10	3.51E-08	ENSG00000204257	6	32948617	32953097
ENST00000374859	55.496986	3.313688982	0.518977	6.385041	1.71E-10	1.39E-08	ENSG00000240065	6	32854191	32859851
ENST00000374865	517.74896	-1.277308038	0.26585	-4.804623	1.55E-06	3.95E-05	ENSG00000136897	9	101387632	101398618
ENST00000374867	565.13745	2.541918852	0.221527	11.47453	1.77E-30	2.18E-27	ENSG00000099250	10	33177492	33334667
ENST00000374941	328.14133	1.184703539	0.243215	4.871021	1.11E-06	2.95E-05	ENSG00000083750	X	55717748	55758774
ENST00000375011	161.79387	-1.09840315	0.302322	-3.633217	0.00027991	0.003110784	ENSG00000119514	9	98807669	98850081
ENST00000375078	71.393804	-2.922252538	0.440635	-6.631908	3.31E-11	3.10E-09	ENSG00000162545	1	20482390	20486210
ENST00000375180	77.381869	1.265168227	0.385334	3.283299	0.001025998	0.008647538	ENSG00000184083	X	54068323	54183254
ENST00000375200	114.6111	2.396499298	0.358279	6.688918	2.25E-11	2.17E-09	ENSG00000131061	20	33731995	33792269
ENST00000375254	8292.3897	1.404048886	0.185302	7.577072	3.53E-14	5.93E-12	ENSG00000127481	1	19074509	19210266
ENST00000375256	578.37063	-1.093894389	0.247925	-4.412192	1.02E-05	0.000197971	ENSG00000165244	9	96385940	96418370
ENST00000375263	45.376124	2.368970666	0.518119	4.57225	4.83E-06	0.000104898	ENSG00000130948	9	96235305	96302176
ENST00000375344	1073.4373	-1.147160476	0.186434	-6.153187	7.59E-10	5.10E-08	ENSG00000148110	9	94374568	94461042
ENST00000375370	1233.2644	-1.011278998	0.196662	-5.142226	2.72E-07	8.81E-06	ENSG00000198176	13	113584752	113641473
ENST00000375431	120.65636	-1.735939991	0.328499	-5.284465	1.26E-07	4.54E-06	ENSG00000139835	13	113324162	113364130
ENST00000375442	141.59742	1.109429348	0.302675	3.665413	0.00024694	0.002807005	ENSG00000184205	X	53082366	53088540
ENST00000375446	253.98105	-1.378226295	0.256243	-5.378596	7.51E-08	2.92E-06	ENSG00000131669	9	93121495	93134251
ENST00000375472	37.501323	-1.749472162	0.5395	-3.242769	0.001183742	0.009711236	ENSG00000157303	9	93058700	93085133
ENST00000375527	64.754226	2.149838405	0.432521	4.970486	6.68E-07	1.91E-05	ENSG00000204366	6	31899612	31902086
ENST00000375533	120.55976	-2.047998564	0.46278	-4.425429	9.63E-06	0.000187736	ENSG00000095739	10	28677520	28682932
ENST00000375631	1603.126	2.17563859	0.183069	11.88425	1.43E-32	2.05E-29	ENSG00000204386	6	31857658	31862821
ENST00000375654	37.418856	1.434122923	0.530292	2.704401	0.00684277	0.038263627	ENSG00000204390	6	31809618	31815283
ENST00000375688	55.442599	2.146978802	0.503202	4.266635	1.98E-05	0.000346858	ENSG00000204396	6	31765589	31777328
ENST00000375731	266.02237	-1.142146907	0.2449	-4.663737	3.11E-06	7.17E-05	ENSG00000148090	9	91213822	91361918
ENST00000375749	1328.1075	-1.185608838	0.188072	-6.304032	2.90E-10	2.20E-08	ENSG00000101346	20	32207879	32238658
ENST00000375754	379.9315	-4.127908719	0.270908	-15.23732	2.00E-52	1.52E-48	ENSG00000165025	9	90801818	90898549
ENST00000375766	89.36605	-2.242523471	0.396846	-5.650866	1.60E-08	7.36E-07	ENSG00000162458	1	15758794	15786589
ENST00000375864	60.352093	1.25919018	0.433339	2.905788	0.003663298	0.023545182	ENSG00000240053	6	31669975	31673546
ENST00000375898	292.27845	-1.136708878	0.260357	-4.365961	1.27E-05	0.00023737	ENSG00000139826	13	108218391	108234243
ENST00000375915	3.8199375	-5.406272071	2.074727	-2.605775	0.009166675	0.047583599	ENSG00000204442	13	107163509	107867496
ENST00000375980	217.77416	-1.420713936	0.32848	-4.325115	1.52E-05	0.000278335	ENSG00000142634	1	15409887	15430339
ENST00000376008	330.33145	-1.176417625	0.231098	-5.090559	3.57E-07	1.11E-05	ENSG00000171729	1	15153746	15220478

ABCA1	protein_coding	Yes	NM_005502.4	108
ZC4H2	protein_coding	Yes	NM_018684.4	108
HLA-DMA	protein_coding	Yes	NM_006120.4	108
PSMB9	protein_coding	Yes	NM_002800.5	108
MRPL50	protein_coding	Yes	NM_019051.3	108
NRP1	protein_coding	Yes	NM_003873.7	108
RRAGB	protein_coding	Yes	NM_006064.5	108
GALNT12	protein_coding	Yes	NM_024642.5	108
CAMK2N1	protein_coding	Yes	NM_018584.6	108
FAM120C	protein_coding	Yes	NM_017848.6	108
ZNF341	protein_coding	Yes	NM_001282933.2	108
UBR4	protein_coding	Yes	NM_020765.3	108
ZNF367	protein_coding	Yes	NM_153695.4	108
HSD17B3	protein_coding	Yes	NM_000197.2	108
MFSD14B	protein_coding	Yes	NM_032558.3	108
TFDP1	protein_coding	Yes	NM_007111.5	108
GRTP1	protein_coding	Yes	NM_024719.4	108
TSPYL2	protein_coding	Yes	NM_022117.4	108
NINJ1	protein_coding	Yes	NM_004148.4	108
SUSD3	protein_coding	Yes	NM_145006.4	108
ZBTB12	protein_coding	Yes	NM_181842.3	108
BAMBI	protein_coding	Yes	NM_012342.3	108
NEU1	protein_coding	Yes	NM_000434.4	108
HSPA1L	protein_coding	Yes	NM_005527.4	108
VWA7	protein_coding	Yes	NM_025258.3	108
AUH	protein_coding	Yes	NM_001698.3	108
POFUT1	protein_coding	Yes	NM_015352.2	108
SYK	protein_coding	Yes	NM_003177.7	108
FBLIM1	protein_coding	Yes	NM_017556.4	108
LY6G5B	protein_coding	Yes	NM_021221.3	108
ABHD13	protein_coding	Yes	NM_032859.3	108
NALF1	protein_coding	Yes	NM_001080396.3	108
EFHD2	protein_coding	Yes	NM_024329.6	108
TMEM51	protein_coding	Yes	NM_001136218.2	108

ENST00000376032	162.85327	-1.114008964	0.290365	-3.836588	0.000124756	0.001604298	ENSG00000151287	13	102765887	102773786
ENST00000376104	35.253483	1.629548159	0.548501	2.970912	0.002969168	0.019992663	ENSG00000150672	11	83455172	85627344
ENST00000376112	314.54525	-3.071675936	0.808463	-3.7994	0.000145047	0.001819185	ENSG00000125968	20	31605288	31606510
ENST00000376358	293.63238	1.14238364	0.243219	4.696936	2.64E-06	6.24E-05	ENSG00000288053	X	49071469	49079887
ENST00000376372	531.28717	1.192189948	0.2105	5.663604	1.48E-08	6.92E-07	ENSG00000196998	X	49074441	49079887
ENST00000376447	699.02444	-1.403517705	0.195822	-7.1673	7.65E-13	9.80E-11	ENSG00000165105	9	82979589	83063142
ENST00000376568	3824.8856	1.006799092	0.189495	5.313055	1.08E-07	3.97E-06	ENSG00000204580	6	30884518	30900156
ENST00000376588	373.26216	-6.758810105	0.44654	-15.13595	9.38E-52	5.96E-48	ENSG00000135069	9	78297124	78330093
ENST00000376730	9.6223353	-4.22250441	1.364947	-3.09353	0.001977905	0.014548632	ENSG00000187210	9	76459181	76507416
ENST00000376854	155.83044	-1.947761567	0.359359	-5.420105	5.96E-08	2.40E-06	ENSG00000135045	9	74946582	74952912
ENST00000377104	48.259732	-1.276075022	0.471758	-2.704938	0.006831724	0.038213075	ENSG00000136158	13	80335975	80341126
ENST00000377245	2533.8209	-1.144295933	0.178141	-6.423553	1.33E-10	1.11E-08	ENSG00000119139	9	69174276	69255208
ENST00000377369	15.654252	7.392614731	1.620783	4.561137	5.09E-06	0.000109772	ENSG00000148482	10	17951917	18043285
ENST00000377401	1598.5135	-1.753200581	0.18232	-9.61605	6.84E-22	3.27E-19	ENSG00000185130	6	27807478	27807929
ENST00000377459	1753.9137	-1.426138855	0.17906	-7.964583	1.66E-15	3.48E-13	ENSG00000274997	6	27147105	27147562
ENST00000377482	6179.0307	-1.234263341	0.189092	-6.527321	6.70E-11	5.84E-09	ENSG00000116285	1	8011726	8026309
ENST00000377495	45.17026	1.79641952	0.499237	3.59833	0.000320267	0.0034621	ENSG00000148483	10	17752200	17800868
ENST00000377602	4.8748837	5.710071607	1.949769	2.928589	0.003405044	0.022197012	ENSG00000148488	10	17315420	17454595
ENST00000377658	1382.8023	2.060221962	0.235286	8.756229	2.02E-18	6.28E-16	ENSG00000162413	1	6590723	6602869
ENST00000377693	14.003501	-3.408713787	1.023122	-3.33168	0.000863235	0.007579241	ENSG00000204866	19	46148237	46161289
ENST00000377708	699.65647	1.155395776	0.221203	5.223246	1.76E-07	6.05E-06	ENSG00000186470	6	26365197	26378312
ENST00000377727	1210.324	-1.675010067	0.211326	-7.926176	2.26E-15	4.65E-13	ENSG00000158406	6	26285125	26285534
ENST00000377733	1237.4862	-1.708442279	0.181208	-9.428088	4.18E-21	1.72E-18	ENSG00000278588	6	26272930	26273412
ENST00000377777	2582.6518	-1.077962425	0.172826	-6.237257	4.45E-10	3.16E-08	ENSG00000158373	6	26158121	26158607
ENST00000377803	3832.7025	-1.078319138	0.17078	-6.314099	2.72E-10	2.09E-08	ENSG00000197061	6	26103932	26104337
ENST00000377818	783.47113	-1.715368417	0.225524	-7.606131	2.82E-14	4.84E-12	ENSG00000204899	13	72708366	72727629
ENST00000377834	129.84957	-1.531227733	0.341241	-4.487232	7.22E-06	0.000147613	ENSG00000069812	1	6415231	6419919
ENST00000377838	643.7299	1.155240053	0.202384	5.708151	1.14E-08	5.58E-07	ENSG00000066827	8	134477787	134713031
ENST00000377893	80.435234	-1.367367039	0.377294	-3.624144	0.00028992	0.00320404	ENSG00000158292	1	6247352	6261098
ENST00000377939	360.75438	1.772902831	0.301677	5.876824	4.18E-09	2.30E-07	ENSG00000158286	1	6206118	6221299
ENST00000377953	119.80909	-2.147463362	0.405891	-5.290741	1.22E-07	4.41E-06	ENSG00000204922	11	62671672	62673686
ENST00000377990	305.21617	1.045743495	0.254617	4.107116	4.01E-05	0.000626067	ENSG00000011523	2	65056415	65087004
ENST00000378026	156.53857	-1.956633628	0.301682	-6.485757	8.83E-11	7.59E-09	ENSG00000136026	12	106237880	106248020
ENST00000378043	11.960626	2.761429053	1.047175	2.637028	0.00836359	0.044531113	ENSG00000167995	11	61950350	61964461

TEX30	protein_coding	Yes	NM_138779.5	108
DLG2	protein_coding	Yes	NM_001142699.3	108
ID1	protein_coding	Yes	NM_002165.4	108
-	protein_coding	Yes	-	108
WDR45	protein_coding	Yes	NM_001029896.2	108
RASEF	protein_coding	Yes	NM_152573.4	108
DDR1	protein_coding	Yes	NM_001297654.2	108
PSAT1	protein_coding	Yes	NM_058179.4	108
GCNT1	protein_coding	Yes	NM_001490.5	108
C9orf40	protein_coding	Yes	NM_017998.3	108
SPRY2	protein_coding	Yes	NM_005842.4	108
TJP2	protein_coding	Yes	NM_004817.4	108
SLC39A12	protein_coding	Yes	NM_001145195.2	108
H2BC13	protein_coding	Yes	NM_003519.4	108
H2AC12	protein_coding	Yes	NM_080596.3	108
ERRFI1	protein_coding	Yes	NM_018948.4	108
TMEM236	protein_coding	Yes	NM_001098844.3	108
ST8SIA6	protein_coding	Yes	NM_001004470.3	108
KLHL21	protein_coding	Yes	NM_014851.4	108
IGFL2	protein_coding	Yes	NM_001135113.2	108
BTN3A2	protein_coding	Yes	NM_007047.5	108
H4C8	protein_coding	Yes	NM_003543.4	108
H2BC10	protein_coding	Yes	NM_003525.3	108
H2BC5	protein_coding	Yes	NM_021063.4	108
H4C3	protein_coding	Yes	NM_003542.4	108
MZT1	protein_coding	Yes	NM_001071775.3	108
HES2	protein_coding	Yes	NM_019089.5	108
ZFAT	protein_coding	Yes	NM_020863.4	108
GPR153	protein_coding	Yes	NM_207370.4	108
RNF207	protein_coding	Yes	NM_207396.3	108
UQCC3	protein_coding	Yes	NM_001085372.3	108
CEP68	protein_coding	Yes	NM_015147.3	108
CKAP4	protein_coding	Yes	NM_006825.4	108
BEST1	protein_coding	Yes	NM_004183.4	108

ENST00000378198	558.60955	1.142587483	0.20648	5.533634	3.14E-08	1.34E-06	ENSG00000111802	6	24649978	24666899
ENST00000378214	87.92723	2.270463891	0.407406	5.572973	2.50E-08	1.10E-06	ENSG00000137261	6	24544106	24646191
ENST00000378247	71.52691	1.568514178	0.409882	3.826742	0.00012985	0.001658755	ENSG00000204991	16	89828474	89871319
ENST00000378387	347.35204	-1.619630827	0.24262	-6.675599	2.46E-11	2.35E-09	ENSG00000137135	9	35659342	35665195
ENST00000378486	17.970814	3.711335065	0.945512	3.925213	8.67E-05	0.001183835	ENSG00000149527	1	2476288	2505532
ENST00000378509	137.78098	-1.344445054	0.307724	-4.368991	1.25E-05	0.000234561	ENSG00000205060	7	134289331	134316930
ENST00000378555	95.684897	-3.702965049	0.443842	-8.342988	7.24E-17	1.81E-14	ENSG00000128567	7	131500270	131556628
ENST00000378585	8.3383499	-6.533808944	1.758928	-3.714655	0.000203481	0.002397403	ENSG00000187730	1	2019344	2030758
ENST00000378616	70.743132	-2.853648641	0.497355	-5.737654	9.60E-09	4.80E-07	ENSG00000047597	X	37685790	37732130
ENST00000378623	24.893706	-3.370152285	0.755671	-4.459817	8.20E-06	0.000163963	ENSG00000134569	11	46856716	46918550
ENST00000378693	15.608407	-4.967749489	1.264993	-3.927098	8.60E-05	0.001176158	ENSG00000198944	5	132813301	132816786
ENST00000378743	592.14791	1.00210437	0.210885	4.751902	2.02E-06	4.97E-05	ENSG00000133858	12	71609598	71663848
ENST00000378747	1249.2545	1.001180197	0.233706	4.283928	1.84E-05	0.000325722	ENSG00000123240	10	13100162	13138308
ENST00000378750	351.46382	1.01928456	0.230379	4.424379	9.67E-06	0.000188531	ENSG00000121680	11	45909662	45917877
ENST00000379028	12.975014	2.441759501	0.934457	2.613024	0.008974505	0.046794836	ENSG00000091129	7	108147648	108456436
ENST00000379116	22.978222	2.441557257	0.732698	3.332283	0.000861367	0.007565799	ENSG00000162572	1	1280435	1292025
ENST00000379214	1771.1722	-1.26386424	0.176222	-7.171991	7.39E-13	9.51E-11	ENSG00000205213	11	27365960	27472790
ENST00000379359	27.948391	2.563906587	0.654301	3.918542	8.91E-05	0.001211106	ENSG00000102760	13	41457549	41470871
ENST00000379400	889.46685	-1.48083801	0.206773	-7.161652	7.97E-13	1.01E-10	ENSG00000101265	20	4780022	4823608
ENST00000379410	14.170299	-2.628327904	0.912507	-2.880337	0.003972505	0.025111089	ENSG00000187583	1	966481	975865
ENST00000379426	105.45378	-1.541614363	0.351004	-4.392008	1.12E-05	0.000214419	ENSG00000205269	6	11537748	11583524
ENST00000379446	1001.4039	1.049813751	0.188758	5.561703	2.67E-08	1.17E-06	ENSG00000111859	6	11183297	11232668
ENST00000379589	9.4334314	-6.71034869	1.734379	-3.869022	0.000109273	0.001434066	ENSG00000183722	13	39342891	39603193
ENST00000379672	49.751657	-3.272037455	0.541339	-6.044345	1.50E-09	9.28E-08	ENSG00000006740	17	12789497	12991643
ENST00000379727	1727.1178	2.076447519	0.766948	2.707415	0.006780935	0.038016889	ENSG00000140465	15	74719541	74725528
ENST00000379936	4.0778538	5.455089577	2.050019	2.660995	0.007791012	0.042153999	ENSG00000132259	11	6239088	6244479
ENST00000380050	103.67744	-1.145036728	0.33831	-3.384579	0.000712874	0.006515565	ENSG00000058091	7	90596320	91210590
ENST00000380071	618.55188	-1.661410426	0.213085	-7.796924	6.34E-15	1.23E-12	ENSG00000133119	13	33818148	33837500
ENST00000380079	64.645279	-1.48404538	0.493418	-3.007682	0.002632486	0.018235604	ENSG00000127954	7	88270891	88306894
ENST00000380097	112.17078	-2.987219869	0.382579	-7.80811	5.81E-15	1.13E-12	ENSG00000121236	11	5596636	5612952
ENST00000380130	23.438135	2.042729541	0.688172	2.968343	0.002994098	0.020124962	ENSG00000139597	13	32400722	32428130
ENST00000380243	377.39407	-1.113256715	0.224799	-4.952224	7.34E-07	2.08E-05	ENSG00000205476	14	99500189	99604207
ENST00000380412	489.96281	-1.390636079	0.226325	-6.144417	8.03E-10	5.35E-08	ENSG00000169957	16	30524003	30526566
ENST00000380526	52.653592	1.992610621	0.484421	4.113389	3.90E-05	0.000611925	ENSG00000228175	1	89993592	89994321

TDP2	protein_coding	Yes	NM_016614.3	108
KIAA0319	protein_coding	Yes	NM_014809.4	108
SPIRE2	protein_coding	Yes	NM_032451.2	108
ARHGEF39	protein_coding	Yes	NM_032818.3	108
PLCH2	protein_coding	Yes	NM_014638.4	108
SLC35B4	protein_coding	Yes	NM_032826.5	108
PODXL	protein_coding	Yes	NM_001018111.3	108
GABRD	protein_coding	Yes	NM_000815.5	108
XK	protein_coding	Yes	NM_021083.4	108
LRP4	protein_coding	Yes	NM_002334.4	108
SOWAHA	protein_coding	Yes	NM_175873.6	108
ZFC3H1	protein_coding	Yes	NM_144982.5	108
OPTN	protein_coding	Yes	NM_001008212.2	108
PEX16	protein_coding	Yes	NM_004813.4	108
NRCAM	protein_coding	Yes	NM_001037132.4	108
SCNN1D	protein_coding	Yes	NM_001130413.4	108
LGR4	protein_coding	Yes	NM_018490.5	108
RGCC	protein_coding	Yes	NM_014059.3	108
RASSF2	protein_coding	Yes	NM_014737.3	108
PLEKHN1	protein_coding	Yes	NM_032129.3	108
TMEM170B	protein_coding	Yes	NM_001100829.3	108
NEDD9	protein_coding	Yes	NM_006403.4	108
LHFPL6	protein_coding	Yes	NM_005780.3	108
ARHGAP44	protein_coding	Yes	NM_014859.6	108
CYP1A1	protein_coding	Yes	NM_001319217.2	108
CNGA4	protein_coding	Yes	NM_001037329.4	108
CDK14	protein_coding	Yes	NM_001287135.2	108
RFC3	protein_coding	Yes	NM_002915.4	108
STEAP4	protein_coding	Yes	NM_024636.4	108
TRIM6	protein_coding	Yes	NM_001003818.3	108
N4BP2L1	protein_coding	Yes	NM_052818.3	108
CCDC85C	protein_coding	Yes	NM_001144995.2	108
ZNF768	protein_coding	Yes	NM_024671.4	108
GEMIN8P4	essed_pseudog	Yes	-	108

ENST00000380554	117.10742	-1.143566266	0.401277	-2.849814	0.004374478	0.026998119	ENSG00000196139	10	5094413	5107686
ENST00000380668	1243.0773	-1.346133577	0.179893	-7.482958	7.27E-14	1.14E-11	ENSG00000101911	X	12791411	12824222
ENST00000380698	66.326836	-2.523330408	0.457181	-5.519327	3.40E-08	1.44E-06	ENSG00000170542	6	2887269	2903309
ENST00000380702	25.807113	-2.272879928	0.671994	-3.382293	0.000718833	0.00655958	ENSG00000065989	19	10420537	10469630
ENST00000380861	136.5275	-2.908728749	0.445026	-6.536089	6.31E-11	5.54E-09	ENSG00000047644	X	10015253	10144474
ENST00000380950	484.40629	-1.768443445	0.213799	-8.271527	1.32E-16	3.23E-14	ENSG00000103995	15	48737937	48811069
ENST00000380956	11.759311	6.979602753	1.673875	4.169727	3.05E-05	0.0004969	ENSG00000137265	6	391751	411443
ENST00000381055	57.49249	1.220809365	0.439344	2.778711	0.005457506	0.032049627	ENSG00000049192	5	65148737	65481920
ENST00000381160	10.630393	6.836238135	1.702285	4.015918	5.92E-05	0.000864307	ENSG00000015520	7	44512534	44541330
ENST00000381273	40.45409	2.494502936	0.554188	4.501183	6.76E-06	0.000139709	ENSG00000205693	12	27762426	27780236
ENST00000381317	90.674418	-1.106064058	0.388877	-2.844249	0.004451627	0.027364036	ENSG00000169093	X	1403138	1452909
ENST00000381323	118.36161	-1.626164985	0.435521	-3.733838	0.000188584	0.002255559	ENSG00000197872	2	16549458	16665834
ENST00000381486	39.754384	3.352393909	0.605729	5.534481	3.12E-08	1.34E-06	ENSG00000196208	2	11534044	11642788
ENST00000381668	29.462553	1.995335633	0.705962	2.826408	0.004707326	0.028607529	ENSG00000124406	4	42408372	42657105
ENST00000381801	931.11785	-1.386009018	0.195319	-7.096139	1.28E-12	1.58E-10	ENSG00000139505	13	25246221	25287488
ENST00000381867	60.037174	-1.543791351	0.461919	-3.342123	0.000831401	0.00735004	ENSG00000101298	20	1266293	1309327
ENST00000381962	2686.2072	1.152137232	0.211308	5.4524	4.97E-08	2.04E-06	ENSG00000271303	20	646614	653200
ENST00000381967	893.08023	-1.256921346	0.190695	-6.591264	4.36E-11	3.97E-09	ENSG00000169299	4	37826685	37862937
ENST00000381980	57.163379	-9.310879111	1.500068	-6.206972	5.40E-10	3.75E-08	ENSG00000154274	4	37453924	37593510
ENST00000382285	56.883073	-1.430192873	0.438097	-3.264561	0.001096337	0.009128319	ENSG00000125841	20	347110	354862
ENST00000382361	1576.3061	-1.587774068	0.266309	-5.962148	2.49E-09	1.45E-07	ENSG00000075618	7	5592815	5606655
ENST00000382554	191.7307	-2.557105737	0.315088	-8.115534	4.84E-16	1.09E-13	ENSG00000205978	14	24399002	24419283
ENST00000382774	5.3151966	5.836269963	1.918801	3.041623	0.002353063	0.016683551	ENSG00000109758	4	3441967	3449486
ENST00000382848	114.33881	-2.628070654	0.356058	-7.381015	1.57E-13	2.32E-11	ENSG00000165474	13	20187469	20192938
ENST00000383366	125.75708	1.178318618	0.318266	3.702313	0.000213643	0.002495523	ENSG00000114670	3	131026876	131350465
ENST00000383472	59.095548	-2.423679417	0.45754	-5.297199	1.18E-07	4.27E-06	ENSG00000088756	18	6729715	6915716
ENST00000383678	219.53659	-1.171221694	0.275875	-4.245483	2.18E-05	0.000374584	ENSG00000163607	3	112990983	113001969
ENST00000383759	91.006603	1.374054144	0.365977	3.754484	0.000173699	0.002108274	ENSG00000136059	3	37990785	38007185
ENST00000383869	128.147	-1.192296001	0.317573	-3.754399	0.000173758	0.002108822	ENSG00000206596	14	34546713	34546877
ENST00000383898	3.9445995	5.40270887	2.054252	2.630013	0.008538167	0.045174985	ENSG00000206625	15	67839938	67840045
ENST00000384010	46.669744	-1.274876508	0.483874	-2.63473	0.008420416	0.044761746	ENSG00000206737	1	143729406	143729570
ENST00000384101	26.183388	-3.638486523	0.765002	-4.756181	1.97E-06	4.88E-05	ENSG00000206828	1	149636765	149636929
ENST00000384111	47.433205	1.413973952	0.478789	2.953232	0.00314466	0.020919243	ENSG00000206838	7	45104348	45104482
ENST00000384476	29.775009	-2.339683884	0.62353	-3.75232	0.000175205	0.00212301	ENSG00000207205	1	144412575	144412740

AKR1C3	protein_coding	Yes	NM_003739.6	108
PRPS2	protein_coding	Yes	NM_002765.5	108
SERPINB9	protein_coding	Yes	NM_004155.6	108
PDE4A	protein_coding	Yes	NM_001111307.2	108
WWC3	protein_coding	Yes	-	108
CEP152	protein_coding	Yes	NM_001194998.2	108
IRF4	protein_coding	Yes	NM_002460.4	108
ADAMTS6	protein_coding	Yes	NM_197941.4	108
NPC1L1	protein_coding	Yes	NM_001101648.2	108
MANSC4	protein_coding	Yes	NM_001146221.5	108
ASMTL	protein_coding	Yes	NM_004192.4	108
CYRIA	protein_coding	Yes	NM_030797.4	108
GREB1	protein_coding	Yes	NM_014668.4	108
ATP8A1	protein_coding	Yes	NM_006095.2	108
MTMR6	protein_coding	Yes	NM_004685.5	108
SNPH	protein_coding	Yes	NM_001318234.2	108
SRXN1	protein_coding	Yes	NM_080725.3	108
PGM2	protein_coding	Yes	NM_018290.4	108
C4orf19	protein_coding	Yes	NM_001104629.2	108
NRSN2	protein_coding	Yes	NM_001323682.2	108
FSCN1	protein_coding	Yes	NM_003088.4	108
NYNRIN	protein_coding	Yes	NM_025081.3	108
HGFAC	protein_coding	Yes	NM_001528.4	108
GJB2	protein_coding	Yes	NM_004004.6	108
NEK11	protein_coding	Yes	NM_024800.5	108
ARHGAP28	protein_coding	Yes	NM_001366230.1	108
GTPBP8	protein_coding	Yes	NM_014170.4	108
VILL	protein_coding	Yes	NM_015873.4	108
RNU1-27P	snRNA	Yes	-	108
RNU6-1	snRNA	Yes	-	108
RNVU1-18	snRNA	Yes	-	108
RNVU1-30	snRNA	Yes	-	108
SNORA5A	snoRNA	Yes	-	108
RNVU1-15	snRNA	Yes	-	108

ENST00000384770	40.720062	-2.628576194	0.562269	-4.67494	2.94E-06	6.84E-05	ENSG00000207501	1	145281115	145281279
ENST00000384994	48.62798	-1.841378136	0.587132	-3.136226	0.00171137	0.012986389	ENSG00000283791	11	65444457	65444557
ENST00000385104	4.7168003	5.659552855	1.975514	2.864851	0.004172059	0.02606122	ENSG00000207839	17	17813835	17813931
ENST00000388747	75.309584	-2.100385457	0.421165	-4.98709	6.13E-07	1.78E-05	ENSG00000167103	9	127920880	127930777
ENST00000388934	99.814936	-1.826101512	0.354186	-5.155772	2.53E-07	8.26E-06	ENSG00000172478	2	240886047	240896156
ENST00000389063	2016.6562	-1.011097968	0.180562	-5.599717	2.15E-08	9.58E-07	ENSG00000172795	5	112976797	113022195
ENST00000389082	133.02413	1.093104663	0.309868	3.527643	0.000419276	0.004292205	ENSG00000170456	12	31382225	31591136
ENST00000389176	17.472384	3.371345975	0.95879	3.516252	0.000437686	0.004443357	ENSG00000197599	16	1434382	1444556
ENST00000389313	43.311203	-1.553771768	0.501457	-3.098514	0.001944936	0.014360064	ENSG00000188176	17	4584528	4608319
ENST00000389658	219.2543	1.870458258	0.295639	6.326825	2.50E-10	1.93E-08	ENSG00000101680	18	6941741	7117797
ENST00000389758	16.792278	7.494674249	1.610655	4.653183	3.27E-06	7.50E-05	ENSG00000185038	2	233778329	233833418
ENST00000389793	515.54494	1.140025372	0.258648	4.407635	1.05E-05	0.000201643	ENSG00000143669	1	235661040	235866906
ENST00000389805	8580.2108	1.276457848	0.233956	5.455983	4.87E-08	2.00E-06	ENSG00000161011	5	179820904	179838078
ENST00000389840	30.521966	4.868645167	0.902952	5.391921	6.97E-08	2.74E-06	ENSG00000187775	17	78423696	78577396
ENST00000389857	105.21908	-1.143065582	0.336783	-3.394074	0.00068861	0.006335752	ENSG00000015133	14	91271322	91417820
ENST00000389912	375.03552	-1.359719899	0.232434	-5.849922	4.92E-09	2.66E-07	ENSG00000133983	14	70325080	70359683
ENST00000390168	1948.0387	-2.824131758	0.178839	-15.79144	3.56E-56	3.19E-52	ENSG00000284010	11	1996758	1996831
ENST00000390667	496.9921	-1.580446067	0.216319	-7.306106	2.75E-13	3.86E-11	ENSG00000136122	13	72727922	72756196
ENST00000390893	34.383303	-2.1389567	0.574796	-3.721244	0.000198244	0.002349182	ENSG00000212195	17	58631640	58631836
ENST00000391759	74.426654	1.404724563	0.407091	3.450639	0.000559261	0.005385241	ENSG00000105619	19	54107019	54115657
ENST00000391854	154.0383	-1.171062367	0.305151	-3.837644	0.000124221	0.001598851	ENSG00000168243	1	235547684	235649784
ENST00000391967	623.23027	-1.68258778	0.237129	-7.095664	1.29E-12	1.58E-10	ENSG00000159166	1	201380832	201399324
ENST00000392027	988.73255	2.791671839	0.271905	10.2671	9.91E-25	7.26E-22	ENSG00000163283	2	232378750	232382889
ENST00000392219	377.28559	-1.261552869	0.226893	-5.560129	2.70E-08	1.18E-06	ENSG00000089327	19	35154734	35169881
ENST00000392318	45.809988	-1.634647211	0.522182	-3.130418	0.001745579	0.013182965	ENSG00000128641	2	191245403	191425386
ENST00000392413	7.9727949	6.421219065	1.777032	3.613452	0.000302147	0.003308115	ENSG00000130518	19	18257097	18274452
ENST00000392452	773.77335	-1.409813726	0.196964	-7.157727	8.20E-13	1.04E-10	ENSG00000180611	3	192796814	192917856
ENST00000392487	4.4097416	5.564557071	1.995673	2.788312	0.005298356	0.031321376	ENSG00000163352	1	154993585	154994315
ENST00000392647	310.91289	-1.195469748	0.236493	-5.05498	4.30E-07	1.31E-05	ENSG00000213160	2	169733831	169751878
ENST00000392668	284.27268	1.111849656	0.303498	3.66345	0.000248841	0.002825246	ENSG00000134954	11	128458764	128587558
ENST00000392692	3160.8827	-1.130703884	0.166566	-6.788305	1.13E-11	1.16E-09	ENSG00000114346	3	172750725	172821474
ENST00000392929	20.11089	4.230582951	0.969069	4.365615	1.27E-05	0.000237644	ENSG00000224043	2	134735463	134918670
ENST00000392951	49.425624	2.308004749	0.510397	4.521979	6.13E-06	0.00012856	ENSG00000149591	11	117199369	117207464
ENST00000392986	3.9445995	5.40270887	2.054252	2.630013	0.008538167	0.045174985	ENSG00000012504	12	100473865	100564414

RNVU1-14	snRNA	Yes	-	108
MIR612	miRNA	Yes	-	108
MIR33B	miRNA	Yes	-	108
PIP5KL1	protein_coding	Yes	NM_001135219.2	108
MAB21L4	protein_coding	Yes	NM_001085437.3	108
DCP2	protein_coding	Yes	NM_152624.6	108
DENND5B	protein_coding	Yes	NM_144973.4	108
CCDC154	protein_coding	Yes	NM_001143980.3	108
SMTNL2	protein_coding	Yes	NM_001114974.2	108
LAMA1	protein_coding	Yes	NM_005559.4	108
MROH2A	protein_coding	Yes	NM_001394639.1	108
LYST	protein_coding	Yes	NM_000081.4	108
SQSTM1	protein_coding	Yes	NM_003900.5	108
DNAH17	protein_coding	Yes	NM_173628.4	108
CCDC88C	protein_coding	Yes	NM_001080414.4	108
COX16	protein_coding	Yes	NM_016468.7	108
MIR675	miRNA	Yes	-	108
BORA	protein_coding	Yes	NM_024808.5	108
U3	snoRNA	Yes	-	108
TFPT	protein_coding	Yes	NM_013342.4	108
GNG4	protein_coding	Yes	NM_001098722.2	108
LAD1	protein_coding	Yes	NM_005558.4	108
ALPP	protein_coding	Yes	NM_001632.5	108
FXYD5	protein_coding	Yes	NM_014164.6	108
MYO1B	protein_coding	Yes	NM_001130158.3	108
IQCN	protein_coding	Yes	NM_001145304.2	108
MB21D2	protein_coding	Yes	NM_178496.4	108
LENEP	protein_coding	Yes	NM_001394530.1	108
KLHL23	protein_coding	Yes	NM_144711.6	108
ETS1	protein_coding	Yes	NM_001143820.2	108
ECT2	protein_coding	Yes	NM_001258315.2	108
CCNT2-AS1	lncRNA	Yes	-	108
TAGLN	protein_coding	Yes	NM_003186.5	108
NR1H4	protein_coding	Yes	NM_001206979.2	108

ENST00000393196	1237.8129	-1.540198536	0.278196	-5.536382	3.09E-08	1.33E-06	ENSG00000239672	17	51153558	51162168
ENST00000393200	134.94068	-5.617869987	0.535378	-10.49328	9.28E-26	7.72E-23	ENSG00000136695	2	113059197	113064744
ENST00000393217	6.5445397	-4.697211964	1.806489	-2.600189	0.009317247	0.048183055	ENSG00000133640	12	85036350	85245105
ENST00000393306	77.548124	1.540668358	0.4314	3.571322	0.000355184	0.003752783	ENSG00000140961	16	83953239	83966332
ENST00000393366	314.93532	-1.193859021	0.288385	-4.139807	3.48E-05	0.000555264	ENSG00000159199	17	48892786	48895871
ENST00000393483	90.13255	1.25307449	0.364415	3.438595	0.000584741	0.0055694	ENSG00000135951	2	98997260	99154942
ENST00000393565	295.54631	-3.542561398	0.317636	-11.15291	6.93E-29	7.71E-26	ENSG00000113758	5	177456609	177473634
ENST00000393567	21.982123	1.829323506	0.69605	2.628151	0.008585031	0.045363919	ENSG00000157423	16	70802083	71230722
ENST00000393597	117.2344	-3.479270793	0.409822	-8.489705	2.07E-17	5.51E-15	ENSG00000175591	11	73218280	73242427
ENST00000393674	284.99046	-1.026567581	0.241229	-4.255578	2.09E-05	0.00036088	ENSG00000164117	4	174236657	174283667
ENST00000393784	814.35519	-1.059113456	0.216657	-4.888434	1.02E-06	2.73E-05	ENSG00000113719	5	172834250	172952683
ENST00000393791	7.0673399	6.245901292	1.808364	3.453896	0.00055255	0.005331391	ENSG00000123329	12	57472268	57479866
ENST00000393868	682.62303	-1.170662559	0.197555	-5.925753	3.11E-09	1.76E-07	ENSG00000163541	2	84423527	84459280
ENST00000393909	946.37844	-1.147874544	0.18561	-6.18435	6.24E-10	4.27E-08	ENSG00000115364	2	75646782	75662206
ENST00000393965	297.43774	1.247223743	0.239001	5.218494	1.80E-07	6.18E-06	ENSG00000135637	2	74472831	74482998
ENST00000394019	40.311393	1.91897422	0.572939	3.349354	0.000810002	0.007201329	ENSG00000188687	2	74216241	74343416
ENST00000394133	4.1523414	5.47828107	2.024699	2.705726	0.00681552	0.038152509	ENSG00000213432	10	93284003	93284558
ENST00000394166	3879.0321	-1.149939262	0.241809	-4.755565	1.98E-06	4.90E-05	ENSG00000185551	15	96330699	96340258
ENST00000394231	241.81866	1.114945793	0.252056	4.423413	9.72E-06	0.000189303	ENSG00000141741	17	39728509	39730532
ENST00000394249	3005.9943	-1.00482747	0.195906	-5.129143	2.91E-07	9.33E-06	ENSG00000198901	15	90966039	90994535
ENST00000394264	139.11765	-1.058085503	0.314447	-3.36491	0.000765687	0.006896298	ENSG00000187866	9	68780064	68785566
ENST00000394313	51.129239	-1.418888924	0.487109	-2.91288	0.003581126	0.023131641	ENSG00000161642	12	54369135	54384733
ENST00000394334	679.00677	-1.188123626	0.198016	-6.000138	1.97E-09	1.18E-07	ENSG00000134602	X	132023301	132075943
ENST00000394412	77.136848	-1.326718539	0.401388	-3.305331	0.000948644	0.008152155	ENSG00000166813	15	89627976	89655467
ENST00000394485	36.155582	-4.148262358	0.870008	-4.768075	1.86E-06	4.63E-05	ENSG00000187193	16	56682469	56684196
ENST00000394576	55.375719	1.209429591	0.440571	2.745143	0.006048445	0.034712464	ENSG00000081853	5	141338759	141512975
ENST00000394610	228.56585	1.028664238	0.307697	3.34311	0.00082845	0.007330749	ENSG00000125354	X	119616944	119693168
ENST00000394650	367.17181	-1.116564996	0.223661	-4.992209	5.97E-07	1.74E-05	ENSG00000005059	4	109560245	109688719
ENST00000394662	14.474596	-3.415026966	1.004043	-3.401276	0.00067072	0.00621107	ENSG00000225492	1	89410318	89426243
ENST00000394685	89.54263	3.352778358	1.126385	2.976583	0.002914802	0.019710185	ENSG00000103888	15	80779369	80951771
ENST00000394718	250.84668	-1.568870645	0.258033	-6.080127	1.20E-09	7.67E-08	ENSG00000179841	14	64465498	64474503
ENST00000394815	370.75251	-1.680257722	0.232924	-7.213755	5.44E-13	7.18E-11	ENSG00000167767	12	52168995	52192014
ENST00000394854	1044.5255	-1.192000816	0.232363	-5.129918	2.90E-07	9.30E-06	ENSG00000138814	4	101023417	101347526
ENST00000394945	25.857492	-3.628067143	0.780921	-4.645882	3.39E-06	7.73E-05	ENSG00000152377	5	136975297	137499326

NME1	protein_coding	Yes	NM_000269.3	108
IL36RN	protein_coding	Yes	NM_012275.3	108
LRRIQ1	protein_coding	Yes	NM_001079910.2	108
OSGIN1	protein_coding	Yes	NM_182981.3	108
ATP5MC1	protein_coding	Yes	NM_005175.3	108
TSGA10	protein_coding	Yes	NM_025244.4	108
DBN1	protein_coding	Yes	NM_001363541.2	108
HYDIN	protein_coding	Yes	NM_001270974.2	108
P2RY2	protein_coding	Yes	NM_002564.4	108
FBXO8	protein_coding	Yes	NM_012180.3	108
ERGIC1	protein_coding	Yes	NM_001031711.3	108
ARHGAP9	protein_coding	Yes	NM_032496.4	108
SUCLG1	protein_coding	Yes	NM_003849.4	108
MRPL19	protein_coding	Yes	NM_014763.4	108
CCDC142	protein_coding	Yes	NM_001365575.2	108
SLC4A5	protein_coding	Yes	NM_133478.3	108
RPL17P34	essed_pseudo[illegible]	Yes	-	108
NR2F2	protein_coding	Yes	NM_021005.4	108
MIEN1	protein_coding	Yes	NM_032339.5	108
PRC1	protein_coding	Yes	NM_003981.4	108
PABIR1	protein_coding	Yes	NM_138333.5	108
ZNF385A	protein_coding	Yes	NM_015481.3	108
STK26	protein_coding	Yes	NM_016542.4	108
KIF7	protein_coding	Yes	NM_198525.3	108
MT1X	protein_coding	Yes	NM_005952.4	108
PCDHGA2	protein_coding	Yes	NM_018915.4	108
SEPTIN6	protein_coding	Yes	NM_145799.4	108
MCUB	protein_coding	Yes	NM_017918.5	108
GBP1P1	_unprocessed_[illegible]	Yes	-	108
CEMIP	protein_coding	Yes	NM_001293298.2	108
AKAP5	protein_coding	Yes	NM_004857.3	108
KRT80	protein_coding	Yes	NM_182507.3	108
PPP3CA	protein_coding	Yes	NM_000944.5	108
SPOCK1	protein_coding	Yes	NM_004598.4	108

ENST00000394957	88.64657	-3.81754909	0.447064	-8.539163	1.35E-17	3.67E-15	ENSG00000107738	10	71747555	71773520
ENST00000395018	8.1272134	3.896059113	1.436123	2.712901	0.006669696	0.037525752	ENSG00000213578	15	74826626	74831802
ENST00000395042	40.627799	-1.621513828	0.537909	-3.014475	0.002574242	0.017914381	ENSG00000171700	20	64073180	64079518
ENST00000395067	588.59339	-1.328417622	0.210067	-6.323781	2.55E-10	1.97E-08	ENSG00000066697	9	100427142	100451734
ENST00000395080	70.33284	-4.095353604	0.598608	-6.841463	7.84E-12	8.24E-10	ENSG00000118785	4	87975713	87983411
ENST00000395105	85.224011	-2.903992316	0.415043	-6.996854	2.62E-12	3.02E-10	ENSG00000137868	15	74179465	74202787
ENST00000395145	76.937915	-1.750245782	0.392399	-4.460368	8.18E-06	0.000163585	ENSG00000165215	7	73768996	73770270
ENST00000395184	4.039114	-5.488239552	2.041085	-2.688884	0.007169132	0.039590369	ENSG00000138639	4	85475149	86002666
ENST00000395323	508.77335	-1.389881471	0.208989	-6.6505	2.92E-11	2.76E-09	ENSG00000213626	2	30231533	30260028
ENST00000395348	14.224639	-2.44056749	0.912789	-2.673748	0.007500883	0.040944704	ENSG00000165449	10	59650763	59709850
ENST00000395407	193.36691	-2.616622958	0.291729	-8.969367	2.98E-19	1.03E-16	ENSG00000137819	15	69298911	69407780
ENST00000395743	53.112554	1.21080383	0.463097	2.61458	0.008933723	0.046642834	ENSG00000174808	4	74744758	74794523
ENST00000395748	101.32052	-2.384344929	0.373354	-6.386282	1.70E-10	1.38E-08	ENSG00000109321	4	74445135	74455005
ENST00000395762	429.33966	-1.36980375	0.217234	-6.305658	2.87E-10	2.18E-08	ENSG00000077238	16	27313973	27364778
ENST00000395823	22.669026	-1.853191455	0.683743	-2.710362	0.006720975	0.037752938	ENSG00000213707	22	26560525	26561088
ENST00000395842	64.675517	1.427212395	0.425951	3.350651	0.000806218	0.007174434	ENSG00000073150	22	50170730	50180295
ENST00000395906	180.07012	1.086334635	0.277235	3.918462	8.91E-05	0.001211377	ENSG00000241322	17	15583885	15619704
ENST00000395965	615.95544	1.160553888	0.215644	5.381814	7.37E-08	2.87E-06	ENSG00000113391	5	93617724	94111663
ENST00000396105	2798.829	1.072545994	0.181359	5.913945	3.34E-09	1.88E-07	ENSG00000124201	20	49245899	49278057
ENST00000396308	37.027266	2.205888284	0.560187	3.937772	8.22E-05	0.001134551	ENSG00000139190	12	6462236	6470677
ENST00000396396	31.131035	5.913156997	1.151081	5.137048	2.79E-07	9.01E-06	ENSG00000154330	9	68356610	68531061
ENST00000396402	6.8347689	6.197357197	1.819129	3.406771	0.000657362	0.006111707	ENSG00000137869	15	51208056	51338596
ENST00000396602	1212.3614	1.644831967	0.192576	8.541202	1.33E-17	3.62E-15	ENSG00000166478	11	9461011	9528524
ENST00000396618	14.870042	-5.911101454	1.694429	-3.488551	0.000485646	0.004818994	ENSG00000100473	14	30874558	30890618
ENST00000396654	165.03837	-1.48760085	0.28981	-5.133026	2.85E-07	9.17E-06	ENSG00000152056	2	223755325	223837582
ENST00000396679	566.21818	-1.235036828	0.21071	-5.861309	4.59E-09	2.51E-07	ENSG00000123219	5	65517765	65563168
ENST00000396872	30.270424	-2.373582532	0.618763	-3.836013	0.000125048	0.001606563	ENSG00000164638	7	5282942	5306912
ENST00000396946	393.53934	-4.202431849	0.268396	-15.65757	2.95E-55	2.37E-51	ENSG00000198286	7	2906141	3043867
ENST00000396959	253.90557	-1.097966905	0.273211	-4.018758	5.85E-05	0.000856819	ENSG00000213943	3	12787392	12788671
ENST00000396984	3051.8437	-1.328562848	0.173414	-7.661237	1.84E-14	3.32E-12	ENSG00000180596	6	26123466	26123926
ENST00000397061	222.97548	-1.837022334	0.268762	-6.835129	8.19E-12	8.59E-10	ENSG00000213965	19	32691820	32713792
ENST00000397066	535.78565	-1.161963942	0.229823	-5.055902	4.28E-07	1.31E-05	ENSG00000162063	16	2429446	2458854
ENST00000397098	1057.4178	-1.216547413	0.25693	-4.734929	2.19E-06	5.35E-05	ENSG00000146540	7	997005	1138247
ENST00000397128	1089.3359	-1.04288483	0.186876	-5.580626	2.40E-08	1.06E-06	ENSG00000132356	5	40759388	40798374

VSIR	protein_coding	Yes	NM_022153.2	108
CPLX3	protein_coding	Yes	NM_001030005.3	108
RGS19	protein_coding	Yes	NM_005873.3	108
MSANTD3	protein_coding	Yes	NM_080655.3	108
SPP1	protein_coding	Yes	NM_001040058.2	108
STRA6	protein_coding	Yes	NM_022369.4	108
CLDN3	protein_coding	Yes	NM_001306.4	108
ARHGAP24	protein_coding	Yes	NM_001025616.3	108
LBH	protein_coding	Yes	NM_030915.4	108
SLC16A9	protein_coding	Yes	NM_194298.3	108
PAQR5	protein_coding	Yes	NM_017705.4	108
BTC	protein_coding	Yes	NM_001729.4	108
AREG	protein_coding	Yes	NM_001657.4	108
IL4R	protein_coding	Yes	NM_000418.4	108
HMGB1P10	essed_pseudog	Yes	-	108
PANX2	protein_coding	Yes	NM_052839.4	108
FBXW10B	protein_coding	Yes	NM_006382.4	108
FAM172A	protein_coding	Yes	NM_032042.6	108
ZNFX1	protein_coding	Yes	NM_021035.3	108
VAMP1	protein_coding	Yes	NM_014231.5	108
PGM5	protein_coding	Yes	NM_021965.4	108
CYP19A1	protein_coding	Yes	NM_000103.4	108
ZNF143	protein_coding	Yes	NM_003442.6	108
COCH	protein_coding	Yes	NM_004086.3	108
AP1S3	protein_coding	Yes	NM_001039569.2	108
CENPK	protein_coding	Yes	NM_022145.5	108
SLC29A4	protein_coding	Yes	NM_153247.4	108
CARD11	protein_coding	Yes	NM_032415.7	108
KRT18P17	essed_pseudog	Yes	-	108
H2BC4	protein_coding	Yes	NM_003526.3	108
NUDT19	protein_coding	Yes	NM_001105570.2	108
CCNF	protein_coding	Yes	NM_001761.3	108
C7orf50	protein_coding	Yes	NM_001318252.2	108
PRKAA1	protein_coding	Yes	NM_006251.6	108

ENST00000397146	217.75312	-2.237546251	0.315816	-7.084962	1.39E-12	1.70E-10	ENSG00000123243	10	7559269	7666966
ENST00000397163	35.759316	1.703246381	0.564322	3.018219	0.002542654	0.017739104	ENSG00000092529	15	42359500	42412317
ENST00000397278	8.4617331	-5.081317247	1.769969	-2.870851	0.004093686	0.025701218	ENSG00000100342	22	36253132	36267525
ENST00000397298	168.29576	-1.096941564	0.287565	-3.814592	0.000136408	0.001728476	ENSG00000214026	11	1947331	1956600
ENST00000397375	577.58112	-1.112789787	0.251875	-4.418031	9.96E-06	0.000193339	ENSG00000074071	16	1771894	1773134
ENST00000397541	3.8949411	5.386514595	2.058112	2.617211	0.008865141	0.04637373	ENSG00000214102	7	141708352	141731271
ENST00000397545	34.602199	1.762311362	0.564728	3.120635	0.001804617	0.013536271	ENSG00000141519	17	80036641	80100613
ENST00000397572	430.97233	-1.448397387	0.224834	-6.442066	1.18E-10	9.88E-09	ENSG00000214114	1	38862492	38873348
ENST00000397701	79.379329	1.41675543	0.402333	3.521354	0.000429349	0.004373293	ENSG00000182362	21	46286341	46297751
ENST00000397764	129.8964	-1.522031119	0.335882	-4.531452	5.86E-06	0.000123844	ENSG00000214194	7	113116717	113118554
ENST00000397766	137.38551	1.01759105	0.310981	3.272202	0.001067135	0.008931525	ENSG00000182405	15	34102082	34108686
ENST00000397799	4.1523414	5.47828107	2.024699	2.705726	0.00681552	0.038152509	ENSG00000171812	1	36095238	36125222
ENST00000397820	21.348701	2.738770543	0.754694	3.62898	0.000284543	0.003153546	ENSG00000214212	19	10848414	10869790
ENST00000397821	704.28296	-1.241844756	0.194923	-6.370958	1.88E-10	1.50E-08	ENSG00000147010	X	19533976	19887600
ENST00000397910	201.26709	-4.133542545	1.246809	-3.315297	0.000915457	0.007933825	ENSG00000181143	19	8848843	8981342
ENST00000397942	1869.2197	-1.096455926	0.197034	-5.564792	2.62E-08	1.15E-06	ENSG00000170677	18	70289044	70330199
ENST00000398297	6.9092565	6.211229184	1.819994	3.412774	0.000643052	0.006010193	ENSG00000215120	X	71527813	71530225
ENST00000398357	354.64962	-1.287714629	0.227908	-5.650141	1.60E-08	7.39E-07	ENSG00000214455	13	45390352	45391267
ENST00000398449	21.376582	-2.052039374	0.707938	-2.898617	0.003748129	0.023967938	ENSG00000160179	21	42219139	42297244
ENST00000398458	12.739254	7.095081025	1.657697	4.280084	1.87E-05	0.000330131	ENSG00000186452	12	50842925	50887884
ENST00000398516	24.507157	2.441974696	0.694155	3.517911	0.000434958	0.004420377	ENSG00000145217	4	987656	993404
ENST00000398540	377.8699	-1.957677555	0.229474	-8.531144	1.45E-17	3.91E-15	ENSG00000114541	3	69168781	69386088
ENST00000398545	35.332946	1.923646319	0.565575	3.401223	0.00067085	0.00621107	ENSG00000172361	18	50227192	50266495
ENST00000398594	155.90468	1.421613152	0.301209	4.719689	2.36E-06	5.69E-05	ENSG00000254122	5	141417676	141512975
ENST00000398604	64.201113	1.25151786	0.413632	3.025676	0.002480781	0.017389888	ENSG00000253767	5	141392632	141512975
ENST00000398631	687.05077	1.323849162	0.207217	6.3887	1.67E-10	1.36E-08	ENSG00000251664	5	140875307	141012347
ENST00000398640	527.91833	1.24476268	0.22287	5.585161	2.33E-08	1.03E-06	ENSG00000249158	5	140868956	141012347
ENST00000398806	71.547255	-1.981636513	0.412465	-4.804373	1.55E-06	3.95E-05	ENSG00000056998	X	2828929	2882818
ENST00000398832	20.352602	2.017022214	0.731967	2.755619	0.005858117	0.033883837	ENSG00000214708	17	32141225	32143135
ENST00000398841	297.65623	1.514094346	0.335999	4.506249	6.60E-06	0.000136955	ENSG00000090238	16	30092313	30096213
ENST00000398992	5.6222553	5.915377793	1.890077	3.129702	0.001749835	0.013209876	ENSG00000214788	11	59942878	59970146
ENST00000399002	104.31575	-1.605201324	0.358092	-4.482654	7.37E-06	0.000150111	ENSG00000172296	20	13008971	13169103
ENST00000399134	11.031285	3.752168802	1.189143	3.155354	0.001603034	0.012340006	ENSG00000214860	17	18377777	18389647
ENST00000399220	11.26934	6.918201685	1.682962	4.110729	3.94E-05	0.000618018	ENSG00000168329	3	39263495	39280036

ITIH5	protein_coding	Yes	NM_030569.7	108
CAPN3	protein_coding	Yes	NM_000070.3	108
APOL1	protein_coding	Yes	NM_003661.4	108
MRPL23	protein_coding	Yes	NM_021134.4	108
MRPS34	protein_coding	Yes	NM_023936.2	108
WEE2	protein_coding	Yes	NM_001105558.1	108
CCDC40	protein_coding	Yes	NM_017950.4	108
MYCBP	protein_coding	Yes	NM_012333.5	108
YBEY	protein_coding	Yes	NM_001314025.2	108
SMIM30	protein_coding	Yes	NM_001352688.2	108
PGBD4	protein_coding	Yes	NM_152595.5	108
COL8A2	protein_coding	Yes	NM_005202.4	108
C19orf38	protein_coding	Yes	NM_001136482.3	108
SH3KBP1	protein_coding	Yes	NM_031892.3	108
MUC16	protein_coding	Yes	-	108
SOCS6	protein_coding	Yes	NM_004232.4	108
SOCS6P1	essed_pseudog	Yes	-	108
RCN1P2	essed_pseudog	Yes	-	108
ABCG1	protein_coding	Yes	NM_016818.3	108
TMPRSS12	protein_coding	Yes	NM_182559.3	108
SLC26A1	protein_coding	Yes	NM_022042.4	108
FRMD4B	protein_coding	Yes	NM_015123.3	108
CFAP53	protein_coding	Yes	NM_145020.5	108
PCDHGB7	protein_coding	Yes	NM_018927.4	108
PCDHGA8	protein_coding	Yes	NM_032088.2	108
PCDHA12	protein_coding	Yes	NM_018903.4	108
PCDHA11	protein_coding	Yes	NM_018902.5	108
GYG2	protein_coding	Yes	NM_001079855.2	108
-	lncRNA	Yes	-	108
YPEL3	protein_coding	Yes	NM_031477.5	108
-	lncRNA	Yes	-	108
SPTLC3	protein_coding	Yes	NM_018327.4	108
EVPLL	protein_coding	Yes	NM_001145127.2	108
CX3CR1	protein_coding	Yes	NM_001337.4	108

ENST00000399506	77.374891	2.412437378	0.440654	5.47468	4.38E-08	1.82E-06	ENSG00000132535	17	7187186	7217627
ENST00000399539	23.49582	-1.846597455	0.677818	-2.724328	0.006443249	0.036533557	ENSG00000237517	22	18985835	18994501
ENST00000399815	144.55288	1.474388149	0.357499	4.124178	3.72E-05	0.000587994	ENSG00000288681	11	308407	315272
ENST00000400324	586.00271	-1.659125675	0.204522	-8.112227	4.97E-16	1.12E-13	ENSG00000139734	13	59665582	60163928
ENST00000400382	23.969306	1.980504046	0.692243	2.860996	0.004223124	0.026311186	ENSG00000100031	22	24603192	24628996
ENST00000400463	47.127891	1.615804124	0.491105	3.290138	0.001001383	0.008491626	ENSG00000117245	1	20664013	20718007
ENST00000400521	182.43413	-1.0357011	0.303261	-3.415209	0.000637331	0.00596953	ENSG00000184470	22	19875521	19941818
ENST00000400522	12.221945	3.118100266	1.031615	3.022542	0.002506612	0.017534938	ENSG00000215529	20	32858922	32961845
ENST00000400590	3596.919	-1.11983338	0.194681	-5.752152	8.81E-09	4.46E-07	ENSG00000180776	13	21372570	21459303
ENST00000400897	11.160874	2.95075187	1.08407	2.72192	0.006490386	0.036748876	ENSG00000009724	1	11026522	11047239
ENST00000401061	78.720379	-1.148590656	0.379548	-3.026207	0.002476423	0.017366801	ENSG00000132763	1	45500299	45513382
ENST00000401533	13.551754	-3.700425998	1.067622	-3.466045	0.000528174	0.005148344	ENSG00000157833	2	26173087	26189663
ENST00000401827	657.15999	-1.188148447	0.205059	-5.79417	6.87E-09	3.59E-07	ENSG00000205268	8	65714333	65842064
ENST00000402142	9.5171964	6.675047853	1.722403	3.875426	0.000106438	0.001402104	ENSG00000100346	22	39570752	39689735
ENST00000402676	36.118628	-6.188659001	1.282904	-4.823946	1.41E-06	3.63E-05	ENSG00000131016	6	151239966	151358559
ENST00000402738	236.56373	1.095533994	0.300777	3.642342	0.000270169	0.003022806	ENSG00000138641	4	88592479	88708539
ENST00000402918	35.190712	-2.114923028	0.563727	-3.751679	0.000175654	0.002127263	ENSG00000166897	22	37367959	37427479
ENST00000402938	73.092624	1.992567605	0.405819	4.909992	9.11E-07	2.49E-05	ENSG00000111886	6	89254463	89315299
ENST00000403245	220.18861	1.124242316	0.265048	4.241655	2.22E-05	0.00038009	ENSG00000135315	6	84124249	84227643
ENST00000403683	379.0177	-1.560095016	0.232526	-6.709332	1.96E-11	1.92E-09	ENSG00000203852	1	149852607	149853125
ENST00000403729	15.252453	-7.404016053	1.628744	-4.545845	5.47E-06	0.000117075	ENSG00000163297	4	79901145	80073229
ENST00000403903	107.68376	1.92964595	0.368254	5.239987	1.61E-07	5.60E-06	ENSG00000220201	19	10304802	10309880
ENST00000403994	483.64216	-1.228948676	0.271064	-4.533799	5.79E-06	0.000122683	ENSG00000140416	15	63042746	63066178
ENST00000404077	41.453378	2.333778265	0.54225	4.303875	1.68E-05	0.000302043	ENSG00000169402	7	6754102	6798765
ENST00000404406	25.894237	2.235936452	0.67331	3.320813	0.000897557	0.007812539	ENSG00000155026	7	5926135	5970689
ENST00000404568	29.581871	3.003388178	0.670084	4.482108	7.39E-06	0.000150395	ENSG00000144045	2	74518130	74526231
ENST00000404589	33.422492	-2.150988151	0.586402	-3.66811	0.000244351	0.002783298	ENSG00000168301	3	58492095	58502356
ENST00000404826	4.2992359	-5.577977538	2.008926	-2.776596	0.005493135	0.032198071	ENSG00000146555	7	3301251	4269000
ENST00000405166	3.9197703	5.394631232	2.054307	2.626011	0.008639213	0.045513691	ENSG00000218803	6	111046867	111047521
ENST00000405655	87.895854	-1.84861332	0.377946	-4.891205	1.00E-06	2.70E-05	ENSG00000185686	22	22547700	22559265
ENST00000405876	112.76749	-2.067268954	0.341184	-6.059097	1.37E-09	8.61E-08	ENSG00000139437	12	109900273	109918069
ENST00000406329	5.132284	5.783864116	1.927267	3.00107	0.002690326	0.018531834	ENSG00000219863	6	53354714	53355065
ENST00000406427	17.40138	2.559442718	0.819499	3.12318	0.001789083	0.013453521	ENSG00000130653	9	137459951	137550402
ENST00000406438	1132.6814	1.17184355	0.181545	6.454826	1.08E-10	9.12E-09	ENSG00000176994	17	18315292	18328056

DLG4	protein_coding	Yes	NM_001321075.3	108
CELSR1P1	unprocessed_p	Yes	-	108
-	protein_coding	Yes	-	108
DIAPH3	protein_coding	Yes	NM_001042517.2	108
GGT1	protein_coding	Yes	NM_001288833.2	108
KIF17	protein_coding	Yes	NM_001122819.3	108
TXNRD2	protein_coding	Yes	NM_006440.5	108
EFCAB8	protein_coding	Yes	NM_001143967.2	108
ZDHHC20	protein_coding	Yes	NM_001330059.2	108
MASP2	protein_coding	Yes	NM_006610.4	108
MMACHC	protein_coding	Yes	NM_015506.3	108
GAREM2	protein_coding	Yes	NM_001168241.2	108
PDE7A	protein_coding	Yes	NM_001242318.3	108
CACNA1I	protein_coding	Yes	NM_021096.4	108
AKAP12	protein_coding	Yes	NM_005100.4	108
HERC3	protein_coding	Yes	NM_014606.3	108
ELFN2	protein_coding	Yes	NM_052906.5	108
GABRR2	protein_coding	Yes	NM_002043.5	108
CEP162	protein_coding	Yes	NM_014895.4	108
H3C15	protein_coding	Yes	NM_001005464.3	108
ANTXR2	protein_coding	Yes	NM_058172.6	108
ZGLP1	protein_coding	Yes	-	108
TPM1	protein_coding	Yes	NM_001018005.2	108
RSPH10B2	protein_coding	Yes	NM_001099697.2	108
RSPH10B	protein_coding	Yes	NM_173565.5	108
DQX1	protein_coding	Yes	NM_133637.3	108
KCTD6	protein_coding	Yes	NM_001128214.2	108
SDK1	protein_coding	Yes	NM_152744.4	108
GSTM2P1	essed_pseudog	Yes	-	108
PRAME	protein_coding	Yes	NM_206956.3	108
TCHP	protein_coding	Yes	NM_001143852.2	108
RPL31P28	essed_pseudog	Yes	-	108
PNPLA7	protein_coding	Yes	NM_001098537.3	108
SMCR8	protein_coding	Yes	NM_144775.3	108

ENST00000406489	51.766507	2.228165669	0.479968	4.642322	3.45E-06	7.85E-05	ENSG00000219891	6	28092426	28092845
ENST00000406819	9.2846254	6.63915141	1.728632	3.840698	0.000122685	0.001582479	ENSG00000219249	6	158725746	158726803
ENST00000406875	184.976	-1.452205764	0.309428	-4.693188	2.69E-06	6.35E-05	ENSG00000064393	7	139561569	139777998
ENST00000407464	1532.6373	-1.660451889	0.179082	-9.272034	1.83E-20	7.03E-18	ENSG00000169635	22	21417370	21451463
ENST00000407977	1163.4024	1.041026368	0.197583	5.268797	1.37E-07	4.89E-06	ENSG00000108375	17	58353675	58417534
ENST00000407997	204.75882	-5.043141527	0.98436	-5.123267	3.00E-07	9.59E-06	ENSG00000239713	22	39077274	39087743
ENST00000408954	273.01036	-5.337440212	0.383724	-13.90958	5.54E-44	1.69E-40	ENSG00000196730	9	87497866	87708634
ENST00000408957	215.28861	1.158453223	0.261411	4.431541	9.36E-06	0.000183313	ENSG00000221944	2	232543882	232550557
ENST00000408968	300.25164	1.466086335	0.299055	4.902395	9.47E-07	2.57E-05	ENSG00000185885	11	314039	315272
ENST00000409150	127.72186	-1.254300139	0.317007	-3.956697	7.60E-05	0.001062881	ENSG00000189362	2	190504337	190534722
ENST00000409235	91.014523	2.751122673	0.46977	5.856316	4.73E-09	2.58E-07	ENSG00000099338	19	38335829	38370943
ENST00000409508	23.068064	2.469158114	0.715479	3.451056	0.000558398	0.005377946	ENSG00000105877	7	21543038	21901839
ENST00000409548	1036.3454	2.825107969	0.209676	13.47368	2.23E-41	4.54E-38	ENSG00000142102	11	289125	296107
ENST00000409561	7.7789555	4.899232981	1.759724	2.784092	0.005367782	0.031643421	ENSG00000187833	2	73784182	73817148
ENST00000409687	1790.9887	-1.731899192	0.256347	-6.756078	1.42E-11	1.43E-09	ENSG00000186193	9	137062126	137070557
ENST00000409709	510.26079	-1.775920263	0.303349	-5.854385	4.79E-09	2.60E-07	ENSG00000137474	11	77128245	77215241
ENST00000409746	670.18993	1.163501862	0.213468	5.450465	5.02E-08	2.06E-06	ENSG00000152229	18	45983535	46072260
ENST00000409873	3.8472345	-5.41543792	2.093812	-2.586401	0.009698407	0.049680498	ENSG00000290738	16	90039760	90047625
ENST00000409878	51.54725	1.810656751	0.483243	3.74689	0.000179041	0.002160553	ENSG00000213901	2	219161464	219170029
ENST00000410023	88.957971	-2.22626522	0.380967	-5.843726	5.10E-09	2.75E-07	ENSG00000115594	2	102142745	102179874
ENST00000410062	268.64249	-1.198962785	0.256496	-4.674394	2.95E-06	6.86E-05	ENSG00000170035	2	180980604	181063425
ENST00000410067	147.55761	-1.079014557	0.304077	-3.548495	0.00038744	0.004025504	ENSG00000197223	2	68041129	68063004
ENST00000410792	13.91885	-4.159256518	1.12916	-3.683495	0.000230057	0.002649997	ENSG00000222724	2	88016353	88016547
ENST00000411421	133.19111	-1.021340239	0.308818	-3.307252	0.000942162	0.008114595	ENSG00000232380	13	68985057	68986064
ENST00000412232	18.680506	-3.551848224	0.88413	-4.017336	5.89E-05	0.000860599	ENSG00000020181	8	37796882	37844896
ENST00000412916	1079.6947	-1.115608862	0.187156	-5.960847	2.51E-09	1.46E-07	ENSG00000067955	16	67029148	67101058
ENST00000412976	98.17755	-1.422065802	0.376327	-3.7788	0.000157586	0.001945711	ENSG00000235489	10	64168958	64170850
ENST00000413053	24.017702	8.01144232	1.566425	5.114475	3.15E-07	9.98E-06	ENSG00000229719	11	64889559	64893449
ENST00000413139	30.654144	-2.661139382	0.643652	-4.134441	3.56E-05	0.000565803	ENSG00000008277	7	87934250	88202889
ENST00000414150	257.33602	-1.014633255	0.257944	-3.933543	8.37E-05	0.001150648	ENSG00000271723	1	54641799	54742231
ENST00000414583	4.6423127	5.639148354	1.971416	2.860456	0.004230322	0.026324826	ENSG00000290696	22	24251827	24265525
ENST00000416167	61.457395	-3.885541179	0.536373	-7.244104	4.35E-13	5.84E-11	ENSG00000167244	11	2129116	2139389
ENST00000416283	578.72669	-1.287648108	0.205558	-6.26417	3.75E-10	2.73E-08	ENSG00000006576	7	77798791	77957503
ENST00000416471	96.998526	1.11303947	0.348884	3.190284	0.001421331	0.011222425	ENSG00000241790	2	201621645	201623430

ZSCAN12P1	unprocessed_p	Yes	-	108
AMZ2P2	essed_pseudog	Yes	-	108
HIPK2	protein_coding	Yes	NM_022740.5	108
HIC2	protein_coding	Yes	NM_015094.3	108
RNF43	protein_coding	Yes	NM_017763.6	108
APOBEC3G	protein_coding	Yes	NM_021822.4	108
DAPK1	protein_coding	Yes	NM_004938.4	108
TIGD1	protein_coding	Yes	NM_145702.4	108
IFITM1	protein_coding	Yes	NM_003641.5	108
NEMP2	protein_coding	Yes	NM_001142645.2	108
CATSPERG	protein_coding	Yes	NM_021185.5	108
DNAH11	protein_coding	Yes	NM_001277115.2	108
PGGHG	protein_coding	Yes	NM_025092.5	108
C2orf78	protein_coding	Yes	NM_001080474.3	108
SAPCD2	protein_coding	Yes	NM_178448.4	108
MYO7A	protein_coding	Yes	NM_000260.4	108
PSTPIP2	protein_coding	Yes	NM_024430.4	108
URAHP	lncRNA	Yes	-	108
SLC23A3	protein_coding	Yes	NM_001144889.2	108
IL1R1	protein_coding	Yes	NM_000877.4	108
UBE2E3	protein_coding	Yes	NM_006357.4	108
C1D	protein_coding	Yes	NM_173177.3	108
RNU2-63P	snRNA	Yes	-	108
ZDHHC20P4	essed_pseudog	Yes	-	108
ADGRA2	protein_coding	Yes	NM_032777.10	108
CBFB	protein_coding	Yes	NM_022845.3	108
DBF4P1	essed_pseudog	Yes	-	108
MIR194-2HG	lncRNA	Yes	-	108
ADAM22	protein_coding	Yes	NM_001324418.2	108
MROH7-TTC4	protein_coding	Yes	-	108
POM121L9P	lncRNA	Yes	-	108
IGF2	protein_coding	Yes	NM_000612.6	108
PHTF2	protein_coding	Yes	-	108
ENO1P4	essed_pseudog	Yes	-	108

ENST00000416575	7.0921691	6.250381496	1.806901	3.459171	0.00054184	0.005252334	ENSG00000227210	2	19990210	20004795
ENST00000416841	150.96742	1.032755343	0.311805	3.312188	0.000925695	0.007999889	ENSG00000179304	X	52897329	52908560
ENST00000417257	285.50203	-1.096037597	0.277933	-3.943532	8.03E-05	0.001112062	ENSG00000171877	15	43870763	44195271
ENST00000417626	330.31605	-1.14895055	0.309035	-3.717863	0.000200915	0.00237506	ENSG00000214706	3	50287731	50292429
ENST00000417761	32.875138	1.608943673	0.568254	2.831381	0.004634749	0.028253207	ENSG00000262560	15	43772616	43799133
ENST00000417927	95.274914	2.307580952	0.384323	6.004268	1.92E-09	1.15E-07	ENSG00000227145	4	122618982	122689156
ENST00000418273	215.60696	1.186167797	0.281813	4.209056	2.56E-05	0.000429403	ENSG00000272734	10	86965739	86971311
ENST00000418331	723.89589	-1.000844464	0.194954	-5.133751	2.84E-07	9.14E-06	ENSG00000149177	11	47980558	48170839
ENST00000418539	157.50121	-2.688310948	0.363319	-7.399308	1.37E-13	2.04E-11	ENSG00000236824	2	47335314	47335514
ENST00000419089	38.013869	-1.997238051	0.543677	-3.673573	0.000239182	0.002737199	ENSG00000225071	X	24429572	24429920
ENST00000419457	88.289261	-2.214679468	0.379304	-5.838795	5.26E-09	2.82E-07	ENSG00000233588	13	28722385	28724021
ENST00000420022	29.251032	1.776523089	0.613515	2.89565	0.003783745	0.024151287	ENSG00000224420	19	49689593	49690575
ENST00000420195	117.41733	1.145550123	0.391401	2.926794	0.00342476	0.022306459	ENSG00000224660	3	15254183	15264493
ENST00000420323	65.921509	2.247798078	0.459146	4.895605	9.80E-07	2.65E-05	ENSG00000114841	3	52316318	52400492
ENST00000420330	38.516626	1.70191384	0.559192	3.043522	0.002338261	0.016596506	ENSG00000236255	2	117833936	117841658
ENST00000420658	213.24099	-1.635509651	0.267805	-6.107102	1.01E-09	6.61E-08	ENSG00000196526	4	7758713	7939861
ENST00000420699	1074.8469	1.162799473	0.201295	5.776595	7.62E-09	3.94E-07	ENSG00000135976	2	97113152	97264521
ENST00000421003	26.104152	1.875820356	0.645648	2.905331	0.003668649	0.023570629	ENSG00000227953	1	246776012	246792385
ENST00000421404	27.107508	-2.017970746	0.632441	-3.190763	0.001418974	0.011207229	ENSG00000236570	3	18538672	18539899
ENST00000421865	215.36514	-1.915557273	0.354452	-5.404286	6.51E-08	2.59E-06	ENSG00000196569	6	128883137	129516566
ENST00000421999	356.54048	-1.912553106	0.277797	-6.884727	5.79E-12	6.29E-10	ENSG00000184220	3	99817861	100181732
ENST00000422538	7.9551378	-4.990126863	1.792891	-2.783285	0.005381145	0.031707841	ENSG00000111405	12	47709733	47725490
ENST00000422689	28.26786	1.931305139	0.649993	2.971271	0.002965702	0.019973509	ENSG00000221923	19	52369922	52385795
ENST00000422704	176.9623	-1.052422186	0.280713	-3.749106	0.000177466	0.002144949	ENSG00000187764	9	89377234	89498113
ENST00000422816	720.0615	-1.343448365	0.214545	-6.261838	3.80E-10	2.77E-08	ENSG00000229944	17	49424268	49424922
ENST00000422946	3.8701119	5.378338053	2.06548	2.603917	0.009216513	0.047791845	ENSG00000223882	3	184006337	184011419
ENST00000423158	5.6470845	5.921009198	1.889355	3.133879	0.00172512	0.013059518	ENSG00000185652	12	5432107	5495299
ENST00000423313	151.08983	-1.201752715	0.295127	-4.071979	4.66E-05	0.000711678	ENSG00000235750	1	175156985	175192776
ENST00000423609	143.0071	-1.079139356	0.316617	-3.408346	0.00065358	0.006089177	ENSG00000213856	X	49632499	49633349
ENST00000423855	435.92042	1.416944581	0.27847	5.088313	3.61E-07	1.13E-05	ENSG00000185946	1	103525698	103555239
ENST00000424215	4.1523414	5.47828107	2.024699	2.705726	0.00681552	0.038152509	ENSG00000228037	1	2581559	2584533
ENST00000424296	78.358663	1.661769032	0.401662	4.137229	3.52E-05	0.000560656	ENSG00000155085	6	109492854	109691202
ENST00000424848	2718.5187	-1.569040592	0.169502	-9.256758	2.11E-20	8.03E-18	ENSG00000105810	7	92604920	92836573
ENST00000425159	95.623289	1.202314624	0.36695	3.276509	0.00105099	0.008817702	ENSG00000291120	17	47450567	47492493

WDR35-DT	lncRNA	Yes	-	108
FAM156B	protein_coding	Yes	NM_001321178.3	108
FRMD5	protein_coding	Yes	NM_032892.5	108
IFRD2	protein_coding	Yes	NM_006764.5	108
-	protein_coding	Yes	-	108
IL21-AS1	lncRNA	Yes	-	108
ADIRF-AS1	lncRNA	Yes	-	108
PTPRJ	protein_coding	Yes	NM_002843.4	108
BCYRN1	scRNA	Yes	-	108
RPS26P58	essed_pseudo	Yes	-	108
CYP51A1P2	essed_pseudo	Yes	-	108
ADM5	protein_coding	Yes	NM_001101340.2	108
SH3BP5-AS1	lncRNA	Yes	-	108
DNAH1	protein_coding	Yes	NM_015512.5	108
-	lncRNA	Yes	-	108
AFAP1	protein_coding	Yes	NM_001134647.2	108
ANKRD36	protein_coding	Yes	NM_001354587.1	108
LINC01341	lncRNA	Yes	-	108
RAD23BP1	essed_pseudo	Yes	-	108
LAMA2	protein_coding	Yes	NM_000426.4	108
CMSS1	protein_coding	Yes	NM_032359.4	108
ENDOU	protein_coding	Yes	NM_001172439.2	108
ZNF880	protein_coding	Yes	NM_001145434.2	108
SEMA4D	protein_coding	Yes	NM_001371194.2	108
EIF4EP2	essed_pseudo	Yes	-	108
ABCC5-AS1	lncRNA	Yes	-	108
NTF3	protein_coding	Yes	NM_001102654.2	108
KIAA0040	protein_coding	Yes	NM_014656.3	108
VDAC1P2	essed_pseudo	Yes	-	108
RNPC3	protein_coding	Yes	NM_017619.4	108
-	lncRNA	Yes	-	108
AK9	protein_coding	Yes	NM_001145128.3	108
CDK6	protein_coding	Yes	NM_001145306.2	108
MRPL45P2	lncRNA	Yes	-	108

ENST00000425248	340.38092	1.049182412	0.23709	4.425241	9.63E-06	0.000187876	ENSG00000158623	7	130506237	130668748
ENST00000425346	87.828364	1.296279905	0.388315	3.338216	0.000843182	0.007436061	ENSG00000114395	3	50350861	50354069
ENST00000425534	32.516664	-3.778924676	0.716616	-5.273288	1.34E-07	4.78E-06	ENSG00000163412	3	71675413	71725402
ENST00000425597	91.672132	1.868859973	0.373427	5.004619	5.60E-07	1.64E-05	ENSG00000198794	15	74995562	75021495
ENST00000425629	408.55636	-2.111147468	0.6432	-3.282259	0.00102979	0.008672779	ENSG00000105492	19	51517818	51531670
ENST00000425666	22.50019	-3.606027587	0.816678	-4.415482	1.01E-05	0.00019533	ENSG00000231107	9	90300895	90433566
ENST00000425740	4.4345708	5.571739292	1.995746	2.791808	0.005241441	0.031089836	ENSG00000236017	X	1401810	1413915
ENST00000425779	6.037739	6.020211337	1.869475	3.220269	0.001280706	0.010336457	ENSG00000236498	2	61868431	61886082
ENST00000426361	980.42487	-1.217718684	0.185086	-6.579196	4.73E-11	4.28E-09	ENSG00000291080	8	144973588	145002895
ENST00000426991	107.4205	-1.184015639	0.429862	-2.75441	0.005879813	0.033972272	ENSG00000229152	13	110894638	110899172
ENST00000427219	75.373819	-1.347697863	0.409542	-3.29074	0.000999241	0.008477806	ENSG00000234354	13	100539900	100540248
ENST00000427277	37.498561	-1.980447508	0.543902	-3.641186	0.000271385	0.003035084	ENSG00000165238	9	93184138	93320569
ENST00000427587	93.843312	-1.759234257	0.388878	-4.523873	6.07E-06	0.00012752	ENSG00000118894	16	5084283	5097795
ENST00000428066	27679.786	-2.584803876	0.189215	-13.66066	1.74E-42	4.03E-39	ENSG00000130600	11	1995175	2001470
ENST00000428091	108.68085	-1.327983216	0.341663	-3.886824	0.000101564	0.001349801	ENSG00000224126	17	18677260	18677757
ENST00000428289	39.263897	2.115754117	0.543069	3.895924	9.78E-05	0.00130763	ENSG00000233030	1	149785658	149793020
ENST00000428301	5.5974261	5.909724272	1.89259	3.122559	0.001792859	0.013471947	ENSG00000290541	21	13843132	13848364
ENST00000428443	34.422344	-5.038106297	1.845641	-2.729733	0.006338573	0.036044635	ENSG00000187231	2	179101677	179264832
ENST00000428512	185.09164	-1.06291427	0.305368	-3.48077	0.000499975	0.004928001	ENSG00000229097	10	70163684	70164124
ENST00000428667	5.3896842	5.85406504	1.907255	3.069367	0.002145128	0.015504393	ENSG00000233818	21	36445730	36532408
ENST00000428826	575.87928	1.025210336	0.26532	3.864054	0.000111521	0.001459344	ENSG00000108799	17	42700274	42745040
ENST00000428956	23.549576	2.380728339	0.696688	3.417207	0.000632672	0.005935745	ENSG00000244731	6	31982056	32002681
ENST00000429032	5.132284	5.783864116	1.927267	3.00107	0.002690326	0.018531834	ENSG00000234515	6	32879215	32879833
ENST00000429588	6.8347689	6.197357197	1.819129	3.406771	0.000657362	0.006111707	ENSG00000230479	21	36430359	36481070
ENST00000429844	4.6174835	5.632298037	1.975236	2.851455	0.004351962	0.026886003	ENSG00000230592	X	100155884	100156773
ENST00000430034	20.632907	-4.353436005	0.986325	-4.413794	1.02E-05	0.000196685	ENSG00000240801	11	2129120	2129964
ENST00000430070	605.13667	1.469772911	0.201689	7.287325	3.16E-13	4.36E-11	ENSG00000123444	11	47572196	47578970
ENST00000430334	291.02282	1.209597811	0.280366	4.314356	1.60E-05	0.000290086	ENSG00000225190	17	45435899	45490721
ENST00000430970	15.604594	7.388547272	1.621498	4.556619	5.20E-06	0.000111921	ENSG00000234134	10	125718770	125719365
ENST00000431042	103.59254	-1.119250487	0.355577	-3.147701	0.001645598	0.012587564	ENSG00000173262	12	7812513	7872915
ENST00000431232	1029.3113	-1.278047344	0.218982	-5.836318	5.34E-09	2.86E-07	ENSG00000129925	16	370787	381978
ENST00000431877	9110.3776	-1.316369188	0.160827	-8.184977	2.72E-16	6.38E-14	ENSG00000138434	2	181891729	181930738
ENST00000431955	15.944647	4.30870678	1.114968	3.864422	0.000111353	0.001457396	ENSG00000241720	1	109725819	109775252
ENST00000432120	69.263196	-1.124614032	0.428249	-2.626073	0.008637627	0.045513691	ENSG00000244332	10	94577438	94611238

COPG2	protein_coding	Yes	NM_012133.6	108
CYB561D2	protein_coding	Yes	NM_001291284.2	108
EIF4E3	protein_coding	Yes	NM_001134651.2	108
SCAMP5	protein_coding	Yes	NM_138967.4	108
SIGLEC6	protein_coding	Yes	NM_001245.7	108
LINC01508	lncRNA	Yes	-	108
ASMTL-AS1	lncRNA	Yes	-	108
-	lncRNA	Yes	-	108
ZNF252P	lncRNA	Yes	-	108
ANKRD10-IT1	lncRNA	Yes	-	108
RPS26P47	essed_pseudog	Yes	-	108
WNK2	protein_coding	Yes	NM_006648.4	108
EEF2KMT	protein_coding	Yes	NM_201400.4	108
H19	lncRNA	Yes	-	108
UBE2SP2	essed_pseudog	Yes	-	108
-	lncRNA	Yes	-	108
CYP4F29P	lncRNA	Yes	-	108
SESTD1	protein_coding	Yes	NM_178123.5	108
CALM2P2	essed_pseudog	Yes	-	108
CLDN14-AS1	lncRNA	Yes	-	108
EZH1	protein_coding	Yes	NM_001991.5	108
C4A	protein_coding	Yes	NM_007293.3	108
PPP1R2P1	d_processed_ps	Yes	-	108
-	lncRNA	Yes	-	108
RPSAP8	essed_pseudog	Yes	-	108
-	lncRNA	Yes	-	108
KBTBD4	protein_coding	Yes	NM_018095.6	108
PLEKHM1	protein_coding	Yes	NM_014798.3	108
-	lncRNA	Yes	-	108
SLC2A14	protein_coding	Yes	NM_001286234.2	108
PGAP6	protein_coding	Yes	NM_021259.3	108
ITPRID2	protein_coding	Yes	NM_001130445.3	108
-	lncRNA	Yes	-	108
-	lncRNA	Yes	-	108

ENST00000432176	1290.2428	-1.359838734	0.181257	-7.502273	6.27E-14	9.89E-12	ENSG00000122591	7	22934210	23014130
ENST00000432264	80.258406	-1.809663355	0.392217	-4.613934	3.95E-06	8.83E-05	ENSG00000177873	3	40477121	40491053
ENST00000432294	132.83836	-2.961011371	0.989257	-2.993166	0.002760998	0.018912054	ENSG00000241073	1	100057989	100084471
ENST00000432377	4.6174835	5.632298037	1.975236	2.851455	0.004351962	0.026886003	ENSG00000225611	3	42770599	42773785
ENST00000432564	245.47001	-3.490434209	0.29482	-11.83919	2.45E-32	3.42E-29	ENSG00000196917	12	122726075	122730844
ENST00000432829	307.62772	1.055832678	0.235399	4.485285	7.28E-06	0.000148647	ENSG00000107099	9	214864	465255
ENST00000432994	12.822849	7.106530081	1.661174	4.278016	1.89E-05	0.000332712	ENSG00000236908	12	3318717	3325343
ENST00000433050	4.8997129	5.716560879	1.947521	2.935301	0.00333224	0.021846691	ENSG00000198633	19	52429147	52442499
ENST00000433060	1568.9353	-1.358368272	0.185679	-7.315666	2.56E-13	3.63E-11	ENSG00000166025	11	94768355	94876748
ENST00000433976	167.81277	1.416536077	0.288852	4.904015	9.39E-07	2.56E-05	ENSG00000170100	16	89217702	89237141
ENST00000434687	729.2714	-1.435151085	0.259946	-5.520949	3.37E-08	1.43E-06	ENSG00000030419	2	212999697	213151612
ENST00000434898	54.181282	1.217061449	0.465883	2.612377	0.008991501	0.046869034	ENSG00000235944	7	5823206	5847743
ENST00000435033	41.984213	1.6610713	0.515264	3.223728	0.001265335	0.010242777	ENSG00000268744	19	12379188	12401274
ENST00000435363	39.554211	1.431122035	0.537295	2.663568	0.007731669	0.041929629	ENSG00000224389	6	32014794	32035418
ENST00000435735	7.3413642	3.754891683	1.446394	2.596036	0.00943062	0.048629485	ENSG00000233217	1	200917459	200966536
ENST00000435919	4.9336504	5.73237924	2.040816	2.808867	0.004971625	0.029868355	ENSG00000231700	1	157709085	157709643
ENST00000435944	10.826265	3.266126238	1.125637	2.901579	0.00371287	0.02379556	ENSG00000177640	10	118046823	118210153
ENST00000435962	236.87689	-2.074482973	0.369016	-5.621661	1.89E-08	8.54E-07	ENSG00000165929	14	91779745	91867536
ENST00000436346	181.00168	-1.493171416	0.324572	-4.600427	4.22E-06	9.35E-05	ENSG00000115355	2	55287841	55419856
ENST00000436367	1113.9856	1.028468882	0.19054	5.397665	6.75E-08	2.67E-06	ENSG00000124788	6	16299111	16761460
ENST00000436636	11.059683	-5.455827607	1.710639	-3.189351	0.001425926	0.011251132	ENSG00000154310	3	171058413	171460405
ENST00000436786	6.3539059	6.097046892	1.93088	3.157652	0.001590454	0.012261756	ENSG00000234840	9	22646199	22824213
ENST00000436885	10.779368	6.854070827	1.692828	4.048887	5.15E-05	0.000772742	ENSG00000228251	2	112590795	112591939
ENST00000437139	278.50958	-1.098909423	0.244406	-4.496243	6.92E-06	0.000142414	ENSG00000179029	17	8172456	8176380
ENST00000437212	142.67725	-1.068459922	0.303362	-3.522066	0.000428198	0.004364483	ENSG00000137648	11	118077077	118121890
ENST00000437667	4.5429959	5.611391132	2.004459	2.799454	0.005118912	0.030564074	ENSG00000229814	9	71589600	71589933
ENST00000438793	960.28638	-1.099767072	0.200496	-5.485226	4.13E-08	1.72E-06	ENSG00000145730	5	102755304	103029716
ENST00000438980	270.68795	1.109574222	0.24625	4.505882	6.61E-06	0.000137118	ENSG00000173264	11	64285847	64289494
ENST00000439099	5.6065343	5.916880357	2.044375	2.894225	0.003800954	0.024235755	ENSG00000227383	9	34191554	34191881
ENST00000439140	14.841066	3.435086958	0.995593	3.450294	0.000559976	0.005391441	ENSG00000155754	2	201487420	201619178
ENST00000439298	32.248062	1.828966821	0.590449	3.097586	0.001951037	0.014390913	ENSG00000225032	9	127816065	127822520
ENST00000439741	132.65695	-1.158016341	0.335293	-3.453747	0.000552856	0.005333673	ENSG00000014914	1	149928650	149936879
ENST00000439742	112.97838	-1.375927066	0.334652	-4.111515	3.93E-05	0.000616405	ENSG00000213347	5	177307209	177311898
ENST00000440460	52.721145	1.819309427	0.462683	3.932083	8.42E-05	0.001156616	ENSG00000150764	11	111937342	112022653

FAM126A	protein_coding	Yes	NM_032581.4	108
ZNF619	protein_coding	Yes	NM_001145093.4	108
-	lncRNA	Yes	-	108
CCDC13-AS2	lncRNA	Yes	-	108
HCAR1	protein_coding	Yes	NM_032554.4	108
DOCK8	protein_coding	Yes	NM_203447.4	108
LINC02827	lncRNA	Yes	-	108
ZNF534	protein_coding	Yes	NM_001143938.3	108
AMOTL1	protein_coding	Yes	NM_130847.3	108
ZNF778	protein_coding	Yes	NM_001201407.2	108
IKZF2	protein_coding	Yes	NM_001387220.1	108
ZNF815P	_unprocessed_p	Yes	-	108
-	lncRNA	Yes	-	108
C4B	protein_coding	Yes	NM_001002029.4	108
MROH3P	ed_unitary_pse	Yes	-	108
-	essed_pseudog	Yes	-	108
CASC2	lncRNA	Yes	-	108
TC2N	protein_coding	Yes	NM_001128596.3	108
CCDC88A	protein_coding	Yes	NM_001365480.1	108
ATXN1	protein_coding	Yes	NM_001128164.2	108
TNIK	protein_coding	Yes	NM_015028.4	108
LINC01239	lncRNA	Yes	-	108
-	lncRNA	Yes	-	108
TMEM107	protein_coding	Yes	NM_183065.4	108
TMPRSS4	protein_coding	Yes	NM_019894.4	108
RPL35AP21	essed_pseudog	Yes	-	108
PAM	protein_coding	Yes	NM_001177306.2	108
GPR137	protein_coding	Yes	NM_001170880.2	108
RPL35AP2	essed_pseudog	Yes	-	108
C2CD6	protein_coding	Yes	NM_001168221.2	108
-	lncRNA	Yes	-	108
MTMR11	protein_coding	Yes	NM_001145862.2	108
MXD3	protein_coding	Yes	NM_031300.4	108
DIXDC1	protein_coding	Yes	NM_001037954.4	108

ENST00000440944	82.242624	-3.583353839	0.474726	-7.548262	4.41E-14	7.19E-12	ENSG00000160360	9	136327538	136359601
ENST00000441178	47.820943	-1.539915642	0.481121	-3.200685	0.001371013	0.010900839	ENSG00000235376	10	103245886	103248016
ENST00000441322	35.970356	-1.674757165	0.545871	-3.068048	0.002154622	0.015561206	ENSG00000237289	15	43594026	43599406
ENST00000441545	80.836162	1.154331506	0.376629	3.0649	0.002177428	0.015687441	ENSG00000137070	9	34652184	34661902
ENST00000441788	303.33836	-1.140981008	0.246245	-4.633521	3.59E-06	8.15E-05	ENSG00000105325	19	3506310	3538334
ENST00000442218	85.166871	-1.396443078	0.369171	-3.782647	0.000155169	0.001921786	ENSG00000211584	12	47773237	47782751
ENST00000442241	177.09861	-1.732004358	0.310084	-5.585597	2.33E-08	1.03E-06	ENSG00000073536	17	35128729	35142303
ENST00000442253	58.43356	-1.883048911	0.44981	-4.186322	2.84E-05	0.00046739	ENSG00000170915	6	52362150	52407777
ENST00000442305	6.0873974	6.030698599	1.860391	3.24163	0.001188481	0.009740153	ENSG00000229393	1	2493436	2494479
ENST00000442673	28.222669	2.290641466	0.641962	3.568187	0.00035946	0.00379087	ENSG00000236423	1	3900402	3917225
ENST00000442697	1517.8092	-1.353685869	0.215346	-6.286096	3.26E-10	2.42E-08	ENSG00000164031	4	99896247	99946618
ENST00000443279	7.3247402	6.297202409	1.796564	3.505137	0.000456372	0.004584703	ENSG00000213338	X	154622427	154623500
ENST00000443439	1113.0863	1.040137447	0.222276	4.679489	2.88E-06	6.71E-05	ENSG00000164307	5	96774483	96807945
ENST00000443585	593.30018	-1.251384183	0.255641	-4.895093	9.83E-07	2.66E-05	ENSG00000235162	12	105330690	105371518
ENST00000443786	309.35685	-1.00866666	0.23487	-4.294573	1.75E-05	0.000312884	ENSG00000214367	4	2228348	2242121
ENST00000443935	24.883909	2.583514875	0.709323	3.642224	0.000270293	0.003023972	ENSG00000236754	22	17580156	17589192
ENST00000444037	8.5923555	3.982725077	1.41631	2.812043	0.004922788	0.029655612	ENSG00000240219	1	204626774	204629712
ENST00000444158	3.9197703	5.394631232	2.054307	2.626011	0.008639213	0.045513691	ENSG00000234210	7	157466230	157499716
ENST00000444623	66.131255	2.06157968	0.500603	4.118192	3.82E-05	0.000601232	ENSG00000139631	12	53157662	53180925
ENST00000444722	3.9197703	5.394631232	2.054307	2.626011	0.008639213	0.045513691	ENSG00000224216	X	155334857	155351926
ENST00000445000	20.111358	2.639497193	0.768736	3.433554	0.000595723	0.005647477	ENSG00000244041	6	2987966	2991173
ENST00000445118	88.667244	1.32228964	0.365478	3.617973	0.00029692	0.003264363	ENSG00000228794	1	827597	859446
ENST00000445224	270.07317	-1.214367115	0.247394	-4.908632	9.17E-07	2.51E-05	ENSG00000151967	3	159839860	159897360
ENST00000445323	316.06377	-2.116120958	0.243816	-8.679159	3.99E-18	1.16E-15	ENSG00000063601	X	150693395	150765108
ENST00000445498	6.1370558	6.041079267	1.857764	3.251801	0.001146762	0.009476202	ENSG00000225778	10	11849607	11894700
ENST00000446176	279.42794	-1.316937934	0.251255	-5.241433	1.59E-07	5.57E-06	ENSG00000187239	9	129887186	130043189
ENST00000446378	71.072968	1.131620916	0.403063	2.807553	0.004991944	0.029967985	ENSG00000164309	5	79689835	79800222
ENST00000446477	7.8454031	-6.445625875	1.776181	-3.628923	0.000284606	0.003153546	ENSG00000224940	7	128350325	128361685
ENST00000446969	11.967053	7.005375847	1.669931	4.195008	2.73E-05	0.000451872	ENSG00000235959	2	95640180	95640604
ENST00000447146	593.97278	1.06846107	0.214903	4.971818	6.63E-07	1.90E-05	ENSG00000102103	X	48897929	48903143
ENST00000447166	20.786536	7.801744701	1.580507	4.93623	7.96E-07	2.23E-05	ENSG00000221843	2	27537385	27582722
ENST00000447535	24.394729	-4.258527838	0.898699	-4.738549	2.15E-06	5.27E-05	ENSG00000233056	21	42916802	42925630
ENST00000448097	131.2582	1.965559743	0.403395	4.872545	1.10E-06	2.93E-05	ENSG00000132510	17	7834216	7854796
ENST00000448387	55.603002	1.553008858	0.57705	2.691289	0.007117645	0.039374522	ENSG00000232653	15	32593455	32607310

GPSM1	protein_coding	Yes	NM_001145638.3	108
RPEL1	protein_coding	Yes	NM_001143909.1	108
CKMT1B	protein_coding	Yes	NM_001375484.1	108
IL11RA	protein_coding	Yes	NM_001142784.3	108
FZR1	protein_coding	Yes	NM_016263.4	108
SLC48A1	protein_coding	Yes	NM_017842.3	108
NLE1	protein_coding	Yes	NM_018096.5	108
PAQR8	protein_coding	Yes	NM_133367.5	108
-	lncRNA	Yes	-	108
LINC01134	lncRNA	Yes	-	108
DNAJB14	protein_coding	Yes	NM_001031723.4	108
ATF4P1	essed_pseudog	Yes	-	108
ERAP1	protein_coding	Yes	NM_001040458.3	108
C12orf75	protein_coding	Yes	NM_001145199.2	108
HAUS3	protein_coding	Yes	NM_001303143.2	108
-	lncRNA	Yes	-	108
-	lncRNA	Yes	-	108
-	lncRNA	Yes	-	108
CSAD	protein_coding	Yes	NM_001244705.2	108
-	lncRNA	Yes	-	108
LINC01011	lncRNA	Yes	-	108
LINC01128	lncRNA	Yes	-	108
SCHIP1	protein_coding	Yes	-	108
MTMR1	protein_coding	Yes	NM_001306144.3	108
PROSER2-AS1	lncRNA	Yes	-	108
FNBP1	protein_coding	Yes	NM_015033.3	108
CMYA5	protein_coding	Yes	NM_153610.5	108
PRRT4	protein_coding	Yes	-	108
-	essed_pseudog	Yes	-	108
PQBP1	protein_coding	Yes	NM_001032382.2	108
C2orf16	protein_coding	Yes	NM_032266.5	108
ERVH48-1	protein_coding	Yes	NM_001308491.2	108
KDM6B	protein_coding	Yes	NM_001348716.2	108
GOLGA8N	protein_coding	Yes	-	108

ENST00000449111	28.648096	1.741248636	0.609568	2.85653	0.004282989	0.026563627	ENSG00000235703	X	149938627	150224580
ENST00000449339	6.8595981	6.201990018	1.818157	3.411141	0.000646916	0.006038901	ENSG00000234509	21	31653592	31659500
ENST00000449428	596.20394	-2.059003857	0.244522	-8.420542	3.75E-17	9.66E-15	ENSG00000182534	17	76679565	76710965
ENST00000449496	244.93175	-1.180785638	0.280566	-4.20858	2.57E-05	0.000430166	ENSG00000214012	6	19612754	19614036
ENST00000450114	957.74633	-1.14394849	0.188377	-6.072655	1.26E-09	7.99E-08	ENSG00000166483	11	9573669	9589985
ENST00000450253	748.12738	-1.315086532	0.215831	-6.093128	1.11E-09	7.13E-08	ENSG00000151247	4	98879275	98929133
ENST00000450708	234.36345	-1.01037065	0.25328	-3.98915	6.63E-05	0.000949763	ENSG00000133895	11	64803515	64810551
ENST00000450892	16.561699	2.457865813	0.847461	2.900271	0.003728398	0.023872598	ENSG00000242866	15	43599562	43618800
ENST00000451775	24.801075	2.260107136	0.68146	3.316564	0.000911318	0.007906579	ENSG00000224738	17	59106597	59118453
ENST00000451794	56.816138	2.540541625	0.475042	5.348039	8.89E-08	3.38E-06	ENSG00000163121	2	96497645	96505357
ENST00000452015	34.84137	1.493720761	0.559521	2.669641	0.007593247	0.041340798	ENSG00000235194	14	23295651	23302859
ENST00000452097	10.447481	6.810309626	1.701378	4.002819	6.26E-05	0.00090441	ENSG00000237017	19	54119510	54125343
ENST00000452361	482.53706	1.180370149	0.224663	5.253962	1.49E-07	5.26E-06	ENSG00000274049	2	74455087	74460884
ENST00000453097	81.326344	2.299247495	0.410816	5.596782	2.18E-08	9.73E-07	ENSG00000050438	12	51424830	51515763
ENST00000453115	340.28857	-1.307378821	0.231928	-5.637006	1.73E-08	7.91E-07	ENSG00000233830	7	27458162	27458849
ENST00000453269	224.84681	-1.046792586	0.257107	-4.071434	4.67E-05	0.000712935	ENSG00000106290	7	100107069	100119358
ENST00000453347	5.4145134	5.85994416	1.907483	3.072083	0.002125708	0.015391333	ENSG00000228420	1	109828354	109871436
ENST00000453424	204.85708	1.687333904	0.353293	4.776016	1.79E-06	4.47E-05	ENSG00000165406	10	45454584	45535371
ENST00000453634	681.66292	1.400330928	0.209707	6.677568	2.43E-11	2.33E-09	ENSG00000254413	22	50568994	50579752
ENST00000453848	333.62108	-1.933971358	0.249182	-7.761293	8.41E-15	1.60E-12	ENSG00000006118	11	60924459	60937159
ENST00000453895	6.0625682	6.025472468	1.864132	3.232321	0.001227891	0.009998619	ENSG00000240710	1	204603034	204616565
ENST00000453996	59.633226	-1.749455111	0.438508	-3.98956	6.62E-05	0.000948659	ENSG00000174177	16	88706502	88715396
ENST00000454078	139.36641	-1.041027007	0.315583	-3.298741	0.000971194	0.008297587	ENSG00000232389	6	70608233	70609334
ENST00000454158	242.83236	-1.775637409	0.258694	-6.863852	6.70E-12	7.18E-10	ENSG00000181826	4	37610633	37686376
ENST00000454584	56.184954	1.770009024	0.473267	3.739979	0.000184036	0.002209107	ENSG00000148935	11	22666610	22813001
ENST00000455537	7.8983073	6.409057007	1.795406	3.569699	0.000357392	0.003772456	ENSG00000155980	12	57550043	57586633
ENST00000455843	5.3397235	-5.889746233	1.915202	-3.075261	0.002103184	0.015270756	ENSG00000284874	22	19717219	19724216
ENST00000455965	38.249002	2.313032968	0.626677	3.690949	0.000223419	0.002586908	ENSG00000235092	2	8666662	8681864
ENST00000456555	4.8997129	5.716560879	1.947521	2.935301	0.00333224	0.021846691	ENSG00000233487	3	196986675	196987558
ENST00000456632	176.34568	1.110847905	0.283722	3.91527	9.03E-05	0.001222742	ENSG00000234322	X	93287732	93288759
ENST00000456687	4.6423127	5.639148354	1.971416	2.860456	0.004230322	0.026324826	ENSG00000225931	1	2566409	2569888
ENST00000456718	121.88998	1.190178741	0.341863	3.481454	0.000498699	0.004917017	ENSG00000123870	19	52592037	52597345
ENST00000456790	71.585503	1.887860787	0.412125	4.580796	4.63E-06	0.000101332	ENSG00000126005	20	35276351	35278122
ENST00000456829	23.481882	3.204779842	0.788616	4.063803	4.83E-05	0.000732101	ENSG00000110002	11	124115449	124147721

EOLA2-DT	lncRNA	Yes	-	108
SOD1-DT	lncRNA	Yes	-	108
MXRA7	protein_coding	Yes	NM_198530.4	108
KRT18P38	essed_pseudog	Yes	-	108
WEE1	protein_coding	Yes	NM_003390.4	108
EIF4E	protein_coding	Yes	NM_001968.5	108
MEN1	protein_coding	Yes	NM_001370259.2	108
STRC	protein_coding	Yes	NM_153700.2	108
-	lncRNA	Yes	-	108
NEURL3	protein_coding	Yes	NM_001285485.2	108
PPP1R3E	protein_coding	Yes	NM_001276318.2	108
PRPF31-AS1	lncRNA	Yes	-	108
INO80B-WBP1	protein_coding	Yes	-	108
SLC4A8	protein_coding	Yes	NM_001039960.3	108
EIF4HP1	essed_pseudog	Yes	-	108
TAF6	protein_coding	Yes	NM_139315.3	108
LINC01768	lncRNA	Yes	-	108
MARCHF8	protein_coding	Yes	NM_001282866.2	108
CHKB-CPT1B	protein_coding	Yes	-	108
TMEM132A	protein_coding	Yes	NM_178031.3	108
-	lncRNA	Yes	-	108
CTU2	protein_coding	Yes	NM_001012759.3	108
-	essed_pseudog	Yes	-	108
RELL1	protein_coding	Yes	NM_001085400.2	108
GAS2	protein_coding	Yes	NM_001143830.3	108
KIF5A	protein_coding	Yes	NM_004984.4	108
-	protein_coding	Yes	-	108
ID2-AS1	lncRNA	Yes	-	108
RPSAP69	d_processed_ps	Yes	-	108
ST13P18	essed_pseudog	Yes	-	108
-	TEC	Yes	-	108
ZNF137P	_unprocessed_p	Yes	-	108
MMP24OS	protein_coding	Yes	NM_001355003.2	108
VWA5A	protein_coding	Yes	NM_001130142.2	108

ENST00000456849	7.5989296	-6.399426641	1.785938	-3.583231	0.00033937	0.003617743	ENSG00000198682	10	87659877	87747705
ENST00000456936	2365.3763	-1.022648216	0.234564	-4.359789	1.30E-05	0.000243247	ENSG00000134970	5	115613209	115626036
ENST00000457511	17.299127	4.457030394	1.099233	4.054673	5.02E-05	0.000756473	ENSG00000041515	13	108629610	109208005
ENST00000457653	65.323023	1.290608221	0.412704	3.127197	0.001764814	0.013303663	ENSG00000161912	6	41101021	41138727
ENST00000457982	38.527412	1.844143975	0.54431	3.388042	0.000703936	0.006449118	ENSG00000186301	1	16645621	16650289
ENST00000458108	332.48057	-1.215518375	0.264839	-4.589644	4.44E-06	9.77E-05	ENSG00000234737	3	32258939	32260230
ENST00000458198	260.02133	-2.32571168	0.266749	-8.71872	2.81E-18	8.58E-16	ENSG00000237190	5	134402064	134411881
ENST00000459742	6.5525395	6.137473412	1.835772	3.343266	0.000827985	0.007328343	ENSG00000259628	7	77043720	77198626
ENST00000461366	11.723555	5.505054685	1.659941	3.316416	0.000911801	0.00790942	ENSG00000128482	17	19411231	19417276
ENST00000461771	7.9976241	6.425211053	1.773124	3.623668	0.000290455	0.003207862	ENSG00000243352	7	104911927	104912193
ENST00000462248	25.943895	2.238899241	0.67621	3.31095	0.000929797	0.008028969	ENSG00000241170	11	74919586	74919965
ENST00000462792	52.565389	1.877140495	0.526538	3.565064	0.000363767	0.003826338	ENSG00000239572	3	87641411	87793629
ENST00000463745	216.80328	-1.064184315	0.268585	-3.962187	7.43E-05	0.001042956	ENSG00000113966	3	97764757	97801229
ENST00000464755	42.8937	1.789413688	0.506459	3.533183	0.000410587	0.004219526	ENSG00000276490	10	94688153	94853073
ENST00000464848	145.627	-2.670946801	0.331711	-8.052039	8.14E-16	1.78E-13	ENSG00000105948	7	139133777	139191986
ENST00000465139	1598.6918	-1.419338547	0.189388	-7.494331	6.66E-14	1.05E-11	ENSG00000140350	15	68778534	68820895
ENST00000465784	419.56036	-1.088044214	0.216843	-5.017657	5.23E-07	1.55E-05	ENSG00000095383	9	98199010	98255649
ENST00000466070	5.3896842	5.85406504	1.907255	3.069367	0.002145128	0.015504393	ENSG00000239622	7	25689579	25689959
ENST00000467148	717.63023	1.296696282	0.257049	5.044542	4.55E-07	1.37E-05	ENSG00000130589	20	63558085	63574239
ENST00000467460	7.8147115	6.390582615	1.776621	3.597043	0.000321856	0.003474102	ENSG00000114757	3	179794957	180036937
ENST00000470205	66.631555	-1.466120032	0.417889	-3.508395	0.000450819	0.004542892	ENSG00000212829	9	9090897	9091245
ENST00000470487	502.54065	-1.507037433	0.241885	-6.230382	4.65E-10	3.28E-08	ENSG00000173905	3	168008688	168095924
ENST00000471229	178.9438	-5.680085595	0.471126	-12.05641	1.79E-33	2.76E-30	ENSG00000102401	X	101623150	101627843
ENST00000471502	142.67597	-1.272081792	0.30168	-4.216659	2.48E-05	0.000417293	ENSG00000272679	9	136800365	136829466
ENST00000472297	203.88087	1.113699061	0.272172	4.091895	4.28E-05	0.000662263	ENSG00000233836	19	23790771	23833314
ENST00000474759	24.386608	2.104384746	0.713591	2.949008	0.003187957	0.021123569	ENSG00000157445	3	54122551	55074557
ENST00000474844	234.23326	-1.305719055	0.25485	-5.123479	3.00E-07	9.58E-06	ENSG00000117481	1	46340806	46365018
ENST00000474889	6.8322124	-6.246152562	1.82005	-3.431858	0.000599461	0.00567693	ENSG00000144724	3	61561570	62297609
ENST00000474905	6.4035643	6.107577541	1.889944	3.231619	0.00123091	0.010015177	ENSG00000158525	7	130344815	130368730
ENST00000475156	205.86859	-1.22732231	0.27992	-4.384543	1.16E-05	0.000220936	ENSG00000212994	8	100895770	100896118
ENST00000475381	791.85765	-1.013600728	0.191467	-5.293879	1.20E-07	4.34E-06	ENSG00000144959	3	172630248	172711067
ENST00000476320	73.99999	1.392055485	0.403738	3.447914	0.000564934	0.005428542	ENSG00000186577	6	34246394	34249006
ENST00000477922	7.8147115	6.390582615	1.776621	3.597043	0.000321856	0.003474102	ENSG00000257743	7	142111717	142222324
ENST00000478745	4.6671419	5.645964836	1.970163	2.865735	0.004160419	0.026003428	ENSG00000244345	3	72504805	72550552

PAPSS2	protein_coding	Yes	NM_001015880.2	108
TMED7	protein_coding	Yes	NM_181836.6	108
MYO16	protein_coding	Yes	NM_001198950.3	108
ADCY10P1	_unprocessed_p	Yes	-	108
MST1P2	cessed_pseudo	Yes	-	108
KRT18P15	essed_pseudog	Yes	-	108
CDKN2AIPNL	protein_coding	Yes	NM_080656.3	108
-	lncRNA	Yes	-	108
RNF112	protein_coding	Yes	NM_007148.5	108
RN7SL8P	misc_RNA	Yes	-	108
RPL31P46	d_processed_ps	Yes	-	108
-	lncRNA	Yes	-	108
ARL6	protein_coding	Yes	NM_001278293.3	108
-	protein_coding	Yes	-	108
TTC26	protein_coding	Yes	NM_024926.4	108
ANP32A	protein_coding	Yes	NM_006305.4	108
TBC1D2	protein_coding	Yes	NM_001267571.2	108
-	essed_pseudog	Yes	-	108
HELZ2	protein_coding	Yes	NM_001037335.2	108
PEX5L	protein_coding	Yes	NM_016559.3	108
RPS26P3	essed_pseudog	Yes	-	108
GOLIM4	protein_coding	Yes	NM_014498.5	108
ARMCX3	protein_coding	Yes	NM_177947.3	108
-	lncRNA	Yes	-	108
-	_unprocessed_p	Yes	-	108
CACNA2D3	protein_coding	Yes	NM_018398.3	108
NSUN4	protein_coding	Yes	NM_199044.4	108
PTPRG	protein_coding	Yes	NM_002841.4	108
CPA5	protein_coding	Yes	NM_080385.5	108
RPS26P6	essed_pseudog	Yes	-	108
NCEH1	protein_coding	Yes	NM_020792.6	108
SMIM29	protein_coding	Yes	NM_001008703.4	108
MGAM2	protein_coding	Yes	NM_001293626.2	108
-	lncRNA	Yes	-	108

ENST00000480456	4.9742005	5.735936539	1.955082	2.933859	0.003347758	0.021910732	ENSG00000154721	21	25639257	25717562
ENST00000481490	375.12244	-1.457851184	0.240962	-6.050129	1.45E-09	9.01E-08	ENSG00000265817	8	94427715	94436944
ENST00000481510	4.6762501	5.655334035	2.10906	2.681448	0.007330432	0.040233297	ENSG00000290594	2	131758030	131767107
ENST00000481698	30.206795	1.764793762	0.61122	2.887332	0.003885241	0.024657652	ENSG00000241527	22	19031563	19034564
ENST00000481739	1092.9338	-1.101730833	0.183085	-6.017587	1.77E-09	1.07E-07	ENSG00000186350	9	134326454	134440585
ENST00000482477	44.138464	-1.874220146	0.529566	-3.53916	0.000401403	0.004144869	ENSG00000232320	2	161340815	161341326
ENST00000484259	115.42246	-1.187377393	0.340399	-3.488195	0.000486294	0.004823727	ENSG00000165060	9	69035751	69079076
ENST00000485303	74.595428	-6.610659044	1.569332	-4.212404	2.53E-05	0.000424191	ENSG00000177707	3	111071815	111137560
ENST00000485827	731.60917	-1.146378641	0.212913	-5.384248	7.27E-08	2.84E-06	ENSG00000196656	4	113214045	113214393
ENST00000486874	5.4145134	5.85994416	1.907483	3.072083	0.002125708	0.015391333	ENSG00000240270	17	80976453	80976948
ENST00000487418	24.056933	1.924282711	0.699963	2.74912	0.005975556	0.034371458	ENSG00000128928	15	40405794	40421308
ENST00000488034	633.93057	1.144689579	0.220722	5.186121	2.15E-07	7.18E-06	ENSG00000112514	6	33416441	33418107
ENST00000488300	164.24498	-1.405856092	0.322005	-4.365948	1.27E-05	0.00023737	ENSG00000180537	6	13924951	13980310
ENST00000488788	2679.643	1.151387838	0.210934	5.458525	4.80E-08	1.98E-06	ENSG00000270299	20	646625	675800
ENST00000489418	1483.7049	-1.044072738	0.205676	-5.076308	3.85E-07	1.19E-05	ENSG00000163950	4	1692730	1712319
ENST00000489612	99.590624	-1.14918939	0.389275	-2.952129	0.003155914	0.020977156	ENSG00000231167	7	105582257	105583256
ENST00000491143	17.315868	-2.027860322	0.781821	-2.593764	0.009493164	0.048862679	ENSG00000119547	18	57435373	57491298
ENST00000492267	62.664299	-2.058123312	0.442961	-4.646286	3.38E-06	7.72E-05	ENSG00000241095	3	82806514	82808021
ENST00000494359	4.6423127	5.639148354	1.971416	2.860456	0.004230322	0.026324826	ENSG00000197149	11	17228278	17229151
ENST00000494426	847.51541	-1.448199527	0.207004	-6.995995	2.63E-12	3.04E-10	ENSG00000169583	9	136994607	136996568
ENST00000494857	76.85603	-3.414303654	0.471349	-7.243688	4.37E-13	5.85E-11	ENSG00000003989	8	17497087	17570566
ENST00000495723	53.542629	1.328820926	0.459502	2.891872	0.003829537	0.024369074	ENSG00000258461	15	42348102	42412317
ENST00000495936	202.84461	-1.503640244	0.287113	-5.237103	1.63E-07	5.68E-06	ENSG00000160991	7	102433574	102456825
ENST00000497576	30.486169	3.46413622	0.697453	4.966837	6.81E-07	1.94E-05	ENSG00000224864	19	20125043	20125484
ENST00000498907	192.79661	-1.679256384	0.293894	-5.713821	1.10E-08	5.42E-07	ENSG00000245848	19	33299933	33302534
ENST00000500612	3.9197703	5.394631232	2.054307	2.626011	0.008639213	0.045513691	ENSG00000247324	16	71462277	71465941
ENST00000501931	3.8949411	5.386514595	2.058112	2.617211	0.008865141	0.04637373	ENSG00000246877	15	75727669	75738623
ENST00000502390	12.457024	7.063247405	1.661797	4.250368	2.13E-05	0.000367999	ENSG00000231683	6	53561288	53617007
ENST00000502773	197.4774	-3.187504125	0.302336	-10.54292	5.48E-26	4.69E-23	ENSG00000145431	4	156760453	156971799
ENST00000503057	115.75169	-2.954402571	0.367068	-8.048656	8.37E-16	1.83E-13	ENSG00000064042	4	41538156	41700044
ENST00000503176	204.59449	1.335983502	0.299624	4.458867	8.24E-06	0.000164627	ENSG00000005882	17	50095350	50112152
ENST00000503362	2672.0509	-1.185049993	0.190214	-6.23008	4.66E-10	3.28E-08	ENSG00000129128	4	176319965	176332245
ENST00000503395	4.8058312	-5.738655542	1.956982	-2.932401	0.003363524	0.021987476	ENSG00000290588	9	138150112	138176508
ENST00000503452	30.354694	2.909452902	0.683596	4.2561	2.08E-05	0.000360326	ENSG00000251141	5	44744899	44808777

JAM2	protein_coding	Yes	NM_021219.4	108
FSBP	protein_coding	Yes	NM_001256141.2	108
-	lncRNA	Yes	-	108
CA15P1	itary_pseudoge	Yes	-	108
RXRA	protein_coding	Yes	NM_002957.6	108
MXRA7P1	essed_pseudog	Yes	-	108
FXN	protein_coding	Yes	NM_000144.5	108
NECTIN3	protein_coding	Yes	NM_015480.3	108
-	d_processed_ps	Yes	-	108
RPL12P37	essed_pseudog	Yes	-	108
IVD	protein_coding	Yes	NM_002225.5	108
CUTA	protein_coding	Yes	NM_001014840.2	108
RNF182	protein_coding	Yes	NM_152737.4	108
-	protein_coding	Yes	-	108
SLBP	protein_coding	Yes	NM_006527.4	108
YBX1P2	essed_pseudog	Yes	-	108
ONECUT2	protein_coding	Yes	NM_004852.3	108
CYP51A1P1	essed_pseudog	Yes	-	108
-	essed_pseudog	Yes	-	108
CLIC3	protein_coding	Yes	NM_004669.3	108
SLC7A2	protein_coding	Yes	NM_001370338.1	108
-	protein_coding	Yes	-	108
ORAI2	protein_coding	Yes	NM_001126340.3	108
-	d_processed_ps	Yes	-	108
CEBPA	protein_coding	Yes	NM_004364.5	108
-	lncRNA	Yes	-	108
DNM1P35	lncRNA	Yes	-	108
-	lncRNA	Yes	-	108
PDGFC	protein_coding	Yes	NM_016205.3	108
LIMCH1	protein_coding	Yes	NM_001330672.2	108
PDK2	protein_coding	Yes	NM_002611.5	108
SPCS3	protein_coding	Yes	NM_021928.4	108
-	lncRNA	Yes	-	108
MRPS30-DT	lncRNA	Yes	-	108

ENST00000503488	5.1074548	5.777669537	1.930343	2.993079	0.00276178	0.018912054	ENSG00000249359	5	21323872	21341375
ENST00000503731	39.319032	-7.31620665	1.51644	-4.824595	1.40E-06	3.62E-05	ENSG00000104723	8	15540262	15766638
ENST00000504120	457.31808	1.212462329	0.229185	5.29031	1.22E-07	4.42E-06	ENSG00000204970	5	140786139	141012347
ENST00000504610	12.509364	2.839211925	1.017705	2.789819	0.00527375	0.031240025	ENSG00000250303	11	112270748	112362534
ENST00000504771	526.32249	-1.158017524	0.205975	-5.622129	1.89E-08	8.52E-07	ENSG00000145779	5	119356018	119399688
ENST00000504986	1393.4677	-1.035777763	0.180764	-5.730002	1.00E-08	4.99E-07	ENSG00000109133	4	41935428	41960803
ENST00000507003	31.411905	1.85266885	0.590798	3.135876	0.001713414	0.012998661	ENSG00000248406	19	12379745	12383687
ENST00000507248	7.6620713	3.803844133	1.459365	2.606507	0.009147094	0.047501373	ENSG00000234492	4	108538189	108620413
ENST00000507279	30.347565	1.638198253	0.600709	2.727108	0.006389205	0.036278414	ENSG00000283155	5	140562442	140592123
ENST00000507444	4.9088212	5.725307972	2.090756	2.738391	0.006174069	0.035297498	ENSG00000250490	5	6310440	6337292
ENST00000508195	9.3930504	6.658190035	1.736301	3.834698	0.000125719	0.00161314	ENSG00000250130	15	44354690	44355692
ENST00000508859	18.350502	2.47087426	0.816461	3.026324	0.002475469	0.017363375	ENSG00000206077	5	710354	784729
ENST00000509176	100.08752	-1.491759997	0.353695	-4.217641	2.47E-05	0.00041571	ENSG00000184305	4	90127393	91605295
ENST00000509437	451.27467	-1.055264117	0.257529	-4.097656	4.17E-05	0.000648018	ENSG00000155329	5	132996984	133026549
ENST00000509479	16.831555	-3.385720508	0.946336	-3.577717	0.000346609	0.003678248	ENSG00000196782	4	139716752	140154184
ENST00000509685	31.740083	-2.088964272	0.61052	-3.421616	0.000622501	0.005857262	ENSG00000250299	13	52617557	52637446
ENST00000509834	22.071711	1.949954278	0.700677	2.782958	0.005386578	0.031735842	ENSG00000251432	4	128292932	128474996
ENST00000510898	29.939665	1.829454071	0.623905	2.932264	0.003365009	0.021992274	ENSG00000146094	5	177501903	177509826
ENST00000511437	205.40286	-1.418552462	0.267132	-5.310301	1.09E-07	4.02E-06	ENSG00000150756	5	10225506	10249888
ENST00000511689	1797.5085	-1.023807368	0.175351	-5.838624	5.26E-09	2.82E-07	ENSG00000113648	5	135334380	135399231
ENST00000511893	87.052795	1.422105739	0.386673	3.677804	0.000235251	0.002698552	ENSG00000251660	7	30157530	30159534
ENST00000511912	444.09773	1.249564525	0.248814	5.022082	5.11E-07	1.52E-05	ENSG00000171503	4	158672295	158709623
ENST00000512123	719.75586	1.342055185	0.200975	6.677739	2.43E-11	2.33E-09	ENSG00000138759	4	78057322	78544269
ENST00000512194	235.57286	1.079682611	0.299849	3.600759	0.00031729	0.003438946	ENSG00000287542	4	88523809	88708450
ENST00000512467	18.150022	2.29171229	0.845419	2.710742	0.006713294	0.037716744	ENSG00000183775	5	144170872	144485686
ENST00000512551	106.88941	1.793569778	0.36932	4.856417	1.20E-06	3.15E-05	ENSG00000288559	10	73247341	73248268
ENST00000513185	80.172898	-1.380291647	0.378442	-3.647299	0.000265012	0.002977739	ENSG00000174136	5	98773662	98796494
ENST00000513610	800.55453	-1.289780425	0.192387	-6.704097	2.03E-11	1.98E-09	ENSG00000145555	5	16661906	16936288
ENST00000513638	6.8046616	-4.755075659	1.79324	-2.651667	0.008009557	0.043076487	ENSG00000290525	1	89407678	89424934
ENST00000513986	140.26581	1.766111557	0.357603	4.938747	7.86E-07	2.20E-05	ENSG00000168916	5	124636914	124746639
ENST00000514254	64.070141	1.417678766	0.440685	3.216992	0.001295421	0.010433672	ENSG00000250091	12	123925460	123934984
ENST00000515279	412.12417	-1.566572442	0.222217	-7.049735	1.79E-12	2.14E-10	ENSG00000138780	4	105708783	105847725
ENST00000515393	17.648437	-5.148580437	1.239432	-4.153983	3.27E-05	0.000527079	ENSG00000175471	5	94703689	95285094
ENST00000515425	442.23225	-1.587548633	0.224778	-7.06275	1.63E-12	1.97E-10	ENSG00000169247	5	148982149	149063062

-	lncRNA	Yes	-	108
TUSC3	protein_coding	Yes	NM_006765.4	108
PCDHA1	protein_coding	Yes	NM_018900.4	108
LINC02762	lncRNA	Yes	-	108
TNFAIP8	protein_coding	Yes	NM_014350.4	108
TMEM33	protein_coding	Yes	NM_018126.3	108
-	_unprocessed_p	Yes	-	108
RPL34-DT	lncRNA	Yes	-	108
-	lncRNA	Yes	-	108
LINC02145	lncRNA	Yes	-	108
GAPDHP43	essed_pseudog	Yes	-	108
ZDHHC11B	protein_coding	Yes	NM_001351303.2	108
CCSER1	protein_coding	Yes	NM_001145065.2	108
ZCCHC10	protein_coding	Yes	NM_001300816.3	108
MAML3	protein_coding	Yes	NM_018717.5	108
MRPS31P4	_unprocessed_p	Yes	-	108
LINC02615	lncRNA	Yes	-	108
DOK3	protein_coding	Yes	NM_001308236.3	108
ATPSCKMT	protein_coding	Yes	NM_199133.4	108
MACROH2A1	protein_coding	Yes	NM_138610.3	108
-	lncRNA	Yes	-	108
ETFDH	protein_coding	Yes	NM_004453.4	108
FRAS1	protein_coding	Yes	NM_025074.7	108
HERC3	protein_coding	Yes	-	108
KCTD16	protein_coding	Yes	NM_020768.4	108
-	lncRNA	Yes	-	108
RGMB	protein_coding	Yes	NM_001366508.1	108
MYO10	protein_coding	Yes	NM_012334.3	108
GBP1P1	lncRNA	Yes	-	108
ZNF608	protein_coding	Yes	NM_020747.3	108
DNAH10OS	lncRNA	Yes	-	108
GSTCD	protein_coding	Yes	NM_001370181.1	108
MCTP1	protein_coding	Yes	NM_024717.7	108
SH3TC2	protein_coding	Yes	NM_024577.4	108

ENST00000515712	49.290095	-1.274713667	0.464789	-2.742565	0.00609613	0.034945035	ENSG00000213763	5	77784880	77786003
ENST00000517417	65.031137	1.195204827	0.411609	2.903739	0.003687354	0.023663872	ENSG00000204956	5	141330513	141512975
ENST00000517434	76.906816	1.200817456	0.383827	3.128536	0.001756796	0.013255198	ENSG00000253731	5	141373890	141512975
ENST00000517746	87.677274	-1.286497626	0.369523	-3.481507	0.000498602	0.004916372	ENSG00000154188	8	107249481	107497918
ENST00000517833	60.437662	-1.530058799	0.469778	-3.256986	0.001126022	0.009335694	ENSG00000247317	8	142981737	143018447
ENST00000517854	4.9088212	5.725307972	2.090756	2.738391	0.006174069	0.035297498	ENSG00000254271	10	60734341	60741828
ENST00000517956	661.23549	1.836479686	0.226743	8.099401	5.52E-16	1.24E-13	ENSG00000156804	8	123497888	123541206
ENST00000518325	64.480367	1.314038171	0.414428	3.170726	0.001520585	0.01182659	ENSG00000253537	5	141382741	141512975
ENST00000518937	1487.579	-1.133499101	0.190195	-5.95966	2.53E-09	1.47E-07	ENSG00000076554	8	80034744	80171564
ENST00000519106	43.282637	-1.351975458	0.493879	-2.737463	0.006191508	0.035375965	ENSG00000253958	8	8701936	8704096
ENST00000519479	68.593247	1.22542273	0.405922	3.018866	0.002537227	0.017710155	ENSG00000253953	5	141387697	141512975
ENST00000519564	8.4131078	6.499536809	1.768876	3.674387	0.000238421	0.002729715	ENSG00000178187	5	178941197	178966433
ENST00000519726	4.6423127	5.639148354	1.971416	2.860456	0.004230322	0.026324826	ENSG00000253893	8	8167818	8226614
ENST00000519762	4.1523414	5.47828107	2.024699	2.705726	0.00681552	0.038152509	ENSG00000254338	8	143417678	143419150
ENST00000519811	233.49945	-1.499227398	0.262954	-5.701472	1.19E-08	5.76E-07	ENSG00000105339	8	141128588	141195804
ENST00000520239	210.01872	-1.103133257	0.264715	-4.167241	3.08E-05	0.000501115	ENSG00000254332	8	69129597	69130345
ENST00000520248	141.77837	-1.040975914	0.313301	-3.322611	0.000891792	0.007776126	ENSG00000253919	8	78760141	78762431
ENST00000520530	27.132725	-3.913571912	0.815392	-4.799621	1.59E-06	4.03E-05	ENSG00000163013	2	73254689	73284478
ENST00000520547	338.57407	-1.000950632	0.256709	-3.899161	9.65E-05	0.001293109	ENSG00000253626	10	79512532	79516440
ENST00000521381	175.13418	-1.137784597	0.294021	-3.869745	0.000108949	0.001430239	ENSG00000145675	5	68215755	68301821
ENST00000521604	2393.8374	-1.268292259	0.20415	-6.21255	5.21E-10	3.63E-08	ENSG00000187735	8	53966555	54022448
ENST00000522232	197.53464	1.443740256	0.271905	5.309724	1.10E-07	4.03E-06	ENSG00000231989	5	156850294	156852528
ENST00000522353	528.11768	1.010564387	0.221871	4.554734	5.25E-06	0.000112754	ENSG00000255408	5	140801056	141012347
ENST00000524156	42.503849	2.01532895	0.561225	3.590949	0.000329477	0.003536078	ENSG00000228451	8	26379391	26381519
ENST00000524555	5.8548263	5.974190633	1.874526	3.187041	0.001437365	0.011313862	ENSG00000255071	11	18231422	18248635
ENST00000524732	32.392382	-2.438239415	0.655224	-3.721232	0.000198253	0.002349182	ENSG00000234964	11	59781317	59781722
ENST00000524803	210.27479	-2.062212717	0.282405	-7.302312	2.83E-13	3.95E-11	ENSG00000166788	11	18080291	18106082
ENST00000525123	39.737458	1.905178938	0.531021	3.587768	0.000333521	0.003568433	ENSG00000165923	11	47659590	47715369
ENST00000525539	43.430109	3.082146032	0.584616	5.272089	1.35E-07	4.81E-06	ENSG00000166473	16	81101122	81220370
ENST00000525643	24.857482	-2.532951721	0.688588	-3.67847	0.000234637	0.002692262	ENSG00000008517	16	3065639	3069530
ENST00000525929	537.72425	1.026520331	0.226209	4.537938	5.68E-06	0.000120753	ENSG00000204963	5	140834268	141012347
ENST00000526136	482.10143	1.18109712	0.228009	5.180048	2.22E-07	7.38E-06	ENSG00000204969	5	140794851	141012347
ENST00000526355	41.360314	3.94195809	0.649903	6.065452	1.32E-09	8.31E-08	ENSG00000152402	11	106674018	107018476
ENST00000526635	50.127576	1.510355421	0.508197	2.971987	0.002958787	0.019940383	ENSG00000245849	15	40686182	40695101

ACTBP2	essed_pseudog	Yes	-	108
PCDHGA1	protein_coding	Yes	NM_018912.3	108
PCDHGA6	protein_coding	Yes	NM_018919.3	108
ANGPT1	protein_coding	Yes	NM_001146.5	108
LY6E-DT	lncRNA	Yes	-	108
ANK3-DT	lncRNA	Yes	-	108
FBXO32	protein_coding	Yes	NM_058229.4	108
PCDHGA7	protein_coding	Yes	NM_018920.4	108
TPD52	protein_coding	Yes	NM_001025253.3	108
CLDN23	protein_coding	Yes	NM_194284.3	108
PCDHGB4	protein_coding	Yes	NM_003736.4	108
ZNF454	protein_coding	Yes	NM_001178089.3	108
FAM85B	lncRNA	Yes	-	108
MAFA-AS1	lncRNA	Yes	-	108
DENND3	protein_coding	Yes	NM_001352890.3	108
-	essed_pseudog	Yes	-	108
THAP12P7	essed_pseudog	Yes	-	108
FBXO41	protein_coding	Yes	NM_001371389.2	108
EIF5AL1	protein_coding	Yes	NM_001099692.2	108
PIK3R1	protein_coding	Yes	NM_181523.3	108
TCEA1	protein_coding	Yes	NM_006756.4	108
PPP1R2B	protein_coding	Yes	NM_206858.3	108
PCDHA3	protein_coding	Yes	NM_018906.3	108
SDAD1P1	d_processed_ps	Yes	-	108
SAA2-SAA4	protein_coding	Yes	-	108
FABP5P7	essed_pseudog	Yes	-	108
SAAL1	protein_coding	Yes	NM_138421.3	108
AGBL2	protein_coding	Yes	NM_024783.4	108
PKD1L2	protein_coding	Yes	-	108
IL32	protein_coding	Yes	NM_001376923.1	108
PCDHA7	protein_coding	Yes	NM_018910.3	108
PCDHA2	protein_coding	Yes	NM_018905.3	108
GUCY1A2	protein_coding	Yes	NM_000855.3	108
RAD51-AS1	lncRNA	Yes	-	108

ENST00000526683	2116.8393	-1.209331502	0.228123	-5.301227	1.15E-07	4.19E-06	ENSG00000179950	8	143816343	143829315
ENST00000527483	14.66385	2.654809458	0.913218	2.907092	0.003648064	0.023477961	ENSG00000224023	10	125700435	125719460
ENST00000527620	142.58878	1.537053117	0.372675	4.124375	3.72E-05	0.000587673	ENSG00000254815	11	557594	560106
ENST00000527799	5.3400258	5.842238811	1.912914	3.054104	0.002257337	0.016129535	ENSG00000255158	11	665909	678391
ENST00000529104	145.33554	2.125602036	0.32998	6.441605	1.18E-10	9.91E-09	ENSG00000291008	6	28091136	28093664
ENST00000529310	485.45814	1.122312756	0.222962	5.033652	4.81E-07	1.44E-05	ENSG00000081842	5	140827959	141012347
ENST00000529859	528.1412	1.001898873	0.218642	4.582373	4.60E-06	0.000100672	ENSG00000204965	5	140821612	141012347
ENST00000530122	691.93222	1.024782656	0.20169	5.080968	3.76E-07	1.17E-05	ENSG00000254995	20	58651433	58715410
ENST00000530167	1019.9716	-1.158834124	0.22196	-5.220911	1.78E-07	6.11E-06	ENSG00000188486	11	119093873	119095465
ENST00000530339	614.12022	1.062974493	0.215845	4.924705	8.45E-07	2.34E-05	ENSG00000204967	5	140807067	141012347
ENST00000530835	109.25021	-1.187583998	0.384325	-3.09005	0.002001228	0.014674906	ENSG00000214391	11	90282559	90284172
ENST00000530997	1340.7911	-1.049211193	0.181935	-5.766948	8.07E-09	4.14E-07	ENSG00000100503	14	50719762	50831162
ENST00000531066	1520.1522	1.235983323	0.22192	5.569505	2.55E-08	1.12E-06	ENSG00000166689	11	16777296	17014414
ENST00000531301	44.909155	1.472131848	0.487893	3.017326	0.002550156	0.017782487	ENSG00000213252	11	106826391	106827890
ENST00000531380	40.892477	-3.319494131	0.671393	-4.944186	7.65E-07	2.15E-05	ENSG00000174804	11	86945678	86955395
ENST00000531613	491.80378	1.183523554	0.227486	5.202629	1.96E-07	6.65E-06	ENSG00000204962	5	140841186	141012347
ENST00000531738	109.05295	1.126167039	0.339187	3.320191	0.000899559	0.007823707	ENSG00000064309	11	125956820	126062866
ENST00000532414	62.383086	-2.148587857	0.437583	-4.910124	9.10E-07	2.49E-05	ENSG00000254726	1	156072012	156082465
ENST00000532602	478.37281	1.190348108	0.227802	5.225358	1.74E-07	6.00E-06	ENSG00000204961	5	140848381	141012347
ENST00000532615	77.442557	-1.434447154	0.39286	-3.651291	0.000260925	0.002940972	ENSG00000254719	11	10272051	10272259
ENST00000532864	197.50211	-1.020138702	0.272294	-3.746458	0.000179349	0.002163412	ENSG00000283341	11	43578888	43840030
ENST00000532942	733.74752	-1.319528037	0.208632	-6.324681	2.54E-10	1.96E-08	ENSG00000285283	11	31816383	32104665
ENST00000532971	95.61778	1.655314308	0.356686	4.640813	3.47E-06	7.90E-05	ENSG00000214756	11	62665311	62668108
ENST00000533601	63.752952	-2.430281042	0.447793	-5.427238	5.72E-08	2.32E-06	ENSG00000139998	14	64945815	64972336
ENST00000533924	55.410737	3.879460773	0.577662	6.715796	1.87E-11	1.84E-09	ENSG00000255026	11	287304	288298
ENST00000533945	9.6165132	6.688341789	1.723725	3.880167	0.000104385	0.001379703	ENSG00000255507	11	75803430	75814576
ENST00000534431	4.3849124	5.55734488	1.998527	2.780721	0.005423835	0.03189308	ENSG00000255202	11	33665219	33696701
ENST00000535094	49.709999	-1.725705421	0.487152	-3.542439	0.000396445	0.004100616	ENSG00000126217	13	112969213	113099742
ENST00000535113	71.676281	1.569576827	0.416654	3.767099	0.000165155	0.002022295	ENSG00000184949	22	38578117	38656392
ENST00000535199	9.2846254	6.63915141	1.728632	3.840698	0.000122685	0.001582479	ENSG00000230212	21	36069641	36126640
ENST00000535358	15.712076	4.286158147	1.118663	3.831501	0.000127364	0.001630955	ENSG00000198520	1	44674713	44725591
ENST00000535456	242.89253	-1.397638042	0.25418	-5.498616	3.83E-08	1.61E-06	ENSG00000149328	11	134331987	134376324
ENST00000535817	6.3447976	6.090084753	1.844875	3.301083	0.000963123	0.008246194	ENSG00000256690	11	62832233	62834043
ENST00000535829	52.432084	-3.05425839	0.512906	-5.954806	2.60E-09	1.51E-07	ENSG00000166532	12	8697990	8777191

PUF60	protein_coding	Yes	NM_078480.3	108
EDRF1-DT	lncRNA	Yes	-	108
LMNTD2-AS1	lncRNA	Yes	-	108
-	lncRNA	Yes	-	108
-	lncRNA	Yes	-	108
PCDHA6	protein_coding	Yes	NM_018909.4	108
PCDHA5	protein_coding	Yes	NM_018908.3	108
STX16-NPEPL1	protein_coding	Yes	-	108
H2AX	protein_coding	Yes	NM_002105.3	108
PCDHA4	protein_coding	Yes	NM_018907.4	108
TUBAP2	essed_pseudog	Yes	-	108
NIN	protein_coding	Yes	NM_020921.4	108
PLEKHA7	protein_coding	Yes	NM_001329630.2	108
-	essed_pseudog	Yes	-	108
FZD4	protein_coding	Yes	NM_012193.4	108
PCDHA8	protein_coding	Yes	NM_018911.3	108
CDON	protein_coding	Yes	NM_001378964.1	108
MEX3A	protein_coding	Yes	NM_001093725.2	108
PCDHA9	protein_coding	Yes	NM_031857.2	108
-	essed_pseudog	Yes	-	108
-	lncRNA	Yes	-	108
-	protein_coding	Yes	-	108
CSKMT	protein_coding	Yes	NM_001043229.2	108
RAB15	protein_coding	Yes	NM_001308154.2	108
-	lncRNA	Yes	-	108
UVRAG-DT	lncRNA	Yes	-	108
-	lncRNA	Yes	-	108
MCF2L	protein_coding	Yes	NM_001112732.3	108
FAM227A	protein_coding	Yes	NM_001013647.2	108
CBR1-AS1	lncRNA	Yes	-	108
ARMH1	protein_coding	Yes	NM_001145636.2	108
GLB1L2	protein_coding	Yes	NM_001370461.1	108
STX5-DT	lncRNA	Yes	-	108
RIMKLB	protein_coding	Yes	NM_001297776.2	108

ENST00000537262	258.09743	-2.147785659	0.291215	-7.375246	1.64E-13	2.41E-11	ENSG00000256632	12	132688048	132714912
ENST00000537269	29.453077	2.012020134	0.622345	3.232968	0.001225114	0.009981332	ENSG00000257084	12	6963225	6964447
ENST00000537384	697.19478	-1.243843809	0.303168	-4.102819	4.08E-05	0.000635923	ENSG00000188191	7	549197	727281
ENST00000538779	23.010999	-2.40684931	0.722904	-3.329416	0.000870283	0.00762478	ENSG00000139144	12	18261517	18648416
ENST00000538780	26.882888	-1.838275024	0.627408	-2.929953	0.003390132	0.022121155	ENSG00000139438	12	109713824	109770495
ENST00000538862	298.13022	-1.460376066	0.246365	-5.927682	3.07E-09	1.75E-07	ENSG00000111665	12	6848827	6851286
ENST00000538904	224.38138	-1.001400335	0.291145	-3.439527	0.000582731	0.005554735	ENSG00000142459	19	7830217	7864976
ENST00000538936	661.13077	1.184070332	0.252037	4.697993	2.63E-06	6.23E-05	ENSG00000185813	17	81900957	81911399
ENST00000539111	165.85317	1.128773979	0.29513	3.824673	0.000130946	0.001670368	ENSG00000256525	17	64477784	64497054
ENST00000539745	658.7846	-1.217955583	0.238166	-5.113886	3.16E-07	1.00E-05	ENSG00000197837	12	14770719	14771131
ENST00000540175	166.85305	-1.553978755	0.376146	-4.131316	3.61E-05	0.00057258	ENSG00000256663	12	20551589	20553012
ENST00000541036	328.07756	-2.60442133	0.247845	-10.50826	7.91E-26	6.66E-23	ENSG00000156299	21	31118417	31344261
ENST00000541790	2293.0109	-1.243451976	0.169952	-7.316504	2.55E-13	3.61E-11	ENSG00000273802	6	26216199	26216688
ENST00000542869	64.955452	1.136048802	0.417277	2.722531	0.006478387	0.036698662	ENSG00000101213	20	63528000	63537376
ENST00000544040	4459.3602	-1.021734643	0.163063	-6.265902	3.71E-10	2.71E-08	ENSG00000111642	12	6570081	6607379
ENST00000544421	5.2903674	5.830249366	1.927025	3.025518	0.002482073	0.017394483	ENSG00000257052	11	60906788	60909742
ENST00000544920	13.746961	2.752762316	0.934437	2.945904	0.003220124	0.021283118	ENSG00000256338	15	28315650	28316059
ENST00000545069	11.203525	2.987237096	1.077682	2.77191	0.005572852	0.032561393	ENSG00000256625	12	27121909	27147664
ENST00000545202	40.279458	8.758049221	1.528562	5.729599	1.01E-08	5.00E-07	ENSG00000255980	11	69425677	69429621
ENST00000545904	12.492994	3.144864639	1.024942	3.068335	0.002152554	0.015549955	ENSG00000256747	12	27779820	27781067
ENST00000548055	17.913242	-3.761515412	0.986173	-3.814256	0.000136594	0.001730399	ENSG00000111344	12	113099277	113135761
ENST00000548203	21.254213	-2.961481785	0.795443	-3.723058	0.000196824	0.002337699	ENSG00000257622	14	105152193	105181194
ENST00000548782	4.1275122	5.470619365	2.029646	2.695356	0.007031337	0.039020136	ENSG00000257489	12	100180552	100199762
ENST00000549284	6.1370558	6.041079267	1.857764	3.251801	0.001146762	0.009476202	ENSG00000257356	14	18418774	18419273
ENST00000549521	4.4345708	5.571739292	1.995746	2.791808	0.005241441	0.031089836	ENSG00000258112	12	75257634	75258634
ENST00000549807	9.2597962	6.635729155	1.729705	3.836336	0.000124884	0.001604959	ENSG00000257732	12	104262313	104280722
ENST00000549994	190.44565	2.124531612	0.375341	5.660265	1.51E-08	7.03E-07	ENSG00000135407	12	57797379	57818734
ENST00000550096	4.1275122	5.470619365	2.029646	2.695356	0.007031337	0.039020136	ENSG00000290576	12	100173311	100199783
ENST00000550402	7.299911	6.292863864	1.798459	3.499032	0.000466951	0.00466923	ENSG00000174600	12	108288045	108339311
ENST00000550804	686.99651	1.144229047	0.255068	4.485975	7.26E-06	0.000148267	ENSG00000012822	12	53708516	53727452
ENST00000550948	28.609618	1.741734583	0.607753	2.865861	0.00415877	0.026003428	ENSG00000110876	12	108621894	108633894
ENST00000551270	20.192446	2.00088439	0.745751	2.683047	0.007295471	0.040084719	ENSG00000214198	12	103843755	103915867
ENST00000551380	10.21491	6.777666262	1.706077	3.972661	7.11E-05	0.001004775	ENSG00000166704	19	57977075	58002820
ENST00000551568	7.9655177	-4.967169685	1.783358	-2.78529	0.005347994	0.03154719	ENSG00000135678	12	68851174	68933171

-	lncRNA	Yes	-	108
MIR200CHG	lncRNA	Yes	-	108
PRKAR1B	protein_coding	Yes	NM_001164760.2	108
PIK3C2G	protein_coding	Yes	NM_001288772.2	108
FAM222A	protein_coding	Yes	NM_032829.3	108
CDCA3	protein_coding	Yes	NM_031299.7	108
EVI5L	protein_coding	Yes	NM_001159944.3	108
PCYT2	protein_coding	Yes	NM_002861.5	108
POLG2	protein_coding	Yes	NM_007215.4	108
H4-16	protein_coding	Yes	NM_175054.2	108
-	essed_pseudog	Yes	-	108
TIAM1	protein_coding	Yes	NM_001353694.2	108
H2BC8	protein_coding	Yes	NM_003518.4	108
PTK6	protein_coding	Yes	NM_005975.4	108
CHD4	protein_coding	Yes	NM_001273.5	108
PRPF19-DT	lncRNA	Yes	-	108
RPL41P2	essed_pseudog	Yes	-	108
-	cessed_pseud	Yes	-	108
LINC02953	lncRNA	Yes	-	108
-	lncRNA	Yes	-	108
RASAL1	protein_coding	Yes	NM_001301202.2	108
-	lncRNA	Yes	-	108
-	_unprocessed_p	Yes	-	108
BNIP3P6	essed_pseudog	Yes	-	108
CCNG2P1	essed_pseudog	Yes	-	108
-	lncRNA	Yes	-	108
AVIL	protein_coding	Yes	NM_006576.4	108
-	lncRNA	Yes	-	108
CMKLR1	protein_coding	Yes	NM_001142343.2	108
CALCOCO1	protein_coding	Yes	NM_020898.3	108
SELPLG	protein_coding	Yes	NM_003006.4	108
TTC41P	ed_unitary_pse	Yes	-	108
ZNF606	protein_coding	Yes	NM_001348022.3	108
CPM	protein_coding	Yes	NM_198320.5	108

ENST00000553443	22.556492	2.101433137	0.729382	2.881113	0.003962739	0.025062866	ENSG00000139865	14	37595628	37842717
ENST00000554659	928.10614	-1.418102028	0.195127	-7.267592	3.66E-13	4.96E-11	ENSG00000100568	14	67647084	67674632
ENST00000554737	14.622397	3.123874692	0.9705	3.218831	0.001287145	0.010380182	ENSG00000258813	14	73057924	73059415
ENST00000554922	23.628773	-5.563881182	1.187512	-4.685328	2.80E-06	6.56E-05	ENSG00000165521	14	88612430	88792953
ENST00000554937	118.92506	1.116320744	0.384248	2.905208	0.003670092	0.023578904	ENSG00000179899	12	55411726	55414787
ENST00000555043	6.161885	6.046250309	1.858827	3.252723	0.001143047	0.009451156	ENSG00000258377	14	49620814	49623480
ENST00000555147	73.911967	1.40743325	0.394722	3.565631	0.000362982	0.003820872	ENSG00000258839	16	89918861	89920972
ENST00000555572	4436.5328	-1.052239288	0.196487	-5.355259	8.54E-08	3.27E-06	ENSG00000011052	17	51153635	51171742
ENST00000555815	17.253831	-2.779583745	0.855698	-3.248325	0.001160865	0.009570495	ENSG00000206190	15	25678711	25863327
ENST00000556029	5632.1081	-1.323766545	0.167123	-7.920898	2.36E-15	4.83E-13	ENSG00000120802	12	98515589	98550351
ENST00000556143	294.92495	1.256894333	0.269035	4.671861	2.98E-06	6.93E-05	ENSG00000165861	14	72969450	73027106
ENST00000556147	35.838504	-3.357332107	0.632962	-5.304158	1.13E-07	4.13E-06	ENSG00000165795	14	21016771	21025061
ENST00000556509	36.509983	-1.840641605	0.605823	-3.03825	0.002379564	0.016829313	ENSG00000205683	14	72609033	72894101
ENST00000556529	33.79025	1.857969272	0.578041	3.214254	0.001307838	0.010508141	ENSG00000258102	12	116559380	116576606
ENST00000556919	3.9445995	5.40270887	2.054252	2.630013	0.008538167	0.045174985	ENSG00000258623	14	68683410	68685565
ENST00000557284	116.79709	1.24911015	0.330173	3.783202	0.000154823	0.001918283	ENSG00000259030	1	74198234	74543982
ENST00000558325	417.63117	1.087430888	0.221534	4.908649	9.17E-07	2.51E-05	ENSG00000259371	14	24120955	24131829
ENST00000558480	112.50163	-2.154526076	0.356063	-6.050973	1.44E-09	8.98E-08	ENSG00000058335	15	78959905	79090780
ENST00000558555	56.963688	-1.781659447	0.485811	-3.667392	0.000245037	0.002789408	ENSG00000105419	19	47403123	47419527
ENST00000559228	2396.3946	-1.104214843	0.175083	-6.306796	2.85E-10	2.17E-08	ENSG00000128923	15	58771301	58861900
ENST00000559239	260.16446	-1.170808018	0.271718	-4.308902	1.64E-05	0.000296215	ENSG00000140451	15	64815631	64825647
ENST00000559435	157.42396	-1.10769537	0.31405	-3.527135	0.000420082	0.004298139	ENSG00000259288	15	40218499	40264890
ENST00000559596	3109.5571	-1.052857236	0.207326	-5.078258	3.81E-07	1.18E-05	ENSG00000137804	15	41332880	41381046
ENST00000560346	57.469296	-2.423535945	0.468486	-5.173124	2.30E-07	7.60E-06	ENSG00000137843	15	40239062	40277487
ENST00000561678	622.47215	-1.432394742	0.200306	-7.15103	8.61E-13	1.09E-10	ENSG00000121274	16	50153305	50235310
ENST00000562631	361.44535	-1.490857453	0.263992	-5.647355	1.63E-08	7.49E-07	ENSG00000205336	16	57628629	57665567
ENST00000562783	33.806693	1.533460447	0.591933	2.590598	0.009580926	0.049206045	ENSG00000178115	15	30552049	30565684
ENST00000562790	128.3071	10.42772846	1.473617	7.076282	1.48E-12	1.80E-10	ENSG00000260999	16	68927546	68948261
ENST00000563172	14.486283	-5.871307599	1.641279	-3.577275	0.000347195	0.003682926	ENSG00000261780	18	73324940	73349879
ENST00000563225	54.20938	1.288146495	0.452957	2.843863	0.004457015	0.027383326	ENSG00000261211	6	6680308	6686948
ENST00000563449	25.718106	2.318987769	0.689797	3.361839	0.000774254	0.00695742	ENSG00000261613	16	2554059	2556060
ENST00000563867	4.8997129	5.716560879	1.947521	2.935301	0.00333224	0.021846691	ENSG00000214646	15	77941441	77942747
ENST00000563924	7.2889842	4.802455985	1.778972	2.699569	0.006942945	0.038698843	ENSG00000261371	17	64319414	64390860
ENST00000564741	7.3247402	6.297202409	1.796564	3.505137	0.000456372	0.004584703	ENSG00000261037	5	6019028	6022283

TTC6	protein_coding	Yes	NM_001310135.5	108
VTI1B	protein_coding	Yes	NM_006370.3	108
RBM25-AS1	lncRNA	Yes	-	108
EML5	protein_coding	Yes	NM_183387.3	108
PHC1P1	essed_pseudog	Yes	-	108
-	lncRNA	Yes	-	108
MC1R	protein_coding	Yes	NM_002386.4	108
NME1-NME2	protein_coding	Yes	-	108
ATP10A	protein_coding	Yes	NM_024490.4	108
TMPO	protein_coding	Yes	NM_001032283.3	108
ZFYVE1	protein_coding	Yes	NM_021260.4	108
NDRG2	protein_coding	Yes	NM_001320329.2	108
DPF3	protein_coding	Yes	NM_001280542.3	108
MAP1LC3B2	protein_coding	Yes	NM_001085481.3	108
-	lncRNA	Yes	-	108
FPGT-TNNI3K	protein_coding	Yes	-	108
-	protein_coding	Yes	-	108
RASGRF1	protein_coding	Yes	NM_001145648.3	108
MEIS3	protein_coding	Yes	NM_001301059.2	108
MINDY2	protein_coding	Yes	NM_001040450.3	108
PIF1	protein_coding	Yes	NM_001286496.2	108
BUB1B-PAK6	protein_coding	Yes	-	108
NUSAP1	protein_coding	Yes	NM_016359.5	108
PAK6	protein_coding	Yes	NM_001395430.1	108
TENT4B	protein_coding	Yes	NM_001365324.3	108
ADGRG1	protein_coding	Yes	NM_201525.4	108
GOLGA8Q	protein_coding	Yes	NM_001355476.2	108
-	lncRNA	Yes	-	108
LINC02582	lncRNA	Yes	-	108
-	lncRNA	Yes	-	108
-	lncRNA	Yes	-	108
COMMD4P1	unprocessed_p	Yes	-	108
PECAM1	protein_coding	Yes	NM_000442.5	108
-	lncRNA	Yes	-	108

ENST00000565246	379.80741	2.224353205	0.234516	9.484886	2.43E-21	1.03E-18	ENSG00000261556	16	70219869	70246610
ENST00000565283	149.64768	1.202128176	0.29687	4.049343	5.14E-05	0.000771564	ENSG00000260025	2	36354743	36355471
ENST00000565433	31.503512	-3.288992031	0.666125	-4.937501	7.91E-07	2.21E-05	ENSG00000260401	11	73238974	73242335
ENST00000565519	61.076668	1.139815264	0.434436	2.623668	0.008698849	0.045765617	ENSG00000261572	3	32236687	32238599
ENST00000566085	5.9293139	5.990369929	1.875215	3.194498	0.001400741	0.011089155	ENSG00000260874	16	2456251	2459966
ENST00000566714	14.693615	2.632602469	0.916122	2.873638	0.004057743	0.025522601	ENSG00000261349	1	25266101	25267136
ENST00000566732	251.23876	1.006663521	0.2582	3.898778	9.67E-05	0.00129436	ENSG00000103168	16	84177854	84187057
ENST00000567255	77.444454	1.449494811	0.385148	3.76347	0.000167572	0.002047931	ENSG00000290554	6	41101033	41140835
ENST00000567563	23.462348	-3.300744733	0.947225	-3.484646	0.000492789	0.004873249	ENSG00000259827	16	56682469	56687807
ENST00000567572	79.095785	-3.585110853	0.471682	-7.600701	2.95E-14	5.02E-12	ENSG00000140931	16	66604703	66613887
ENST00000567819	49.122149	1.908116927	0.513628	3.714976	0.000203223	0.002394729	ENSG00000260942	2	240582699	240586699
ENST00000567832	31.58894	1.833840815	0.652844	2.809005	0.004969484	0.029861852	ENSG00000260855	1	246772300	246775772
ENST00000568093	9.4518171	6.669379199	1.789582	3.72678	0.000193942	0.002308744	ENSG00000260648	15	40232081	40236109
ENST00000568179	15.040135	7.336209446	1.630089	4.500497	6.78E-06	0.000140085	ENSG00000266801	16	68933819	68937725
ENST00000568184	3.9197703	5.394631232	2.054307	2.626011	0.008639213	0.045513691	ENSG00000290666	15	77941898	77944582
ENST00000568223	71.375356	-1.338594184	0.409446	-3.269278	0.001078225	0.009007527	ENSG00000103260	16	715117	719655
ENST00000568248	291.76318	-2.157974533	0.285933	-7.547138	4.45E-14	7.24E-12	ENSG00000259820	8	134792019	134798272
ENST00000568855	251.02266	2.239491021	0.263132	8.51089	1.73E-17	4.63E-15	ENSG00000291219	16	70229803	70246612
ENST00000568943	4.1771706	5.485899544	2.023138	2.711579	0.006696351	0.037642369	ENSG00000103546	16	55655987	55706192
ENST00000569046	606.10833	-1.174698308	0.229712	-5.113777	3.16E-07	1.00E-05	ENSG00000259781	15	71164769	71165415
ENST00000569188	28.253599	4.408569804	0.874165	5.043179	4.58E-07	1.38E-05	ENSG00000103044	16	69105652	69117660
ENST00000569417	147.11833	1.192327252	0.328941	3.624747	0.000289244	0.003197869	ENSG00000167965	16	2205453	2209453
ENST00000569580	8.2550244	6.470616567	1.762642	3.670977	0.000241625	0.00275915	ENSG00000260927	16	58129518	58163246
ENST00000569591	31.152259	2.752932353	0.652938	4.216221	2.48E-05	0.000417965	ENSG00000260314	10	17809347	17911164
ENST00000569669	870.93457	-1.414986308	0.273058	-5.182004	2.20E-07	7.32E-06	ENSG00000261236	8	144262044	144291438
ENST00000569988	4.8748837	5.710071607	1.949769	2.928589	0.003405044	0.022197012	ENSG00000260714	16	25015977	25021023
ENST00000570309	46.150022	1.682294533	0.516679	3.255974	0.001130041	0.009363414	ENSG00000262580	17	80200672	80205071
ENST00000571252	95.11321	1.298620156	0.378412	3.431761	0.000599676	0.005678609	ENSG00000262576	5	141355020	141512975
ENST00000571422	4.4097416	5.564557071	1.995673	2.788312	0.005298356	0.031321376	ENSG00000262539	17	46259550	46260606
ENST00000572272	34.666195	2.016939341	0.579947	3.477801	0.000505544	0.0049714	ENSG00000091592	17	5514117	5584509
ENST00000572447	415.25494	-1.409143801	0.228815	-6.158454	7.35E-10	4.96E-08	ENSG00000129195	17	6444454	6451469
ENST00000572705	217.3083	1.019351617	0.265718	3.83621	0.000124948	0.001605549	ENSG00000196689	17	3565445	3609411
ENST00000573312	61.975632	1.700631451	0.493885	3.443373	0.000574506	0.005495231	ENSG00000261888	17	83104254	83106910
ENST00000573318	85.871316	1.292891426	0.380063	3.401782	0.000669481	0.006202058	ENSG00000228782	17	47482991	47492459

SMG1P7	unprocessed_p	Yes	-	108
CRIM1-DT	lncRNA	Yes	-	108
-	lncRNA	Yes	-	108
-	lncRNA	Yes	-	108
-	lncRNA	Yes	-	108
-	cessed_pseudo	Yes	-	108
TAF1C	protein_coding	Yes	NM_001243156.2	108
ADCY10P1	lncRNA	Yes	-	108
-	lncRNA	Yes	-	108
CMTM3	protein_coding	Yes	NM_181553.4	108
CAPN10-DT	lncRNA	Yes	-	108
-	lncRNA	Yes	-	108
-	lncRNA	Yes	-	108
-	lncRNA	Yes	-	108
-	lncRNA	Yes	-	108
METRN	protein_coding	Yes	NM_024042.4	108
MIR30DHG	lncRNA	Yes	-	108
-	lncRNA	Yes	-	108
SLC6A2	protein_coding	Yes	NM_001172501.3	108
HMGB1P6	essed_pseudog	Yes	-	108
HAS3	protein_coding	Yes	NM_001199280.2	108
MLST8	protein_coding	Yes	NM_022372.6	108
-	lncRNA	Yes	-	108
MRC1	protein_coding	Yes	NM_002438.4	108
BOP1	protein_coding	Yes	NM_015201.5	108
-	essed_pseudog	Yes	-	108
-	lncRNA	Yes	-	108
PCDHGA4	protein_coding	Yes	NM_018917.4	108
-	essed_pseudog	Yes	-	108
NLRP1	protein_coding	Yes	NM_033004.4	108
PIMREG	protein_coding	Yes	NM_019013.3	108
TRPV1	protein_coding	Yes	NM_080704.4	108
-	lncRNA	Yes	-	108
-	unprocessed_p	Yes	-	108

ENST00000574428	311.70056	-1.161571492	0.248709	-4.670409	3.01E-06	6.97E-05	ENSG00000123472	1	46632736	46668364
ENST00000574616	1548.0948	-1.574951479	0.184327	-8.544341	1.29E-17	3.53E-15	ENSG00000263244	16	9104847	9113181
ENST00000575018	83.220158	-1.822050692	0.385925	-4.72126	2.34E-06	5.66E-05	ENSG00000262519	17	4572205	4572515
ENST00000575310	96.656632	2.684465741	0.429529	6.249784	4.11E-10	2.95E-08	ENSG00000262880	17	7420102	7444081
ENST00000575331	253.16698	1.97330931	0.286378	6.890578	5.56E-12	6.05E-10	ENSG00000272884	17	7439505	7445966
ENST00000576060	7.6409071	6.361575644	1.811108	3.512533	0.000443858	0.00449046	ENSG00000182896	17	7355155	7357219
ENST00000576222	81.31532	1.159696451	0.382686	3.03041	0.002442221	0.017183146	ENSG00000262209	5	141370241	141512975
ENST00000576762	3.9197703	5.394631232	2.054307	2.626011	0.008639213	0.045513691	ENSG00000262521	16	3181232	3184018
ENST00000577781	45.707076	3.450265832	0.61445	5.615213	1.96E-08	8.83E-07	ENSG00000263826	3	186781779	186784179
ENST00000577809	283.59129	-1.162331728	0.245902	-4.726815	2.28E-06	5.53E-05	ENSG00000108666	17	32328440	32342173
ENST00000577970	29.959975	4.831923581	0.901749	5.358391	8.40E-08	3.22E-06	ENSG00000263567	17	31762439	31769048
ENST00000577988	165.63093	-1.235016663	0.285352	-4.328042	1.50E-05	0.000275289	ENSG00000265185	17	19061911	19062669
ENST00000578036	454.79031	1.272264775	0.263035	4.836866	1.32E-06	3.43E-05	ENSG00000291117	17	64779258	64837139
ENST00000578186	4747.8644	-1.376660717	0.172875	-7.963309	1.67E-15	3.51E-13	ENSG00000270882	1	149832656	149833052
ENST00000578492	411.42805	1.665500481	0.317061	5.252934	1.50E-07	5.28E-06	ENSG00000215769	17	64749662	64781626
ENST00000579209	99.604322	2.071968763	0.412778	5.019566	5.18E-07	1.53E-05	ENSG00000263647	17	64761911	64762710
ENST00000579212	202.02847	1.088709975	0.291952	3.729069	0.000192189	0.002292171	ENSG00000284368	17	64501213	64501313
ENST00000579299	4.7755669	5.683608333	1.984913	2.863404	0.004191157	0.026156931	ENSG00000264958	17	30529687	30542416
ENST00000579774	527.1743	1.841354814	0.2523	7.29828	2.91E-13	4.06E-11	ENSG00000265107	1	147756198	147760602
ENST00000579849	25.368799	-1.979511555	0.650021	-3.045302	0.002324467	0.016513975	ENSG00000006042	17	32928152	32944315
ENST00000580170	36.126605	-6.178179026	1.140488	-5.417139	6.06E-08	2.43E-06	ENSG00000173482	18	7567315	8406856
ENST00000580341	5.5974261	5.909724272	1.89259	3.122559	0.001792859	0.013471947	ENSG00000277452	1	150566561	150566860
ENST00000580533	318.34838	2.032109374	0.309967	6.555897	5.53E-11	4.93E-09	ENSG00000264940	17	19189664	19190245
ENST00000580960	7.9388575	6.410816918	1.78307	3.595382	0.000323915	0.003490403	ENSG00000265542	17	57771945	57834749
ENST00000581050	102.16224	2.123270698	0.372359	5.702218	1.18E-08	5.74E-07	ENSG00000291263	16	70219580	70226033
ENST00000581130	129.65504	1.316935384	0.333999	3.942928	8.05E-05	0.001113956	ENSG00000284564	17	64500773	64500839
ENST00000581296	193.4392	1.459524589	0.347915	4.195065	2.73E-05	0.000451872	ENSG00000214425	17	45508108	45550335
ENST00000581633	4.4594	5.578907621	1.99861	2.791394	0.005248159	0.03111273	ENSG00000264125	17	30204317	30206468
ENST00000581792	91.959575	1.245202128	0.359113	3.467436	0.000525448	0.005127021	ENSG00000264462	21	8986998	8987178
ENST00000582201	311.12757	1.601127796	0.279513	5.72828	1.01E-08	5.03E-07	ENSG00000214176	17	64785258	64829208
ENST00000582394	66.551177	1.064746365	0.405229	2.62752	0.008600988	0.045429445	ENSG00000263887	17	62282701	62297026
ENST00000582558	6.7602813	6.183328809	1.829831	3.37918	0.000727024	0.00661729	ENSG00000264569	17	82037871	82039380
ENST00000582970	3229.0791	1.725100058	0.237175	7.273526	3.50E-13	4.78E-11	ENSG00000173821	17	80260851	80398794
ENST00000583452	5.8051679	5.963296083	1.882633	3.16753	0.001537398	0.011931177	ENSG00000264421	17	67224302	67225541

ATPAF1	protein_coding	Yes	NM_001394565.1	108
-	lncRNA	Yes	-	108
TXNP4	essed_pseudog	Yes	-	108
-	lncRNA	Yes	-	108
-	lncRNA	Yes	-	108
TMEM95	protein_coding	Yes	NM_001320436.2	108
PCDHGB3	protein_coding	Yes	NM_018924.5	108
-	lncRNA	Yes	-	108
-	lncRNA	Yes	-	108
C17orf75	protein_coding	Yes	NM_022344.4	108
-	lncRNA	Yes	-	108
SNORD3B-1	snoRNA	Yes	-	108
-	lncRNA	Yes	-	108
H4C14	protein_coding	Yes	NM_003548.2	108
ARHGAP27P1-BPTFP1-KPNA2P3	lncRNA	Yes	-	108
BPTFP1	cessed_pseud	Yes	-	108
MIR5047	miRNA	Yes	-	108
ALOX12P1	cessed_pseud	Yes	-	108
GJA5	protein_coding	Yes	NM_181703.4	108
TMEM98	protein_coding	Yes	NM_015544.3	108
PTPRM	protein_coding	Yes	NM_001105244.2	108
RN7SL473P	misc_RNA	Yes	-	108
SNORD3C	snoRNA	Yes	-	108
-	lncRNA	Yes	-	108
SMG1P7	lncRNA	Yes	-	108
MIR3064	miRNA	Yes	-	108
LRRC37A4P	_unprocessed_	Yes	-	108
-	lncRNA	Yes	-	108
MIR3648-2	miRNA	Yes	-	108
PLEKHM1P1	_unprocessed_	Yes	-	108
USP32P4	_unprocessed_	Yes	-	108
DCXR-DT	lncRNA	Yes	-	108
RNF213	protein_coding	Yes	NM_001256071.3	108
-	lncRNA	Yes	-	108

ENST00000583931	2397.3669	-1.66364588	0.188926	-8.805812	1.30E-18	4.13E-16	ENSG00000143401	1	150218416	150236112
ENST00000584190	187.46727	1.971574203	0.374154	5.26942	1.37E-07	4.87E-06	ENSG00000266820	17	64749917	64758603
ENST00000584253	4.2855874	-5.573929283	2.013034	-2.76892	0.005624245	0.032777485	ENSG00000265565	6	27147625	27147688
ENST00000585193	4.6174835	5.632298037	1.975236	2.851455	0.004351962	0.026886003	ENSG00000266473	17	67244836	67245806
ENST00000585482	35.833123	2.58486674	0.638312	4.049535	5.13E-05	0.000771216	ENSG00000004139	17	28371693	28404049
ENST00000585807	203.62211	1.440449903	0.272921	5.277893	1.31E-07	4.68E-06	ENSG00000108786	17	42552922	42555214
ENST00000586582	10.892926	-3.781064675	1.194198	-3.166195	0.001544473	0.011972064	ENSG00000167680	19	4542592	4559684
ENST00000587682	106.61122	1.2589283	0.395033	3.186894	0.001438093	0.011318258	ENSG00000159905	19	43951227	43967709
ENST00000588129	63.810714	1.279954677	0.448071	2.856589	0.004282204	0.026559843	ENSG00000283236	19	51860241	51863236
ENST00000588776	108.73856	-1.400111199	0.429575	-3.259297	0.001116888	0.009274097	ENSG00000105519	19	5914253	5916211
ENST00000588981	103.66584	-3.419654257	0.453974	-7.532706	4.97E-14	8.02E-12	ENSG00000105048	19	55132697	55149206
ENST00000589764	27.156912	-1.9321601	0.629016	-3.071717	0.002128311	0.01540403	ENSG00000267533	18	12067172	12068417
ENST00000590474	114.43474	-6.038443783	0.665247	-9.076995	1.12E-19	4.03E-17	ENSG00000108984	17	69414696	69553865
ENST00000590508	145.02181	1.100698859	0.339357	3.243484	0.001180773	0.009692613	ENSG00000132003	19	13795442	13832254
ENST00000590800	29.254765	-2.309233314	0.626438	-3.686292	0.000227545	0.002626112	ENSG00000184828	18	48026671	48409383
ENST00000591146	3.9694287	5.410767483	2.057658	2.629576	0.008549154	0.045217418	ENSG00000267736	19	1203208	1203842
ENST00000591504	38.890749	-2.63275704	0.565678	-4.654165	3.25E-06	7.46E-05	ENSG00000166510	18	54901508	54959461
ENST00000591866	4.8997129	5.716560879	1.947521	2.935301	0.00333224	0.021846691	ENSG00000267666	19	663481	669500
ENST00000592087	30.606243	2.619334019	0.637567	4.108328	3.99E-05	0.000623367	ENSG00000267309	19	36489648	36491040
ENST00000592431	184.89773	3.210364054	0.321597	9.982562	1.82E-23	1.14E-20	ENSG00000267475	19	32686917	32691764
ENST00000592524	27.236695	1.717430468	0.63441	2.707132	0.006786722	0.038042332	ENSG00000182963	17	44798447	44830287
ENST00000592540	570.09199	-1.954807124	0.23735	-8.235974	1.78E-16	4.25E-14	ENSG00000161888	19	11145492	11155782
ENST00000592792	587.85107	-1.127055536	0.201639	-5.589484	2.28E-08	1.01E-06	ENSG00000196922	8	144976189	144999723
ENST00000593393	37.820297	1.586654142	0.570184	2.782706	0.00539076	0.031750634	ENSG00000232098	19	58404237	58407296
ENST00000593945	68.895051	-3.635232866	0.486633	-7.470173	8.01E-14	1.24E-11	ENSG00000074219	19	49340594	49362416
ENST00000594254	61.956825	1.296608247	0.425826	3.044928	0.002327358	0.016531431	ENSG00000213976	19	21382864	21387177
ENST00000595010	54.598172	3.29232109	0.520216	6.328761	2.47E-10	1.92E-08	ENSG00000269235	19	51949115	51981367
ENST00000595224	12.326705	2.569464723	0.967745	2.655106	0.007928359	0.042739329	ENSG00000269313	X	49162757	49168774
ENST00000595607	320.50247	4.035580851	0.292546	13.79467	2.74E-43	7.21E-40	ENSG00000178150	19	48270080	48287608
ENST00000597229	136.63258	-2.041601974	0.324095	-6.299392	2.99E-10	2.26E-08	ENSG00000198816	19	7516102	7521025
ENST00000597420	3.7926406	-5.397134966	2.073037	-2.603492	0.009227952	0.047833259	ENSG00000269564	19	53788781	53789168
ENST00000598293	37.294282	2.095780669	0.552836	3.790965	0.000150063	0.001869965	ENSG00000169169	19	49691115	49713731
ENST00000599078	21.926768	2.13342702	0.71529	2.982603	0.002858084	0.019412783	ENSG00000268658	19	21483714	21503238
ENST00000599312	37.213396	-1.633826055	0.536032	-3.048003	0.002303674	0.01639759	ENSG00000267952	19	7507081	7519526

ANP32E	protein_coding	Yes	NM_030920.5	108
KPNA2P3	unprocessed_p	Yes	-	108
MIR3143	miRNA	Yes	-	108
HELZ-AS1	lncRNA	Yes	-	108
SARM1	protein_coding	Yes	NM_015077.4	108
HSD17B1	protein_coding	Yes	NM_000413.4	108
SEMA6B	protein_coding	Yes	NM_032108.4	108
ZNF221	protein_coding	Yes	NM_001297588.2	108
-	unprocessed_p	Yes	-	108
CAPS	protein_coding	Yes	NM_004058.5	108
TNNT1	protein_coding	Yes	NM_003283.6	108
-	essed_pseudog	Yes	-	108
MAP2K6	protein_coding	Yes	NM_002758.4	108
ZSWIM4	protein_coding	Yes	NM_001367834.3	108
ZBTB7C	protein_coding	Yes	NM_001318841.2	108
HMGB2P1	d_processed_ps	Yes	-	108
CCDC68	protein_coding	Yes	NM_025214.3	108
-	lncRNA	Yes	-	108
ZNF566-AS1	lncRNA	Yes	-	108
NUDT19-DT	lncRNA	Yes	-	108
GJC1	protein_coding	Yes	NM_005497.4	108
SPC24	protein_coding	Yes	NM_182513.4	108
ZNF252P	unprocessed_p	Yes	-	108
ZNF584-DT	lncRNA	Yes	-	108
TEAD2	protein_coding	Yes	NM_001256660.2	108
-	cessed_pseudo	Yes	-	108
ZNF350-AS1	lncRNA	Yes	-	108
MAGIX	protein_coding	Yes	NM_024859.4	108
ZNF114	protein_coding	Yes	NM_153608.4	108
ZNF358	protein_coding	Yes	NM_018083.5	108
-	lncRNA	Yes	-	108
CPT1C	protein_coding	Yes	NM_001199753.2	108
LINC00664	lncRNA	Yes	-	108
-	protein_coding	Yes	-	108

ENST00000599640	3.9445995	5.40270887	2.054252	2.630013	0.008538167	0.045174985	ENSG00000227733	1	148159212	148246747
ENST00000599904	4.5593578	-5.662458128	1.980957	-2.858446	0.004257221	0.026441471	ENSG00000267882	20	47318501	47320754
ENST00000599921	101.82621	4.104108229	0.462235	8.878827	6.76E-19	2.23E-16	ENSG00000105499	19	48047845	48110817
ENST00000599944	7.3992278	6.310151505	1.797481	3.510552	0.000447177	0.004516245	ENSG00000267924	19	23817598	23874701
ENST00000600128	54.111209	-4.271534736	0.72308	-5.907413	3.48E-09	1.94E-07	ENSG00000142449	19	8065401	8149592
ENST00000600729	57.10873	1.181801472	0.434161	2.722035	0.00648812	0.036742869	ENSG00000268975	19	40771647	40796943
ENST00000600882	58.380782	-2.115620829	0.489046	-4.32602	1.52E-05	0.000277427	ENSG00000019144	11	118607614	118658028
ENST00000600996	107.66204	-1.099799029	0.343214	-3.204416	0.001353366	0.010796541	ENSG00000268034	19	41506151	41506898
ENST00000602195	12.183903	7.034514586	1.693431	4.154002	3.27E-05	0.000527079	ENSG00000269437	X	102360394	102440008
ENST00000602318	456.97773	1.271993108	0.221523	5.742035	9.35E-09	4.69E-07	ENSG00000134248	1	110401252	110407694
ENST00000602347	6.7263439	6.171089666	1.842437	3.349418	0.000809816	0.007200567	ENSG00000270130	12	123960716	123961244
ENST00000602478	95.589384	-1.838954816	0.371056	-4.956009	7.20E-07	2.04E-05	ENSG00000270022	22	42615243	42615907
ENST00000602595	6.286031	6.072216831	1.889163	3.214236	0.001307919	0.01050824	ENSG00000270016	15	30607694	30608193
ENST00000602739	10.765426	3.247286108	1.169649	2.776291	0.005498292	0.032222098	ENSG00000270020	16	86520382	86523897
ENST00000603191	5.6379762	5.913956072	2.051049	2.883381	0.003934316	0.024923439	ENSG00000271551	6	156776359	156778422
ENST00000603208	31.261914	2.118717935	0.626829	3.38006	0.000724701	0.006602447	ENSG00000270393	22	19018042	19018916
ENST00000603357	330.241	-1.749834753	0.2505	-6.985376	2.84E-12	3.26E-10	ENSG00000180346	4	89111532	89114901
ENST00000603504	32.663404	1.79703589	0.587782	3.057319	0.002233264	0.015992268	ENSG00000271394	17	35889557	35889804
ENST00000603540	16.329128	2.433452683	0.852304	2.855146	0.004301708	0.026654779	ENSG00000139985	14	70452173	70459905
ENST00000603937	3.8949411	5.386514595	2.058112	2.617211	0.008865141	0.04637373	ENSG00000271283	19	19699202	19699409
ENST00000604030	621.20412	-1.11481191	0.201909	-5.521353	3.36E-08	1.43E-06	ENSG00000109686	4	151120280	151325605
ENST00000604082	5.2067716	5.802330943	1.930916	3.004963	0.002656136	0.018353552	ENSG00000271265	6	156774216	156774662
ENST00000604716	46.398323	2.57625977	0.593767	4.338841	1.43E-05	0.000264135	ENSG00000270605	1	28239508	28241453
ENST00000604729	912.48531	1.462236114	0.20748	7.047589	1.82E-12	2.17E-10	ENSG00000214655	10	73785605	73801793
ENST00000605757	6.3696268	6.09507428	1.84369	3.30591	0.000946686	0.008137461	ENSG00000270326	6	28319659	28319852
ENST00000606173	93.635011	1.045124201	0.380366	2.747682	0.006001823	0.034496401	ENSG00000290683	1	247199398	247210856
ENST00000606358	4.4097416	5.564557071	1.995673	2.788312	0.005298356	0.031321376	ENSG00000272459	5	177554823	177555364
ENST00000606379	247.36637	1.456210316	0.254772	5.715749	1.09E-08	5.36E-07	ENSG00000272084	1	19072109	19075511
ENST00000606729	6.146164	6.048747967	1.91833	3.153132	0.001615288	0.0124099	ENSG00000271931	6	89080163	89080667
ENST00000606888	9.645221	3.105373994	1.182924	2.625168	0.008660632	0.045604464	ENSG00000272031	1	145959440	145964575
ENST00000606924	77.566133	1.513810532	0.41111	3.682253	0.000231182	0.002660119	ENSG00000272341	6	16764345	16766883
ENST00000607056	75.215411	-1.072674085	0.387073	-2.771248	0.005584188	0.032613877	ENSG00000272335	5	44826075	44828592
ENST00000607176	3.9445995	5.40270887	2.054252	2.630013	0.008538167	0.045174985	ENSG00000271882	8	101208147	101208558
ENST00000607355	608.05332	-1.670599822	0.248519	-6.72221	1.79E-11	1.77E-09	ENSG00000288859	1	149851060	149851594

-	lncRNA	Yes	-	108
-	lncRNA	Yes	-	108
PLA2G4C	protein_coding	Yes	NM_003706.3	108
-	lncRNA	Yes	-	108
FBN3	protein_coding	Yes	NM_032447.5	108
MIA-RAB4B	protein_coding	Yes	-	108
PHLDB1	protein_coding	Yes	NM_001144758.3	108
TPM3P5	essed_pseudog	Yes	-	108
NXF2B	protein_coding	Yes	NM_001099686.3	108
LAMTOR5	protein_coding	Yes	NM_001382293.1	108
-	lncRNA	Yes	-	108
-	lncRNA	Yes	-	108
-	lncRNA	Yes	-	108
-	lncRNA	Yes	-	108
-	lncRNA	Yes	-	108
-	essed_pseudog	Yes	-	108
TIGD2	protein_coding	Yes	NM_145715.3	108
7SK	misc_RNA	Yes	-	108
ADAM21	protein_coding	Yes	NM_003813.4	108
-	essed_pseudog	Yes	-	108
SH3D19	protein_coding	Yes	NM_001378122.1	108
-	lncRNA	Yes	-	108
-	lncRNA	Yes	-	108
ZSWIM8	protein_coding	Yes	NM_001367799.1	108
SMIM15P2	essed_pseudog	Yes	-	108
-	lncRNA	Yes	-	108
FAM193B-DT	lncRNA	Yes	-	108
-	lncRNA	Yes	-	108
PNRC1-DT	lncRNA	Yes	-	108
ANKRD34A	protein_coding	Yes	NM_001039888.4	108
-	lncRNA	Yes	-	108
-	lncRNA	Yes	-	108
-	lncRNA	Yes	-	108
H2AC19	protein_coding	Yes	NM_001040874.1	108

ENST00000607452	126.68665	1.51309348	0.318618	4.748934	2.04E-06	5.04E-05	ENSG00000170634	2	53971112	54305300
ENST00000607727	20.099963	3.892026685	0.908602	4.283532	1.84E-05	0.000326095	ENSG00000271755	6	27404009	27406964
ENST00000607876	11.34796	3.01972385	1.133774	2.663428	0.007734906	0.041939759	ENSG00000272848	6	169426419	169452475
ENST00000607927	33.413244	1.834101598	0.572534	3.203481	0.001357771	0.01082503	ENSG00000225335	22	18077185	18078884
ENST00000608005	18.474394	2.632167496	0.86741	3.034514	0.002409237	0.016991637	ENSG00000272630	10	73098043	73101297
ENST00000608013	79.423504	1.34829376	0.402711	3.348041	0.000813849	0.007227987	ENSG00000291126	2	95526650	95532405
ENST00000608016	7.316535	3.750334524	1.448647	2.588852	0.009629635	0.049405219	ENSG00000273137	22	50208460	50209542
ENST00000608162	5.9044847	5.984986571	1.873264	3.19495	0.00139855	0.011075257	ENSG00000273335	18	9473420	9474006
ENST00000608574	21.979148	2.027440712	0.713769	2.840473	0.004504667	0.027621535	ENSG00000273382	1	109087970	109090858
ENST00000608684	10.118258	-6.812748997	1.707919	-3.988918	6.64E-05	0.000950513	ENSG00000273338	1	78004345	78004554
ENST00000608770	49.999115	-2.537922373	0.501742	-5.058226	4.23E-07	1.29E-05	ENSG00000272732	7	7550103	7552440
ENST00000608984	5.1571132	5.790031587	1.926318	3.005751	0.002649259	0.018320612	ENSG00000272911	17	2688472	2688960
ENST00000609162	24.65856	1.934650895	0.700646	2.761239	0.005758257	0.033406482	ENSG00000272720	22	38090126	38091559
ENST00000609256	22.561575	-3.87211884	0.966436	-4.006596	6.16E-05	0.000892361	ENSG00000180535	7	98211438	98215457
ENST00000609514	4.1523414	5.47828107	2.024699	2.705726	0.00681552	0.038152509	ENSG00000273143	10	110428839	110496187
ENST00000609791	3.9197703	5.394631232	2.054307	2.626011	0.008639213	0.045513691	ENSG00000273442	22	17080700	17081456
ENST00000609924	11.346549	5.448166773	1.668829	3.264665	0.001095935	0.009126461	ENSG00000272688	18	3245287	3247433
ENST00000609936	7.8395407	6.394636132	1.775957	3.600671	0.000317397	0.003439132	ENSG00000272682	22	19124308	19128449
ENST00000610050	14.292763	7.262893339	1.63995	4.428729	9.48E-06	0.000185457	ENSG00000272836	22	50205584	50206062
ENST00000610331	28.733546	-1.599175224	0.609521	-2.623661	0.008699044	0.045765617	ENSG00000276334	2	32521926	32523547
ENST00000610533	767.95142	-1.234883843	0.203135	-6.079133	1.21E-09	7.71E-08	ENSG00000106628	7	44114679	44123548
ENST00000610828	10.032884	-3.229749259	1.154123	-2.798444	0.00513495	0.030634626	ENSG00000136098	13	52132646	52159597
ENST00000610841	15.164281	7.34674532	1.625993	4.518314	6.23E-06	0.0001305	ENSG00000275504	16	68926893	68926957
ENST00000611161	69.074759	1.213096054	0.406274	2.985903	0.002827427	0.019252603	ENSG00000275183	19	54461731	54463778
ENST00000611299	73.370338	1.285056377	0.403451	3.185158	0.001446752	0.011371888	ENSG00000164674	6	158650053	158764871
ENST00000611366	55.250965	1.4839871	0.490887	3.02307	0.002502242	0.017511671	ENSG00000100918	14	24172079	24180254
ENST00000611590	41.672982	1.638568449	0.508752	3.220763	0.001278499	0.010324113	ENSG00000182612	17	81637195	81648749
ENST00000611745	8.4966883	-4.00017719	1.484246	-2.695091	0.007036945	0.039042878	ENSG00000177679	7	76201895	76287287
ENST00000611884	545.51826	-1.033530714	0.205152	-5.037869	4.71E-07	1.42E-05	ENSG00000174891	3	158110088	158545730
ENST00000612078	6.4192852	6.105021644	1.845666	3.307761	0.000940449	0.008103505	ENSG00000186526	19	15615217	15630639
ENST00000612480	152.92752	1.154780222	0.295282	3.910772	9.20E-05	0.001242868	ENSG00000275131	1	120489859	120701289
ENST00000612813	111.1169	1.109074287	0.379293	2.92406	0.003454979	0.022473507	ENSG00000165949	14	94110814	94116695
ENST00000612814	430.61142	-1.227389738	0.242806	-5.05503	4.30E-07	1.31E-05	ENSG00000076351	17	28394641	28406212
ENST00000612820	4.8500545	5.703545452	1.954491	2.918175	0.003520872	0.022814493	ENSG00000276626	3	124792318	124792562

ACYP2	protein_coding	Yes	NM_001320586.2	108
-	lncRNA	Yes	-	108
-	lncRNA	Yes	-	108
-	lncRNA	Yes	-	108
-	lncRNA	Yes	-	108
-	lncRNA	Yes	-	108
SELENOO-AS1	lncRNA	Yes	-	108
-	lncRNA	Yes	-	108
TMEM167B-DT	lncRNA	Yes	-	108
-	lncRNA	Yes	-	108
-	lncRNA	Yes	-	108
-	lncRNA	Yes	-	108
-	lncRNA	Yes	-	108
BHLHA15	protein_coding	Yes	NM_177455.4	108
DUSP5-DT	lncRNA	Yes	-	108
-	lncRNA	Yes	-	108
-	lncRNA	Yes	-	108
-	lncRNA	Yes	-	108
-	lncRNA	Yes	-	108
-	lncRNA	Yes	-	108
POLD2	protein_coding	Yes	NM_006230.4	108
NEK3	protein_coding	Yes	NM_002498.3	108
U7	snRNA	Yes	-	108
LENG9	protein_coding	Yes	NM_001301782.2	108
SYTL3	protein_coding	Yes	NM_001242394.2	108
REC8	protein_coding	Yes	NM_001048205.2	108
TSPAN10	protein_coding	Yes	NM_001290212.2	108
SRRM3	protein_coding	Yes	NM_001110199.3	108
RSRC1	protein_coding	Yes	NM_001271838.2	108
CYP4F8	protein_coding	Yes	NM_007253.4	108
PDE4DIPP2	unprocessed_p	Yes	-	108
IFI27	protein_coding	Yes	-	108
SLC46A1	protein_coding	Yes	NM_080669.6	108
7SK	misc_RNA	Yes	-	108

ENST00000612829	748.454	-1.338298318	0.22341	-5.990326	2.09E-09	1.24E-07	ENSG00000284378	2	231713313	231713398
ENST00000612899	182.72251	1.381304246	0.291885	4.732354	2.22E-06	5.40E-05	ENSG00000118503	6	137867258	137883312
ENST00000612966	1111.3686	-1.357028311	0.184501	-7.355113	1.91E-13	2.78E-11	ENSG00000287080	6	26045383	26045869
ENST00000612985	9.734284	-3.204057795	1.215205	-2.636641	0.008373147	0.044571098	ENSG00000277610	1	120942599	120942763
ENST00000613174	852.83302	-1.378479033	0.196775	-7.005368	2.46E-12	2.87E-10	ENSG00000276903	6	27865316	27865798
ENST00000613769	22.620851	-3.218084369	0.775542	-4.149467	3.33E-05	0.000535882	ENSG00000290856	13	52600118	52637368
ENST00000613854	1112.475	-1.187069414	0.182369	-6.509158	7.56E-11	6.56E-09	ENSG00000275714	6	26020450	26020958
ENST00000613868	15.607275	3.200351361	0.95665	3.345373	0.000821719	0.007283879	ENSG00000275491	20	3808356	3812434
ENST00000614097	29.875028	3.146528276	0.675529	4.657871	3.19E-06	7.35E-05	ENSG00000274290	6	26183760	26184230
ENST00000614247	2874.0437	-1.369334292	0.171818	-7.969694	1.59E-15	3.34E-13	ENSG00000277157	6	26188709	26189112
ENST00000614302	35.222983	-1.94289708	0.573913	-3.385351	0.000710873	0.00649939	ENSG00000176715	16	89093851	89156233
ENST00000614378	1883.2344	-1.47640658	0.172558	-8.556005	1.17E-17	3.21E-15	ENSG00000273983	6	26270917	26271413
ENST00000614492	35.627541	1.772879045	0.654681	2.708003	0.00676894	0.037958017	ENSG00000277437	21	8208843	8208904
ENST00000614586	40.304425	2.222285851	0.545121	4.076682	4.57E-05	0.000699616	ENSG00000276975	1	146547366	146898974
ENST00000614891	6338.0688	-1.44309566	0.164572	-8.768781	1.81E-18	5.65E-16	ENSG00000147676	8	119208362	119245673
ENST00000615164	1932.2435	-1.706059694	0.172297	-9.901862	4.09E-23	2.36E-20	ENSG00000276966	6	26204609	26205021
ENST00000615353	395.90065	-1.010617246	0.221102	-4.570822	4.86E-06	0.00010552	ENSG00000276180	6	27139281	27139678
ENST00000615531	67.578146	1.964610156	0.460826	4.263237	2.01E-05	0.000350878	ENSG00000088340	20	35558865	35607494
ENST00000615665	70.888752	-1.636483159	0.455491	-3.592785	0.000327162	0.003518028	ENSG00000145687	5	81412803	81751097
ENST00000615674	5.132284	5.783864116	1.927267	3.00107	0.002690326	0.018531834	ENSG00000273712	6	28315612	28315883
ENST00000615868	774.07765	-1.591640527	0.199524	-7.977208	1.50E-15	3.16E-13	ENSG00000278463	6	26033091	26033618
ENST00000615959	19.623415	4.195235606	1.101258	3.809495	0.000139251	0.001758941	ENSG00000275708	21	8208472	8208652
ENST00000615966	1057.8089	-1.248649509	0.190912	-6.540435	6.13E-11	5.40E-09	ENSG00000276410	6	26043226	26043713
ENST00000616106	25.673201	1.76927422	0.651209	2.716908	0.0065895	0.037191239	ENSG00000278341	16	88708955	88710437
ENST00000616182	1189.6941	-1.429427983	0.183507	-7.789491	6.73E-15	1.29E-12	ENSG00000274641	6	27893424	27893891
ENST00000616428	55.56845	2.161352939	0.468277	4.615541	3.92E-06	8.77E-05	ENSG00000274828	18	79677284	79679364
ENST00000616572	4.3600832	5.550086197	2.004404	2.768946	0.005623788	0.032777457	ENSG00000273998	20	16576067	16579615
ENST00000616721	37.94191	3.070250829	0.602056	5.099613	3.40E-07	1.07E-05	ENSG00000275395	19	39863322	39934626
ENST00000617175	5.326075	-5.886483505	1.913477	-3.076328	0.002095671	0.015227809	ENSG00000276386	9	67059559	67293468
ENST00000617380	61.617495	1.218636267	0.423806	2.87546	0.004034394	0.025403265	ENSG00000276547	5	141397946	141512975
ENST00000617569	537.97043	-1.250682478	0.20548	-6.086643	1.15E-09	7.39E-08	ENSG00000278637	6	26021648	26022050
ENST00000617896	8.7201665	6.5500757	1.749115	3.744795	0.000180541	0.002175212	ENSG00000275329	9	136122520	136124363
ENST00000618052	2308.6972	-1.103413158	0.169941	-6.492937	8.42E-11	7.25E-09	ENSG00000277775	6	26250141	26250635
ENST00000618081	47.749525	1.658885848	0.519948	3.190481	0.001420361	0.011215348	ENSG00000291090	17	18537799	18625620

MIR1244-1	miRNA	Yes	-	108
TNFAIP3	protein_coding	Yes	NM_001270508.2	108
H3C3	protein_coding	Yes	NM_003531.3	108
RNVU1-4	snRNA	Yes	-	108
H2AC16	protein_coding	Yes	NM_003511.3	108
-	lncRNA	Yes	-	108
H3C1	protein_coding	Yes	NM_003529.3	108
LINC01730	lncRNA	Yes	-	108
H2BC6	protein_coding	Yes	NM_003523.3	108
H4C4	protein_coding	Yes	NM_003539.4	108
ACSF3	protein_coding	Yes	NM_001243279.3	108
H3C8	protein_coding	Yes	NM_003534.3	108
-	miRNA	Yes	-	108
HYDIN2	_unprocessed_p	Yes	-	108
MAL2	protein_coding	Yes	NM_052886.3	108
H4C5	protein_coding	Yes	NM_003545.4	108
H4C9	protein_coding	Yes	NM_003495.3	108
FER1L4	ed_unitary_pse	Yes	-	108
SSBP2	protein_coding	Yes	NM_001256732.3	108
-	essed_pseudo	Yes	-	108
H2AC4	protein_coding	Yes	NM_003513.3	108
MIR3648-1	miRNA	Yes	-	108
H2BC3	protein_coding	Yes	NM_021062.3	108
-	lncRNA	Yes	-	108
H2BC17	protein_coding	Yes	NM_003527.4	108
-	lncRNA	Yes	-	108
-	lncRNA	Yes	-	108
FCGBP	protein_coding	Yes	-	108
CNTNAP3P2	cessed_pseud	Yes	-	108
PCDHGB5	protein_coding	Yes	NM_018925.3	108
H4C1	protein_coding	Yes	NM_003538.4	108
-	lncRNA	Yes	-	108
H3C7	protein_coding	Yes	NM_021018.3	108
CCDC144BP	lncRNA	Yes	-	108

ENST00000618302	12.773191	7.101509334	1.666771	4.260639	2.04E-05	0.000354531	ENSG00000284800	X	102360397	102463164
ENST00000618305	500.62819	-2.686339996	0.667976	-4.021611	5.78E-05	0.000848135	ENSG00000275126	6	27873147	27873534
ENST00000618589	86.697488	-2.492172676	0.411892	-6.050551	1.44E-09	8.99E-08	ENSG00000276216	1	145281115	145281462
ENST00000619360	202.28495	1.675137905	0.32628	5.134054	2.84E-07	9.13E-06	ENSG00000146232	6	44258165	44265551
ENST00000619466	2568.783	-1.057826617	0.16936	-6.246021	4.21E-10	3.01E-08	ENSG00000275713	6	26251613	26252075
ENST00000619647	4.6919711	5.652761962	1.971579	2.867124	0.004142211	0.025926118	ENSG00000277235	20	36064242	36064563
ENST00000619654	9.4518171	6.669379199	1.789582	3.72678	0.000193942	0.002308744	ENSG00000274383	15	64950915	64951435
ENST00000619781	6.1370558	6.041079267	1.857764	3.251801	0.001146762	0.009476202	ENSG00000278737	15	65083041	65083663
ENST00000620149	22.314594	-3.013989143	0.782904	-3.849757	0.000118235	0.001533904	ENSG00000196542	3	161344797	161371517
ENST00000620414	85.121936	-1.229080587	0.37383	-3.287807	0.001009709	0.00854616	ENSG00000277534	18	26542970	26545791
ENST00000620793	11.615722	-3.07859033	1.047089	-2.940141	0.003280626	0.021600882	ENSG00000274286	2	96112875	96116571
ENST00000621103	48.331044	1.808393065	0.529215	3.417126	0.00063286	0.005937143	ENSG00000233006	5	132311284	132369916
ENST00000621112	752.25329	-1.636481614	0.195764	-8.359474	6.30E-17	1.59E-14	ENSG00000273703	6	27815021	27815489
ENST00000621139	19.980317	2.768805681	0.815813	3.393923	0.00068899	0.006338485	ENSG00000290399	15	22664678	22686213
ENST00000621141	11.70331	-4.517958647	1.322611	-3.415939	0.000635624	0.005955518	ENSG00000183671	2	206175315	206213371
ENST00000621166	5.5974261	5.909724272	1.89259	3.122559	0.001792859	0.013471947	ENSG00000283836	1	43364647	43364715
ENST00000621281	139.76697	-1.147977331	0.303703	-3.779937	0.000156868	0.001938416	ENSG00000274944	1	38864528	38881602
ENST00000621282	82.977097	-1.680813368	0.379652	-4.427251	9.54E-06	0.00018642	ENSG00000231607	13	49982551	50125541
ENST00000621411	3083.4932	-1.298356366	0.17692	-7.338677	2.16E-13	3.11E-11	ENSG00000286522	6	26031588	26032099
ENST00000621426	44.808334	-1.707371341	0.501447	-3.404892	0.000661903	0.00614791	ENSG00000197183	20	32443058	32585333
ENST00000621525	406.27854	-1.237763195	0.227426	-5.442498	5.25E-08	2.14E-06	ENSG00000258644	14	70326063	70417074
ENST00000621583	12.02582	7.0148215	1.681932	4.170693	3.04E-05	0.000495275	ENSG00000274678	16	30821337	30821884
ENST00000621925	4.1275122	5.470619365	2.029646	2.695356	0.007031337	0.039020136	ENSG00000278472	15	33972058	33972515
ENST00000621997	6.4937728	6.11996677	1.859641	3.29094	0.000998534	0.008474163	ENSG00000277368	13	26221926	26222654
ENST00000621999	1330.1893	-1.183277632	0.249864	-4.735691	2.18E-06	5.33E-05	ENSG00000085365	5	78360616	78480739
ENST00000622447	169.00351	1.387981505	0.295499	4.697076	2.64E-06	6.24E-05	ENSG00000268350	X	52947253	52958518
ENST00000622683	886.55032	-1.854438516	0.209724	-8.84227	9.38E-19	3.03E-16	ENSG00000275832	17	38428463	38512385
ENST00000622694	779.47751	-1.568743552	0.242762	-6.462075	1.03E-10	8.74E-09	ENSG00000015475	22	17734137	17774469
ENST00000622750	60.152443	1.291549915	0.434765	2.970682	0.002971394	0.020004112	ENSG00000277476	17	68133200	68135935
ENST00000622931	58.715564	1.376690593	0.472276	2.915014	0.00355673	0.022996003	ENSG00000280248	17	80339897	80342058
ENST00000623192	20.445927	1.900754067	0.723251	2.62807	0.008587075	0.045370749	ENSG00000278952	11	65118309	65119111
ENST00000623243	6.9183647	6.218405648	1.844871	3.370646	0.000749922	0.006786389	ENSG00000280228	2	112840327	112844195
ENST00000623400	876.10604	2.069565217	0.209371	9.884666	4.85E-23	2.77E-20	ENSG00000172661	10	45727265	45792964
ENST00000623543	21.168691	3.675837478	0.914073	4.021384	5.79E-05	0.000848708	ENSG00000280007	22	18110758	18131154

-	protein_coding	Yes	-	108
H4C13	protein_coding	Yes	NM_003546.3	108
-	lncRNA	Yes	-	108
NFKBIE	protein_coding	Yes	NM_004556.3	108
H2BC9	protein_coding	Yes	NM_003524.3	108
-	lncRNA	Yes	-	108
-	lncRNA	Yes	-	108
-	lncRNA	Yes	-	108
SPTSSB	protein_coding	Yes	NM_001040100.2	108
-	lncRNA	Yes	-	108
ADRA2B	protein_coding	Yes	NM_000682.7	108
MIR3936HG	lncRNA	Yes	-	108
H2BC14	protein_coding	Yes	NM_003521.3	108
-	lncRNA	Yes	-	108
CMKLR2	protein_coding	Yes	NM_001389445.1	108
MIR6734	miRNA	Yes	-	108
-	protein_coding	Yes	-	108
DLEU2	lncRNA	Yes	-	108
H3C2	protein_coding	Yes	NM_003537.4	108
NOL4L	protein_coding	Yes	NM_001256798.2	108
SYNJ2BP-COX16	protein_coding	Yes	-	108
-	lncRNA	Yes	-	108
-	lncRNA	Yes	-	108
-	lncRNA	Yes	-	108
SCAMP1	protein_coding	Yes	NM_004866.6	108
FAM156A	protein_coding	Yes	NM_001387706.1	108
ARHGAP23	protein_coding	Yes	NM_001199417.2	108
BID	protein_coding	Yes	NM_001196.4	108
-	lncRNA	Yes	-	108
-	TEC	Yes	-	108
-	TEC	Yes	-	108
-	TEC	Yes	-	108
WASHC2C	protein_coding	Yes	NM_001330074.2	108
-	lncRNA	Yes	-	108

ENST00000623679	24.454378	-2.185524387	0.673332	-3.245834	0.001171072	0.009623349	ENSG00000279518	8	134783693	134785879
ENST00000623745	4.4594	5.578907621	1.99861	2.791394	0.005248159	0.03111273	ENSG00000279747	16	19503860	19504625
ENST00000623789	9.0023959	6.595307849	1.737616	3.795608	0.000147282	0.001840553	ENSG00000278920	22	31051629	31068327
ENST00000624087	43.160442	-2.083785373	0.523907	-3.977393	6.97E-05	0.000988323	ENSG00000280287	12	132550728	132554947
ENST00000624153	39.26484	2.311791514	0.546595	4.229444	2.34E-05	0.000397254	ENSG00000280330	21	7129102	7130287
ENST00000624171	92.578423	1.778467024	0.39503	4.502102	6.73E-06	0.00013922	ENSG00000280046	17	7858942	7866083
ENST00000624173	18.328648	2.186133543	0.786129	2.780885	0.005421089	0.031888597	ENSG00000279968	6	138725210	138773652
ENST00000624246	12.000809	2.812152357	1.080323	2.603067	0.00923938	0.047880182	ENSG00000279539	19	42242774	42244973
ENST00000624248	3.9197703	5.394631232	2.054307	2.626011	0.008639213	0.045513691	ENSG00000278998	7	155222902	155223851
ENST00000624682	55.1058	-2.386400431	0.481848	-4.952597	7.32E-07	2.07E-05	ENSG00000280206	16	15701236	15702118
ENST00000624705	299.53441	-1.591897023	0.261055	-6.097936	1.07E-09	6.95E-08	ENSG00000279602	17	43360032	43361361
ENST00000624818	6.8347689	6.197357197	1.819129	3.406771	0.000657362	0.006111707	ENSG00000280384	22	46163302	46165347
ENST00000624861	4.3849124	5.55734488	1.998527	2.780721	0.005423835	0.03189308	ENSG00000279853	7	44367139	44369292
ENST00000624924	6.2206516	6.064676285	1.874143	3.235973	0.001212289	0.00989483	ENSG00000280277	6	88964526	88966587
ENST00000625022	5.8548263	5.974190633	1.874526	3.187041	0.001437365	0.011313862	ENSG00000280053	3	126973064	126976426
ENST00000625293	68.152909	1.968516886	0.426917	4.611002	4.01E-06	8.94E-05	ENSG00000213689	3	48466229	48467645
ENST00000626300	11.684823	6.971475369	1.67561	4.160559	3.17E-05	0.000514086	ENSG00000281491	9	34985409	34989379
ENST00000626948	4.5926543	5.625398721	1.981882	2.838412	0.004533861	0.02775253	ENSG00000242808	3	180989769	181791029
ENST00000627116	4.6174835	5.632298037	1.975236	2.851455	0.004351962	0.026886003	ENSG00000228242	3	14148401	14165978
ENST00000627565	6.3447976	6.090084753	1.844875	3.301083	0.000963123	0.008246194	ENSG00000203876	10	109945558	110008381
ENST00000627981	2147.7955	-7.228927363	0.663469	-10.89566	1.21E-27	1.21E-24	ENSG00000281181	21	8437628	8438551
ENST00000628077	89.942629	1.251750752	0.360149	3.47565	0.000509617	0.005003624	ENSG00000110011	11	64230547	64234281
ENST00000629807	44.056342	2.149317261	0.512086	4.197179	2.70E-05	0.000448519	ENSG00000242086	3	195708182	195739964
ENST00000629969	4448.3096	-9.210167968	0.577402	-15.95104	2.80E-57	2.67E-53	ENSG00000281383	21	8254591	8255514
ENST00000630721	371.70141	-1.034040605	0.227495	-4.545328	5.48E-06	0.000117278	ENSG00000198689	X	135985434	136047269
ENST00000633077	61.350539	-3.00618763	0.501939	-5.989155	2.11E-09	1.25E-07	ENSG00000048740	10	11017871	11336675
ENST00000633556	580.23312	-1.127030763	0.205614	-5.481285	4.22E-08	1.76E-06	ENSG00000112297	6	106360716	106572017
ENST00000634942	62.575959	1.692882384	0.459415	3.684866	0.000228823	0.002638461	ENSG00000282851	19	17406103	17419324
ENST00000635120	94.849763	-1.183114207	0.399673	-2.960205	0.003074344	0.02056277	ENSG00000183117	8	2935360	4994914
ENST00000635200	3516.8543	-1.014512941	0.169399	-5.98889	2.11E-09	1.25E-07	ENSG00000282988	6	26196753	26199293
ENST00000635767	364.6413	-1.070537127	0.230739	-4.639603	3.49E-06	7.94E-05	ENSG00000178177	4	17841186	18021755
ENST00000635826	16.695578	-3.992279101	1.009215	-3.955826	7.63E-05	0.001065781	ENSG00000283676	1	148334478	148334554
ENST00000635852	54.023484	9.180996033	1.506658	6.093617	1.10E-09	7.11E-08	ENSG00000283646	3	46416523	46423591
ENST00000636449	61.064737	-1.681801445	0.520088	-3.233686	0.001222036	0.009961054	ENSG00000283498	5	118974585	118974670

-	TEC	Yes	-	108
-	TEC	Yes	-	108
-	lncRNA	Yes	-	108
-	TEC	Yes	-	108
CTBP2P10	essed_pseudog	Yes	-	108
-	TEC	Yes	-	108
CCDC28A-AS1	lncRNA	Yes	-	108
-	TEC	Yes	-	108
-	TEC	Yes	-	108
-	lncRNA	Yes	-	108
-	TEC	Yes	-	108
-	lncRNA	Yes	-	108
-	TEC	Yes	-	108
-	TEC	Yes	-	108
-	TEC	Yes	-	108
TREX1	protein_coding	Yes	NM_033629.6	108
DNAJB5-DT	lncRNA	Yes	-	108
SOX2-OT	lncRNA	Yes	-	108
XPC-AS1	lncRNA	Yes	-	108
ADD3-AS1	lncRNA	Yes	-	108
-	lncRNA	Yes	-	108
DNAJC4	protein_coding	Yes	NM_005528.4	108
MUC20-OT1	lncRNA	Yes	-	108
-	lncRNA	Yes	-	108
SLC9A6	protein_coding	Yes	NM_001379110.1	108
CELF2	protein_coding	Yes	NM_001326342.2	108
CRYBG1	protein_coding	Yes	NM_001371242.2	108
BISPR	lncRNA	Yes	-	108
CSMD1	protein_coding	Yes	NM_033225.6	108
-	protein_coding	Yes	-	108
LCORL	protein_coding	Yes	NM_001394446.1	108
MIR5087	miRNA	Yes	-	108
LINC02009	lncRNA	Yes	-	108
MIR1244-2	miRNA	Yes	-	108

ENST00000636498	5.5974261	5.909724272	1.89259	3.122559	0.001792859	0.013471947	ENSG00000283441	20	34990399	34990472
ENST00000637030	7.0176815	6.236877671	1.814974	3.436345	0.00058962	0.005607074	ENSG00000283409	6	134979337	134979432
ENST00000637412	47.477896	1.92635807	0.490059	3.930871	8.46E-05	0.001160998	ENSG00000283426	3	195701617	195711643
ENST00000638164	21.983329	7.884377382	1.580648	4.988066	6.10E-07	1.77E-05	ENSG00000214725	16	29864307	29865442
ENST00000638170	292.96317	-1.857372681	0.263904	-7.038059	1.95E-12	2.30E-10	ENSG00000259330	15	40323691	40326715
ENST00000638394	99.661377	2.177845359	0.431004	5.052962	4.35E-07	1.32E-05	ENSG00000163637	3	64092235	64225466
ENST00000638749	354.54607	-1.228984497	0.239827	-5.124458	2.98E-07	9.53E-06	ENSG00000283154	3	159273243	159897359
ENST00000639008	6.9928523	6.232322729	1.820135	3.424099	0.000616842	0.005816945	ENSG00000204791	8	144049078	144051203
ENST00000639629	106.38315	1.093712211	0.335527	3.259682	0.001115373	0.009264042	ENSG00000216937	10	32446139	32882864
ENST00000640226	86.323817	-1.100599105	0.36882	-2.984111	0.00284404	0.019337212	ENSG00000284543	1	31506274	31583306
ENST00000640237	45.04245	1.811110841	0.56582	3.20086	0.001370182	0.010895331	ENSG00000214279	10	133453944	133469760
ENST00000641433	84.801259	-1.605644009	0.380429	-4.220613	2.44E-05	0.00041104	ENSG00000262454	16	14302287	14331067
ENST00000641539	471.18062	-1.146192842	0.217748	-5.263849	1.41E-07	5.01E-06	ENSG00000167747	19	50798193	50798910
ENST00000641729	12.287974	2.817466914	0.999265	2.819539	0.004809271	0.029102904	ENSG00000284602	1	25232585	25234775
ENST00000641841	45.218649	2.028153359	0.518283	3.913214	9.11E-05	0.001231862	ENSG00000243896	7	144257662	144264792
ENST00000641844	31.559905	2.559928003	0.625422	4.093119	4.26E-05	0.00065949	ENSG00000177359	12	31112235	31201235
ENST00000641863	259.84399	-1.015340822	0.2488	-4.080956	4.49E-05	0.000689298	ENSG00000196369	1	144887287	145095328
ENST00000642238	5672.3778	1.092026969	0.174504	6.257878	3.90E-10	2.83E-08	ENSG00000132849	1	61742479	62163915
ENST00000642329	4.7912879	5.680042389	2.006949	2.830187	0.004652078	0.028333915	ENSG00000285159	6	158853587	158864502
ENST00000642385	4072.591	-1.063707088	0.164777	-6.455418	1.08E-10	9.09E-09	ENSG00000159921	9	36214440	36258448
ENST00000642399	25.861746	-2.014366695	0.646146	-3.117509	0.001823861	0.013650387	ENSG00000285035	15	30644638	30658868
ENST00000642412	262.01952	-1.174830744	0.260722	-4.506074	6.60E-06	0.000137012	ENSG00000128274	22	42692120	42720870
ENST00000642586	6.7015147	6.166287805	1.837366	3.356048	0.00079065	0.007070983	ENSG00000224478	6	159094092	159116254
ENST00000642889	2578.5569	-1.1419535	0.178712	-6.389896	1.66E-10	1.35E-08	ENSG00000285130	9	69035749	69255187
ENST00000643090	5.5974261	5.909724272	1.89259	3.122559	0.001792859	0.013471947	ENSG00000237773	7	16970319	17299288
ENST00000643273	1252.8162	-1.138355873	0.190049	-5.989792	2.10E-09	1.25E-07	ENSG00000148019	9	78236074	78279690
ENST00000643349	14.011159	-3.092886019	0.989273	-3.126423	0.001769471	0.013327402	ENSG00000284779	11	2131394	2149603
ENST00000643651	6.1122266	6.035898091	1.858312	3.248054	0.001161974	0.009572323	ENSG00000291098	12	31131454	31135627
ENST00000643898	43.751364	3.393224343	0.584147	5.808856	6.29E-09	3.31E-07	ENSG00000111913	6	24804283	24935960
ENST00000643932	54.287401	2.056992823	0.556964	3.693222	0.00022143	0.002569156	ENSG00000137103	9	35829227	35865515
ENST00000644032	13.626492	7.188586805	1.70057	4.227163	2.37E-05	0.000400901	ENSG00000142347	19	8520777	8577442
ENST00000644384	170.19264	1.717087322	0.404113	4.24903	2.15E-05	0.000369702	ENSG00000160183	21	42371889	42396052
ENST00000644823	29.840796	2.663001632	0.649296	4.10137	4.11E-05	0.000639395	ENSG00000119715	14	76376136	76501837
ENST00000645453	61.765817	3.388514547	0.594743	5.697446	1.22E-08	5.87E-07	ENSG00000266714	17	75587799	75626849

MIR499B	miRNA	Yes	-	108
MIR3662	miRNA	Yes	-	108
SMBD1P	ed_unitary_pse	Yes	-	108
CDIPTOSP	ed_unitary_pse	Yes	-	108
INAFM2	protein_coding	Yes	NM_001301268.2	108
PRICKLE2	protein_coding	Yes	NM_198859.4	108
IQCJ-SCHIP1	protein_coding	Yes	NM_014575.4	108
SMPD5	ed_unitary_pse	Yes	-	108
CCDC7	protein_coding	Yes	NM_001395015.1	108
LINC01226	lncRNA	Yes	-	108
SCART1	protein_coding	Yes	NM_001396050.1	108
MIR193BHG	lncRNA	Yes	-	108
C19orf48	ed_unitary_pse	Yes	-	108
-	lncRNA	Yes	-	108
OR2A7	protein_coding	Yes	NM_001005328.2	108
-	_unprocessed_	Yes	-	108
SRGAP2B	protein_coding	Yes	NM_001271870.5	108
PATJ	protein_coding	Yes	NM_001350145.3	108
-	lncRNA	Yes	-	108
GNE	protein_coding	Yes	NM_005476.7	108
-	_unprocessed_	Yes	-	108
A4GALT	protein_coding	Yes	NM_017436.7	108
-	lncRNA	Yes	-	108
-	protein_coding	Yes	-	108
-	lncRNA	Yes	-	108
CEP78	protein_coding	Yes	NM_001330691.3	108
-	protein_coding	Yes	-	108
-	lncRNA	Yes	-	108
RIPOR2	protein_coding	Yes	NM_001286445.3	108
TMEM8B	protein_coding	Yes	NM_001042590.4	108
MYO1F	protein_coding	Yes	NM_012335.4	108
TMPRSS3	protein_coding	Yes	NM_001256317.3	108
ESRRB	protein_coding	Yes	NM_001379180.1	108
MYO15B	protein_coding	Yes	NM_001395058.1	108

ENST00000645495	3981.2809	-1.104006744	0.164847	-6.697167	2.12E-11	2.07E-09	ENSG00000137710	11	110229440	110296614
ENST00000645635	181.94586	-1.115619494	0.275331	-4.051926	5.08E-05	0.000764654	ENSG00000285330	4	109713915	109801999
ENST00000645668	635.33204	-1.816766733	0.202115	-8.988756	2.50E-19	8.76E-17	ENSG00000198860	1	184051729	184074212
ENST00000645805	182.55809	-1.194733425	0.388102	-3.078402	0.002081139	0.015154726	ENSG00000214049	19	15828205	15836136
ENST00000645831	143.5145	-1.033773658	0.305451	-3.384416	0.000713299	0.006518444	ENSG00000054598	6	1609914	1613897
ENST00000646241	1382.2512	1.707267525	0.178683	9.554703	1.24E-21	5.57E-19	ENSG00000064601	20	45891334	45898820
ENST00000646357	107.56094	-1.07327262	0.374192	-2.868239	0.004127639	0.025865375	ENSG00000285382	20	32632182	32743523
ENST00000646449	144.44269	1.080284755	0.301964	3.577528	0.000346859	0.003680388	ENSG00000197728	12	56041917	56044697
ENST00000646932	1068.2297	-1.209579887	0.182697	-6.620676	3.58E-11	3.31E-09	ENSG00000136143	13	47942655	48001273
ENST00000646952	12.174795	7.030694764	1.667021	4.21752	2.47E-05	0.000415841	ENSG00000291251	12	31158896	31172377
ENST00000647029	14.82855	-7.365193609	1.639266	-4.492981	7.02E-06	0.000144146	ENSG00000121742	13	20221961	20232319
ENST00000647043	143.55727	1.10711692	0.31783	3.483367	0.000495149	0.004892458	ENSG00000290854	1	1724837	1745919
ENST00000647468	333.36041	1.176814633	0.239533	4.912948	8.97E-07	2.46E-05	ENSG00000122335	6	158109518	158168262
ENST00000647653	4.1523414	5.47828107	2.024699	2.705726	0.00681552	0.038152509	ENSG00000285612	14	71586268	71590354
ENST00000647725	62.753157	-2.270716105	0.442083	-5.136399	2.80E-07	9.03E-06	ENSG00000285708	3	70959225	71754229
ENST00000647738	14.748366	-3.444410699	1.002551	-3.435646	0.000591142	0.005612796	ENSG00000186487	2	1789112	2331275
ENST00000647856	80.229255	1.782715651	0.406481	4.385724	1.16E-05	0.000219878	ENSG00000285796	20	45926517	45935055
ENST00000647893	54.214967	-3.25401127	0.517047	-6.293453	3.10E-10	2.33E-08	ENSG00000197594	6	131808019	131895155
ENST00000647941	372.24608	-1.140516674	0.255758	-4.459354	8.22E-06	0.000164275	ENSG00000080819	3	98579445	98593611
ENST00000647952	6.037739	6.020211337	1.869475	3.220269	0.001280706	0.010336457	ENSG00000290870	6	29849549	29885615
ENST00000647956	186.6507	1.337819628	0.303517	4.407725	1.04E-05	0.000201585	ENSG00000159403	12	7080218	7092445
ENST00000648076	30.43925	-3.378824719	0.73511	-4.596352	4.30E-06	9.51E-05	ENSG00000065618	10	104031285	104085880
ENST00000648193	10.704881	6.845203891	1.695036	4.038383	5.38E-05	0.000801533	ENSG00000285777	1	173596059	173669837
ENST00000648319	910.33251	-1.005366978	0.210742	-4.770595	1.84E-06	4.58E-05	ENSG00000104783	19	43766532	43780973
ENST00000648736	4.4345708	5.571739292	1.995746	2.791808	0.005241441	0.031089836	ENSG00000262223	17	81375143	81385464
ENST00000648922	175.44796	-1.074945215	0.278324	-3.862209	0.000112366	0.00146789	ENSG00000285547	X	72301810	72572843
ENST00000648936	8.8419697	-4.094019748	1.388385	-2.948763	0.003190482	0.021123569	ENSG00000127990	7	94584979	94656133
ENST00000649150	5.132284	5.783864116	1.927267	3.00107	0.002690326	0.018531834	ENSG00000261645	11	90251203	90915048
ENST00000649169	36.980418	8.63264	1.525612	5.658478	1.53E-08	7.09E-07	ENSG00000268926	9	95772588	95776282
ENST00000649286	174.4387	-2.38142831	0.296547	-8.030513	9.71E-16	2.08E-13	ENSG00000006530	7	141551409	141655244
ENST00000649368	674.5027	-1.336984873	0.206996	-6.458989	1.05E-10	8.90E-09	ENSG00000092758	20	62817049	62841159
ENST00000649488	8.3791704	6.489713504	1.759848	3.687656	0.000226329	0.002614675	ENSG00000108556	17	4897770	4903098
ENST00000649528	126.81981	-2.188096255	0.345846	-6.326793	2.50E-10	1.93E-08	ENSG00000114861	3	70954707	71583728
ENST00000649556	174.32498	1.352184046	0.302833	4.465117	8.00E-06	0.000160798	ENSG00000285952	16	4777938	4801423

RDX	protein_coding	Yes	NM_002906.4	108
-	protein_coding	Yes	-	108
TSEN15	protein_coding	Yes	NM_052965.4	108
UCA1	lncRNA	Yes	-	108
FOXC1	protein_coding	Yes	NM_001453.3	108
CTSA	protein_coding	Yes	NM_000308.4	108
-	protein_coding	Yes	-	108
RPS26	protein_coding	Yes	NM_001029.5	108
SUCLA2	protein_coding	Yes	NM_003850.3	108
-	lncRNA	Yes	-	108
GJB6	protein_coding	Yes	NM_001110219.3	108
-	lncRNA	Yes	-	108
SERAC1	protein_coding	Yes	NM_032861.4	108
-	lncRNA	Yes	-	108
-	protein_coding	Yes	-	108
MYT1L	protein_coding	Yes	NM_001303052.2	108
-	lncRNA	Yes	-	108
ENPP1	protein_coding	Yes	NM_006208.3	108
CPOX	protein_coding	Yes	NM_000097.7	108
-	lncRNA	Yes	-	108
C1R	protein_coding	Yes	NM_001733.7	108
COL17A1	protein_coding	Yes	NM_000494.4	108
-	protein_coding	Yes	-	108
KCNN4	protein_coding	Yes	NM_002250.3	108
-	lncRNA	Yes	-	108
-	protein_coding	Yes	-	108
SGCE	protein_coding	Yes	NM_003919.3	108
DISC1FP1	lncRNA	Yes	-	108
-	lncRNA	Yes	-	108
AGK	protein_coding	Yes	NM_018238.4	108
COL9A3	protein_coding	Yes	NM_001853.4	108
CHRNE	protein_coding	Yes	NM_000080.4	108
FOXP1	protein_coding	Yes	NM_001349338.3	108
-	lncRNA	Yes	-	108

ENST00000649792	32.129218	1.63827848	0.582351	2.813216	0.004904868	0.029569869	ENSG00000175356	11	9019475	9091599
ENST00000649796	87.925018	-1.340438333	0.385536	-3.476821	0.000507397	0.004986004	ENSG00000127955	7	80134830	80226181
ENST00000649979	1522.0876	1.506017201	0.541413	2.781645	0.005408423	0.031832751	ENSG00000115267	2	162267073	162318684
ENST00000650255	5.5725969	5.904038679	1.897053	3.112216	0.001856888	0.013844541	ENSG00000285871	10	68896919	68900768
ENST00000650284	192.73764	-1.419532988	0.290243	-4.89085	1.00E-06	2.70E-05	ENSG00000285645	9	35812959	35828732
ENST00000650340	4.335254	5.542764563	2.013314	2.753055	0.005904191	0.034056399	ENSG00000285712	10	43325832	43350792
ENST00000650446	12.698268	3.187717965	1.036817	3.074524	0.002108389	0.015301253	ENSG00000285877	17	67244281	67273503
ENST00000650711	51.338789	-2.261386575	0.490637	-4.609087	4.04E-06	9.01E-05	ENSG00000108576	17	30194318	30235697
ENST00000650905	183.71151	2.224625047	0.340765	6.528333	6.65E-11	5.81E-09	ENSG00000172936	3	38138660	38143022
ENST00000650932	413.77299	-2.036185427	0.2764	-7.366813	1.75E-13	2.56E-11	ENSG00000276043	19	4909500	4962154
ENST00000650944	30.202916	2.051442096	0.633947	3.235983	0.001212245	0.00989483	ENSG00000286207	2	152635267	152641275
ENST00000651192	83.536991	1.048676555	0.380475	2.756231	0.005847165	0.033832137	ENSG00000164011	1	42846612	42852477
ENST00000651205	11.303277	6.925450708	1.695113	4.08554	4.40E-05	0.000677464	ENSG00000286067	12	120218069	120222668
ENST00000651272	5.1231757	5.775684372	2.083732	2.771799	0.005574752	0.032569995	ENSG00000258216	12	89712047	90100763
ENST00000651636	13.87728	7.219543009	1.642061	4.396636	1.10E-05	0.000210533	ENSG00000267549	19	56315261	56365316
ENST00000651663	39.641633	2.77444925	0.568694	4.878634	1.07E-06	2.85E-05	ENSG00000286042	6	79307668	79313384
ENST00000651671	535.04497	-1.487389403	0.21456	-6.932264	4.14E-12	4.61E-10	ENSG00000148400	9	136494432	136546048
ENST00000651687	96.898795	-2.692030501	0.393129	-6.847694	7.50E-12	7.94E-10	ENSG00000113356	5	90474863	90514557
ENST00000651706	40.263805	-4.311308813	0.713798	-6.03996	1.54E-09	9.48E-08	ENSG00000286231	1	220786989	220884503
ENST00000651735	140.55461	-1.052618269	0.334597	-3.145925	0.001655623	0.01264521	ENSG00000132170	3	12289069	12434344
ENST00000651973	9.6444429	-3.567626758	1.262852	-2.825056	0.004727242	0.028704529	ENSG00000286172	1	147084615	147084756
ENST00000651982	24.744304	-1.753335347	0.661831	-2.64922	0.008067778	0.043307084	ENSG00000178662	2	165469697	165689407
ENST00000652248	961.26679	-1.051205654	0.231673	-4.537446	5.69E-06	0.000120968	ENSG00000286070	22	24556006	24628987
ENST00000652333	4.9742005	5.735936539	1.955082	2.933859	0.003347758	0.021910732	ENSG00000267107	19	41449519	41500644
ENST00000652477	69.740911	1.885860767	0.496579	3.797702	0.000146044	0.001828226	ENSG00000180155	8	142771201	142777202
ENST00000652894	5.6470845	5.921009198	1.889355	3.133879	0.00172512	0.013059518	ENSG00000272690	3	75443563	75538029
ENST00000653014	4.2019998	5.493492792	2.024643	2.713315	0.006661379	0.037489128	ENSG00000287513	1	241640554	241641835
ENST00000654017	4.6083752	5.622775254	2.123133	2.648339	0.008088835	0.043394135	ENSG00000258647	15	92567816	92574529
ENST00000654602	151.20716	2.343530119	0.320565	7.310618	2.66E-13	3.74E-11	ENSG00000233461	1	231520728	231528604
ENST00000654773	4.6423127	5.639148354	1.971416	2.860456	0.004230322	0.026324826	ENSG00000288002	5	8207018	8366437
ENST00000655235	23.095147	3.378293307	0.785402	4.301353	1.70E-05	0.000305107	ENSG00000279726	5	140785697	140828549
ENST00000655618	9.3839422	6.652788571	1.731312	3.842627	0.000121724	0.001572748	ENSG00000237596	6	136053350	136225618
ENST00000655709	22.775751	4.069944873	0.92459	4.401891	1.07E-05	0.00020617	ENSG00000276462	9	41101041	41119909
ENST00000655747	5.9541431	5.995754227	1.878846	3.19119	0.001416881	0.011192507	ENSG00000287676	1	219294981	219297668

SCUBE2	protein_coding	Yes	NM_001367977.2	108
GNAI1	protein_coding	Yes	NM_002069.6	108
IFIH1	protein_coding	Yes	NM_022168.4	108
-	lncRNA	Yes	-	108
-	protein_coding	Yes	-	108
-	lncRNA	Yes	-	108
-	lncRNA	Yes	-	108
SLC6A4	protein_coding	Yes	NM_001045.6	108
MYD88	protein_coding	Yes	NM_002468.5	108
UHRF1	protein_coding	Yes	NM_001048201.3	108
-	lncRNA	Yes	-	108
ZNF691	protein_coding	Yes	NM_001242739.2	108
-	lncRNA	Yes	-	108
-	lncRNA	Yes	-	108
ZSCAN5A-AS1	lncRNA	Yes	-	108
LCAL1	lncRNA	Yes	-	108
NOTCH1	protein_coding	Yes	NM_017617.5	108
POLR3G	protein_coding	Yes	NM_006467.3	108
-	protein_coding	Yes	-	108
PPARG	protein_coding	Yes	NM_138711.6	108
RNVU1-8	snRNA	Yes	-	108
CSRNP3	protein_coding	Yes	NM_001172173.2	108
-	protein_coding	Yes	-	108
PCAT19	lncRNA	Yes	-	108
LYNX1	protein_coding	Yes	NM_177477.4	108
LINC02018	lncRNA	Yes	-	108
-	lncRNA	Yes	-	108
LINC00930	lncRNA	Yes	-	108
-	lncRNA	Yes	-	108
-	lncRNA	Yes	-	108
-	lncRNA	Yes	-	108
-	lncRNA	Yes	-	108
LINC03025	lncRNA	Yes	-	108
-	lncRNA	Yes	-	108

ENST00000655811	7.7650531	6.382432372	1.780947	3.583729	0.000338724	0.003613507	ENSG00000287530	13	23954492	23970188
ENST00000655847	277.55372	1.146070586	0.243047	4.715427	2.41E-06	5.79E-05	ENSG00000203875	6	85650490	85678748
ENST00000656037	3.8949411	5.386514595	2.058112	2.617211	0.008865141	0.04637373	ENSG00000228221	3	177441926	177767379
ENST00000656081	91.820878	1.328265435	0.365241	3.636685	0.000276169	0.003078869	ENSG00000286388	5	1345196	1350786
ENST00000656249	5.3400258	5.842238811	1.912914	3.054104	0.002257337	0.016129535	ENSG00000245293	4	107863667	107978799
ENST00000656264	72.213343	1.77245191	0.465357	3.808803	0.000139641	0.00176226	ENSG00000277701	2	97281355	97291849
ENST00000656384	110.43932	1.818799261	0.351381	5.176145	2.27E-07	7.51E-06	ENSG00000230606	2	97416164	97429862
ENST00000656825	4674.6799	-1.013800609	0.171366	-5.915995	3.30E-09	1.86E-07	ENSG00000070831	1	22052708	22101360
ENST00000656931	4.1275122	5.470619365	2.029646	2.695356	0.007031337	0.039020136	ENSG00000227431	20	49040463	49046168
ENST00000656939	39.752893	2.758869101	0.617889	4.464989	8.01E-06	0.000160854	ENSG00000287126	2	3568504	3575100
ENST00000657329	15.799058	-2.140771747	0.824632	-2.596033	0.009430695	0.048629485	ENSG00000227885	6	53739265	53794904
ENST00000657573	38.848373	1.804991001	0.540519	3.339368	0.000839692	0.007411729	ENSG00000288009	6	89130967	89145985
ENST00000657742	6.4441144	6.109993113	1.848755	3.304923	0.000950025	0.008160641	ENSG00000236404	9	2421604	2622373
ENST00000658424	5.6470845	5.921009198	1.889355	3.133879	0.00172512	0.013059518	ENSG00000288079	3	113259785	113266824
ENST00000658444	69.576166	1.266549876	0.448165	2.826077	0.004712191	0.028629107	ENSG00000244055	7	92460836	92495601
ENST00000658780	25.574541	-2.594466904	0.690887	-3.755268	0.000173156	0.00210336	ENSG00000110427	11	33376107	33674102
ENST00000658933	7.6317988	6.355094261	1.786447	3.557394	0.000374552	0.003915353	ENSG00000255553	11	62049862	62081968
ENST00000659037	4.4345708	5.571739292	1.995746	2.791808	0.005241441	0.031089836	ENSG00000286747	1	247105404	247108929
ENST00000659152	88.227389	-3.462329298	0.428481	-8.08047	6.45E-16	1.43E-13	ENSG00000287190	1	146050440	146052244
ENST00000659528	44.832735	-1.297339765	0.484423	-2.678114	0.007403812	0.040528047	ENSG00000233184	1	101025845	101090513
ENST00000659885	7.8946424	3.850688933	1.447265	2.660667	0.007798613	0.042181646	ENSG00000286692	1	94417742	94418228
ENST00000660029	6.6270271	6.151919684	1.830449	3.36088	0.000776946	0.006976673	ENSG00000259359	15	96171574	96174339
ENST00000660618	171.27029	-1.868815495	0.319498	-5.849225	4.94E-09	2.67E-07	ENSG00000285646	1	11908151	11914298
ENST00000660920	6.0873974	6.030698599	1.860391	3.24163	0.001188481	0.009740153	ENSG00000286431	X	3819923	3823898
ENST00000661181	20.479477	7.780763105	1.582608	4.916417	8.81E-07	2.42E-05	ENSG00000286299	6	125268086	125274828
ENST00000661238	4.8997129	5.716560879	1.947521	2.935301	0.00333224	0.021846691	ENSG00000286507	7	26598599	26617295
ENST00000661266	9.0023959	6.595307849	1.737616	3.795608	0.000147282	0.001840553	ENSG00000288067	8	134190382	134390487
ENST00000661438	3199.35	-1.041758232	0.20695	-5.033876	4.81E-07	1.44E-05	ENSG00000285920	15	41286010	41380996
ENST00000661493	22.412348	-2.87733977	0.744445	-3.86508	0.000111053	0.001454222	ENSG00000245694	16	54852902	54928843
ENST00000661543	1234.6797	2.727469643	0.185341	14.71592	5.09E-49	2.59E-45	ENSG00000077150	10	102395704	102402524
ENST00000661545	3.9445995	5.40270887	2.054252	2.630013	0.008538167	0.045174985	ENSG00000226985	X	13334179	13403019
ENST00000661897	6.8238421	4.703472476	1.797799	2.616239	0.008890432	0.04646775	ENSG00000287078	1	52365442	52367865
ENST00000662121	27.304288	1.784634623	0.672899	2.652158	0.007997913	0.043027529	ENSG00000232931	2	95807117	95833963
ENST00000662437	57.950087	-2.05770616	0.451581	-4.556671	5.20E-06	0.000111909	ENSG00000250682	5	102608478	102671765

-	lncRNA	Yes	-	108
SNHG5	lncRNA	Yes	-	108
LINC00578	lncRNA	Yes	-	108
-	lncRNA	Yes	-	108
CYP2U1-AS1	lncRNA	Yes	-	108
-	lncRNA	Yes	-	108
LINC02969	lncRNA	Yes	-	108
CDC42	protein_coding	Yes	NM_001791.4	108
CSE1L-DT	lncRNA	Yes	-	108
-	lncRNA	Yes	-	108
-	lncRNA	Yes	-	108
-	lncRNA	Yes	-	108
VLDLR-AS1	lncRNA	Yes	-	108
-	lncRNA	Yes	-	108
-	lncRNA	Yes	-	108
KIAA1549L	protein_coding	Yes	NM_012194.3	108
LINC02733	lncRNA	Yes	-	108
-	lncRNA	Yes	-	108
-	lncRNA	Yes	-	108
DPH5-DT	lncRNA	Yes	-	108
-	lncRNA	Yes	-	108
-	lncRNA	Yes	-	108
-	lncRNA	Yes	-	108
-	lncRNA	Yes	-	108
-	lncRNA	Yes	-	108
-	lncRNA	Yes	-	108
-	lncRNA	Yes	-	108
-	protein_coding	Yes	-	108
CRNDE	lncRNA	Yes	-	108
NFKB2	protein_coding	Yes	NM_001322934.2	108
LINC01203	lncRNA	Yes	-	108
-	lncRNA	Yes	-	108
LINC00342	lncRNA	Yes	-	108
LINC00491	lncRNA	Yes	-	108

ENST00000662507	11.402808	2.704288904	1.028406	2.629593	0.008548712	0.045217418	ENSG00000286782	9	35012962	35016076
ENST00000662856	14.037141	3.666749632	1.082331	3.387827	0.000704488	0.006452239	ENSG00000233058	3	194487145	194521154
ENST00000663040	31.276405	2.758627783	0.645717	4.272192	1.94E-05	0.000339832	ENSG00000287245	11	109946580	109975996
ENST00000663232	470.77539	1.905007744	0.227002	8.392015	4.78E-17	1.22E-14	ENSG00000224032	5	112160525	112164818
ENST00000663724	9.5807572	5.206290624	1.705384	3.052856	0.002266746	0.016183867	ENSG00000232618	6	142946405	142957945
ENST00000664962	7.3992278	6.310151505	1.797481	3.510552	0.000447177	0.004516245	ENSG00000179935	20	18786085	18794574
ENST00000665340	17.314768	2.082338137	0.796391	2.614717	0.008930147	0.046632152	ENSG00000228775	7	141704002	141738230
ENST00000665637	80.863899	-1.141571295	0.383565	-2.976211	0.002918336	0.019727083	ENSG00000136237	7	22118235	22357154
ENST00000666136	187.57028	2.278019039	0.293567	7.759789	8.51E-15	1.62E-12	ENSG00000268621	19	46189028	46203083
ENST00000666402	10.798714	3.718823391	1.195315	3.111166	0.001863502	0.013880951	ENSG00000268460	19	46201579	46214838
ENST00000666693	7.299911	6.292863864	1.798459	3.499032	0.000466951	0.00466923	ENSG00000286833	2	132347472	132365130
ENST00000666926	5.4393426	5.865814449	1.909598	3.071753	0.002128058	0.01540293	ENSG00000255197	11	47381508	47409271
ENST00000666966	6.8595981	6.201990018	1.818157	3.411141	0.000646916	0.006038901	ENSG00000287170	19	55116419	55123169
ENST00000667541	7.5167611	6.339450526	1.888263	3.357292	0.0007871	0.007045857	ENSG00000287181	4	88352221	88358423
ENST00000669835	20.271736	7.765768344	1.58364	4.903745	9.40E-07	2.56E-05	ENSG00000287382	4	55938152	55947996
ENST00000670397	148.78167	1.00758949	0.313177	3.217312	0.001293977	0.010426454	ENSG00000231074	6	30286702	30327362
ENST00000670665	61.520664	-1.21014417	0.421811	-2.868924	0.004118711	0.02582656	ENSG00000260289	16	7878798	8112756
ENST00000670794	4.9742005	5.735936539	1.955082	2.933859	0.003347758	0.021910732	ENSG00000254689	8	81841951	81924346
ENST00000671199	96.850566	1.045540413	0.347623	3.007683	0.002632473	0.018235604	ENSG00000223891	20	44210906	44226027
ENST00000673801	646.73676	-1.604974515	0.199542	-8.043289	8.75E-16	1.90E-13	ENSG00000121281	16	50266550	50318135
ENST00000675367	196.31118	1.732007292	0.281512	6.152524	7.63E-10	5.12E-08	ENSG00000129667	17	76470895	76501423
ENST00000675830	16.929992	2.484826447	0.895704	2.77416	0.005534447	0.032394127	ENSG00000290691	15	43699440	43718260
ENST00000677713	4.1523414	5.47828107	2.024699	2.705726	0.00681552	0.038152509	ENSG00000083067	9	70529059	71121621
ENST00000678225	781.4542	-1.139959223	0.209144	-5.450603	5.02E-08	2.06E-06	ENSG00000085982	2	233475525	233566782
ENST00000679262	13.911699	-7.27428437	1.713846	-4.24442	2.19E-05	0.000376236	ENSG00000289754	16	28537039	28620133
ENST00000679887	26.859261	2.825854868	0.714046	3.957525	7.57E-05	0.001060174	ENSG00000087589	20	56412259	56460382
ENST00000680143	128.33877	-1.109595092	0.318645	-3.482225	0.000497265	0.004906289	ENSG00000112787	12	132490150	132585188
ENST00000681320	224.68447	1.280358179	0.316242	4.048663	5.15E-05	0.000773178	ENSG00000114270	3	48564072	48595329
ENST00000682017	2912.8782	-1.061105697	0.184409	-5.754079	8.71E-09	4.42E-07	ENSG00000088387	13	98793428	98978064
ENST00000682079	220.13581	1.186880522	0.346942	3.420978	0.000623963	0.005868122	ENSG00000078018	2	209424046	209734112
ENST00000682384	331.81916	-1.395869514	0.253106	-5.514964	3.49E-08	1.48E-06	ENSG00000162714	1	247297414	247331867
ENST00000682506	177.24	-2.344454419	0.294502	-7.960737	1.71E-15	3.57E-13	ENSG00000160145	3	124033368	124726325
ENST00000682553	392.29613	-1.198966329	0.222416	-5.390647	7.02E-08	2.75E-06	ENSG00000179046	4	188091271	188109603
ENST00000682763	125.61122	-1.959428066	0.346213	-5.659598	1.52E-08	7.05E-07	ENSG00000176438	14	95407265	95516650

-	lncRNA	Yes	-	108
ATP13A3-DT	lncRNA	Yes	-	108
-	lncRNA	Yes	-	108
EPB41L4A-AS1	lncRNA	Yes	-	108
HIVEP2-DT	lncRNA	Yes	-	108
LINC00652	lncRNA	Yes	-	108
WEE2-AS1	lncRNA	Yes	-	108
RAPGEF5	protein_coding	Yes	NM_012294.5	108
IGFL2-AS1	lncRNA	Yes	-	108
-	lncRNA	Yes	-	108
-	lncRNA	Yes	-	108
-	lncRNA	Yes	-	108
-	lncRNA	Yes	-	108
-	lncRNA	Yes	-	108
-	lncRNA	Yes	-	108
HCG18	lncRNA	Yes	-	108
-	lncRNA	Yes	-	108
LINC02235	lncRNA	Yes	-	108
OSER1-DT	lncRNA	Yes	-	108
ADCY7	protein_coding	Yes	NM_001114.5	108
RHBDF2	protein_coding	Yes	NM_001005498.4	108
STRCP1	lncRNA	Yes	-	108
TRPM3	protein_coding	Yes	NM_001366145.2	108
USP40	protein_coding	Yes	NM_001365479.2	108
-	lncRNA	Yes	-	108
CASS4	protein_coding	Yes	NM_020356.4	108
FBRSL1	protein_coding	Yes	NM_001367871.1	108
COL7A1	protein_coding	Yes	NM_000094.4	108
DOCK9	protein_coding	Yes	NM_001366683.2	108
MAP2	protein_coding	Yes	NM_001375505.1	108
ZNF496	protein_coding	Yes	NM_032752.3	108
KALRN	protein_coding	Yes	NM_001388419.1	108
TRIML2	protein_coding	Yes	NM_173553.4	108
SYNE3	protein_coding	Yes	NM_152592.6	108

ENST00000682840	38.677572	-1.517459001	0.524447	-2.893444	0.003810427	0.024283962	ENSG00000150722	2	181985852	182117756
ENST00000682850	28.06284	1.838478583	0.65509	2.806451	0.005009056	0.03005414	ENSG00000229474	15	44665731	44711323
ENST00000682931	114.17465	-1.913327903	0.338044	-5.659989	1.51E-08	7.04E-07	ENSG00000143147	1	168079541	168136930
ENST00000682950	40.277564	2.561117789	0.554548	4.618392	3.87E-06	8.68E-05	ENSG00000140839	16	74408630	74421478
ENST00000683046	140.31197	-1.279736473	0.305416	-4.190143	2.79E-05	0.000460734	ENSG00000186314	5	145756343	145835342
ENST00000683327	8.9215217	5.090527943	1.72808	2.945771	0.00322151	0.021291081	ENSG00000174945	7	2688201	2719683
ENST00000683767	15.369769	3.203207494	0.95561	3.352005	0.000802287	0.007149973	ENSG00000110693	11	15966448	16356546
ENST00000683778	843.3823	1.350243654	0.196991	6.85435	7.16E-12	7.64E-10	ENSG00000197279	6	28080567	28089563
ENST00000683779	237.52482	1.027443886	0.252573	4.067907	4.74E-05	0.00072141	ENSG00000172687	19	21358999	21388582
ENST00000683810	70.090277	1.180954852	0.454003	2.601202	0.009289765	0.048083354	ENSG00000159915	19	44259885	44275317
ENST00000683842	34.006724	-3.40416459	0.653409	-5.20985	1.89E-07	6.43E-06	ENSG00000139973	14	61812702	62112550
ENST00000683975	32.986203	-1.965570921	0.749982	-2.620823	0.008771776	0.046046828	ENSG00000134864	13	100530179	100588789
ENST00000684293	2649.2824	-1.237569833	0.169424	-7.304567	2.78E-13	3.90E-11	ENSG00000091409	2	172427585	172506459
ENST00000684302	7.620872	4.866640122	1.765751	2.75613	0.005848974	0.033841319	ENSG00000111181	12	190080	214157
ENST00000684388	2869.8227	-1.011272679	0.166884	-6.05974	1.36E-09	8.58E-08	ENSG00000288725	16	56430555	56501497
ENST00000684423	50.485449	1.327394736	0.465184	2.853481	0.004324309	0.026755553	ENSG00000078487	7	100400871	100428703
ENST00000684572	294.59326	1.024047256	0.262998	3.893745	9.87E-05	0.001317934	ENSG00000121903	1	33472644	33501643
ENST00000684583	100.96428	2.416601189	0.367706	6.572105	4.96E-11	4.48E-09	ENSG00000063438	5	321713	438285
ENST00000684770	111.52781	-1.418209157	0.367477	-3.85931	0.000113708	0.001482743	ENSG00000170379	7	143620973	143730410
ENST00000684848	216.92863	1.252652087	0.26397	4.745441	2.08E-06	5.11E-05	ENSG00000188385	10	132065945	132184858
ENST00000685041	2186.4382	-1.006102611	0.170019	-5.917593	3.27E-09	1.84E-07	ENSG00000278828	6	27810050	27810536
ENST00000685175	781.10446	1.181130752	0.192531	6.134745	8.53E-10	5.66E-08	ENSG00000092931	17	76738118	76779341
ENST00000685409	40.512622	-1.457543792	0.516635	-2.821227	0.00478404	0.028982446	ENSG00000288924	9	129891880	129893638
ENST00000685419	170.72729	1.24216443	0.326902	3.799802	0.000144812	0.001816982	ENSG00000214021	3	9810261	9836365
ENST00000685586	7.3247402	6.297202409	1.796564	3.505137	0.000456372	0.004584703	ENSG00000288806	3	123701307	123713035
ENST00000685598	6.8347689	6.197357197	1.819129	3.406771	0.000657362	0.006111707	ENSG00000290960	11	3380917	3408981
ENST00000685781	124.05949	1.046050676	0.315442	3.316139	0.000912703	0.007914087	ENSG00000204344	6	31972362	31981446
ENST00000685924	461.25075	-1.124603177	0.214436	-5.244472	1.57E-07	5.49E-06	ENSG00000284906	15	30626128	30773002
ENST00000686237	29.743979	1.671552781	0.601671	2.778183	0.005466375	0.03208564	ENSG00000284713	11	69155934	69162440
ENST00000686413	191.69536	2.352996545	0.342101	6.878081	6.07E-12	6.56E-10	ENSG00000048052	7	18495748	19002416
ENST00000686456	46.436384	1.488918659	0.489861	3.039473	0.002369924	0.016773439	ENSG00000291099	7	5823127	5854439
ENST00000686636	683.58141	-1.052919823	0.199269	-5.283906	1.26E-07	4.55E-06	ENSG00000117114	1	81800933	81993932
ENST00000687221	65.276889	1.39072856	0.418028	3.32688	0.000878241	0.007683465	ENSG00000288826	1	100036631	100038042
ENST00000688159	4.4594	5.578907621	1.99861	2.791394	0.005248159	0.03111273	ENSG00000289141	1	161389546	161389965

PPP1R1C	protein_coding	Yes	NM_001080545.3	108
PATL2	protein_coding	Yes	NM_001387263.1	108
GPR161	protein_coding	Yes	NM_001375883.1	108
CLEC18B	protein_coding	Yes	NM_001385193.1	108
PRELID2	protein_coding	Yes	NM_205846.3	108
AMZ1	protein_coding	Yes	NM_001384743.1	108
SOX6	protein_coding	Yes	NM_001367873.1	108
ZNF165	protein_coding	Yes	NM_001376491.1	108
ZNF738	protein_coding	Yes	NM_001355237.2	108
ZNF233	protein_coding	Yes	NM_001207005.2	108
SYT16	protein_coding	Yes	NM_001367656.1	108
GGACT	protein_coding	Yes	NM_001195087.2	108
ITGA6	protein_coding	Yes	NM_000210.4	108
SLC6A12	protein_coding	Yes	NM_001122848.3	108
-	protein_coding	Yes	-	108
ZCWPW1	protein_coding	Yes	NM_001386010.1	108
ZSCAN20	protein_coding	Yes	NM_001377376.1	108
AHRR	protein_coding	Yes	NM_001377236.1	108
TCAF2	protein_coding	Yes	NM_001363538.2	108
JAKMIP3	protein_coding	Yes	NM_001323087.2	108
H3C10	protein_coding	Yes	NM_003536.3	108
MFSD11	protein_coding	Yes	NM_001242532.5	108
-	lncRNA	Yes	-	108
TTLL3	protein_coding	Yes	NM_001387446.1	108
-	lncRNA	Yes	-	108
-	lncRNA	Yes	-	108
STK19	protein_coding	Yes	NM_004197.2	108
-	protein_coding	Yes	-	108
SMIM38	protein_coding	Yes	NM_001369201.2	108
HDAC9	protein_coding	Yes	NM_178425.4	108
-	lncRNA	Yes	-	108
ADGRL2	protein_coding	Yes	NM_001366006.2	108
-	lncRNA	Yes	-	108
-	lncRNA	Yes	-	108

ENST00000688186	26.647342	-1.743084036	0.627519	-2.777741	0.005473819	0.032118201	ENSG00000289357	8	25508778	25509735
ENST00000688192	62.387441	1.081731573	0.417644	2.590083	0.009595283	0.049271474	ENSG00000290921	10	45862608	45972422
ENST00000688475	27.929045	2.909487556	0.680284	4.276871	1.90E-05	0.000334156	ENSG00000271133	7	20328288	20331781
ENST00000688547	156.95276	1.340185862	0.365163	3.6701	0.000242456	0.002765189	ENSG00000140853	16	56989556	57083520
ENST00000688554	10.505812	2.882110112	1.0967	2.627984	0.008589264	0.0453776	ENSG00000144130	2	112721019	112739095
ENST00000688720	31.113159	-1.710308157	0.617449	-2.769958	0.005606358	0.032702153	ENSG00000196562	20	47657405	47785481
ENST00000688817	70.909199	-1.22226768	0.395694	-3.088923	0.002008835	0.014719639	ENSG00000276170	17	38450393	38452439
ENST00000689186	11.964799	2.798458181	1.030605	2.715354	0.006620497	0.037324723	ENSG00000289100	6	33299478	33301090
ENST00000689319	8.2550244	6.470616567	1.762642	3.670977	0.000241625	0.00275915	ENSG00000288892	5	141039992	141040905
ENST00000689428	4.6423127	5.639148354	1.971416	2.860456	0.004230322	0.026324826	ENSG00000289410	2	61728807	61764201
ENST00000689635	251.71931	-4.873181824	0.34932	-13.95049	3.12E-44	1.06E-40	ENSG00000123094	12	25958681	26072869
ENST00000689782	17.422116	7.544469873	1.621545	4.652644	3.28E-06	7.51E-05	ENSG00000215039	12	6439160	6451568
ENST00000690982	1619.7326	-1.848235902	0.197635	-9.351776	8.62E-21	3.41E-18	ENSG00000164023	4	107824931	107915047
ENST00000691001	12.174795	7.030694764	1.667021	4.21752	2.47E-05	0.000415841	ENSG00000228063	1	218976671	219173768
ENST00000691431	17.754944	-2.990252639	0.866754	-3.449944	0.000560703	0.005396053	ENSG00000289145	8	17226028	17228085
ENST00000691602	4.6174835	5.632298037	1.975236	2.851455	0.004351962	0.026886003	ENSG00000289460	3	33275246	33277376
ENST00000691638	22.011928	2.363096477	0.761252	3.104224	0.00190779	0.014141732	ENSG00000188766	19	38388696	38399587
ENST00000691676	313.32936	3.651018058	0.337422	10.82033	2.76E-27	2.64E-24	ENSG00000182261	11	7957536	7965447
ENST00000691740	18.214272	2.785630244	0.874254	3.186294	0.001441079	0.011335483	ENSG00000289291	1	28736043	28737670
ENST00000692248	546.16118	-1.52890835	0.233549	-6.546418	5.89E-11	5.20E-09	ENSG00000288864	15	32615447	32697095
ENST00000692281	2281.442	-1.077276514	0.171355	-6.286794	3.24E-10	2.41E-08	ENSG00000289027	7	92112175	92245924
ENST00000692306	67.044418	1.134877147	0.408239	2.779933	0.00543701	0.031948961	ENSG00000229931	6	16761723	16763947
ENST00000692570	137.63732	-1.141062999	0.314152	-3.632202	0.000281013	0.003121449	ENSG00000289194	11	62153729	62154783
ENST00000692904	6.9431939	6.223094443	1.834798	3.391705	0.000694591	0.006379523	ENSG00000289446	3	196857590	196867801
ENST00000693075	62.533114	1.287109618	0.423557	3.038812	0.002375126	0.016803693	ENSG00000169955	16	30530366	30534858
ENST00000693695	6.9431939	6.223094443	1.834798	3.391705	0.000694591	0.006379523	ENSG00000289083	15	30427079	30470946
ENST00000693711	199.38123	-1.562661632	0.271845	-5.748345	9.01E-09	4.54E-07	ENSG00000285077	15	30625965	30649529
ENST00000694912	4431.0185	-1.188590351	0.183013	-6.494578	8.33E-11	7.18E-09	ENSG00000153310	8	129839592	130016560
ENST00000695795	379.78094	1.425928333	0.223455	6.381284	1.76E-10	1.42E-08	ENSG00000178104	1	148844427	149033016
ENST00000695919	87.491188	-2.165690196	0.519139	-4.1717	3.02E-05	0.000493354	ENSG00000169851	4	30720368	31146800
ENST00000695948	66.666664	-3.662366734	0.502697	-7.285433	3.21E-13	4.41E-11	ENSG00000124496	6	42224930	42451926
ENST00000697736	28.997083	2.419657837	0.635897	3.805107	0.000141743	0.001785681	ENSG00000196381	19	37668578	37676393
ENST00000697990	1140.4271	1.81244242	0.183896	9.855815	6.47E-23	3.65E-20	ENSG00000289738	10	70815988	70958701
ENST00000698129	150.34483	1.010553822	0.377569	2.676478	0.007440056	0.040686988	ENSG00000289740	11	65499311	65507432

-	lncRNA	Yes	-	108
-	lncRNA	Yes	-	108
ITGB8-AS1	lncRNA	Yes	-	108
NLRC5	protein_coding	Yes	NM_001384950.1	108
NT5DC4	protein_coding	Yes	NM_001393655.1	108
SULF2	protein_coding	Yes	NM_001387048.1	108
-	lncRNA	Yes	-	108
-	lncRNA	Yes	-	108
-	lncRNA	Yes	-	108
-	lncRNA	Yes	-	108
RASSF8	protein_coding	Yes	NM_001394098.1	108
CD27-AS1	lncRNA	Yes	-	108
SGMS2	protein_coding	Yes	NM_001375905.1	108
LYPLAL1-DT	lncRNA	Yes	-	108
-	lncRNA	Yes	-	108
-	lncRNA	Yes	-	108
SPRED3	protein_coding	Yes	NM_001394336.1	108
NLRP10	protein_coding	Yes	NM_001391958.1	108
-	lncRNA	Yes	-	108
ARHGAP11A-SCG5	protein_coding	Yes	-	108
-	protein_coding	Yes	-	108
ATXN1-AS1	lncRNA	Yes	-	108
-	lncRNA	Yes	-	108
-	lncRNA	Yes	-	108
ZNF747	protein_coding	Yes	NM_001305018.2	108
LINC02256	lncRNA	Yes	-	108
ARHGAP11B	protein_coding	Yes	-	108
CYRIB	protein_coding	Yes	NM_001353258.2	108
PDE4DIP	protein_coding	Yes	NM_001395426.1	108
PCDH7	protein_coding	Yes	NM_001173523.2	108
TRERF1	protein_coding	Yes	NM_001395490.1	108
ZNF781	unprocessed_p	Yes	-	108
-	lncRNA	Yes	-	108
TALAM1	lncRNA	Yes	-	108

ENST00000698562	90.377752	1.283297092	0.35936	3.571058	0.000355542	0.003756308	ENSG00000289748	19	37696862	37697402
ENST00000698857	457.13774	-2.97928047	0.250161	-11.90943	1.06E-32	1.53E-29	ENSG00000065882	4	37891083	38139173
ENST00000699294	166.70156	1.734804563	0.343065	5.056776	4.26E-07	1.30E-05	ENSG00000180881	12	75275978	75330324
ENST00000701183	9.8436007	3.573714563	1.223588	2.920684	0.003492638	0.022664301	ENSG00000287865	7	155203670	155215013
ENST00000701218	55.829253	1.622129056	0.448406	3.617543	0.000297413	0.003268133	ENSG00000204682	10	21490271	21497071
ENST00000701348	10.804198	6.857016357	1.693199	4.04974	5.13E-05	0.000770616	ENSG00000289885	3	153161418	153162036
ENST00000701580	25.120715	-6.662652304	1.569048	-4.246302	2.17E-05	0.000373386	ENSG00000224307	9	129282509	129313220
ENST00000702066	4.335254	5.542764563	2.013314	2.753055	0.005904191	0.034056399	ENSG00000281655	11	102641077	102683911
ENST00000702614	11.301261	-3.382148256	1.127936	-2.998528	0.002712874	0.018651741	ENSG00000290039	17	58659124	58659306
ENST00000702711	313.83509	1.899454475	0.258798	7.339537	2.14E-13	3.09E-11	ENSG00000290058	19	32693040	32693529
ENST00000702873	33.060091	2.457540302	0.613164	4.007964	6.12E-05	0.000887547	ENSG00000290082	17	29567822	29568533
ENST00000702916	41.582181	8.802912425	1.518663	5.796487	6.77E-09	3.55E-07	ENSG00000269834	19	52388835	52397713
ENST00000703413	5.1074548	5.777669537	1.930343	2.993079	0.00276178	0.018912054	ENSG00000290163	X	119815163	119815307

-	d_processed_ps	Yes	-	108
TBC1D1	protein_coding	Yes	NM_001396959.1	108
CAPS2	protein_coding	Yes	NM_001355024.4	108
-	lncRNA	Yes	-	108
MIR1915HG	lncRNA	Yes	-	108
-	lncRNA	Yes	-	108
LINC02975	lncRNA	Yes	-	108
-	lncRNA	Yes	-	108
-	lncRNA	Yes	-	108
-	lncRNA	Yes	-	108
-	lncRNA	Yes	-	108
ZNF528-AS1	lncRNA	Yes	-	108
-	ocessed_pseudo	Yes	-	108
