# Supplementary material for: Combined loss of CDH1 and downstream regulatory sequences drive early-onset diffuse gastric cancer and increase penetrance of hereditary diffuse gastric cancer
Source: Gastric Cancer. 2023 May 30;26(5):653–66. doi: 10.1007/s10120-023-01395-0 (PMC10361908; doi:10.1007/s10120-023-01395-0)
Supplement: Supplementary file 8 — Supplementary file8 (PDF 397 KB) [file 10120_2023_1395_MOESM8_ESM.pdf]

**Supplementary table 7.** Differentially expressed genes in CDH1-TANGO6 del vs CDH1 del

| Transcript.stable.ID | baseMean | log2FoldChange | lfcSE | stat | pvalue | padj | Gene.stable.ID | chr | start | end |
|---|---|---|---|---|---|---|---|---|---|---|
| ENST00000008527 | 318.35016 | -1.008032365 | 0.218062 | -4.62269 | 3.79E-06 | 9.29E-05 | ENSG00000008405 | 12 | 106991363 | 107093549 |
| ENST00000009530 | 5.55650472 | -5.924850703 | 1.904995 | -3.11017 | 0.001869821 | 0.016103119 | ENSG00000019582 | 5 | 150401638 | 150412910 |
| ENST00000035307 | 673.058945 | -1.628983675 | 0.160751 | -10.1336 | 3.92E-24 | 1.31E-21 | ENSG00000033100 | 7 | 151232482 | 151238822 |
| ENST00000064780 | 138.115755 | -1.151963521 | 0.306359 | -3.76017 | 0.000169798 | 0.002283272 | ENSG00000054967 | 11 | 73376398 | 73397474 |
| ENST00000155926 | 241.974466 | -1.878460019 | 0.260248 | -7.21796 | 5.28E-13 | 5.41E-11 | ENSG00000071575 | 2 | 12716935 | 12742734 |
| ENST00000194155 | 262.805655 | 1.214433144 | 0.220173 | 5.515806 | 3.47E-08 | 1.50E-06 | ENSG00000112852 | 5 | 141094614 | 141098703 |
| ENST00000201647 | 857.094384 | -1.626056103 | 0.153166 | -10.6163 | 2.50E-26 | 1.02E-23 | ENSG00000131037 | 19 | 55075868 | 55087923 |
| ENST00000202917 | 399.653973 | 5.338348049 | 0.644163 | 8.287265 | 1.16E-16 | 1.85E-14 | ENSG00000089127 | 12 | 112906961 | 112919903 |
| ENST00000204679 | 732.010102 | -1.023288952 | 0.155466 | -6.58206 | 4.64E-11 | 3.42E-09 | ENSG00000090581 | 16 | 1351930 | 1364113 |
| ENST00000215912 | 37.2207834 | 1.593366482 | 0.528196 | 3.016617 | 0.002556123 | 0.020533081 | ENSG00000100100 | 22 | 31281599 | 31292488 |
| ENST00000216714 | 1220.20183 | 1.469233211 | 0.145651 | 10.08738 | 6.28E-24 | 2.06E-21 | ENSG00000100823 | 14 | 20455225 | 20457767 |
| ENST00000216832 | 966.601222 | 1.007569144 | 0.146701 | 6.868199 | 6.50E-12 | 5.71E-10 | ENSG00000100941 | 14 | 39175253 | 39183220 |
| ENST00000217131 | 49.970069 | 1.997090515 | 0.473577 | 4.21703 | 2.48E-05 | 0.000456233 | ENSG00000101160 | 20 | 58995184 | 59007254 |
| ENST00000217159 | 217.525367 | -1.923973135 | 0.272398 | -7.0631 | 1.63E-12 | 1.57E-10 | ENSG00000101187 | 20 | 62642502 | 62672293 |
| ENST00000217182 | 197.483174 | -4.7200027 | 0.36838 | -12.8129 | 1.39E-37 | 1.75E-34 | ENSG00000101210 | 20 | 63488013 | 63499083 |
| ENST00000217909 | 151.854597 | 1.922961003 | 0.286057 | 6.722307 | 1.79E-11 | 1.43E-09 | ENSG00000077713 | X | 119399335 | 119454478 |
| ENST00000219150 | 31.8847532 | -2.271888185 | 0.592308 | -3.83565 | 0.000125231 | 0.001772957 | ENSG00000102879 | 16 | 30183601 | 30189076 |
| ENST00000219207 | 8.4986465 | 5.04143172 | 1.736388 | 2.903401 | 0.003691331 | 0.027286602 | ENSG00000102934 | 16 | 57256096 | 57284672 |
| ENST00000219299 | 98.763051 | 1.860382577 | 0.338891 | 5.489627 | 4.03E-08 | 1.71E-06 | ENSG00000103021 | 16 | 58249935 | 58283836 |
| ENST00000220509 | 1038.60731 | -1.115924582 | 0.165815 | -6.72995 | 1.70E-11 | 1.36E-09 | ENSG00000104142 | 15 | 40894449 | 40903975 |
| ENST00000220616 | 73.1630612 | -1.397195274 | 0.37947 | -3.68197 | 0.000231443 | 0.002973323 | ENSG00000042832 | 8 | 132866957 | 133134899 |
| ENST00000221418 | 131.482654 | 1.564706294 | 0.299468 | 5.224945 | 1.74E-07 | 6.33E-06 | ENSG00000104823 | 19 | 38815421 | 38831794 |
| ENST00000221466 | 186.531081 | -4.315995526 | 0.337232 | -12.7983 | 1.68E-37 | 2.03E-34 | ENSG00000104870 | 19 | 49512660 | 49526428 |
| ENST00000221980 | 127.444333 | -2.184224674 | 0.321394 | -6.79609 | 1.07E-11 | 9.02E-10 | ENSG00000105376 | 19 | 10289951 | 10296778 |
| ENST00000222120 | 149.590063 | 1.283206158 | 0.282101 | 4.548747 | 5.40E-06 | 0.000125015 | ENSG00000105514 | 19 | 11322067 | 11339657 |
| ENST00000222122 | 355.197786 | -1.498926219 | 0.225884 | -6.63583 | 3.23E-11 | 2.46E-09 | ENSG00000105516 | 19 | 48630029 | 48637379 |
| ENST00000222248 | 14.1980249 | -2.426978058 | 0.893632 | -2.71586 | 0.006610425 | 0.042812947 | ENSG00000105641 | 19 | 17871944 | 17895174 |
| ENST00000222374 | 251.819412 | -2.543920948 | 0.248244 | -10.2477 | 1.21E-24 | 4.22E-22 | ENSG00000105767 | 19 | 43622367 | 43639850 |
| ENST00000222800 | 161.195468 | -1.33018332 | 0.272496 | -4.88148 | 1.05E-06 | 3.10E-05 | ENSG00000106077 | 7 | 73736093 | 73738802 |
| ENST00000222982 | 11.2369187 | -3.827829358 | 1.189605 | -3.21773 | 0.001292091 | 0.012109701 | ENSG00000106258 | 7 | 99648193 | 99679996 |
| ENST00000223076 | 7.0802514 | -4.791201019 | 1.788894 | -2.6783 | 0.007399609 | 0.046667752 | ENSG00000184414 | 7 | 100570130 | 100571136 |
| ENST00000223095 | 32.9380584 | -3.098683142 | 0.663651 | -4.66915 | 3.02E-06 | 7.65E-05 | ENSG00000106366 | 7 | 101127103 | 101139247 |
| ENST00000223136 | 301.886048 | -1.005084135 | 0.207597 | -4.84151 | 1.29E-06 | 3.69E-05 | ENSG00000214253 | 7 | 101239471 | 101245081 |
| ENST00000223145 | 182.440046 | 1.235027925 | 0.261683 | 4.71955 | 2.36E-06 | 6.19E-05 | ENSG00000106415 | 7 | 7968795 | 8089080 |
| ENST00000223271 | 114.928255 | -2.02880175 | 0.353894 | -5.73279 | 9.88E-09 | 4.69E-07 | ENSG00000106538 | 7 | 150338328 | 150341629 |
| ENST00000223368 | 166.998796 | -1.171343954 | 0.275348 | -4.25405 | 2.10E-05 | 0.000397202 | ENSG00000106635 | 7 | 73536355 | 73557690 |
| ENST00000223398 | 55.6386626 | 1.488759908 | 0.442351 | 3.365565 | 0.000763871 | 0.007883407 | ENSG00000106665 | 7 | 74289406 | 74405935 |

| Gene.name | Gene.type | Canonical | MANE.Select | ensembl.version |
|---|---|---|---|---|
| CRY1 | protein_coding | Yes | NM_004075.5 | 108 |
| CD74 | protein_coding | Yes | NM_001025159.3 | 108 |
| CHPF2 | protein_coding | Yes | NM_019015.3 | 108 |
| RELT | protein_coding | Yes | NM_152222.2 | 108 |
| TRIB2 | protein_coding | Yes | NM_021643.4 | 108 |
| PCDHB2 | protein_coding | Yes | NM_018936.4 | 108 |
| EPS8L1 | protein_coding | Yes | NM_133180.3 | 108 |
| OAS1 | protein_coding | Yes | NM_016816.4 | 108 |
| GNPTG | protein_coding | Yes | NM_032520.5 | 108 |
| PIK3IP1 | protein_coding | Yes | NM_052880.5 | 108 |
| APEX1 | protein_coding | Yes | NM_001641.4 | 108 |
| PNN | protein_coding | Yes | NM_002687.4 | 108 |
| CTSZ | protein_coding | Yes | NM_001336.4 | 108 |
| SLCO4A1 | protein_coding | Yes | NM_016354.4 | 108 |
| EEF1A2 | protein_coding | Yes | NM_001958.5 | 108 |
| SLC25A43 | protein_coding | Yes | NM_145305.3 | 108 |
| CORO1A | protein_coding | Yes | NM_007074.4 | 108 |
| PLLP | protein_coding | Yes | NM_015993.3 | 108 |
| CCDC113 | protein_coding | Yes | NM_014157.4 | 108 |
| VPS18 | protein_coding | Yes | NM_020857.3 | 108 |
| TG | protein_coding | Yes | NM_003235.5 | 108 |
| ECH1 | protein_coding | Yes | NM_001398.3 | 108 |
| FCGRT | protein_coding | Yes | NM_001136019.3 | 108 |
| ICAM5 | protein_coding | Yes | NM_003259.4 | 108 |
| RAB3D | protein_coding | Yes | NM_004283.4 | 108 |
| DBP | protein_coding | Yes | NM_001352.5 | 108 |
| SLC5A5 | protein_coding | Yes | NM_000453.3 | 108 |
| CADM4 | protein_coding | Yes | NM_145296.2 | 108 |
| ABHD11 | protein_coding | Yes | NM_148912.4 | 108 |
| CYP3A5 | protein_coding | Yes | NM_000777.5 | 108 |
| IRS3P | essed_pseudog | Yes | - | 108 |
| SERPINE1 | protein_coding | Yes | NM_000602.5 | 108 |
| FIS1 | protein_coding | Yes | NM_016068.3 | 108 |
| GLCCI1 | protein_coding | Yes | NM_138426.4 | 108 |
| RARRES2 | protein_coding | Yes | NM_002889.4 | 108 |
| BCL7B | protein_coding | Yes | NM_001707.4 | 108 |
| CLIP2 | protein_coding | Yes | NM_003388.5 | 108 |

| | | | | | | | | | | |
|---|---|---|---|---|---|---|---|---|---|---|
| ENST00000224949 | 952.340497 | 1.313045081 | 0.150789 | 8.707828 | 3.10E-18 | 5.96E-16 | ENSG00000107959 | 10 | 3137727 | 3172782 |
| ENST00000225792 | 7553.48414 | 1.121146128 | 0.117636 | 9.530648 | 1.56E-21 | 4.17E-19 | ENSG00000108654 | 17 | 64498253 | 64506289 |
| ENST00000227155 | 382.152461 | -1.381631104 | 0.216159 | -6.39174 | 1.64E-10 | 1.09E-08 | ENSG00000085117 | 11 | 44565662 | 44620358 |
| ENST00000227880 | 36.1283413 | 3.319446892 | 0.655427 | 5.064558 | 4.09E-07 | 1.35E-05 | ENSG00000110446 | 11 | 60937083 | 60952176 |
| ENST00000228865 | 365.819802 | 1.113721583 | 0.20692 | 5.382374 | 7.35E-08 | 2.97E-06 | ENSG00000111269 | 12 | 12611875 | 12645108 |
| ENST00000228928 | 1957.76552 | 1.612684481 | 0.372002 | 4.335153 | 1.46E-05 | 0.000292656 | ENSG00000111331 | 12 | 112938473 | 112973251 |
| ENST00000229264 | 16.6206541 | -6.047782775 | 1.590494 | -3.80246 | 0.000143269 | 0.001977206 | ENSG00000111664 | 12 | 6840924 | 6847393 |
| ENST00000229277 | 149.553696 | 1.16973306 | 0.275689 | 4.242939 | 2.21E-05 | 0.000413971 | ENSG00000111674 | 12 | 6914579 | 6923697 |
| ENST00000229708 | 101.732816 | 1.016004604 | 0.322281 | 3.15254 | 0.001618566 | 0.014441813 | ENSG00000111981 | 6 | 149963942 | 149973715 |
| ENST00000231484 | 7.2444086 | -6.309363331 | 1.80773 | -3.49021 | 0.000482634 | 0.0054245 | ENSG00000113555 | 5 | 141943580 | 141958202 |
| ENST00000232975 | 137.529055 | -1.330091955 | 0.28995 | -4.58732 | 4.49E-06 | 0.000107027 | ENSG00000114854 | 3 | 52451099 | 52454041 |
| ENST00000233057 | 2274.09061 | 1.044379444 | 0.174761 | 5.97605 | 2.29E-09 | 1.23E-07 | ENSG00000055332 | 2 | 37099209 | 37156980 |
| ENST00000233948 | 615.323432 | -1.306148204 | 0.163616 | -7.98302 | 1.43E-15 | 2.00E-13 | ENSG00000115596 | 2 | 218859804 | 218874233 |
| ENST00000234179 | 584.116686 | -1.462866405 | 0.166492 | -8.78641 | 1.54E-18 | 3.11E-16 | ENSG00000115825 | 2 | 37250501 | 37324833 |
| ENST00000236147 | 4.538799 | 5.614285089 | 2.00097 | 2.805781 | 0.005019472 | 0.034639173 | ENSG00000188404 | 1 | 169690666 | 169711620 |
| ENST00000237353 | 51.997586 | 1.377657214 | 0.484402 | 2.844039 | 0.004454563 | 0.031611236 | ENSG00000118557 | 16 | 72119096 | 72172155 |
| ENST00000238721 | 129.648926 | 1.160781801 | 0.294231 | 3.945134 | 7.98E-05 | 0.001218757 | ENSG00000115129 | 2 | 24077432 | 24084834 |
| ENST00000238936 | 138.181097 | -1.00722685 | 0.291008 | -3.46116 | 0.00053785 | 0.005914274 | ENSG00000138111 | 10 | 102461394 | 102477045 |
| ENST00000239243 | 186.289409 | -1.202513794 | 0.255928 | -4.69865 | 2.62E-06 | 6.77E-05 | ENSG00000120149 | 5 | 174724581 | 174730896 |
| ENST00000239444 | 10.7533658 | 5.388951677 | 1.678617 | 3.210353 | 0.001325721 | 0.012354789 | ENSG00000120322 | 5 | 141177789 | 141180539 |
| ENST00000239449 | 68.1849706 | 2.746380332 | 0.456849 | 6.011574 | 1.84E-09 | 1.01E-07 | ENSG00000120327 | 5 | 141223342 | 141227759 |
| ENST00000239938 | 69.085502 | 1.229338893 | 0.393506 | 3.124064 | 0.001783714 | 0.01554397 | ENSG00000120738 | 5 | 138465478 | 138469303 |
| ENST00000240100 | 273.320956 | -1.775452055 | 0.255771 | -6.94156 | 3.88E-12 | 3.55E-10 | ENSG00000120875 | 8 | 29333063 | 29350684 |
| ENST00000241052 | 440.181731 | 1.160935079 | 0.211925 | 5.478049 | 4.30E-08 | 1.82E-06 | ENSG00000121691 | 11 | 34438933 | 34472060 |
| ENST00000241261 | 39.2325016 | 3.449766536 | 0.648599 | 5.318799 | 1.04E-07 | 4.05E-06 | ENSG00000121858 | 3 | 172505507 | 172523430 |
| ENST00000241463 | 140.793881 | -1.001719732 | 0.293623 | -3.41158 | 0.000645866 | 0.006875417 | ENSG00000122035 | 13 | 27270829 | 27275192 |
| ENST00000242577 | 891.53639 | 1.16028536 | 0.151661 | 7.650508 | 2.00E-14 | 2.46E-12 | ENSG00000088986 | 12 | 120496112 | 120498493 |
| ENST00000242728 | 24.0149374 | -3.931819348 | 0.857707 | -4.5841 | 4.56E-06 | 0.000108326 | ENSG00000123095 | 12 | 26120029 | 26125037 |
| ENST00000243167 | 82.5274294 | 2.164975262 | 0.375387 | 5.767318 | 8.05E-09 | 3.90E-07 | ENSG00000117480 | 1 | 46394316 | 46413845 |
| ENST00000243349 | 64.064623 | 2.062681013 | 0.438696 | 4.701847 | 2.58E-06 | 6.67E-05 | ENSG00000123612 | 2 | 157526766 | 157628864 |
| ENST00000243776 | 1659.31394 | -1.073041057 | 0.129244 | -8.30242 | 1.02E-16 | 1.65E-14 | ENSG00000123989 | 2 | 219538953 | 219543809 |
| ENST00000245105 | 72.8628049 | -2.358746576 | 0.409243 | -5.76368 | 8.23E-09 | 3.97E-07 | ENSG00000125089 | 4 | 8199289 | 8241103 |
| ENST00000245157 | 499.071028 | 1.18691153 | 0.175696 | 6.75548 | 1.42E-11 | 1.16E-09 | ENSG00000125124 | 16 | 56484384 | 56520024 |
| ENST00000245185 | 58.7024319 | 1.602264162 | 0.558556 | 2.868582 | 0.004123159 | 0.029736916 | ENSG00000125148 | 16 | 56608583 | 56609497 |
| ENST00000245451 | 280.901358 | -2.58356702 | 0.260068 | -9.93421 | 2.96E-23 | 9.11E-21 | ENSG00000125378 | 14 | 53949735 | 53956825 |
| ENST00000245817 | 223.821205 | -2.014588846 | 0.242382 | -8.31164 | 9.44E-17 | 1.54E-14 | ENSG00000125657 | 19 | 6531025 | 6535924 |
| ENST00000245907 | 97.52075 | -4.986829805 | 0.534176 | -9.33556 | 1.00E-20 | 2.52E-18 | ENSG00000125730 | 19 | 6677703 | 6720650 |
| ENST00000245923 | 45.8671278 | -1.272043807 | 0.469938 | -2.70683 | 0.006792881 | 0.043717365 | ENSG00000125744 | 19 | 45485293 | 45497047 |
| ENST00000246672 | 61.7727801 | -1.503415753 | 0.412013 | -3.64895 | 0.000263313 | 0.003316781 | ENSG00000126368 | 17 | 40092792 | 40100589 |
| ENST00000247470 | 22.6502665 | 2.326025247 | 0.702379 | 3.311639 | 0.000927511 | 0.00925496 | ENSG00000103490 | 16 | 31201485 | 31202760 |

| | | | | |
|---|---|---|---|---|
| PITRM1 | protein_coding | Yes | NM_014889.4 | 108 |
| DDX5 | protein_coding | Yes | NM_004396.5 | 108 |
| CD82 | protein_coding | Yes | NM_002231.4 | 108 |
| SLC15A3 | protein_coding | Yes | NM_016582.3 | 108 |
| CREBL2 | protein_coding | Yes | NM_001310.4 | 108 |
| OAS3 | protein_coding | Yes | NM_006187.4 | 108 |
| GNB3 | protein_coding | Yes | NM_002075.4 | 108 |
| ENO2 | protein_coding | Yes | NM_001975.3 | 108 |
| ULBP1 | protein_coding | Yes | NM_025218.4 | 108 |
| PCDH12 | protein_coding | Yes | NM_016580.4 | 108 |
| TNNC1 | protein_coding | Yes | NM_003280.3 | 108 |
| EIF2AK2 | protein_coding | Yes | NM_001135651.3 | 108 |
| WNT6 | protein_coding | Yes | NM_006522.4 | 108 |
| PRKD3 | protein_coding | Yes | NM_005813.6 | 108 |
| SELL | protein_coding | Yes | NM_000655.5 | 108 |
| PMFBP1 | protein_coding | Yes | NM_031293.3 | 108 |
| TP53I3 | protein_coding | Yes | NM_004881.5 | 108 |
| MFSD13A | protein_coding | Yes | NM_024789.4 | 108 |
| MSX2 | protein_coding | Yes | NM_002449.5 | 108 |
| PCDHB8 | protein_coding | Yes | NM_019120.5 | 108 |
| PCDHB14 | protein_coding | Yes | NM_018934.4 | 108 |
| EGR1 | protein_coding | Yes | NM_001964.3 | 108 |
| DUSP4 | protein_coding | Yes | NM_001394.7 | 108 |
| CAT | protein_coding | Yes | NM_001752.4 | 108 |
| TNFSF10 | protein_coding | Yes | NM_003810.4 | 108 |
| RASL11A | protein_coding | Yes | NM_206827.2 | 108 |
| DYNLL1 | protein_coding | Yes | NM_003746.3 | 108 |
| BHLHE41 | protein_coding | Yes | NM_030762.3 | 108 |
| FAAH | protein_coding | Yes | NM_001441.3 | 108 |
| ACVR1C | protein_coding | Yes | NM_145259.3 | 108 |
| CHPF | protein_coding | Yes | NM_024536.6 | 108 |
| SH3TC1 | protein_coding | Yes | NM_018986.5 | 108 |
| BBS2 | protein_coding | Yes | NM_031885.5 | 108 |
| MT2A | protein_coding | Yes | NM_005953.5 | 108 |
| BMP4 | protein_coding | Yes | NM_001202.6 | 108 |
| TNFSF9 | protein_coding | Yes | NM_003811.4 | 108 |
| C3 | protein_coding | Yes | NM_000064.4 | 108 |
| RTN2 | protein_coding | Yes | NM_005619.5 | 108 |
| NR1D1 | protein_coding | Yes | NM_021724.5 | 108 |
| PYCARD | protein_coding | Yes | NM_013258.5 | 108 |

| | | | | | | | | | | |
|---|---|---|---|---|---|---|---|---|---|---|
| ENST00000248306 | 93.5970232 | 1.181804118 | 0.338006 | 3.496405 | 0.000471572 | 0.005324956 | ENSG00000127720 | 12 | 82358528 | 82479239 |
| ENST00000248484 | 551.321596 | 1.285672707 | 0.171968 | 7.476243 | 7.65E-14 | 8.60E-12 | ENSG00000127863 | 13 | 23570411 | 23676093 |
| ENST00000249330 | 121.840993 | -2.631817245 | 0.382194 | -6.88607 | 5.74E-12 | 5.10E-10 | ENSG00000128564 | 7 | 101162508 | 101165569 |
| ENST00000249700 | 28.1663901 | 1.789910682 | 0.624696 | 2.865251 | 0.004166788 | 0.029977301 | ENSG00000128872 | 15 | 51751596 | 51816363 |
| ENST00000250383 | 1214.82402 | -1.613999586 | 0.137985 | -11.6969 | 1.32E-31 | 9.88E-29 | ENSG00000100867 | 14 | 23636363 | 23645639 |
| ENST00000251166 | 266.970239 | -1.136794143 | 0.221795 | -5.12543 | 2.97E-07 | 1.01E-05 | ENSG00000262246 | 16 | 4354541 | 4416596 |
| ENST00000251363 | 64.1830914 | 1.887087139 | 0.442159 | 4.267895 | 1.97E-05 | 0.00037678 | ENSG00000090661 | 19 | 8209369 | 8262421 |
| ENST00000251453 | 1920.87542 | 1.713511363 | 0.13683 | 12.52295 | 5.59E-36 | 5.99E-33 | ENSG00000105193 | 19 | 39433136 | 39435949 |
| ENST00000252029 | 105.839422 | 1.549305258 | 0.330956 | 4.681305 | 2.85E-06 | 7.28E-05 | ENSG00000025708 | 22 | 50525751 | 50530012 |
| ENST00000252242 | 229.226187 | -2.878160643 | 0.328816 | -8.7531 | 2.08E-18 | 4.06E-16 | ENSG00000186081 | 12 | 52514574 | 52520394 |
| ENST00000252486 | 372.75986 | -1.847619658 | 0.255086 | -7.24312 | 4.38E-13 | 4.53E-11 | ENSG00000130203 | 19 | 44905795 | 44909393 |
| ENST00000252506 | 116.623568 | -1.053990468 | 0.331477 | -3.17968 | 0.001474385 | 0.0134224 | ENSG00000130222 | 9 | 89605011 | 89606555 |
| ENST00000252809 | 140.295076 | -1.30340272 | 0.282683 | -4.61082 | 4.01E-06 | 9.74E-05 | ENSG00000130513 | 19 | 18386157 | 18389176 |
| ENST00000252971 | 51.1203905 | -1.794825708 | 0.457662 | -3.92172 | 8.79E-05 | 0.001322096 | ENSG00000130675 | 7 | 157004853 | 157010663 |
| ENST00000253122 | 147.727517 | -2.361609279 | 0.292922 | -8.06224 | 7.49E-16 | 1.09E-13 | ENSG00000130821 | X | 153687925 | 153696588 |
| ENST00000254667 | 297.56914 | -1.149500519 | 0.217354 | -5.2886 | 1.23E-07 | 4.67E-06 | ENSG00000132334 | 10 | 127907102 | 128085855 |
| ENST00000254691 | 32.0945094 | -4.641765336 | 0.834994 | -5.55904 | 2.71E-08 | 1.19E-06 | ENSG00000132357 | 5 | 40841366 | 40855354 |
| ENST00000254898 | 269.310297 | 2.999604978 | 0.249629 | 12.01623 | 2.92E-33 | 2.55E-30 | ENSG00000132561 | 8 | 97869063 | 98036724 |
| ENST00000254928 | 115.057436 | 1.357731993 | 0.313798 | 4.326771 | 1.51E-05 | 0.000302445 | ENSG00000132591 | 17 | 28855015 | 28861061 |
| ENST00000256084 | 65.0774636 | 1.400487488 | 0.40719 | 3.439396 | 0.000583013 | 0.006314158 | ENSG00000133710 | 5 | 148063979 | 148137382 |
| ENST00000256544 | 1968.89699 | -1.343225647 | 0.144784 | -9.27747 | 1.74E-20 | 4.31E-18 | ENSG00000134152 | 15 | 34140673 | 34210096 |
| ENST00000256722 | 129.18126 | 6.402541927 | 0.668324 | 9.580004 | 9.70E-22 | 2.63E-19 | ENSG00000134326 | 2 | 6848315 | 6865907 |
| ENST00000256733 | 31.023174 | -2.000225483 | 0.588309 | -3.39996 | 0.000673963 | 0.007122844 | ENSG00000134339 | 11 | 18245239 | 18248668 |
| ENST00000256996 | 86.8286694 | 1.201063999 | 0.349147 | 3.439993 | 0.000581729 | 0.006305023 | ENSG00000134574 | 11 | 47214973 | 47239217 |
| ENST00000257570 | 68.2273145 | 8.075831176 | 1.485943 | 5.434817 | 5.49E-08 | 2.28E-06 | ENSG00000135114 | 12 | 121019110 | 121039246 |
| ENST00000257770 | 41.7011254 | -5.778944484 | 0.966111 | -5.98166 | 2.21E-09 | 1.19E-07 | ENSG00000135318 | 6 | 85450082 | 85495784 |
| ENST00000258381 | 302.261459 | 1.013116224 | 0.281221 | 3.602567 | 0.00031509 | 0.003844341 | ENSG00000135899 | 2 | 230165185 | 230219984 |
| ENST00000258411 | 590.353285 | -2.541181451 | 0.187263 | -13.5701 | 6.02E-42 | 1.10E-38 | ENSG00000135925 | 2 | 218880851 | 218893928 |
| ENST00000258443 | 314.153405 | 1.989443206 | 0.277839 | 7.160425 | 8.04E-13 | 8.05E-11 | ENSG00000135960 | 2 | 108894470 | 108989220 |
| ENST00000258821 | 95.1156804 | 1.238824015 | 0.336849 | 3.677682 | 0.000235363 | 0.003018039 | ENSG00000136319 | 14 | 20286226 | 20305951 |
| ENST00000259870 | 21.8852469 | -4.075683449 | 0.907412 | -4.49155 | 7.07E-06 | 0.000157774 | ENSG00000204542 | 6 | 31111222 | 31112575 |
| ENST00000259939 | 275.715582 | 1.052908293 | 0.226203 | 4.65471 | 3.24E-06 | 8.12E-05 | ENSG00000137393 | 6 | 18387349 | 18468870 |
| ENST00000259951 | 158.860514 | 1.994588843 | 0.319726 | 6.238431 | 4.42E-10 | 2.74E-08 | ENSG00000204642 | 6 | 29723433 | 29727296 |
| ENST00000260113 | 83.8704686 | 1.295403951 | 0.388475 | 3.33459 | 0.000854253 | 0.008645558 | ENSG00000137558 | 8 | 74824533 | 74855029 |
| ENST00000260356 | 4392.3929 | -1.65349368 | 0.549029 | -3.01167 | 0.002598159 | 0.020798894 | ENSG00000137801 | 15 | 39581078 | 39599466 |
| ENST00000260453 | 24.8068203 | 2.075757875 | 0.660745 | 3.141542 | 0.001680605 | 0.014850188 | ENSG00000138587 | 15 | 56428723 | 56465137 |
| ENST00000260702 | 1036.90955 | -2.179869185 | 0.16264 | -13.403 | 5.80E-41 | 1.02E-37 | ENSG00000138131 | 10 | 98247689 | 98268194 |
| ENST00000261205 | 346.032075 | 1.222167888 | 0.215447 | 5.672714 | 1.41E-08 | 6.50E-07 | ENSG00000067715 | 12 | 78864773 | 79452008 |
| ENST00000261292 | 95.2461436 | -1.377020309 | 0.386607 | -3.56181 | 0.000368303 | 0.004369077 | ENSG00000101670 | 18 | 49562056 | 49599185 |
| ENST00000261464 | 14.1327895 | 3.71930385 | 1.050917 | 3.539104 | 0.000401488 | 0.004684785 | ENSG00000082512 | 1 | 211326832 | 211374946 |

| | | | | |
|---|---|---|---|---|
| METTL25 | protein_coding | Yes | NM_032230.3 | 108 |
| TNFRSF19 | protein_coding | Yes | NM_148957.4 | 108 |
| VGF | protein_coding | Yes | NM_003378.4 | 108 |
| TMOD2 | protein_coding | Yes | NM_014548.4 | 108 |
| DHRS2 | protein_coding | Yes | NM_005794.4 | 108 |
| CORO7 | protein_coding | Yes | NM_024535.5 | 108 |
| CERS4 | protein_coding | Yes | NM_024552.3 | 108 |
| RPS16 | protein_coding | Yes | NM_001020.6 | 108 |
| TYMP | protein_coding | Yes | NM_001953.5 | 108 |
| KRT5 | protein_coding | Yes | NM_000424.4 | 108 |
| APOE | protein_coding | Yes | NM_000041.4 | 108 |
| GADD45G | protein_coding | Yes | NM_006705.4 | 108 |
| GDF15 | protein_coding | Yes | NM_004864.4 | 108 |
| MNX1 | protein_coding | Yes | NM_005515.4 | 108 |
| SLC6A8 | protein_coding | Yes | NM_005629.4 | 108 |
| PTPRE | protein_coding | Yes | NM_006504.6 | 108 |
| CARD6 | protein_coding | Yes | NM_032587.4 | 108 |
| MATN2 | protein_coding | Yes | NM_002380.5 | 108 |
| ERAL1 | protein_coding | Yes | NM_005702.4 | 108 |
| SPINK5 | protein_coding | Yes | NM_006846.4 | 108 |
| KATNBL1 | protein_coding | Yes | NM_024713.3 | 108 |
| CMPK2 | protein_coding | Yes | NM_207315.4 | 108 |
| SAA2 | protein_coding | Yes | NM_030754.5 | 108 |
| DDB2 | protein_coding | Yes | NM_000107.3 | 108 |
| OASL | protein_coding | Yes | NM_003733.4 | 108 |
| NT5E | protein_coding | Yes | NM_002526.4 | 108 |
| SP110 | protein_coding | Yes | NM_080424.4 | 108 |
| WNT10A | protein_coding | Yes | NM_025216.3 | 108 |
| EDAR | protein_coding | Yes | NM_022336.4 | 108 |
| TTC5 | protein_coding | Yes | NM_138376.3 | 108 |
| C6orf15 | protein_coding | Yes | NM_014070.3 | 108 |
| RNF144B | protein_coding | Yes | NM_182757.4 | 108 |
| HLA-F | protein_coding | Yes | NM_001098479.2 | 108 |
| PI15 | protein_coding | Yes | NM_015886.5 | 108 |
| THBS1 | protein_coding | Yes | NM_003246.4 | 108 |
| MNS1 | protein_coding | Yes | NM_018365.4 | 108 |
| LOXL4 | protein_coding | Yes | NM_032211.7 | 108 |
| SYT1 | protein_coding | Yes | NM_005639.3 | 108 |
| LIPG | protein_coding | Yes | NM_006033.4 | 108 |
| TRAF5 | protein_coding | Yes | NM_001033910.3 | 108 |

| | | | | | | | | | | |
|---|---|---|---|---|---|---|---|---|---|---|
| ENST00000261486 | 18.6808705 | 6.189752978 | 1.587331 | 3.899472 | 9.64E-05 | 0.001428096 | ENSG00000129595 | 5 | 112162618 | 112419277 |
| ENST00000261769 | 1768.12489 | -1.820360091 | 0.138515 | -13.142 | 1.89E-39 | 2.83E-36 | ENSG00000039068 | 16 | 68737291 | 68835537 |
| ENST00000261908 | 115.871599 | -1.635236536 | 0.326476 | -5.00874 | 5.48E-07 | 1.74E-05 | ENSG00000067141 | 15 | 73052462 | 73305205 |
| ENST00000262094 | 68.8572687 | 1.619116257 | 0.414848 | 3.902912 | 9.50E-05 | 0.001411681 | ENSG00000041353 | 18 | 54828476 | 54895516 |
| ENST00000262096 | 116.407135 | -1.607562567 | 0.328362 | -4.89571 | 9.80E-07 | 2.92E-05 | ENSG00000104219 | 8 | 17156481 | 17224799 |
| ENST00000262133 | 517.125295 | 1.61659929 | 0.184666 | 8.754191 | 2.06E-18 | 4.03E-16 | ENSG00000103479 | 16 | 53434470 | 53491648 |
| ENST00000262207 | 89.4779749 | 1.16481726 | 0.382608 | 3.044417 | 0.002331315 | 0.01913553 | ENSG00000121005 | 8 | 74984504 | 75034558 |
| ENST00000262301 | 69.5635702 | -1.070394058 | 0.388722 | -2.75362 | 0.00589401 | 0.039140855 | ENSG00000103227 | 16 | 853633 | 970984 |
| ENST00000262315 | 359.292252 | -1.07593408 | 0.208936 | -5.1496 | 2.61E-07 | 9.04E-06 | ENSG00000127586 | 16 | 788619 | 798074 |
| ENST00000262424 | 298.413536 | 1.138310045 | 0.209553 | 5.432087 | 5.57E-08 | 2.31E-06 | ENSG00000103196 | 16 | 84819984 | 84909508 |
| ENST00000262580 | 402.185172 | -1.990021016 | 0.192667 | -10.3288 | 5.22E-25 | 1.85E-22 | ENSG00000104518 | 8 | 143558345 | 143563062 |
| ENST00000262848 | 305.014474 | 1.056234827 | 0.205959 | 5.128374 | 2.92E-07 | 9.95E-06 | ENSG00000183943 | X | 3604339 | 3713649 |
| ENST00000262992 | 108.567665 | -1.370125349 | 0.335578 | -4.08288 | 4.45E-05 | 0.00074995 | ENSG00000109452 | 4 | 142023159 | 142846301 |
| ENST00000263035 | 1483.05986 | 1.159121585 | 0.137675 | 8.419256 | 3.79E-17 | 6.42E-15 | ENSG00000181192 | 10 | 12068953 | 12123221 |
| ENST00000263038 | 313.192573 | 2.154044861 | 0.233856 | 9.211 | 3.23E-20 | 7.74E-18 | ENSG00000107537 | 10 | 13277798 | 13300064 |
| ENST00000263125 | 16.1463797 | 4.357460504 | 1.096124 | 3.975337 | 7.03E-05 | 0.001095333 | ENSG00000065675 | 10 | 6427147 | 6580276 |
| ENST00000263150 | 220.396352 | 1.302347885 | 0.236571 | 5.505113 | 3.69E-08 | 1.59E-06 | ENSG00000047056 | 10 | 1056384 | 1132372 |
| ENST00000263205 | 1259.27851 | -1.09738492 | 0.13652 | -8.03829 | 9.11E-16 | 1.31E-13 | ENSG00000099917 | 22 | 20507609 | 20587619 |
| ENST00000263266 | 62.5937537 | -1.103952279 | 0.403114 | -2.73856 | 0.006170934 | 0.040567731 | ENSG00000105523 | 19 | 48599960 | 48615076 |
| ENST00000263277 | 77.5856202 | 1.688320979 | 0.376208 | 4.48773 | 7.20E-06 | 0.000160226 | ENSG00000024422 | 19 | 47713421 | 47743134 |
| ENST00000263280 | 1147.50605 | -1.094974253 | 0.138841 | -7.88654 | 3.11E-15 | 4.23E-13 | ENSG00000090372 | 19 | 46719510 | 46746450 |
| ENST00000263372 | 43.1912391 | -1.371632861 | 0.507697 | -2.70167 | 0.00689914 | 0.044252355 | ENSG00000099337 | 19 | 38319844 | 38332076 |
| ENST00000263966 | 134.890367 | 1.01209741 | 0.284642 | 3.555681 | 0.000377001 | 0.004451288 | ENSG00000058056 | 3 | 179653039 | 179789401 |
| ENST00000264021 | 50.396685 | 1.347256357 | 0.465253 | 2.895748 | 0.003782554 | 0.027774815 | ENSG00000118096 | 11 | 118544542 | 118565931 |
| ENST00000264025 | 89.4336608 | 1.649458147 | 0.350266 | 4.709162 | 2.49E-06 | 6.47E-05 | ENSG00000110400 | 11 | 119660991 | 119729200 |
| ENST00000264228 | 56.0804485 | 1.794467484 | 0.439276 | 4.085057 | 4.41E-05 | 0.000744022 | ENSG00000128039 | 4 | 55346241 | 55373100 |
| ENST00000264234 | 54.519665 | 1.572625101 | 0.456371 | 3.445933 | 0.000569092 | 0.006187138 | ENSG00000114638 | 3 | 119173597 | 119205143 |
| ENST00000264245 | 26.0382593 | -1.832668015 | 0.63263 | -2.8969 | 0.003768657 | 0.027706424 | ENSG00000031081 | 3 | 119294382 | 119420714 |
| ENST00000264346 | 616.673141 | 1.804363905 | 0.196923 | 9.162805 | 5.06E-20 | 1.18E-17 | ENSG00000138642 | 4 | 88378851 | 88443097 |
| ENST00000264350 | 1352.0814 | 2.592209989 | 0.473782 | 5.471317 | 4.47E-08 | 1.89E-06 | ENSG00000138646 | 4 | 88457118 | 88506163 |
| ENST00000264409 | 576.833407 | -1.074045859 | 0.203108 | -5.28804 | 1.24E-07 | 4.68E-06 | ENSG00000138678 | 4 | 83536107 | 83605875 |
| ENST00000264434 | 109.682096 | 1.05124284 | 0.31484 | 3.338979 | 0.00084087 | 0.008536354 | ENSG00000115998 | 2 | 70149884 | 70191019 |
| ENST00000264555 | 1091.40469 | -1.031125935 | 0.150915 | -6.83248 | 8.35E-12 | 7.15E-10 | ENSG00000070047 | 11 | 576469 | 612222 |
| ENST00000264712 | 141.988242 | 1.389858261 | 0.283681 | 4.899372 | 9.61E-07 | 2.87E-05 | ENSG00000084731 | 2 | 25926597 | 25982497 |
| ENST00000264735 | 14.6041143 | -7.322147968 | 1.649311 | -4.43952 | 9.02E-06 | 0.000194101 | ENSG00000127252 | 3 | 193241221 | 193270855 |
| ENST00000264930 | 505.887448 | -2.254611848 | 0.183396 | -12.2937 | 9.79E-35 | 9.43E-32 | ENSG00000113504 | 5 | 1050383 | 1112063 |
| ENST00000264956 | 71.4577954 | 1.325569945 | 0.382975 | 3.461241 | 0.00053769 | 0.005912949 | ENSG00000072840 | 4 | 5711200 | 5814305 |
| ENST00000264982 | 788.824764 | 1.062311724 | 0.163086 | 6.513813 | 7.33E-11 | 5.21E-09 | ENSG00000114107 | 3 | 138494343 | 138594260 |
| ENST00000264992 | 73.8452606 | 1.368582349 | 0.378224 | 3.618445 | 0.000296378 | 0.0036541 | ENSG00000034533 | 3 | 131013874 | 131026825 |
| ENST00000265087 | 767.096162 | -1.499603755 | 0.155658 | -9.63394 | 5.75E-22 | 1.62E-19 | ENSG00000113739 | 5 | 173314722 | 173328414 |

| | | | | |
|---|---|---|---|---|
| EPB41L4A | protein_coding | Yes | NM_022140.5 | 108 |
| CDH1 | protein_coding | Yes | NM_004360.5 | 108 |
| NEO1 | protein_coding | Yes | NM_002499.4 | 108 |
| RAB27B | protein_coding | Yes | NM_004163.4 | 108 |
| ZDHHC2 | protein_coding | Yes | NM_016353.5 | 108 |
| RBL2 | protein_coding | Yes | NM_005611.4 | 108 |
| CRISPLD1 | protein_coding | Yes | NM_031461.6 | 108 |
| LMF1 | protein_coding | Yes | NM_022773.4 | 108 |
| CHTF18 | protein_coding | Yes | NM_022092.3 | 108 |
| CRISPLD2 | protein_coding | Yes | NM_031476.4 | 108 |
| GSDMD | protein_coding | Yes | NM_024736.7 | 108 |
| PRKX | protein_coding | Yes | NM_005044.5 | 108 |
| INPP4B | protein_coding | Yes | NM_001101669.3 | 108 |
| DHTKD1 | protein_coding | Yes | NM_018706.7 | 108 |
| PHYH | protein_coding | Yes | NM_006214.4 | 108 |
| PRKCQ | protein_coding | Yes | NM_006257.5 | 108 |
| WDR37 | protein_coding | Yes | NM_014023.4 | 108 |
| MED15 | protein_coding | Yes | NM_001003891.3 | 108 |
| FAM83E | protein_coding | Yes | NM_017708.4 | 108 |
| EHD2 | protein_coding | Yes | NM_014601.4 | 108 |
| STRN4 | protein_coding | Yes | NM_013403.3 | 108 |
| KCNK6 | protein_coding | Yes | NM_004823.3 | 108 |
| USP13 | protein_coding | Yes | NM_003940.3 | 108 |
| IFT46 | protein_coding | Yes | NM_001168618.2 | 108 |
| NECTIN1 | protein_coding | Yes | NM_002855.5 | 108 |
| SRD5A3 | protein_coding | Yes | NM_024592.5 | 108 |
| UPK1B | protein_coding | Yes | NM_006952.4 | 108 |
| ARHGAP31 | protein_coding | Yes | NM_020754.4 | 108 |
| HERC6 | protein_coding | Yes | NM_017912.4 | 108 |
| HERC5 | protein_coding | Yes | NM_016323.4 | 108 |
| GPAT3 | protein_coding | Yes | NM_032717.5 | 108 |
| C2orf42 | protein_coding | Yes | NM_017880.3 | 108 |
| PHRF1 | protein_coding | Yes | NM_001286581.2 | 108 |
| KIF3C | protein_coding | Yes | NM_002254.8 | 108 |
| PLAAT1 | protein_coding | Yes | NM_020386.5 | 108 |
| SLC12A7 | protein_coding | Yes | NM_006598.3 | 108 |
| EVC | protein_coding | Yes | NM_153717.3 | 108 |
| CEP70 | protein_coding | Yes | NM_024491.4 | 108 |
| ASTE1 | protein_coding | Yes | NM_014065.4 | 108 |
| STC2 | protein_coding | Yes | NM_003714.3 | 108 |

| | | | | | | | | | | |
|---|---|---|---|---|---|---|---|---|---|---|
| ENST00000265113 | 292.356926 | -1.079766855 | 0.222013 | -4.86353 | 1.15E-06 | 3.35E-05 | ENSG00000079215 | 5 | 36606605 | 36688334 |
| ENST00000265310 | 9.1329699 | -5.153077126 | 1.735873 | -2.96858 | 0.002991804 | 0.023179081 | ENSG00000127412 | 7 | 142908100 | 142933746 |
| ENST00000265322 | 45.6223824 | 1.596615048 | 0.486996 | 3.278494 | 0.001043625 | 0.010184287 | ENSG00000115425 | 2 | 216038387 | 216081809 |
| ENST00000265361 | 175.237833 | -1.753630946 | 0.300738 | -5.83109 | 5.51E-09 | 2.76E-07 | ENSG00000075223 | 7 | 80742537 | 80919051 |
| ENST00000265523 | 225.093462 | 1.155912434 | 0.231752 | 4.987714 | 6.11E-07 | 1.92E-05 | ENSG00000106605 | 7 | 43758679 | 43807342 |
| ENST00000265598 | 192.488419 | 1.403058287 | 0.331422 | 4.233454 | 2.30E-05 | 0.000428884 | ENSG00000078081 | 3 | 183122214 | 183162734 |
| ENST00000265758 | 255.195258 | -1.032169521 | 0.245484 | -4.20463 | 2.62E-05 | 0.000478308 | ENSG00000071462 | 7 | 73683596 | 73698212 |
| ENST00000265806 | 67.7820457 | 1.097785638 | 0.388818 | 2.823393 | 0.004751831 | 0.033224563 | ENSG00000104679 | 8 | 23288107 | 23296279 |
| ENST00000266263 | 62.9930565 | 1.21495962 | 0.42001 | 2.892695 | 0.003819515 | 0.027982978 | ENSG00000242114 | 22 | 30425767 | 30429054 |
| ENST00000266383 | 17.1251766 | 2.214422638 | 0.814341 | 2.719283 | 0.006542361 | 0.042470406 | ENSG00000139044 | 12 | 459938 | 563509 |
| ENST00000266556 | 24.190656 | 4.953139994 | 1.019644 | 4.857716 | 1.19E-06 | 3.44E-05 | ENSG00000139192 | 12 | 6452041 | 6462316 |
| ENST00000266671 | 2241.58422 | -1.039457301 | 0.143092 | -7.26428 | 3.75E-13 | 3.90E-11 | ENSG00000139289 | 12 | 76025446 | 76031776 |
| ENST00000267328 | 195.776399 | -1.091550541 | 0.254389 | -4.29088 | 1.78E-05 | 0.000344819 | ENSG00000139832 | 13 | 110523065 | 110561722 |
| ENST00000268097 | 24.4102369 | -6.607979987 | 1.539971 | -4.29098 | 1.78E-05 | 0.000344717 | ENSG00000213614 | 15 | 72340923 | 72376014 |
| ENST00000268261 | 456.842396 | -1.208604262 | 0.182937 | -6.60668 | 3.93E-11 | 2.93E-09 | ENSG00000140650 | 16 | 8797838 | 8849325 |
| ENST00000268281 | 30.6961081 | -1.973998422 | 0.593704 | -3.32488 | 0.000884553 | 0.008898579 | ENSG00000178226 | 16 | 31138925 | 31150066 |
| ENST00000268459 | 112.242108 | 1.191310693 | 0.310707 | 3.834197 | 0.000125975 | 0.001782323 | ENSG00000140807 | 16 | 50548395 | 50649249 |
| ENST00000269389 | 54.6182368 | 1.966983396 | 0.47439 | 4.146342 | 3.38E-05 | 0.000595717 | ENSG00000141574 | 17 | 82321023 | 82333766 |
| ENST00000269593 | 330.993426 | -1.82807024 | 0.21578 | -8.47191 | 2.41E-17 | 4.19E-15 | ENSG00000141753 | 17 | 40443449 | 40457725 |
| ENST00000270176 | 2981.04054 | -1.079153967 | 0.130348 | -8.27902 | 1.24E-16 | 1.96E-14 | ENSG00000142186 | 11 | 65525082 | 65538704 |
| ENST00000270645 | 13.3475599 | -5.713731812 | 1.62518 | -3.51575 | 0.000438509 | 0.005025574 | ENSG00000142552 | 19 | 49528002 | 49543633 |
| ENST00000271588 | 104.555332 | -1.07375712 | 0.321194 | -3.34302 | 0.000828721 | 0.008436872 | ENSG00000143341 | 1 | 185734390 | 186190949 |
| ENST00000271643 | 118.637572 | 1.263671479 | 0.331441 | 3.812662 | 0.000137478 | 0.001912831 | ENSG00000143382 | 1 | 150549407 | 150560933 |
| ENST00000271764 | 548.323457 | 1.175795956 | 0.169849 | 6.922612 | 4.43E-12 | 4.01E-10 | ENSG00000143486 | 1 | 206591641 | 206612465 |
| ENST00000272252 | 645.712447 | 1.043630398 | 0.182028 | 5.733363 | 9.85E-09 | 4.68E-07 | ENSG00000143891 | 2 | 38666113 | 38734765 |
| ENST00000272438 | 186.400735 | 1.278344612 | 0.251364 | 5.085635 | 3.66E-07 | 1.22E-05 | ENSG00000144043 | 2 | 70985941 | 70994873 |
| ENST00000272542 | 872.640124 | -1.017515035 | 0.180734 | -5.6299 | 1.80E-08 | 8.16E-07 | ENSG00000144136 | 2 | 112645938 | 112663825 |
| ENST00000272895 | 227.996035 | 1.025781968 | 0.243498 | 4.212689 | 2.52E-05 | 0.000463568 | ENSG00000144452 | 2 | 214931541 | 215138626 |
| ENST00000273067 | 35.4119129 | -2.803052145 | 0.597649 | -4.69013 | 2.73E-06 | 7.02E-05 | ENSG00000144583 | 2 | 216257864 | 216372483 |
| ENST00000274773 | 52.4810921 | 1.584351364 | 0.46242 | 3.426221 | 0.000612043 | 0.006573243 | ENSG00000146054 | 5 | 181193923 | 181205196 |
| ENST00000276646 | 230.625644 | 1.636173312 | 0.235452 | 6.94908 | 3.68E-12 | 3.39E-10 | ENSG00000147642 | 8 | 109573977 | 109644822 |
| ENST00000277632 | 234.726456 | 1.001817079 | 0.228711 | 4.380271 | 1.19E-05 | 0.000244633 | ENSG00000148481 | 10 | 15778173 | 15860507 |
| ENST00000278505 | 494.887179 | 1.958225689 | 0.180143 | 10.8704 | 1.59E-27 | 8.14E-25 | ENSG00000149218 | 11 | 95089845 | 95132645 |
| ENST00000280258 | 10558.6975 | -1.390102891 | 0.110331 | -12.5994 | 2.13E-36 | 2.33E-33 | ENSG00000150687 | 11 | 86800541 | 86811233 |
| ENST00000280551 | 121.427517 | -1.25424878 | 0.303541 | -4.13206 | 3.60E-05 | 0.000627426 | ENSG00000150961 | 4 | 118722822 | 118836126 |
| ENST00000280665 | 7.26194443 | 6.293426578 | 1.807692 | 3.481471 | 0.000498668 | 0.005564808 | ENSG00000151065 | 12 | 1946052 | 2004457 |
| ENST00000280734 | 41.1900059 | -1.374378465 | 0.499234 | -2.75297 | 0.005905654 | 0.03919235 | ENSG00000151117 | 11 | 18698778 | 18704785 |
| ENST00000281141 | 1418.51517 | 1.10158464 | 0.138883 | 7.931759 | 2.16E-15 | 2.98E-13 | ENSG00000151465 | 10 | 12196187 | 12250589 |
| ENST00000281172 | 312.847517 | 1.256107864 | 0.204355 | 6.146696 | 7.91E-10 | 4.67E-08 | ENSG00000151491 | 12 | 15620133 | 15789388 |
| ENST00000281441 | 441.09477 | -2.313758824 | 0.190901 | -12.1202 | 8.25E-34 | 7.42E-31 | ENSG00000151715 | 11 | 129815847 | 129860003 |

| | | | | |
|---|---|---|---|---|
| SLC1A3 | protein_coding | Yes | NM_004172.5 | 108 |
| TRPV5 | protein_coding | Yes | NM_019841.7 | 108 |
| PECR | protein_coding | Yes | NM_018441.6 | 108 |
| SEMA3C | protein_coding | Yes | NM_006379.5 | 108 |
| BLVRA | protein_coding | Yes | NM_000712.4 | 108 |
| LAMP3 | protein_coding | Yes | NM_014398.4 | 108 |
| BUD23 | protein_coding | Yes | NM_017528.5 | 108 |
| R3HCC1 | protein_coding | Yes | NM_001136108.3 | 108 |
| MTFP1 | protein_coding | Yes | NM_016498.5 | 108 |
| B4GALNT3 | protein_coding | Yes | NM_173593.4 | 108 |
| TAPBPL | protein_coding | Yes | NM_018009.5 | 108 |
| PHLDA1 | protein_coding | Yes | - | 108 |
| RAB20 | protein_coding | Yes | NM_017817.3 | 108 |
| HEXA | protein_coding | Yes | NM_000520.6 | 108 |
| PMM2 | protein_coding | Yes | NM_000303.3 | 108 |
| PRSS36 | protein_coding | Yes | NM_173502.5 | 108 |
| NKD1 | protein_coding | Yes | NM_033119.5 | 108 |
| SECTM1 | protein_coding | Yes | NM_003004.3 | 108 |
| IGFBP4 | protein_coding | Yes | NM_001552.3 | 108 |
| SCYL1 | protein_coding | Yes | NM_020680.4 | 108 |
| RCN3 | protein_coding | Yes | NM_020650.3 | 108 |
| HMCN1 | protein_coding | Yes | NM_031935.3 | 108 |
| ADAMTSL4 | protein_coding | Yes | NM_019032.6 | 108 |
| EIF2D | protein_coding | Yes | NM_006893.3 | 108 |
| GALM | protein_coding | Yes | NM_138801.3 | 108 |
| TEX261 | protein_coding | Yes | NM_144582.3 | 108 |
| SLC20A1 | protein_coding | Yes | NM_005415.5 | 108 |
| ABCA12 | protein_coding | Yes | NM_173076.3 | 108 |
| MARCHF4 | protein_coding | Yes | NM_020814.3 | 108 |
| TRIM7 | protein_coding | Yes | NM_203293.3 | 108 |
| SYBU | protein_coding | Yes | NM_001099754.2 | 108 |
| MINDY3 | protein_coding | Yes | NM_024948.4 | 108 |
| ENDOD1 | protein_coding | Yes | NM_015036.3 | 108 |
| PRSS23 | protein_coding | Yes | NM_007173.6 | 108 |
| SEC24D | protein_coding | Yes | NM_014822.4 | 108 |
| DCP1B | protein_coding | Yes | NM_152640.5 | 108 |
| TMEM86A | protein_coding | Yes | NM_153347.3 | 108 |
| CDC123 | protein_coding | Yes | NM_006023.3 | 108 |
| EPS8 | protein_coding | Yes | NM_004447.6 | 108 |
| TMEM45B | protein_coding | Yes | NM_138788.5 | 108 |

| | | | | | | | | | | |
|---|---|---|---|---|---|---|---|---|---|---|
| ENST00000281631 | 132.097186 | -6.033804012 | 0.580305 | -10.3976 | 2.54E-25 | 9.28E-23 | ENSG00000151883 | 5 | 50666636 | 50846519 |
| ENST00000281923 | 624.732235 | -1.033934283 | 0.161751 | -6.39215 | 1.64E-10 | 1.09E-08 | ENSG00000152127 | 2 | 134254071 | 134454621 |
| ENST00000281938 | 134.505817 | -2.006212691 | 0.335647 | -5.97714 | 2.27E-09 | 1.22E-07 | ENSG00000152137 | 12 | 119178930 | 119194746 |
| ENST00000282388 | 246.041103 | 1.082520568 | 0.223874 | 4.835392 | 1.33E-06 | 3.79E-05 | ENSG00000152518 | 2 | 43222401 | 43226606 |
| ENST00000282406 | 49.0014519 | 1.442107226 | 0.500247 | 2.882791 | 0.003941687 | 0.028679943 | ENSG00000152527 | 2 | 43637259 | 43767987 |
| ENST00000282588 | 17.7904687 | -2.991290979 | 0.84289 | -3.54885 | 0.000386917 | 0.004538944 | ENSG00000213949 | 5 | 52787915 | 52959209 |
| ENST00000282633 | 1303.41213 | 1.058500909 | 0.145318 | 7.284038 | 3.24E-13 | 3.41E-11 | ENSG00000099290 | 10 | 50067953 | 50133509 |
| ENST00000283233 | 33.2339415 | 1.644802791 | 0.559684 | 2.938806 | 0.003294791 | 0.0249734 | ENSG00000153237 | 2 | 158171072 | 158456753 |
| ENST00000285039 | 281.985925 | 1.082757607 | 0.225066 | 4.810842 | 1.50E-06 | 4.21E-05 | ENSG00000167306 | 18 | 49822788 | 50195147 |
| ENST00000285238 | 118.298952 | -2.878735631 | 0.338462 | -8.50535 | 1.81E-17 | 3.19E-15 | ENSG00000108846 | 17 | 50634880 | 50692253 |
| ENST00000285947 | 62.9369262 | 1.149885232 | 0.4037 | 2.848364 | 0.004394458 | 0.031286655 | ENSG00000155542 | 5 | 56909595 | 56917348 |
| ENST00000286201 | 475.530623 | -1.639209819 | 0.189168 | -8.66534 | 4.50E-18 | 8.36E-16 | ENSG00000155760 | 2 | 202033854 | 202038441 |
| ENST00000290472 | 4.96938648 | -5.766431035 | 1.95871 | -2.944 | 0.003240052 | 0.024671117 | ENSG00000159337 | 15 | 42067008 | 42094562 |
| ENST00000290510 | 71.5914354 | -3.827234424 | 0.48325 | -7.91978 | 2.38E-15 | 3.27E-13 | ENSG00000110811 | 12 | 6828406 | 6839847 |
| ENST00000290551 | 15.1895824 | 2.711443234 | 0.884635 | 3.06504 | 0.002176408 | 0.018136453 | ENSG00000159388 | 1 | 203305518 | 203309602 |
| ENST00000290573 | 20.8090471 | -2.081243927 | 0.717264 | -2.90164 | 0.003712117 | 0.027401383 | ENSG00000159399 | 2 | 74834126 | 74893359 |
| ENST00000290902 | 36.1238363 | -2.413674685 | 0.563669 | -4.28208 | 1.85E-05 | 0.000356656 | ENSG00000159674 | 4 | 1166931 | 1172583 |
| ENST00000292140 | 492.440348 | -1.13415062 | 0.175079 | -6.47792 | 9.30E-11 | 6.46E-09 | ENSG00000176531 | 19 | 43475102 | 43504746 |
| ENST00000293195 | 264.086265 | 1.02652471 | 0.218097 | 4.706745 | 2.52E-06 | 6.54E-05 | ENSG00000161513 | 17 | 74862496 | 74872994 |
| ENST00000293471 | 507.581144 | 1.137065838 | 0.175888 | 6.464733 | 1.01E-10 | 6.99E-09 | ENSG00000176024 | 19 | 51927471 | 51946621 |
| ENST00000294244 | 114.813006 | -1.229108674 | 0.316751 | -3.88036 | 0.0001043 | 0.00152565 | ENSG00000168005 | 11 | 63813455 | 63827716 |
| ENST00000295156 | 4.04681398 | -5.467228023 | 2.052778 | -2.66333 | 0.007737118 | 0.048289009 | ENSG00000163032 | 2 | 17540695 | 17657018 |
| ENST00000295448 | 77.4651482 | -5.248742015 | 0.613653 | -8.55327 | 1.20E-17 | 2.13E-15 | ENSG00000163281 | 4 | 44701794 | 44726556 |
| ENST00000295809 | 9.077598 | 6.614319008 | 1.747698 | 3.78459 | 0.000153962 | 0.002101864 | ENSG00000163565 | 1 | 159009906 | 159055151 |
| ENST00000296161 | 1548.69098 | 1.225990027 | 0.175566 | 6.983084 | 2.89E-12 | 2.70E-10 | ENSG00000163840 | 3 | 122564337 | 122575203 |
| ENST00000296318 | 109.089982 | 1.192962119 | 0.315743 | 3.778275 | 0.000157918 | 0.002148574 | ENSG00000144730 | 3 | 57089981 | 57165353 |
| ENST00000296358 | 4.46615624 | -5.612620884 | 2.01042 | -2.79177 | 0.00524213 | 0.03572226 | ENSG00000163982 | 4 | 4188725 | 4226929 |
| ENST00000296370 | 414.884197 | -1.949897616 | 0.202072 | -9.64952 | 4.94E-22 | 1.41E-19 | ENSG00000163993 | 4 | 6693877 | 6697170 |
| ENST00000296498 | 61.8505607 | -2.460868307 | 0.454527 | -5.41412 | 6.16E-08 | 2.53E-06 | ENSG00000164099 | 4 | 118280037 | 118353003 |
| ENST00000296978 | 109.135179 | 5.016233917 | 0.500384 | 10.02478 | 1.19E-23 | 3.80E-21 | ENSG00000164484 | 6 | 130366016 | 130443067 |
| ENST00000297012 | 6.80409434 | -6.217444682 | 1.827404 | -3.40234 | 0.00066812 | 0.007070986 | ENSG00000164508 | 6 | 25726062 | 25726562 |
| ENST00000297261 | 8.96189663 | -3.477457808 | 1.262607 | -2.75419 | 0.005883767 | 0.039090013 | ENSG00000164690 | 7 | 155799979 | 155812463 |
| ENST00000297785 | 4.97786475 | 5.74958366 | 1.949778 | 2.94884 | 0.003189689 | 0.024372674 | ENSG00000165092 | 9 | 72900670 | 72953053 |
| ENST00000297990 | 1665.58298 | -1.773476922 | 0.433714 | -4.08905 | 4.33E-05 | 0.000732969 | ENSG00000165271 | 9 | 33461352 | 33473924 |
| ENST00000297991 | 201.416512 | -2.552476068 | 0.291775 | -8.74811 | 2.17E-18 | 4.23E-16 | ENSG00000165272 | 9 | 33441159 | 33447593 |
| ENST00000298130 | 249.153609 | 1.237006638 | 0.231625 | 5.340563 | 9.27E-08 | 3.63E-06 | ENSG00000165389 | 14 | 34432787 | 34462240 |
| ENST00000298299 | 95.8277313 | 1.507206627 | 0.35779 | 4.212542 | 2.53E-05 | 0.000463813 | ENSG00000165512 | 10 | 45000922 | 45005326 |
| ENST00000298428 | 112.10142 | 1.157992993 | 0.311847 | 3.713333 | 0.000204547 | 0.002673248 | ENSG00000065665 | 10 | 12129676 | 12165407 |
| ENST00000298596 | 175.00903 | 1.757512283 | 0.263585 | 6.667717 | 2.60E-11 | 2.01E-09 | ENSG00000165730 | 10 | 68827530 | 68893060 |
| ENST00000299001 | 29.5065672 | -1.839955081 | 0.596356 | -3.08533 | 0.002033278 | 0.017196409 | ENSG00000134627 | 11 | 94567367 | 94621421 |

| | | | | |
|---|---|---|---|---|
| PARP8 | protein_coding | Yes | NM_024615.4 | 108 |
| MGAT5 | protein_coding | Yes | NM_002410.5 | 108 |
| HSPB8 | protein_coding | Yes | NM_014365.3 | 108 |
| ZFP36L2 | protein_coding | Yes | NM_006887.5 | 108 |
| PLEKHH2 | protein_coding | Yes | NM_172069.4 | 108 |
| ITGA1 | protein_coding | Yes | NM_181501.2 | 108 |
| WASHC2A | protein_coding | Yes | NM_001005751.3 | 108 |
| CCDC148 | protein_coding | Yes | NM_138803.4 | 108 |
| MYO5B | protein_coding | Yes | NM_001080467.3 | 108 |
| ABCC3 | protein_coding | Yes | NM_003786.4 | 108 |
| SETD9 | protein_coding | Yes | NM_153706.4 | 108 |
| FZD7 | protein_coding | Yes | NM_003507.2 | 108 |
| PLA2G4D | protein_coding | Yes | NM_178034.4 | 108 |
| P3H3 | protein_coding | Yes | NM_014262.5 | 108 |
| BTG2 | protein_coding | Yes | NM_006763.3 | 108 |
| HK2 | protein_coding | Yes | NM_000189.5 | 108 |
| SPON2 | protein_coding | Yes | NM_012445.4 | 108 |
| PHLDB3 | protein_coding | Yes | NM_198850.4 | 108 |
| FDXR | protein_coding | Yes | NM_024417.5 | 108 |
| ZNF613 | protein_coding | Yes | NM_001031721.4 | 108 |
| SPINDOC | protein_coding | Yes | NM_138471.3 | 108 |
| VSNL1 | protein_coding | Yes | NM_003385.5 | 108 |
| GNPDA2 | protein_coding | Yes | NM_138335.3 | 108 |
| IFI16 | protein_coding | Yes | NM_001376587.1 | 108 |
| DTX3L | protein_coding | Yes | NM_138287.3 | 108 |
| IL17RD | protein_coding | Yes | NM_017563.5 | 108 |
| OTOP1 | protein_coding | Yes | NM_177998.3 | 108 |
| S100P | protein_coding | Yes | NM_005980.3 | 108 |
| PRSS12 | protein_coding | Yes | NM_003619.4 | 108 |
| TMEM200A | protein_coding | Yes | NM_001258277.2 | 108 |
| H2AC1 | protein_coding | Yes | NM_170745.3 | 108 |
| SHH | protein_coding | Yes | NM_000193.4 | 108 |
| ALDH1A1 | protein_coding | Yes | NM_000689.5 | 108 |
| NOL6 | protein_coding | Yes | NM_022917.5 | 108 |
| AQP3 | protein_coding | Yes | NM_004925.5 | 108 |
| SPTSSA | protein_coding | Yes | NM_138288.4 | 108 |
| ZNF22 | protein_coding | Yes | NM_006963.5 | 108 |
| SEC61A2 | protein_coding | Yes | NM_018144.4 | 108 |
| STOX1 | protein_coding | Yes | NM_152709.5 | 108 |
| PIWIL4 | protein_coding | Yes | NM_152431.3 | 108 |

| | | | | | | | | | | |
|---|---|---|---|---|---|---|---|---|---|---|
| ENST00000299084 | 198.492948 | -1.069891918 | 0.242951 | -4.40374 | 1.06E-05 | 0.000223186 | ENSG00000166068 | 15 | 38252835 | 38357249 |
| ENST00000299237 | 78.9675647 | 1.007453974 | 0.372262 | 2.706307 | 0.006803616 | 0.043765952 | ENSG00000166188 | 16 | 57994675 | 58000672 |
| ENST00000299440 | 197.446866 | 1.29132095 | 0.266746 | 4.841022 | 1.29E-06 | 3.70E-05 | ENSG00000166349 | 11 | 36568006 | 36579762 |
| ENST00000299698 | 4.02584198 | -5.460585771 | 2.05251 | -2.66044 | 0.007803783 | 0.048574694 | ENSG00000166535 | 12 | 8822620 | 8876787 |
| ENST00000300179 | 51.1761638 | -1.498287829 | 0.452477 | -3.3113 | 0.000928641 | 0.009264091 | ENSG00000166924 | 7 | 100483926 | 100494802 |
| ENST00000300181 | 511.315319 | -1.013750171 | 0.173093 | -5.85668 | 4.72E-09 | 2.39E-07 | ENSG00000166925 | 7 | 100466518 | 100479214 |
| ENST00000300291 | 1602.71583 | 1.16304591 | 0.130749 | 8.895269 | 5.83E-19 | 1.22E-16 | ENSG00000167005 | 16 | 56429132 | 56451332 |
| ENST00000300527 | 30.1247447 | -2.324177521 | 0.632615 | -3.67392 | 0.000238856 | 0.003056373 | ENSG00000142173 | 21 | 46098111 | 46132848 |
| ENST00000300784 | 30.3066126 | 2.080200635 | 0.60528 | 3.436758 | 0.000588721 | 0.006364837 | ENSG00000167363 | 17 | 82735614 | 82751196 |
| ENST00000300849 | 220.43441 | -1.00178006 | 0.242333 | -4.1339 | 3.57E-05 | 0.000623001 | ENSG00000167394 | 16 | 31060846 | 31074240 |
| ENST00000301732 | 38.9934689 | 1.473227359 | 0.514808 | 2.861701 | 0.00421374 | 0.030218596 | ENSG00000167972 | 16 | 2275880 | 2340728 |
| ENST00000302060 | 65.6404415 | 1.224987159 | 0.405227 | 3.022962 | 0.002503133 | 0.020204069 | ENSG00000170464 | 5 | 139410202 | 139439525 |
| ENST00000302274 | 10.0381892 | -4.264236758 | 1.354803 | -3.1475 | 0.001646753 | 0.014628714 | ENSG00000170522 | 4 | 110045845 | 110198615 |
| ENST00000302628 | 42.2227866 | -1.532985798 | 0.510503 | -3.00289 | 0.002674267 | 0.021270707 | ENSG00000172137 | 16 | 71358722 | 71390433 |
| ENST00000302850 | 488.937099 | 1.426048414 | 0.215629 | 6.613422 | 3.76E-11 | 2.82E-09 | ENSG00000171105 | 19 | 7112264 | 7294414 |
| ENST00000303225 | 86.645549 | -1.900037792 | 0.36343 | -5.22806 | 1.71E-07 | 6.24E-06 | ENSG00000171124 | 19 | 5842887 | 5851449 |
| ENST00000303460 | 70.5392393 | -1.843599181 | 0.394741 | -4.6704 | 3.01E-06 | 7.61E-05 | ENSG00000180818 | 12 | 53985145 | 53990279 |
| ENST00000303721 | 65.5728842 | 1.310203254 | 0.397968 | 3.292235 | 0.000993943 | 0.009796248 | ENSG00000172456 | 1 | 59297093 | 59762730 |
| ENST00000303746 | 300.443298 | -2.970091168 | 0.229691 | -12.9308 | 3.02E-38 | 4.14E-35 | ENSG00000131650 | 16 | 2964274 | 2968380 |
| ENST00000304735 | 695.498767 | -1.596457362 | 0.172636 | -9.24752 | 2.30E-20 | 5.60E-18 | ENSG00000168140 | 16 | 4371847 | 4383538 |
| ENST00000304749 | 8.64923935 | -6.566721345 | 1.771398 | -3.70708 | 0.000209659 | 0.002731554 | ENSG00000170373 | 20 | 23747561 | 23750935 |
| ENST00000304800 | 333.174402 | -1.162521521 | 0.201422 | -5.77158 | 7.85E-09 | 3.81E-07 | ENSG00000168701 | 16 | 67227129 | 67229278 |
| ENST00000305352 | 16.436323 | -3.943674654 | 1.024317 | -3.85005 | 0.000118092 | 0.001690086 | ENSG00000170989 | 1 | 101237018 | 101241518 |
| ENST00000305428 | 326.321709 | -1.189876334 | 0.210281 | -5.6585 | 1.53E-08 | 7.02E-07 | ENSG00000169330 | 15 | 79432335 | 79472304 |
| ENST00000305544 | 1049.51512 | 1.306743766 | 0.190916 | 6.844597 | 7.67E-12 | 6.62E-10 | ENSG00000172037 | 3 | 49121113 | 49133050 |
| ENST00000305560 | 392.895637 | -1.004373276 | 0.227366 | -4.41743 | 9.99E-06 | 0.000211617 | ENSG00000171763 | 15 | 45402335 | 45421415 |
| ENST00000305709 | 40.1674788 | 1.630766881 | 0.509436 | 3.201121 | 0.00136894 | 0.012667726 | ENSG00000170161 | 9 | 62897448 | 62900104 |
| ENST00000305747 | 457.10302 | 2.472951032 | 0.206615 | 11.96887 | 5.17E-33 | 4.38E-30 | ENSG00000172292 | 2 | 168456271 | 168775134 |
| ENST00000305798 | 90.8875354 | -1.331804014 | 0.342643 | -3.88685 | 0.000101553 | 0.001491238 | ENSG00000168785 | 4 | 98470366 | 98658611 |
| ENST00000306390 | 68.1771149 | -9.543665291 | 1.49078 | -6.40179 | 1.54E-10 | 1.03E-08 | ENSG00000171236 | 19 | 4536401 | 4540036 |
| ENST00000307102 | 1349.04253 | -1.069098918 | 0.16732 | -6.38953 | 1.66E-10 | 1.10E-08 | ENSG00000169032 | 15 | 66386911 | 66491544 |
| ENST00000307365 | 1502.53647 | -1.26615713 | 0.134362 | -9.42351 | 4.36E-21 | 1.12E-18 | ENSG00000168209 | 10 | 72273923 | 72276036 |
| ENST00000307407 | 50.3479604 | -3.457770236 | 0.549325 | -6.29458 | 3.08E-10 | 1.95E-08 | ENSG00000169429 | 4 | 73740568 | 73743716 |
| ENST00000307428 | 218.453126 | 9.761321444 | 1.451992 | 6.72271 | 1.78E-11 | 1.42E-09 | ENSG00000169116 | 4 | 74933115 | 75050113 |
| ENST00000307465 | 54.929461 | 1.995131424 | 0.452579 | 4.408363 | 1.04E-05 | 0.000219204 | ENSG00000138769 | 4 | 75576495 | 75630528 |
| ENST00000307729 | 519.1164 | 1.071484625 | 0.180212 | 5.945678 | 2.75E-09 | 1.46E-07 | ENSG00000078403 | 10 | 21534231 | 21743630 |
| ENST00000308874 | 163.336529 | -1.412912152 | 0.268179 | -5.26855 | 1.38E-07 | 5.15E-06 | ENSG00000172889 | 9 | 136662915 | 136672678 |
| ENST00000308919 | 41.1367729 | -4.506393547 | 0.709871 | -6.34818 | 2.18E-10 | 1.41E-08 | ENSG00000073067 | 7 | 983180 | 989640 |
| ENST00000309328 | 439.944669 | -1.248397879 | 0.218771 | -5.70642 | 1.15E-08 | 5.41E-07 | ENSG00000173465 | 11 | 65570476 | 65571888 |
| ENST00000309422 | 156.796639 | -1.032666821 | 0.267954 | -3.85389 | 0.000116256 | 0.001669218 | ENSG00000173511 | 11 | 64234583 | 64239264 |

| | | | | |
|---|---|---|---|---|
| SPRED1 | protein_coding | Yes | NM_152594.3 | 108 |
| ZNF319 | protein_coding | Yes | NM_020807.3 | 108 |
| RAG1 | protein_coding | Yes | NM_000448.3 | 108 |
| A2ML1 | protein_coding | Yes | NM_144670.6 | 108 |
| NYAP1 | protein_coding | Yes | NM_173564.4 | 108 |
| TSC22D4 | protein_coding | Yes | NM_030935.5 | 108 |
| NUDT21 | protein_coding | Yes | NM_007006.3 | 108 |
| COL6A2 | protein_coding | Yes | NM_001849.4 | 108 |
| FN3K | protein_coding | Yes | NM_022158.4 | 108 |
| ZNF668 | protein_coding | Yes | NM_024706.5 | 108 |
| ABCA3 | protein_coding | Yes | NM_001089.3 | 108 |
| DNAJC18 | protein_coding | Yes | NM_152686.4 | 108 |
| ELOVL6 | protein_coding | Yes | NM_024090.3 | 108 |
| CALB2 | protein_coding | Yes | NM_001740.5 | 108 |
| INSR | protein_coding | Yes | NM_000208.4 | 108 |
| FUT3 | protein_coding | Yes | - | 108 |
| HOXC10 | protein_coding | Yes | NM_017409.4 | 108 |
| FGGY | protein_coding | Yes | NM_018291.5 | 108 |
| KREMEN2 | protein_coding | Yes | NM_172229.3 | 108 |
| VASN | protein_coding | Yes | NM_138440.3 | 108 |
| CST1 | protein_coding | Yes | NM_001898.3 | 108 |
| TMEM208 | protein_coding | Yes | NM_014187.4 | 108 |
| S1PR1 | protein_coding | Yes | NM_001400.5 | 108 |
| MINAR1 | protein_coding | Yes | NM_015206.3 | 108 |
| LAMB2 | protein_coding | Yes | NM_002292.4 | 108 |
| SPATA5L1 | protein_coding | Yes | NM_024063.3 | 108 |
| FAM88B | lncRNA | Yes | - | 108 |
| CERS6 | protein_coding | Yes | NM_203463.3 | 108 |
| TSPAN5 | protein_coding | Yes | NM_005723.4 | 108 |
| LRG1 | protein_coding | Yes | NM_052972.3 | 108 |
| MAP2K1 | protein_coding | Yes | NM_002755.4 | 108 |
| DDIT4 | protein_coding | Yes | NM_019058.4 | 108 |
| CXCL8 | protein_coding | Yes | NM_000584.4 | 108 |
| PARM1 | protein_coding | Yes | NM_015393.4 | 108 |
| CDKL2 | protein_coding | Yes | NM_001330724.2 | 108 |
| MLLT10 | protein_coding | Yes | NM_001195626.3 | 108 |
| EGFL7 | protein_coding | Yes | NM_016215.5 | 108 |
| CYP2W1 | protein_coding | Yes | NM_017781.3 | 108 |
| ZNRD2 | protein_coding | Yes | NM_006396.3 | 108 |
| VEGFB | protein_coding | Yes | NM_003377.5 | 108 |

| | | | | | | | | | | |
|---|---|---|---|---|---|---|---|---|---|---|
| ENST00000309731 | 304.959056 | 1.416907404 | 0.20779 | 6.818932 | 9.17E-12 | 7.79E-10 | ENSG00000175318 | 15 | 72159805 | 72197787 |
| ENST00000309951 | 637.422828 | -1.166393812 | 0.170709 | -6.83266 | 8.34E-12 | 7.15E-10 | ENSG00000122729 | 9 | 32384642 | 32454769 |
| ENST00000310833 | 4.52907223 | -5.630511359 | 1.993554 | -2.82436 | 0.004737537 | 0.033149239 | ENSG00000174059 | 1 | 207880971 | 207911125 |
| ENST00000311052 | 1646.43453 | -1.36446277 | 0.142048 | -9.60566 | 7.57E-22 | 2.10E-19 | ENSG00000153048 | 16 | 8852946 | 8869006 |
| ENST00000311177 | 37.6752444 | -2.028863147 | 0.540433 | -3.75414 | 0.000173936 | 0.00233063 | ENSG00000170689 | 17 | 48621155 | 48626358 |
| ENST00000311208 | 386.145115 | -2.120846305 | 0.26465 | -8.01379 | 1.11E-15 | 1.57E-13 | ENSG00000128422 | 17 | 41619441 | 41624575 |
| ENST00000311852 | 3012.66641 | -1.198771137 | 0.121607 | -9.85776 | 6.35E-23 | 1.92E-20 | ENSG00000157227 | 14 | 22836584 | 22847758 |
| ENST00000312108 | 645.016003 | 1.011218683 | 0.168572 | 5.998748 | 1.99E-09 | 1.09E-07 | ENSG00000205560 | 22 | 50568860 | 50578427 |
| ENST00000312134 | 158.408412 | -1.237939807 | 0.278115 | -4.45118 | 8.54E-06 | 0.000185468 | ENSG00000175315 | 11 | 66012007 | 66013505 |
| ENST00000312438 | 63.8940793 | -1.267741109 | 0.415658 | -3.04996 | 0.002288691 | 0.018856724 | ENSG00000175505 | 11 | 67364167 | 67373598 |
| ENST00000312562 | 82.8716797 | -1.088991232 | 0.355725 | -3.06133 | 0.002203589 | 0.018317504 | ENSG00000175592 | 11 | 65892048 | 65900388 |
| ENST00000313005 | 78.7064443 | 1.763161993 | 0.386547 | 4.56131 | 5.08E-06 | 0.000118708 | ENSG00000186318 | 11 | 117285697 | 117316256 |
| ENST00000313093 | 159.553556 | -1.759513516 | 0.27682 | -6.35617 | 2.07E-10 | 1.35E-08 | ENSG00000180448 | 19 | 1067166 | 1086628 |
| ENST00000313401 | 186.304976 | -1.347629132 | 0.271122 | -4.97057 | 6.68E-07 | 2.08E-05 | ENSG00000176749 | 17 | 32486992 | 32491253 |
| ENST00000313546 | 117.301272 | 1.050451155 | 0.304003 | 3.455399 | 0.00054948 | 0.006024185 | ENSG00000139323 | 12 | 89419717 | 89526047 |
| ENST00000316105 | 20.9146179 | 6.362150943 | 1.572747 | 4.045246 | 5.23E-05 | 0.000857505 | ENSG00000177839 | 5 | 141187160 | 141191541 |
| ENST00000316157 | 1138.23246 | 1.301920892 | 0.145856 | 8.926072 | 4.41E-19 | 9.32E-17 | ENSG00000107929 | 10 | 809496 | 931705 |
| ENST00000317008 | 11.4782717 | -2.780515201 | 1.045587 | -2.65929 | 0.007830661 | 0.048702558 | ENSG00000178412 | 18 | 79638927 | 79679745 |
| ENST00000317571 | 132.774603 | -1.073808178 | 0.287725 | -3.73207 | 0.000189914 | 0.002510694 | ENSG00000168234 | 18 | 24014753 | 24135600 |
| ENST00000318443 | 468.295552 | -1.102552872 | 0.177503 | -6.21144 | 5.25E-10 | 3.20E-08 | ENSG00000103855 | 15 | 73684382 | 73714514 |
| ENST00000318579 | 104.183994 | -7.708305817 | 1.069939 | -7.20444 | 5.83E-13 | 5.96E-11 | ENSG00000005102 | 17 | 43640388 | 43661922 |
| ENST00000318683 | 369.568968 | -1.085952383 | 0.198353 | -5.47485 | 4.38E-08 | 1.85E-06 | ENSG00000179913 | 19 | 17795137 | 17813576 |
| ENST00000318948 | 121.868803 | 8.917402295 | 1.455978 | 6.124682 | 9.09E-10 | 5.32E-08 | ENSG00000180530 | 21 | 14961234 | 15065000 |
| ENST00000319017 | 99.7340423 | 1.149532856 | 0.337359 | 3.407451 | 0.000655728 | 0.006960793 | ENSG00000178537 | 3 | 48856925 | 48898882 |
| ENST00000319349 | 204.892178 | -2.226567975 | 0.304454 | -7.31332 | 2.61E-13 | 2.77E-11 | ENSG00000175832 | 17 | 43527845 | 43546340 |
| ENST00000319359 | 27.2039246 | 1.968931542 | 0.627071 | 3.139886 | 0.001690137 | 0.014919169 | ENSG00000128886 | 15 | 43772619 | 43776966 |
| ENST00000319550 | 404.503511 | 1.385142541 | 0.19627 | 7.05733 | 1.70E-12 | 1.63E-10 | ENSG00000178425 | 6 | 116100852 | 116249497 |
| ENST00000319933 | 102.021645 | 1.356743004 | 0.326554 | 4.154725 | 3.26E-05 | 0.000577974 | ENSG00000181751 | 5 | 103258762 | 103278660 |
| ENST00000320892 | 70.0854907 | 6.523476897 | 0.923586 | 7.063208 | 1.63E-12 | 1.57E-10 | ENSG00000151692 | 2 | 6917411 | 7044179 |
| ENST00000320895 | 2496.50913 | -1.382784446 | 0.156084 | -8.85922 | 8.06E-19 | 1.67E-16 | ENSG00000049656 | 5 | 1317751 | 1345099 |
| ENST00000320954 | 1143.26741 | 1.014194718 | 0.151518 | 6.693551 | 2.18E-11 | 1.70E-09 | ENSG00000170275 | 3 | 33114013 | 33147773 |
| ENST00000321562 | 464.714676 | -1.204178931 | 0.177969 | -6.76622 | 1.32E-11 | 1.08E-09 | ENSG00000141756 | 17 | 41813003 | 41823213 |
| ENST00000322563 | 115.059719 | -1.86547314 | 0.31657 | -5.89278 | 3.80E-09 | 1.96E-07 | ENSG00000085741 | 11 | 76186324 | 76206502 |
| ENST00000323468 | 410.210468 | 1.083172777 | 0.184253 | 5.878739 | 4.13E-09 | 2.12E-07 | ENSG00000178202 | 11 | 108472115 | 108498384 |
| ENST00000323851 | 3538.98912 | -1.931905915 | 0.122882 | -15.7217 | 1.07E-55 | 5.07E-52 | ENSG00000104419 | 8 | 133237174 | 133297252 |
| ENST00000323963 | 3261.9461 | 1.099594719 | 0.118735 | 9.260881 | 2.03E-20 | 4.97E-18 | ENSG00000156976 | 3 | 186783576 | 186789897 |
| ENST00000324001 | 68.9383425 | 1.598810489 | 0.396576 | 4.031539 | 5.54E-05 | 0.000899526 | ENSG00000105227 | 19 | 40393767 | 40413378 |
| ENST00000324093 | 123.868881 | -1.56787984 | 0.300994 | -5.20901 | 1.90E-07 | 6.85E-06 | ENSG00000004399 | 3 | 129555213 | 129606676 |
| ENST00000324219 | 429.459497 | 1.164201211 | 0.18574 | 6.267904 | 3.66E-10 | 2.29E-08 | ENSG00000071054 | 2 | 101697706 | 101894690 |
| ENST00000324348 | 87.5132805 | 1.443986769 | 0.35556 | 4.061159 | 4.88E-05 | 0.000811009 | ENSG00000179859 | 17 | 7913338 | 7916289 |

| | | | | |
|---|---|---|---|---|
| GRAMD2A | protein_coding | Yes | NM_001012642.3 | 108 |
| ACO1 | protein_coding | Yes | NM_002197.3 | 108 |
| CD34 | protein_coding | Yes | NM_001025109.2 | 108 |
| CARHSP1 | protein_coding | Yes | NM_014316.4 | 108 |
| HOXB9 | protein_coding | Yes | NM_024017.5 | 108 |
| KRT17 | protein_coding | Yes | NM_000422.3 | 108 |
| MMP14 | protein_coding | Yes | NM_004995.4 | 108 |
| CPT1B | protein_coding | Yes | NM_152246.3 | 108 |
| CST6 | protein_coding | Yes | NM_001323.4 | 108 |
| CLCF1 | protein_coding | Yes | NM_013246.3 | 108 |
| FOSL1 | protein_coding | Yes | NM_005438.5 | 108 |
| BACE1 | protein_coding | Yes | NM_012104.6 | 108 |
| ARHGAP45 | protein_coding | Yes | NM_012292.5 | 108 |
| CDK5R1 | protein_coding | Yes | NM_003885.3 | 108 |
| POC1B | protein_coding | Yes | NM_172240.3 | 108 |
| PCDHB9 | protein_coding | Yes | NM_019119.5 | 108 |
| LARP4B | protein_coding | Yes | NM_015155.3 | 108 |
| CTDP1-DT | lncRNA | Yes | - | 108 |
| TTC39C | protein_coding | Yes | NM_001135993.2 | 108 |
| CD276 | protein_coding | Yes | NM_001024736.2 | 108 |
| MEOX1 | protein_coding | Yes | NM_004527.4 | 108 |
| B3GNT3 | protein_coding | Yes | NM_014256.4 | 108 |
| NRIP1 | protein_coding | Yes | NM_003489.4 | 108 |
| SLC25A20 | protein_coding | Yes | NM_000387.6 | 108 |
| ETV4 | protein_coding | Yes | NM_001079675.5 | 108 |
| ELL3 | protein_coding | Yes | NM_025165.3 | 108 |
| NT5DC1 | protein_coding | Yes | NM_152729.3 | 108 |
| MACIR | protein_coding | Yes | NM_033211.4 | 108 |
| RNF144A | protein_coding | Yes | NM_014746.6 | 108 |
| CLPTM1L | protein_coding | Yes | NM_030782.5 | 108 |
| CRTAP | protein_coding | Yes | NM_006371.5 | 108 |
| FKBP10 | protein_coding | Yes | NM_021939.4 | 108 |
| WNT11 | protein_coding | Yes | NM_004626.3 | 108 |
| POGLUT3 | protein_coding | Yes | NM_153705.5 | 108 |
| NDRG1 | protein_coding | Yes | NM_006096.4 | 108 |
| EIF4A2 | protein_coding | Yes | NM_001967.4 | 108 |
| PRX | protein_coding | Yes | NM_181882.3 | 108 |
| PLXND1 | protein_coding | Yes | NM_015103.3 | 108 |
| MAP4K4 | protein_coding | Yes | NM_001395002.1 | 108 |
| RNF227 | protein_coding | Yes | NM_001358699.2 | 108 |

| | | | | | | | | | | |
|---|---|---|---|---|---|---|---|---|---|---|
| ENST00000324765 | 209.525749 | -3.29123577 | 0.28193 | -11.674 | 1.73E-31 | 1.29E-28 | ENSG00000147202 | X | 96684841 | 97604997 |
| ENST00000325102 | 121.419033 | 1.093937833 | 0.301575 | 3.627415 | 0.000286273 | 0.00355036 | ENSG00000125457 | 17 | 75266227 | 75271231 |
| ENST00000325888 | 12.9600743 | -2.730118631 | 0.98969 | -2.75856 | 0.005805665 | 0.038690185 | ENSG00000128591 | 7 | 128830405 | 128859272 |
| ENST00000326134 | 929.083612 | -1.016427557 | 0.157754 | -6.44311 | 1.17E-10 | 7.99E-09 | ENSG00000179832 | 8 | 144148015 | 144261926 |
| ENST00000326427 | 338.903745 | -1.787113446 | 0.235733 | -7.58109 | 3.43E-14 | 4.06E-12 | ENSG00000135916 | 2 | 230864941 | 230879248 |
| ENST00000327111 | 15.6823268 | -3.282060724 | 0.951625 | -3.4489 | 0.000562872 | 0.006137625 | ENSG00000175745 | 5 | 93583221 | 93594611 |
| ENST00000327347 | 1239.26275 | 1.975450951 | 0.141802 | 13.93106 | 4.10E-44 | 1.09E-40 | ENSG00000086475 | 10 | 13317427 | 13348293 |
| ENST00000327490 | 68.3802725 | 1.267280389 | 0.389494 | 3.253659 | 0.001139288 | 0.010952342 | ENSG00000185262 | 17 | 76265347 | 76271298 |
| ENST00000327669 | 226.515847 | -1.0002413 | 0.234948 | -4.25729 | 2.07E-05 | 0.000392713 | ENSG00000184731 | 2 | 38813 | 46505 |
| ENST00000327741 | 840.741814 | -1.33469775 | 0.151252 | -8.82431 | 1.10E-18 | 2.26E-16 | ENSG00000205426 | 12 | 52285912 | 52291534 |
| ENST00000327773 | 421.162616 | -1.526214063 | 0.20086 | -7.59838 | 3.00E-14 | 3.57E-12 | ENSG00000183087 | 13 | 113820548 | 113864076 |
| ENST00000328459 | 76.168919 | 1.130665278 | 0.37058 | 3.051072 | 0.002280262 | 0.018802827 | ENSG00000165914 | 14 | 90524563 | 90816430 |
| ENST00000329078 | 382.785119 | -3.582054393 | 0.22205 | -16.1317 | 1.53E-58 | 1.05E-54 | ENSG00000183018 | 17 | 4498880 | 4539035 |
| ENST00000329363 | 36.3829904 | 1.928958433 | 0.553904 | 3.48248 | 0.000496792 | 0.005548785 | ENSG00000177989 | 22 | 50530425 | 50532498 |
| ENST00000329608 | 54.2320427 | -1.220037927 | 0.443091 | -2.75347 | 0.005896726 | 0.039153731 | ENSG00000184371 | 1 | 109910848 | 109930992 |
| ENST00000329875 | 985.863812 | -1.230979557 | 0.162607 | -7.57029 | 3.72E-14 | 4.40E-12 | ENSG00000183010 | 17 | 81932390 | 81937300 |
| ENST00000329962 | 63.8671339 | -2.817445937 | 0.446617 | -6.30842 | 2.82E-10 | 1.80E-08 | ENSG00000182272 | 11 | 369498 | 382117 |
| ENST00000330055 | 252.390654 | -1.062418096 | 0.220636 | -4.81524 | 1.47E-06 | 4.14E-05 | ENSG00000184160 | 4 | 3766384 | 3768526 |
| ENST00000330651 | 121.475743 | -1.518874667 | 0.309191 | -4.91241 | 9.00E-07 | 2.71E-05 | ENSG00000185386 | 22 | 50263712 | 50270380 |
| ENST00000330714 | 52.7091454 | 3.916581492 | 0.571964 | 6.847599 | 7.51E-12 | 6.49E-10 | ENSG00000183486 | 21 | 41362026 | 41409393 |
| ENST00000330722 | 20.7722304 | -6.376206817 | 1.575752 | -4.04645 | 5.20E-05 | 0.000853841 | ENSG00000205420 | 12 | 52487175 | 52493257 |
| ENST00000330794 | 68.9549853 | 2.129652208 | 0.410643 | 5.186143 | 2.15E-07 | 7.63E-06 | ENSG00000184584 | 5 | 139475532 | 139482758 |
| ENST00000330889 | 30.1536978 | 1.582959843 | 0.582093 | 2.719426 | 0.006539526 | 0.042455652 | ENSG00000184060 | 17 | 30921944 | 30959322 |
| ENST00000330891 | 9.64102127 | 5.225864396 | 1.721664 | 3.035358 | 0.002402507 | 0.019575874 | ENSG00000127325 | 12 | 69653608 | 69699303 |
| ENST00000331366 | 77.5600519 | 3.159775414 | 0.432464 | 7.30645 | 2.74E-13 | 2.90E-11 | ENSG00000139117 | 12 | 38652202 | 38905591 |
| ENST00000331872 | 1152.00934 | 1.461571888 | 0.191119 | 7.64744 | 2.05E-14 | 2.51E-12 | ENSG00000135821 | 1 | 182378097 | 182391790 |
| ENST00000332145 | 4.26697111 | -5.544915273 | 2.022592 | -2.74149 | 0.006116115 | 0.040284792 | ENSG00000182950 | 15 | 75724040 | 75727688 |
| ENST00000332585 | 50.8138459 | 1.27487117 | 0.453152 | 2.813341 | 0.004902957 | 0.034021322 | ENSG00000184792 | 22 | 30694873 | 30907813 |
| ENST00000333050 | 98.4234954 | -1.13328989 | 0.328374 | -3.45122 | 0.000558057 | 0.006097195 | ENSG00000184857 | 16 | 8795179 | 8797639 |
| ENST00000333437 | 30.3395867 | 3.177166691 | 0.674678 | 4.70916 | 2.49E-06 | 6.47E-05 | ENSG00000182459 | 17 | 82359246 | 82363775 |
| ENST00000333467 | 789.819783 | 1.458941391 | 0.153182 | 9.524249 | 1.66E-21 | 4.42E-19 | ENSG00000179750 | 22 | 38982346 | 38992779 |
| ENST00000333482 | 4.57101623 | -5.642314047 | 1.996969 | -2.82544 | 0.004721582 | 0.033094212 | ENSG00000249021 | 5 | 115691461 | 115692167 |
| ENST00000334062 | 28.7784493 | -8.299112121 | 1.544422 | -5.3736 | 7.72E-08 | 3.10E-06 | ENSG00000185989 | 13 | 113977782 | 114132623 |
| ENST00000334166 | 13.5939527 | 5.732451991 | 1.637153 | 3.501476 | 0.000462688 | 0.005248101 | ENSG00000187123 | 2 | 149330586 | 149474138 |
| ENST00000334186 | 260.904523 | -1.180678751 | 0.220237 | -5.36094 | 8.28E-08 | 3.30E-06 | ENSG00000177380 | 19 | 49119543 | 49151026 |
| ENST00000335174 | 37.5650334 | -1.376715935 | 0.518726 | -2.65403 | 0.007953601 | 0.049269913 | ENSG00000186352 | 4 | 185396840 | 185400241 |
| ENST00000335181 | 18213.6983 | -1.041874157 | 0.348642 | -2.98838 | 0.002804625 | 0.022066001 | ENSG00000067225 | 15 | 72199028 | 72231190 |
| ENST00000335678 | 8.73312734 | -6.57918312 | 1.752004 | -3.75523 | 0.00017318 | 0.002322279 | ENSG00000185974 | 13 | 113667218 | 113737736 |
| ENST00000336119 | 39.299653 | -1.529429098 | 0.521491 | -2.9328 | 0.003359218 | 0.025362538 | ENSG00000162711 | 1 | 247416172 | 247448817 |
| ENST00000336174 | 73.5609396 | 1.214852055 | 0.38133 | 3.185828 | 0.001443403 | 0.013203178 | ENSG00000266173 | 17 | 63702831 | 63741799 |

| | | | | |
|---|---|---|---|---|
| DIAPH2 | protein_coding | Yes | NM_006729.5 | 108 |
| MIF4GD | protein_coding | Yes | NM_001370592.1 | 108 |
| FLNC | protein_coding | Yes | NM_001458.5 | 108 |
| MROH1 | protein_coding | Yes | NM_032450.3 | 108 |
| ITM2C | protein_coding | Yes | NM_030926.6 | 108 |
| NR2F1 | protein_coding | Yes | NM_005654.6 | 108 |
| SEPHS1 | protein_coding | Yes | NM_012247.5 | 108 |
| UBALD2 | protein_coding | Yes | NM_182565.4 | 108 |
| FAM110C | protein_coding | Yes | NM_001077710.3 | 108 |
| KRT81 | protein_coding | Yes | NM_002281.4 | 108 |
| GAS6 | protein_coding | Yes | NM_000820.4 | 108 |
| TTC7B | protein_coding | Yes | NM_001010854.2 | 108 |
| SPNS2 | protein_coding | Yes | NM_001124758.3 | 108 |
| ODF3B | protein_coding | Yes | NM_001014440.4 | 108 |
| CSF1 | protein_coding | Yes | NM_000757.6 | 108 |
| PYCR1 | protein_coding | Yes | NM_006907.4 | 108 |
| B4GALNT4 | protein_coding | Yes | NM_178537.5 | 108 |
| ADRA2C | protein_coding | Yes | NM_000683.4 | 108 |
| MAPK11 | protein_coding | Yes | NM_002751.7 | 108 |
| MX2 | protein_coding | Yes | NM_002463.2 | 108 |
| KRT6A | protein_coding | Yes | NM_005554.4 | 108 |
| STING1 | protein_coding | Yes | NM_198282.4 | 108 |
| ADAP2 | protein_coding | Yes | NM_018404.3 | 108 |
| BEST3 | protein_coding | Yes | NM_032735.3 | 108 |
| CPNE8 | protein_coding | Yes | NM_153634.3 | 108 |
| GLUL | protein_coding | Yes | NM_001033044.4 | 108 |
| ODF3L1 | protein_coding | Yes | NM_175881.5 | 108 |
| OSBP2 | protein_coding | Yes | NM_030758.4 | 108 |
| TMEM186 | protein_coding | Yes | NM_015421.4 | 108 |
| TEX19 | protein_coding | Yes | NM_207459.4 | 108 |
| APOBEC3B | protein_coding | Yes | NM_004900.5 | 108 |
| - | lncRNA | Yes | - | 108 |
| RASA3 | protein_coding | Yes | NM_007368.4 | 108 |
| LYPD6 | protein_coding | Yes | NM_194317.5 | 108 |
| PPFIA3 | protein_coding | Yes | NM_003660.4 | 108 |
| ANKRD37 | protein_coding | Yes | NM_181726.4 | 108 |
| PKM | protein_coding | Yes | NM_002654.6 | 108 |
| GRK1 | protein_coding | Yes | NM_002929.3 | 108 |
| NLRP3 | protein_coding | Yes | NM_001243133.2 | 108 |
| STRADA | protein_coding | Yes | NM_001003787.4 | 108 |

| | | | | | | | | | | |
|---|---|---|---|---|---|---|---|---|---|---|
| ENST00000336334 | 48.1821703 | 2.269283004 | 0.486291 | 4.666513 | 3.06E-06 | 7.73E-05 | ENSG00000167754 | 19 | 50943302 | 50953038 |
| ENST00000336374 | 17.26569 | -2.727712065 | 0.857976 | -3.17924 | 0.001476615 | 0.01343461 | ENSG00000160870 | 7 | 99705035 | 99735196 |
| ENST00000336395 | 521.114906 | -1.298531881 | 0.175852 | -7.38424 | 1.53E-13 | 1.67E-11 | ENSG00000107140 | 9 | 35605261 | 35610033 |
| ENST00000336689 | 69.520957 | -1.289523387 | 0.389781 | -3.30833 | 0.000938538 | 0.009346479 | ENSG00000088280 | 1 | 23428562 | 23484179 |
| ENST00000337619 | 1745.08893 | -1.235344296 | 0.138519 | -8.91824 | 4.74E-19 | 9.96E-17 | ENSG00000106367 | 7 | 101154475 | 101161276 |
| ENST00000337702 | 223.51841 | 1.230867843 | 0.248269 | 4.957807 | 7.13E-07 | 2.20E-05 | ENSG00000113638 | 5 | 40711575 | 40755961 |
| ENST00000337910 | 1660.22302 | 1.029056205 | 0.361941 | 2.84316 | 0.004466857 | 0.03167757 | ENSG00000072422 | 10 | 60869437 | 60944185 |
| ENST00000337975 | 160.205813 | -1.095916946 | 0.280648 | -3.90496 | 9.42E-05 | 0.001401448 | ENSG00000183655 | 15 | 85759325 | 85794925 |
| ENST00000338037 | 795.961521 | 1.438596992 | 0.207594 | 6.929845 | 4.21E-12 | 3.83E-10 | ENSG00000182621 | 20 | 8132265 | 8884900 |
| ENST00000338343 | 624.793715 | -1.057624245 | 0.164359 | -6.43483 | 1.24E-10 | 8.42E-09 | ENSG00000099364 | 16 | 30923571 | 30948783 |
| ENST00000338380 | 54.9353595 | -1.838472477 | 0.455322 | -4.03774 | 5.40E-05 | 0.000880513 | ENSG00000124107 | 20 | 45252238 | 45254564 |
| ENST00000338415 | 6.05973497 | -6.049979257 | 1.870544 | -3.23434 | 0.001219233 | 0.011578734 | ENSG00000115828 | 2 | 37344629 | 37373322 |
| ENST00000338483 | 328.867576 | -1.228850292 | 0.21329 | -5.7614 | 8.34E-09 | 4.02E-07 | ENSG00000185022 | 22 | 38201993 | 38216507 |
| ENST00000338523 | 306.887875 | 1.104481376 | 0.213369 | 5.17639 | 2.26E-07 | 7.98E-06 | ENSG00000086300 | 7 | 26291861 | 26374383 |
| ENST00000339464 | 97.8529323 | -1.174956568 | 0.329056 | -3.57069 | 0.000356045 | 0.004254047 | ENSG00000188064 | 22 | 45920365 | 45977162 |
| ENST00000339732 | 24.1811072 | -1.782552155 | 0.658506 | -2.70696 | 0.006790151 | 0.0437091 | ENSG00000131386 | 3 | 16174679 | 16230165 |
| ENST00000339852 | 8.03022039 | 4.956713692 | 1.75521 | 2.824 | 0.004742843 | 0.033170931 | ENSG00000188505 | 19 | 39196963 | 39201884 |
| ENST00000340093 | 627.82311 | -1.039997579 | 0.16306 | -6.37801 | 1.79E-10 | 1.18E-08 | ENSG00000011422 | 19 | 43648578 | 43670169 |
| ENST00000340126 | 1141.29761 | 1.131735344 | 0.187443 | 6.037762 | 1.56E-09 | 8.76E-08 | ENSG00000067066 | 2 | 230416200 | 230545606 |
| ENST00000340249 | 14.9939673 | -2.966743007 | 0.943668 | -3.14384 | 0.001667449 | 0.01475947 | ENSG00000291004 | 15 | 22610352 | 22617854 |
| ENST00000340368 | 508.522855 | -1.004620304 | 0.179745 | -5.58916 | 2.28E-08 | 1.02E-06 | ENSG00000186480 | 7 | 155297877 | 155310235 |
| ENST00000340607 | 120.68279 | 1.94843052 | 0.311937 | 6.246233 | 4.20E-10 | 2.61E-08 | ENSG00000148344 | 9 | 129738348 | 129753042 |
| ENST00000340958 | 2491.86513 | -1.60014528 | 0.128317 | -12.4702 | 1.09E-35 | 1.10E-32 | ENSG00000189143 | 7 | 73830995 | 73832690 |
| ENST00000340967 | 30.208447 | 1.787123114 | 0.5897 | 3.030563 | 0.002440979 | 0.019835871 | ENSG00000177076 | 9 | 19409008 | 19452505 |
| ENST00000341184 | 222.793968 | -2.101345233 | 0.249399 | -8.42563 | 3.59E-17 | 6.09E-15 | ENSG00000128268 | 22 | 39457011 | 39492194 |
| ENST00000341259 | 126.33671 | -1.35223928 | 0.296962 | -4.55357 | 5.27E-06 | 0.000122537 | ENSG00000111252 | 12 | 111405922 | 111451623 |
| ENST00000341469 | 170.909524 | 1.435617315 | 0.271628 | 5.285227 | 1.26E-07 | 4.74E-06 | ENSG00000106785 | 9 | 98084352 | 98119222 |
| ENST00000341744 | 283.606919 | -1.470490482 | 0.259225 | -5.67264 | 1.41E-08 | 6.50E-07 | ENSG00000124225 | 20 | 57648395 | 57710015 |
| ENST00000341864 | 180.304016 | 1.28006701 | 0.260759 | 4.908999 | 9.15E-07 | 2.75E-05 | ENSG00000187325 | X | 78129747 | 78139650 |
| ENST00000341948 | 10.2834674 | 4.280432032 | 1.352868 | 3.163969 | 0.001556334 | 0.014007286 | ENSG00000187372 | 5 | 141213918 | 141218979 |
| ENST00000342435 | 1115.65735 | 2.226827585 | 0.202217 | 11.01208 | 3.34E-28 | 1.95E-25 | ENSG00000188313 | 3 | 146515179 | 146544607 |
| ENST00000342456 | 61.7514945 | 3.071117993 | 0.484948 | 6.332878 | 2.41E-10 | 1.55E-08 | ENSG00000006534 | 11 | 68010326 | 68029276 |
| ENST00000343959 | 67.5795155 | -2.66855152 | 0.429659 | -6.21086 | 5.27E-10 | 3.21E-08 | ENSG00000188100 | 10 | 87020293 | 87024730 |
| ENST00000344293 | 82.3832648 | 1.019188119 | 0.362056 | 2.815004 | 0.004877664 | 0.033897375 | ENSG00000165632 | 10 | 7818504 | 8016631 |
| ENST00000344320 | 403.706132 | -1.462347423 | 0.202673 | -7.2153 | 5.38E-13 | 5.51E-11 | ENSG00000188277 | 15 | 40769979 | 40772449 |
| ENST00000344363 | 23.5714566 | 7.993540763 | 1.563982 | 5.111018 | 3.20E-07 | 1.08E-05 | ENSG00000221880 | 17 | 41033883 | 41034874 |
| ENST00000344646 | 108.380384 | 1.503364727 | 0.336737 | 4.464505 | 8.03E-06 | 0.000175934 | ENSG00000172671 | 10 | 45615499 | 45672772 |
| ENST00000344754 | 5.83957784 | -5.995698975 | 1.890982 | -3.17068 | 0.001520823 | 0.013761437 | ENSG00000088827 | 20 | 3686969 | 3712600 |
| ENST00000345136 | 15712.8633 | -1.328397912 | 0.125409 | -10.5925 | 3.23E-26 | 1.29E-23 | ENSG00000178209 | 8 | 143915152 | 143939597 |
| ENST00000345146 | 2490.59847 | 1.159740877 | 0.157156 | 7.379541 | 1.59E-13 | 1.72E-11 | ENSG00000138413 | 2 | 208236228 | 208255071 |

| | | | | |
|---|---|---|---|---|
| KLK5 | protein_coding | Yes | NM_012427.5 | 108 |
| CYP3A7 | protein_coding | Yes | NM_000765.5 | 108 |
| TESK1 | protein_coding | Yes | NM_006285.3 | 108 |
| ASAP3 | protein_coding | Yes | NM_017707.4 | 108 |
| AP1S1 | protein_coding | Yes | NM_001283.5 | 108 |
| TTC33 | protein_coding | Yes | NM_012382.3 | 108 |
| RHOBTB1 | protein_coding | Yes | NM_014836.5 | 108 |
| KLHL25 | protein_coding | Yes | NM_022480.4 | 108 |
| PLCB1 | protein_coding | Yes | NM_015192.4 | 108 |
| FBXL19 | protein_coding | Yes | NM_001382779.1 | 108 |
| SLPI | protein_coding | Yes | NM_003064.4 | 108 |
| QPCT | protein_coding | Yes | NM_012413.4 | 108 |
| MAFF | protein_coding | Yes | NM_012323.4 | 108 |
| SNX10 | protein_coding | Yes | NM_013322.3 | 108 |
| WNT7B | protein_coding | Yes | NM_058238.3 | 108 |
| GALNT15 | protein_coding | Yes | NM_054110.5 | 108 |
| NCCRP1 | protein_coding | Yes | NM_001001414.2 | 108 |
| PLAUR | protein_coding | Yes | NM_002659.4 | 108 |
| SP100 | protein_coding | Yes | NM_001080391.2 | 108 |
| GOLGA8IP | lncRNA | Yes | - | 108 |
| INSIG1 | protein_coding | Yes | NM_005542.6 | 108 |
| PTGES | protein_coding | Yes | NM_004878.5 | 108 |
| CLDN4 | protein_coding | Yes | NM_001305.5 | 108 |
| ACER2 | protein_coding | Yes | NM_001010887.3 | 108 |
| MGAT3 | protein_coding | Yes | NM_002409.5 | 108 |
| SH2B3 | protein_coding | Yes | NM_005475.3 | 108 |
| TRIM14 | protein_coding | Yes | NM_014788.4 | 108 |
| PMEPA1 | protein_coding | Yes | NM_020182.5 | 108 |
| TAF9B | protein_coding | Yes | NM_015975.5 | 108 |
| PCDHB13 | protein_coding | Yes | NM_018933.4 | 108 |
| PLSCR1 | protein_coding | Yes | NM_021105.3 | 108 |
| ALDH3B1 | protein_coding | Yes | NM_000694.4 | 108 |
| FAM25A | protein_coding | Yes | NM_001146157.3 | 108 |
| TAF3 | protein_coding | Yes | NM_031923.4 | 108 |
| C15orf62 | protein_coding | Yes | NM_001130448.3 | 108 |
| KRTAP1-3 | protein_coding | Yes | NM_030966.2 | 108 |
| ZFAND4 | protein_coding | Yes | NM_174890.4 | 108 |
| SIGLEC1 | protein_coding | Yes | NM_023068.4 | 108 |
| PLEC | protein_coding | Yes | NM_201384.3 | 108 |
| IDH1 | protein_coding | Yes | NM_005896.4 | 108 |

| | | | | | | | | | | |
|---|---|---|---|---|---|---|---|---|---|---|
| ENST00000345264 | 401.419063 | 1.637658867 | 0.18985 | 8.626068 | 6.35E-18 | 1.16E-15 | ENSG00000148484 | 10 | 16590610 | 16817424 |
| ENST00000345988 | 5268.91645 | -1.007441213 | 0.113474 | -8.87818 | 6.80E-19 | 1.42E-16 | ENSG00000118898 | 16 | 4882506 | 4937148 |
| ENST00000346872 | 4.46615624 | -5.612620884 | 2.01042 | -2.79177 | 0.00524213 | 0.03572226 | ENSG00000161405 | 17 | 39757717 | 39864312 |
| ENST00000347055 | 160.659523 | 1.025874929 | 0.278154 | 3.68816 | 0.000225881 | 0.002912273 | ENSG00000172086 | 2 | 88027204 | 88055783 |
| ENST00000348335 | 58.0578066 | -1.745825757 | 0.437103 | -3.99409 | 6.49E-05 | 0.001027688 | ENSG00000004660 | 17 | 3860314 | 3893053 |
| ENST00000348831 | 240.512272 | -1.431769821 | 0.226879 | -6.31071 | 2.78E-10 | 1.77E-08 | ENSG00000197381 | 21 | 45074579 | 45226560 |
| ENST00000348993 | 350.187682 | -1.095039354 | 0.196537 | -5.57167 | 2.52E-08 | 1.12E-06 | ENSG00000291159 | 15 | 84631450 | 84647478 |
| ENST00000349485 | 415.604169 | -1.198365197 | 0.184164 | -6.50707 | 7.66E-11 | 5.43E-09 | ENSG00000186866 | 21 | 45263934 | 45287895 |
| ENST00000351298 | 121.704645 | 2.253459665 | 0.316536 | 7.119133 | 1.09E-12 | 1.07E-10 | ENSG00000075651 | 3 | 171600403 | 171810483 |
| ENST00000353704 | 1380.12594 | -1.096111715 | 0.170859 | -6.41529 | 1.41E-10 | 9.47E-09 | ENSG00000107175 | 9 | 35732665 | 35736999 |
| ENST00000354190 | 72.5404745 | 1.534808569 | 0.490801 | 3.127153 | 0.00176508 | 0.015424012 | ENSG00000021300 | 11 | 73646580 | 73662819 |
| ENST00000354532 | 526.859452 | -1.322847009 | 0.171524 | -7.7123 | 1.24E-14 | 1.58E-12 | ENSG00000076928 | 19 | 41883183 | 41907452 |
| ENST00000354919 | 283.46254 | 1.316188108 | 0.213821 | 6.155565 | 7.48E-10 | 4.43E-08 | ENSG00000152455 | 10 | 14878865 | 14904315 |
| ENST00000354957 | 221.704716 | 1.194998117 | 0.235277 | 5.079115 | 3.79E-07 | 1.26E-05 | ENSG00000198093 | 19 | 51889234 | 51905017 |
| ENST00000354960 | 48.7669239 | 1.263769901 | 0.45987 | 2.748103 | 0.005994109 | 0.039667894 | ENSG00000138735 | 4 | 119494402 | 119628804 |
| ENST00000355029 | 1213.55095 | 1.353997821 | 0.146094 | 9.267998 | 1.90E-20 | 4.67E-18 | ENSG00000173848 | 10 | 5412556 | 5459056 |
| ENST00000355522 | 131.982657 | -2.132326383 | 0.359856 | -5.9255 | 3.11E-09 | 1.63E-07 | ENSG00000198535 | 15 | 62066976 | 62070917 |
| ENST00000355716 | 112.339337 | 1.508841274 | 0.321721 | 4.689909 | 2.73E-06 | 7.03E-05 | ENSG00000157873 | 1 | 2556370 | 2563829 |
| ENST00000355897 | 58.3174554 | 2.129441644 | 0.459793 | 4.631303 | 3.63E-06 | 8.96E-05 | ENSG00000173890 | 3 | 170037994 | 170085392 |
| ENST00000355994 | 67.2476666 | -1.323714291 | 0.417079 | -3.17377 | 0.001504711 | 0.013643418 | ENSG00000197971 | 18 | 76978832 | 77132783 |
| ENST00000356031 | 23.9580485 | 3.100212348 | 0.743142 | 4.171764 | 3.02E-05 | 0.000542024 | ENSG00000152582 | 5 | 35617862 | 35814611 |
| ENST00000356082 | 726.861305 | -1.882074099 | 0.159332 | -11.8123 | 3.37E-32 | 2.65E-29 | ENSG00000196878 | 1 | 209614869 | 209652425 |
| ENST00000356083 | 111.35545 | 1.937233659 | 0.324516 | 5.969607 | 2.38E-09 | 1.28E-07 | ENSG00000196739 | 9 | 114155536 | 114312511 |
| ENST00000356189 | 517.618638 | 1.311679512 | 0.223675 | 5.864208 | 4.51E-09 | 2.29E-07 | ENSG00000176244 | 10 | 15075474 | 15088776 |
| ENST00000356524 | 207.414867 | -1.475700773 | 0.241445 | -6.11195 | 9.84E-10 | 5.71E-08 | ENSG00000173432 | 11 | 18266263 | 18269967 |
| ENST00000356660 | 118.290059 | 1.104189995 | 0.305628 | 3.612853 | 0.000302846 | 0.003719109 | ENSG00000176697 | 11 | 27654892 | 27700455 |
| ENST00000356708 | 2141.64559 | 1.499157258 | 0.125499 | 11.94557 | 6.85E-33 | 5.72E-30 | ENSG00000165629 | 10 | 7788176 | 7807801 |
| ENST00000356736 | 406.492344 | -1.342276913 | 0.187316 | -7.16585 | 7.73E-13 | 7.78E-11 | ENSG00000138821 | 4 | 102261665 | 102345482 |
| ENST00000356839 | 851.26068 | 1.807279711 | 0.161156 | 11.2145 | 3.46E-29 | 2.27E-26 | ENSG00000072778 | 17 | 7219937 | 7225266 |
| ENST00000356955 | 2372.31473 | -1.017093902 | 0.123273 | -8.25074 | 1.57E-16 | 2.43E-14 | ENSG00000143537 | 1 | 155051315 | 155062775 |
| ENST00000357077 | 142.067983 | 1.792175942 | 0.312155 | 5.741303 | 9.40E-09 | 4.48E-07 | ENSG00000145362 | 4 | 113049652 | 113383736 |
| ENST00000357447 | 142.956376 | 1.262413974 | 0.289719 | 4.357378 | 1.32E-05 | 0.000267707 | ENSG00000151474 | 10 | 13643705 | 14330924 |
| ENST00000357484 | 197.988371 | 1.247128065 | 0.244489 | 5.100964 | 3.38E-07 | 1.13E-05 | ENSG00000242498 | 15 | 89895005 | 89912952 |
| ENST00000357578 | 208.297495 | 1.151349096 | 0.240117 | 4.794947 | 1.63E-06 | 4.51E-05 | ENSG00000112294 | 6 | 24494968 | 24537207 |
| ENST00000357604 | 1339.81935 | 1.062076015 | 0.13962 | 7.606889 | 2.81E-14 | 3.36E-12 | ENSG00000151461 | 10 | 11920021 | 12042809 |
| ENST00000357681 | 289.71411 | 1.244251091 | 0.229607 | 5.419038 | 5.99E-08 | 2.47E-06 | ENSG00000058668 | 1 | 203626831 | 203744081 |
| ENST00000357700 | 92.5878385 | 1.614267024 | 0.34631 | 4.661342 | 3.14E-06 | 7.90E-05 | ENSG00000196372 | 10 | 5638866 | 5666595 |
| ENST00000357727 | 26.6249731 | 1.851432622 | 0.627111 | 2.95232 | 0.003153957 | 0.024156737 | ENSG00000146592 | 7 | 28412517 | 28825894 |
| ENST00000357877 | 100.064634 | 1.128187157 | 0.326787 | 3.452365 | 0.000555696 | 0.006078227 | ENSG00000135622 | 2 | 74654246 | 74683841 |
| ENST00000358056 | 504.320092 | 1.174099247 | 0.174221 | 6.739155 | 1.59E-11 | 1.29E-09 | ENSG00000197045 | 14 | 54474484 | 54488980 |

| | | | | |
|---|---|---|---|---|
| RSU1 | protein_coding | Yes | NM_012425.4 | 108 |
| PPL | protein_coding | Yes | NM_002705.5 | 108 |
| IKZF3 | protein_coding | Yes | NM_012481.5 | 108 |
| KRCC1 | protein_coding | Yes | NM_016618.3 | 108 |
| CAMKK1 | protein_coding | Yes | NM_032294.3 | 108 |
| ADARB1 | protein_coding | Yes | NM_001112.4 | 108 |
| - | lncRNA | Yes | - | 108 |
| POFUT2 | protein_coding | Yes | NM_133635.6 | 108 |
| PLD1 | protein_coding | Yes | NM_002662.5 | 108 |
| CREB3 | protein_coding | Yes | NM_006368.5 | 108 |
| PLEKHB1 | protein_coding | Yes | NM_021200.3 | 108 |
| ARHGEF1 | protein_coding | Yes | NM_004706.4 | 108 |
| SUV39H2 | protein_coding | Yes | NM_001193424.2 | 108 |
| ZNF649 | protein_coding | Yes | NM_023074.4 | 108 |
| PDE5A | protein_coding | Yes | NM_001083.4 | 108 |
| NET1 | protein_coding | Yes | NM_001047160.3 | 108 |
| C2CD4A | protein_coding | Yes | NM_207322.3 | 108 |
| TNFRSF14 | protein_coding | Yes | NM_003820.4 | 108 |
| GPR160 | protein_coding | Yes | NM_014373.3 | 108 |
| MBP | protein_coding | Yes | NM_001025101.2 | 108 |
| SPEF2 | protein_coding | Yes | NM_024867.4 | 108 |
| LAMB3 | protein_coding | Yes | NM_000228.3 | 108 |
| COL27A1 | protein_coding | Yes | NM_032888.4 | 108 |
| ACBD7 | protein_coding | Yes | NM_001039844.3 | 108 |
| SAA1 | protein_coding | Yes | NM_199161.5 | 108 |
| BDNF | protein_coding | Yes | NM_001709.5 | 108 |
| ATP5F1C | protein_coding | Yes | NM_001001973.3 | 108 |
| SLC39A8 | protein_coding | Yes | NM_001135146.2 | 108 |
| ACADVL | protein_coding | Yes | NM_000018.4 | 108 |
| ADAM15 | protein_coding | Yes | NM_207197.3 | 108 |
| ANK2 | protein_coding | Yes | NM_001148.6 | 108 |
| FRMD4A | protein_coding | Yes | NM_018027.5 | 108 |
| ARPIN | protein_coding | Yes | NM_182616.4 | 108 |
| ALDH5A1 | protein_coding | Yes | NM_001080.3 | 108 |
| UPF2 | protein_coding | Yes | NM_015542.4 | 108 |
| ATP2B4 | protein_coding | Yes | NM_001684.5 | 108 |
| ASB13 | protein_coding | Yes | NM_024701.4 | 108 |
| CREB5 | protein_coding | Yes | NM_182898.4 | 108 |
| SEMA4F | protein_coding | Yes | NM_004263.5 | 108 |
| GMFB | protein_coding | Yes | NM_004124.3 | 108 |

| | | | | | | | | | | |
|---|---|---|---|---|---|---|---|---|---|---|
| ENST00000358171 | 740.362958 | -1.016990364 | 0.192084 | -5.2945 | 1.19E-07 | 4.55E-06 | ENSG00000149257 | 11 | 75562252 | 75572783 |
| ENST00000358173 | 375.113684 | -1.457506707 | 0.19273 | -7.56242 | 3.96E-14 | 4.65E-12 | ENSG00000196411 | 7 | 100802564 | 100827523 |
| ENST00000358196 | 54.1322742 | 1.236584389 | 0.442427 | 2.795004 | 0.005189909 | 0.035533185 | ENSG00000128683 | 2 | 170816886 | 170861151 |
| ENST00000358495 | 75.9339028 | 1.304401241 | 0.374012 | 3.48759 | 0.000487396 | 0.005464436 | ENSG00000002016 | 12 | 911735 | 949694 |
| ENST00000358771 | 53.6466262 | -1.728977852 | 0.445736 | -3.87893 | 0.000104917 | 0.001532151 | ENSG00000198053 | 20 | 1895406 | 1940592 |
| ENST00000358789 | 79.345643 | -1.195629906 | 0.398009 | -3.00403 | 0.002664297 | 0.02121485 | ENSG00000129451 | 19 | 51012738 | 51019709 |
| ENST00000358901 | 16665.5666 | -1.221741822 | 0.346792 | -3.52298 | 0.000426724 | 0.004924502 | ENSG00000165280 | 9 | 35056063 | 35072625 |
| ENST00000359062 | 161.611149 | -9.344092272 | 1.466463 | -6.37186 | 1.87E-10 | 1.23E-08 | ENSG00000172572 | 12 | 20368536 | 20688583 |
| ENST00000359204 | 113.435485 | 1.350697813 | 0.314298 | 4.2975 | 1.73E-05 | 0.000336586 | ENSG00000148690 | 10 | 93667882 | 93702592 |
| ENST00000359315 | 52.3091255 | -1.230116945 | 0.440032 | -2.79552 | 0.005181627 | 0.035492404 | ENSG00000141933 | 19 | 507496 | 519654 |
| ENST00000359396 | 17.9475319 | 2.617132357 | 0.80827 | 3.237945 | 0.001203942 | 0.011466656 | ENSG00000165125 | 7 | 142871207 | 142885745 |
| ENST00000359467 | 896.242401 | 1.323993746 | 0.151558 | 8.735869 | 2.42E-18 | 4.69E-16 | ENSG00000196693 | 10 | 42589108 | 42638570 |
| ENST00000359470 | 508.665163 | -2.356144893 | 0.499001 | -4.72172 | 2.34E-06 | 6.13E-05 | ENSG00000182795 | 1 | 207018521 | 207032756 |
| ENST00000359534 | 14.6437383 | 3.148031473 | 0.984617 | 3.197215 | 0.001387612 | 0.01280523 | ENSG00000164626 | 6 | 39188970 | 39229475 |
| ENST00000359543 | 333.522219 | 1.702675756 | 0.223975 | 7.602069 | 2.91E-14 | 3.48E-12 | ENSG00000213853 | 16 | 10528421 | 10580598 |
| ENST00000359742 | 109.506115 | -1.121291072 | 0.312732 | -3.58547 | 0.000336475 | 0.004059052 | ENSG00000155974 | 12 | 66347430 | 66679076 |
| ENST00000359775 | 84.9389017 | -1.010574971 | 0.35581 | -2.84021 | 0.004508378 | 0.031917011 | ENSG00000175048 | 6 | 157381189 | 157678157 |
| ENST00000359988 | 8.93378477 | -5.11979184 | 1.727663 | -2.96342 | 0.003042405 | 0.023477239 | ENSG00000197893 | 10 | 113588713 | 113664041 |
| ENST00000360004 | 57.3268809 | 1.784698267 | 0.45634 | 3.910895 | 9.20E-05 | 0.001373243 | ENSG00000196126 | 6 | 32578774 | 32589848 |
| ENST00000360273 | 7.10813946 | -6.27933177 | 1.81974 | -3.45067 | 0.000559187 | 0.006104486 | ENSG00000157087 | 3 | 10324022 | 10505586 |
| ENST00000360476 | 34.1215762 | -2.109304945 | 0.573928 | -3.67521 | 0.000237658 | 0.003043385 | ENSG00000197696 | 15 | 84655131 | 84658199 |
| ENST00000360490 | 214.114189 | 2.819504518 | 0.311134 | 9.062018 | 1.28E-19 | 2.82E-17 | ENSG00000196549 | 3 | 155080318 | 155183704 |
| ENST00000360564 | 43.4876062 | -2.267673693 | 0.512854 | -4.42168 | 9.79E-06 | 0.000208559 | ENSG00000198598 | 12 | 131828392 | 131851771 |
| ENST00000360570 | 203.228247 | 1.12435436 | 0.24224 | 4.641488 | 3.46E-06 | 8.59E-05 | ENSG00000158234 | 3 | 138608770 | 138633376 |
| ENST00000360631 | 21.5689709 | -6.432427025 | 1.600546 | -4.0189 | 5.85E-05 | 0.000941493 | ENSG00000133083 | 13 | 35768651 | 36131382 |
| ENST00000360774 | 39.2352647 | -1.5768657 | 0.529437 | -2.97838 | 0.002897725 | 0.022625339 | ENSG00000119121 | 9 | 74722494 | 74887921 |
| ENST00000360779 | 258.409614 | -1.457908302 | 0.224682 | -6.48876 | 8.65E-11 | 6.06E-09 | ENSG00000125775 | 20 | 1309908 | 1329139 |
| ENST00000360803 | 1169.2032 | 1.13246614 | 0.159532 | 7.098687 | 1.26E-12 | 1.23E-10 | ENSG00000107937 | 10 | 988433 | 1019932 |
| ENST00000361099 | 4952.44007 | 1.818119668 | 0.167163 | 10.87632 | 1.49E-27 | 7.76E-25 | ENSG00000115415 | 2 | 190969148 | 191014171 |
| ENST00000361143 | 62.708023 | 1.569058105 | 0.410837 | 3.819177 | 0.000133898 | 0.001872325 | ENSG00000114779 | 3 | 51968514 | 51974049 |
| ENST00000361157 | 4311.98429 | 1.171658668 | 0.359511 | 3.259038 | 0.001117909 | 0.010772854 | ENSG00000126709 | 1 | 27666063 | 27672192 |
| ENST00000361226 | 443.972812 | -1.655897441 | 0.188834 | -8.76906 | 1.80E-18 | 3.56E-16 | ENSG00000198853 | 9 | 35490110 | 35561895 |
| ENST00000361271 | 204.436257 | 2.21938789 | 0.252476 | 8.790487 | 1.49E-18 | 3.00E-16 | ENSG00000165996 | 10 | 17589031 | 17617374 |
| ENST00000361807 | 37.1465345 | 1.4907748 | 0.529743 | 2.814147 | 0.004890688 | 0.033962896 | ENSG00000198298 | 10 | 43606437 | 43617904 |
| ENST00000361842 | 85.0053854 | 9.842971978 | 1.482519 | 6.639358 | 3.15E-11 | 2.41E-09 | ENSG00000132530 | 17 | 6756045 | 6775647 |
| ENST00000361866 | 125.746066 | -2.951526199 | 0.331868 | -8.89367 | 5.91E-19 | 1.24E-16 | ENSG00000142156 | 21 | 45981769 | 46005048 |
| ENST00000361910 | 68.4287789 | -9.54844547 | 1.488327 | -6.41556 | 1.40E-10 | 9.46E-09 | ENSG00000198960 | X | 101615124 | 101618000 |
| ENST00000362007 | 44.8412965 | 3.93657804 | 0.632804 | 6.220851 | 4.94E-10 | 3.03E-08 | ENSG00000198715 | 1 | 156292687 | 156295679 |
| ENST00000362077 | 178.078245 | -1.117330495 | 0.281061 | -3.97541 | 7.03E-05 | 0.001095243 | ENSG00000272657 | 21 | 34073591 | 34360033 |
| ENST00000362091 | 1148.92218 | 1.244163341 | 0.139035 | 8.948593 | 3.60E-19 | 7.69E-17 | ENSG00000134452 | 10 | 5890242 | 5937593 |

| | | | | |
|---|---|---|---|---|
| SERPINH1 | protein_coding | Yes | NM_001235.5 | 108 |
| EPHB4 | protein_coding | Yes | NM_004444.5 | 108 |
| GAD1 | protein_coding | Yes | NM_000817.3 | 108 |
| RAD52 | protein_coding | Yes | NM_134424.4 | 108 |
| SIRPA | protein_coding | Yes | NM_001040023.2 | 108 |
| KLK10 | protein_coding | Yes | NM_145888.3 | 108 |
| VCP | protein_coding | Yes | NM_007126.5 | 108 |
| PDE3A | protein_coding | Yes | NM_000921.5 | 108 |
| FRA10AC1 | protein_coding | Yes | NM_145246.5 | 108 |
| TPGS1 | protein_coding | Yes | NM_033513.3 | 108 |
| TRPV6 | protein_coding | Yes | NM_018646.6 | 108 |
| ZNF33B | protein_coding | Yes | NM_006955.3 | 108 |
| C1orf116 | protein_coding | Yes | NM_023938.6 | 108 |
| KCNK5 | protein_coding | Yes | NM_003740.4 | 108 |
| EMP2 | protein_coding | Yes | NM_001424.6 | 108 |
| GRIP1 | protein_coding | Yes | NM_001366722.1 | 108 |
| ZDHHC14 | protein_coding | Yes | NM_024630.3 | 108 |
| NRAP | protein_coding | Yes | NM_198060.4 | 108 |
| HLA-DRB1 | protein_coding | Yes | NM_002124.4 | 108 |
| ATP2B2 | protein_coding | Yes | NM_001001331.4 | 108 |
| NMB | protein_coding | Yes | NM_021077.4 | 108 |
| MME | protein_coding | Yes | NM_007289.4 | 108 |
| MMP17 | protein_coding | Yes | NM_016155.7 | 108 |
| FAIM | protein_coding | Yes | NM_001033031.2 | 108 |
| DCLK1 | protein_coding | Yes | NM_001330071.2 | 108 |
| TRPM6 | protein_coding | Yes | NM_017662.5 | 108 |
| SDCBP2 | protein_coding | Yes | NM_080489.5 | 108 |
| GTPBP4 | protein_coding | Yes | NM_012341.3 | 108 |
| STAT1 | protein_coding | Yes | NM_007315.4 | 108 |
| ABHD14B | protein_coding | Yes | NM_001146314.2 | 108 |
| IFI6 | protein_coding | Yes | NM_002038.4 | 108 |
| RUSC2 | protein_coding | Yes | NM_014806.5 | 108 |
| HACD1 | protein_coding | Yes | NM_014241.4 | 108 |
| ZNF485 | protein_coding | Yes | NM_145312.4 | 108 |
| XAF1 | protein_coding | Yes | NM_017523.5 | 108 |
| COL6A1 | protein_coding | Yes | NM_001848.3 | 108 |
| ARMCX6 | protein_coding | Yes | NM_019007.4 | 108 |
| GLMP | protein_coding | Yes | NM_144580.3 | 108 |
| - | lncRNA | Yes | - | 108 |
| FBH1 | protein_coding | Yes | NM_178150.3 | 108 |

| | | | | | | | | | | |
|---|---|---|---|---|---|---|---|---|---|---|
| ENST00000364043 | 52.1354601 | 1.435360247 | 0.445851 | 3.219373 | 0.001284714 | 0.012054391 | ENSG00000200913 | 1 | 44776489 | 44776593 |
| ENST00000364488 | 66.1117247 | -1.376743742 | 0.396931 | -3.46847 | 0.000523429 | 0.005788547 | ENSG00000201358 | 14 | 50068567 | 50068873 |
| ENST00000366376 | 4.46615624 | -5.612620884 | 2.01042 | -2.79177 | 0.00524213 | 0.03572226 | ENSG00000203565 | 10 | 33684754 | 33709868 |
| ENST00000366592 | 74.3018628 | 1.909734089 | 0.39403 | 4.846672 | 1.26E-06 | 3.61E-05 | ENSG00000077585 | 1 | 236142538 | 236208907 |
| ENST00000367162 | 174.599504 | 1.177438099 | 0.257032 | 4.580907 | 4.63E-06 | 0.000109634 | ENSG00000133059 | 1 | 205142504 | 205211702 |
| ENST00000367463 | 81.1686549 | -3.105406185 | 0.412532 | -7.52767 | 5.17E-14 | 5.99E-12 | ENSG00000111962 | 6 | 148747029 | 149076990 |
| ENST00000367468 | 19.5203166 | -3.141566515 | 0.821074 | -3.82617 | 0.000130155 | 0.00183015 | ENSG00000073756 | 1 | 186671790 | 186680423 |
| ENST00000367660 | 174.351145 | 1.062372317 | 0.255834 | 4.152579 | 3.29E-05 | 0.00058226 | ENSG00000146386 | 6 | 139028744 | 139043302 |
| ENST00000367815 | 1875.0072 | -1.162809628 | 0.147349 | -7.89152 | 2.99E-15 | 4.08E-13 | ENSG00000143153 | 1 | 169106689 | 169132719 |
| ENST00000368440 | 65.7171434 | -2.454071964 | 0.428616 | -5.72557 | 1.03E-08 | 4.88E-07 | ENSG00000172594 | 6 | 122789257 | 122809720 |
| ENST00000368476 | 14.5395971 | -3.160805901 | 0.97176 | -3.25266 | 0.001143295 | 0.010983874 | ENSG00000160716 | 1 | 154567777 | 154580013 |
| ENST00000368564 | 193.349227 | 1.001307666 | 0.251252 | 3.985267 | 6.74E-05 | 0.001058486 | ENSG00000196911 | 6 | 116681210 | 116741867 |
| ENST00000368685 | 112.535621 | 1.169783274 | 0.309558 | 3.778886 | 0.000157531 | 0.002144079 | ENSG00000143553 | 1 | 153658702 | 153661852 |
| ENST00000368708 | 15.1556253 | -2.364201584 | 0.866822 | -2.72744 | 0.006382833 | 0.041694467 | ENSG00000196754 | 1 | 153561107 | 153565844 |
| ENST00000368713 | 100.882914 | -7.070715893 | 0.908814 | -7.78016 | 7.24E-15 | 9.45E-13 | ENSG00000188015 | 1 | 153547328 | 153549258 |
| ENST00000368716 | 724.816864 | -2.044437684 | 0.161507 | -12.6585 | 1.00E-36 | 1.12E-33 | ENSG00000196154 | 1 | 153543620 | 153545806 |
| ENST00000368738 | 62.9256382 | -2.796924467 | 0.453434 | -6.16832 | 6.90E-10 | 4.13E-08 | ENSG00000163220 | 1 | 153357853 | 153361023 |
| ENST00000368799 | 15.349946 | -5.922070864 | 1.618663 | -3.65862 | 0.000253579 | 0.003208689 | ENSG00000143631 | 1 | 152302164 | 152325239 |
| ENST00000368836 | 120.746108 | 1.143235935 | 0.30007 | 3.809898 | 0.000139024 | 0.001929685 | ENSG00000203791 | 10 | 124757833 | 124791887 |
| ENST00000368851 | 117.878971 | 1.371604606 | 0.305862 | 4.484394 | 7.31E-06 | 0.000162347 | ENSG00000112394 | 6 | 111087502 | 111231194 |
| ENST00000368904 | 326.467776 | -1.254124257 | 0.207634 | -6.04008 | 1.54E-09 | 8.64E-08 | ENSG00000286088 | 10 | 122832166 | 122855773 |
| ENST00000368908 | 147.843934 | -1.02202997 | 0.27561 | -3.70825 | 0.000208698 | 0.002720673 | ENSG00000163155 | 1 | 151159747 | 151165902 |
| ENST00000369458 | 294.08005 | 2.730860007 | 0.298104 | 9.160765 | 5.15E-20 | 1.20E-17 | ENSG00000134258 | 1 | 117143586 | 117210927 |
| ENST00000369516 | 147.292513 | -2.103549612 | 0.28767 | -7.31238 | 2.62E-13 | 2.79E-11 | ENSG00000134198 | 1 | 115048010 | 115089503 |
| ENST00000369534 | 105.711672 | 1.587600113 | 0.32448 | 4.892748 | 9.94E-07 | 2.95E-05 | ENSG00000130830 | X | 154778683 | 154805485 |
| ENST00000369684 | 230.973844 | -1.161357834 | 0.241214 | -4.81464 | 1.47E-06 | 4.15E-05 | ENSG00000134245 | 1 | 112508964 | 112530165 |
| ENST00000369909 | 171.036688 | 1.040619817 | 0.257603 | 4.039633 | 5.35E-05 | 0.000875153 | ENSG00000134222 | 1 | 109279555 | 109283145 |
| ENST00000369940 | 34.2519587 | 1.759742514 | 0.556403 | 3.162711 | 0.001563076 | 0.014058756 | ENSG00000146243 | 6 | 78867550 | 78903102 |
| ENST00000369977 | 3526.59895 | 1.217866452 | 0.145303 | 8.381566 | 5.22E-17 | 8.73E-15 | ENSG00000196586 | 6 | 75749238 | 75919537 |
| ENST00000370035 | 475.951396 | 1.553683299 | 0.184004 | 8.443768 | 3.07E-17 | 5.28E-15 | ENSG00000162636 | 1 | 108560099 | 108639322 |
| ENST00000370078 | 112.068533 | 1.443897805 | 0.313508 | 4.605616 | 4.11E-06 | 9.95E-05 | ENSG00000198890 | 1 | 107056673 | 107059294 |
| ENST00000370096 | 14.9778136 | 2.903027136 | 0.912371 | 3.18185 | 0.001463375 | 0.013346289 | ENSG00000060718 | 1 | 102876472 | 103108522 |
| ENST00000370206 | 2675.9804 | -1.10505534 | 0.165195 | -6.68939 | 2.24E-11 | 1.75E-09 | ENSG00000117519 | 1 | 94896956 | 94927110 |
| ENST00000370278 | 10.6636326 | -5.383443906 | 1.685826 | -3.19336 | 0.001406286 | 0.012937638 | ENSG00000221867 | X | 152698793 | 152702347 |
| ENST00000370747 | 723.218402 | 6.770452183 | 0.583457 | 11.60403 | 3.93E-31 | 2.87E-28 | ENSG00000137965 | 1 | 78649830 | 78664073 |
| ENST00000370751 | 1389.65324 | 7.122233487 | 0.708519 | 10.05229 | 8.98E-24 | 2.90E-21 | ENSG00000137959 | 1 | 78620447 | 78646145 |
| ENST00000370859 | 457.679712 | 1.313802616 | 0.21827 | 6.019161 | 1.75E-09 | 9.71E-08 | ENSG00000137968 | 1 | 75202128 | 75611114 |
| ENST00000370982 | 477.277304 | -1.184176229 | 0.177682 | -6.66457 | 2.65E-11 | 2.05E-09 | ENSG00000172380 | 1 | 67701474 | 67833467 |
| ENST00000370992 | 1332.93551 | -1.099777552 | 0.378891 | -2.90262 | 0.003700498 | 0.027342328 | ENSG00000052749 | 10 | 97356700 | 97401340 |
| ENST00000371026 | 69.1094186 | 1.280468441 | 0.395204 | 3.240021 | 0.001195209 | 0.011395697 | ENSG00000152763 | 1 | 66812884 | 66924856 |

| | | | | |
|---|---|---|---|---|
| SNORD46 | snoRNA | Yes | - | 108 |
| RN7SKP193 | misc_RNA | Yes | - | 108 |
| - | lncRNA | Yes | - | 108 |
| GPR137B | protein_coding | Yes | NM_003272.4 | 108 |
| DSTYK | protein_coding | Yes | NM_015375.3 | 108 |
| UST | protein_coding | Yes | NM_005715.3 | 108 |
| PTGS2 | protein_coding | Yes | NM_000963.4 | 108 |
| ABRACL | protein_coding | Yes | NM_021243.3 | 108 |
| ATP1B1 | protein_coding | Yes | NM_001677.4 | 108 |
| SMPDL3A | protein_coding | Yes | NM_006714.5 | 108 |
| CHRNB2 | protein_coding | Yes | NM_000748.3 | 108 |
| KPNA5 | protein_coding | Yes | NM_001366306.2 | 108 |
| SNAPIN | protein_coding | Yes | NM_012437.6 | 108 |
| S100A2 | protein_coding | Yes | NM_005978.4 | 108 |
| S100A3 | protein_coding | Yes | NM_002960.2 | 108 |
| S100A4 | protein_coding | Yes | NM_002961.3 | 108 |
| S100A9 | protein_coding | Yes | NM_002965.4 | 108 |
| FLG | protein_coding | Yes | NM_002016.2 | 108 |
| EEF1AKMT2 | protein_coding | Yes | NM_212554.4 | 108 |
| SLC16A10 | protein_coding | Yes | NM_018593.5 | 108 |
| - | protein_coding | Yes | - | 108 |
| LYSMD1 | protein_coding | Yes | NM_212551.5 | 108 |
| VTCN1 | protein_coding | Yes | NM_024626.4 | 108 |
| TSPAN2 | protein_coding | Yes | NM_005725.6 | 108 |
| MPP1 | protein_coding | Yes | NM_002436.4 | 108 |
| WNT2B | protein_coding | Yes | NM_024494.3 | 108 |
| PSRC1 | protein_coding | Yes | NM_001032291.3 | 108 |
| IRAK1BP1 | protein_coding | Yes | NM_001010844.4 | 108 |
| MYO6 | protein_coding | Yes | NM_004999.4 | 108 |
| FAM102B | protein_coding | Yes | NM_001010883.3 | 108 |
| PRMT6 | protein_coding | Yes | NM_018137.3 | 108 |
| COL11A1 | protein_coding | Yes | NM_001854.4 | 108 |
| CNN3 | protein_coding | Yes | NM_001839.5 | 108 |
| MAGEA3 | protein_coding | Yes | NM_005362.4 | 108 |
| IFI44 | protein_coding | Yes | NM_006417.5 | 108 |
| IFI44L | protein_coding | Yes | NM_006820.4 | 108 |
| SLC44A5 | protein_coding | Yes | NM_001130058.2 | 108 |
| GNG12 | protein_coding | Yes | NM_018841.6 | 108 |
| RRP12 | protein_coding | Yes | NM_015179.4 | 108 |
| DNAI4 | protein_coding | Yes | NM_024763.5 | 108 |

| | | | | | | | | | | |
|---|---|---|---|---|---|---|---|---|---|---|
| ENST00000371121 | 497.914499 | -5.175537827 | 0.257393 | -20.1075 | 6.34E-90 | 2.40E-85 | ENSG00000102038 | X | 129446505 | 129523490 |
| ENST00000371204 | 19.0989643 | 6.230020429 | 1.581491 | 3.939335 | 8.17E-05 | 0.001243565 | ENSG00000134716 | 1 | 59893307 | 59926773 |
| ENST00000371205 | 30.3151342 | 1.536240077 | 0.579344 | 2.651689 | 0.008009018 | 0.049552173 | ENSG00000138185 | 10 | 95756162 | 95877266 |
| ENST00000371253 | 36.4356006 | -3.243967809 | 0.61349 | -5.28773 | 1.24E-07 | 4.69E-06 | ENSG00000153292 | 6 | 46997707 | 47042332 |
| ENST00000371269 | 214.630884 | 1.200112477 | 0.242062 | 4.957873 | 7.13E-07 | 2.20E-05 | ENSG00000116133 | 1 | 54849626 | 54887195 |
| ENST00000371384 | 275.964741 | 1.190115984 | 0.223587 | 5.322841 | 1.02E-07 | 3.97E-06 | ENSG00000124098 | 20 | 56358973 | 56368663 |
| ENST00000371410 | 82.7027448 | 1.017603239 | 0.354181 | 2.873114 | 0.004064476 | 0.029386607 | ENSG00000101882 | X | 119920671 | 119943751 |
| ENST00000371655 | 1457.76906 | -1.395228573 | 0.177418 | -7.86408 | 3.72E-15 | 5.03E-13 | ENSG00000169213 | 1 | 51907955 | 51990700 |
| ENST00000371697 | 588.746494 | -2.900489418 | 0.859815 | -3.37339 | 0.000742488 | 0.007701642 | ENSG00000148677 | 10 | 90912095 | 90921087 |
| ENST00000371703 | 237.212308 | -1.219806421 | 0.232515 | -5.24615 | 1.55E-07 | 5.72E-06 | ENSG00000148688 | 10 | 90871973 | 90902191 |
| ENST00000371795 | 983.495314 | 1.66217942 | 0.206729 | 8.04036 | 8.96E-16 | 1.29E-13 | ENSG00000152778 | 10 | 89414567 | 89420997 |
| ENST00000371804 | 3240.15208 | 2.552982071 | 0.412247 | 6.192842 | 5.91E-10 | 3.58E-08 | ENSG00000185745 | 10 | 89392622 | 89406487 |
| ENST00000371818 | 605.118022 | 4.196023095 | 0.251817 | 16.66298 | 2.44E-62 | 3.58E-58 | ENSG00000119917 | 10 | 89327996 | 89340968 |
| ENST00000371826 | 1013.55979 | 2.390713147 | 0.195896 | 12.20402 | 2.96E-34 | 2.74E-31 | ENSG00000119922 | 10 | 89302045 | 89309271 |
| ENST00000371850 | 80.806105 | -2.245132464 | 0.388305 | -5.78188 | 7.39E-09 | 3.60E-07 | ENSG00000160293 | 9 | 133761893 | 133992324 |
| ENST00000371930 | 432.82239 | -1.061328978 | 0.186739 | -5.68348 | 1.32E-08 | 6.13E-07 | ENSG00000152766 | 10 | 88819895 | 88851844 |
| ENST00000372017 | 17.7936371 | -2.176978248 | 0.779086 | -2.79427 | 0.005201671 | 0.035586154 | ENSG00000173267 | 10 | 86958598 | 86963258 |
| ENST00000372201 | 203.138024 | -1.11364248 | 0.253954 | -4.38521 | 1.16E-05 | 0.000240055 | ENSG00000173846 | 1 | 44800376 | 44805990 |
| ENST00000372271 | 69.0116073 | 1.289642401 | 0.390645 | 3.301319 | 0.000962313 | 0.009535494 | ENSG00000126882 | 9 | 131258077 | 131276510 |
| ENST00000372338 | 92.3685762 | -1.739171318 | 0.350223 | -4.9659 | 6.84E-07 | 2.12E-05 | ENSG00000130720 | 9 | 130902439 | 130939247 |
| ENST00000372431 | 60.0181226 | -1.90813522 | 0.427449 | -4.46401 | 8.04E-06 | 0.000176166 | ENSG00000100979 | 20 | 45898621 | 45912155 |
| ENST00000372517 | 107.649899 | 1.196913952 | 0.318561 | 3.757255 | 0.000171787 | 0.002306333 | ENSG00000164010 | 1 | 42817121 | 42844991 |
| ENST00000372625 | 377.457326 | 1.036276403 | 0.209511 | 4.946162 | 7.57E-07 | 2.33E-05 | ENSG00000172465 | X | 103628974 | 103630953 |
| ENST00000372706 | 44.2109905 | 1.624447581 | 0.488231 | 3.327208 | 0.000877208 | 0.008838224 | ENSG00000187815 | 1 | 40477289 | 40496343 |
| ENST00000372816 | 32.874344 | 2.013437138 | 0.573218 | 3.512517 | 0.000443883 | 0.005074855 | ENSG00000116990 | 1 | 39895427 | 39901917 |
| ENST00000372980 | 62.9691415 | -1.610276859 | 0.421903 | -3.8167 | 0.000135249 | 0.001886329 | ENSG00000149596 | 20 | 44106589 | 44187188 |
| ENST00000372989 | 421.968244 | 2.251311612 | 0.203999 | 11.03589 | 2.56E-28 | 1.53E-25 | ENSG00000102362 | X | 100674490 | 100732121 |
| ENST00000373004 | 7.52056565 | -4.882325189 | 1.764332 | -2.76724 | 0.005653353 | 0.037897402 | ENSG00000102359 | X | 100644198 | 100675788 |
| ENST00000373233 | 321.024097 | 1.651550189 | 0.209924 | 7.867374 | 3.62E-15 | 4.91E-13 | ENSG00000124177 | 20 | 41402082 | 41618377 |
| ENST00000373247 | 435.584897 | -1.079458316 | 0.204386 | -5.28146 | 1.28E-07 | 4.83E-06 | ENSG00000136877 | 9 | 127802881 | 127814081 |
| ENST00000373327 | 93.2407281 | 1.067199428 | 0.336076 | 3.175472 | 0.001495931 | 0.013576026 | ENSG00000204104 | 2 | 238320517 | 238400897 |
| ENST00000373383 | 12.3402501 | 3.94644316 | 1.174087 | 3.361288 | 0.000775799 | 0.007985241 | ENSG00000094841 | X | 75274084 | 75304885 |
| ENST00000373394 | 220.636454 | 1.411527365 | 0.235747 | 5.987455 | 2.13E-09 | 1.16E-07 | ENSG00000131269 | X | 75051047 | 75156283 |
| ENST00000373509 | 323.598698 | -1.321243336 | 0.212253 | -6.22485 | 4.82E-10 | 2.96E-08 | ENSG00000137193 | 6 | 37170151 | 37175428 |
| ENST00000373510 | 133.940576 | 1.05468599 | 0.289248 | 3.646309 | 0.000266034 | 0.003344926 | ENSG00000160062 | 1 | 32539426 | 32605941 |
| ENST00000373658 | 12.9805987 | -5.673982358 | 1.63405 | -3.47234 | 0.000515935 | 0.00571865 | ENSG00000121753 | 1 | 31727116 | 31764340 |
| ENST00000373672 | 56.4302268 | -1.915971144 | 0.456888 | -4.19352 | 2.75E-05 | 0.000498907 | ENSG00000084636 | 1 | 31652262 | 31704017 |
| ENST00000373709 | 111.862526 | -1.10457299 | 0.309386 | -3.57021 | 0.000356699 | 0.00426085 | ENSG00000168528 | 1 | 31413212 | 31434678 |
| ENST00000373883 | 222.438506 | -1.360735506 | 0.23409 | -5.81288 | 6.14E-09 | 3.03E-07 | ENSG00000188760 | 2 | 219544027 | 219550595 |
| ENST00000374212 | 530.383707 | -1.110906111 | 0.171431 | -6.48021 | 9.16E-11 | 6.39E-09 | ENSG00000136868 | 9 | 113221543 | 113264492 |

| | | | | |
|---|---|---|---|---|
| SMARCA1 | protein_coding | Yes | NM_001282874.2 | 108 |
| CYP2J2 | protein_coding | Yes | NM_000775.4 | 108 |
| ENTPD1 | protein_coding | Yes | NM_001776.6 | 108 |
| ADGRF1 | protein_coding | Yes | NM_153840.4 | 108 |
| DHCR24 | protein_coding | Yes | NM_014762.4 | 108 |
| FAM210B | protein_coding | Yes | NM_080821.3 | 108 |
| NKAP | protein_coding | Yes | NM_024528.4 | 108 |
| RAB3B | protein_coding | Yes | NM_002867.4 | 108 |
| ANKRD1 | protein_coding | Yes | NM_014391.3 | 108 |
| RPP30 | protein_coding | Yes | NM_006413.5 | 108 |
| IFIT5 | protein_coding | Yes | NM_012420.3 | 108 |
| IFIT1 | protein_coding | Yes | NM_001548.5 | 108 |
| IFIT3 | protein_coding | Yes | NM_001549.6 | 108 |
| IFIT2 | protein_coding | Yes | NM_001547.5 | 108 |
| VAV2 | protein_coding | Yes | NM_001134398.2 | 108 |
| ANKRD22 | protein_coding | Yes | NM_144590.3 | 108 |
| SNCG | protein_coding | Yes | NM_003087.3 | 108 |
| PLK3 | protein_coding | Yes | NM_004073.4 | 108 |
| FAM78A | protein_coding | Yes | NM_033387.4 | 108 |
| FIBCD1 | protein_coding | Yes | NM_032843.5 | 108 |
| PLTP | protein_coding | Yes | NM_006227.4 | 108 |
| ERMAP | protein_coding | Yes | NM_001017922.2 | 108 |
| TCEAL1 | protein_coding | Yes | NM_004780.3 | 108 |
| ZFP69 | protein_coding | Yes | NM_001320179.2 | 108 |
| MYCL | protein_coding | Yes | NM_001033081.3 | 108 |
| JPH2 | protein_coding | Yes | NM_020433.5 | 108 |
| SYTL4 | protein_coding | Yes | NM_001370165.1 | 108 |
| SRPX2 | protein_coding | Yes | NM_014467.3 | 108 |
| CHD6 | protein_coding | Yes | NM_032221.5 | 108 |
| FPGS | protein_coding | Yes | NM_004957.6 | 108 |
| TRAF3IP1 | protein_coding | Yes | NM_015650.4 | 108 |
| UPRT | protein_coding | Yes | NM_145052.4 | 108 |
| ABCB7 | protein_coding | Yes | NM_001271696.3 | 108 |
| PIM1 | protein_coding | Yes | NM_002648.4 | 108 |
| ZBTB8A | protein_coding | Yes | NM_001040441.3 | 108 |
| ADGRB2 | protein_coding | Yes | NM_001364857.2 | 108 |
| COL16A1 | protein_coding | Yes | NM_001856.4 | 108 |
| SERINC2 | protein_coding | Yes | NM_178865.5 | 108 |
| TMEM198 | protein_coding | Yes | NM_001005209.3 | 108 |
| SLC31A1 | protein_coding | Yes | NM_001859.4 | 108 |

| | | | | | | | | | | |
|---|---|---|---|---|---|---|---|---|---|---|
| ENST00000374433 | 69.6091221 | 1.734222745 | 0.396238 | 4.376721 | 1.20E-05 | 0.000248167 | ENSG00000169740 | 10 | 43643861 | 43648881 |
| ENST00000374446 | 20.0522811 | 2.120202522 | 0.736698 | 2.877981 | 0.004002296 | 0.029021737 | ENSG00000196793 | 10 | 43556343 | 43574616 |
| ENST00000374466 | 133.172746 | 1.131298289 | 0.290679 | 3.891919 | 9.95E-05 | 0.001465833 | ENSG00000169826 | 10 | 43138444 | 43185302 |
| ENST00000374469 | 172.06738 | 1.078013403 | 0.259724 | 4.150608 | 3.32E-05 | 0.000586402 | ENSG00000165124 | 9 | 110365247 | 110579741 |
| ENST00000374530 | 445.563698 | 3.000755172 | 0.198825 | 15.09245 | 1.82E-51 | 5.84E-48 | ENSG00000157654 | 9 | 109780197 | 110172512 |
| ENST00000374672 | 574.683132 | -1.457868445 | 0.171883 | -8.48173 | 2.22E-17 | 3.88E-15 | ENSG00000136826 | 9 | 107484851 | 107489769 |
| ENST00000374736 | 73.5858819 | 4.204043864 | 0.535739 | 7.847185 | 4.25E-15 | 5.71E-13 | ENSG00000165029 | 9 | 104781005 | 104928155 |
| ENST00000374810 | 93.2107129 | -1.423288306 | 0.3716 | -3.83016 | 0.000128057 | 0.001806201 | ENSG00000078804 | 20 | 34704343 | 34713436 |
| ENST00000374843 | 109.717975 | 1.403883979 | 0.349312 | 4.019001 | 5.84E-05 | 0.000941175 | ENSG00000204257 | 6 | 32948617 | 32953097 |
| ENST00000375078 | 65.4426852 | -2.753968428 | 0.438823 | -6.27581 | 3.48E-10 | 2.19E-08 | ENSG00000162545 | 1 | 20482390 | 20486210 |
| ENST00000375180 | 78.028343 | 1.298630786 | 0.36827 | 3.526301 | 0.000421408 | 0.004871708 | ENSG00000184083 | X | 54068323 | 54183254 |
| ENST00000375215 | 32.919457 | 1.740054622 | 0.578705 | 3.006808 | 0.00264006 | 0.021056255 | ENSG00000204323 | 17 | 75633433 | 75641404 |
| ENST00000375281 | 58.52485 | 1.340308788 | 0.419719 | 3.193349 | 0.001406326 | 0.012937638 | ENSG00000204335 | 2 | 170715336 | 170718078 |
| ENST00000375527 | 75.7730451 | 1.279649605 | 0.371648 | 3.443172 | 0.000574933 | 0.006240319 | ENSG00000204366 | 6 | 31899612 | 31902086 |
| ENST00000375533 | 89.3631523 | -1.461305718 | 0.392538 | -3.72272 | 0.000197091 | 0.002590142 | ENSG00000095739 | 10 | 28677520 | 28682932 |
| ENST00000375645 | 1701.7854 | -1.361490805 | 0.149882 | -9.08376 | 1.05E-19 | 2.34E-17 | ENSG00000068650 | 13 | 112690037 | 112887168 |
| ENST00000375754 | 323.579005 | -3.858551701 | 0.24682 | -15.6331 | 4.33E-55 | 1.87E-51 | ENSG00000165025 | 9 | 90801818 | 90898549 |
| ENST00000375766 | 94.7768329 | -2.318813943 | 0.364086 | -6.36886 | 1.90E-10 | 1.25E-08 | ENSG00000162458 | 1 | 15758794 | 15786589 |
| ENST00000376454 | 541.30838 | 1.101672055 | 0.198201 | 5.558362 | 2.72E-08 | 1.20E-06 | ENSG00000120549 | 10 | 24209137 | 24547843 |
| ENST00000376901 | 807.185065 | -1.258242201 | 0.163175 | -7.711 | 1.25E-14 | 1.59E-12 | ENSG00000174851 | 11 | 66284579 | 66289143 |
| ENST00000377275 | 1189.59854 | 1.034141665 | 0.152 | 6.803575 | 1.02E-11 | 8.62E-10 | ENSG00000165997 | 10 | 18659430 | 18681639 |
| ENST00000377294 | 31.0989384 | 1.64616382 | 0.575025 | 2.862769 | 0.004199566 | 0.030159355 | ENSG00000187626 | 6 | 28241696 | 28252269 |
| ENST00000377304 | 246.66936 | 1.12856853 | 0.225283 | 5.009562 | 5.46E-07 | 1.74E-05 | ENSG00000241058 | 10 | 18545560 | 18651587 |
| ENST00000377369 | 16.4030371 | 4.983398361 | 1.240533 | 4.017144 | 5.89E-05 | 0.000947104 | ENSG00000148482 | 10 | 17951917 | 18043285 |
| ENST00000377420 | 7.45560087 | -4.842101586 | 1.800461 | -2.68937 | 0.007158768 | 0.045519678 | ENSG00000204779 | 9 | 65282100 | 65285209 |
| ENST00000377495 | 41.3614331 | 2.627010635 | 0.541082 | 4.855106 | 1.20E-06 | 3.48E-05 | ENSG00000148483 | 10 | 17752200 | 17800868 |
| ENST00000377507 | 57.7800873 | -2.65952031 | 0.473058 | -5.62197 | 1.89E-08 | 8.52E-07 | ENSG00000049249 | 1 | 7915870 | 7940839 |
| ENST00000377524 | 653.8629 | 1.436881213 | 0.162736 | 8.829512 | 1.05E-18 | 2.16E-16 | ENSG00000136738 | 10 | 17644150 | 17716824 |
| ENST00000377669 | 341.914986 | 2.083791739 | 0.252919 | 8.238976 | 1.74E-16 | 2.66E-14 | ENSG00000118922 | 13 | 73686088 | 74133929 |
| ENST00000377693 | 17.2618496 | -3.711445982 | 0.953548 | -3.89225 | 9.93E-05 | 0.001464119 | ENSG00000204866 | 19 | 46148237 | 46161289 |
| ENST00000377697 | 160.601948 | -2.777789924 | 0.323814 | -8.57836 | 9.62E-18 | 1.73E-15 | ENSG00000204869 | 19 | 46039181 | 46041002 |
| ENST00000377799 | 37.7564092 | 2.254151537 | 0.55 | 4.098456 | 4.16E-05 | 0.000708574 | ENSG00000107614 | 10 | 17137335 | 17201672 |
| ENST00000377833 | 14.9822305 | 3.80800893 | 1.038948 | 3.665256 | 0.000247091 | 0.003142041 | ENSG00000107611 | 10 | 16823965 | 17129811 |
| ENST00000377834 | 40.7416557 | 2.319311919 | 0.533139 | 4.350292 | 1.36E-05 | 0.000275548 | ENSG00000069812 | 1 | 6415231 | 6419919 |
| ENST00000377990 | 300.292605 | 1.193062931 | 0.214757 | 5.555415 | 2.77E-08 | 1.21E-06 | ENSG00000011523 | 2 | 65056415 | 65087004 |
| ENST00000378026 | 200.417573 | -2.36617991 | 0.257481 | -9.18974 | 3.94E-20 | 9.26E-18 | ENSG00000136026 | 12 | 106237880 | 106248020 |
| ENST00000378078 | 1502.69218 | -1.041417088 | 0.135659 | -7.6767 | 1.63E-14 | 2.03E-12 | ENSG00000107185 | 9 | 35749286 | 35758585 |
| ENST00000378083 | 47.8576045 | -1.39716538 | 0.462426 | -3.02138 | 0.002516241 | 0.020279548 | ENSG00000069424 | 1 | 6045887 | 6101180 |
| ENST00000378165 | 81.8611135 | 1.032339124 | 0.356689 | 2.894231 | 0.00380088 | 0.027884993 | ENSG00000152465 | 10 | 15105769 | 15168693 |
| ENST00000378197 | 216.653713 | 1.479641984 | 0.237776 | 6.222836 | 4.88E-10 | 3.00E-08 | ENSG00000152464 | 10 | 15097354 | 15104257 |

| | | | | |
|---|---|---|---|---|
| ZNF32 | protein_coding | Yes | NM_006973.3 | 108 |
| ZNF239 | protein_coding | Yes | NM_001099282.2 | 108 |
| CSGALNACT2 | protein_coding | Yes | NM_018590.5 | 108 |
| SVEP1 | protein_coding | Yes | NM_153366.4 | 108 |
| PALM2AKAP2 | protein_coding | Yes | NM_007203.5 | 108 |
| KLF4 | protein_coding | Yes | NM_004235.6 | 108 |
| ABCA1 | protein_coding | Yes | NM_005502.4 | 108 |
| TP53INP2 | protein_coding | Yes | NM_021202.3 | 108 |
| HLA-DMA | protein_coding | Yes | NM_006120.4 | 108 |
| CAMK2N1 | protein_coding | Yes | NM_018584.6 | 108 |
| FAM120C | protein_coding | Yes | NM_017848.6 | 108 |
| SMIM5 | protein_coding | Yes | NM_001162995.3 | 108 |
| SP5 | protein_coding | Yes | NM_001003845.3 | 108 |
| ZBTB12 | protein_coding | Yes | NM_181842.3 | 108 |
| BAMBI | protein_coding | Yes | NM_012342.3 | 108 |
| ATP11A | protein_coding | Yes | NM_015205.3 | 108 |
| SYK | protein_coding | Yes | NM_003177.7 | 108 |
| FBLIM1 | protein_coding | Yes | NM_017556.4 | 108 |
| KIAA1217 | protein_coding | Yes | NM_019590.5 | 108 |
| YIF1A | protein_coding | Yes | NM_020470.3 | 108 |
| ARL5B | protein_coding | Yes | NM_178815.5 | 108 |
| ZKSCAN4 | protein_coding | Yes | NM_019110.5 | 108 |
| NSUN6 | protein_coding | Yes | NM_182543.5 | 108 |
| SLC39A12 | protein_coding | Yes | NM_001145195.2 | 108 |
| FOXD4L5 | protein_coding | Yes | NM_001126334.1 | 108 |
| TMEM236 | protein_coding | Yes | NM_001098844.3 | 108 |
| TNFRSF9 | protein_coding | Yes | NM_001561.6 | 108 |
| STAM | protein_coding | Yes | NM_003473.4 | 108 |
| KLF12 | protein_coding | Yes | - | 108 |
| IGFL2 | protein_coding | Yes | NM_001135113.2 | 108 |
| IGFL4 | protein_coding | Yes | NM_001002923.3 | 108 |
| TRDMT1 | protein_coding | Yes | NM_004412.7 | 108 |
| CUBN | protein_coding | Yes | NM_001081.4 | 108 |
| HES2 | protein_coding | Yes | NM_019089.5 | 108 |
| CEP68 | protein_coding | Yes | NM_015147.3 | 108 |
| CKAP4 | protein_coding | Yes | NM_006825.4 | 108 |
| RGP1 | protein_coding | Yes | NM_001080496.3 | 108 |
| KCNAB2 | protein_coding | Yes | NM_001199862.2 | 108 |
| NMT2 | protein_coding | Yes | NM_004808.3 | 108 |
| RPP38 | protein_coding | Yes | NM_183005.5 | 108 |

| | | | | | | | | | | |
|---|---|---|---|---|---|---|---|---|---|---|
| ENST00000378278 | 599.374789 | 1.461633549 | 0.181865 | 8.036907 | 9.21E-16 | 1.32E-13 | ENSG00000152457 | 10 | 14904612 | 14954096 |
| ENST00000378295 | 155.489519 | 1.064921424 | 0.269443 | 3.952311 | 7.74E-05 | 0.00118793 | ENSG00000078900 | 1 | 3652515 | 3736201 |
| ENST00000378372 | 285.279575 | 1.269023154 | 0.215134 | 5.898746 | 3.66E-09 | 1.89E-07 | ENSG00000187522 | 10 | 14838305 | 14871741 |
| ENST00000378473 | 298.477231 | -1.048269737 | 0.209042 | -5.01465 | 5.31E-07 | 1.70E-05 | ENSG00000101333 | 20 | 9069086 | 9480808 |
| ENST00000378555 | 56.5203645 | -2.840584615 | 0.476078 | -5.96664 | 2.42E-09 | 1.30E-07 | ENSG00000128567 | 7 | 131500270 | 131556628 |
| ENST00000378572 | 296.812914 | 1.055037066 | 0.208042 | 5.071266 | 3.95E-07 | 1.31E-05 | ENSG00000165630 | 10 | 13586964 | 13630859 |
| ENST00000378714 | 512.918854 | 1.142672156 | 0.183988 | 6.210585 | 5.28E-10 | 3.22E-08 | ENSG00000065328 | 10 | 13161557 | 13211110 |
| ENST00000378747 | 1144.3134 | 1.509568313 | 0.169018 | 8.931389 | 4.21E-19 | 8.90E-17 | ENSG00000123240 | 10 | 13100162 | 13138308 |
| ENST00000379400 | 858.665643 | -1.381248971 | 0.154814 | -8.92201 | 4.58E-19 | 9.64E-17 | ENSG00000101265 | 20 | 4780022 | 4823608 |
| ENST00000379416 | 64.5985077 | 1.114064822 | 0.404423 | 2.754699 | 0.005874622 | 0.039056727 | ENSG00000158125 | 2 | 31334320 | 31414742 |
| ENST00000379562 | 353.068808 | 1.392515085 | 0.197091 | 7.065352 | 1.60E-12 | 1.55E-10 | ENSG00000151657 | 10 | 7750961 | 7787993 |
| ENST00000379611 | 80.6659978 | 1.314351482 | 0.36385 | 3.612342 | 0.000303443 | 0.003724193 | ENSG00000164221 | 5 | 115267189 | 115296654 |
| ENST00000379613 | 182.999989 | 1.056897975 | 0.255807 | 4.131628 | 3.60E-05 | 0.000628248 | ENSG00000137203 | 6 | 10396676 | 10415074 |
| ENST00000379644 | 267.758311 | 1.436430071 | 0.223143 | 6.437251 | 1.22E-10 | 8.30E-09 | ENSG00000165102 | 8 | 43140463 | 43202855 |
| ENST00000379888 | 560.290675 | 1.787589805 | 0.172576 | 10.35828 | 3.84E-25 | 1.38E-22 | ENSG00000134453 | 10 | 6089033 | 6117447 |
| ENST00000379915 | 12.1942954 | -5.58195075 | 1.650453 | -3.38207 | 0.000719413 | 0.007517613 | ENSG00000260729 | 15 | 72284726 | 72375981 |
| ENST00000380079 | 77.5760388 | -1.801685917 | 0.404349 | -4.45577 | 8.36E-06 | 0.000182292 | ENSG00000127954 | 7 | 88270891 | 88306894 |
| ENST00000380094 | 79.8708571 | 1.652478436 | 0.372332 | 4.438184 | 9.07E-06 | 0.000195143 | ENSG00000134461 | 10 | 5861615 | 5889893 |
| ENST00000380191 | 5635.29715 | 1.540694458 | 0.113892 | 13.52762 | 1.07E-41 | 1.93E-38 | ENSG00000057608 | 10 | 5765222 | 5813434 |
| ENST00000380338 | 37.4765044 | 2.688735237 | 0.572666 | 4.695123 | 2.66E-06 | 6.87E-05 | ENSG00000171843 | 9 | 20341668 | 20622499 |
| ENST00000380625 | 46.1503788 | -8.980091216 | 1.507718 | -5.95608 | 2.58E-09 | 1.37E-07 | ENSG00000187268 | X | 13035616 | 13044620 |
| ENST00000380698 | 11.524141 | 2.729184296 | 1.018555 | 2.679466 | 0.007373976 | 0.046539565 | ENSG00000170542 | 6 | 2887269 | 2903309 |
| ENST00000380739 | 409.642142 | 1.366603392 | 0.194123 | 7.039889 | 1.92E-12 | 1.84E-10 | ENSG00000021355 | 6 | 2832331 | 2841863 |
| ENST00000381125 | 1303.80223 | 1.396247056 | 0.153803 | 9.078142 | 1.10E-19 | 2.46E-17 | ENSG00000067057 | 10 | 3067547 | 3136802 |
| ENST00000381151 | 5677.16102 | -1.253356183 | 0.11617 | -10.789 | 3.88E-27 | 1.81E-24 | ENSG00000198743 | 21 | 34073577 | 34106260 |
| ENST00000381344 | 916.630847 | 1.175268793 | 0.147321 | 7.977625 | 1.49E-15 | 2.09E-13 | ENSG00000067064 | 10 | 1039418 | 1049119 |
| ENST00000381356 | 101.999649 | 1.129542447 | 0.334305 | 3.378775 | 0.000728096 | 0.007588984 | ENSG00000205707 | 12 | 25195236 | 25205143 |
| ENST00000381604 | 1486.86984 | 1.831004619 | 0.133933 | 13.67107 | 1.51E-42 | 2.93E-39 | ENSG00000015171 | 10 | 135454 | 254637 |
| ENST00000381620 | 15.2136769 | -5.908260986 | 1.606752 | -3.67714 | 0.000235859 | 0.003023638 | ENSG00000151834 | 4 | 46243547 | 46390128 |
| ENST00000381657 | 273.497756 | -1.04360198 | 0.220895 | -4.72443 | 2.31E-06 | 6.06E-05 | ENSG00000182378 | X | 281380 | 303356 |
| ENST00000381962 | 5708.40306 | -1.025726911 | 0.140177 | -7.31739 | 2.53E-13 | 2.70E-11 | ENSG00000271303 | 20 | 646614 | 653200 |
| ENST00000382040 | 297.210083 | 6.870772598 | 0.534529 | 12.85387 | 8.18E-38 | 1.07E-34 | ENSG00000134321 | 2 | 6877776 | 6898239 |
| ENST00000382349 | 22.3986544 | -1.898866276 | 0.687488 | -2.76204 | 0.0057442 | 0.038360071 | ENSG00000205922 | 19 | 1753505 | 1780988 |
| ENST00000382723 | 629.543663 | -4.450844974 | 0.723964 | -6.14788 | 7.85E-10 | 4.64E-08 | ENSG00000163132 | 4 | 4859664 | 4863936 |
| ENST00000382771 | 434.218933 | -1.462086506 | 0.200678 | -7.28572 | 3.20E-13 | 3.37E-11 | ENSG00000206075 | 18 | 63476957 | 63505085 |
| ENST00000382848 | 126.221952 | -2.767070107 | 0.327398 | -8.45171 | 2.87E-17 | 4.95E-15 | ENSG00000165474 | 13 | 20187469 | 20192938 |
| ENST00000388913 | 2643.02376 | -1.064880537 | 0.121429 | -8.76955 | 1.79E-18 | 3.56E-16 | ENSG00000180921 | 8 | 143723932 | 143733779 |
| ENST00000389221 | 577.633152 | -1.046838292 | 0.187167 | -5.59308 | 2.23E-08 | 9.97E-07 | ENSG00000059145 | 16 | 1363204 | 1414704 |
| ENST00000389420 | 239.658365 | 1.132444347 | 0.238321 | 4.751755 | 2.02E-06 | 5.41E-05 | ENSG00000173157 | 12 | 43353865 | 43552203 |
| ENST00000389737 | 182.940556 | -1.034277852 | 0.25044 | -4.12984 | 3.63E-05 | 0.000632206 | ENSG00000155275 | 4 | 8440776 | 8476555 |

| | | | | |
|---|---|---|---|---|
| DCLRE1C | protein_coding | Yes | NM_001033855.3 | 108 |
| TP73 | protein_coding | Yes | NM_005427.4 | 108 |
| HSPA14 | protein_coding | Yes | NM_016299.4 | 108 |
| PLCB4 | protein_coding | Yes | NM_001377142.1 | 108 |
| PODXL | protein_coding | Yes | NM_001018111.3 | 108 |
| PRPF18 | protein_coding | Yes | NM_003675.4 | 108 |
| MCM10 | protein_coding | Yes | NM_018518.5 | 108 |
| OPTN | protein_coding | Yes | NM_001008212.2 | 108 |
| RASSF2 | protein_coding | Yes | NM_014737.3 | 108 |
| XDH | protein_coding | Yes | NM_000379.4 | 108 |
| KIN | protein_coding | Yes | NM_012311.4 | 108 |
| CCDC112 | protein_coding | Yes | NM_001040440.3 | 108 |
| TFAP2A | protein_coding | Yes | NM_001372066.1 | 108 |
| HGSNAT | protein_coding | Yes | NM_152419.3 | 108 |
| RBM17 | protein_coding | Yes | NM_032905.5 | 108 |
| - | protein_coding | Yes | - | 108 |
| STEAP4 | protein_coding | Yes | NM_024636.4 | 108 |
| ANKRD16 | protein_coding | Yes | NM_019046.3 | 108 |
| GDI2 | protein_coding | Yes | NM_001494.4 | 108 |
| MLLT3 | protein_coding | Yes | NM_004529.4 | 108 |
| FAM9C | protein_coding | Yes | NM_174901.6 | 108 |
| SERPINB9 | protein_coding | Yes | NM_004155.6 | 108 |
| SERPINB1 | protein_coding | Yes | NM_030666.4 | 108 |
| PFKP | protein_coding | Yes | NM_002627.5 | 108 |
| SLC5A3 | protein_coding | Yes | NM_006933.7 | 108 |
| IDI1 | protein_coding | Yes | NM_004508.4 | 108 |
| ETFRF1 | protein_coding | Yes | NM_001001660.3 | 108 |
| ZMYND11 | protein_coding | Yes | NM_001370100.5 | 108 |
| GABRA2 | protein_coding | Yes | NM_000807.4 | 108 |
| PLCXD1 | protein_coding | Yes | NM_018390.4 | 108 |
| SRXN1 | protein_coding | Yes | NM_080725.3 | 108 |
| RSAD2 | protein_coding | Yes | NM_080657.5 | 108 |
| ONECUT3 | protein_coding | Yes | NM_001080488.2 | 108 |
| MSX1 | protein_coding | Yes | NM_002448.3 | 108 |
| SERPINB5 | protein_coding | Yes | NM_002639.5 | 108 |
| GJB2 | protein_coding | Yes | NM_004004.6 | 108 |
| FAM83H | protein_coding | Yes | NM_198488.5 | 108 |
| UNKL | protein_coding | Yes | NM_001372107.1 | 108 |
| ADAMTS20 | protein_coding | Yes | NM_025003.5 | 108 |
| TRMT44 | protein_coding | Yes | NM_152544.3 | 108 |

| | | | | | | | | | | |
|---|---|---|---|---|---|---|---|---|---|---|
| ENST00000389797 | 118.170306 | -1.232887502 | 0.303405 | -4.06351 | 4.83E-05 | 0.000803548 | ENSG00000136141 | 13 | 46553169 | 46744898 |
| ENST00000390168 | 761.226363 | -1.076915384 | 0.15396 | -6.99476 | 2.66E-12 | 2.50E-10 | ENSG00000284010 | 11 | 1996758 | 1996831 |
| ENST00000390183 | 19.502513 | -2.337563008 | 0.755741 | -3.09307 | 0.001980957 | 0.016845826 | ENSG00000284191 | 7 | 151238420 | 151238538 |
| ENST00000390687 | 8.67168365 | -5.075439762 | 1.739234 | -2.9182 | 0.003520536 | 0.026291162 | ENSG00000128739 | 15 | 24954986 | 24978723 |
| ENST00000391141 | 95.7506837 | 1.319246856 | 0.39172 | 3.367832 | 0.000757617 | 0.007830089 | ENSG00000212443 | 12 | 98599634 | 98599883 |
| ENST00000392370 | 71.5885815 | 1.368072564 | 0.41872 | 3.267273 | 0.00108589 | 0.010525374 | ENSG00000064989 | 2 | 187341963 | 187448252 |
| ENST00000392583 | 134.079657 | 5.437660534 | 0.522368 | 10.40963 | 2.24E-25 | 8.22E-23 | ENSG00000111335 | 12 | 112978518 | 113011723 |
| ENST00000392644 | 46.6992537 | 1.361243786 | 0.469071 | 2.902003 | 0.003707856 | 0.027374547 | ENSG00000118690 | 6 | 108848421 | 108974476 |
| ENST00000392790 | 315.870718 | -1.332915373 | 0.212628 | -6.26875 | 3.64E-10 | 2.28E-08 | ENSG00000138161 | 10 | 122832157 | 122845857 |
| ENST00000392863 | 56.0137883 | 1.365602779 | 0.44625 | 3.060176 | 0.002212067 | 0.018365758 | ENSG00000111850 | 6 | 87322587 | 87342329 |
| ENST00000393306 | 226.493108 | -1.518334195 | 0.268654 | -5.65164 | 1.59E-08 | 7.29E-07 | ENSG00000140961 | 16 | 83953239 | 83966332 |
| ENST00000393316 | 23.8833089 | -2.819725225 | 0.726793 | -3.87968 | 0.000104593 | 0.001529186 | ENSG00000188761 | 1 | 113876815 | 113887581 |
| ENST00000393565 | 86.5625511 | -1.409267494 | 0.36583 | -3.85225 | 0.000117037 | 0.001677726 | ENSG00000113758 | 5 | 177456609 | 177473634 |
| ENST00000393658 | 17.5084662 | 2.574682111 | 0.817571 | 3.149186 | 0.001637259 | 0.014567322 | ENSG00000150625 | 4 | 175632936 | 175812264 |
| ENST00000393743 | 2110.5848 | 2.604992588 | 0.512353 | 5.084368 | 3.69E-07 | 1.23E-05 | ENSG00000137628 | 4 | 168216293 | 168318752 |
| ENST00000394009 | 35.7700378 | 1.507995698 | 0.538267 | 2.801575 | 0.005085382 | 0.034994939 | ENSG00000119636 | 14 | 74019348 | 74066092 |
| ENST00000394019 | 42.0398021 | 1.753551108 | 0.525488 | 3.336998 | 0.000846886 | 0.008585946 | ENSG00000188687 | 2 | 74216241 | 74343416 |
| ENST00000394610 | 224.575651 | 1.183652969 | 0.255373 | 4.635001 | 3.57E-06 | 8.82E-05 | ENSG00000125354 | X | 119616944 | 119693168 |
| ENST00000394621 | 51.7975125 | -1.929030817 | 0.47991 | -4.01957 | 5.83E-05 | 0.000939401 | ENSG00000157214 | 7 | 90211739 | 90237683 |
| ENST00000394685 | 88.1205257 | 4.038838488 | 0.503379 | 8.023458 | 1.03E-15 | 1.47E-13 | ENSG00000103888 | 15 | 80779369 | 80951771 |
| ENST00000394815 | 288.999468 | -1.155670864 | 0.20963 | -5.51291 | 3.53E-08 | 1.52E-06 | ENSG00000167767 | 12 | 52168995 | 52192014 |
| ENST00000394852 | 631.240222 | -1.272213407 | 0.164331 | -7.74177 | 9.80E-15 | 1.26E-12 | ENSG00000140403 | 15 | 78264561 | 78282191 |
| ENST00000395080 | 21.809168 | -2.183914946 | 0.728585 | -2.99747 | 0.002722284 | 0.021555065 | ENSG00000118785 | 4 | 87975713 | 87983411 |
| ENST00000395105 | 74.6537466 | -2.659561246 | 0.410545 | -6.47812 | 9.29E-11 | 6.46E-09 | ENSG00000137868 | 15 | 74179465 | 74202787 |
| ENST00000395145 | 71.2627194 | -1.575023128 | 0.388317 | -4.05603 | 4.99E-05 | 0.000825347 | ENSG00000165215 | 7 | 73768996 | 73770270 |
| ENST00000395323 | 465.28764 | -1.176893381 | 0.177889 | -6.61588 | 3.69E-11 | 2.78E-09 | ENSG00000213626 | 2 | 30231533 | 30260028 |
| ENST00000395348 | 19.4484357 | -2.937359455 | 0.809592 | -3.6282 | 0.000285409 | 0.003541685 | ENSG00000165449 | 10 | 59650763 | 59709850 |
| ENST00000395407 | 183.965981 | -2.507007652 | 0.270299 | -9.27495 | 1.78E-20 | 4.40E-18 | ENSG00000137819 | 15 | 69298911 | 69407780 |
| ENST00000395473 | 20.2594997 | 2.656209694 | 0.768796 | 3.455024 | 0.000550244 | 0.006029504 | ENSG00000168961 | 17 | 27631187 | 27649560 |
| ENST00000395743 | 42.4768135 | 2.977472046 | 0.556981 | 5.345733 | 9.01E-08 | 3.55E-06 | ENSG00000174808 | 4 | 74744758 | 74794523 |
| ENST00000395762 | 366.328211 | -1.008410005 | 0.191618 | -5.26259 | 1.42E-07 | 5.29E-06 | ENSG00000077238 | 16 | 27313973 | 27364778 |
| ENST00000395810 | 59.5473747 | 1.862576126 | 0.434053 | 4.291122 | 1.78E-05 | 0.000344717 | ENSG00000198915 | 10 | 43194534 | 43267065 |
| ENST00000395925 | 24.0588041 | -4.975040574 | 1.021129 | -4.8721 | 1.10E-06 | 3.23E-05 | ENSG00000106571 | 7 | 41960948 | 42237209 |
| ENST00000396005 | 29.5759514 | 4.481926823 | 0.84751 | 5.288345 | 1.23E-07 | 4.67E-06 | ENSG00000148942 | 11 | 26667019 | 26722060 |
| ENST00000396402 | 29.7212806 | -1.715277037 | 0.591224 | -2.90123 | 0.003717031 | 0.027426939 | ENSG00000137869 | 15 | 51208056 | 51338596 |
| ENST00000396410 | 3665.59937 | -1.027542843 | 0.11705 | -8.77865 | 1.65E-18 | 3.32E-16 | ENSG00000179889 | 16 | 14975025 | 15038332 |
| ENST00000396618 | 40.3037872 | -7.338868053 | 1.514418 | -4.846 | 1.26E-06 | 3.62E-05 | ENSG00000100473 | 14 | 30874558 | 30890618 |
| ENST00000396864 | 1090.31459 | 2.214803829 | 0.196909 | 11.24785 | 2.37E-29 | 1.62E-26 | ENSG00000213928 | 14 | 24161264 | 24166565 |
| ENST00000396895 | 5.71374586 | -5.967827756 | 1.900251 | -3.14055 | 0.001686324 | 0.014891661 | ENSG00000213931 | 11 | 5268344 | 5269945 |
| ENST00000396946 | 182.72964 | -2.977699115 | 0.281547 | -10.5762 | 3.84E-26 | 1.50E-23 | ENSG00000198286 | 7 | 2906141 | 3043867 |

| | | | | |
|---|---|---|---|---|
| LRCH1 | protein_coding | Yes | NM_001164211.2 | 108 |
| MIR675 | miRNA | Yes | - | 108 |
| MIR671 | miRNA | Yes | - | 108 |
| SNRPN | protein_coding | Yes | NM_003097.6 | 108 |
| SNORA53 | snoRNA | Yes | - | 108 |
| CALCRL | protein_coding | Yes | NM_005795.6 | 108 |
| OAS2 | protein_coding | Yes | NM_002535.3 | 108 |
| ARMC2 | protein_coding | Yes | NM_032131.6 | 108 |
| CUZD1 | protein_coding | Yes | NM_022034.6 | 108 |
| SMIM8 | protein_coding | Yes | NM_001042493.3 | 108 |
| OSGIN1 | protein_coding | Yes | NM_182981.3 | 108 |
| BCL2L15 | protein_coding | Yes | NM_001010922.3 | 108 |
| DBN1 | protein_coding | Yes | NM_001363541.2 | 108 |
| GPM6A | protein_coding | Yes | NM_201591.3 | 108 |
| DDX60 | protein_coding | Yes | NM_017631.6 | 108 |
| BBOF1 | protein_coding | Yes | NM_025057.3 | 108 |
| SLC4A5 | protein_coding | Yes | NM_133478.3 | 108 |
| SEPTIN6 | protein_coding | Yes | NM_145799.4 | 108 |
| STEAP2 | protein_coding | Yes | NM_001244944.2 | 108 |
| CEMIP | protein_coding | Yes | NM_001293298.2 | 108 |
| KRT80 | protein_coding | Yes | NM_182507.3 | 108 |
| DNAJA4 | protein_coding | Yes | NM_001130182.2 | 108 |
| SPP1 | protein_coding | Yes | NM_001040058.2 | 108 |
| STRA6 | protein_coding | Yes | NM_022369.4 | 108 |
| CLDN3 | protein_coding | Yes | NM_001306.4 | 108 |
| LBH | protein_coding | Yes | NM_030915.4 | 108 |
| SLC16A9 | protein_coding | Yes | NM_194298.3 | 108 |
| PAQR5 | protein_coding | Yes | NM_017705.4 | 108 |
| LGALS9 | protein_coding | Yes | NM_009587.3 | 108 |
| BTC | protein_coding | Yes | NM_001729.4 | 108 |
| IL4R | protein_coding | Yes | NM_000418.4 | 108 |
| RASGEF1A | protein_coding | Yes | NM_145313.4 | 108 |
| GLI3 | protein_coding | Yes | NM_000168.6 | 108 |
| SLC5A12 | protein_coding | Yes | NM_178498.4 | 108 |
| CYP19A1 | protein_coding | Yes | NM_000103.4 | 108 |
| PDXDC1 | protein_coding | Yes | NM_015027.4 | 108 |
| COCH | protein_coding | Yes | NM_004086.3 | 108 |
| IRF9 | protein_coding | Yes | NM_006084.5 | 108 |
| HBE1 | protein_coding | Yes | NM_005330.4 | 108 |
| CARD11 | protein_coding | Yes | NM_032415.7 | 108 |

| | | | | | | | | | | |
|---|---|---|---|---|---|---|---|---|---|---|
| ENST00000397061 | 67.1658814 | 1.493971262 | 0.398602 | 3.748031 | 0.000178229 | 0.002380755 | ENSG00000213965 | 19 | 32691820 | 32713792 |
| ENST00000397146 | 38.7692941 | 8.710166493 | 1.521595 | 5.724366 | 1.04E-08 | 4.91E-07 | ENSG00000123243 | 10 | 7559269 | 7666966 |
| ENST00000397238 | 562.450183 | -1.023453547 | 0.178591 | -5.73071 | 1.00E-08 | 4.74E-07 | ENSG00000133612 | 7 | 151086474 | 151144434 |
| ENST00000397397 | 315.597313 | -1.279721333 | 0.203959 | -6.27442 | 3.51E-10 | 2.20E-08 | ENSG00000214063 | 11 | 842851 | 867111 |
| ENST00000397536 | 342.098487 | -1.269947657 | 0.211922 | -5.99253 | 2.07E-09 | 1.13E-07 | ENSG00000188549 | 15 | 40331451 | 40340939 |
| ENST00000397545 | 37.208648 | 1.431277254 | 0.523863 | 2.732161 | 0.006292037 | 0.041218977 | ENSG00000141519 | 17 | 80036641 | 80100613 |
| ENST00000397560 | 1264.79584 | -2.64311031 | 0.148682 | -17.7769 | 1.07E-70 | 3.23E-66 | ENSG00000006459 | 7 | 140084745 | 140176983 |
| ENST00000397820 | 73.0802001 | -1.524101616 | 0.381211 | -3.99805 | 6.39E-05 | 0.001012697 | ENSG00000214212 | 19 | 10848414 | 10869790 |
| ENST00000397909 | 26.6883297 | -4.093408065 | 0.846862 | -4.83362 | 1.34E-06 | 3.82E-05 | ENSG00000067798 | 12 | 77830893 | 78213010 |
| ENST00000397910 | 172.199188 | -3.875185401 | 0.370252 | -10.4663 | 1.23E-25 | 4.62E-23 | ENSG00000181143 | 19 | 8848843 | 8981342 |
| ENST00000397944 | 30.791468 | -8.394166511 | 1.550158 | -5.41504 | 6.13E-08 | 2.52E-06 | ENSG00000118492 | 6 | 146598971 | 146815462 |
| ENST00000397985 | 46.8433321 | -1.402367797 | 0.473053 | -2.9645 | 0.003031725 | 0.023415147 | ENSG00000166401 | 18 | 63970080 | 63989374 |
| ENST00000398449 | 21.8085915 | -2.060692363 | 0.699697 | -2.94512 | 0.003228284 | 0.024605066 | ENSG00000160179 | 21 | 42219139 | 42297244 |
| ENST00000398594 | 168.902894 | 1.106150028 | 0.263043 | 4.205204 | 2.61E-05 | 0.000477322 | ENSG00000254122 | 5 | 141417676 | 141512975 |
| ENST00000398755 | 62.7509946 | 1.161516548 | 0.407113 | 2.853058 | 0.004330078 | 0.030912092 | ENSG00000041880 | 3 | 51942362 | 51948867 |
| ENST00000398882 | 752.082692 | -1.940835409 | 0.176439 | -11.0001 | 3.82E-28 | 2.21E-25 | ENSG00000204839 | 8 | 143566191 | 143572772 |
| ENST00000399002 | 33.5732361 | 1.84574094 | 0.5683 | 3.247831 | 0.001162885 | 0.011135267 | ENSG00000172296 | 20 | 13008971 | 13169103 |
| ENST00000399808 | 488.540592 | 1.452345644 | 0.194832 | 7.454336 | 9.03E-14 | 1.01E-11 | ENSG00000142089 | 11 | 319675 | 320860 |
| ENST00000399815 | 112.368338 | 4.65857359 | 0.467665 | 9.961344 | 2.25E-23 | 7.00E-21 | ENSG00000288681 | 11 | 308407 | 315272 |
| ENST00000399878 | 1272.41071 | -1.625019702 | 0.152419 | -10.6615 | 1.54E-26 | 6.65E-24 | ENSG00000091490 | 4 | 25747432 | 25862988 |
| ENST00000400056 | 11.3576597 | -5.493843175 | 1.678153 | -3.27374 | 0.001061325 | 0.010327945 | ENSG00000214417 | 9 | 97698921 | 97700734 |
| ENST00000400897 | 11.0712431 | 3.306014172 | 1.128439 | 2.929723 | 0.003392647 | 0.025547012 | ENSG00000009724 | 1 | 11026522 | 11047239 |
| ENST00000401408 | 78.3952621 | 1.50090093 | 0.391566 | 3.833074 | 0.000126552 | 0.001789309 | ENSG00000162994 | 2 | 55172546 | 55232293 |
| ENST00000402676 | 368.357632 | -9.534958368 | 1.068181 | -8.92635 | 4.40E-19 | 9.31E-17 | ENSG00000131016 | 6 | 151239966 | 151358559 |
| ENST00000402918 | 43.9025316 | -2.472541178 | 0.516003 | -4.79172 | 1.65E-06 | 4.57E-05 | ENSG00000166897 | 22 | 37367959 | 37427479 |
| ENST00000403687 | 356.24144 | -1.292942791 | 0.197523 | -6.54578 | 5.92E-11 | 4.27E-09 | ENSG00000152689 | 2 | 33476648 | 33564731 |
| ENST00000403729 | 4.04681398 | -5.467228023 | 2.052778 | -2.66333 | 0.007737118 | 0.048289009 | ENSG00000163297 | 4 | 79901145 | 80073229 |
| ENST00000404327 | 91.5818269 | 1.422341684 | 0.344261 | 4.131574 | 3.60E-05 | 0.000628252 | ENSG00000218416 | 2 | 240449417 | 240456714 |
| ENST00000404568 | 33.2043567 | 2.036399504 | 0.573473 | 3.550993 | 0.00038378 | 0.004513351 | ENSG00000144045 | 2 | 74518130 | 74526231 |
| ENST00000404816 | 309.922376 | 1.088016305 | 0.20388 | 5.336543 | 9.47E-08 | 3.71E-06 | ENSG00000049323 | 2 | 32946952 | 33399509 |
| ENST00000405655 | 80.4198296 | -1.653731781 | 0.36808 | -4.49286 | 7.03E-06 | 0.00015697 | ENSG00000185686 | 22 | 22547700 | 22559265 |
| ENST00000406819 | 9.42858265 | 6.671611181 | 1.727764 | 3.861414 | 0.000112732 | 0.001626087 | ENSG00000219249 | 6 | 158725746 | 158726803 |
| ENST00000406875 | 63.6518819 | 1.91488222 | 0.418465 | 4.575965 | 4.74E-06 | 0.000111764 | ENSG00000064393 | 7 | 139561569 | 139777998 |
| ENST00000407010 | 194.168682 | 1.437217558 | 0.257456 | 5.582375 | 2.37E-08 | 1.05E-06 | ENSG00000214960 | 7 | 16087524 | 16421538 |
| ENST00000407071 | 61.4758776 | -1.739798976 | 0.418778 | -4.15447 | 3.26E-05 | 0.000578347 | ENSG00000162849 | 1 | 245154984 | 245709432 |
| ENST00000407693 | 148.358845 | 1.14793788 | 0.275404 | 4.168203 | 3.07E-05 | 0.000549619 | ENSG00000106853 | 9 | 111562566 | 111599647 |
| ENST00000407997 | 1397.22423 | -7.827223154 | 0.665816 | -11.7558 | 6.59E-32 | 5.06E-29 | ENSG00000239713 | 22 | 39077274 | 39087743 |
| ENST00000408903 | 40.2595313 | 1.48991069 | 0.507991 | 2.932945 | 0.003357639 | 0.025355683 | ENSG00000171444 | 5 | 113022105 | 113488453 |
| ENST00000408925 | 8.99643297 | 5.125745383 | 1.721147 | 2.978098 | 0.002900428 | 0.022644108 | ENSG00000221821 | 6 | 42890264 | 42890821 |
| ENST00000408954 | 59.9450323 | -2.994640736 | 0.473177 | -6.3288 | 2.47E-10 | 1.59E-08 | ENSG00000196730 | 9 | 87497866 | 87708634 |

| | | | | |
|---|---|---|---|---|
| NUDT19 | protein_coding | Yes | NM_001105570.2 | 108 |
| ITIH5 | protein_coding | Yes | NM_030569.7 | 108 |
| AGAP3 | protein_coding | Yes | NM_031946.7 | 108 |
| TSPAN4 | protein_coding | Yes | NM_003271.5 | 108 |
| CCDC9B | protein_coding | Yes | NM_207380.3 | 108 |
| CCDC40 | protein_coding | Yes | NM_017950.4 | 108 |
| KDM7A | protein_coding | Yes | NM_030647.2 | 108 |
| C19orf38 | protein_coding | Yes | NM_001136482.3 | 108 |
| NAV3 | protein_coding | Yes | NM_001024383.2 | 108 |
| MUC16 | protein_coding | Yes | - | 108 |
| ADGB | protein_coding | Yes | NM_024694.4 | 108 |
| SERPINB8 | protein_coding | Yes | NM_002640.4 | 108 |
| ABCG1 | protein_coding | Yes | NM_016818.3 | 108 |
| PCDHGB7 | protein_coding | Yes | NM_018927.4 | 108 |
| PARP3 | protein_coding | Yes | NM_001003931.4 | 108 |
| MROH6 | protein_coding | Yes | NM_001100878.2 | 108 |
| SPTLC3 | protein_coding | Yes | NM_018327.4 | 108 |
| IFITM3 | protein_coding | Yes | NM_021034.3 | 108 |
| - | protein_coding | Yes | - | 108 |
| SEL1L3 | protein_coding | Yes | NM_015187.5 | 108 |
| KRT18P13 | essed_pseudo( | Yes | - | 108 |
| MASP2 | protein_coding | Yes | NM_006610.4 | 108 |
| CLHC1 | protein_coding | Yes | NM_152385.4 | 108 |
| AKAP12 | protein_coding | Yes | NM_005100.4 | 108 |
| ELFN2 | protein_coding | Yes | NM_052906.5 | 108 |
| RASGRP3 | protein_coding | Yes | NM_001139488.2 | 108 |
| ANTXR2 | protein_coding | Yes | NM_058172.6 | 108 |
| GPC1-AS1 | lncRNA | Yes | - | 108 |
| DQX1 | protein_coding | Yes | NM_133637.3 | 108 |
| LTBP1 | protein_coding | Yes | NM_206943.4 | 108 |
| PRAME | protein_coding | Yes | NM_206956.3 | 108 |
| AMZ2P2 | essed_pseudo( | Yes | - | 108 |
| HIPK2 | protein_coding | Yes | NM_022740.5 | 108 |
| CRPPA | protein_coding | Yes | NM_001101426.4 | 108 |
| KIF26B | protein_coding | Yes | NM_018012.4 | 108 |
| PTGR1 | protein_coding | Yes | NM_001146108.2 | 108 |
| APOBEC3G | protein_coding | Yes | NM_021822.4 | 108 |
| MCC | protein_coding | Yes | NM_001085377.2 | 108 |
| C6orf226 | protein_coding | Yes | NM_001008739.2 | 108 |
| DAPK1 | protein_coding | Yes | NM_004938.4 | 108 |

| | | | | | | | | | | |
|---|---|---|---|---|---|---|---|---|---|---|
| ENST00000408965 | 104.994977 | -1.227341256 | 0.324328 | -3.78426 | 0.000154167 | 0.002104162 | ENSG00000221869 | 8 | 47736912 | 47738164 |
| ENST00000408968 | 230.687669 | 5.098043233 | 0.370157 | 13.77267 | 3.72E-43 | 8.34E-40 | ENSG00000185885 | 11 | 314039 | 315272 |
| ENST00000409110 | 56.9805314 | 9.267880262 | 1.497305 | 6.189707 | 6.03E-10 | 3.64E-08 | ENSG00000130561 | 2 | 233307815 | 233347055 |
| ENST00000409379 | 1436.51223 | -1.152270635 | 0.138679 | -8.3089 | 9.66E-17 | 1.57E-14 | ENSG00000160949 | 8 | 144428774 | 144444440 |
| ENST00000409548 | 4965.57272 | -2.132741082 | 0.131085 | -16.27 | 1.61E-59 | 1.22E-55 | ENSG00000142102 | 11 | 289125 | 296107 |
| ENST00000409678 | 289.733978 | -1.311923821 | 0.27268 | -4.81123 | 1.50E-06 | 4.20E-05 | ENSG00000185269 | 17 | 81952506 | 81961187 |
| ENST00000413027 | 207.915859 | 1.241544126 | 0.241753 | 5.1356 | 2.81E-07 | 9.62E-06 | ENSG00000227939 | 6 | 31280316 | 31281519 |
| ENST00000414634 | 6.50004923 | -6.152817268 | 1.844335 | -3.33606 | 0.000849738 | 0.008606205 | ENSG00000236508 | 3 | 193307243 | 193314362 |
| ENST00000414799 | 4.57101623 | -5.642314047 | 1.996969 | -2.82544 | 0.004721582 | 0.033094212 | ENSG00000227113 | 7 | 65075022 | 65078780 |
| ENST00000415229 | 112.456507 | 1.14291434 | 0.313344 | 3.647477 | 0.000264828 | 0.00333306 | ENSG00000162174 | 11 | 62337447 | 62393412 |
| ENST00000415816 | 401.682572 | 1.607536357 | 0.254642 | 6.312931 | 2.74E-10 | 1.75E-08 | ENSG00000068079 | 17 | 43006783 | 43014456 |
| ENST00000417362 | 230.846534 | -1.029202537 | 0.230649 | -4.4622 | 8.11E-06 | 0.000177428 | ENSG00000103544 | 16 | 19555702 | 19701163 |
| ENST00000417410 | 93.3243923 | 1.014144389 | 0.335669 | 3.021266 | 0.002517197 | 0.020286168 | ENSG00000176209 | 8 | 42541584 | 42555195 |
| ENST00000417761 | 32.94248 | 1.690420522 | 0.562124 | 3.007201 | 0.002636657 | 0.021039339 | ENSG00000262560 | 15 | 43772616 | 43799133 |
| ENST00000417985 | 8.03022039 | 4.956713692 | 1.75521 | 2.824 | 0.004742843 | 0.033170931 | ENSG00000229145 | X | 46288015 | 46289128 |
| ENST00000418927 | 40.8975559 | -1.960287728 | 0.541902 | -3.61742 | 0.000297554 | 0.003666196 | ENSG00000235269 | 14 | 53685138 | 53850882 |
| ENST00000419025 | 25.1286231 | -2.123549674 | 0.666184 | -3.18763 | 0.001434429 | 0.013139385 | ENSG00000223529 | 3 | 184026368 | 184027756 |
| ENST00000420433 | 4.61296022 | -5.654139163 | 2.012719 | -2.8092 | 0.004966405 | 0.034374985 | ENSG00000171476 | 4 | 56647997 | 56681296 |
| ENST00000420470 | 5.07424647 | -5.793138941 | 1.948089 | -2.97376 | 0.002941797 | 0.022877263 | ENSG00000236699 | 4 | 105552619 | 105680914 |
| ENST00000421553 | 57.9411239 | 1.642961315 | 0.427704 | 3.84135 | 0.00012236 | 0.001738653 | ENSG00000197062 | 6 | 28267142 | 28278204 |
| ENST00000421828 | 78.8776073 | -1.504558768 | 0.371174 | -4.05351 | 5.05E-05 | 0.000832711 | ENSG00000187186 | 9 | 34664162 | 34666045 |
| ENST00000421865 | 63.1585157 | 1.417221356 | 0.433609 | 3.268433 | 0.001081446 | 0.010489026 | ENSG00000196569 | 6 | 128883137 | 129516566 |
| ENST00000422704 | 188.757337 | -1.155887885 | 0.250042 | -4.62277 | 3.79E-06 | 9.29E-05 | ENSG00000187764 | 9 | 89377234 | 89498113 |
| ENST00000424120 | 59.6292953 | 6.289400451 | 0.918285 | 6.849072 | 7.43E-12 | 6.45E-10 | ENSG00000048342 | 4 | 15469881 | 15601552 |
| ENST00000424460 | 36.4378729 | 1.651328915 | 0.534563 | 3.089117 | 0.002007524 | 0.017025768 | ENSG00000229970 | 7 | 8262263 | 8344516 |
| ENST00000424542 | 294.992314 | -1.7680726 | 0.215102 | -8.21968 | 2.04E-16 | 3.11E-14 | ENSG00000065054 | 16 | 2026901 | 2039026 |
| ENST00000425534 | 24.2186371 | -3.291688697 | 0.75762 | -4.34478 | 1.39E-05 | 0.000281423 | ENSG00000163412 | 3 | 71675413 | 71725402 |
| ENST00000425629 | 298.355111 | -1.500251829 | 0.260515 | -5.75879 | 8.47E-09 | 4.08E-07 | ENSG00000105492 | 19 | 51517818 | 51531670 |
| ENST00000425699 | 1133.47239 | -1.028270201 | 0.144012 | -7.14015 | 9.32E-13 | 9.26E-11 | ENSG00000188613 | 10 | 119029713 | 119033730 |
| ENST00000425753 | 1044.91081 | -1.208587045 | 0.160653 | -7.52296 | 5.35E-14 | 6.19E-12 | ENSG00000104522 | 8 | 143612617 | 143617549 |
| ENST00000427199 | 275.860864 | -1.176832847 | 0.215205 | -5.46843 | 4.54E-08 | 1.92E-06 | ENSG00000243414 | 5 | 115578495 | 115602479 |
| ENST00000428149 | 197.522355 | 1.038708778 | 0.244135 | 4.254653 | 2.09E-05 | 0.000396527 | ENSG00000137760 | 11 | 107502726 | 107565735 |
| ENST00000428301 | 5.68050391 | 5.941347995 | 1.894273 | 3.13648 | 0.001709892 | 0.015051413 | ENSG00000290541 | 21 | 13843132 | 13848364 |
| ENST00000429299 | 10.2847621 | -6.814244348 | 1.706145 | -3.99394 | 6.50E-05 | 0.001028062 | ENSG00000227507 | 6 | 31580557 | 31582424 |
| ENST00000429317 | 18.7977323 | -7.684434256 | 1.593152 | -4.82342 | 1.41E-06 | 4.00E-05 | ENSG00000231420 | 2 | 150234891 | 150257007 |
| ENST00000429345 | 6.94036348 | -6.24874061 | 1.832723 | -3.40954 | 0.000650726 | 0.00691595 | ENSG00000216863 | 6 | 6346464 | 6622744 |
| ENST00000429538 | 43.8860664 | -1.338441138 | 0.481398 | -2.78032 | 0.005430476 | 0.036721643 | ENSG00000125618 | 2 | 113215996 | 113278921 |
| ENST00000429722 | 163.303409 | 1.012865711 | 0.291359 | 3.476345 | 0.000508297 | 0.005653061 | ENSG00000189046 | 12 | 109088188 | 109093472 |
| ENST00000431083 | 9.23782989 | -5.169025177 | 1.709266 | -3.02412 | 0.002493576 | 0.020149187 | ENSG00000217825 | 7 | 155196562 | 155198455 |
| ENST00000431232 | 1122.48756 | -1.422092556 | 0.156113 | -9.10936 | 8.29E-20 | 1.89E-17 | ENSG00000129925 | 16 | 370787 | 381978 |

| | | | | |
|---|---|---|---|---|
| CEBPD | protein_coding | Yes | NM_005195.4 | 108 |
| IFITM1 | protein_coding | Yes | NM_003641.5 | 108 |
| SAG | protein_coding | Yes | NM_000541.5 | 108 |
| TONSL | protein_coding | Yes | NM_013432.5 | 108 |
| PGGHG | protein_coding | Yes | NM_025092.5 | 108 |
| NOTUM | protein_coding | Yes | NM_178493.6 | 108 |
| RPL3P2 | essed_pseudog | Yes | - | 108 |
| ATP13A5-AS1 | lncRNA | Yes | - | 108 |
| - | cessed_pseudo | Yes | - | 108 |
| ASRGL1 | protein_coding | Yes | NM_001083926.2 | 108 |
| IFI35 | protein_coding | Yes | NM_001330230.2 | 108 |
| VPS35L | protein_coding | Yes | NM_020314.7 | 108 |
| SMIM19 | protein_coding | Yes | NM_001135674.2 | 108 |
| - | protein_coding | Yes | - | 108 |
| ACTBP1 | essed_pseudog | Yes | - | 108 |
| LINC02331 | lncRNA | Yes | - | 108 |
| EEF1A1P8 | essed_pseudog | Yes | - | 108 |
| HOPX | protein_coding | Yes | NM_032495.6 | 108 |
| ARHGEF38 | protein_coding | Yes | NM_001242729.2 | 108 |
| ZSCAN26 | protein_coding | Yes | NM_001023560.4 | 108 |
| - | protein_coding | Yes | NM_001320038.2 | 108 |
| LAMA2 | protein_coding | Yes | NM_000426.4 | 108 |
| SEMA4D | protein_coding | Yes | NM_001371194.2 | 108 |
| CC2D2A | protein_coding | Yes | NM_001378615.1 | 108 |
| - | lncRNA | Yes | - | 108 |
| SLC9A3R2 | protein_coding | Yes | NM_001130012.3 | 108 |
| EIF4E3 | protein_coding | Yes | NM_001134651.2 | 108 |
| SIGLEC6 | protein_coding | Yes | NM_001245.7 | 108 |
| NANOS1 | protein_coding | Yes | NM_199461.4 | 108 |
| GFUS | protein_coding | Yes | NM_003313.4 | 108 |
| TICAM2 | protein_coding | Yes | NM_021649.7 | 108 |
| ALKBH8 | protein_coding | Yes | NM_138775.3 | 108 |
| CYP4F29P | lncRNA | Yes | - | 108 |
| LTB | protein_coding | Yes | NM_002341.2 | 108 |
| LINC01817 | lncRNA | Yes | - | 108 |
| LY86-AS1 | lncRNA | Yes | - | 108 |
| PAX8 | protein_coding | Yes | NM_003466.4 | 108 |
| ALKBH2 | protein_coding | Yes | NM_001145374.2 | 108 |
| - | lncRNA | Yes | - | 108 |
| PGAP6 | protein_coding | Yes | NM_021259.3 | 108 |

| | | | | | | | | | | |
|---|---|---|---|---|---|---|---|---|---|---|
| ENST00000431378 | 42.6516369 | -1.332200274 | 0.489912 | -2.71926 | 0.006542759 | 0.042471165 | ENSG00000225380 | 9 | 40641840 | 40642117 |
| ENST00000431679 | 28.7900959 | -1.863946305 | 0.603931 | -3.08636 | 0.002026262 | 0.017150992 | ENSG00000244219 | 7 | 99598266 | 99611045 |
| ENST00000432230 | 7.67164981 | 6.374538635 | 1.787093 | 3.566987 | 0.00036111 | 0.004303333 | ENSG00000235609 | 21 | 14818842 | 15014430 |
| ENST00000432264 | 21.5890568 | 2.360212259 | 0.721175 | 3.27273 | 0.001065141 | 0.010358805 | ENSG00000177873 | 3 | 40477121 | 40491053 |
| ENST00000433050 | 4.97786475 | 5.74958366 | 1.949778 | 2.94884 | 0.003189689 | 0.024372674 | ENSG00000198633 | 19 | 52429147 | 52442499 |
| ENST00000433688 | 262.881104 | 1.084689866 | 0.218394 | 4.966674 | 6.81E-07 | 2.12E-05 | ENSG00000188070 | 11 | 63759891 | 63768775 |
| ENST00000434127 | 87.7837278 | -1.601002697 | 0.357981 | -4.47232 | 7.74E-06 | 0.000170666 | ENSG00000187678 | 5 | 142310429 | 142325021 |
| ENST00000434498 | 33.7432847 | 1.985961405 | 0.568302 | 3.494551 | 0.00047486 | 0.005352886 | ENSG00000214429 | 2 | 78412792 | 78413094 |
| ENST00000434651 | 9.31480181 | -4.149771397 | 1.376855 | -3.01395 | 0.0025787 | 0.020673787 | ENSG00000179344 | 6 | 32659466 | 32666657 |
| ENST00000435962 | 53.6237784 | 2.660908565 | 0.503033 | 5.289725 | 1.23E-07 | 4.64E-06 | ENSG00000165929 | 14 | 91779745 | 91867536 |
| ENST00000435967 | 14.0478775 | 2.578936512 | 0.913488 | 2.823176 | 0.004755051 | 0.033240929 | ENSG00000272328 | 7 | 8303740 | 8341343 |
| ENST00000436066 | 824.140914 | -1.28781455 | 0.157549 | -8.17404 | 2.98E-16 | 4.47E-14 | ENSG00000166289 | 19 | 29665460 | 29675477 |
| ENST00000436346 | 61.8857582 | 1.824959213 | 0.429855 | 4.245527 | 2.18E-05 | 0.000409908 | ENSG00000115355 | 2 | 55287841 | 55419856 |
| ENST00000437936 | 41.8145947 | -3.271147245 | 0.616751 | -5.30384 | 1.13E-07 | 4.36E-06 | ENSG00000188293 | 19 | 46229741 | 46231243 |
| ENST00000438747 | 209.382306 | -1.022515605 | 0.256066 | -3.99318 | 6.52E-05 | 0.001030735 | ENSG00000130305 | 7 | 73302515 | 73308826 |
| ENST00000438938 | 7.52748172 | -6.363928544 | 1.793231 | -3.54886 | 0.0003869 | 0.004538944 | ENSG00000223657 | 7 | 136938279 | 136938553 |
| ENST00000439140 | 16.5275278 | 2.328516542 | 0.826931 | 2.815853 | 0.004864787 | 0.033832732 | ENSG00000155754 | 2 | 201487420 | 201619178 |
| ENST00000439838 | 81.6031136 | 1.202013564 | 0.37239 | 3.227838 | 0.001247295 | 0.01178819 | ENSG00000249590 | 22 | 30409261 | 30428987 |
| ENST00000440480 | 88.9763089 | -1.589256459 | 0.355857 | -4.466 | 7.97E-06 | 0.000174964 | ENSG00000129757 | 11 | 2883217 | 2885775 |
| ENST00000440944 | 40.2022832 | -2.394633155 | 0.538182 | -4.44949 | 8.61E-06 | 0.000186747 | ENSG00000160360 | 9 | 136327538 | 136359601 |
| ENST00000441095 | 11.0500935 | -6.917601392 | 1.689107 | -4.09542 | 4.21E-05 | 0.000716394 | ENSG00000233922 | 21 | 45593653 | 45603056 |
| ENST00000441160 | 4.04681398 | -5.467228023 | 2.052778 | -2.66333 | 0.007737118 | 0.048289009 | ENSG00000228437 | 1 | 221966409 | 221984964 |
| ENST00000441377 | 103.565466 | 1.575498121 | 0.333105 | 4.729728 | 2.25E-06 | 5.93E-05 | ENSG00000278419 | 10 | 3134565 | 3145166 |
| ENST00000442396 | 38.7822788 | 1.423692641 | 0.512157 | 2.779797 | 0.005439297 | 0.036767438 | ENSG00000022976 | 14 | 102319745 | 102342367 |
| ENST00000442673 | 81.7714733 | -1.28402241 | 0.358529 | -3.58136 | 0.000341806 | 0.004111869 | ENSG00000236423 | 1 | 3900402 | 3917225 |
| ENST00000443035 | 1355.13053 | -1.005958851 | 0.138222 | -7.27785 | 3.39E-13 | 3.55E-11 | ENSG00000174485 | 15 | 65659122 | 65792293 |
| ENST00000443374 | 26.2416834 | 1.662810538 | 0.627673 | 2.649169 | 0.008069008 | 0.049847826 | ENSG00000226200 | 10 | 50624942 | 50641451 |
| ENST00000444180 | 21.9868047 | -2.076116763 | 0.699538 | -2.96784 | 0.002998989 | 0.023226758 | ENSG00000272508 | 10 | 86993431 | 87024732 |
| ENST00000445105 | 109.846701 | 1.115382686 | 0.31397 | 3.552514 | 0.000381568 | 0.004492934 | ENSG00000114279 | 3 | 192139389 | 192727541 |
| ENST00000445131 | 77.9970188 | 2.066600532 | 0.383822 | 5.384272 | 7.27E-08 | 2.94E-06 | ENSG00000233994 | 1 | 72274551 | 72275159 |
| ENST00000445355 | 1188.79432 | -2.955044586 | 0.193524 | -15.2697 | 1.22E-52 | 4.18E-49 | ENSG00000151651 | 10 | 133262422 | 133276868 |
| ENST00000446597 | 423.79256 | 1.916492518 | 0.210444 | 9.10688 | 8.48E-20 | 1.93E-17 | ENSG00000111731 | 12 | 22448582 | 22544542 |
| ENST00000447110 | 7.0173354 | -4.779447445 | 1.78572 | -2.67648 | 0.007439952 | 0.046828843 | ENSG00000141506 | 17 | 8878915 | 8965707 |
| ENST00000447944 | 1189.99054 | 1.039078195 | 0.138201 | 7.518608 | 5.54E-14 | 6.37E-12 | ENSG00000138035 | 2 | 55634060 | 55693844 |
| ENST00000448875 | 219.112085 | 1.005685108 | 0.234097 | 4.296022 | 1.74E-05 | 0.000338575 | ENSG00000234648 | 14 | 98973313 | 98973471 |
| ENST00000449264 | 14.5307612 | -7.313057029 | 1.634223 | -4.47495 | 7.64E-06 | 0.000168851 | ENSG00000232810 | 6 | 31575564 | 31578336 |
| ENST00000449428 | 354.285562 | -1.018472615 | 0.209366 | -4.86455 | 1.15E-06 | 3.34E-05 | ENSG00000182534 | 17 | 76679565 | 76710965 |
| ENST00000450053 | 614.237926 | -1.128082303 | 0.178504 | -6.31966 | 2.62E-10 | 1.68E-08 | ENSG00000160796 | 3 | 46979665 | 47009701 |
| ENST00000450791 | 5214.15591 | -1.018103201 | 0.128829 | -7.90274 | 2.73E-15 | 3.73E-13 | ENSG00000090615 | 12 | 132768913 | 132828869 |
| ENST00000451137 | 219.004817 | -1.461140934 | 0.242999 | -6.01294 | 1.82E-09 | 1.00E-07 | ENSG00000142871 | 1 | 85580760 | 85583950 |

| | | | | |
|---|---|---|---|---|
| IGKV1OR9-2 | G_V_pseudoger | Yes | - | 108 |
| TMEM225B | protein_coding | Yes | NM_001195541.3 | 108 |
| - | lncRNA | Yes | - | 108 |
| ZNF619 | protein_coding | Yes | NM_001145093.4 | 108 |
| ZNF534 | protein_coding | Yes | NM_001143938.3 | 108 |
| ZFTA | protein_coding | Yes | NM_001144936.2 | 108 |
| SPRY4 | protein_coding | Yes | NM_001127496.3 | 108 |
| CYCSP6 | essed_pseudog | Yes | - | 108 |
| HLA-DQB1 | protein_coding | Yes | NM_002123.5 | 108 |
| TC2N | protein_coding | Yes | NM_001128596.3 | 108 |
| - | lncRNA | Yes | - | 108 |
| PLEKHF1 | protein_coding | Yes | NM_024310.5 | 108 |
| CCDC88A | protein_coding | Yes | NM_001365480.1 | 108 |
| IGFL1 | protein_coding | Yes | NM_198541.2 | 108 |
| NSUN5 | protein_coding | Yes | NM_148956.4 | 108 |
| KRT8P51 | essed_pseudog | Yes | - | 108 |
| C2CD6 | protein_coding | Yes | NM_001168221.2 | 108 |
| - | protein_coding | Yes | - | 108 |
| CDKN1C | protein_coding | Yes | NM_001122630.2 | 108 |
| GPSM1 | protein_coding | Yes | NM_001145638.3 | 108 |
| LINC01694 | lncRNA | Yes | - | 108 |
| LINC02474 | lncRNA | Yes | - | 108 |
| - | lncRNA | Yes | - | 108 |
| ZNF839 | protein_coding | Yes | NM_018335.6 | 108 |
| LINC01134 | lncRNA | Yes | - | 108 |
| DENND4A | protein_coding | Yes | NM_001320835.1 | 108 |
| SGMS1-AS1 | lncRNA | Yes | - | 108 |
| - | lncRNA | Yes | - | 108 |
| FGF12 | protein_coding | Yes | NM_004113.6 | 108 |
| GDI2P2 | essed_pseudog | Yes | - | 108 |
| ADAM8 | protein_coding | Yes | NM_001109.5 | 108 |
| C2CD5 | protein_coding | Yes | NM_001286176.2 | 108 |
| PIK3R5 | protein_coding | Yes | NM_001142633.3 | 108 |
| PNPT1 | protein_coding | Yes | NM_033109.5 | 108 |
| - | essed_pseudog | Yes | - | 108 |
| TNF | protein_coding | Yes | NM_000594.4 | 108 |
| MXRA7 | protein_coding | Yes | NM_198530.4 | 108 |
| NBEAL2 | protein_coding | Yes | NM_015175.3 | 108 |
| GOLGA3 | protein_coding | Yes | NM_001389683.1 | 108 |
| CCN1 | protein_coding | Yes | NM_001554.5 | 108 |

| | | | | | | | | | | |
|---|---|---|---|---|---|---|---|---|---|---|
| ENST00000451775 | 27.3483604 | 1.678743433 | 0.614869 | 2.730245 | 0.00632872 | 0.041410825 | ENSG00000224738 | 17 | 59106597 | 59118453 |
| ENST00000452145 | 30.0979216 | 1.715087622 | 0.586421 | 2.924668 | 0.003448235 | 0.02585552 | ENSG00000204172 | 10 | 47501853 | 47523638 |
| ENST00000453424 | 195.132622 | 2.137651763 | 0.294193 | 7.26615 | 3.70E-13 | 3.85E-11 | ENSG00000165406 | 10 | 45454584 | 45535371 |
| ENST00000453433 | 301.673067 | -1.642844825 | 0.222045 | -7.39872 | 1.38E-13 | 1.50E-11 | ENSG00000228283 | 6 | 146802794 | 146803709 |
| ENST00000454799 | 7.73728661 | 4.90242822 | 1.762098 | 2.782153 | 0.005399951 | 0.036541222 | ENSG00000226711 | 12 | 8180208 | 8216151 |
| ENST00000454872 | 234.858403 | 1.477335925 | 0.254771 | 5.798675 | 6.68E-09 | 3.28E-07 | ENSG00000177694 | 3 | 174859333 | 175810548 |
| ENST00000456660 | 4.68493096 | 5.663623189 | 1.978177 | 2.863052 | 0.004195813 | 0.030141529 | ENSG00000235454 | 7 | 53862232 | 53863339 |
| ENST00000456806 | 107.915126 | 1.58163629 | 0.341379 | 4.633078 | 3.60E-06 | 8.89E-05 | ENSG00000214652 | 7 | 64045433 | 64085339 |
| ENST00000457996 | 29.9554936 | -1.666398369 | 0.587746 | -2.83523 | 0.004579217 | 0.032318189 | ENSG00000233029 | 1 | 121090288 | 121097655 |
| ENST00000458198 | 60.4656635 | 1.407577235 | 0.415014 | 3.391634 | 0.000694772 | 0.007305979 | ENSG00000237190 | 5 | 134402064 | 134411881 |
| ENST00000458200 | 51.460709 | 1.372778534 | 0.448887 | 3.05818 | 0.002226857 | 0.018452301 | ENSG00000231752 | 1 | 121519344 | 121568791 |
| ENST00000458549 | 1748.8245 | -1.244428634 | 0.132736 | -9.37525 | 6.90E-21 | 1.76E-18 | ENSG00000180694 | 8 | 90621999 | 90646083 |
| ENST00000461517 | 11.5202157 | -2.785755204 | 1.045886 | -2.66354 | 0.007732417 | 0.048273622 | ENSG00000241175 | 15 | 50244627 | 50244931 |
| ENST00000462792 | 43.7920481 | 4.57381143 | 0.717389 | 6.375635 | 1.82E-10 | 1.20E-08 | ENSG00000239572 | 3 | 87641411 | 87793629 |
| ENST00000463745 | 102.983441 | 1.169569826 | 0.32603 | 3.587303 | 0.000334116 | 0.004034782 | ENSG00000113966 | 3 | 97764757 | 97801229 |
| ENST00000463781 | 15.0826249 | -3.206809055 | 0.965464 | -3.32152 | 0.000895284 | 0.008986084 | ENSG00000145113 | 3 | 195746770 | 195811929 |
| ENST00000465530 | 12.0512878 | 2.802752697 | 1.007406 | 2.782149 | 0.005400028 | 0.036541222 | ENSG00000185267 | 10 | 14819244 | 14838037 |
| ENST00000466133 | 151.352838 | 1.023257775 | 0.271434 | 3.769823 | 0.000163363 | 0.002212681 | ENSG00000184100 | 3 | 160100849 | 160102793 |
| ENST00000466271 | 65.6464639 | 1.127175774 | 0.39581 | 2.847773 | 0.004402633 | 0.031332376 | ENSG00000165626 | 10 | 13441087 | 13529014 |
| ENST00000467482 | 4.26697111 | -5.544915273 | 2.022592 | -2.74149 | 0.006116115 | 0.040284792 | ENSG00000101850 | X | 9725345 | 9765847 |
| ENST00000471229 | 362.497294 | -6.691354752 | 0.427884 | -15.6382 | 4.00E-55 | 1.78E-51 | ENSG00000102401 | X | 101623150 | 101627843 |
| ENST00000472837 | 69.7490571 | -1.452452977 | 0.390321 | -3.72118 | 0.000198297 | 0.002603972 | ENSG00000174428 | 7 | 75092589 | 75149817 |
| ENST00000474759 | 24.6670258 | 2.145560213 | 0.681684 | 3.147441 | 0.001647062 | 0.014628714 | ENSG00000157445 | 3 | 54122551 | 55074557 |
| ENST00000476941 | 134.857706 | 2.138808854 | 0.316859 | 6.750034 | 1.48E-11 | 1.20E-09 | ENSG00000144935 | 3 | 142724033 | 142807888 |
| ENST00000477258 | 13.1900921 | -2.510331816 | 0.934961 | -2.68496 | 0.007253859 | 0.045979241 | ENSG00000168386 | 3 | 99828810 | 100114501 |
| ENST00000479066 | 8.0042962 | -3.918752747 | 1.42437 | -2.75122 | 0.005937416 | 0.039368559 | ENSG00000243537 | 8 | 133325996 | 133326404 |
| ENST00000484902 | 135.473157 | 1.092354254 | 0.289056 | 3.779036 | 0.000157437 | 0.002143178 | ENSG00000227063 | 20 | 21755269 | 21755350 |
| ENST00000486442 | 103.145678 | -1.667165905 | 0.338567 | -4.92418 | 8.47E-07 | 2.57E-05 | ENSG00000119771 | 2 | 23385178 | 23708606 |
| ENST00000488300 | 137.068915 | -1.001243779 | 0.296463 | -3.3773 | 0.000732019 | 0.007624455 | ENSG00000180537 | 6 | 13924951 | 13980310 |
| ENST00000488788 | 5692.10676 | -1.02517899 | 0.139947 | -7.3255 | 2.38E-13 | 2.55E-11 | ENSG00000270299 | 20 | 646625 | 675800 |
| ENST00000490466 | 225.348829 | -1.238160325 | 0.235621 | -5.25489 | 1.48E-07 | 5.48E-06 | ENSG00000142694 | 1 | 36322029 | 36323645 |
| ENST00000491614 | 1139.82713 | 1.344909268 | 0.140001 | 9.606395 | 7.51E-22 | 2.09E-19 | ENSG00000165609 | 10 | 12165329 | 12195891 |
| ENST00000494426 | 712.86684 | -1.061658986 | 0.162428 | -6.53617 | 6.31E-11 | 4.53E-09 | ENSG00000169583 | 9 | 136994607 | 136996568 |
| ENST00000495936 | 182.792544 | -1.266119008 | 0.258617 | -4.89574 | 9.79E-07 | 2.92E-05 | ENSG00000160991 | 7 | 102433574 | 102456825 |
| ENST00000497571 | 383.705169 | 1.518003952 | 0.193987 | 7.825283 | 5.07E-15 | 6.71E-13 | ENSG00000067082 | 10 | 3775995 | 3785209 |
| ENST00000498907 | 56.7104016 | 2.212406577 | 0.45197 | 4.895034 | 9.83E-07 | 2.92E-05 | ENSG00000245848 | 19 | 33299933 | 33302534 |
| ENST00000499418 | 71.9247529 | -1.077620763 | 0.378534 | -2.84683 | 0.004415687 | 0.031402496 | ENSG00000245149 | 8 | 124462484 | 124474564 |
| ENST00000499966 | 16.511421 | 2.30766052 | 0.822215 | 2.80664 | 0.005006116 | 0.03456594 | ENSG00000246898 | 16 | 66408515 | 66412135 |
| ENST00000501665 | 253.868165 | -1.011960093 | 0.225244 | -4.49273 | 7.03E-06 | 0.000157038 | ENSG00000247556 | 15 | 41284031 | 41309737 |
| ENST00000504924 | 152.515212 | 1.109587509 | 0.308373 | 3.598202 | 0.000320424 | 0.003894647 | ENSG00000185305 | 5 | 53883941 | 54310573 |

| | | | | |
|---|---|---|---|---|
| - | lncRNA | Yes | - | 108 |
| AGAP9 | protein_coding | Yes | NM_001190810.1 | 108 |
| MARCHF8 | protein_coding | Yes | NM_001282866.2 | 108 |
| KATNBL1P6 | d_processed_ps | Yes | - | 108 |
| FAM66C | lncRNA | Yes | - | 108 |
| NAALADL2 | protein_coding | Yes | NM_207015.3 | 108 |
| HAUS6P3 | essed_pseudog | Yes | - | 108 |
| ZNF727 | protein_coding | Yes | NM_001159522.3 | 108 |
| - | lncRNA | Yes | - | 108 |
| CDKN2AIPNL | protein_coding | Yes | NM_080656.3 | 108 |
| EMBP1 | _unprocessed_p | Yes | - | 108 |
| TMEM64 | protein_coding | Yes | NM_001008495.4 | 108 |
| RN7SL494P | misc_RNA | Yes | - | 108 |
| - | lncRNA | Yes | - | 108 |
| ARL6 | protein_coding | Yes | NM_001278293.3 | 108 |
| MUC4 | protein_coding | Yes | NM_018406.7 | 108 |
| CDNF | protein_coding | Yes | NM_001029954.3 | 108 |
| BRD7P2 | essed_pseudog | Yes | - | 108 |
| BEND7 | protein_coding | Yes | NM_001369863.1 | 108 |
| GPR143 | protein_coding | Yes | NM_000273.3 | 108 |
| ARMCX3 | protein_coding | Yes | NM_177947.3 | 108 |
| GTF2IRD2B | protein_coding | Yes | NM_001003795.3 | 108 |
| CACNA2D3 | protein_coding | Yes | NM_018398.3 | 108 |
| TRPC1 | protein_coding | Yes | NM_001251845.2 | 108 |
| FILIP1L | protein_coding | Yes | NM_001387850.1 | 108 |
| RPL32P20 | essed_pseudog | Yes | - | 108 |
| RPL41P1 | essed_pseudog | Yes | - | 108 |
| KLHL29 | protein_coding | Yes | NM_052920.2 | 108 |
| RNF182 | protein_coding | Yes | NM_152737.4 | 108 |
| - | protein_coding | Yes | - | 108 |
| EVA1B | protein_coding | Yes | NM_001304762.2 | 108 |
| NUDT5 | protein_coding | Yes | NM_014142.4 | 108 |
| CLIC3 | protein_coding | Yes | NM_004669.3 | 108 |
| ORAI2 | protein_coding | Yes | NM_001126340.3 | 108 |
| KLF6 | protein_coding | Yes | NM_001300.6 | 108 |
| CEBPA | protein_coding | Yes | NM_004364.5 | 108 |
| RNF139-DT | lncRNA | Yes | - | 108 |
| LINC00920 | lncRNA | Yes | - | 108 |
| OIP5-AS1 | lncRNA | Yes | - | 108 |
| ARL15 | protein_coding | Yes | NM_019087.3 | 108 |

| | | | | | | | | | | |
|---|---|---|---|---|---|---|---|---|---|---|
| ENST00000505667 | 1047.0289 | -1.033092086 | 0.141232 | -7.31487 | 2.58E-13 | 2.75E-11 | ENSG00000129116 | 4 | 168497051 | 168928441 |
| ENST00000507866 | 6.05973497 | -6.049979257 | 1.870544 | -3.23434 | 0.001219233 | 0.011578734 | ENSG00000184985 | 4 | 7192537 | 7742827 |
| ENST00000509176 | 35.5010206 | 1.601089565 | 0.541973 | 2.954188 | 0.003134931 | 0.024045654 | ENSG00000184305 | 4 | 90127393 | 91605295 |
| ENST00000509479 | 14.3991327 | -3.112807106 | 0.954444 | -3.26138 | 0.001108702 | 0.01070529 | ENSG00000196782 | 4 | 139716752 | 140154184 |
| ENST00000509706 | 52.5984424 | 1.524218608 | 0.445375 | 3.422328 | 0.000620874 | 0.006654843 | ENSG00000206228 | 8 | 82291623 | 82292379 |
| ENST00000510708 | 147.951241 | 1.368172923 | 0.287059 | 4.766171 | 1.88E-06 | 5.09E-05 | ENSG00000169570 | 5 | 118836073 | 118988547 |
| ENST00000511928 | 169.358887 | -2.279230775 | 0.283444 | -8.04121 | 8.90E-16 | 1.28E-13 | ENSG00000250986 | 4 | 3758747 | 3763390 |
| ENST00000512369 | 1046.88922 | -1.168450792 | 0.14922 | -7.83042 | 4.86E-15 | 6.45E-13 | ENSG00000070190 | 4 | 99816826 | 99870190 |
| ENST00000514985 | 67.0878428 | 1.901394095 | 0.443005 | 4.292034 | 1.77E-05 | 0.000343741 | ENSG00000250722 | 5 | 42799879 | 42811892 |
| ENST00000517992 | 2977.99154 | -1.964357544 | 0.126288 | -15.5546 | 1.48E-54 | 5.46E-51 | ENSG00000172164 | 8 | 120535755 | 120812046 |
| ENST00000518322 | 37.7270488 | 2.252978611 | 0.549005 | 4.103751 | 4.07E-05 | 0.000695364 | ENSG00000188343 | 8 | 93700549 | 93731527 |
| ENST00000519676 | 118.227019 | -1.650332573 | 0.308727 | -5.34561 | 9.01E-08 | 3.55E-06 | ENSG00000174032 | 13 | 45393315 | 45418373 |
| ENST00000519762 | 4.21650485 | 5.510674427 | 2.028128 | 2.717124 | 0.006585201 | 0.042677947 | ENSG00000254338 | 8 | 143417678 | 143419150 |
| ENST00000522557 | 45.3627805 | 1.458990346 | 0.484545 | 3.011051 | 0.002603454 | 0.020831352 | ENSG00000124097 | 20 | 57487958 | 57495366 |
| ENST00000522652 | 2006.66335 | -1.613614855 | 0.126029 | -12.8035 | 1.57E-37 | 1.94E-34 | ENSG00000008513 | 8 | 133454847 | 133571887 |
| ENST00000523390 | 126.949212 | 1.064229895 | 0.301826 | 3.525968 | 0.000421939 | 0.004877095 | ENSG00000254221 | 5 | 141350098 | 141512975 |
| ENST00000523860 | 61.591486 | 1.260297296 | 0.415506 | 3.033166 | 0.002420026 | 0.019697384 | ENSG00000253706 | 8 | 74798783 | 74866925 |
| ENST00000524555 | 26.2305284 | -1.770843584 | 0.629373 | -2.81366 | 0.004898025 | 0.033991569 | ENSG00000255071 | 11 | 18231422 | 18248635 |
| ENST00000525123 | 43.1705289 | 1.493413848 | 0.488975 | 3.054172 | 0.002256827 | 0.018645193 | ENSG00000165923 | 11 | 47659590 | 47715369 |
| ENST00000525643 | 62.5651505 | -3.984156951 | 0.528579 | -7.53749 | 4.79E-14 | 5.59E-12 | ENSG00000008517 | 16 | 3065639 | 3069530 |
| ENST00000527879 | 79.1402001 | 1.407688167 | 0.375029 | 3.753549 | 0.000174349 | 0.002335325 | ENSG00000255150 | 12 | 104303738 | 104305205 |
| ENST00000528667 | 2237.08724 | -1.085763605 | 0.124041 | -8.75329 | 2.07E-18 | 4.06E-16 | ENSG00000116337 | 1 | 109619836 | 109632051 |
| ENST00000529415 | 158.654059 | -1.415695079 | 0.269746 | -5.24824 | 1.54E-07 | 5.66E-06 | ENSG00000254402 | 8 | 144522387 | 144527033 |
| ENST00000530639 | 100.467741 | -1.725109492 | 0.332966 | -5.18103 | 2.21E-07 | 7.81E-06 | ENSG00000135362 | 11 | 36296287 | 36465204 |
| ENST00000531730 | 19.5088525 | -2.315793109 | 0.774653 | -2.98946 | 0.002794726 | 0.022001863 | ENSG00000255050 | 8 | 143573489 | 143577397 |
| ENST00000533544 | 438.74025 | -1.010182165 | 0.186521 | -5.41591 | 6.10E-08 | 2.51E-06 | ENSG00000175376 | 11 | 65996544 | 66002157 |
| ENST00000533924 | 277.296507 | -2.091140059 | 0.260706 | -8.02107 | 1.05E-15 | 1.50E-13 | ENSG00000255026 | 11 | 287304 | 288298 |
| ENST00000534000 | 393.519495 | -1.270103663 | 0.197686 | -6.42486 | 1.32E-10 | 8.95E-09 | ENSG00000111907 | 6 | 125153772 | 125264407 |
| ENST00000534313 | 179.337099 | -1.055155645 | 0.25642 | -4.11496 | 3.87E-05 | 0.000667871 | ENSG00000213445 | 11 | 65638100 | 65650912 |
| ENST00000535456 | 301.035687 | -1.780611988 | 0.212732 | -8.3702 | 5.75E-17 | 9.56E-15 | ENSG00000149328 | 11 | 134331987 | 134376324 |
| ENST00000535784 | 964.641322 | 1.740369643 | 0.153619 | 11.3291 | 9.42E-30 | 6.68E-27 | ENSG00000165983 | 10 | 16437009 | 16513745 |
| ENST00000538077 | 74.4208192 | 1.438308228 | 0.37792 | 3.805858 | 0.000141314 | 0.001956689 | ENSG00000255874 | 13 | 110863986 | 110870330 |
| ENST00000541103 | 68.3230278 | -1.235235709 | 0.38894 | -3.1759 | 0.001493705 | 0.013559897 | ENSG00000176700 | 15 | 84631962 | 84642066 |
| ENST00000544301 | 4.71429133 | 5.671608216 | 1.974105 | 2.873002 | 0.004065912 | 0.029389963 | ENSG00000026025 | 10 | 17228240 | 17237593 |
| ENST00000544898 | 90.6090524 | 1.510404958 | 0.348399 | 4.335277 | 1.46E-05 | 0.000292568 | ENSG00000166548 | 16 | 66508002 | 66550122 |
| ENST00000545001 | 75.1604511 | -1.268404249 | 0.374141 | -3.39017 | 0.000698482 | 0.007338864 | ENSG00000256293 | 12 | 64338177 | 64338797 |
| ENST00000549284 | 6.23701111 | 6.074526403 | 1.85872 | 3.268123 | 0.001082632 | 0.010496488 | ENSG00000257356 | 14 | 18418774 | 18419273 |
| ENST00000549336 | 76.7018675 | -1.023177938 | 0.366489 | -2.79184 | 0.005240975 | 0.03572226 | ENSG00000111424 | 12 | 47841536 | 47904994 |
| ENST00000551568 | 9.54187501 | -5.216405557 | 1.697358 | -3.07325 | 0.002117416 | 0.017739741 | ENSG00000135678 | 12 | 68851174 | 68933171 |
| ENST00000551956 | 21.1578881 | -3.282324679 | 0.811887 | -4.04283 | 5.28E-05 | 0.000864974 | ENSG00000170477 | 12 | 52806548 | 52814116 |

| | | | | |
|---|---|---|---|---|
| PALLD | protein_coding | Yes | NM_001166108.2 | 108 |
| SORCS2 | protein_coding | Yes | NM_020777.3 | 108 |
| CCSER1 | protein_coding | Yes | NM_001145065.2 | 108 |
| MAML3 | protein_coding | Yes | NM_018717.5 | 108 |
| HNRNPA1P4 | essed_pseudog | Yes | - | 108 |
| DTWD2 | protein_coding | Yes | NM_173666.4 | 108 |
| LINC02600 | lncRNA | Yes | - | 108 |
| DAPP1 | protein_coding | Yes | NM_014395.3 | 108 |
| SELENOP | protein_coding | Yes | NM_005410.4 | 108 |
| SNTB1 | protein_coding | Yes | NM_021021.4 | 108 |
| CIBAR1 | protein_coding | Yes | NM_145269.5 | 108 |
| SLC25A30 | protein_coding | Yes | NM_001010875.4 | 108 |
| MAFA-AS1 | lncRNA | Yes | - | 108 |
| HMGB1P1 | protein_coding | Yes | - | 108 |
| ST3GAL1 | protein_coding | Yes | NM_173344.3 | 108 |
| PCDHGB1 | protein_coding | Yes | NM_018922.3 | 108 |
| - | lncRNA | Yes | - | 108 |
| SAA2-SAA4 | protein_coding | Yes | - | 108 |
| AGBL2 | protein_coding | Yes | NM_024783.4 | 108 |
| IL32 | protein_coding | Yes | NM_001376923.1 | 108 |
| EID3 | protein_coding | Yes | NM_001008394.3 | 108 |
| AMPD2 | protein_coding | Yes | NM_001368809.2 | 108 |
| LRRC24 | protein_coding | Yes | NM_001024678.4 | 108 |
| PRR5L | protein_coding | Yes | NM_001160167.2 | 108 |
| - | lncRNA | Yes | - | 108 |
| EIF1AD | protein_coding | Yes | NM_001242481.2 | 108 |
| - | lncRNA | Yes | - | 108 |
| TPD52L1 | protein_coding | Yes | NM_003287.4 | 108 |
| SIPA1 | protein_coding | Yes | NM_006747.4 | 108 |
| GLB1L2 | protein_coding | Yes | NM_001370461.1 | 108 |
| PTER | protein_coding | Yes | NM_001261836.2 | 108 |
| PRECSIT | lncRNA | Yes | - | 108 |
| SCAND2P | _unprocessed_p | Yes | - | 108 |
| VIM | protein_coding | Yes | NM_003380.5 | 108 |
| TK2 | protein_coding | Yes | NM_004614.5 | 108 |
| ATP6V1E1P3 | essed_pseudog | Yes | - | 108 |
| BNIP3P6 | essed_pseudog | Yes | - | 108 |
| VDR | protein_coding | Yes | NM_000376.3 | 108 |
| CPM | protein_coding | Yes | NM_198320.5 | 108 |
| KRT4 | protein_coding | Yes | NM_002272.4 | 108 |

| | | | | | | | | | | |
|---|---|---|---|---|---|---|---|---|---|---|
| ENST00000552810 | 387.464762 | 1.251310666 | 0.192695 | 6.493753 | 8.37E-11 | 5.88E-09 | ENSG00000198707 | 12 | 88049015 | 88142088 |
| ENST00000554452 | 10.5623914 | -4.341043254 | 1.341134 | -3.23685 | 0.001208586 | 0.011501025 | ENSG00000258590 | 15 | 20657637 | 20688408 |
| ENST00000554659 | 379.548806 | 1.060189395 | 0.192956 | 5.494469 | 3.92E-08 | 1.67E-06 | ENSG00000100568 | 14 | 67647084 | 67674632 |
| ENST00000555815 | 25.1432584 | -3.363219367 | 0.75558 | -4.45118 | 8.54E-06 | 0.000185468 | ENSG00000206190 | 15 | 25678711 | 25863327 |
| ENST00000558375 | 4.99035848 | -5.771826396 | 1.951724 | -2.9573 | 0.003103493 | 0.02385242 | ENSG00000259407 | 15 | 85744108 | 85750281 |
| ENST00000558391 | 5.07424647 | -5.793138941 | 1.948089 | -2.97376 | 0.002941797 | 0.022877263 | ENSG00000259315 | 15 | 82726306 | 82727425 |
| ENST00000559052 | 9.75726255 | -4.248939893 | 1.454836 | -2.92056 | 0.003493999 | 0.026130821 | ENSG00000259186 | 15 | 73483195 | 73483943 |
| ENST00000560346 | 45.030626 | -1.96794951 | 0.490952 | -4.00844 | 6.11E-05 | 0.000976266 | ENSG00000137843 | 15 | 40239062 | 40277487 |
| ENST00000560869 | 38.7745577 | -2.144102423 | 0.549237 | -3.90378 | 9.47E-05 | 0.001407298 | ENSG00000185215 | 14 | 103123460 | 103137439 |
| ENST00000561421 | 90.4169622 | -1.180612809 | 0.349272 | -3.38021 | 0.000724294 | 0.007559616 | ENSG00000103876 | 15 | 80152998 | 80186349 |
| ENST00000561705 | 8.69658312 | 6.55478384 | 1.749812 | 3.745994 | 0.000179681 | 0.00239761 | ENSG00000260265 | 4 | 75081701 | 75084717 |
| ENST00000562038 | 101.762177 | 1.01652913 | 0.322515 | 3.151886 | 0.001622193 | 0.014465642 | ENSG00000260804 | 2 | 216217044 | 216220192 |
| ENST00000562057 | 4016.27234 | -1.183580984 | 0.118317 | -10.0035 | 1.47E-23 | 4.63E-21 | ENSG00000166145 | 15 | 40844047 | 40858207 |
| ENST00000562280 | 12.2048302 | -5.579505418 | 1.651601 | -3.37824 | 0.000729511 | 0.00760095 | ENSG00000260034 | 16 | 25140576 | 25149032 |
| ENST00000562760 | 60.0475698 | 1.391563405 | 0.415877 | 3.346093 | 0.00081959 | 0.008360026 | ENSG00000261437 | 8 | 94637284 | 94639467 |
| ENST00000562790 | 130.301945 | 10.46024818 | 1.468534 | 7.122916 | 1.06E-12 | 1.04E-10 | ENSG00000260999 | 16 | 68927546 | 68948261 |
| ENST00000563018 | 15.5412369 | 2.747947068 | 0.883888 | 3.108931 | 0.001877653 | 0.016154713 | ENSG00000260193 | 9 | 136108104 | 136109424 |
| ENST00000563172 | 21.9674389 | -6.455963143 | 1.553784 | -4.15499 | 3.25E-05 | 0.000577448 | ENSG00000261780 | 18 | 73324940 | 73349879 |
| ENST00000563601 | 14.5827428 | 2.654647961 | 0.901133 | 2.945899 | 0.003220171 | 0.024560927 | ENSG00000260589 | 10 | 17641283 | 17643878 |
| ENST00000564224 | 97.0898735 | -1.111740853 | 0.332071 | -3.3479 | 0.000814257 | 0.00831539 | ENSG00000261215 | 9 | 34661902 | 34666029 |
| ENST00000565283 | 153.168582 | 1.166030331 | 0.27199 | 4.287035 | 1.81E-05 | 0.000350081 | ENSG00000260025 | 2 | 36354743 | 36355471 |
| ENST00000566339 | 607.400144 | -1.021062224 | 0.1664 | -6.1362 | 8.45E-10 | 4.97E-08 | ENSG00000162032 | 16 | 1776711 | 1782571 |
| ENST00000566383 | 6.76415795 | 6.190908804 | 1.831928 | 3.379451 | 0.000726308 | 0.007574889 | ENSG00000261804 | 16 | 53373478 | 53385142 |
| ENST00000566699 | 99.7509968 | -3.063461823 | 0.376067 | -8.14606 | 3.76E-16 | 5.59E-14 | ENSG00000260231 | 7 | 140177183 | 140179640 |
| ENST00000567180 | 50.6525047 | -1.257767544 | 0.464015 | -2.71062 | 0.006715782 | 0.043354098 | ENSG00000260428 | 8 | 144266452 | 144268481 |
| ENST00000568179 | 15.5173197 | 5.921561319 | 1.606665 | 3.685623 | 0.000228144 | 0.002935437 | ENSG00000266801 | 16 | 68933819 | 68937725 |
| ENST00000569591 | 34.0027954 | 2.084005614 | 0.579501 | 3.596206 | 0.000322892 | 0.003920697 | ENSG00000260314 | 10 | 17809347 | 17911164 |
| ENST00000569831 | 8.28736932 | -3.971389959 | 1.411976 | -2.81265 | 0.004913547 | 0.034076231 | ENSG00000260425 | 16 | 1358899 | 1361405 |
| ENST00000571340 | 6.00279806 | 6.018894125 | 1.87445 | 3.211019 | 0.001322652 | 0.012331658 | ENSG00000262714 | 16 | 53386943 | 53389085 |
| ENST00000571422 | 4.48007827 | 5.597580283 | 1.998575 | 2.800785 | 0.00509784 | 0.035050348 | ENSG00000262539 | 17 | 46259550 | 46260606 |
| ENST00000574428 | 146.039871 | 1.01606493 | 0.279426 | 3.636263 | 0.000276622 | 0.00345135 | ENSG00000123472 | 1 | 46632736 | 46668364 |
| ENST00000576742 | 378.641862 | -1.381252845 | 0.194458 | -7.10308 | 1.22E-12 | 1.19E-10 | ENSG00000167723 | 17 | 3510501 | 3557812 |
| ENST00000577809 | 116.559141 | 1.688892836 | 0.312774 | 5.399724 | 6.67E-08 | 2.72E-06 | ENSG00000108666 | 17 | 32328440 | 32342173 |
| ENST00000577863 | 5.99681898 | -6.036652153 | 1.874613 | -3.22021 | 0.001280958 | 0.012028864 | ENSG00000179136 | 17 | 12549940 | 12642854 |
| ENST00000577970 | 89.2831619 | -1.027812355 | 0.344331 | -2.98496 | 0.002836171 | 0.022253968 | ENSG00000263567 | 17 | 31762439 | 31769048 |
| ENST00000578774 | 28.7081715 | 1.760603238 | 0.603147 | 2.919028 | 0.00351125 | 0.02623776 | ENSG00000266208 | 17 | 40360654 | 40364693 |
| ENST00000579212 | 167.334703 | 2.33348335 | 0.283737 | 8.224116 | 1.97E-16 | 3.00E-14 | ENSG00000284368 | 17 | 64501213 | 64501313 |
| ENST00000580960 | 8.32315417 | 5.009100819 | 1.750756 | 2.861107 | 0.004221647 | 0.030265249 | ENSG00000265542 | 17 | 57771945 | 57834749 |
| ENST00000581130 | 112.393594 | 2.354082567 | 0.336177 | 7.002502 | 2.51E-12 | 2.37E-10 | ENSG00000284564 | 17 | 64500773 | 64500839 |
| ENST00000581792 | 681.150033 | -3.227349806 | 0.20592 | -15.6728 | 2.32E-55 | 1.06E-51 | ENSG00000264462 | 21 | 8986998 | 8987178 |

| | | | | |
|---|---|---|---|---|
| CEP290 | protein_coding | Yes | NM_025114.4 | 108 |
| NBEAP1 | _unprocessed_p | Yes | - | 108 |
| VTI1B | protein_coding | Yes | NM_006370.3 | 108 |
| ATP10A | protein_coding | Yes | NM_024490.4 | 108 |
| - | lncRNA | Yes | - | 108 |
| ACTG1P17 | d_processed_ps | Yes | - | 108 |
| MRPS15P1 | essed_pseudog | Yes | - | 108 |
| PAK6 | protein_coding | Yes | NM_001395430.1 | 108 |
| TNFAIP2 | protein_coding | Yes | NM_006291.4 | 108 |
| FAH | protein_coding | Yes | NM_000137.4 | 108 |
| LINC02562 | lncRNA | Yes | - | 108 |
| LINC01963 | lncRNA | Yes | - | 108 |
| SPINT1 | protein_coding | Yes | NM_003710.4 | 108 |
| LCMT1-AS2 | lncRNA | Yes | - | 108 |
| LINC02894 | lncRNA | Yes | - | 108 |
| - | lncRNA | Yes | - | 108 |
| LINC02846 | lncRNA | Yes | - | 108 |
| LINC02582 | lncRNA | Yes | - | 108 |
| STAM-DT | lncRNA | Yes | - | 108 |
| - | lncRNA | Yes | - | 108 |
| CRIM1-DT | lncRNA | Yes | - | 108 |
| SPSB3 | protein_coding | Yes | NM_080861.4 | 108 |
| - | lncRNA | Yes | - | 108 |
| KDM7A-DT | lncRNA | Yes | - | 108 |
| SCX | protein_coding | Yes | NM_001080514.3 | 108 |
| - | lncRNA | Yes | - | 108 |
| MRC1 | protein_coding | Yes | NM_002438.4 | 108 |
| - | lncRNA | Yes | - | 108 |
| - | lncRNA | Yes | - | 108 |
| - | essed_pseudog | Yes | - | 108 |
| ATPAF1 | protein_coding | Yes | NM_001394565.1 | 108 |
| TRPV3 | protein_coding | Yes | NM_145068.4 | 108 |
| C17orf75 | protein_coding | Yes | NM_022344.4 | 108 |
| LINC00670 | lncRNA | Yes | - | 108 |
| - | lncRNA | Yes | - | 108 |
| GJD3-AS1 | lncRNA | Yes | - | 108 |
| MIR5047 | miRNA | Yes | - | 108 |
| - | lncRNA | Yes | - | 108 |
| MIR3064 | miRNA | Yes | - | 108 |
| MIR3648-2 | miRNA | Yes | - | 108 |

| | | | | | | | | | | |
|---|---|---|---|---|---|---|---|---|---|---|
| ENST00000584605 | 31.2025477 | 1.777808948 | 0.5801 | 3.06466 | 0.002179177 | 0.01815352 | ENSG00000266728 | 17 | 27623363 | 27640777 |
| ENST00000585281 | 106.58954 | 1.556232729 | 0.350519 | 4.43979 | 9.00E-06 | 0.000194052 | ENSG00000265190 | 10 | 47467992 | 47484115 |
| ENST00000585682 | 246.389051 | 2.388686499 | 0.242826 | 9.837032 | 7.80E-23 | 2.34E-20 | ENSG00000164125 | 4 | 158124473 | 158173025 |
| ENST00000586569 | 18.1926326 | 2.05868413 | 0.765535 | 2.68921 | 0.007162132 | 0.045535323 | ENSG00000141655 | 18 | 62325309 | 62391288 |
| ENST00000586726 | 16.749381 | 2.884046954 | 0.865872 | 3.330802 | 0.000865962 | 0.008745901 | ENSG00000225339 | 6 | 34248534 | 34284453 |
| ENST00000586748 | 1334.54614 | -1.015051542 | 0.139683 | -7.26681 | 3.68E-13 | 3.84E-11 | ENSG00000130733 | 19 | 10922192 | 10928639 |
| ENST00000587128 | 7.90635228 | -4.937274429 | 1.772711 | -2.78516 | 0.00535021 | 0.036267438 | ENSG00000267058 | 19 | 43891803 | 43901805 |
| ENST00000589332 | 10.7432814 | 2.935579641 | 1.096451 | 2.677347 | 0.007420782 | 0.046754919 | ENSG00000186496 | 18 | 35366693 | 35377337 |
| ENST00000589666 | 132.211622 | 1.149216837 | 0.298434 | 3.850828 | 0.000117719 | 0.001686253 | ENSG00000132478 | 17 | 75784805 | 75825799 |
| ENST00000590508 | 312.730868 | -1.079505519 | 0.225438 | -4.78849 | 1.68E-06 | 4.63E-05 | ENSG00000132003 | 19 | 13795442 | 13832254 |
| ENST00000591368 | 9.98241305 | -3.200256077 | 1.15631 | -2.76765 | 0.00564627 | 0.037863356 | ENSG00000204706 | 9 | 70087418 | 70175883 |
| ENST00000593945 | 43.0906155 | -2.862209212 | 0.540888 | -5.29168 | 1.21E-07 | 4.61E-06 | ENSG00000074219 | 19 | 49340594 | 49362416 |
| ENST00000595010 | 62.0786053 | 2.093735299 | 0.426401 | 4.910254 | 9.10E-07 | 2.74E-05 | ENSG00000269235 | 19 | 51949115 | 51981367 |
| ENST00000597336 | 89.8570244 | -1.028211069 | 0.34069 | -3.01802 | 0.002544292 | 0.020463065 | ENSG00000269069 | 19 | 40023383 | 40025502 |
| ENST00000597850 | 14.0084788 | -2.856536913 | 0.965288 | -2.95926 | 0.003083801 | 0.023735111 | ENSG00000171649 | 19 | 57584144 | 57593890 |
| ENST00000600059 | 119.890479 | -1.119633339 | 0.321016 | -3.48778 | 0.000487044 | 0.005461298 | ENSG00000142235 | 19 | 48485270 | 48511835 |
| ENST00000600882 | 41.9307487 | -1.471918025 | 0.496467 | -2.96478 | 0.00302895 | 0.023402085 | ENSG00000019144 | 11 | 118607614 | 118658028 |
| ENST00000602508 | 7.2653806 | -6.313055698 | 1.805064 | -3.49741 | 0.000469794 | 0.005309629 | ENSG00000269911 | X | 72777607 | 72779097 |
| ENST00000604082 | 5.30015889 | 5.837873963 | 1.932426 | 3.021008 | 0.002519346 | 0.02029591 | ENSG00000271265 | 6 | 156774216 | 156774662 |
| ENST00000604404 | 346.590416 | 1.071281153 | 0.22375 | 4.787839 | 1.69E-06 | 4.64E-05 | ENSG00000089356 | 19 | 35115822 | 35124324 |
| ENST00000607357 | 114.240301 | 1.236697404 | 0.312769 | 3.954027 | 7.68E-05 | 0.001180276 | ENSG00000172428 | 2 | 240130827 | 240136305 |
| ENST00000608012 | 92.1514963 | 1.39667337 | 0.341235 | 4.09299 | 4.26E-05 | 0.000722641 | ENSG00000273084 | 7 | 5428183 | 5430566 |
| ENST00000609104 | 756.486399 | 1.080957206 | 0.162604 | 6.647776 | 2.98E-11 | 2.28E-09 | ENSG00000148429 | 10 | 11460509 | 11611650 |
| ENST00000609475 | 16.3660914 | 2.462385768 | 0.832827 | 2.95666 | 0.003109908 | 0.023888362 | ENSG00000272977 | 22 | 25476217 | 25479971 |
| ENST00000609713 | 29.2537503 | -1.666001668 | 0.604562 | -2.75572 | 0.005856384 | 0.038961182 | ENSG00000157542 | 21 | 37607372 | 37916457 |
| ENST00000609836 | 5.09521847 | -5.798452607 | 1.953229 | -2.96865 | 0.002991118 | 0.023176144 | ENSG00000242021 | X | 27042906 | 27158993 |
| ENST00000610122 | 69.7623946 | 1.537335057 | 0.395186 | 3.890157 | 0.00010018 | 0.001474507 | ENSG00000234432 | 7 | 5426276 | 5428927 |
| ENST00000610322 | 333.395932 | -1.067510606 | 0.213529 | -4.99936 | 5.75E-07 | 1.82E-05 | ENSG00000274523 | 7 | 75042182 | 75073802 |
| ENST00000610578 | 78.492135 | -1.191425247 | 0.36635 | -3.25215 | 0.001145358 | 0.011001591 | ENSG00000291158 | 1 | 149607343 | 149679523 |
| ENST00000610674 | 39208.7322 | -1.061665112 | 0.130679 | -8.12425 | 4.50E-16 | 6.65E-14 | ENSG00000278771 | 14 | 49853615 | 49853914 |
| ENST00000610745 | 25.9991697 | 4.309737515 | 0.870393 | 4.951483 | 7.37E-07 | 2.27E-05 | ENSG00000138061 | 2 | 38067508 | 38076151 |
| ENST00000610776 | 11.0666017 | 5.422962412 | 1.671603 | 3.244168 | 0.001177942 | 0.01125663 | ENSG00000135253 | 7 | 128876864 | 128910709 |
| ENST00000610841 | 15.4020203 | 7.37942027 | 1.6235 | 4.545376 | 5.48E-06 | 0.00012678 | ENSG00000275504 | 16 | 68926893 | 68926957 |
| ENST00000611582 | 202.427088 | 1.12162637 | 0.240757 | 4.658742 | 3.18E-06 | 7.98E-05 | ENSG00000135931 | 2 | 231198630 | 231376848 |
| ENST00000611639 | 33.5939837 | 2.267344408 | 0.577532 | 3.925921 | 8.64E-05 | 0.001302407 | ENSG00000189366 | 3 | 125929271 | 125990537 |
| ENST00000611716 | 564.028283 | -1.20105217 | 0.174763 | -6.87245 | 6.31E-12 | 5.56E-10 | ENSG00000140479 | 15 | 101303932 | 101489707 |
| ENST00000611956 | 7.2653806 | -6.313055698 | 1.805064 | -3.49741 | 0.000469794 | 0.005309629 | ENSG00000273980 | 10 | 133257143 | 133257551 |
| ENST00000612286 | 33.5757353 | 1.599716077 | 0.561391 | 2.849555 | 0.004378039 | 0.031192601 | ENSG00000105997 | 7 | 27107009 | 27152583 |
| ENST00000612813 | 78.9593955 | 5.452796981 | 0.650666 | 8.380331 | 5.28E-17 | 8.81E-15 | ENSG00000165949 | 14 | 94110814 | 94116695 |
| ENST00000612829 | 303.797592 | 1.29119406 | 0.222241 | 5.809872 | 6.25E-09 | 3.08E-07 | ENSG00000284378 | 2 | 231713313 | 231713398 |

| | | | | |
|---|---|---|---|---|
| - | protein_coding | Yes | - | 108 |
| ANXA8 | protein_coding | Yes | NM_001040084.3 | 108 |
| GASK1B | protein_coding | Yes | NM_001128424.2 | 108 |
| TNFRSF11A | protein_coding | Yes | NM_003839.4 | 108 |
| - | lncRNA | Yes | - | 108 |
| YIPF2 | protein_coding | Yes | NM_001321439.2 | 108 |
| - | lncRNA | Yes | - | 108 |
| ZNF396 | protein_coding | Yes | NM_001322286.2 | 108 |
| UNK | protein_coding | Yes | NM_001080419.3 | 108 |
| ZSWIM4 | protein_coding | Yes | NM_001367834.3 | 108 |
| MAMDC2-AS1 | lncRNA | Yes | - | 108 |
| TEAD2 | protein_coding | Yes | NM_001256660.2 | 108 |
| ZNF350-AS1 | lncRNA | Yes | - | 108 |
| - | essed_pseudog | Yes | - | 108 |
| ZIK1 | protein_coding | Yes | NM_001010879.4 | 108 |
| LMTK3 | protein_coding | Yes | NM_001388485.1 | 108 |
| PHLDB1 | protein_coding | Yes | NM_001144758.3 | 108 |
| FAM226B | lncRNA | Yes | - | 108 |
| - | lncRNA | Yes | - | 108 |
| FXYD3 | protein_coding | Yes | NM_005971.4 | 108 |
| COPS9 | protein_coding | Yes | NM_001163424.2 | 108 |
| - | lncRNA | Yes | - | 108 |
| USP6NL | protein_coding | Yes | NM_014688.5 | 108 |
| - | lncRNA | Yes | - | 108 |
| KCNJ6 | protein_coding | Yes | NM_002240.5 | 108 |
| - | lncRNA | Yes | - | 108 |
| LINC02983 | lncRNA | Yes | - | 108 |
| RCC1L | protein_coding | Yes | NM_030798.5 | 108 |
| LINC00869 | lncRNA | Yes | - | 108 |
| RN7SL3 | misc_RNA | Yes | - | 108 |
| CYP1B1 | protein_coding | Yes | NM_000104.4 | 108 |
| KCP | protein_coding | Yes | NM_001366122.1 | 108 |
| U7 | snRNA | Yes | - | 108 |
| ARMC9 | protein_coding | Yes | NM_001352754.2 | 108 |
| ALG1L | _unprocessed_p | Yes | - | 108 |
| PCSK6 | protein_coding | Yes | NM_002570.5 | 108 |
| - | lncRNA | Yes | - | 108 |
| HOXA3 | protein_coding | Yes | NM_153631.3 | 108 |
| IFI27 | protein_coding | Yes | - | 108 |
| MIR1244-1 | miRNA | Yes | - | 108 |

| | | | | | | | | | | |
|---|---|---|---|---|---|---|---|---|---|---|
| ENST00000613161 | 8.71362764 | -5.082159722 | 1.728389 | -2.9404 | 0.003277866 | 0.024876323 | ENSG00000273674 | 15 | 50839875 | 50908599 |
| ENST00000613986 | 104.725696 | 1.710674147 | 0.350991 | 4.87384 | 1.09E-06 | 3.21E-05 | ENSG00000196684 | 19 | 16143684 | 16158575 |
| ENST00000614341 | 4.68493096 | 5.663623189 | 1.978177 | 2.863052 | 0.004195813 | 0.030141529 | ENSG00000182040 | 17 | 74916082 | 74923255 |
| ENST00000614521 | 57.5968344 | 1.208860746 | 0.428012 | 2.824365 | 0.00473744 | 0.033149239 | ENSG00000157680 | 7 | 137381036 | 137846974 |
| ENST00000614758 | 166.793954 | 1.655089449 | 0.312662 | 5.293549 | 1.20E-07 | 4.57E-06 | ENSG00000284570 | 8 | 100702967 | 100703024 |
| ENST00000614771 | 34.0046757 | -1.704703998 | 0.560133 | -3.04339 | 0.002339293 | 0.019186999 | ENSG00000278709 | 20 | 57710155 | 57712780 |
| ENST00000615189 | 60.0597052 | 1.489771757 | 0.420469 | 3.54312 | 0.000395423 | 0.004622922 | ENSG00000100003 | 22 | 30397017 | 30425303 |
| ENST00000615353 | 134.886436 | 6.467185832 | 0.647427 | 9.98905 | 1.70E-23 | 5.34E-21 | ENSG00000276180 | 6 | 27139281 | 27139678 |
| ENST00000615648 | 4398.33835 | -1.04042279 | 0.114196 | -9.11086 | 8.17E-20 | 1.88E-17 | ENSG00000261150 | 8 | 143857323 | 143878467 |
| ENST00000615674 | 24.0098101 | -1.851485161 | 0.659299 | -2.80826 | 0.004980947 | 0.034440934 | ENSG00000273712 | 6 | 28315612 | 28315883 |
| ENST00000616316 | 22.7360313 | -2.227882094 | 0.706508 | -3.15337 | 0.001613969 | 0.014411118 | ENSG00000185201 | 11 | 307815 | 309395 |
| ENST00000617533 | 137.358654 | -1.311109797 | 0.298269 | -4.39573 | 1.10E-05 | 0.000230419 | ENSG00000174938 | 16 | 29871158 | 29899550 |
| ENST00000617673 | 4.26697111 | -5.544915273 | 2.022592 | -2.74149 | 0.006116115 | 0.040284792 | ENSG00000284280 | 8 | 27610600 | 27610751 |
| ENST00000617714 | 57.0565124 | -4.195438632 | 0.622368 | -6.74109 | 1.57E-11 | 1.27E-09 | ENSG00000237412 | 2 | 232520387 | 232525716 |
| ENST00000618081 | 51.5228225 | 1.331063928 | 0.464175 | 2.867589 | 0.004136129 | 0.02980202 | ENSG00000291090 | 17 | 18537799 | 18625620 |
| ENST00000618183 | 1774.81111 | 1.236876641 | 0.134031 | 9.228307 | 2.75E-20 | 6.65E-18 | ENSG00000122566 | 7 | 26189926 | 26200746 |
| ENST00000618526 | 10.5818911 | -5.386043925 | 1.676262 | -3.21313 | 0.001312983 | 0.012264889 | ENSG00000275756 | 9 | 92104424 | 92105451 |
| ENST00000618835 | 90.5104515 | -1.068641731 | 0.350284 | -3.05078 | 0.002282448 | 0.018817775 | ENSG00000278600 | 15 | 79920194 | 79922455 |
| ENST00000619162 | 98.2296614 | 1.495014773 | 0.345747 | 4.324017 | 1.53E-05 | 0.000305721 | ENSG00000264230 | 10 | 46375775 | 46391778 |
| ENST00000619168 | 208.432343 | 2.026690478 | 0.260383 | 7.783483 | 7.06E-15 | 9.22E-13 | ENSG00000183049 | 10 | 12349546 | 12835545 |
| ENST00000619208 | 81.18288 | -1.089916738 | 0.365223 | -2.98425 | 0.00284272 | 0.022293764 | ENSG00000186862 | 10 | 101007678 | 101031129 |
| ENST00000619781 | 6.23701111 | 6.074526403 | 1.85872 | 3.268123 | 0.001082632 | 0.010496488 | ENSG00000278737 | 15 | 65083041 | 65083663 |
| ENST00000619870 | 43.6302121 | -1.759824061 | 0.492307 | -3.57465 | 0.0003507 | 0.004199813 | ENSG00000290951 | 7 | 75359193 | 75395368 |
| ENST00000620220 | 10.3242069 | -3.20863541 | 1.143854 | -2.80511 | 0.005029949 | 0.034687713 | ENSG00000282301 | 7 | 99684956 | 99735102 |
| ENST00000621053 | 9.65271126 | 3.120861867 | 1.166538 | 2.675319 | 0.007465826 | 0.046942823 | ENSG00000276302 | 6 | 28267120 | 28281580 |
| ENST00000621141 | 9.35674581 | -4.155739991 | 1.379638 | -3.0122 | 0.002593654 | 0.020769432 | ENSG00000183671 | 2 | 206175315 | 206213371 |
| ENST00000622683 | 617.861104 | -1.115333529 | 0.17198 | -6.48524 | 8.86E-11 | 6.19E-09 | ENSG00000275832 | 17 | 38428463 | 38512385 |
| ENST00000623243 | 7.25980228 | 4.796455464 | 1.805403 | 2.656723 | 0.007890429 | 0.048983384 | ENSG00000280228 | 2 | 112840327 | 112844195 |
| ENST00000623250 | 65.5319671 | 1.177394284 | 0.395659 | 2.97578 | 0.002922444 | 0.022761901 | ENSG00000279348 | 2 | 216211403 | 216213519 |
| ENST00000624440 | 7.32976889 | -4.820264511 | 1.771353 | -2.72123 | 0.006503898 | 0.042280654 | ENSG00000279235 | 15 | 78906126 | 78906809 |
| ENST00000626934 | 11.9395579 | -2.845621021 | 1.034269 | -2.75133 | 0.005935302 | 0.039359725 | ENSG00000281909 | 15 | 22480438 | 22484840 |
| ENST00000627620 | 224.323095 | 1.231758503 | 0.239812 | 5.136341 | 2.80E-07 | 9.59E-06 | ENSG00000196876 | 12 | 51591232 | 51812864 |
| ENST00000630421 | 12.641173 | -3.530779114 | 1.084136 | -3.25677 | 0.001126889 | 0.01085109 | ENSG00000280734 | 13 | 99486961 | 99496772 |
| ENST00000631190 | 34.0671844 | 1.580841657 | 0.548453 | 2.882363 | 0.003947053 | 0.028710549 | ENSG00000280798 | 11 | 33076148 | 33079454 |
| ENST00000631195 | 18.7298201 | -3.111607827 | 0.867365 | -3.58742 | 0.000333961 | 0.004033227 | ENSG00000281097 | 11 | 129612115 | 129617269 |
| ENST00000634942 | 68.2677873 | 1.303075413 | 0.405606 | 3.212666 | 0.00131509 | 0.012276905 | ENSG00000282851 | 19 | 17406103 | 17419324 |
| ENST00000635031 | 307.448305 | -1.396471361 | 0.212239 | -6.57972 | 4.71E-11 | 3.46E-09 | ENSG00000170846 | 4 | 6674074 | 6676047 |
| ENST00000635120 | 37.3183703 | 1.914393334 | 0.543924 | 3.519596 | 0.000432204 | 0.004973684 | ENSG00000183117 | 8 | 2935360 | 4994914 |
| ENST00000636215 | 284.42184 | 1.161226762 | 0.211453 | 5.491656 | 3.98E-08 | 1.70E-06 | ENSG00000175105 | 3 | 88059254 | 88144660 |
| ENST00000636341 | 56.9588045 | 1.605032513 | 0.433359 | 3.703704 | 0.000212474 | 0.002763462 | ENSG00000225868 | 19 | 37829049 | 37836964 |

| | | | | |
|---|---|---|---|---|
| - | lncRNA | Yes | - | 108 |
| HSH2D | protein_coding | Yes | NM_001382417.1 | 108 |
| USH1G | protein_coding | Yes | NM_173477.5 | 108 |
| DGKI | protein_coding | Yes | NM_001321708.2 | 108 |
| MIR7705 | miRNA | Yes | - | 108 |
| NKILA | lncRNA | Yes | - | 108 |
| SEC14L2 | protein_coding | Yes | NM_012429.5 | 108 |
| H4C9 | protein_coding | Yes | NM_003495.3 | 108 |
| EPPK1 | protein_coding | Yes | NM_031308.4 | 108 |
| - | essed_pseudo | Yes | - | 108 |
| IFITM2 | protein_coding | Yes | NM_006435.3 | 108 |
| SEZ6L2 | protein_coding | Yes | NM_001243332.2 | 108 |
| MIR6843 | miRNA | Yes | - | 108 |
| PRSS56 | protein_coding | Yes | NM_001195129.2 | 108 |
| CCDC144BP | lncRNA | Yes | - | 108 |
| HNRNPA2B1 | protein_coding | Yes | NM_002137.4 | 108 |
| - | essed_pseudo | Yes | - | 108 |
| - | lncRNA | Yes | - | 108 |
| ANXA8L1 | protein_coding | Yes | NM_001098845.3 | 108 |
| CAMK1D | protein_coding | Yes | NM_153498.4 | 108 |
| PDZD7 | protein_coding | Yes | NM_001195263.2 | 108 |
| - | lncRNA | Yes | - | 108 |
| - | lncRNA | Yes | - | 108 |
| CYP3A7-CYP3A51P | protein_coding | Yes | - | 108 |
| - | protein_coding | Yes | - | 108 |
| CMKLR2 | protein_coding | Yes | NM_001389445.1 | 108 |
| ARHGAP23 | protein_coding | Yes | NM_001199417.2 | 108 |
| - | TEC | Yes | - | 108 |
| - | TEC | Yes | - | 108 |
| - | TEC | Yes | - | 108 |
| HERC2P7 | cessed_pseud | Yes | - | 108 |
| SCN8A | protein_coding | Yes | NM_001330260.2 | 108 |
| LINC01232 | lncRNA | Yes | - | 108 |
| LINC00294 | lncRNA | Yes | - | 108 |
| LINC01395 | lncRNA | Yes | - | 108 |
| BISPR | lncRNA | Yes | - | 108 |
| - | protein_coding | Yes | NM_138699.3 | 108 |
| CSMD1 | protein_coding | Yes | NM_033225.6 | 108 |
| ZNF654 | protein_coding | Yes | NM_001350134.2 | 108 |
| WDR87BP | _unprocessed_ | Yes | - | 108 |

| | | | | | | | | | | |
|---|---|---|---|---|---|---|---|---|---|---|
| ENST00000636498 | 5.68050391 | 5.941347995 | 1.894273 | 3.13648 | 0.001709892 | 0.015051413 | ENSG00000283441 | 20 | 34990399 | 34990472 |
| ENST00000638170 | 219.987896 | -1.275293564 | 0.240194 | -5.30942 | 1.10E-07 | 4.24E-06 | ENSG00000259330 | 15 | 40323691 | 40326715 |
| ENST00000639008 | 7.34788337 | 4.813047146 | 1.781854 | 2.701145 | 0.006910118 | 0.044307722 | ENSG00000204791 | 8 | 144049078 | 144051203 |
| ENST00000640019 | 480.975388 | 1.17956767 | 0.179483 | 6.572012 | 4.96E-11 | 3.63E-09 | ENSG00000284024 | 10 | 14838305 | 14846999 |
| ENST00000642384 | 125.849451 | 1.043567166 | 0.307503 | 3.393686 | 0.000689587 | 0.007261061 | ENSG00000119866 | 2 | 60457193 | 60553654 |
| ENST00000642385 | 6336.12461 | -1.900675486 | 0.11472 | -16.5679 | 1.19E-61 | 1.06E-57 | ENSG00000159921 | 9 | 36214440 | 36258448 |
| ENST00000642603 | 11.3392334 | 5.465918588 | 1.674189 | 3.264815 | 0.001095354 | 0.010597401 | ENSG00000187554 | 1 | 223109403 | 223143248 |
| ENST00000643898 | 49.3803212 | 2.205561538 | 0.479561 | 4.599128 | 4.24E-06 | 0.000102126 | ENSG00000111913 | 6 | 24804283 | 24935960 |
| ENST00000643944 | 1586.03753 | -1.168271505 | 0.167883 | -6.95884 | 3.43E-12 | 3.17E-10 | ENSG00000185803 | 8 | 144358612 | 144361272 |
| ENST00000644032 | 15.1156463 | 3.492438628 | 1.112836 | 3.138324 | 0.001699172 | 0.014980724 | ENSG00000142347 | 19 | 8520777 | 8577442 |
| ENST00000644917 | 33.2029756 | 2.089588113 | 0.591469 | 3.532881 | 0.000411057 | 0.004776889 | ENSG00000070182 | 14 | 64746282 | 64879907 |
| ENST00000644974 | 307.383969 | 1.035746781 | 0.216351 | 4.787348 | 1.69E-06 | 4.65E-05 | ENSG00000141068 | 17 | 27456447 | 27626435 |
| ENST00000645235 | 7.30040852 | -4.837452239 | 1.774899 | -2.72548 | 0.006420801 | 0.041861093 | ENSG00000261040 | 17 | 60085108 | 60088286 |
| ENST00000645453 | 77.9449546 | 1.480208372 | 0.434233 | 3.408786 | 0.000652527 | 0.006930219 | ENSG00000266714 | 17 | 75587799 | 75626849 |
| ENST00000645805 | 258.439024 | -1.843016247 | 0.27644 | -6.66696 | 2.61E-11 | 2.02E-09 | ENSG00000214049 | 19 | 15828205 | 15836136 |
| ENST00000646209 | 165.136292 | 3.114809022 | 0.300797 | 10.3552 | 3.96E-25 | 1.43E-22 | ENSG00000151090 | 3 | 24117152 | 24494850 |
| ENST00000646441 | 209.114805 | -1.30036146 | 0.261881 | -4.96547 | 6.85E-07 | 2.13E-05 | ENSG00000125266 | 13 | 106489744 | 106535662 |
| ENST00000646952 | 14.0654566 | 2.833815518 | 0.964374 | 2.938503 | 0.00329801 | 0.024987795 | ENSG00000291251 | 12 | 31158896 | 31172377 |
| ENST00000647584 | 174.455872 | 1.136016175 | 0.257585 | 4.410256 | 1.03E-05 | 0.00021767 | ENSG00000132199 | 18 | 670317 | 712630 |
| ENST00000647725 | 58.5380189 | -2.121041206 | 0.439784 | -4.82291 | 1.41E-06 | 4.00E-05 | ENSG00000285708 | 3 | 70959225 | 71754229 |
| ENST00000647814 | 259.98835 | -1.739881705 | 0.248681 | -6.99644 | 2.63E-12 | 2.47E-10 | ENSG00000023839 | 10 | 99782639 | 99852594 |
| ENST00000647941 | 165.136468 | 1.317781801 | 0.271674 | 4.850603 | 1.23E-06 | 3.55E-05 | ENSG00000080819 | 3 | 98579445 | 98593611 |
| ENST00000647956 | 192.372083 | 1.267770708 | 0.260686 | 4.863203 | 1.16E-06 | 3.36E-05 | ENSG00000159403 | 12 | 7080218 | 7092445 |
| ENST00000648011 | 79.2353305 | 1.211235255 | 0.36664 | 3.30361 | 0.000954484 | 0.0094734 | ENSG00000291141 | 1 | 121519122 | 121575702 |
| ENST00000648240 | 348.352792 | 1.072508209 | 0.223542 | 4.797793 | 1.60E-06 | 4.45E-05 | ENSG00000285526 | 19 | 35106509 | 35124297 |
| ENST00000649015 | 154.305469 | -1.220027169 | 0.272879 | -4.47095 | 7.79E-06 | 0.000171511 | ENSG00000150995 | 3 | 4493347 | 4847506 |
| ENST00000649528 | 103.359913 | -1.78978211 | 0.337258 | -5.30686 | 1.12E-07 | 4.30E-06 | ENSG00000114861 | 3 | 70954707 | 71583728 |
| ENST00000649529 | 1159.01898 | 2.30369978 | 0.193373 | 11.91324 | 1.01E-32 | 8.20E-30 | ENSG00000187608 | 1 | 1013496 | 1014540 |
| ENST00000650340 | 4.39199718 | 5.572166461 | 2.017142 | 2.762406 | 0.005737708 | 0.038325182 | ENSG00000285712 | 10 | 43325832 | 43350792 |
| ENST00000650553 | 18.3376917 | -3.054647266 | 0.857847 | -3.56083 | 0.000369686 | 0.004379799 | ENSG00000234147 | 6 | 140575811 | 140898381 |
| ENST00000650579 | 8.34881301 | -5.035652247 | 1.740455 | -2.8933 | 0.003812216 | 0.027941026 | ENSG00000205293 | 8 | 57855499 | 57984114 |
| ENST00000650711 | 9.28936676 | 5.172383992 | 1.716593 | 3.013168 | 0.00258536 | 0.020715093 | ENSG00000108576 | 17 | 30194318 | 30235697 |
| ENST00000650905 | 200.333991 | 1.727438795 | 0.276119 | 6.256133 | 3.95E-10 | 2.46E-08 | ENSG00000172936 | 3 | 38138660 | 38143022 |
| ENST00000651006 | 24.7280212 | 2.362433265 | 0.677531 | 3.486825 | 0.000488792 | 0.005476838 | ENSG00000251363 | 14 | 40954692 | 41149309 |
| ENST00000651358 | 925.967211 | -1.679445397 | 0.156661 | -10.7203 | 8.18E-27 | 3.66E-24 | ENSG00000164970 | 9 | 34398183 | 34458570 |
| ENST00000651449 | 10.9257338 | -5.419379897 | 1.677673 | -3.2303 | 0.00123662 | 0.011707799 | ENSG00000244128 | 3 | 165460538 | 165846519 |
| ENST00000651514 | 8.12865589 | -4.995533567 | 1.754803 | -2.84678 | 0.004416434 | 0.031402496 | ENSG00000160868 | 7 | 99756966 | 99784184 |
| ENST00000652361 | 82.3173574 | 2.041590328 | 0.37406 | 5.457917 | 4.82E-08 | 2.02E-06 | ENSG00000176928 | 5 | 75025345 | 75052558 |
| ENST00000653200 | 35.3116962 | 1.699168425 | 0.549277 | 3.093465 | 0.001978337 | 0.016828276 | ENSG00000227533 | 1 | 42959077 | 42996800 |
| ENST00000653713 | 23.695104 | -2.093974913 | 0.681371 | -3.07318 | 0.00211791 | 0.017742897 | ENSG00000287985 | 7 | 64369240 | 64375584 |

| | | | | |
|---|---|---|---|---|
| MIR499B | miRNA | Yes | - | 108 |
| INAFM2 | protein_coding | Yes | NM_001301268.2 | 108 |
| SMPD5 | ed_unitary_pse | Yes | - | 108 |
| - | protein_coding | Yes | NM_001378785.1 | 108 |
| BCL11A | protein_coding | Yes | NM_022893.4 | 108 |
| GNE | protein_coding | Yes | NM_005476.7 | 108 |
| TLR5 | protein_coding | Yes | NM_003268.6 | 108 |
| RIPOR2 | protein_coding | Yes | NM_001286445.3 | 108 |
| SLC52A2 | protein_coding | Yes | NM_001363118.2 | 108 |
| MYO1F | protein_coding | Yes | NM_012335.4 | 108 |
| SPTB | protein_coding | Yes | NM_001355436.2 | 108 |
| KSR1 | protein_coding | Yes | NM_001394583.1 | 108 |
| WFDC21P | ed_unitary_pse | Yes | - | 108 |
| MYO15B | protein_coding | Yes | NM_001395058.1 | 108 |
| UCA1 | lncRNA | Yes | - | 108 |
| THRB | protein_coding | Yes | NM_001354712.2 | 108 |
| EFNB2 | protein_coding | Yes | NM_004093.4 | 108 |
| - | lncRNA | Yes | - | 108 |
| ENOSF1 | protein_coding | Yes | NM_017512.7 | 108 |
| - | protein_coding | Yes | - | 108 |
| ABCC2 | protein_coding | Yes | NM_000392.5 | 108 |
| CPOX | protein_coding | Yes | NM_000097.7 | 108 |
| C1R | protein_coding | Yes | NM_001733.7 | 108 |
| EMBP1 | lncRNA | Yes | - | 108 |
| - | protein_coding | Yes | - | 108 |
| ITPR1 | protein_coding | Yes | NM_001378452.1 | 108 |
| FOXP1 | protein_coding | Yes | NM_001349338.3 | 108 |
| ISG15 | protein_coding | Yes | NM_005101.4 | 108 |
| - | lncRNA | Yes | - | 108 |
| - | lncRNA | Yes | - | 108 |
| LINC01602 | lncRNA | Yes | - | 108 |
| SLC6A4 | protein_coding | Yes | NM_001045.6 | 108 |
| MYD88 | protein_coding | Yes | NM_002468.5 | 108 |
| LINC02315 | lncRNA | Yes | - | 108 |
| FAM219A | protein_coding | Yes | NM_001184940.2 | 108 |
| LINC01322 | lncRNA | Yes | - | 108 |
| CYP3A4 | protein_coding | Yes | NM_017460.6 | 108 |
| GCNT4 | protein_coding | Yes | NM_001366737.1 | 108 |
| SLC2A1-DT | lncRNA | Yes | - | 108 |
| - | lncRNA | Yes | - | 108 |

| | | | | | | | | | | |
|---|---|---|---|---|---|---|---|---|---|---|
| ENST00000655075 | 21.4322617 | 2.098038751 | 0.734143 | 2.857807 | 0.004265794 | 0.030529606 | ENSG00000288029 | 3 | 54033987 | 54058864 |
| ENST00000655684 | 51.0795608 | 1.650227837 | 0.461334 | 3.577076 | 0.000347459 | 0.004170255 | ENSG00000227403 | 2 | 161244719 | 161249095 |
| ENST00000656698 | 2107.25263 | -1.61348447 | 0.13298 | -12.1332 | 7.04E-34 | 6.37E-31 | ENSG00000250920 | 4 | 103550588 | 103559277 |
| ENST00000659750 | 35.6049439 | -1.468548866 | 0.534426 | -2.7479 | 0.005997803 | 0.039688864 | ENSG00000228192 | 1 | 42832521 | 42846414 |
| ENST00000660029 | 6.97592671 | 4.748575312 | 1.792917 | 2.648519 | 0.008084538 | 0.049923358 | ENSG00000259359 | 15 | 96171574 | 96174339 |
| ENST00000661181 | 105.835405 | -2.031992076 | 0.336975 | -6.0301 | 1.64E-09 | 9.12E-08 | ENSG00000286299 | 6 | 125268086 | 125274828 |
| ENST00000661238 | 4.97786475 | 5.74958366 | 1.949778 | 2.94884 | 0.003189689 | 0.024372674 | ENSG00000286507 | 7 | 26598599 | 26617295 |
| ENST00000661790 | 68.3125854 | 1.083034927 | 0.392253 | 2.761063 | 0.005761354 | 0.038449141 | ENSG00000226476 | 1 | 60515734 | 60622712 |
| ENST00000662642 | 20.5580601 | -3.937504837 | 0.923121 | -4.26543 | 2.00E-05 | 0.000380248 | ENSG00000258743 | 14 | 82642619 | 82746664 |
| ENST00000662918 | 6.34280809 | -6.115070995 | 1.858676 | -3.29001 | 0.001001822 | 0.009853335 | ENSG00000286590 | 6 | 20321460 | 20333193 |
| ENST00000663543 | 59.8975569 | 1.836181099 | 0.436298 | 4.208549 | 2.57E-05 | 0.000471225 | ENSG00000226453 | 6 | 81844603 | 82169391 |
| ENST00000663616 | 13.2179802 | -2.731014238 | 0.950415 | -2.8735 | 0.004059551 | 0.029355217 | ENSG00000203999 | 20 | 50292708 | 50315488 |
| ENST00000665637 | 28.1374745 | 3.349027546 | 0.706801 | 4.738291 | 2.16E-06 | 5.72E-05 | ENSG00000136237 | 7 | 22118235 | 22357154 |
| ENST00000666136 | 526.163977 | -1.221846177 | 0.181053 | -6.74857 | 1.49E-11 | 1.21E-09 | ENSG00000268621 | 19 | 46189028 | 46203083 |
| ENST00000667279 | 4.46615624 | -5.612620884 | 2.01042 | -2.79177 | 0.00524213 | 0.03572226 | ENSG00000277047 | 13 | 54938603 | 54986717 |
| ENST00000669890 | 7.52748172 | -6.363928544 | 1.793231 | -3.54886 | 0.0003869 | 0.004538944 | ENSG00000235142 | 6 | 106705333 | 106787521 |
| ENST00000671505 | 9.75726255 | -4.248939893 | 1.454836 | -2.92056 | 0.003493999 | 0.026130821 | ENSG00000287853 | 20 | 34132269 | 34136485 |
| ENST00000673966 | 8.72594349 | 6.559092951 | 1.749682 | 3.748735 | 0.000177729 | 0.002374922 | ENSG00000132746 | 11 | 67662154 | 67674623 |
| ENST00000674313 | 57.6777781 | -1.359054153 | 0.424165 | -3.20407 | 0.001354981 | 0.012563171 | ENSG00000155465 | 14 | 22773221 | 22815435 |
| ENST00000674635 | 63.9025601 | 1.229307601 | 0.402477 | 3.054357 | 0.002255435 | 0.018637512 | ENSG00000116991 | 1 | 232397964 | 232630133 |
| ENST00000675268 | 84.4091658 | -1.355029136 | 0.364666 | -3.71581 | 0.000202554 | 0.002651789 | ENSG00000288634 | 7 | 112450486 | 112450645 |
| ENST00000676095 | 9.98071696 | -6.77182737 | 1.716693 | -3.94469 | 7.99E-05 | 0.001220511 | ENSG00000228509 | 2 | 190672296 | 190841864 |
| ENST00000676302 | 103.297925 | 1.145636354 | 0.322416 | 3.553283 | 0.000380455 | 0.004484371 | ENSG00000188158 | X | 17375199 | 17735994 |
| ENST00000680972 | 654.899695 | -1.130660357 | 0.163437 | -6.91803 | 4.58E-12 | 4.13E-10 | ENSG00000089060 | 12 | 113298770 | 113335109 |
| ENST00000681370 | 2067.19481 | 1.284270935 | 0.361542 | 3.552209 | 0.000382012 | 0.004497457 | ENSG00000233975 | 1 | 27660383 | 27666279 |
| ENST00000681845 | 2846.01609 | -1.275395765 | 0.367481 | -3.47064 | 0.00051922 | 0.005749154 | ENSG00000288699 | 9 | 35065338 | 35074175 |
| ENST00000682323 | 1573.8313 | 1.36870315 | 0.188143 | 7.274808 | 3.47E-13 | 3.63E-11 | ENSG00000138496 | 3 | 122527923 | 122564300 |
| ENST00000682931 | 76.5429303 | -1.102033942 | 0.367155 | -3.00155 | 0.002686073 | 0.021338778 | ENSG00000143147 | 1 | 168079541 | 168136930 |
| ENST00000683033 | 113.560826 | 1.558390628 | 0.337979 | 4.610915 | 4.01E-06 | 9.74E-05 | ENSG00000150471 | 4 | 61200325 | 62078335 |
| ENST00000683051 | 29.9325138 | 1.984593239 | 0.59912 | 3.312512 | 0.000924623 | 0.009233461 | ENSG00000228956 | 3 | 18527206 | 18923280 |
| ENST00000683152 | 50.9375336 | 1.247325832 | 0.447955 | 2.784491 | 0.005361174 | 0.036327095 | ENSG00000019485 | 11 | 45146638 | 45235109 |
| ENST00000683327 | 9.0551537 | 5.13433886 | 1.725004 | 2.976422 | 0.002916334 | 0.022727198 | ENSG00000174945 | 7 | 2688201 | 2719683 |
| ENST00000683555 | 25.5042401 | 1.765841194 | 0.640737 | 2.755953 | 0.005852149 | 0.038938147 | ENSG00000188747 | 9 | 137423392 | 137434406 |
| ENST00000683666 | 390.48323 | -1.191403911 | 0.196599 | -6.06008 | 1.36E-09 | 7.73E-08 | ENSG00000143409 | 1 | 150996834 | 151006902 |
| ENST00000684388 | 1394.12343 | 1.175781469 | 0.13368 | 8.795514 | 1.42E-18 | 2.88E-16 | ENSG00000288725 | 16 | 56430555 | 56501497 |
| ENST00000685332 | 1008.79574 | 1.103412338 | 0.144818 | 7.619298 | 2.55E-14 | 3.07E-12 | ENSG00000075407 | 10 | 38094336 | 38124625 |
| ENST00000685964 | 219.548611 | 1.10981505 | 0.244012 | 4.548194 | 5.41E-06 | 0.000125305 | ENSG00000089682 | X | 107061884 | 107118822 |
| ENST00000686162 | 65.8265045 | -1.125544574 | 0.410817 | -2.73977 | 0.006148163 | 0.040441242 | ENSG00000289562 | 11 | 62574173 | 62575097 |
| ENST00000687343 | 10.3694064 | 3.665486325 | 1.21225 | 3.023704 | 0.002497006 | 0.020169699 | ENSG00000237943 | 10 | 6580505 | 6615954 |
| ENST00000687513 | 44.8241671 | -1.302360027 | 0.475566 | -2.73855 | 0.00617115 | 0.040567731 | ENSG00000288744 | 12 | 103965892 | 103966794 |

| | | | | |
|---|---|---|---|---|
| - | lncRNA | Yes | - | 108 |
| LINC01806 | lncRNA | Yes | - | 108 |
| - | lncRNA | Yes | - | 108 |
| ZNF691-DT | lncRNA | Yes | - | 108 |
| - | lncRNA | Yes | - | 108 |
| - | lncRNA | Yes | - | 108 |
| - | lncRNA | Yes | - | 108 |
| LINC01748 | lncRNA | Yes | - | 108 |
| LINC02301 | lncRNA | Yes | - | 108 |
| - | lncRNA | Yes | - | 108 |
| LINC02542 | lncRNA | Yes | - | 108 |
| LINC01270 | lncRNA | Yes | - | 108 |
| RAPGEF5 | protein_coding | Yes | NM_012294.5 | 108 |
| IGFL2-AS1 | lncRNA | Yes | - | 108 |
| - | lncRNA | Yes | - | 108 |
| LINC02532 | lncRNA | Yes | - | 108 |
| - | lncRNA | Yes | - | 108 |
| ALDH3B2 | protein_coding | Yes | NM_001393402.2 | 108 |
| SLC7A7 | protein_coding | Yes | NM_003982.4 | 108 |
| SIPA1L2 | protein_coding | Yes | NM_020808.5 | 108 |
| - | protein_coding | Yes | - | 108 |
| - | lncRNA | Yes | - | 108 |
| NHS | protein_coding | Yes | NM_001291867.2 | 108 |
| SLC8B1 | protein_coding | Yes | NM_001358345.2 | 108 |
| LINC02574 | lncRNA | Yes | - | 108 |
| - | protein_coding | Yes | - | 108 |
| PARP9 | protein_coding | Yes | NM_001146105.2 | 108 |
| GPR161 | protein_coding | Yes | NM_001375883.1 | 108 |
| ADGRL3 | protein_coding | Yes | NM_001387552.1 | 108 |
| SATB1-AS1 | lncRNA | Yes | - | 108 |
| PRDM11 | protein_coding | Yes | NM_001384648.1 | 108 |
| AMZ1 | protein_coding | Yes | NM_001384743.1 | 108 |
| NOXA1 | protein_coding | Yes | NM_001256067.2 | 108 |
| MINDY1 | protein_coding | Yes | NM_001376665.1 | 108 |
| - | protein_coding | Yes | - | 108 |
| ZNF37A | protein_coding | Yes | NM_001324250.3 | 108 |
| RBM41 | protein_coding | Yes | NM_001324242.2 | 108 |
| - | lncRNA | Yes | - | 108 |
| PRKCQ-AS1 | lncRNA | Yes | - | 108 |
| - | lncRNA | Yes | - | 108 |

| | | | | | | | | | | |
|---|---|---|---|---|---|---|---|---|---|---|
| ENST00000688159 | 4.538799 | 5.614285089 | 2.00097 | 2.805781 | 0.005019472 | 0.034639173 | ENSG00000289141 | 1 | 161389546 | 161389965 |
| ENST00000688547 | 154.873627 | 1.500078159 | 0.306838 | 4.888821 | 1.01E-06 | 3.00E-05 | ENSG00000140853 | 16 | 56989556 | 57083520 |
| ENST00000688585 | 702.692935 | -1.841041863 | 0.162741 | -11.3127 | 1.14E-29 | 8.02E-27 | ENSG00000289316 | 8 | 133237170 | 133237927 |
| ENST00000688720 | 140.4619 | -4.16610263 | 0.390528 | -10.6679 | 1.44E-26 | 6.29E-24 | ENSG00000196562 | 20 | 47657405 | 47785481 |
| ENST00000688948 | 38.0211024 | -1.639638061 | 0.529635 | -3.09579 | 0.001962887 | 0.016722269 | ENSG00000064787 | 20 | 53943540 | 54070594 |
| ENST00000689451 | 33.782639 | 1.568297734 | 0.551466 | 2.843873 | 0.004456884 | 0.031624414 | ENSG00000289174 | 12 | 88781255 | 88847391 |
| ENST00000689635 | 63.7987741 | -2.715372355 | 0.443457 | -6.12319 | 9.17E-10 | 5.36E-08 | ENSG00000123094 | 12 | 25958681 | 26072869 |
| ENST00000690102 | 44.3552956 | -1.614291269 | 0.484044 | -3.33501 | 0.000852958 | 0.008635341 | ENSG00000291043 | 10 | 73730572 | 73737790 |
| ENST00000691526 | 46.6036753 | -1.352199289 | 0.472927 | -2.85921 | 0.004246951 | 0.030407711 | ENSG00000285533 | 11 | 65662987 | 65671718 |
| ENST00000691676 | 322.967254 | 3.358188386 | 0.268659 | 12.49981 | 7.48E-36 | 7.85E-33 | ENSG00000182261 | 11 | 7957536 | 7965447 |
| ENST00000692801 | 38.8463094 | 2.24058826 | 0.537745 | 4.166635 | 3.09E-05 | 0.000552821 | ENSG00000291096 | 3 | 125928688 | 125990573 |
| ENST00000693548 | 112.972003 | -1.807665963 | 0.348307 | -5.18987 | 2.10E-07 | 7.51E-06 | ENSG00000144821 | 3 | 108380367 | 108510596 |
| ENST00000694881 | 100.10805 | 1.144236698 | 0.327362 | 3.495321 | 0.000473493 | 0.005340653 | ENSG00000176155 | 17 | 82101469 | 82212842 |
| ENST00000695401 | 63.3164719 | -1.374618139 | 0.404807 | -3.39574 | 0.000684433 | 0.007217247 | ENSG00000138835 | 9 | 113463726 | 113597738 |
| ENST00000695562 | 48.0173448 | -1.561701579 | 0.465237 | -3.35679 | 0.000788535 | 0.008104183 | ENSG00000289688 | 13 | 23505237 | 23535314 |
| ENST00000695919 | 203.953041 | -3.533300442 | 0.346294 | -10.2032 | 1.92E-24 | 6.53E-22 | ENSG00000169851 | 4 | 30720368 | 31146800 |
| ENST00000695948 | 31.9341898 | -2.446546391 | 0.604749 | -4.04556 | 5.22E-05 | 0.000856552 | ENSG00000124496 | 6 | 42224930 | 42451926 |
| ENST00000696247 | 323.866893 | -1.482445139 | 0.248971 | -5.95429 | 2.61E-09 | 1.39E-07 | ENSG00000188707 | 7 | 150329870 | 150331115 |
| ENST00000697408 | 39.5896422 | -1.589284078 | 0.518261 | -3.06657 | 0.002165299 | 0.018056823 | ENSG00000289728 | 1 | 247332330 | 247448603 |
| ENST00000701030 | 19.7990989 | -3.207369332 | 0.937515 | -3.42114 | 0.000623596 | 0.006677392 | ENSG00000289850 | 6 | 144922314 | 144929987 |
| ENST00000701218 | 59.395263 | 1.367444067 | 0.418606 | 3.266663 | 0.001088232 | 0.010545749 | ENSG00000204682 | 10 | 21490271 | 21497071 |
| ENST00000701580 | 22.1016592 | -6.455354084 | 1.549485 | -4.16613 | 3.10E-05 | 0.000553719 | ENSG00000224307 | 9 | 129282509 | 129313220 |
| ENST00000702066 | 4.39199718 | 5.572166461 | 2.017142 | 2.762406 | 0.005737708 | 0.038325182 | ENSG00000281655 | 11 | 102641077 | 102683911 |
| ENST00000702115 | 12.2767111 | -7.069861435 | 1.666641 | -4.24198 | 2.22E-05 | 0.000415433 | ENSG00000289959 | X | 13089739 | 13091216 |
| ENST00000702916 | 42.7179413 | 6.387569961 | 1.116342 | 5.721876 | 1.05E-08 | 4.98E-07 | ENSG00000269834 | 19 | 52388835 | 52397713 |

| | | | | |
|---|---|---|---|---|
| - | lncRNA | Yes | - | 108 |
| NLRC5 | protein_coding | Yes | NM_001384950.1 | 108 |
| - | lncRNA | Yes | - | 108 |
| SULF2 | protein_coding | Yes | NM_001387048.1 | 108 |
| BCAS1 | protein_coding | Yes | NM_001366298.2 | 108 |
| - | lncRNA | Yes | - | 108 |
| RASSF8 | protein_coding | Yes | NM_001394098.1 | 108 |
| GLUD1P3 | lncRNA | Yes | - | 108 |
| RELA-DT | lncRNA | Yes | - | 108 |
| NLRP10 | protein_coding | Yes | NM_001391958.1 | 108 |
| - | lncRNA | Yes | - | 108 |
| MYH15 | protein_coding | Yes | NM_014981.3 | 108 |
| CCDC57 | protein_coding | Yes | NM_001394669.1 | 108 |
| RGS3 | protein_coding | Yes | NM_001394167.1 | 108 |
| - | lncRNA | Yes | - | 108 |
| PCDH7 | protein_coding | Yes | NM_001173523.2 | 108 |
| TRERF1 | protein_coding | Yes | NM_001395490.1 | 108 |
| ZBED10P | ed_unitary_pse | Yes | - | 108 |
| - | lncRNA | Yes | - | 108 |
| - | lncRNA | Yes | - | 108 |
| MIR1915HG | lncRNA | Yes | - | 108 |
| LINC02975 | lncRNA | Yes | - | 108 |
| - | lncRNA | Yes | - | 108 |
| - | lncRNA | Yes | - | 108 |
| ZNF528-AS1 | lncRNA | Yes | - | 108 |
