# Supplementary material for: Combined loss of CDH1 and downstream regulatory sequences drive early-onset diffuse gastric cancer and increase penetrance of hereditary diffuse gastric cancer
Source: Gastric Cancer. 2023 May 30;26(5):653–66. doi: 10.1007/s10120-023-01395-0 (PMC10361908; doi:10.1007/s10120-023-01395-0)
Supplement: Supplementary file 9 — Supplementary file9 (PDF 131 KB) [file 10120_2023_1395_MOESM9_ESM.pdf]

**Supplementary table 8.** Enriched go terms of differentially expressed genes

| ID | Comparison.ID | Description | GeneRatio | BgRatio | pvalue |
|---|---|---|---|---|---|
| GO:0000786 | CDH1-TANGO6 del vs CDH1 WT | nucleosome | 59/1858 | 129/19594 | 7.26E-27 |
| GO:0044815 | CDH1-TANGO6 del vs CDH1 WT | DNA packaging complex | 68/1858 | 198/19594 | 4.77E-22 |
| GO:0032993 | CDH1-TANGO6 del vs CDH1 WT | protein-DNA complex | 71/1858 | 220/19594 | 3.48E-21 |
| GO:0000786 | CDH1 del vs CDH1 WT | nucleosome | 53/2417 | 129/19594 | 1.70E-16 |
| GO:0006334 | CDH1-TANGO6 del vs CDH1 WT | nucleosome assembly | 47/1788 | 126/18800 | 3.00E-17 |
| GO:0044815 | CDH1 del vs CDH1 WT | DNA packaging complex | 66/2417 | 198/19594 | 8.78E-15 |
| GO:0034728 | CDH1-TANGO6 del vs CDH1 WT | nucleosome organization | 51/1788 | 159/18800 | 1.96E-15 |
| GO:0032993 | CDH1 del vs CDH1 WT | protein-DNA complex | 69/2417 | 220/19594 | 6.61E-14 |
| GO:0065004 | CDH1-TANGO6 del vs CDH1 WT | protein-DNA complex assembly | 53/1788 | 203/18800 | 5.70E-12 |
| GO:0031497 | CDH1-TANGO6 del vs CDH1 WT | chromatin assembly | 53/1788 | 205/18800 | 8.58E-12 |
| GO:0043505 | CDH1-TANGO6 del vs CDH1 WT | CENP-A containing nucleosome | 13/1858 | 18/19594 | 2.62E-10 |
| GO:0061638 | CDH1-TANGO6 del vs CDH1 WT | CENP-A containing chromatin | 13/1858 | 18/19594 | 2.62E-10 |
| GO:0071824 | CDH1-TANGO6 del vs CDH1 WT | protein-DNA complex subunit organization | 57/1788 | 237/18800 | 3.30E-11 |
| GO:0043505 | CDH1 del vs CDH1 WT | CENP-A containing nucleosome | 14/2417 | 18/19594 | 3.44E-10 |
| GO:0061638 | CDH1 del vs CDH1 WT | CENP-A containing chromatin | 14/2417 | 18/19594 | 3.44E-10 |
| GO:0045071 | CDH1-TANGO6 del vs CDH1 del | negative regulation of viral genome replication | 18/854 | 57/18800 | 3.31E-11 |
| GO:0006338 | CDH1-TANGO6 del vs CDH1 WT | chromatin remodeling | 60/1788 | 266/18800 | 1.64E-10 |
| GO:0007156 | CDH1-TANGO6 del vs CDH1 WT | homophilic cell adhesion via plasma membrane adhesion molecules | 42/1788 | 168/18800 | 3.65E-09 |
| GO:0009615 | CDH1-TANGO6 del vs CDH1 del | response to virus | 46/854 | 392/18800 | 3.77E-09 |
| GO:0019058 | CDH1-TANGO6 del vs CDH1 del | viral life cycle | 39/854 | 317/18800 | 1.60E-08 |
| GO:0051607 | CDH1-TANGO6 del vs CDH1 del | defense response to virus | 37/854 | 290/18800 | 1.42E-08 |
| GO:0140546 | CDH1-TANGO6 del vs CDH1 del | defense response to symbiont | 37/854 | 291/18800 | 1.55E-08 |
| GO:0046982 | CDH1-TANGO6 del vs CDH1 WT | protein heterodimerization activity | 66/1834 | 332/18410 | 3.29E-08 |
| GO:0035455 | CDH1-TANGO6 del vs CDH1 del | response to interferon-alpha | 9/854 | 20/18800 | 8.44E-08 |
| GO:0006334 | CDH1 del vs CDH1 WT | nucleosome assembly | 40/2311 | 126/18800 | 7.10E-09 |
| GO:0034728 | CDH1 del vs CDH1 WT | nucleosome organization | 46/2311 | 159/18800 | 1.47E-08 |
| GO:0071357 | CDH1-TANGO6 del vs CDH1 del | cellular response to type I interferon | 14/854 | 58/18800 | 2.25E-07 |
| GO:0065004 | CDH1 del vs CDH1 WT | protein-DNA complex assembly | 53/2311 | 203/18800 | 5.72E-08 |
| GO:0071824 | CDH1 del vs CDH1 WT | protein-DNA complex subunit organization | 59/2311 | 237/18800 | 6.60E-08 |
| GO:0005788 | CDH1-TANGO6 del vs CDH1 del | endoplasmic reticulum lumen | 36/876 | 311/19594 | 1.82E-07 |
| GO:0052547 | CDH1-TANGO6 del vs CDH1 del | regulation of peptidase activity | 46/854 | 456/18800 | 3.66E-07 |
| GO:0072567 | CDH1-TANGO6 del vs CDH1 del | chemokine (C-X-C motif) ligand 2 production | 8/854 | 18/18800 | 5.12E-07 |
| GO:2000341 | CDH1-TANGO6 del vs CDH1 del | regulation of chemokine (C-X-C motif) ligand 2 production | 8/854 | 18/18800 | 5.12E-07 |
| GO:0034340 | CDH1-TANGO6 del vs CDH1 del | response to type I interferon | 14/854 | 64/18800 | 8.24E-07 |
| GO:0035456 | CDH1-TANGO6 del vs CDH1 del | response to interferon-beta | 10/854 | 32/18800 | 9.20E-07 |
| GO:0060337 | CDH1-TANGO6 del vs CDH1 del | type I interferon signaling pathway | 13/854 | 56/18800 | 9.86E-07 |
| GO:0006338 | CDH1 del vs CDH1 WT | chromatin remodeling | 62/2311 | 266/18800 | 3.88E-07 |

| p.adjust | qvalue |
|---|---|
| 5.06E-24 | 4.61E-24 |
| 1.66E-19 | 1.51E-19 |
| 8.10E-19 | 7.36E-19 |
| 1.21E-13 | 1.09E-13 |
| 1.74E-13 | 1.65E-13 |
| 3.13E-12 | 2.81E-12 |
| 5.69E-12 | 5.39E-12 |
| 1.57E-11 | 1.41E-11 |
| 1.10E-08 | 1.05E-08 |
| 1.25E-08 | 1.18E-08 |
| 3.65E-08 | 3.32E-08 |
| 3.65E-08 | 3.32E-08 |
| 3.83E-08 | 3.63E-08 |
| 4.90E-08 | 4.41E-08 |
| 4.90E-08 | 4.41E-08 |
| 1.67E-07 | 1.48E-07 |
| 1.59E-07 | 1.51E-07 |
| 2.36E-06 | 2.24E-06 |
| 3.80E-06 | 3.38E-06 |
| 8.97E-06 | 7.97E-06 |
| 8.97E-06 | 7.97E-06 |
| 8.97E-06 | 7.97E-06 |
| 3.62E-05 | 3.40E-05 |
| 3.87E-05 | 3.44E-05 |
| 4.27E-05 | 3.93E-05 |
| 4.42E-05 | 4.07E-05 |
| 8.73E-05 | 7.76E-05 |
| 9.91E-05 | 9.13E-05 |
| 9.91E-05 | 9.13E-05 |
| 0.0001 | 9.50E-05 |
| 0.00013 | 0.00012 |
| 0.00016 | 0.00014 |
| 0.00016 | 0.00014 |
| 0.00024 | 0.00022 |
| 0.00026 | 0.00023 |
| 0.00026 | 0.00023 |
| 0.00047 | 0.00043 |

| geneID |
| --- |
| H1-3/SPHK2/H2AZ1/H2AC8/H1-4/RUVBL1/H2AC21/H2AC20/H1-5/H3C13/H1-10/H2BC11/H1-0/H2AC7/H1-2/H4C11/H2BC7/H2BC12/H2AC13/H3C12/H2AC17/H2BC21/H3C14/H2AC18/H2B |
| H1-3/SPHK2/NCAPG/NUF2/SPC25/H2AZ1/H2AC8/H1-4/TERT/RUVBL1/H2AC21/H2AC20/H1-5/H3C13/H1-10/H2BC11/H1-0/H2AC7/H1-2/H4C11/DPF1/H2BC7/H2BC12/BCL11B/H2AC13/H3 |
| RPA3/H1-3/SPHK2/GINS2/GINS1/POLD3/H2AZ1/FOS/H2AC8/H1-4/TERT/GINS3/RUVBL1/NUPR1/H2AC21/H2AC20/H1-5/H3C13/H1-10/H2BC11/H1-0/H2AC7/SOX12/H1-2/DDIT3/H4C11/ |
| H1-3/SPHK2/H2BC1/H2AZ1/H2AC1/H2AC8/H1-4/RUVBL1/H2AC21/H2AC20/H1-5/H1-10/CENPA/H2BC11/H1-0/H2AC7/H1-2/H4C11/H2BC7/H2BC12/H2AC13/H3C12/VPS72/H2BC21/H2AC |
| H1-3/ITGB3BP/HMGB2/H1-4/NAP1L5/H1-5/H3C13/H1-10/H2BC11/H1-0/H1-2/H4C11/H2BC7/H2BC12/H3C12/H2BC21/H3C14/H2BC18/TSPYL2/H2BC13/H4C8/H2BC10/H2BC5/H4C3/H2BC |
| H1-3/SPHK2/NCAPG/NDC80/NUF2/H2BC1/SPC25/H2AZ1/H2AC1/H2AC8/H1-4/RUVBL1/H2AC21/H2AC20/H1-5/H1-10/CENPA/H2BC11/H1-0/H2AC7/H1-2/SMARCA2/H4C11/DPF1/H2BC7/ |
| H1-3/CHD5/ITGB3BP/SSRP1/HMGB2/H1-4/NAP1L5/H1-5/H3C13/H1-10/H2BC11/H1-0/H1-2/H4C11/H2BC7/H2BC12/H3C12/H2BC21/H3C14/H2BC18/DMAP1/TSPYL2/H2BC13/H4C8/H2BC |
| BRF2/RPA3/H1-3/SPHK2/GINS2/GINS1/POLD3/H2BC1/H2AZ1/NPM1/H2AC1/RAG1/FOS/H2AC8/H1-4/GINS3/RUVBL1/SUZ12/H2AC21/H2AC20/H1-5/H1-10/CENPA/H2BC11/H1-0/H2AC7 |
| H1-3/DLGAP5/CENPE/ITGB3BP/HMGB2/H1-4/NAP1L5/H1-5/H3C13/H1-10/H2BC11/H1-0/HMGB1/H1-2/H4C11/H2BC7/H2BC12/H3C12/H2BC21/H3C14/H2BC18/TSPYL2/H2BC13/H4C8/H2 |
| H1-3/CDK2/ITGB3BP/HMGB2/H1-4/NAP1L5/H1-5/H3C13/H1-10/H2BC11/H1-0/H1-2/H4C11/H2BC7/H2BC12/PHF2/H3C12/L3MBTL3/H2BC21/H3C14/H2BC18/TSPYL2/H2BC13/H4C8/H2BC |
| H2AC8/H2BC11/H4C11/H4C8/H4C3/H4-16/H4C14/H4C4/H4C5/H4C9/H2AC4/H4C1/H4C13 |
| H2AC8/H2BC11/H4C11/H4C8/H4C3/H4-16/H4C14/H4C4/H4C5/H4C9/H2AC4/H4C1/H4C13 |
| H1-3/DLGAP5/CHD5/CENPE/ITGB3BP/SSRP1/HMGB2/H1-4/NAP1L5/H1-5/H3C13/H1-10/H2BC11/H1-0/HMGB1/H1-2/H4C11/H2BC7/H2BC12/H3C12/H2BC21/H3C14/H2BC18/DMAP1/TSP |
| H2AC8/CENPA/H2BC11/H4C11/H4C8/H4C2/H4C3/H4-16/H4C14/H4C4/H4C5/H4C9/H2AC4/H4C13 |
| H2AC8/CENPA/H2BC11/H4C11/H4C8/H4C2/H4C3/H4-16/H4C14/H4C4/H4C5/H4C9/H2AC4/H4C13 |
| OAS1/OAS3/EIF2AK2/OASL/IFI16/APOBEC3B/SLPI/PLSCR1/IFIT5/IFIT1/RSAD2/OAS2/IFITM3/APOBEC3G/IFITM1/TNF/IFITM2/ISG15 |
| INO80B/H1-3/CHD5/ITGB3BP/SSRP1/HMGB2/H1-4/RUVBL1/NAP1L5/H1-5/H3C13/H1-10/SATB1/H2BC11/H1-0/MYB/H1-2/H4C11/H2BC7/H2BC12/H3C12/H2BC21/H3C14/H2BC18/SMARC |
| CELSR3/PCDHB5/PCDHGC5/PCDHAC1/PCDHGA3/CDH1/PTPN23/MPZL2/PCDHAC2/PCDHA13/PCDHGC4/PCDHA10/PCDHGC3/MYPN/AMIGO1/L1CAM/PCDHGA2/PCDHGB7/PCDHGA8/ |
| OAS1/OAS3/EIF2AK2/PYCARD/OASL/HERC5/SPON2/IFI16/DTX3L/DDIT4/FOSL1/MAPK11/MX2/STING1/APOBEC3B/NLRP3/AP1S1/PLSCR1/ADARB1/DCLK1/STAT1/IFI6/IFI44/IFI44L/ |
| CD74/OAS1/VPS18/OAS3/EIF2AK2/APOE/OASL/NECTIN1/LAMP3/IFI16/INSR/CXCL8/GAS6/APOBEC3B/SLPI/TRIM14/PLSCR1/SIGLEC1/ADARB1/TNFRSF14/VCP/HLA-DRB1/IFIT5/IFI |
| OAS1/OAS3/EIF2AK2/PYCARD/OASL/HERC5/SPON2/IFI16/DTX3L/DDIT4/MX2/STING1/APOBEC3B/PLSCR1/ADARB1/STAT1/IFI6/IFI44L/IFIT5/IFIT1/IFIT3/IFIT2/TRAF3IP1/ZMYND11/ |
| OAS1/OAS3/EIF2AK2/PYCARD/OASL/HERC5/SPON2/IFI16/DTX3L/DDIT4/MX2/STING1/APOBEC3B/PLSCR1/ADARB1/STAT1/IFI6/IFI44L/IFIT5/IFIT1/IFIT3/IFIT2/TRAF3IP1/ZMYND11/ |
| CAV2/IRAK2/BCL2L10/BARD1/H2AZ1/LSM6/H2AC8/AOC3/BOK/H2AC21/H2AC20/H3C13/NR4A2/H2BC11/H2AC7/DDIT3/H4C11/H2BC7/H2BC12/H2AC13/H3C12/H2AC17/SDCBP2/ATF6/ |
| EIF2AK2/LAMP3/GAS6/MX2/IFIT3/IFIT2/IFITM3/IFITM1/IFITM2 |
| ASF1A/H1-3/ITGB3BP/H2BC1/HMGB2/NPM1/H1-4/H1-5/H1-10/ANP32B/H2BC11/H1-0/H1-2/NASP/H4C11/H2BC7/H2BC12/H3C12/H2BC21/H2BC18/H2BC13/H4C8/H2BC10/H4C2/H4C3/H |
| ASF1A/H1-3/CHD5/ITGB3BP/H2BC1/SSRP1/HMGB2/NPM1/H1-4/H1-5/H1-10/ANP32B/H2BC11/H1-0/H1-2/NASP/H4C11/H2BC7/H2BC12/H3C12/VPS72/H2BC21/H2BC18/H2BC13/H4C8/ |
| OAS1/OAS3/STING1/SP100/STAT1/IFIT1/OAS2/IFITM3/IFITM1/IFI27/IFITM2/ISG15/MYD88/NLRC5 |
| BRF2/ASF1A/H1-3/DLGAP5/TAF1B/CENPE/ITGB3BP/H2BC1/HMGB2/NPM1/H1-4/CENPS/H1-5/H1-10/CENPA/ANP32B/H2BC11/H1-0/HMGB1/H1-2/NASP/H4C11/H2BC7/H2BC12/RAD52/ |
| BRF2/ASF1A/H1-3/DLGAP5/CHD5/TAF1B/CENPE/ITGB3BP/H2BC1/SSRP1/HMGB2/NPM1/H1-4/CENPS/H1-5/H1-10/CENPA/ANP32B/H2BC11/H1-0/HMGB1/H1-2/NASP/H4C11/H2BC7/H |
| CTSZ/WNT6/BMP4/C3/VGF/APOE/THBS1/STC2/IGFBP4/RCN3/ADAMTSL4/PRSS23/SHH/COL6A2/LAMB2/TSPAN5/CYP2W1/BACE1/CRTAP/FKBP10/POGLUT3/GAS6/CSF1/WNT7B/PL |
| SERPINE1/FIS1/TNFSF10/ACVR1C/C3/PYCARD/SPINK5/PI15/THBS1/LAMP3/RCN3/IFI16/RAG1/A2ML1/CST1/CST6/GAS6/NLRP3/SLPI/PLAUR/CLDN4/ACER2/MBP/SERPINH1/VCP/IFI |
| CD74/OAS1/OAS3/MBP/KLF4/TNF/FOXP1/MYD88 |
| CD74/OAS1/OAS3/MBP/KLF4/TNF/FOXP1/MYD88 |
| OAS1/OAS3/STING1/SP100/STAT1/IFIT1/OAS2/IFITM3/IFITM1/IFI27/IFITM2/ISG15/MYD88/NLRC5 |
| OAS1/IFI16/STING1/PLSCR1/STAT1/XAF1/IFITM3/IFITM1/PNPT1/IFITM2 |
| OAS1/OAS3/STING1/SP100/STAT1/OAS2/IFITM3/IFITM1/IFI27/IFITM2/ISG15/MYD88/NLRC5 |
| HCFC2/ASF1A/H1-3/MBD2/CHD5/KAT2B/ITGB3BP/H2BC1/SSRP1/HMGB2/NPM1/H1-4/RUVBL1/H1-5/H1-10/SATB1/ANP32B/H2BC11/H1-0/MYB/H1-2/SMARCA2/NASP/SUV39H2/H4C11/ |

| Count | GO | zscore |
|---|---|---|
| 59 | CC | -6.63963446 |
| 68 | CC | -7.03353313 |
| 71 | CC | -6.76465545 |
| 53 | CC | -5.90650425 |
| 47 | BP | -6.27219463 |
| 66 | CC | -6.89312349 |
| 51 | BP | -6.02120436 |
| 69 | CC | -6.13967851 |
| 53 | BP | -6.45594651 |
| 53 | BP | -5.90650425 |
| 13 | CC | -3.60555128 |
| 13 | CC | -3.60555128 |
| 57 | BP | -6.22530208 |
| 14 | CC | -3.74165739 |
| 14 | CC | -3.74165739 |
| 18 | BP | 2.3570226 |
| 60 | BP | -5.68037557 |
| 42 | BP | 4.3204938 |
| 46 | BP | 3.24372304 |
| 39 | BP | 1.76140969 |
| 37 | BP | 4.43877266 |
| 37 | BP | 4.43877266 |
| 66 | MF | -6.15457455 |
| 9 | BP | 1.66666667 |
| 40 | BP | -5.69209979 |
| 46 | BP | -5.89767825 |
| 14 | BP | 3.2071349 |
| 53 | BP | -6.18122538 |
| 59 | BP | -6.37925664 |
| 36 | CC | -3 |
| 46 | BP | -1.47441956 |
| 8 | BP | -0.70710678 |
| 8 | BP | -0.70710678 |
| 14 | BP | 3.2071349 |
| 10 | BP | 2.52982213 |
| 13 | BP | 3.05085108 |
| 62 | BP | -6.6040066 |

**Supplementary table 5 legend**

Highlight yellow: Selected pathways
Red text: Common pathways

| | | | | | |
|---|---|---|---|---|---|
| GO:0062023 | CDH1-TANGO6 del vs CDH1 del | collagen-containing extracellular matrix | 42/876 | 429/19594 | 1.73E-06 |
| GO:0031497 | CDH1 del vs CDH1 WT | chromatin assembly | 51/2311 | 205/18800 | 5.12E-07 |
| GO:0001894 | CDH1-TANGO6 del vs CDH1 del | tissue homeostasis | 31/854 | 272/18800 | 2.46E-06 |
| GO:0001819 | CDH1-TANGO6 del vs CDH1 del | positive regulation of cytokine production | 45/854 | 475/18800 | 2.72E-06 |
| GO:0045653 | CDH1-TANGO6 del vs CDH1 WT | negative regulation of megakaryocyte differentiation | 10/1788 | 18/18800 | 1.26E-06 |
| GO:0060249 | CDH1-TANGO6 del vs CDH1 del | anatomical structure homeostasis | 34/854 | 319/18800 | 3.69E-06 |
| GO:0046982 | CDH1 del vs CDH1 WT | protein heterodimerization activity | 74/2359 | 332/18410 | 1.03E-06 |
| GO:0010466 | CDH1-TANGO6 del vs CDH1 del | negative regulation of peptidase activity | 29/854 | 262/18800 | 9.14E-06 |
| GO:0051056 | CDH1 del vs CDH1 WT | regulation of small GTPase mediated signal transduction | 65/2311 | 299/18800 | 2.74E-06 |
| GO:0050900 | CDH1-TANGO6 del vs CDH1 del | leukocyte migration | 37/854 | 384/18800 | 1.43E-05 |
| GO:0005774 | CDH1 del vs CDH1 WT | vacuolar membrane | 85/2417 | 449/19594 | 3.33E-05 |
| GO:0051056 | CDH1-TANGO6 del vs CDH1 WT | regulation of small GTPase mediated signal transduction | 53/1788 | 299/18800 | 6.47E-06 |
| GO:0015629 | CDH1-TANGO6 del vs CDH1 WT | actin cytoskeleton | 75/1858 | 499/19594 | 4.06E-05 |
| GO:0042476 | CDH1-TANGO6 del vs CDH1 del | odontogenesis | 18/854 | 130/18800 | 2.40E-05 |
| GO:0007162 | CDH1-TANGO6 del vs CDH1 del | negative regulation of cell adhesion | 31/854 | 305/18800 | 2.51E-05 |
| GO:0008544 | CDH1-TANGO6 del vs CDH1 del | epidermis development | 34/854 | 355/18800 | 3.56E-05 |
| GO:0048732 | CDH1-TANGO6 del vs CDH1 del | gland development | 39/854 | 431/18800 | 3.53E-05 |
| GO:0062197 | CDH1 del vs CDH1 WT | cellular response to chemical stress | 69/2311 | 332/18800 | 7.13E-06 |
| GO:0030496 | CDH1 del vs CDH1 WT | midbody | 44/2417 | 203/19594 | 0.000123 |
| GO:0045653 | CDH1 del vs CDH1 WT | negative regulation of megakaryocyte differentiation | out/11 | 18/18800 | 1.32E-05 |
| GO:0005884 | CDH1-TANGO6 del vs CDH1 WT | actin filament | 24/1858 | 113/19594 | 0.000128 |
| GO:0005911 | CDH1-TANGO6 del vs CDH1 WT | cell-cell junction | 73/1858 | 497/19594 | 0.000107 |
| GO:0009898 | CDH1-TANGO6 del vs CDH1 WT | cytoplasmic side of plasma membrane | 32/1858 | 169/19594 | 0.000112 |
| GO:0031252 | CDH1-TANGO6 del vs CDH1 WT | cell leading edge | 63/1858 | 416/19594 | 0.000131 |
| GO:0098562 | CDH1-TANGO6 del vs CDH1 WT | cytoplasmic side of membrane | 35/1858 | 193/19594 | 0.000135 |
| GO:0002685 | CDH1-TANGO6 del vs CDH1 del | regulation of leukocyte migration | 24/854 | 218/18800 | 5.76E-05 |
| GO:0000922 | CDH1 del vs CDH1 WT | spindle pole | 38/2417 | 169/19594 | 0.000157 |
| GO:0016125 | CDH1 del vs CDH1 WT | sterol metabolic process | 38/2311 | 154/18800 | 1.69E-05 |
| GO:0030198 | CDH1-TANGO6 del vs CDH1 del | extracellular matrix organization | 30/854 | 307/18800 | 7.00E-05 |
| GO:0002831 | CDH1-TANGO6 del vs CDH1 del | regulation of response to biotic stimulus | 33/854 | 351/18800 | 6.64E-05 |
| GO:0042481 | CDH1-TANGO6 del vs CDH1 del | regulation of odontogenesis | 6/854 | 17/18800 | 6.94E-05 |
| GO:0044409 | CDH1-TANGO6 del vs CDH1 del | entry into host | 19/854 | 153/18800 | 6.69E-05 |
| GO:0002687 | CDH1-TANGO6 del vs CDH1 del | positive regulation of leukocyte migration | 18/854 | 141/18800 | 7.19E-05 |
| GO:0043062 | CDH1-TANGO6 del vs CDH1 del | extracellular structure organization | 30/854 | 308/18800 | 7.43E-05 |
| GO:0030595 | CDH1-TANGO6 del vs CDH1 del | leukocyte chemotaxis | 25/854 | 236/18800 | 7.66E-05 |
| GO:0045229 | CDH1-TANGO6 del vs CDH1 del | external encapsulating structure organization | 30/854 | 310/18800 | 8.36E-05 |
| GO:0051346 | CDH1-TANGO6 del vs CDH1 del | negative regulation of hydrolase activity | 34/854 | 371/18800 | 8.58E-05 |
| GO:0005782 | CDH1 del vs CDH1 WT | peroxisomal matrix | 16/2417 | 50/19594 | 0.000217 |
| GO:0031907 | CDH1 del vs CDH1 WT | microbody lumen | 16/2417 | 50/19594 | 0.000217 |
| GO:0000228 | CDH1-TANGO6 del vs CDH1 WT | nuclear chromosome | 39/1858 | 228/19594 | 0.000209 |

| | |
|---|---|
| 0.00048 | 0.00045 |
| 0.00051 | 0.00047 |
| 0.00059 | 0.00053 |
| 0.00062 | 0.00056 |
| 0.00073 | 0.0007 |
| 0.00081 | 0.00072 |
| 0.00119 | 0.00113 |
| 0.00184 | 0.00164 |
| 0.00235 | 0.00217 |
| 0.00267 | 0.00237 |
| 0.00288 | 0.00259 |
| 0.00313 | 0.00297 |
| 0.00405 | 0.00368 |
| 0.00418 | 0.00372 |
| 0.00422 | 0.00375 |
| 0.00528 | 0.0047 |
| 0.00528 | 0.0047 |
| 0.00536 | 0.00494 |
| 0.0073 | 0.00657 |
| 0.00723 | 0.00666 |
| 0.00788 | 0.00717 |
| 0.00788 | 0.00717 |
| 0.00788 | 0.00717 |
| 0.00788 | 0.00717 |
| 0.00788 | 0.00717 |
| 0.00829 | 0.00737 |
| 0.00859 | 0.00773 |
| 0.00847 | 0.00781 |
| 0.00883 | 0.00785 |
| 0.00883 | 0.00785 |
| 0.00883 | 0.00785 |
| 0.00883 | 0.00785 |
| 0.00884 | 0.00786 |
| 0.00892 | 0.00793 |
| 0.00898 | 0.00799 |
| 0.00958 | 0.00851 |
| 0.00962 | 0.00855 |
| 0.01029 | 0.00926 |
| 0.01029 | 0.00926 |
| 0.01123 | 0.01021 |

| |
|---|
| CTSZ/SERPINE1/RARRES2/APOE/GDF15/MATN2/THBS1/LOXL4/HMCN1/ADAMTSL4/SHH/COL6A2/LAMB2/EGFL7/PKM/SLPI/PLSCR1/LAMB3/COL27A1/SERPINH1/COL6A1/S100A4/S |
| ASF1A/H1-3/MBD2/CDK2/ITGB3BP/H2BC1/HMGB2/NPM1/H1-4/H1-5/H1-10/SIRT6/ANP32B/H2BC11/H1-0/H1-2/NASP/H4C11/CDAN1/H2BC7/H2BC12/PHF2/H3C12/L3MBTL3/H2BC21/H2 |
| OAS1/CORO1A/RAB3D/BBS2/RCN3/ABCA12/ALDH1A1/PIWIL4/ABCA3/S1PR1/CD34/POC1B/CSF1/SNX10/MBP/SLC39A8/GPR137B/PTGS2/TP53INP2/SYK/CUBN/SPP1/CLDN3/LAMA |
| CD74/OAS1/SERPINE1/OAS3/EIF2AK2/EGR1/C3/PYCARD/HLA-F/THBS1/GSDMD/PRKCQ/SPON2/IFI16/CD34/CD276/WNT11/MAPK11/STING1/NLRP3/PLCB1/TNFRSF14/MBP/SAA1/S |
| H4C11/H4C8/H4C3/H4-16/H4C14/H4C4/H4C5/H4C9/H4C1/H4C13 |
| OAS1/CORO1A/RAB3D/BBS2/RCN3/ABCA12/ALDH1A1/PIWIL4/ABCA3/CALB2/FGGY/S1PR1/CD34/POC1B/CSF1/SNX10/MBP/SLC39A8/ATP2B2/GPR137B/PTGS2/TP53INP2/SYK/CU |
| ABCD1/CAV2/BHLHE41/IRAK2/TUBB2B/BCL2L10/BARD1/HEXA/H2BC1/NAE1/H2AZ1/LSM6/H2AC1/H2AC8/BCL2L1/AOC3/CENPS/H2AC21/H2AC20/CENPA/NR4A2/H2BC11/H2AC7/CA |
| SERPINE1/C3/SPINK5/PI15/THBS1/LAMP3/IFI16/RAG1/A2ML1/CST1/CST6/GAS6/SLPI/PLAUR/SERPINH1/IFI6/PTGS2/MAGEA3/DHCR24/KLF4/SERPINB9/SERPINB1/SERPINB5/ITIH |
| PPP2CB/RASIP1/ARHGDIB/RAP1GAP2/ARHGAP29/PDGFRB/ARHGEF19/RABL3/EPS8/CGNL1/FGD5/ARF6/RASGRP1/KRAS/RACGAP1/EPS8L2/F2R/MAP4K4/PSD3/CSF1/RASA3/SCA |
| CD74/CORO1A/SERPINE1/RARRES2/SELL/PYCARD/THBS1/EPS8/ITGA1/S1PR1/CXCL8/VEGFB/CD34/MMP14/GAS6/SPNS2/CSF1/PLCB1/PLEC/CREB3/TNFRSF14/SAA1/SIRPA/S100 |
| CTNS/GNA11/SNAP29/ABCD1/VPS18/RAB3D/ITFG2/SPHK2/ATP6V1E1/VPS4A/AP5M1/SLC30A4/WIPI1/RAB2A/MCOLN1/ENPEP/SLC46A3/MAP1LC3B/NPC1/ARL8A/GNAQ/CYB561A3 |
| PPP2CB/CADM4/ARHGDIB/APOE/RAP1GAP2/NGEF/ARHGAP31/RABL3/EPS8/CGNL1/ARF6/RASGRP1/ARHGAP45/F2R/MAP4K4/PSD3/RASA3/CYTH3/CD2AP/ARHGAP11A/RHOU/KI |
| CORO1A/ACTA2/AIF1L/RAC2/TMOD2/ANXA1/CDH1/COTL1/STK17B/SEPTIN11/MYZAP/CGN/CORIN/IQGAP2/TNNT3/CGNL1/MYO1E/MST1R/CFL2/MYO1A/AXL/RAC3/RND1/ABLIM3/AR |
| RELT/SERPINE1/WNT6/MSX2/BMP4/WNT10A/EDAR/NECTIN1/SHH/AQP3/ZNF22/CD34/CSF1/KLK5/SNX10/TFAP2A/MSX1/GLI3 |
| CD74/SERPINE1/BMP4/THBS1/CDH1/ABCA12/FZD7/SHH/MMP14/PLXND1/MAP4K4/ACER2/SH2B3/PDE5A/TNFRSF14/MBP/ADAM15/HLA-DRB1/GTPBP4/VTCN1/KLF4/PODXL/MIR675/ |
| MSX2/BMP4/TNFRSF19/KRT5/SPINK5/WNT10A/EDAR/ABCA12/SHH/AQP3/INSR/MAP2K1/KRT17/CST6/ETV4/KRT81/KRT6A/KLK5/MAFF/PLEC/PPL/LAMB3/HLA-DRB1/FLG/MYO6/MY |
| TG/MSX2/BMP4/WNT10A/EDAR/CDH1/SEMA3C/HK2/SHH/RAG1/INSR/MAP2K1/HOXB9/WNT11/PLXND1/CSF1/WNT7B/UPF2/FPGS/UPRT/XDH/MSX1/SERPINB5/OAS2/STRA6/LBH/GLI |
| ABCD1/SMPD3/BRF2/RELB/PKD2/PYROXD1/CAT/DHRS2/OSER1/ANXA1/HTRA2/EFHD1/FZD1/NOS3/AXL/FOS/GPR37/PPARGC1B/STK25/PINK1/ATP13A2/PAWR/TSPO/ERMP1/NR4A |
| ARL2BP/VPS4A/KATNBL1/YPEL5/CAPG/CENPE/JTB/ARL8A/RALB/ARF6/PLK1/SHCBP1/RACGAP1/TACC1/EML4/CHMP6/KLHDC8B/ANKRD45/PPP1CC/ZFYVE26/BIRC5/SEPTIN7/PIT |
| H4C11/H4C8/H4C2/H4C3/H4-16/H4C14/H4C4/H4C5/H4C9/H4C13 |
| CORO1A/AIF1L/RAC2/ANXA1/COTL1/MYO1A/RAC3/DIAPH2/MYO18B/PDLIM7/PLS3/DMTN/CD2AP/MYO6/SMTNL2/TFPT/MYO1B/DBN1/DIAPH3/TPM1/PSTPIP2/GAS2/AVIL/GJB6 |
| CORO1A/CADM4/PKD2/GJA3/KLHL24/WNK4/ANXA1/CDH1/ILDR2/CGN/ANK3/CGNL1/MYO1E/ITGA5/STEAP1/TMEM65/AQP3/RND1/PLXDC1/STXBP6/MAPK15/CLDN4/FLRT3/FERMT2/ |
| GNA11/CAV2/CDH1/ATP2C2/MYZAP/GNAQ/ANK1/SAMD12/FES/RASA3/MTSS2/FERMT2/CYTH3/TH/S100A6/NTSR1/GNG12/GNAS/TRAF1/GNG10/CNR2/SYK/ERRFI1/GNG4/MIEN1/AK |
| ITGB4/PLEK2/FERMT1/CORO1A/CADM4/ACTA2/PKD2/AIF1L/RAC2/CDH1/ARHGAP31/IQGAP2/EPS8/PDE9A/ITGA5/ARF6/RAC3/ABLIM3/ARHGAP45/ARPC2/PLXND1/PSD3/ASAP3/AD |
| GNA11/EEF1A2/CAV2/PKD2/CDH1/ATP2C2/MYZAP/GNAQ/ANK1/SAMD12/FES/RASA3/MTSS2/FERMT2/CYTH3/TH/S100A6/NTSR1/GNG12/GNAS/TRAF1/GNG10/CNR2/SYK/ERRFI1/G |
| CD74/SERPINE1/RARRES2/PYCARD/THBS1/CXCL8/VEGFB/MMP14/GAS6/CSF1/PLCB1/CREB3/TNFRSF14/MPP1/LRCH1/LGALS9/CYP19A1/ADAM8/TNF/PRSS56/CAMK1D/RIPOR2/M |
| PPP2CB/DLGAP5/TUBGCP6/VPS4A/CCNB1/KATNBL1/CKAP2/KIF11/YPEL5/TUBGCP3/EML1/CALM2/MAD2L1/NPM1/PLK1/TPX2/CKAP2L/POC1B/CALM1/RASSF1/ANKRD53/CENPF/N |
| CYP51A1/FDFT1/DGAT2/MVK/GNB3/CAT/EBPL/ERG28/CYP27A1/CYP2C9/MSMO1/SCARF1/OSBPL5/SQLE/NPC1/LBR/TM7SF2/HMGCR/PCSK9/OSBPL1A/HMGCS1/TSKU/DHCR7/ACA |
| SPINK5/C6orf15/LOXL4/CRISPLD2/HMCN1/ADAMTSL4/LAMB2/MMP14/CRTAP/FKBP10/GAS6/KLK5/LAMB3/COL27A1/SLC39A8/ADAM15/SERPINH1/MMP17/COL11A1/COL16A1/SERP |
| OAS1/OAS3/PYCARD/APOE/SPINK5/OASL/HLA-F/HERC5/IFI16/DTX3L/OTOP1/STING1/KLK5/PLSCR1/HLA-DRB1/STAT1/IFIT1/TRAF3IP1/SYK/OPTN/SERPINB9/DDX60/LGALS9/COC |
| BMP4/WNT10A/SHH/CD34/CSF1/MSX1 |
| CD74/VPS18/NECTIN1/INSR/CXCL8/GAS6/KRT6A/TRIM14/PLSCR1/SIGLEC1/TNFRSF14/HLA-DRB1/LGALS9/IFITM3/IFITM1/MRC1/IFITM2/SLC52A2/EFNB2 |
| CD74/SERPINE1/RARRES2/PYCARD/THBS1/CXCL8/VEGFB/MMP14/GAS6/CSF1/CREB3/TNFRSF14/LGALS9/ADAM8/TNF/PRSS56/CAMK1D/RIPOR2 |
| SPINK5/C6orf15/LOXL4/CRISPLD2/HMCN1/ADAMTSL4/LAMB2/MMP14/CRTAP/FKBP10/GAS6/KLK5/LAMB3/COL27A1/SLC39A8/ADAM15/SERPINH1/MMP17/COL11A1/COL16A1/SERP |
| CD74/CORO1A/SERPINE1/RARRES2/THBS1/ITGA1/S1PR1/CXCL8/VEGFB/GAS6/CSF1/PLEC/CREB3/SAA1/S100A9/MPP1/SYK/LGALS9/CYP19A1/ADAM8/TNFRSF11A/PRSS56/CAM |
| SPINK5/C6orf15/LOXL4/CRISPLD2/HMCN1/ADAMTSL4/LAMB2/MMP14/CRTAP/FKBP10/GAS6/KLK5/LAMB3/COL27A1/SLC39A8/ADAM15/SERPINH1/MMP17/COL11A1/COL16A1/SERP |
| SERPINE1/C3/SPINK5/PI15/THBS1/LAMP3/MGAT5/IFI16/RAG1/A2ML1/CST1/CST6/GAS6/SLPI/PLAUR/SERPINH1/IFI6/PTGS2/MAGEA3/DHCR24/KLF4/SERPINB9/SERPINB1/SERPIN |
| ECH1/NUDT12/CAT/AGPS/PAOX/LONP2/TYSND1/DHRS4/PIPOX/IDH1/DDO/HMGCL/ECI2/NUDT19/HSD17B4/EPHX2 |
| ECH1/NUDT12/CAT/AGPS/PAOX/LONP2/TYSND1/DHRS4/PIPOX/IDH1/DDO/HMGCL/ECI2/NUDT19/HSD17B4/EPHX2 |
| POLE2/BRMS1L/RPA3/INO80B/GINS2/GINS1/POLD3/ZRANB3/PLK1/ING2/GINS3/RUVBL1/BIRC5/H4C11/H3C12/DMAP1/HDAC8/H4C8/H4C3/TFPT/MACROH2A1/H2AX/H4-16/H4C14/A |

| | | |
|---|---|---|
| 42 | CC | -1.8516402 |
| 51 | BP | -5.46109233 |
| 31 | BP | -0.89802651 |
| 45 | BP | -0.4472136 |
| 10 | BP | -3.16227766 |
| 34 | BP | -1.02899151 |
| 74 | MF | -4.41741027 |
| 29 | BP | -0.92847669 |
| 65 | BP | -3.10086836 |
| 37 | BP | -2.46598481 |
| 85 | CC | 0.97618706 |
| 53 | BP | -4.80761974 |
| 75 | CC | -4.04145188 |
| 18 | BP | -1.41421356 |
| 31 | BP | -1.61644772 |
| 34 | BP | -2.40098019 |
| 39 | BP | -1.76140969 |
| 69 | BP | -0.36115756 |
| 44 | CC | -4.82418151 |
| 10 | BP | -3.16227766 |
| 24 | CC | -2.04124145 |
| 73 | CC | -4.33052245 |
| 32 | CC | -3.18198052 |
| 63 | CC | -5.16551446 |
| 35 | CC | -3.21158617 |
| 24 | BP | -1.63299316 |
| 38 | CC | -3.89331411 |
| 38 | BP | -2.59554274 |
| 30 | BP | -1.82574186 |
| 33 | BP | 2.95932015 |
| 6 | BP | -2.44948974 |
| 19 | BP | 0.22941573 |
| 18 | BP | -1.88561808 |
| 30 | BP | -1.82574186 |
| 25 | BP | -3 |
| 30 | BP | -1.82574186 |
| 34 | BP | -1.71498585 |
| 16 | CC | -1.5 |
| 16 | CC | -1.5 |
| 39 | CC | -4.32346015 |

| | | | | | |
|---|---|---|---|---|---|
| GO:0030133 | CDH1 del vs CDH1 WT | transport vesicle | 74/2417 | 402/19594 | 0.000255 |
| GO:0042599 | CDH1-TANGO6 del vs CDH1 del | lamellar body | 6/876 | 17/19594 | 6.35E-05 |
| GO:0001822 | CDH1-TANGO6 del vs CDH1 del | kidney development | 29/854 | 303/18800 | 0.000132 |
| GO:0009913 | CDH1-TANGO6 del vs CDH1 del | epidermal cell differentiation | 24/854 | 230/18800 | 0.000134 |
| GO:0032103 | CDH1-TANGO6 del vs CDH1 del | positive regulation of response to external stimulus | 38/854 | 442/18800 | 0.00013 |
| GO:0007159 | CDH1-TANGO6 del vs CDH1 del | leukocyte cell-cell adhesion | 34/854 | 381/18800 | 0.000144 |
| GO:0005776 | CDH1 del vs CDH1 WT | autophagosome | 27/2417 | 112/19594 | 0.000421 |
| GO:0005777 | CDH1 del vs CDH1 WT | peroxisome | 32/2417 | 141/19594 | 0.000419 |
| GO:0005795 | CDH1 del vs CDH1 WT | Golgi stack | 34/2417 | 153/19594 | 0.000426 |
| GO:0042579 | CDH1 del vs CDH1 WT | microbody | 32/2417 | 141/19594 | 0.000419 |
| GO:0002688 | CDH1-TANGO6 del vs CDH1 del | regulation of leukocyte chemotaxis | 16/854 | 124/18800 | 0.000157 |
| GO:0022409 | CDH1-TANGO6 del vs CDH1 del | positive regulation of cell-cell adhesion | 28/854 | 291/18800 | 0.000157 |
| GO:0019627 | CDH1-TANGO6 del vs CDH1 WT | urea metabolic process | 7/1788 | 12/18800 | 3.58E-05 |
| GO:0071941 | CDH1-TANGO6 del vs CDH1 WT | nitrogen cycle metabolic process | 7/1788 | 12/18800 | 3.58E-05 |
| GO:0043154 | CDH1-TANGO6 del vs CDH1 del | negative regulation of cysteine-type endopeptidase activity involved in apoptotic process | 12/854 | 77/18800 | 0.000173 |
| GO:0033700 | CDH1-TANGO6 del vs CDH1 del | phospholipid efflux | 5/854 | 13/18800 | 0.000181 |
| GO:0030324 | CDH1-TANGO6 del vs CDH1 del | lung development | 20/854 | 179/18800 | 0.00019 |
| GO:0050870 | CDH1-TANGO6 del vs CDH1 del | positive regulation of T cell activation | 23/854 | 223/18800 | 0.000217 |
| GO:0042178 | CDH1-TANGO6 del vs CDH1 WT | xenobiotic catabolic process | 10/1788 | 25/18800 | 5.06E-05 |
| GO:0022407 | CDH1-TANGO6 del vs CDH1 del | regulation of cell-cell adhesion | 38/854 | 456/18800 | 0.000245 |
| GO:0006665 | CDH1 del vs CDH1 WT | sphingolipid metabolic process | 38/2311 | 161/18800 | 4.87E-05 |
| GO:1902652 | CDH1 del vs CDH1 WT | secondary alcohol metabolic process | 36/2311 | 149/18800 | 4.50E-05 |
| GO:0055037 | CDH1-TANGO6 del vs CDH1 WT | recycling endosome | 34/1858 | 196/19594 | 0.000396 |
| GO:0008203 | CDH1 del vs CDH1 WT | cholesterol metabolic process | 34/2311 | 139/18800 | 5.55E-05 |
| GO:0016042 | CDH1 del vs CDH1 WT | lipid catabolic process | 65/2311 | 327/18800 | 5.51E-05 |
| GO:0072331 | CDH1-TANGO6 del vs CDH1 del | signal transduction by p53 class mediator | 19/854 | 170/18800 | 0.000272 |
| GO:0031301 | CDH1 del vs CDH1 WT | integral component of organelle membrane | 69/2417 | 382/19594 | 0.000694 |
| GO:0032102 | CDH1-TANGO6 del vs CDH1 del | negative regulation of response to external stimulus | 36/854 | 429/18800 | 0.000309 |
| GO:0032874 | CDH1-TANGO6 del vs CDH1 del | positive regulation of stress-activated MAPK cascade | 16/854 | 132/18800 | 0.000325 |
| GO:0002690 | CDH1-TANGO6 del vs CDH1 del | positive regulation of leukocyte chemotaxis | 13/854 | 94/18800 | 0.00032 |
| GO:0045621 | CDH1-TANGO6 del vs CDH1 del | positive regulation of lymphocyte differentiation | 14/854 | 107/18800 | 0.000343 |
| GO:0045926 | CDH1-TANGO6 del vs CDH1 del | negative regulation of growth | 24/854 | 245/18800 | 0.000346 |
| GO:0046718 | CDH1-TANGO6 del vs CDH1 del | viral entry into host cell | 17/854 | 146/18800 | 0.000346 |
| GO:0006670 | CDH1 del vs CDH1 WT | sphingosine metabolic process | out/11 | 21/18800 | 7.51E-05 |
| GO:0030330 | CDH1-TANGO6 del vs CDH1 del | DNA damage response, signal transduction by p53 class mediator | 11/854 | 72/18800 | 0.000381 |
| GO:0052372 | CDH1-TANGO6 del vs CDH1 del | modulation by symbiont of entry into host | 9/854 | 50/18800 | 0.00037 |
| GO:0055091 | CDH1-TANGO6 del vs CDH1 del | phospholipid homeostasis | 5/854 | 15/18800 | 0.000392 |
| GO:0060541 | CDH1-TANGO6 del vs CDH1 del | respiratory system development | 21/854 | 203/18800 | 0.000385 |
| GO:0005801 | CDH1 del vs CDH1 WT | cis-Golgi network | 19/2417 | 72/19594 | 0.000905 |
| GO:0001503 | CDH1-TANGO6 del vs CDH1 del | ossification | 35/854 | 420/18800 | 0.000424 |

| | |
|---|---|
| 0.01135 | 0.01022 |
| 0.01178 | 0.01106 |
| 0.01348 | 0.01198 |
| 0.01348 | 0.01198 |
| 0.01348 | 0.01198 |
| 0.01392 | 0.01238 |
| 0.01444 | 0.013 |
| 0.01444 | 0.013 |
| 0.01444 | 0.013 |
| 0.01444 | 0.013 |
| 0.01466 | 0.01303 |
| 0.01466 | 0.01303 |
| 0.01388 | 0.01315 |
| 0.01388 | 0.01315 |
| 0.01584 | 0.01408 |
| 0.01633 | 0.01452 |
| 0.01683 | 0.01496 |
| 0.01837 | 0.01633 |
| 0.01838 | 0.01741 |
| 0.01989 | 0.01768 |
| 0.0195 | 0.01797 |
| 0.0195 | 0.01797 |
| 0.01976 | 0.01797 |
| 0.01963 | 0.01809 |
| 0.01963 | 0.01809 |
| 0.0214 | 0.01902 |
| 0.02247 | 0.02023 |
| 0.02398 | 0.02132 |
| 0.02408 | 0.02141 |
| 0.02408 | 0.02141 |
| 0.0241 | 0.02143 |
| 0.0241 | 0.02143 |
| 0.0241 | 0.02143 |
| 0.02376 | 0.0219 |
| 0.02517 | 0.02238 |
| 0.02517 | 0.02238 |
| 0.02517 | 0.02238 |
| 0.02517 | 0.02238 |
| 0.02576 | 0.0232 |
| 0.02637 | 0.02344 |

| |
|---|
| KDELR3/RAB3D/CAV2/VGF/NTS/ERG28/SORL1/SLC40A1/APH1B/RAB27B/PCSK2/SYP/COPS4/APBA1/CNIH3/ABCA12/SEC24D/TMEM163/NCSTN/SYTL5/BCL2L1/TMEM168/BACE1/PI |
| SPINK5/LAMP3/ABCA12/ABCA3/KLK5/CKAP4 |
| WNT6/EGR1/CAT/BMP4/PRKX/SHH/LAMB2/CD34/WNT11/PLXND1/WNT7B/MME/MMP17/STAT1/WNT2B/TRAF3IP1/TP73/PODXL/TFAP2A/STRA6/GLI3/KIF26B/CC2D2A/PAX8/CDKN1C/( |
| MSX2/BMP4/KRT5/SPINK5/ABCA12/AQP3/MAP2K1/KRT17/ETV4/KRT81/KRT6A/KLK5/MAFF/PLEC/PPL/FLG/MYO6/MYCL/KLF4/KRT80/PALLD/VDR/KRT4/PDZD7 |
| CD74/SERPINE1/RARRES2/C3/PYCARD/OASL/HLA-F/THBS1/IFI16/S1PR1/CXCL8/VEGFB/GAS6/CSF1/STING1/KLK5/PLSCR1/CREB3/C2CD4A/PTGS2/S100A9/CAMK2N1/SYK/OPTN/[ |
| CD74/CORO1A/SELL/BMP4/TNFSF9/PYCARD/NT5E/PRKCQ/SHH/RAG1/CD276/NLRP3/PDE5A/TNFRSF14/SLC39A8/SIRPA/HLA-DRB1/S100A9/VTCN1/NKAP/KLF4/HLA-DMA/SYK/LGAI |
| SNAP29/VPS18/TBC1D17/WIPI1/MAP1LC3B/FTH1/RAB24/CHMP6/ATP13A2/STING1/MAPK15/ADPRH/MAP1LC3A/MCOLN3/TP53INP2/PIP4K2A/AUP1/SQSTM1/WDR81/RUBCNL/ULK3/ |
| ISOC1/ABCD1/ECH1/MVK/NUDT12/CAT/AGPS/PAOX/LONP2/TYSND1/HMGCR/PEX13/TMEM135/DHRS4/PXMP2/PIPOX/IDH2/STING1/DHRS4L2/IDH1/FAR1/ACSL3/DDO/PEX11B/HMG |
| CHPF2/SMPD3/RASIP1/CHPF/NECAB3/CHSY1/SORL1/MAN2A1/RAB27B/LPCAT2/B4GALNT3/GALNT1/MARCHF4/GOLGA6A/NAGPA/GOLGA8IP/PITPNM1/TMBIM4/GOLGA8A/FUT11/B |
| ISOC1/ABCD1/ECH1/MVK/NUDT12/CAT/AGPS/PAOX/LONP2/TYSND1/HMGCR/PEX13/TMEM135/DHRS4/PXMP2/PIPOX/IDH2/STING1/DHRS4L2/IDH1/FAR1/ACSL3/DDO/PEX11B/HMG |
| CD74/SERPINE1/RARRES2/THBS1/CXCL8/VEGFB/GAS6/CSF1/CREB3/MPP1/LGALS9/CYP19A1/PRSS56/CAMK1D/RIPOR2/SLC8B1 |
| CD74/CORO1A/TNFSF9/PYCARD/PRKCQ/SHH/RAG1/FUT3/CD276/NLRP3/PLAUR/TNFRSF14/SIRPA/HLA-DRB1/VTCN1/NKAP/HLA-DMA/SYK/PODXL/LGALS9/IL4R/GLI3/CARD11/KIF2( |
| CPS1/CYP2C9/ARG2/ASL/SLC25A15/NR1H4/CEBPA |
| CPS1/CYP2C9/ARG2/ASL/SLC25A15/NR1H4/CEBPA |
| THBS1/LAMP3/RAG1/GAS6/PLAUR/IFI6/PTGS2/MAGEA3/DHCR24/KLF4/SERPINB9/TNF |
| APOE/ABCA12/ABCA3/ABCA1/ABCG1 |
| CTSZ/BMP4/CRISPLD2/RCN3/ABCA12/SHH/ABCA3/MAP2K1/MMP14/WNT11/WNT7B/MME/WNT2B/STRA6/GLI3/CCDC40/HOPX/TNF/CEBPA/THRB |
| CD74/CORO1A/TNFSF9/PYCARD/PRKCQ/SHH/RAG1/CD276/NLRP3/TNFRSF14/SIRPA/HLA-DRB1/VTCN1/NKAP/HLA-DMA/SYK/LGALS9/IL4R/GLI3/CARD11/HLA-DQB1/ADAM8/EFNB2 |
| GSTM2/NUDT15/CYP2C9/ACSL1/ACAA1/CRYZ/GSTM3/GSTM4/CYP2C19/CYP1A1 |
| CD74/CORO1A/BMP4/TNFSF9/PYCARD/CDH1/ZDHHC2/PRKCQ/ABCA12/SHH/RAG1/FUT3/CD276/NLRP3/PLAUR/SH2B3/PDE5A/TNFRSF14/MBP/SIRPA/HLA-DRB1/GTPBP4/VTCN1/NK/ |
| GLA/SMPD3/ITGB8/SPHK2/SGPP1/CERS4/PEMT/HTRA2/HEXA/ABCA12/NAAA/SPTSSA/ACER1/CERS6/UGT8/GLTP/SGPP2/SPNS2/B4GALNT1/ALDH3B1/HACD1/GBA/ELOVL3/SGPL |
| CYP51A1/FDFT1/DGAT2/MVK/GNB3/CAT/CYP27A1/CYP2C9/MSMO1/SCARF1/OSBPL5/SQLE/NPC1/LBR/TM7SF2/HMGCR/PCSK9/OSBPL1A/HMGCS1/IDH2/TSKU/IDH1/DHCR7/ACADV |
| TBC1D17/SLC31A2/HLA-F/SORL1/ZDHHC2/VAMP8/SLC26A7/VPS51/TMUB1/ARF6/TPP1/SLC39A4/SCAMP3/STX8/OR2A4/BOK/ABHD17B/ATP9A/SYT11/MCOLN2/SLC31A1/OPTN/ARH |
| CYP51A1/FDFT1/DGAT2/MVK/GNB3/CAT/CYP27A1/CYP2C9/MSMO1/SCARF1/OSBPL5/SQLE/NPC1/LBR/TM7SF2/HMGCR/PCSK9/OSBPL1A/HMGCS1/TSKU/DHCR7/ACADVL/APOL2/G |
| CYP24A1/ABCD1/GLA/SMPD3/ECH1/CPS1/FAAH/CYP27A1/HINT2/SORL1/SCARF1/SRD5A3/PLAAT1/MGLL/HEXA/PLBD2/LONP2/NAAA/TYSND1/PEX13/ENPP6/PLIN1/SLC27A4/ACER |
| CD74/DDX5/PYCARD/SETD9/IFI16/SPRED1/DDIT4/ELL3/NDRG1/SP100/ACER2/TAF9B/MYO6/PRMT6/ANKRD1/PLK3/TP73/MSX1/HIPK2 |
| ABCD1/COA1/DGAT2/PKD2/SLC35A2/TRAM1/SYP/UBXN8/SLC46A3/NPC1/LBR/TEX261/TMEM177/LNPK/TM7SF2/TMEM163/AMFR/PEX13/COX11/ARL6IP1/PINK1/PORCN/ATP13A2/C( |
| OAS1/SERPINE1/OAS3/BBS2/NR1D1/APOE/SPINK5/NT5E/HLA-F/THBS1/SEMA3C/IFI16/OTOP1/CD34/BACE1/MACIR/NLRP3/SH2B3/SAA1/SEMA4F/SIRPA/HLA-DRB1/TRAF3IP1/KLF4 |
| EIF2AK2/BMP4/PYCARD/TNFRSF19/GADD45G/EDAR/TRAF5/FZD7/PLCB1/WNT7B/RASSF2/XDH/HIPK2/TNF/TPD52L1/MYD88 |
| CD74/SERPINE1/RARRES2/THBS1/CXCL8/VEGFB/GAS6/CSF1/CREB3/LGALS9/PRSS56/CAMK1D/RIPOR2 |
| CD74/TNFSF9/SHH/RAG1/MMP14/GAS6/NLRP3/HLA-DRB1/NKAP/SYK/LGALS9/IL4R/GLI3/ADAM8 |
| BBS2/MT2A/BMP4/GDF15/STC2/SEMA3C/NKD1/MINAR1/CDK5R1/WNT11/MAPK11/ADAM15/SEMA4F/PSRC1/TP73/MSX1/OSGIN1/SPP1/SEMA4D/PNPT1/SIPA1/BCL11A/SLC6A4/PRD |
| CD74/VPS18/NECTIN1/INSR/GAS6/TRIM14/PLSCR1/SIGLEC1/TNFRSF14/HLA-DRB1/LGALS9/IFITM3/IFITM1/MRC1/IFITM2/SLC52A2/EFNB2 |
| SPHK2/SGPP1/NAAA/SPTSSA/ACER1/SGPP2/GBA/SPTLC3/PLPP2/SPTSSB |
| CD74/DDX5/SPRED1/NDRG1/SP100/ACER2/MYO6/ANKRD1/PLK3/MSX1/HIPK2 |
| CD74/CXCL8/KRT6A/TRIM14/HLA-DRB1/LGALS9/IFITM3/IFITM1/IFITM2 |
| LIPG/RCN3/ABCA3/ABCA1/ABCG1 |
| CTSZ/BMP4/CRISPLD2/RCN3/ABCA12/SHH/ABCA3/MAP2K1/MMP14/WNT11/WNT7B/SPEF2/MME/WNT2B/STRA6/GLI3/CCDC40/HOPX/TNF/CEBPA/THRB |
| KDELR3/MAN2A1/GPR108/B3GAT3/SCYL1/GOLGA6A/GOLGA8IP/FKTN/GOLGA8A/PHTF1/TMED5/SLC35C2/GOLGA8N/GOLIM4/PIK3R1/GOLGA8Q/GOLGA8M/GOLGA8H/GOLGA8B |
| DDX5/MSX2/CAT/BMP4/SHH/KREMEN2/S1PR1/MMP14/WNT11/CSF1/MAPK11/SNX10/WNT7B/GTPBP4/COL6A1/PTGS2/COL11A1/TP53INP2/BAMBI/RASSF2/TFAP2A/MIR675/SPP1/GI |

| | | |
|---|---|---|
| 74 | CC | 0.23249528 |
| 6 | CC | 1.63299316 |
| 29 | BP | -0.92847669 |
| 24 | BP | -2.85773803 |
| 38 | BP | -0.32444284 |
| 34 | BP | -1.71498585 |
| 27 | CC | 2.11695099 |
| 32 | CC | -2.82842712 |
| 34 | CC | 2.05798302 |
| 32 | CC | -2.82842712 |
| 16 | BP | -2 |
| 28 | BP | -1.88982237 |
| 7 | BP | -0.37796447 |
| 7 | BP | -0.37796447 |
| 12 | BP | -0.57735027 |
| 5 | BP | 0.4472136 |
| 20 | BP | -0.89442719 |
| 23 | BP | -1.04257207 |
| 10 | BP | 1.26491106 |
| 38 | BP | -1.94665705 |
| 38 | BP | -0.32444284 |
| 36 | BP | -2.66666667 |
| 34 | CC | -2.05798302 |
| 34 | BP | -2.40098019 |
| 65 | BP | 0.86824314 |
| 19 | BP | 1.14707867 |
| 69 | CC | -1.32424438 |
| 36 | BP | -0.66666667 |
| 16 | BP | 0.5 |
| 13 | BP | -1.94145069 |
| 14 | BP | -1.60356745 |
| 24 | BP | -0.81649658 |
| 17 | BP | 0.72760688 |
| 10 | BP | 0 |
| 11 | BP | -0.30151134 |
| 9 | BP | 0.33333333 |
| 5 | BP | -0.4472136 |
| 21 | BP | -0.65465367 |
| 19 | CC | 1.60591014 |
| 35 | BP | -2.53546276 |

| | | | | | |
|---|---|---|---|---|---|
| GO:0000079 | CDH1 del vs CDH1 WT | regulation of cyclin-dependent protein serine/threonine kinase activity | 26/2311 | 98/18800 | 9.71E-05 |
| GO:0090175 | CDH1 del vs CDH1 WT | regulation of establishment of planar polarity | 18/2311 | 57/18800 | 0.000103 |
| GO:0030148 | CDH1 del vs CDH1 WT | sphingolipid biosynthetic process | 28/2311 | 110/18800 | 0.000117 |
| GO:0048640 | CDH1-TANGO6 del vs CDH1 del | negative regulation of developmental growth | 14/854 | 111/18800 | 0.000502 |
| GO:0036064 | CDH1 del vs CDH1 WT | ciliary basal body | 34/2417 | 161/19594 | 0.001108 |
| GO:0030148 | CDH1-TANGO6 del vs CDH1 WT | sphingolipid biosynthetic process | 24/1788 | 110/18800 | 8.57E-05 |
| GO:0019058 | CDH1 del vs CDH1 WT | viral life cycle | 62/2311 | 317/18800 | 0.000131 |
| GO:0007265 | CDH1 del vs CDH1 WT | Ras protein signal transduction | 66/2311 | 347/18800 | 0.000188 |
| GO:0032956 | CDH1 del vs CDH1 WT | regulation of actin cytoskeleton organization | 67/2311 | 352/18800 | 0.000165 |
| GO:0070734 | CDH1 del vs CDH1 WT | histone H3-K27 methylation | set/11 | 19/18800 | 0.000183 |
| GO:0140014 | CDH1 del vs CDH1 WT | mitotic nuclear division | 58/2311 | 293/18800 | 0.000152 |
| GO:0006643 | CDH1 del vs CDH1 WT | membrane lipid metabolic process | 44/2311 | 207/18800 | 0.00018 |
| GO:0010638 | CDH1 del vs CDH1 WT | positive regulation of organelle organization | 87/2311 | 487/18800 | 0.000196 |
| GO:0032392 | CDH1 del vs CDH1 WT | DNA geometric change | 25/2311 | 96/18800 | 0.000181 |
| GO:0032970 | CDH1 del vs CDH1 WT | regulation of actin filament-based process | 73/2311 | 393/18800 | 0.000189 |
| GO:0043087 | CDH1 del vs CDH1 WT | regulation of GTPase activity | 69/2311 | 364/18800 | 0.000151 |
| GO:0005635 | CDH1 del vs CDH1 WT | nuclear envelope | 82/2417 | 479/19594 | 0.001244 |
| GO:0045780 | CDH1-TANGO6 del vs CDH1 del | positive regulation of bone resorption | 5/854 | 16/18800 | 0.000549 |
| GO:0030336 | CDH1-TANGO6 del vs CDH1 del | negative regulation of cell migration | 30/854 | 346/18800 | 0.000559 |
| GO:0031638 | CDH1-TANGO6 del vs CDH1 del | zymogen activation | 10/854 | 64/18800 | 0.000579 |
| GO:0035458 | CDH1-TANGO6 del vs CDH1 del | cellular response to interferon-beta | 6/854 | 24/18800 | 0.000575 |
| GO:0070229 | CDH1-TANGO6 del vs CDH1 del | negative regulation of lymphocyte apoptotic process | 7/854 | 33/18800 | 0.00059 |
| GO:0045104 | CDH1-TANGO6 del vs CDH1 del | intermediate filament cytoskeleton organization | 12/854 | 88/18800 | 0.000612 |
| GO:0040013 | CDH1-TANGO6 del vs CDH1 del | negative regulation of locomotion | 33/854 | 396/18800 | 0.000611 |
| GO:0042771 | CDH1-TANGO6 del vs CDH1 del | intrinsic apoptotic signaling pathway in response to DNA damage by p53 class mediator | 8/854 | 43/18800 | 0.000618 |
| GO:0045619 | CDH1-TANGO6 del vs CDH1 del | regulation of lymphocyte differentiation | 19/854 | 181/18800 | 0.000599 |
| GO:0005685 | CDH1 del vs CDH1 WT | U1 snRNP | nov/17 | 33/19594 | 0.001417 |
| GO:0006665 | CDH1-TANGO6 del vs CDH1 WT | sphingolipid metabolic process | 31/1788 | 161/18800 | 0.000109 |
| GO:0006753 | CDH1-TANGO6 del vs CDH1 WT | nucleoside phosphate metabolic process | 73/1788 | 495/18800 | 0.000103 |
| GO:0051235 | CDH1-TANGO6 del vs CDH1 WT | maintenance of location | 53/1788 | 331/18800 | 0.000112 |
| GO:0001649 | CDH1-TANGO6 del vs CDH1 del | osteoblast differentiation | 23/854 | 241/18800 | 0.000657 |
| GO:0045103 | CDH1-TANGO6 del vs CDH1 del | intermediate filament-based process | 12/854 | 89/18800 | 0.000679 |
| GO:0050878 | CDH1-TANGO6 del vs CDH1 del | regulation of body fluid levels | 32/854 | 382/18800 | 0.000672 |
| GO:0060439 | CDH1-TANGO6 del vs CDH1 del | trachea morphogenesis | 4/854 | 10/18800 | 0.000713 |
| GO:0000940 | CDH1 del vs CDH1 WT | outer kinetochore | jun/17 | 12/19594 | 0.001666 |
| GO:0008021 | CDH1 del vs CDH1 WT | synaptic vesicle | 39/2417 | 196/19594 | 0.00164 |
| GO:0019898 | CDH1 del vs CDH1 WT | extrinsic component of membrane | 57/2417 | 314/19594 | 0.001714 |
| GO:0005814 | CDH1 del vs CDH1 WT | centriole | 31/2417 | 147/19594 | 0.001832 |
| GO:0005938 | CDH1-TANGO6 del vs CDH1 WT | cell cortex | 47/1858 | 310/19594 | 0.00086 |
| GO:1902107 | CDH1-TANGO6 del vs CDH1 del | positive regulation of leukocyte differentiation | 17/854 | 157/18800 | 0.000803 |

| | |
|---|---|
| 0.02821 | 0.026 |
| 0.02821 | 0.026 |
| 0.02821 | 0.026 |
| 0.03013 | 0.02678 |
| 0.03035 | 0.02733 |
| 0.02931 | 0.02777 |
| 0.03019 | 0.02782 |
| 0.03104 | 0.0286 |
| 0.03104 | 0.0286 |
| 0.03104 | 0.0286 |
| 0.03104 | 0.0286 |
| 0.03104 | 0.0286 |
| 0.03104 | 0.0286 |
| 0.03104 | 0.0286 |
| 0.03104 | 0.0286 |
| 0.03104 | 0.0286 |
| 0.03208 | 0.02888 |
| 0.03258 | 0.02896 |
| 0.03277 | 0.02913 |
| 0.03319 | 0.02951 |
| 0.03319 | 0.02951 |
| 0.03344 | 0.02972 |
| 0.03351 | 0.02979 |
| 0.03351 | 0.02979 |
| 0.03351 | 0.02979 |
| 0.03351 | 0.02979 |
| 0.03362 | 0.03027 |
| 0.03263 | 0.03092 |
| 0.03263 | 0.03092 |
| 0.03263 | 0.03092 |
| 0.03487 | 0.031 |
| 0.035 | 0.03112 |
| 0.035 | 0.03112 |
| 0.03594 | 0.03195 |
| 0.03589 | 0.03231 |
| 0.03589 | 0.03231 |
| 0.03589 | 0.03231 |
| 0.03726 | 0.03355 |
| 0.03751 | 0.03411 |
| 0.03893 | 0.03461 |

| |
|---|
| TFAP4/CDKN1B/PKD2/CDKN1A/CCNB1/MNAT1/CCND2/KAT2B/CCNA2/PLK1/CDKN2A/CCNE2/CDK5R1/CDC25C/CDKN3/SFN/CCNG1/GTPBP4/PROX1/PSRC1/CDKN2C/NR2F2/CCNF/ME |
| CELSR3/FZD3/NKD1/ARHGEF19/CELSR2/FZD7/FZD1/DVL3/WNT11/VANGL1/FZD6/DAAM1/PKHD1/MLLT3/SAPCD2/TIAM1/PRICKLE2/CDC42 |
| SPHK2/SGPP1/CERS4/PEMT/SPTSSA/ACER1/CERS6/UGT8/SGPP2/SPNS2/B4GALNT1/ALDH3B1/HACD1/GBA/ELOVL3/SGPL1/UGCG/PRKAA1/SPTLC3/PLPP2/CCN1/ST6GALNAC5/E |
| BBS2/BMP4/GDF15/STC2/SEMA3C/NKD1/CDK5R1/MAPK11/SEMA4F/TP73/SPP1/SEMA4D/BCL11A/SLC6A4 |
| CCDC113/PKD2/BBS2/CFAP20/IFT46/PTPN23/BBS4/RILPL2/POC1B/C2CD3/MAPK15/CFAP410/SSX2IP/CFAP70/DAAM1/CENPF/CETN2/PKHD1/TRAF3IP1/IQCD/CCDC88A/ODF2L/TTC |
| PRKD3/SPHK2/SGPP1/CERS4/PEMT/ELOVL6/UGT8/ST8SIA5/CERS5/SPNS2/PLA2G6/B4GALNT1/ALDH3B1/GBA/ELOVL3/SGPL1/UGCG/ST8SIA6/PRKAA1/SPTLC3/SPTSSB/A4GALT |
| OAS1/LGALS1/VPS18/CAV2/EFNB3/ZC3HAV1/VPS4A/OASL/N4BP1/NECTIN1/ICAM1/TARBP2/NPC1/CXADR/LY6E/ITGA5/AXL/CXCR6/ISG20/CXCL8/APOBEC3F/ROCK2/EEA1/CHMP6 |
| PPP2CB/RASIP1/ARHGDIB/CDKN1A/RAC2/RRAS2/ARHGAP29/PDGFRB/CDK2/RALB/RABL3/CCNA2/CDC42EP2/EPS8/ARF6/CDKN2A/RASGRP1/KRAS/ROCK2/EPS8L2/F2R/MAP4K4/F |
| CORO1A/ARHGDIB/TMSB10/SORBS3/PYCARD/RAC2/TMOD2/RHOBTB2/PDGFRB/CAPG/CAPZB/BBS4/ARHGEF19/IQGAP2/CDC42EP2/EPS8/CGNL1/WHAMM/CFL2/ARF6/S1PR1/CDK |
| H1-3/CHD5/H1-4/H1-2/MTF2/PHF19/PHF1/EZH1/MACROH2A1 |
| SMPD3/CAV2/CDKN1B/FBXO5/DUSP1/BMP4/DLGAP5/NCAPG/VPS4A/CCNB1/ESPL1/KIF11/NDC80/PDGFRB/CENPE/NUF2/EPS8/RIOK2/BUB1B/MAD2L1/PLK1/TPX2/RACGAP1/PSMG |
| PIGB/PIGH/GLA/SMPD3/ITGB8/SPHK2/SGPP1/CERS4/PEMT/HTRA2/HEXA/ABCA12/NAAA/SPTSSA/ACER1/CERS6/UGT8/GLTP/SGPP2/B3GNT5/SPNS2/B4GALNT1/ALDH3B1/PGAP1 |
| ZNF205/SMPD3/CDKN1B/SORBS3/DLGAP5/PYCARD/NCAPG/KATNBL1/ANXA1/HRK/ESPL1/HTRA2/CCT7/MNS1/PDGFRB/WIPI1/PRKCQ/RAB3GAP1/CDK2/BBS4/RALB/LNPK/CDC42EI |
| DDX1/GINS2/IGHMBP2/ANXA1/MNAT1/CHD5/GINS1/ZRANB3/HMGB2/TWNK/GINS3/RUVBL1/HMGB3/HMGB1/NAV2/CHD1L/CHD6/RFC3/TOP2A/CHD9/CHD4/PIF1/MCM3/HNRNPA2B1/T |
| CORO1A/ACTA2/ARHGDIB/TNNC1/TMSB10/SORBS3/PYCARD/RAC2/TMOD2/RHOBTB2/TRPM4/PDGFRB/CAPG/CAPZB/BBS4/ARHGEF19/IQGAP2/CDC42EP2/EPS8/CGNL1/WHAMM/C |
| TBC1D17/RASIP1/CAV2/PYCARD/RAP1GAP2/ARHGAP29/RAB3GAP1/BBS4/ARHGEF19/IQGAP2/MMUT/DOCK11/FGD5/SLC27A4/S1PR1/RASGRP1/DVL3/BIN1/F2R/WNT11/PLXND1/M |
| OSBPL6/RAC2/DHRS2/RAP1GAP2/SORL1/BCL2L10/MNS1/CCND2/LMNB1/PLAAT1/NPC1/CALR3/LBR/OTULINL/EI24/TM7SF2/NUP205/GNAQ/ATP5MF/NXF1/MAD2L1/CLMN/BCL2L1/NI |
| SYK/SPP1/ADAM8/TMEM64/TNFRSF11A |
| CD74/APEX1/SERPINE1/ACVR1C/APOE/THBS1/CDH1/SHH/SPRED1/MACIR/WNT11/PLCB1/SP100/ADARB1/ADAM15/ARPIN/ATP2B4/GTPBP4/KLF4/LRCH1/DNAJA4/CLDN3/CYP19A1/ |
| CTSZ/SERPINE1/PYCARD/THBS1/IFI16/PRSS12/MMP14/DHCR24/CUZD1/C1R |
| OAS1/IFI16/STING1/STAT1/PNPT1/IFITM2 |
| CD74/BMP4/PRKCQ/RAG1/ST3GAL1/HSH2D/FOXP1 |
| KRT5/SHH/KRT17/KRT81/KRT6A/PLEC/PPL/FAM83H/KRT80/VIM/KRT4/EPPK1 |
| CD74/APEX1/SERPINE1/ACVR1C/APOE/THBS1/CDH1/SEMA3C/SHH/SPRED1/MACIR/WNT11/PLCB1/SP100/ADARB1/ADAM15/ARPIN/ATP2B4/SEMA4F/GTPBP4/KLF4/LRCH1/DNAJA4 |
| CD74/PYCARD/IFI16/DDIT4/ELL3/TAF9B/TP73/HIPK2 |
| CD74/BMP4/TNFSF9/SPINK5/ZFP36L2/SHH/RAG1/MMP14/GAS6/NLRP3/IKZF3/HLA-DRB1/NKAP/SYK/LGALS9/IL4R/GLI3/CARD11/ADAM8 |
| SNRPD3/SNRPA/SNRPD1/RNVU1-3/RNVU1-6/RNVU1-15/RNVU1-14/LUC7L3/RNVU1-4/RNVU1-8/WEE2-AS1 |
| GLA/ITGB8/PRKD3/SPHK2/SGPP1/CERS4/PEMT/ABCA12/NAAA/ELOVL6/UGT8/ST8SIA5/CERS5/SPNS2/PLA2G6/B4GALNT1/ALDH3B1/TH/GBA/ELOVL3/SGPL1/SERINC2/UGCG/NEU |
| NME4/TGFB1/MVK/ENO2/NUDT12/SPHK2/TYMP/CMPK2/NT5E/NUDT15/PPAT/HSD17B12/ACSL1/SLC26A2/DCK/HK2/PDE9A/ATP5MF/NOS3/ELOVL6/ADORA2B/ATP5ME/RRM2/DTYMK |
| KDELR3/CORO1A/MEST/TMSB10/PKD2/GSTM2/ACVR1C/C3/APOE/SORL1/BARD1/CASQ2/SLC30A4/MCOLN1/SQLE/ANK3/UBASH3B/HK2/CDS1/GAA/XCR1/NRIP1/F2R/RASA3/TSPO/ |
| DDX5/MSX2/CAT/BMP4/SHH/WNT11/MAPK11/WNT7B/GTPBP4/COL6A1/TP53INP2/BAMBI/RASSF2/MIR675/SPP1/GLI3/CEBPD/IFITM1/SEMA4D/TNF/CCN1/TMEM64/CEBPA |
| KRT5/SHH/KRT17/KRT81/KRT6A/PLEC/PPL/FAM83H/KRT80/VIM/KRT4/EPPK1 |
| SERPINE1/APOE/THBS1/PRKCQ/TRPV5/ABCA12/MYO5B/HK2/SHH/AQP3/CD34/GAS6/ADRA2C/PLAUR/CLDN4/SH2B3/PLSCR1/PLEC/SAA1/EMP2/FLG/ENTPD1/VAV2/UPRT/SYK/XDI |
| BMP4/SHH/MAP2K1/WNT7B |
| CCNB1/NDC80/BUB1B/PLK1/SKA2/CENPF |
| RAB3D/SLC40A1/RAB27B/SYP/COPS4/APBA1/TMEM163/NCSTN/BCL2L1/BACE1/VAMP2/BIN1/ROGDI/TMED9/SLC17A5/BDNF/MCTP2/SYN3/MME/SYT11/SNAPIN/RAB3B/GABRA2/DL |
| GNA11/CAV2/GNB3/COQ3/ANXA1/CDH1/WIPI1/EHD2/MGLL/MYZAP/OTULINL/GNAQ/NAAA/COQ5/PCSK9/GRAMD2A/KRAS/RACGAP1/YES1/BIN1/EEA1/CARMIL2/SNX10/PLAUR/STK |
| TUBGCP3/CFAP20/CAPG/BBS4/PLK1/TCP10L/CEP120/HSPA6/POC1B/C2CD3/MAPK15/CETN2/HSPA1B/HSPA1A/CEP152/CCNF/TOP2A/CCDC88A/ODF2L/CIBAR1/NIN/CEP290/CEP1 |
| CORO1A/ENO2/PKD2/RAC2/RHOBTB2/CDH1/COTL1/SEPTIN11/RAI14/MYZAP/EPS8/MYO1E/BSN/MELK/ARF6/STOX1/MYO1A/RAC3/RND1/CABP1/STXBP6/CRIP2/PCLO/MTSS2/FER |
| CD74/TNFSF9/SHH/RAG1/MMP14/GAS6/CSF1/NLRP3/HLA-DRB1/NKAP/SYK/LGALS9/IL4R/GLI3/ADAM8/TNF/TMEM64 |

| | | |
|---|---|---|
| 26 | BP | -3.9223227 |
| 18 | BP | -1.88561808 |
| 28 | BP | -1.13389342 |
| 14 | BP | -0.53452248 |
| 34 | CC | -3.08697453 |
| 24 | BP | -0.81649658 |
| 62 | BP | -2.54000254 |
| 66 | BP | -2.95419578 |
| 67 | BP | -3.298575 |
| 9 | BP | -1 |
| 58 | BP | -4.72703158 |
| 44 | BP | -0.60302269 |
| 87 | BP | -2.03701382 |
| 25 | BP | -3 |
| 73 | BP | -3.39419327 |
| 69 | BP | -3.49118974 |
| 82 | CC | -3.97553494 |
| 5 | BP | -1.34164079 |
| 30 | BP | -1.09544512 |
| 10 | BP | 0 |
| 6 | BP | 1.63299316 |
| 7 | BP | -0.37796447 |
| 12 | BP | -2.88675135 |
| 33 | BP | -1.21854359 |
| 8 | BP | 1.41421356 |
| 19 | BP | -1.60591014 |
| 11 | CC | -2.7136021 |
| 31 | BP | -0.89802651 |
| 73 | BP | -2.69194639 |
| 53 | BP | -2.33512959 |
| 23 | BP | -2.71068738 |
| 12 | BP | -2.88675135 |
| 32 | BP | -1.41421356 |
| 4 | BP | -2 |
| 6 | CC | -2.44948974 |
| 39 | CC | 1.12089708 |
| 57 | CC | -3.04642442 |
| 31 | CC | -3.05329013 |
| 47 | CC | -3.06316482 |
| 17 | BP | -2.18282063 |

| | | | | | |
|---|---|---|---|---|---|
| GO:0030856 | CDH1-TANGO6 del vs CDH1 del | regulation of epithelial cell differentiation | 17/854 | 157/18800 | 0.000803 |
| GO:1903708 | CDH1-TANGO6 del vs CDH1 del | positive regulation of hemopoiesis | 17/854 | 157/18800 | 0.000803 |
| GO:0072521 | CDH1-TANGO6 del vs CDH1 WT | purine-containing compound metabolic process | 63/1788 | 416/18800 | 0.000141 |
| GO:0043903 | CDH1-TANGO6 del vs CDH1 del | regulation of biological process involved in symbiotic interaction | 9/854 | 56/18800 | 0.000876 |
| GO:0000395 | CDH1 del vs CDH1 WT | mRNA 5'-splice site recognition | out/11 | 24/18800 | 0.000295 |
| GO:0001736 | CDH1 del vs CDH1 WT | establishment of planar polarity | 20/2311 | 72/18800 | 0.000309 |
| GO:0007164 | CDH1 del vs CDH1 WT | establishment of tissue polarity | 20/2311 | 72/18800 | 0.000309 |
| GO:0036010 | CDH1 del vs CDH1 WT | protein localization to endosome | out/11 | 24/18800 | 0.000295 |
| GO:0051084 | CDH1 del vs CDH1 WT | 'de novo' post-translational protein folding | 13/2311 | 37/18800 | 0.000287 |
| GO:0030219 | CDH1 del vs CDH1 WT | megakaryocyte differentiation | 17/2311 | 57/18800 | 0.000344 |
| GO:0045071 | CDH1 del vs CDH1 WT | negative regulation of viral genome replication | 17/2311 | 57/18800 | 0.000344 |
| GO:1904029 | CDH1-TANGO6 del vs CDH1 WT | regulation of cyclin-dependent protein kinase activity | 22/1788 | 101/18800 | 0.00017 |
| GO:0006644 | CDH1 del vs CDH1 WT | phospholipid metabolic process | 71/2311 | 388/18800 | 0.000361 |
| GO:0022038 | CDH1 del vs CDH1 WT | corpus callosum development | jul/11 | 13/18800 | 0.000367 |
| GO:2000027 | CDH1 del vs CDH1 WT | regulation of animal organ morphogenesis | 30/2311 | 129/18800 | 0.00038 |
| GO:0002504 | CDH1-TANGO6 del vs CDH1 del | antigen processing and presentation of peptide or polysaccharide antigen via MHC class II | 7/854 | 36/18800 | 0.001024 |
| GO:0060993 | CDH1-TANGO6 del vs CDH1 del | kidney morphogenesis | 12/854 | 93/18800 | 0.001009 |
| GO:0070228 | CDH1-TANGO6 del vs CDH1 del | regulation of lymphocyte apoptotic process | 9/854 | 57/18800 | 0.000999 |
| GO:0097094 | CDH1-TANGO6 del vs CDH1 del | craniofacial suture morphogenesis | 5/854 | 18/18800 | 0.000999 |
| GO:0043588 | CDH1-TANGO6 del vs CDH1 del | skin development | 26/854 | 296/18800 | 0.001052 |
| GO:0001660 | CDH1-TANGO6 del vs CDH1 del | fever generation | 4/854 | 11/18800 | 0.00108 |
| GO:0006665 | CDH1-TANGO6 del vs CDH1 del | sphingolipid metabolic process | 17/854 | 161/18800 | 0.001065 |
| GO:0072205 | CDH1-TANGO6 del vs CDH1 del | metanephric collecting duct development | 4/854 | 11/18800 | 0.00108 |
| GO:0046486 | CDH1 del vs CDH1 WT | glycerolipid metabolic process | 72/2311 | 396/18800 | 0.000399 |
| GO:0002673 | CDH1-TANGO6 del vs CDH1 del | regulation of acute inflammatory response | 8/854 | 47/18800 | 0.001142 |
| GO:0002675 | CDH1-TANGO6 del vs CDH1 del | positive regulation of acute inflammatory response | 6/854 | 27/18800 | 0.001125 |
| GO:0010875 | CDH1-TANGO6 del vs CDH1 del | positive regulation of cholesterol efflux | 6/854 | 27/18800 | 0.001125 |
| GO:0061005 | CDH1-TANGO6 del vs CDH1 del | cell differentiation involved in kidney development | 9/854 | 58/18800 | 0.001136 |
| GO:0005874 | CDH1 del vs CDH1 WT | microtubule | 74/2417 | 435/19594 | 0.002468 |
| GO:0060071 | CDH1 del vs CDH1 WT | Wnt signaling pathway, planar cell polarity pathway | 16/2311 | 53/18800 | 0.000438 |
| GO:0090224 | CDH1 del vs CDH1 WT | regulation of spindle organization | 14/2311 | 43/18800 | 0.000419 |
| GO:0019369 | CDH1 del vs CDH1 WT | arachidonic acid metabolic process | 17/2311 | 58/18800 | 0.000431 |
| GO:0051235 | CDH1 del vs CDH1 WT | maintenance of location | 62/2311 | 331/18800 | 0.000442 |
| GO:0010952 | CDH1-TANGO6 del vs CDH1 del | positive regulation of peptidase activity | 19/854 | 192/18800 | 0.001221 |
| GO:0036293 | CDH1-TANGO6 del vs CDH1 del | response to decreased oxygen levels | 26/854 | 299/18800 | 0.001216 |
| GO:0005543 | CDH1 del vs CDH1 WT | phospholipid binding | 88/2359 | 467/18410 | 0.000114 |
| GO:0016628 | CDH1 del vs CDH1 WT | oxidoreductase activity, acting on the CH-CH group of donors, NAD or NADP as acceptor | dez/59 | 29/18410 | 0.00012 |
| GO:0001667 | CDH1-TANGO6 del vs CDH1 del | ameboidal-type cell migration | 37/854 | 480/18800 | 0.001249 |

| | |
|---|---|
| 0.03893 | 0.03461 |
| 0.03893 | 0.03461 |
| 0.03904 | 0.03699 |
| 0.04169 | 0.03707 |
| 0.04131 | 0.03808 |
| 0.04131 | 0.03808 |
| 0.04131 | 0.03808 |
| 0.04131 | 0.03808 |
| 0.04131 | 0.03808 |
| 0.04217 | 0.03887 |
| 0.04217 | 0.03887 |
| 0.04129 | 0.03912 |
| 0.04322 | 0.03984 |
| 0.04322 | 0.03984 |
| 0.04388 | 0.04044 |
| 0.04612 | 0.041 |
| 0.04612 | 0.041 |
| 0.04612 | 0.041 |
| 0.04612 | 0.041 |
| 0.04693 | 0.04172 |
| 0.04694 | 0.04173 |
| 0.04694 | 0.04173 |
| 0.04694 | 0.04173 |
| 0.04528 | 0.04173 |
| 0.0472 | 0.04196 |
| 0.0472 | 0.04196 |
| 0.0472 | 0.04196 |
| 0.0472 | 0.04196 |
| 0.0475 | 0.04276 |
| 0.04658 | 0.04293 |
| 0.04658 | 0.04293 |
| 0.04658 | 0.04293 |
| 0.04658 | 0.04293 |
| 0.04898 | 0.04354 |
| 0.04898 | 0.04354 |
| 0.0462 | 0.04359 |
| 0.0462 | 0.04359 |
| 0.04961 | 0.0441 |

| |
|---|
| SERPINE1/MSX2/BMP4/PRKX/ABCA12/AQP3/SPRED1/ETV4/PLCB1/MAFF/STAT1/MYCL/XDH/PAX8/CDKN1C/TNF/VDR |
| CD74/TNFSF9/SHH/RAG1/MMP14/GAS6/CSF1/NLRP3/HLA-DRB1/NKAP/SYK/LGALS9/IL4R/GLI3/ADAM8/TNF/TMEM64 |
| NME4/TGFB1/MVK/ENO2/SPHK2/MACROD1/NT5E/NUDT15/PPAT/HSD17B12/ACSL1/SLC26A2/DCK/HK2/PDE9A/ATP5MF/NOS3/ELOVL6/ADORA2B/ATP5ME/PIPOX/NUPR1/HMGCS1/[illegible] |
| CD74/CXCL8/KRT6A/TRIM14/HLA-DRB1/LGALS9/IFITM3/IFITM1/IFITM2 |
| SRSF1/RNVU1-3/RNVU1-6/PSIP1/RNVU1-15/RNVU1-14/RNU11/RNVU1-4/RNVU1-8/WEE2-AS1 |
| CELSR3/FZD3/NKD1/ARHGEF19/CELSR2/FZD7/FZD1/DVL3/WNT11/VANGL1/FZD6/DAAM1/PKHD1/MLLT3/SAPCD2/TIAM1/EXOC5/PRICKLE2/RPGRIP1L/CDC42 |
| CELSR3/FZD3/NKD1/ARHGEF19/CELSR2/FZD7/FZD1/DVL3/WNT11/VANGL1/FZD6/DAAM1/PKHD1/MLLT3/SAPCD2/TIAM1/EXOC5/PRICKLE2/RPGRIP1L/CDC42 |
| SORL1/WASHC2A/ARF6/VPS35/ROCK2/ABHD17B/MGAT3/NRP1/WASHC2C/RDX |
| HSPA13/DNAJC18/HSPA6/SELENOF/ENTPD5/TOR2A/HSPA1B/HSPA1A/HSPA1L/HSPA14/DNAJB14/DNAJB12/BAG1 |
| PITHD1/HMGB2/MTURN/SH2B3/H4C11/UBA5/PRMT6/PIP4K2A/H4C8/H4C2/H4C3/H4-16/H4C14/H4C4/H4C5/H4C9/H4C13 |
| OAS1/ZC3HAV1/OASL/N4BP1/ISG20/APOBEC3F/APOBEC3B/PLSCR1/PROX1/TRIM6/RSAD2/OAS2/IFITM3/APOBEC3G/IFITM1/TNF/ISG15 |
| TFAP4/PKD2/MNAT1/CCND2/CCNA2/STOX1/PLK1/CDKN2A/CCNE2/CDC25C/HEXIM1/SFN/PROX1/PSRC1/CCND3/RGCC/NR2F2/CCNF/WEE2/MEN1/CEBPA/TNFAIP3 |
| PIGB/PIGH/SMPD3/FDFT1/MTMR9/MVK/GNB3/CHPT1/SPHK2/SGPP1/ABHD8/PEMT/HTR2B/PDGFRB/LPCAT2/OSBPL5/SRD5A3/PLAAT1/PIP5K1B/CHKA/ETNK1/PLBD2/NAAA/ENPP6/ |
| C12orf57/RTN4RL1/TSKU/EPHB2/NIN/SZT2/RPGRIP1L |
| CELSR3/FZD3/BMP4/WNT10A/NKD1/ARHGEF19/CELSR2/FZD7/FZD1/CD34/DVL3/WNT11/MAGED1/CSF1/APCDD1/VANGL1/FZD6/DAAM1/WNT2B/PKHD1/LGR4/MLLT3/MSX1/PDGFA/S[illegible] |
| CD74/PYCARD/THBS1/HLA-DRB1/HLA-DMA/HLA-DQB1/MARCHF8 |
| WNT6/BMP4/PRKX/SHH/WNT11/WNT7B/STAT1/WNT2B/GLI3/KIF26B/PAX8/GCNT4 |
| CD74/BMP4/PRKCQ/RAG1/LGALS9/ADAM8/ST3GAL1/HSH2D/FOXP1 |
| MSX2/BMP4/MMP14/INSIG1/GLI3 |
| MSX2/TNFRSF19/KRT5/SPINK5/WNT10A/EDAR/ABCA12/SHH/AQP3/MAP2K1/KRT17/ETV4/KRT81/KRT6A/KLK5/CLDN4/PLEC/PPL/FLG/DHCR24/KRT80/LTB/TNF/PALLD/VDR/KRT4 |
| PTGES/PTGS2/TNF/TNFRSF11A |
| PRKD3/CERS4/HEXA/ABCA12/SPTSSA/ELOVL6/FUT3/CERS6/SPNS2/ACER2/ALDH3B1/HACD1/SERINC2/SPTLC3/CCN1/ST3GAL1/ALDH3B2 |
| BMP4/SHH/WNT7B/PAX8 |
| PIGB/PIGH/MTMR9/DGAT2/GNB3/CHPT1/CPS1/CAT/FAAH/C3/ABHD8/CYP2E1/PEMT/HTR2B/SORL1/PDGFRB/LPCAT2/OSBPL5/PLAAT1/MGLL/PIP5K1B/CHKA/ETNK1/NAAA/ENPP6/[illegible] |
| C3/NLRP3/PTGES/C2CD4A/PTGS2/ADAM8/TNF/TNFRSF11A |
| C3/C2CD4A/PTGS2/ADAM8/TNF/TNFRSF11A |
| APOE/ABCA12/ABCA3/PLTP/ABCA1/ABCG1 |
| BMP4/PRKX/SHH/LAMB2/CD34/STAT1/PODXL/GLI3/PAX8 |
| FSD1/RAB3D/CLIP2/TTL/DYNLL1/TUBGCP6/CCT7/DYNC1LI2/CKAP2/TUBB2B/LRRC49/TTLL7/KIF11/TUBGCP3/EML1/CFAP20/KIF3C/CENPE/MAP1LC3B/CALM2/FAM161B/WHAMM/PL[illegible] |
| CELSR3/FZD3/NKD1/ARHGEF19/CELSR2/FZD7/FZD1/DVL3/WNT11/VANGL1/FZD6/DAAM1/MLLT3/TIAM1/PRICKLE2/CDC42 |
| FSD1/PLK1/TPX2/CHMP6/KIF15/MAPK15/ANKRD53/PSRC1/HSPA1B/HSPA1A/BORA/RAE1/DCTN1/GNAI1 |
| FAAH/CYP2E1/CYP2C9/MGLL/CYP2C18/CYP2S1/CYP2U1/PTGES2/PTGS1/PLA2G4A/CYP2J2/CYP2C8/AKR1C3/CYP4F11/PLA2G4B/PLA2G4C/CYP1B1 |
| KDELR3/CORO1A/ARL2BP/MEST/DGAT2/TMSB10/PKD2/ACVR1C/C3/HTR2B/SORL1/BARD1/CASQ2/SLC30A4/MCOLN1/SQLE/BBS4/CALM2/FTH1/HK2/XCR1/NFKBIB/NRIP1/F2R/PINK |
| FIS1/TNFSF10/ACVR1C/PYCARD/RCN3/IFI16/NLRP3/CLDN4/ACER2/MBP/VCP/S100A9/SYK/XDH/CLDN3/LGALS9/DAPK1/TNF/CCN1 |
| EGR1/CAT/THBS1/STC2/HK2/AQP3/STOX1/VASN/DDIT4/VEGFB/CD34/MMP14/NDRG1/SUV39H2/ADAM15/PTGS2/ATP1B1/CHRNB2/PLK3/SLC39A12/CLDN3/HIPK2/ADAM8/TNF/ITPR1 |
| SMPD3/NME4/SNX8/CPS1/FAAH/ANXA1/THBS1/WIPI1/OSBPL5/CAPG/IQGAP2/WASHC2A/FZD7/CPNE2/SYTL5/PACSIN3/TPP1/AXL/SNX7/GRAMD2A/RASGRP1/SNX31/RACGAP1/PL |
| BLVRB/SRD5A3/LBR/TM7SF2/CYP2S1/PTGES2/DHCR7/ACADSB/DPYD/DHCR24/AKR1C3/PTGR1 |
| ACVR1C/BMP4/APOE/THBS1/PRKX/SEMA3C/S100P/SHH/SPRED1/MACIR/WNT11/PLXND1/MAP4K4/GLUL/TESK1/SP100/PLEC/ATP2B4/SEMA4F/EPHB4/EMP2/PTGS2/S100A2/SRPX |

| | | |
|---|---|---|
| 17 | BP | -1.21267813 |
| 17 | BP | -2.18282063 |
| 63 | BP | -3.14970394 |
| 9 | BP | 0.33333333 |
| 10 | BP | -2.52982213 |
| 20 | BP | -2.23606798 |
| 20 | BP | -2.23606798 |
| 10 | BP | -0.63245553 |
| 13 | BP | -0.2773501 |
| 17 | BP | -2.66789188 |
| 17 | BP | -0.72760688 |
| 22 | BP | -2.13200716 |
| 71 | BP | -2.25488515 |
| 7 | BP | -1.13389342 |
| 30 | BP | -0.36514837 |
| 7 | BP | 0.37796447 |
| 12 | BP | -1.73205081 |
| 9 | BP | -0.33333333 |
| 5 | BP | -2.23606798 |
| 26 | BP | -2.74562589 |
| 4 | BP | 0 |
| 17 | BP | 0.24253563 |
| 4 | BP | -2 |
| 72 | BP | -2.12132034 |
| 8 | BP | -1.41421356 |
| 6 | BP | -1.63299316 |
| 6 | BP | 0 |
| 9 | BP | -1 |
| 74 | CC | -3.48742916 |
| 16 | BP | -1.5 |
| 14 | BP | -1.60356745 |
| 17 | BP | -0.24253563 |
| 62 | BP | -1.77800178 |
| 19 | BP | -1.14707867 |
| 26 | BP | -2.35339362 |
| 88 | MF | -1.06600358 |
| 12 | MF | -1.73205081 |
| 37 | BP | -2.46598481 |
