# Supplementary figures and images for: Combined loss of CDH1 and downstream regulatory sequences drive early-onset diffuse gastric cancer and increase penetrance of hereditary diffuse gastric cancer
Source: Gastric Cancer. 2023 May 30;26(5):653–66. doi: 10.1007/s10120-023-01395-0 (PMC10361908; doi:10.1007/s10120-023-01395-0)

Supplementary figure 1. Evolution of HDGC clinical criteria

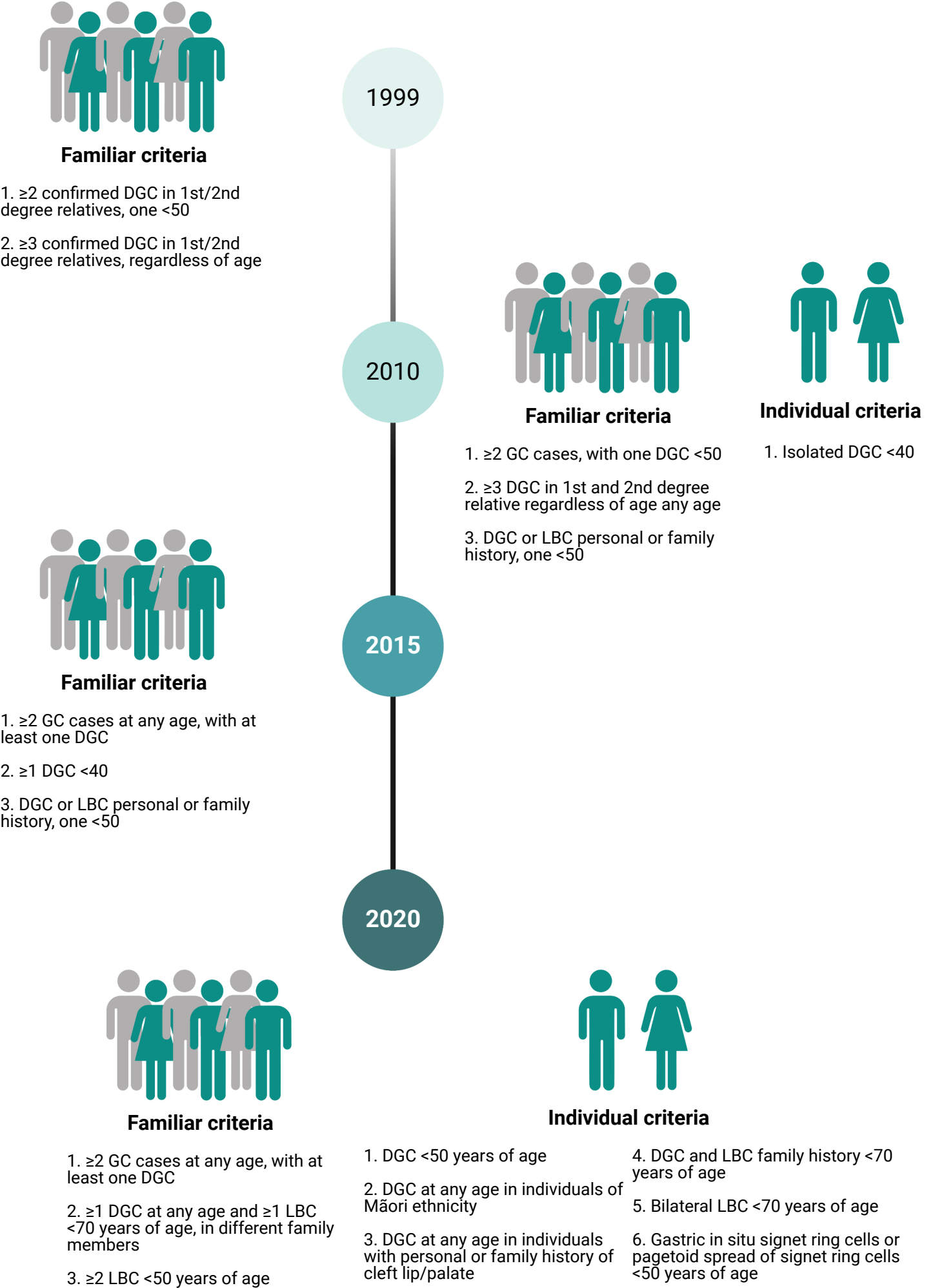

Supplement: Supplementary file 10 — Supplementary file10 (PDF 233 KB) [file 10120_2023_1395_MOESM10_ESM.pdf]
